# Sporulation capability and amylosome conservation among diverse human colonic and rumen isolates of the keystone starch‐degrader *Ruminococcus bromii*

**DOI:** 10.1111/1462-2920.14000

**Published:** 2017-12-07

**Authors:** Indrani Mukhopadhya, Sarah Moraïs, Jenny Laverde‐Gomez, Paul O. Sheridan, Alan W. Walker, William Kelly, Athol V. Klieve, Diane Ouwerkerk, Sylvia H. Duncan, Petra Louis, Nicole Koropatkin, Darrell Cockburn, Ryan Kibler, Philip J. Cooper, Carlos Sandoval, Emmanuelle Crost, Nathalie Juge, Edward A. Bayer, Harry J. Flint

**Affiliations:** ^1^ Microbiology Group The Rowett Institute, University of Aberdeen Aberdeen UK; ^2^ Department of Biomolecular Sciences The Weizmann Institute of Science Rehovot Israel; ^3^ AgResearch Limited, Grasslands Research Centre, Palmerston North 4442 New Zealand; ^4^ School of Agriculture and Food Sciences The University of Queensland QLD St Lucia, Australia; ^5^ Queensland Alliance for Agriculture and Food Innovation The University of Queensland QLD St Lucia, Australia; ^6^ Department of Agriculture and Fisheries Agri‐Science Queensland Brisbane QLD Australia; ^7^ Department of Microbiology and Immunology University of Michigan Medical School Ann Arbor MI USA; ^8^ Hospital Cantonal “Padre Alberto Buffoni”, Avenida 3 de Julio y Victor Villegas Quininde Esmeraldas Province Ecuador; ^9^ The Gut Health and Food Safety Institute Strategic Programme, Institute of Food Research Norwich UK; ^10^ Present address: Faculty of Natural Sciences, Ben‐Gurion University of the Negev Beer‐Sheva 8499000 Israel

## Abstract

*Ruminococcus bromii* is a dominant member of the human colonic microbiota that plays a ‘keystone’ role in degrading dietary resistant starch. Recent evidence from one strain has uncovered a unique cell surface ‘amylosome’ complex that organizes starch‐degrading enzymes. New genome analysis presented here reveals further features of this complex and shows remarkable conservation of amylosome components between human colonic strains from three different continents and a *R. bromii* strain from the rumen of Australian cattle. These *R. bromii* strains encode a narrow spectrum of carbohydrate active enzymes (CAZymes) that reflect extreme specialization in starch utilization. Starch hydrolysis products are taken up mainly as oligosaccharides, with only one strain able to grow on glucose. The human strains, but not the rumen strain, also possess transporters that allow growth on galactose and fructose. *R. bromii* strains possess a full complement of sporulation and spore germination genes and we demonstrate the ability to form spores that survive exposure to air. Spore formation is likely to be a critical factor in the ecology of this nutritionally highly specialized bacterium, which was previously regarded as ‘non‐sporing’, helping to explain its widespread occurrence in the gut microbiota through the ability to transmit between hosts.

## Introduction

Ruminococcaceae are an important family of Firmicutes bacteria within gut microbial communities (La Reau *et al*., 2016). These bacteria account for around 20% of the healthy human colonic microbiota based on molecular surveys (Suau *et al*., 2001) with two species, *Faecalibacterium prausnitzii* and *Ruminococcus bromii*, among the four most abundant contributors to the human faecal metagenome in European adults (Zhernakova *et al*., 2016). While *F. prausnitzii* in particular has been implicated in health maintenance (Sokol *et al*., 2008) a scarcity of cultured isolates, partly due to fastidious growth requirements (Herbeck and Bryant, 1974; Ze *et al*., 2012) has limited the information available on other Ruminococcaceae. *R. bromii* is thought to be a specialist starch‐utilizing bacterium, with at least one strain showing superior ability to degrade insoluble starches when compared with other amylolytic human gut bacteria (Ze *et al*., 2012). Resistant starch (RS), i.e., dietary starch that escapes digestion by host amylases, often provides the largest single source of energy for microbial growth in the human colon and its fermentation is considered to provide health benefits (Nugent, 2005). Significantly, the relative abundance of *R. bromii* 16S rRNA gene sequences has been found to increase rapidly and dramatically in faecal samples from human volunteers after switching onto diets high in RS (Walker *et al*., 2011; Salonen *et al*., 2014), indicating an outstanding ability to compete for RS *in vivo*. Similar rapid population increases have been reported for *R. bromii* within the rumen microbiota in cattle fed starch‐enriched diets (Klieve *et al*., 2007). The primary role played by *R. bromii* in releasing energy from RS to other members of the microbial community, and the drop in RS fermentation when this species is absent from the community, justifies designating it as a ‘keystone’ species within the human colonic microbiota (Ze *et al*., 2012, 2013).

An initial investigation into the amylases of the human *R. bromii* strain L2–63 concluded that several starch‐degrading enzymes are organized into unique multienzyme complexes that we have termed ‘amylosomes’, which may explain the exceptional RS‐degrading activity of this strain (Ze *et al*., 2015). Amylosome complexes are assembled via interactions between dockerin and cohesin modules present in enzymes and structural proteins (scaffoldins) (Ze *et al*., 2015) in a manner suggested by the organization of cellulosome complexes implicated in bacterial degradation of lignocellulose (Bayer *et al*., 2008). Highly elaborate forms of cellulosome organization are found in the related rumen cellulolytic species *Ruminococcus flavefaciens* (Ding *et al*., 2001; Rincon *et al*., 2010) and in the human cellulolytic species *Ruminococcus champanellensis* (Ben David *et al*., 2015; Moraïs *et al*., 2016). Amylosome organization has been demonstrated so far only in *R. bromii* L2–63 however and its significance and occurrence across other strains of *R. bromii* is unknown. Indeed, with only a single genome available for analysis until now there has been no information on genetic variation and limited opportunity to probe the microbial ecology of this species. Here we examine the genomic and phenotypic characteristics of four human *R. bromii* strains of diverse origin and of one rumen *R. bromii* strain. This investigation reveals remarkable new insights into the conservation of systems involved in substrate utilization and degradation and into survival mechanisms in this important, but little known, gut symbiont.

## Results

### Comparative genomics of *R. bromii* strains

**Figure 1:**
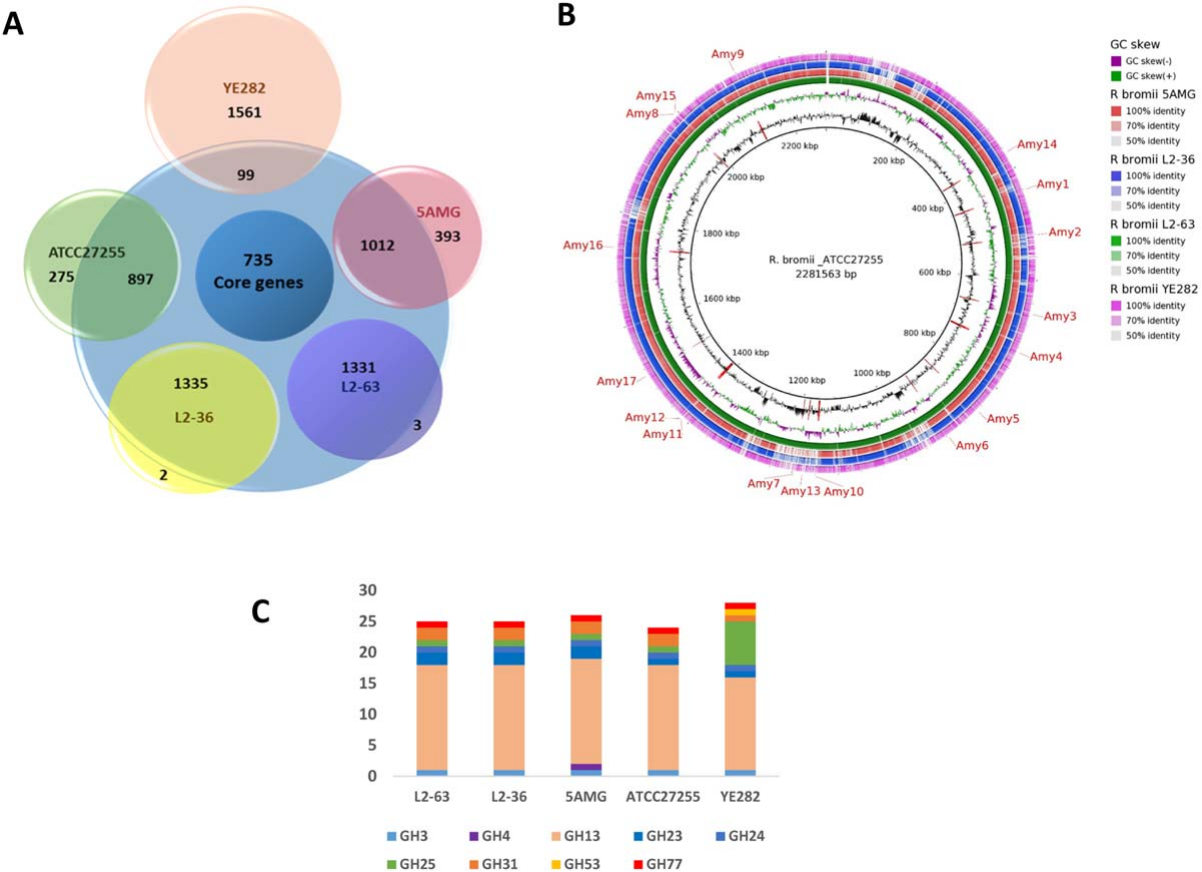
Comparison of five *R. bromii* genomes. A. Pan‐genome analysis of core, accessory and unique genes among five *R. bromii* strains. A total of 735 core orthologous groups (OGs), 1456 variable OGs and 2240 unique genes were detected in the *R. bromii* pan‐genome. Overall, the *R. bromii* YE282 strain genome has the highest number of unique genes (1561) compared to the human *R. bromii* genomes suggesting that these may be associated with colonization of the rumen. B. Genomic comparison of four *R. bromii* genomes to reference strain ATCC27255. Diagram represents BLASTn results of each genome against ATCC2755 strain with results rendered using the BRIG program. The inner circle represents the reference genome *R. bromii* ATCC27255. Each genome is colour coded as indicated by the legend. Relative shading density (from darker to lighter) within each circle represents relative levels of nucleotide homology. White regions indicate regions with no identity to the reference. The location of genes encoding GH13 enzymes (Amy1–17) is also indicated. C. Representation (number per genome) of glycoside hydrolase families encoded by *R. bromii* genomes.

Draft genomes were obtained here for three human *R. bromii* strains L2–36, 5AMG, ATCC27255 and for the rumen strain YE282. The existing draft genome of the human strain *R. bromii* L2–63 was re‐annotated for comparison (Supporting Information Table S1, Fig. 1, Experimental procedures). Genome size estimates for the four human strains (2151–2400 kb) are slightly lower than for the rumen YE282 strain (2539 kb). The genomic sequences of the *R. bromii* human strains share 95%–100% average nucleotide identity with each other and 86% identity with the rumen strain. Genomes of L2–63, L2–36, 5AMG, ATCC27255 and YE282 *R. bromii* strains were compared using BLAST and the atlases were generated using Blast Ring Image generator (BRIG) software (Fig. 1A). A pan‐genome analysis identified 735 core genes common to all 5 strains, with *R. bromii* YE282 possessing by far the highest number (1561) of unique genes thus establishing its divergence from the human strains (Fig. 1B, Supporting Information Fig. S1, Data File S1).

### Conservation of glycoside hydrolases and carbohydrate‐binding modules among *R. bromii* strains

**Figure 2:**
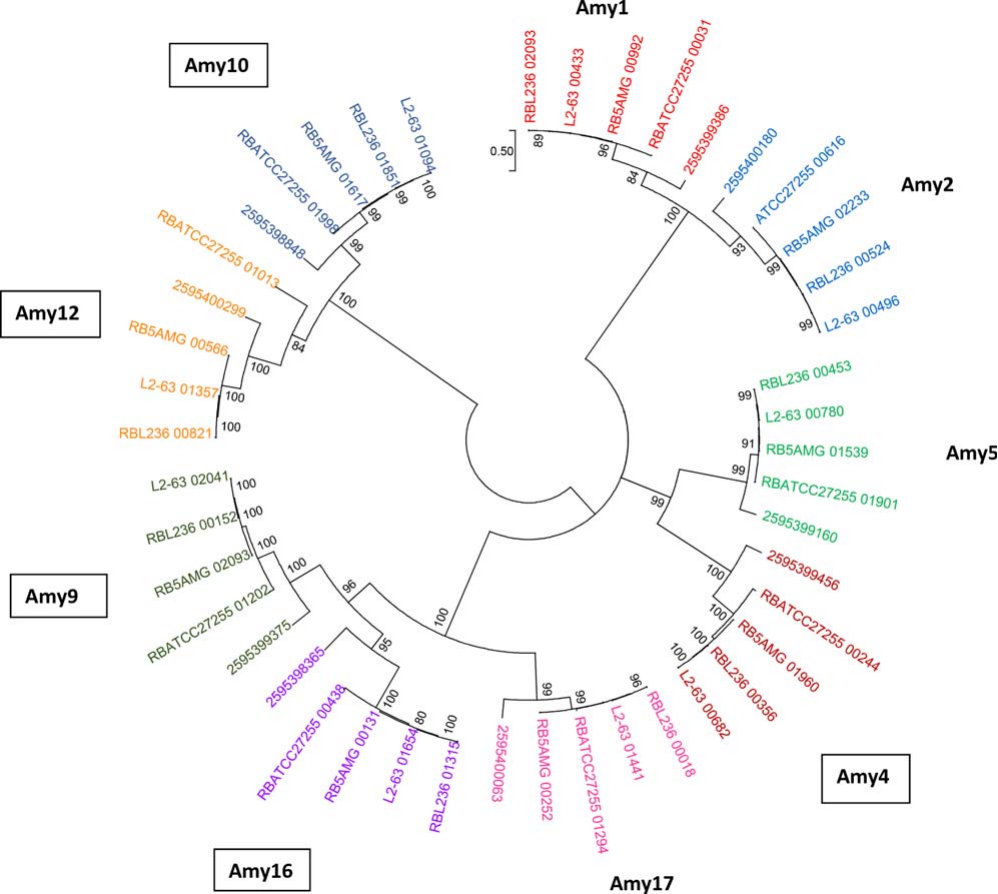
Phylogenetic tree based on the amino acid sequences of GH13 catalytic domains of the 9 extracellular GH13 enzymes (that carry signal peptides) from 5 *R. bromii* strains. The sequences of the GH13 genes shown here from the five *R. bromii* strains fall into 9 significant clusters, with sequences from the four human *R. bromii* strains clustering more closely with each other than the YE282 rumen strain. YE282 sequences are identified here by their numerical identifiers in the JGI database, whereas genes from the human strains are prefixed by the strain designation. Boxes indicate GH13 domains that are associated with dockerins. Bootstrap values, expressed as a percentage of 1000 replications, are given at the branching nodes. This tree is unrooted and reconstructed using the maximum‐likelihood method. The scale bar refers to the number of amino acid differences per position. Similar clustering was also observed for the remaining GH13 enzymes listed in Table 1 that are common to all five strains (not shown).

The genomes of the four human *R. bromii* strains encode between 24 and 26 glycoside hydrolases (GHs), and the rumen strain YE282 28 GHs (Supporting Information Table S2), with no polysaccharide lyases detectable at the known polypeptide sequence level. This represents an unusually limited CAZyme repertoire for a carbohydrate‐utilizing gut bacterium (El Kaoutari *et al*., 2013) with the great majority of CAZymes belonging to family GH13, and to families (GH23, GH24, GH25) that encode lysozymes (Fig. 1C) although the GH23 family is also known to encode chitinases. Among the families that are represented, GH13, GH31 and GH77 enzymes are mostly concerned with starch utilization (Fig. 1C). 17 GH13 enzymes were found in all four human *R. bromii* genomes and 15 in the rumen strain YE282 which lacked homologues of Amy3 and Amy7 (Table 1). With three exceptions, genes encoding GH13 enzymes were not clustered (Fig. 1A). Phylogenetic analysis revealed remarkable conservation of homologous GH13 sequences as shown in Fig. 2 for the 9 extracellular GH13 enzymes that carry N‐terminal signal peptides. Furthermore CBM26 modules that have been implicated in starch binding (Boraston *et al*., 2006) are present in the same 7 extracellular GH13 proteins in all five strains (Table 1).

**Table 1:** Conserved modular organization of *Ruminococcus bromii* gene products that include GH13 glycoside hydrolase domains.

	Size (aa) human	Size (aa) rumen	Signal peptide	Cohesin domain	Dockerin domain	CBM 26	CBM 48	Presumed function	Domain architecture (GH13 domains in bold)
Amy 1	805–804	797	**+**	–	–	**+ (1)**	–	amylase	SP CBM26 **GH13**
Amy 2	752–751	748	**+**	–	–	**+ (1)**	–	amylase	SP CBM26 **GH13**
Amy 3^a^	630–629	–	–	–	–	–	–	amylase	CBM34 **GH13**
Amy 4	1357–1356	1528	**+**	**+**	**+**	**+ (2)**	–	amylase	SP **GH13** CBM26 CBM26 Coh Doc
Amy 5	565–551	554	**+**	–	–	–	–	amylase	SP **GH13**
Amy 6	512–511	510	–	–	–	–	–	amylase	**GH13**
Amy 7^a^	428–427	–	–	–	–	–		amylase	**GH13**
Amy 8	565–552	545	–	–	–	–	–	amylase	**GH13** DUF
Amy 9	1064–1056	801	**+**	–	**+**	**+ (1)** ^b^	–	amylase	SP **GH13** CBM26 Doc
Amy 10	1234–1233	1333	**+**	–	**+**	**+ (2)** ^c^	**+ (1)**	pullulanase	SP CBM48 **GH13** MucBP MucBP CBM26 MucBP Doc CBM26
Amy 11	941–940	961	–	–	–	–	**+ (1)**	pullulanase	CBM48 **GH13** MucBP MucBP MucBP
Amy 12	1060–1059	1059	**+**	–	**+**	**+ (1)**	**+ (1)**	pullulanase	SP CBM48 **GH13** MucBP Doc MucBP CBM26
Amy 13	641–639	632	–	–	–	–	**+ (1)**	glycogen	CBM48 **GH13**
Amy 14	767–762	717	–	–	–	–	**+ (1)**	glycogen	CBM48 **GH13**
Amy 15	695–694	695	–	–	–	–	**+ (1)**	glycogen	CBM48 **GH13**
Amy 16	877–867	995	**+**	–	**+**	**+ (2)** ^c^	–	amylase	SP **GH13** CBM26 Doc CBM26
Amy 17	569–556	665	**+**	–	–	**+ (1)** ^d^	–	amylase	SP **GH13**

**a.** Amy3 and Amy7 were absent in rumen strain YE282.

**b.** absent in YE282.

**c.** 3 modules present in YE282.

**d.** only present in YE282.

SP, signal peptide; CBM, carbohydrate binding module; GH, glycoside hydrolase, DUF, domain of unknown function; MucBP, Mucin binding protein.

The four human *R. bromii* genomes, but not the rumen YE282 strain, encode multiple copies of a novel family of accessory (‘X’) module closest in sequence to CBM37 (Xu *et al*., 2004) (Supporting Information Table S2). In contrast to *R. albus* CBM37‐like modules (Xu *et al*., 2004), none of the *R. bromii* X modules was found in association with GH domains, cohesins or dockerins. Furthermore, bioinformatic analysis of 50 X modules from *R. bromii* strains revealed that they belong to a phylogenetic clade distinct from the CBM37 modules of *R. albus* (Supporting Information Fig. S2). Several of these new *R. bromii* X modules are adjacent to domains annotated as invasin/intimin cell adhesion fragments, cell wall hydrolase/autolysins and a L,D‐transpeptidase.

### New insights into amylosome structure and conservation of amylosome components

The formation of enzyme complexes (amylosomes) in *R. bromii* L2–63 was previously inferred from the interactions of dockerin modules present in four extracellular GH13 enzymes with cohesin modules found in four scaffoldin proteins (Ze *et al*., 2015). The present analysis identifies a fifth dockerin‐containing GH13 enzyme, Amy16, and shows that the same five dockerin‐carrying enzymes (Amy4, Amy9, Amy10, Amy12 and Amy16) are encoded by all five *R. bromii* strains (Table 1, Supporting Information Data File S2). Not only the GH13 sequences, but also the size and modular organization of these five enzymes show remarkable conservation between all five strains. Notably, the Amy4 enzyme carries both a dockerin and cohesin module in all strains, although the YE282 enzyme differs in carrying two cohesins and there is some variation also in the number of CBM26 modules carried. Comparison of the activities of the Amy16, Amy4 and Amy9 enzymes from *R. bromii* L2–63 following expression in *E. coli* showed that they acted as α–(1,4)‐ amylases with no activity against pullulan, indicating a lack of activity against α‐(1,6)‐linked glucose residues (Supporting Information Fig. S3). In contrast, recombinant Amy10 and Amy12 enzymes act as pullulanases that are more active against pullulan than against glycogen and show minimal activity against α‐(1,4) linkages.

**Figure 3:**
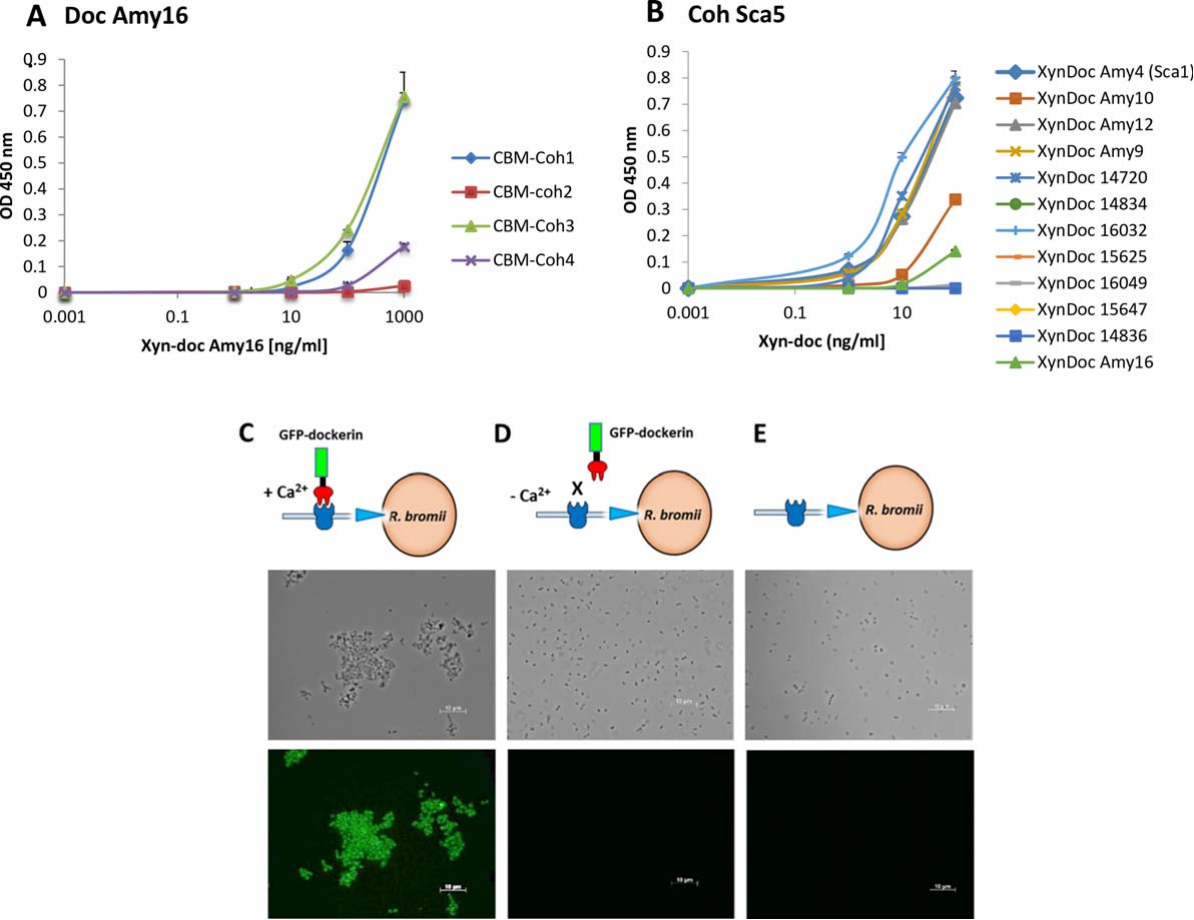
Cohesin‐dockerin binding measured by ELISA. A. A microtiter plate was coated with XyndocAmy16. Positive interactions of the Amy16 dockerin were observed with coh1 and 3; coh1, 2, 3 and 4 are from Sca1 (Amy4), 2, 3 and 4, respectively. B. ELISA plates were coated with CBM‐Coh from Sca5. Positive interactions of the cohesin from Sca5 were observed with Xyndocs 14720, 16032, Amy4, Amy12 and Amy9, low interaction was also observed with Xyndoc Amy10. (Please note that to allow comparison the CBL numbering for Xyndocs given here is consistent with that given previously (Ze *et al.*, 2015) and does not refer to the L2–63 genome re‐annotation). Error bars indicate SD from the mean of duplicate samples from one experiment. C. Recombinant gfp‐Amy4doc protein incubated with EDTA‐pretreated, washed *R. bromii* L2–63 cells in the presence of Ca^2+^. (D) as (C) but in the absence of Ca^2+^. E. *R. bromii* L2–63 cells in the absence of gfp‐amy4doc.

**Figure 4:**
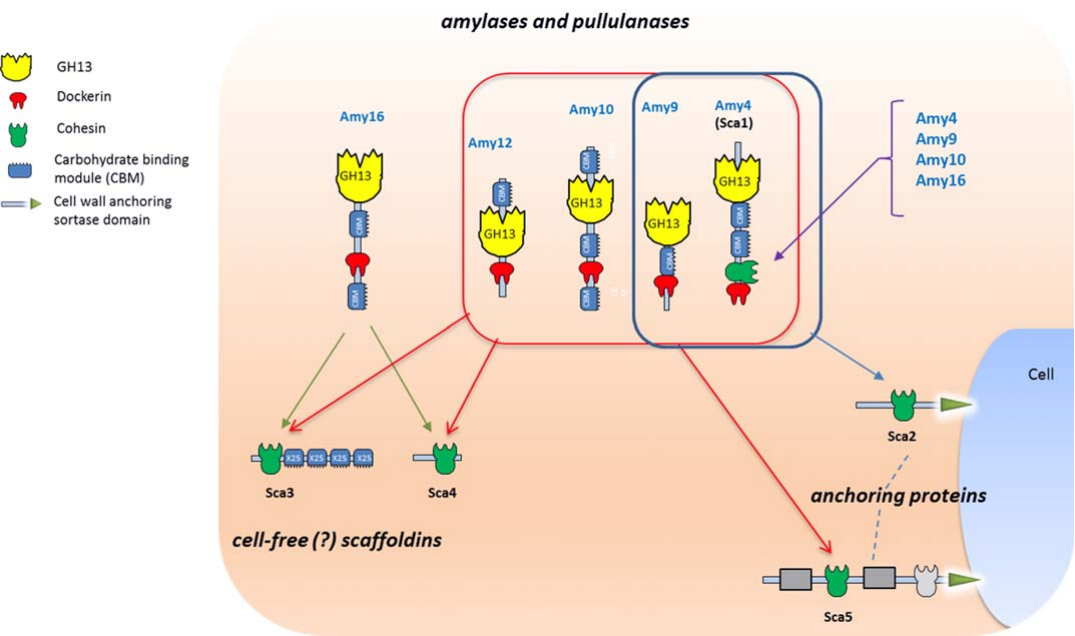
Updated model for cell‐bound and cell‐free amylosome complexes in *R. bromii* L2–63. The Amy4 and Amy9 enzymes are likely to bind to the cell surface via the Sca2 scaffoldin protein. Amy4 has then the potential to self‐aggregate through interactions between its own cohesin and dockerin or to integrate Amy9, Amy10 or Amy16. Further complexes are likely to form between the Amy4, Amy9, Amy10, Amy12, Amy16 and either the Sca3 or Sca4 proteins to form cell‐free amylosomes. In addition, Amy4, Amy9, Amy10, Amy12 can be integrated to the first cohesin of Sca5 and attached to the cell‐surface. The binding specificities of cohesin modules of the second Sca5 cohesin (shown in light grey) have yet to be determined.

A fifth scaffoldin, Sca5 was also detected here in the re‐annotated L2–63 genome. This comprises two similar modules (51% amino‐acid identity) of unknown function, two cohesins and a C‐terminal sortase signal and therefore represents a second potential mode of anchorage, along with Sca2 (Ze *et al*., 2015), to the bacterial cell surface. Bioinformatic analysis (BLASTP) using L2–63 cohesins and dockerins as queries showed that five similar scaffoldin proteins are present in all five strains (Supporting Information Fig. S4). A cohesin from *R. bromii* L2–63 Sca5 was expressed as a fusion protein in *E. coli* to determine its interactions with selected dockerins. The Sca5 cohesin bound strongly to dockerins from Amy4, Amy12 and Amy9, but only weakly to those from Amy10 and Amy16 (Fig. 3A and B). The Amy16 dockerin did however exhibit strong binding activity for the cohesins from Amy4 (Sca1) and Sca3 (Fig. 3A and B). We also investigated the interaction of a GFP‐tagged dockerin from *R. bromii* L2–63 Amy4 (GFP‐doc‐13a) with *R. bromii* L2–63 cells. Since dockerin:cohesin interactions require calcium, cells were first treated with EDTA to disrupt protein complexes; subsequent incubation with GFP‐doc‐13a showed binding in the presence but not in the absence of calcium (Fig. 3C, D, and E), thus strongly supporting the hypothesis that binding of Amy4 to the cell surface occurs through dockerin:cohesin interactions. Based on these new data we can now propose a comprehensive scheme for the *R. bromii* L2–63 amylosome that includes Amy16 and a presumed role for the Sca5 scaffoldin whose C‐terminal sortase signal indicates that is attached to the cell surface (Fig. 4).

**Figure 5:**
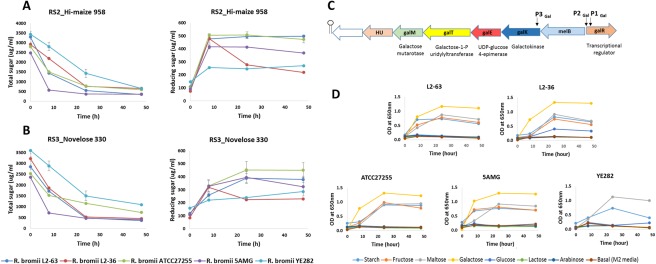
Carbohydrate utilization in *R. bromii* strains. Total sugar utilized and the concentration of free soluble reducing sugar (glucose equivalents) are shown after 48 h incubation in M2 medium containing (A) RS2 (High‐maize 958) or (B) RS3 starch (Novelose 330). Data plotted in graphs are the mean ± SD OD readings (OD_490_ for total sugar assay and OD_415_ for reducing sugar assay) of three biological replicates and three technical replicates for each time‐point studied for each strain. (C) Organization of the galactose operon in the human *R. bromii* strains (P indicates likely promoters and the hairpin indicates the likely transcriptional terminator). (D) Growth of the five *R. bromii* strains with soluble potato starch, maltose, fructose, galactose, lactose, glucose and arabinose (0.2% w/v) as sole sources of carbon. Data plotted in graphs are the mean OD_650_ readings of three replicates of each strain grown on different sugar substrates. As the SD values were very small in all cases they were not plotted on to the graph.

We also compared the ability of *R. bromii* strains to utilize RS. The genomes of all five strains indicated a very limited capacity for vitamin synthesis, with only niacin predicted to be synthesized (Magnúsdóttir *et al*., 2015). For this reason the growth tests were carried out in rumen fluid (M2) medium which was used for the initial strain isolations (Herbeck and Bryant, 1974; Ze *et al*., 2015). Consistent with the conservation of their amylase systems, the five *R. bromii* strains showed a similar ability to utilize pre‐boiled resistant starches. The disappearance of total sugar after 48 h incubation ranged from 77% to 89% for RS2 (mean 82.4%) and from 71% to 87% for RS3 (mean 78.9%) (Fig. 5A and B). These figures slightly underestimate the extent of degradation since there was some accumulation of unabsorbed reducing sugar, as noted previously for *R. bromii* L2–63 (Ze *et al*., 2012).

### Capacity for sugar utilization by *Ruminococcus bromii* strains

The five *R. bromii* genomes encode limited sets of carbohydrate transporters. An ABC transport system predicted to take up maltose and maltodextrins, the major products of starch hydrolysis, is encoded by genes upstream of the Amy5 amylase (Supporting Information Fig. S5). Separately, linked *malP* and *malQ* genes encode maltodextrin phosphorylase and a GH77 glucanotransferase, suggesting that maltose is metabolized via a phosphorylase/glucanotransferase cycle similar to that in *E. coli* (Boos and Shuman, 1998). A fructose‐specific phosphotransferase gene cluster (Supporting Information Fig. S5) and a galactose utilization gene cluster including a Na^+^ galactoside symporter (melB) are also present in the four human *R. bromii* genomes (Fig. 5C). The ability to grow with galactose as sole energy source, not previously identified in this species, was confirmed experimentally, while only strain L2–36 grew with glucose as sole energy source (Fig. 5D). In contrast, the genome of the rumen strain YE282 lacks a close homologue of *melB* and carries an incomplete fructose utilization cluster. YE282 grew well on maltose and starch but failed to utilize any of the monosaccharides tested suggesting that it must rely solely on the uptake of oligosaccharides (Fig. 5D).

### Sporulation, spore survival and germination

**Figure 6:**
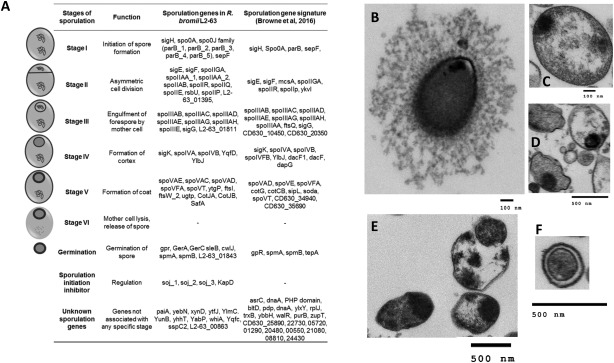
Sporulation gene signature and TEM of *R. bromii* sporulation. (A) A set of 65 genes with known sporulation function were detected in the genome of *R. bromii* L2–63 strain. These genes corresponded to: initiation of spore formation (Stage I), asymmetrical division into a larger mother cell and a smaller forespore (Stage II), engulfment of the forespore by the mother cell (Stage III), formation of the spore cortex (Stage IV), deposition of the spore coat (Stage V) followed by lysis of the mother cell and release of the endospore (Stage VI). A set of 8 genes related to germination of the endospore were also detected. Spo0A, which is a critical transcription factor to initiate sporulation and the specialized sporulation sigma factors (σH, σF, σE, σG and σK; small proteins that direct RNA polymerase to specific sites on DNA to initiate gene expression) along with the regulators of these sigma factors (such as spoIIAA, spoIIGA and the spoIIIA operon) were also present. Key regulatory proteins involved in chromosome partitioning, belonging to the soj (parA_1, parA_2, parA_3,) and spo0J (parB_1, parB_2, parB_3, parB_4, parB_5) families were also detected. In the absence of spoJ, soj is known to negatively regulate expression of several sporulation genes by binding to the promoter regions and inhibiting transcription indicating a tightly regulated energy‐intensive process for survival. Comparison with core sporulation and germination related genes from *Clostridium difficile* strain 630 and *Bacillus subtilis* strain 168 described recently by Browne and colleagues (2016) showed that all the key sporulation genes were present. (B) TEM image of a *R. bromii* L2–63 strain after 72 h growth on M2S medium. (C and D) Endospores were visible from *R. bromii* cells (E) Release of endospores from *R. bromii* after lysis of the mother cell. (F) Endospores released into the surrounding medium.

The *R. bromii* L2–63 genome encodes 73 genes with inferred functions in sporulation and spore germination. These include the key regulator Spo0A, along with sporulation sigma factors (σH, σF, σE, σG and σK) and their regulators [the spoIIAA, spoIIGA and spoIIIA operons (Hutchison *et al*., 2014)] and regulatory proteins (soj and spo0J families) involved in chromosome partitioning (Quisel and Grossman, 2000) (Fig. 6A, Supporting Information Data File S2). These 73 genes were also detected in *R. bromii* L2–36 and their products were identical in sequence. *R. bromii* 5AMG, ATCC27255 and YE282 lack 3, 8 and 6 of these genes and the corresponding gene products share 98%–100%, 81%–100% and 30%–98% amino acid identity, respectively, with L2–63 homologues (Supporting Information Data File S2). The majority of core sporulation genes described recently in *Clostridium difficile* strain 630 and *Bacillus subtilis* strain 168 (Browne *et al*., 2016) are present in the five *R. bromii* genomes.

We tested the sporulation capabilities of *R. bromii* L2–63 strain by subjecting cultures pre‐grown for 72 h in M2S liquid medium to heat inactivation (80°C for 20 min) and exposure to air. Restoration of anaerobic conditions resulted in good growth (OD_650_ > 0.6) after 24 h incubation in liquid M2S medium containing 0.1% sodium taurocholate. This indicated germination of functional spores that had survived these conditions and 16S rRNA gene sequencing confirmed the identity of the re‐grown cultures as *R. bromii*. *R. bromii* endospores were detected by TEM in images obtained after heat treatment of 72 h and 30 day old cultures. *R. bromii* cells showed diffuse cell surface features after 72 h growth in liquid M2S medium (Fig. 6B), whereas endospore‐like structures were detected in cells grown for 30 days (Fig. 6C and D) along with release of endospores from *R. bromii* cells (Fig. 6E and F).

### Indications of horizontal gene transfer

An 8‐gene vancomycin resistance cluster (Depardieu *et al*., 2003) was detected in *R. bromii* L2–63 and L2–36 but not in 5AMG, ATCC27255 or YE282 (Supporting Information Fig. S6AB). All four human *R. bromii* strains were however fully inhibited by vancomycin at 1 μg/ml, while growth on 0.4 μg/ml vancomycin was similar for all five strains, showing that resistance was not expressed (Supporting Information Fig. S6C). The regions flanking the VanG cluster contain genes (XerC site‐specific recombinase, TraC, VirB4 and VirD4 Type IV secretion) that suggest acquisition through conjugative‐transposon‐like elements. Unambiguous CRISPR arrays were found in three of the 5 *R. bromii* strains (ATCC27255, 5AMG and YE282) and possible CRISPR structures in all 5 strains. CRISPR structures are also prevalent in related *Ruminococcus* species (Berg Miller *et al*., 2012; Wegmann *et al*., 2014).

## Discussion

The organization of carbohydrate‐degrading enzyme systems and transporters in polysaccharide‐utilizing micro‐organisms appears fundamental to their niche specificity and to their competitive ability within the microbial community (Flint *et al*., 2008). In *R. bromii* the unique organization of starch‐degrading enzymes into extracellular amylosome complexes is proposed to underpin the exceptional ability to degrade dietary resistant starches (Ze *et al*., 2012, 2013). We have now shown that *R. bromii* L2–63 encodes five dockerin‐carrying amylases and five cohesin‐carrying scaffoldins (one of which, Amy4, is also an amylase) that participate in these complexes. Two scaffoldins (Sca2, Sca5) carry C terminal sortase signals likely to mediate attachment to the cell surface and a gfp‐tagged dockerin from Amy4 (that recognizes cohesins in Sca2 and Sca5) was shown here to bind *R. bromii* L2–63 cells in a calcium‐dependent manner. Our evidence suggests the formation of alternative cell surface and cell‐free scaffoldin‐based amylase complexes (Fig. 4).

We find that the modular organization of the five dockerin‐carrying amylases and of the five scaffoldin proteins is very highly conserved across the five available *R. bromii* strains. These include human strains from the USA (ATCC27255), Ecuador (5AMG) and the UK (L2–63, L2–36), but also a rumen strain from Australia (YE282). While the rumen strain shows the most divergence, its amylosome components nevertheless share the main features found in their homologues in the human strains. This degree of similarity points to the likelihood of an early evolutionary origin for such complexes among specialist starch‐utilizing bacteria that colonized the animal gut that predates acquisition of these bacteria by humans. Starch present naturally in the diets of hind‐gut fermenting animals will be in the raw state, making it more resistant to the action of mammalian amylases than is starch present in cooked foods consumed by modern day humans. In pre‐gastric fermenting ruminants, raw starch present in ingested feed is exposed only to salivary amylases before entering the microbial community of the rumen. Thus the evolution of sophisticated microbial systems capable of degrading a wide range of starches including raw starch particles in the animal gut may well explain the high particulate starch‐degrading capacity of human *R. bromii* strains. The significance of fructose and galactose utilization by the human strains, although not in the rumen strain, is unknown, but suggests that these sugars might provide alternatives to starch as an energy source in the human colon. Some *Ruminococcus* strains from the rumen have been shown to possess an unusual transformation mechanism mediated by membrane vesicles that also contain cellulosome complexes (Klieve *et al*., 2005). In view of this the possibility that horizontal gene transfer might be mediated by amylosome‐containing membrane vesicles in *Ruminococcus bromii* deserves investigation.

Until recently, most of the predominant anaerobic gut bacteria found in gut communities were regarded as ‘non‐sporing’. The detection of large sets of sporulation genes in many of these species (Browne *et al*., 2016) has however called this assumption into question. We have shown here that all five *R. bromii* strains possess a complete set of genes required for spore formation and spore germination. In the presence of taurocholate as an inducer of spore germination (Browne *et al*., 2016), *R. bromii* L2–63 was able to recover from 80°C heat treatment and exposure to air, conditions that kill vegetative cells but are resisted through spore formation. Spore formation has profound consequences for gut microbial ecology as it may help to explain the acquisition of this strictly anaerobic species in human infants around the time of weaning. Sporulation must also increase the potential for acquisition of strains from environmental sources, even in adults; thus although *Ruminococcus* spp. are strongly associated with gut communities rather than non‐host‐associated environments (La Reau *et al*., 2016) this does not preclude their transmission between hosts via spores of faecal origin. It has been proposed that for human populations in many industrialized countries gut microbiota diversity is decreasing with each generation as members of more diverse (typically rural) microbial communities are lost (Bokulich *et al*., 2016). The transmissibility of key anaerobic species between hosts appears crucial in assessing this hypothesis.

As one of the most dominant bacterial species in the human colon, *R. bromii* has the potential to influence host physiology and health via primary and secondary metabolite production (Donia *et al*., 2014) and via interactions with the immune system. Furthermore since we know that diet composition has a profound impact on the population of this nutritionally specialized species, it is essential to gain a better understanding of its impact on the host. This has to start with better understanding of the microbial ecology, physiology and genomics of this little studied bacterium and this work represents an important step towards this.

## Experimental procedures

### Bacterial isolates and growth medium

Human *R. bromii* isolates include ATCC2755 (Moore *et al*., 1972) and two strains (L2–63 and L2–36) isolated from a healthy male UK child (Barcenilla, 1999; Ze *et al*., 2012) with approval from Grampian Research Ethics (project no 00/00133). Strain 5AMG strain was isolated at the Rowett from a faecal sample of a healthy individual from rural Ecuador. The rumen strain YE282 was isolated from a steer fed on high grain diet from Australia (Klieve *et al*., 2007) Strains were routinely maintained in M2GSC medium containing 30% clarified bovine rumen fluid and 0.75% agar (Miyazaki *et al*., 1997) in Hungate tubes (7.5 ml aliquots). M2 medium supplemented with 0.2% carbohydrate (Miyazaki *et al*., 1997) was used for growth tests based on OD_650_ of triplicate cultures. M2S medium contained 0.2% soluble potato starch.

### Resistant starch utilization

Hi‐maize 958 is a natural RS2 corn starch and Novelose 330 a retrograded RS3 corn starch (Ze *et al*., 2012) Raw starch solutions (2% wt per vol) were boiled for 10 min before addition to M2 medium (final concentration 0.2%). Total sugar was determined by the phenol sulfuric acid assay (Dubois *et al*., 1956) and reducing sugar according to (Lever, 1977).

### Sequencing, assembly and annotation of *Ruminococcus bromii* draft genomes

Overnight cultures of *R. bromii* human strains were centrifuged at 14 000 X g for 10 min and genomic DNA extracted from cell pellets using FastDNA® Spin Kit for Soil (MP Biomedicals, Cambridge, UK). Sequencing of the *R. bromii* L2–63 genome was done at the Wellcome Trust Sanger Institute, Cambridge UK, by the Pathogen Genomic group (http://www.sanger.ac.uk/resources/downloads/bacteria/metahit/) and of the *R. bromii* YE282 genome at the U.S. Department of Energy Joint Genome Institute (DOE JGI, California) as part of the Hungate 1000 project. These sequences were publicly available. The *R. bromii* L2–63 genome was re‐annotated using Prokka (Seemann, 2014) which resulted in an increase of 14.2% in identified coding sequences (CDS) to a total of 2111 compared with 1811 reported previously (Ze *et al*., 2015).

*R. bromii* strains L2–36, 5AMG and ATCC2755 were newly sequenced at the Earlham Institute (Norwich, UK), using Illumina HiSeq (Illumina, San Diego, CA, USA) generating paired end reads with read lengths of 50 bp. Libraries were prepared from genomic DNA using the KAPA high throughout Library Prep Kit (Part No: KK8234). Genomic DNA sonicated to an average size of 500 bp, end repaired, tailed, adaptor‐ligated, fractionated, purified and enriched. The constructed libraries were normalized and equimolar amounts pooled into one final pool of 7.7 nM (8.1 nM for strain 5AMG) using elution buffer (Qiagen). The library pools were then diluted, transferred into a 200 μl strip tube, spiked with 1% PhiX Control and placed on ice before loading onto the Illumina cBot. Template hybridization and first extension was carried out on the cBot utilizing the TruSeq Rapid PE Cluster Kit v1 or HiSeq Rapid PE Cluster Kit v2 prior to the flowcell being transferred onto the HiSeq2500 for the remainder of the clustering process. The sequencing chemistry used was TruSeq Rapid SBS Kit v1 or HiSeq Rapid SBS Kit v2 using HiSeq Control Software 2.2.58 and RTA 1.18.64. The library pool was run in a single lane for 50 cycles of each paired end read. Reads in bcl format were de‐multiplexed based on the 6 bp Illumina index by CASAVA 1.8, allowing for a one base‐pair mismatch per library, and converted to FASTQ format by bcl2fastq.

The raw data reads were aligned against the *R. bromii* L2–63 genome using the mapping tool Bowtie2 (version 2.2.6). De‐novo assembly of the three *R. bromii* strains used ABYSS (version 1.9.0). Assemblies were annotated using Prokka (version1.7.2)(Seemann, 2014) and employing Pfam (Punta *et al*., 2012), Prosite (Sigrist *et al*., 2010) and RNAmmer (Lagesen *et al*., 2007) to identify protein families and functional protein sites and rRNA.

### Data availability

This Whole Genome Shotgun project has been deposited at DDBJ/ENA/GenBank under the accession numbers NPHY00000000 (*R. bromii* L2–36 strain), NNSR00000000 (*R. bromii* ATCC27255 strain) and NNBY00000000 (*R. bromii* 5AMG strain). The version described in this article is version NPHY01000000, NNSR01000000 and NNBY01000000 respectively.

### Genome analysis

Blast Ring Image generator (BRIG) software was used for genome comparisons (Alikhan *et al*., 2011). Pangenome analysis used BPGA software Version 1.3 (Chaudhari *et al*., 2016). Orthologous genes (OGs) present in all five *R. bromii* genomes were defined as core genes, sequences with orthologues present in 2–4 strains were considered as variable or accessory genes and sequences that were found only in one strain were considered unique genes. Analysis of the Carbohydrate‐active enzymes (CAZymes) relied on the CAZY website (http://www.cazy.org/) supplemented by Hidden Markov models (Sheridan *et al*., 2016). Cohesin and dockerin sequences were predicted using the BLASTP and tBLASTn algorithm (Altschul *et al*., 1997) utilizing known dockerin and cohesin query sequences. Hits of *E*‐value < 10^−4^ were individually examined. Signal Peptide sequences were predicted using the SIGNALP server (http://www.cbs.dtu.dk/services/SignalP /). ClustalW was used for multiple sequence alignments and phylogenetic trees constructed by the maximum‐likelihood method, using MEGA7.0 software (Kumar *et al*., 2016). Promoter sequences were identified using BPROM (http://www.softberry.com) and Rho‐independent transcription terminators predicted using ARNold (http://rna.igmors.u-psud.fr/toolbox/arnold/index.php). Antibiotic resistance genes were analysed through the Comprehensive Antibiotic Resistance Database (CARD) (version 1.0.3) using BLASTP (McArthur *et al*., 2013). Clustered Regularly Interspaced Short Palindromic Repeats (CRISPR) arrays were detected by CRISPRFinder web tool (Grissa *et al*., 2007).

### Expression of cohesins and dockerins in *E. coli*

Cloning of the xylanase fused‐dockerin from Amy16, GFP fused‐dockerin from Amy4 (GFP‐doc‐13a) and CBM‐fused Sca5 cohesin, expression in *E.coli* BL21 (DE3) cells and purification of the recombinant proteins were performed following previously published protocol (Barak *et al*., 2005). Primers used in this study are listed in Supporting Information Table S3.

### Expression of *R. bromii* amylases in *E. coli*

Full length Amy4, Amy9 and Amy16 sequences were amplified from genomic DNA using the primers listed in Supporting Information Table S3 and cloned into the vectors indicated. Following transformation into *E. coli* BL21 (DE3) and over‐expression (Ben David *et al*., 2015), 6His‐tagged products were purified and their activities against alpha‐glucan substrates (Supporting Information Fig. S3AB) assayed by reducing sugar release (Ze *et al*., 2015). The Amy10 and Amy12 sequences were amplified from genomic DNA to express the soluble portions of the proteins minus the signal peptides or dockerin sequences. These gene fragments were cloned into the LIC vector pETite‐Chis (Lucigen, Madison, WI) to include a 6His tag at the C‐terminus as well as a TEV cleavage site. The proteins were expressed in Rosetta (DE3) pLysS cells, purified via Ni^2+^‐affinity chromatography, and the 6His tag removed via TEV using methodology previously described (Cockburn *et al*., 2015). Activity on 0.3% or 0.075% pullulan, and 0.3% glycogen was measured in triplicate (Supporting Information Fig. S3C). For activity assays with polysaccharide substrates the production of free reducing ends was monitored using the bicinchoninic acid (BCA) method (Waffenschmidt and Jaenicke, 1987; Cockburn *et al*., 2015). Assay substrates were: potato starch (Sigma S2004); corn starch (Sigma S4180); glycogen (Sigma G8751); Novelose 330 (National Starch & Chemical); pullulan (Megazyme).

### Binding assays

Cohesin‐dockerin binding was measured by ELISA (Barak *et al*., 2005). The interaction between the GFP‐tagged Amy4 dockerin (GFP‐doc‐13a) protein and *R. bromii* cells was assessed using 24 h cultures of *R. bromii* L2–63 grown in M2S medium (OD_650_=0.6). After centrifugation the cell bacterial pellet was washed with sterile PBS and divided into three separate tubes. In the first tube, the pellet was re‐suspended in PBS, treated with 10 mM EDTA and washed with PBS three times. Cells were then treated with GFP‐doc‐13a protein (40 μl) along with 10mM calcium and incubated for 1 h at room temperature on a rotating platform. The cells were then washed with PBS three times to eliminate any unbound protein, centrifuged at 5000 rpm for 10 min, re‐suspended in PBS, smeared on microscope slides, air‐dried, overlaid with Vectashield anti‐fade mounting Medium (Vector Laboratories, Peterborough, UK) and examined with a Zeiss Axio Observer Z1 inverted fluorescent microscope (Carl Zeiss, Cambridge, UK) using a Zeiss 10 FITC filter. The *R. bromii* pellet from the second tube was treated exactly as the first tube but without the addition of calcium. The third tube was a control where the bacterial pellet was simply washed in PBS.

### Spore germination assay

*R. bromii* L2–63 cultures grown for 72 h were heated to 80°C for 20 min to kill vegetative cells. Subsequently, the cells were exposed to air, pelleted by centrifugation, washed in sterile PBS and used to inoculate fresh M2S medium containing 0.1% sodium taurocholate. Inoculated tubes were incubated anaerobically at 37°C and OD_650_ checked after 24 h. PCR amplification and sequencing of the full‐length 16S rRNA gene was used to confirm species identity. Sporulation images of *R. bromii* strains cultured for 72 h and 30 days were captured using transmission electron microscopy (TEM) (Lawley *et al*., 2009).

## Author Contributions

HJF, NJ, WK devised the study. IM, JLG, SM performed the experiments. IM, POS, SM, JLG, HJF, EAB analysed the data and prepared figures and tables. SHD, PL, AK, DO, WK, AWW, EC, PC, CS, NK, RK, DC provided critical resources and support. HJF, IM, EAB, SM wrote the paper. All authors read and approved the final manuscript.

## Supporting information

Additional Supporting Information may be found in the online version of this article at the publisher's web‐site:

**Fig. S1.** Pan genome analysis of the five *R. bromii* genomes. (A) Phylogenetic analysis using BPGA software based on concatenated core gene alignments. (B) COG distribution of core, accessory and unique genes of *R. bromii* strains as deduced by the pan‐genome analysis.

**Fig. S2.** Phylogenetic tree of CBM37 modules from *R. albus* and ‘X’ modules from *R. bromii*.

**Fig. S3.** Activities of recombinant enzymes expressed in *E. coli*.

**Fig. S4.** Phylogenetic tree comparing the 30 cohesin modules from *R. bromii* L2‐63, L2‐36, 5AMG, ATCC27255 and YE282 strains.

**Fig. S5.** Carbohydrate transport/utilization gene clusters in *R. bromii* genomes. Organization of (A) maltose and fructose transport systems and (B) glycogen biosynthesis gene cluster for the human *Ruminococcus bromii* L2‐63, L2‐36, ATCC27255 and 5AMG strains.

**Fig. S6.** Potential vancomycin resistance gene cluster in *R. bromii* L2‐63. (A), (B) This VanG cluster is present in *R. bromii* L2‐63 and L2‐36, but not in the other three genomes. However, we found no evidence for resistance to vancomycin in the two human strains that possess the VanG cluster compared with the two that lack the cluster (C).

**Table S1.** *Ruminococcus bromii* genome assembly statistics.

**Table S2.** Distribution of GH, dockerins, cohesins and CBM modules in human and rumen *Ruminococcus bromii* strains

**Table S3.** List of primers used for the different constructs produced.

**Data File S1.** List of core, accessory and unique genes from pan‐genome analysis of 5 *R. bromii* strains.

**Data File S2.** List of locus tag numbers for amylase scaffoldin and dockerin genes for *R. bromii* L2‐63 strain before and after re‐annotation of the genome (Worksheet1), list of sporulation genes detected in *R. bromii* L2‐63 strain (Worksheet 2), conservation of L2‐63 sporulation genes in 4 other *R. bromii* strains (Worksheet 3), comparison of spore signature genes described by Browne *et al*. with *R. bromii* strains (Worksheet 4).
