# Supplementary material for: Sporulation capability and amylosome conservation among diverse human colonic and rumen isolates of the keystone starch‐degrader Ruminococcus bromii
Source: Environ Microbiol. 2017 Dec 7;20(1):324–36. doi: 10.1111/1462-2920.14000 (PMC5814915; doi:10.1111/1462-2920.14000)

Figure S1: Pan genome analysis of the five *R. bromii* genomes. A) Phylogenetic analysis using BPGA software based on concatenated core gene alignments. B) COG distribution of core, accessory and unique genes of *R. bromii* strains as deduced by the pan-genome analysis. The COG distribution pattern showed involvement of more core genes in translation, ribosomal structure and biogenesis, carbohydrate/amino-acid/nucleotide transport and metabolism, while accessory and unique genes appeared to be enriched in transcription, replication, recombination, repair and cell wall/membrane/envelope biogenesis and defence related functions.

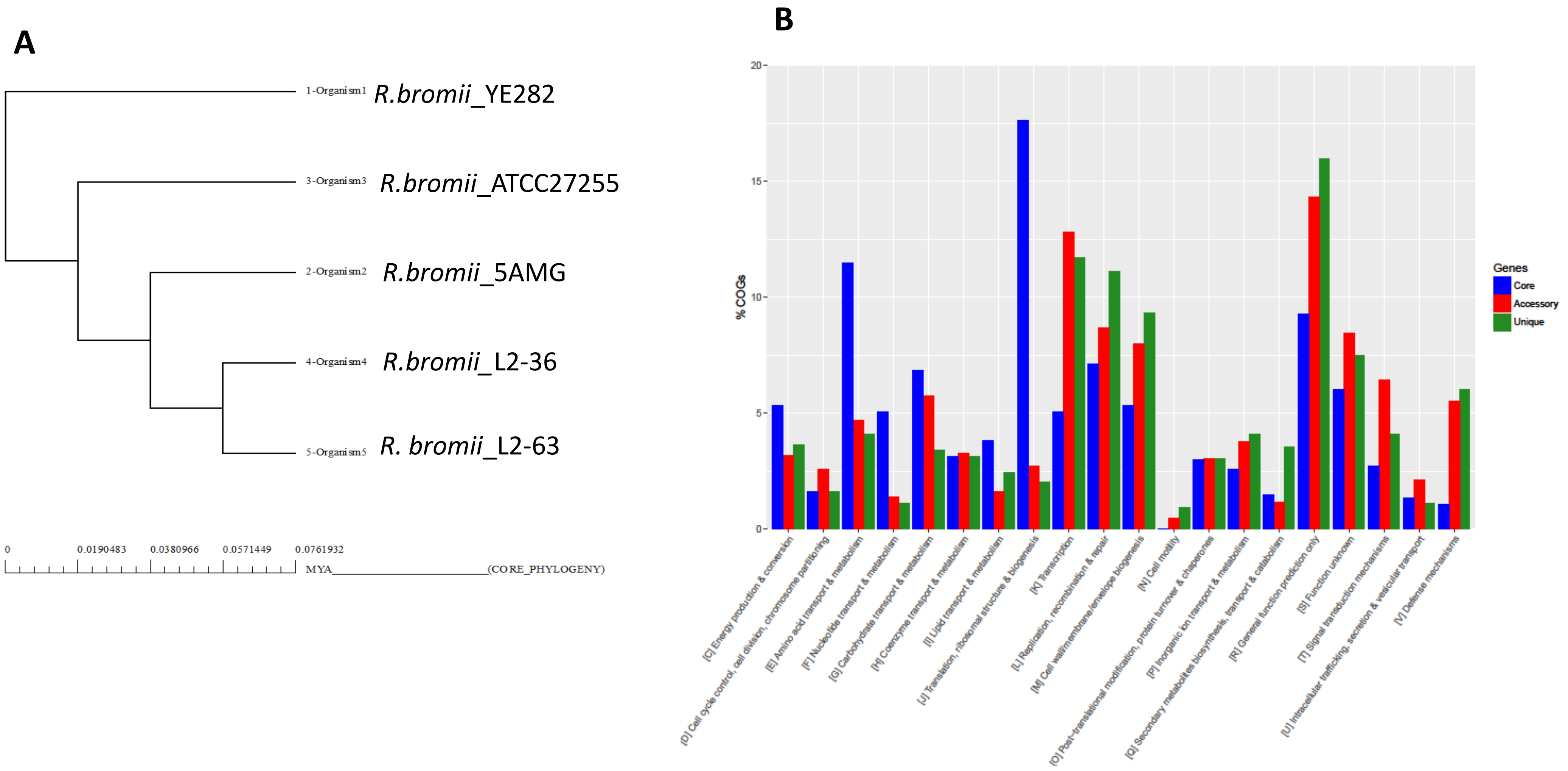

Supplement: Supplementary file 1 — Fig. S1. Pan genome analysis of the five R. bromii genomes. (A) Phylogenetic analysis using BPGA software based on concatenated core gene alignments. (B) COG distribution of core, accessory and unique genes of R. bromii strains as deduced by the pan‐genome analysis. [file EMI-20-324-s001.pdf]
