# Supplementary material for: Sporulation capability and amylosome conservation among diverse human colonic and rumen isolates of the keystone starch‐degrader Ruminococcus bromii
Source: Environ Microbiol. 2017 Dec 7;20(1):324–36. doi: 10.1111/1462-2920.14000 (PMC5814915; doi:10.1111/1462-2920.14000)

Figure S2. **Phylogenetic tree of CBM37 modules from *R. albus* and ‘X’ modules from *R. bromii*.**
We observed that the *R. bromii* ‘X’ modules were on a different phylogenetic clade (cyan clade) than the classical CBM37 from *R. albus* (orange and red clades). In the cyan clade, 49 ‘X’ modules were all clustered together, suggesting a potential new function for these modules. Bootstrapping confidence values higher than 0.6 are shown.

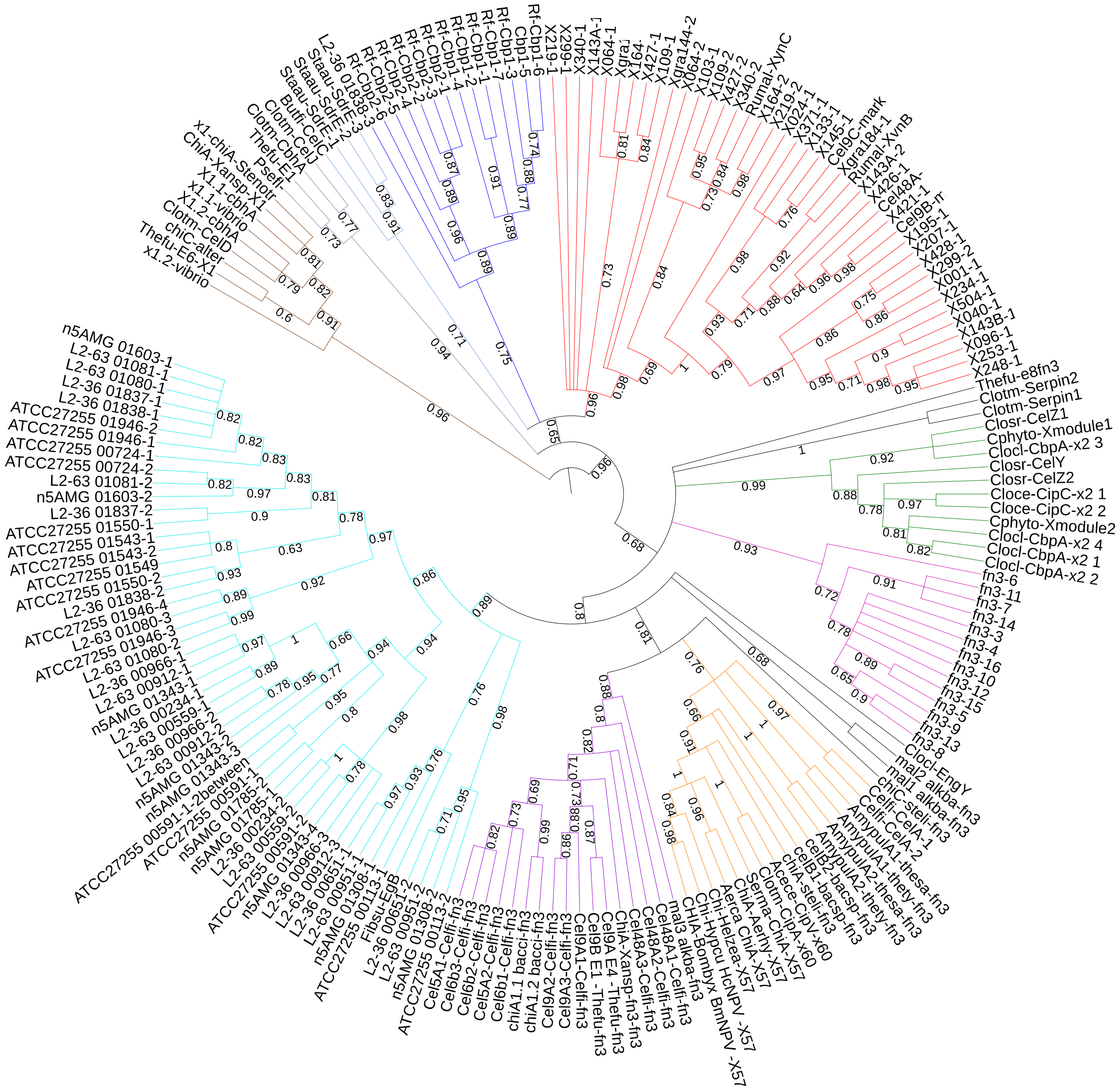

Supplement: Supplementary file 2 — Fig. S2. Phylogenetic tree of CBM37 modules from R. albus and ‘X’ modules from R. bromii. [file EMI-20-324-s002.pdf]
