# Supplementary material for: Sporulation capability and amylosome conservation among diverse human colonic and rumen isolates of the keystone starch‐degrader Ruminococcus bromii
Source: Environ Microbiol. 2017 Dec 7;20(1):324–36. doi: 10.1111/1462-2920.14000 (PMC5814915; doi:10.1111/1462-2920.14000)

Figure S3. Activities of recombinant enzymes expressed in *E. coli*. Sequences encoding full length gene products were amplified from *R. bromii* L2-63 genomic DNA and cloned into expression vectors followed by expression in *E. coli* (Table S3). Overexpressed enzymes carrying His_6 tags were purified and assayed for their activity (release of reducing sugar) against different starch substrates, as described in the Materials and Methods (except for the Amy9 enzyme which lacked a His_6 tag, where activities refer to cell lysates).

a) Comparison of activities for Amy4, Amy9 and Amy16 against corn starch, Novelose 330 and glycogen relative to the activity of each enzyme against potato starch. Substrates were pretreated by boiling for 10 minutes. Activities are based on 3h incubation for Amy16 or 6h incubation for Amy4 and Amy9. Standard deviations for triplicate readings for single time points were < 2 % of the mean value (not shown). **b)** Log_{10} ratio of activities against pullulan/glycogen for the recombinant overexpressed Amy4, Amy9, Amy10, Amy12 and Amy16 enzymes. Standard deviations for triplicate assays of Amy10 and Amy12 pullulanase activity in were < 6% of the mean (not shown).

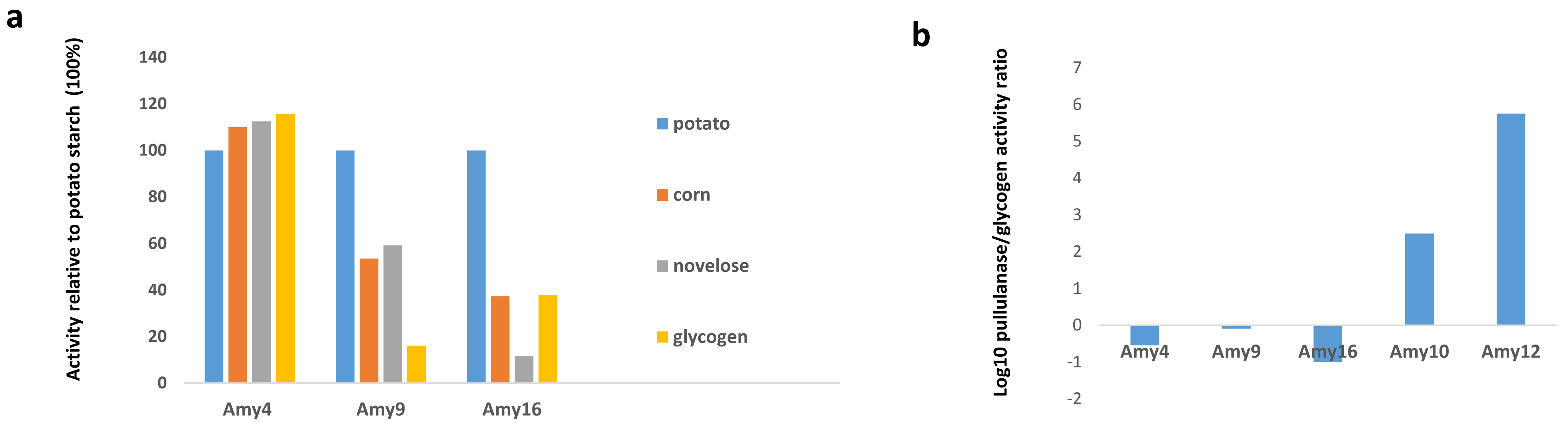

Supplement: Supplementary file 3 — Fig. S3. Activities of recombinant enzymes expressed in E. coli. [file EMI-20-324-s003.pdf]
