# Supplementary material for: Sporulation capability and amylosome conservation among diverse human colonic and rumen isolates of the keystone starch‐degrader Ruminococcus bromii
Source: Environ Microbiol. 2017 Dec 7;20(1):324–36. doi: 10.1111/1462-2920.14000 (PMC5814915; doi:10.1111/1462-2920.14000)

Figure S4. Phylogenetic tree comparing the 30 cohesin modules from *R. bromii* L2-63, L2-36, 5AMG, ATCC27255 and YE282 strains. Bootstrapping confidence values higher than 0.6 are shown in black.
.

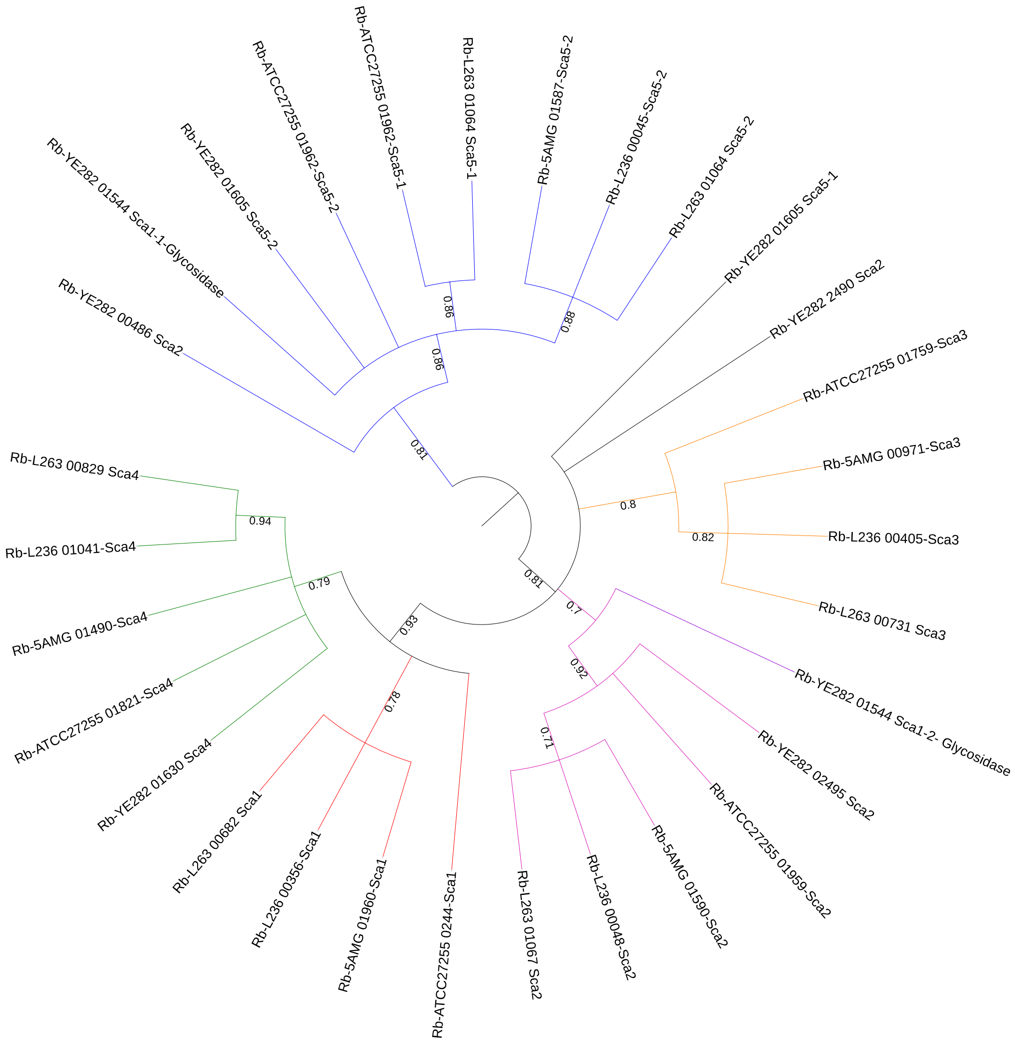

Supplement: Supplementary file 4 — Fig. S4. Phylogenetic tree comparing the 30 cohesin modules from R. bromii L2‐63, L2‐36, 5AMG, ATCC27255 and YE282 strains. [file EMI-20-324-s004.pdf]
