# Supplementary material for: Sporulation capability and amylosome conservation among diverse human colonic and rumen isolates of the keystone starch‐degrader Ruminococcus bromii
Source: Environ Microbiol. 2017 Dec 7;20(1):324–36. doi: 10.1111/1462-2920.14000 (PMC5814915; doi:10.1111/1462-2920.14000)

Figure S5. Carbohydrate transport/utilisation gene clusters in *R. bromii* genomes. Organisation of (A) maltose and fructose transport systems and (B) glycogen biosynthesis gene cluster for the human *Ruminococcus bromii* L2-63, L2-36, ATCC27255 and 5AMG strains. A separate galactose utilization cluster is also present (see Figure 5).

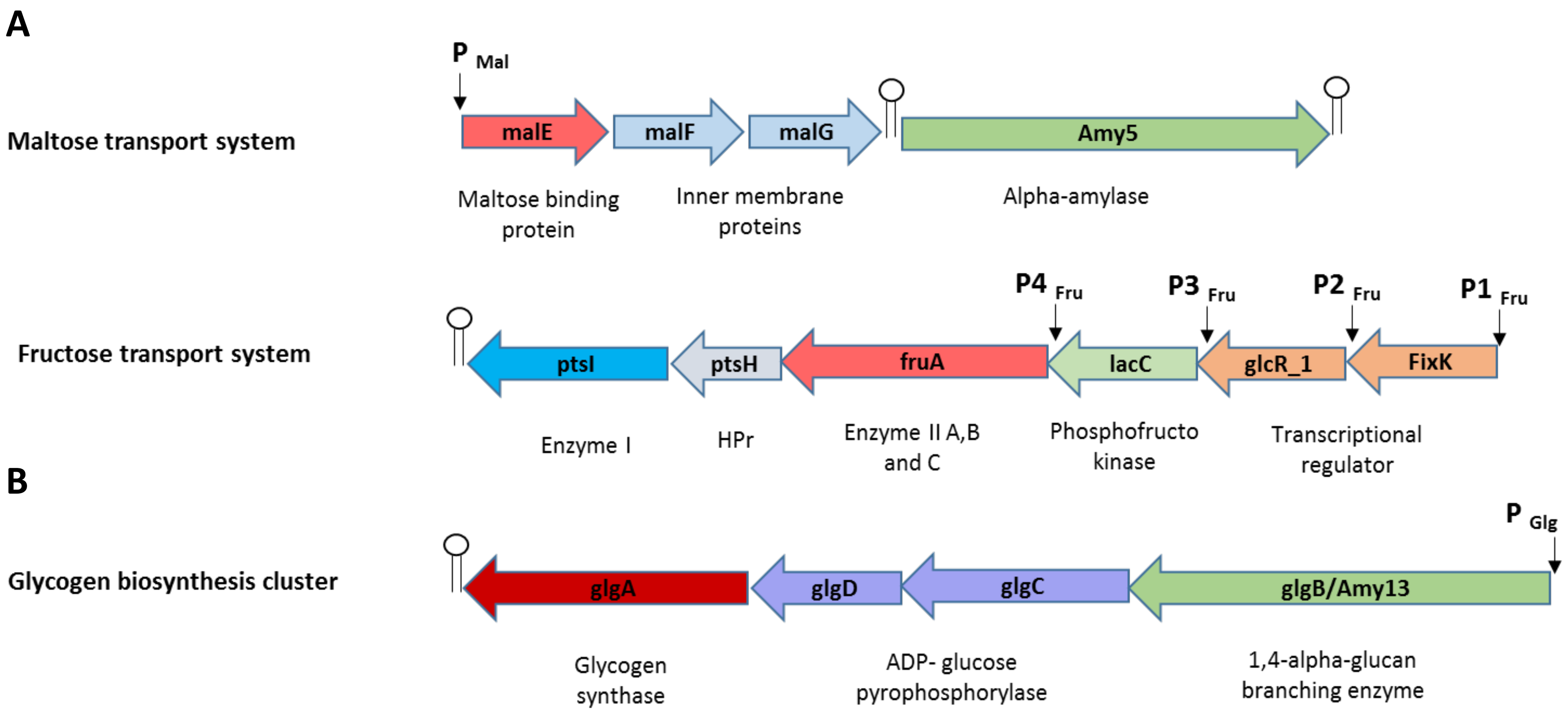

Supplement: Supplementary file 5 — Fig. S5. Carbohydrate transport/utilization gene clusters in R. bromii genomes. Organization of (A) maltose and fructose transport systems and (B) glycogen biosynthesis gene cluster for the human Ruminococcus bromii L2‐63, L2‐36, ATCC27255 and 5AMG strains. [file EMI-20-324-s005.pdf]
