# Supplementary material for: Sporulation capability and amylosome conservation among diverse human colonic and rumen isolates of the keystone starch‐degrader Ruminococcus bromii
Source: Environ Microbiol. 2017 Dec 7;20(1):324–36. doi: 10.1111/1462-2920.14000 (PMC5814915; doi:10.1111/1462-2920.14000)
Supplement: Supplementary file 7 — Table S1. Ruminococcus bromii genome assembly statistics. [file EMI-20-324-s007.docx]

**Supplementary Table 1**. *Ruminococcus bromii* genome assembly statistics

| ***R. bromii* strain** | **Source** | **Sample origin** | **Genome source** | **N50** | **Contigs** | **Genome size** | **Gene** | **CDS** | **Genome Reference** | **Accession number** |
| --- | --- | --- | --- | --- | --- | --- | --- | --- | --- | --- |
| L2-63 | Human faeces | UK | Wellcome Trust, Sanger Institute | 146298 | 27 | 2249085 | 2152 | 2111 | Ze *et al* 2015 (updated) | GCA_000209875.1 |
| L2-36 | Human faeces | UK | RI, University of Aberdeen | 144375 | 30 | 2281553 | 2216 | 2127 | This study | NPHY00000000 |
| 5AMG | Human faeces | Ecuador | RI, University of Aberdeen | 122098 | 52 | 2400097 | 2257 | 2186 | This Study | NNBY00000000 |
| ATCC 27255 | Human faeces | US | RI, University of Aberdeen | 101237 | 74 | 2151595 | 2009 | 1949 | This study | NNSR00000000 |
| YE282 | Bovine rumen | Australia | University of Queensland | 140000 | 81 | 2539482 | 2571 | 2491 | Hungate1000 (available through JGI) | GCA_900101355.1 |

RI= Rowett Institute.
