# Supplementary material for: Sporulation capability and amylosome conservation among diverse human colonic and rumen isolates of the keystone starch‐degrader Ruminococcus bromii
Source: Environ Microbiol. 2017 Dec 7;20(1):324–36. doi: 10.1111/1462-2920.14000 (PMC5814915; doi:10.1111/1462-2920.14000)
Supplement: Supplementary file 8 — Table S2. Distribution of GH, dockerins, cohesins and CBM modules in human and rumen Ruminococcus bromii strains [file EMI-20-324-s008.docx]

**Supplementary Table 2.** Distribution of GH, dockerins, cohesins and CBM modules in human and rumen *Ruminococcus bromii* strains

|  | **L2-63** | **L2-36** | **5AMG** | **ATCC27255** | **YE282** |
| --- | --- | --- | --- | --- | --- |
| **Total GH proteins** | 25 | 25 | 26 | 24 | 28 |
| **GH3** | 1 | 1 | 1 | 1 | 1 |
| **GH4** | 0 | 0 | 1 | 0 | 0 |
| **GH13** | 17 | 17 | 17 | 17 | 15 |
| **GH23** | 2 | 2 | 2 | 1 | 1 |
| **GH24** | 1 | 1 | 1 | 1 | 1 |
| **GH25** | 1 | 1 | 1 | 1 | 7 |
| **GH31** | 2 | 2 | 2 | 2 | 1 |
| **GH53** | 0 | 0 | 0 | 0 | 1 |
| **GH77** | 1 | 1 | 1 | 1 | 1 |
| **Dockerins** | 27 | 30 | 27 | 20 | 26 |
| **Cohesins** | 6 | 5* | 5* | 6 | 8 |
| **CBM13** | 1 | 1 | 0 | 0 | 0 |
| **CBM21** | 0 | 0 | 0 | 0 | 1 |
| **CBM25** | 0 | 0 | 0 | 0 | 2 |
| **CBM26** | 11 | 11 | 15 | 15 | 17 |
| **CBM30** | 0 | 0 | 0 | 0 | 1 |
| **CBM32** | 0 | 0 | 0 | 1 | 0 |
| **CBM34** | 1 | 1 | 1 | 1 | 0 |
| **‘X’ (CBM37-like)** | 12 | 12 | 10 | 16 | 0 |
| **CBM44** | 0 | 0 | 0 | 0 | 2 |
| **CBM46** | 1 | 1 | 1 | 0 | 0 |
| **CBM48** | 6 | 6 | 6 | 6 | 5 |
| **CBM50** | 3 | 3 | 3 | 3 | 4 |
| **CBM56** | 1 | 1 | 0 | 0 | 0 |

GH= Glycoside hydrolase, CBM=Carbohydrate binding module.

* = possibly 6 cohesins present
