# Supplementary material for: Sporulation capability and amylosome conservation among diverse human colonic and rumen isolates of the keystone starch‐degrader Ruminococcus bromii
Source: Environ Microbiol. 2017 Dec 7;20(1):324–36. doi: 10.1111/1462-2920.14000 (PMC5814915; doi:10.1111/1462-2920.14000)
Supplement: Supplementary file 9 — Table S3. List of primers used for the different constructs produced. [file EMI-20-324-s009.docx]

**Supplementary Table 3:** List of primers used for the different constructs produced (restriction enzyme sequences marked in uppercase). GFP-doc13a (from Sca1) was produced by restriction free. Ligation-independent cloning was used for Amy10 and Amy12; overhang regions are marked in uppercase and the TEV site is underlined.

| **Construct Name** | **Vector** | **Forward Primer(5’-3’)** | **Reverse Primer (5’-3’)** | **PCR Template** |
| --- | --- | --- | --- | --- |
| **Amy4** | pET28a | atatcaCCATGGgagcaacagtttccgatgacag | atatcaCTCGAGaaagttgctgatgaggtctac | *R. bromii L2-63* genomic DNA |
| **Amy9** | pIVEX2.4d | taaaatccatggtaaatgctgccgaggttgattc | aaaAGTACTttattctga atagcttacccagac | *R. bromii L2-63* genomic DNA |
| **Amy10** | pETite-Chis | GAAGGAGATATACATATGgcagtaacaagcgatgaatcggtttcagc | GTGATGGTGGTGATGATGgccctggaagtacaggttctctttttccgtcggctgtgtaacgggg | *R. bromii L2-63* genomic DNA |
| **Amy12** | pETite-Chis | GAAGGAGATATACATATGgcaacagccgat gacagttcggcag | GTGATGGTGGTGATGATGgccctggaagtacaggttctcgagtgattcagg aatgtagtctgtgtagtag | *R. bromii L2-63* genomic DNA |
| **Amy16** | pET28a | gatataCCATGGcacaccatcaccatcaccatgcagaaagctcgccaaccgtatc | tatctaCTCGAGttacttggagtatttactccactg | *R. bromii L2-63* genomic DNA |
| **GFP-doc13a**  **(from Sca1)** | pET9d  Xyn-doc13a  (Ze et al, 2015) | TATACCATGAgccaccatcaccatcaccatagtaaaggagaagaacttttc | Aaatgccgttgatgcgtttggtaccacttttttgtagagctcatccatgc | *R. bromii L2-63* genomic DNA |
| **Xyn-doc 1654** | pET9d  Xyn-doc13a  (Ze et al, 2015) | tacttaGGTACCaccttcaaacagaattcttatcgg | tacttaGGATCCttatgcaacattttctgtgtaagtac | *R. bromii L2-63* genomic DNA |
| **CBM-coh5** | pET28a CBM-CohA1  (Morais, 2015) | ttatcaGGATCCGCAAAGGGTCTCACAGTTACAGCTA | ttatcaCTCGAGttaCTCTTCTGAACCGTCGGGATCTGTTA | *R. bromii L2-63* genomic DNA |
| **CBM-coh6** | pET28a CBM-CohA1  (Morais, 2015) | ttatcaGGATCCGCAGTTGATAATTTAACAATCAACG | ttacaaCTCGAGttaCTCAACATATGCCTCAACCTTAGCG | *R. bromii L2-63* genomic DNA |
