# Supplementary material for: Sporulation capability and amylosome conservation among diverse human colonic and rumen isolates of the keystone starch‐degrader Ruminococcus bromii
Source: Environ Microbiol. 2017 Dec 7;20(1):324–36. doi: 10.1111/1462-2920.14000 (PMC5814915; doi:10.1111/1462-2920.14000)
Supplement: Supplementary file 6 — Fig. S6. Potential vancomycin resistance gene cluster in R. bromii L2‐63. (A), (B) This VanG cluster is present in R. bromii L2‐63 and L2‐36, but not in the other three genomes. However, we found no evidence for resistance to vancomycin in the two human strains that possess the VanG cluster compared with the two that lack the cluster (C). [file EMI-20-324-s006.pdf]

Figure S6. Vancomycin gene cluster in R. bromii L2-63. A) B) This VanG cluster is present in *R. bromii* L2-63 and L2-36, but not in the other three genomes. However we found no evidence for resistance to vancomycin in the two human strains that possess the VanG cluster compared with the two that lack the cluster (C).

A

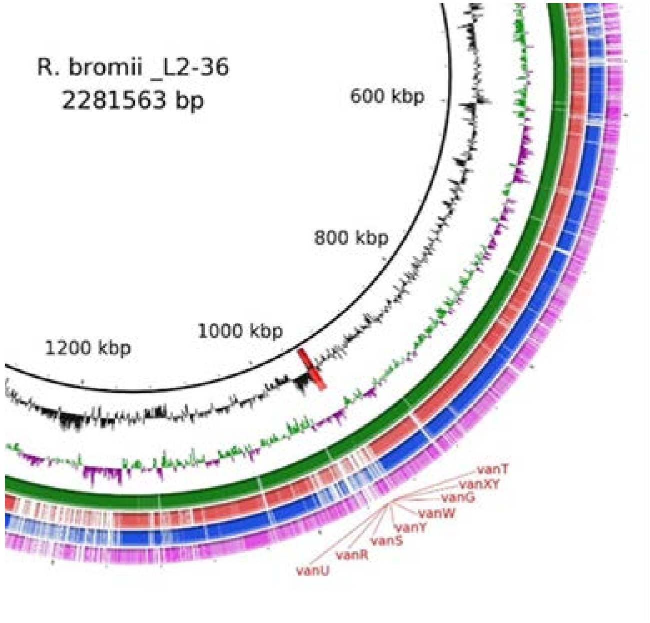

B

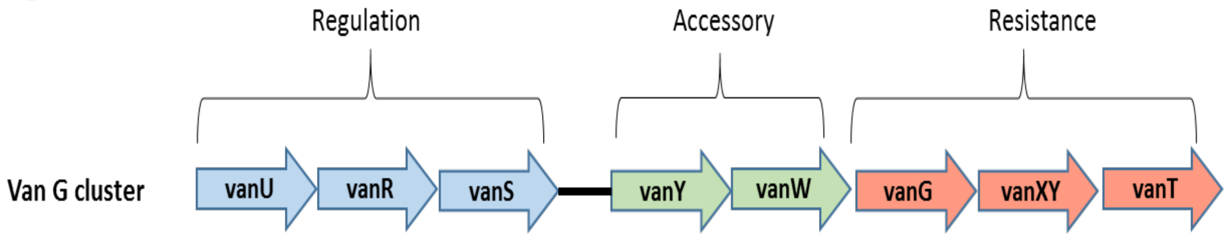

C

	Vancomycin concentration (µg/ml)										
	**142**	**71**	**35**	**17**	**14**	**7**	**3**	**2**	**1**	**0.4**	**0**
***R. bromii* ATCC27255**	X	X	X	X	X	X	X	X	X	✓	✓
***R. bromii* L2-63**	X	X	X	X	X	X	X	X	X	✓	✓
***R. bromii* L2-36**	X	X	X	X	X	X	X	X	X	✓	✓
***R. bromii* 5AMG**	X	X	X	X	X	X	X	X	X	✓	✓
