# Supplementary material for: Sporulation capability and amylosome conservation among diverse human colonic and rumen isolates of the keystone starch‐degrader Ruminococcus bromii
Source: Environ Microbiol. 2017 Dec 7;20(1):324–36. doi: 10.1111/1462-2920.14000 (PMC5814915; doi:10.1111/1462-2920.14000)
Supplement: Supplementary file 11 — Data File S2. List of locus tag numbers for amylase scaffoldin and dockerin genes for R. bromii L2‐63 strain before and after re‐annotation of the genome (Worksheet1), list of sporulation genes detected in R. bromii L2‐63 strain (Worksheet 2), conservation of L2‐63 sporulation genes in 4 other R. bromii strains (Worksheet 3), comparison of spore signature genes described by Browne et al. with R. bromii strains (Worksheet 4). [file EMI-20-324-s011.pdf]

	R. bromii L2-63 locus tag number after re-annotation	*R. bromii* L2-63 previous locus tag number	Synonym
**Amy 1**	L2-63_00433	RBR_04300	
**Amy 2**	L2-63_00496	RBR_04930	
**Amy 3**	L2-63_00613	RBR_06100	
**Amy 4**	L2-63_00682	RBR_06780	
**Amy 5**	L2-63_00780	RBR_07800	
**Amy 6**	L2-63_00862	RBR_08620	
**Amy 7**	L2-63_01126	RBR_11290	
**Amy 8**	L2-63_01910	RBR_19140	
**Amy 9**	L2-63_02041	RBR_20490	
**Amy 10**	L2-63_01094	RBR_10980	
**Amy 11**	L2-63_01356	RBR_13550	
**Amy 12**	L2-63_01357	RBR_13560	
**Amy 13**	L2-63_01115	RBR_11170	
**Amy 14**	L2-63_00370	RBR_03680	
**Amy 15**	L2-63_01911	RBR_19150	
**Amy 16**	L2-63_01654	Not detected	
**Amy 17**	L2-63_01441	Not detected	
**Sca1**	L2-63_00682	RBR_06780	Amy 4
**Sca2**	L2-63_01067	RBR_10710	
**Sca3**	L2-63_00731	RBR_07310	
**Sca4**	L2-63_00829	RBR_08290	
**Sca5**	L2-63_01064	Not detected	
**Doc 1**	L2-63_00285	RBR_02970	
**Doc 2**	L2-63_00287	RBR_02990	
**Doc 3**	L2-63_00546	RBR_05420	
**Doc 4**	L2-63_00576	RBR_05720	
**Doc 5**	L2-63_01402	RBR_14010	
**Doc 6**	L2-63_01443	RBR_14490	
**Doc 7**	L2-63_01793	RBR_17960	
**Doc 8**	L2-63_01861	RBR_18670	
**Doc 9**	L2-63_02145	RBR_21580	
**Doc 10**	L2-63_00682	RBR_06780	Amy 4
**Doc 11**	L2-63_02041	RBR_20490	Amy 9
**Doc 12**	L2-63_01094	RBR_10980	Amy 10
**Doc 13**	L2-63_01357	RBR_13560	Amy 12
**Doc 14**	L2-63_00436	RBR_04330	
**Doc 15**	L2-63_00485	RBR_04820	
**Doc 16**	L2-63_01616	RBR_16240	
**Doc 17**	L2-63_01862	RBR_18680	
**Doc 18**	L2-63_01881	RBR_18860	
**Doc 19**	L2-63_01944	RBR_19520	
**Doc 20**	L2-63_00125	RBR_01410	
**Doc 21**	L2-63_00158	RBR_01740	
**Doc 22**	L2-63_00434	RBR_04310	
**Doc 23**	L2-63_00717	RBR_07140	
**Doc 24**	L2-63_01374	RBR_13730	
**Doc 25**	L2-63_01451	RBR_14570	
**Doc 26**	L2-63_01574	RBR_15800	
**Doc 27**	L2-63_01654	Not detected	Amy 16

R. bromii L2-63 locus tag	Gene annotation
L2-63 00110	soj 1 Sporulation initiation inhibitor protein soj,plasmid-partitioning protein RepA,Septum formation inhibitor-activating ATPase,plasmid partitioning protein RepA,CobQ/CobB/MinD/ParA nucleotide binding domain 117275:118102 reverse MW:30184
L2-63 00593	soj 2 Sporulation initiation inhibitor protein soj,plasmid-partitioning protein RepA,Septum formation inhibitor-activating ATPase,cell division ATPase MinD,CobQ/CobB/MinD/ParA nucleotide binding domain 636409:637176 reverse MW:27874
L2-63 01701	soj 3 Sporulation initiation inhibitor protein soj,plasmid-partitioning protein RepA,Septum formation inhibitor-activating ATPase,plasmid partitioning protein RepA,CobQ/CobB/MinD/ParA nucleotide binding domain 1791642:1792466 forward MW:29817
L2-63 01362	L2-63 01362 sporulation inhibitor KapD,Inhibitor of the KinA pathway to sporulation, predicted exonuclease,DNA polymerase III, alpha subunit, Gram-positive type,Exonuclease 1423530:1424420 forward MW:34040
L2-63 00972	sigH Stage 0 sporulation protein H,RNA polymerase factor sigma-70,RNA polymerase sigma-H factor,Sigma-70, region 4 1035179:1035769 forward MW:21824
L2-63 01663	spo0A Stage 0 sporulation protein G,chemotaxis-specific methylesterase,Response regulator of citrate/malate metabolism,sporulation transcription factor Spo0A,Sporulation initiation factor Spo0A C terminal 1749036:1749806 forward MW:28974
L2-63 00354	mrkE hypothetical protein,putative two-component response-regulatory protein YehT,Response regulator of the LytR/AlgR family,sporulation transcription factor Spo0A,Response regulator receiver domain 355792:356523 forward MW:28162
L2-63 00109	parB 1 Chromosome-partitioning protein parB,plasmid partitioning protein RepB,ParB/RepB/Spo0J family partition protein,ParB-like nuclease domain 116374:117321 reverse MW:36513
L2-63 00592	parB 2 Probable chromosome-partitioning protein parB,plasmid partitioning protein RepB,ParB/RepB/Spo0J family partition protein,ParB-like nuclease domain 635559:636407 reverse MW:31201
L2-63 01702	parB 3 Probable chromosome-partitioning protein parB,plasmid partitioning protein,ParB/RepB/Spo0J family partition protein,ParB-like nuclease domain 1792423:1793364 forward MW:35407
L2-63 02000	parB 4 Probable chromosome-partitioning protein parB,plasmid partitioning protein RepB,ParB/RepB/Spo0J family partition protein,ParB-like nuclease domain 2083908:2084753 reverse MW:32796
L2-63 02125	parB 5 Probable chromosome-partitioning protein parB,plasmid partitioning protein RepB,ParB/RepB/Spo0J family partition protein,ParB-like nuclease domain 2188827:2189648 reverse MW:31731
L2-63 01542	sepF Cell division protein SepF,Protein of unknown function (DUF552) 1620226:1620693 forward MW:17612
L2-63 01819	L2-63 01819 Septum formation initiator 1900610:1901056 reverse MW:17079
L2-63 01504	sigE RNA polymerase sigma-35 factor precursor,sporulation sigma factor SigE,RNA polymerase sigma-E factor,Sigma-70 region 2 1581366:1582067 reverse MW:26566
L2-63 00833	sigF 2 Stage II sporulation protein AC,sporulation sigma factor SigF,RNA polymerase sigma-F factor,Sigma-70, region 4 891831:892490 reverse MW:24772
L2-63 01505	L2-63 01505 sigma-E processing peptidase SpoIIGA,Sporulation factor SpoIIGA 1582078:1582881 reverse MW:29621
L2-63 00834	spoIIAB Anti-sigma F factor,anti-sigma F factor,Signal transduction histidine kinase,anti-sigma F factor,Histidine kinase-, DNA gyrase B-, and HSP90-like ATPase 892480:892920 reverse MW:15963
L2-63 00835	spoIIAA 1 Stage II sporulation protein AA,Anti-anti-sigma regulatory factor (antagonist of anti-sigma factor),anti-sigma F factor antagonist,STAS domain 892917:893219 reverse MW:11077
L2-63 01168	spoIIAA 2 Stage II sporulation protein AA,Anti-anti-sigma regulatory factor (antagonist of anti-sigma factor),anti-anti-sigma factor,STAS domain 1241266:1241574 reverse MW:11313
L2-63 01395	L2-63 01395 stage II sporulation protein M 1459044:1459712 forward MW:24721
L2-63 01486	L2-63 01486 stage II sporulation protein R,Stage II sporulation protein R (spore II R) 1553681:1554283 forward MW:23015
L2-63 00295	spoIIQ Stage II sporulation protein Q,Membrane-bound metallopeptidase,Peptidase family M23 296471:297199 forward MW:26396
L2-63 00381	spoIIE Stage II sporulation protein E,stage II sporulation protein E,Stage II sporulation protein E (SpoIIE) 380299:382623 reverse MW:82850
L2-63 00388	rsbU Phosphoserine phosphatase rsbU,stage II sporulation protein E,Stage II sporulation protein E (SpoIIE) 393426:395069 reverse MW:61809
L2-63 00438	L2-63 00438 stage II sporulation protein P,Stage II sporulation protein P (SpoIIP) 449541:450638 forward MW:40615
L2-63 00209	sigF 1 Stage II sporulation protein AC,sporulation sigma factor SigG,RNA polymerase sigma-G factor,Sigma-70, region 4 228385:229167 forward MW:29578
L2-63 01805	L2-63 01805 stage III sporulation protein AG 1889807:1890256 reverse MW:16203
L2-63 01807	L2-63 01807 stage III sporulation protein AE,Stage III sporulation protein AE (spore III AE) 1890730:1891848 reverse MW:38770
L2-63 01808	L2-63 01808 stage III sporulation protein AD,Stage III sporulation protein AC/AD protein family 1891858:1892247 reverse MW:13366
L2-63 01809	L2-63 01809 stage III sporulation protein AC,Stage III sporulation protein AC/AD protein family 1892249:1892443 reverse MW:6989
L2-63 01810	L2-63 01810 stage III sporulation protein SpoAB,stage III sporulation protein AB,Stage III sporulation protein AB (spore III AB) 1892452:1892946 reverse MW:18375
L2-63 01804	L2-63 01804 SpoIIIAH-like protein 1889250:1889792 reverse MW:19553
L2-63 01950	spoIIIE Stage III sporulation protein E,DNA translocase FtsK,DNA segregation ATPase FtsK/SpoIIIE and related proteins,type VII secretion protein EssC,FtsK/SpoIIIE family 2032173:2034929 reverse MW:100193
L2-63 00486	spoIIID 14 kDa transcription factor,sporulation transcriptional regulator SpoIIID,Stage III sporulation protein D 509822:510094 forward MW:10071
L2-63 01811	L2-63 01811 Uncharacterized protein conserved in bacteria,stage III sporulation protein AA 1892936:1893886 reverse MW:34365
L2-63 01295	sigK RNA polymerase sigma-28 factor precursor,sporulation sigma factor SigK,RNA polymerase sigma-K factor,Sigma-70 region 2 1353319:1354014 reverse MW:26172
L2-63 01857	spoIVA Stage IV sporulation protein A,stage IV sporulation protein A,Stage IV sporulation protein A (spore IV A) 1938504:1939982 reverse MW:55914
L2-63 01662	spoIVB SpoIVB peptidase precursor,Predicted secreted protein containing a PDZ domain,stage IV sporulation protein B,SpoIVB peptidase S55 1747865:1748887 forward MW:37314
L2-63 01939	L2-63 01939 sporulation protein YqfD,Putative stage IV sporulation protein YqfD 2019519:2020697 reverse MW:44472
L2-63 01941	L2-63 01941 Uncharacterized protein conserved in bacteria,sporulation integral membrane protein YlbJ,Nucleoside recognition 2021053:2022123 forward MW:38307
L2-63 00575	L2-63 00575 stage V sporulation protein AE,SpoVA protein 620709:621062 forward MW:11671
L2-63 00785	L2-63 00785 stage V sporulation protein AC,SpoVA protein 857225:857680 reverse MW:15951
L2-63 00769	spoVAD Stage V sporulation protein AD,stage V sporulation protein AD,stage V sporulation protein AD,Stage V sporulation protein AD (SpoVAD) 833612:834622 reverse MW:35677
L2-63 00220	spoVT Stage V sporulation protein T,stage V sporulation protein T,SpoVT / AbrB like domain 237414:237956 forward MW:19830
L2-63 00283	spoVFA Stage V sporulation protein FA,dipicolinate synthase subunit A,dipicolinic acid synthetase, A subunit 284892:285767 forward MW:31857
L2-63 01257	L2-63 01257 Spore coat associated protein JA (CotJA) 1323069:1323269 reverse MW:7358
L2-63 01256	L2-63 01256 CotJB protein 1322822:1323085 reverse MW:10452
L2-63 01354	L2-63 01354 Muramidase-2 precursor,membrane-bound lytic murein transglycosylase D,Oxidation resistance protein,spore coat assembly protein SafA,LysM domain 1409244:1409549 reverse MW:11190
L2-63_00771	ugtP Processive diacylglycerol glucosyltransferase,diacylglycerol glucosyltransferase,Spore coat polysaccharide biosynthesis protein, predicted glycosyltransferase,undecaprenyldiphospho-muramoylpentapeptide beta-N-acetylglucosaminyltransferase,Monogalactosyldiacylglycerol (MGDG) synthase 836861:838003 forward MW:42729
L2-63 01824	ytgP Probable cell division protein ytgP,stage V sporulation protein B,MatE 1903801:1905657 reverse MW:66913
L2-63 01012	ftsW 2 Cell division protein FtsW,cell wall shape-determining protein,stage V sporulation protein E,Cell cycle protein 1069360:1070616 forward MW:46915
L2-63 01010	ftsI Peptidoglycan synthase FtsI precursor,peptidoglycan synthase FtsI,Membrane carboxypeptidase/penicillin-binding protein,stage V sporulation protein D,Penicillin binding protein transpeptidase domain 1065978:1068248 forward MW:81515
L2-63 00437	gpr Germination protease precursor,germination protease,GPR endopeptidase,Germination protease 448572:449447 forward MW:31539
L2-63 01844	L2-63 01844 Bacillus/Clostridium GerA spore germination protein 1925442:1926896 reverse MW:53679
L2-63 01843	L2-63 01843 spore germination protein (amino acid permease) 1924375:1925442 reverse MW:39757
L2-63 01842	L2-63 01842 germination protein, Ger(x)C family 1923792:1924382 reverse MW:21622
L2-63 00389	sleB Germination-specific amidase,Uncharacterized conserved protein,spore cortex-lytic enzyme,Putative peptidoglycan binding domain 395673:397265 reverse MW:56229
L2-63 01585	cwlJ Cell wall hydrolase CwlJ,Cell wall hydrolyses involved in spore germination,spore cortex-lytic enzyme,Cell Wall Hydrolase 1668618:1669055 reverse MW:16059
L2-63 00319	spmA Spore maturation protein A,Uncharacterized membrane protein,Nucleoside recognition 320363:320947 forward MW:20788
L2-63 00320	spmB Spore maturation protein B,hypothetical protein,Uncharacterized membrane protein 320976:321476 forward MW:17657
L2-63 00174	paiA Protease synthase and sporulation negative regulatory protein PAI 1,ribosomal-protein-alanine acetyltransferase,Acetyltransferase (GNAT) family 188586:189110 forward MW:19857
L2-63 00444	yebN hypothetical protein,hypothetical protein,putative sporulation protein YtaF,Domain of unknown function DUF 458034:458597 reverse MW:20110
L2-63 00863	L2-63 00863 Negative regulator of genetic competence, sporulation and motility 917748:918302 forward MW:20856
L2-63 00955	xynD Bifunctional xylanase/deacetylase precursor,polysaccharide deacetylase family sporulation protein PdaB,Polysaccharide deacetylase 1018553:1019338 reverse MW:29076
L2-63 01073	ytfJ Uncharacterized spore protein ytfJ,Uncharacterized conserved protein,sporulation protein YtfJ,Sporulation protein YtfJ (Spore YtfJ) 1129826:1130245 forward MW:14853
L2-63 01507	L2-63 01507 Uncharacterized protein conserved in bacteria,sporulation protein, YlmC/YmxH family,PRC-barrel domain 1584146:1584433 forward MW:10662
L2-63 01534	L2-63 01534 sporulation protein YunB,Sporulation protein YunB (Spo YunB) 1609947:1610600 forward MW:24285
L2-63 01630	yhhT hypothetical protein,pheromone autoinducer 2 transporter,sporulation integral membrane protein YtvI,Domain of unknown function DUF20 1711378:1712697 reverse MW:49073
L2-63 01820	L2-63 01820 sporulation protein YabP,YabP family 1901459:1901731 reverse MW:9788
L2-63 01838	whiA Putative sporulation transcription regulator WhiA,Uncharacterized protein conserved in bacteria,conserved hypothetical protein,Sporulation Regulator WhiA C terminal domain 1919846:1920781 reverse MW:35064
L2-63 01940	L2-63 01940 sporulation protein YqfC,YabP family 2020707:2020979 reverse MW:10041
L2-63 00312	sspC2 SSP-3,Small, acid-soluble spore proteins, alpha/beta type 312723:312920 reverse MW:7173

Key	Stages of sporulation	Function
	Sporulation initiation inhibitor	Regulation
	Stage I	Initiation of spore formation
	Stage II	Asymmetric cell division
	Stage III	Engulfment of forespore by mother cell
	Stage IV	Formation of cortex
	Stage V	Formation of coat
	Germination	Germination of spore
	Unknown sporulation genes	Genes not associated with any specific stage of sporulation

qseqid query (e.g., gene) sequence id	sseqid subject (e.g., reference genome) sequence id	pident percentage of identical matches	length alignment length	mismatch number of mismatches	gapopen number of gap openings	qstart start of alignment in query	qend end of alignment in query	sstart start of alignment in subject	send end of alignment in subject	evalue expect value	bitscore bit score
L2-63_00109	RBL236_01215	100	315	0	0	1	315	1	315	0	640
L2-63_00110	RBL236_01216	100	275	0	0	1	275	1	275	0	561
L2-63_00174	RBL236_01565	100	174	0	0	1	174	1	174	1.00E-128	360
L2-63_00209	RBL236_01600	100	260	0	0	1	260	1	260	0	525
L2-63_00220	RBL236_01740	100	180	0	0	1	180	1	180	6.00E-129	362
L2-63_00283	RBL236_01803	100	291	0	0	1	291	1	291	0	582
L2-63_00295	RBL236_01815	100	242	0	0	1	242	1	242	1.00E-179	496
L2-63_00312	RBL236_02214	100	65	0	0	1	65	1	65	1.00E-41	131
L2-63_00319	RBL236_02207	100	194	0	0	1	194	1	194	4.00E-139	389
L2-63_00320	RBL236_02206	100	166	0	0	1	166	1	166	1.00E-117	332
L2-63_00354	RBL236_02172	100	243	0	0	1	243	1	243	0	499
L2-63_00381	RBL236_02145	100	774	0	0	1	774	1	774	0	1576
L2-63_00388	RBL236_02138	100	547	0	0	1	547	1	547	0	1129
L2-63_00389	RBL236_02137	100	530	0	0	1	530	1	530	0	1074
L2-63_00437	RBL236_02089	100	291	0	0	1	291	1	291	0	578
L2-63_00438	RBL236_02088	100	365	0	0	1	365	1	365	0	749
L2-63_00444	RBL236_02082	100	187	0	0	1	187	1	187	2.00E-130	366
L2-63_00486	RBL236_00534	100	90	0	0	1	90	1	90	8.00E-60	179
L2-63_00575	RBL236_00250	100	117	0	0	1	117	1	117	1.00E-76	224
L2-63_00592	RBL236_00267	100	282	0	0	1	282	1	282	0	560
L2-63_00593	RBL236_00268	100	255	0	0	1	255	1	255	0	520
L2-63_00769	RBL236_00442	100	336	0	0	1	336	1	336	0	696
L2-63_00771	RBL236_00444	100	380	0	0	1	380	1	380	0	781
L2-63_00785	RBL236_00458	100	151	0	0	1	151	1	151	6.00E-105	298
L2-63_00833	RBL236_01045	100	219	0	0	1	219	1	219	1.00E-160	445
L2-63_00834	RBL236_01046	100	146	0	0	1	146	1	146	3.00E-106	301
L2-63_00835	RBL236_01047	100	100	0	0	1	100	1	100	6.00E-69	203
L2-63_00863	RBL236_01075	100	184	0	0	1	184	1	184	2.00E-134	376
L2-63_00955	RBL236_00647	100	261	0	0	1	261	1	261	0	545
L2-63_00972	RBL236_00630	100	196	0	0	1	196	1	196	1.00E-142	398
L2-63_01010	RBL236_00592	100	756	0	0	1	756	1	756	0	1548
L2-63_01012	RBL236_00590	100	418	0	0	1	418	1	418	0	836
L2-63_01073	RBL236_00054	100	139	0	0	1	139	1	139	8.00E-97	277
L2-63_01168	RBL236_01925	100	102	0	0	1	102	1	102	9.00E-69	203
L2-63_01256	RBL236_00720	100	87	0	0	1	87	1	87	3.00E-60	180
L2-63_01257	RBL236_00721	100	66	0	0	1	66	1	66	1.00E-43	136
L2-63_01295	RBL236_00759	100	231	0	0	1	231	1	231	6.00E-170	470
L2-63_01354	RBL236_00818	100	101	0	0	1	101	1	101	5.00E-71	208
L2-63_01362	RBL236_00826	100	296	0	0	1	296	1	296	0	610
L2-63_01395	RBL236_02016	100	222	0	0	1	222	1	222	2.00E-158	440
L2-63_01486	RBL236_01482	100	200	0	0	1	200	1	200	8.00E-149	414
L2-63_01504	RBL236_01465	100	233	0	0	1	233	1	233	6.00E-171	473
L2-63_01505	RBL236_01464	100	267	0	0	1	267	1	267	0	522
L2-63_01507	RBL236_01462	100	95	0	0	1	95	1	95	1.00E-64	192
L2-63_01534	RBL236_01434	100	217	0	0	1	217	1	217	3.00E-158	439
L2-63_01542	RBL236_01426	100	155	0	0	1	155	1	155	8.00E-111	314
L2-63_01585	RBL236_01383	100	145	0	0	1	145	1	145	5.00E-107	303
L2-63_01630	RBL236_01338	100	439	0	0	1	439	1	439	0	879
L2-63_01662	RBL236_01308	100	340	0	0	1	340	1	340	0	694
L2-63_01663	RBL236_01307	100	256	0	0	1	256	1	256	0	526
L2-63_01701	RBL236_01269	100	274	0	0	1	274	1	274	0	561
L2-63_01702	RBL236_01268	100	313	0	0	1	313	1	313	0	640
L2-63_01804	RBL236_01615	100	180	0	0	1	180	1	180	2.00E-127	358
L2-63_01805	RBL236_01616	100	149	0	0	1	149	1	149	2.00E-102	292
L2-63_01807	RBL236_01618	100	372	0	0	1	372	1	372	0	717
L2-63_01808	RBL236_01619	100	129	0	0	1	129	1	129	2.00E-82	240
L2-63_01809	RBL236_01620	100	64	0	0	1	64	1	64	2.00E-37	120
L2-63_01810	RBL236_01621	100	164	0	0	1	164	1	164	3.00E-117	331
L2-63_01811	RBL236_01622	100	316	0	0	1	316	1	316	0	645
L2-63_01819	RBL236_01630	100	148	0	0	1	148	1	148	9.00E-100	285
L2-63_01820	RBL236_01631	100	90	0	0	1	90	1	90	2.00E-61	183
L2-63_01824	RBL236_01635	100	618	0	0	1	618	1	618	0	1241
L2-63_01838	RBL236_01649	100	311	0	0	1	311	1	311	0	642
L2-63_01842	RBL236_01653	100	196	0	0	1	196	1	196	4.00E-137	384
L2-63_01843	RBL236_01654	100	355	0	0	1	355	1	355	0	692
L2-63_01844	RBL236_01655	100	484	0	0	1	484	1	484	0	990
L2-63_01857	RBL236_01668	100	492	0	0	1	492	1	492	0	1004
L2-63_01939	RBL236_00668	100	392	0	0	1	392	1	392	0	800
L2-63_01940	RBL236_00669	100	90	0	0	1	90	1	90	2.00E-61	183
L2-63_01941	RBL236_00670	100	356	0	0	1	356	1	356	0	702
L2-63_01950	RBL236_00679	100	918	0	0	1	918	1	918	0	1869
L2-63_02000	RBL236_00192	100	281	0	0	1	281	1	281	0	583
L2-63_02125	RBL236_00069	100	273	0	0	1	273	1	273	0	556
L2-63_00109	RB5AMG_00413	60.86	304	114	2	5	308	4	302	1.00E-125	364
L2-63_00110	RB5AMG_01432	52.57	272	129	0	1	272	1	272	2.00E-105	310
L2-63_00174	RB5AMG_01839	98.27	173	3	0	1	173	1	173	4.00E-126	354
L2-63_00209	RB5AMG_01804	100	260	0	0	1	260	1	260	0	525
L2-63_00220	RB5AMG_00070	99.33	150	1	0	31	180	589	738	4.00E-100	306
L2-63_00283	RB5AMG_00088	98.97	291	3	0	1	291	1	291	0	576
L2-63_00295	RB5AMG_00100	99.17	242	2	0	1	242	1	242	1.00E-177	491
L2-63_00312	RB5AMG_00328	100	65	0	0	1	65	1	65	1.00E-41	131
L2-63_00319	RB5AMG_00335	100	194	0	0	1	194	1	194	4.00E-139	389
L2-63_00320	RB5AMG_00336	99.4	166	1	0	1	166	1	166	9.00E-116	327
L2-63_00354	RB5AMG_00443	98.77	243	3	0	1	243	1	243	2.00E-178	493
L2-63_00381	RB5AMG_00468	99.1	774	7	0	1	774	1	774	0	1561

L2-63_00388	RB5AMG_00476	98.17	547	10	0	1	547	1	547	0	1106
L2-63_00389	RB5AMG_00922	99.25	530	1	1	1	530	1	527	0	1061
L2-63_00437	RB5AMG_00988	99.66	291	1	0	1	291	1	291	0	576
L2-63_00438	RB5AMG_00987	98.63	365	5	0	1	365	1	365	0	741
L2-63_00444	RB5AMG_00981	98.4	187	3	0	1	187	1	187	8.00E-129	362
L2-63_00486	RB5AMG_02241	98.89	90	1	0	1	90	1	90	2.00E-58	176
L2-63_00575	RB5AMG_01771	100	117	0	0	1	117	1	117	1.00E-76	224
L2-63_00592	RB5AMG_01752	99.29	282	2	0	1	282	1	282	0	555
L2-63_00593	RB5AMG_01751	99.61	255	1	0	1	255	1	255	0	518
L2-63_00769	RB5AMG_01550	99.7	336	1	0	1	336	1	336	0	693
L2-63_00771	RB5AMG_01548	98.42	380	6	0	1	380	1	380	0	773
L2-63_00785	RB5AMG_01534	99.34	151	1	0	1	151	1	151	3.00E-104	296
L2-63_00833	RB5AMG_01486	99.54	219	1	0	1	219	1	219	4.00E-160	444
L2-63_00834	RB5AMG_01485	99.32	146	1	0	1	146	1	146	3.00E-105	299
L2-63_00835	RB5AMG_01484	100	100	0	0	1	100	1	100	6.00E-69	203
L2-63_00863	RB5AMG_01456	98.91	184	2	0	1	184	1	184	4.00E-133	373
L2-63_00955	RB5AMG_01304	100	261	0	0	1	261	1	261	0	545
L2-63_00972	RB5AMG_01287	98.98	196	2	0	1	196	1	196	1.00E-141	395
L2-63_01010	RB5AMG_01251	98.68	756	10	0	1	756	1	756	0	1530
L2-63_01012	RB5AMG_01249	99.28	418	3	0	1	418	1	418	0	832
L2-63_01073	RB5AMG_01596	99.28	139	1	0	1	139	1	139	4.00E-96	275
L2-63_01168	RB5AMG_01064	98.02	101	2	0	1	101	1	101	1.00E-66	197
L2-63_01256	RB5AMG_01168	97.67	86	2	0	1	86	1	86	7.00E-59	176
L2-63_01257	RB5AMG_01169	98.48	66	1	0	1	66	1	66	8.00E-43	134
L2-63_01295	RB5AMG_00626	99.13	231	2	0	1	231	1	231	3.00E-169	468
L2-63_01354	RB5AMG_00569	98.02	101	2	0	1	101	1	101	7.00E-70	206
L2-63_01362	RB5AMG_00561	99.32	296	2	0	1	296	1	296	0	607
L2-63_01395	RB5AMG_00525	99.55	222	1	0	1	222	1	222	1.00E-157	438
L2-63_01486	RB5AMG_00206	100	200	0	0	1	200	1	200	8.00E-149	414
L2-63_01504	RB5AMG_00794	98.71	233	3	0	1	233	1	233	4.00E-169	468
L2-63_01505	RB5AMG_00795	99.25	267	2	0	1	267	1	267	0	516
L2-63_01507	RB5AMG_00797	100	95	0	0	1	95	1	95	1.00E-64	192
L2-63_01534	RB5AMG_00826	99.08	217	2	0	1	217	1	217	8.00E-157	436
L2-63_01542	RB5AMG_00834	100	155	0	0	1	155	1	155	8.00E-111	314
L2-63_01585	RB5AMG_00878	100	145	0	0	1	145	1	145	5.00E-107	303
L2-63_01630	RB5AMG_00155	99.09	439	4	0	1	439	1	439	0	870
L2-63_01662	RB5AMG_01655	97.65	340	8	0	1	340	1	340	0	677
L2-63_01663	RB5AMG_01656	100	256	0	0	1	256	1	256	0	526
L2-63_01701	RB5AMG_00414	57.66	274	116	0	1	274	1	274	3.00E-111	324
L2-63_01804	RB5AMG_00706	99.44	180	1	0	1	180	1	180	8.00E-126	354
L2-63_01805	RB5AMG_00705	97.99	149	3	0	1	149	1	149	3.00E-101	289
L2-63_01807	RB5AMG_00703	99.73	372	1	0	1	372	1	372	0	715
L2-63_01808	RB5AMG_00702	100	129	0	0	1	129	1	129	2.00E-82	240
L2-63_01809	RB5AMG_00701	100	64	0	0	1	64	1	64	2.00E-37	120
L2-63_01810	RB5AMG_00700	99.39	164	1	0	1	164	1	164	2.00E-116	328
L2-63_01811	RB5AMG_00699	98.42	316	5	0	1	316	1	316	0	639
L2-63_01819	RB5AMG_00691	100	148	0	0	1	148	1	148	9.00E-100	285
L2-63_01820	RB5AMG_00690	100	90	0	0	1	90	1	90	2.00E-61	183
L2-63_01824	RB5AMG_00686	99.84	618	1	0	1	618	1	618	0	1240
L2-63_01838	RB5AMG_00672	100	311	0	0	1	311	1	311	0	642
L2-63_01842	RB5AMG_00668	98.47	196	3	0	1	196	1	196	1.00E-134	377
L2-63_01843	RB5AMG_00667	98.59	355	5	0	1	355	1	355	0	684
L2-63_01844	RB5AMG_00666	99.17	484	4	0	1	484	1	484	0	985
L2-63_01857	RB5AMG_00651	99.19	492	4	0	1	492	1	492	0	994
L2-63_01939	RB5AMG_02145	98.47	392	6	0	1	392	1	392	0	791
L2-63_01940	RB5AMG_02146	100	90	0	0	1	90	1	90	2.00E-61	183
L2-63_01941	RB5AMG_02147	97.75	356	8	0	1	356	1	356	0	692
L2-63_01950	RB5AMG_02154	99.35	918	6	0	1	918	1	918	0	1858
L2-63_00209	RBATCC27255_01940	95.77	260	11	0	1	260	1	260	0	510
L2-63_00220	RBATCC27255_00711	98.89	180	2	0	1	180	1	180	8.00E-128	359
L2-63_00283	RBATCC27255_00276	97.94	291	6	0	1	291	1	291	0	573
L2-63_00295	RBATCC27255_00288	89.3	243	25	1	1	242	1	243	4.00E-154	431
L2-63_00319	RBATCC27255_00930	100	194	0	0	1	194	1	194	4.00E-139	389
L2-63_00320	RBATCC27255_00931	100	166	0	0	1	166	1	166	1.00E-117	332
L2-63_00354	RBATCC27255_00833	98.36	244	3	1	1	243	1	244	8.00E-177	488
L2-63_00381	RBATCC27255_01820	98.71	774	10	0	1	774	1	774	0	1553
L2-63_00388	RBATCC27255_01879	94.88	547	28	0	1	547	1	547	0	1051
L2-63_00389	RBATCC27255_00013	90.71	538	42	2	1	530	1	538	0	921
L2-63_00437	RBATCC27255_01877	97.25	291	8	0	1	291	1	291	0	566
L2-63_00438	RBATCC27255_01876	93.13	364	25	0	1	364	1	364	0	693
L2-63_00444	RBATCC27255_00268	97.86	187	4	0	1	187	1	187	1.00E-127	359
L2-63_00486	RBATCC27255_00018	96.67	90	3	0	1	90	1	90	4.00E-57	172
L2-63_00575	RBATCC27255_00264	94.02	117	7	0	1	117	1	117	7.00E-66	197
L2-63_00592	RBATCC27255_00140	98.58	282	4	0	1	282	1	282	0	552
L2-63_00593	RBATCC27255_00141	98.04	255	5	0	1	255	1	255	0	513
L2-63_00769	RBATCC27255_01887	96.43	336	12	0	1	336	1	336	0	678
L2-63_00771	RBATCC27255_01889	95.53	380	17	0	1	380	1	380	0	754
L2-63_00785	RBATCC27255_00613	93.38	151	10	0	1	151	1	151	4.00E-99	284
L2-63_00833	RBATCC27255_01851	96.8	219	7	0	1	219	1	219	8.00E-157	436
L2-63_00834	RBATCC27255_01850	91.1	146	13	0	1	146	1	146	5.00E-96	276
L2-63_00835	RBATCC27255_01849	98	100	2	0	1	100	1	100	2.00E-67	199
L2-63_00863	RBATCC27255_01811	95.11	184	9	0	1	184	1	184	3.00E-128	360
L2-63_00955	RBATCC27255_00979	100	261	0	0	1	261	1	261	0	545
L2-63_00972	RBATCC27255_00962	93.3	194	13	0	1	194	1	194	6.00E-131	368
L2-63_01010	RBATCC27255_00789	95.24	756	36	0	1	756	1	756	0	1399
L2-63_01012	RBATCC27255_00791	98.56	418	6	0	1	418	1	418	0	828
L2-63_01073	RBATCC27255_01953	95.68	139	6	0	1	139	1	139	1.00E-92	266
L2-63_01168	RBATCC27255_01426	100	102	0	0	1	102	1	102	9.00E-69	203
L2-63_01256	RBATCC27255_00511	97.67	86	2	0	1	86	1	86	1.00E-58	176

L2-63_01257	RBATCC27255_00510	100	66	0	0	1	66	1	66	1.00E-43	136
L2-63_01295	RBATCC27255_00917	98.27	231	4	0	1	231	1	231	2.00E-167	464
L2-63_01354	RBATCC27255_01015	99.01	101	1	0	1	101	1	101	2.00E-70	207
L2-63_01362	RBATCC27255_01007	98.65	296	4	0	1	296	1	296	0	605
L2-63_01395	RBATCC27255_00419	90.54	222	21	0	1	222	1	222	1.00E-142	400
L2-63_01486	RBATCC27255_01249	91.46	199	17	0	1	199	1	199	1.00E-138	388
L2-63_01504	RBATCC27255_00318	94.42	233	13	0	1	233	1	233	9.00E-162	449
L2-63_01505	RBATCC27255_00319	81.27	267	50	0	1	267	1	267	1.00E-155	437
L2-63_01507	RBATCC27255_00321	93.68	95	6	0	1	95	1	95	1.00E-51	159
L2-63_01534	RBATCC27255_01678	98.62	217	3	0	1	217	1	217	3.00E-156	434
L2-63_01542	RBATCC27255_01686	99.35	155	1	0	1	155	1	155	4.00E-110	312
L2-63_01585	RBATCC27255_00370	100	145	0	0	1	145	1	145	5.00E-107	303
L2-63_01630	RBATCC27255_00334	93.93	428	26	0	12	439	12	439	0	760
L2-63_01662	RBATCC27255_00445	95.88	340	14	0	1	340	1	340	0	670
L2-63_01663	RBATCC27255_00446	96.88	256	8	0	1	256	1	256	0	516
L2-63_01804	RBATCC27255_01654	98.89	180	2	0	1	180	1	180	4.00E-125	352
L2-63_01805	RBATCC27255_01653	97.32	149	4	0	1	149	1	149	9.00E-100	285
L2-63_01807	RBATCC27255_01651	98.66	372	5	0	1	372	1	372	0	706
L2-63_01808	RBATCC27255_01650	99.22	129	1	0	1	129	1	129	2.00E-82	239
L2-63_01809	RBATCC27255_01649	100	64	0	0	1	64	1	64	2.00E-37	120
L2-63_01810	RBATCC27255_01648	98.78	164	2	0	1	164	1	164	5.00E-116	328
L2-63_01811	RBATCC27255_01647	98.42	316	5	0	1	316	1	316	0	639
L2-63_01819	RBATCC27255_01638	88.71	124	14	0	25	148	1	124	2.00E-73	217
L2-63_01820	RBATCC27255_01636	94.44	90	5	0	1	90	1	90	1.00E-57	173
L2-63_01824	RBATCC27255_01632	97.57	618	15	0	1	618	1	618	0	1219
L2-63_01838	RBATCC27255_01615	94.86	311	16	0	1	311	1	311	0	614
L2-63_01842	RBATCC27255_01611	87.11	194	25	0	1	194	1	194	4.00E-116	330
L2-63_01843	RBATCC27255_01610	95.21	355	17	0	1	355	1	355	0	665
L2-63_01844	RBATCC27255_01609	96.07	484	19	0	1	484	1	484	0	957
L2-63_01857	RBATCC27255_01596	95.73	492	21	0	1	492	1	492	0	969
L2-63_01939	RBATCC27255_01142	92.6	392	29	0	1	392	1	392	0	751
L2-63_01940	RBATCC27255_01143	96.67	90	3	0	1	90	1	90	7.00E-60	179
L2-63_01941	RBATCC27255_01144	93.22	354	24	0	3	356	1	354	0	662
L2-63_01950	RBATCC27255_01151	96.51	918	32	0	1	918	1	918	0	1815
L2-63_00174	2595398400	24.29	140	97	3	32	171	7	137	1.00E-06	43.9
L2-63_00209	2595399457	93.85	260	16	0	1	260	1	260	1.00E-176	489
L2-63_00220	2595399501	84.44	180	28	0	1	180	1	180	1.00E-112	321
L2-63_00283	2595398729	68.73	291	91	0	1	291	1	291	7.00E-148	419
L2-63_00295	2595398956	56.97	251	99	4	1	242	1	251	6.00E-94	278
L2-63_00312	2595400464	98.46	65	1	0	1	65	1	65	2.00E-41	130
L2-63_00319	2595399509	75.92	191	46	0	1	191	1	191	6.00E-108	310
L2-63_00320	2595399510	72.73	165	45	0	1	165	1	165	3.00E-85	250
L2-63_00381	2595399462	67.62	772	248	2	1	772	1	770	0	1090
L2-63_00388	2595399148	23.15	337	222	14	106	435	73	379	5.00E-05	43.9
L2-63_00389	2595399706	59.89	531	186	7	1	530	1	505	0	558
L2-63_00437	2595399393	79.73	291	59	0	1	291	1	291	3.00E-168	471
L2-63_00438	2595399392	59.73	365	136	3	8	363	4	366	9.00E-160	455
L2-63_00444	2595399920	74.29	175	45	0	13	187	13	187	5.00E-90	263
L2-63_00486	2595398834	81.11	90	17	0	1	90	1	90	3.00E-49	152
L2-63_00575	2595400172	82.91	117	20	0	1	117	1	117	2.00E-64	193
L2-63_00592	2595398421	80.3	269	53	0	14	282	14	282	4.00E-158	444
L2-63_00593	2595398422	85.83	254	36	0	1	254	1	254	1.00E-164	458
L2-63_00769	2595399459	73.21	336	90	0	1	336	1	336	3.00E-170	479
L2-63_00771	2595399461	66.84	374	123	1	1	373	1	374	0	523
L2-63_00785	2595398783	76.82	151	35	0	1	151	1	151	4.00E-77	228
L2-63_00833	2595399524	76.04	217	52	0	2	218	3	219	1.00E-123	352
L2-63_00834	2595399525	81.75	137	25	0	5	141	5	141	5.00E-81	237
L2-63_00835	2595399526	77	100	23	0	1	100	7	106	6.00E-53	162
L2-63_00863	2595398441	42.39	184	105	1	1	183	1	184	9.00E-52	166
L2-63_00955	2595399157	78.12	256	56	0	1	256	1	256	1.00E-157	441
L2-63_00972	2595398017	60.44	182	72	0	8	189	8	189	7.00E-79	236
L2-63_01010	2595399249	73.13	748	199	2	10	756	11	757	0	1151
L2-63_01012	2595399251	79	419	87	1	1	418	1	419	0	664
L2-63_01073	2595400105	79.43	141	26	1	1	138	1	141	1.00E-66	201
L2-63_01256	2595398202	68.29	82	26	0	4	85	1	82	2.00E-39	127
L2-63_01257	2595398201	55.74	61	27	0	6	66	1	61	5.00E-21	78.6
L2-63_01295	2595398702	86.22	225	31	0	7	231	1	225	2.00E-142	400
L2-63_01354	2595400301	61.39	101	39	0	1	101	1	101	9.00E-44	139
L2-63_01362	2595398229	50.86	291	139	1	1	291	1	287	2.00E-105	311
L2-63_01395	2595397949	58.3	223	92	1	1	222	1	223	2.00E-85	254
L2-63_01486	2595399310	58.42	190	79	0	8	197	3	192	1.00E-82	246
L2-63_01504	2595400087	88.89	216	24	0	18	233	15	230	3.00E-138	390
L2-63_01505	2595400086	57.46	228	97	0	37	264	1	228	1.00E-93	278
L2-63_01507	2595398181	72.34	94	26	0	1	94	1	94	1.00E-46	146
L2-63_01534	2595398261	57.14	217	89	2	1	213	1	217	3.00E-87	259
L2-63_01542	2595398253	79.35	155	31	1	1	154	1	155	8.00E-82	240
L2-63_01630	2595399725	63.85	426	154	0	1	426	1	426	0	544
L2-63_01662	2595399596	59.23	336	134	2	1	335	5	338	4.00E-130	378
L2-63_01663	2595399595	79.22	255	53	0	1	255	1	255	6.00E-149	419
L2-63_01804	2595399285	67.4	181	58	1	1	180	1	181	5.00E-84	248
L2-63_01805	2595399284	72.97	148	40	0	2	149	3	150	2.00E-65	198
L2-63_01807	2595399282	59.52	373	150	1	1	372	1	373	1.00E-152	437
L2-63_01808	2595399281	69.84	126	38	0	1	126	1	126	1.00E-54	169
L2-63_01809	2595399280	92.19	64	5	0	1	64	1	64	2.00E-34	112
L2-63_01810	2595399279	47.77	157	80	1	10	164	1	157	4.00E-45	147
L2-63_01811	2595399278	60.86	304	119	0	9	312	9	312	2.00E-139	399
L2-63_01819	2595400262	70.09	107	31	1	43	148	2	108	2.00E-47	150
L2-63_01820	2595400260	77.78	90	20	0	1	90	1	90	3.00E-47	147
L2-63_01824	2595398673	61.83	613	217	4	6	617	2	598	0	760

L2-63_01838	2595398659	74.19	310	80	0	1	310	1	310	1.00E-173	486
L2-63_01842	2595398655	33.51	194	125	2	1	191	1	193	3.00E-36	126
L2-63_01843	2595398654	30.51	354	239	2	1	352	1	349	2.00E-49	171
L2-63_01844	2595398653	71.64	483	137	0	1	483	4	486	0	709
L2-63_01857	2595398642	79.88	492	99	0	1	492	1	492	0	840
L2-63_01939	2595398762	49.23	392	199	0	1	392	1	392	9.00E-138	401
L2-63_01940	2595398761	63.33	90	31	1	3	90	2	91	1.00E-32	110
L2-63_01941	2595398760	51.81	359	166	2	1	356	1	355	4.00E-124	363
L2-63_01950	2595398753	78.67	661	119	6	266	918	344	990	0	1032
L2-63_02000	2595398708	62.95	278	88	3	1	278	1	263	2.00E-117	340
L2-63_02125	2595400026	49.82	273	134	2	2	272	9	280	7.00E-91	273
L2-63_02125	2595399002	69.18	279	72	5	2	273	5	276	2.00E-132	379
L2-63_00109	L2-63_00109	100	315	0	0	1	315	1	315	0	640
L2-63_00110	L2-63_00110	100	275	0	0	1	275	1	275	0	561
L2-63_00174	L2-63_00174	100	174	0	0	1	174	1	174	1.00E-128	360
L2-63_00209	L2-63_00209	100	260	0	0	1	260	1	260	0	525
L2-63_00220	L2-63_00220	100	180	0	0	1	180	1	180	6.00E-129	362
L2-63_00283	L2-63_00283	100	291	0	0	1	291	1	291	0	582
L2-63_00295	L2-63_00295	100	242	0	0	1	242	1	242	1.00E-179	496
L2-63_00312	L2-63_00312	100	65	0	0	1	65	1	65	1.00E-41	131
L2-63_00319	L2-63_00319	100	194	0	0	1	194	1	194	4.00E-139	389
L2-63_00320	L2-63_00320	100	166	0	0	1	166	1	166	1.00E-117	332
L2-63_00354	L2-63_00354	100	243	0	0	1	243	1	243	0	499
L2-63_00381	L2-63_00381	100	774	0	0	1	774	1	774	0	1576
L2-63_00388	L2-63_00388	100	547	0	0	1	547	1	547	0	1129
L2-63_00389	L2-63_00389	100	530	0	0	1	530	1	530	0	1074
L2-63_00437	L2-63_00437	100	291	0	0	1	291	1	291	0	578
L2-63_00438	L2-63_00438	100	365	0	0	1	365	1	365	0	749
L2-63_00444	L2-63_00444	100	187	0	0	1	187	1	187	2.00E-130	366
L2-63_00486	L2-63_00486	100	90	0	0	1	90	1	90	8.00E-60	179
L2-63_00575	L2-63_00575	100	117	0	0	1	117	1	117	9.00E-77	224
L2-63_00592	L2-63_00592	100	282	0	0	1	282	1	282	0	560
L2-63_00593	L2-63_00593	100	255	0	0	1	255	1	255	0	520
L2-63_00769	L2-63_00769	100	336	0	0	1	336	1	336	0	696
L2-63_00771	L2-63_00771	100	380	0	0	1	380	1	380	0	781
L2-63_00785	L2-63_00785	100	151	0	0	1	151	1	151	6.00E-105	298
L2-63_00833	L2-63_00833	100	219	0	0	1	219	1	219	1.00E-160	445
L2-63_00834	L2-63_00834	100	146	0	0	1	146	1	146	3.00E-106	301
L2-63_00835	L2-63_00835	100	100	0	0	1	100	1	100	5.00E-69	203
L2-63_00863	L2-63_00863	100	184	0	0	1	184	1	184	1.00E-134	376
L2-63_00955	L2-63_00955	100	261	0	0	1	261	1	261	0	545
L2-63_00972	L2-63_00972	100	196	0	0	1	196	1	196	1.00E-142	398
L2-63_01010	L2-63_01010	100	756	0	0	1	756	1	756	0	1548
L2-63_01012	L2-63_01012	100	418	0	0	1	418	1	418	0	836
L2-63_01073	L2-63_01073	100	139	0	0	1	139	1	139	8.00E-97	277
L2-63_01168	L2-63_01168	100	102	0	0	1	102	1	102	8.00E-69	203
L2-63_01256	L2-63_01256	100	87	0	0	1	87	1	87	3.00E-60	180
L2-63_01257	L2-63_01257	100	66	0	0	1	66	1	66	1.00E-43	136
L2-63_01295	L2-63_01295	100	231	0	0	1	231	1	231	6.00E-170	470
L2-63_01354	L2-63_01354	100	101	0	0	1	101	1	101	5.00E-71	208
L2-63_01362	L2-63_01362	100	296	0	0	1	296	1	296	0	610
L2-63_01395	L2-63_01395	100	222	0	0	1	222	1	222	2.00E-158	440
L2-63_01486	L2-63_01486	100	200	0	0	1	200	1	200	8.00E-149	414
L2-63_01504	L2-63_01504	100	233	0	0	1	233	1	233	6.00E-171	473
L2-63_01505	L2-63_01505	100	267	0	0	1	267	1	267	0	522
L2-63_01507	L2-63_01507	100	95	0	0	1	95	1	95	1.00E-64	192
L2-63_01534	L2-63_01534	100	217	0	0	1	217	1	217	3.00E-158	439
L2-63_01542	L2-63_01542	100	155	0	0	1	155	1	155	8.00E-111	314
L2-63_01585	L2-63_01585	100	145	0	0	1	145	1	145	5.00E-107	303
L2-63_01630	L2-63_01630	100	439	0	0	1	439	1	439	0	879
L2-63_01662	L2-63_01662	100	340	0	0	1	340	1	340	0	694
L2-63_01663	L2-63_01663	100	256	0	0	1	256	1	256	0	526
L2-63_01701	L2-63_01701	100	274	0	0	1	274	1	274	0	561
L2-63_01702	L2-63_01702	100	313	0	0	1	313	1	313	0	640
L2-63_01804	L2-63_01804	100	180	0	0	1	180	1	180	2.00E-127	358
L2-63_01805	L2-63_01805	100	149	0	0	1	149	1	149	2.00E-102	292
L2-63_01807	L2-63_01807	100	372	0	0	1	372	1	372	0	717
L2-63_01808	L2-63_01808	100	129	0	0	1	129	1	129	1.00E-82	240
L2-63_01809	L2-63_01809	100	64	0	0	1	64	1	64	2.00E-37	120
L2-63_01810	L2-63_01810	100	164	0	0	1	164	1	164	3.00E-117	331
L2-63_01811	L2-63_01811	100	316	0	0	1	316	1	316	0	645
L2-63_01819	L2-63_01819	100	148	0	0	1	148	1	148	8.00E-100	285
L2-63_01820	L2-63_01820	100	90	0	0	1	90	1	90	2.00E-61	183
L2-63_01824	L2-63_01824	100	618	0	0	1	618	1	618	0	1241
L2-63_01838	L2-63_01838	100	311	0	0	1	311	1	311	0	642
L2-63_01842	L2-63_01842	100	196	0	0	1	196	1	196	4.00E-137	384
L2-63_01843	L2-63_01843	100	355	0	0	1	355	1	355	0	692
L2-63_01844	L2-63_01844	100	484	0	0	1	484	1	484	0	990
L2-63_01857	L2-63_01857	100	492	0	0	1	492	1	492	0	1004
L2-63_01939	L2-63_01939	100	392	0	0	1	392	1	392	0	800
L2-63_01940	L2-63_01940	100	90	0	0	1	90	1	90	2.00E-61	183
L2-63_01941	L2-63_01941	100	356	0	0	1	356	1	356	0	702
L2-63_01950	L2-63_01950	100	918	0	0	1	918	1	918	0	1869
L2-63_02000	L2-63_02000	100	281	0	0	1	281	1	281	0	583
L2-63_02125	L2-63_02125	100	273	0	0	1	273	1	273	0	556

qseqid query (e.g., gene) sequence id	sseqid subject (e.g., reference genome) sequence id	pident percentage of identical matches	length alignment length	mismatch number of mismatches	gapopen number of gap openings	qstart start of alignment in query	qend end of alignment in query	sstart start of alignment in subject	send end of alignment in subject	evalue expect value	bitscore bit score
CD630_00010	L2-63_00603	42.02	445	243	4	7	438	21	463	1.00E-121	364
CD630_00010	RBL236_00278	42.02	445	243	4	7	438	21	463	1.00E-121	364
CD630_00010	RB5AMG_01741	42.02	445	243	4	7	438	21	463	1.00E-121	364
CD630_00010	RBATCC27255_00151	42.79	444	243	3	7	439	8	451	8.00E-124	369
CD630_00010	2595398460	44.67	441	226	4	7	438	8	439	2.00E-131	389
CD630_00240	L2-63_00984	32.94	170	100	4	1	160	1	166	1.00E-22	89
CD630_00240	RBL236_00618	32.94	170	100	4	1	160	1	166	1.00E-22	89
CD630_00240	RB5AMG_01276	32.94	170	100	4	1	160	1	166	1.00E-22	89
CD630_00240	RBATCC27255_00956	33.53	170	99	4	1	160	1	166	7.00E-23	89.4
CD630_00240	2595398677	34.52	168	103	1	1	161	1	168	9.00E-29	105
CD630_00550	L2-63_00456	51.9	237	114	0	7	243	17	253	8.00E-89	265
CD630_00550	RBL236_02070	51.9	237	114	0	7	243	17	253	9.00E-89	265
CD630_00550	RB5AMG_00968	51.9	237	114	0	7	243	17	253	9.00E-89	265
CD630_00550	RBATCC27255_01756	52.32	237	113	0	7	243	17	253	2.00E-89	267
CD630_00550	2595399512	52.74	237	112	0	7	243	13	249	6.00E-88	263
CD630_00570	RB5AMG_01287	30.48	210	120	4	11	215	5	193	1.00E-23	93.6
CD630_00570	RBATCC27255_00962	30.81	211	120	4	11	216	5	194	2.00E-26	101
CD630_00570	L2-63_00972	30.95	210	119	4	11	215	5	193	7.00E-24	94.4
CD630_00570	RBL236_00630	30.95	210	119	4	11	215	5	193	7.00E-24	94.4
CD630_00570	2595398017	33.01	209	117	4	6	211	1	189	2.00E-26	101
CD630_00630	2595399580	40.76	157	92	1	5	161	7	162	2.00E-35	123
CD630_00630	L2-63_00169	40.76	157	92	1	5	161	7	162	1.00E-34	120
CD630_00630	RBL236_01560	40.76	157	92	1	5	161	7	162	1.00E-34	120
CD630_00630	RB5AMG_01844	40.76	157	92	1	5	161	7	162	1.00E-34	120
CD630_00630	RBATCC27255_00759	41.4	157	91	1	5	161	7	162	4.00E-35	122
CD630_05720	L2-63_01601	27.63	76	48	2	137	207	144	217	9.2	25.8
CD630_05720	2595400425	30	60	39	2	154	213	428	484	5.3	26.6
CD630_05720	RB5AMG_00626	32.5	40	27	0	148	187	41	80	6.3	26.2
CD630_05720	L2-63_01295	32.5	40	27	0	148	187	41	80	6.6	26.2
CD630_05720	RBL236_00759	32.5	40	27	0	148	187	41	80	6.6	26.2
CD630_05980	RB5AMG_01153	47.29	129	68	0	49	177	1	129	3.00E-36	125
CD630_05980	L2-63_01241	48.06	129	67	0	49	177	1	129	3.00E-37	127
CD630_05980	RBL236_00705	48.06	129	67	0	49	177	1	129	3.00E-37	127
CD630_05980	RBATCC27255_00524	48.06	129	67	0	49	177	1	129	4.00E-37	127
CD630_05980	2595398203	53.11	177	83	0	1	177	1	177	6.00E-67	206
CD630_07720	L2-63_00209	40.82	245	139	2	13	253	18	260	1.00E-55	181
CD630_07720	RBL236_01600	40.82	245	139	2	13	253	18	260	1.00E-55	181
CD630_07720	RB5AMG_01804	40.82	245	139	2	13	253	18	260	1.00E-55	181
CD630_07720	RBATCC27255_01940	41.22	245	138	2	13	253	18	260	7.00E-57	184
CD630_07720	2595399457	43.27	245	133	2	13	253	18	260	6.00E-58	186
CD630_07740	RB5AMG_01550	45.29	329	180	0	4	332	3	331	5.00E-97	293
CD630_07740	L2-63_00769	45.29	329	180	0	4	332	3	331	1.00E-96	291
CD630_07740	RBL236_00442	45.29	329	180	0	4	332	3	331	1.00E-96	291
CD630_07740	RB5AMG_01550	45.29	329	180	0	4	332	3	331	5.00E-97	293
CD630_07740	L2-63_00769	45.29	329	180	0	4	332	3	331	1.00E-96	291
CD630_07740	RBL236_00442	45.29	329	180	0	4	332	3	331	1.00E-96	291
CD630_07740	RBATCC27255_01887	45.32	331	181	0	4	334	3	333	1.00E-97	294
CD630_07740	RBATCC27255_01887	45.32	331	181	0	4	334	3	333	1.00E-97	294
CD630_07740	2595399459	47.43	331	174	0	4	334	3	333	3.00E-97	293
CD630_07740	2595399459	47.43	331	174	0	4	334	3	333	3.00E-97	293
CD630_08810	2595399340	50.15	329	129	5	9	337	16	309	2.00E-103	309
CD630_08810	L2-63_01672	53.57	308	113	4	21	328	20	297	1.00E-103	309
CD630_08810	RBL236_01298	53.57	308	113	4	21	328	20	297	1.00E-103	309
CD630_08810	RB5AMG_01665	54.69	245	81	4	84	328	1	215	9.00E-77	237
CD630_08810	RBATCC27255_00455	55.87	281	94	4	48	328	1	251	1.00E-96	289
CD630_10450	2595399725	21.21	330	220	8	36	336	41	359	5.00E-05	43.1
CD630_10450	L2-63_01630	21.67	263	170	8	87	332	114	357	0.007	36.2
CD630_10450	RBL236_01338	21.67	263	170	8	87	332	114	357	0.007	36.2
CD630_10450	RB5AMG_00155	21.67	263	170	8	87	332	114	357	0.007	36.2
CD630_10450	RBATCC27255_00334	22.05	263	169	8	87	332	114	357	0.004	37
CD630_10870	L2-63_01176	26.82	261	156	10	16	261	14	254	5.00E-21	88.2
CD630_10870	RBL236_01933	26.82	261	156	10	16	261	14	254	5.00E-21	88.2
CD630_10870	RB5AMG_01070	26.82	261	156	10	16	261	14	254	7.00E-21	87.8
CD630_10870	RBATCC27255_01420	27.2	261	155	10	16	261	14	254	8.00E-22	90.5
CD630_10870	2595398363	27.42	62	45	0	7	68	183	244	6.2	26.2
CD630_10870	2595398363	30.54	239	142	10	16	245	17	240	2.00E-20	86.7
CD630_11920	RBATCC27255_01647	34.86	284	172	5	29	310	35	307	6.00E-52	175
CD630_11920	RB5AMG_00699	34.86	284	172	5	29	310	35	307	6.00E-52	175
CD630_11920	L2-63_01811	34.86	284	172	5	29	310	35	307	9.00E-51	172
CD630_11920	RBL236_01622	34.86	284	172	5	29	310	35	307	1.00E-50	172
CD630_11920	2595399278	37.58	306	179	5	12	315	17	312	2.00E-62	202
CD630_11930	2595399279	22.7	163	119	2	11	173	2	157	3.00E-06	43.1
CD630_11930	RB5AMG_00700	23.7	173	123	2	1	173	1	164	4.00E-06	43.1
CD630_11930	L2-63_01810	23.7	173	123	2	1	173	1	164	5.00E-06	42.7
CD630_11930	RBL236_01621	23.7	173	123	2	1	173	1	164	5.00E-06	42.7
CD630_11930	RBATCC27255_01648	23.7	173	123	2	1	173	1	164	7.00E-06	42.4
CD630_11940	2595399280	46.77	62	33	0	1	62	1	62	6.00E-16	65.5
CD630_11940	L2-63_01809	48.39	62	32	0	1	62	1	62	4.00E-16	65.9
CD630_11940	RBL236_01620	48.39	62	32	0	1	62	1	62	4.00E-16	65.9
CD630_11940	RBATCC27255_01649	48.39	62	32	0	1	62	1	62	4.00E-16	65.9
CD630_11940	RB5AMG_00701	48.39	62	32	0	1	62	1	62	4.00E-16	65.9
CD630_11950	RBATCC27255_01650	30.16	126	88	0	1	126	1	126	6.00E-16	68.9
CD630_11950	L2-63_01808	30.16	126	88	0	1	126	1	126	9.00E-16	68.6
CD630_11950	RBL236_01619	30.16	126	88	0	1	126	1	126	9.00E-16	68.6
CD630_11950	RB5AMG_00702	30.16	126	88	0	1	126	1	126	9.00E-16	68.6
CD630_11950	2595399281	31.5	127	84	1	4	127	1	127	2.00E-15	67.8
CD630_11960	2595399282	27.03	111	76	2	230	338	201	308	8.00E-07	48.9
CD630_11960	L2-63_01807	30.36	112	71	4	230	338	200	307	1.00E-06	48.1
CD630_11960	RBL236_01618	30.36	112	71	4	230	338	200	307	1.00E-06	48.1
CD630_11960	RB5AMG_00703	30.36	112	71	4	230	338	200	307	1.00E-06	48.1
CD630_11960	RBATCC27255_01651	30.36	112	71	4	230	338	200	307	1.00E-06	48.1
CD630_11980	2595399284	26.5	117	51	2	63	179	62	143	8.00E-06	42
CD630_11980	L2-63_01805	28.81	118	49	2	64	181	62	144	2.00E-07	46.6
CD630_11980	RBL236_01616	28.81	118	49	2	64	181	62	144	2.00E-07	46.6
CD630_11980	RBATCC27255_01653	28.81	118	49	2	64	181	62	144	2.00E-07	46.6
CD630_11980	RB5AMG_00705	28.81	118	49	2	64	181	62	144	3.00E-07	45.8
CD630_11990	2595399285	27.83	115	83	0	110	224	66	180	3.00E-08	50.1
CD630_11990	RB5AMG_00706	27.86	140	95	2	85	222	42	177	2.00E-09	53.5
CD630_11990	RBATCC27255_01654	27.86	140	95	2	85	222	42	177	2.00E-09	53.5
CD630_11990	L2-63_01804	27.86	140	95	2	85	222	42	177	2.00E-09	53.1
CD630_11990	RBL236_01615	27.86	140	95	2	85	222	42	177	2.00E-09	53.1
CD630_12120	L2-63_01251	27.38	84	57	2	47	127	62	144	3.00E-04	37
CD630_12120	RBL236_00715	27.38	84	57	2	47	127	62	144	3.00E-04	37
CD630_12120	2595398409	28.05	82	57	2	48	127	64	145	8.00E-04	36.2
CD630_12120	RB5AMG_01163	32.69	52	35	0	76	127	31	82	6.00E-04	35.8
CD630_12120	2595398387	33.33	81	50	3	48	127	53	130	1.00E-04	38.1
CD630_12130	2595399596	34.19	313	188	7	48	351	34	337	2.00E-49	170
CD630_12130	RBATCC27255_00445	38.49	317	172	8	48	352	30	335	9.00E-57	189
CD630_12130	RB5AMG_01655	39.49	314	167	8	48	349	30	332	6.00E-57	190
CD630_12130	L2-63_01662	39.81	314	166	8	48	349	30	332	1.00E-57	191
CD630_12130	RBL236_01308	39.81	314	166	8	48	349	30	332	1.00E-57	191
CD630_12140	2595399595	42.26	265	139	4	5	269	1	251	1.00E-69	217
CD630_12140	L2-63_01663	44.27	262	132	6	8	269	4	251	9.00E-73	225
CD630_12140	RBL236_01307	44.27	262	132	6	8	269	4	251	9.00E-73	225
CD630_12140	RB5AMG_01656	44.27	262	132	6	8	269	4	251	9.00E-73	225
CD630_12140	RBATCC27255_00446	44.66	262	131	6	8	269	4	251	1.00E-72	225
CD630_12150	RBATCC27255_00859	32.2	59	39	1	159	217	47	104	1	29.3
CD630_12150	L2-63_00990	32.2	59	39	1	159	217	47	104	1.1	29.3
CD630_12150	RBL236_00612	32.2	59	39	1	159	217	47	104	1.1	29.3
CD630_12150	RB5AMG_01270	32.2	59	39	1	159	217	47	104	1.1	29.3
CD630_12150	RBATCC27255_01269	40	35	19	1	190	222	19	53	1.9	28.1
CD630_12300	RB5AMG_00626	49.05	210	99	3	6	215	24	225	1.00E-58	186
CD630_12300	L2-63_01295	49.05	210	99	3	6	215	24	225	2.00E-58	186
CD630_12300	RBL236_00759	49.05	210	99	3	6	215	24	225	2.00E-58	186
CD630_12300	RBATCC27255_00917	49.52	210	98	3	6	215	24	225	1.00E-58	186

CD630_12300	2595398702	51.43	210	94	3	6	215	18	219	5.00E-62	195
CD630_12910	L2-63_00292	41.71	386	216	3	2	387	4	380	1.00E-98	300
CD630_12910	RBL236_01812	41.71	386	216	3	2	387	4	380	1.00E-98	300
CD630_12910	RB5AMG_00097	41.97	386	215	3	2	387	4	380	3.00E-99	302
CD630_12910	RBATCC27255_00285	41.97	386	215	3	2	387	4	380	3.00E-98	299
CD630_12910	2595398736	46.23	318	167	1	35	352	30	343	2.00E-95	292
CD630_13220	RBATCC27255_00773	32.78	421	246	9	1	405	1	400	3.00E-62	207
CD630_13220	2595399046	32.78	421	246	10	1	405	1	400	1.00E-61	206
CD630_13220	L2-63_00138	33.02	421	245	9	1	405	1	400	1.00E-57	195
CD630_13220	RBL236_01529	33.02	421	245	9	1	405	1	400	1.00E-57	195
CD630_13220	RB5AMG_01873	33.02	421	245	9	1	405	1	400	1.00E-57	195
CD630_13230	RBATCC27255_01153	55.96	193	85	0	45	237	60	252	4.00E-74	228
CD630_13230	RB5AMG_02156	56.06	198	87	0	44	241	59	256	6.00E-77	235
CD630_13230	2595398751	56.35	197	86	0	44	240	56	252	2.00E-75	231
CD630_13230	L2-63_01952	56.57	198	86	0	44	241	59	256	2.00E-77	237
CD630_13230	RBL236_00681	56.57	198	86	0	44	241	59	256	2.00E-77	237
CD630_13340	2595399328	61.85	464	176	1	8	470	5	468	0	612
CD630_13340	RBATCC27255_01240	62.34	462	173	1	8	468	5	466	0	611
CD630_13340	L2-63_01694	62.55	462	172	1	8	468	5	466	0	613
CD630_13340	RBL236_01276	62.55	462	172	1	8	468	5	466	0	613
CD630_13340	RB5AMG_00760	62.77	462	171	1	8	468	5	466	0	613
CD630_15670	RB5AMG_01153	31.21	141	91	3	52	189	4	141	2.00E-15	70.1
CD630_15670	L2-63_01241	31.91	141	90	3	52	189	4	141	9.00E-16	70.9
CD630_15670	RBL236_00705	31.91	141	90	3	52	189	4	141	9.00E-16	70.9
CD630_15670	RBATCC27255_00524	31.91	141	90	3	52	189	4	141	1.00E-15	70.5
CD630_15670	2595398203	32.29	192	124	3	1	189	1	189	3.00E-29	109
CD630_16310	L2-63_00922	25.71	140	85	5	68	195	6	138	0.12	31.6
CD630_16310	RBL236_00976	25.71	140	85	5	68	195	6	138	0.12	31.6
CD630_16310	RBATCC27255_00838	25.71	140	85	5	68	195	6	138	0.12	31.6
CD630_16310	RB5AMG_01333	25.71	140	85	5	68	195	6	138	0.12	31.6
CD630_16310	2595398971	27.03	111	67	4	68	166	6	114	0.72	29.3
CD630_16910	L2-63_01154	32.34	303	185	11	6	291	2	301	1.00E-36	133
CD630_16910	RBL236_01911	32.34	303	185	11	6	291	2	301	1.00E-36	133
CD630_16910	RB5AMG_01052	32.67	303	184	11	6	291	2	301	2.00E-37	135
CD630_16910	RBATCC27255_01473	33	303	183	11	6	291	2	301	3.00E-38	137
CD630_16910	2595399026	51.37	292	137	2	3	294	1	287	4.00E-104	308
CD630_20350	2595399725	26.02	342	201	14	38	345	39	362	4.00E-05	43.5
CD630_20350	RBATCC27255_00334	28.28	198	120	6	154	345	181	362	0.001	38.9
CD630_20350	L2-63_01630	28.79	198	119	6	154	345	181	362	0.006	36.6
CD630_20350	RBL236_01338	28.79	198	119	6	154	345	181	362	0.006	36.6
CD630_20350	RB5AMG_00155	28.79	198	119	6	154	345	181	362	0.006	36.6
CD630_20480	2595399270	48.75	279	142	1	12	289	4	282	2.00E-94	283
CD630_20480	L2-63_01446	49.1	279	141	1	12	289	4	282	9.00E-95	284
CD630_20480	RBL236_00023	49.1	279	141	1	12	289	4	282	9.00E-95	284
CD630_20480	RB5AMG_00246	49.1	279	141	1	12	289	4	282	9.00E-95	284
CD630_20480	RBATCC27255_01289	49.1	279	141	1	12	289	4	282	9.00E-95	284
CD630_21080	RBATCC27255_01415	30.7	456	296	6	1	444	7	454	4.00E-75	244
CD630_21080	RB5AMG_01076	30.7	456	296	6	1	444	7	454	5.00E-75	243
CD630_21080	L2-63_01183	30.7	456	296	6	1	444	7	454	1.00E-74	243
CD630_21080	RBL236_01939	30.7	456	296	6	1	444	7	454	1.00E-74	243
CD630_21080	L2-63_00325	33.71	448	286	6	2	443	3	445	2.00E-72	236
CD630_22310	L2-63_01148	30.14	292	183	6	12	301	10	282	3.00E-38	137
CD630_22310	RBL236_01905	30.14	292	183	6	12	301	10	282	3.00E-38	137
CD630_22310	RB5AMG_01046	30.48	292	182	6	12	301	10	282	6.00E-39	139
CD630_22310	RBATCC27255_01479	30.48	292	182	6	12	301	10	282	1.00E-38	139
CD630_22310	RB5AMG_00488	35.14	74	38	3	147	220	221	284	4.00E-06	45.8
CD630_22730	2595399725	24.05	370	233	14	7	339	8	366	4.00E-05	43.1
CD630_22730	RBATCC27255_00334	30.05	203	122	10	148	339	173	366	3.00E-05	43.5
CD630_22730	L2-63_01630	40	45	26	1	296	339	322	366	0.007	36.2
CD630_22730	RBL236_01338	40	45	26	1	296	339	322	366	0.007	36.2
CD630_22730	RB5AMG_00155	40	45	26	1	296	339	322	366	0.007	36.2
CD630_24430	2595399692	25.86	58	40	1	9	63	9	66	2.1	24.6
CD630_24430	2595398761	30.77	65	45	0	15	79	26	90	1.00E-05	37.7
CD630_24430	2595398632	36.36	33	21	0	39	71	142	174	0.29	27.3
CD630_24430	2595398629	37.84	37	23	0	9	45	736	772	2.1	24.6
CD630_24430	2595399516	40	20	12	0	48	67	181	200	1.5	25
CD630_24690	RB5AMG_00987	26.84	313	195	9	42	331	58	359	7.00E-22	93.2
CD630_24690	L2-63_00438	27.01	311	197	8	42	331	58	359	1.00E-21	92.4
CD630_24690	RBL236_02088	27.01	311	197	8	42	331	58	359	1.00E-21	92.4
CD630_24690	2595399392	28.64	213	130	7	132	331	159	362	6.00E-22	93.2
CD630_24690	RBATCC27255_01876	30	210	127	6	134	331	158	359	1.00E-21	92.4
CD630_24700	L2-63_00437	36.94	314	160	6	2	312	1	279	2.00E-54	180
CD630_24700	RBL236_02089	36.94	314	160	6	2	312	1	279	2.00E-54	180
CD630_24700	RB5AMG_00988	36.94	314	160	6	2	312	1	279	8.00E-54	179
CD630_24700	2595399393	37.58	314	158	6	2	312	1	279	6.00E-56	184
CD630_24700	RBATCC27255_01877	38.22	314	156	6	2	312	1	279	1.00E-55	184
CD630_24980	RBATCC27255_01952	31.58	380	237	7	1	379	1	358	9.00E-46	163
CD630_24980	RB5AMG_02141	32.19	379	224	12	1	360	5	369	2.00E-46	166
CD630_24980	L2-63_01935	32.19	379	224	12	1	360	5	369	4.00E-46	166
CD630_24980	RBL236_00664	32.19	379	224	12	1	360	5	369	4.00E-46	166
CD630_24980	RBATCC27255_01138	32.63	377	221	12	3	360	7	369	7.00E-47	167
CD630_25890	RBATCC27255_01282	40.82	294	173	1	1	293	1	294	3.00E-74	231
CD630_25890	2595400084	40.82	294	173	1	1	293	1	294	4.00E-73	228
CD630_25890	L2-63_01453	41.5	294	171	1	1	293	1	294	6.00E-77	238
CD630_25890	RBL236_01515	41.5	294	171	1	1	293	1	294	6.00E-77	238
CD630_25890	RB5AMG_00239	41.5	294	171	1	1	293	1	294	7.00E-77	238
CD630_26220	2595398253	29.41	153	98	4	4	147	3	154	3.00E-16	70.9
CD630_26220	RBATCC27255_01686	29.61	152	98	5	4	147	3	153	4.00E-17	73.2
CD630_26220	L2-63_01542	29.61	152	98	5	4	147	3	153	8.00E-17	72.4
CD630_26220	RBL236_01426	29.61	152	98	5	4	147	3	153	9.00E-17	72.4
CD630_26220	RB5AMG_00834	29.61	152	98	5	4	147	3	153	9.00E-17	72.4
CD630_26290	RBATCC27255_01596	47.34	488	257	0	4	491	5	492	0	521
CD630_26290	L2-63_01857	47.95	488	254	0	4	491	5	492	0	520
CD630_26290	RBL236_01668	47.95	488	254	0	4	491	5	492	0	520
CD630_26290	RB5AMG_00651	48.16	488	253	0	4	491	5	492	0	522
CD630_26290	2595398642	49.8	488	245	0	4	491	5	492	0	528
CD630_26420	RBATCC27255_01940	60.54	261	98	2	1	257	1	260	7.00E-113	327
CD630_26420	L2-63_00209	60.92	261	97	2	1	257	1	260	2.00E-112	326
CD630_26420	RBL236_01600	60.92	261	97	2	1	257	1	260	2.00E-112	326
CD630_26420	RB5AMG_01804	60.92	261	97	2	1	257	1	260	2.00E-112	326
CD630_26420	2595399457	62.07	261	94	2	1	257	1	260	1.00E-113	329
CD630_26430	RB5AMG_00794	57.78	225	93	2	17	240	10	233	9.00E-81	244
CD630_26430	RBATCC27255_00318	58.22	225	92	2	17	240	10	233	5.00E-82	247
CD630_26430	L2-63_01504	58.22	225	92	2	17	240	10	233	3.00E-81	245
CD630_26430	RBL236_01465	58.22	225	92	2	17	240	10	233	3.00E-81	245
CD630_26430	2595400087	60.93	215	82	2	27	240	17	230	8.00E-83	249
CD630_26440	2595400086	23.21	237	131	7	36	256	2	203	2.00E-08	52
CD630_26440	RBATCC27255_00319	23.43	286	153	6	2	263	4	247	1.00E-13	67.4
CD630_26440	L2-63_01505	23.43	286	153	6	2	263	4	247	2.00E-11	61.2
CD630_26440	RBL236_01464	23.43	286	153	6	2	263	4	247	2.00E-11	61.2
CD630_26440	RB5AMG_00795	23.78	286	152	6	2	263	4	247	5.00E-12	62.8
CD630_26500	2595399254	27.06	218	151	4	34	244	168	384	8.00E-12	62.4
CD630_26500	L2-63_01015	27.83	115	79	2	15	128	157	268	2.00E-08	52.4
CD630_26500	RBL236_00587	27.83	115	79	2	15	128	157	268	2.00E-08	52.4
CD630_26500	RB5AMG_01246	27.83	115	79	2	15	128	157	268	3.00E-08	52
CD630_26500	RBATCC27255_00794	28.46	130	84	3	1	128	146	268	2.00E-08	52.8
CD630_26520	2595399251	39.33	356	209	3	27	376	63	417	6.00E-68	221
CD630_26520	RBATCC27255_00791	39.39	358	205	3	27	376	63	416	1.00E-66	218
CD630_26520	L2-63_01012	39.66	358	204	3	27	376	63	416	3.00E-67	220
CD630_26520	RBL236_00590	39.66	358	204	3	27	376	63	416	3.00E-67	220
CD630_26520	RB5AMG_01249	39.77	352	200	3	33	376	69	416	7.00E-66	216
CD630_29680	2595398729	31.19	295	171	6	9	288	11	288	2.00E-39	140
CD630_29680	RB5AMG_00088	34.84	287	171	6	9	288	11	288	2.00E-43	150
CD630_29680	L2-63_00283	35.17	290	166	7	9	288	11	288	8.00E-44	152
CD630_29680	RBL236_01803	35.17	290	166	7	9	288	11	288	8.00E-44	152
CD630_29680	RBATCC27255_00276	35.52	290	165	7	9	288	11	288	4.00E-44	152
CD630_31200	L2-63_00668	50.4	125	59	2	1	122	1	125	7.00E-38	127
CD630_31200	RBL236_00342	50.4	125	59	2	1	122	1	125	8.00E-38	127
CD630_31200	RB5AMG_01976	50.4	125	59	2	1	122	1	125	8.00E-38	127

CD630_31200	2595398499	50.77	65	32	0	2	66	5	69	2.00E-13	63.9
CD630_31200	RBATCC27255_01718	52	125	57	2	1	122	1	125	1.00E-38	128
CD630_34940	2595400260	39.76	83	49	1	2	84	9	90	1.00E-14	63.2
CD630_34940	L2-63_01820	41.46	82	47	1	3	84	10	90	6.00E-16	66.6
CD630_34940	RBL236_01631	41.46	82	47	1	3	84	10	90	6.00E-16	66.6
CD630_34940	RB5AMG_00690	41.46	82	47	1	3	84	10	90	6.00E-16	66.6
CD630_34940	RBATCC27255_01636	41.46	82	47	1	3	84	10	90	9.00E-16	66.2
CD630_34990	RB5AMG_00070	38.67	150	90	2	31	179	589	737	1.00E-27	108
CD630_34990	2595399501	48.33	180	91	2	1	179	1	179	2.00E-50	162
CD630_34990	L2-63_00220	48.89	180	90	2	1	179	1	179	5.00E-53	168
CD630_34990	RBL236_01740	48.89	180	90	2	1	179	1	179	6.00E-53	168
CD630_34990	RBATCC27255_00711	48.89	180	90	2	1	179	1	179	6.00E-53	168
CD630_35410	RB5AMG_00336	31.71	164	112	0	9	172	1	164	6.00E-32	113
CD630_35410	L2-63_00320	32.48	157	106	0	9	165	1	157	1.00E-31	112
CD630_35410	RBL236_02206	32.48	157	106	0	9	165	1	157	1.00E-31	112
CD630_35410	RBATCC27255_00931	32.48	157	106	0	9	165	1	157	1.00E-31	112
CD630_35410	2595399510	33.54	164	109	0	9	172	1	164	2.00E-33	117
CD630_35420	L2-63_00319	37.43	187	116	1	7	192	5	191	2.00E-43	144
CD630_35420	RBL236_02207	37.43	187	116	1	7	192	5	191	3.00E-43	144
CD630_35420	RBATCC27255_00930	37.43	187	116	1	7	192	5	191	3.00E-43	144
CD630_35420	RB5AMG_00335	37.43	187	116	1	7	192	5	191	3.00E-43	144
CD630_35420	2595399509	41.88	191	110	1	3	192	1	191	3.00E-48	157
CD630_35640	L2-63_01486	37.75	204	112	3	10	213	8	196	2.00E-43	145
CD630_35640	RBL236_01482	37.75	204	112	3	10	213	8	196	2.00E-43	145
CD630_35640	RB5AMG_00206	37.75	204	112	3	10	213	8	196	2.00E-43	145
CD630_35640	2595399310	38.76	178	97	2	42	219	32	197	1.00E-42	144
CD630_35640	RBATCC27255_01249	39.08	174	90	3	42	213	37	196	3.00E-40	137
CD630_35670	RBATCC27255_01595	19.04	478	354	15	20	483	22	480	3.00E-07	51.2
CD630_35670	L2-63_01858	19.46	478	352	15	20	483	22	480	3.00E-09	57.4
CD630_35670	RBL236_01669	19.46	478	352	15	20	483	22	480	3.00E-09	57.4
CD630_35670	RB5AMG_00650	19.79	470	344	15	28	483	30	480	9.00E-10	58.9
CD630_35670	2595400301	30.43	46	32	0	463	508	3	48	0.078	31.6
CD630_35670	2595400301	39.02	41	25	0	466	506	58	98	0.002	36.2
CD630_35690	2595399797	26.19	42	31	0	191	232	267	308	9.6	25.8
CD630_35690	RBATCC27255_00247	31.25	64	40	2	51	111	62	124	7.3	26.6
CD630_35690	RB5AMG_01957	31.25	64	40	2	51	111	62	124	7.7	26.2
CD630_35690	L2-63_00974	56.25	16	7	0	147	162	7	22	8.3	23.9
CD630_36730	L2-63_00594	41.38	261	149	3	3	261	17	275	8.00E-64	202
CD630_36730	RBL236_00269	41.38	261	149	3	3	261	17	275	8.00E-64	202
CD630_36730	RB5AMG_01750	41.38	261	149	3	3	261	17	275	9.00E-64	202
CD630_36730	RBATCC27255_00142	41.98	262	146	3	3	261	17	275	2.00E-65	207
CD630_36730	2595398423	46.12	258	133	3	6	260	13	267	4.00E-70	219
sp|O31674|YKVI_BACSU	2595400104	36.36	33	17	1	309	341	127	155	3	27.3
sp|O31674|YKVI_BACSU	L2-63_01499	40.3	67	37	3	17	80	121	187	0.016	35
sp|O31674|YKVI_BACSU	RBL236_01470	40.3	67	37	3	17	80	121	187	0.016	35
sp|O31674|YKVI_BACSU	RB5AMG_00788	40.3	67	37	3	17	80	121	187	0.016	34.7
sp|O31674|YKVI_BACSU	RBATCC27255_00313	40.3	67	37	3	17	80	121	187	0.02	34.7
sp|O34765|YLBJ_BACSU	RBATCC27255_01144	21.35	356	237	6	2	356	4	317	2.00E-15	75.1
sp|O34765|YLBJ_BACSU	2595398760	21.59	389	246	8	16	400	20	353	5.00E-17	80.1
sp|O34765|YLBJ_BACSU	L2-63_01941	21.9	347	226	9	11	356	17	319	1.00E-15	75.9
sp|O34765|YLBJ_BACSU	RBL236_00670	21.9	347	226	9	11	356	17	319	1.00E-15	75.9
sp|O34765|YLBJ_BACSU	RB5AMG_02147	21.9	347	226	7	11	356	17	319	4.00E-15	74.3
sp|P26937|SP4FB_BACSU	L2-63_01645	22.5	120	88	2	40	158	3	118	0.003	35.8
sp|P26937|SP4FB_BACSU	RBL236_01323	22.5	120	88	2	40	158	3	118	0.003	35.8
sp|P26937|SP4FB_BACSU	RB5AMG_00140	22.5	120	88	2	40	158	3	118	0.003	35.8
sp|P26937|SP4FB_BACSU	L2-63_01069	25.53	94	56	3	70	149	86	179	0.026	33.5
sp|P26937|SP4FB_BACSU	2595400221	28.87	142	98	2	17	158	16	154	3.00E-10	57
sp|P37478|WALR_BACSU	L2-63_01735	41.13	231	132	2	1	227	1	231	2.00E-56	181
sp|P37478|WALR_BACSU	RBL236_00915	41.13	231	132	2	1	227	1	231	3.00E-56	181
sp|P37478|WALR_BACSU	L2-63_00649	43.3	224	123	2	4	227	3	222	6.00E-56	179
sp|P37478|WALR_BACSU	RBL236_00323	43.3	224	123	2	4	227	3	222	6.00E-56	179
sp|P37478|WALR_BACSU	RBATCC27255_00119	43.3	224	123	2	4	227	3	222	6.00E-56	179
sp|P50850|YLXY_BACSU	2595399157	38.86	193	117	1	121	312	46	238	7.00E-46	157
sp|P50850|YLXY_BACSU	L2-63_00955	38.86	193	117	1	121	312	46	238	1.00E-45	156
sp|P50850|YLXY_BACSU	RBL236_00647	38.86	193	117	1	121	312	46	238	1.00E-45	156
sp|P50850|YLXY_BACSU	RBATCC27255_00979	38.86	193	117	1	121	312	46	238	1.00E-45	156
sp|P50850|YLXY_BACSU	RB5AMG_01304	38.86	193	117	1	121	312	46	238	1.00E-45	156
sp|Q45581|YBBH_BACSU	2595399270	19.53	215	169	2	1	211	1	215	8.00E-04	38.5
sp|Q45581|YBBH_BACSU	RB5AMG_00951	26.47	102	73	1	179	280	339	438	0.003	37
sp|Q45581|YBBH_BACSU	L2-63_00475	26.47	102	73	1	179	280	339	438	0.003	37
sp|Q45581|YBBH_BACSU	RB5AMG_01148	33.75	80	52	1	176	254	89	168	7.00E-04	38.5
sp|Q45581|YBBH_BACSU	2595398802	34.25	73	46	1	179	251	339	409	3.00E-04	40.4
