# Supplementary material for: Sporulation capability and amylosome conservation among diverse human colonic and rumen isolates of the keystone starch‐degrader Ruminococcus bromii
Source: Environ Microbiol. 2017 Dec 7;20(1):324–36. doi: 10.1111/1462-2920.14000 (PMC5814915; doi:10.1111/1462-2920.14000)
Supplement: Supplementary file 10 — Data File S1. List of core, accessory and unique genes from pan‐genome analysis of 5 R. bromii strains. [file EMI-20-324-s010.pdf]

Genome_no.	ID	*R.bromii* strain
1	Organism1	Rbromii 2593339225_YE282
2	Organism2	Rbromii 5AMG
3	Organism3	Rbromii ATCC27255
4	Organism4	Rbromii L2-36
5	Organism5	Rbromii L2-63

BPGA ID	type	cluster	seq ID	strain
Org1_Gene1083	accessory	2712	2595399040_IE32DRAFT_01127_Kynurenine_formamidase_(EC_3.5.1.9)_[Ruminococcus_bromii_YE282]	Ruminococcus_bromii_YE282
Org1_Gene1255	accessory	1689	2595399213_IE32DRAFT_01301_Glycosyl_transferase_family_2_[Ruminococcus_bromii_YE282]	Ruminococcus_bromii_YE282
Org1_Gene1256	accessory	547	2595399214_IE32DRAFT_01302_Membrane_protein_involved_in_the_export_of_O-antigen_and_teichoic_acid_[Ruminococcus_bromii_YE282]	Ruminococcus_bromii_YE282
Org1_Gene1257	accessory	1203	2595399215_IE32DRAFT_01303_Polysaccharide_pyruvyl_transferase_[Ruminococcus_bromii_YE282]	Ruminococcus_bromii_YE282
Org1_Gene1259	accessory	1491	2595399217_IE32DRAFT_01305_Glycosyl_transferase_family_2_[Ruminococcus_bromii_YE282]	Ruminococcus_bromii_YE282
Org1_Gene1261	accessory	1097	2595399219_IE32DRAFT_01307_Polysaccharide_pyruvyl_transferase_[Ruminococcus_bromii_YE282]	Ruminococcus_bromii_YE282
Org1_Gene1262	accessory	3119	2595399220_IE32DRAFT_01308_Bacterial_transferase_hexapeptide_(six_repeats)_[Ruminococcus_bromii_YE282]	Ruminococcus_bromii_YE282
Org1_Gene1263	accessory	934	2595399221_IE32DRAFT_01309_Glycosyltransferase_[Ruminococcus_bromii_YE282]	Ruminococcus_bromii_YE282
Org1_Gene1264	accessory	1411	2595399222_IE32DRAFT_01310_Lipopolysaccharide_biosynthesis_proteins,_LPS:glycosyltransferases_[Ruminococcus_bromii_YE282]	Ruminococcus_bromii_YE282
Org1_Gene1351	accessory	903	2595399313_IE32DRAFT_01401_Transposase_[Ruminococcus_bromii_YE282]	Ruminococcus_bromii_YE282
Org1_Gene1357	accessory	3544	2595399319_IE32DRAFT_01407_Transposon-encoded_protein_TnpV_[Ruminococcus_bromii_YE282]	Ruminococcus_bromii_YE282
Org1_Gene1361	accessory	1261	2595399323_IE32DRAFT_01411_AP2-like_DNA-binding_integrase_domain/Phage_integrase_family/Phage_integrase,_N-terminal_SAM-like_domain_[Rum	Ruminococcus_bromii_YE282
Org1_Gene1486	accessory	3553	2595399450_IE32DRAFT_01538_Transposon-encoded_protein_TnpV_[Ruminococcus_bromii_YE282]	Ruminococcus_bromii_YE282
Org1_Gene1594	accessory	813	2595399558_IE32DRAFT_01646_Adenosylmethionine-8-amino-7-oxononanoate_aminotransferase_[Ruminococcus_bromii_YE282]	Ruminococcus_bromii_YE282
Org1_Gene1595	accessory	1135	2595399559_IE32DRAFT_01647_Alcohol_dehydrogenase,_class_IV_[Ruminococcus_bromii_YE282]	Ruminococcus_bromii_YE282
Org1_Gene1596	accessory	2032	2595399560_IE32DRAFT_01648_ABC-type_nitrate/sulfonate/bicarbonate_transport_system,_ATPase_component_[Ruminococcus_bromii_YE282]	Ruminococcus_bromii_YE282
Org1_Gene1597	accessory	1900	2595399561_IE32DRAFT_01649_ABC-type_nitrate/sulfonate/bicarbonate_transport_system,_permease_component_[Ruminococcus_bromii_YE282]	Ruminococcus_bromii_YE282
Org1_Gene1598	accessory	1917	2595399562_IE32DRAFT_01650_ABC-type_nitrate/sulfonate/bicarbonate_transport_system,_permease_component_[Ruminococcus_bromii_YE282]	Ruminococcus_bromii_YE282
Org1_Gene1599	accessory	1171	2595399563_IE32DRAFT_01651_NMT1/THI5_like_[Ruminococcus_bromii_YE282]	Ruminococcus_bromii_YE282
Org1_Gene1600	accessory	736	2595399564_IE32DRAFT_01652_D-hydantoinase_[Ruminococcus_bromii_YE282]	Ruminococcus_bromii_YE282
Org1_Gene1601	accessory	579	2595399565_IE32DRAFT_01653_dihydroorotate_dehydrogenase_(subfamily_1)_family_protein_[Ruminococcus_bromii_YE282]	Ruminococcus_bromii_YE282
Org1_Gene1602	accessory	923	2595399566_IE32DRAFT_01654_amidase,_hydantoinase/carbamoylase_family_[Ruminococcus_bromii_YE282]	Ruminococcus_bromii_YE282
Org1_Gene1603	accessory	2616	2595399567_IE32DRAFT_01655_Cytidylate_kinase-like_family_[Ruminococcus_bromii_YE282]	Ruminococcus_bromii_YE282
Org1_Gene1639	accessory	4178	2595399603_IE32DRAFT_01691_Helix-turn-helix_domain_[Ruminococcus_bromii_YE282]	Ruminococcus_bromii_YE282
Org1_Gene1716	accessory	3760	2595399680_IE32DRAFT_01768_TfoX_N-terminal_domain_[Ruminococcus_bromii_YE282]	Ruminococcus_bromii_YE282
Org1_Gene1718	accessory	2160	2595399683_IE32DRAFT_01771_Putative_zinc_ribbon_domain/Helix-turn-helix_[Ruminococcus_bromii_YE282]	Ruminococcus_bromii_YE282
Org1_Gene1721	accessory	2950	2595399686_IE32DRAFT_01774_Fructose-2,6-bisphosphatase_[Ruminococcus_bromii_YE282]	Ruminococcus_bromii_YE282
Org1_Gene1723	accessory	275	2595399688_IE32DRAFT_01776_ATP-dependent_DNA_helicase_RecQ_[Ruminococcus_bromii_YE282]	Ruminococcus_bromii_YE282
Org1_Gene1854	accessory	4414	2595399828_IE32DRAFT_01917_hypothetical_protein_[Ruminococcus_bromii_YE282]	Ruminococcus_bromii_YE282
Org1_Gene1860	accessory	3868	2595399834_IE32DRAFT_01923_looped-hinge_helix_DNA_binding_domain,_AbrB_family_[Ruminococcus_bromii_YE282]	Ruminococcus_bromii_YE282
Org1_Gene1866	accessory	3654	2595399840_IE32DRAFT_01929_Protein_of_unknown_function_(DUF3852)_[Ruminococcus_bromii_YE282]	Ruminococcus_bromii_YE282
Org1_Gene1876	accessory	1185	2595399850_IE32DRAFT_01939_hypothetical_protein_[Ruminococcus_bromii_YE282]	Ruminococcus_bromii_YE282
Org1_Gene1880	accessory	2952	2595399854_IE32DRAFT_01943_Uncharacterized_conserved_protein_[Ruminococcus_bromii_YE282]	Ruminococcus_bromii_YE282
Org1_Gene1885	accessory	299	2595399859_IE32DRAFT_01948_Type_IV_secretory_pathway,_VirD4_components_[Ruminococcus_bromii_YE282]	Ruminococcus_bromii_YE282
Org1_Gene1886	accessory	3956	2595399860_IE32DRAFT_01949_Helix-turn-helix_domain_[Ruminococcus_bromii_YE282]	Ruminococcus_bromii_YE282
Org1_Gene1889	accessory	4366	2595399868_IE32DRAFT_01957_hypothetical_protein_[Ruminococcus_bromii_YE282]	Ruminococcus_bromii_YE282
Org1_Gene1890	accessory	3776	2595399869_IE32DRAFT_01958_hypothetical_protein_[Ruminococcus_bromii_YE282]	Ruminococcus_bromii_YE282
Org1_Gene1891	accessory	2120	2595399870_IE32DRAFT_01959_DNA-methyltransferase_(dcm)_[Ruminococcus_bromii_YE282]	Ruminococcus_bromii_YE282
Org1_Gene1892	accessory	2057	2595399871_IE32DRAFT_01960_C-5_cytosine-specific_DNA_methylase_[Ruminococcus_bromii_YE282]	Ruminococcus_bromii_YE282
Org1_Gene1893	accessory	1451	2595399872_IE32DRAFT_01961_Site-specific_DNA_methylase_[Ruminococcus_bromii_YE282]	Ruminococcus_bromii_YE282
Org1_Gene1894	accessory	4167	2595399873_IE32DRAFT_01962_hypothetical_protein_[Ruminococcus_bromii_YE282]	Ruminococcus_bromii_YE282
Org1_Gene1896	accessory	3470	2595399875_IE32DRAFT_01964_Uncharacterized_protein,_involved_in_the_regulation_of_septum_location_[Ruminococcus_bromii_YE282]	Ruminococcus_bromii_YE282
Org1_Gene1899	accessory	2696	2595399878_IE32DRAFT_01967_hypothetical_protein_[Ruminococcus_bromii_YE282]	Ruminococcus_bromii_YE282
Org1_Gene1900	accessory	22	2595399879_IE32DRAFT_01968_Cna_protein_B-type_domain/Carboxypeptidase_regulatory-like_domain_[Ruminococcus_bromii_YE282]	Ruminococcus_bromii_YE282
Org1_Gene1901	accessory	3636	2595399880_IE32DRAFT_01969_hypothetical_protein_[Ruminococcus_bromii_YE282]	Ruminococcus_bromii_YE282
Org1_Gene1903	accessory	1228	2595399882_IE32DRAFT_01971_hypothetical_protein_[Ruminococcus_bromii_YE282]	Ruminococcus_bromii_YE282
Org1_Gene1905	accessory	2691	2595399884_IE32DRAFT_01973_hypothetical_protein_[Ruminococcus_bromii_YE282]	Ruminococcus_bromii_YE282
Org1_Gene1906	accessory	1966	2595399885_IE32DRAFT_01974_sortase,_SrtB_family_[Ruminococcus_bromii_YE282]	Ruminococcus_bromii_YE282
Org1_Gene1910	accessory	4059	2595399889_IE32DRAFT_01978_hypothetical_protein_[Ruminococcus_bromii_YE282]	Ruminococcus_bromii_YE282
Org1_Gene1911	accessory	252	2595399890_IE32DRAFT_01979_Putative_amidoligase_enzyme_[Ruminococcus_bromii_YE282]	Ruminococcus_bromii_YE282
Org1_Gene1913	accessory	3467	2595399892_IE32DRAFT_01981_hypothetical_protein_[Ruminococcus_bromii_YE282]	Ruminococcus_bromii_YE282
Org1_Gene2000	accessory	3172	2595399979_IE32DRAFT_02068_Sigma-70,_region_4_[Ruminococcus_bromii_YE282]	Ruminococcus_bromii_YE282
Org1_Gene2002	accessory	4306	2595399981_IE32DRAFT_02070_hypothetical_protein_[Ruminococcus_bromii_YE282]	Ruminococcus_bromii_YE282
Org1_Gene2003	accessory	524	2595399982_IE32DRAFT_02071_Site-specific_recombinases,_DNA_invertase_Pin_homologs_[Ruminococcus_bromii_YE282]	Ruminococcus_bromii_YE282
Org1_Gene2042	accessory	3512	2595400022_IE32DRAFT_02111_Cysteine-rich_VLP_[Ruminococcus_bromii_YE282]	Ruminococcus_bromii_YE282
Org1_Gene2051	accessory	850	2595400031_IE32DRAFT_02120_GHKL_domain_[Ruminococcus_bromii_YE282]	Ruminococcus_bromii_YE282
Org1_Gene2052	accessory	2349	2595400032_IE32DRAFT_02121_two_component_transcriptional_regulator,_LytTR_family_[Ruminococcus_bromii_YE282]	Ruminococcus_bromii_YE282
Org1_Gene2053	accessory	4379	2595400033_IE32DRAFT_02122_cyclic_lactone_autoinducer_peptide_[Ruminococcus_bromii_YE282]	Ruminococcus_bromii_YE282
Org1_Gene2054	accessory	2721	2595400034_IE32DRAFT_02123_Membrane_protein_putatively_involved_in_post-translational_modification_of_the_autoinducing_quorum-sensing_pept	Ruminococcus_bromii_YE282
Org1_Gene22	accessory	2650	2595397945_IE32DRAFT_00026_DnaJ_domain_[Ruminococcus_bromii_YE282]	Ruminococcus_bromii_YE282
Org1_Gene2209	accessory	4328	2595400195_IE32DRAFT_02285_DNA_binding_domain,_excisionase_family_[Ruminococcus_bromii_YE282]	Ruminococcus_bromii_YE282
Org1_Gene2219	accessory	4311	2595400205_IE32DRAFT_02295_hypothetical_protein_[Ruminococcus_bromii_YE282]	Ruminococcus_bromii_YE282
Org1_Gene2253	accessory	980	2595400241_IE32DRAFT_02331_Transposase_and_inactivated_derivatives_[Ruminococcus_bromii_YE282]	Ruminococcus_bromii_YE282
Org1_Gene2285	accessory	1672	2595400274_IE32DRAFT_02364_Nucleoside-diphosphate-sugar_epimerases_[Ruminococcus_bromii_YE282]	Ruminococcus_bromii_YE282
Org1_Gene2295	accessory	4213	2595400284_IE32DRAFT_02374_hypothetical_protein_[Ruminococcus_bromii_YE282]	Ruminococcus_bromii_YE282
Org1_Gene2296	accessory	1244	2595400285_IE32DRAFT_02375_Site-specific_recombinase_XerD_[Ruminococcus_bromii_YE282]	Ruminococcus_bromii_YE282
Org1_Gene2297	accessory	3695	2595400286_IE32DRAFT_02376_hypothetical_protein_[Ruminococcus_bromii_YE282]	Ruminococcus_bromii_YE282
Org1_Gene2324	accessory	3189	2595400314_IE32DRAFT_02404_Predicted_transcriptional_regulators_[Ruminococcus_bromii_YE282]	Ruminococcus_bromii_YE282
Org1_Gene2325	accessory	4232	2595400315_IE32DRAFT_02405_hypothetical_protein_[Ruminococcus_bromii_YE282]	Ruminococcus_bromii_YE282
Org1_Gene2326	accessory	1264	2595400316_IE32DRAFT_02406_Site-specific_recombinase_XerD_[Ruminococcus_bromii_YE282]	Ruminococcus_bromii_YE282
Org1_Gene2327	accessory	3692	2595400317_IE32DRAFT_02407_hypothetical_protein_[Ruminococcus_bromii_YE282]	Ruminococcus_bromii_YE282
Org1_Gene2328	accessory	1136	2595400318_IE32DRAFT_02408_AAA_domain_[Ruminococcus_bromii_YE282]	Ruminococcus_bromii_YE282
Org1_Gene2331	accessory	1283	2595400321_IE32DRAFT_02411_DNA_adenine_methylase_(dam)_[Ruminococcus_bromii_YE282]	Ruminococcus_bromii_YE282
Org1_Gene2350	accessory	993	2595400341_IE32DRAFT_02431_Transposase_[Ruminococcus_bromii_YE282]	Ruminococcus_bromii_YE282
Org1_Gene2391	accessory	1640	2595400383_IE32DRAFT_02473_Protein_of_unknown_function_(DUF3991)/Toprim-like_[Ruminococcus_bromii_YE282]	Ruminococcus_bromii_YE282
Org1_Gene2465	accessory	4229	2595400464_IE32DRAFT_02555_Small,_acid-soluble_spore_proteins,_alpha/beta_type_[Ruminococcus_bromii_YE282]	Ruminococcus_bromii_YE282
Org1_Gene2484	accessory	2065	2595400483_IE32DRAFT_02574_hypothetical_protein_[Ruminococcus_bromii_YE282]	Ruminococcus_bromii_YE282
Org1_Gene2490	accessory	2825	2595400489_IE32DRAFT_02580_Acetyltransferases,_including_N-acetylases_of_ribosomal_proteins_[Ruminococcus_bromii_YE282]	Ruminococcus_bromii_YE282
Org1_Gene281	accessory	2439	2595398225_IE32DRAFT_00306_ABC-type_antimicrobial_peptide_transport_system,_ATPase_component_[Ruminococcus_bromii_YE282]	Ruminococcus_bromii_YE282
Org1_Gene340	accessory	2729	2595398285_IE32DRAFT_00366_translation_initiation_factor_IF-3_[Ruminococcus_bromii_YE282]	Ruminococcus_bromii_YE282
Org1_Gene417	accessory	858	2595398362_IE32DRAFT_00443_phosphopyruvate_hydratase_[Ruminococcus_bromii_YE282]	Ruminococcus_bromii_YE282
Org1_Gene462	accessory	3013	2595398409_IE32DRAFT_00490_Acetyltransferases,_including_N-acetylases_of_ribosomal_proteins_[Ruminococcus_bromii_YE282]	Ruminococcus_bromii_YE282
Org1_Gene52	accessory	4291	2595397975_IE32DRAFT_00056_hypothetical_protein_[Ruminococcus_bromii_YE282]	Ruminococcus_bromii_YE282
Org1_Gene591	accessory	3598	2595398542_IE32DRAFT_00623_Protein_of_unknown_function_(DUF3852)_[Ruminococcus_bromii_YE282]	Ruminococcus_bromii_YE282
Org1_Gene597	accessory	350	2595398548_IE32DRAFT_00629_AAA-like_domain_[Ruminococcus_bromii_YE282]	Ruminococcus_bromii_YE282
Org1_Gene756	accessory	692	2595398709_IE32DRAFT_00791_Relaxase/Mobilisation_nuclease_domain_[Ruminococcus_bromii_YE282]	Ruminococcus_bromii_YE282
Org1_Gene757	accessory	3610	2595398710_IE32DRAFT_00792_Ribbon-helix-helix_protein,_copG_family_[Ruminococcus_bromii_YE282]	Ruminococcus_bromii_YE282
Org1_Gene758	accessory	3706	2595398711_IE32DRAFT_00793_hypothetical_protein_[Ruminococcus_bromii_YE282]	Ruminococcus_bromii_YE282
Org1_Gene759	accessory	1613	2595398712_IE32DRAFT_00794_hypothetical_protein_[Ruminococcus_bromii_YE282]	Ruminococcus_bromii_YE282
Org1_Gene760	accessory	4069	2595398713_IE32DRAFT_00795_Cysteine-rich_VLP_[Ruminococcus_bromii_YE282]	Ruminococcus_bromii_YE282
Org1_Gene761	accessory	1168	2595398714_IE32DRAFT_00796_hypothetical_protein_[Ruminococcus_bromii_YE282]	Ruminococcus_bromii_YE282
Org1_Gene762	accessory	1739	2595398715_IE32DRAFT_00797_DNA-directed_RNA_polymerase_specialized_sigma_subunit_[Ruminococcus_bromii_YE282]	Ruminococcus_bromii_YE282
Org1_Gene844	accessory	2669	2595398797_IE32DRAFT_00879_dTDP-4-dehydrorhamnose_3,5-epimerase_[Ruminococcus_bromii_YE282]	Ruminococcus_bromii_YE282
Org1_Gene945	accessory	976	2595398898_IE32DRAFT_00982_Site-specific_recombinase_XerD_[Ruminococcus_bromii_YE282]	Ruminococcus_bromii_YE282
Org2_Gene1	accessory	2528	[Rbromii5AMG]RB5AMG_00001_5'-nucleotidase	Rbromii5AMG
Org2_Gene100	accessory	993	[Rbromii5AMG]RB5AMG_00106_Transposase	Rbromii5AMG
Org2_Gene1000	accessory	1481	[Rbromii5AMG]RB5AMG_01031_1,5-anhydro-D-fructose_reductase	Rbromii5AMG
Org2_Gene1002	accessory	1342	[Rbromii5AMG]RB5AMG_01033_Sulfate_starvation-induced_protein_2	Rbromii5AMG
Org2_Gene1003	accessory	1867	[Rbromii5AMG]RB5AMG_01034_Sulfate_transport_system_permease_protein_CysT	Rbromii5AMG
Org2_Gene1004	accessory	1971	[Rbromii5AMG]RB5AMG_01035_Sulfate_transport_system_permease_protein_CysW	Rbromii5AMG
Org2_Gene1005	accessory	1303	[Rbromii5AMG]RB5AMG_01036_Sulfate/thiosulfate_import_ATP-binding_protein_CysA	Rbromii5AMG
Org2_Gene1007	accessory	1165	[Rbromii5AMG]RB5AMG_01038_Glutamyl-tRNA_reductase	Rbromii5AMG
Org2_Gene1008	accessory	2659	[Rbromii5AMG]RB5AMG_01039_Siroheme_synthase	Rbromii5AMG
Org2_Gene1009	accessory	1836	[Rbromii5AMG]RB5AMG_01040_Porphobilinogen_deaminase	Rbromii5AMG
Org2_Gene101	accessory	4306	[Rbromii5AMG]RB5AMG_00107_hypothetical_protein	Rbromii5AMG
Org2_Gene1010	accessory	591	[Rbromii5AMG]RB5AMG_01041_Uroporphyrinogen-III_C-methyltransferase	Rbromii5AMG
Org2_Gene1011	accessory	1492	[Rbromii5AMG]RB5AMG_01042_Delta-aminolevulinic_acid_dehydratase	Rbromii5AMG
Org2_Gene1012	accessory	920	[Rbromii5AMG]RB5AMG_01043_Glutamate-1-semialdehyde_2,1-aminomutase_2	Rbromii5AMG
Org2_Gene1013	accessory	3248	[Rbromii5AMG]RB5AMG_01044_HTH-type_transcriptional_regulator_IscR	Rbromii5AMG
Org2_Gene1014	accessory	940	[Rbromii5AMG]RB5AMG_01045_Methionine_gamma-lyase	Rbromii5AMG
Org2_Gene1015	accessory	1835	[Rbromii5AMG]RB5AMG_01046_Anaerobic_sulfite_reductase_subunit_C	Rbromii5AMG
Org2_Gene1016	accessory	4035	[Rbromii5AMG]RB5AMG_01047_SirA-like_protein	Rbromii5AMG
Org2_Gene1017	accessory	3759	[Rbromii5AMG]RB5AMG_01048_Thioredoxin-M	Rbromii5AMG

Org2_Gene1018	accessory	4189	[Rbromii5AMG]RB5AMG_01049_bifunctional_sulfur_carrier_protein/thiazole_synthase_protein	Rbromii5AMG
Org2_Gene1019	accessory	2001	[Rbromii5AMG]RB5AMG_01050_putative_adenylyltransferase/sulfurtransferase_MoeZ	Rbromii5AMG
Org2_Gene1020	accessory	3425	[Rbromii5AMG]RB5AMG_01051_CysO-cysteine_peptidase	Rbromii5AMG
Org2_Gene1021	accessory	1657	[Rbromii5AMG]RB5AMG_01052_Thioredoxin_reductase	Rbromii5AMG
Org2_Gene1022	accessory	442	[Rbromii5AMG]RB5AMG_01053_Fumarate_reductase_flavoprotein_subunit_precursor	Rbromii5AMG
Org2_Gene1023	accessory	3783	[Rbromii5AMG]RB5AMG_01054_Ferredoxin_II	Rbromii5AMG
Org2_Gene1024	accessory	1700	[Rbromii5AMG]RB5AMG_01055_Sulfate_adenylyltransferase_subunit_2	Rbromii5AMG
Org2_Gene1025	accessory	439	[Rbromii5AMG]RB5AMG_01056_Sulfate_adenylyltransferase_subunit_1	Rbromii5AMG
Org2_Gene1026	accessory	4217	[Rbromii5AMG]RB5AMG_01057_TM2_domain-containing_protein	Rbromii5AMG
Org2_Gene1027	accessory	3515	[Rbromii5AMG]RB5AMG_01058_hypothetical_protein	Rbromii5AMG
Org2_Gene1028	accessory	2955	[Rbromii5AMG]RB5AMG_01059_Methylated-DNA--protein-cysteine_methyltransferase,_inducible	Rbromii5AMG
Org2_Gene1029	accessory	893	[Rbromii5AMG]RB5AMG_01060_hypothetical_protein	Rbromii5AMG
Org2_Gene103	accessory	2957	[Rbromii5AMG]RB5AMG_00111_hypothetical_protein	Rbromii5AMG
Org2_Gene1030	accessory	2352	[Rbromii5AMG]RB5AMG_01061_Sensory_transduction_protein_LytR	Rbromii5AMG
Org2_Gene1031	accessory	6	[Rbromii5AMG]RB5AMG_01062_Tyrocidine_synthase_III	Rbromii5AMG
Org2_Gene1032	accessory	389	[Rbromii5AMG]RB5AMG_01063_Multidrug_export_protein_MepA	Rbromii5AMG
Org2_Gene1033	accessory	3806	[Rbromii5AMG]RB5AMG_01064_Stage_II_sporulation_protein_AA	Rbromii5AMG
Org2_Gene1034	accessory	3468	[Rbromii5AMG]RB5AMG_01065_serine-protein_kinase_RsbW	Rbromii5AMG
Org2_Gene1035	accessory	2215	[Rbromii5AMG]RB5AMG_01066_Inner_membrane_protein_YgaZ	Rbromii5AMG
Org2_Gene1036	accessory	3815	[Rbromii5AMG]RB5AMG_01067_putative_membrane_protein	Rbromii5AMG
Org2_Gene1037	accessory	1104	[Rbromii5AMG]RB5AMG_01068_Nuclease_SbcCD_subunit_D	Rbromii5AMG
Org2_Gene1038	accessory	73	[Rbromii5AMG]RB5AMG_01069_Nuclease_SbcCD_subunit_C	Rbromii5AMG
Org2_Gene1039	accessory	2100	[Rbromii5AMG]RB5AMG_01070_Zinc_transporter_ZupT	Rbromii5AMG
Org2_Gene104	accessory	2437	[Rbromii5AMG]RB5AMG_00112_hypothetical_protein	Rbromii5AMG
Org2_Gene1040	accessory	3997	[Rbromii5AMG]RB5AMG_01071_hypothetical_protein	Rbromii5AMG
Org2_Gene1042	accessory	2479	[Rbromii5AMG]RB5AMG_01073_Immunity_protein_SdpI	Rbromii5AMG
Org2_Gene1043	accessory	4003	[Rbromii5AMG]RB5AMG_01074_Transcriptional_repressor_SdpR	Rbromii5AMG
Org2_Gene1044	accessory	858	[Rbromii5AMG]RB5AMG_01075_Enolase	Rbromii5AMG
Org2_Gene1045	accessory	742	[Rbromii5AMG]RB5AMG_01076_Staphylococcal_virulence_regulator_protein_A	Rbromii5AMG
Org2_Gene1046	accessory	2826	[Rbromii5AMG]RB5AMG_01077_hypothetical_protein	Rbromii5AMG
Org2_Gene1047	accessory	1220	[Rbromii5AMG]RB5AMG_01078_Acyltransferase_family_protein	Rbromii5AMG
Org2_Gene1048	accessory	108	[Rbromii5AMG]RB5AMG_01079_CDP-Glycerol:Poly(glycerophosphate)_glycerophosphotransferase	Rbromii5AMG
Org2_Gene105	accessory	861	[Rbromii5AMG]RB5AMG_00113_hypothetical_protein	Rbromii5AMG
Org2_Gene1050	accessory	3156	[Rbromii5AMG]RB5AMG_01081_putative_acetyltransferase	Rbromii5AMG
Org2_Gene1051	accessory	125	[Rbromii5AMG]RB5AMG_01082_Elongation_factor_G	Rbromii5AMG
Org2_Gene1052	accessory	3693	[Rbromii5AMG]RB5AMG_01083_anaerobic_benzoate_catabolism_transcriptional_regulator	Rbromii5AMG
Org2_Gene1053	accessory	2779	[Rbromii5AMG]RB5AMG_01084_Zn_finger_protein_HypA/HybF_(possibly_regulating_hydrogenase_expression)	Rbromii5AMG
Org2_Gene1055	accessory	4006	[Rbromii5AMG]RB5AMG_01086_hypothetical_protein	Rbromii5AMG
Org2_Gene1056	accessory	2931	[Rbromii5AMG]RB5AMG_01087_hypothetical_protein	Rbromii5AMG
Org2_Gene1057	accessory	2861	[Rbromii5AMG]RB5AMG_01088_hypothetical_protein	Rbromii5AMG
Org2_Gene1059	accessory	3419	[Rbromii5AMG]RB5AMG_01090_Peptide_deformylase_2	Rbromii5AMG
Org2_Gene1060	accessory	2311	[Rbromii5AMG]RB5AMG_01091_hypothetical_protein	Rbromii5AMG
Org2_Gene1061	accessory	3131	[Rbromii5AMG]RB5AMG_01092_RNA_polymerase_sigma_factor_SigM	Rbromii5AMG
Org2_Gene1062	accessory	4263	[Rbromii5AMG]RB5AMG_01093_hypothetical_protein	Rbromii5AMG
Org2_Gene1063	accessory	3263	[Rbromii5AMG]RB5AMG_01094_HTH-type_transcriptional_regulator_AdhR	Rbromii5AMG
Org2_Gene1064	accessory	2874	[Rbromii5AMG]RB5AMG_01095_hypothetical_protein	Rbromii5AMG
Org2_Gene1065	accessory	1905	[Rbromii5AMG]RB5AMG_01096_Patatin-like_phospholipase	Rbromii5AMG
Org2_Gene1066	accessory	2662	[Rbromii5AMG]RB5AMG_01097_mycofactocin_system_transcriptional_regulator	Rbromii5AMG
Org2_Gene1067	accessory	2363	[Rbromii5AMG]RB5AMG_01098_azoreductase	Rbromii5AMG
Org2_Gene1070	accessory	213	[Rbromii5AMG]RB5AMG_01101_3-oxoacyl-[acyl-carrier-protein]_synthase_2	Rbromii5AMG
Org2_Gene1072	accessory	2422	[Rbromii5AMG]RB5AMG_01103_Bifunctional_protein_aas	Rbromii5AMG
Org2_Gene1073	accessory	1299	[Rbromii5AMG]RB5AMG_01104_Tryptophan--tRNA_ligase_2	Rbromii5AMG
Org2_Gene1074	accessory	1381	[Rbromii5AMG]RB5AMG_01105_Penicillin_V_acylase	Rbromii5AMG
Org2_Gene1075	accessory	840	[Rbromii5AMG]RB5AMG_01106_sensory_histidine_kinase_DcuS	Rbromii5AMG
Org2_Gene1076	accessory	2284	[Rbromii5AMG]RB5AMG_01107_Transcriptional_regulatory_protein_YpdB	Rbromii5AMG
Org2_Gene1077	accessory	272	[Rbromii5AMG]RB5AMG_01108_Metallo-beta-lactamase_superfamily_protein	Rbromii5AMG
Org2_Gene1078	accessory	2887	[Rbromii5AMG]RB5AMG_01109_hypothetical_protein	Rbromii5AMG
Org2_Gene1083	accessory	1493	[Rbromii5AMG]RB5AMG_01117_V-type_sodium_pump_subunit_C	Rbromii5AMG
Org2_Gene1084	accessory	2760	[Rbromii5AMG]RB5AMG_01118_V-type_ATP_synthase_subunit_E	Rbromii5AMG
Org2_Gene1086	accessory	287	[Rbromii5AMG]RB5AMG_01120_V-type_ATP_synthase_subunit_I	Rbromii5AMG
Org2_Gene1087	accessory	3801	[Rbromii5AMG]RB5AMG_01121_V-type_ATP_synthase_subunit_H	Rbromii5AMG
Org2_Gene1088	accessory	1429	[Rbromii5AMG]RB5AMG_01122_mobile_mystery_protein_B	Rbromii5AMG
Org2_Gene109	accessory	799	[Rbromii5AMG]RB5AMG_00117_Maltose-binding_periplasmic_proteins/domains	Rbromii5AMG
Org2_Gene1091	accessory	32	[Rbromii5AMG]RB5AMG_01125_Swarming_motility_protein_SwrC	Rbromii5AMG
Org2_Gene1092	accessory	2637	[Rbromii5AMG]RB5AMG_01126_DNA-binding_transcriptional_repressor_AcrR	Rbromii5AMG
Org2_Gene1093	accessory	3183	[Rbromii5AMG]RB5AMG_01127_Low_molecular_weight_protein-tyrosine-phosphatase_YfkJ	Rbromii5AMG
Org2_Gene1094	accessory	4432	[Rbromii5AMG]RB5AMG_01128_hypothetical_protein	Rbromii5AMG
Org2_Gene1095	accessory	2604	[Rbromii5AMG]RB5AMG_01129_putative_sugar_transferase_EpsL	Rbromii5AMG
Org2_Gene1096	accessory	1241	[Rbromii5AMG]RB5AMG_01130_N,N'-diacetylbacillosaminyl-diphospho-undecaprenol_alpha-1,3-N-acetylgalactosaminyltransferase	Rbromii5AMG
Org2_Gene1097	accessory	1138	[Rbromii5AMG]RB5AMG_01131_Transmembrane_protein_EpsG	Rbromii5AMG
Org2_Gene110	accessory	776	[Rbromii5AMG]RB5AMG_00118_Maltose-binding_periplasmic_proteins/domains	Rbromii5AMG
Org2_Gene112	accessory	1830	[Rbromii5AMG]RB5AMG_00120_L-arabinose_transport_system_permease_protein_AraP	Rbromii5AMG
Org2_Gene1120	accessory	1025	[Rbromii5AMG]RB5AMG_01154_hypothetical_protein	Rbromii5AMG
Org2_Gene1121	accessory	2002	[Rbromii5AMG]RB5AMG_01155_Sulfate_starvation-induced_protein_7	Rbromii5AMG
Org2_Gene1124	accessory	3499	[Rbromii5AMG]RB5AMG_01158_hypothetical_protein	Rbromii5AMG
Org2_Gene1125	accessory	878	[Rbromii5AMG]RB5AMG_01159_Putative_metal_chaperone_YciC	Rbromii5AMG
Org2_Gene1126	accessory	2837	[Rbromii5AMG]RB5AMG_01160_Septum_formation_protein_Maf	Rbromii5AMG
Org2_Gene1127	accessory	1608	[Rbromii5AMG]RB5AMG_01161_Serine_acetyltransferase	Rbromii5AMG
Org2_Gene113	accessory	1912	[Rbromii5AMG]RB5AMG_00121_Lactose_transport_system_permease_protein_LacG	Rbromii5AMG
Org2_Gene1130	accessory	3151	[Rbromii5AMG]RB5AMG_01164_putative_acetyltransferase	Rbromii5AMG
Org2_Gene1131	accessory	789	[Rbromii5AMG]RB5AMG_01165_Na+-dependent_transporters_of_the_SNF_family_protein	Rbromii5AMG
Org2_Gene1132	accessory	1741	[Rbromii5AMG]RB5AMG_01166_Cyn_operon_transcriptional_activator	Rbromii5AMG
Org2_Gene1134	accessory	3963	[Rbromii5AMG]RB5AMG_01168_CotJB_protein	Rbromii5AMG
Org2_Gene1135	accessory	4215	[Rbromii5AMG]RB5AMG_01169_Spore_coat_associated_protein_JA_(CotJA)	Rbromii5AMG
Org2_Gene1136	accessory	1933	[Rbromii5AMG]RB5AMG_01170_Thiamine_ECF_transporter_S_component_ThiT	Rbromii5AMG
Org2_Gene1139	accessory	1712	[Rbromii5AMG]RB5AMG_01174_hypothetical_protein	Rbromii5AMG
Org2_Gene114	accessory	3364	[Rbromii5AMG]RB5AMG_00122_hypothetical_protein	Rbromii5AMG
Org2_Gene1141	accessory	2700	[Rbromii5AMG]RB5AMG_01176_Alpha/beta_hydrolase_family_protein	Rbromii5AMG
Org2_Gene1146	accessory	4335	[Rbromii5AMG]RB5AMG_01181_Virus_attachment_protein_p12_family_protein	Rbromii5AMG
Org2_Gene1147	accessory	2386	[Rbromii5AMG]RB5AMG_01182_Bacitracin_export_ATP-binding_protein_BceA	Rbromii5AMG
Org2_Gene1148	accessory	202	[Rbromii5AMG]RB5AMG_01183_FtsX-like_permease_family_protein	Rbromii5AMG
Org2_Gene1149	accessory	3556	[Rbromii5AMG]RB5AMG_01184_copper_ion_binding_protein	Rbromii5AMG
Org2_Gene115	accessory	1144	[Rbromii5AMG]RB5AMG_00123_acidobacterial_duplicated_orphan_permease	Rbromii5AMG
Org2_Gene1150	accessory	1531	[Rbromii5AMG]RB5AMG_01185_Regulatory_protein_SoxS	Rbromii5AMG
Org2_Gene1151	accessory	2862	[Rbromii5AMG]RB5AMG_01186_hypothetical_protein	Rbromii5AMG
Org2_Gene1156	accessory	3105	[Rbromii5AMG]RB5AMG_01192_Putative_phosphinothricin_acetyltransferase_YwnH	Rbromii5AMG
Org2_Gene1157	accessory	3696	[Rbromii5AMG]RB5AMG_01193_hypothetical_protein	Rbromii5AMG
Org2_Gene1158	accessory	4130	[Rbromii5AMG]RB5AMG_01194_hypothetical_protein	Rbromii5AMG
Org2_Gene1159	accessory	3287	[Rbromii5AMG]RB5AMG_01195_hypothetical_protein	Rbromii5AMG
Org2_Gene1160	accessory	4066	[Rbromii5AMG]RB5AMG_01196_Antitoxin_YefM	Rbromii5AMG
Org2_Gene1161	accessory	3921	[Rbromii5AMG]RB5AMG_01197_addiction_module_toxin,_Txe/YoeB_family	Rbromii5AMG
Org2_Gene1162	accessory	1853	[Rbromii5AMG]RB5AMG_01198_Pyridoxine_kinase	Rbromii5AMG
Org2_Gene1163	accessory	2872	[Rbromii5AMG]RB5AMG_01199_putative_membrane_protein	Rbromii5AMG
Org2_Gene1164	accessory	2678	[Rbromii5AMG]RB5AMG_01200_molybdenum_cofactor_biosynthesis_protein_A	Rbromii5AMG
Org2_Gene1165	accessory	2713	[Rbromii5AMG]RB5AMG_01201_Ion_channel	Rbromii5AMG
Org2_Gene1166	accessory	752	[Rbromii5AMG]RB5AMG_01202_Na+-dependent_transporters_of_the_SNF_family_protein	Rbromii5AMG
Org2_Gene1167	accessory	1713	[Rbromii5AMG]RB5AMG_01203_hypothetical_protein	Rbromii5AMG
Org2_Gene1168	accessory	3510	[Rbromii5AMG]RB5AMG_01204_hypothetical_protein	Rbromii5AMG
Org2_Gene1169	accessory	2571	[Rbromii5AMG]RB5AMG_01205_hypothetical_protein	Rbromii5AMG
Org2_Gene1170	accessory	2783	[Rbromii5AMG]RB5AMG_01206_Putative_beta-lactamase_HcpC_precursor	Rbromii5AMG
Org2_Gene1174	accessory	3318	[Rbromii5AMG]RB5AMG_01210_Protease_synthase_and_sporulation_negative_regulatory_protein_PAI_1	Rbromii5AMG
Org2_Gene1175	accessory	3313	[Rbromii5AMG]RB5AMG_01211_Low_molecular_weight_protein-tyrosine-phosphatase_YwlE	Rbromii5AMG
Org2_Gene1178	accessory	530	[Rbromii5AMG]RB5AMG_01214_Proline_permease	Rbromii5AMG
Org2_Gene1179	accessory	2772	[Rbromii5AMG]RB5AMG_01215_Xanthine_phosphoribosyltransferase	Rbromii5AMG
Org2_Gene118	accessory	980	[Rbromii5AMG]RB5AMG_00126_Transposase	Rbromii5AMG
Org2_Gene1181	accessory	2616	[Rbromii5AMG]RB5AMG_01218_cytidylate_kinase	Rbromii5AMG
Org2_Gene1182	accessory	923	[Rbromii5AMG]RB5AMG_01219_Allantoate_amidohydrolase	Rbromii5AMG
Org2_Gene1183	accessory	579	[Rbromii5AMG]RB5AMG_01220_NAD-dependent_dihydropyrimidine_dehydrogenase_subunit_PreA	Rbromii5AMG
Org2_Gene1184	accessory	736	[Rbromii5AMG]RB5AMG_01221_D-hydantoinase	Rbromii5AMG
Org2_Gene1185	accessory	1171	[Rbromii5AMG]RB5AMG_01222_ABC_transporter,_substrate-binding_protein,_aliphatic_sulfonates_family	Rbromii5AMG
Org2_Gene1186	accessory	1917	[Rbromii5AMG]RB5AMG_01223_ABC-type_anion_transport_system,_duplicated_permease_component	Rbromii5AMG
Org2_Gene1187	accessory	1900	[Rbromii5AMG]RB5AMG_01224_Bicarbonate_transport_system_permease_protein_CmpB	Rbromii5AMG

Org2_Gene1188	accessory	2032	[Rbromii5AMG]RB5AMG_01225_Aliphatic_sulfonates_import_ATP-binding_protein_SsuB	Rbromii5AMG
Org2_Gene1189	accessory	1135	[Rbromii5AMG]RB5AMG_01226_NAD-dependent_methanol_dehydrogenase	Rbromii5AMG
Org2_Gene1190	accessory	813	[Rbromii5AMG]RB5AMG_01227_Taurine--pyruvate_aminotransferase	Rbromii5AMG
Org2_Gene1191	accessory	1699	[Rbromii5AMG]RB5AMG_01228_hypothetical_protein	Rbromii5AMG
Org2_Gene1192	accessory	2534	[Rbromii5AMG]RB5AMG_01229_Response_regulator_ArlR	Rbromii5AMG
Org2_Gene1193	accessory	646	[Rbromii5AMG]RB5AMG_01230_Signal_transduction_histidine-protein_kinase_ArlS	Rbromii5AMG
Org2_Gene1194	accessory	2117	[Rbromii5AMG]RB5AMG_01231_hypothetical_protein	Rbromii5AMG
Org2_Gene1195	accessory	1420	[Rbromii5AMG]RB5AMG_01232_Lipid_II:glycine_glycyltransferase	Rbromii5AMG
Org2_Gene1196	accessory	1862	[Rbromii5AMG]RB5AMG_01233_Error-prone_DNA_polymerase	Rbromii5AMG
Org2_Gene1197	accessory	935	[Rbromii5AMG]RB5AMG_01234_hypothetical_protein	Rbromii5AMG
Org2_Gene120	accessory	560	[Rbromii5AMG]RB5AMG_00128_Arginine_transport_system_permease_protein_ArtQ	Rbromii5AMG
Org2_Gene1200	accessory	4288	[Rbromii5AMG]RB5AMG_01237_hypothetical_protein	Rbromii5AMG
Org2_Gene1201	accessory	4285	[Rbromii5AMG]RB5AMG_01238_hypothetical_protein	Rbromii5AMG
Org2_Gene1202	accessory	3461	[Rbromii5AMG]RB5AMG_01239_hypothetical_protein	Rbromii5AMG
Org2_Gene1203	accessory	994	[Rbromii5AMG]RB5AMG_01240_H(+)/Cl(-)_exchange_transporter_ClcA	Rbromii5AMG
Org2_Gene1204	accessory	1482	[Rbromii5AMG]RB5AMG_01241_Choloylglycine_hydrolase	Rbromii5AMG
Org2_Gene1205	accessory	4268	[Rbromii5AMG]RB5AMG_01242_hypothetical_protein	Rbromii5AMG
Org2_Gene1206	accessory	1055	[Rbromii5AMG]RB5AMG_01243_Glutamate_permease	Rbromii5AMG
Org2_Gene1207	accessory	1530	[Rbromii5AMG]RB5AMG_01244_TRAP_transporter_solute_receptor,_TAXI_family	Rbromii5AMG
Org2_Gene1209	accessory	631	[Rbromii5AMG]RB5AMG_01246_cell_division_protein_FtsQ	Rbromii5AMG
Org2_Gene1215	accessory	2882	[Rbromii5AMG]RB5AMG_01252_hypothetical_protein	Rbromii5AMG
Org2_Gene1217	accessory	3379	[Rbromii5AMG]RB5AMG_01254_cell_division_protein_MraZ	Rbromii5AMG
Org2_Gene1219	accessory	3116	[Rbromii5AMG]RB5AMG_01256_Phosphopantetheine_adenylyltransferase	Rbromii5AMG
Org2_Gene122	accessory	119	[Rbromii5AMG]RB5AMG_00131_Alpha-amylase_precursor	Rbromii5AMG
Org2_Gene1229	accessory	3080	[Rbromii5AMG]RB5AMG_01266_Undecaprenol_kinase	Rbromii5AMG
Org2_Gene1236	accessory	3082	[Rbromii5AMG]RB5AMG_01273_hypothetical_protein	Rbromii5AMG
Org2_Gene124	accessory	1672	[Rbromii5AMG]RB5AMG_00133_UDP-glucose_4-epimerase	Rbromii5AMG
Org2_Gene1241	accessory	3185	[Rbromii5AMG]RB5AMG_01278_hypothetical_protein	Rbromii5AMG
Org2_Gene1244	accessory	3198	[Rbromii5AMG]RB5AMG_01281_hypothetical_protein	Rbromii5AMG
Org2_Gene1245	accessory	3124	[Rbromii5AMG]RB5AMG_01282_hypothetical_protein	Rbromii5AMG
Org2_Gene1246	accessory	1187	[Rbromii5AMG]RB5AMG_01283_hypothetical_protein	Rbromii5AMG
Org2_Gene1248	accessory	493	[Rbromii5AMG]RB5AMG_01285_hypothetical_protein	Rbromii5AMG
Org2_Gene1250	accessory	2728	[Rbromii5AMG]RB5AMG_01287_Stage_0_sporulation_protein_H	Rbromii5AMG
Org2_Gene1251	accessory	556	[Rbromii5AMG]RB5AMG_01288_Competence_protein_ComM	Rbromii5AMG
Org2_Gene1253	accessory	2486	[Rbromii5AMG]RB5AMG_01290_tRNA_(adenine(22)-N(1))-methyltransferase	Rbromii5AMG
Org2_Gene1254	accessory	2171	[Rbromii5AMG]RB5AMG_01291_metal-binding_protein	Rbromii5AMG
Org2_Gene1256	accessory	785	[Rbromii5AMG]RB5AMG_01293_hypothetical_protein	Rbromii5AMG
Org2_Gene126	accessory	966	[Rbromii5AMG]RB5AMG_00135_mercuric_reductase	Rbromii5AMG
Org2_Gene1260	accessory	1895	[Rbromii5AMG]RB5AMG_01297_Release_factor_glutamine_methyltransferase	Rbromii5AMG
Org2_Gene127	accessory	3152	[Rbromii5AMG]RB5AMG_00136_putative_membrane_protein	Rbromii5AMG
Org2_Gene1270	accessory	2135	[Rbromii5AMG]RB5AMG_01307_hypothetical_protein	Rbromii5AMG
Org2_Gene1271	accessory	588	[Rbromii5AMG]RB5AMG_01308_Fibronectin_type_III_domain_protein	Rbromii5AMG
Org2_Gene1272	accessory	3701	[Rbromii5AMG]RB5AMG_01309_hypothetical_protein	Rbromii5AMG
Org2_Gene1273	accessory	1670	[Rbromii5AMG]RB5AMG_01310_Autolytic_lysozyme	Rbromii5AMG
Org2_Gene1275	accessory	526	[Rbromii5AMG]RB5AMG_01312_Murein_hydrolase_activator_NlpD_precursor	Rbromii5AMG
Org2_Gene1276	accessory	4300	[Rbromii5AMG]RB5AMG_01313_hypothetical_protein	Rbromii5AMG
Org2_Gene128	accessory	3182	[Rbromii5AMG]RB5AMG_00137_putative_integral_membrane_protein	Rbromii5AMG
Org2_Gene1283	accessory	1072	[Rbromii5AMG]RB5AMG_01320_Ferrous-iron_efflux_pump_FieF	Rbromii5AMG
Org2_Gene1284	accessory	1710	[Rbromii5AMG]RB5AMG_01321_hypothetical_protein	Rbromii5AMG
Org2_Gene1287	accessory	2709	[Rbromii5AMG]RB5AMG_01324_hypothetical_protein	Rbromii5AMG
Org2_Gene1288	accessory	653	[Rbromii5AMG]RB5AMG_01325_Amino-acid_carrier_protein_AlsT	Rbromii5AMG
Org2_Gene1290	accessory	1625	[Rbromii5AMG]RB5AMG_01327_putative_amino-acid_metabolite_efflux_pump	Rbromii5AMG
Org2_Gene1291	accessory	1726	[Rbromii5AMG]RB5AMG_01328_Thioredoxin_reductase	Rbromii5AMG
Org2_Gene1292	accessory	3720	[Rbromii5AMG]RB5AMG_01329_Thioredoxin	Rbromii5AMG
Org2_Gene1293	accessory	1932	[Rbromii5AMG]RB5AMG_01330_putative_endonuclease_4	Rbromii5AMG
Org2_Gene1294	accessory	1159	[Rbromii5AMG]RB5AMG_01332_hypothetical_protein	Rbromii5AMG
Org2_Gene1297	accessory	749	[Rbromii5AMG]RB5AMG_01336_putative_aminodeoxychorismate_lyase	Rbromii5AMG
Org2_Gene1298	accessory	2684	[Rbromii5AMG]RB5AMG_01337_5-formyltetrahydrofolate_cyclo-ligase_family_protein	Rbromii5AMG
Org2_Gene13	accessory	3983	[Rbromii5AMG]RB5AMG_00013_hypothetical_protein	Rbromii5AMG
Org2_Gene130	accessory	3520	[Rbromii5AMG]RB5AMG_00139_hypothetical_protein	Rbromii5AMG
Org2_Gene1300	accessory	2072	[Rbromii5AMG]RB5AMG_01339_putative_acetyltransferase_involved_in_intracellular_survival	Rbromii5AMG
Org2_Gene1301	accessory	1737	[Rbromii5AMG]RB5AMG_01340_hypothetical_protein	Rbromii5AMG
Org2_Gene1302	accessory	2455	[Rbromii5AMG]RB5AMG_01341_Thymidylate_kinase	Rbromii5AMG
Org2_Gene1305	accessory	3750	[Rbromii5AMG]RB5AMG_01344_HTH-type_transcriptional_regulator_ImmR	Rbromii5AMG
Org2_Gene1307	accessory	4355	[Rbromii5AMG]RB5AMG_01346_hypothetical_protein	Rbromii5AMG
Org2_Gene131	accessory	3057	[Rbromii5AMG]RB5AMG_00140_Zn-dependent_proteases	Rbromii5AMG
Org2_Gene1336	accessory	372	[Rbromii5AMG]RB5AMG_01376_type_IV_secretion_system_component_VirD4	Rbromii5AMG
Org2_Gene134	accessory	2429	[Rbromii5AMG]RB5AMG_00143_hypothetical_protein	Rbromii5AMG
Org2_Gene1348	accessory	1465	[Rbromii5AMG]RB5AMG_01388_hypothetical_protein	Rbromii5AMG
Org2_Gene1349	accessory	4120	[Rbromii5AMG]RB5AMG_01389_hypothetical_protein	Rbromii5AMG
Org2_Gene135	accessory	1687	[Rbromii5AMG]RB5AMG_00144_radical_SAM_protein	Rbromii5AMG
Org2_Gene1350	accessory	3155	[Rbromii5AMG]RB5AMG_01390_hypothetical_protein	Rbromii5AMG
Org2_Gene1351	accessory	364	[Rbromii5AMG]RB5AMG_01391_Conjugal_transfer_protein_TraG	Rbromii5AMG
Org2_Gene1352	accessory	4163	[Rbromii5AMG]RB5AMG_01392_Maff2_family_protein	Rbromii5AMG
Org2_Gene1353	accessory	1803	[Rbromii5AMG]RB5AMG_01393_hypothetical_protein	Rbromii5AMG
Org2_Gene1354	accessory	3451	[Rbromii5AMG]RB5AMG_01394_PrgI_family_protein	Rbromii5AMG
Org2_Gene1355	accessory	146	[Rbromii5AMG]RB5AMG_01395_type-IV_secretion_system_protein_TraC	Rbromii5AMG
Org2_Gene1356	accessory	1526	[Rbromii5AMG]RB5AMG_01396_DNA_adenine_methyltransferase_YhdJ	Rbromii5AMG
Org2_Gene1357	accessory	225	[Rbromii5AMG]RB5AMG_01397_hypothetical_protein	Rbromii5AMG
Org2_Gene1358	accessory	3912	[Rbromii5AMG]RB5AMG_01398_hypothetical_protein	Rbromii5AMG
Org2_Gene1359	accessory	2334	[Rbromii5AMG]RB5AMG_01399_hypothetical_protein	Rbromii5AMG
Org2_Gene136	accessory	2091	[Rbromii5AMG]RB5AMG_00145_hypothetical_protein	Rbromii5AMG
Org2_Gene1360	accessory	4274	[Rbromii5AMG]RB5AMG_01400_hypothetical_protein	Rbromii5AMG
Org2_Gene1362	accessory	250	[Rbromii5AMG]RB5AMG_01402_DNA_topoisomerase_3	Rbromii5AMG
Org2_Gene1364	accessory	1542	[Rbromii5AMG]RB5AMG_01404_hypothetical_protein	Rbromii5AMG
Org2_Gene1365	accessory	4199	[Rbromii5AMG]RB5AMG_01405_hypothetical_protein	Rbromii5AMG
Org2_Gene1366	accessory	4018	[Rbromii5AMG]RB5AMG_01406_Helix-turn-helix_domain_protein	Rbromii5AMG
Org2_Gene1367	accessory	771	[Rbromii5AMG]RB5AMG_01407_Relaxase/Mobilisation_nuclease_domain_protein	Rbromii5AMG
Org2_Gene1368	accessory	3724	[Rbromii5AMG]RB5AMG_01408_Bacterial_mobilisation_protein_(MobC)	Rbromii5AMG
Org2_Gene1369	accessory	3615	[Rbromii5AMG]RB5AMG_01409_anaerobic_benzoate_catabolism_transcriptional_regulator	Rbromii5AMG
Org2_Gene1370	accessory	4294	[Rbromii5AMG]RB5AMG_01410_hypothetical_protein	Rbromii5AMG
Org2_Gene1371	accessory	1452	[Rbromii5AMG]RB5AMG_01411_putative_dual-specificity_RNA_methyltransferase_RlmN	Rbromii5AMG
Org2_Gene1372	accessory	3304	[Rbromii5AMG]RB5AMG_01412_RNA_polymerase_sigma_factor	Rbromii5AMG
Org2_Gene1373	accessory	3978	[Rbromii5AMG]RB5AMG_01413_Helix-turn-helix_domain_protein	Rbromii5AMG
Org2_Gene1374	accessory	447	[Rbromii5AMG]RB5AMG_01414_hypothetical_protein	Rbromii5AMG
Org2_Gene1395	accessory	1488	[Rbromii5AMG]RB5AMG_01435_hypothetical_protein	Rbromii5AMG
Org2_Gene1396	accessory	1432	[Rbromii5AMG]RB5AMG_01436_hypothetical_protein	Rbromii5AMG
Org2_Gene1397	accessory	2621	[Rbromii5AMG]RB5AMG_01437_putative_ABC_transporter_ATP-binding_protein_YxlF	Rbromii5AMG
Org2_Gene1398	accessory	2402	[Rbromii5AMG]RB5AMG_01438_hypothetical_protein	Rbromii5AMG
Org2_Gene1399	accessory	2172	[Rbromii5AMG]RB5AMG_01439_hypothetical_protein	Rbromii5AMG
Org2_Gene14	accessory	4127	[Rbromii5AMG]RB5AMG_00014_hypothetical_protein	Rbromii5AMG
Org2_Gene140	accessory	1939	[Rbromii5AMG]RB5AMG_00150_Phosphatidate_cytidylyltransferase	Rbromii5AMG
Org2_Gene1400	accessory	2165	[Rbromii5AMG]RB5AMG_01440_hypothetical_protein	Rbromii5AMG
Org2_Gene1401	accessory	373	[Rbromii5AMG]RB5AMG_01441_Adenine_deaminase	Rbromii5AMG
Org2_Gene1402	accessory	1189	[Rbromii5AMG]RB5AMG_01442_Glycerol_dehydrogenase	Rbromii5AMG
Org2_Gene1403	accessory	728	[Rbromii5AMG]RB5AMG_01443_Glutamate-aspartate_carrier_protein	Rbromii5AMG
Org2_Gene1404	accessory	2185	[Rbromii5AMG]RB5AMG_01444_HTH-type_transcriptional_repressor_GlcR	Rbromii5AMG
Org2_Gene1405	accessory	1438	[Rbromii5AMG]RB5AMG_01445_General_stress_protein_69	Rbromii5AMG
Org2_Gene1406	accessory	1487	[Rbromii5AMG]RB5AMG_01446_putative_sugar_kinase_YdjH	Rbromii5AMG
Org2_Gene1407	accessory	1913	[Rbromii5AMG]RB5AMG_01447_D-tagatose-1,6-bisphosphate_aldolase_subunit_GatY	Rbromii5AMG
Org2_Gene1408	accessory	1308	[Rbromii5AMG]RB5AMG_01448_Sorbitol_dehydrogenase	Rbromii5AMG
Org2_Gene1409	accessory	704	[Rbromii5AMG]RB5AMG_01449_Inner_membrane_metabolite_transport_protein_YdjE	Rbromii5AMG
Org2_Gene1410	accessory	2373	[Rbromii5AMG]RB5AMG_01450_D-allulose-6-phosphate_3-epimerase	Rbromii5AMG
Org2_Gene1411	accessory	1232	[Rbromii5AMG]RB5AMG_01451_D-arabitol-phosphate_dehydrogenase	Rbromii5AMG
Org2_Gene1413	accessory	3532	[Rbromii5AMG]RB5AMG_01453_hypothetical_protein	Rbromii5AMG
Org2_Gene1414	accessory	3672	[Rbromii5AMG]RB5AMG_01454_hypothetical_protein	Rbromii5AMG
Org2_Gene1415	accessory	4325	[Rbromii5AMG]RB5AMG_01455_hypothetical_protein	Rbromii5AMG
Org2_Gene1416	accessory	2863	[Rbromii5AMG]RB5AMG_01456_Negative_regulator_of_genetic_competence,_sporulation_and_motility	Rbromii5AMG
Org2_Gene1419	accessory	3612	[Rbromii5AMG]RB5AMG_01459_HTH-type_transcriptional_regulator_ImmR	Rbromii5AMG
Org2_Gene142	accessory	708	[Rbromii5AMG]RB5AMG_00152_Zinc_metalloprotease_RasP	Rbromii5AMG
Org2_Gene1426	accessory	2932	[Rbromii5AMG]RB5AMG_01466_Shikimate_kinase	Rbromii5AMG
Org2_Gene1430	accessory	4282	[Rbromii5AMG]RB5AMG_01470_hypothetical_protein	Rbromii5AMG

Org2_Gene1434	accessory	3891	[Rbromii5AMG]RB5AMG_01474_hypothetical_protein	Rbromii5AMG
Org2_Gene1435	accessory	3622	[Rbromii5AMG]RB5AMG_01475_hypothetical_protein	Rbromii5AMG
Org2_Gene1436	accessory	4070	[Rbromii5AMG]RB5AMG_01476_hypothetical_protein	Rbromii5AMG
Org2_Gene1437	accessory	3178	[Rbromii5AMG]RB5AMG_01477_Phosphodiesterase_YfcE	Rbromii5AMG
Org2_Gene1438	accessory	1827	[Rbromii5AMG]RB5AMG_01478_DNA_polymerase_III_subunit_tau	Rbromii5AMG
Org2_Gene1441	accessory	2222	[Rbromii5AMG]RB5AMG_01481_tRNA1(Val)_(adenine(37)-N6)-methyltransferase	Rbromii5AMG
Org2_Gene1443	accessory	845	[Rbromii5AMG]RB5AMG_01483_Folylpolyglutamate_synthase	Rbromii5AMG
Org2_Gene1447	accessory	3031	[Rbromii5AMG]RB5AMG_01487_Shikimate_kinase_2	Rbromii5AMG
Org2_Gene1449	accessory	281	[Rbromii5AMG]RB5AMG_01489_CotH_protein	Rbromii5AMG
Org2_Gene145	accessory	827	[Rbromii5AMG]RB5AMG_00155_pheromone_autoinducer_2_transporter	Rbromii5AMG
Org2_Gene1450	accessory	1513	[Rbromii5AMG]RB5AMG_01490_hypothetical_protein	Rbromii5AMG
Org2_Gene1456	accessory	4431	[Rbromii5AMG]RB5AMG_01496_Ribosomal_protein_II	Rbromii5AMG
Org2_Gene1483	accessory	3012	[Rbromii5AMG]RB5AMG_01523_Cob(I)yrinic_acid_a,c-diamide_adenosyltransferase	Rbromii5AMG
Org2_Gene1484	accessory	1121	[Rbromii5AMG]RB5AMG_01524_hypothetical_protein	Rbromii5AMG
Org2_Gene1487	accessory	1447	[Rbromii5AMG]RB5AMG_01527_L-Ala--D-Glu_endopeptidase_precursor	Rbromii5AMG
Org2_Gene1488	accessory	4128	[Rbromii5AMG]RB5AMG_01528_hypothetical_protein	Rbromii5AMG
Org2_Gene1489	accessory	1041	[Rbromii5AMG]RB5AMG_01529_Cystathionine_gamma-synthase	Rbromii5AMG
Org2_Gene1490	accessory	3382	[Rbromii5AMG]RB5AMG_01530_hypothetical_protein	Rbromii5AMG
Org2_Gene1491	accessory	295	[Rbromii5AMG]RB5AMG_01531_sortase,_SrtB_family	Rbromii5AMG
Org2_Gene1502	accessory	786	[Rbromii5AMG]RB5AMG_01542_Cyclodextrin-binding_protein_precursor	Rbromii5AMG
Org2_Gene1507	accessory	762	[Rbromii5AMG]RB5AMG_01547_Serine_protease_inhibitor	Rbromii5AMG
Org2_Gene1508	accessory	1154	[Rbromii5AMG]RB5AMG_01548_Processive_diacylglycerol_glucosyltransferase	Rbromii5AMG
Org2_Gene1509	accessory	422	[Rbromii5AMG]RB5AMG_01549_Vancomycin_B-type_resistance_protein_VanW	Rbromii5AMG
Org2_Gene1512	accessory	3666	[Rbromii5AMG]RB5AMG_01552_hypothetical_protein	Rbromii5AMG
Org2_Gene1513	accessory	4279	[Rbromii5AMG]RB5AMG_01553_hypothetical_protein	Rbromii5AMG
Org2_Gene1516	accessory	2930	[Rbromii5AMG]RB5AMG_01556_hypothetical_protein	Rbromii5AMG
Org2_Gene1517	accessory	3449	[Rbromii5AMG]RB5AMG_01557_hypothetical_protein	Rbromii5AMG
Org2_Gene1518	accessory	4322	[Rbromii5AMG]RB5AMG_01558_hypothetical_protein	Rbromii5AMG
Org2_Gene1519	accessory	1269	[Rbromii5AMG]RB5AMG_01559_6-phosphofructokinase	Rbromii5AMG
Org2_Gene1520	accessory	218	[Rbromii5AMG]RB5AMG_01560_Polyphosphate_kinase	Rbromii5AMG
Org2_Gene1521	accessory	751	[Rbromii5AMG]RB5AMG_01561_Na+-dependent_transporters_of_the_SNF_family_protein	Rbromii5AMG
Org2_Gene1524	accessory	1941	[Rbromii5AMG]RB5AMG_01564_hypothetical_protein	Rbromii5AMG
Org2_Gene1525	accessory	2281	[Rbromii5AMG]RB5AMG_01565_sortase,_marine_proteobacterial_type	Rbromii5AMG
Org2_Gene1526	accessory	2727	[Rbromii5AMG]RB5AMG_01566_hypothetical_protein	Rbromii5AMG
Org2_Gene1527	accessory	2288	[Rbromii5AMG]RB5AMG_01567_Capsular_polysaccharide_type_8_biosynthesis_protein_cap8A	Rbromii5AMG
Org2_Gene1528	accessory	2326	[Rbromii5AMG]RB5AMG_01568_Tyrosine-protein_kinase_YwqD	Rbromii5AMG
Org2_Gene1529	accessory	2462	[Rbromii5AMG]RB5AMG_01569_Undecaprenyl_phosphate_N,N'-diacetylbacillosamine_1-phosphate_transferase	Rbromii5AMG
Org2_Gene154	accessory	4057	[Rbromii5AMG]RB5AMG_00164_hypothetical_protein	Rbromii5AMG
Org2_Gene1547	accessory	846	[Rbromii5AMG]RB5AMG_01587_Ribonucleases_G_and_E	Rbromii5AMG
Org2_Gene1548	accessory	1778	[Rbromii5AMG]RB5AMG_01588_hypothetical_protein	Rbromii5AMG
Org2_Gene1549	accessory	1268	[Rbromii5AMG]RB5AMG_01589_hypothetical_protein	Rbromii5AMG
Org2_Gene1550	accessory	512	[Rbromii5AMG]RB5AMG_01590_hypothetical_protein	Rbromii5AMG
Org2_Gene1552	accessory	2385	[Rbromii5AMG]RB5AMG_01592_Zn-dependent_proteases	Rbromii5AMG
Org2_Gene1555	accessory	2625	[Rbromii5AMG]RB5AMG_01595_hypothetical_protein	Rbromii5AMG
Org2_Gene1557	accessory	1186	[Rbromii5AMG]RB5AMG_01597_D-alanyl-D-alanine_carboxypeptidase_DacB_precursor	Rbromii5AMG
Org2_Gene156	accessory	965	[Rbromii5AMG]RB5AMG_00166_hypothetical_protein	Rbromii5AMG
Org2_Gene1562	accessory	2447	[Rbromii5AMG]RB5AMG_01602_hypothetical_protein	Rbromii5AMG
Org2_Gene1563	accessory	351	[Rbromii5AMG]RB5AMG_01603_Fibronectin_type_III_domain_protein	Rbromii5AMG
Org2_Gene1567	accessory	947	[Rbromii5AMG]RB5AMG_01607_hypothetical_protein	Rbromii5AMG
Org2_Gene1568	accessory	185	[Rbromii5AMG]RB5AMG_01608_Cadmium,_zinc_and_cobalt-transporting_ATPase	Rbromii5AMG
Org2_Gene157	accessory	3938	[Rbromii5AMG]RB5AMG_00167_hypothetical_protein	Rbromii5AMG
Org2_Gene1576	accessory	4154	[Rbromii5AMG]RB5AMG_01616_hypothetical_protein	Rbromii5AMG
Org2_Gene1577	accessory	41	[Rbromii5AMG]RB5AMG_01617_Pullulanase_precursor	Rbromii5AMG
Org2_Gene158	accessory	1779	[Rbromii5AMG]RB5AMG_00168_Ribosomal_large_subunit_pseudouridine_synthase_D	Rbromii5AMG
Org2_Gene1580	accessory	3059	[Rbromii5AMG]RB5AMG_01620_hypothetical_protein	Rbromii5AMG
Org2_Gene1581	accessory	2006	[Rbromii5AMG]RB5AMG_01621_sortase,_SrtB_family	Rbromii5AMG
Org2_Gene1582	accessory	816	[Rbromii5AMG]RB5AMG_01622_Putative_transcriptional_regulator_YvhJ	Rbromii5AMG
Org2_Gene1585	accessory	143	[Rbromii5AMG]RB5AMG_01625_putative_membrane_protein	Rbromii5AMG
Org2_Gene1587	accessory	2081	[Rbromii5AMG]RB5AMG_01627_hypothetical_protein	Rbromii5AMG
Org2_Gene1590	accessory	2274	[Rbromii5AMG]RB5AMG_01630_DNA_alkylation_repair_enzyme	Rbromii5AMG
Org2_Gene1591	accessory	2502	[Rbromii5AMG]RB5AMG_01632_Riboflavin_ECF_transporter_S_component_RibU	Rbromii5AMG
Org2_Gene1592	accessory	2798	[Rbromii5AMG]RB5AMG_01634_Folate_ECF_transporter_S_component_FolT	Rbromii5AMG
Org2_Gene1596	accessory	311	[Rbromii5AMG]RB5AMG_01638_1,4-alpha-glucan_branching_enzyme_GlgB	Rbromii5AMG
Org2_Gene1601	accessory	3284	[Rbromii5AMG]RB5AMG_01643_acyl-CoA_thioesterase_YbgC	Rbromii5AMG
Org2_Gene1604	accessory	1661	[Rbromii5AMG]RB5AMG_01646_HTH-type_transcriptional_regulator_GltC	Rbromii5AMG
Org2_Gene1611	accessory	768	[Rbromii5AMG]RB5AMG_01653_CCA-adding_enzyme	Rbromii5AMG
Org2_Gene1612	accessory	167	[Rbromii5AMG]RB5AMG_01654_Calcium-transporting_ATPase	Rbromii5AMG
Org2_Gene1613	accessory	1382	[Rbromii5AMG]RB5AMG_01655_SpoIVB_peptidase_precursor	Rbromii5AMG
Org2_Gene1622	accessory	571	[Rbromii5AMG]RB5AMG_01664_putative_membrane_protein	Rbromii5AMG
Org2_Gene1636	accessory	4379	[Rbromii5AMG]RB5AMG_01678_cyclic_lactone_autoinducer_peptide	Rbromii5AMG
Org2_Gene1637	accessory	2349	[Rbromii5AMG]RB5AMG_01679_Sensory_transduction_protein_LytR	Rbromii5AMG
Org2_Gene1638	accessory	850	[Rbromii5AMG]RB5AMG_01680_hypothetical_protein	Rbromii5AMG
Org2_Gene1639	accessory	3956	[Rbromii5AMG]RB5AMG_01681_putative_transcriptional_regulator	Rbromii5AMG
Org2_Gene164	accessory	944	[Rbromii5AMG]RB5AMG_00174_UDP-glucose_6-dehydrogenase	Rbromii5AMG
Org2_Gene1640	accessory	299	[Rbromii5AMG]RB5AMG_01682_Conjugal_transfer_protein_TraG	Rbromii5AMG
Org2_Gene1641	accessory	1640	[Rbromii5AMG]RB5AMG_01683_DNA_primase	Rbromii5AMG
Org2_Gene1643	accessory	252	[Rbromii5AMG]RB5AMG_01685_hypothetical_protein	Rbromii5AMG
Org2_Gene1645	accessory	3467	[Rbromii5AMG]RB5AMG_01687_hypothetical_protein	Rbromii5AMG
Org2_Gene1646	accessory	2952	[Rbromii5AMG]RB5AMG_01688_AIG2-like_family_protein	Rbromii5AMG
Org2_Gene1648	accessory	4059	[Rbromii5AMG]RB5AMG_01690_hypothetical_protein	Rbromii5AMG
Org2_Gene1649	accessory	1185	[Rbromii5AMG]RB5AMG_01691_hypothetical_protein	Rbromii5AMG
Org2_Gene1651	accessory	350	[Rbromii5AMG]RB5AMG_01693_conjugal_transfer_ATP-binding_protein_TraC	Rbromii5AMG
Org2_Gene1652	accessory	1966	[Rbromii5AMG]RB5AMG_01694_sortase,_SrtB_family	Rbromii5AMG
Org2_Gene1653	accessory	2691	[Rbromii5AMG]RB5AMG_01695_hypothetical_protein	Rbromii5AMG
Org2_Gene1654	accessory	1228	[Rbromii5AMG]RB5AMG_01696_hypothetical_protein	Rbromii5AMG
Org2_Gene1655	accessory	3598	[Rbromii5AMG]RB5AMG_01697_hypothetical_protein	Rbromii5AMG
Org2_Gene1656	accessory	3636	[Rbromii5AMG]RB5AMG_01698_hypothetical_protein	Rbromii5AMG
Org2_Gene1657	accessory	22	[Rbromii5AMG]RB5AMG_01699_putative_outer_membrane_protein	Rbromii5AMG
Org2_Gene1658	accessory	2696	[Rbromii5AMG]RB5AMG_01700_hypothetical_protein	Rbromii5AMG
Org2_Gene1661	accessory	3470	[Rbromii5AMG]RB5AMG_01703_Putative_septation_protein_SpoVG	Rbromii5AMG
Org2_Gene1662	accessory	3868	[Rbromii5AMG]RB5AMG_01704_transcriptional_regulator,_AbrB_family	Rbromii5AMG
Org2_Gene1663	accessory	4167	[Rbromii5AMG]RB5AMG_01705_hypothetical_protein	Rbromii5AMG
Org2_Gene1665	accessory	1451	[Rbromii5AMG]RB5AMG_01707_Modification_methylase_DpnIIA	Rbromii5AMG
Org2_Gene1666	accessory	2057	[Rbromii5AMG]RB5AMG_01708_DNA_(cytosine-5-)-methyltransferase	Rbromii5AMG
Org2_Gene1667	accessory	2120	[Rbromii5AMG]RB5AMG_01709_Modification_methylase_HpaII	Rbromii5AMG
Org2_Gene1668	accessory	3776	[Rbromii5AMG]RB5AMG_01710_hypothetical_protein	Rbromii5AMG
Org2_Gene1669	accessory	4366	[Rbromii5AMG]RB5AMG_01711_hypothetical_protein	Rbromii5AMG
Org2_Gene1670	accessory	4414	[Rbromii5AMG]RB5AMG_01712_hypothetical_protein	Rbromii5AMG
Org2_Gene1671	accessory	1066	[Rbromii5AMG]RB5AMG_01713_tRNA_modification_GTPase_MnmE	Rbromii5AMG
Org2_Gene1673	accessory	1405	[Rbromii5AMG]RB5AMG_01715_Biotin_synthase	Rbromii5AMG
Org2_Gene1674	accessory	1977	[Rbromii5AMG]RB5AMG_01716_hypothetical_protein	Rbromii5AMG
Org2_Gene1675	accessory	1188	[Rbromii5AMG]RB5AMG_01717_hypothetical_protein	Rbromii5AMG
Org2_Gene1677	accessory	2096	[Rbromii5AMG]RB5AMG_01719_hypothetical_protein	Rbromii5AMG
Org2_Gene168	accessory	2669	[Rbromii5AMG]RB5AMG_00178_dTDP-4-dehydrorhamnose_3,5-epimerase	Rbromii5AMG
Org2_Gene1682	accessory	276	[Rbromii5AMG]RB5AMG_01724_Penicillin-binding_protein_2B	Rbromii5AMG
Org2_Gene1683	accessory	3046	[Rbromii5AMG]RB5AMG_01725_rod_shape-determining_protein_MreD	Rbromii5AMG
Org2_Gene1684	accessory	1973	[Rbromii5AMG]RB5AMG_01726_Rod_shape-determining_protein_MreC	Rbromii5AMG
Org2_Gene1687	accessory	262	[Rbromii5AMG]RB5AMG_01729_putative_protease_YhbU_precursor	Rbromii5AMG
Org2_Gene1688	accessory	2560	[Rbromii5AMG]RB5AMG_01730_cell_division_protein_ZapA	Rbromii5AMG
Org2_Gene1689	accessory	327	[Rbromii5AMG]RB5AMG_01731_Alpha-amylase/pullulanase	Rbromii5AMG
Org2_Gene1690	accessory	2176	[Rbromii5AMG]RB5AMG_01732_hypothetical_protein	Rbromii5AMG
Org2_Gene1692	accessory	3328	[Rbromii5AMG]RB5AMG_01734_acyl-CoA_thioesterase_YbgC	Rbromii5AMG
Org2_Gene1694	accessory	2258	[Rbromii5AMG]RB5AMG_01736_cxxc_20_cxxc_protein	Rbromii5AMG
Org2_Gene17	accessory	3492	[Rbromii5AMG]RB5AMG_00017_Putative_PBSX_repressor	Rbromii5AMG
Org2_Gene1703	accessory	1410	[Rbromii5AMG]RB5AMG_01745_Oxa1Ec	Rbromii5AMG
Org2_Gene1707	accessory	2320	[Rbromii5AMG]RB5AMG_01749_Ribosomal_RNA_small_subunit_methyltransferase_G	Rbromii5AMG
Org2_Gene1712	accessory	3623	[Rbromii5AMG]RB5AMG_01754_hypothetical_protein	Rbromii5AMG
Org2_Gene1713	accessory	3466	[Rbromii5AMG]RB5AMG_01755_hypothetical_protein	Rbromii5AMG
Org2_Gene1714	accessory	1987	[Rbromii5AMG]RB5AMG_01756_hypothetical_protein	Rbromii5AMG
Org2_Gene1717	accessory	4367	[Rbromii5AMG]RB5AMG_01762_hypothetical_protein	Rbromii5AMG
Org2_Gene1718	accessory	2977	[Rbromii5AMG]RB5AMG_01763_hypothetical_protein	Rbromii5AMG
Org2_Gene1719	accessory	3407	[Rbromii5AMG]RB5AMG_01764_hypothetical_protein	Rbromii5AMG

Org2_Gene1722	accessory	3213	[Rbromii5AMG]RB5AMG_01767_GtrA-like_protein	Rbromii5AMG
Org2_Gene1725	accessory	106	[Rbromii5AMG]RB5AMG_01770_putative_membrane_protein	Rbromii5AMG
Org2_Gene1729	accessory	497	[Rbromii5AMG]RB5AMG_01774_gliding-associated_putative_ABC_transporter_substrate-binding_component_GldG	Rbromii5AMG
Org2_Gene1730	accessory	548	[Rbromii5AMG]RB5AMG_01775_hypothetical_protein	Rbromii5AMG
Org2_Gene1731	accessory	2193	[Rbromii5AMG]RB5AMG_01776_Sensory_transduction_protein_LytR	Rbromii5AMG
Org2_Gene1744	accessory	2531	[Rbromii5AMG]RB5AMG_01789_cAMP_regulatory_protein	Rbromii5AMG
Org2_Gene1745	accessory	1607	[Rbromii5AMG]RB5AMG_01790_Bacterial_Ig-like_domain_(group_2)	Rbromii5AMG
Org2_Gene1746	accessory	1873	[Rbromii5AMG]RB5AMG_01791_Carboxylesterase_NlhH	Rbromii5AMG
Org2_Gene1747	accessory	1921	[Rbromii5AMG]RB5AMG_01792_Melibiose_operon_regulatory_protein	Rbromii5AMG
Org2_Gene1748	accessory	1999	[Rbromii5AMG]RB5AMG_01793_Arabinose_operon_regulatory_protein	Rbromii5AMG
Org2_Gene1749	accessory	1143	[Rbromii5AMG]RB5AMG_01794_Resuscitation-promoting_factor_Rpf2_precursor	Rbromii5AMG
Org2_Gene1750	accessory	2852	[Rbromii5AMG]RB5AMG_01795_hypothetical_protein	Rbromii5AMG
Org2_Gene1751	accessory	1120	[Rbromii5AMG]RB5AMG_01796_hypothetical_protein	Rbromii5AMG
Org2_Gene1753	accessory	2121	[Rbromii5AMG]RB5AMG_01802_Glycine/sarcosine_N-methyltransferase	Rbromii5AMG
Org2_Gene1756	accessory	3536	[Rbromii5AMG]RB5AMG_01805_HTH-type_transcriptional_repressor_YtrA	Rbromii5AMG
Org2_Gene1757	accessory	1716	[Rbromii5AMG]RB5AMG_01806_ABC_transporter_ATP-binding_protein_YtrB	Rbromii5AMG
Org2_Gene1758	accessory	219	[Rbromii5AMG]RB5AMG_01807_hypothetical_protein	Rbromii5AMG
Org2_Gene1759	accessory	2886	[Rbromii5AMG]RB5AMG_01808_Prolyl-tRNA_deacylase_ProX	Rbromii5AMG
Org2_Gene1760	accessory	1112	[Rbromii5AMG]RB5AMG_01809_hypothetical_protein	Rbromii5AMG
Org2_Gene1761	accessory	417	[Rbromii5AMG]RB5AMG_01810_Sensor_protein_QseC	Rbromii5AMG
Org2_Gene1762	accessory	2433	[Rbromii5AMG]RB5AMG_01811_Staphylococcal_respiratory_response_protein_A	Rbromii5AMG
Org2_Gene1763	accessory	3462	[Rbromii5AMG]RB5AMG_01812_putative_manganese-dependent_inorganic_pyrophosphatase	Rbromii5AMG
Org2_Gene1764	accessory	3229	[Rbromii5AMG]RB5AMG_01813_hypothetical_protein	Rbromii5AMG
Org2_Gene1765	accessory	2951	[Rbromii5AMG]RB5AMG_01814_serine/threonine_protein_kinase	Rbromii5AMG
Org2_Gene1766	accessory	1140	[Rbromii5AMG]RB5AMG_01815_hypothetical_protein	Rbromii5AMG
Org2_Gene1768	accessory	509	[Rbromii5AMG]RB5AMG_01817_hypothetical_protein	Rbromii5AMG
Org2_Gene1769	accessory	2321	[Rbromii5AMG]RB5AMG_01818_ABC-type_transporter_ATP-binding_protein_EcsA	Rbromii5AMG
Org2_Gene1770	accessory	2554	[Rbromii5AMG]RB5AMG_01819_hemolysin	Rbromii5AMG
Org2_Gene1771	accessory	369	[Rbromii5AMG]RB5AMG_01820_PhoU-like_phosphate_uptake_regulator	Rbromii5AMG
Org2_Gene1772	accessory	466	[Rbromii5AMG]RB5AMG_01821_Alkaline_phosphatase_synthesis_sensor_protein_PhoR	Rbromii5AMG
Org2_Gene1773	accessory	2472	[Rbromii5AMG]RB5AMG_01822_Sensory_transduction_protein_regX3	Rbromii5AMG
Org2_Gene1774	accessory	2551	[Rbromii5AMG]RB5AMG_01823_Phosphate_uptake_regulator	Rbromii5AMG
Org2_Gene1776	accessory	1612	[Rbromii5AMG]RB5AMG_01825_Phosphate_transport_system_permease_protein_PstA	Rbromii5AMG
Org2_Gene1777	accessory	1802	[Rbromii5AMG]RB5AMG_01826_Phosphate_transport_system_permease_protein_PstC	Rbromii5AMG
Org2_Gene1778	accessory	1642	[Rbromii5AMG]RB5AMG_01827_Phosphate-binding_protein_PstS_2_precursor	Rbromii5AMG
Org2_Gene1780	accessory	3369	[Rbromii5AMG]RB5AMG_01829_hypothetical_protein	Rbromii5AMG
Org2_Gene1785	accessory	57	[Rbromii5AMG]RB5AMG_01834_outer_membrane-specific_lipoprotein_transporter_subunit_LolE	Rbromii5AMG
Org2_Gene1786	accessory	2375	[Rbromii5AMG]RB5AMG_01835_putative_ABC_transporter_ATP-binding_protein/MT1014	Rbromii5AMG
Org2_Gene1790	accessory	2969	[Rbromii5AMG]RB5AMG_01839_Protease_synthase_and_sporulation_negative_regulatory_protein_PAI_1	Rbromii5AMG
Org2_Gene1791	accessory	2099	[Rbromii5AMG]RB5AMG_01840_Endo-1,4-beta-xylanase_Z_precursor	Rbromii5AMG
Org2_Gene1799	accessory	3989	[Rbromii5AMG]RB5AMG_01849_Preprotein_translocase_subunit_SecE	Rbromii5AMG
Org2_Gene18	accessory	1839	[Rbromii5AMG]RB5AMG_00019_putative_integral_membrane_protein	Rbromii5AMG
Org2_Gene1805	accessory	3763	[Rbromii5AMG]RB5AMG_01855_Endoglucanase_A_precursor	Rbromii5AMG
Org2_Gene1806	accessory	4185	[Rbromii5AMG]RB5AMG_01856_HTH-type_transcriptional_regulator_ImmR	Rbromii5AMG
Org2_Gene1807	accessory	3996	[Rbromii5AMG]RB5AMG_01857_hypothetical_protein	Rbromii5AMG
Org2_Gene1808	accessory	2134	[Rbromii5AMG]RB5AMG_01858_vancomycin_high_temperature_exclusion_protein	Rbromii5AMG
Org2_Gene1809	accessory	545	[Rbromii5AMG]RB5AMG_01859_Iron_hydrogenase_1	Rbromii5AMG
Org2_Gene1810	accessory	3314	[Rbromii5AMG]RB5AMG_01860_putative_membrane_protein	Rbromii5AMG
Org2_Gene1815	accessory	3163	[Rbromii5AMG]RB5AMG_01865_Biotin_carboxyl_carrier_protein_of_acetyl-CoA_carboxylase	Rbromii5AMG
Org2_Gene1820	accessory	3725	[Rbromii5AMG]RB5AMG_01870_hypothetical_protein	Rbromii5AMG
Org2_Gene1821	accessory	140	[Rbromii5AMG]RB5AMG_01871_Penicillin-binding_protein_F	Rbromii5AMG
Org2_Gene1824	accessory	2329	[Rbromii5AMG]RB5AMG_01874_hypothetical_protein	Rbromii5AMG
Org2_Gene1827	accessory	2569	[Rbromii5AMG]RB5AMG_01877_DNA-binding_transcriptional_dual_regulator_Crp	Rbromii5AMG
Org2_Gene1828	accessory	2142	[Rbromii5AMG]RB5AMG_01878_HTH-type_transcriptional_repressor_GlcR	Rbromii5AMG
Org2_Gene1829	accessory	1678	[Rbromii5AMG]RB5AMG_01879_Tagatose-6-phosphate_kinase	Rbromii5AMG
Org2_Gene1830	accessory	308	[Rbromii5AMG]RB5AMG_01880_EIIABC-Fru	Rbromii5AMG
Org2_Gene1832	accessory	430	[Rbromii5AMG]RB5AMG_01882_Phosphoenolpyruvate-protein_phosphotransferase	Rbromii5AMG
Org2_Gene1833	accessory	2420	[Rbromii5AMG]RB5AMG_01883_hypothetical_protein	Rbromii5AMG
Org2_Gene1834	accessory	2517	[Rbromii5AMG]RB5AMG_01884_Phosphoglycolate_phosphatase	Rbromii5AMG
Org2_Gene1835	accessory	2712	[Rbromii5AMG]RB5AMG_01885_Kynurenine_formamidase	Rbromii5AMG
Org2_Gene1836	accessory	324	[Rbromii5AMG]RB5AMG_01886_hypothetical_protein	Rbromii5AMG
Org2_Gene1849	accessory	1829	[Rbromii5AMG]RB5AMG_01901_DNA-damage-inducible_protein_D	Rbromii5AMG
Org2_Gene1851	accessory	524	[Rbromii5AMG]RB5AMG_01903_Recombinase	Rbromii5AMG
Org2_Gene1853	accessory	4146	[Rbromii5AMG]RB5AMG_01905_hypothetical_protein	Rbromii5AMG
Org2_Gene1854	accessory	348	[Rbromii5AMG]RB5AMG_01906_Trifunctional_nucleotide_phosphoesterase_protein_YfkN_precursor	Rbromii5AMG
Org2_Gene1855	accessory	2180	[Rbromii5AMG]RB5AMG_01907_Pyruvate_formate-lyase-activating_enzyme	Rbromii5AMG
Org2_Gene1857	accessory	3085	[Rbromii5AMG]RB5AMG_01909_putative_HD_superfamily_hydrolase	Rbromii5AMG
Org2_Gene1859	accessory	1054	[Rbromii5AMG]RB5AMG_01911_Putative_L,D-transpeptidase_YciB_precursor	Rbromii5AMG
Org2_Gene1862	accessory	3393	[Rbromii5AMG]RB5AMG_01914_hypothetical_protein	Rbromii5AMG
Org2_Gene1864	accessory	2561	[Rbromii5AMG]RB5AMG_01916_Vitamin_B12_dependent_methionine_synthase,_activation_domain	Rbromii5AMG
Org2_Gene1867	accessory	2556	[Rbromii5AMG]RB5AMG_01919_hypothetical_protein	Rbromii5AMG
Org2_Gene187	accessory	3211	[Rbromii5AMG]RB5AMG_00197_Transposase	Rbromii5AMG
Org2_Gene1870	accessory	1665	[Rbromii5AMG]RB5AMG_01922_hypothetical_protein	Rbromii5AMG
Org2_Gene1871	accessory	2076	[Rbromii5AMG]RB5AMG_01923_sortase	Rbromii5AMG
Org2_Gene1872	accessory	1569	[Rbromii5AMG]RB5AMG_01924_sortase	Rbromii5AMG
Org2_Gene1874	accessory	19	[Rbromii5AMG]RB5AMG_01926_fibro-slime_domain_protein	Rbromii5AMG
Org2_Gene1879	accessory	3590	[Rbromii5AMG]RB5AMG_01931_hypothetical_protein	Rbromii5AMG
Org2_Gene1880	accessory	2881	[Rbromii5AMG]RB5AMG_01932_Biotin_ECF_transporter_S_component_BioY	Rbromii5AMG
Org2_Gene1882	accessory	406	[Rbromii5AMG]RB5AMG_01934_Serine_protease_Do-like_HtrA	Rbromii5AMG
Org2_Gene1883	accessory	2043	[Rbromii5AMG]RB5AMG_01935_hypothetical_protein	Rbromii5AMG
Org2_Gene1886	accessory	2636	[Rbromii5AMG]RB5AMG_01938_hypothetical_protein	Rbromii5AMG
Org2_Gene189	accessory	328	[Rbromii5AMG]RB5AMG_00199_LPS_biosynthesis_protein	Rbromii5AMG
Org2_Gene1891	accessory	2984	[Rbromii5AMG]RB5AMG_01943_hypothetical_protein	Rbromii5AMG
Org2_Gene1892	accessory	4240	[Rbromii5AMG]RB5AMG_01944_hypothetical_protein	Rbromii5AMG
Org2_Gene1897	accessory	3805	[Rbromii5AMG]RB5AMG_01950_hypothetical_protein	Rbromii5AMG
Org2_Gene1898	accessory	3711	[Rbromii5AMG]RB5AMG_01951_putative_ribosomal_protein_YlxQ	Rbromii5AMG
Org2_Gene190	accessory	1266	[Rbromii5AMG]RB5AMG_00200_dTDP-4-dehydro-6-deoxyglucose_reductase	Rbromii5AMG
Org2_Gene1901	accessory	1575	[Rbromii5AMG]RB5AMG_01954_Bifunctional_oligoribonuclease_and_PAP_phosphatase_NrnA	Rbromii5AMG
Org2_Gene1902	accessory	1648	[Rbromii5AMG]RB5AMG_01955_tRNA_pseudouridine_synthase_B	Rbromii5AMG
Org2_Gene1903	accessory	2465	[Rbromii5AMG]RB5AMG_01956_putative_ABC_transporter_ATP-binding_protein/MT1014	Rbromii5AMG
Org2_Gene1904	accessory	116	[Rbromii5AMG]RB5AMG_01957_ABC_transporter_permease_YtrF_precursor	Rbromii5AMG
Org2_Gene1905	accessory	2549	[Rbromii5AMG]RB5AMG_01958_Phosphate_regulon_transcriptional_regulatory_protein_PhoB	Rbromii5AMG
Org2_Gene1906	accessory	991	[Rbromii5AMG]RB5AMG_01959_Signal-transduction_histidine_kinase_senX3	Rbromii5AMG
Org2_Gene1907	accessory	30	[Rbromii5AMG]RB5AMG_01960_Alpha-amylase_precursor	Rbromii5AMG
Org2_Gene1909	accessory	3576	[Rbromii5AMG]RB5AMG_01962_putative_protein_encoded_in_hypervariable_junctions_of_pilus_gene_clusters	Rbromii5AMG
Org2_Gene191	accessory	2225	[Rbromii5AMG]RB5AMG_00201_2-C-methyl-D-erythritol_4-phosphate_cytidylyltransferase_1	Rbromii5AMG
Org2_Gene1919	accessory	2358	[Rbromii5AMG]RB5AMG_01972_putative_membrane_protein	Rbromii5AMG
Org2_Gene1920	accessory	2146	[Rbromii5AMG]RB5AMG_01973_putative_membrane_protein	Rbromii5AMG
Org2_Gene1921	accessory	2210	[Rbromii5AMG]RB5AMG_01974_ABC_transporter_efflux_protein,_DrrB_family	Rbromii5AMG
Org2_Gene1922	accessory	2271	[Rbromii5AMG]RB5AMG_01975_Daunorubicin/doxorubicin_resistance_ATP-binding_protein_DrrA	Rbromii5AMG
Org2_Gene1923	accessory	3416	[Rbromii5AMG]RB5AMG_01976_transcriptional_repressor_DicA	Rbromii5AMG
Org2_Gene1924	accessory	2746	[Rbromii5AMG]RB5AMG_01977_hypothetical_protein	Rbromii5AMG
Org2_Gene1925	accessory	1937	[Rbromii5AMG]RB5AMG_01978_hypothetical_protein	Rbromii5AMG
Org2_Gene1927	accessory	1225	[Rbromii5AMG]RB5AMG_01980_hypothetical_protein	Rbromii5AMG
Org2_Gene193	accessory	2734	[Rbromii5AMG]RB5AMG_00203_hypothetical_protein	Rbromii5AMG
Org2_Gene1931	accessory	2855	[Rbromii5AMG]RB5AMG_01984_HSP-70_cofactor	Rbromii5AMG
Org2_Gene1932	accessory	1368	[Rbromii5AMG]RB5AMG_01985_Heat-inducible_transcription_repressor_HrcA	Rbromii5AMG
Org2_Gene1933	accessory	3920	[Rbromii5AMG]RB5AMG_01986_hypothetical_protein	Rbromii5AMG
Org2_Gene1934	accessory	2668	[Rbromii5AMG]RB5AMG_01987_Adenosine_monophosphate-protein_transferase_NmFic	Rbromii5AMG
Org2_Gene1936	accessory	3442	[Rbromii5AMG]RB5AMG_01990_Putative_8-oxo-dGTP_diphosphatase_YtkD	Rbromii5AMG
Org2_Gene1937	accessory	3998	[Rbromii5AMG]RB5AMG_01991_hypothetical_protein	Rbromii5AMG
Org2_Gene194	accessory	51	[Rbromii5AMG]RB5AMG_00204_ATP-dependent_helicase/nuclease_subunit_A	Rbromii5AMG
Org2_Gene1942	accessory	1348	[Rbromii5AMG]RB5AMG_01996_Flagellar_biosynthetic_protein_FliU	Rbromii5AMG
Org2_Gene1943	accessory	3792	[Rbromii5AMG]RB5AMG_01997_hypothetical_protein	Rbromii5AMG
Org2_Gene1949	accessory	2046	[Rbromii5AMG]RB5AMG_02003_Indole-3-glycerol_phosphate_synthase	Rbromii5AMG
Org2_Gene195	accessory	60	[Rbromii5AMG]RB5AMG_00205_ATP-dependent_helicase/deoxyribonuclease_subunit_B	Rbromii5AMG
Org2_Gene1950	accessory	1401	[Rbromii5AMG]RB5AMG_02004_Anthranilate_phosphoribosyltransferase	Rbromii5AMG
Org2_Gene1951	accessory	2800	[Rbromii5AMG]RB5AMG_02005_Anthranilate_synthase_component_II	Rbromii5AMG
Org2_Gene1952	accessory	620	[Rbromii5AMG]RB5AMG_02006_Anthranilate_synthase_component_1	Rbromii5AMG
Org2_Gene1955	accessory	476	[Rbromii5AMG]RB5AMG_02010_Acetyl-coenzyme_A_synthetase	Rbromii5AMG
Org2_Gene196	accessory	2689	[Rbromii5AMG]RB5AMG_00206_stage_II_sporulation_protein_R	Rbromii5AMG
Org2_Gene1962	accessory	2338	[Rbromii5AMG]RB5AMG_02017_hypothetical_protein	Rbromii5AMG

Org2_Gene1963	accessory	1475	[Rbromii5AMG]RB5AMG_02018_O-acetyl-ADP-ribose_deacetylase	Rbromii5AMG
Org2_Gene1964	accessory	2562	[Rbromii5AMG]RB5AMG_02019_putative_protein_encoded_in_toxicity_protection_region_of_plasmid_R478,_contains_von_Willebrand_factor_(vWF)_do	Rbromii5AMG
Org2_Gene1965	accessory	735	[Rbromii5AMG]RB5AMG_02020_putative_membrane_protein	Rbromii5AMG
Org2_Gene1966	accessory	3712	[Rbromii5AMG]RB5AMG_02021_Transcriptional_regulator_YqjI	Rbromii5AMG
Org2_Gene1968	accessory	652	[Rbromii5AMG]RB5AMG_02023_hypothetical_protein	Rbromii5AMG
Org2_Gene1970	accessory	284	[Rbromii5AMG]RB5AMG_02025_Fructose-1,6-bisphosphatase_class_3	Rbromii5AMG
Org2_Gene1972	accessory	242	[Rbromii5AMG]RB5AMG_02027_Lipoteichoic_acid_synthase_1	Rbromii5AMG
Org2_Gene1973	accessory	896	[Rbromii5AMG]RB5AMG_02028_putative_protein_involved_in_cytokinesis,_contains_TGc_(transglutaminase/protease-like)_domain	Rbromii5AMG
Org2_Gene1974	accessory	969	[Rbromii5AMG]RB5AMG_02029_putative_protein_involved_in_cytokinesis,_contains_TGc_(transglutaminase/protease-like)_domain	Rbromii5AMG
Org2_Gene1977	accessory	2357	[Rbromii5AMG]RB5AMG_02032_hypothetical_protein	Rbromii5AMG
Org2_Gene1979	accessory	1899	[Rbromii5AMG]RB5AMG_02034_Putative_PBSX_repressor	Rbromii5AMG
Org2_Gene1983	accessory	3362	[Rbromii5AMG]RB5AMG_02038_hypothetical_protein	Rbromii5AMG
Org2_Gene1991	accessory	3184	[Rbromii5AMG]RB5AMG_02048_hypothetical_protein	Rbromii5AMG
Org2_Gene1992	accessory	1403	[Rbromii5AMG]RB5AMG_02049_bacterial_surface_protein_26-residue_repeat	Rbromii5AMG
Org2_Gene1993	accessory	1765	[Rbromii5AMG]RB5AMG_02050_hypothetical_protein	Rbromii5AMG
Org2_Gene1996	accessory	454	[Rbromii5AMG]RB5AMG_02053_hypothetical_protein	Rbromii5AMG
Org2_Gene1997	accessory	847	[Rbromii5AMG]RB5AMG_02054_sensory_histidine_kinase_UhpB	Rbromii5AMG
Org2_Gene1998	accessory	2580	[Rbromii5AMG]RB5AMG_02055_Response_regulator_protein_VraR	Rbromii5AMG
Org2_Gene1999	accessory	3446	[Rbromii5AMG]RB5AMG_02056_hypothetical_protein	Rbromii5AMG
Org2_Gene2	accessory	518	[Rbromii5AMG]RB5AMG_00002_hypothetical_protein	Rbromii5AMG
Org2_Gene200	accessory	3054	[Rbromii5AMG]RB5AMG_00210_hypothetical_protein	Rbromii5AMG
Org2_Gene2000	accessory	3507	[Rbromii5AMG]RB5AMG_02057_hypothetical_protein	Rbromii5AMG
Org2_Gene2001	accessory	2754	[Rbromii5AMG]RB5AMG_02058_hypothetical_protein	Rbromii5AMG
Org2_Gene2002	accessory	534	[Rbromii5AMG]RB5AMG_02059_hypothetical_protein	Rbromii5AMG
Org2_Gene2003	accessory	1086	[Rbromii5AMG]RB5AMG_02060_hypothetical_protein	Rbromii5AMG
Org2_Gene2004	accessory	3506	[Rbromii5AMG]RB5AMG_02061_hypothetical_protein	Rbromii5AMG
Org2_Gene2005	accessory	3546	[Rbromii5AMG]RB5AMG_02062_hypothetical_protein	Rbromii5AMG
Org2_Gene2006	accessory	357	[Rbromii5AMG]RB5AMG_02063_ATP-dependent_DNA_helicase_RecQ	Rbromii5AMG
Org2_Gene2007	accessory	1208	[Rbromii5AMG]RB5AMG_02064_hypothetical_protein	Rbromii5AMG
Org2_Gene2009	accessory	4372	[Rbromii5AMG]RB5AMG_02066_Rubredoxin	Rbromii5AMG
Org2_Gene2011	accessory	3483	[Rbromii5AMG]RB5AMG_02068_Peroxide-responsive_repressor_PerR	Rbromii5AMG
Org2_Gene2012	accessory	1828	[Rbromii5AMG]RB5AMG_02069_Poly-beta-1,6-N-acetyl-D-glucosamine_N-deacetylase_precursor	Rbromii5AMG
Org2_Gene2013	accessory	504	[Rbromii5AMG]RB5AMG_02070_putative_bicarbonate_transporter,_IctB_family	Rbromii5AMG
Org2_Gene2014	accessory	2543	[Rbromii5AMG]RB5AMG_02071_5'-nucleotidase	Rbromii5AMG
Org2_Gene2015	accessory	455	[Rbromii5AMG]RB5AMG_02072_Sigma-24	Rbromii5AMG
Org2_Gene2016	accessory	3782	[Rbromii5AMG]RB5AMG_02073_hypothetical_protein	Rbromii5AMG
Org2_Gene202	accessory	580	[Rbromii5AMG]RB5AMG_00212_Lipid_A_core_-_O-antigen_ligase	Rbromii5AMG
Org2_Gene2020	accessory	4426	[Rbromii5AMG]RB5AMG_02077_hypothetical_protein	Rbromii5AMG
Org2_Gene2026	accessory	337	[Rbromii5AMG]RB5AMG_02085_LPS_biosynthesis_protein	Rbromii5AMG
Org2_Gene2027	accessory	1239	[Rbromii5AMG]RB5AMG_02086_dTDP-glucose_4,6-dehydratase	Rbromii5AMG
Org2_Gene2034	accessory	69	[Rbromii5AMG]RB5AMG_02093_Alpha-amylase_precursor	Rbromii5AMG
Org2_Gene2035	accessory	2808	[Rbromii5AMG]RB5AMG_02094_hypothetical_protein	Rbromii5AMG
Org2_Gene2036	accessory	1392	[Rbromii5AMG]RB5AMG_02095_Rod_shape-determining_protein_MreB	Rbromii5AMG
Org2_Gene2037	accessory	2496	[Rbromii5AMG]RB5AMG_02096_DNA_utilization_protein_GntX	Rbromii5AMG
Org2_Gene2039	accessory	2287	[Rbromii5AMG]RB5AMG_02098_putative_glutamine_amidotransferase	Rbromii5AMG
Org2_Gene204	accessory	3844	[Rbromii5AMG]RB5AMG_00214_hypothetical_protein	Rbromii5AMG
Org2_Gene2041	accessory	4251	[Rbromii5AMG]RB5AMG_02100_hypothetical_protein	Rbromii5AMG
Org2_Gene2042	accessory	2697	[Rbromii5AMG]RB5AMG_02101_Putative_acetyltransferase	Rbromii5AMG
Org2_Gene2044	accessory	2667	[Rbromii5AMG]RB5AMG_02103_Undecaprenyl-diphosphatase_BcrC	Rbromii5AMG
Org2_Gene2045	accessory	4123	[Rbromii5AMG]RB5AMG_02104_hypothetical_protein	Rbromii5AMG
Org2_Gene2047	accessory	713	[Rbromii5AMG]RB5AMG_02107_dihydrolipoamide_dehydrogenase	Rbromii5AMG
Org2_Gene2048	accessory	395	[Rbromii5AMG]RB5AMG_02110_DNA_polymerase_III_subunit_gamma/tau	Rbromii5AMG
Org2_Gene2052	accessory	1614	[Rbromii5AMG]RB5AMG_02114_hypothetical_protein	Rbromii5AMG
Org2_Gene206	accessory	438	[Rbromii5AMG]RB5AMG_00217_hypothetical_protein	Rbromii5AMG
Org2_Gene2060	accessory	754	[Rbromii5AMG]RB5AMG_02123_putative_ATPase	Rbromii5AMG
Org2_Gene2061	accessory	2377	[Rbromii5AMG]RB5AMG_02124_hypothetical_protein	Rbromii5AMG
Org2_Gene2062	accessory	3656	[Rbromii5AMG]RB5AMG_02125_hypothetical_protein	Rbromii5AMG
Org2_Gene2063	accessory	3212	[Rbromii5AMG]RB5AMG_02126_hypothetical_protein	Rbromii5AMG
Org2_Gene2064	accessory	2409	[Rbromii5AMG]RB5AMG_02127_hypothetical_protein	Rbromii5AMG
Org2_Gene2065	accessory	1954	[Rbromii5AMG]RB5AMG_02128_ABC-2_family_transporter_protein	Rbromii5AMG
Org2_Gene2066	accessory	2381	[Rbromii5AMG]RB5AMG_02129_hypothetical_protein	Rbromii5AMG
Org2_Gene2067	accessory	1919	[Rbromii5AMG]RB5AMG_02130_ABC-2_family_transporter_protein	Rbromii5AMG
Org2_Gene2068	accessory	2523	[Rbromii5AMG]RB5AMG_02131_Fluoroquinolones_export_ATP-binding_proteinc/MT2762	Rbromii5AMG
Org2_Gene2069	accessory	2776	[Rbromii5AMG]RB5AMG_02132_hypothetical_protein	Rbromii5AMG
Org2_Gene207	accessory	1815	[Rbromii5AMG]RB5AMG_00218_Fatty_acid-binding_protein	Rbromii5AMG
Org2_Gene2070	accessory	4342	[Rbromii5AMG]RB5AMG_02133_hypothetical_protein	Rbromii5AMG
Org2_Gene2074	accessory	3165	[Rbromii5AMG]RB5AMG_02137_flavodoxin	Rbromii5AMG
Org2_Gene2076	accessory	3036	[Rbromii5AMG]RB5AMG_02139_flavodoxin	Rbromii5AMG
Org2_Gene2078	accessory	759	[Rbromii5AMG]RB5AMG_02141_D-alanyl-D-alanine_carboxypeptidase_DacB_precursor	Rbromii5AMG
Org2_Gene208	accessory	987	[Rbromii5AMG]RB5AMG_00219_hypothetical_protein	Rbromii5AMG
Org2_Gene2082	accessory	1077	[Rbromii5AMG]RB5AMG_02145_sporulation_protein_YqfD	Rbromii5AMG
Org2_Gene2083	accessory	3933	[Rbromii5AMG]RB5AMG_02146_sporulation_protein_YqfC	Rbromii5AMG
Org2_Gene2084	accessory	1279	[Rbromii5AMG]RB5AMG_02147_Sporulation_integral_membrane_protein_YlbJ	Rbromii5AMG
Org2_Gene2086	accessory	3822	[Rbromii5AMG]RB5AMG_02149_Putative_undecaprenyl-diphosphatase_YbjG	Rbromii5AMG
Org2_Gene2089	accessory	1906	[Rbromii5AMG]RB5AMG_02152_ComEC_family_competence_protein	Rbromii5AMG
Org2_Gene209	accessory	3045	[Rbromii5AMG]RB5AMG_00220_hypothetical_protein	Rbromii5AMG
Org2_Gene2091	accessory	104	[Rbromii5AMG]RB5AMG_02154_Stage_III_sporulation_protein_E	Rbromii5AMG
Org2_Gene2095	accessory	3481	[Rbromii5AMG]RB5AMG_02158_putative_membrane_protein	Rbromii5AMG
Org2_Gene2097	accessory	1334	[Rbromii5AMG]RB5AMG_02160_DNA_polymerase_III_subunit_delta	Rbromii5AMG
Org2_Gene2098	accessory	204	[Rbromii5AMG]RB5AMG_02161_DNA_internalization-related_competence_protein_ComEC/Rec2	Rbromii5AMG
Org2_Gene2101	accessory	2279	[Rbromii5AMG]RB5AMG_02164_G3P_acyltransferase	Rbromii5AMG
Org2_Gene2103	accessory	848	[Rbromii5AMG]RB5AMG_02166_putative_FeS-containing_Cyanobacterial-specific_oxidoreductase	Rbromii5AMG
Org2_Gene2106	accessory	2390	[Rbromii5AMG]RB5AMG_02169_hypothetical_protein	Rbromii5AMG
Org2_Gene2107	accessory	2328	[Rbromii5AMG]RB5AMG_02170_hypothetical_protein	Rbromii5AMG
Org2_Gene2108	accessory	3526	[Rbromii5AMG]RB5AMG_02171_Penicillinase_repressor	Rbromii5AMG
Org2_Gene2109	accessory	1033	[Rbromii5AMG]RB5AMG_02172_Regulatory_protein_BlaR1	Rbromii5AMG
Org2_Gene2110	accessory	3816	[Rbromii5AMG]RB5AMG_02173_hypothetical_protein	Rbromii5AMG
Org2_Gene2111	accessory	1092	[Rbromii5AMG]RB5AMG_02174_RmuC_family_protein	Rbromii5AMG
Org2_Gene2113	accessory	1394	[Rbromii5AMG]RB5AMG_02176_Putative_bifunctional_phosphatase/peptidyl-prolyl_cis-trans_isomerase	Rbromii5AMG
Org2_Gene2115	accessory	1616	[Rbromii5AMG]RB5AMG_02178_regulatory_ATPase_RavA	Rbromii5AMG
Org2_Gene2116	accessory	1511	[Rbromii5AMG]RB5AMG_02179_hypothetical_protein	Rbromii5AMG
Org2_Gene2117	accessory	226	[Rbromii5AMG]RB5AMG_02180_Protein-glutamine_gamma-glutamyltransferase	Rbromii5AMG
Org2_Gene2118	accessory	4304	[Rbromii5AMG]RB5AMG_02181_hypothetical_protein	Rbromii5AMG
Org2_Gene2119	accessory	3302	[Rbromii5AMG]RB5AMG_02182_Transcriptional_regulatory_protein_YpdB	Rbromii5AMG
Org2_Gene2120	accessory	3275	[Rbromii5AMG]RB5AMG_02183_hypothetical_protein	Rbromii5AMG
Org2_Gene2122	accessory	1598	[Rbromii5AMG]RB5AMG_02185_ABC-2_family_transporter_protein	Rbromii5AMG
Org2_Gene2123	accessory	1207	[Rbromii5AMG]RB5AMG_02186_5-methyltetrahydropteroyltriglutamate--homocysteine_methyltransferase	Rbromii5AMG
Org2_Gene2124	accessory	1711	[Rbromii5AMG]RB5AMG_02187_Cys_regulon_transcriptional_activator	Rbromii5AMG
Org2_Gene2125	accessory	2985	[Rbromii5AMG]RB5AMG_02188_FMN_reductase_[NAD(P)H]	Rbromii5AMG
Org2_Gene2126	accessory	2909	[Rbromii5AMG]RB5AMG_02189_malonic_semialdehyde_reductase	Rbromii5AMG
Org2_Gene2127	accessory	1553	[Rbromii5AMG]RB5AMG_02190_auxin_efflux_carrier	Rbromii5AMG
Org2_Gene2128	accessory	4303	[Rbromii5AMG]RB5AMG_02191_hypothetical_protein	Rbromii5AMG
Org2_Gene2133	accessory	4179	[Rbromii5AMG]RB5AMG_02204_hypothetical_protein	Rbromii5AMG
Org2_Gene2137	accessory	1928	[Rbromii5AMG]RB5AMG_02208_putative_phosphatase_YwpJ	Rbromii5AMG
Org2_Gene2138	accessory	3405	[Rbromii5AMG]RB5AMG_02209_hypothetical_protein	Rbromii5AMG
Org2_Gene214	accessory	2602	[Rbromii5AMG]RB5AMG_00226_Thiamine_pyrophosphokinase	Rbromii5AMG
Org2_Gene2140	accessory	3242	[Rbromii5AMG]RB5AMG_02211_C_GCAxxG_C_C_family_protein	Rbromii5AMG
Org2_Gene2143	accessory	1729	[Rbromii5AMG]RB5AMG_02214_Gamma-D-glutamyl-L-diamino_acid_endopeptidase_1	Rbromii5AMG
Org2_Gene2145	accessory	1600	[Rbromii5AMG]RB5AMG_02216_Riboflavin_biosynthesis_protein_RibF	Rbromii5AMG
Org2_Gene2146	accessory	1990	[Rbromii5AMG]RB5AMG_02217_Nucleotidyltransferase_domain_protein	Rbromii5AMG
Org2_Gene2147	accessory	678	[Rbromii5AMG]RB5AMG_02218_Na(+)/drug_antiporter	Rbromii5AMG
Org2_Gene2148	accessory	2715	[Rbromii5AMG]RB5AMG_02219_LOG_family_protein_YvdD	Rbromii5AMG
Org2_Gene2150	accessory	1981	[Rbromii5AMG]RB5AMG_02221_hypothetical_protein	Rbromii5AMG
Org2_Gene2151	accessory	2017	[Rbromii5AMG]RB5AMG_02222_hypothetical_protein	Rbromii5AMG
Org2_Gene2152	accessory	1962	[Rbromii5AMG]RB5AMG_02223_hypothetical_protein	Rbromii5AMG
Org2_Gene2153	accessory	2212	[Rbromii5AMG]RB5AMG_02224_Signal_peptidase_I_W	Rbromii5AMG
Org2_Gene2154	accessory	552	[Rbromii5AMG]RB5AMG_02225_hypothetical_protein	Rbromii5AMG
Org2_Gene2155	accessory	130	[Rbromii5AMG]RB5AMG_02226_chromosome_segregation_protein_SMC	Rbromii5AMG
Org2_Gene2156	accessory	1423	[Rbromii5AMG]RB5AMG_02227_hypothetical_protein	Rbromii5AMG
Org2_Gene2157	accessory	314	[Rbromii5AMG]RB5AMG_02228_hypothetical_protein	Rbromii5AMG
Org2_Gene2158	accessory	67	[Rbromii5AMG]RB5AMG_02229_repeat	Rbromii5AMG
Org2_Gene2159	accessory	948	[Rbromii5AMG]RB5AMG_02230_Bacterial_Ig-like_domain_(group_2)	Rbromii5AMG

Org2_Gene2160	accessory	2216	[Rbromii5AMG]RB5AMG_02231_Signal_peptidase_I_W	Rbromii5AMG
Org2_Gene2162	accessory	198	[Rbromii5AMG]RB5AMG_02233_Beta/alpha-amylase_precursor	Rbromii5AMG
Org2_Gene2164	accessory	3241	[Rbromii5AMG]RB5AMG_02235_hypothetical_protein	Rbromii5AMG
Org2_Gene2165	accessory	1580	[Rbromii5AMG]RB5AMG_02236_auxin_efflux_carrier	Rbromii5AMG
Org2_Gene2166	accessory	1425	[Rbromii5AMG]RB5AMG_02237_hypothetical_protein	Rbromii5AMG
Org2_Gene2167	accessory	1840	[Rbromii5AMG]RB5AMG_02238_Regulatory_protein_SoxS	Rbromii5AMG
Org2_Gene2168	accessory	2025	[Rbromii5AMG]RB5AMG_02239_hypothetical_protein	Rbromii5AMG
Org2_Gene2171	accessory	557	[Rbromii5AMG]RB5AMG_02242_putative_protein_involved_in_cytokinesis,_contains_TGc_(transglutaminase/protease-like)_domain	Rbromii5AMG
Org2_Gene2173	accessory	1060	[Rbromii5AMG]RB5AMG_02244_Alanine_racemase	Rbromii5AMG
Org2_Gene2174	accessory	1372	[Rbromii5AMG]RB5AMG_02245_hypothetical_protein	Rbromii5AMG
Org2_Gene219	accessory	779	[Rbromii5AMG]RB5AMG_00231_Ribosomal_RNA_small_subunit_methyltransferase_B	Rbromii5AMG
Org2_Gene225	accessory	2761	[Rbromii5AMG]RB5AMG_00237_Guanylate_kinase	Rbromii5AMG
Org2_Gene229	accessory	797	[Rbromii5AMG]RB5AMG_00241_hypothetical_protein	Rbromii5AMG
Org2_Gene23	accessory	2628	[Rbromii5AMG]RB5AMG_00024_CDP-diacylglycerol--glycerol-3-phosphate_3-phosphatidyltransferase	Rbromii5AMG
Org2_Gene237	accessory	211	[Rbromii5AMG]RB5AMG_00249_Dockerin_type_I_repeat	Rbromii5AMG
Org2_Gene24	accessory	2453	[Rbromii5AMG]RB5AMG_00025_Colicin_V_production_protein	Rbromii5AMG
Org2_Gene240	accessory	425	[Rbromii5AMG]RB5AMG_00252_Alpha-amylase_precursor	Rbromii5AMG
Org2_Gene241	accessory	1295	[Rbromii5AMG]RB5AMG_00253_glycosyltransferase	Rbromii5AMG
Org2_Gene242	accessory	1725	[Rbromii5AMG]RB5AMG_00254_hypothetical_protein	Rbromii5AMG
Org2_Gene246	accessory	3777	[Rbromii5AMG]RB5AMG_00258_putative_ribosomal_protein	Rbromii5AMG
Org2_Gene25	accessory	865	[Rbromii5AMG]RB5AMG_00026_hypothetical_protein	Rbromii5AMG
Org2_Gene250	accessory	915	[Rbromii5AMG]RB5AMG_00262_Peptidase_M16_inactive_domain_protein	Rbromii5AMG
Org2_Gene251	accessory	514	[Rbromii5AMG]RB5AMG_00263_Internalin-A_precursor	Rbromii5AMG
Org2_Gene253	accessory	1114	[Rbromii5AMG]RB5AMG_00265_Fucose_4-O-acetylase	Rbromii5AMG
Org2_Gene254	accessory	838	[Rbromii5AMG]RB5AMG_00266_sensor_histidine_kinase_DpiB	Rbromii5AMG
Org2_Gene255	accessory	2309	[Rbromii5AMG]RB5AMG_00267_Transcriptional_regulatory_protein_YpdB	Rbromii5AMG
Org2_Gene259	accessory	888	[Rbromii5AMG]RB5AMG_00271_Galactokinase	Rbromii5AMG
Org2_Gene260	accessory	604	[Rbromii5AMG]RB5AMG_00272_Thiomethylgalactoside_permease_II	Rbromii5AMG
Org2_Gene261	accessory	1395	[Rbromii5AMG]RB5AMG_00273_Glucose-resistance_amylase_regulator	Rbromii5AMG
Org2_Gene262	accessory	2113	[Rbromii5AMG]RB5AMG_00274_Sulfite_exporter_TauE/SafE	Rbromii5AMG
Org2_Gene263	accessory	1577	[Rbromii5AMG]RB5AMG_00275_hypothetical_protein	Rbromii5AMG
Org2_Gene271	accessory	3525	[Rbromii5AMG]RB5AMG_00283_hypothetical_protein	Rbromii5AMG
Org2_Gene272	accessory	1116	[Rbromii5AMG]RB5AMG_00284_Plasmid_recombination_enzyme	Rbromii5AMG
Org2_Gene273	accessory	1136	[Rbromii5AMG]RB5AMG_00285_Replicative_DNA_helicase	Rbromii5AMG
Org2_Gene274	accessory	3692	[Rbromii5AMG]RB5AMG_00286_hypothetical_protein	Rbromii5AMG
Org2_Gene297	accessory	234	[Rbromii5AMG]RB5AMG_00309_Recombinase	Rbromii5AMG
Org2_Gene299	accessory	1387	[Rbromii5AMG]RB5AMG_00311_DNA_repair_protein_RadA	Rbromii5AMG
Org2_Gene3	accessory	247	[Rbromii5AMG]RB5AMG_00003_HAD_hydrolase,_family_IA,_variant_1	Rbromii5AMG
Org2_Gene300	accessory	917	[Rbromii5AMG]RB5AMG_00312_Plasmid_recombination_enzyme	Rbromii5AMG
Org2_Gene314	accessory	760	[Rbromii5AMG]RB5AMG_00327_hypothetical_protein	Rbromii5AMG
Org2_Gene315	accessory	4229	[Rbromii5AMG]RB5AMG_00328_hypothetical_protein	Rbromii5AMG
Org2_Gene316	accessory	151	[Rbromii5AMG]RB5AMG_00329_X-X-X-Leu-X-X-Gly_heptad_repeats	Rbromii5AMG
Org2_Gene318	accessory	2828	[Rbromii5AMG]RB5AMG_00331_putative_dihydroxyacetone_kinase_regulator	Rbromii5AMG
Org2_Gene319	accessory	2600	[Rbromii5AMG]RB5AMG_00332_4'-phosphopantetheinyl_transferase_sfp	Rbromii5AMG
Org2_Gene320	accessory	3106	[Rbromii5AMG]RB5AMG_00333_deoxycytidylate_deaminase	Rbromii5AMG
Org2_Gene324	accessory	3624	[Rbromii5AMG]RB5AMG_00337_hypothetical_protein	Rbromii5AMG
Org2_Gene325	accessory	1245	[Rbromii5AMG]RB5AMG_00338_hypothetical_protein	Rbromii5AMG
Org2_Gene326	accessory	1336	[Rbromii5AMG]RB5AMG_00339_Signal_transduction_histidine-protein_kinase_BaeS	Rbromii5AMG
Org2_Gene327	accessory	2466	[Rbromii5AMG]RB5AMG_00340_Heme_response_regulator_HssR	Rbromii5AMG
Org2_Gene328	accessory	777	[Rbromii5AMG]RB5AMG_00341_Multidrug_export_protein_MepA	Rbromii5AMG
Org2_Gene35	accessory	3575	[Rbromii5AMG]RB5AMG_00036_hypothetical_protein	Rbromii5AMG
Org2_Gene353	accessory	1071	[Rbromii5AMG]RB5AMG_00366_NADH_peroxidase	Rbromii5AMG
Org2_Gene361	accessory	2360	[Rbromii5AMG]RB5AMG_00374_Transglutaminase-like_superfamily_protein	Rbromii5AMG
Org2_Gene37	accessory	3090	[Rbromii5AMG]RB5AMG_00038_hypothetical_protein	Rbromii5AMG
Org2_Gene378	accessory	181	[Rbromii5AMG]RB5AMG_00391_type-IV_secretion_system_protein_TraC	Rbromii5AMG
Org2_Gene379	accessory	3255	[Rbromii5AMG]RB5AMG_00392_PrgI_family_protein	Rbromii5AMG
Org2_Gene382	accessory	1907	[Rbromii5AMG]RB5AMG_00395_hypothetical_protein	Rbromii5AMG
Org2_Gene383	accessory	3601	[Rbromii5AMG]RB5AMG_00396_hypothetical_protein	Rbromii5AMG
Org2_Gene4	accessory	503	[Rbromii5AMG]RB5AMG_00004_4-hydroxy-2-oxovalerate_aldolase	Rbromii5AMG
Org2_Gene40	accessory	4435	[Rbromii5AMG]RB5AMG_00041_hypothetical_protein	Rbromii5AMG
Org2_Gene409	accessory	1945	[Rbromii5AMG]RB5AMG_00422_hypothetical_protein	Rbromii5AMG
Org2_Gene41	accessory	1020	[Rbromii5AMG]RB5AMG_00042_FtsX-like_permease_family_protein	Rbromii5AMG
Org2_Gene410	accessory	825	[Rbromii5AMG]RB5AMG_00423_Septal_ring_factor	Rbromii5AMG
Org2_Gene411	accessory	1709	[Rbromii5AMG]RB5AMG_00424_Cell_division_protein_FtsX	Rbromii5AMG
Org2_Gene415	accessory	2298	[Rbromii5AMG]RB5AMG_00428_Ribosomal_small_subunit_pseudouridine_synthase_A	Rbromii5AMG
Org2_Gene416	accessory	3180	[Rbromii5AMG]RB5AMG_00429_hypothetical_protein	Rbromii5AMG
Org2_Gene417	accessory	2489	[Rbromii5AMG]RB5AMG_00430_5'-nucleotidase	Rbromii5AMG
Org2_Gene42	accessory	2439	[Rbromii5AMG]RB5AMG_00043_Lipoprotein-releasing_system_ATP-binding_protein_LolD	Rbromii5AMG
Org2_Gene420	accessory	4416	[Rbromii5AMG]RB5AMG_00433_hypothetical_protein	Rbromii5AMG
Org2_Gene424	accessory	2630	[Rbromii5AMG]RB5AMG_00437_putative_membrane_protein	Rbromii5AMG
Org2_Gene425	accessory	1502	[Rbromii5AMG]RB5AMG_00438_Bifunctional_protein_BirA	Rbromii5AMG
Org2_Gene427	accessory	2306	[Rbromii5AMG]RB5AMG_00440_phosphoglycolate_phosphatase	Rbromii5AMG
Org2_Gene429	accessory	3047	[Rbromii5AMG]RB5AMG_00442_hypothetical_protein	Rbromii5AMG
Org2_Gene43	accessory	773	[Rbromii5AMG]RB5AMG_00044_FtsX-like_permease_family_protein	Rbromii5AMG
Org2_Gene430	accessory	2250	[Rbromii5AMG]RB5AMG_00443_Transcriptional_regulatory_protein_YpdB	Rbromii5AMG
Org2_Gene431	accessory	841	[Rbromii5AMG]RB5AMG_00444_hypothetical_protein	Rbromii5AMG
Org2_Gene432	accessory	968	[Rbromii5AMG]RB5AMG_00445_CHAP_domain_protein	Rbromii5AMG
Org2_Gene433	accessory	3584	[Rbromii5AMG]RB5AMG_00446_four_helix_bundle_protein	Rbromii5AMG
Org2_Gene435	accessory	2624	[Rbromii5AMG]RB5AMG_00450_Soluble_lytic_murein_transglycosylase_precursor	Rbromii5AMG
Org2_Gene436	accessory	2647	[Rbromii5AMG]RB5AMG_00451_Dephospho-CoA_kinase	Rbromii5AMG
Org2_Gene440	accessory	2548	[Rbromii5AMG]RB5AMG_00455_hypothetical_protein	Rbromii5AMG
Org2_Gene444	accessory	2780	[Rbromii5AMG]RB5AMG_00459_hypothetical_protein	Rbromii5AMG
Org2_Gene445	accessory	856	[Rbromii5AMG]RB5AMG_00460_Inner_membrane_protein_YbaL	Rbromii5AMG
Org2_Gene446	accessory	3398	[Rbromii5AMG]RB5AMG_00461_hypothetical_protein	Rbromii5AMG
Org2_Gene447	accessory	1471	[Rbromii5AMG]RB5AMG_00462_L-asparaginase_1	Rbromii5AMG
Org2_Gene448	accessory	3201	[Rbromii5AMG]RB5AMG_00463_PPOX_class_probable_F420-dependent_enzyme	Rbromii5AMG
Org2_Gene449	accessory	872	[Rbromii5AMG]RB5AMG_00464_hypothetical_protein	Rbromii5AMG
Org2_Gene450	accessory	1573	[Rbromii5AMG]RB5AMG_00466_hypothetical_protein	Rbromii5AMG
Org2_Gene451	accessory	3748	[Rbromii5AMG]RB5AMG_00467_hypothetical_protein	Rbromii5AMG
Org2_Gene452	accessory	184	[Rbromii5AMG]RB5AMG_00468_Stage_II_sporulation_protein_E	Rbromii5AMG
Org2_Gene454	accessory	4058	[Rbromii5AMG]RB5AMG_00470_HTH-type_transcriptional_regulator_ImmR	Rbromii5AMG
Org2_Gene46	accessory	1850	[Rbromii5AMG]RB5AMG_00047_hypothetical_protein	Rbromii5AMG
Org2_Gene460	accessory	478	[Rbromii5AMG]RB5AMG_00476_Phosphoserine_phosphatase_RsbU	Rbromii5AMG
Org2_Gene463	accessory	1585	[Rbromii5AMG]RB5AMG_00479_Tyrosine_recombinase_XerC	Rbromii5AMG
Org2_Gene464	accessory	293	[Rbromii5AMG]RB5AMG_00484_Collagen_triple_helix_repeat_(20_copies)	Rbromii5AMG
Org2_Gene465	accessory	2680	[Rbromii5AMG]RB5AMG_00485_DNA-binding_transcriptional_repressor_AcrR	Rbromii5AMG
Org2_Gene466	accessory	1157	[Rbromii5AMG]RB5AMG_00486_Signal_transduction_histidine-protein_kinase_ArlS	Rbromii5AMG
Org2_Gene467	accessory	2442	[Rbromii5AMG]RB5AMG_00487_Transcriptional_regulatory_protein_QseB	Rbromii5AMG
Org2_Gene468	accessory	1457	[Rbromii5AMG]RB5AMG_00488_Putative_electron_transport_protein_YccM	Rbromii5AMG
Org2_Gene469	accessory	1508	[Rbromii5AMG]RB5AMG_00489_Cytochrome_c_biogenesis_protein_CycY	Rbromii5AMG
Org2_Gene47	accessory	1911	[Rbromii5AMG]RB5AMG_00048_putative_membrane_protein	Rbromii5AMG
Org2_Gene471	accessory	3625	[Rbromii5AMG]RB5AMG_00491_transcriptional_regulator,_Acidobacterial,_PadR-family	Rbromii5AMG
Org2_Gene472	accessory	2753	[Rbromii5AMG]RB5AMG_00492_hypothetical_protein	Rbromii5AMG
Org2_Gene474	accessory	2504	[Rbromii5AMG]RB5AMG_00494_hypothetical_protein	Rbromii5AMG
Org2_Gene476	accessory	2978	[Rbromii5AMG]RB5AMG_00496_hypothetical_protein	Rbromii5AMG
Org2_Gene477	accessory	2042	[Rbromii5AMG]RB5AMG_00497_CAAX_prenyl_protease-related_protein	Rbromii5AMG
Org2_Gene478	accessory	3168	[Rbromii5AMG]RB5AMG_00498_hypothetical_protein	Rbromii5AMG
Org2_Gene479	accessory	2961	[Rbromii5AMG]RB5AMG_00499_hypothetical_protein	Rbromii5AMG
Org2_Gene48	accessory	1967	[Rbromii5AMG]RB5AMG_00050_Hydroxyethylthiazole_kinase	Rbromii5AMG
Org2_Gene480	accessory	1758	[Rbromii5AMG]RB5AMG_00500_hypothetical_protein	Rbromii5AMG
Org2_Gene481	accessory	3433	[Rbromii5AMG]RB5AMG_00501_hypothetical_protein	Rbromii5AMG
Org2_Gene483	accessory	3356	[Rbromii5AMG]RB5AMG_00503_NifU-like_protein	Rbromii5AMG
Org2_Gene484	accessory	989	[Rbromii5AMG]RB5AMG_00504_putative_cysteine_desulfurase	Rbromii5AMG
Org2_Gene485	accessory	1257	[Rbromii5AMG]RB5AMG_00505_FeS_cluster_assembly_protein_SufD	Rbromii5AMG
Org2_Gene486	accessory	682	[Rbromii5AMG]RB5AMG_00506_FeS_cluster_assembly_protein_SufB	Rbromii5AMG
Org2_Gene487	accessory	2167	[Rbromii5AMG]RB5AMG_00507_putative_ABC_transporter_ATP-binding_protein	Rbromii5AMG
Org2_Gene488	accessory	3306	[Rbromii5AMG]RB5AMG_00508_HTH-type_transcriptional_regulator_IscR	Rbromii5AMG
Org2_Gene489	accessory	2708	[Rbromii5AMG]RB5AMG_00509_hypothetical_protein	Rbromii5AMG
Org2_Gene49	accessory	2565	[Rbromii5AMG]RB5AMG_00051_Thiamine-phosphate_synthase	Rbromii5AMG
Org2_Gene490	accessory	3972	[Rbromii5AMG]RB5AMG_00510_pro-sigmaK_processing_inhibitor_BofA	Rbromii5AMG
Org2_Gene5	accessory	2407	[Rbromii5AMG]RB5AMG_00005_3-deoxy-manno-octulosonate_cytidylyltransferase	Rbromii5AMG
Org2_Gene50	accessory	2498	[Rbromii5AMG]RB5AMG_00052_Phosphorylated_carbohydrates_phosphatase	Rbromii5AMG

Org2_Gene501	accessory	1790	[Rbromii5AMG]RB5AMG_00521_Radical_SAM_superfamily_protein	Rbromii5AMG
Org2_Gene502	accessory	2915	[Rbromii5AMG]RB5AMG_00522_hypothetical_protein	Rbromii5AMG
Org2_Gene503	accessory	2541	[Rbromii5AMG]RB5AMG_00523_hypothetical_protein	Rbromii5AMG
Org2_Gene504	accessory	1547	[Rbromii5AMG]RB5AMG_00524_putative_integral_membrane_protein	Rbromii5AMG
Org2_Gene505	accessory	2477	[Rbromii5AMG]RB5AMG_00525_stage_II_sporulation_protein_M	Rbromii5AMG
Org2_Gene507	accessory	138	[Rbromii5AMG]RB5AMG_00527_invasion_associated_secreted_endopeptidase	Rbromii5AMG
Org2_Gene508	accessory	2699	[Rbromii5AMG]RB5AMG_00528_hypothetical_protein	Rbromii5AMG
Org2_Gene509	accessory	2650	[Rbromii5AMG]RB5AMG_00529_Chaperone_protein_DnaJ	Rbromii5AMG
Org2_Gene51	accessory	1982	[Rbromii5AMG]RB5AMG_00053_Hydroxymethylpyrimidine/phosphomethylpyrimidine_kinase	Rbromii5AMG
Org2_Gene510	accessory	1817	[Rbromii5AMG]RB5AMG_00530_hypothetical_protein	Rbromii5AMG
Org2_Gene511	accessory	2829	[Rbromii5AMG]RB5AMG_00531_HAD_hydrolase,_family_IB	Rbromii5AMG
Org2_Gene513	accessory	2585	[Rbromii5AMG]RB5AMG_00534_phosphoglycolate_phosphatase	Rbromii5AMG
Org2_Gene514	accessory	1470	[Rbromii5AMG]RB5AMG_00536_hypothetical_protein	Rbromii5AMG
Org2_Gene515	accessory	482	[Rbromii5AMG]RB5AMG_00537_D-alanyl-lipoteichoic_acid_biosynthesis_protein_DltB	Rbromii5AMG
Org2_Gene516	accessory	4091	[Rbromii5AMG]RB5AMG_00538_D-alanine--poly(phosphoribitol)_ligase_subunit_2	Rbromii5AMG
Org2_Gene517	accessory	1073	[Rbromii5AMG]RB5AMG_00539_L-glutamyl-[BtrI_acyl-carrier_protein]_decarboxylase	Rbromii5AMG
Org2_Gene518	accessory	567	[Rbromii5AMG]RB5AMG_00540_Tyrocidine_synthase_I	Rbromii5AMG
Org2_Gene519	accessory	790	[Rbromii5AMG]RB5AMG_00541_Sensor_histidine_kinase_YycG	Rbromii5AMG
Org2_Gene52	accessory	1032	[Rbromii5AMG]RB5AMG_00054_Cytosine_permease	Rbromii5AMG
Org2_Gene520	accessory	2085	[Rbromii5AMG]RB5AMG_00542_Transcriptional_regulatory_protein_WalR	Rbromii5AMG
Org2_Gene521	accessory	3133	[Rbromii5AMG]RB5AMG_00543_hypothetical_protein	Rbromii5AMG
Org2_Gene522	accessory	4143	[Rbromii5AMG]RB5AMG_00544_hypothetical_protein	Rbromii5AMG
Org2_Gene523	accessory	1630	[Rbromii5AMG]RB5AMG_00545_ComEC_family_competence_protein	Rbromii5AMG
Org2_Gene526	accessory	3132	[Rbromii5AMG]RB5AMG_00548_transcriptional_regulator_SlyA	Rbromii5AMG
Org2_Gene527	accessory	2276	[Rbromii5AMG]RB5AMG_00549_hypothetical_protein	Rbromii5AMG
Org2_Gene528	accessory	1507	[Rbromii5AMG]RB5AMG_00550_ribonuclease_BN/unknown_domain_fusion_protein	Rbromii5AMG
Org2_Gene530	accessory	2563	[Rbromii5AMG]RB5AMG_00552_Demethylrebeccamycin-D-glucose_O-methyltransferase	Rbromii5AMG
Org2_Gene531	accessory	2542	[Rbromii5AMG]RB5AMG_00553_hypothetical_protein	Rbromii5AMG
Org2_Gene537	accessory	427	[Rbromii5AMG]RB5AMG_00559_Serine_protease_Do-like_HtrB	Rbromii5AMG
Org2_Gene538	accessory	3137	[Rbromii5AMG]RB5AMG_00560_hypothetical_protein	Rbromii5AMG
Org2_Gene539	accessory	1731	[Rbromii5AMG]RB5AMG_00561_sporulation_inhibitor_KapD	Rbromii5AMG
Org2_Gene54	accessory	688	[Rbromii5AMG]RB5AMG_00056_putative_ATPase_(AAA+_superfamily)	Rbromii5AMG
Org2_Gene544	accessory	71	[Rbromii5AMG]RB5AMG_00566_Pullulanase_precursor	Rbromii5AMG
Org2_Gene546	accessory	930	[Rbromii5AMG]RB5AMG_00568_Enolase	Rbromii5AMG
Org2_Gene547	accessory	3818	[Rbromii5AMG]RB5AMG_00569_Muramidase-2_precursor	Rbromii5AMG
Org2_Gene553	accessory	2014	[Rbromii5AMG]RB5AMG_00575_D-alanyl-D-alanine_carboxypeptidase	Rbromii5AMG
Org2_Gene555	accessory	842	[Rbromii5AMG]RB5AMG_00578_hypothetical_protein	Rbromii5AMG
Org2_Gene556	accessory	2657	[Rbromii5AMG]RB5AMG_00579_hypothetical_protein	Rbromii5AMG
Org2_Gene557	accessory	3835	[Rbromii5AMG]RB5AMG_00580_RNA_polymerase_sigma_factor	Rbromii5AMG
Org2_Gene558	accessory	4191	[Rbromii5AMG]RB5AMG_00581_hypothetical_protein	Rbromii5AMG
Org2_Gene559	accessory	696	[Rbromii5AMG]RB5AMG_00582_Multidrug_export_protein_MepA	Rbromii5AMG
Org2_Gene560	accessory	2112	[Rbromii5AMG]RB5AMG_00583_N-acetylmuramoyl-L-alanine_amidase	Rbromii5AMG
Org2_Gene561	accessory	4071	[Rbromii5AMG]RB5AMG_00584_hypothetical_protein	Rbromii5AMG
Org2_Gene563	accessory	2065	[Rbromii5AMG]RB5AMG_00586_hypothetical_protein	Rbromii5AMG
Org2_Gene567	accessory	804	[Rbromii5AMG]RB5AMG_00590_tRNA(Ile)-lysidine_synthase	Rbromii5AMG
Org2_Gene570	accessory	294	[Rbromii5AMG]RB5AMG_00593_Bifunctional_oligoribonuclease_and_PAP_phosphatase_NrnA	Rbromii5AMG
Org2_Gene575	accessory	2242	[Rbromii5AMG]RB5AMG_00598_Tyrosine-protein_phosphatase_CpsB	Rbromii5AMG
Org2_Gene578	accessory	2478	[Rbromii5AMG]RB5AMG_00601_Putative_PBSX_repressor	Rbromii5AMG
Org2_Gene580	accessory	2988	[Rbromii5AMG]RB5AMG_00603_hypothetical_protein	Rbromii5AMG
Org2_Gene581	accessory	2884	[Rbromii5AMG]RB5AMG_00604_Cd1	Rbromii5AMG
Org2_Gene582	accessory	699	[Rbromii5AMG]RB5AMG_00605_Magnesium_transporter_MgtE	Rbromii5AMG
Org2_Gene583	accessory	2787	[Rbromii5AMG]RB5AMG_00606_putative_nicotinate-nucleotide_adenylyltransferase	Rbromii5AMG
Org2_Gene584	accessory	1792	[Rbromii5AMG]RB5AMG_00607_Nicotinamide_nucleotide_repair_protein	Rbromii5AMG
Org2_Gene585	accessory	2902	[Rbromii5AMG]RB5AMG_00608_hypothetical_protein	Rbromii5AMG
Org2_Gene592	accessory	3952	[Rbromii5AMG]RB5AMG_00616_addiction_module_antitoxin,_RelB/DinJ_family	Rbromii5AMG
Org2_Gene593	accessory	3927	[Rbromii5AMG]RB5AMG_00617_mRNA_interferase_YafQ	Rbromii5AMG
Org2_Gene597	accessory	2651	[Rbromii5AMG]RB5AMG_00621_N-(5'-phosphoribosyl)anthranilate_isomerase	Rbromii5AMG
Org2_Gene598	accessory	1599	[Rbromii5AMG]RB5AMG_00622_Cysteine_synthase	Rbromii5AMG
Org2_Gene6	accessory	575	[Rbromii5AMG]RB5AMG_00006_hypothetical_protein	Rbromii5AMG
Org2_Gene600	accessory	3351	[Rbromii5AMG]RB5AMG_00624_Cysteine_metabolism_repressor	Rbromii5AMG
Org2_Gene601	accessory	698	[Rbromii5AMG]RB5AMG_00625_putative_adenine_permease_PurP	Rbromii5AMG
Org2_Gene603	accessory	3109	[Rbromii5AMG]RB5AMG_00628_CarD-like/TRCF_domain_protein	Rbromii5AMG
Org2_Gene604	accessory	273	[Rbromii5AMG]RB5AMG_00629_Bacterial_Ig-like_domain_(group_2)	Rbromii5AMG
Org2_Gene605	accessory	959	[Rbromii5AMG]RB5AMG_00630_Beta-hexosaminidase_A_precursor	Rbromii5AMG
Org2_Gene606	accessory	3179	[Rbromii5AMG]RB5AMG_00631_hypothetical_protein	Rbromii5AMG
Org2_Gene607	accessory	2471	[Rbromii5AMG]RB5AMG_00632_Peptidyl-prolyl_cis-trans_isomerase_A_precursor	Rbromii5AMG
Org2_Gene608	accessory	1891	[Rbromii5AMG]RB5AMG_00633_Teichoic_acid_translocation_permease_protein_TagG	Rbromii5AMG
Org2_Gene609	accessory	901	[Rbromii5AMG]RB5AMG_00634_Teichoic_acids_export_ATP-binding_protein_TagH	Rbromii5AMG
Org2_Gene612	accessory	4415	[Rbromii5AMG]RB5AMG_00637_hypothetical_protein	Rbromii5AMG
Org2_Gene615	accessory	3899	[Rbromii5AMG]RB5AMG_00640_hypothetical_protein	Rbromii5AMG
Org2_Gene617	accessory	4135	[Rbromii5AMG]RB5AMG_00643_hypothetical_protein	Rbromii5AMG
Org2_Gene618	accessory	3944	[Rbromii5AMG]RB5AMG_00644_addiction_module_toxin,_RelE/StbE_family	Rbromii5AMG
Org2_Gene620	accessory	4192	[Rbromii5AMG]RB5AMG_00646_hypothetical_protein	Rbromii5AMG
Org2_Gene623	accessory	4437	[Rbromii5AMG]RB5AMG_00649_hypothetical_protein	Rbromii5AMG
Org2_Gene624	accessory	564	[Rbromii5AMG]RB5AMG_00650_hypothetical_protein	Rbromii5AMG
Org2_Gene626	accessory	3365	[Rbromii5AMG]RB5AMG_00652_oxaloacetate_decarboxylase_subunit_gamma	Rbromii5AMG
Org2_Gene628	accessory	2257	[Rbromii5AMG]RB5AMG_00654_polysaccharide_export_protein,_MPA1_family	Rbromii5AMG
Org2_Gene629	accessory	2289	[Rbromii5AMG]RB5AMG_00655_Tyrosine-protein_kinase_YwqD	Rbromii5AMG
Org2_Gene633	accessory	1456	[Rbromii5AMG]RB5AMG_00659_putative_zinc_transport_system_zinc-binding_lipoprotein_AdcA_precursor	Rbromii5AMG
Org2_Gene634	accessory	2203	[Rbromii5AMG]RB5AMG_00660_putative_zinc_transport_system_ATP-binding_protein_AdcC	Rbromii5AMG
Org2_Gene635	accessory	1915	[Rbromii5AMG]RB5AMG_00661_High-affinity_zinc_uptake_system_membrane_protein_ZnuB	Rbromii5AMG
Org2_Gene638	accessory	3524	[Rbromii5AMG]RB5AMG_00664_hypothetical_protein	Rbromii5AMG
Org2_Gene639	accessory	2312	[Rbromii5AMG]RB5AMG_00665_hypothetical_protein	Rbromii5AMG
Org2_Gene641	accessory	1290	[Rbromii5AMG]RB5AMG_00667_spore_germination_protein_(amino_acid_permease)	Rbromii5AMG
Org2_Gene642	accessory	2738	[Rbromii5AMG]RB5AMG_00668_germination_protein,_Ger(x)C_family	Rbromii5AMG
Org2_Gene647	accessory	4117	[Rbromii5AMG]RB5AMG_00673_exodeoxyribonuclease_VII_small_subunit	Rbromii5AMG
Org2_Gene651	accessory	1849	[Rbromii5AMG]RB5AMG_00677_putative_inorganic_polyphosphate/ATP-NAD_kinase	Rbromii5AMG
Org2_Gene652	accessory	3272	[Rbromii5AMG]RB5AMG_00678_Arginine_hydroxamate_resistance_protein	Rbromii5AMG
Org2_Gene654	accessory	267	[Rbromii5AMG]RB5AMG_00680_Single-stranded-DNA-specific_exonuclease_RecJ	Rbromii5AMG
Org2_Gene659	accessory	3434	[Rbromii5AMG]RB5AMG_00685_hypothetical_protein	Rbromii5AMG
Org2_Gene660	accessory	345	[Rbromii5AMG]RB5AMG_00686_Stage_III_sporulation_protein_F	Rbromii5AMG
Org2_Gene665	accessory	3277	[Rbromii5AMG]RB5AMG_00691_Septum_formation_initiator	Rbromii5AMG
Org2_Gene669	accessory	1561	[Rbromii5AMG]RB5AMG_00695_hypothetical_protein	Rbromii5AMG
Org2_Gene672	accessory	3034	[Rbromii5AMG]RB5AMG_00698_preprotein_translocase_subunit_SecD	Rbromii5AMG
Org2_Gene673	accessory	1549	[Rbromii5AMG]RB5AMG_00699_stage_III_sporulation_protein_AA	Rbromii5AMG
Org2_Gene674	accessory	3075	[Rbromii5AMG]RB5AMG_00700_stage_III_sporulation_protein_SpoAB	Rbromii5AMG
Org2_Gene676	accessory	3501	[Rbromii5AMG]RB5AMG_00702_stage_III_sporulation_protein_AD	Rbromii5AMG
Org2_Gene677	accessory	1199	[Rbromii5AMG]RB5AMG_00703_stage_III_sporulation_protein_AE	Rbromii5AMG
Org2_Gene678	accessory	3177	[Rbromii5AMG]RB5AMG_00704_hypothetical_protein	Rbromii5AMG
Org2_Gene68	accessory	208	[Rbromii5AMG]RB5AMG_00070_Stage_V_sporulation_protein_T	Rbromii5AMG
Org2_Gene680	accessory	2903	[Rbromii5AMG]RB5AMG_00706_SpoIIIAH-like_protein	Rbromii5AMG
Org2_Gene681	accessory	3081	[Rbromii5AMG]RB5AMG_00707_hypothetical_protein	Rbromii5AMG
Org2_Gene683	accessory	995	[Rbromii5AMG]RB5AMG_00709_Exodeoxyribonuclease_7_large_subunit	Rbromii5AMG
Org2_Gene685	accessory	1421	[Rbromii5AMG]RB5AMG_00711_ABC-type_uncharacterized_transport_system,_periplasmic_component	Rbromii5AMG
Org2_Gene69	accessory	3063	[Rbromii5AMG]RB5AMG_00071_nicotinamidase/pyrazinamidase	Rbromii5AMG
Org2_Gene690	accessory	1704	[Rbromii5AMG]RB5AMG_00716_hypothetical_protein	Rbromii5AMG
Org2_Gene691	accessory	2323	[Rbromii5AMG]RB5AMG_00717_SynChlorMet_cassette_protein_ScmC	Rbromii5AMG
Org2_Gene693	accessory	1087	[Rbromii5AMG]RB5AMG_00719_CYTH_domain_protein	Rbromii5AMG
Org2_Gene694	accessory	392	[Rbromii5AMG]RB5AMG_00720_1-deoxy-D-xylulose-5-phosphate_synthase	Rbromii5AMG
Org2_Gene696	accessory	3755	[Rbromii5AMG]RB5AMG_00722_hypothetical_protein	Rbromii5AMG
Org2_Gene697	accessory	3278	[Rbromii5AMG]RB5AMG_00723_hypothetical_protein	Rbromii5AMG
Org2_Gene7	accessory	424	[Rbromii5AMG]RB5AMG_00007_hypothetical_protein	Rbromii5AMG
Org2_Gene70	accessory	2950	[Rbromii5AMG]RB5AMG_00072_2,3-bisphosphoglycerate-dependent_phosphoglycerate_mutase	Rbromii5AMG
Org2_Gene701	accessory	2707	[Rbromii5AMG]RB5AMG_00727_ATP_synthase_(E/31_kDa)_subunit	Rbromii5AMG
Org2_Gene704	accessory	305	[Rbromii5AMG]RB5AMG_00730_V-type_ATP_synthase_subunit_I	Rbromii5AMG
Org2_Gene706	accessory	3794	[Rbromii5AMG]RB5AMG_00732_hypothetical_protein	Rbromii5AMG
Org2_Gene709	accessory	1969	[Rbromii5AMG]RB5AMG_00735_Histidinol-phosphatase	Rbromii5AMG
Org2_Gene71	accessory	788	[Rbromii5AMG]RB5AMG_00073_Ktr_system_potassium_uptake_protein_B	Rbromii5AMG
Org2_Gene711	accessory	1338	[Rbromii5AMG]RB5AMG_00737_hypothetical_protein	Rbromii5AMG
Org2_Gene714	accessory	4008	[Rbromii5AMG]RB5AMG_00740_TSCPD_domain_protein	Rbromii5AMG
Org2_Gene715	accessory	1231	[Rbromii5AMG]RB5AMG_00741_DnaD_domain_protein	Rbromii5AMG
Org2_Gene716	accessory	1458	[Rbromii5AMG]RB5AMG_00742_DNA_replication_protein_DnaC	Rbromii5AMG

Org2_Gene717	accessory	3200	[Rbromii5AMG]RB5AMG_00745_hypothetical_protein	Rbromii5AMG
Org2_Gene719	accessory	1008	[Rbromii5AMG]RB5AMG_00747_Putative_penicillin-binding_protein_PbpX	Rbromii5AMG
Org2_Gene72	accessory	2553	[Rbromii5AMG]RB5AMG_00074_Ktr_system_potassium_uptake_protein_A	Rbromii5AMG
Org2_Gene720	accessory	1814	[Rbromii5AMG]RB5AMG_00748_hypothetical_protein	Rbromii5AMG
Org2_Gene721	accessory	739	[Rbromii5AMG]RB5AMG_00749_HTH-type_transcriptional_regulatory_protein_GabR	Rbromii5AMG
Org2_Gene723	accessory	1370	[Rbromii5AMG]RB5AMG_00751_Putative_aminopeptidase_YsdC	Rbromii5AMG
Org2_Gene724	accessory	1322	[Rbromii5AMG]RB5AMG_00752_Putative_aminopeptidase_YsdC	Rbromii5AMG
Org2_Gene725	accessory	2123	[Rbromii5AMG]RB5AMG_00753_ribosomal-protein-alanine_acetyltransferase	Rbromii5AMG
Org2_Gene728	accessory	1309	[Rbromii5AMG]RB5AMG_00756_CAAX_amino_terminal_protease_self-_immunity	Rbromii5AMG
Org2_Gene729	accessory	3053	[Rbromii5AMG]RB5AMG_00757_hypothetical_protein	Rbromii5AMG
Org2_Gene73	accessory	4010	[Rbromii5AMG]RB5AMG_00075_hypothetical_protein	Rbromii5AMG
Org2_Gene736	accessory	4213	[Rbromii5AMG]RB5AMG_00764_Recombination_directionality_factor_xis	Rbromii5AMG
Org2_Gene737	accessory	1244	[Rbromii5AMG]RB5AMG_00765_Putative_prophage_phiRv2_integrase	Rbromii5AMG
Org2_Gene738	accessory	3695	[Rbromii5AMG]RB5AMG_00766_hypothetical_protein	Rbromii5AMG
Org2_Gene739	accessory	1040	[Rbromii5AMG]RB5AMG_00767_Replicative_DNA_helicase	Rbromii5AMG
Org2_Gene74	accessory	286	[Rbromii5AMG]RB5AMG_00076_Sensor_protein_KdpD	Rbromii5AMG
Org2_Gene744	accessory	3189	[Rbromii5AMG]RB5AMG_00772_Helix-turn-helix_domain_protein	Rbromii5AMG
Org2_Gene745	accessory	4232	[Rbromii5AMG]RB5AMG_00773_hypothetical_protein	Rbromii5AMG
Org2_Gene746	accessory	1264	[Rbromii5AMG]RB5AMG_00774_Putative_prophage_phiRv2_integrase	Rbromii5AMG
Org2_Gene75	accessory	2378	[Rbromii5AMG]RB5AMG_00077_KDP_operon_transcriptional_regulatory_protein_KdpE	Rbromii5AMG
Org2_Gene758	accessory	96	[Rbromii5AMG]RB5AMG_00786_Acyltransferase_family_protein	Rbromii5AMG
Org2_Gene759	accessory	507	[Rbromii5AMG]RB5AMG_00787_putative_integral_membrane_protein	Rbromii5AMG
Org2_Gene762	accessory	467	[Rbromii5AMG]RB5AMG_00790_hypothetical_protein	Rbromii5AMG
Org2_Gene763	accessory	2074	[Rbromii5AMG]RB5AMG_00791_Small-conductance_mechanosensitive_channel	Rbromii5AMG
Org2_Gene764	accessory	3756	[Rbromii5AMG]RB5AMG_00792_hypothetical_protein	Rbromii5AMG
Org2_Gene767	accessory	2030	[Rbromii5AMG]RB5AMG_00795_sigma-E_processing_peptidase_SpoIIGA	Rbromii5AMG
Org2_Gene770	accessory	1497	[Rbromii5AMG]RB5AMG_00798_hypothetical_protein	Rbromii5AMG
Org2_Gene774	accessory	2729	[Rbromii5AMG]RB5AMG_00803_Translation_initiation_factor_IF-3	Rbromii5AMG
Org2_Gene777	accessory	2102	[Rbromii5AMG]RB5AMG_00806_23S_rRNA_(uridine(2479)-2'-O)-methyltransferase	Rbromii5AMG
Org2_Gene778	accessory	927	[Rbromii5AMG]RB5AMG_00807_DNA_protecting_protein_DprA	Rbromii5AMG
Org2_Gene783	accessory	1574	[Rbromii5AMG]RB5AMG_00812_Oxygen-independent_coproporphyrinogen-III_oxidase_1	Rbromii5AMG
Org2_Gene788	accessory	2681	[Rbromii5AMG]RB5AMG_00817_putative_nicotinate-nucleotide_adenylyltransferase	Rbromii5AMG
Org2_Gene789	accessory	2743	[Rbromii5AMG]RB5AMG_00818_putative_nicotinate-nucleotide_adenylyltransferase	Rbromii5AMG
Org2_Gene794	accessory	1242	[Rbromii5AMG]RB5AMG_00823_putative_peptidase	Rbromii5AMG
Org2_Gene796	accessory	3110	[Rbromii5AMG]RB5AMG_00825_hypothetical_protein	Rbromii5AMG
Org2_Gene797	accessory	2532	[Rbromii5AMG]RB5AMG_00826_Sporulation_protein_YunB	Rbromii5AMG
Org2_Gene80	accessory	2039	[Rbromii5AMG]RB5AMG_00086_General_stress_protein_39	Rbromii5AMG
Org2_Gene801	accessory	229	[Rbromii5AMG]RB5AMG_00830_DNA_mismatch_repair_protein_MutL	Rbromii5AMG
Org2_Gene802	accessory	1550	[Rbromii5AMG]RB5AMG_00831_tRNA_dimethylallyltransferase	Rbromii5AMG
Org2_Gene803	accessory	877	[Rbromii5AMG]RB5AMG_00832_putative_membrane_fusion_protein	Rbromii5AMG
Org2_Gene806	accessory	2147	[Rbromii5AMG]RB5AMG_00835_photosystem_II_S4_domain_protein	Rbromii5AMG
Org2_Gene807	accessory	2446	[Rbromii5AMG]RB5AMG_00836_Minicell-associated_protein_DivIVA	Rbromii5AMG
Org2_Gene81	accessory	4345	[Rbromii5AMG]RB5AMG_00087_hypothetical_protein	Rbromii5AMG
Org2_Gene812	accessory	2568	[Rbromii5AMG]RB5AMG_00841_Signal_peptidase_I_P	Rbromii5AMG
Org2_Gene813	accessory	2785	[Rbromii5AMG]RB5AMG_00842_Signal_peptidase_I_T	Rbromii5AMG
Org2_Gene816	accessory	3585	[Rbromii5AMG]RB5AMG_00845_hypothetical_protein	Rbromii5AMG
Org2_Gene817	accessory	1578	[Rbromii5AMG]RB5AMG_00846_Membrane_dipeptidase_(Peptidase_family_M19)	Rbromii5AMG
Org2_Gene82	accessory	1777	[Rbromii5AMG]RB5AMG_00088_Dipicolinate_synthase_subunit_A	Rbromii5AMG
Org2_Gene827	accessory	4178	[Rbromii5AMG]RB5AMG_00856_HTH-type_transcriptional_regulator_ImmR	Rbromii5AMG
Org2_Gene830	accessory	2396	[Rbromii5AMG]RB5AMG_00859_hypothetical_protein	Rbromii5AMG
Org2_Gene832	accessory	2333	[Rbromii5AMG]RB5AMG_00861_N-acetylmuramoyl-L-alanine_amidase_AmiC_precursor	Rbromii5AMG
Org2_Gene834	accessory	679	[Rbromii5AMG]RB5AMG_00863_Amidophosphoribosyltransferase_precursor	Rbromii5AMG
Org2_Gene835	accessory	114	[Rbromii5AMG]RB5AMG_00864_Putative_HMP/thiamine_import_ATP-binding_protein_YkoD	Rbromii5AMG
Org2_Gene836	accessory	1732	[Rbromii5AMG]RB5AMG_00865_Energy-coupling_factor_transporter_transmembrane_protein_EcfT	Rbromii5AMG
Org2_Gene837	accessory	1565	[Rbromii5AMG]RB5AMG_00866_hypothetical_protein	Rbromii5AMG
Org2_Gene838	accessory	326	[Rbromii5AMG]RB5AMG_00867_hypothetical_protein	Rbromii5AMG
Org2_Gene839	accessory	4072	[Rbromii5AMG]RB5AMG_00868_hypothetical_protein	Rbromii5AMG
Org2_Gene84	accessory	4177	[Rbromii5AMG]RB5AMG_00090_putative_zinc_finger/helix-turn-helix_protein,_YgiT_family	Rbromii5AMG
Org2_Gene841	accessory	2009	[Rbromii5AMG]RB5AMG_00870_ribonuclease_Z	Rbromii5AMG
Org2_Gene844	accessory	686	[Rbromii5AMG]RB5AMG_00873_Arginine_decarboxylase	Rbromii5AMG
Org2_Gene846	accessory	1229	[Rbromii5AMG]RB5AMG_00875_Oxygen-independent_coproporphyrinogen-III_oxidase_1	Rbromii5AMG
Org2_Gene847	accessory	1690	[Rbromii5AMG]RB5AMG_00876_hypothetical_protein	Rbromii5AMG
Org2_Gene848	accessory	2343	[Rbromii5AMG]RB5AMG_00877_hypothetical_protein	Rbromii5AMG
Org2_Gene849	accessory	3307	[Rbromii5AMG]RB5AMG_00878_Cell_wall_hydrolase_CwlJ	Rbromii5AMG
Org2_Gene85	accessory	4113	[Rbromii5AMG]RB5AMG_00091_HTH-type_transcriptional_regulator_ImmR	Rbromii5AMG
Org2_Gene857	accessory	655	[Rbromii5AMG]RB5AMG_00886_Divergent_AAA_domain_protein	Rbromii5AMG
Org2_Gene858	accessory	410	[Rbromii5AMG]RB5AMG_00887_putative_ABC_transporter_ATP-binding_protein	Rbromii5AMG
Org2_Gene859	accessory	377	[Rbromii5AMG]RB5AMG_00888_Iron_import_ATP-binding/permease_protein_IrtA	Rbromii5AMG
Org2_Gene860	accessory	2626	[Rbromii5AMG]RB5AMG_00889_DNA-binding_transcriptional_repressor_AcrR	Rbromii5AMG
Org2_Gene861	accessory	3319	[Rbromii5AMG]RB5AMG_00890_ribosomal-protein-alanine_acetyltransferase	Rbromii5AMG
Org2_Gene864	accessory	2337	[Rbromii5AMG]RB5AMG_00893_t(6)A37_threonylcarbamoyladenosine_biosynthesis_protein	Rbromii5AMG
Org2_Gene867	accessory	1095	[Rbromii5AMG]RB5AMG_00896_Cell_division_protein_FtsW	Rbromii5AMG
Org2_Gene868	accessory	925	[Rbromii5AMG]RB5AMG_00897_YbbR-like_protein	Rbromii5AMG
Org2_Gene87	accessory	2966	[Rbromii5AMG]RB5AMG_00093_hypothetical_protein	Rbromii5AMG
Org2_Gene870	accessory	1106	[Rbromii5AMG]RB5AMG_00899_putative_cysteine_desulfurase	Rbromii5AMG
Org2_Gene871	accessory	4103	[Rbromii5AMG]RB5AMG_00900_hypothetical_protein	Rbromii5AMG
Org2_Gene877	accessory	3360	[Rbromii5AMG]RB5AMG_00906_preprotein_translocase_subunit_YajC	Rbromii5AMG
Org2_Gene878	accessory	3480	[Rbromii5AMG]RB5AMG_00907_putative_membrane_protein	Rbromii5AMG
Org2_Gene879	accessory	150	[Rbromii5AMG]RB5AMG_00908_Serine/threonine-protein_kinase_PrkC	Rbromii5AMG
Org2_Gene880	accessory	414	[Rbromii5AMG]RB5AMG_00909_hypothetical_protein	Rbromii5AMG
Org2_Gene882	accessory	4007	[Rbromii5AMG]RB5AMG_00911_hypothetical_protein	Rbromii5AMG
Org2_Gene883	accessory	301	[Rbromii5AMG]RB5AMG_00912_Bacterial_Ig-like_domain_(group_2)	Rbromii5AMG
Org2_Gene893	accessory	500	[Rbromii5AMG]RB5AMG_00922_Germination-specific_amidase	Rbromii5AMG
Org2_Gene896	accessory	2061	[Rbromii5AMG]RB5AMG_00925_hypothetical_protein	Rbromii5AMG
Org2_Gene897	accessory	1169	[Rbromii5AMG]RB5AMG_00926_hypothetical_protein	Rbromii5AMG
Org2_Gene900	accessory	3522	[Rbromii5AMG]RB5AMG_00929_hypothetical_protein	Rbromii5AMG
Org2_Gene901	accessory	1584	[Rbromii5AMG]RB5AMG_00930_auxin_efflux_carrier	Rbromii5AMG
Org2_Gene905	accessory	2742	[Rbromii5AMG]RB5AMG_00934_Plasmid_pRiA4b_ORF-3-like_protein	Rbromii5AMG
Org2_Gene907	accessory	3347	[Rbromii5AMG]RB5AMG_00936_hypothetical_protein	Rbromii5AMG
Org2_Gene908	accessory	2366	[Rbromii5AMG]RB5AMG_00937_Serine/threonine-protein_kinase_PknB	Rbromii5AMG
Org2_Gene909	accessory	3688	[Rbromii5AMG]RB5AMG_00938_HTH-type_transcriptional_regulator_ImmR	Rbromii5AMG
Org2_Gene910	accessory	3435	[Rbromii5AMG]RB5AMG_00939_hypothetical_protein	Rbromii5AMG
Org2_Gene911	accessory	2970	[Rbromii5AMG]RB5AMG_00940_putative_membrane_protein	Rbromii5AMG
Org2_Gene912	accessory	2994	[Rbromii5AMG]RB5AMG_00941_hypothetical_protein	Rbromii5AMG
Org2_Gene913	accessory	826	[Rbromii5AMG]RB5AMG_00942_putative_membrane_protein	Rbromii5AMG
Org2_Gene914	accessory	1312	[Rbromii5AMG]RB5AMG_00943_hypothetical_protein	Rbromii5AMG
Org2_Gene915	accessory	950	[Rbromii5AMG]RB5AMG_00945_Fucose_4-O-acetylase	Rbromii5AMG
Org2_Gene916	accessory	2299	[Rbromii5AMG]RB5AMG_00946_SynChlorMet_cassette_protein_ScmC	Rbromii5AMG
Org2_Gene918	accessory	1931	[Rbromii5AMG]RB5AMG_00948_transcriptional_activator_RhaS	Rbromii5AMG
Org2_Gene919	accessory	1882	[Rbromii5AMG]RB5AMG_00949_putative_metallophosphoesterase	Rbromii5AMG
Org2_Gene922	accessory	285	[Rbromii5AMG]RB5AMG_00952_hypothetical_protein	Rbromii5AMG
Org2_Gene928	accessory	740	[Rbromii5AMG]RB5AMG_00958_Staphylococcal_virulence_regulator_protein_A	Rbromii5AMG
Org2_Gene929	accessory	2552	[Rbromii5AMG]RB5AMG_00959_Phosphorylated_carbohydrates_phosphatase	Rbromii5AMG
Org2_Gene930	accessory	370	[Rbromii5AMG]RB5AMG_00960_hypothetical_protein	Rbromii5AMG
Org2_Gene931	accessory	623	[Rbromii5AMG]RB5AMG_00961_Multidrug_export_protein_MepA	Rbromii5AMG
Org2_Gene932	accessory	1024	[Rbromii5AMG]RB5AMG_00962_putative_integral_membrane_protein	Rbromii5AMG
Org2_Gene935	accessory	855	[Rbromii5AMG]RB5AMG_00965_hypothetical_protein	Rbromii5AMG
Org2_Gene937	accessory	2765	[Rbromii5AMG]RB5AMG_00967_hypothetical_protein	Rbromii5AMG
Org2_Gene939	accessory	113	[Rbromii5AMG]RB5AMG_00969_copper_exporting_ATPase	Rbromii5AMG
Org2_Gene94	accessory	2261	[Rbromii5AMG]RB5AMG_00100_Stage_II_sporulation_protein_Q	Rbromii5AMG
Org2_Gene944	accessory	897	[Rbromii5AMG]RB5AMG_00975_corrinoid_ABC_transporter_substrate-binding_protein	Rbromii5AMG
Org2_Gene948	accessory	1310	[Rbromii5AMG]RB5AMG_00979_hypothetical_protein	Rbromii5AMG
Org2_Gene949	accessory	2344	[Rbromii5AMG]RB5AMG_00980_transcriptional_regulator_FixK	Rbromii5AMG
Org2_Gene95	accessory	2592	[Rbromii5AMG]RB5AMG_00101_cytidylate_kinase	Rbromii5AMG
Org2_Gene951	accessory	1545	[Rbromii5AMG]RB5AMG_00982_L-lactate_dehydrogenase_P	Rbromii5AMG
Org2_Gene954	accessory	651	[Rbromii5AMG]RB5AMG_00985_Trk_system_potassium_uptake_protein_TrkA	Rbromii5AMG
Org2_Gene956	accessory	1233	[Rbromii5AMG]RB5AMG_00987_stage_II_sporulation_protein_P	Rbromii5AMG
Org2_Gene958	accessory	462	[Rbromii5AMG]RB5AMG_00989_Papain_family_cysteine_protease	Rbromii5AMG
Org2_Gene960	accessory	468	[Rbromii5AMG]RB5AMG_00991_Alpha-amylase_precursor	Rbromii5AMG
Org2_Gene962	accessory	3195	[Rbromii5AMG]RB5AMG_00993_Acetyltransferase_(GNAT)_family_protein	Rbromii5AMG
Org2_Gene964	accessory	3803	[Rbromii5AMG]RB5AMG_00995_Thioredoxin-M	Rbromii5AMG
Org2_Gene965	accessory	3187	[Rbromii5AMG]RB5AMG_00996_Terminase_small_subunit	Rbromii5AMG

Org2_Gene967	accessory	1126	[Rbromii5AMG]RB5AMG_00998_phage_portal_protein,_HK97_family	Rbromii5AMG
Org2_Gene968	accessory	1431	[Rbromii5AMG]RB5AMG_00999_hypothetical_protein	Rbromii5AMG
Org2_Gene969	accessory	3684	[Rbromii5AMG]RB5AMG_01000_hypothetical_protein	Rbromii5AMG
Org2_Gene97	accessory	584	[Rbromii5AMG]RB5AMG_00103_Cell_wall-associated_polypeptide_CWBP200	Rbromii5AMG
Org2_Gene971	accessory	3620	[Rbromii5AMG]RB5AMG_01002_hypothetical_protein	Rbromii5AMG
Org2_Gene972	accessory	3603	[Rbromii5AMG]RB5AMG_01003_hypothetical_protein	Rbromii5AMG
Org2_Gene973	accessory	3144	[Rbromii5AMG]RB5AMG_01004_hypothetical_protein	Rbromii5AMG
Org2_Gene974	accessory	3579	[Rbromii5AMG]RB5AMG_01005_hypothetical_protein	Rbromii5AMG
Org2_Gene975	accessory	4389	[Rbromii5AMG]RB5AMG_01006_hypothetical_protein	Rbromii5AMG
Org2_Gene976	accessory	3009	[Rbromii5AMG]RB5AMG_01007_spore_coat_assembly_protein_SafA	Rbromii5AMG
Org2_Gene977	accessory	1534	[Rbromii5AMG]RB5AMG_01008_Mu-like_prophage_tail_protein_gpP	Rbromii5AMG
Org2_Gene978	accessory	3726	[Rbromii5AMG]RB5AMG_01009_Mu-like_prophage_protein_gp45	Rbromii5AMG
Org2_Gene979	accessory	3727	[Rbromii5AMG]RB5AMG_01010_hypothetical_protein	Rbromii5AMG
Org2_Gene980	accessory	1302	[Rbromii5AMG]RB5AMG_01011_baseplate_assembly_protein	Rbromii5AMG
Org2_Gene981	accessory	3005	[Rbromii5AMG]RB5AMG_01012_hypothetical_protein	Rbromii5AMG
Org2_Gene986	accessory	889	[Rbromii5AMG]RB5AMG_01017_hypothetical_protein	Rbromii5AMG
Org2_Gene989	accessory	1035	[Rbromii5AMG]RB5AMG_01020_hypothetical_protein	Rbromii5AMG
Org2_Gene99	accessory	4417	[Rbromii5AMG]RB5AMG_00105_hypothetical_protein	Rbromii5AMG
Org2_Gene990	accessory	1752	[Rbromii5AMG]RB5AMG_01021_hypothetical_protein	Rbromii5AMG
Org2_Gene995	accessory	2392	[Rbromii5AMG]RB5AMG_01026_putative_flavoprotein	Rbromii5AMG
Org2_Gene996	accessory	879	[Rbromii5AMG]RB5AMG_01027_Alpha-1,4-glucan:maltose-1-phosphate_maltosyltransferase_1	Rbromii5AMG
Org2_Gene998	accessory	634	[Rbromii5AMG]RB5AMG_01029_Staphylococcal_virulence_regulator_protein_A	Rbromii5AMG
Org2_Gene999	accessory	3490	[Rbromii5AMG]RB5AMG_01030_hypothetical_protein	Rbromii5AMG
Org3_Gene100	accessory	4300	[RbromiiATCC27255]RBATCC27255_00104_hypothetical_protein	RbromiiATCC27255
Org3_Gene1000	accessory	696	[RbromiiATCC27255]RBATCC27255_01028_Multidrug_export_protein_MepA	RbromiiATCC27255
Org3_Gene1001	accessory	2112	[RbromiiATCC27255]RBATCC27255_01029_N-acetylmuramoyl-L-alanine_amidase	RbromiiATCC27255
Org3_Gene1002	accessory	4071	[RbromiiATCC27255]RBATCC27255_01030_hypothetical_protein	RbromiiATCC27255
Org3_Gene1007	accessory	804	[RbromiiATCC27255]RBATCC27255_01035_tRNA(Ile)-lysidine_synthase	RbromiiATCC27255
Org3_Gene101	accessory	3516	[RbromiiATCC27255]RBATCC27255_00105_hypothetical_protein	RbromiiATCC27255
Org3_Gene1010	accessory	294	[RbromiiATCC27255]RBATCC27255_01038_Bifunctional_oligoribonuclease_and_PAP_phosphatase_NrnA	RbromiiATCC27255
Org3_Gene1016	accessory	2242	[RbromiiATCC27255]RBATCC27255_01044_Tyrosine-protein_phosphatase_CpsB	RbromiiATCC27255
Org3_Gene1019	accessory	2478	[RbromiiATCC27255]RBATCC27255_01047_Putative_PBSX_repressor	RbromiiATCC27255
Org3_Gene102	accessory	3762	[RbromiiATCC27255]RBATCC27255_00106_hypothetical_protein	RbromiiATCC27255
Org3_Gene1021	accessory	2988	[RbromiiATCC27255]RBATCC27255_01049_hypothetical_protein	RbromiiATCC27255
Org3_Gene1022	accessory	3437	[RbromiiATCC27255]RBATCC27255_01050_hypothetical_protein	RbromiiATCC27255
Org3_Gene1023	accessory	2787	[RbromiiATCC27255]RBATCC27255_01051_putative_nicotinate-nucleotide_adenylyltransferase	RbromiiATCC27255
Org3_Gene103	accessory	3577	[RbromiiATCC27255]RBATCC27255_00107_hypothetical_protein	RbromiiATCC27255
Org3_Gene1030	accessory	3492	[RbromiiATCC27255]RBATCC27255_01058_Putative_PBSX_repressor	RbromiiATCC27255
Org3_Gene1037	accessory	1283	[RbromiiATCC27255]RBATCC27255_01066_Modification_methylase_DpnIIA	RbromiiATCC27255
Org3_Gene1038	accessory	3525	[RbromiiATCC27255]RBATCC27255_01067_hypothetical_protein	RbromiiATCC27255
Org3_Gene1039	accessory	1116	[RbromiiATCC27255]RBATCC27255_01068_Plasmid_recombination_enzyme	RbromiiATCC27255
Org3_Gene104	accessory	4291	[RbromiiATCC27255]RBATCC27255_00108_hypothetical_protein	RbromiiATCC27255
Org3_Gene105	accessory	526	[RbromiiATCC27255]RBATCC27255_00109_Murein_hydrolase_activator_NlpD_precursor	RbromiiATCC27255
Org3_Gene1052	accessory	4179	[RbromiiATCC27255]RBATCC27255_01082_hypothetical_protein	RbromiiATCC27255
Org3_Gene1055	accessory	1928	[RbromiiATCC27255]RBATCC27255_01085_putative_phosphatase_YwpJ	RbromiiATCC27255
Org3_Gene1056	accessory	2556	[RbromiiATCC27255]RBATCC27255_01086_hypothetical_protein	RbromiiATCC27255
Org3_Gene1058	accessory	406	[RbromiiATCC27255]RBATCC27255_01088_Serine_protease_Do-like_HtrA	RbromiiATCC27255
Org3_Gene1060	accessory	2881	[RbromiiATCC27255]RBATCC27255_01090_Biotin_ECF_transporter_S_component_BioY	RbromiiATCC27255
Org3_Gene1061	accessory	3590	[RbromiiATCC27255]RBATCC27255_01091_hypothetical_protein	RbromiiATCC27255
Org3_Gene1062	accessory	2338	[RbromiiATCC27255]RBATCC27255_01096_hypothetical_protein	RbromiiATCC27255
Org3_Gene1065	accessory	760	[RbromiiATCC27255]RBATCC27255_01100_hypothetical_protein	RbromiiATCC27255
Org3_Gene1066	accessory	1770	[RbromiiATCC27255]RBATCC27255_01101_putative_ABC_transporter_ATP-binding_protein_YxlF	RbromiiATCC27255
Org3_Gene1069	accessory	190	[RbromiiATCC27255]RBATCC27255_01104_ABC-type_transport_system_involved_in_multi-copper_enzyme_maturation,_permease_component	RbromiiATCC27255
Org3_Gene107	accessory	1670	[RbromiiATCC27255]RBATCC27255_00111_Autolytic_lysozyme	RbromiiATCC27255
Org3_Gene1070	accessory	921	[RbromiiATCC27255]RBATCC27255_01105_hypothetical_protein	RbromiiATCC27255
Org3_Gene1071	accessory	3270	[RbromiiATCC27255]RBATCC27255_01106_hypothetical_protein	RbromiiATCC27255
Org3_Gene1072	accessory	1114	[RbromiiATCC27255]RBATCC27255_01107_Fucose_4-O-acetylase	RbromiiATCC27255
Org3_Gene1076	accessory	888	[RbromiiATCC27255]RBATCC27255_01111_Galactokinase	RbromiiATCC27255
Org3_Gene1077	accessory	604	[RbromiiATCC27255]RBATCC27255_01112_Thiomethylgalactoside_permease_II	RbromiiATCC27255
Org3_Gene1078	accessory	1395	[RbromiiATCC27255]RBATCC27255_01113_Glucose-resistance_amylase_regulator	RbromiiATCC27255
Org3_Gene108	accessory	3701	[RbromiiATCC27255]RBATCC27255_00112_hypothetical_protein	RbromiiATCC27255
Org3_Gene109	accessory	588	[RbromiiATCC27255]RBATCC27255_00113_Phage-related_lysozyme_(muraminidase)	RbromiiATCC27255
Org3_Gene1091	accessory	861	[RbromiiATCC27255]RBATCC27255_01126_hypothetical_protein	RbromiiATCC27255
Org3_Gene1096	accessory	1244	[RbromiiATCC27255]RBATCC27255_01131_Putative_prophage_phiRv2_integrase	RbromiiATCC27255
Org3_Gene1099	accessory	3165	[RbromiiATCC27255]RBATCC27255_01134_flavodoxin	RbromiiATCC27255
Org3_Gene11	accessory	500	[RbromiiATCC27255]RBATCC27255_00013_Germination-specific_amidase	RbromiiATCC27255
Org3_Gene110	accessory	2135	[RbromiiATCC27255]RBATCC27255_00114_hypothetical_protein	RbromiiATCC27255
Org3_Gene1101	accessory	3036	[RbromiiATCC27255]RBATCC27255_01136_flavodoxin	RbromiiATCC27255
Org3_Gene1103	accessory	759	[RbromiiATCC27255]RBATCC27255_01138_D-alanyl-D-alanine_carboxypeptidase_DacB_precursor	RbromiiATCC27255
Org3_Gene1107	accessory	1077	[RbromiiATCC27255]RBATCC27255_01142_sporulation_protein_YqfD	RbromiiATCC27255
Org3_Gene1108	accessory	3933	[RbromiiATCC27255]RBATCC27255_01143_sporulation_protein_YqfC	RbromiiATCC27255
Org3_Gene1109	accessory	1279	[RbromiiATCC27255]RBATCC27255_01144_Sporulation_integral_membrane_protein_YlbJ	RbromiiATCC27255
Org3_Gene1114	accessory	1906	[RbromiiATCC27255]RBATCC27255_01149_ComEC_family_competence_protein	RbromiiATCC27255
Org3_Gene1116	accessory	104	[RbromiiATCC27255]RBATCC27255_01151_Stage_III_sporulation_protein_E	RbromiiATCC27255
Org3_Gene112	accessory	3213	[RbromiiATCC27255]RBATCC27255_00116_GtrA-like_protein	RbromiiATCC27255
Org3_Gene1120	accessory	3481	[RbromiiATCC27255]RBATCC27255_01155_putative_membrane_protein	RbromiiATCC27255
Org3_Gene1122	accessory	1334	[RbromiiATCC27255]RBATCC27255_01157_DNA_polymerase_III_subunit_delta	RbromiiATCC27255
Org3_Gene1123	accessory	204	[RbromiiATCC27255]RBATCC27255_01158_DNA_internalization-related_competence_protein_ComEC/Rec2	RbromiiATCC27255
Org3_Gene1124	accessory	2974	[RbromiiATCC27255]RBATCC27255_01159_hypothetical_protein	RbromiiATCC27255
Org3_Gene1127	accessory	2279	[RbromiiATCC27255]RBATCC27255_01162_G3P_acyltransferase	RbromiiATCC27255
Org3_Gene1129	accessory	848	[RbromiiATCC27255]RBATCC27255_01164_putative_FeS-containing_Cyanobacterial-specific_oxidoreductase	RbromiiATCC27255
Org3_Gene1132	accessory	2390	[RbromiiATCC27255]RBATCC27255_01167_hypothetical_protein	RbromiiATCC27255
Org3_Gene1133	accessory	2328	[RbromiiATCC27255]RBATCC27255_01168_hypothetical_protein	RbromiiATCC27255
Org3_Gene1134	accessory	3526	[RbromiiATCC27255]RBATCC27255_01169_Penicillinase_repressor	RbromiiATCC27255
Org3_Gene1135	accessory	1033	[RbromiiATCC27255]RBATCC27255_01170_Regulatory_protein_BlaR1	RbromiiATCC27255
Org3_Gene1136	accessory	3816	[RbromiiATCC27255]RBATCC27255_01171_hypothetical_protein	RbromiiATCC27255
Org3_Gene1137	accessory	1092	[RbromiiATCC27255]RBATCC27255_01172_RmuC_family_protein	RbromiiATCC27255
Org3_Gene1139	accessory	1394	[RbromiiATCC27255]RBATCC27255_01174_Putative_bifunctional_phosphatase/peptidyl-prolyl_cis-trans_isomerase	RbromiiATCC27255
Org3_Gene1141	accessory	1616	[RbromiiATCC27255]RBATCC27255_01176_regulatory_ATPase_RavA	RbromiiATCC27255
Org3_Gene1142	accessory	1511	[RbromiiATCC27255]RBATCC27255_01177_hypothetical_protein	RbromiiATCC27255
Org3_Gene1143	accessory	226	[RbromiiATCC27255]RBATCC27255_01178_Protein-glutamine_gamma-glutamyltransferase	RbromiiATCC27255
Org3_Gene1144	accessory	4304	[RbromiiATCC27255]RBATCC27255_01179_hypothetical_protein	RbromiiATCC27255
Org3_Gene1147	accessory	3302	[RbromiiATCC27255]RBATCC27255_01182_Transcriptional_regulatory_protein_YpdB	RbromiiATCC27255
Org3_Gene1148	accessory	3275	[RbromiiATCC27255]RBATCC27255_01183_hypothetical_protein	RbromiiATCC27255
Org3_Gene1150	accessory	1598	[RbromiiATCC27255]RBATCC27255_01185_ABC-2_family_transporter_protein	RbromiiATCC27255
Org3_Gene1151	accessory	1207	[RbromiiATCC27255]RBATCC27255_01186_5-methyltetrahydropteroyltriglutamate--homocysteine_methyltransferase	RbromiiATCC27255
Org3_Gene1152	accessory	1711	[RbromiiATCC27255]RBATCC27255_01187_Cys_regulon_transcriptional_activator	RbromiiATCC27255
Org3_Gene1153	accessory	2985	[RbromiiATCC27255]RBATCC27255_01188_FMN_reductase_[NAD(P)H]	RbromiiATCC27255
Org3_Gene1154	accessory	2909	[RbromiiATCC27255]RBATCC27255_01189_malonic_semialdehyde_reductase	RbromiiATCC27255
Org3_Gene1155	accessory	1553	[RbromiiATCC27255]RBATCC27255_01190_auxin_efflux_carrier	RbromiiATCC27255
Org3_Gene1156	accessory	4303	[RbromiiATCC27255]RBATCC27255_01191_hypothetical_protein	RbromiiATCC27255
Org3_Gene1162	accessory	2287	[RbromiiATCC27255]RBATCC27255_01197_putative_glutamine_amidotransferase	RbromiiATCC27255
Org3_Gene1164	accessory	2496	[RbromiiATCC27255]RBATCC27255_01199_DNA_utilization_protein_GntX	RbromiiATCC27255
Org3_Gene1165	accessory	1392	[RbromiiATCC27255]RBATCC27255_01200_Rod_shape-determining_protein_MreB	RbromiiATCC27255
Org3_Gene1167	accessory	69	[RbromiiATCC27255]RBATCC27255_01202_Alpha-amylase_precursor	RbromiiATCC27255
Org3_Gene117	accessory	1348	[RbromiiATCC27255]RBATCC27255_00121_Flagellar_biosynthetic_protein_FliU	RbromiiATCC27255
Org3_Gene1174	accessory	1239	[RbromiiATCC27255]RBATCC27255_01209_dTDP-glucose_4,6-dehydratase	RbromiiATCC27255
Org3_Gene1175	accessory	337	[RbromiiATCC27255]RBATCC27255_01210_LPS_biosynthesis_protein	RbromiiATCC27255
Org3_Gene1176	accessory	455	[RbromiiATCC27255]RBATCC27255_01212_hypothetical_protein	RbromiiATCC27255
Org3_Gene118	accessory	3792	[RbromiiATCC27255]RBATCC27255_00122_hypothetical_protein	RbromiiATCC27255
Org3_Gene1181	accessory	980	[RbromiiATCC27255]RBATCC27255_01217_Transposase	RbromiiATCC27255
Org3_Gene1183	accessory	424	[RbromiiATCC27255]RBATCC27255_01219_hypothetical_protein	RbromiiATCC27255
Org3_Gene1184	accessory	575	[RbromiiATCC27255]RBATCC27255_01220_hypothetical_protein	RbromiiATCC27255
Org3_Gene1185	accessory	2407	[RbromiiATCC27255]RBATCC27255_01221_3-deoxy-manno-octulosonate_cytidylyltransferase	RbromiiATCC27255
Org3_Gene1186	accessory	503	[RbromiiATCC27255]RBATCC27255_01222_4-hydroxy-2-oxovalerate_aldolase	RbromiiATCC27255
Org3_Gene1187	accessory	247	[RbromiiATCC27255]RBATCC27255_01223_HAD_hydrolase,_family_IA,_variant_1	RbromiiATCC27255
Org3_Gene1188	accessory	518	[RbromiiATCC27255]RBATCC27255_01224_hypothetical_protein	RbromiiATCC27255
Org3_Gene1189	accessory	2528	[RbromiiATCC27255]RBATCC27255_01225_5'-nucleotidase	RbromiiATCC27255
Org3_Gene1193	accessory	1712	[RbromiiATCC27255]RBATCC27255_01229_hypothetical_protein	RbromiiATCC27255
Org3_Gene1207	accessory	3053	[RbromiiATCC27255]RBATCC27255_01243_hypothetical_protein	RbromiiATCC27255
Org3_Gene1209	accessory	2734	[RbromiiATCC27255]RBATCC27255_01246_hypothetical_protein	RbromiiATCC27255
Org3_Gene1210	accessory	51	[RbromiiATCC27255]RBATCC27255_01247_ATP-dependent_helicase/nuclease_subunit_A	RbromiiATCC27255

Org3_Gene1211	accessory	60	[RbromiiATCC27255]RBATCC27255_01248_ATP-dependent_helicase/deoxyribonuclease_subunit_B	RbromiiATCC27255
Org3_Gene1212	accessory	2689	[RbromiiATCC27255]RBATCC27255_01249_stage_II_sporulation_protein_R	RbromiiATCC27255
Org3_Gene1216	accessory	3054	[RbromiiATCC27255]RBATCC27255_01253_hypothetical_protein	RbromiiATCC27255
Org3_Gene1218	accessory	580	[RbromiiATCC27255]RBATCC27255_01255_Lipid_A_core_-_O-antigen_ligase	RbromiiATCC27255
Org3_Gene1220	accessory	3844	[RbromiiATCC27255]RBATCC27255_01257_hypothetical_protein	RbromiiATCC27255
Org3_Gene1222	accessory	438	[RbromiiATCC27255]RBATCC27255_01260_putative_integral_membrane_protein	RbromiiATCC27255
Org3_Gene1223	accessory	1815	[RbromiiATCC27255]RBATCC27255_01261_DegV_domain-containing_protein	RbromiiATCC27255
Org3_Gene1224	accessory	987	[RbromiiATCC27255]RBATCC27255_01262_hypothetical_protein	RbromiiATCC27255
Org3_Gene1225	accessory	3045	[RbromiiATCC27255]RBATCC27255_01263_hypothetical_protein	RbromiiATCC27255
Org3_Gene1230	accessory	2602	[RbromiiATCC27255]RBATCC27255_01269_Thiamine_pyrophosphokinase	RbromiiATCC27255
Org3_Gene1235	accessory	779	[RbromiiATCC27255]RBATCC27255_01274_Ribosomal_RNA_small_subunit_methyltransferase_B	RbromiiATCC27255
Org3_Gene124	accessory	2046	[RbromiiATCC27255]RBATCC27255_00128_Indole-3-glycerol_phosphate_synthase	RbromiiATCC27255
Org3_Gene1241	accessory	2761	[RbromiiATCC27255]RBATCC27255_01280_Guanylate_kinase	RbromiiATCC27255
Org3_Gene125	accessory	1401	[RbromiiATCC27255]RBATCC27255_00129_Anthranilate_phosphoribosyltransferase	RbromiiATCC27255
Org3_Gene1253	accessory	211	[RbromiiATCC27255]RBATCC27255_01292_Endoglucanase_D_precursor	RbromiiATCC27255
Org3_Gene1255	accessory	425	[RbromiiATCC27255]RBATCC27255_01294_Alpha-amylase_precursor	RbromiiATCC27255
Org3_Gene1256	accessory	1295	[RbromiiATCC27255]RBATCC27255_01295_glycosyltransferase	RbromiiATCC27255
Org3_Gene1259	accessory	1421	[RbromiiATCC27255]RBATCC27255_01298_ABC-type_uncharacterized_transport_system,_periplasmic_component	RbromiiATCC27255
Org3_Gene126	accessory	2800	[RbromiiATCC27255]RBATCC27255_00130_Anthranilate_synthase_component_II	RbromiiATCC27255
Org3_Gene1261	accessory	995	[RbromiiATCC27255]RBATCC27255_01300_Exodeoxyribonuclease_7_large_subunit	RbromiiATCC27255
Org3_Gene1263	accessory	3081	[RbromiiATCC27255]RBATCC27255_01302_hypothetical_protein	RbromiiATCC27255
Org3_Gene1264	accessory	328	[RbromiiATCC27255]RBATCC27255_01303_LPS_biosynthesis_protein	RbromiiATCC27255
Org3_Gene1265	accessory	1266	[RbromiiATCC27255]RBATCC27255_01304_dTDP-4-dehydro-6-deoxyglucose_reductase	RbromiiATCC27255
Org3_Gene1266	accessory	2225	[RbromiiATCC27255]RBATCC27255_01305_2-C-methyl-D-erythritol_4-phosphate_cytidylyltransferase_1	RbromiiATCC27255
Org3_Gene1267	accessory	1309	[RbromiiATCC27255]RBATCC27255_01306_CAAX_amino_terminal_protease_self-_immunity	RbromiiATCC27255
Org3_Gene127	accessory	620	[RbromiiATCC27255]RBATCC27255_00131_Anthranilate_synthase_component_1	RbromiiATCC27255
Org3_Gene1270	accessory	2123	[RbromiiATCC27255]RBATCC27255_01309_ribosomal-protein-alanine_acetyltransferase	RbromiiATCC27255
Org3_Gene1271	accessory	1322	[RbromiiATCC27255]RBATCC27255_01310_Putative_aminopeptidase_YsdC	RbromiiATCC27255
Org3_Gene1272	accessory	1370	[RbromiiATCC27255]RBATCC27255_01311_Putative_aminopeptidase_YsdC	RbromiiATCC27255
Org3_Gene1274	accessory	739	[RbromiiATCC27255]RBATCC27255_01313_HTH-type_transcriptional_regulatory_protein_GabR	RbromiiATCC27255
Org3_Gene1275	accessory	1814	[RbromiiATCC27255]RBATCC27255_01314_hypothetical_protein	RbromiiATCC27255
Org3_Gene1276	accessory	1008	[RbromiiATCC27255]RBATCC27255_01315_Penicillin-binding_protein_E	RbromiiATCC27255
Org3_Gene1278	accessory	3200	[RbromiiATCC27255]RBATCC27255_01317_hypothetical_protein	RbromiiATCC27255
Org3_Gene1279	accessory	1458	[RbromiiATCC27255]RBATCC27255_01318_DNA_replication_protein_DnaC	RbromiiATCC27255
Org3_Gene1280	accessory	1231	[RbromiiATCC27255]RBATCC27255_01319_DnaD_domain_protein	RbromiiATCC27255
Org3_Gene1281	accessory	4008	[RbromiiATCC27255]RBATCC27255_01320_TSCPD_domain_protein	RbromiiATCC27255
Org3_Gene1284	accessory	1338	[RbromiiATCC27255]RBATCC27255_01323_hypothetical_protein	RbromiiATCC27255
Org3_Gene1286	accessory	1969	[RbromiiATCC27255]RBATCC27255_01325_Histidinol-phosphatase	RbromiiATCC27255
Org3_Gene1289	accessory	3794	[RbromiiATCC27255]RBATCC27255_01328_hypothetical_protein	RbromiiATCC27255
Org3_Gene1291	accessory	305	[RbromiiATCC27255]RBATCC27255_01330_V-type_ATP_synthase_subunit_I	RbromiiATCC27255
Org3_Gene1294	accessory	2707	[RbromiiATCC27255]RBATCC27255_01333_V-type_ATP_synthase_subunit_E	RbromiiATCC27255
Org3_Gene1298	accessory	3278	[RbromiiATCC27255]RBATCC27255_01337_hypothetical_protein	RbromiiATCC27255
Org3_Gene1299	accessory	3755	[RbromiiATCC27255]RBATCC27255_01338_hypothetical_protein	RbromiiATCC27255
Org3_Gene130	accessory	476	[RbromiiATCC27255]RBATCC27255_00135_Acetyl-coenzyme_A_synthetase	RbromiiATCC27255
Org3_Gene1301	accessory	392	[RbromiiATCC27255]RBATCC27255_01340_1-deoxy-D-xylulose-5-phosphate_synthase	RbromiiATCC27255
Org3_Gene1302	accessory	1087	[RbromiiATCC27255]RBATCC27255_01345_CYTH_domain_protein	RbromiiATCC27255
Org3_Gene1304	accessory	2323	[RbromiiATCC27255]RBATCC27255_01347_hypothetical_protein	RbromiiATCC27255
Org3_Gene1305	accessory	1704	[RbromiiATCC27255]RBATCC27255_01348_hypothetical_protein	RbromiiATCC27255
Org3_Gene1308	accessory	1725	[RbromiiATCC27255]RBATCC27255_01351_hypothetical_protein	RbromiiATCC27255
Org3_Gene131	accessory	1987	[RbromiiATCC27255]RBATCC27255_00136_hypothetical_protein	RbromiiATCC27255
Org3_Gene1312	accessory	3777	[RbromiiATCC27255]RBATCC27255_01355_putative_ribosomal_protein	RbromiiATCC27255
Org3_Gene1316	accessory	915	[RbromiiATCC27255]RBATCC27255_01359_Peptidase_M16_inactive_domain_protein	RbromiiATCC27255
Org3_Gene1317	accessory	3090	[RbromiiATCC27255]RBATCC27255_01360_hypothetical_protein	RbromiiATCC27255
Org3_Gene132	accessory	3466	[RbromiiATCC27255]RBATCC27255_00137_hypothetical_protein	RbromiiATCC27255
Org3_Gene1321	accessory	1261	[RbromiiATCC27255]RBATCC27255_01364_Putative_prophage_phiRv2_integrase	RbromiiATCC27255
Org3_Gene1322	accessory	3692	[RbromiiATCC27255]RBATCC27255_01365_hypothetical_protein	RbromiiATCC27255
Org3_Gene1323	accessory	1040	[RbromiiATCC27255]RBATCC27255_01366_Replicative_DNA_helicase	RbromiiATCC27255
Org3_Gene133	accessory	3623	[RbromiiATCC27255]RBATCC27255_00138_hypothetical_protein	RbromiiATCC27255
Org3_Gene1331	accessory	3212	[RbromiiATCC27255]RBATCC27255_01374_hypothetical_protein	RbromiiATCC27255
Org3_Gene1332	accessory	2409	[RbromiiATCC27255]RBATCC27255_01375_hypothetical_protein	RbromiiATCC27255
Org3_Gene1333	accessory	1954	[RbromiiATCC27255]RBATCC27255_01376_ABC-2_family_transporter_protein	RbromiiATCC27255
Org3_Gene1334	accessory	2381	[RbromiiATCC27255]RBATCC27255_01377_hypothetical_protein	RbromiiATCC27255
Org3_Gene1335	accessory	1919	[RbromiiATCC27255]RBATCC27255_01378_ABC-2_family_transporter_protein	RbromiiATCC27255
Org3_Gene1336	accessory	2523	[RbromiiATCC27255]RBATCC27255_01379_Fluoroquinolones_export_ATP-binding_proteinc/MT2762	RbromiiATCC27255
Org3_Gene1337	accessory	2776	[RbromiiATCC27255]RBATCC27255_01380_hypothetical_protein	RbromiiATCC27255
Org3_Gene1338	accessory	4342	[RbromiiATCC27255]RBATCC27255_01381_hypothetical_protein	RbromiiATCC27255
Org3_Gene1340	accessory	752	[RbromiiATCC27255]RBATCC27255_01383_Na+-dependent_transporters_of_the_SNF_family_protein	RbromiiATCC27255
Org3_Gene1354	accessory	3295	[RbromiiATCC27255]RBATCC27255_01397_hypothetical_protein	RbromiiATCC27255
Org3_Gene1358	accessory	3183	[RbromiiATCC27255]RBATCC27255_01401_Low_molecular_weight_protein-tyrosine-phosphatase_YfkJ	RbromiiATCC27255
Org3_Gene1359	accessory	2637	[RbromiiATCC27255]RBATCC27255_01402_DNA-binding_transcriptional_repressor_AcrR	RbromiiATCC27255
Org3_Gene1360	accessory	32	[RbromiiATCC27255]RBATCC27255_01403_Swarming_motility_protein_SwrC	RbromiiATCC27255
Org3_Gene1362	accessory	3801	[RbromiiATCC27255]RBATCC27255_01405_V-type_ATP_synthase_subunit_H	RbromiiATCC27255
Org3_Gene1363	accessory	287	[RbromiiATCC27255]RBATCC27255_01406_V-type_ATP_synthase_subunit_I	RbromiiATCC27255
Org3_Gene1365	accessory	2760	[RbromiiATCC27255]RBATCC27255_01408_V-type_ATP_synthase_subunit_E	RbromiiATCC27255
Org3_Gene1366	accessory	1493	[RbromiiATCC27255]RBATCC27255_01409_V-type_sodium_pump_subunit_C	RbromiiATCC27255
Org3_Gene1371	accessory	2826	[RbromiiATCC27255]RBATCC27255_01414_hypothetical_protein	RbromiiATCC27255
Org3_Gene1372	accessory	742	[RbromiiATCC27255]RBATCC27255_01415_Staphylococcal_virulence_regulator_protein_A	RbromiiATCC27255
Org3_Gene1373	accessory	4003	[RbromiiATCC27255]RBATCC27255_01416_Transcriptional_repressor_SdpR	RbromiiATCC27255
Org3_Gene1374	accessory	2479	[RbromiiATCC27255]RBATCC27255_01417_Immunity_protein_SdpI	RbromiiATCC27255
Org3_Gene1376	accessory	3997	[RbromiiATCC27255]RBATCC27255_01419_hypothetical_protein	RbromiiATCC27255
Org3_Gene1377	accessory	2100	[RbromiiATCC27255]RBATCC27255_01420_Zinc_transporter_ZupT	RbromiiATCC27255
Org3_Gene1378	accessory	73	[RbromiiATCC27255]RBATCC27255_01421_Nuclease_SbcCD_subunit_C	RbromiiATCC27255
Org3_Gene1379	accessory	1104	[RbromiiATCC27255]RBATCC27255_01422_Nuclease_SbcCD_subunit_D	RbromiiATCC27255
Org3_Gene138	accessory	2320	[RbromiiATCC27255]RBATCC27255_00143_Ribosomal_RNA_small_subunit_methyltransferase_G	RbromiiATCC27255
Org3_Gene1380	accessory	3815	[RbromiiATCC27255]RBATCC27255_01423_putative_membrane_protein	RbromiiATCC27255
Org3_Gene1381	accessory	2215	[RbromiiATCC27255]RBATCC27255_01424_Inner_membrane_protein_YgaZ	RbromiiATCC27255
Org3_Gene1382	accessory	3468	[RbromiiATCC27255]RBATCC27255_01425_serine-protein_kinase_RsbW	RbromiiATCC27255
Org3_Gene1383	accessory	3806	[RbromiiATCC27255]RBATCC27255_01426_Stage_II_sporulation_protein_AA	RbromiiATCC27255
Org3_Gene1384	accessory	389	[RbromiiATCC27255]RBATCC27255_01427_Multidrug_export_protein_MepA	RbromiiATCC27255
Org3_Gene1385	accessory	6	[RbromiiATCC27255]RBATCC27255_01428_Tyrocidine_synthase_III	RbromiiATCC27255
Org3_Gene1386	accessory	2901	[RbromiiATCC27255]RBATCC27255_01429_VanZ_like_family_protein	RbromiiATCC27255
Org3_Gene1387	accessory	2352	[RbromiiATCC27255]RBATCC27255_01430_Sensory_transduction_protein_LytR	RbromiiATCC27255
Org3_Gene1388	accessory	893	[RbromiiATCC27255]RBATCC27255_01431_hypothetical_protein	RbromiiATCC27255
Org3_Gene1389	accessory	2955	[RbromiiATCC27255]RBATCC27255_01432_Methylated-DNA--protein-cysteine_methyltransferase,_inducible	RbromiiATCC27255
Org3_Gene1390	accessory	2887	[RbromiiATCC27255]RBATCC27255_01436_hypothetical_protein	RbromiiATCC27255
Org3_Gene1391	accessory	272	[RbromiiATCC27255]RBATCC27255_01437_Metallo-beta-lactamase_superfamily_protein	RbromiiATCC27255
Org3_Gene1392	accessory	2284	[RbromiiATCC27255]RBATCC27255_01438_Transcriptional_regulatory_protein_YpdB	RbromiiATCC27255
Org3_Gene1393	accessory	840	[RbromiiATCC27255]RBATCC27255_01439_sensor_histidine_kinase_DpiB	RbromiiATCC27255
Org3_Gene1394	accessory	1381	[RbromiiATCC27255]RBATCC27255_01440_Penicillin_V_acylase	RbromiiATCC27255
Org3_Gene1395	accessory	1299	[RbromiiATCC27255]RBATCC27255_01441_Tryptophan--tRNA_ligase_2	RbromiiATCC27255
Org3_Gene1396	accessory	2422	[RbromiiATCC27255]RBATCC27255_01442_Bifunctional_protein_aas	RbromiiATCC27255
Org3_Gene1398	accessory	213	[RbromiiATCC27255]RBATCC27255_01444_3-oxoacyl-[acyl-carrier-protein]_synthase_2	RbromiiATCC27255
Org3_Gene14	accessory	1580	[RbromiiATCC27255]RBATCC27255_00016_auxin_efflux_carrier	RbromiiATCC27255
Org3_Gene1401	accessory	2363	[RbromiiATCC27255]RBATCC27255_01447_azoreductase	RbromiiATCC27255
Org3_Gene1402	accessory	2662	[RbromiiATCC27255]RBATCC27255_01448_mycofactocin_system_transcriptional_regulator	RbromiiATCC27255
Org3_Gene1403	accessory	1905	[RbromiiATCC27255]RBATCC27255_01449_Patatin-like_phospholipase	RbromiiATCC27255
Org3_Gene1404	accessory	2874	[RbromiiATCC27255]RBATCC27255_01450_hypothetical_protein	RbromiiATCC27255
Org3_Gene1405	accessory	3263	[RbromiiATCC27255]RBATCC27255_01451_HTH-type_transcriptional_regulator_AdhR	RbromiiATCC27255
Org3_Gene1406	accessory	4263	[RbromiiATCC27255]RBATCC27255_01452_hypothetical_protein	RbromiiATCC27255
Org3_Gene1407	accessory	3131	[RbromiiATCC27255]RBATCC27255_01453_RNA_polymerase_sigma_factor_SigM	RbromiiATCC27255
Org3_Gene1408	accessory	2311	[RbromiiATCC27255]RBATCC27255_01454_hypothetical_protein	RbromiiATCC27255
Org3_Gene1409	accessory	3419	[RbromiiATCC27255]RBATCC27255_01455_Peptide_deformylase_2	RbromiiATCC27255
Org3_Gene1410	accessory	2861	[RbromiiATCC27255]RBATCC27255_01456_hypothetical_protein	RbromiiATCC27255
Org3_Gene1411	accessory	2931	[RbromiiATCC27255]RBATCC27255_01457_hypothetical_protein	RbromiiATCC27255
Org3_Gene1412	accessory	4006	[RbromiiATCC27255]RBATCC27255_01458_hypothetical_protein	RbromiiATCC27255
Org3_Gene1414	accessory	2779	[RbromiiATCC27255]RBATCC27255_01460_putative_membrane_protein	RbromiiATCC27255
Org3_Gene1415	accessory	3693	[RbromiiATCC27255]RBATCC27255_01461_anaerobic_benzoate_catabolism_transcriptional_regulator	RbromiiATCC27255
Org3_Gene1416	accessory	125	[RbromiiATCC27255]RBATCC27255_01462_Elongation_factor_G	RbromiiATCC27255
Org3_Gene1417	accessory	3156	[RbromiiATCC27255]RBATCC27255_01463_putative_acetyltransferase	RbromiiATCC27255
Org3_Gene1419	accessory	108	[RbromiiATCC27255]RBATCC27255_01465_CDP-Glycerol:Poly(glycerophosphate)_glycerophosphotransferase	RbromiiATCC27255
Org3_Gene142	accessory	1410	[RbromiiATCC27255]RBATCC27255_00147_Oxa1Ec	RbromiiATCC27255
Org3_Gene1420	accessory	1220	[RbromiiATCC27255]RBATCC27255_01466_Acyltransferase_family_protein	RbromiiATCC27255

Org3_Gene1421	accessory	3515	[RbromiiATCC27255]RBATCC27255_01467_hypothetical_protein	RbromiiATCC27255
Org3_Gene1422	accessory	4217	[RbromiiATCC27255]RBATCC27255_01468_TM2_domain-containing_protein	RbromiiATCC27255
Org3_Gene1423	accessory	439	[RbromiiATCC27255]RBATCC27255_01469_Sulfate_adenylyltransferase_subunit_1	RbromiiATCC27255
Org3_Gene1424	accessory	1700	[RbromiiATCC27255]RBATCC27255_01470_Sulfate_adenylyltransferase_subunit_2	RbromiiATCC27255
Org3_Gene1425	accessory	3783	[RbromiiATCC27255]RBATCC27255_01471_Ferredoxin_II	RbromiiATCC27255
Org3_Gene1426	accessory	442	[RbromiiATCC27255]RBATCC27255_01472_Fumarate_reductase_flavoprotein_subunit_precursor	RbromiiATCC27255
Org3_Gene1427	accessory	1657	[RbromiiATCC27255]RBATCC27255_01473_Thioredoxin_reductase	RbromiiATCC27255
Org3_Gene1428	accessory	3425	[RbromiiATCC27255]RBATCC27255_01474_CysO-cysteine_peptidase	RbromiiATCC27255
Org3_Gene1429	accessory	2001	[RbromiiATCC27255]RBATCC27255_01475_putative_adenylyltransferase/sulfurtransferase_MoeZ	RbromiiATCC27255
Org3_Gene1430	accessory	4189	[RbromiiATCC27255]RBATCC27255_01476_bifunctional_sulfur_carrier_protein/thiazole_synthase_protein	RbromiiATCC27255
Org3_Gene1431	accessory	3759	[RbromiiATCC27255]RBATCC27255_01477_Thioredoxin-M	RbromiiATCC27255
Org3_Gene1432	accessory	4035	[RbromiiATCC27255]RBATCC27255_01478_selenium_metabolism_protein_YedF	RbromiiATCC27255
Org3_Gene1433	accessory	1835	[RbromiiATCC27255]RBATCC27255_01479_Anaerobic_sulfite_reductase_subunit_C	RbromiiATCC27255
Org3_Gene1434	accessory	940	[RbromiiATCC27255]RBATCC27255_01480_Methionine_gamma-lyase	RbromiiATCC27255
Org3_Gene1435	accessory	3248	[RbromiiATCC27255]RBATCC27255_01481_HTH-type_transcriptional_regulator_IscR	RbromiiATCC27255
Org3_Gene1436	accessory	920	[RbromiiATCC27255]RBATCC27255_01482_Glutamate-1-semialdehyde_2,1-aminomutase_2	RbromiiATCC27255
Org3_Gene1437	accessory	1492	[RbromiiATCC27255]RBATCC27255_01483_Delta-aminolevulinic_acid_dehydratase	RbromiiATCC27255
Org3_Gene1438	accessory	591	[RbromiiATCC27255]RBATCC27255_01484_Uroporphyrinogen-III_C-methyltransferase	RbromiiATCC27255
Org3_Gene1439	accessory	1836	[RbromiiATCC27255]RBATCC27255_01485_Porphobilinogen_deaminase	RbromiiATCC27255
Org3_Gene1440	accessory	2659	[RbromiiATCC27255]RBATCC27255_01486_Siroheme_synthase	RbromiiATCC27255
Org3_Gene1441	accessory	1165	[RbromiiATCC27255]RBATCC27255_01487_Glutamyl-tRNA_reductase	RbromiiATCC27255
Org3_Gene1443	accessory	1303	[RbromiiATCC27255]RBATCC27255_01489_Sulfate/thiosulfate_import_ATP-binding_protein_CysA	RbromiiATCC27255
Org3_Gene1444	accessory	1971	[RbromiiATCC27255]RBATCC27255_01490_Sulfate_transport_system_permease_protein_CysW	RbromiiATCC27255
Org3_Gene1445	accessory	1867	[RbromiiATCC27255]RBATCC27255_01491_Sulfate_transport_system_permease_protein_CysT	RbromiiATCC27255
Org3_Gene1446	accessory	1342	[RbromiiATCC27255]RBATCC27255_01492_Sulfate_starvation-induced_protein_2	RbromiiATCC27255
Org3_Gene1447	accessory	1071	[RbromiiATCC27255]RBATCC27255_01493_NADH_peroxidase	RbromiiATCC27255
Org3_Gene1448	accessory	1481	[RbromiiATCC27255]RBATCC27255_01494_1,5-anhydro-D-fructose_reductase	RbromiiATCC27255
Org3_Gene1449	accessory	3490	[RbromiiATCC27255]RBATCC27255_01495_hypothetical_protein	RbromiiATCC27255
Org3_Gene1450	accessory	634	[RbromiiATCC27255]RBATCC27255_01496_Staphylococcal_virulence_regulator_protein_A	RbromiiATCC27255
Org3_Gene1452	accessory	3591	[RbromiiATCC27255]RBATCC27255_01498_hypothetical_protein	RbromiiATCC27255
Org3_Gene1453	accessory	2825	[RbromiiATCC27255]RBATCC27255_01499_Putative_ribosomal_N-acetyltransferase_YdaF	RbromiiATCC27255
Org3_Gene1454	accessory	879	[RbromiiATCC27255]RBATCC27255_01500_Alpha-1,4-glucan:maltose-1-phosphate_maltosyltransferase_1	RbromiiATCC27255
Org3_Gene1455	accessory	2392	[RbromiiATCC27255]RBATCC27255_01501_putative_flavoprotein	RbromiiATCC27255
Org3_Gene1457	accessory	1661	[RbromiiATCC27255]RBATCC27255_01503_HTH-type_transcriptional_regulator_GltC	RbromiiATCC27255
Org3_Gene1460	accessory	3284	[RbromiiATCC27255]RBATCC27255_01506_Acyl-CoA_thioester_hydrolase_YbgC	RbromiiATCC27255
Org3_Gene1465	accessory	311	[RbromiiATCC27255]RBATCC27255_01511_1,4-alpha-glucan_branching_enzyme_GlgB	RbromiiATCC27255
Org3_Gene1469	accessory	2798	[RbromiiATCC27255]RBATCC27255_01515_Folate_ECF_transporter_S_component_FolT	RbromiiATCC27255
Org3_Gene1470	accessory	2502	[RbromiiATCC27255]RBATCC27255_01517_Riboflavin_ECF_transporter_S_component_RibU	RbromiiATCC27255
Org3_Gene1471	accessory	2274	[RbromiiATCC27255]RBATCC27255_01519_DNA_alkylation_repair_enzyme	RbromiiATCC27255
Org3_Gene1474	accessory	2081	[RbromiiATCC27255]RBATCC27255_01522_hypothetical_protein	RbromiiATCC27255
Org3_Gene1476	accessory	143	[RbromiiATCC27255]RBATCC27255_01524_putative_membrane_protein	RbromiiATCC27255
Org3_Gene1479	accessory	816	[RbromiiATCC27255]RBATCC27255_01527_Putative_transcriptional_regulator_YvhJ	RbromiiATCC27255
Org3_Gene1480	accessory	2006	[RbromiiATCC27255]RBATCC27255_01528_sortase,_SrtB_family	RbromiiATCC27255
Org3_Gene1481	accessory	3059	[RbromiiATCC27255]RBATCC27255_01529_hypothetical_protein	RbromiiATCC27255
Org3_Gene1485	accessory	3108	[RbromiiATCC27255]RBATCC27255_01533_Small_T_antigen	RbromiiATCC27255
Org3_Gene1490	accessory	860	[RbromiiATCC27255]RBATCC27255_01538_Replication_initiation_factor	RbromiiATCC27255
Org3_Gene1491	accessory	3140	[RbromiiATCC27255]RBATCC27255_01539_hypothetical_protein	RbromiiATCC27255
Org3_Gene1492	accessory	3345	[RbromiiATCC27255]RBATCC27255_01540_hypothetical_protein	RbromiiATCC27255
Org3_Gene1493	accessory	4328	[RbromiiATCC27255]RBATCC27255_01541_hypothetical_protein	RbromiiATCC27255
Org3_Gene1494	accessory	1019	[RbromiiATCC27255]RBATCC27255_01542_Integrase	RbromiiATCC27255
Org3_Gene1496	accessory	2678	[RbromiiATCC27255]RBATCC27255_01545_molybdenum_cofactor_biosynthesis_protein_A	RbromiiATCC27255
Org3_Gene1497	accessory	2872	[RbromiiATCC27255]RBATCC27255_01546_putative_membrane_protein	RbromiiATCC27255
Org3_Gene1498	accessory	1853	[RbromiiATCC27255]RBATCC27255_01547_Pyridoxine_kinase	RbromiiATCC27255
Org3_Gene1502	accessory	2543	[RbromiiATCC27255]RBATCC27255_01551_5'-nucleotidase	RbromiiATCC27255
Org3_Gene1503	accessory	504	[RbromiiATCC27255]RBATCC27255_01552_putative_bicarbonate_transporter,_IctB_family	RbromiiATCC27255
Org3_Gene1504	accessory	1828	[RbromiiATCC27255]RBATCC27255_01553_Poly-beta-1,6-N-acetyl-D-glucosamine_N-deacetylase_precursor	RbromiiATCC27255
Org3_Gene1505	accessory	3483	[RbromiiATCC27255]RBATCC27255_01554_Peroxide-responsive_repressor_PerR	RbromiiATCC27255
Org3_Gene1507	accessory	4372	[RbromiiATCC27255]RBATCC27255_01556_Rubredoxin	RbromiiATCC27255
Org3_Gene151	accessory	2258	[RbromiiATCC27255]RBATCC27255_00156_cxxc_20_cxxc_protein	RbromiiATCC27255
Org3_Gene1513	accessory	3575	[RbromiiATCC27255]RBATCC27255_01562_hypothetical_protein	RbromiiATCC27255
Org3_Gene1523	accessory	865	[RbromiiATCC27255]RBATCC27255_01572_hypothetical_protein	RbromiiATCC27255
Org3_Gene1524	accessory	2453	[RbromiiATCC27255]RBATCC27255_01573_Colicin_V_production_protein	RbromiiATCC27255
Org3_Gene1525	accessory	2628	[RbromiiATCC27255]RBATCC27255_01574_CDP-diacylglycerol--glycerol-3-phosphate_3-phosphatidyltransferase	RbromiiATCC27255
Org3_Gene153	accessory	3328	[RbromiiATCC27255]RBATCC27255_00158_acyl-CoA_thioesterase_YbgC	RbromiiATCC27255
Org3_Gene1530	accessory	1839	[RbromiiATCC27255]RBATCC27255_01579_putative_integral_membrane_protein	RbromiiATCC27255
Org3_Gene1534	accessory	4415	[RbromiiATCC27255]RBATCC27255_01583_hypothetical_protein	RbromiiATCC27255
Org3_Gene1537	accessory	3899	[RbromiiATCC27255]RBATCC27255_01586_hypothetical_protein	RbromiiATCC27255
Org3_Gene1539	accessory	4135	[RbromiiATCC27255]RBATCC27255_01589_hypothetical_protein	RbromiiATCC27255
Org3_Gene1540	accessory	3944	[RbromiiATCC27255]RBATCC27255_01590_Toxin_RelG	RbromiiATCC27255
Org3_Gene1542	accessory	4192	[RbromiiATCC27255]RBATCC27255_01592_hypothetical_protein	RbromiiATCC27255
Org3_Gene1545	accessory	564	[RbromiiATCC27255]RBATCC27255_01595_hypothetical_protein	RbromiiATCC27255
Org3_Gene1547	accessory	3365	[RbromiiATCC27255]RBATCC27255_01597_oxaloacetate_decarboxylase_subunit_gamma	RbromiiATCC27255
Org3_Gene1549	accessory	2257	[RbromiiATCC27255]RBATCC27255_01599_polysaccharide_export_protein,_MPA1_family	RbromiiATCC27255
Org3_Gene155	accessory	2176	[RbromiiATCC27255]RBATCC27255_00160_hypothetical_protein	RbromiiATCC27255
Org3_Gene1550	accessory	2289	[RbromiiATCC27255]RBATCC27255_01600_Tyrosine-protein_kinase_YwqD	RbromiiATCC27255
Org3_Gene1554	accessory	1456	[RbromiiATCC27255]RBATCC27255_01604_putative_zinc_transport_system_zinc-binding_lipoprotein_AdcA_precursor	RbromiiATCC27255
Org3_Gene1555	accessory	2203	[RbromiiATCC27255]RBATCC27255_01605_putative_zinc_transport_system_ATP-binding_protein_AdcC	RbromiiATCC27255
Org3_Gene1556	accessory	1915	[RbromiiATCC27255]RBATCC27255_01606_High-affinity_zinc_uptake_system_membrane_protein_ZnuB	RbromiiATCC27255
Org3_Gene1557	accessory	3524	[RbromiiATCC27255]RBATCC27255_01607_hypothetical_protein	RbromiiATCC27255
Org3_Gene1558	accessory	2312	[RbromiiATCC27255]RBATCC27255_01608_hypothetical_protein	RbromiiATCC27255
Org3_Gene1560	accessory	1290	[RbromiiATCC27255]RBATCC27255_01610_spore_germination_protein_(amino_acid_permease)	RbromiiATCC27255
Org3_Gene1561	accessory	2738	[RbromiiATCC27255]RBATCC27255_01611_germination_protein,_Ger(x)C_family	RbromiiATCC27255
Org3_Gene1566	accessory	4117	[RbromiiATCC27255]RBATCC27255_01616_exodeoxyribonuclease_VII_small_subunit	RbromiiATCC27255
Org3_Gene1570	accessory	1849	[RbromiiATCC27255]RBATCC27255_01620_putative_inorganic_polyphosphate/ATP-NAD_kinase	RbromiiATCC27255
Org3_Gene1571	accessory	3272	[RbromiiATCC27255]RBATCC27255_01621_Arginine_hydroxamate_resistance_protein	RbromiiATCC27255
Org3_Gene1576	accessory	267	[RbromiiATCC27255]RBATCC27255_01626_Single-stranded-DNA-specific_exonuclease_RecJ	RbromiiATCC27255
Org3_Gene158	accessory	327	[RbromiiATCC27255]RBATCC27255_00163_Alpha-amylase/pullulanase	RbromiiATCC27255
Org3_Gene1581	accessory	3434	[RbromiiATCC27255]RBATCC27255_01631_hypothetical_protein	RbromiiATCC27255
Org3_Gene1582	accessory	345	[RbromiiATCC27255]RBATCC27255_01632_Stage_III_sporulation_protein_F	RbromiiATCC27255
Org3_Gene1588	accessory	3277	[RbromiiATCC27255]RBATCC27255_01638_Septum_formation_initiator	RbromiiATCC27255
Org3_Gene159	accessory	2560	[RbromiiATCC27255]RBATCC27255_00164_cell_division_protein_ZapA	RbromiiATCC27255
Org3_Gene1595	accessory	3034	[RbromiiATCC27255]RBATCC27255_01645_preprotein_translocase_subunit_SecD	RbromiiATCC27255
Org3_Gene1597	accessory	1549	[RbromiiATCC27255]RBATCC27255_01647_stage_III_sporulation_protein_AA	RbromiiATCC27255
Org3_Gene1598	accessory	3075	[RbromiiATCC27255]RBATCC27255_01648_stage_III_sporulation_protein_SpoAB	RbromiiATCC27255
Org3_Gene160	accessory	262	[RbromiiATCC27255]RBATCC27255_00165_putative_protease_YhbU_precursor	RbromiiATCC27255
Org3_Gene1600	accessory	3501	[RbromiiATCC27255]RBATCC27255_01650_stage_III_sporulation_protein_AD	RbromiiATCC27255
Org3_Gene1601	accessory	1199	[RbromiiATCC27255]RBATCC27255_01651_stage_III_sporulation_protein_AE	RbromiiATCC27255
Org3_Gene1602	accessory	3177	[RbromiiATCC27255]RBATCC27255_01652_hypothetical_protein	RbromiiATCC27255
Org3_Gene1604	accessory	2903	[RbromiiATCC27255]RBATCC27255_01654_SpoIIIAH-like_protein	RbromiiATCC27255
Org3_Gene1605	accessory	2729	[RbromiiATCC27255]RBATCC27255_01655_Translation_initiation_factor_IF-3	RbromiiATCC27255
Org3_Gene1608	accessory	2102	[RbromiiATCC27255]RBATCC27255_01658_23S_rRNA_(uridine(2479)-2'-O)-methyltransferase	RbromiiATCC27255
Org3_Gene1609	accessory	927	[RbromiiATCC27255]RBATCC27255_01659_DNA_protecting_protein_DprA	RbromiiATCC27255
Org3_Gene1614	accessory	1574	[RbromiiATCC27255]RBATCC27255_01664_Oxygen-independent_coproporphyrinogen-III_oxidase_1	RbromiiATCC27255
Org3_Gene1619	accessory	2681	[RbromiiATCC27255]RBATCC27255_01669_putative_nicotinate-nucleotide_adenylyltransferase	RbromiiATCC27255
Org3_Gene1620	accessory	2743	[RbromiiATCC27255]RBATCC27255_01670_putative_nicotinate-nucleotide_adenylyltransferase	RbromiiATCC27255
Org3_Gene1625	accessory	1242	[RbromiiATCC27255]RBATCC27255_01675_putative_peptidase	RbromiiATCC27255
Org3_Gene1627	accessory	3110	[RbromiiATCC27255]RBATCC27255_01677_hypothetical_protein	RbromiiATCC27255
Org3_Gene1628	accessory	2532	[RbromiiATCC27255]RBATCC27255_01678_Sporulation_protein_YunB	RbromiiATCC27255
Org3_Gene163	accessory	1973	[RbromiiATCC27255]RBATCC27255_00168_Rod_shape-determining_protein_MreC	RbromiiATCC27255
Org3_Gene1632	accessory	229	[RbromiiATCC27255]RBATCC27255_01682_DNA_mismatch_repair_protein_MutL	RbromiiATCC27255
Org3_Gene1633	accessory	1550	[RbromiiATCC27255]RBATCC27255_01683_tRNA_dimethylallyltransferase	RbromiiATCC27255
Org3_Gene1634	accessory	877	[RbromiiATCC27255]RBATCC27255_01684_putative_membrane_fusion_protein	RbromiiATCC27255
Org3_Gene1637	accessory	2147	[RbromiiATCC27255]RBATCC27255_01687_photosystem_II_S4_domain_protein	RbromiiATCC27255
Org3_Gene1638	accessory	2446	[RbromiiATCC27255]RBATCC27255_01688_Minicell-associated_protein_DivIVA	RbromiiATCC27255
Org3_Gene164	accessory	3046	[RbromiiATCC27255]RBATCC27255_00169_rod_shape-determining_protein_MreD	RbromiiATCC27255
Org3_Gene1643	accessory	2568	[RbromiiATCC27255]RBATCC27255_01693_Signal_peptidase_I_P	RbromiiATCC27255
Org3_Gene1644	accessory	2785	[RbromiiATCC27255]RBATCC27255_01694_Signal_peptidase_I_T	RbromiiATCC27255
Org3_Gene1647	accessory	3585	[RbromiiATCC27255]RBATCC27255_01697_hypothetical_protein	RbromiiATCC27255
Org3_Gene1648	accessory	1578	[RbromiiATCC27255]RBATCC27255_01698_Membrane_dipeptidase_(Peptidase_family_M19)	RbromiiATCC27255
Org3_Gene165	accessory	276	[RbromiiATCC27255]RBATCC27255_00170_Penicillin-binding_protein_2B	RbromiiATCC27255
Org3_Gene1659	accessory	1508	[RbromiiATCC27255]RBATCC27255_01709_Cytochrome_c_biogenesis_protein_CycY	RbromiiATCC27255
Org3_Gene1660	accessory	1457	[RbromiiATCC27255]RBATCC27255_01710_Putative_electron_transport_protein_YccM	RbromiiATCC27255

Org3_Gene1661	accessory	2442	[RbromiiATCC27255]RBATCC27255_01711_Transcriptional_regulatory_protein_QseB	RbromiiATCC27255
Org3_Gene1662	accessory	1157	[RbromiiATCC27255]RBATCC27255_01712_Signal_transduction_histidine-protein_kinase_ArlS	RbromiiATCC27255
Org3_Gene1663	accessory	2680	[RbromiiATCC27255]RBATCC27255_01713_DNA-binding_transcriptional_repressor_AcrR	RbromiiATCC27255
Org3_Gene1664	accessory	293	[RbromiiATCC27255]RBATCC27255_01714_Collagen_triple_helix_repeat_(20_copies)	RbromiiATCC27255
Org3_Gene1665	accessory	2146	[RbromiiATCC27255]RBATCC27255_01715_putative_membrane_protein	RbromiiATCC27255
Org3_Gene1666	accessory	2210	[RbromiiATCC27255]RBATCC27255_01716_ABC_transporter_efflux_protein,_DrrB_family	RbromiiATCC27255
Org3_Gene1667	accessory	2271	[RbromiiATCC27255]RBATCC27255_01717_Daunorubicin/doxorubicin_resistance_ATP-binding_protein_DrrA	RbromiiATCC27255
Org3_Gene1668	accessory	3416	[RbromiiATCC27255]RBATCC27255_01718_transcriptional_repressor_DicA	RbromiiATCC27255
Org3_Gene1669	accessory	2746	[RbromiiATCC27255]RBATCC27255_01719_hypothetical_protein	RbromiiATCC27255
Org3_Gene1670	accessory	1937	[RbromiiATCC27255]RBATCC27255_01720_hypothetical_protein	RbromiiATCC27255
Org3_Gene1673	accessory	1648	[RbromiiATCC27255]RBATCC27255_01723_tRNA_pseudouridine_synthase_B	RbromiiATCC27255
Org3_Gene1674	accessory	1575	[RbromiiATCC27255]RBATCC27255_01724_Bifunctional_oligoribonuclease_and_PAP_phosphatase_NrnA	RbromiiATCC27255
Org3_Gene1677	accessory	3711	[RbromiiATCC27255]RBATCC27255_01727_putative_ribosomal_protein_YlxQ	RbromiiATCC27255
Org3_Gene1678	accessory	3805	[RbromiiATCC27255]RBATCC27255_01728_hypothetical_protein	RbromiiATCC27255
Org3_Gene1681	accessory	4240	[RbromiiATCC27255]RBATCC27255_01732_hypothetical_protein	RbromiiATCC27255
Org3_Gene1682	accessory	2984	[RbromiiATCC27255]RBATCC27255_01733_hypothetical_protein	RbromiiATCC27255
Org3_Gene1686	accessory	3393	[RbromiiATCC27255]RBATCC27255_01737_hypothetical_protein	RbromiiATCC27255
Org3_Gene1688	accessory	2561	[RbromiiATCC27255]RBATCC27255_01739_Vitamin_B12_dependent_methionine_synthase,_activation_domain	RbromiiATCC27255
Org3_Gene1697	accessory	370	[RbromiiATCC27255]RBATCC27255_01748_hypothetical_protein	RbromiiATCC27255
Org3_Gene1698	accessory	623	[RbromiiATCC27255]RBATCC27255_01749_Multidrug_export_protein_MepA	RbromiiATCC27255
Org3_Gene1699	accessory	1024	[RbromiiATCC27255]RBATCC27255_01750_putative_integral_membrane_protein	RbromiiATCC27255
Org3_Gene17	accessory	557	[RbromiiATCC27255]RBATCC27255_00019_putative_protein_involved_in_cytokinesis,_contains_TGc_(transglutaminase/protease-like)_domain	RbromiiATCC27255
Org3_Gene170	accessory	2096	[RbromiiATCC27255]RBATCC27255_00175_hypothetical_protein	RbromiiATCC27255
Org3_Gene1702	accessory	855	[RbromiiATCC27255]RBATCC27255_01753_hypothetical_protein	RbromiiATCC27255
Org3_Gene1704	accessory	2765	[RbromiiATCC27255]RBATCC27255_01755_hypothetical_protein	RbromiiATCC27255
Org3_Gene1706	accessory	113	[RbromiiATCC27255]RBATCC27255_01757_copper_exporting_ATPase	RbromiiATCC27255
Org3_Gene1715	accessory	3119	[RbromiiATCC27255]RBATCC27255_01766_Maltose_O-acetyltransferase	RbromiiATCC27255
Org3_Gene1716	accessory	934	[RbromiiATCC27255]RBATCC27255_01767_Glycosyltransferase_KanE	RbromiiATCC27255
Org3_Gene1717	accessory	1411	[RbromiiATCC27255]RBATCC27255_01768_General_stress_protein_A	RbromiiATCC27255
Org3_Gene172	accessory	1188	[RbromiiATCC27255]RBATCC27255_00177_hypothetical_protein	RbromiiATCC27255
Org3_Gene1722	accessory	2462	[RbromiiATCC27255]RBATCC27255_01773_Undecaprenyl_phosphate_N,N'-diacetylbacillosamine_1-phosphate_transferase	RbromiiATCC27255
Org3_Gene1723	accessory	2326	[RbromiiATCC27255]RBATCC27255_01774_Tyrosine-protein_kinase_YwqD	RbromiiATCC27255
Org3_Gene1724	accessory	2288	[RbromiiATCC27255]RBATCC27255_01775_Capsular_polysaccharide_type_8_biosynthesis_protein_cap8A	RbromiiATCC27255
Org3_Gene1725	accessory	2727	[RbromiiATCC27255]RBATCC27255_01776_hypothetical_protein	RbromiiATCC27255
Org3_Gene1726	accessory	2281	[RbromiiATCC27255]RBATCC27255_01777_sortase,_marine_proteobacterial_type	RbromiiATCC27255
Org3_Gene1727	accessory	1941	[RbromiiATCC27255]RBATCC27255_01778_hypothetical_protein	RbromiiATCC27255
Org3_Gene173	accessory	1977	[RbromiiATCC27255]RBATCC27255_00178_hypothetical_protein	RbromiiATCC27255
Org3_Gene1730	accessory	751	[RbromiiATCC27255]RBATCC27255_01781_Na+-dependent_transporters_of_the_SNF_family_protein	RbromiiATCC27255
Org3_Gene1731	accessory	218	[RbromiiATCC27255]RBATCC27255_01782_Polyphosphate_kinase	RbromiiATCC27255
Org3_Gene1732	accessory	1269	[RbromiiATCC27255]RBATCC27255_01783_Pyrophosphate--fructose_6-phosphate_1-phosphotransferase	RbromiiATCC27255
Org3_Gene1733	accessory	4322	[RbromiiATCC27255]RBATCC27255_01784_hypothetical_protein	RbromiiATCC27255
Org3_Gene1735	accessory	3449	[RbromiiATCC27255]RBATCC27255_01786_hypothetical_protein	RbromiiATCC27255
Org3_Gene1736	accessory	2930	[RbromiiATCC27255]RBATCC27255_01787_hypothetical_protein	RbromiiATCC27255
Org3_Gene174	accessory	1405	[RbromiiATCC27255]RBATCC27255_00179_Biotin_synthase	RbromiiATCC27255
Org3_Gene1741	accessory	3553	[RbromiiATCC27255]RBATCC27255_01792_hypothetical_protein	RbromiiATCC27255
Org3_Gene1752	accessory	3512	[RbromiiATCC27255]RBATCC27255_01803_hypothetical_protein	RbromiiATCC27255
Org3_Gene1753	accessory	976	[RbromiiATCC27255]RBATCC27255_01804_site-specific_tyrosine_recombinase_XerC	RbromiiATCC27255
Org3_Gene1756	accessory	4355	[RbromiiATCC27255]RBATCC27255_01808_hypothetical_protein	RbromiiATCC27255
Org3_Gene1759	accessory	2863	[RbromiiATCC27255]RBATCC27255_01811_Negative_regulator_of_genetic_competence,_sporulation_and_motility	RbromiiATCC27255
Org3_Gene176	accessory	1066	[RbromiiATCC27255]RBATCC27255_00181_tRNA_modification_GTPase_MnmE	RbromiiATCC27255
Org3_Gene1760	accessory	4325	[RbromiiATCC27255]RBATCC27255_01812_hypothetical_protein	RbromiiATCC27255
Org3_Gene1761	accessory	3672	[RbromiiATCC27255]RBATCC27255_01813_hypothetical_protein	RbromiiATCC27255
Org3_Gene1762	accessory	3532	[RbromiiATCC27255]RBATCC27255_01814_hypothetical_protein	RbromiiATCC27255
Org3_Gene1764	accessory	2185	[RbromiiATCC27255]RBATCC27255_01816_HTH-type_transcriptional_repressor_GlcR	RbromiiATCC27255
Org3_Gene1765	accessory	4058	[RbromiiATCC27255]RBATCC27255_01817_HTH-type_transcriptional_regulator_ImmR	RbromiiATCC27255
Org3_Gene1768	accessory	184	[RbromiiATCC27255]RBATCC27255_01820_Stage_II_sporulation_protein_E	RbromiiATCC27255
Org3_Gene1770	accessory	281	[RbromiiATCC27255]RBATCC27255_01822_CotH_protein	RbromiiATCC27255
Org3_Gene1772	accessory	3031	[RbromiiATCC27255]RBATCC27255_01824_Shikimate_kinase_2	RbromiiATCC27255
Org3_Gene1779	accessory	2932	[RbromiiATCC27255]RBATCC27255_01831_Shikimate_kinase	RbromiiATCC27255
Org3_Gene1783	accessory	4282	[RbromiiATCC27255]RBATCC27255_01835_hypothetical_protein	RbromiiATCC27255
Org3_Gene1787	accessory	3891	[RbromiiATCC27255]RBATCC27255_01839_hypothetical_protein	RbromiiATCC27255
Org3_Gene1788	accessory	3622	[RbromiiATCC27255]RBATCC27255_01840_hypothetical_protein	RbromiiATCC27255
Org3_Gene1789	accessory	4070	[RbromiiATCC27255]RBATCC27255_01841_hypothetical_protein	RbromiiATCC27255
Org3_Gene179	accessory	4367	[RbromiiATCC27255]RBATCC27255_00186_hypothetical_protein	RbromiiATCC27255
Org3_Gene1790	accessory	3178	[RbromiiATCC27255]RBATCC27255_01842_Phosphodiesterase_YfcE	RbromiiATCC27255
Org3_Gene1791	accessory	1827	[RbromiiATCC27255]RBATCC27255_01843_DNA_polymerase_III_subunit_tau	RbromiiATCC27255
Org3_Gene1794	accessory	2222	[RbromiiATCC27255]RBATCC27255_01846_tRNA1(Val)_(adenine(37)-N6)-methyltransferase	RbromiiATCC27255
Org3_Gene1796	accessory	845	[RbromiiATCC27255]RBATCC27255_01848_Folylpolyglutamate_synthase	RbromiiATCC27255
Org3_Gene18	accessory	2299	[RbromiiATCC27255]RBATCC27255_00021_hypothetical_protein	RbromiiATCC27255
Org3_Gene180	accessory	2977	[RbromiiATCC27255]RBATCC27255_00187_hypothetical_protein	RbromiiATCC27255
Org3_Gene1801	accessory	3748	[RbromiiATCC27255]RBATCC27255_01853_HTH-type_transcriptional_regulator_ImmR	RbromiiATCC27255
Org3_Gene1805	accessory	889	[RbromiiATCC27255]RBATCC27255_01858_hypothetical_protein	RbromiiATCC27255
Org3_Gene1808	accessory	1035	[RbromiiATCC27255]RBATCC27255_01861_hypothetical_protein	RbromiiATCC27255
Org3_Gene1809	accessory	1752	[RbromiiATCC27255]RBATCC27255_01862_hypothetical_protein	RbromiiATCC27255
Org3_Gene181	accessory	3407	[RbromiiATCC27255]RBATCC27255_00188_hypothetical_protein	RbromiiATCC27255
Org3_Gene1816	accessory	1584	[RbromiiATCC27255]RBATCC27255_01869_auxin_efflux_carrier	RbromiiATCC27255
Org3_Gene1817	accessory	3522	[RbromiiATCC27255]RBATCC27255_01870_hypothetical_protein	RbromiiATCC27255
Org3_Gene1820	accessory	1169	[RbromiiATCC27255]RBATCC27255_01873_cytidyltransferase-like_domain_protein	RbromiiATCC27255
Org3_Gene1821	accessory	2061	[RbromiiATCC27255]RBATCC27255_01874_hypothetical_protein	RbromiiATCC27255
Org3_Gene1823	accessory	1233	[RbromiiATCC27255]RBATCC27255_01876_stage_II_sporulation_protein_P	RbromiiATCC27255
Org3_Gene1825	accessory	462	[RbromiiATCC27255]RBATCC27255_01878_Papain_family_cysteine_protease	RbromiiATCC27255
Org3_Gene1826	accessory	478	[RbromiiATCC27255]RBATCC27255_01879_Phosphoserine_phosphatase_RsbU	RbromiiATCC27255
Org3_Gene1828	accessory	651	[RbromiiATCC27255]RBATCC27255_01881_Trk_system_potassium_uptake_protein_TrkA	RbromiiATCC27255
Org3_Gene1830	accessory	1060	[RbromiiATCC27255]RBATCC27255_01883_Alanine_racemase	RbromiiATCC27255
Org3_Gene1831	accessory	1372	[RbromiiATCC27255]RBATCC27255_01884_hypothetical_protein	RbromiiATCC27255
Org3_Gene1835	accessory	422	[RbromiiATCC27255]RBATCC27255_01888_Vancomycin_B-type_resistance_protein_VanW	RbromiiATCC27255
Org3_Gene1836	accessory	1154	[RbromiiATCC27255]RBATCC27255_01889_Processive_diacylglycerol_glucosyltransferase	RbromiiATCC27255
Org3_Gene1837	accessory	762	[RbromiiATCC27255]RBATCC27255_01890_Serine_protease_inhibitor	RbromiiATCC27255
Org3_Gene1838	accessory	1425	[RbromiiATCC27255]RBATCC27255_01891_hypothetical_protein	RbromiiATCC27255
Org3_Gene1839	accessory	1840	[RbromiiATCC27255]RBATCC27255_01892_Regulatory_protein_SoxS	RbromiiATCC27255
Org3_Gene1840	accessory	2025	[RbromiiATCC27255]RBATCC27255_01893_hypothetical_protein	RbromiiATCC27255
Org3_Gene1845	accessory	786	[RbromiiATCC27255]RBATCC27255_01898_Cyclodextrin-binding_protein_precursor	RbromiiATCC27255
Org3_Gene1855	accessory	872	[RbromiiATCC27255]RBATCC27255_01908_hypothetical_protein	RbromiiATCC27255
Org3_Gene1856	accessory	3201	[RbromiiATCC27255]RBATCC27255_01909_PPOX_class_probable_F420-dependent_enzyme	RbromiiATCC27255
Org3_Gene1857	accessory	1471	[RbromiiATCC27255]RBATCC27255_01910_L-asparaginase_1	RbromiiATCC27255
Org3_Gene1858	accessory	856	[RbromiiATCC27255]RBATCC27255_01911_Inner_membrane_protein_YbaL	RbromiiATCC27255
Org3_Gene1859	accessory	2780	[RbromiiATCC27255]RBATCC27255_01912_hypothetical_protein	RbromiiATCC27255
Org3_Gene1863	accessory	2548	[RbromiiATCC27255]RBATCC27255_01916_hypothetical_protein	RbromiiATCC27255
Org3_Gene1867	accessory	2647	[RbromiiATCC27255]RBATCC27255_01920_Dephospho-CoA_kinase	RbromiiATCC27255
Org3_Gene1868	accessory	2624	[RbromiiATCC27255]RBATCC27255_01921_Soluble_lytic_murein_transglycosylase_precursor	RbromiiATCC27255
Org3_Gene1870	accessory	3720	[RbromiiATCC27255]RBATCC27255_01924_Thioredoxin	RbromiiATCC27255
Org3_Gene1871	accessory	1726	[RbromiiATCC27255]RBATCC27255_01925_Thioredoxin_reductase	RbromiiATCC27255
Org3_Gene1872	accessory	1625	[RbromiiATCC27255]RBATCC27255_01926_putative_amino-acid_metabolite_efflux_pump	RbromiiATCC27255
Org3_Gene1874	accessory	653	[RbromiiATCC27255]RBATCC27255_01928_Amino-acid_carrier_protein_AlsT	RbromiiATCC27255
Org3_Gene1875	accessory	2709	[RbromiiATCC27255]RBATCC27255_01929_hypothetical_protein	RbromiiATCC27255
Org3_Gene1878	accessory	1054	[RbromiiATCC27255]RBATCC27255_01932_Putative_L,D-transpeptidase_YciB_precursor	RbromiiATCC27255
Org3_Gene1884	accessory	2121	[RbromiiATCC27255]RBATCC27255_01938_Glycine/sarcosine_N-methyltransferase	RbromiiATCC27255
Org3_Gene1887	accessory	3536	[RbromiiATCC27255]RBATCC27255_01941_HTH-type_transcriptional_repressor_YtrA	RbromiiATCC27255
Org3_Gene1888	accessory	1716	[RbromiiATCC27255]RBATCC27255_01942_ABC_transporter_ATP-binding_protein_YtrB	RbromiiATCC27255
Org3_Gene1890	accessory	2886	[RbromiiATCC27255]RBATCC27255_01944_Prolyl-tRNA_deacylase_ProX	RbromiiATCC27255
Org3_Gene1892	accessory	162	[RbromiiATCC27255]RBATCC27255_01946_Fibronectin_type_III_domain_protein	RbromiiATCC27255
Org3_Gene1893	accessory	2447	[RbromiiATCC27255]RBATCC27255_01947_hypothetical_protein	RbromiiATCC27255
Org3_Gene1898	accessory	1186	[RbromiiATCC27255]RBATCC27255_01952_D-alanyl-D-alanine_carboxypeptidase_DacB_precursor	RbromiiATCC27255
Org3_Gene1900	accessory	2625	[RbromiiATCC27255]RBATCC27255_01954_hypothetical_protein	RbromiiATCC27255
Org3_Gene1903	accessory	2385	[RbromiiATCC27255]RBATCC27255_01957_Zn-dependent_proteases	RbromiiATCC27255
Org3_Gene1912	accessory	3318	[RbromiiATCC27255]RBATCC27255_01966_Protease_synthase_and_sporulation_negative_regulatory_protein_PAI_1	RbromiiATCC27255
Org3_Gene1913	accessory	3313	[RbromiiATCC27255]RBATCC27255_01967_Low_molecular_weight_protein-tyrosine-phosphatase_YwlE	RbromiiATCC27255
Org3_Gene1916	accessory	530	[RbromiiATCC27255]RBATCC27255_01970_Proline_permease	RbromiiATCC27255
Org3_Gene1917	accessory	2772	[RbromiiATCC27255]RBATCC27255_01971_Xanthine_phosphoribosyltransferase	RbromiiATCC27255
Org3_Gene1919	accessory	2616	[RbromiiATCC27255]RBATCC27255_01974_cytidylate_kinase	RbromiiATCC27255
Org3_Gene1920	accessory	923	[RbromiiATCC27255]RBATCC27255_01975_Allantoate_amidohydrolase	RbromiiATCC27255
Org3_Gene1921	accessory	579	[RbromiiATCC27255]RBATCC27255_01976_NAD-dependent_dihydropyrimidine_dehydrogenase_subunit_PreA	RbromiiATCC27255

Org3_Gene1922	accessory	736	[RbromiiATCC27255]RBATCC27255_01977_D-hydantoinase	RbromiiATCC27255
Org3_Gene1923	accessory	1171	[RbromiiATCC27255]RBATCC27255_01978_ABC_transporter,_substrate-binding_protein,_aliphatic_sulfonates_family	RbromiiATCC27255
Org3_Gene1924	accessory	1917	[RbromiiATCC27255]RBATCC27255_01979_ABC-type_anion_transport_system,_duplicated_permease_component	RbromiiATCC27255
Org3_Gene1925	accessory	1900	[RbromiiATCC27255]RBATCC27255_01980_Bicarbonate_transport_system_permease_protein_CmpB	RbromiiATCC27255
Org3_Gene1926	accessory	2032	[RbromiiATCC27255]RBATCC27255_01981_Aliphatic_sulfonates_import_ATP-binding_protein_SsuB	RbromiiATCC27255
Org3_Gene1927	accessory	1135	[RbromiiATCC27255]RBATCC27255_01982_NAD-dependent_methanol_dehydrogenase	RbromiiATCC27255
Org3_Gene1928	accessory	813	[RbromiiATCC27255]RBATCC27255_01983_Taurine--pyruvate_aminotransferase	RbromiiATCC27255
Org3_Gene1929	accessory	1699	[RbromiiATCC27255]RBATCC27255_01984_hypothetical_protein	RbromiiATCC27255
Org3_Gene1930	accessory	2534	[RbromiiATCC27255]RBATCC27255_01985_Response_regulator_ArlR	RbromiiATCC27255
Org3_Gene1931	accessory	646	[RbromiiATCC27255]RBATCC27255_01986_Signal_transduction_histidine-protein_kinase_ArlS	RbromiiATCC27255
Org3_Gene1932	accessory	2117	[RbromiiATCC27255]RBATCC27255_01987_hypothetical_protein	RbromiiATCC27255
Org3_Gene1933	accessory	1420	[RbromiiATCC27255]RBATCC27255_01988_Lipid_II:glycine_glycyltransferase	RbromiiATCC27255
Org3_Gene1934	accessory	1862	[RbromiiATCC27255]RBATCC27255_01989_Error-prone_DNA_polymerase	RbromiiATCC27255
Org3_Gene1937	accessory	4288	[RbromiiATCC27255]RBATCC27255_01992_hypothetical_protein	RbromiiATCC27255
Org3_Gene1938	accessory	4285	[RbromiiATCC27255]RBATCC27255_01993_hypothetical_protein	RbromiiATCC27255
Org3_Gene1939	accessory	3461	[RbromiiATCC27255]RBATCC27255_01994_hypothetical_protein	RbromiiATCC27255
Org3_Gene1940	accessory	1843	[RbromiiATCC27255]RBATCC27255_01995_hypothetical_protein	RbromiiATCC27255
Org3_Gene1941	accessory	41	[RbromiiATCC27255]RBATCC27255_01998_Pullulanase_precursor	RbromiiATCC27255
Org3_Gene1942	accessory	4154	[RbromiiATCC27255]RBATCC27255_01999_hypothetical_protein	RbromiiATCC27255
Org3_Gene2	accessory	275	[RbromiiATCC27255]RBATCC27255_00002_ATP-dependent_DNA_helicase_RecQ	RbromiiATCC27255
Org3_Gene20	accessory	1931	[RbromiiATCC27255]RBATCC27255_00023_transcriptional_activator_RhaS	RbromiiATCC27255
Org3_Gene203	accessory	3211	[RbromiiATCC27255]RBATCC27255_00214_Transposase	RbromiiATCC27255
Org3_Gene209	accessory	2531	[RbromiiATCC27255]RBATCC27255_00221_cAMP_regulatory_protein	RbromiiATCC27255
Org3_Gene21	accessory	1447	[RbromiiATCC27255]RBATCC27255_00024_L-Ala--D-Glu_endopeptidase_precursor	RbromiiATCC27255
Org3_Gene210	accessory	1607	[RbromiiATCC27255]RBATCC27255_00222_Bacterial_Ig-like_domain_(group_2)	RbromiiATCC27255
Org3_Gene211	accessory	1873	[RbromiiATCC27255]RBATCC27255_00223_Carboxylesterase_NlhH	RbromiiATCC27255
Org3_Gene212	accessory	1921	[RbromiiATCC27255]RBATCC27255_00224_Melibiose_operon_regulatory_protein	RbromiiATCC27255
Org3_Gene213	accessory	1999	[RbromiiATCC27255]RBATCC27255_00225_Arabinose_operon_regulatory_protein	RbromiiATCC27255
Org3_Gene214	accessory	1143	[RbromiiATCC27255]RBATCC27255_00226_Cell_wall-binding_protein_YocH_precursor	RbromiiATCC27255
Org3_Gene215	accessory	2852	[RbromiiATCC27255]RBATCC27255_00227_hypothetical_protein	RbromiiATCC27255
Org3_Gene216	accessory	2043	[RbromiiATCC27255]RBATCC27255_00228_hypothetical_protein	RbromiiATCC27255
Org3_Gene219	accessory	2636	[RbromiiATCC27255]RBATCC27255_00231_hypothetical_protein	RbromiiATCC27255
Org3_Gene221	accessory	2358	[RbromiiATCC27255]RBATCC27255_00233_putative_membrane_protein	RbromiiATCC27255
Org3_Gene23	accessory	1121	[RbromiiATCC27255]RBATCC27255_00026_hypothetical_protein	RbromiiATCC27255
Org3_Gene230	accessory	3576	[RbromiiATCC27255]RBATCC27255_00242_putative_protein_encoded_in_hypervariable_junctions_of_pilus_gene_clusters	RbromiiATCC27255
Org3_Gene232	accessory	30	[RbromiiATCC27255]RBATCC27255_00244_Alpha-amylase_precursor	RbromiiATCC27255
Org3_Gene233	accessory	991	[RbromiiATCC27255]RBATCC27255_00245_Signal-transduction_histidine_kinase_senX3	RbromiiATCC27255
Org3_Gene234	accessory	2549	[RbromiiATCC27255]RBATCC27255_00246_Phosphate_regulon_transcriptional_regulatory_protein_PhoB	RbromiiATCC27255
Org3_Gene235	accessory	116	[RbromiiATCC27255]RBATCC27255_00247_ABC_transporter_permease_YtrF_precursor	RbromiiATCC27255
Org3_Gene236	accessory	2465	[RbromiiATCC27255]RBATCC27255_00248_putative_ABC_transporter_ATP-binding_protein/MT1014	RbromiiATCC27255
Org3_Gene240	accessory	2855	[RbromiiATCC27255]RBATCC27255_00252_HSP-70_cofactor	RbromiiATCC27255
Org3_Gene241	accessory	1368	[RbromiiATCC27255]RBATCC27255_00253_Heat-inducible_transcription_repressor_HrcA	RbromiiATCC27255
Org3_Gene242	accessory	3920	[RbromiiATCC27255]RBATCC27255_00254_hypothetical_protein	RbromiiATCC27255
Org3_Gene243	accessory	3998	[RbromiiATCC27255]RBATCC27255_00255_hypothetical_protein	RbromiiATCC27255
Org3_Gene244	accessory	3442	[RbromiiATCC27255]RBATCC27255_00256_Putative_8-oxo-dGTP_diphosphatase_YtkD	RbromiiATCC27255
Org3_Gene247	accessory	2193	[RbromiiATCC27255]RBATCC27255_00259_Sensory_transduction_protein_LytR	RbromiiATCC27255
Org3_Gene248	accessory	548	[RbromiiATCC27255]RBATCC27255_00260_hypothetical_protein	RbromiiATCC27255
Org3_Gene249	accessory	497	[RbromiiATCC27255]RBATCC27255_00261_gliding-associated_putative_ABC_transporter_substrate-binding_component_GldG	RbromiiATCC27255
Org3_Gene25	accessory	740	[RbromiiATCC27255]RBATCC27255_00028_Multidrug_export_protein_MepA	RbromiiATCC27255
Org3_Gene253	accessory	106	[RbromiiATCC27255]RBATCC27255_00265_putative_membrane_protein	RbromiiATCC27255
Org3_Gene255	accessory	2344	[RbromiiATCC27255]RBATCC27255_00267_transcriptional_regulator_FixK	RbromiiATCC27255
Org3_Gene257	accessory	1545	[RbromiiATCC27255]RBATCC27255_00269_L-lactate_dehydrogenase_P	RbromiiATCC27255
Org3_Gene262	accessory	2039	[RbromiiATCC27255]RBATCC27255_00274_General_stress_protein_39	RbromiiATCC27255
Org3_Gene263	accessory	4345	[RbromiiATCC27255]RBATCC27255_00275_hypothetical_protein	RbromiiATCC27255
Org3_Gene264	accessory	1777	[RbromiiATCC27255]RBATCC27255_00276_Dipicolinate_synthase_subunit_A	RbromiiATCC27255
Org3_Gene266	accessory	4177	[RbromiiATCC27255]RBATCC27255_00278_putative_zinc_finger/helix-turn-helix_protein,_YgiT_family	RbromiiATCC27255
Org3_Gene267	accessory	4113	[RbromiiATCC27255]RBATCC27255_00279_HTH-type_transcriptional_regulator_ImmR	RbromiiATCC27255
Org3_Gene269	accessory	2966	[RbromiiATCC27255]RBATCC27255_00281_hypothetical_protein	RbromiiATCC27255
Org3_Gene27	accessory	468	[RbromiiATCC27255]RBATCC27255_00030_triple_tyrosine_motif-containing_protein	RbromiiATCC27255
Org3_Gene276	accessory	2261	[RbromiiATCC27255]RBATCC27255_00288_Stage_II_sporulation_protein_Q	RbromiiATCC27255
Org3_Gene277	accessory	2592	[RbromiiATCC27255]RBATCC27255_00289_cytidylate_kinase	RbromiiATCC27255
Org3_Gene278	accessory	1577	[RbromiiATCC27255]RBATCC27255_00290_hypothetical_protein	RbromiiATCC27255
Org3_Gene279	accessory	2113	[RbromiiATCC27255]RBATCC27255_00291_Sulfite_exporter_TauE/SafE	RbromiiATCC27255
Org3_Gene281	accessory	2742	[RbromiiATCC27255]RBATCC27255_00293_hypothetical_protein	RbromiiATCC27255
Org3_Gene282	accessory	3347	[RbromiiATCC27255]RBATCC27255_00294_hypothetical_protein	RbromiiATCC27255
Org3_Gene283	accessory	2366	[RbromiiATCC27255]RBATCC27255_00295_Serine/threonine-protein_kinase_PknB	RbromiiATCC27255
Org3_Gene284	accessory	3688	[RbromiiATCC27255]RBATCC27255_00296_anaerobic_benzoate_catabolism_transcriptional_regulator	RbromiiATCC27255
Org3_Gene285	accessory	3435	[RbromiiATCC27255]RBATCC27255_00297_hypothetical_protein	RbromiiATCC27255
Org3_Gene286	accessory	2970	[RbromiiATCC27255]RBATCC27255_00298_putative_membrane_protein	RbromiiATCC27255
Org3_Gene287	accessory	2994	[RbromiiATCC27255]RBATCC27255_00299_hypothetical_protein	RbromiiATCC27255
Org3_Gene288	accessory	826	[RbromiiATCC27255]RBATCC27255_00300_putative_membrane_protein	RbromiiATCC27255
Org3_Gene289	accessory	1312	[RbromiiATCC27255]RBATCC27255_00301_hypothetical_protein	RbromiiATCC27255
Org3_Gene29	accessory	373	[RbromiiATCC27255]RBATCC27255_00032_Adenine_deaminase	RbromiiATCC27255
Org3_Gene291	accessory	3109	[RbromiiATCC27255]RBATCC27255_00303_RNA_polymerase-binding_transcription_factor_CarD	RbromiiATCC27255
Org3_Gene293	accessory	273	[RbromiiATCC27255]RBATCC27255_00305_Bacterial_Ig-like_domain_(group_2)	RbromiiATCC27255
Org3_Gene294	accessory	959	[RbromiiATCC27255]RBATCC27255_00306_Beta-hexosaminidase_A_precursor	RbromiiATCC27255
Org3_Gene295	accessory	3179	[RbromiiATCC27255]RBATCC27255_00307_hypothetical_protein	RbromiiATCC27255
Org3_Gene296	accessory	2471	[RbromiiATCC27255]RBATCC27255_00308_Peptidyl-prolyl_cis-trans_isomerase_A_precursor	RbromiiATCC27255
Org3_Gene297	accessory	1891	[RbromiiATCC27255]RBATCC27255_00309_Teichoic_acid_translocation_permease_protein_TagG	RbromiiATCC27255
Org3_Gene298	accessory	901	[RbromiiATCC27255]RBATCC27255_00310_Teichoic_acids_export_ATP-binding_protein_TagH	RbromiiATCC27255
Org3_Gene299	accessory	96	[RbromiiATCC27255]RBATCC27255_00311_Acyltransferase_family_protein	RbromiiATCC27255
Org3_Gene30	accessory	3269	[RbromiiATCC27255]RBATCC27255_00033_hypothetical_protein	RbromiiATCC27255
Org3_Gene300	accessory	507	[RbromiiATCC27255]RBATCC27255_00312_hypothetical_protein	RbromiiATCC27255
Org3_Gene303	accessory	467	[RbromiiATCC27255]RBATCC27255_00315_hypothetical_protein	RbromiiATCC27255
Org3_Gene304	accessory	2074	[RbromiiATCC27255]RBATCC27255_00316_Small-conductance_mechanosensitive_channel	RbromiiATCC27255
Org3_Gene305	accessory	3756	[RbromiiATCC27255]RBATCC27255_00317_hypothetical_protein	RbromiiATCC27255
Org3_Gene307	accessory	2030	[RbromiiATCC27255]RBATCC27255_00319_sigma-E_processing_peptidase_SpoIIGA	RbromiiATCC27255
Org3_Gene31	accessory	2165	[RbromiiATCC27255]RBATCC27255_00034_hypothetical_protein	RbromiiATCC27255
Org3_Gene310	accessory	1497	[RbromiiATCC27255]RBATCC27255_00322_hypothetical_protein	RbromiiATCC27255
Org3_Gene313	accessory	2829	[RbromiiATCC27255]RBATCC27255_00325_HAD_hydrolase,_family_IB	RbromiiATCC27255
Org3_Gene317	accessory	1939	[RbromiiATCC27255]RBATCC27255_00329_Phosphatidate_cytidylyltransferase	RbromiiATCC27255
Org3_Gene319	accessory	708	[RbromiiATCC27255]RBATCC27255_00331_Zinc_metalloprotease_RasP	RbromiiATCC27255
Org3_Gene32	accessory	2172	[RbromiiATCC27255]RBATCC27255_00035_hypothetical_protein	RbromiiATCC27255
Org3_Gene322	accessory	827	[RbromiiATCC27255]RBATCC27255_00334_pheromone_autoinducer_2_transporter	RbromiiATCC27255
Org3_Gene33	accessory	2402	[RbromiiATCC27255]RBATCC27255_00036_hypothetical_protein	RbromiiATCC27255
Org3_Gene331	accessory	4057	[RbromiiATCC27255]RBATCC27255_00343_hypothetical_protein	RbromiiATCC27255
Org3_Gene333	accessory	965	[RbromiiATCC27255]RBATCC27255_00345_hypothetical_protein	RbromiiATCC27255
Org3_Gene334	accessory	3938	[RbromiiATCC27255]RBATCC27255_00346_hypothetical_protein	RbromiiATCC27255
Org3_Gene335	accessory	1779	[RbromiiATCC27255]RBATCC27255_00347_Ribosomal_large_subunit_pseudouridine_synthase_D	RbromiiATCC27255
Org3_Gene336	accessory	414	[RbromiiATCC27255]RBATCC27255_00348_hypothetical_protein	RbromiiATCC27255
Org3_Gene337	accessory	150	[RbromiiATCC27255]RBATCC27255_00349_Serine/threonine-protein_kinase_StkP	RbromiiATCC27255
Org3_Gene338	accessory	3480	[RbromiiATCC27255]RBATCC27255_00350_putative_membrane_protein	RbromiiATCC27255
Org3_Gene339	accessory	3360	[RbromiiATCC27255]RBATCC27255_00351_preprotein_translocase_subunit_YajC	RbromiiATCC27255
Org3_Gene34	accessory	2621	[RbromiiATCC27255]RBATCC27255_00037_putative_ABC_transporter_ATP-binding_protein_YxlF	RbromiiATCC27255
Org3_Gene345	accessory	4103	[RbromiiATCC27255]RBATCC27255_00357_hypothetical_protein	RbromiiATCC27255
Org3_Gene346	accessory	1106	[RbromiiATCC27255]RBATCC27255_00358_putative_cysteine_desulfurase	RbromiiATCC27255
Org3_Gene348	accessory	925	[RbromiiATCC27255]RBATCC27255_00360_YbbR-like_protein	RbromiiATCC27255
Org3_Gene349	accessory	1095	[RbromiiATCC27255]RBATCC27255_00361_Cell_division_protein_FtsW	RbromiiATCC27255
Org3_Gene35	accessory	1432	[RbromiiATCC27255]RBATCC27255_00038_hypothetical_protein	RbromiiATCC27255
Org3_Gene352	accessory	2337	[RbromiiATCC27255]RBATCC27255_00364_t(6)A37_threonylcarbamoyladenosine_biosynthesis_protein	RbromiiATCC27255
Org3_Gene355	accessory	655	[RbromiiATCC27255]RBATCC27255_00367_Divergent_AAA_domain_protein	RbromiiATCC27255
Org3_Gene356	accessory	1996	[RbromiiATCC27255]RBATCC27255_00369_hypothetical_protein	RbromiiATCC27255
Org3_Gene357	accessory	3307	[RbromiiATCC27255]RBATCC27255_00370_Cell_wall_hydrolase_CwlJ	RbromiiATCC27255
Org3_Gene358	accessory	2343	[RbromiiATCC27255]RBATCC27255_00371_hypothetical_protein	RbromiiATCC27255
Org3_Gene359	accessory	1690	[RbromiiATCC27255]RBATCC27255_00372_hypothetical_protein	RbromiiATCC27255
Org3_Gene360	accessory	1229	[RbromiiATCC27255]RBATCC27255_00373_Oxygen-independent_coproporphyrinogen-III_oxidase_1	RbromiiATCC27255
Org3_Gene362	accessory	686	[RbromiiATCC27255]RBATCC27255_00375_Arginine_decarboxylase	RbromiiATCC27255
Org3_Gene365	accessory	2009	[RbromiiATCC27255]RBATCC27255_00378_ribonuclease_Z	RbromiiATCC27255
Org3_Gene367	accessory	4072	[RbromiiATCC27255]RBATCC27255_00380_hypothetical_protein	RbromiiATCC27255
Org3_Gene368	accessory	326	[RbromiiATCC27255]RBATCC27255_00381_Dockerin_type_I_repeat	RbromiiATCC27255
Org3_Gene369	accessory	1565	[RbromiiATCC27255]RBATCC27255_00382_hypothetical_protein	RbromiiATCC27255
Org3_Gene37	accessory	1614	[RbromiiATCC27255]RBATCC27255_00040_hypothetical_protein	RbromiiATCC27255

Org3_Gene370	accessory	1732	[RbromiiATCC27255]RBATCC27255_00383_Energy-coupling_factor_transporter_transmembrane_protein_EcfT	RbromiiATCC27255
Org3_Gene371	accessory	114	[RbromiiATCC27255]RBATCC27255_00384_Putative_HMP/thiamine_import_ATP-binding_protein_YkoD	RbromiiATCC27255
Org3_Gene372	accessory	679	[RbromiiATCC27255]RBATCC27255_00385_Amidophosphoribosyltransferase_precursor	RbromiiATCC27255
Org3_Gene374	accessory	2333	[RbromiiATCC27255]RBATCC27255_00387_N-acetylmuramoyl-L-alanine_amidase_AmiC_precursor	RbromiiATCC27255
Org3_Gene376	accessory	2396	[RbromiiATCC27255]RBATCC27255_00389_hypothetical_protein	RbromiiATCC27255
Org3_Gene379	accessory	2753	[RbromiiATCC27255]RBATCC27255_00392_hypothetical_protein	RbromiiATCC27255
Org3_Gene384	accessory	2978	[RbromiiATCC27255]RBATCC27255_00397_hypothetical_protein	RbromiiATCC27255
Org3_Gene385	accessory	2042	[RbromiiATCC27255]RBATCC27255_00398_CAAX_amino_terminal_protease_self-_immunity	RbromiiATCC27255
Org3_Gene386	accessory	3168	[RbromiiATCC27255]RBATCC27255_00399_hypothetical_protein	RbromiiATCC27255
Org3_Gene387	accessory	1758	[RbromiiATCC27255]RBATCC27255_00400_hypothetical_protein	RbromiiATCC27255
Org3_Gene388	accessory	3433	[RbromiiATCC27255]RBATCC27255_00401_hypothetical_protein	RbromiiATCC27255
Org3_Gene392	accessory	2708	[RbromiiATCC27255]RBATCC27255_00405_hypothetical_protein	RbromiiATCC27255
Org3_Gene393	accessory	3972	[RbromiiATCC27255]RBATCC27255_00406_pro-sigmaK_processing_inhibitor_BofA	RbromiiATCC27255
Org3_Gene402	accessory	1790	[RbromiiATCC27255]RBATCC27255_00415_Radical_SAM_superfamily_protein	RbromiiATCC27255
Org3_Gene403	accessory	2915	[RbromiiATCC27255]RBATCC27255_00416_hypothetical_protein	RbromiiATCC27255
Org3_Gene404	accessory	2541	[RbromiiATCC27255]RBATCC27255_00417_hypothetical_protein	RbromiiATCC27255
Org3_Gene405	accessory	1547	[RbromiiATCC27255]RBATCC27255_00418_putative_integral_membrane_protein	RbromiiATCC27255
Org3_Gene406	accessory	2477	[RbromiiATCC27255]RBATCC27255_00419_stage_II_sporulation_protein_M	RbromiiATCC27255
Org3_Gene408	accessory	138	[RbromiiATCC27255]RBATCC27255_00421_invasion_associated_secreted_endopeptidase	RbromiiATCC27255
Org3_Gene409	accessory	2699	[RbromiiATCC27255]RBATCC27255_00422_hypothetical_protein	RbromiiATCC27255
Org3_Gene41	accessory	3803	[RbromiiATCC27255]RBATCC27255_00045_Thioredoxin-M	RbromiiATCC27255
Org3_Gene411	accessory	1817	[RbromiiATCC27255]RBATCC27255_00424_hypothetical_protein	RbromiiATCC27255
Org3_Gene412	accessory	2091	[RbromiiATCC27255]RBATCC27255_00426_hypothetical_protein	RbromiiATCC27255
Org3_Gene413	accessory	1687	[RbromiiATCC27255]RBATCC27255_00427_radical_SAM_protein	RbromiiATCC27255
Org3_Gene414	accessory	2429	[RbromiiATCC27255]RBATCC27255_00428_hypothetical_protein	RbromiiATCC27255
Org3_Gene417	accessory	3057	[RbromiiATCC27255]RBATCC27255_00431_Zn-dependent_proteases	RbromiiATCC27255
Org3_Gene418	accessory	3520	[RbromiiATCC27255]RBATCC27255_00432_hypothetical_protein	RbromiiATCC27255
Org3_Gene42	accessory	3187	[RbromiiATCC27255]RBATCC27255_00046_Terminase_small_subunit	RbromiiATCC27255
Org3_Gene420	accessory	3182	[RbromiiATCC27255]RBATCC27255_00434_putative_integral_membrane_protein	RbromiiATCC27255
Org3_Gene421	accessory	3152	[RbromiiATCC27255]RBATCC27255_00435_putative_membrane_protein	RbromiiATCC27255
Org3_Gene422	accessory	966	[RbromiiATCC27255]RBATCC27255_00436_mercuric_reductase	RbromiiATCC27255
Org3_Gene424	accessory	119	[RbromiiATCC27255]RBATCC27255_00438_Alpha-amylase_precursor	RbromiiATCC27255
Org3_Gene426	accessory	560	[RbromiiATCC27255]RBATCC27255_00441_Arginine_transport_system_permease_protein_ArtQ	RbromiiATCC27255
Org3_Gene428	accessory	768	[RbromiiATCC27255]RBATCC27255_00443_CCA-adding_enzyme	RbromiiATCC27255
Org3_Gene429	accessory	167	[RbromiiATCC27255]RBATCC27255_00444_putative_cation-transporting_ATPase_F	RbromiiATCC27255
Org3_Gene430	accessory	1382	[RbromiiATCC27255]RBATCC27255_00445_SpoIVB_peptidase_precursor	RbromiiATCC27255
Org3_Gene439	accessory	571	[RbromiiATCC27255]RBATCC27255_00454_putative_membrane_protein	RbromiiATCC27255
Org3_Gene44	accessory	1126	[RbromiiATCC27255]RBATCC27255_00048_hypothetical_protein	RbromiiATCC27255
Org3_Gene441	accessory	2957	[RbromiiATCC27255]RBATCC27255_00456_hypothetical_protein	RbromiiATCC27255
Org3_Gene442	accessory	2437	[RbromiiATCC27255]RBATCC27255_00457_hypothetical_protein	RbromiiATCC27255
Org3_Gene446	accessory	1208	[RbromiiATCC27255]RBATCC27255_00461_hypothetical_protein	RbromiiATCC27255
Org3_Gene447	accessory	357	[RbromiiATCC27255]RBATCC27255_00462_ATP-dependent_DNA_helicase_RecQ	RbromiiATCC27255
Org3_Gene448	accessory	3546	[RbromiiATCC27255]RBATCC27255_00463_hypothetical_protein	RbromiiATCC27255
Org3_Gene449	accessory	3506	[RbromiiATCC27255]RBATCC27255_00464_hypothetical_protein	RbromiiATCC27255
Org3_Gene45	accessory	1431	[RbromiiATCC27255]RBATCC27255_00049_hypothetical_protein	RbromiiATCC27255
Org3_Gene450	accessory	1086	[RbromiiATCC27255]RBATCC27255_00465_hypothetical_protein	RbromiiATCC27255
Org3_Gene451	accessory	534	[RbromiiATCC27255]RBATCC27255_00466_hypothetical_protein	RbromiiATCC27255
Org3_Gene452	accessory	2754	[RbromiiATCC27255]RBATCC27255_00467_hypothetical_protein	RbromiiATCC27255
Org3_Gene453	accessory	3507	[RbromiiATCC27255]RBATCC27255_00468_hypothetical_protein	RbromiiATCC27255
Org3_Gene454	accessory	3446	[RbromiiATCC27255]RBATCC27255_00469_hypothetical_protein	RbromiiATCC27255
Org3_Gene455	accessory	2580	[RbromiiATCC27255]RBATCC27255_00470_Response_regulator_protein_VraR	RbromiiATCC27255
Org3_Gene456	accessory	847	[RbromiiATCC27255]RBATCC27255_00471_sensory_histidine_kinase_UhpB	RbromiiATCC27255
Org3_Gene457	accessory	454	[RbromiiATCC27255]RBATCC27255_00472_hypothetical_protein	RbromiiATCC27255
Org3_Gene46	accessory	3684	[RbromiiATCC27255]RBATCC27255_00050_hypothetical_protein	RbromiiATCC27255
Org3_Gene461	accessory	395	[RbromiiATCC27255]RBATCC27255_00476_DNA_polymerase_III_subunit_gamma/tau	RbromiiATCC27255
Org3_Gene462	accessory	713	[RbromiiATCC27255]RBATCC27255_00479_dihydrolipoamide_dehydrogenase	RbromiiATCC27255
Org3_Gene464	accessory	4123	[RbromiiATCC27255]RBATCC27255_00482_hypothetical_protein	RbromiiATCC27255
Org3_Gene465	accessory	2667	[RbromiiATCC27255]RBATCC27255_00483_Undecaprenyl-diphosphatase_BcrC	RbromiiATCC27255
Org3_Gene467	accessory	2697	[RbromiiATCC27255]RBATCC27255_00485_Putative_acetyltransferase	RbromiiATCC27255
Org3_Gene468	accessory	4251	[RbromiiATCC27255]RBATCC27255_00486_hypothetical_protein	RbromiiATCC27255
Org3_Gene470	accessory	3287	[RbromiiATCC27255]RBATCC27255_00488_hypothetical_protein	RbromiiATCC27255
Org3_Gene471	accessory	4130	[RbromiiATCC27255]RBATCC27255_00489_hypothetical_protein	RbromiiATCC27255
Org3_Gene472	accessory	3696	[RbromiiATCC27255]RBATCC27255_00490_hypothetical_protein	RbromiiATCC27255
Org3_Gene473	accessory	3105	[RbromiiATCC27255]RBATCC27255_00491_Putative_phosphinothricin_acetyltransferase_YwnH	RbromiiATCC27255
Org3_Gene478	accessory	2862	[RbromiiATCC27255]RBATCC27255_00497_hypothetical_protein	RbromiiATCC27255
Org3_Gene479	accessory	1531	[RbromiiATCC27255]RBATCC27255_00498_Regulatory_protein_SoxS	RbromiiATCC27255
Org3_Gene48	accessory	3620	[RbromiiATCC27255]RBATCC27255_00052_hypothetical_protein	RbromiiATCC27255
Org3_Gene480	accessory	3556	[RbromiiATCC27255]RBATCC27255_00499_copper_ion_binding_protein	RbromiiATCC27255
Org3_Gene481	accessory	4335	[RbromiiATCC27255]RBATCC27255_00500_Virus_attachment_protein_p12_family_protein	RbromiiATCC27255
Org3_Gene486	accessory	2700	[RbromiiATCC27255]RBATCC27255_00505_Alpha/beta_hydrolase_family_protein	RbromiiATCC27255
Org3_Gene489	accessory	1933	[RbromiiATCC27255]RBATCC27255_00509_Thiamine_ECF_transporter_S_component_ThiT	RbromiiATCC27255
Org3_Gene49	accessory	3603	[RbromiiATCC27255]RBATCC27255_00053_hypothetical_protein	RbromiiATCC27255
Org3_Gene490	accessory	4215	[RbromiiATCC27255]RBATCC27255_00510_Spore_coat_associated_protein_JA_(CotJA)	RbromiiATCC27255
Org3_Gene491	accessory	3963	[RbromiiATCC27255]RBATCC27255_00511_CotJB_protein	RbromiiATCC27255
Org3_Gene493	accessory	1741	[RbromiiATCC27255]RBATCC27255_00513_Cyn_operon_transcriptional_activator	RbromiiATCC27255
Org3_Gene494	accessory	789	[RbromiiATCC27255]RBATCC27255_00514_Na+-dependent_transporters_of_the_SNF_family_protein	RbromiiATCC27255
Org3_Gene495	accessory	3151	[RbromiiATCC27255]RBATCC27255_00515_putative_acetyltransferase	RbromiiATCC27255
Org3_Gene496	accessory	1608	[RbromiiATCC27255]RBATCC27255_00516_Serine_acetyltransferase	RbromiiATCC27255
Org3_Gene497	accessory	2837	[RbromiiATCC27255]RBATCC27255_00517_Septum_formation_protein_Maf	RbromiiATCC27255
Org3_Gene498	accessory	878	[RbromiiATCC27255]RBATCC27255_00518_Putative_metal_chaperone_YciC	RbromiiATCC27255
Org3_Gene499	accessory	3499	[RbromiiATCC27255]RBATCC27255_00519_hypothetical_protein	RbromiiATCC27255
Org3_Gene50	accessory	3144	[RbromiiATCC27255]RBATCC27255_00054_hypothetical_protein	RbromiiATCC27255
Org3_Gene502	accessory	2002	[RbromiiATCC27255]RBATCC27255_00522_Sulfate_starvation-induced_protein_7	RbromiiATCC27255
Org3_Gene503	accessory	1025	[RbromiiATCC27255]RBATCC27255_00523_hypothetical_protein	RbromiiATCC27255
Org3_Gene506	accessory	944	[RbromiiATCC27255]RBATCC27255_00526_UDP-glucose_6-dehydrogenase	RbromiiATCC27255
Org3_Gene51	accessory	3579	[RbromiiATCC27255]RBATCC27255_00055_hypothetical_protein	RbromiiATCC27255
Org3_Gene518	accessory	1645	[RbromiiATCC27255]RBATCC27255_00538_Hyaluronan_synthase	RbromiiATCC27255
Org3_Gene52	accessory	4389	[RbromiiATCC27255]RBATCC27255_00056_hypothetical_protein	RbromiiATCC27255
Org3_Gene520	accessory	1248	[RbromiiATCC27255]RBATCC27255_00540_Putative_glycosyltransferase_EpsF	RbromiiATCC27255
Org3_Gene521	accessory	1138	[RbromiiATCC27255]RBATCC27255_00541_Transmembrane_protein_EpsG	RbromiiATCC27255
Org3_Gene522	accessory	1241	[RbromiiATCC27255]RBATCC27255_00542_N,N'-diacetylbacillosaminyl-diphospho-undecaprenol_alpha-1,3-N-acetylgalactosaminyltransferase	RbromiiATCC27255
Org3_Gene523	accessory	2604	[RbromiiATCC27255]RBATCC27255_00543_putative_sugar_transferase_EpsL	RbromiiATCC27255
Org3_Gene53	accessory	3009	[RbromiiATCC27255]RBATCC27255_00057_Mu-like_prophage_DNA_circulation_protein	RbromiiATCC27255
Org3_Gene530	accessory	3544	[RbromiiATCC27255]RBATCC27255_00550_hypothetical_protein	RbromiiATCC27255
Org3_Gene54	accessory	1534	[RbromiiATCC27255]RBATCC27255_00058_Mu-like_prophage_tail_protein_gpP	RbromiiATCC27255
Org3_Gene544	accessory	1585	[RbromiiATCC27255]RBATCC27255_00564_Tyrosine_recombinase_XerC	RbromiiATCC27255
Org3_Gene545	accessory	1829	[RbromiiATCC27255]RBATCC27255_00565_DNA-damage-inducible_protein_D	RbromiiATCC27255
Org3_Gene546	accessory	524	[RbromiiATCC27255]RBATCC27255_00566_Transposase	RbromiiATCC27255
Org3_Gene547	accessory	4306	[RbromiiATCC27255]RBATCC27255_00567_hypothetical_protein	RbromiiATCC27255
Org3_Gene548	accessory	4417	[RbromiiATCC27255]RBATCC27255_00568_hypothetical_protein	RbromiiATCC27255
Org3_Gene549	accessory	3172	[RbromiiATCC27255]RBATCC27255_00569_RNA_polymerase_factor_sigma-70	RbromiiATCC27255
Org3_Gene55	accessory	3726	[RbromiiATCC27255]RBATCC27255_00059_Mu-like_prophage_protein_gp45	RbromiiATCC27255
Org3_Gene56	accessory	3727	[RbromiiATCC27255]RBATCC27255_00060_hypothetical_protein	RbromiiATCC27255
Org3_Gene569	accessory	2449	[RbromiiATCC27255]RBATCC27255_00589_hypothetical_protein	RbromiiATCC27255
Org3_Gene57	accessory	1302	[RbromiiATCC27255]RBATCC27255_00061_baseplate_assembly_protein	RbromiiATCC27255
Org3_Gene570	accessory	584	[RbromiiATCC27255]RBATCC27255_00590_Cell_wall-associated_polypeptide_CWBP200	RbromiiATCC27255
Org3_Gene574	accessory	285	[RbromiiATCC27255]RBATCC27255_00594_hypothetical_protein	RbromiiATCC27255
Org3_Gene575	accessory	897	[RbromiiATCC27255]RBATCC27255_00595_corrinoid_ABC_transporter_substrate-binding_protein	RbromiiATCC27255
Org3_Gene578	accessory	4146	[RbromiiATCC27255]RBATCC27255_00599_hypothetical_protein	RbromiiATCC27255
Org3_Gene579	accessory	348	[RbromiiATCC27255]RBATCC27255_00600_hypothetical_protein	RbromiiATCC27255
Org3_Gene58	accessory	3005	[RbromiiATCC27255]RBATCC27255_00062_hypothetical_protein	RbromiiATCC27255
Org3_Gene580	accessory	2180	[RbromiiATCC27255]RBATCC27255_00601_Pyruvate_formate-lyase-activating_enzyme	RbromiiATCC27255
Org3_Gene582	accessory	3085	[RbromiiATCC27255]RBATCC27255_00603_putative_HD_superfamily_hydrolase	RbromiiATCC27255
Org3_Gene586	accessory	1882	[RbromiiATCC27255]RBATCC27255_00607_phosphodiesterase_YaeI	RbromiiATCC27255
Org3_Gene588	accessory	3382	[RbromiiATCC27255]RBATCC27255_00609_hypothetical_protein	RbromiiATCC27255
Org3_Gene589	accessory	295	[RbromiiATCC27255]RBATCC27255_00610_sortase,_SrtB_family	RbromiiATCC27255
Org3_Gene59	accessory	1710	[RbromiiATCC27255]RBATCC27255_00063_hypothetical_protein	RbromiiATCC27255
Org3_Gene593	accessory	2160	[RbromiiATCC27255]RBATCC27255_00614_HTH-type_transcriptional_regulator_ImmR	RbromiiATCC27255
Org3_Gene595	accessory	198	[RbromiiATCC27255]RBATCC27255_00616_Beta/alpha-amylase_precursor	RbromiiATCC27255
Org3_Gene597	accessory	2216	[RbromiiATCC27255]RBATCC27255_00618_Signal_peptidase_I_W	RbromiiATCC27255
Org3_Gene598	accessory	948	[RbromiiATCC27255]RBATCC27255_00619_Bacterial_Ig-like_domain_(group_2)	RbromiiATCC27255
Org3_Gene599	accessory	67	[RbromiiATCC27255]RBATCC27255_00620_repeat	RbromiiATCC27255

Org3_Gene60	accessory	1072	[RbromiiATCC27255]RBATCC27255_00064_Ferrous-iron_efflux_pump_FieF	RbromiiATCC27255
Org3_Gene600	accessory	314	[RbromiiATCC27255]RBATCC27255_00621_hypothetical_protein	RbromiiATCC27255
Org3_Gene601	accessory	1423	[RbromiiATCC27255]RBATCC27255_00622_hypothetical_protein	RbromiiATCC27255
Org3_Gene602	accessory	130	[RbromiiATCC27255]RBATCC27255_00623_chromosome_segregation_protein	RbromiiATCC27255
Org3_Gene603	accessory	552	[RbromiiATCC27255]RBATCC27255_00624_hypothetical_protein	RbromiiATCC27255
Org3_Gene604	accessory	2212	[RbromiiATCC27255]RBATCC27255_00625_Signal_peptidase_I_W	RbromiiATCC27255
Org3_Gene605	accessory	1962	[RbromiiATCC27255]RBATCC27255_00626_hypothetical_protein	RbromiiATCC27255
Org3_Gene606	accessory	2017	[RbromiiATCC27255]RBATCC27255_00627_hypothetical_protein	RbromiiATCC27255
Org3_Gene607	accessory	1981	[RbromiiATCC27255]RBATCC27255_00628_hypothetical_protein	RbromiiATCC27255
Org3_Gene609	accessory	2715	[RbromiiATCC27255]RBATCC27255_00630_LOG_family_protein_YvdD	RbromiiATCC27255
Org3_Gene61	accessory	3012	[RbromiiATCC27255]RBATCC27255_00065_Cob(I)yrinic_acid_a,c-diamide_adenosyltransferase	RbromiiATCC27255
Org3_Gene610	accessory	678	[RbromiiATCC27255]RBATCC27255_00631_Na(+)/drug_antiporter	RbromiiATCC27255
Org3_Gene611	accessory	1990	[RbromiiATCC27255]RBATCC27255_00632_Nucleotidyltransferase_domain_protein	RbromiiATCC27255
Org3_Gene612	accessory	1600	[RbromiiATCC27255]RBATCC27255_00633_Riboflavin_biosynthesis_protein_RibF	RbromiiATCC27255
Org3_Gene614	accessory	1729	[RbromiiATCC27255]RBATCC27255_00635_Gamma-D-glutamyl-L-diamino_acid_endopeptidase_1	RbromiiATCC27255
Org3_Gene616	accessory	3242	[RbromiiATCC27255]RBATCC27255_00637_C_GCAxxG_C_C_family_protein	RbromiiATCC27255
Org3_Gene618	accessory	3405	[RbromiiATCC27255]RBATCC27255_00639_hypothetical_protein	RbromiiATCC27255
Org3_Gene622	accessory	2783	[RbromiiATCC27255]RBATCC27255_00643_Putative_beta-lactamase_HcpC_precursor	RbromiiATCC27255
Org3_Gene623	accessory	2571	[RbromiiATCC27255]RBATCC27255_00644_hypothetical_protein	RbromiiATCC27255
Org3_Gene624	accessory	2713	[RbromiiATCC27255]RBATCC27255_00645_Ion_channel	RbromiiATCC27255
Org3_Gene626	accessory	903	[RbromiiATCC27255]RBATCC27255_00647_Transposase	RbromiiATCC27255
Org3_Gene629	accessory	1850	[RbromiiATCC27255]RBATCC27255_00650_hypothetical_protein	RbromiiATCC27255
Org3_Gene630	accessory	1144	[RbromiiATCC27255]RBATCC27255_00651_acidobacterial_duplicated_orphan_permease	RbromiiATCC27255
Org3_Gene631	accessory	3364	[RbromiiATCC27255]RBATCC27255_00652_hypothetical_protein	RbromiiATCC27255
Org3_Gene632	accessory	1912	[RbromiiATCC27255]RBATCC27255_00653_Lactose_transport_system_permease_protein_LacG	RbromiiATCC27255
Org3_Gene633	accessory	1830	[RbromiiATCC27255]RBATCC27255_00654_L-arabinose_transport_system_permease_protein_AraP	RbromiiATCC27255
Org3_Gene634	accessory	776	[RbromiiATCC27255]RBATCC27255_00655_Maltose-binding_periplasmic_proteins/domains	RbromiiATCC27255
Org3_Gene635	accessory	799	[RbromiiATCC27255]RBATCC27255_00656_Maltose-binding_periplasmic_proteins/domains	RbromiiATCC27255
Org3_Gene640	accessory	2721	[RbromiiATCC27255]RBATCC27255_00661_putative_accessory_gene_regulator_protein	RbromiiATCC27255
Org3_Gene641	accessory	4379	[RbromiiATCC27255]RBATCC27255_00662_hypothetical_protein	RbromiiATCC27255
Org3_Gene642	accessory	2349	[RbromiiATCC27255]RBATCC27255_00663_Sensory_transduction_protein_LytR	RbromiiATCC27255
Org3_Gene643	accessory	850	[RbromiiATCC27255]RBATCC27255_00664_hypothetical_protein	RbromiiATCC27255
Org3_Gene649	accessory	2585	[RbromiiATCC27255]RBATCC27255_00670_phosphoglycolate_phosphatase	RbromiiATCC27255
Org3_Gene656	accessory	3414	[RbromiiATCC27255]RBATCC27255_00677_hypothetical_protein	RbromiiATCC27255
Org3_Gene670	accessory	4228	[RbromiiATCC27255]RBATCC27255_00691_DNA_binding_domain,_excisionase_family	RbromiiATCC27255
Org3_Gene676	accessory	1029	[RbromiiATCC27255]RBATCC27255_00697_Integrase	RbromiiATCC27255
Org3_Gene677	accessory	2378	[RbromiiATCC27255]RBATCC27255_00698_KDP_operon_transcriptional_regulatory_protein_KdpE	RbromiiATCC27255
Org3_Gene678	accessory	286	[RbromiiATCC27255]RBATCC27255_00699_Sensor_protein_KdpD	RbromiiATCC27255
Org3_Gene679	accessory	4010	[RbromiiATCC27255]RBATCC27255_00700_hypothetical_protein	RbromiiATCC27255
Org3_Gene680	accessory	2553	[RbromiiATCC27255]RBATCC27255_00701_Ktr_system_potassium_uptake_protein_A	RbromiiATCC27255
Org3_Gene681	accessory	788	[RbromiiATCC27255]RBATCC27255_00702_Ktr_system_potassium_uptake_protein_B	RbromiiATCC27255
Org3_Gene682	accessory	1911	[RbromiiATCC27255]RBATCC27255_00703_putative_membrane_protein	RbromiiATCC27255
Org3_Gene683	accessory	1967	[RbromiiATCC27255]RBATCC27255_00705_Hydroxyethylthiazole_kinase	RbromiiATCC27255
Org3_Gene684	accessory	2565	[RbromiiATCC27255]RBATCC27255_00706_Thiamine-phosphate_synthase	RbromiiATCC27255
Org3_Gene685	accessory	2498	[RbromiiATCC27255]RBATCC27255_00707_Phosphorylated_carbohydrates_phosphatase	RbromiiATCC27255
Org3_Gene686	accessory	1982	[RbromiiATCC27255]RBATCC27255_00708_Hydroxymethylpyrimidine/phosphomethylpyrimidine_kinase	RbromiiATCC27255
Org3_Gene687	accessory	1032	[RbromiiATCC27255]RBATCC27255_00709_Cytosine_permease	RbromiiATCC27255
Org3_Gene688	accessory	688	[RbromiiATCC27255]RBATCC27255_00710_putative_ATPase_(AAA+_superfamily)	RbromiiATCC27255
Org3_Gene689	accessory	208	[RbromiiATCC27255]RBATCC27255_00711_Stage_V_sporulation_protein_T	RbromiiATCC27255
Org3_Gene690	accessory	1246	[RbromiiATCC27255]RBATCC27255_00712_hypothetical_protein	RbromiiATCC27255
Org3_Gene691	accessory	3063	[RbromiiATCC27255]RBATCC27255_00713_nicotinamidase/pyrazinamidase	RbromiiATCC27255
Org3_Gene697	accessory	185	[RbromiiATCC27255]RBATCC27255_00719_Cadmium,_zinc_and_cobalt-transporting_ATPase	RbromiiATCC27255
Org3_Gene698	accessory	947	[RbromiiATCC27255]RBATCC27255_00720_hypothetical_protein	RbromiiATCC27255
Org3_Gene702	accessory	351	[RbromiiATCC27255]RBATCC27255_00724_Fibronectin_type_III_domain_protein	RbromiiATCC27255
Org3_Gene703	accessory	1112	[RbromiiATCC27255]RBATCC27255_00725_hypothetical_protein	RbromiiATCC27255
Org3_Gene704	accessory	417	[RbromiiATCC27255]RBATCC27255_00726_Sensor_protein_QseC	RbromiiATCC27255
Org3_Gene705	accessory	2433	[RbromiiATCC27255]RBATCC27255_00727_Staphylococcal_respiratory_response_protein_A	RbromiiATCC27255
Org3_Gene706	accessory	3462	[RbromiiATCC27255]RBATCC27255_00728_putative_manganese-dependent_inorganic_pyrophosphatase	RbromiiATCC27255
Org3_Gene707	accessory	3229	[RbromiiATCC27255]RBATCC27255_00729_hypothetical_protein	RbromiiATCC27255
Org3_Gene708	accessory	2951	[RbromiiATCC27255]RBATCC27255_00730_serine/threonine_protein_kinase	RbromiiATCC27255
Org3_Gene709	accessory	1140	[RbromiiATCC27255]RBATCC27255_00731_hypothetical_protein	RbromiiATCC27255
Org3_Gene712	accessory	509	[RbromiiATCC27255]RBATCC27255_00734_hypothetical_protein	RbromiiATCC27255
Org3_Gene713	accessory	2321	[RbromiiATCC27255]RBATCC27255_00735_ABC-type_transporter_ATP-binding_protein_EcsA	RbromiiATCC27255
Org3_Gene714	accessory	2554	[RbromiiATCC27255]RBATCC27255_00736_hemolysin	RbromiiATCC27255
Org3_Gene715	accessory	369	[RbromiiATCC27255]RBATCC27255_00737_PhoU-like_phosphate_uptake_regulator	RbromiiATCC27255
Org3_Gene716	accessory	466	[RbromiiATCC27255]RBATCC27255_00738_Alkaline_phosphatase_synthesis_sensor_protein_PhoR	RbromiiATCC27255
Org3_Gene717	accessory	2472	[RbromiiATCC27255]RBATCC27255_00739_Sensory_transduction_protein_regX3	RbromiiATCC27255
Org3_Gene718	accessory	2551	[RbromiiATCC27255]RBATCC27255_00740_Phosphate_uptake_regulator	RbromiiATCC27255
Org3_Gene720	accessory	1612	[RbromiiATCC27255]RBATCC27255_00742_Phosphate_transport_system_permease_protein_PstA	RbromiiATCC27255
Org3_Gene721	accessory	1802	[RbromiiATCC27255]RBATCC27255_00743_Phosphate_transport_system_permease_protein_PstC	RbromiiATCC27255
Org3_Gene722	accessory	1642	[RbromiiATCC27255]RBATCC27255_00744_Phosphate-binding_protein_PstS_2_precursor	RbromiiATCC27255
Org3_Gene724	accessory	3369	[RbromiiATCC27255]RBATCC27255_00746_hypothetical_protein	RbromiiATCC27255
Org3_Gene729	accessory	57	[RbromiiATCC27255]RBATCC27255_00751_ABC_transporter_permease_YtrF_precursor	RbromiiATCC27255
Org3_Gene730	accessory	2375	[RbromiiATCC27255]RBATCC27255_00752_putative_ABC_transporter_ATP-binding_protein/MT1014	RbromiiATCC27255
Org3_Gene733	accessory	2099	[RbromiiATCC27255]RBATCC27255_00755_Endo-1,4-beta-xylanase_Z_precursor	RbromiiATCC27255
Org3_Gene741	accessory	3989	[RbromiiATCC27255]RBATCC27255_00764_Preprotein_translocase_subunit_SecE	RbromiiATCC27255
Org3_Gene747	accessory	1765	[RbromiiATCC27255]RBATCC27255_00770_hypothetical_protein	RbromiiATCC27255
Org3_Gene752	accessory	140	[RbromiiATCC27255]RBATCC27255_00775_Penicillin-binding_protein_F	RbromiiATCC27255
Org3_Gene753	accessory	3725	[RbromiiATCC27255]RBATCC27255_00776_hypothetical_protein	RbromiiATCC27255
Org3_Gene758	accessory	3163	[RbromiiATCC27255]RBATCC27255_00781_Biotin_carboxyl_carrier_protein_of_acetyl-CoA_carboxylase	RbromiiATCC27255
Org3_Gene763	accessory	3379	[RbromiiATCC27255]RBATCC27255_00786_cell_division_protein_MraZ	RbromiiATCC27255
Org3_Gene765	accessory	2882	[RbromiiATCC27255]RBATCC27255_00788_hypothetical_protein	RbromiiATCC27255
Org3_Gene771	accessory	631	[RbromiiATCC27255]RBATCC27255_00794_cell_division_protein_FtsQ	RbromiiATCC27255
Org3_Gene773	accessory	1530	[RbromiiATCC27255]RBATCC27255_00796_TRAP_transporter_solute_receptor,_TAXI_family	RbromiiATCC27255
Org3_Gene774	accessory	1055	[RbromiiATCC27255]RBATCC27255_00797_Glutamate_permease	RbromiiATCC27255
Org3_Gene775	accessory	4268	[RbromiiATCC27255]RBATCC27255_00798_hypothetical_protein	RbromiiATCC27255
Org3_Gene776	accessory	1482	[RbromiiATCC27255]RBATCC27255_00799_Choloylglycine_hydrolase	RbromiiATCC27255
Org3_Gene777	accessory	994	[RbromiiATCC27255]RBATCC27255_00800_H(+)/Cl(-)_exchange_transporter_ClcA	RbromiiATCC27255
Org3_Gene778	accessory	3314	[RbromiiATCC27255]RBATCC27255_00801_hypothetical_protein	RbromiiATCC27255
Org3_Gene779	accessory	545	[RbromiiATCC27255]RBATCC27255_00802_Iron_hydrogenase_1	RbromiiATCC27255
Org3_Gene780	accessory	2134	[RbromiiATCC27255]RBATCC27255_00803_vancomycin_high_temperature_exclusion_protein	RbromiiATCC27255
Org3_Gene781	accessory	3996	[RbromiiATCC27255]RBATCC27255_00804_hypothetical_protein	RbromiiATCC27255
Org3_Gene789	accessory	1475	[RbromiiATCC27255]RBATCC27255_00812_O-acetyl-ADP-ribose_deacetylase	RbromiiATCC27255
Org3_Gene790	accessory	2562	[RbromiiATCC27255]RBATCC27255_00813_putative_protein_encoded_in_toxicity_protection_region_of_plasmid_R478,_contains_von_Willebrand_factor_	RbromiiATCC27255
Org3_Gene791	accessory	735	[RbromiiATCC27255]RBATCC27255_00814_putative_membrane_protein	RbromiiATCC27255
Org3_Gene793	accessory	284	[RbromiiATCC27255]RBATCC27255_00816_Fructose-1,6-bisphosphatase_class_3	RbromiiATCC27255
Org3_Gene795	accessory	242	[RbromiiATCC27255]RBATCC27255_00818_Lipoteichoic_acid_synthase_1	RbromiiATCC27255
Org3_Gene796	accessory	969	[RbromiiATCC27255]RBATCC27255_00819_putative_protein_involved_in_cytokinesis,_contains_TGc_(transglutaminase/protease-like)_domain	RbromiiATCC27255
Org3_Gene799	accessory	2357	[RbromiiATCC27255]RBATCC27255_00822_hypothetical_protein	RbromiiATCC27255
Org3_Gene8	accessory	4218	[RbromiiATCC27255]RBATCC27255_00010_hypothetical_protein	RbromiiATCC27255
Org3_Gene801	accessory	1899	[RbromiiATCC27255]RBATCC27255_00824_transcriptional_repressor_DicA	RbromiiATCC27255
Org3_Gene805	accessory	3362	[RbromiiATCC27255]RBATCC27255_00828_hypothetical_protein	RbromiiATCC27255
Org3_Gene808	accessory	3047	[RbromiiATCC27255]RBATCC27255_00832_hypothetical_protein	RbromiiATCC27255
Org3_Gene809	accessory	2250	[RbromiiATCC27255]RBATCC27255_00833_Sensory_transduction_protein_LytR	RbromiiATCC27255
Org3_Gene810	accessory	841	[RbromiiATCC27255]RBATCC27255_00834_sensory_histidine_kinase_DcuS	RbromiiATCC27255
Org3_Gene811	accessory	968	[RbromiiATCC27255]RBATCC27255_00835_CHAP_domain_protein	RbromiiATCC27255
Org3_Gene812	accessory	1159	[RbromiiATCC27255]RBATCC27255_00837_hypothetical_protein	RbromiiATCC27255
Org3_Gene815	accessory	749	[RbromiiATCC27255]RBATCC27255_00841_putative_aminodeoxychorismate_lyase	RbromiiATCC27255
Org3_Gene816	accessory	2684	[RbromiiATCC27255]RBATCC27255_00842_5-formyltetrahydrofolate_cyclo-ligase_family_protein	RbromiiATCC27255
Org3_Gene818	accessory	2072	[RbromiiATCC27255]RBATCC27255_00844_putative_acetyltransferase_involved_in_intracellular_survival	RbromiiATCC27255
Org3_Gene819	accessory	1737	[RbromiiATCC27255]RBATCC27255_00845_hypothetical_protein	RbromiiATCC27255
Org3_Gene820	accessory	2455	[RbromiiATCC27255]RBATCC27255_00846_Thymidylate_kinase	RbromiiATCC27255
Org3_Gene824	accessory	2306	[RbromiiATCC27255]RBATCC27255_00850_phosphoglycolate_phosphatase	RbromiiATCC27255
Org3_Gene826	accessory	1502	[RbromiiATCC27255]RBATCC27255_00852_Bifunctional_protein_BirA	RbromiiATCC27255
Org3_Gene837	accessory	3080	[RbromiiATCC27255]RBATCC27255_00863_Undecaprenol_kinase	RbromiiATCC27255
Org3_Gene847	accessory	3116	[RbromiiATCC27255]RBATCC27255_00873_Phosphopantetheine_adenylyltransferase	RbromiiATCC27255
Org3_Gene857	accessory	2517	[RbromiiATCC27255]RBATCC27255_00883_Phosphoglycolate_phosphatase	RbromiiATCC27255
Org3_Gene858	accessory	2420	[RbromiiATCC27255]RBATCC27255_00884_hypothetical_protein	RbromiiATCC27255
Org3_Gene859	accessory	430	[RbromiiATCC27255]RBATCC27255_00885_Phosphoenolpyruvate-protein_phosphotransferase	RbromiiATCC27255
Org3_Gene861	accessory	308	[RbromiiATCC27255]RBATCC27255_00887_EIIABC-Fru	RbromiiATCC27255
Org3_Gene862	accessory	1678	[RbromiiATCC27255]RBATCC27255_00888_Tagatose-6-phosphate_kinase	RbromiiATCC27255
Org3_Gene863	accessory	2142	[RbromiiATCC27255]RBATCC27255_00889_HTH-type_transcriptional_repressor_GlcR	RbromiiATCC27255

Org3_Gene864	accessory	2569	[RbromiiATCC27255]RBATCC27255_00890_DNA-binding_transcriptional_dual_regulator_Crp	RbromiiATCC27255
Org3_Gene867	accessory	2329	[RbromiiATCC27255]RBATCC27255_00893_hypothetical_protein	RbromiiATCC27255
Org3_Gene870	accessory	1792	[RbromiiATCC27255]RBATCC27255_00896_Nicotinamide_nucleotide_repair_protein	RbromiiATCC27255
Org3_Gene871	accessory	2902	[RbromiiATCC27255]RBATCC27255_00897_hypothetical_protein	RbromiiATCC27255
Org3_Gene877	accessory	3941	[RbromiiATCC27255]RBATCC27255_00904_Antitoxin_DinJ	RbromiiATCC27255
Org3_Gene88	accessory	4431	[RbromiiATCC27255]RBATCC27255_00092_Ribosomal_protein_II	RbromiiATCC27255
Org3_Gene885	accessory	2651	[RbromiiATCC27255]RBATCC27255_00912_N-(5'-phosphoribosyl)anthranilate_isomerase	RbromiiATCC27255
Org3_Gene886	accessory	1599	[RbromiiATCC27255]RBATCC27255_00913_Cysteine_synthase	RbromiiATCC27255
Org3_Gene888	accessory	3351	[RbromiiATCC27255]RBATCC27255_00915_Cysteine_metabolism_repressor	RbromiiATCC27255
Org3_Gene889	accessory	698	[RbromiiATCC27255]RBATCC27255_00916_putative_adenine_permease_PurP	RbromiiATCC27255
Org3_Gene891	accessory	3184	[RbromiiATCC27255]RBATCC27255_00918_hypothetical_protein	RbromiiATCC27255
Org3_Gene892	accessory	1403	[RbromiiATCC27255]RBATCC27255_00919_bacterial_surface_protein_26-residue_repeat	RbromiiATCC27255
Org3_Gene897	accessory	151	[RbromiiATCC27255]RBATCC27255_00924_X-X-X-Leu-X-X-Gly_heptad_repeats	RbromiiATCC27255
Org3_Gene899	accessory	2828	[RbromiiATCC27255]RBATCC27255_00926_putative_dihydroxyacetone_kinase_regulator	RbromiiATCC27255
Org3_Gene900	accessory	2600	[RbromiiATCC27255]RBATCC27255_00927_4'-phosphopantetheinyl_transferase_sfp	RbromiiATCC27255
Org3_Gene901	accessory	3106	[RbromiiATCC27255]RBATCC27255_00928_deoxycytidylate_deaminase	RbromiiATCC27255
Org3_Gene906	accessory	4416	[RbromiiATCC27255]RBATCC27255_00933_hypothetical_protein	RbromiiATCC27255
Org3_Gene909	accessory	2489	[RbromiiATCC27255]RBATCC27255_00936_5'-nucleotidase	RbromiiATCC27255
Org3_Gene910	accessory	3180	[RbromiiATCC27255]RBATCC27255_00937_hypothetical_protein	RbromiiATCC27255
Org3_Gene911	accessory	2298	[RbromiiATCC27255]RBATCC27255_00938_Ribosomal_small_subunit_pseudouridine_synthase_A	RbromiiATCC27255
Org3_Gene915	accessory	1709	[RbromiiATCC27255]RBATCC27255_00942_Cell_division_protein_FtsX	RbromiiATCC27255
Org3_Gene916	accessory	825	[RbromiiATCC27255]RBATCC27255_00943_Septal_ring_factor	RbromiiATCC27255
Org3_Gene917	accessory	1945	[RbromiiATCC27255]RBATCC27255_00944_hypothetical_protein	RbromiiATCC27255
Org3_Gene921	accessory	777	[RbromiiATCC27255]RBATCC27255_00948_Multidrug_export_protein_MepA	RbromiiATCC27255
Org3_Gene922	accessory	2466	[RbromiiATCC27255]RBATCC27255_00949_Heme_response_regulator_HssR	RbromiiATCC27255
Org3_Gene923	accessory	1336	[RbromiiATCC27255]RBATCC27255_00950_Sensor_kinase_CusS	RbromiiATCC27255
Org3_Gene924	accessory	1245	[RbromiiATCC27255]RBATCC27255_00951_hypothetical_protein	RbromiiATCC27255
Org3_Gene925	accessory	3624	[RbromiiATCC27255]RBATCC27255_00952_arsenate_reductase,_glutathione/glutaredoxin_type	RbromiiATCC27255
Org3_Gene926	accessory	3082	[RbromiiATCC27255]RBATCC27255_00953_hypothetical_protein	RbromiiATCC27255
Org3_Gene931	accessory	3185	[RbromiiATCC27255]RBATCC27255_00958_hypothetical_protein	RbromiiATCC27255
Org3_Gene933	accessory	3141	[RbromiiATCC27255]RBATCC27255_00960_hypothetical_protein	RbromiiATCC27255
Org3_Gene935	accessory	2728	[RbromiiATCC27255]RBATCC27255_00962_Stage_0_sporulation_protein_H	RbromiiATCC27255
Org3_Gene936	accessory	556	[RbromiiATCC27255]RBATCC27255_00963_Competence_protein_ComM	RbromiiATCC27255
Org3_Gene938	accessory	2486	[RbromiiATCC27255]RBATCC27255_00965_tRNA_(adenine(22)-N(1))-methyltransferase	RbromiiATCC27255
Org3_Gene939	accessory	2171	[RbromiiATCC27255]RBATCC27255_00966_metal-binding_protein	RbromiiATCC27255
Org3_Gene941	accessory	785	[RbromiiATCC27255]RBATCC27255_00968_hypothetical_protein	RbromiiATCC27255
Org3_Gene945	accessory	1895	[RbromiiATCC27255]RBATCC27255_00972_Release_factor_glutamine_methyltransferase	RbromiiATCC27255
Org3_Gene953	accessory	2812	[RbromiiATCC27255]RBATCC27255_00980_hypothetical_protein	RbromiiATCC27255
Org3_Gene954	accessory	3751	[RbromiiATCC27255]RBATCC27255_00981_Putative_PBSX_repressor	RbromiiATCC27255
Org3_Gene955	accessory	1470	[RbromiiATCC27255]RBATCC27255_00982_hypothetical_protein	RbromiiATCC27255
Org3_Gene956	accessory	482	[RbromiiATCC27255]RBATCC27255_00983_D-alanyl-lipoteichoic_acid_biosynthesis_protein_DltB	RbromiiATCC27255
Org3_Gene957	accessory	4091	[RbromiiATCC27255]RBATCC27255_00984_D-alanine--poly(phosphoribitol)_ligase_subunit_2	RbromiiATCC27255
Org3_Gene958	accessory	1073	[RbromiiATCC27255]RBATCC27255_00985_L-glutamyl-[BtrI_acyl-carrier_protein]_decarboxylase	RbromiiATCC27255
Org3_Gene959	accessory	567	[RbromiiATCC27255]RBATCC27255_00986_Tyrocidine_synthase_I	RbromiiATCC27255
Org3_Gene960	accessory	790	[RbromiiATCC27255]RBATCC27255_00987_Alkaline_phosphatase_synthesis_sensor_protein_PhoR	RbromiiATCC27255
Org3_Gene961	accessory	2085	[RbromiiATCC27255]RBATCC27255_00988_Sensory_transduction_protein_regX3	RbromiiATCC27255
Org3_Gene962	accessory	3133	[RbromiiATCC27255]RBATCC27255_00989_hypothetical_protein	RbromiiATCC27255
Org3_Gene963	accessory	4143	[RbromiiATCC27255]RBATCC27255_00990_hypothetical_protein	RbromiiATCC27255
Org3_Gene964	accessory	1630	[RbromiiATCC27255]RBATCC27255_00991_ComEC_family_competence_protein	RbromiiATCC27255
Org3_Gene967	accessory	3132	[RbromiiATCC27255]RBATCC27255_00994_transcriptional_regulator_SlyA	RbromiiATCC27255
Org3_Gene968	accessory	2276	[RbromiiATCC27255]RBATCC27255_00995_hypothetical_protein	RbromiiATCC27255
Org3_Gene969	accessory	1507	[RbromiiATCC27255]RBATCC27255_00996_ribonuclease_BN/unknown_domain_fusion_protein	RbromiiATCC27255
Org3_Gene971	accessory	2563	[RbromiiATCC27255]RBATCC27255_00998_Demethylrebeccamycin-D-glucose_O-methyltransferase	RbromiiATCC27255
Org3_Gene972	accessory	2542	[RbromiiATCC27255]RBATCC27255_00999_hypothetical_protein	RbromiiATCC27255
Org3_Gene975	accessory	1194	[RbromiiATCC27255]RBATCC27255_01002_hypothetical_protein	RbromiiATCC27255
Org3_Gene976	accessory	303	[RbromiiATCC27255]RBATCC27255_01003_hypothetical_protein	RbromiiATCC27255
Org3_Gene977	accessory	2313	[RbromiiATCC27255]RBATCC27255_01004_Sensory_transduction_protein_LytR	RbromiiATCC27255
Org3_Gene978	accessory	427	[RbromiiATCC27255]RBATCC27255_01005_Serine_protease_Do-like_HtrB	RbromiiATCC27255
Org3_Gene979	accessory	3137	[RbromiiATCC27255]RBATCC27255_01006_hypothetical_protein	RbromiiATCC27255
Org3_Gene980	accessory	1731	[RbromiiATCC27255]RBATCC27255_01007_sporulation_inhibitor_KapD	RbromiiATCC27255
Org3_Gene985	accessory	71	[RbromiiATCC27255]RBATCC27255_01012_Pullulanase_precursor	RbromiiATCC27255
Org3_Gene987	accessory	930	[RbromiiATCC27255]RBATCC27255_01014_Enolase	RbromiiATCC27255
Org3_Gene988	accessory	3818	[RbromiiATCC27255]RBATCC27255_01015_Muramidase-2_precursor	RbromiiATCC27255
Org3_Gene994	accessory	2014	[RbromiiATCC27255]RBATCC27255_01021_D-alanyl-D-alanine_carboxypeptidase	RbromiiATCC27255
Org3_Gene997	accessory	2657	[RbromiiATCC27255]RBATCC27255_01025_hypothetical_protein	RbromiiATCC27255
Org3_Gene998	accessory	3835	[RbromiiATCC27255]RBATCC27255_01026_RNA_polymerase_sigma_factor	RbromiiATCC27255
Org3_Gene999	accessory	4191	[RbromiiATCC27255]RBATCC27255_01027_hypothetical_protein	RbromiiATCC27255
Org4_Gene100	accessory	4281	[RbromiiL2-36]RBL236_00101_hypothetical_protein	RbromiiL2-36
Org4_Gene1000	accessory	2222	[RbromiiL2-36]RBL236_01050_tRNA1(Val)_(adenine(37)-N6)-methyltransferase	RbromiiL2-36
Org4_Gene1003	accessory	1827	[RbromiiL2-36]RBL236_01053_DNA_polymerase_III_subunit_tau	RbromiiL2-36
Org4_Gene1004	accessory	3178	[RbromiiL2-36]RBL236_01054_Phosphodiesterase_YfcE	RbromiiL2-36
Org4_Gene1005	accessory	4070	[RbromiiL2-36]RBL236_01055_hypothetical_protein	RbromiiL2-36
Org4_Gene1006	accessory	3622	[RbromiiL2-36]RBL236_01056_hypothetical_protein	RbromiiL2-36
Org4_Gene1007	accessory	3891	[RbromiiL2-36]RBL236_01057_hypothetical_protein	RbromiiL2-36
Org4_Gene101	accessory	2016	[RbromiiL2-36]RBL236_00102_Helix-turn-helix_domain_protein	RbromiiL2-36
Org4_Gene1014	accessory	2932	[RbromiiL2-36]RBL236_01064_Shikimate_kinase	RbromiiL2-36
Org4_Gene102	accessory	3173	[RbromiiL2-36]RBL236_00103_putative_acetyltransferase	RbromiiL2-36
Org4_Gene1021	accessory	3612	[RbromiiL2-36]RBL236_01071_HTH-type_transcriptional_regulator_ImmR	RbromiiL2-36
Org4_Gene1022	accessory	629	[RbromiiL2-36]RBL236_01072_hypothetical_protein	RbromiiL2-36
Org4_Gene1023	accessory	3079	[RbromiiL2-36]RBL236_01073_Double_zinc_ribbon	RbromiiL2-36
Org4_Gene1025	accessory	2863	[RbromiiL2-36]RBL236_01075_Negative_regulator_of_genetic_competence,_sporulation_and_motility	RbromiiL2-36
Org4_Gene1026	accessory	4325	[RbromiiL2-36]RBL236_01076_hypothetical_protein	RbromiiL2-36
Org4_Gene1027	accessory	3672	[RbromiiL2-36]RBL236_01077_hypothetical_protein	RbromiiL2-36
Org4_Gene1028	accessory	3532	[RbromiiL2-36]RBL236_01078_hypothetical_protein	RbromiiL2-36
Org4_Gene103	accessory	4025	[RbromiiL2-36]RBL236_00104_hypothetical_protein	RbromiiL2-36
Org4_Gene1030	accessory	1232	[RbromiiL2-36]RBL236_01080_D-arabitol-phosphate_dehydrogenase	RbromiiL2-36
Org4_Gene1031	accessory	2373	[RbromiiL2-36]RBL236_01081_D-allulose-6-phosphate_3-epimerase	RbromiiL2-36
Org4_Gene1032	accessory	704	[RbromiiL2-36]RBL236_01082_Inner_membrane_metabolite_transport_protein_YdjE	RbromiiL2-36
Org4_Gene1033	accessory	1308	[RbromiiL2-36]RBL236_01083_Sorbitol_dehydrogenase	RbromiiL2-36
Org4_Gene1034	accessory	1913	[RbromiiL2-36]RBL236_01084_D-tagatose-1,6-bisphosphate_aldolase_subunit_GatY	RbromiiL2-36
Org4_Gene1035	accessory	1487	[RbromiiL2-36]RBL236_01085_putative_sugar_kinase_YdjH	RbromiiL2-36
Org4_Gene1036	accessory	1438	[RbromiiL2-36]RBL236_01086_General_stress_protein_69	RbromiiL2-36
Org4_Gene1037	accessory	2185	[RbromiiL2-36]RBL236_01087_HTH-type_transcriptional_repressor_GlcR	RbromiiL2-36
Org4_Gene1038	accessory	728	[RbromiiL2-36]RBL236_01088_Glutamate-aspartate_carrier_protein	RbromiiL2-36
Org4_Gene1039	accessory	1189	[RbromiiL2-36]RBL236_01089_Glycerol_dehydrogenase	RbromiiL2-36
Org4_Gene104	accessory	3760	[RbromiiL2-36]RBL236_00105_Regulator_of_competence-specific_genes	RbromiiL2-36
Org4_Gene1040	accessory	373	[RbromiiL2-36]RBL236_01090_Adenine_deaminase	RbromiiL2-36
Org4_Gene1041	accessory	3269	[RbromiiL2-36]RBL236_01091_hypothetical_protein	RbromiiL2-36
Org4_Gene1042	accessory	2165	[RbromiiL2-36]RBL236_01092_hypothetical_protein	RbromiiL2-36
Org4_Gene1043	accessory	2172	[RbromiiL2-36]RBL236_01093_hypothetical_protein	RbromiiL2-36
Org4_Gene1044	accessory	2402	[RbromiiL2-36]RBL236_01094_hypothetical_protein	RbromiiL2-36
Org4_Gene1045	accessory	2621	[RbromiiL2-36]RBL236_01095_putative_ABC_transporter_ATP-binding_protein_YxlF	RbromiiL2-36
Org4_Gene1046	accessory	1432	[RbromiiL2-36]RBL236_01096_hypothetical_protein	RbromiiL2-36
Org4_Gene1048	accessory	1052	[RbromiiL2-36]RBL236_01098_HAD_phosphoserine_phosphatase-like_hydrolase,_family_IB	RbromiiL2-36
Org4_Gene1049	accessory	616	[RbromiiL2-36]RBL236_01099_Deoxyguanosinetriphosphate_triphosphohydrolase	RbromiiL2-36
Org4_Gene105	accessory	3246	[RbromiiL2-36]RBL236_00106_Bacterial_transcription_activator,_effector_binding_domain	RbromiiL2-36
Org4_Gene1050	accessory	2464	[RbromiiL2-36]RBL236_01100_hypothetical_protein	RbromiiL2-36
Org4_Gene1051	accessory	2456	[RbromiiL2-36]RBL236_01101_hypothetical_protein	RbromiiL2-36
Org4_Gene1052	accessory	854	[RbromiiL2-36]RBL236_01102_putative_ATPase	RbromiiL2-36
Org4_Gene1053	accessory	235	[RbromiiL2-36]RBL236_01103_Cell_cycle_control_protein	RbromiiL2-36
Org4_Gene1054	accessory	2338	[RbromiiL2-36]RBL236_01106_hypothetical_protein	RbromiiL2-36
Org4_Gene1055	accessory	1475	[RbromiiL2-36]RBL236_01107_O-acetyl-ADP-ribose_deacetylase	RbromiiL2-36
Org4_Gene1056	accessory	2562	[RbromiiL2-36]RBL236_01108_putative_protein_encoded_in_toxicity_protection_region_of_plasmid_R478,_contains_von_Willebrand_factor_(vWF)_doma	RbromiiL2-36
Org4_Gene1057	accessory	735	[RbromiiL2-36]RBL236_01109_putative_membrane_protein	RbromiiL2-36
Org4_Gene1058	accessory	3712	[RbromiiL2-36]RBL236_01110_Transcriptional_regulator_YqjI	RbromiiL2-36
Org4_Gene1059	accessory	652	[RbromiiL2-36]RBL236_01111_hypothetical_protein	RbromiiL2-36
Org4_Gene1060	accessory	680	[RbromiiL2-36]RBL236_01112_hypothetical_protein	RbromiiL2-36
Org4_Gene1061	accessory	660	[RbromiiL2-36]RBL236_01113_hypothetical_protein	RbromiiL2-36
Org4_Gene1062	accessory	284	[RbromiiL2-36]RBL236_01114_Fructose-1,6-bisphosphatase_class_3	RbromiiL2-36
Org4_Gene1064	accessory	242	[RbromiiL2-36]RBL236_01116_Lipoteichoic_acid_synthase_1	RbromiiL2-36
Org4_Gene1065	accessory	896	[RbromiiL2-36]RBL236_01117_putative_protein_involved_in_cytokinesis,_contains_TGc_(transglutaminase/protease-like)_domain	RbromiiL2-36
Org4_Gene1066	accessory	969	[RbromiiL2-36]RBL236_01118_putative_protein_involved_in_cytokinesis,_contains_TGc_(transglutaminase/protease-like)_domain	RbromiiL2-36

Org4_Gene1069	accessory	2357	[RbromiiL2-36]RBL236_01121_hypothetical_protein	RbromiiL2-36
Org4_Gene107	accessory	3529	[RbromiiL2-36]RBL236_00108_hypothetical_protein	RbromiiL2-36
Org4_Gene1071	accessory	1899	[RbromiiL2-36]RBL236_01123_Putative_PBSX_repressor	RbromiiL2-36
Org4_Gene1075	accessory	3362	[RbromiiL2-36]RBL236_01127_hypothetical_protein	RbromiiL2-36
Org4_Gene1077	accessory	3380	[RbromiiL2-36]RBL236_01132_SnoaL-like_domain_protein	RbromiiL2-36
Org4_Gene1078	accessory	452	[RbromiiL2-36]RBL236_01133_Putative_DNA-invertase_from_lambdoid_prophage_Rac	RbromiiL2-36
Org4_Gene1079	accessory	4383	[RbromiiL2-36]RBL236_01134_hypothetical_protein	RbromiiL2-36
Org4_Gene108	accessory	3937	[RbromiiL2-36]RBL236_00109_putative_transcriptional_regulator	RbromiiL2-36
Org4_Gene1080	accessory	3609	[RbromiiL2-36]RBL236_01135_mRNA_interferase_EndoA	RbromiiL2-36
Org4_Gene1081	accessory	3346	[RbromiiL2-36]RBL236_01136_RNA_polymerase_sigma_factor_SigX	RbromiiL2-36
Org4_Gene1082	accessory	4275	[RbromiiL2-36]RBL236_01137_hypothetical_protein	RbromiiL2-36
Org4_Gene1083	accessory	711	[RbromiiL2-36]RBL236_01138_Multidrug_export_protein_MepA	RbromiiL2-36
Org4_Gene1084	accessory	2973	[RbromiiL2-36]RBL236_01139_transcriptional_regulator,_Acidobacterial,_PadR-family	RbromiiL2-36
Org4_Gene1085	accessory	1326	[RbromiiL2-36]RBL236_01140_putative_choloylglycine_hydrolase	RbromiiL2-36
Org4_Gene1086	accessory	1816	[RbromiiL2-36]RBL236_01141_Multiple_antibiotic_resistance_protein_MarA	RbromiiL2-36
Org4_Gene1087	accessory	3604	[RbromiiL2-36]RBL236_01142_anaerobic_benzoate_catabolism_transcriptional_regulator	RbromiiL2-36
Org4_Gene1088	accessory	381	[RbromiiL2-36]RBL236_01143_CHAP_domain_protein	RbromiiL2-36
Org4_Gene1089	accessory	2129	[RbromiiL2-36]RBL236_01144_sortase,_SrtB_family	RbromiiL2-36
Org4_Gene109	accessory	4163	[RbromiiL2-36]RBL236_00110_Maff2_family_protein	RbromiiL2-36
Org4_Gene1090	accessory	3432	[RbromiiL2-36]RBL236_01145_hypothetical_protein	RbromiiL2-36
Org4_Gene1091	accessory	181	[RbromiiL2-36]RBL236_01146_conjugal_transfer_ATP-binding_protein_TraC	RbromiiL2-36
Org4_Gene1092	accessory	3255	[RbromiiL2-36]RBL236_01147_PrgI_family_protein	RbromiiL2-36
Org4_Gene1094	accessory	2436	[RbromiiL2-36]RBL236_01149_hypothetical_protein	RbromiiL2-36
Org4_Gene1095	accessory	3601	[RbromiiL2-36]RBL236_01150_hypothetical_protein	RbromiiL2-36
Org4_Gene1096	accessory	372	[RbromiiL2-36]RBL236_01151_type_IV_secretion_system_component_VirD4	RbromiiL2-36
Org4_Gene1097	accessory	4239	[RbromiiL2-36]RBL236_01152_hypothetical_protein	RbromiiL2-36
Org4_Gene1098	accessory	1822	[RbromiiL2-36]RBL236_01153_hypothetical_protein	RbromiiL2-36
Org4_Gene1099	accessory	2000	[RbromiiL2-36]RBL236_01154_hypothetical_protein	RbromiiL2-36
Org4_Gene110	accessory	4347	[RbromiiL2-36]RBL236_00111_hypothetical_protein	RbromiiL2-36
Org4_Gene1100	accessory	674	[RbromiiL2-36]RBL236_01155_Relaxase/Mobilisation_nuclease_domain_protein	RbromiiL2-36
Org4_Gene1101	accessory	3733	[RbromiiL2-36]RBL236_01156_Ribbon-helix-helix_protein,_copG_family	RbromiiL2-36
Org4_Gene1102	accessory	4384	[RbromiiL2-36]RBL236_01157_hypothetical_protein	RbromiiL2-36
Org4_Gene1103	accessory	3862	[RbromiiL2-36]RBL236_01158_hypothetical_protein	RbromiiL2-36
Org4_Gene1104	accessory	1448	[RbromiiL2-36]RBL236_01159_hypothetical_protein	RbromiiL2-36
Org4_Gene1105	accessory	1413	[RbromiiL2-36]RBL236_01160_hypothetical_protein	RbromiiL2-36
Org4_Gene1106	accessory	1016	[RbromiiL2-36]RBL236_01161_hypothetical_protein	RbromiiL2-36
Org4_Gene1107	accessory	3732	[RbromiiL2-36]RBL236_01162_hypothetical_protein	RbromiiL2-36
Org4_Gene1108	accessory	539	[RbromiiL2-36]RBL236_01163_DNA_methylase	RbromiiL2-36
Org4_Gene1109	accessory	4272	[RbromiiL2-36]RBL236_01164_hypothetical_protein	RbromiiL2-36
Org4_Gene1110	accessory	917	[RbromiiL2-36]RBL236_01165_Plasmid_recombination_enzyme	RbromiiL2-36
Org4_Gene1111	accessory	1387	[RbromiiL2-36]RBL236_01166_DNA_repair_protein_RadA	RbromiiL2-36
Org4_Gene1112	accessory	1559	[RbromiiL2-36]RBL236_01167_Adenine_deaminase	RbromiiL2-36
Org4_Gene1113	accessory	4112	[RbromiiL2-36]RBL236_01168_hypothetical_protein	RbromiiL2-36
Org4_Gene1114	accessory	234	[RbromiiL2-36]RBL236_01169_Recombinase	RbromiiL2-36
Org4_Gene1115	accessory	4145	[RbromiiL2-36]RBL236_01170_hypothetical_protein	RbromiiL2-36
Org4_Gene1117	accessory	3171	[RbromiiL2-36]RBL236_01172_hypothetical_protein	RbromiiL2-36
Org4_Gene1118	accessory	364	[RbromiiL2-36]RBL236_01173_conjugal_transfer_coupling_protein_TraG	RbromiiL2-36
Org4_Gene112	accessory	3573	[RbromiiL2-36]RBL236_00113_hypothetical_protein	RbromiiL2-36
Org4_Gene1121	accessory	2975	[RbromiiL2-36]RBL236_01176_hypothetical_protein	RbromiiL2-36
Org4_Gene1122	accessory	3861	[RbromiiL2-36]RBL236_01177_hypothetical_protein	RbromiiL2-36
Org4_Gene1123	accessory	1972	[RbromiiL2-36]RBL236_01178_HTH-type_transcriptional_regulator_ImmR	RbromiiL2-36
Org4_Gene1124	accessory	3703	[RbromiiL2-36]RBL236_01179_hypothetical_protein	RbromiiL2-36
Org4_Gene1125	accessory	3830	[RbromiiL2-36]RBL236_01180_hypothetical_protein	RbromiiL2-36
Org4_Gene1126	accessory	3599	[RbromiiL2-36]RBL236_01181_hypothetical_protein	RbromiiL2-36
Org4_Gene1127	accessory	519	[RbromiiL2-36]RBL236_01182_DNA_strand_transferase	RbromiiL2-36
Org4_Gene1128	accessory	2577	[RbromiiL2-36]RBL236_01183_DNA_primase	RbromiiL2-36
Org4_Gene113	accessory	4252	[RbromiiL2-36]RBL236_00114_hypothetical_protein	RbromiiL2-36
Org4_Gene1132	accessory	1819	[RbromiiL2-36]RBL236_01187_hypothetical_protein	RbromiiL2-36
Org4_Gene1134	accessory	3485	[RbromiiL2-36]RBL236_01189_PrgI_family_protein	RbromiiL2-36
Org4_Gene1136	accessory	1536	[RbromiiL2-36]RBL236_01191_Modification_methylase_DpnIIB	RbromiiL2-36
Org4_Gene1137	accessory	289	[RbromiiL2-36]RBL236_01192_D-gamma-glutamyl-meso-diaminopimelic_acid_endopeptidase_CwlS_precursor	RbromiiL2-36
Org4_Gene1138	accessory	2290	[RbromiiL2-36]RBL236_01193_PUA_domain_(predicted_RNA-binding_domain)	RbromiiL2-36
Org4_Gene1139	accessory	3959	[RbromiiL2-36]RBL236_01194_hypothetical_protein	RbromiiL2-36
Org4_Gene114	accessory	1823	[RbromiiL2-36]RBL236_00115_DNA_replication_protein_DnaC	RbromiiL2-36
Org4_Gene1140	accessory	2199	[RbromiiL2-36]RBL236_01195_hypothetical_protein	RbromiiL2-36
Org4_Gene1141	accessory	255	[RbromiiL2-36]RBL236_01196_DNA_topoisomerase_3	RbromiiL2-36
Org4_Gene1142	accessory	58	[RbromiiL2-36]RBL236_01197_Antirestriction_protein_(ArdA)	RbromiiL2-36
Org4_Gene1143	accessory	4183	[RbromiiL2-36]RBL236_01198_hypothetical_protein	RbromiiL2-36
Org4_Gene1144	accessory	792	[RbromiiL2-36]RBL236_01199_Relaxase/Mobilisation_nuclease_domain_protein	RbromiiL2-36
Org4_Gene1145	accessory	3735	[RbromiiL2-36]RBL236_01200_Bacterial_mobilisation_protein_(MobC)	RbromiiL2-36
Org4_Gene1146	accessory	3615	[RbromiiL2-36]RBL236_01201_anaerobic_benzoate_catabolism_transcriptional_regulator	RbromiiL2-36
Org4_Gene1148	accessory	3309	[RbromiiL2-36]RBL236_01203_RNA_polymerase_sigma_factor	RbromiiL2-36
Org4_Gene1149	accessory	4045	[RbromiiL2-36]RBL236_01204_Helix-turn-helix_domain_protein	RbromiiL2-36
Org4_Gene115	accessory	2240	[RbromiiL2-36]RBL236_00116_hypothetical_protein	RbromiiL2-36
Org4_Gene1150	accessory	4334	[RbromiiL2-36]RBL236_01205_hypothetical_protein	RbromiiL2-36
Org4_Gene1153	accessory	11	[RbromiiL2-36]RBL236_01208_DNA_methylase	RbromiiL2-36
Org4_Gene1154	accessory	4195	[RbromiiL2-36]RBL236_01209_hypothetical_protein	RbromiiL2-36
Org4_Gene1155	accessory	2346	[RbromiiL2-36]RBL236_01210_hypothetical_protein	RbromiiL2-36
Org4_Gene1156	accessory	3934	[RbromiiL2-36]RBL236_01211_hypothetical_protein	RbromiiL2-36
Org4_Gene1157	accessory	3833	[RbromiiL2-36]RBL236_01212_hypothetical_protein	RbromiiL2-36
Org4_Gene1158	accessory	4434	[RbromiiL2-36]RBL236_01213_hypothetical_protein	RbromiiL2-36
Org4_Gene1159	accessory	27	[RbromiiL2-36]RBL236_01214_putative_outer_membrane_protein	RbromiiL2-36
Org4_Gene116	accessory	4324	[RbromiiL2-36]RBL236_00117_hypothetical_protein	RbromiiL2-36
Org4_Gene1160	accessory	1560	[RbromiiL2-36]RBL236_01215_Chromosome-partitioning_protein_ParB	RbromiiL2-36
Org4_Gene1161	accessory	1947	[RbromiiL2-36]RBL236_01216_Sporulation_initiation_inhibitor_protein_soj	RbromiiL2-36
Org4_Gene1162	accessory	4318	[RbromiiL2-36]RBL236_01217_hypothetical_protein	RbromiiL2-36
Org4_Gene1168	accessory	2840	[RbromiiL2-36]RBL236_01223_Helix-turn-helix_domain_of_resolvase	RbromiiL2-36
Org4_Gene1169	accessory	1979	[RbromiiL2-36]RBL236_01224_hypothetical_protein	RbromiiL2-36
Org4_Gene117	accessory	3570	[RbromiiL2-36]RBL236_00118_addiction_module_antitoxin,_RelB/DinJ_family	RbromiiL2-36
Org4_Gene1176	accessory	324	[RbromiiL2-36]RBL236_01231_hypothetical_protein	RbromiiL2-36
Org4_Gene1177	accessory	2712	[RbromiiL2-36]RBL236_01232_Kynurenine_formamidase	RbromiiL2-36
Org4_Gene1178	accessory	2517	[RbromiiL2-36]RBL236_01233_Phosphoglycolate_phosphatase	RbromiiL2-36
Org4_Gene1179	accessory	2420	[RbromiiL2-36]RBL236_01234_hypothetical_protein	RbromiiL2-36
Org4_Gene118	accessory	3873	[RbromiiL2-36]RBL236_00119_mRNA_interferase_YafQ	RbromiiL2-36
Org4_Gene1181	accessory	4414	[RbromiiL2-36]RBL236_01238_hypothetical_protein	RbromiiL2-36
Org4_Gene1182	accessory	4366	[RbromiiL2-36]RBL236_01239_hypothetical_protein	RbromiiL2-36
Org4_Gene1183	accessory	3776	[RbromiiL2-36]RBL236_01240_hypothetical_protein	RbromiiL2-36
Org4_Gene1184	accessory	3266	[RbromiiL2-36]RBL236_01241_Modification_methylase_HhaI	RbromiiL2-36
Org4_Gene1185	accessory	2057	[RbromiiL2-36]RBL236_01242_DNA_cytosine_methylase	RbromiiL2-36
Org4_Gene1186	accessory	1451	[RbromiiL2-36]RBL236_01243_Modification_methylase_DpnIIA	RbromiiL2-36
Org4_Gene1187	accessory	4230	[RbromiiL2-36]RBL236_01244_hypothetical_protein	RbromiiL2-36
Org4_Gene1188	accessory	3868	[RbromiiL2-36]RBL236_01245_transcriptional_regulator,_AbrB_family	RbromiiL2-36
Org4_Gene1189	accessory	2148	[RbromiiL2-36]RBL236_01246_hypothetical_protein	RbromiiL2-36
Org4_Gene119	accessory	638	[RbromiiL2-36]RBL236_00120_DNA_strand_transferase	RbromiiL2-36
Org4_Gene1190	accessory	2696	[RbromiiL2-36]RBL236_01247_hypothetical_protein	RbromiiL2-36
Org4_Gene1191	accessory	22	[RbromiiL2-36]RBL236_01248_putative_outer_membrane_protein	RbromiiL2-36
Org4_Gene1192	accessory	2244	[RbromiiL2-36]RBL236_01249_putative_outer_membrane_protein	RbromiiL2-36
Org4_Gene1193	accessory	3636	[RbromiiL2-36]RBL236_01250_hypothetical_protein	RbromiiL2-36
Org4_Gene1194	accessory	3654	[RbromiiL2-36]RBL236_01251_hypothetical_protein	RbromiiL2-36
Org4_Gene1195	accessory	1228	[RbromiiL2-36]RBL236_01252_hypothetical_protein	RbromiiL2-36
Org4_Gene1196	accessory	3077	[RbromiiL2-36]RBL236_01253_Putative_PBSX_repressor	RbromiiL2-36
Org4_Gene1197	accessory	1185	[RbromiiL2-36]RBL236_01254_hypothetical_protein	RbromiiL2-36
Org4_Gene1198	accessory	4059	[RbromiiL2-36]RBL236_01255_hypothetical_protein	RbromiiL2-36
Org4_Gene1199	accessory	252	[RbromiiL2-36]RBL236_01256_Putative_amidoligase_enzyme	RbromiiL2-36
Org4_Gene12	accessory	3777	[RbromiiL2-36]RBL236_00012_putative_ribosomal_protein	RbromiiL2-36
Org4_Gene120	accessory	3869	[RbromiiL2-36]RBL236_00121_hypothetical_protein	RbromiiL2-36
Org4_Gene1200	accessory	1464	[RbromiiL2-36]RBL236_01257_hypothetical_protein	RbromiiL2-36
Org4_Gene1201	accessory	1640	[RbromiiL2-36]RBL236_01258_DNA_primase	RbromiiL2-36
Org4_Gene1202	accessory	299	[RbromiiL2-36]RBL236_01259_Conjugal_transfer_protein_TraG	RbromiiL2-36
Org4_Gene1205	accessory	2428	[RbromiiL2-36]RBL236_01262_Antirestriction_protein	RbromiiL2-36
Org4_Gene1206	accessory	2890	[RbromiiL2-36]RBL236_01263_hypothetical_protein	RbromiiL2-36
Org4_Gene1207	accessory	1581	[RbromiiL2-36]RBL236_01264_hypothetical_protein	RbromiiL2-36

Org4_Gene1208	accessory	3685	[RbromiiL2-36]RBL236_01265_hypothetical_protein	RbromiiL2-36
Org4_Gene1209	accessory	24	[RbromiiL2-36]RBL236_01266_putative_outer_membrane_protein	RbromiiL2-36
Org4_Gene121	accessory	3215	[RbromiiL2-36]RBL236_00122_RNA_polymerase_sigma_factor,_sigma-70_family	RbromiiL2-36
Org4_Gene1210	accessory	2597	[RbromiiL2-36]RBL236_01267_hypothetical_protein	RbromiiL2-36
Org4_Gene1211	accessory	1576	[RbromiiL2-36]RBL236_01268_putative_chromosome-partitioning_protein_ParB	RbromiiL2-36
Org4_Gene1212	accessory	1957	[RbromiiL2-36]RBL236_01269_Sporulation_initiation_inhibitor_protein_soj	RbromiiL2-36
Org4_Gene1213	accessory	4050	[RbromiiL2-36]RBL236_01270_hypothetical_protein	RbromiiL2-36
Org4_Gene1214	accessory	3053	[RbromiiL2-36]RBL236_01271_hypothetical_protein	RbromiiL2-36
Org4_Gene1215	accessory	3381	[RbromiiL2-36]RBL236_01272_hypothetical_protein	RbromiiL2-36
Org4_Gene1216	accessory	3123	[RbromiiL2-36]RBL236_01273_hypothetical_protein	RbromiiL2-36
Org4_Gene122	accessory	3257	[RbromiiL2-36]RBL236_00123_RNA_polymerase_sigma_factor	RbromiiL2-36
Org4_Gene1222	accessory	3441	[RbromiiL2-36]RBL236_01279_Transposase	RbromiiL2-36
Org4_Gene1223	accessory	2997	[RbromiiL2-36]RBL236_01280_dephospho-CoA_kinase/protein_folding_accessory_domain-containing_protein	RbromiiL2-36
Org4_Gene1224	accessory	1577	[RbromiiL2-36]RBL236_01281_hypothetical_protein	RbromiiL2-36
Org4_Gene1225	accessory	2113	[RbromiiL2-36]RBL236_01282_Sulfite_exporter_TauE/SafE	RbromiiL2-36
Org4_Gene1226	accessory	1395	[RbromiiL2-36]RBL236_01283_Glucose-resistance_amylase_regulator	RbromiiL2-36
Org4_Gene1227	accessory	604	[RbromiiL2-36]RBL236_01284_Thiomethylgalactoside_permease_II	RbromiiL2-36
Org4_Gene1228	accessory	888	[RbromiiL2-36]RBL236_01285_Galactokinase	RbromiiL2-36
Org4_Gene123	accessory	3406	[RbromiiL2-36]RBL236_00124_RNA_polymerase_sigma_factor,_sigma-70_family	RbromiiL2-36
Org4_Gene1232	accessory	2309	[RbromiiL2-36]RBL236_01289_Transcriptional_regulatory_protein_YpdB	RbromiiL2-36
Org4_Gene1233	accessory	838	[RbromiiL2-36]RBL236_01290_sensory_histidine_kinase_DcuS	RbromiiL2-36
Org4_Gene1234	accessory	810	[RbromiiL2-36]RBL236_01291_hypothetical_protein	RbromiiL2-36
Org4_Gene1235	accessory	1114	[RbromiiL2-36]RBL236_01292_Fucose_4-O-acetylase	RbromiiL2-36
Org4_Gene1236	accessory	3270	[RbromiiL2-36]RBL236_01293_hypothetical_protein	RbromiiL2-36
Org4_Gene1237	accessory	921	[RbromiiL2-36]RBL236_01294_hypothetical_protein	RbromiiL2-36
Org4_Gene1238	accessory	190	[RbromiiL2-36]RBL236_01295_ABC-type_transport_system_involved_in_multi-copper_enzyme_maturation,_permease_component	RbromiiL2-36
Org4_Gene1239	accessory	1770	[RbromiiL2-36]RBL236_01296_putative_ABC_transporter_ATP-binding_protein_YxlF	RbromiiL2-36
Org4_Gene124	accessory	2243	[RbromiiL2-36]RBL236_00125_ABC-type_transport_system_involved_in_multi-copper_enzyme_maturation,_permease_component	RbromiiL2-36
Org4_Gene1240	accessory	3295	[RbromiiL2-36]RBL236_01297_hypothetical_protein	RbromiiL2-36
Org4_Gene1242	accessory	571	[RbromiiL2-36]RBL236_01299_putative_membrane_protein	RbromiiL2-36
Org4_Gene125	accessory	2259	[RbromiiL2-36]RBL236_00126_lantibiotic_protection_ABC_transporter_permease_subunit,_MutG_family	RbromiiL2-36
Org4_Gene1251	accessory	1382	[RbromiiL2-36]RBL236_01308_SpoIVB_peptidase_precursor	RbromiiL2-36
Org4_Gene1252	accessory	167	[RbromiiL2-36]RBL236_01309_Calcium-transporting_ATPase	RbromiiL2-36
Org4_Gene1253	accessory	768	[RbromiiL2-36]RBL236_01310_CCA-adding_enzyme	RbromiiL2-36
Org4_Gene1255	accessory	560	[RbromiiL2-36]RBL236_01312_Arginine_transport_system_permease_protein_ArtQ	RbromiiL2-36
Org4_Gene1257	accessory	119	[RbromiiL2-36]RBL236_01315_Alpha-amylase_precursor	RbromiiL2-36
Org4_Gene1258	accessory	1672	[RbromiiL2-36]RBL236_01316_UDP-glucose_4-epimerase	RbromiiL2-36
Org4_Gene126	accessory	1629	[RbromiiL2-36]RBL236_00127_Daunorubicin/doxorubicin_resistance_ATP-binding_protein_DrrA	RbromiiL2-36
Org4_Gene1260	accessory	966	[RbromiiL2-36]RBL236_01318_mercuric_reductase	RbromiiL2-36
Org4_Gene1261	accessory	3152	[RbromiiL2-36]RBL236_01319_putative_membrane_protein	RbromiiL2-36
Org4_Gene1262	accessory	3182	[RbromiiL2-36]RBL236_01320_putative_integral_membrane_protein	RbromiiL2-36
Org4_Gene1264	accessory	3520	[RbromiiL2-36]RBL236_01322_hypothetical_protein	RbromiiL2-36
Org4_Gene1265	accessory	3057	[RbromiiL2-36]RBL236_01323_Zn-dependent_proteases	RbromiiL2-36
Org4_Gene1268	accessory	2429	[RbromiiL2-36]RBL236_01326_hypothetical_protein	RbromiiL2-36
Org4_Gene1269	accessory	1687	[RbromiiL2-36]RBL236_01327_radical_SAM_protein	RbromiiL2-36
Org4_Gene127	accessory	3355	[RbromiiL2-36]RBL236_00128_hypothetical_protein	RbromiiL2-36
Org4_Gene1270	accessory	2091	[RbromiiL2-36]RBL236_01328_hypothetical_protein	RbromiiL2-36
Org4_Gene1274	accessory	1939	[RbromiiL2-36]RBL236_01333_Phosphatidate_cytidylyltransferase	RbromiiL2-36
Org4_Gene1276	accessory	708	[RbromiiL2-36]RBL236_01335_Zinc_metalloprotease_RasP	RbromiiL2-36
Org4_Gene1279	accessory	827	[RbromiiL2-36]RBL236_01338_pheromone_autoinducer_2_transporter	RbromiiL2-36
Org4_Gene128	accessory	2150	[RbromiiL2-36]RBL236_00129_lantibiotic_protection_ABC_transporter_permease_subunit,_MutE/EpiE_family	RbromiiL2-36
Org4_Gene1288	accessory	4057	[RbromiiL2-36]RBL236_01347_hypothetical_protein	RbromiiL2-36
Org4_Gene129	accessory	1627	[RbromiiL2-36]RBL236_00130_Sensor_protein_kinase_WalK	RbromiiL2-36
Org4_Gene1290	accessory	965	[RbromiiL2-36]RBL236_01349_hypothetical_protein	RbromiiL2-36
Org4_Gene1291	accessory	3938	[RbromiiL2-36]RBL236_01350_hypothetical_protein	RbromiiL2-36
Org4_Gene1292	accessory	1779	[RbromiiL2-36]RBL236_01351_Ribosomal_large_subunit_pseudouridine_synthase_D	RbromiiL2-36
Org4_Gene1293	accessory	301	[RbromiiL2-36]RBL236_01352_Bacterial_Ig-like_domain_(group_2)	RbromiiL2-36
Org4_Gene1294	accessory	3911	[RbromiiL2-36]RBL236_01353_mRNA_interferase_YafQ	RbromiiL2-36
Org4_Gene1295	accessory	4007	[RbromiiL2-36]RBL236_01354_hypothetical_protein	RbromiiL2-36
Org4_Gene1296	accessory	414	[RbromiiL2-36]RBL236_01355_hypothetical_protein	RbromiiL2-36
Org4_Gene1297	accessory	150	[RbromiiL2-36]RBL236_01356_Serine/threonine-protein_kinase_PrkC	RbromiiL2-36
Org4_Gene1298	accessory	3480	[RbromiiL2-36]RBL236_01357_putative_membrane_protein	RbromiiL2-36
Org4_Gene1299	accessory	3360	[RbromiiL2-36]RBL236_01358_preprotein_translocase_subunit_YajC	RbromiiL2-36
Org4_Gene130	accessory	2383	[RbromiiL2-36]RBL236_00131_Transcriptional_regulatory_protein_YycF	RbromiiL2-36
Org4_Gene1305	accessory	4103	[RbromiiL2-36]RBL236_01364_hypothetical_protein	RbromiiL2-36
Org4_Gene1306	accessory	1106	[RbromiiL2-36]RBL236_01365_putative_cysteine_desulfurase	RbromiiL2-36
Org4_Gene1308	accessory	925	[RbromiiL2-36]RBL236_01367_YbbR-like_protein	RbromiiL2-36
Org4_Gene1309	accessory	1095	[RbromiiL2-36]RBL236_01368_Cell_division_protein_FtsW	RbromiiL2-36
Org4_Gene131	accessory	4277	[RbromiiL2-36]RBL236_00132_Helix-turn-helix_domain_protein	RbromiiL2-36
Org4_Gene1312	accessory	2337	[RbromiiL2-36]RBL236_01371_t(6)A37_threonylcarbamoyladenosine_biosynthesis_protein	RbromiiL2-36
Org4_Gene1315	accessory	3319	[RbromiiL2-36]RBL236_01374_ribosomal-protein-alanine_acetyltransferase	RbromiiL2-36
Org4_Gene1317	accessory	2626	[RbromiiL2-36]RBL236_01376_hypothetical_protein	RbromiiL2-36
Org4_Gene1318	accessory	377	[RbromiiL2-36]RBL236_01377_Iron_import_ATP-binding/permease_protein_IrtA	RbromiiL2-36
Org4_Gene1319	accessory	410	[RbromiiL2-36]RBL236_01378_Putative_multidrug_export_ATP-binding/permease_protein	RbromiiL2-36
Org4_Gene132	accessory	3571	[RbromiiL2-36]RBL236_00133_anaerobic_benzoate_catabolism_transcriptional_regulator	RbromiiL2-36
Org4_Gene1320	accessory	655	[RbromiiL2-36]RBL236_01379_Divergent_AAA_domain_protein	RbromiiL2-36
Org4_Gene1321	accessory	4223	[RbromiiL2-36]RBL236_01381_hypothetical_protein	RbromiiL2-36
Org4_Gene1322	accessory	1996	[RbromiiL2-36]RBL236_01382_hypothetical_protein	RbromiiL2-36
Org4_Gene1323	accessory	3307	[RbromiiL2-36]RBL236_01383_Cell_wall_hydrolase_CwlJ	RbromiiL2-36
Org4_Gene1324	accessory	2343	[RbromiiL2-36]RBL236_01384_hypothetical_protein	RbromiiL2-36
Org4_Gene1325	accessory	1690	[RbromiiL2-36]RBL236_01385_hypothetical_protein	RbromiiL2-36
Org4_Gene1326	accessory	1229	[RbromiiL2-36]RBL236_01386_Oxygen-independent_coproporphyrinogen-III_oxidase_1	RbromiiL2-36
Org4_Gene1328	accessory	686	[RbromiiL2-36]RBL236_01388_Arginine_decarboxylase	RbromiiL2-36
Org4_Gene133	accessory	3837	[RbromiiL2-36]RBL236_00134_hypothetical_protein	RbromiiL2-36
Org4_Gene1331	accessory	2009	[RbromiiL2-36]RBL236_01391_ribonuclease_Z	RbromiiL2-36
Org4_Gene1333	accessory	4072	[RbromiiL2-36]RBL236_01393_hypothetical_protein	RbromiiL2-36
Org4_Gene1334	accessory	326	[RbromiiL2-36]RBL236_01394_hypothetical_protein	RbromiiL2-36
Org4_Gene1335	accessory	1565	[RbromiiL2-36]RBL236_01395_hypothetical_protein	RbromiiL2-36
Org4_Gene1336	accessory	1732	[RbromiiL2-36]RBL236_01396_Energy-coupling_factor_transporter_transmembrane_protein_EcfT	RbromiiL2-36
Org4_Gene1337	accessory	114	[RbromiiL2-36]RBL236_01397_Putative_HMP/thiamine_import_ATP-binding_protein_YkoD	RbromiiL2-36
Org4_Gene1338	accessory	679	[RbromiiL2-36]RBL236_01398_Amidophosphoribosyltransferase_precursor	RbromiiL2-36
Org4_Gene134	accessory	3627	[RbromiiL2-36]RBL236_00135_hypothetical_protein	RbromiiL2-36
Org4_Gene1340	accessory	2333	[RbromiiL2-36]RBL236_01400_N-acetylmuramoyl-L-alanine_amidase_AmiC_precursor	RbromiiL2-36
Org4_Gene1342	accessory	2396	[RbromiiL2-36]RBL236_01402_hypothetical_protein	RbromiiL2-36
Org4_Gene135	accessory	335	[RbromiiL2-36]RBL236_00136_Recombinase	RbromiiL2-36
Org4_Gene1354	accessory	1578	[RbromiiL2-36]RBL236_01414_Membrane_dipeptidase_(Peptidase_family_M19)	RbromiiL2-36
Org4_Gene1355	accessory	3585	[RbromiiL2-36]RBL236_01415_hypothetical_protein	RbromiiL2-36
Org4_Gene1358	accessory	2785	[RbromiiL2-36]RBL236_01418_Signal_peptidase_I_T	RbromiiL2-36
Org4_Gene1359	accessory	2568	[RbromiiL2-36]RBL236_01419_Signal_peptidase_I_P	RbromiiL2-36
Org4_Gene1364	accessory	2446	[RbromiiL2-36]RBL236_01424_Minicell-associated_protein_DivIVA	RbromiiL2-36
Org4_Gene1365	accessory	2147	[RbromiiL2-36]RBL236_01425_photosystem_II_S4_domain_protein	RbromiiL2-36
Org4_Gene1368	accessory	877	[RbromiiL2-36]RBL236_01428_putative_membrane_fusion_protein	RbromiiL2-36
Org4_Gene1369	accessory	1550	[RbromiiL2-36]RBL236_01429_tRNA_dimethylallyltransferase	RbromiiL2-36
Org4_Gene137	accessory	3155	[RbromiiL2-36]RBL236_00138_hypothetical_protein	RbromiiL2-36
Org4_Gene1370	accessory	229	[RbromiiL2-36]RBL236_01430_DNA_mismatch_repair_protein_MutL	RbromiiL2-36
Org4_Gene1374	accessory	2532	[RbromiiL2-36]RBL236_01434_Sporulation_protein_YunB	RbromiiL2-36
Org4_Gene1375	accessory	3110	[RbromiiL2-36]RBL236_01435_hypothetical_protein	RbromiiL2-36
Org4_Gene1377	accessory	1242	[RbromiiL2-36]RBL236_01437_putative_peptidase	RbromiiL2-36
Org4_Gene138	accessory	4120	[RbromiiL2-36]RBL236_00139_hypothetical_protein	RbromiiL2-36
Org4_Gene1382	accessory	2743	[RbromiiL2-36]RBL236_01442_putative_nicotinate-nucleotide_adenylyltransferase	RbromiiL2-36
Org4_Gene1383	accessory	2681	[RbromiiL2-36]RBL236_01443_putative_nicotinate-nucleotide_adenylyltransferase	RbromiiL2-36
Org4_Gene1388	accessory	1574	[RbromiiL2-36]RBL236_01448_Oxygen-independent_coproporphyrinogen-III_oxidase_1	RbromiiL2-36
Org4_Gene139	accessory	1465	[RbromiiL2-36]RBL236_00140_hypothetical_protein	RbromiiL2-36
Org4_Gene1393	accessory	927	[RbromiiL2-36]RBL236_01453_DNA_protecting_protein_DprA	RbromiiL2-36
Org4_Gene1394	accessory	2102	[RbromiiL2-36]RBL236_01454_23S_rRNA_(uridine(2479)-2'-O)-methyltransferase	RbromiiL2-36
Org4_Gene1397	accessory	2729	[RbromiiL2-36]RBL236_01457_Translation_initiation_factor_IF-3	RbromiiL2-36
Org4_Gene140	accessory	531	[RbromiiL2-36]RBL236_00141_Recombinase	RbromiiL2-36
Org4_Gene1400	accessory	1497	[RbromiiL2-36]RBL236_01461_hypothetical_protein	RbromiiL2-36
Org4_Gene1403	accessory	2030	[RbromiiL2-36]RBL236_01464_sigma-E_processing_peptidase_SpoIIGA	RbromiiL2-36
Org4_Gene1405	accessory	3756	[RbromiiL2-36]RBL236_01466_hypothetical_protein	RbromiiL2-36
Org4_Gene1406	accessory	2074	[RbromiiL2-36]RBL236_01467_Small-conductance_mechanosensitive_channel	RbromiiL2-36
Org4_Gene1407	accessory	467	[RbromiiL2-36]RBL236_01468_hypothetical_protein	RbromiiL2-36

Org4_Gene141	accessory	337	[RbromiiL2-36]RBL236_00144_LPS_biosynthesis_protein	RbromiiL2-36
Org4_Gene1410	accessory	507	[RbromiiL2-36]RBL236_01471_putative_integral_membrane_protein	RbromiiL2-36
Org4_Gene1411	accessory	96	[RbromiiL2-36]RBL236_01472_Fucose_4-O-acetylase	RbromiiL2-36
Org4_Gene1413	accessory	2802	[RbromiiL2-36]RBL236_01474_Phosphinothricin_N-acetyltransferase	RbromiiL2-36
Org4_Gene1414	accessory	328	[RbromiiL2-36]RBL236_01475_LPS_biosynthesis_protein	RbromiiL2-36
Org4_Gene1415	accessory	1266	[RbromiiL2-36]RBL236_01476_dTDP-4-dehydro-6-deoxyglucose_reductase	RbromiiL2-36
Org4_Gene1416	accessory	2225	[RbromiiL2-36]RBL236_01477_2-C-methyl-D-erythritol_4-phosphate_cytidylyltransferase_1	RbromiiL2-36
Org4_Gene1418	accessory	2734	[RbromiiL2-36]RBL236_01479_hypothetical_protein	RbromiiL2-36
Org4_Gene1419	accessory	51	[RbromiiL2-36]RBL236_01480_ATP-dependent_helicase/nuclease_subunit_A	RbromiiL2-36
Org4_Gene142	accessory	1239	[RbromiiL2-36]RBL236_00145_dTDP-glucose_4,6-dehydratase	RbromiiL2-36
Org4_Gene1420	accessory	60	[RbromiiL2-36]RBL236_01481_ATP-dependent_helicase/deoxyribonuclease_subunit_B	RbromiiL2-36
Org4_Gene1421	accessory	2689	[RbromiiL2-36]RBL236_01482_stage_II_sporulation_protein_R	RbromiiL2-36
Org4_Gene1425	accessory	3054	[RbromiiL2-36]RBL236_01486_hypothetical_protein	RbromiiL2-36
Org4_Gene1427	accessory	580	[RbromiiL2-36]RBL236_01488_Lipid_A_core_-_O-antigen_ligase	RbromiiL2-36
Org4_Gene1429	accessory	3844	[RbromiiL2-36]RBL236_01490_hypothetical_protein	RbromiiL2-36
Org4_Gene1431	accessory	438	[RbromiiL2-36]RBL236_01493_hypothetical_protein	RbromiiL2-36
Org4_Gene1432	accessory	1815	[RbromiiL2-36]RBL236_01494_DegV_domain-containing_protein	RbromiiL2-36
Org4_Gene1433	accessory	987	[RbromiiL2-36]RBL236_01495_hypothetical_protein	RbromiiL2-36
Org4_Gene1434	accessory	3045	[RbromiiL2-36]RBL236_01496_hypothetical_protein	RbromiiL2-36
Org4_Gene1439	accessory	2602	[RbromiiL2-36]RBL236_01502_Thiamine_pyrophosphokinase	RbromiiL2-36
Org4_Gene1444	accessory	779	[RbromiiL2-36]RBL236_01507_Ribosomal_RNA_small_subunit_methyltransferase_B	RbromiiL2-36
Org4_Gene1450	accessory	2761	[RbromiiL2-36]RBL236_01513_Guanylate_kinase	RbromiiL2-36
Org4_Gene1454	accessory	797	[RbromiiL2-36]RBL236_01517_hypothetical_protein	RbromiiL2-36
Org4_Gene1457	accessory	430	[RbromiiL2-36]RBL236_01520_Phosphoenolpyruvate-protein_phosphotransferase	RbromiiL2-36
Org4_Gene1459	accessory	308	[RbromiiL2-36]RBL236_01522_EIIABC-Fru	RbromiiL2-36
Org4_Gene1460	accessory	1678	[RbromiiL2-36]RBL236_01523_Tagatose-6-phosphate_kinase	RbromiiL2-36
Org4_Gene1461	accessory	2142	[RbromiiL2-36]RBL236_01524_HTH-type_transcriptional_repressor_GlcR	RbromiiL2-36
Org4_Gene1462	accessory	2569	[RbromiiL2-36]RBL236_01525_transcriptional_regulator_FixK	RbromiiL2-36
Org4_Gene1465	accessory	2329	[RbromiiL2-36]RBL236_01528_hypothetical_protein	RbromiiL2-36
Org4_Gene1468	accessory	140	[RbromiiL2-36]RBL236_01531_Penicillin-binding_protein_F	RbromiiL2-36
Org4_Gene1469	accessory	3725	[RbromiiL2-36]RBL236_01532_hypothetical_protein	RbromiiL2-36
Org4_Gene1474	accessory	3163	[RbromiiL2-36]RBL236_01537_Biotin_carboxyl_carrier_protein_of_acetyl-CoA_carboxylase	RbromiiL2-36
Org4_Gene1479	accessory	3314	[RbromiiL2-36]RBL236_01542_putative_membrane_protein	RbromiiL2-36
Org4_Gene1480	accessory	545	[RbromiiL2-36]RBL236_01543_Iron_hydrogenase_1	RbromiiL2-36
Org4_Gene1481	accessory	2134	[RbromiiL2-36]RBL236_01544_vancomycin_high_temperature_exclusion_protein	RbromiiL2-36
Org4_Gene1482	accessory	3996	[RbromiiL2-36]RBL236_01545_hypothetical_protein	RbromiiL2-36
Org4_Gene1484	accessory	3028	[RbromiiL2-36]RBL236_01547_hypothetical_protein	RbromiiL2-36
Org4_Gene1485	accessory	4185	[RbromiiL2-36]RBL236_01548_HTH-type_transcriptional_regulator_ImmR	RbromiiL2-36
Org4_Gene1486	accessory	3763	[RbromiiL2-36]RBL236_01549_Endoglucanase_A_precursor	RbromiiL2-36
Org4_Gene149	accessory	69	[RbromiiL2-36]RBL236_00152_Alpha-amylase_precursor	RbromiiL2-36
Org4_Gene1492	accessory	3989	[RbromiiL2-36]RBL236_01555_Preprotein_translocase_subunit_SecE	RbromiiL2-36
Org4_Gene150	accessory	2808	[RbromiiL2-36]RBL236_00153_hypothetical_protein	RbromiiL2-36
Org4_Gene1500	accessory	2099	[RbromiiL2-36]RBL236_01564_Endo-1,4-beta-xylanase_Z_precursor	RbromiiL2-36
Org4_Gene1501	accessory	2969	[RbromiiL2-36]RBL236_01565_Protease_synthase_and_sporulation_negative_regulatory_protein_PAI_1	RbromiiL2-36
Org4_Gene1504	accessory	2375	[RbromiiL2-36]RBL236_01568_putative_ABC_transporter_ATP-binding_protein/MT1014	RbromiiL2-36
Org4_Gene1505	accessory	57	[RbromiiL2-36]RBL236_01569_outer_membrane-specific_lipoprotein_transporter_subunit_LolE	RbromiiL2-36
Org4_Gene151	accessory	1392	[RbromiiL2-36]RBL236_00154_Rod_shape-determining_protein_MreB	RbromiiL2-36
Org4_Gene1510	accessory	3369	[RbromiiL2-36]RBL236_01574_hypothetical_protein	RbromiiL2-36
Org4_Gene1511	accessory	2981	[RbromiiL2-36]RBL236_01575_anhydro-N-acetylmuramic_acid_kinase	RbromiiL2-36
Org4_Gene1513	accessory	1642	[RbromiiL2-36]RBL236_01577_Phosphate-binding_protein_PstS_2_precursor	RbromiiL2-36
Org4_Gene1514	accessory	1802	[RbromiiL2-36]RBL236_01578_Phosphate_transport_system_permease_protein_PstC	RbromiiL2-36
Org4_Gene1515	accessory	1612	[RbromiiL2-36]RBL236_01579_Phosphate_transport_system_permease_protein_PstA	RbromiiL2-36
Org4_Gene1517	accessory	2551	[RbromiiL2-36]RBL236_01581_Phosphate_uptake_regulator	RbromiiL2-36
Org4_Gene1518	accessory	2472	[RbromiiL2-36]RBL236_01582_Sensory_transduction_protein_regX3	RbromiiL2-36
Org4_Gene1519	accessory	466	[RbromiiL2-36]RBL236_01583_Alkaline_phosphatase_synthesis_sensor_protein_PhoR	RbromiiL2-36
Org4_Gene152	accessory	2496	[RbromiiL2-36]RBL236_00155_DNA_utilization_protein_GntX	RbromiiL2-36
Org4_Gene1520	accessory	369	[RbromiiL2-36]RBL236_01584_PhoU-like_phosphate_uptake_regulator	RbromiiL2-36
Org4_Gene1521	accessory	2554	[RbromiiL2-36]RBL236_01585_hemolysin	RbromiiL2-36
Org4_Gene1522	accessory	2321	[RbromiiL2-36]RBL236_01586_ABC-type_transporter_ATP-binding_protein_EcsA	RbromiiL2-36
Org4_Gene1523	accessory	509	[RbromiiL2-36]RBL236_01587_putative_permease	RbromiiL2-36
Org4_Gene1525	accessory	1140	[RbromiiL2-36]RBL236_01589_hypothetical_protein	RbromiiL2-36
Org4_Gene1526	accessory	2951	[RbromiiL2-36]RBL236_01590_serine/threonine_protein_kinase	RbromiiL2-36
Org4_Gene1527	accessory	3229	[RbromiiL2-36]RBL236_01591_hypothetical_protein	RbromiiL2-36
Org4_Gene1528	accessory	3462	[RbromiiL2-36]RBL236_01592_putative_manganese-dependent_inorganic_pyrophosphatase	RbromiiL2-36
Org4_Gene1529	accessory	2433	[RbromiiL2-36]RBL236_01593_Staphylococcal_respiratory_response_protein_A	RbromiiL2-36
Org4_Gene1530	accessory	417	[RbromiiL2-36]RBL236_01594_Sensor_protein_QseC	RbromiiL2-36
Org4_Gene1531	accessory	1112	[RbromiiL2-36]RBL236_01595_hypothetical_protein	RbromiiL2-36
Org4_Gene1532	accessory	2886	[RbromiiL2-36]RBL236_01596_Prolyl-tRNA_deacylase_ProX	RbromiiL2-36
Org4_Gene1533	accessory	219	[RbromiiL2-36]RBL236_01597_hypothetical_protein	RbromiiL2-36
Org4_Gene1534	accessory	1716	[RbromiiL2-36]RBL236_01598_ABC_transporter_ATP-binding_protein_YtrB	RbromiiL2-36
Org4_Gene1535	accessory	3536	[RbromiiL2-36]RBL236_01599_HTH-type_transcriptional_repressor_YtrA	RbromiiL2-36
Org4_Gene1538	accessory	2121	[RbromiiL2-36]RBL236_01602_Glycine/sarcosine_N-methyltransferase	RbromiiL2-36
Org4_Gene154	accessory	2287	[RbromiiL2-36]RBL236_00157_putative_glutamine_amidotransferase	RbromiiL2-36
Org4_Gene1543	accessory	1421	[RbromiiL2-36]RBL236_01610_ABC-type_uncharacterized_transport_system,_periplasmic_component	RbromiiL2-36
Org4_Gene1545	accessory	995	[RbromiiL2-36]RBL236_01612_Exodeoxyribonuclease_7_large_subunit	RbromiiL2-36
Org4_Gene1547	accessory	3081	[RbromiiL2-36]RBL236_01614_hypothetical_protein	RbromiiL2-36
Org4_Gene1548	accessory	2903	[RbromiiL2-36]RBL236_01615_SpoIIIAH-like_protein	RbromiiL2-36
Org4_Gene1550	accessory	3177	[RbromiiL2-36]RBL236_01617_hypothetical_protein	RbromiiL2-36
Org4_Gene1551	accessory	1199	[RbromiiL2-36]RBL236_01618_stage_III_sporulation_protein_AE	RbromiiL2-36
Org4_Gene1552	accessory	3501	[RbromiiL2-36]RBL236_01619_stage_III_sporulation_protein_AD	RbromiiL2-36
Org4_Gene1554	accessory	3075	[RbromiiL2-36]RBL236_01621_stage_III_sporulation_protein_SpoAB	RbromiiL2-36
Org4_Gene1555	accessory	1549	[RbromiiL2-36]RBL236_01622_stage_III_sporulation_protein_AA	RbromiiL2-36
Org4_Gene1556	accessory	3034	[RbromiiL2-36]RBL236_01623_preprotein_translocase_subunit_SecD	RbromiiL2-36
Org4_Gene1559	accessory	1561	[RbromiiL2-36]RBL236_01626_hypothetical_protein	RbromiiL2-36
Org4_Gene156	accessory	4251	[RbromiiL2-36]RBL236_00159_hypothetical_protein	RbromiiL2-36
Org4_Gene1563	accessory	3277	[RbromiiL2-36]RBL236_01630_Septum_formation_initiator	RbromiiL2-36
Org4_Gene1568	accessory	345	[RbromiiL2-36]RBL236_01635_Stage_III_sporulation_protein_F	RbromiiL2-36
Org4_Gene1569	accessory	3434	[RbromiiL2-36]RBL236_01636_hypothetical_protein	RbromiiL2-36
Org4_Gene157	accessory	2697	[RbromiiL2-36]RBL236_00160_Putative_acetyltransferase	RbromiiL2-36
Org4_Gene1574	accessory	267	[RbromiiL2-36]RBL236_01641_Single-stranded-DNA-specific_exonuclease_RecJ	RbromiiL2-36
Org4_Gene1576	accessory	3272	[RbromiiL2-36]RBL236_01643_Arginine_hydroxamate_resistance_protein	RbromiiL2-36
Org4_Gene1577	accessory	1849	[RbromiiL2-36]RBL236_01644_putative_inorganic_polyphosphate/ATP-NAD_kinase	RbromiiL2-36
Org4_Gene1581	accessory	4117	[RbromiiL2-36]RBL236_01648_exodeoxyribonuclease_VII_small_subunit	RbromiiL2-36
Org4_Gene1586	accessory	2738	[RbromiiL2-36]RBL236_01653_germination_protein,_Ger(x)C_family	RbromiiL2-36
Org4_Gene1587	accessory	1290	[RbromiiL2-36]RBL236_01654_spore_germination_protein_(amino_acid_permease)	RbromiiL2-36
Org4_Gene1589	accessory	2312	[RbromiiL2-36]RBL236_01656_hypothetical_protein	RbromiiL2-36
Org4_Gene159	accessory	2667	[RbromiiL2-36]RBL236_00162_Undecaprenyl-diphosphatase_BcrC	RbromiiL2-36
Org4_Gene1590	accessory	3524	[RbromiiL2-36]RBL236_01657_hypothetical_protein	RbromiiL2-36
Org4_Gene1591	accessory	1915	[RbromiiL2-36]RBL236_01658_High-affinity_zinc_uptake_system_membrane_protein_ZnuB	RbromiiL2-36
Org4_Gene1592	accessory	2203	[RbromiiL2-36]RBL236_01659_putative_zinc_transport_system_ATP-binding_protein_AdcC	RbromiiL2-36
Org4_Gene1593	accessory	1456	[RbromiiL2-36]RBL236_01660_putative_zinc_transport_system_zinc-binding_lipoprotein_AdcA_precursor	RbromiiL2-36
Org4_Gene1597	accessory	2289	[RbromiiL2-36]RBL236_01664_Tyrosine-protein_kinase_YwqD	RbromiiL2-36
Org4_Gene1598	accessory	2257	[RbromiiL2-36]RBL236_01665_tyrosine_kinase	RbromiiL2-36
Org4_Gene16	accessory	1725	[RbromiiL2-36]RBL236_00016_hypothetical_protein	RbromiiL2-36
Org4_Gene160	accessory	4123	[RbromiiL2-36]RBL236_00163_hypothetical_protein	RbromiiL2-36
Org4_Gene1600	accessory	3365	[RbromiiL2-36]RBL236_01667_oxaloacetate_decarboxylase_subunit_gamma	RbromiiL2-36
Org4_Gene1602	accessory	564	[RbromiiL2-36]RBL236_01669_hypothetical_protein	RbromiiL2-36
Org4_Gene1603	accessory	4437	[RbromiiL2-36]RBL236_01670_hypothetical_protein	RbromiiL2-36
Org4_Gene1605	accessory	2236	[RbromiiL2-36]RBL236_01672_hypothetical_protein	RbromiiL2-36
Org4_Gene1606	accessory	245	[RbromiiL2-36]RBL236_01673_Subtilisin_BL	RbromiiL2-36
Org4_Gene1608	accessory	4192	[RbromiiL2-36]RBL236_01675_hypothetical_protein	RbromiiL2-36
Org4_Gene1610	accessory	3944	[RbromiiL2-36]RBL236_01677_Toxin_RelG	RbromiiL2-36
Org4_Gene1611	accessory	4135	[RbromiiL2-36]RBL236_01678_hypothetical_protein	RbromiiL2-36
Org4_Gene1613	accessory	3899	[RbromiiL2-36]RBL236_01681_hypothetical_protein	RbromiiL2-36
Org4_Gene1616	accessory	4415	[RbromiiL2-36]RBL236_01684_hypothetical_protein	RbromiiL2-36
Org4_Gene1619	accessory	901	[RbromiiL2-36]RBL236_01687_Teichoic_acids_export_ATP-binding_protein_TagH	RbromiiL2-36
Org4_Gene162	accessory	713	[RbromiiL2-36]RBL236_00166_dihydrolipoamide_dehydrogenase	RbromiiL2-36
Org4_Gene1620	accessory	1891	[RbromiiL2-36]RBL236_01688_Teichoic_acid_translocation_permease_protein_TagG	RbromiiL2-36
Org4_Gene1621	accessory	2471	[RbromiiL2-36]RBL236_01689_Peptidyl-prolyl_cis-trans_isomerase_A_precursor	RbromiiL2-36
Org4_Gene1622	accessory	3179	[RbromiiL2-36]RBL236_01690_hypothetical_protein	RbromiiL2-36
Org4_Gene1623	accessory	959	[RbromiiL2-36]RBL236_01691_Beta-hexosaminidase_A_precursor	RbromiiL2-36
Org4_Gene1624	accessory	273	[RbromiiL2-36]RBL236_01692_Bacterial_Ig-like_domain_(group_2)	RbromiiL2-36

Org4_Gene1625	accessory	3109	[RbromiiL2-36]RBL236_01693_CarD-like/TRCF_domain_protein	RbromiiL2-36
Org4_Gene1627	accessory	3983	[RbromiiL2-36]RBL236_01695_hypothetical_protein	RbromiiL2-36
Org4_Gene1628	accessory	4127	[RbromiiL2-36]RBL236_01696_hypothetical_protein	RbromiiL2-36
Org4_Gene163	accessory	395	[RbromiiL2-36]RBL236_00169_DNA_polymerase_III_subunit_gamma/tau	RbromiiL2-36
Org4_Gene1631	accessory	3492	[RbromiiL2-36]RBL236_01699_Putative_PBSX_repressor	RbromiiL2-36
Org4_Gene1632	accessory	1839	[RbromiiL2-36]RBL236_01701_putative_integral_membrane_protein	RbromiiL2-36
Org4_Gene1637	accessory	2628	[RbromiiL2-36]RBL236_01706_CDP-diacylglycerol--glycerol-3-phosphate_3-phosphatidyltransferase	RbromiiL2-36
Org4_Gene1638	accessory	2453	[RbromiiL2-36]RBL236_01707_Colicin_V_production_protein	RbromiiL2-36
Org4_Gene1639	accessory	865	[RbromiiL2-36]RBL236_01708_hypothetical_protein	RbromiiL2-36
Org4_Gene1649	accessory	3575	[RbromiiL2-36]RBL236_01718_hypothetical_protein	RbromiiL2-36
Org4_Gene1651	accessory	3090	[RbromiiL2-36]RBL236_01720_hypothetical_protein	RbromiiL2-36
Org4_Gene1654	accessory	4435	[RbromiiL2-36]RBL236_01723_hypothetical_protein	RbromiiL2-36
Org4_Gene1655	accessory	1020	[RbromiiL2-36]RBL236_01724_FtsX-like_permease_family_protein	RbromiiL2-36
Org4_Gene1656	accessory	2439	[RbromiiL2-36]RBL236_01725_Lipoprotein-releasing_system_ATP-binding_protein_LolD	RbromiiL2-36
Org4_Gene1657	accessory	773	[RbromiiL2-36]RBL236_01726_FtsX-like_permease_family_protein	RbromiiL2-36
Org4_Gene1658	accessory	1911	[RbromiiL2-36]RBL236_01733_putative_membrane_protein	RbromiiL2-36
Org4_Gene1659	accessory	1967	[RbromiiL2-36]RBL236_01735_Hydroxyethylthiazole_kinase	RbromiiL2-36
Org4_Gene1660	accessory	2565	[RbromiiL2-36]RBL236_01736_Thiamine-phosphate_synthase	RbromiiL2-36
Org4_Gene1661	accessory	2498	[RbromiiL2-36]RBL236_01737_Phosphorylated_carbohydrates_phosphatase	RbromiiL2-36
Org4_Gene1662	accessory	1982	[RbromiiL2-36]RBL236_01738_Hydroxymethylpyrimidine/phosphomethylpyrimidine_kinase	RbromiiL2-36
Org4_Gene1663	accessory	1032	[RbromiiL2-36]RBL236_01739_Cytosine_permease	RbromiiL2-36
Org4_Gene1664	accessory	208	[RbromiiL2-36]RBL236_01740_Stage_V_sporulation_protein_T	RbromiiL2-36
Org4_Gene1665	accessory	1246	[RbromiiL2-36]RBL236_01741_hypothetical_protein	RbromiiL2-36
Org4_Gene1666	accessory	3063	[RbromiiL2-36]RBL236_01742_nicotinamidase/pyrazinamidase	RbromiiL2-36
Org4_Gene1667	accessory	2950	[RbromiiL2-36]RBL236_01743_2,3-bisphosphoglycerate-dependent_phosphoglycerate_mutase	RbromiiL2-36
Org4_Gene1668	accessory	788	[RbromiiL2-36]RBL236_01744_Ktr_system_potassium_uptake_protein_B	RbromiiL2-36
Org4_Gene1669	accessory	2553	[RbromiiL2-36]RBL236_01745_Ktr_system_potassium_uptake_protein_A	RbromiiL2-36
Org4_Gene167	accessory	1614	[RbromiiL2-36]RBL236_00173_hypothetical_protein	RbromiiL2-36
Org4_Gene1670	accessory	4010	[RbromiiL2-36]RBL236_01746_hypothetical_protein	RbromiiL2-36
Org4_Gene1671	accessory	286	[RbromiiL2-36]RBL236_01747_Sensor_protein_KdpD	RbromiiL2-36
Org4_Gene1672	accessory	2378	[RbromiiL2-36]RBL236_01748_KDP_operon_transcriptional_regulatory_protein_KdpE	RbromiiL2-36
Org4_Gene1673	accessory	1029	[RbromiiL2-36]RBL236_01749_Integrase	RbromiiL2-36
Org4_Gene1674	accessory	3450	[RbromiiL2-36]RBL236_01750_hypothetical_protein	RbromiiL2-36
Org4_Gene1675	accessory	2811	[RbromiiL2-36]RBL236_01751_hypothetical_protein	RbromiiL2-36
Org4_Gene1676	accessory	2415	[RbromiiL2-36]RBL236_01752_HTH-type_transcriptional_regulator_ImmR	RbromiiL2-36
Org4_Gene1677	accessory	4082	[RbromiiL2-36]RBL236_01753_Helix-turn-helix_domain_protein	RbromiiL2-36
Org4_Gene1678	accessory	4144	[RbromiiL2-36]RBL236_01754_hypothetical_protein	RbromiiL2-36
Org4_Gene1679	accessory	4290	[RbromiiL2-36]RBL236_01755_hypothetical_protein	RbromiiL2-36
Org4_Gene168	accessory	4311	[RbromiiL2-36]RBL236_00175_hypothetical_protein	RbromiiL2-36
Org4_Gene1680	accessory	4097	[RbromiiL2-36]RBL236_01756_hypothetical_protein	RbromiiL2-36
Org4_Gene1681	accessory	4181	[RbromiiL2-36]RBL236_01757_helix-turn-helix_protein	RbromiiL2-36
Org4_Gene1682	accessory	4266	[RbromiiL2-36]RBL236_01758_hypothetical_protein	RbromiiL2-36
Org4_Gene1683	accessory	4228	[RbromiiL2-36]RBL236_01759_DNA_binding_domain,_excisionase_family	RbromiiL2-36
Org4_Gene1684	accessory	4073	[RbromiiL2-36]RBL236_01760_hypothetical_protein	RbromiiL2-36
Org4_Gene1685	accessory	3301	[RbromiiL2-36]RBL236_01761_hypothetical_protein	RbromiiL2-36
Org4_Gene1686	accessory	549	[RbromiiL2-36]RBL236_01762_hypothetical_protein	RbromiiL2-36
Org4_Gene1687	accessory	1474	[RbromiiL2-36]RBL236_01763_hypothetical_protein	RbromiiL2-36
Org4_Gene1688	accessory	2716	[RbromiiL2-36]RBL236_01764_Siphovirus_Gp157	RbromiiL2-36
Org4_Gene1689	accessory	1673	[RbromiiL2-36]RBL236_01765_hypothetical_protein	RbromiiL2-36
Org4_Gene169	accessory	3544	[RbromiiL2-36]RBL236_00176_hypothetical_protein	RbromiiL2-36
Org4_Gene1690	accessory	784	[RbromiiL2-36]RBL236_01766_type_I_restriction_enzyme_EcoKI_subunit_R	RbromiiL2-36
Org4_Gene1691	accessory	3784	[RbromiiL2-36]RBL236_01767_VRR-NUC_domain_protein	RbromiiL2-36
Org4_Gene1692	accessory	2845	[RbromiiL2-36]RBL236_01768_hypothetical_protein	RbromiiL2-36
Org4_Gene1693	accessory	186	[RbromiiL2-36]RBL236_01769_hypothetical_protein	RbromiiL2-36
Org4_Gene1694	accessory	3417	[RbromiiL2-36]RBL236_01770_hypothetical_protein	RbromiiL2-36
Org4_Gene1695	accessory	3071	[RbromiiL2-36]RBL236_01771_hypothetical_protein	RbromiiL2-36
Org4_Gene1696	accessory	4336	[RbromiiL2-36]RBL236_01772_hypothetical_protein	RbromiiL2-36
Org4_Gene1697	accessory	3414	[RbromiiL2-36]RBL236_01773_hypothetical_protein	RbromiiL2-36
Org4_Gene1698	accessory	471	[RbromiiL2-36]RBL236_01774_phage_terminase,_large_subunit,_PBSX_family	RbromiiL2-36
Org4_Gene1699	accessory	814	[RbromiiL2-36]RBL236_01775_phage_portal_protein,_putative,_A118_family	RbromiiL2-36
Org4_Gene17	accessory	1295	[RbromiiL2-36]RBL236_00017_glycosyltransferase	RbromiiL2-36
Org4_Gene170	accessory	3775	[RbromiiL2-36]RBL236_00177_Adenine-specific_DNA_methylase	RbromiiL2-36
Org4_Gene1700	accessory	269	[RbromiiL2-36]RBL236_01776_Putative_ADP-ribosyltransferase_Certhrax	RbromiiL2-36
Org4_Gene1701	accessory	3749	[RbromiiL2-36]RBL236_01777_hypothetical_protein	RbromiiL2-36
Org4_Gene1702	accessory	3436	[RbromiiL2-36]RBL236_01778_hypothetical_protein	RbromiiL2-36
Org4_Gene1703	accessory	4218	[RbromiiL2-36]RBL236_01779_hypothetical_protein	RbromiiL2-36
Org4_Gene1704	accessory	2823	[RbromiiL2-36]RBL236_01780_Phage_minor_structural_protein_GP20	RbromiiL2-36
Org4_Gene1705	accessory	1784	[RbromiiL2-36]RBL236_01781_hypothetical_protein	RbromiiL2-36
Org4_Gene1706	accessory	3561	[RbromiiL2-36]RBL236_01782_hypothetical_protein	RbromiiL2-36
Org4_Gene1707	accessory	3372	[RbromiiL2-36]RBL236_01783_hypothetical_protein	RbromiiL2-36
Org4_Gene1708	accessory	3128	[RbromiiL2-36]RBL236_01784_hypothetical_protein	RbromiiL2-36
Org4_Gene1709	accessory	3298	[RbromiiL2-36]RBL236_01785_hypothetical_protein	RbromiiL2-36
Org4_Gene171	accessory	4264	[RbromiiL2-36]RBL236_00178_hypothetical_protein	RbromiiL2-36
Org4_Gene1710	accessory	3051	[RbromiiL2-36]RBL236_01786_hypothetical_protein	RbromiiL2-36
Org4_Gene1711	accessory	3385	[RbromiiL2-36]RBL236_01787_hypothetical_protein	RbromiiL2-36
Org4_Gene1712	accessory	2891	[RbromiiL2-36]RBL236_01788_Bacteriophage_Gp15_protein	RbromiiL2-36
Org4_Gene1713	accessory	2710	[RbromiiL2-36]RBL236_01789_hypothetical_protein	RbromiiL2-36
Org4_Gene1714	accessory	43	[RbromiiL2-36]RBL236_01790_Phage-related_protein	RbromiiL2-36
Org4_Gene1715	accessory	3392	[RbromiiL2-36]RBL236_01791_hypothetical_protein	RbromiiL2-36
Org4_Gene1716	accessory	870	[RbromiiL2-36]RBL236_01792_hypothetical_protein	RbromiiL2-36
Org4_Gene1717	accessory	2127	[RbromiiL2-36]RBL236_01793_hypothetical_protein	RbromiiL2-36
Org4_Gene1719	accessory	3262	[RbromiiL2-36]RBL236_01795_toxin_secretion/phage_lysis_holin	RbromiiL2-36
Org4_Gene172	accessory	2307	[RbromiiL2-36]RBL236_00179_hypothetical_protein	RbromiiL2-36
Org4_Gene1720	accessory	1910	[RbromiiL2-36]RBL236_01796_N-acetylmuramoyl-L-alanine_amidase	RbromiiL2-36
Org4_Gene1721	accessory	2039	[RbromiiL2-36]RBL236_01801_General_stress_protein_39	RbromiiL2-36
Org4_Gene1722	accessory	4345	[RbromiiL2-36]RBL236_01802_hypothetical_protein	RbromiiL2-36
Org4_Gene1723	accessory	1777	[RbromiiL2-36]RBL236_01803_Dipicolinate_synthase_subunit_A	RbromiiL2-36
Org4_Gene1725	accessory	473	[RbromiiL2-36]RBL236_01805_hypothetical_protein	RbromiiL2-36
Org4_Gene1726	accessory	4177	[RbromiiL2-36]RBL236_01806_putative_zinc_finger/helix-turn-helix_protein,_YgiT_family	RbromiiL2-36
Org4_Gene1727	accessory	670	[RbromiiL2-36]RBL236_01807_hypothetical_protein	RbromiiL2-36
Org4_Gene1728	accessory	2966	[RbromiiL2-36]RBL236_01808_hypothetical_protein	RbromiiL2-36
Org4_Gene173	accessory	2376	[RbromiiL2-36]RBL236_00180_AlwI_restriction_endonuclease	RbromiiL2-36
Org4_Gene1735	accessory	2261	[RbromiiL2-36]RBL236_01815_Stage_II_sporulation_protein_Q	RbromiiL2-36
Org4_Gene1736	accessory	2592	[RbromiiL2-36]RBL236_01816_cytidylate_kinase	RbromiiL2-36
Org4_Gene1737	accessory	2543	[RbromiiL2-36]RBL236_01817_5'-nucleotidase	RbromiiL2-36
Org4_Gene1738	accessory	504	[RbromiiL2-36]RBL236_01818_Lipid_A_core_-_O-antigen_ligase	RbromiiL2-36
Org4_Gene1739	accessory	1828	[RbromiiL2-36]RBL236_01819_Poly-beta-1,6-N-acetyl-D-glucosamine_N-deacetylase_precursor	RbromiiL2-36
Org4_Gene174	accessory	237	[RbromiiL2-36]RBL236_00181_hypothetical_protein	RbromiiL2-36
Org4_Gene1740	accessory	3483	[RbromiiL2-36]RBL236_01820_Peroxide-responsive_repressor_PerR	RbromiiL2-36
Org4_Gene1742	accessory	4372	[RbromiiL2-36]RBL236_01822_Rubredoxin	RbromiiL2-36
Org4_Gene1744	accessory	1208	[RbromiiL2-36]RBL236_01824_hypothetical_protein	RbromiiL2-36
Org4_Gene1745	accessory	357	[RbromiiL2-36]RBL236_01825_ATP-dependent_DNA_helicase_RecQ	RbromiiL2-36
Org4_Gene1746	accessory	3546	[RbromiiL2-36]RBL236_01826_hypothetical_protein	RbromiiL2-36
Org4_Gene1747	accessory	3506	[RbromiiL2-36]RBL236_01827_hypothetical_protein	RbromiiL2-36
Org4_Gene1748	accessory	1086	[RbromiiL2-36]RBL236_01828_hypothetical_protein	RbromiiL2-36
Org4_Gene1749	accessory	534	[RbromiiL2-36]RBL236_01829_hypothetical_protein	RbromiiL2-36
Org4_Gene175	accessory	2417	[RbromiiL2-36]RBL236_00182_hypothetical_protein	RbromiiL2-36
Org4_Gene1750	accessory	2754	[RbromiiL2-36]RBL236_01830_hypothetical_protein	RbromiiL2-36
Org4_Gene1751	accessory	3507	[RbromiiL2-36]RBL236_01831_hypothetical_protein	RbromiiL2-36
Org4_Gene1752	accessory	3446	[RbromiiL2-36]RBL236_01832_hypothetical_protein	RbromiiL2-36
Org4_Gene1753	accessory	2580	[RbromiiL2-36]RBL236_01833_Response_regulator_protein_VraR	RbromiiL2-36
Org4_Gene1754	accessory	847	[RbromiiL2-36]RBL236_01834_sensory_histidine_kinase_UhpB	RbromiiL2-36
Org4_Gene1755	accessory	454	[RbromiiL2-36]RBL236_01835_hypothetical_protein	RbromiiL2-36
Org4_Gene1757	accessory	162	[RbromiiL2-36]RBL236_01837_Fibronectin_type_III_domain_protein	RbromiiL2-36
Org4_Gene1758	accessory	351	[RbromiiL2-36]RBL236_01838_Fibronectin_type_III_domain_protein	RbromiiL2-36
Org4_Gene176	accessory	1305	[RbromiiL2-36]RBL236_00183_hypothetical_protein	RbromiiL2-36
Org4_Gene1762	accessory	947	[RbromiiL2-36]RBL236_01842_hypothetical_protein	RbromiiL2-36
Org4_Gene1763	accessory	185	[RbromiiL2-36]RBL236_01843_Cadmium,_zinc_and_cobalt-transporting_ATPase	RbromiiL2-36
Org4_Gene177	accessory	602	[RbromiiL2-36]RBL236_00184_hypothetical_protein	RbromiiL2-36
Org4_Gene1770	accessory	4154	[RbromiiL2-36]RBL236_01850_hypothetical_protein	RbromiiL2-36
Org4_Gene1771	accessory	41	[RbromiiL2-36]RBL236_01851_Pullulanase_precursor	RbromiiL2-36
Org4_Gene1774	accessory	3059	[RbromiiL2-36]RBL236_01854_hypothetical_protein	RbromiiL2-36

Org4_Gene1775	accessory	2006	[RbromiiL2-36]RBL236_01855_sortase,_SrtB_family	RbromiiL2-36
Org4_Gene1776	accessory	816	[RbromiiL2-36]RBL236_01856_Putative_transcriptional_regulator_YvhJ	RbromiiL2-36
Org4_Gene1779	accessory	143	[RbromiiL2-36]RBL236_01859_putative_membrane_protein	RbromiiL2-36
Org4_Gene178	accessory	1739	[RbromiiL2-36]RBL236_00185_RNA_polymerase_sigma_factor,_sigma-70_family	RbromiiL2-36
Org4_Gene1781	accessory	2081	[RbromiiL2-36]RBL236_01861_hypothetical_protein	RbromiiL2-36
Org4_Gene1784	accessory	2274	[RbromiiL2-36]RBL236_01864_DNA_alkylation_repair_enzyme	RbromiiL2-36
Org4_Gene1785	accessory	2502	[RbromiiL2-36]RBL236_01866_Riboflavin_ECF_transporter_S_component_RibU	RbromiiL2-36
Org4_Gene1786	accessory	2798	[RbromiiL2-36]RBL236_01868_Folate_ECF_transporter_S_component_FolT	RbromiiL2-36
Org4_Gene179	accessory	1168	[RbromiiL2-36]RBL236_00186_hypothetical_protein	RbromiiL2-36
Org4_Gene1790	accessory	311	[RbromiiL2-36]RBL236_01872_1,4-alpha-glucan_branching_enzyme_GlgB	RbromiiL2-36
Org4_Gene1795	accessory	3284	[RbromiiL2-36]RBL236_01877_Acyl-CoA_thioester_hydrolase_YbgC	RbromiiL2-36
Org4_Gene1798	accessory	1661	[RbromiiL2-36]RBL236_01880_HTH-type_transcriptional_regulator_GltC	RbromiiL2-36
Org4_Gene18	accessory	425	[RbromiiL2-36]RBL236_00018_Alpha-amylase_precursor	RbromiiL2-36
Org4_Gene180	accessory	4069	[RbromiiL2-36]RBL236_00187_hypothetical_protein	RbromiiL2-36
Org4_Gene1800	accessory	2392	[RbromiiL2-36]RBL236_01882_putative_flavoprotein	RbromiiL2-36
Org4_Gene1801	accessory	879	[RbromiiL2-36]RBL236_01883_Alpha-1,4-glucan:maltose-1-phosphate_maltosyltransferase_1	RbromiiL2-36
Org4_Gene1802	accessory	2825	[RbromiiL2-36]RBL236_01884_Putative_ribosomal_N-acetyltransferase_YdaF	RbromiiL2-36
Org4_Gene1803	accessory	3591	[RbromiiL2-36]RBL236_01885_hypothetical_protein	RbromiiL2-36
Org4_Gene1805	accessory	634	[RbromiiL2-36]RBL236_01887_Staphylococcal_virulence_regulator_protein_A	RbromiiL2-36
Org4_Gene1806	accessory	3490	[RbromiiL2-36]RBL236_01888_hypothetical_protein	RbromiiL2-36
Org4_Gene1807	accessory	1481	[RbromiiL2-36]RBL236_01889_1,5-anhydro-D-fructose_reductase	RbromiiL2-36
Org4_Gene1808	accessory	1071	[RbromiiL2-36]RBL236_01890_NADH_peroxidase	RbromiiL2-36
Org4_Gene1809	accessory	1342	[RbromiiL2-36]RBL236_01891_Sulfate_starvation-induced_protein_2	RbromiiL2-36
Org4_Gene181	accessory	1613	[RbromiiL2-36]RBL236_00188_hypothetical_protein	RbromiiL2-36
Org4_Gene1810	accessory	1867	[RbromiiL2-36]RBL236_01892_Sulfate_transport_system_permease_protein_CysT	RbromiiL2-36
Org4_Gene1811	accessory	1971	[RbromiiL2-36]RBL236_01893_Sulfate_transport_system_permease_protein_CysW	RbromiiL2-36
Org4_Gene1812	accessory	1303	[RbromiiL2-36]RBL236_01894_Sulfate/thiosulfate_import_ATP-binding_protein_CysA	RbromiiL2-36
Org4_Gene1813	accessory	3037	[RbromiiL2-36]RBL236_01895_Zeta_toxin	RbromiiL2-36
Org4_Gene1815	accessory	1165	[RbromiiL2-36]RBL236_01897_Glutamyl-tRNA_reductase	RbromiiL2-36
Org4_Gene1816	accessory	2659	[RbromiiL2-36]RBL236_01898_Siroheme_synthase	RbromiiL2-36
Org4_Gene1817	accessory	1836	[RbromiiL2-36]RBL236_01899_Porphobilinogen_deaminase	RbromiiL2-36
Org4_Gene1818	accessory	591	[RbromiiL2-36]RBL236_01900_Uroporphyrinogen-III_C-methyltransferase	RbromiiL2-36
Org4_Gene1819	accessory	1492	[RbromiiL2-36]RBL236_01901_Delta-aminolevulinic_acid_dehydratase	RbromiiL2-36
Org4_Gene182	accessory	3706	[RbromiiL2-36]RBL236_00189_hypothetical_protein	RbromiiL2-36
Org4_Gene1820	accessory	920	[RbromiiL2-36]RBL236_01902_Glutamate-1-semialdehyde_2,1-aminomutase_2	RbromiiL2-36
Org4_Gene1821	accessory	3248	[RbromiiL2-36]RBL236_01903_HTH-type_transcriptional_regulator_IscR	RbromiiL2-36
Org4_Gene1822	accessory	940	[RbromiiL2-36]RBL236_01904_Methionine_gamma-lyase	RbromiiL2-36
Org4_Gene1823	accessory	1835	[RbromiiL2-36]RBL236_01905_Anaerobic_sulfite_reductase_subunit_C	RbromiiL2-36
Org4_Gene1824	accessory	4035	[RbromiiL2-36]RBL236_01906_SirA-like_protein	RbromiiL2-36
Org4_Gene1825	accessory	3759	[RbromiiL2-36]RBL236_01907_Thioredoxin-M	RbromiiL2-36
Org4_Gene1826	accessory	4189	[RbromiiL2-36]RBL236_01908_bifunctional_sulfur_carrier_protein/thiazole_synthase_protein	RbromiiL2-36
Org4_Gene1827	accessory	2001	[RbromiiL2-36]RBL236_01909_putative_adenylyltransferase/sulfurtransferase_MoeZ	RbromiiL2-36
Org4_Gene1828	accessory	3425	[RbromiiL2-36]RBL236_01910_CysO-cysteine_peptidase	RbromiiL2-36
Org4_Gene1829	accessory	1657	[RbromiiL2-36]RBL236_01911_Thioredoxin_reductase	RbromiiL2-36
Org4_Gene183	accessory	3610	[RbromiiL2-36]RBL236_00190_hypothetical_protein	RbromiiL2-36
Org4_Gene1830	accessory	442	[RbromiiL2-36]RBL236_01912_Fumarate_reductase_flavoprotein_subunit_precursor	RbromiiL2-36
Org4_Gene1831	accessory	3783	[RbromiiL2-36]RBL236_01913_Ferredoxin_II	RbromiiL2-36
Org4_Gene1832	accessory	1700	[RbromiiL2-36]RBL236_01914_Sulfate_adenylyltransferase_subunit_2	RbromiiL2-36
Org4_Gene1833	accessory	439	[RbromiiL2-36]RBL236_01915_Sulfate_adenylyltransferase_subunit_1	RbromiiL2-36
Org4_Gene1834	accessory	4217	[RbromiiL2-36]RBL236_01916_TM2_domain-containing_protein	RbromiiL2-36
Org4_Gene1835	accessory	3515	[RbromiiL2-36]RBL236_01917_hypothetical_protein	RbromiiL2-36
Org4_Gene1836	accessory	2955	[RbromiiL2-36]RBL236_01918_Methylated-DNA--protein-cysteine_methyltransferase,_constitutive	RbromiiL2-36
Org4_Gene1837	accessory	893	[RbromiiL2-36]RBL236_01919_hypothetical_protein	RbromiiL2-36
Org4_Gene1838	accessory	2352	[RbromiiL2-36]RBL236_01920_Sensory_transduction_protein_LytR	RbromiiL2-36
Org4_Gene1839	accessory	3261	[RbromiiL2-36]RBL236_01921_hypothetical_protein	RbromiiL2-36
Org4_Gene184	accessory	692	[RbromiiL2-36]RBL236_00191_Relaxase/Mobilisation_nuclease_domain_protein	RbromiiL2-36
Org4_Gene1840	accessory	2901	[RbromiiL2-36]RBL236_01922_VanZ_like_family_protein	RbromiiL2-36
Org4_Gene1841	accessory	6	[RbromiiL2-36]RBL236_01923_Gramicidin_S_synthase_II	RbromiiL2-36
Org4_Gene1842	accessory	389	[RbromiiL2-36]RBL236_01924_Multidrug_export_protein_MepA	RbromiiL2-36
Org4_Gene1843	accessory	3806	[RbromiiL2-36]RBL236_01925_Stage_II_sporulation_protein_AA	RbromiiL2-36
Org4_Gene1844	accessory	3468	[RbromiiL2-36]RBL236_01926_serine-protein_kinase_RsbW	RbromiiL2-36
Org4_Gene1845	accessory	1952	[RbromiiL2-36]RBL236_01927_hypothetical_protein	RbromiiL2-36
Org4_Gene1846	accessory	3745	[RbromiiL2-36]RBL236_01928_hypothetical_protein	RbromiiL2-36
Org4_Gene1847	accessory	2215	[RbromiiL2-36]RBL236_01929_Inner_membrane_protein_YgaZ	RbromiiL2-36
Org4_Gene1848	accessory	3815	[RbromiiL2-36]RBL236_01930_putative_membrane_protein	RbromiiL2-36
Org4_Gene1849	accessory	1104	[RbromiiL2-36]RBL236_01931_Nuclease_SbcCD_subunit_D	RbromiiL2-36
Org4_Gene185	accessory	1881	[RbromiiL2-36]RBL236_00192_putative_chromosome-partitioning_protein_ParB	RbromiiL2-36
Org4_Gene1850	accessory	73	[RbromiiL2-36]RBL236_01932_Nuclease_SbcCD_subunit_C	RbromiiL2-36
Org4_Gene1851	accessory	2100	[RbromiiL2-36]RBL236_01933_Zinc_transporter_ZupT	RbromiiL2-36
Org4_Gene1852	accessory	3997	[RbromiiL2-36]RBL236_01934_hypothetical_protein	RbromiiL2-36
Org4_Gene1854	accessory	2479	[RbromiiL2-36]RBL236_01936_Immunity_protein_SdpI	RbromiiL2-36
Org4_Gene1855	accessory	4003	[RbromiiL2-36]RBL236_01937_Transcriptional_repressor_SdpR	RbromiiL2-36
Org4_Gene1856	accessory	858	[RbromiiL2-36]RBL236_01938_Enolase	RbromiiL2-36
Org4_Gene1857	accessory	742	[RbromiiL2-36]RBL236_01939_Staphylococcal_virulence_regulator_protein_A	RbromiiL2-36
Org4_Gene1858	accessory	2826	[RbromiiL2-36]RBL236_01940_hypothetical_protein	RbromiiL2-36
Org4_Gene1859	accessory	2632	[RbromiiL2-36]RBL236_01941_Uracil_DNA_glycosylase_superfamily_protein	RbromiiL2-36
Org4_Gene186	accessory	559	[RbromiiL2-36]RBL236_00193_V-type_ATP_synthase_subunit_E	RbromiiL2-36
Org4_Gene1860	accessory	1220	[RbromiiL2-36]RBL236_01942_Acyltransferase_family_protein	RbromiiL2-36
Org4_Gene1861	accessory	108	[RbromiiL2-36]RBL236_01943_CDP-Glycerol:Poly(glycerophosphate)_glycerophosphotransferase	RbromiiL2-36
Org4_Gene1863	accessory	3156	[RbromiiL2-36]RBL236_01945_putative_acetyltransferase	RbromiiL2-36
Org4_Gene1864	accessory	125	[RbromiiL2-36]RBL236_01946_Elongation_factor_G	RbromiiL2-36
Org4_Gene1865	accessory	977	[RbromiiL2-36]RBL236_01947_LL-diaminopimelate_aminotransferase	RbromiiL2-36
Org4_Gene1866	accessory	4006	[RbromiiL2-36]RBL236_01948_hypothetical_protein	RbromiiL2-36
Org4_Gene1867	accessory	2931	[RbromiiL2-36]RBL236_01949_hypothetical_protein	RbromiiL2-36
Org4_Gene1868	accessory	2861	[RbromiiL2-36]RBL236_01950_hypothetical_protein	RbromiiL2-36
Org4_Gene1869	accessory	3092	[RbromiiL2-36]RBL236_01951_hypothetical_protein	RbromiiL2-36
Org4_Gene187	accessory	1570	[RbromiiL2-36]RBL236_00194_hypothetical_protein	RbromiiL2-36
Org4_Gene1870	accessory	2767	[RbromiiL2-36]RBL236_01952_hypothetical_protein	RbromiiL2-36
Org4_Gene1871	accessory	3419	[RbromiiL2-36]RBL236_01953_Peptide_deformylase_1	RbromiiL2-36
Org4_Gene1872	accessory	2311	[RbromiiL2-36]RBL236_01954_hypothetical_protein	RbromiiL2-36
Org4_Gene1873	accessory	3131	[RbromiiL2-36]RBL236_01955_RNA_polymerase_sigma_factor_SigM	RbromiiL2-36
Org4_Gene1874	accessory	4263	[RbromiiL2-36]RBL236_01956_hypothetical_protein	RbromiiL2-36
Org4_Gene1875	accessory	3263	[RbromiiL2-36]RBL236_01957_HTH-type_transcriptional_regulator_AdhR	RbromiiL2-36
Org4_Gene1876	accessory	1905	[RbromiiL2-36]RBL236_01958_Patatin-like_phospholipase	RbromiiL2-36
Org4_Gene1877	accessory	2662	[RbromiiL2-36]RBL236_01959_mycofactocin_system_transcriptional_regulator	RbromiiL2-36
Org4_Gene1878	accessory	2363	[RbromiiL2-36]RBL236_01960_Cd1	RbromiiL2-36
Org4_Gene188	accessory	172	[RbromiiL2-36]RBL236_00195_ski2-like_helicase	RbromiiL2-36
Org4_Gene1881	accessory	213	[RbromiiL2-36]RBL236_01963_3-oxoacyl-[acyl-carrier-protein]_synthase_2	RbromiiL2-36
Org4_Gene1883	accessory	2422	[RbromiiL2-36]RBL236_01965_Bifunctional_protein_aas	RbromiiL2-36
Org4_Gene1884	accessory	1299	[RbromiiL2-36]RBL236_01966_Tryptophan--tRNA_ligase_2	RbromiiL2-36
Org4_Gene1885	accessory	1381	[RbromiiL2-36]RBL236_01967_Penicillin_V_acylase	RbromiiL2-36
Org4_Gene1886	accessory	840	[RbromiiL2-36]RBL236_01968_sensory_histidine_kinase_DcuS	RbromiiL2-36
Org4_Gene1887	accessory	2284	[RbromiiL2-36]RBL236_01969_Transcriptional_regulatory_protein_YpdB	RbromiiL2-36
Org4_Gene1888	accessory	272	[RbromiiL2-36]RBL236_01970_Metallo-beta-lactamase_superfamily_protein	RbromiiL2-36
Org4_Gene1889	accessory	2887	[RbromiiL2-36]RBL236_01971_hypothetical_protein	RbromiiL2-36
Org4_Gene189	accessory	3647	[RbromiiL2-36]RBL236_00196_hypothetical_protein	RbromiiL2-36
Org4_Gene1894	accessory	1493	[RbromiiL2-36]RBL236_01978_V-type_sodium_pump_subunit_C	RbromiiL2-36
Org4_Gene1895	accessory	2760	[RbromiiL2-36]RBL236_01979_V-type_ATP_synthase_subunit_E	RbromiiL2-36
Org4_Gene1897	accessory	287	[RbromiiL2-36]RBL236_01981_V-type_ATP_synthase_subunit_I	RbromiiL2-36
Org4_Gene1898	accessory	3801	[RbromiiL2-36]RBL236_01982_V-type_ATP_synthase_subunit_H	RbromiiL2-36
Org4_Gene1899	accessory	1429	[RbromiiL2-36]RBL236_01983_mobile_mystery_protein_B	RbromiiL2-36
Org4_Gene190	accessory	4426	[RbromiiL2-36]RBL236_00197_hypothetical_protein	RbromiiL2-36
Org4_Gene1901	accessory	32	[RbromiiL2-36]RBL236_01985_Swarming_motility_protein_SwrC	RbromiiL2-36
Org4_Gene1902	accessory	2637	[RbromiiL2-36]RBL236_01986_DNA-binding_transcriptional_repressor_AcrR	RbromiiL2-36
Org4_Gene1903	accessory	3183	[RbromiiL2-36]RBL236_01987_Low_molecular_weight_protein-tyrosine-phosphatase_YfkJ	RbromiiL2-36
Org4_Gene1904	accessory	4432	[RbromiiL2-36]RBL236_01988_hypothetical_protein	RbromiiL2-36
Org4_Gene1905	accessory	3000	[RbromiiL2-36]RBL236_01989_hypothetical_protein	RbromiiL2-36
Org4_Gene1907	accessory	2604	[RbromiiL2-36]RBL236_01991_putative_sugar_transferase_EpsL	RbromiiL2-36
Org4_Gene1909	accessory	1241	[RbromiiL2-36]RBL236_01993_N,N'-diacetylbacillosaminyl-diphospho-undecaprenol_alpha-1,3-N-acetylgalactosaminyltransferase	RbromiiL2-36
Org4_Gene191	accessory	3848	[RbromiiL2-36]RBL236_00198_hypothetical_protein	RbromiiL2-36
Org4_Gene1910	accessory	1248	[RbromiiL2-36]RBL236_01994_Putative_glycosyltransferase_EpsF	RbromiiL2-36
Org4_Gene1911	accessory	1645	[RbromiiL2-36]RBL236_01995_Hyaluronan_synthase	RbromiiL2-36

Org4_Gene1912	accessory	754	[RbromiiL2-36]RBL236_01996_putative_ATPase	RbromiiL2-36
Org4_Gene1913	accessory	2377	[RbromiiL2-36]RBL236_01997_hypothetical_protein	RbromiiL2-36
Org4_Gene1914	accessory	3656	[RbromiiL2-36]RBL236_01998_hypothetical_protein	RbromiiL2-36
Org4_Gene1915	accessory	3212	[RbromiiL2-36]RBL236_01999_hypothetical_protein	RbromiiL2-36
Org4_Gene1916	accessory	2409	[RbromiiL2-36]RBL236_02000_hypothetical_protein	RbromiiL2-36
Org4_Gene1917	accessory	1954	[RbromiiL2-36]RBL236_02001_ABC-2_family_transporter_protein	RbromiiL2-36
Org4_Gene1918	accessory	2381	[RbromiiL2-36]RBL236_02002_hypothetical_protein	RbromiiL2-36
Org4_Gene1919	accessory	1919	[RbromiiL2-36]RBL236_02003_ABC-2_family_transporter_protein	RbromiiL2-36
Org4_Gene192	accessory	2434	[RbromiiL2-36]RBL236_00199_hypothetical_protein	RbromiiL2-36
Org4_Gene1920	accessory	2523	[RbromiiL2-36]RBL236_02004_Fluoroquinolones_export_ATP-binding_proteinc/MT2762	RbromiiL2-36
Org4_Gene1921	accessory	2776	[RbromiiL2-36]RBL236_02005_hypothetical_protein	RbromiiL2-36
Org4_Gene1922	accessory	4342	[RbromiiL2-36]RBL236_02006_hypothetical_protein	RbromiiL2-36
Org4_Gene1925	accessory	2585	[RbromiiL2-36]RBL236_02009_phosphoglycolate_phosphatase	RbromiiL2-36
Org4_Gene1926	accessory	2829	[RbromiiL2-36]RBL236_02010_HAD_hydrolase,_family_IB	RbromiiL2-36
Org4_Gene1927	accessory	1817	[RbromiiL2-36]RBL236_02011_hypothetical_protein	RbromiiL2-36
Org4_Gene1928	accessory	2650	[RbromiiL2-36]RBL236_02012_Chaperone_protein_DnaJ	RbromiiL2-36
Org4_Gene1929	accessory	2699	[RbromiiL2-36]RBL236_02013_hypothetical_protein	RbromiiL2-36
Org4_Gene193	accessory	3782	[RbromiiL2-36]RBL236_00200_hypothetical_protein	RbromiiL2-36
Org4_Gene1930	accessory	138	[RbromiiL2-36]RBL236_02014_invasion_associated_secreted_endopeptidase	RbromiiL2-36
Org4_Gene1932	accessory	2477	[RbromiiL2-36]RBL236_02016_stage_II_sporulation_protein_M	RbromiiL2-36
Org4_Gene1933	accessory	1547	[RbromiiL2-36]RBL236_02017_putative_integral_membrane_protein	RbromiiL2-36
Org4_Gene1934	accessory	2541	[RbromiiL2-36]RBL236_02018_hypothetical_protein	RbromiiL2-36
Org4_Gene1935	accessory	2915	[RbromiiL2-36]RBL236_02019_hypothetical_protein	RbromiiL2-36
Org4_Gene1936	accessory	1790	[RbromiiL2-36]RBL236_02020_Radical_SAM_superfamily_protein	RbromiiL2-36
Org4_Gene1939	accessory	1930	[RbromiiL2-36]RBL236_02023_hypothetical_protein	RbromiiL2-36
Org4_Gene194	accessory	78	[RbromiiL2-36]RBL236_00201_hypothetical_protein	RbromiiL2-36
Org4_Gene1946	accessory	3972	[RbromiiL2-36]RBL236_02030_pro-sigmaK_processing_inhibitor_BofA	RbromiiL2-36
Org4_Gene1947	accessory	2708	[RbromiiL2-36]RBL236_02031_hypothetical_protein	RbromiiL2-36
Org4_Gene1948	accessory	3306	[RbromiiL2-36]RBL236_02032_HTH-type_transcriptional_regulator_IscR	RbromiiL2-36
Org4_Gene1949	accessory	2167	[RbromiiL2-36]RBL236_02033_putative_ABC_transporter_ATP-binding_protein	RbromiiL2-36
Org4_Gene195	accessory	379	[RbromiiL2-36]RBL236_00202_hypothetical_protein	RbromiiL2-36
Org4_Gene1950	accessory	682	[RbromiiL2-36]RBL236_02034_FeS_cluster_assembly_protein_SufB	RbromiiL2-36
Org4_Gene1951	accessory	1257	[RbromiiL2-36]RBL236_02035_FeS_cluster_assembly_protein_SufD	RbromiiL2-36
Org4_Gene1952	accessory	989	[RbromiiL2-36]RBL236_02036_putative_cysteine_desulfurase	RbromiiL2-36
Org4_Gene1953	accessory	3356	[RbromiiL2-36]RBL236_02037_NifU-like_protein	RbromiiL2-36
Org4_Gene1954	accessory	2961	[RbromiiL2-36]RBL236_02038_hypothetical_protein	RbromiiL2-36
Org4_Gene1955	accessory	3168	[RbromiiL2-36]RBL236_02039_hypothetical_protein	RbromiiL2-36
Org4_Gene1956	accessory	2042	[RbromiiL2-36]RBL236_02040_CAAX_prenyl_protease-related_protein	RbromiiL2-36
Org4_Gene1957	accessory	2978	[RbromiiL2-36]RBL236_02041_hypothetical_protein	RbromiiL2-36
Org4_Gene1958	accessory	2504	[RbromiiL2-36]RBL236_02042_hypothetical_protein	RbromiiL2-36
Org4_Gene1959	accessory	3537	[RbromiiL2-36]RBL236_02043_hypothetical_protein	RbromiiL2-36
Org4_Gene1960	accessory	3625	[RbromiiL2-36]RBL236_02044_transcriptional_regulator,_Acidobacterial,_PadR-family	RbromiiL2-36
Org4_Gene1961	accessory	1508	[RbromiiL2-36]RBL236_02045_Cytochrome_c_biogenesis_protein_CycY	RbromiiL2-36
Org4_Gene1962	accessory	1457	[RbromiiL2-36]RBL236_02046_Putative_electron_transport_protein_YccM	RbromiiL2-36
Org4_Gene1963	accessory	2442	[RbromiiL2-36]RBL236_02047_Transcriptional_regulatory_protein_QseB	RbromiiL2-36
Org4_Gene1964	accessory	1157	[RbromiiL2-36]RBL236_02048_Signal_transduction_histidine-protein_kinase_ArlS	RbromiiL2-36
Org4_Gene1965	accessory	2680	[RbromiiL2-36]RBL236_02049_DNA-binding_transcriptional_repressor_AcrR	RbromiiL2-36
Org4_Gene1966	accessory	293	[RbromiiL2-36]RBL236_02050_Collagen_triple_helix_repeat_(20_copies)	RbromiiL2-36
Org4_Gene1969	accessory	285	[RbromiiL2-36]RBL236_02053_hypothetical_protein	RbromiiL2-36
Org4_Gene197	accessory	4303	[RbromiiL2-36]RBL236_00204_hypothetical_protein	RbromiiL2-36
Org4_Gene1975	accessory	740	[RbromiiL2-36]RBL236_02059_Staphylococcal_virulence_regulator_protein_A	RbromiiL2-36
Org4_Gene1976	accessory	2552	[RbromiiL2-36]RBL236_02060_Phosphorylated_carbohydrates_phosphatase	RbromiiL2-36
Org4_Gene1977	accessory	1870	[RbromiiL2-36]RBL236_02061_putative_membrane_protein	RbromiiL2-36
Org4_Gene1978	accessory	370	[RbromiiL2-36]RBL236_02062_hypothetical_protein	RbromiiL2-36
Org4_Gene1979	accessory	623	[RbromiiL2-36]RBL236_02063_Multidrug_export_protein_MepA	RbromiiL2-36
Org4_Gene198	accessory	1553	[RbromiiL2-36]RBL236_00205_auxin_efflux_carrier	RbromiiL2-36
Org4_Gene1980	accessory	1024	[RbromiiL2-36]RBL236_02064_putative_integral_membrane_protein	RbromiiL2-36
Org4_Gene1983	accessory	855	[RbromiiL2-36]RBL236_02067_hypothetical_protein	RbromiiL2-36
Org4_Gene1985	accessory	2765	[RbromiiL2-36]RBL236_02069_hypothetical_protein	RbromiiL2-36
Org4_Gene1987	accessory	113	[RbromiiL2-36]RBL236_02071_copper_exporting_ATPase	RbromiiL2-36
Org4_Gene199	accessory	2909	[RbromiiL2-36]RBL236_00206_malonic_semialdehyde_reductase	RbromiiL2-36
Org4_Gene1991	accessory	897	[RbromiiL2-36]RBL236_02076_corrinoid_ABC_transporter_substrate-binding_protein	RbromiiL2-36
Org4_Gene1995	accessory	1310	[RbromiiL2-36]RBL236_02080_hypothetical_protein	RbromiiL2-36
Org4_Gene1996	accessory	2344	[RbromiiL2-36]RBL236_02081_transcriptional_regulator_FixK	RbromiiL2-36
Org4_Gene1998	accessory	1545	[RbromiiL2-36]RBL236_02083_L-lactate_dehydrogenase_P	RbromiiL2-36
Org4_Gene20	accessory	211	[RbromiiL2-36]RBL236_00020_Dockerin_type_I_repeat	RbromiiL2-36
Org4_Gene200	accessory	2985	[RbromiiL2-36]RBL236_00207_FMN_reductase_[NAD(P)H]	RbromiiL2-36
Org4_Gene2001	accessory	651	[RbromiiL2-36]RBL236_02086_Trk_system_potassium_uptake_protein_TrkA	RbromiiL2-36
Org4_Gene2003	accessory	1233	[RbromiiL2-36]RBL236_02088_stage_II_sporulation_protein_P	RbromiiL2-36
Org4_Gene2005	accessory	462	[RbromiiL2-36]RBL236_02090_Papain_family_cysteine_protease	RbromiiL2-36
Org4_Gene2007	accessory	468	[RbromiiL2-36]RBL236_02092_Alpha-amylase_precursor	RbromiiL2-36
Org4_Gene2009	accessory	3195	[RbromiiL2-36]RBL236_02094_Acetyltransferase_(GNAT)_family_protein	RbromiiL2-36
Org4_Gene201	accessory	1711	[RbromiiL2-36]RBL236_00208_Cys_regulon_transcriptional_activator	RbromiiL2-36
Org4_Gene2010	accessory	3619	[RbromiiL2-36]RBL236_02095_hypothetical_protein	RbromiiL2-36
Org4_Gene2011	accessory	4222	[RbromiiL2-36]RBL236_02096_Ferredoxin-thioredoxin_reductase,_catalytic_subunit	RbromiiL2-36
Org4_Gene2012	accessory	3803	[RbromiiL2-36]RBL236_02097_Thioredoxin-M	RbromiiL2-36
Org4_Gene2013	accessory	3187	[RbromiiL2-36]RBL236_02098_Terminase_small_subunit	RbromiiL2-36
Org4_Gene2015	accessory	1126	[RbromiiL2-36]RBL236_02100_phage_portal_protein,_HK97_family	RbromiiL2-36
Org4_Gene2016	accessory	1431	[RbromiiL2-36]RBL236_02101_hypothetical_protein	RbromiiL2-36
Org4_Gene2017	accessory	3684	[RbromiiL2-36]RBL236_02102_hypothetical_protein	RbromiiL2-36
Org4_Gene2019	accessory	3620	[RbromiiL2-36]RBL236_02104_hypothetical_protein	RbromiiL2-36
Org4_Gene202	accessory	1207	[RbromiiL2-36]RBL236_00209_5-methyltetrahydropteroyltriglutamate--homocysteine_methyltransferase	RbromiiL2-36
Org4_Gene2020	accessory	3603	[RbromiiL2-36]RBL236_02105_hypothetical_protein	RbromiiL2-36
Org4_Gene2021	accessory	3144	[RbromiiL2-36]RBL236_02106_hypothetical_protein	RbromiiL2-36
Org4_Gene2022	accessory	3579	[RbromiiL2-36]RBL236_02107_hypothetical_protein	RbromiiL2-36
Org4_Gene2023	accessory	4389	[RbromiiL2-36]RBL236_02108_hypothetical_protein	RbromiiL2-36
Org4_Gene2024	accessory	3009	[RbromiiL2-36]RBL236_02109_Mu-like_prophage_DNA_circulation_protein	RbromiiL2-36
Org4_Gene2025	accessory	1534	[RbromiiL2-36]RBL236_02110_Mu-like_prophage_tail_protein_gpP	RbromiiL2-36
Org4_Gene2026	accessory	3726	[RbromiiL2-36]RBL236_02111_Mu-like_prophage_protein_gp45	RbromiiL2-36
Org4_Gene2027	accessory	3727	[RbromiiL2-36]RBL236_02112_hypothetical_protein	RbromiiL2-36
Org4_Gene2028	accessory	1302	[RbromiiL2-36]RBL236_02113_baseplate_assembly_protein	RbromiiL2-36
Org4_Gene2029	accessory	3005	[RbromiiL2-36]RBL236_02114_hypothetical_protein	RbromiiL2-36
Org4_Gene203	accessory	1598	[RbromiiL2-36]RBL236_00210_ABC-2_family_transporter_protein	RbromiiL2-36
Org4_Gene2030	accessory	2786	[RbromiiL2-36]RBL236_02115_hypothetical_protein	RbromiiL2-36
Org4_Gene2031	accessory	2400	[RbromiiL2-36]RBL236_02116_putative_ABC_transporter_ATP-binding_protein/MT1014	RbromiiL2-36
Org4_Gene2032	accessory	1151	[RbromiiL2-36]RBL236_02117_FtsX-like_permease_family_protein	RbromiiL2-36
Org4_Gene2033	accessory	635	[RbromiiL2-36]RBL236_02118_putative_ATPase_(AAA+_superfamily)	RbromiiL2-36
Org4_Gene2037	accessory	889	[RbromiiL2-36]RBL236_02122_hypothetical_protein	RbromiiL2-36
Org4_Gene2040	accessory	1035	[RbromiiL2-36]RBL236_02125_hypothetical_protein	RbromiiL2-36
Org4_Gene2041	accessory	1752	[RbromiiL2-36]RBL236_02126_hypothetical_protein	RbromiiL2-36
Org4_Gene2044	accessory	1584	[RbromiiL2-36]RBL236_02129_auxin_efflux_carrier	RbromiiL2-36
Org4_Gene2045	accessory	3522	[RbromiiL2-36]RBL236_02130_hypothetical_protein	RbromiiL2-36
Org4_Gene2048	accessory	1169	[RbromiiL2-36]RBL236_02133_hypothetical_protein	RbromiiL2-36
Org4_Gene2049	accessory	2061	[RbromiiL2-36]RBL236_02134_hypothetical_protein	RbromiiL2-36
Org4_Gene205	accessory	3275	[RbromiiL2-36]RBL236_00212_hypothetical_protein	RbromiiL2-36
Org4_Gene2052	accessory	500	[RbromiiL2-36]RBL236_02137_Germination-specific_amidase	RbromiiL2-36
Org4_Gene2053	accessory	478	[RbromiiL2-36]RBL236_02138_Phosphoserine_phosphatase_RsbU	RbromiiL2-36
Org4_Gene2055	accessory	3227	[RbromiiL2-36]RBL236_02140_hypothetical_protein	RbromiiL2-36
Org4_Gene2058	accessory	4058	[RbromiiL2-36]RBL236_02143_HTH-type_transcriptional_regulator_ImmR	RbromiiL2-36
Org4_Gene206	accessory	3302	[RbromiiL2-36]RBL236_00213_Transcriptional_regulatory_protein_YpdB	RbromiiL2-36
Org4_Gene2060	accessory	184	[RbromiiL2-36]RBL236_02145_Stage_II_sporulation_protein_E	RbromiiL2-36
Org4_Gene2061	accessory	3748	[RbromiiL2-36]RBL236_02146_hypothetical_protein	RbromiiL2-36
Org4_Gene2062	accessory	1573	[RbromiiL2-36]RBL236_02147_hypothetical_protein	RbromiiL2-36
Org4_Gene2063	accessory	872	[RbromiiL2-36]RBL236_02149_hypothetical_protein	RbromiiL2-36
Org4_Gene2064	accessory	3201	[RbromiiL2-36]RBL236_02150_PPOX_class_probable_F420-dependent_enzyme	RbromiiL2-36
Org4_Gene2065	accessory	1471	[RbromiiL2-36]RBL236_02151_L-asparaginase_1	RbromiiL2-36
Org4_Gene2066	accessory	1942	[RbromiiL2-36]RBL236_02152_hypothetical_protein	RbromiiL2-36
Org4_Gene2067	accessory	3398	[RbromiiL2-36]RBL236_02153_hypothetical_protein	RbromiiL2-36
Org4_Gene2068	accessory	856	[RbromiiL2-36]RBL236_02154_Inner_membrane_protein_YbaL	RbromiiL2-36
Org4_Gene2069	accessory	2780	[RbromiiL2-36]RBL236_02155_putative_dihydroxyacetone_kinase_regulator	RbromiiL2-36
Org4_Gene207	accessory	4304	[RbromiiL2-36]RBL236_00214_hypothetical_protein	RbromiiL2-36
Org4_Gene2073	accessory	2548	[RbromiiL2-36]RBL236_02159_hypothetical_protein	RbromiiL2-36

Org4_Gene2077	accessory	2647	[RbromiiL2-36]RBL236_02163_Dephospho-CoA_kinase	RbromiiL2-36
Org4_Gene2078	accessory	2624	[RbromiiL2-36]RBL236_02164_Soluble_lytic_murein_transglycosylase_precursor	RbromiiL2-36
Org4_Gene208	accessory	226	[RbromiiL2-36]RBL236_00215_Protein-glutamine_gamma-glutamyltransferase	RbromiiL2-36
Org4_Gene2080	accessory	3584	[RbromiiL2-36]RBL236_02168_four_helix_bundle_protein	RbromiiL2-36
Org4_Gene2081	accessory	968	[RbromiiL2-36]RBL236_02169_CHAP_domain_protein	RbromiiL2-36
Org4_Gene2082	accessory	841	[RbromiiL2-36]RBL236_02170_hypothetical_protein	RbromiiL2-36
Org4_Gene2084	accessory	2250	[RbromiiL2-36]RBL236_02172_Transcriptional_regulatory_protein_YpdB	RbromiiL2-36
Org4_Gene2085	accessory	3047	[RbromiiL2-36]RBL236_02173_hypothetical_protein	RbromiiL2-36
Org4_Gene2087	accessory	2306	[RbromiiL2-36]RBL236_02175_phosphoglycolate_phosphatase	RbromiiL2-36
Org4_Gene2089	accessory	1412	[RbromiiL2-36]RBL236_02177_hypothetical_protein	RbromiiL2-36
Org4_Gene209	accessory	1511	[RbromiiL2-36]RBL236_00216_hypothetical_protein	RbromiiL2-36
Org4_Gene2090	accessory	2144	[RbromiiL2-36]RBL236_02178_hypothetical_protein	RbromiiL2-36
Org4_Gene2091	accessory	3094	[RbromiiL2-36]RBL236_02179_hypothetical_protein	RbromiiL2-36
Org4_Gene2093	accessory	3961	[RbromiiL2-36]RBL236_02181_hypothetical_protein	RbromiiL2-36
Org4_Gene2094	accessory	1502	[RbromiiL2-36]RBL236_02182_Bifunctional_protein_BirA	RbromiiL2-36
Org4_Gene2095	accessory	2630	[RbromiiL2-36]RBL236_02183_putative_membrane_protein	RbromiiL2-36
Org4_Gene2099	accessory	4416	[RbromiiL2-36]RBL236_02187_hypothetical_protein	RbromiiL2-36
Org4_Gene210	accessory	1616	[RbromiiL2-36]RBL236_00217_regulatory_ATPase_RavA	RbromiiL2-36
Org4_Gene2102	accessory	2489	[RbromiiL2-36]RBL236_02190_5'-nucleotidase	RbromiiL2-36
Org4_Gene2103	accessory	3180	[RbromiiL2-36]RBL236_02191_hypothetical_protein	RbromiiL2-36
Org4_Gene2104	accessory	2298	[RbromiiL2-36]RBL236_02192_Ribosomal_small_subunit_pseudouridine_synthase_A	RbromiiL2-36
Org4_Gene2108	accessory	1709	[RbromiiL2-36]RBL236_02196_Cell_division_protein_FtsX	RbromiiL2-36
Org4_Gene2109	accessory	825	[RbromiiL2-36]RBL236_02197_Septal_ring_factor	RbromiiL2-36
Org4_Gene2110	accessory	1945	[RbromiiL2-36]RBL236_02198_hypothetical_protein	RbromiiL2-36
Org4_Gene2113	accessory	777	[RbromiiL2-36]RBL236_02201_Multidrug_export_protein_MepA	RbromiiL2-36
Org4_Gene2114	accessory	2466	[RbromiiL2-36]RBL236_02202_Heme_response_regulator_HssR	RbromiiL2-36
Org4_Gene2115	accessory	1336	[RbromiiL2-36]RBL236_02203_Signal_transduction_histidine-protein_kinase_BaeS	RbromiiL2-36
Org4_Gene2116	accessory	1245	[RbromiiL2-36]RBL236_02204_putative_membrane_protein	RbromiiL2-36
Org4_Gene2117	accessory	3624	[RbromiiL2-36]RBL236_02205_hypothetical_protein	RbromiiL2-36
Org4_Gene212	accessory	1120	[RbromiiL2-36]RBL236_00223_hypothetical_protein	RbromiiL2-36
Org4_Gene2121	accessory	3106	[RbromiiL2-36]RBL236_02209_deoxycytidylate_deaminase	RbromiiL2-36
Org4_Gene2122	accessory	2600	[RbromiiL2-36]RBL236_02210_4'-phosphopantetheinyl_transferase_sfp	RbromiiL2-36
Org4_Gene2123	accessory	2828	[RbromiiL2-36]RBL236_02211_putative_dihydroxyacetone_kinase_regulator	RbromiiL2-36
Org4_Gene2125	accessory	151	[RbromiiL2-36]RBL236_02213_X-X-X-Leu-X-X-Gly_heptad_repeats	RbromiiL2-36
Org4_Gene2126	accessory	4229	[RbromiiL2-36]RBL236_02214_hypothetical_protein	RbromiiL2-36
Org4_Gene213	accessory	2852	[RbromiiL2-36]RBL236_00224_hypothetical_protein	RbromiiL2-36
Org4_Gene214	accessory	1143	[RbromiiL2-36]RBL236_00225_Resuscitation-promoting_factor_Rpf2_precursor	RbromiiL2-36
Org4_Gene215	accessory	1893	[RbromiiL2-36]RBL236_00226_DNA-damage-inducible_protein_D	RbromiiL2-36
Org4_Gene216	accessory	1999	[RbromiiL2-36]RBL236_00227_Arabinose_operon_regulatory_protein	RbromiiL2-36
Org4_Gene217	accessory	1921	[RbromiiL2-36]RBL236_00228_Melibiose_operon_regulatory_protein	RbromiiL2-36
Org4_Gene218	accessory	1873	[RbromiiL2-36]RBL236_00229_Carboxylesterase_NlhH	RbromiiL2-36
Org4_Gene219	accessory	1607	[RbromiiL2-36]RBL236_00230_Bacterial_Ig-like_domain_(group_2)	RbromiiL2-36
Org4_Gene220	accessory	2531	[RbromiiL2-36]RBL236_00231_cAMP_regulatory_protein	RbromiiL2-36
Org4_Gene223	accessory	64	[RbromiiL2-36]RBL236_00234_Exoglucanase_B_precursor	RbromiiL2-36
Org4_Gene231	accessory	4118	[RbromiiL2-36]RBL236_00242_Helix-turn-helix_domain_protein	RbromiiL2-36
Org4_Gene232	accessory	1524	[RbromiiL2-36]RBL236_00243_hypothetical_protein	RbromiiL2-36
Org4_Gene234	accessory	2193	[RbromiiL2-36]RBL236_00245_Sensory_transduction_protein_LytR	RbromiiL2-36
Org4_Gene235	accessory	548	[RbromiiL2-36]RBL236_00246_hypothetical_protein	RbromiiL2-36
Org4_Gene236	accessory	497	[RbromiiL2-36]RBL236_00247_gliding-associated_putative_ABC_transporter_substrate-binding_component_GldG	RbromiiL2-36
Org4_Gene240	accessory	1656	[RbromiiL2-36]RBL236_00251_hypothetical_protein	RbromiiL2-36
Org4_Gene241	accessory	106	[RbromiiL2-36]RBL236_00252_putative_membrane_protein	RbromiiL2-36
Org4_Gene243	accessory	3213	[RbromiiL2-36]RBL236_00254_GtrA-like_protein	RbromiiL2-36
Org4_Gene246	accessory	3407	[RbromiiL2-36]RBL236_00257_hypothetical_protein	RbromiiL2-36
Org4_Gene249	accessory	1987	[RbromiiL2-36]RBL236_00263_hypothetical_protein	RbromiiL2-36
Org4_Gene250	accessory	3466	[RbromiiL2-36]RBL236_00264_hypothetical_protein	RbromiiL2-36
Org4_Gene251	accessory	3623	[RbromiiL2-36]RBL236_00265_hypothetical_protein	RbromiiL2-36
Org4_Gene256	accessory	2320	[RbromiiL2-36]RBL236_00270_Ribosomal_RNA_small_subunit_methyltransferase_G	RbromiiL2-36
Org4_Gene260	accessory	1410	[RbromiiL2-36]RBL236_00274_Oxa1Ec	RbromiiL2-36
Org4_Gene269	accessory	2258	[RbromiiL2-36]RBL236_00283_cxxc_20_cxxc_protein	RbromiiL2-36
Org4_Gene271	accessory	3328	[RbromiiL2-36]RBL236_00285_acyl-CoA_thioesterase_YbgC	RbromiiL2-36
Org4_Gene273	accessory	2176	[RbromiiL2-36]RBL236_00287_hypothetical_protein	RbromiiL2-36
Org4_Gene274	accessory	327	[RbromiiL2-36]RBL236_00288_Alpha-amylase/pullulanase	RbromiiL2-36
Org4_Gene275	accessory	2560	[RbromiiL2-36]RBL236_00289_cell_division_protein_ZapA	RbromiiL2-36
Org4_Gene276	accessory	262	[RbromiiL2-36]RBL236_00290_putative_protease_YhbU_precursor	RbromiiL2-36
Org4_Gene279	accessory	1973	[RbromiiL2-36]RBL236_00293_Rod_shape-determining_protein_MreC	RbromiiL2-36
Org4_Gene280	accessory	3046	[RbromiiL2-36]RBL236_00294_rod_shape-determining_protein_MreD	RbromiiL2-36
Org4_Gene281	accessory	276	[RbromiiL2-36]RBL236_00295_Penicillin-binding_protein_2B	RbromiiL2-36
Org4_Gene286	accessory	2096	[RbromiiL2-36]RBL236_00300_hypothetical_protein	RbromiiL2-36
Org4_Gene288	accessory	1188	[RbromiiL2-36]RBL236_00302_hypothetical_protein	RbromiiL2-36
Org4_Gene289	accessory	1977	[RbromiiL2-36]RBL236_00303_hypothetical_protein	RbromiiL2-36
Org4_Gene290	accessory	1405	[RbromiiL2-36]RBL236_00304_Biotin_synthase	RbromiiL2-36
Org4_Gene292	accessory	1066	[RbromiiL2-36]RBL236_00306_tRNA_modification_GTPase_MnmE	RbromiiL2-36
Org4_Gene293	accessory	476	[RbromiiL2-36]RBL236_00307_Acetyl-coenzyme_A_synthetase	RbromiiL2-36
Org4_Gene296	accessory	620	[RbromiiL2-36]RBL236_00311_Anthranilate_synthase_component_1	RbromiiL2-36
Org4_Gene297	accessory	2800	[RbromiiL2-36]RBL236_00312_Anthranilate_synthase_component_II	RbromiiL2-36
Org4_Gene298	accessory	1401	[RbromiiL2-36]RBL236_00313_Anthranilate_phosphoribosyltransferase	RbromiiL2-36
Org4_Gene299	accessory	2046	[RbromiiL2-36]RBL236_00314_Indole-3-glycerol_phosphate_synthase	RbromiiL2-36
Org4_Gene305	accessory	3792	[RbromiiL2-36]RBL236_00320_hypothetical_protein	RbromiiL2-36
Org4_Gene306	accessory	1348	[RbromiiL2-36]RBL236_00321_Flagellar_biosynthetic_protein_FliU	RbromiiL2-36
Org4_Gene311	accessory	3909	[RbromiiL2-36]RBL236_00326_hypothetical_protein	RbromiiL2-36
Org4_Gene312	accessory	2706	[RbromiiL2-36]RBL236_00327_KilA-N_domain_protein	RbromiiL2-36
Org4_Gene313	accessory	3998	[RbromiiL2-36]RBL236_00328_hypothetical_protein	RbromiiL2-36
Org4_Gene314	accessory	3442	[RbromiiL2-36]RBL236_00329_Putative_8-oxo-dGTP_diphosphatase_YtkD	RbromiiL2-36
Org4_Gene316	accessory	2668	[RbromiiL2-36]RBL236_00331_Adenosine_monophosphate-protein_transferase_NmFic	RbromiiL2-36
Org4_Gene317	accessory	3920	[RbromiiL2-36]RBL236_00332_hypothetical_protein	RbromiiL2-36
Org4_Gene318	accessory	1368	[RbromiiL2-36]RBL236_00333_Heat-inducible_transcription_repressor_HrcA	RbromiiL2-36
Org4_Gene319	accessory	2855	[RbromiiL2-36]RBL236_00334_HSP-70_cofactor	RbromiiL2-36
Org4_Gene32	accessory	3318	[RbromiiL2-36]RBL236_00032_Acetyltransferase_YpeA	RbromiiL2-36
Org4_Gene323	accessory	1225	[RbromiiL2-36]RBL236_00338_hypothetical_protein	RbromiiL2-36
Org4_Gene325	accessory	1937	[RbromiiL2-36]RBL236_00340_hypothetical_protein	RbromiiL2-36
Org4_Gene326	accessory	2746	[RbromiiL2-36]RBL236_00341_hypothetical_protein	RbromiiL2-36
Org4_Gene327	accessory	3416	[RbromiiL2-36]RBL236_00342_transcriptional_repressor_DicA	RbromiiL2-36
Org4_Gene328	accessory	3929	[RbromiiL2-36]RBL236_00343_Lipopolysaccharide_export_system_ATP-binding_protein_LptB	RbromiiL2-36
Org4_Gene329	accessory	2146	[RbromiiL2-36]RBL236_00344_putative_membrane_protein	RbromiiL2-36
Org4_Gene33	accessory	3313	[RbromiiL2-36]RBL236_00033_Low_molecular_weight_protein-tyrosine-phosphatase_YwlE	RbromiiL2-36
Org4_Gene330	accessory	2358	[RbromiiL2-36]RBL236_00345_putative_membrane_protein	RbromiiL2-36
Org4_Gene339	accessory	3576	[RbromiiL2-36]RBL236_00354_putative_protein_encoded_in_hypervariable_junctions_of_pilus_gene_clusters	RbromiiL2-36
Org4_Gene341	accessory	30	[RbromiiL2-36]RBL236_00356_Alpha-amylase_precursor	RbromiiL2-36
Org4_Gene342	accessory	991	[RbromiiL2-36]RBL236_00357_Signal-transduction_histidine_kinase_senX3	RbromiiL2-36
Org4_Gene343	accessory	2549	[RbromiiL2-36]RBL236_00358_Phosphate_regulon_transcriptional_regulatory_protein_PhoB	RbromiiL2-36
Org4_Gene344	accessory	116	[RbromiiL2-36]RBL236_00359_ABC_transporter_permease_YtrF_precursor	RbromiiL2-36
Org4_Gene345	accessory	2465	[RbromiiL2-36]RBL236_00360_putative_ABC_transporter_ATP-binding_protein/MT1014	RbromiiL2-36
Org4_Gene346	accessory	1648	[RbromiiL2-36]RBL236_00361_tRNA_pseudouridine_synthase_B	RbromiiL2-36
Org4_Gene347	accessory	1575	[RbromiiL2-36]RBL236_00362_Bifunctional_oligoribonuclease_and_PAP_phosphatase_NrnA	RbromiiL2-36
Org4_Gene350	accessory	3711	[RbromiiL2-36]RBL236_00365_putative_ribosomal_protein_YlxQ	RbromiiL2-36
Org4_Gene351	accessory	3805	[RbromiiL2-36]RBL236_00366_hypothetical_protein	RbromiiL2-36
Org4_Gene354	accessory	4240	[RbromiiL2-36]RBL236_00370_hypothetical_protein	RbromiiL2-36
Org4_Gene355	accessory	2984	[RbromiiL2-36]RBL236_00371_hypothetical_protein	RbromiiL2-36
Org4_Gene36	accessory	530	[RbromiiL2-36]RBL236_00036_Proline_permease	RbromiiL2-36
Org4_Gene360	accessory	2636	[RbromiiL2-36]RBL236_00376_hypothetical_protein	RbromiiL2-36
Org4_Gene363	accessory	2043	[RbromiiL2-36]RBL236_00379_hypothetical_protein	RbromiiL2-36
Org4_Gene364	accessory	406	[RbromiiL2-36]RBL236_00380_Serine_protease_Do-like_HtrA	RbromiiL2-36
Org4_Gene366	accessory	2881	[RbromiiL2-36]RBL236_00382_Biotin_ECF_transporter_S_component_BioY	RbromiiL2-36
Org4_Gene367	accessory	3590	[RbromiiL2-36]RBL236_00383_hypothetical_protein	RbromiiL2-36
Org4_Gene37	accessory	2772	[RbromiiL2-36]RBL236_00037_Xanthine_phosphoribosyltransferase	RbromiiL2-36
Org4_Gene371	accessory	19	[RbromiiL2-36]RBL236_00387_fibro-slime_domain_protein	RbromiiL2-36
Org4_Gene372	accessory	662	[RbromiiL2-36]RBL236_00388_fimbrial_isopeptide_formation_D2_domain_protein	RbromiiL2-36
Org4_Gene373	accessory	1569	[RbromiiL2-36]RBL236_00389_sortase	RbromiiL2-36
Org4_Gene374	accessory	2076	[RbromiiL2-36]RBL236_00390_sortase	RbromiiL2-36
Org4_Gene375	accessory	1665	[RbromiiL2-36]RBL236_00391_hypothetical_protein	RbromiiL2-36
Org4_Gene376	accessory	2556	[RbromiiL2-36]RBL236_00392_hypothetical_protein	RbromiiL2-36
Org4_Gene379	accessory	2561	[RbromiiL2-36]RBL236_00395_Vitamin_B12_dependent_methionine_synthase,_activation_domain	RbromiiL2-36

Org4_Gene38	accessory	4308	[RbromiiL2-36]RBL236_00038_hypothetical_protein	RbromiiL2-36
Org4_Gene381	accessory	3393	[RbromiiL2-36]RBL236_00397_hypothetical_protein	RbromiiL2-36
Org4_Gene383	accessory	1054	[RbromiiL2-36]RBL236_00399_Putative_L,D-transpeptidase_YciB_precursor	RbromiiL2-36
Org4_Gene385	accessory	3085	[RbromiiL2-36]RBL236_00401_putative_HD_superfamily_hydrolase	RbromiiL2-36
Org4_Gene387	accessory	2180	[RbromiiL2-36]RBL236_00403_Pyruvate_formate-lyase-activating_enzyme	RbromiiL2-36
Org4_Gene388	accessory	4146	[RbromiiL2-36]RBL236_00404_hypothetical_protein	RbromiiL2-36
Org4_Gene389	accessory	342	[RbromiiL2-36]RBL236_00405_putative_integral_membrane_protein_linked_to_a_cation_pump	RbromiiL2-36
Org4_Gene39	accessory	589	[RbromiiL2-36]RBL236_00040_putative_ATPase	RbromiiL2-36
Org4_Gene390	accessory	3663	[RbromiiL2-36]RBL236_00406_hypothetical_protein	RbromiiL2-36
Org4_Gene391	accessory	2764	[RbromiiL2-36]RBL236_00407_Serine_acetyltransferase	RbromiiL2-36
Org4_Gene392	accessory	1127	[RbromiiL2-36]RBL236_00408_NADH_dehydrogenase_subunit_I	RbromiiL2-36
Org4_Gene393	accessory	1254	[RbromiiL2-36]RBL236_00409_Polysaccharide_pyruvyl_transferase	RbromiiL2-36
Org4_Gene394	accessory	1689	[RbromiiL2-36]RBL236_00410_Chondroitin_polymerase	RbromiiL2-36
Org4_Gene395	accessory	547	[RbromiiL2-36]RBL236_00411_putative_membrane_protein_EpsK	RbromiiL2-36
Org4_Gene396	accessory	1203	[RbromiiL2-36]RBL236_00412_Polysaccharide_pyruvyl_transferase	RbromiiL2-36
Org4_Gene397	accessory	910	[RbromiiL2-36]RBL236_00413_putative_O-glycosylation_ligase,_exosortase_A-associated	RbromiiL2-36
Org4_Gene398	accessory	1491	[RbromiiL2-36]RBL236_00414_Putative_glycosyltransferase_EpsH	RbromiiL2-36
Org4_Gene399	accessory	1049	[RbromiiL2-36]RBL236_00415_NADH_dehydrogenase_subunit_I	RbromiiL2-36
Org4_Gene40	accessory	2416	[RbromiiL2-36]RBL236_00041_hypothetical_protein	RbromiiL2-36
Org4_Gene400	accessory	1097	[RbromiiL2-36]RBL236_00416_Polysaccharide_pyruvyl_transferase	RbromiiL2-36
Org4_Gene401	accessory	942	[RbromiiL2-36]RBL236_00417_Glycosyltransferase_KanE	RbromiiL2-36
Org4_Gene402	accessory	1411	[RbromiiL2-36]RBL236_00418_General_stress_protein_A	RbromiiL2-36
Org4_Gene407	accessory	2462	[RbromiiL2-36]RBL236_00423_Undecaprenyl_phosphate_N,N'-diacetylbacillosamine_1-phosphate_transferase	RbromiiL2-36
Org4_Gene408	accessory	2326	[RbromiiL2-36]RBL236_00424_Tyrosine-protein_kinase_YwqD	RbromiiL2-36
Org4_Gene409	accessory	2288	[RbromiiL2-36]RBL236_00425_Capsular_polysaccharide_type_8_biosynthesis_protein_cap8A	RbromiiL2-36
Org4_Gene41	accessory	4038	[RbromiiL2-36]RBL236_00042_hypothetical_protein	RbromiiL2-36
Org4_Gene410	accessory	2727	[RbromiiL2-36]RBL236_00426_hypothetical_protein	RbromiiL2-36
Org4_Gene411	accessory	2281	[RbromiiL2-36]RBL236_00427_sortase,_marine_proteobacterial_type	RbromiiL2-36
Org4_Gene412	accessory	1941	[RbromiiL2-36]RBL236_00428_hypothetical_protein	RbromiiL2-36
Org4_Gene415	accessory	751	[RbromiiL2-36]RBL236_00431_Na+-dependent_transporters_of_the_SNF_family_protein	RbromiiL2-36
Org4_Gene416	accessory	218	[RbromiiL2-36]RBL236_00432_Polyphosphate_kinase	RbromiiL2-36
Org4_Gene417	accessory	1269	[RbromiiL2-36]RBL236_00433_Pyrophosphate--fructose_6-phosphate_1-phosphotransferase	RbromiiL2-36
Org4_Gene418	accessory	4322	[RbromiiL2-36]RBL236_00434_hypothetical_protein	RbromiiL2-36
Org4_Gene419	accessory	3449	[RbromiiL2-36]RBL236_00435_hypothetical_protein	RbromiiL2-36
Org4_Gene42	accessory	4107	[RbromiiL2-36]RBL236_00043_hypothetical_protein	RbromiiL2-36
Org4_Gene420	accessory	2930	[RbromiiL2-36]RBL236_00436_hypothetical_protein	RbromiiL2-36
Org4_Gene423	accessory	4279	[RbromiiL2-36]RBL236_00439_hypothetical_protein	RbromiiL2-36
Org4_Gene424	accessory	3666	[RbromiiL2-36]RBL236_00440_hypothetical_protein	RbromiiL2-36
Org4_Gene427	accessory	422	[RbromiiL2-36]RBL236_00443_Vancomycin_B-type_resistance_protein_VanW	RbromiiL2-36
Org4_Gene428	accessory	1154	[RbromiiL2-36]RBL236_00444_Processive_diacylglycerol_glucosyltransferase	RbromiiL2-36
Org4_Gene429	accessory	762	[RbromiiL2-36]RBL236_00445_Serine_protease_inhibitor	RbromiiL2-36
Org4_Gene43	accessory	2397	[RbromiiL2-36]RBL236_00044_hypothetical_protein	RbromiiL2-36
Org4_Gene434	accessory	786	[RbromiiL2-36]RBL236_00450_Cyclodextrin-binding_protein_precursor	RbromiiL2-36
Org4_Gene44	accessory	846	[RbromiiL2-36]RBL236_00045_Ribonucleases_G_and_E	RbromiiL2-36
Org4_Gene445	accessory	295	[RbromiiL2-36]RBL236_00461_sortase,_SrtB_family	RbromiiL2-36
Org4_Gene446	accessory	3382	[RbromiiL2-36]RBL236_00462_hypothetical_protein	RbromiiL2-36
Org4_Gene447	accessory	1041	[RbromiiL2-36]RBL236_00463_Cystathionine_gamma-synthase	RbromiiL2-36
Org4_Gene448	accessory	4128	[RbromiiL2-36]RBL236_00464_hypothetical_protein	RbromiiL2-36
Org4_Gene449	accessory	3403	[RbromiiL2-36]RBL236_00473_hypothetical_protein	RbromiiL2-36
Org4_Gene45	accessory	1778	[RbromiiL2-36]RBL236_00046_hypothetical_protein	RbromiiL2-36
Org4_Gene450	accessory	3402	[RbromiiL2-36]RBL236_00474_hypothetical_protein	RbromiiL2-36
Org4_Gene451	accessory	4179	[RbromiiL2-36]RBL236_00475_hypothetical_protein	RbromiiL2-36
Org4_Gene452	accessory	3343	[RbromiiL2-36]RBL236_00476_hypothetical_protein	RbromiiL2-36
Org4_Gene455	accessory	1928	[RbromiiL2-36]RBL236_00479_putative_phosphatase_YwpJ	RbromiiL2-36
Org4_Gene456	accessory	3405	[RbromiiL2-36]RBL236_00480_hypothetical_protein	RbromiiL2-36
Org4_Gene458	accessory	3242	[RbromiiL2-36]RBL236_00482_C_GCAxxG_C_C_family_protein	RbromiiL2-36
Org4_Gene46	accessory	1268	[RbromiiL2-36]RBL236_00047_hypothetical_protein	RbromiiL2-36
Org4_Gene460	accessory	1729	[RbromiiL2-36]RBL236_00484_Gamma-D-glutamyl-L-diamino_acid_endopeptidase_1	RbromiiL2-36
Org4_Gene462	accessory	1600	[RbromiiL2-36]RBL236_00486_Riboflavin_biosynthesis_protein_RibF	RbromiiL2-36
Org4_Gene463	accessory	4126	[RbromiiL2-36]RBL236_00487_hypothetical_protein	RbromiiL2-36
Org4_Gene464	accessory	4226	[RbromiiL2-36]RBL236_00488_hypothetical_protein	RbromiiL2-36
Org4_Gene465	accessory	821	[RbromiiL2-36]RBL236_00489_hypothetical_protein	RbromiiL2-36
Org4_Gene466	accessory	2336	[RbromiiL2-36]RBL236_00490_Sensory_transduction_protein_LytR	RbromiiL2-36
Org4_Gene467	accessory	1296	[RbromiiL2-36]RBL236_00491_Flp_pilus_assembly_protein,_ATPase_CpaE	RbromiiL2-36
Org4_Gene468	accessory	955	[RbromiiL2-36]RBL236_00492_Putative_conjugal_transfer_proteinc/MT3759	RbromiiL2-36
Org4_Gene469	accessory	1831	[RbromiiL2-36]RBL236_00493_Flp_pilus_assembly_protein_TadB	RbromiiL2-36
Org4_Gene47	accessory	512	[RbromiiL2-36]RBL236_00048_hypothetical_protein	RbromiiL2-36
Org4_Gene470	accessory	1787	[RbromiiL2-36]RBL236_00494_Flp_pilus_assembly_protein_TadB	RbromiiL2-36
Org4_Gene471	accessory	4122	[RbromiiL2-36]RBL236_00495_hypothetical_protein	RbromiiL2-36
Org4_Gene472	accessory	2487	[RbromiiL2-36]RBL236_00496_Flp_pilus_assembly_protein_TadG	RbromiiL2-36
Org4_Gene473	accessory	39	[RbromiiL2-36]RBL236_00497_hypothetical_protein	RbromiiL2-36
Org4_Gene474	accessory	2587	[RbromiiL2-36]RBL236_00498_hypothetical_protein	RbromiiL2-36
Org4_Gene475	accessory	803	[RbromiiL2-36]RBL236_00499_hypothetical_protein	RbromiiL2-36
Org4_Gene476	accessory	2470	[RbromiiL2-36]RBL236_00500_Late_competence_protein_ComC	RbromiiL2-36
Org4_Gene477	accessory	843	[RbromiiL2-36]RBL236_00501_hypothetical_protein	RbromiiL2-36
Org4_Gene478	accessory	498	[RbromiiL2-36]RBL236_00502_type_VI_secretion_system_FHA_domain_protein	RbromiiL2-36
Org4_Gene479	accessory	2128	[RbromiiL2-36]RBL236_00503_Putative_protein_phosphatase_2C-type	RbromiiL2-36
Org4_Gene480	accessory	433	[RbromiiL2-36]RBL236_00504_Serine/threonine-protein_kinase_PrkC	RbromiiL2-36
Org4_Gene481	accessory	2956	[RbromiiL2-36]RBL236_00505_hypothetical_protein	RbromiiL2-36
Org4_Gene482	accessory	3125	[RbromiiL2-36]RBL236_00506_hypothetical_protein	RbromiiL2-36
Org4_Gene483	accessory	8	[RbromiiL2-36]RBL236_00507_hypothetical_protein	RbromiiL2-36
Org4_Gene484	accessory	1990	[RbromiiL2-36]RBL236_00508_hypothetical_protein	RbromiiL2-36
Org4_Gene485	accessory	678	[RbromiiL2-36]RBL236_00509_Na(+)/drug_antiporter	RbromiiL2-36
Org4_Gene486	accessory	2715	[RbromiiL2-36]RBL236_00510_LOG_family_protein_YvdD	RbromiiL2-36
Org4_Gene488	accessory	1981	[RbromiiL2-36]RBL236_00512_hypothetical_protein	RbromiiL2-36
Org4_Gene489	accessory	2017	[RbromiiL2-36]RBL236_00513_hypothetical_protein	RbromiiL2-36
Org4_Gene49	accessory	2385	[RbromiiL2-36]RBL236_00050_Zn-dependent_proteases	RbromiiL2-36
Org4_Gene490	accessory	2062	[RbromiiL2-36]RBL236_00514_hypothetical_protein	RbromiiL2-36
Org4_Gene491	accessory	2212	[RbromiiL2-36]RBL236_00515_Signal_peptidase_I_W	RbromiiL2-36
Org4_Gene492	accessory	552	[RbromiiL2-36]RBL236_00516_hypothetical_protein	RbromiiL2-36
Org4_Gene493	accessory	130	[RbromiiL2-36]RBL236_00517_chromosome_segregation_protein_SMC	RbromiiL2-36
Org4_Gene494	accessory	1371	[RbromiiL2-36]RBL236_00518_hypothetical_protein	RbromiiL2-36
Org4_Gene495	accessory	394	[RbromiiL2-36]RBL236_00519_hypothetical_protein	RbromiiL2-36
Org4_Gene496	accessory	20	[RbromiiL2-36]RBL236_00520_repeat	RbromiiL2-36
Org4_Gene497	accessory	982	[RbromiiL2-36]RBL236_00521_hypothetical_protein	RbromiiL2-36
Org4_Gene498	accessory	2216	[RbromiiL2-36]RBL236_00522_Signal_peptidase_I_W	RbromiiL2-36
Org4_Gene500	accessory	198	[RbromiiL2-36]RBL236_00524_Beta/alpha-amylase_precursor	RbromiiL2-36
Org4_Gene502	accessory	3241	[RbromiiL2-36]RBL236_00526_hypothetical_protein	RbromiiL2-36
Org4_Gene503	accessory	1580	[RbromiiL2-36]RBL236_00527_auxin_efflux_carrier	RbromiiL2-36
Org4_Gene504	accessory	1425	[RbromiiL2-36]RBL236_00528_hypothetical_protein	RbromiiL2-36
Org4_Gene505	accessory	1840	[RbromiiL2-36]RBL236_00529_Regulatory_protein_SoxS	RbromiiL2-36
Org4_Gene506	accessory	2025	[RbromiiL2-36]RBL236_00530_hypothetical_protein	RbromiiL2-36
Org4_Gene507	accessory	3569	[RbromiiL2-36]RBL236_00531_hypothetical_protein	RbromiiL2-36
Org4_Gene510	accessory	557	[RbromiiL2-36]RBL236_00535_putative_protein_involved_in_cytokinesis,_contains_TGc_(transglutaminase/protease-like)_domain	RbromiiL2-36
Org4_Gene511	accessory	1060	[RbromiiL2-36]RBL236_00536_Alanine_racemase	RbromiiL2-36
Org4_Gene512	accessory	1372	[RbromiiL2-36]RBL236_00537_hypothetical_protein	RbromiiL2-36
Org4_Gene513	accessory	950	[RbromiiL2-36]RBL236_00539_Fucose_4-O-acetylase	RbromiiL2-36
Org4_Gene514	accessory	2299	[RbromiiL2-36]RBL236_00540_SynChlorMet_cassette_protein_ScmC	RbromiiL2-36
Org4_Gene516	accessory	1931	[RbromiiL2-36]RBL236_00542_transcriptional_activator_RhaS	RbromiiL2-36
Org4_Gene517	accessory	1882	[RbromiiL2-36]RBL236_00543_putative_metallophosphoesterase	RbromiiL2-36
Org4_Gene52	accessory	2625	[RbromiiL2-36]RBL236_00053_hypothetical_protein	RbromiiL2-36
Org4_Gene521	accessory	3108	[RbromiiL2-36]RBL236_00555_Small_T_antigen	RbromiiL2-36
Org4_Gene522	accessory	3292	[RbromiiL2-36]RBL236_00556_hypothetical_protein	RbromiiL2-36
Org4_Gene523	accessory	1740	[RbromiiL2-36]RBL236_00557_hypothetical_protein	RbromiiL2-36
Org4_Gene524	accessory	3421	[RbromiiL2-36]RBL236_00558_hypothetical_protein	RbromiiL2-36
Org4_Gene525	accessory	2217	[RbromiiL2-36]RBL236_00559_hypothetical_protein	RbromiiL2-36
Org4_Gene526	accessory	2292	[RbromiiL2-36]RBL236_00560_type_VII_secretion_protein_EccCa	RbromiiL2-36
Org4_Gene527	accessory	3715	[RbromiiL2-36]RBL236_00561_hypothetical_protein	RbromiiL2-36
Org4_Gene528	accessory	860	[RbromiiL2-36]RBL236_00562_Replication_initiation_factor	RbromiiL2-36
Org4_Gene529	accessory	3140	[RbromiiL2-36]RBL236_00563_hypothetical_protein	RbromiiL2-36
Org4_Gene530	accessory	3345	[RbromiiL2-36]RBL236_00564_hypothetical_protein	RbromiiL2-36
Org4_Gene531	accessory	4328	[RbromiiL2-36]RBL236_00565_hypothetical_protein	RbromiiL2-36

Org4_Gene532	accessory	1019	[RbromiiL2-36]RBL236_00566_Integrase	RbromiiL2-36
Org4_Gene534	accessory	1699	[RbromiiL2-36]RBL236_00568_hypothetical_protein	RbromiiL2-36
Org4_Gene535	accessory	2534	[RbromiiL2-36]RBL236_00569_Response_regulator_ArlR	RbromiiL2-36
Org4_Gene536	accessory	646	[RbromiiL2-36]RBL236_00570_Signal_transduction_histidine-protein_kinase_ArlS	RbromiiL2-36
Org4_Gene537	accessory	2117	[RbromiiL2-36]RBL236_00571_hypothetical_protein	RbromiiL2-36
Org4_Gene538	accessory	1420	[RbromiiL2-36]RBL236_00572_Lipid_II:glycine_glycyltransferase	RbromiiL2-36
Org4_Gene539	accessory	1862	[RbromiiL2-36]RBL236_00573_Error-prone_DNA_polymerase	RbromiiL2-36
Org4_Gene54	accessory	1186	[RbromiiL2-36]RBL236_00055_D-alanyl-D-alanine_carboxypeptidase_DacB_precursor	RbromiiL2-36
Org4_Gene540	accessory	935	[RbromiiL2-36]RBL236_00574_hypothetical_protein	RbromiiL2-36
Org4_Gene543	accessory	4288	[RbromiiL2-36]RBL236_00577_hypothetical_protein	RbromiiL2-36
Org4_Gene544	accessory	4285	[RbromiiL2-36]RBL236_00578_hypothetical_protein	RbromiiL2-36
Org4_Gene545	accessory	3461	[RbromiiL2-36]RBL236_00579_hypothetical_protein	RbromiiL2-36
Org4_Gene546	accessory	1843	[RbromiiL2-36]RBL236_00580_hypothetical_protein	RbromiiL2-36
Org4_Gene547	accessory	994	[RbromiiL2-36]RBL236_00581_H(+)/Cl(-)_exchange_transporter_ClcA	RbromiiL2-36
Org4_Gene548	accessory	1482	[RbromiiL2-36]RBL236_00582_Choloylglycine_hydrolase	RbromiiL2-36
Org4_Gene549	accessory	4268	[RbromiiL2-36]RBL236_00583_hypothetical_protein	RbromiiL2-36
Org4_Gene550	accessory	1055	[RbromiiL2-36]RBL236_00584_Glutamate_permease	RbromiiL2-36
Org4_Gene551	accessory	1530	[RbromiiL2-36]RBL236_00585_TRAP_transporter_solute_receptor,_TAXI_family	RbromiiL2-36
Org4_Gene553	accessory	631	[RbromiiL2-36]RBL236_00587_cell_division_protein_FtsQ	RbromiiL2-36
Org4_Gene559	accessory	2882	[RbromiiL2-36]RBL236_00593_hypothetical_protein	RbromiiL2-36
Org4_Gene561	accessory	3379	[RbromiiL2-36]RBL236_00595_cell_division_protein_MraZ	RbromiiL2-36
Org4_Gene562	accessory	4436	[RbromiiL2-36]RBL236_00596_hypothetical_protein	RbromiiL2-36
Org4_Gene564	accessory	3116	[RbromiiL2-36]RBL236_00598_Phosphopantetheine_adenylyltransferase	RbromiiL2-36
Org4_Gene574	accessory	3080	[RbromiiL2-36]RBL236_00608_Undecaprenol_kinase	RbromiiL2-36
Org4_Gene581	accessory	3082	[RbromiiL2-36]RBL236_00615_hypothetical_protein	RbromiiL2-36
Org4_Gene586	accessory	3185	[RbromiiL2-36]RBL236_00620_hypothetical_protein	RbromiiL2-36
Org4_Gene587	accessory	2938	[RbromiiL2-36]RBL236_00621_hypothetical_protein	RbromiiL2-36
Org4_Gene588	accessory	3141	[RbromiiL2-36]RBL236_00622_hypothetical_protein	RbromiiL2-36
Org4_Gene59	accessory	774	[RbromiiL2-36]RBL236_00060_hypothetical_protein	RbromiiL2-36
Org4_Gene590	accessory	3198	[RbromiiL2-36]RBL236_00624_hypothetical_protein	RbromiiL2-36
Org4_Gene591	accessory	3124	[RbromiiL2-36]RBL236_00625_hypothetical_protein	RbromiiL2-36
Org4_Gene592	accessory	3142	[RbromiiL2-36]RBL236_00626_hypothetical_protein	RbromiiL2-36
Org4_Gene593	accessory	1187	[RbromiiL2-36]RBL236_00627_hypothetical_protein	RbromiiL2-36
Org4_Gene594	accessory	493	[RbromiiL2-36]RBL236_00628_hypothetical_protein	RbromiiL2-36
Org4_Gene596	accessory	2728	[RbromiiL2-36]RBL236_00630_Stage_0_sporulation_protein_H	RbromiiL2-36
Org4_Gene597	accessory	556	[RbromiiL2-36]RBL236_00631_Competence_protein_ComM	RbromiiL2-36
Org4_Gene599	accessory	2486	[RbromiiL2-36]RBL236_00633_tRNA_(adenine(22)-N(1))-methyltransferase	RbromiiL2-36
Org4_Gene60	accessory	2340	[RbromiiL2-36]RBL236_00061_hypothetical_protein	RbromiiL2-36
Org4_Gene600	accessory	2171	[RbromiiL2-36]RBL236_00634_metal-binding_protein	RbromiiL2-36
Org4_Gene602	accessory	785	[RbromiiL2-36]RBL236_00636_hypothetical_protein	RbromiiL2-36
Org4_Gene606	accessory	1895	[RbromiiL2-36]RBL236_00640_Release_factor_glutamine_methyltransferase	RbromiiL2-36
Org4_Gene61	accessory	3525	[RbromiiL2-36]RBL236_00062_hypothetical_protein	RbromiiL2-36
Org4_Gene614	accessory	2812	[RbromiiL2-36]RBL236_00648_hypothetical_protein	RbromiiL2-36
Org4_Gene615	accessory	3751	[RbromiiL2-36]RBL236_00649_Putative_PBSX_repressor	RbromiiL2-36
Org4_Gene616	accessory	2135	[RbromiiL2-36]RBL236_00650_hypothetical_protein	RbromiiL2-36
Org4_Gene617	accessory	588	[RbromiiL2-36]RBL236_00651_Fibronectin_type_III_domain_protein	RbromiiL2-36
Org4_Gene618	accessory	3701	[RbromiiL2-36]RBL236_00652_hypothetical_protein	RbromiiL2-36
Org4_Gene619	accessory	1670	[RbromiiL2-36]RBL236_00653_Autolytic_lysozyme	RbromiiL2-36
Org4_Gene62	accessory	62	[RbromiiL2-36]RBL236_00063_Type_I_restriction_enzyme_EcoR124II_R_protein	RbromiiL2-36
Org4_Gene621	accessory	526	[RbromiiL2-36]RBL236_00655_Murein_hydrolase_activator_NlpD_precursor	RbromiiL2-36
Org4_Gene624	accessory	2449	[RbromiiL2-36]RBL236_00658_hypothetical_protein	RbromiiL2-36
Org4_Gene625	accessory	3641	[RbromiiL2-36]RBL236_00659_hypothetical_protein	RbromiiL2-36
Org4_Gene626	accessory	3165	[RbromiiL2-36]RBL236_00660_flavodoxin	RbromiiL2-36
Org4_Gene628	accessory	3036	[RbromiiL2-36]RBL236_00662_flavodoxin	RbromiiL2-36
Org4_Gene63	accessory	362	[RbromiiL2-36]RBL236_00064_KAP_family_P-loop_domain_protein	RbromiiL2-36
Org4_Gene630	accessory	759	[RbromiiL2-36]RBL236_00664_D-alanyl-D-alanine_carboxypeptidase_DacB_precursor	RbromiiL2-36
Org4_Gene634	accessory	1077	[RbromiiL2-36]RBL236_00668_sporulation_protein_YqfD	RbromiiL2-36
Org4_Gene635	accessory	3933	[RbromiiL2-36]RBL236_00669_sporulation_protein_YqfC	RbromiiL2-36
Org4_Gene636	accessory	1279	[RbromiiL2-36]RBL236_00670_Sporulation_integral_membrane_protein_YlbJ	RbromiiL2-36
Org4_Gene638	accessory	3822	[RbromiiL2-36]RBL236_00672_Putative_undecaprenyl-diphosphatase_YbjG	RbromiiL2-36
Org4_Gene639	accessory	223	[RbromiiL2-36]RBL236_00673_putative_cell_wall_hydrolase_LytN_precursor	RbromiiL2-36
Org4_Gene64	accessory	1209	[RbromiiL2-36]RBL236_00065_Type_I_restriction_modification_DNA_specificity_domain_protein	RbromiiL2-36
Org4_Gene640	accessory	4090	[RbromiiL2-36]RBL236_00674_putative_transcriptional_regulator	RbromiiL2-36
Org4_Gene643	accessory	1906	[RbromiiL2-36]RBL236_00677_ComEC_family_competence_protein	RbromiiL2-36
Org4_Gene645	accessory	104	[RbromiiL2-36]RBL236_00679_Stage_III_sporulation_protein_E	RbromiiL2-36
Org4_Gene649	accessory	3481	[RbromiiL2-36]RBL236_00683_putative_membrane_protein	RbromiiL2-36
Org4_Gene65	accessory	440	[RbromiiL2-36]RBL236_00066_Type_I_restriction_enzyme_EcoKI_M_protein	RbromiiL2-36
Org4_Gene651	accessory	1334	[RbromiiL2-36]RBL236_00685_DNA_polymerase_III_subunit_delta	RbromiiL2-36
Org4_Gene652	accessory	204	[RbromiiL2-36]RBL236_00686_DNA_internalization-related_competence_protein_ComEC/Rec2	RbromiiL2-36
Org4_Gene653	accessory	2974	[RbromiiL2-36]RBL236_00687_hypothetical_protein	RbromiiL2-36
Org4_Gene656	accessory	2279	[RbromiiL2-36]RBL236_00690_G3P_acyltransferase	RbromiiL2-36
Org4_Gene658	accessory	848	[RbromiiL2-36]RBL236_00692_putative_FeS-containing_Cyanobacterial-specific_oxidoreductase	RbromiiL2-36
Org4_Gene66	accessory	4176	[RbromiiL2-36]RBL236_00067_hypothetical_protein	RbromiiL2-36
Org4_Gene661	accessory	2390	[RbromiiL2-36]RBL236_00695_hypothetical_protein	RbromiiL2-36
Org4_Gene662	accessory	2328	[RbromiiL2-36]RBL236_00696_hypothetical_protein	RbromiiL2-36
Org4_Gene663	accessory	3526	[RbromiiL2-36]RBL236_00697_Penicillinase_repressor	RbromiiL2-36
Org4_Gene664	accessory	1033	[RbromiiL2-36]RBL236_00698_Regulatory_protein_BlaR1	RbromiiL2-36
Org4_Gene665	accessory	3816	[RbromiiL2-36]RBL236_00699_hypothetical_protein	RbromiiL2-36
Org4_Gene666	accessory	1092	[RbromiiL2-36]RBL236_00700_RmuC_family_protein	RbromiiL2-36
Org4_Gene668	accessory	1394	[RbromiiL2-36]RBL236_00702_Putative_bifunctional_phosphatase/peptidyl-prolyl_cis-trans_isomerase	RbromiiL2-36
Org4_Gene67	accessory	3679	[RbromiiL2-36]RBL236_00068_hypothetical_protein	RbromiiL2-36
Org4_Gene672	accessory	1025	[RbromiiL2-36]RBL236_00706_hypothetical_protein	RbromiiL2-36
Org4_Gene673	accessory	2002	[RbromiiL2-36]RBL236_00707_Sulfate_starvation-induced_protein_7	RbromiiL2-36
Org4_Gene676	accessory	3499	[RbromiiL2-36]RBL236_00710_hypothetical_protein	RbromiiL2-36
Org4_Gene677	accessory	878	[RbromiiL2-36]RBL236_00711_Putative_metal_chaperone_YciC	RbromiiL2-36
Org4_Gene678	accessory	2837	[RbromiiL2-36]RBL236_00712_Septum_formation_protein_Maf	RbromiiL2-36
Org4_Gene679	accessory	1608	[RbromiiL2-36]RBL236_00713_Serine_acetyltransferase	RbromiiL2-36
Org4_Gene68	accessory	1975	[RbromiiL2-36]RBL236_00069_putative_chromosome-partitioning_protein_ParB	RbromiiL2-36
Org4_Gene680	accessory	2360	[RbromiiL2-36]RBL236_00714_Transglutaminase-like_superfamily_protein	RbromiiL2-36
Org4_Gene681	accessory	3013	[RbromiiL2-36]RBL236_00715_ribosomal-protein-alanine_acetyltransferase	RbromiiL2-36
Org4_Gene682	accessory	3151	[RbromiiL2-36]RBL236_00716_putative_acetyltransferase	RbromiiL2-36
Org4_Gene683	accessory	789	[RbromiiL2-36]RBL236_00717_Na+-dependent_transporters_of_the_SNF_family_protein	RbromiiL2-36
Org4_Gene684	accessory	1741	[RbromiiL2-36]RBL236_00718_Cyn_operon_transcriptional_activator	RbromiiL2-36
Org4_Gene686	accessory	3963	[RbromiiL2-36]RBL236_00720_CotJB_protein	RbromiiL2-36
Org4_Gene687	accessory	4215	[RbromiiL2-36]RBL236_00721_Spore_coat_associated_protein_JA_(CotJA)	RbromiiL2-36
Org4_Gene688	accessory	1933	[RbromiiL2-36]RBL236_00722_Thiamine_ECF_transporter_S_component_ThiT	RbromiiL2-36
Org4_Gene69	accessory	4053	[RbromiiL2-36]RBL236_00070_hypothetical_protein	RbromiiL2-36
Org4_Gene691	accessory	2700	[RbromiiL2-36]RBL236_00726_Alpha/beta_hydrolase_family_protein	RbromiiL2-36
Org4_Gene692	accessory	3193	[RbromiiL2-36]RBL236_00727_hypothetical_protein	RbromiiL2-36
Org4_Gene697	accessory	4335	[RbromiiL2-36]RBL236_00732_Virus_attachment_protein_p12_family_protein	RbromiiL2-36
Org4_Gene698	accessory	2386	[RbromiiL2-36]RBL236_00733_Bacitracin_export_ATP-binding_protein_BceA	RbromiiL2-36
Org4_Gene699	accessory	202	[RbromiiL2-36]RBL236_00734_FtsX-like_permease_family_protein	RbromiiL2-36
Org4_Gene7	accessory	514	[RbromiiL2-36]RBL236_00007_Internalin-A_precursor	RbromiiL2-36
Org4_Gene70	accessory	4206	[RbromiiL2-36]RBL236_00071_hypothetical_protein	RbromiiL2-36
Org4_Gene700	accessory	3556	[RbromiiL2-36]RBL236_00735_putative_mercuric_reductase	RbromiiL2-36
Org4_Gene701	accessory	1531	[RbromiiL2-36]RBL236_00736_Regulatory_protein_SoxS	RbromiiL2-36
Org4_Gene702	accessory	2862	[RbromiiL2-36]RBL236_00737_hypothetical_protein	RbromiiL2-36
Org4_Gene707	accessory	3105	[RbromiiL2-36]RBL236_00743_Putative_phosphinothricin_acetyltransferase_YwnH	RbromiiL2-36
Org4_Gene708	accessory	3696	[RbromiiL2-36]RBL236_00744_hypothetical_protein	RbromiiL2-36
Org4_Gene709	accessory	4130	[RbromiiL2-36]RBL236_00745_hypothetical_protein	RbromiiL2-36
Org4_Gene71	accessory	447	[RbromiiL2-36]RBL236_00072_hypothetical_protein	RbromiiL2-36
Org4_Gene710	accessory	3287	[RbromiiL2-36]RBL236_00746_hypothetical_protein	RbromiiL2-36
Org4_Gene711	accessory	4066	[RbromiiL2-36]RBL236_00747_Antitoxin_YefM	RbromiiL2-36
Org4_Gene712	accessory	3921	[RbromiiL2-36]RBL236_00748_addiction_module_toxin,_Txe/YoeB_family	RbromiiL2-36
Org4_Gene713	accessory	1853	[RbromiiL2-36]RBL236_00749_Pyridoxine_kinase	RbromiiL2-36
Org4_Gene714	accessory	2872	[RbromiiL2-36]RBL236_00750_putative_membrane_protein	RbromiiL2-36
Org4_Gene715	accessory	2678	[RbromiiL2-36]RBL236_00751_molybdenum_cofactor_biosynthesis_protein_A	RbromiiL2-36
Org4_Gene716	accessory	2713	[RbromiiL2-36]RBL236_00752_Ion_channel	RbromiiL2-36
Org4_Gene717	accessory	752	[RbromiiL2-36]RBL236_00753_Na+-dependent_transporters_of_the_SNF_family_protein	RbromiiL2-36
Org4_Gene718	accessory	1713	[RbromiiL2-36]RBL236_00754_hypothetical_protein	RbromiiL2-36
Org4_Gene719	accessory	3510	[RbromiiL2-36]RBL236_00755_hypothetical_protein	RbromiiL2-36
Org4_Gene72	accessory	3978	[RbromiiL2-36]RBL236_00073_Helix-turn-helix_domain_protein	RbromiiL2-36

Org4_Gene720	accessory	2571	[RbromiiL2-36]RBL236_00756_hypothetical_protein	RbromiiL2-36
Org4_Gene721	accessory	2783	[RbromiiL2-36]RBL236_00757_Putative_beta-lactamase_HcpC_precursor	RbromiiL2-36
Org4_Gene723	accessory	698	[RbromiiL2-36]RBL236_00760_putative_adenine_permease_PurP	RbromiiL2-36
Org4_Gene724	accessory	3351	[RbromiiL2-36]RBL236_00761_Cysteine_metabolism_repressor	RbromiiL2-36
Org4_Gene726	accessory	1599	[RbromiiL2-36]RBL236_00763_Cysteine_synthase	RbromiiL2-36
Org4_Gene727	accessory	2651	[RbromiiL2-36]RBL236_00764_N-(5'-phosphoribosyl)anthranilate_isomerase	RbromiiL2-36
Org4_Gene73	accessory	3304	[RbromiiL2-36]RBL236_00074_RNA_polymerase_sigma_factor	RbromiiL2-36
Org4_Gene731	accessory	3927	[RbromiiL2-36]RBL236_00768_mRNA_interferase_YafQ	RbromiiL2-36
Org4_Gene732	accessory	3952	[RbromiiL2-36]RBL236_00769_addiction_module_antitoxin,_RelB/DinJ_family	RbromiiL2-36
Org4_Gene735	accessory	3941	[RbromiiL2-36]RBL236_00772_Antitoxin_DinJ	RbromiiL2-36
Org4_Gene74	accessory	1452	[RbromiiL2-36]RBL236_00075_putative_dual-specificity_RNA_methyltransferase_RlmN	RbromiiL2-36
Org4_Gene740	accessory	2902	[RbromiiL2-36]RBL236_00778_hypothetical_protein	RbromiiL2-36
Org4_Gene741	accessory	1792	[RbromiiL2-36]RBL236_00779_Nicotinamide_nucleotide_repair_protein	RbromiiL2-36
Org4_Gene742	accessory	2787	[RbromiiL2-36]RBL236_00780_putative_nicotinate-nucleotide_adenylyltransferase	RbromiiL2-36
Org4_Gene743	accessory	3437	[RbromiiL2-36]RBL236_00781_hypothetical_protein	RbromiiL2-36
Org4_Gene745	accessory	699	[RbromiiL2-36]RBL236_00783_Magnesium_transporter_MgtE	RbromiiL2-36
Org4_Gene746	accessory	2884	[RbromiiL2-36]RBL236_00784_Cd1	RbromiiL2-36
Org4_Gene747	accessory	2988	[RbromiiL2-36]RBL236_00785_hypothetical_protein	RbromiiL2-36
Org4_Gene749	accessory	2478	[RbromiiL2-36]RBL236_00787_Putative_PBSX_repressor	RbromiiL2-36
Org4_Gene75	accessory	4294	[RbromiiL2-36]RBL236_00076_hypothetical_protein	RbromiiL2-36
Org4_Gene752	accessory	2242	[RbromiiL2-36]RBL236_00790_Tyrosine-protein_phosphatase_CpsB	RbromiiL2-36
Org4_Gene757	accessory	294	[RbromiiL2-36]RBL236_00795_Bifunctional_oligoribonuclease_and_PAP_phosphatase_NrnA	RbromiiL2-36
Org4_Gene760	accessory	804	[RbromiiL2-36]RBL236_00798_tRNA(Ile)-lysidine_synthase	RbromiiL2-36
Org4_Gene765	accessory	4071	[RbromiiL2-36]RBL236_00803_hypothetical_protein	RbromiiL2-36
Org4_Gene766	accessory	2112	[RbromiiL2-36]RBL236_00804_N-acetylmuramoyl-L-alanine_amidase	RbromiiL2-36
Org4_Gene767	accessory	696	[RbromiiL2-36]RBL236_00805_Multidrug_export_protein_MepA	RbromiiL2-36
Org4_Gene768	accessory	4191	[RbromiiL2-36]RBL236_00806_hypothetical_protein	RbromiiL2-36
Org4_Gene769	accessory	3835	[RbromiiL2-36]RBL236_00807_RNA_polymerase_sigma_factor	RbromiiL2-36
Org4_Gene77	accessory	3724	[RbromiiL2-36]RBL236_00078_Bacterial_mobilisation_protein_(MobC)	RbromiiL2-36
Org4_Gene770	accessory	2657	[RbromiiL2-36]RBL236_00808_hypothetical_protein	RbromiiL2-36
Org4_Gene771	accessory	842	[RbromiiL2-36]RBL236_00809_hypothetical_protein	RbromiiL2-36
Org4_Gene773	accessory	2014	[RbromiiL2-36]RBL236_00812_D-alanyl-D-alanine_carboxypeptidase	RbromiiL2-36
Org4_Gene779	accessory	3818	[RbromiiL2-36]RBL236_00818_Muramidase-2_precursor	RbromiiL2-36
Org4_Gene78	accessory	771	[RbromiiL2-36]RBL236_00079_Relaxase/Mobilisation_nuclease_domain_protein	RbromiiL2-36
Org4_Gene780	accessory	930	[RbromiiL2-36]RBL236_00819_Enolase	RbromiiL2-36
Org4_Gene782	accessory	71	[RbromiiL2-36]RBL236_00821_Pullulanase_precursor	RbromiiL2-36
Org4_Gene787	accessory	1731	[RbromiiL2-36]RBL236_00826_sporulation_inhibitor_KapD	RbromiiL2-36
Org4_Gene788	accessory	3137	[RbromiiL2-36]RBL236_00827_hypothetical_protein	RbromiiL2-36
Org4_Gene789	accessory	427	[RbromiiL2-36]RBL236_00828_Serine_protease_Do-like_HtrB	RbromiiL2-36
Org4_Gene79	accessory	4018	[RbromiiL2-36]RBL236_00080_Helix-turn-helix_domain_protein	RbromiiL2-36
Org4_Gene790	accessory	2313	[RbromiiL2-36]RBL236_00829_Sensory_transduction_protein_LytR	RbromiiL2-36
Org4_Gene791	accessory	303	[RbromiiL2-36]RBL236_00830_hypothetical_protein	RbromiiL2-36
Org4_Gene792	accessory	1194	[RbromiiL2-36]RBL236_00831_hypothetical_protein	RbromiiL2-36
Org4_Gene795	accessory	2542	[RbromiiL2-36]RBL236_00834_hypothetical_protein	RbromiiL2-36
Org4_Gene796	accessory	2563	[RbromiiL2-36]RBL236_00835_Demethylrebeccamycin-D-glucose_O-methyltransferase	RbromiiL2-36
Org4_Gene798	accessory	1507	[RbromiiL2-36]RBL236_00837_ribonuclease_BN/unknown_domain_fusion_protein	RbromiiL2-36
Org4_Gene799	accessory	2276	[RbromiiL2-36]RBL236_00838_hypothetical_protein	RbromiiL2-36
Org4_Gene8	accessory	915	[RbromiiL2-36]RBL236_00008_Peptidase_M16_inactive_domain_protein	RbromiiL2-36
Org4_Gene80	accessory	4199	[RbromiiL2-36]RBL236_00081_hypothetical_protein	RbromiiL2-36
Org4_Gene800	accessory	3132	[RbromiiL2-36]RBL236_00839_transcriptional_regulator_SlyA	RbromiiL2-36
Org4_Gene803	accessory	1630	[RbromiiL2-36]RBL236_00842_ComEC_family_competence_protein	RbromiiL2-36
Org4_Gene804	accessory	4143	[RbromiiL2-36]RBL236_00843_hypothetical_protein	RbromiiL2-36
Org4_Gene805	accessory	3133	[RbromiiL2-36]RBL236_00844_hypothetical_protein	RbromiiL2-36
Org4_Gene806	accessory	2085	[RbromiiL2-36]RBL236_00845_Transcriptional_regulatory_protein_YycF	RbromiiL2-36
Org4_Gene808	accessory	790	[RbromiiL2-36]RBL236_00847_Sensor_histidine_kinase_YycG	RbromiiL2-36
Org4_Gene809	accessory	567	[RbromiiL2-36]RBL236_00848_Tyrocidine_synthase_I	RbromiiL2-36
Org4_Gene81	accessory	1542	[RbromiiL2-36]RBL236_00082_hypothetical_protein	RbromiiL2-36
Org4_Gene810	accessory	1073	[RbromiiL2-36]RBL236_00849_L-glutamyl-[BtrI_acyl-carrier_protein]_decarboxylase	RbromiiL2-36
Org4_Gene811	accessory	4091	[RbromiiL2-36]RBL236_00850_D-alanine--poly(phosphoribitol)_ligase_subunit_2	RbromiiL2-36
Org4_Gene812	accessory	482	[RbromiiL2-36]RBL236_00851_D-alanyl-lipoteichoic_acid_biosynthesis_protein_DltB	RbromiiL2-36
Org4_Gene813	accessory	1470	[RbromiiL2-36]RBL236_00852_hypothetical_protein	RbromiiL2-36
Org4_Gene814	accessory	795	[RbromiiL2-36]RBL236_00854_hypothetical_protein	RbromiiL2-36
Org4_Gene815	accessory	45	[RbromiiL2-36]RBL236_00855_hypothetical_protein	RbromiiL2-36
Org4_Gene816	accessory	4400	[RbromiiL2-36]RBL236_00856_hypothetical_protein	RbromiiL2-36
Org4_Gene817	accessory	1811	[RbromiiL2-36]RBL236_00857_hypothetical_protein	RbromiiL2-36
Org4_Gene818	accessory	2323	[RbromiiL2-36]RBL236_00858_hypothetical_protein	RbromiiL2-36
Org4_Gene82	accessory	49	[RbromiiL2-36]RBL236_00083_Antirestriction_protein	RbromiiL2-36
Org4_Gene820	accessory	1087	[RbromiiL2-36]RBL236_00860_CYTH_domain_protein	RbromiiL2-36
Org4_Gene821	accessory	392	[RbromiiL2-36]RBL236_00865_1-deoxy-D-xylulose-5-phosphate_synthase	RbromiiL2-36
Org4_Gene823	accessory	3755	[RbromiiL2-36]RBL236_00867_hypothetical_protein	RbromiiL2-36
Org4_Gene827	accessory	2707	[RbromiiL2-36]RBL236_00871_ATP_synthase_(E/31_kDa)_subunit	RbromiiL2-36
Org4_Gene83	accessory	250	[RbromiiL2-36]RBL236_00084_DNA_topoisomerase_3	RbromiiL2-36
Org4_Gene830	accessory	305	[RbromiiL2-36]RBL236_00874_V-type_ATP_synthase_subunit_I	RbromiiL2-36
Org4_Gene832	accessory	3794	[RbromiiL2-36]RBL236_00876_hypothetical_protein	RbromiiL2-36
Org4_Gene835	accessory	1969	[RbromiiL2-36]RBL236_00879_Histidinol-phosphatase	RbromiiL2-36
Org4_Gene837	accessory	1338	[RbromiiL2-36]RBL236_00881_hypothetical_protein	RbromiiL2-36
Org4_Gene84	accessory	4274	[RbromiiL2-36]RBL236_00085_hypothetical_protein	RbromiiL2-36
Org4_Gene840	accessory	4412	[RbromiiL2-36]RBL236_00884_hypothetical_protein	RbromiiL2-36
Org4_Gene841	accessory	4008	[RbromiiL2-36]RBL236_00885_TSCPD_domain_protein	RbromiiL2-36
Org4_Gene842	accessory	1231	[RbromiiL2-36]RBL236_00886_DnaD_domain_protein	RbromiiL2-36
Org4_Gene843	accessory	1458	[RbromiiL2-36]RBL236_00887_DNA_replication_protein_DnaC	RbromiiL2-36
Org4_Gene844	accessory	3200	[RbromiiL2-36]RBL236_00890_hypothetical_protein	RbromiiL2-36
Org4_Gene847	accessory	1008	[RbromiiL2-36]RBL236_00893_Penicillin-binding_protein_E	RbromiiL2-36
Org4_Gene848	accessory	1814	[RbromiiL2-36]RBL236_00894_hypothetical_protein	RbromiiL2-36
Org4_Gene849	accessory	739	[RbromiiL2-36]RBL236_00895_HTH-type_transcriptional_regulatory_protein_GabR	RbromiiL2-36
Org4_Gene85	accessory	2334	[RbromiiL2-36]RBL236_00086_hypothetical_protein	RbromiiL2-36
Org4_Gene851	accessory	1370	[RbromiiL2-36]RBL236_00897_Putative_aminopeptidase_YsdC	RbromiiL2-36
Org4_Gene852	accessory	1322	[RbromiiL2-36]RBL236_00898_Putative_aminopeptidase_YsdC	RbromiiL2-36
Org4_Gene853	accessory	2123	[RbromiiL2-36]RBL236_00899_ribosomal-protein-alanine_acetyltransferase	RbromiiL2-36
Org4_Gene856	accessory	1309	[RbromiiL2-36]RBL236_00902_CAAX_amino_terminal_protease_self-_immunity	RbromiiL2-36
Org4_Gene857	accessory	1047	[RbromiiL2-36]RBL236_00904_Tyrosine_recombinase_XerC	RbromiiL2-36
Org4_Gene858	accessory	4141	[RbromiiL2-36]RBL236_00905_DNA_binding_domain,_excisionase_family	RbromiiL2-36
Org4_Gene859	accessory	2980	[RbromiiL2-36]RBL236_00906_mRNA_interferase_EndoA	RbromiiL2-36
Org4_Gene86	accessory	3912	[RbromiiL2-36]RBL236_00087_hypothetical_protein	RbromiiL2-36
Org4_Gene860	accessory	3188	[RbromiiL2-36]RBL236_00907_RNA_polymerase_sigma_factor	RbromiiL2-36
Org4_Gene861	accessory	3714	[RbromiiL2-36]RBL236_00908_anaerobic_benzoate_catabolism_transcriptional_regulator	RbromiiL2-36
Org4_Gene862	accessory	230	[RbromiiL2-36]RBL236_00909_Serine/alanine_racemase	RbromiiL2-36
Org4_Gene863	accessory	2108	[RbromiiL2-36]RBL236_00910_D-alanyl-D-alanine_carboxypeptidase	RbromiiL2-36
Org4_Gene864	accessory	1319	[RbromiiL2-36]RBL236_00911_Vancomycin_C-type_resistance_protein_VanC	RbromiiL2-36
Org4_Gene865	accessory	1960	[RbromiiL2-36]RBL236_00912_Vancomycin_B-type_resistance_protein_VanW	RbromiiL2-36
Org4_Gene866	accessory	2926	[RbromiiL2-36]RBL236_00913_D-alanyl-D-alanine_carboxypeptidase	RbromiiL2-36
Org4_Gene867	accessory	1176	[RbromiiL2-36]RBL236_00914_Alkaline_phosphatase_synthesis_sensor_protein_PhoR	RbromiiL2-36
Org4_Gene868	accessory	2347	[RbromiiL2-36]RBL236_00915_Transcriptional_regulatory_protein_WalR	RbromiiL2-36
Org4_Gene869	accessory	4159	[RbromiiL2-36]RBL236_00916_putative_transcriptional_regulator	RbromiiL2-36
Org4_Gene87	accessory	225	[RbromiiL2-36]RBL236_00088_hypothetical_protein	RbromiiL2-36
Org4_Gene870	accessory	401	[RbromiiL2-36]RBL236_00917_hypothetical_protein	RbromiiL2-36
Org4_Gene871	accessory	2051	[RbromiiL2-36]RBL236_00918_sortase,_SrtB_family	RbromiiL2-36
Org4_Gene872	accessory	177	[RbromiiL2-36]RBL236_00919_conjugal_transfer_ATP-binding_protein_TraC	RbromiiL2-36
Org4_Gene873	accessory	3614	[RbromiiL2-36]RBL236_00920_PrgI_family_protein	RbromiiL2-36
Org4_Gene874	accessory	1892	[RbromiiL2-36]RBL236_00921_hypothetical_protein	RbromiiL2-36
Org4_Gene875	accessory	1907	[RbromiiL2-36]RBL236_00922_hypothetical_protein	RbromiiL2-36
Org4_Gene876	accessory	2784	[RbromiiL2-36]RBL236_00923_Transcriptional_activator,_adenine-specific_DNA_methyltransferase	RbromiiL2-36
Org4_Gene877	accessory	4158	[RbromiiL2-36]RBL236_00924_hypothetical_protein	RbromiiL2-36
Org4_Gene88	accessory	1526	[RbromiiL2-36]RBL236_00089_DNA_adenine_methyltransferase_YhdJ	RbromiiL2-36
Org4_Gene880	accessory	4255	[RbromiiL2-36]RBL236_00927_hypothetical_protein	RbromiiL2-36
Org4_Gene881	accessory	1698	[RbromiiL2-36]RBL236_00928_hypothetical_protein	RbromiiL2-36
Org4_Gene882	accessory	1961	[RbromiiL2-36]RBL236_00929_hypothetical_protein	RbromiiL2-36
Org4_Gene883	accessory	664	[RbromiiL2-36]RBL236_00930_Relaxase/Mobilisation_nuclease_domain_protein	RbromiiL2-36
Org4_Gene884	accessory	3722	[RbromiiL2-36]RBL236_00931_Bacterial_mobilisation_protein_(MobC)	RbromiiL2-36
Org4_Gene885	accessory	601	[RbromiiL2-36]RBL236_00932_Modification_methylase_HhaI	RbromiiL2-36
Org4_Gene886	accessory	4302	[RbromiiL2-36]RBL236_00933_hypothetical_protein	RbromiiL2-36
Org4_Gene887	accessory	3443	[RbromiiL2-36]RBL236_00934_hypothetical_protein	RbromiiL2-36

Org4_Gene888	accessory	3729	[RbromiiL2-36]RBL236_00935_hypothetical_protein	RbromiiL2-36
Org4_Gene889	accessory	1499	[RbromiiL2-36]RBL236_00936_DNA_primase_(bacterial_type)	RbromiiL2-36
Org4_Gene89	accessory	146	[RbromiiL2-36]RBL236_00090_type-IV_secretion_system_protein_TraC	RbromiiL2-36
Org4_Gene890	accessory	2316	[RbromiiL2-36]RBL236_00937_hypothetical_protein	RbromiiL2-36
Org4_Gene891	accessory	1331	[RbromiiL2-36]RBL236_00938_Fic/DOC_family_protein	RbromiiL2-36
Org4_Gene892	accessory	3758	[RbromiiL2-36]RBL236_00939_hypothetical_protein	RbromiiL2-36
Org4_Gene893	accessory	3208	[RbromiiL2-36]RBL236_00940_hypothetical_protein	RbromiiL2-36
Org4_Gene894	accessory	3628	[RbromiiL2-36]RBL236_00941_anaerobic_benzoate_catabolism_transcriptional_regulator	RbromiiL2-36
Org4_Gene895	accessory	3211	[RbromiiL2-36]RBL236_00942_Transposase	RbromiiL2-36
Org4_Gene896	accessory	2759	[RbromiiL2-36]RBL236_00943_hypothetical_protein	RbromiiL2-36
Org4_Gene897	accessory	2170	[RbromiiL2-36]RBL236_00944_hypothetical_protein	RbromiiL2-36
Org4_Gene898	accessory	2809	[RbromiiL2-36]RBL236_00945_hypothetical_protein	RbromiiL2-36
Org4_Gene899	accessory	68	[RbromiiL2-36]RBL236_00946_Type-1_restriction_enzyme_R_protein	RbromiiL2-36
Org4_Gene90	accessory	3451	[RbromiiL2-36]RBL236_00091_PrgI_family_protein	RbromiiL2-36
Org4_Gene900	accessory	895	[RbromiiL2-36]RBL236_00947_EcoKI_restriction-modification_system_protein_HsdS	RbromiiL2-36
Org4_Gene901	accessory	515	[RbromiiL2-36]RBL236_00948_putative_type_I_restriction_enzymeP_M_protein	RbromiiL2-36
Org4_Gene902	accessory	1596	[RbromiiL2-36]RBL236_00949_Integrase	RbromiiL2-36
Org4_Gene903	accessory	2663	[RbromiiL2-36]RBL236_00950_Type_I_restriction_modification_DNA_specificity_domain_protein	RbromiiL2-36
Org4_Gene904	accessory	3032	[RbromiiL2-36]RBL236_00951_EcoKI_restriction-modification_system_protein_HsdS	RbromiiL2-36
Org4_Gene905	accessory	2641	[RbromiiL2-36]RBL236_00952_hypothetical_protein	RbromiiL2-36
Org4_Gene906	accessory	3731	[RbromiiL2-36]RBL236_00953_hypothetical_protein	RbromiiL2-36
Org4_Gene907	accessory	1488	[RbromiiL2-36]RBL236_00954_Putative_viral_replication_protein	RbromiiL2-36
Org4_Gene908	accessory	851	[RbromiiL2-36]RBL236_00955_DNA_translocase_FtsK	RbromiiL2-36
Org4_Gene909	accessory	4172	[RbromiiL2-36]RBL236_00956_DNA_binding_domain,_excisionase_family	RbromiiL2-36
Org4_Gene91	accessory	1803	[RbromiiL2-36]RBL236_00092_hypothetical_protein	RbromiiL2-36
Org4_Gene910	accessory	1081	[RbromiiL2-36]RBL236_00957_Integrase	RbromiiL2-36
Org4_Gene911	accessory	4198	[RbromiiL2-36]RBL236_00959_Putative_PBSX_repressor	RbromiiL2-36
Org4_Gene912	accessory	2492	[RbromiiL2-36]RBL236_00960_hypothetical_protein	RbromiiL2-36
Org4_Gene913	accessory	863	[RbromiiL2-36]RBL236_00961_KWG_Leptospira	RbromiiL2-36
Org4_Gene914	accessory	2178	[RbromiiL2-36]RBL236_00962_hypothetical_protein	RbromiiL2-36
Org4_Gene915	accessory	4355	[RbromiiL2-36]RBL236_00963_hypothetical_protein	RbromiiL2-36
Org4_Gene917	accessory	3750	[RbromiiL2-36]RBL236_00965_HTH-type_transcriptional_regulator_ImmR	RbromiiL2-36
Org4_Gene918	accessory	254	[RbromiiL2-36]RBL236_00966_Kappa-carrageenase_precursor	RbromiiL2-36
Org4_Gene92	accessory	451	[RbromiiL2-36]RBL236_00093_hypothetical_protein	RbromiiL2-36
Org4_Gene920	accessory	2455	[RbromiiL2-36]RBL236_00968_Thymidylate_kinase	RbromiiL2-36
Org4_Gene921	accessory	1737	[RbromiiL2-36]RBL236_00969_hypothetical_protein	RbromiiL2-36
Org4_Gene922	accessory	2072	[RbromiiL2-36]RBL236_00970_putative_acetyltransferase_involved_in_intracellular_survival	RbromiiL2-36
Org4_Gene924	accessory	2684	[RbromiiL2-36]RBL236_00972_5-formyltetrahydrofolate_cyclo-ligase_family_protein	RbromiiL2-36
Org4_Gene925	accessory	749	[RbromiiL2-36]RBL236_00973_putative_aminodeoxychorismate_lyase	RbromiiL2-36
Org4_Gene928	accessory	1159	[RbromiiL2-36]RBL236_00977_hypothetical_protein	RbromiiL2-36
Org4_Gene929	accessory	1932	[RbromiiL2-36]RBL236_00979_putative_endonuclease_4	RbromiiL2-36
Org4_Gene93	accessory	4233	[RbromiiL2-36]RBL236_00094_hypothetical_protein	RbromiiL2-36
Org4_Gene930	accessory	3720	[RbromiiL2-36]RBL236_00980_Thioredoxin	RbromiiL2-36
Org4_Gene931	accessory	1726	[RbromiiL2-36]RBL236_00981_Thioredoxin_reductase	RbromiiL2-36
Org4_Gene932	accessory	1625	[RbromiiL2-36]RBL236_00982_putative_amino-acid_metabolite_efflux_pump	RbromiiL2-36
Org4_Gene934	accessory	653	[RbromiiL2-36]RBL236_00984_Amino-acid_carrier_protein_AlsT	RbromiiL2-36
Org4_Gene935	accessory	2709	[RbromiiL2-36]RBL236_00985_hypothetical_protein	RbromiiL2-36
Org4_Gene938	accessory	1710	[RbromiiL2-36]RBL236_00988_hypothetical_protein	RbromiiL2-36
Org4_Gene939	accessory	1072	[RbromiiL2-36]RBL236_00989_Ferrous-iron_efflux_pump_FieF	RbromiiL2-36
Org4_Gene94	accessory	800	[RbromiiL2-36]RBL236_00095_putative_P-loop_ATPase	RbromiiL2-36
Org4_Gene946	accessory	4300	[RbromiiL2-36]RBL236_00996_hypothetical_protein	RbromiiL2-36
Org4_Gene947	accessory	3516	[RbromiiL2-36]RBL236_00997_hypothetical_protein	RbromiiL2-36
Org4_Gene948	accessory	3762	[RbromiiL2-36]RBL236_00998_hypothetical_protein	RbromiiL2-36
Org4_Gene949	accessory	3577	[RbromiiL2-36]RBL236_00999_hypothetical_protein	RbromiiL2-36
Org4_Gene95	accessory	2559	[RbromiiL2-36]RBL236_00096_DNA_primase	RbromiiL2-36
Org4_Gene950	accessory	4291	[RbromiiL2-36]RBL236_01000_hypothetical_protein	RbromiiL2-36
Org4_Gene954	accessory	1447	[RbromiiL2-36]RBL236_01004_L-Ala--D-Glu_endopeptidase_precursor	RbromiiL2-36
Org4_Gene955	accessory	3376	[RbromiiL2-36]RBL236_01005_hypothetical_protein	RbromiiL2-36
Org4_Gene957	accessory	1121	[RbromiiL2-36]RBL236_01007_hypothetical_protein	RbromiiL2-36
Org4_Gene958	accessory	3012	[RbromiiL2-36]RBL236_01008_Cob(I)yrinic_acid_a,c-diamide_adenosyltransferase	RbromiiL2-36
Org4_Gene96	accessory	523	[RbromiiL2-36]RBL236_00097_DNA_strand_transferase	RbromiiL2-36
Org4_Gene97	accessory	4129	[RbromiiL2-36]RBL236_00098_hypothetical_protein	RbromiiL2-36
Org4_Gene98	accessory	3846	[RbromiiL2-36]RBL236_00099_hypothetical_protein	RbromiiL2-36
Org4_Gene985	accessory	4431	[RbromiiL2-36]RBL236_01035_Ribosomal_protein_II	RbromiiL2-36
Org4_Gene99	accessory	3665	[RbromiiL2-36]RBL236_00100_hypothetical_protein	RbromiiL2-36
Org4_Gene991	accessory	1513	[RbromiiL2-36]RBL236_01041_hypothetical_protein	RbromiiL2-36
Org4_Gene992	accessory	281	[RbromiiL2-36]RBL236_01042_CotH_protein	RbromiiL2-36
Org4_Gene994	accessory	3031	[RbromiiL2-36]RBL236_01044_Shikimate_kinase_2	RbromiiL2-36
Org4_Gene998	accessory	845	[RbromiiL2-36]RBL236_01048_Folylpolyglutamate_synthase	RbromiiL2-36
Org5_Gene1	accessory	2338	[RbromiL2-63]L2-63_00001_L2-63_00001_hypothetical_protein_1035:1745_forward_MW:26274	RbromiL2-63
Org5_Gene100	accessory	4195	[RbromiL2-63]L2-63_00103_L2-63_00103_hypothetical_protein_110254:110460_reverse_MW:7210	RbromiL2-63
Org5_Gene1000	accessory	1420	[RbromiL2-63]L2-63_01030_femX_Lipid_II:glycine_glycyltransferase,Uncharacterized_protein_involved_in_methicillin_resistance,FemAB-related_protein,_	RbromiL2-63
Org5_Gene1001	accessory	2117	[RbromiL2-63]L2-63_01031_L2-63_01031_hypothetical_protein_1089080:1089856_reverse_MW:29076	RbromiL2-63
Org5_Gene1002	accessory	646	[RbromiL2-63]L2-63_01032_arlS_1_Signal_transduction_histidine-protein_kinase_ArlS,sensory_histidine_kinase_CreC,Predicted_periplasmic_ligand-bindin	RbromiL2-63
Org5_Gene1003	accessory	2534	[RbromiL2-63]L2-63_01033_arlR_Response_regulator_ArlR,DNA-binding_response_regulator_CreB,Response_regulator_of_citrate/malate_metabolism,ph	RbromiL2-63
Org5_Gene1004	accessory	1699	[RbromiL2-63]L2-63_01034_L2-63_01034_hypothetical_protein_1092175:1093074_forward_MW:31723	RbromiL2-63
Org5_Gene1006	accessory	1019	[RbromiL2-63]L2-63_01036_Int-Tn_4_Integrase,site-specific_tyrosine_recombinase_XerC,Site-specific_recombinase_XerD,tyrosine_recombinase_XerC,Pha	RbromiL2-63
Org5_Gene1007	accessory	4328	[RbromiL2-63]L2-63_01037_L2-63_01037_hypothetical_protein_1095758:1095934_reverse_MW:6573	RbromiL2-63
Org5_Gene1008	accessory	3345	[RbromiL2-63]L2-63_01038_L2-63_01038_hypothetical_protein_1095931:1096359_reverse_MW:16652	RbromiL2-63
Org5_Gene1009	accessory	3140	[RbromiL2-63]L2-63_01039_L2-63_01039_hypothetical_protein_1096356:1096835_reverse_MW:18372	RbromiL2-63
Org5_Gene101	accessory	2346	[RbromiL2-63]L2-63_00104_L2-63_00104_hypothetical_protein_110475:111182_reverse_MW:27720	RbromiL2-63
Org5_Gene1010	accessory	860	[RbromiL2-63]L2-63_01040_L2-63_01040_Replication_initiation_factor_1096987:1098282_reverse_MW:50372	RbromiL2-63
Org5_Gene1011	accessory	3715	[RbromiL2-63]L2-63_01041_L2-63_01041_hypothetical_protein_1098415:1098747_reverse_MW:12383	RbromiL2-63
Org5_Gene1012	accessory	2292	[RbromiL2-63]L2-63_01042_L2-63_01042_DNA_segregation_ATPase_FtsK/SpoIIIE_and_related_proteins,type_VII_secretion_protein_EccCa,FtsK/SpoIIIE_fa	RbromiL2-63
Org5_Gene1013	accessory	2217	[RbromiL2-63]L2-63_01043_L2-63_01043_hypothetical_protein_1099485:1100228_reverse_MW:28402	RbromiL2-63
Org5_Gene1014	accessory	3421	[RbromiL2-63]L2-63_01044_L2-63_01044_hypothetical_protein_1100320:1100730_reverse_MW:15451	RbromiL2-63
Org5_Gene1015	accessory	1740	[RbromiL2-63]L2-63_01045_L2-63_01045_hypothetical_protein_1101152:1102039_forward_MW:34445	RbromiL2-63
Org5_Gene1016	accessory	3292	[RbromiL2-63]L2-63_01046_L2-63_01046_hypothetical_protein_1102093:1102536_forward_MW:17434	RbromiL2-63
Org5_Gene1017	accessory	3108	[RbromiL2-63]L2-63_01047_L2-63_01047_DnaJ_domain_1102959:1103447_forward_MW:19377	RbromiL2-63
Org5_Gene1018	accessory	2397	[RbromiL2-63]L2-63_01048_L2-63_01048_hypothetical_protein_1104525:1105220_forward_MW:25920	RbromiL2-63
Org5_Gene1019	accessory	4107	[RbromiL2-63]L2-63_01049_L2-63_01049_hypothetical_protein_1105770:1105997_forward_MW:8595	RbromiL2-63
Org5_Gene102	accessory	3934	[RbromiL2-63]L2-63_00105_L2-63_00105_hypothetical_protein_111311:111583_reverse_MW:10650	RbromiL2-63
Org5_Gene1020	accessory	4038	[RbromiL2-63]L2-63_01050_L2-63_01050_hypothetical_protein_1105997:1106242_forward_MW:9341	RbromiL2-63
Org5_Gene1021	accessory	2416	[RbromiL2-63]L2-63_01051_L2-63_01051_hypothetical_protein_1106385:1107074_reverse_MW:26784	RbromiL2-63
Org5_Gene1022	accessory	589	[RbromiL2-63]L2-63_01052_L2-63_01052_Predicted_ATPase_1107074:1108561_reverse_MW:57073	RbromiL2-63
Org5_Gene1023	accessory	4308	[RbromiL2-63]L2-63_01054_L2-63_01054_hypothetical_protein_1109148:1109330_reverse_MW:6863	RbromiL2-63
Org5_Gene1024	accessory	2772	[RbromiL2-63]L2-63_01055_xpt_Xanthine_phosphoribosyltransferase,xanthine_phosphoribosyltransferase,xanthine_phosphoribosyltransferase,Phosphoril	RbromiL2-63
Org5_Gene1025	accessory	530	[RbromiL2-63]L2-63_01056_putP_Proline_permease,proline:sodium_symporter_PutP,Na_/panthothenate_symporter,sodium/proline_symporter,Sodium:s	RbromiL2-63
Org5_Gene1028	accessory	3313	[RbromiL2-63]L2-63_01059_ywlE_Low_molecular_weight_protein-tyrosine-phosphatase_ywlE,arsenate_reductase,Protein-tyrosine-phosphatase,arsenate_	RbromiL2-63
Org5_Gene1029	accessory	3318	[RbromiL2-63]L2-63_01060_ypeA_2_Acetyltransferase_YpeA,ribosomal-protein-alanine_N-acetyltransferase,Predicted_acetyltransferase_involved_in_intra	RbromiL2-63
Org5_Gene103	accessory	3833	[RbromiL2-63]L2-63_00106_L2-63_00106_hypothetical_protein_111594:111896_reverse_MW:11503	RbromiL2-63
Org5_Gene1033	accessory	846	[RbromiL2-63]L2-63_01064_L2-63_01064_Ribonucleases_G_and_E_1121168:1122199_forward_MW:35772	RbromiL2-63
Org5_Gene1034	accessory	1778	[RbromiL2-63]L2-63_01065_L2-63_01065_hypothetical_protein_1122467:1123342_forward_MW:31845	RbromiL2-63
Org5_Gene1035	accessory	1268	[RbromiL2-63]L2-63_01066_L2-63_01066_hypothetical_protein_1123525:1124592_forward_MW:38577	RbromiL2-63
Org5_Gene1036	accessory	512	[RbromiL2-63]L2-63_01067_L2-63_01067_hypothetical_protein_1124887:1126491_forward_MW:57811	RbromiL2-63
Org5_Gene1038	accessory	2385	[RbromiL2-63]L2-63_01069_L2-63_01069_Zn-dependent_proteases,Peptidase_family_M50_1127025:1127723_forward_MW:25729	RbromiL2-63
Org5_Gene104	accessory	4434	[RbromiL2-63]L2-63_00107_L2-63_00107_hypothetical_protein_111946:112038_reverse_MW:3367	RbromiL2-63
Org5_Gene1041	accessory	2625	[RbromiL2-63]L2-63_01072_L2-63_01072_Protein_of_unknown_function_(DUF2953)_1129175:1129795_forward_MW:23165	RbromiL2-63
Org5_Gene1043	accessory	1186	[RbromiL2-63]L2-63_01074_dacB_1_D-alanyl-D-alanine_carboxypeptidase_dacB_precursor,D-alanyl-D-alanine_carboxypeptidase_fraction_C,D-alanyl-D-ala	RbromiL2-63
Org5_Gene1048	accessory	774	[RbromiL2-63]L2-63_01079_L2-63_01079_hypothetical_protein_1135790:1137136_forward_MW:49458	RbromiL2-63
Org5_Gene1049	accessory	162	[RbromiL2-63]L2-63_01080_L2-63_01080_Fibronectin_type_III_domain_1137333:1139474_forward_MW:80197	RbromiL2-63
Org5_Gene105	accessory	27	[RbromiL2-63]L2-63_00108_L2-63_00108_Predicted_outer_membrane_protein,Cna_protein_B-type_domain_112042:116184_reverse_MW:149624	RbromiL2-63
Org5_Gene1050	accessory	351	[RbromiL2-63]L2-63_01081_L2-63_01081_Fibronectin_type_III_domain_1139909:1141747_forward_MW:68621	RbromiL2-63
Org5_Gene1054	accessory	947	[RbromiL2-63]L2-63_01085_L2-63_01085_hypothetical_protein_1145474:1146718_reverse_MW:47505	RbromiL2-63
Org5_Gene1055	accessory	185	[RbromiL2-63]L2-63_01086_cadA_Cadmium,_zinc_and_cobalt-transporting_ATPase,zinc/cadmium/mercury/lead-transporting_ATPase,Uncharacterized_pr	RbromiL2-63
Org5_Gene106	accessory	1560	[RbromiL2-63]L2-63_00109_parB_1_Chromosome-partitioning_protein_parB,plasmid_partitioning_protein_RepB,ParB/RepB/Spo0J_family_partition_prote	RbromiL2-63
Org5_Gene1062	accessory	4154	[RbromiL2-63]L2-63_01093_L2-63_01093_hypothetical_protein_1156044:1156259_reverse_MW:8130	RbromiL2-63
Org5_Gene1063	accessory	41	[RbromiL2-63]L2-63_01094_pulA_1_Pullulanase_precursor,putative_bifunctional_4-alpha-glucanotransferase/glycogen_debranching_enzyme,pullulanase,_	RbromiL2-63
Org5_Gene1066	accessory	3059	[RbromiL2-63]L2-63_01097_L2-63_01097_hypothetical_protein_1163024:1163524_forward_MW:17775	RbromiL2-63
Org5_Gene1067	accessory	2006	[RbromiL2-63]L2-63_01098_L2-63_01098_Sortase_(surface_protein_transpeptidase),sortase,_SrtB_family,Sortase_family_1163717:1164526_forward_MW	RbromiL2-63
Org5_Gene1068	accessory	816	[RbromiL2-63]L2-63_01099_yvhJ_1_Putative_transcriptional_regulator_yvhJ,membrane-bound_transcriptional_regulator_LytR,cell_envelope-related_func	RbromiL2-63
Org5_Gene107	accessory	1947	[RbromiL2-63]L2-63_00110_soj_1_Sporulation_initiation_inhibitor_protein_soj,plasmid-partitioning_protein_RepA,Septum_formation_inhibitor-activating	RbromiL2-63

Org5_Gene1071	accessory	143	[RbromiL2-63]L2-63_01102_L2-63_01102_Predicted_membrane_protein,Bacterial_membrane_protein_YfhO_1168388:1170847_forward_MW:93376	RbromiL2-63
Org5_Gene1073	accessory	2081	[RbromiL2-63]L2-63_01104_L2-63_01104_Protein_of_unknown_function_(DUF975)_1172291:1173079_forward_MW:29372	RbromiL2-63
Org5_Gene1076	accessory	2274	[RbromiL2-63]L2-63_01107_L2-63_01107_DNA_alkylation_repair_enzyme_1175088:1175813_forward_MW:28639	RbromiL2-63
Org5_Gene1077	accessory	2502	[RbromiL2-63]L2-63_01109_ribU_Riboflavin_ECF_transporter_S_component_RibU,Protein_of_unknown_function_(DUF3816)_1176076:1176738_forward_	RbromiL2-63
Org5_Gene1078	accessory	2798	[RbromiL2-63]L2-63_01111_folT_Folate_ECF_transporter_S_component_FolT,hypothetical_protein,Predicted_membrane_protein,Protein_of_unknown_fu	RbromiL2-63
Org5_Gene108	accessory	4318	[RbromiL2-63]L2-63_00111_L2-63_00111_hypothetical_protein_118228:118407_reverse_MW:7173	RbromiL2-63
Org5_Gene1082	accessory	311	[RbromiL2-63]L2-63_01115_glgB_2_1,4-alpha-glucan_branching_enzyme_GlgB,glycogen_branching_enzyme,1,4-alpha-glucan_branching_enzyme,Carbohy	RbromiL2-63
Org5_Gene1087	accessory	3284	[RbromiL2-63]L2-63_01120_L2-63_01120_hypothetical_protein,acyl-CoA_thioesterase_YbgC,acyl-CoA_thioester_hydrolase,_YbgC/YbaW_family,Thioestera	RbromiL2-63
Org5_Gene1090	accessory	1661	[RbromiL2-63]L2-63_01123_gltC_HTH-type_transcriptional_regulator_gltC,DNA-binding_transcriptional_regulator_HcaR,aminoethylphosphonate_catabolis	RbromiL2-63
Org5_Gene1092	accessory	2392	[RbromiL2-63]L2-63_01125_L2-63_01125_Predicted_flavoprotein,NADPH-dependent_FMN_reductase_1195452:1196147_forward_MW:26289	RbromiL2-63
Org5_Gene1093	accessory	879	[RbromiL2-63]L2-63_01126_glgE1_Alpha-1,4-glucan:maltose-1-phosphate_maltosyltransferase_1,putative_bifunctional_4-alpha-glucanotransferase/glycog	RbromiL2-63
Org5_Gene1094	accessory	2825	[RbromiL2-63]L2-63_01127_ydaF_Putative_ribosomal_N-acetyltransferase_YdaF,spermidine_N1-acetyltransferase,Acetyltransferase_(GNAT)_family_1197	RbromiL2-63
Org5_Gene1095	accessory	3591	[RbromiL2-63]L2-63_01128_L2-63_01128_hypothetical_protein_1198246:1198608_reverse_MW:14297	RbromiL2-63
Org5_Gene1097	accessory	634	[RbromiL2-63]L2-63_01130_mepA_5_Staphylococcal_virulence_regulator_protein_A,multidrug_efflux_protein,MATE_efflux_family_protein,MatE_120012	RbromiL2-63
Org5_Gene1098	accessory	3490	[RbromiL2-63]L2-63_01131_L2-63_01131_hypothetical_protein_1202018:1202410_forward_MW:14492	RbromiL2-63
Org5_Gene1099	accessory	1481	[RbromiL2-63]L2-63_01132_afr_1,5-anhydro-D-fructose_reductase,putative_oxidoreductase,Uncharacterized_protein_conserved_in_bacteria,Oxidoreduct	RbromiL2-63
Org5_Gene11	accessory	242	[RbromiL2-63]L2-63_00011_ltaS1_Lipoteichoic_acid_synthase_1,arylsulfatase,Phosphoglycerol_transferase_and_related_proteins,_alkaline_phosphatase_	RbromiL2-63
Org5_Gene1100	accessory	1071	[RbromiL2-63]L2-63_01133_rbr1_NADH_peroxidase,Uncharacterized_conserved_protein,Rubrerythrin_1203535:1204080_reverse_MW:20797	RbromiL2-63
Org5_Gene1101	accessory	1342	[RbromiL2-63]L2-63_01134_sbp_Sulfate_starvation-induced_protein_2,sulfate_transporter_subunit,ABC-type_sulfate_transport_system,_periplasmic_com	RbromiL2-63
Org5_Gene1102	accessory	1867	[RbromiL2-63]L2-63_01135_cysW_1_Sulfate_transport_system_permease_protein_CysW,sulfate/thiosulfate_transporter_subunit,ABC-type_molybdate_tr	RbromiL2-63
Org5_Gene1103	accessory	1971	[RbromiL2-63]L2-63_01136_cysW_2_Sulfate_transport_system_permease_protein_CysW,sulfate/thiosulfate_transporter_permease_subunit,ABC-type_sul	RbromiL2-63
Org5_Gene1104	accessory	1303	[RbromiL2-63]L2-63_01137_cysA_Sulfate/thiosulfate_import_ATP-binding_protein_CysA,putrescine/spermidine_ABC_transporter_ATPase_protein,ABC-ty	RbromiL2-63
Org5_Gene1105	accessory	3037	[RbromiL2-63]L2-63_01138_L2-63_01138_Uncharacterized_protein_conserved_in_bacteria,Zeta_toxin_1208558:1209064_reverse_MW:19460	RbromiL2-63
Org5_Gene1107	accessory	1165	[RbromiL2-63]L2-63_01140_hemA_Glutamyl-tRNA_reductase,glutamyl-tRNA_reductase,Glutamyl-tRNA_reductase,glutamyl-tRNA_reductase,Glutamyl-tRN	RbromiL2-63
Org5_Gene1108	accessory	2659	[RbromiL2-63]L2-63_01141_cysG_1_Siroheme_synthase,precorrin-2_dehydrogenase,Uroporphyrinogen-III_methylase,siroheme_synthase,_N-terminal_dor	RbromiL2-63
Org5_Gene1109	accessory	1836	[RbromiL2-63]L2-63_01142_hemC_Porphobilinogen_deaminase,porphobilinogen_deaminase,Uroporphyrinogen-III_synthase,porphobilinogen_deaminase,	RbromiL2-63
Org5_Gene1110	accessory	591	[RbromiL2-63]L2-63_01143_cysG_2_Siroheme_synthase,uroporphyrin-III_C-methyltransferase,Uroporphyrinogen-III_methylase,uroporphyrinogen-III_C-me	RbromiL2-63
Org5_Gene1111	accessory	1492	[RbromiL2-63]L2-63_01144_hemB_Delta-aminolevulinic_acid_dehydratase,delta-aminolevulinic_acid_dehydratase,Delta-aminolevulinic_acid_dehydratase	RbromiL2-63
Org5_Gene1112	accessory	920	[RbromiL2-63]L2-63_01145_hemL2_Glutamate-1-semialdehyde_2,1-aminomutase_2,glutamate-1-semialdehyde_aminotransferase,Glutamate-1-semialdeh	RbromiL2-63
Org5_Gene1113	accessory	3248	[RbromiL2-63]L2-63_01146_iscR_1_HTH-type_transcriptional_regulator_iscR,DNA-binding_transcriptional_regulator_IscR,Rrf2_family_protein,Transcriptio	RbromiL2-63
Org5_Gene1114	accessory	940	[RbromiL2-63]L2-63_01147_mdeA_2_Methionine_gamma-lyase,O-acetylhomoserine_aminocarboxypropyltransferase,Protoheme_ferro-lyase_(ferrochelat	RbromiL2-63
Org5_Gene1115	accessory	1835	[RbromiL2-63]L2-63_01148_dsvB_Sulfite_reductase,_dissimilatory-type_subunit_beta,ferredoxin-nitrite_reductase,Sulfite_reductase,_beta_subunit_(hem	RbromiL2-63
Org5_Gene1116	accessory	4035	[RbromiL2-63]L2-63_01149_L2-63_01149_hypothetical_protein,hypothetical_protein,SirA-like_protein_1219382:1219627_forward_MW:8974	RbromiL2-63
Org5_Gene1117	accessory	3759	[RbromiL2-63]L2-63_01150_trxA_3_Thioredoxin-M,thioredoxin,Thioredoxin-related_protein,thioredoxin,Thioredoxin_1219628:1219951_forward_MW:122	RbromiL2-63
Org5_Gene1118	accessory	4189	[RbromiL2-63]L2-63_01151_L2-63_01151_bifunctional_sulfur_carrier_protein/thiazole_synthase_protein,thiamine_biosynthesis_protein_ThiS,ThiS_family_	RbromiL2-63
Org5_Gene1119	accessory	2001	[RbromiL2-63]L2-63_01152_moeZ_Probable_adenylyltransferase/sulfurtransferase_MoeZ,molybdopterin_biosynthesis_protein_MoeB,thiazole_biosynthes	RbromiL2-63
Org5_Gene1120	accessory	3425	[RbromiL2-63]L2-63_01153_mec_CysO-cysteine_peptidase,Mov34/MPN/PAD-1_family_1220972:1221382_forward_MW:15395	RbromiL2-63
Org5_Gene1121	accessory	1657	[RbromiL2-63]L2-63_01154_trxB_2_Thioredoxin_reductase,alkyl_hydroperoxide_reductase_subunit_F,Alkyl_hydroperoxide_reductase,_large_subunit,thio	RbromiL2-63
Org5_Gene1122	accessory	442	[RbromiL2-63]L2-63_01155_ifcA_Fumarate_reductase_flavoprotein_subunit_precursor,adenylylsulfate_reductase_subunit_alpha,Aspartate_oxidase,adeny	RbromiL2-63
Org5_Gene1123	accessory	3783	[RbromiL2-63]L2-63_01156_L2-63_01156_Ferredoxin_II,NADH-plastoquinone_oxidoreductase_subunit,Uncharacterized_Fe-S_center_protein,adenylylsulfa	RbromiL2-63
Org5_Gene1124	accessory	1700	[RbromiL2-63]L2-63_01157_cysD_Sulfate_adenylyltransferase_subunit_2,sulfate_adenylyltransferase_subunit_2,PUA_domain_(predicted_RNA-binding_do	RbromiL2-63
Org5_Gene1125	accessory	439	[RbromiL2-63]L2-63_01158_cysN_Sulfate_adenylyltransferase_subunit_1,bifunctional_sulfate_adenylyltransferase_subunit_1/adenylylsulfate_kinase_prot	RbromiL2-63
Org5_Gene1126	accessory	4217	[RbromiL2-63]L2-63_01159_L2-63_01159_TM2_domain_1227106:1227306_forward_MW:7151	RbromiL2-63
Org5_Gene1127	accessory	3515	[RbromiL2-63]L2-63_01160_L2-63_01160_hypothetical_protein_1227324:1227707_forward_MW:13763	RbromiL2-63
Org5_Gene1128	accessory	2955	[RbromiL2-63]L2-63_01161_ogt_Methylated-DNA--protein-cysteine_methyltransferase,_constitutive,methylated-DNA--protein-cysteine_methyltransferase	RbromiL2-63
Org5_Gene1129	accessory	893	[RbromiL2-63]L2-63_01162_L2-63_01162_hypothetical_protein_1228313:1229590_reverse_MW:48314	RbromiL2-63
Org5_Gene1130	accessory	2352	[RbromiL2-63]L2-63_01163_lytR_3_Sensory_transduction_protein_lytR,two-component_response_regulator,Response_regulator_of_the_LytR/AlgR_famil	RbromiL2-63
Org5_Gene1131	accessory	3261	[RbromiL2-63]L2-63_01164_L2-63_01164_hypothetical_protein_1230797:1231246_forward_MW:16919	RbromiL2-63
Org5_Gene1132	accessory	2901	[RbromiL2-63]L2-63_01165_L2-63_01165_VanZ_like_family_1231250:1231792_forward_MW:20394	RbromiL2-63
Org5_Gene1133	accessory	6	[RbromiL2-63]L2-63_01166_grsB_Gramicidin_S_synthase_II,peptide_synthase,Acyl_carrier_protein,amino_acid_adenylation_domain,AMP-binding_enzyme	RbromiL2-63
Org5_Gene1134	accessory	389	[RbromiL2-63]L2-63_01167_mepA_6_Multidrug_export_protein_mepA,multidrug_efflux_pump_VmrA,MATE_efflux_family_protein,MatE_1239496:124125	RbromiL2-63
Org5_Gene1135	accessory	3806	[RbromiL2-63]L2-63_01168_spoIIAA_2_Stage_II_sporulation_protein_AA,Anti-anti-sigma_regulatory_factor_(antagonist_of_anti-sigma_factor),anti-anti-si	RbromiL2-63
Org5_Gene1136	accessory	3468	[RbromiL2-63]L2-63_01169_btrW_Serine/threonine-protein_kinase_BtrW,serine-protein_kinase_RsbW_1241742:1242140_reverse_MW:14766	RbromiL2-63
Org5_Gene1137	accessory	1952	[RbromiL2-63]L2-63_01170_L2-63_01170_hypothetical_protein_1242384:1243208_reverse_MW:32246	RbromiL2-63
Org5_Gene1138	accessory	3745	[RbromiL2-63]L2-63_01171_L2-63_01171_hypothetical_protein_1243217:1243543_reverse_MW:11998	RbromiL2-63
Org5_Gene1139	accessory	2215	[RbromiL2-63]L2-63_01172_ygaZ_Inner_membrane_protein_YgaZ,azaleucine_resistance_protein_AzlC,AzlC_protein_1243809:1244552_forward_MW:262	RbromiL2-63
Org5_Gene114	accessory	2840	[RbromiL2-63]L2-63_00117_L2-63_00117_Helix-turn-helix_domain_of_resolvase_130961:131524_forward_MW:21940	RbromiL2-63
Org5_Gene1140	accessory	3815	[RbromiL2-63]L2-63_01173_L2-63_01173_Predicted_membrane_protein,Branched-chain_amino_acid_transport_protein_(AzlD)_1244542:1244847_forwar	RbromiL2-63
Org5_Gene1141	accessory	1104	[RbromiL2-63]L2-63_01174_sbcD_Nuclease_sbcCD_subunit_D,exonuclease_subunit_SbcD,exonuclease_SbcCD,_D_subunit,Calcineurin-like_phosphoestera	RbromiL2-63
Org5_Gene1142	accessory	73	[RbromiL2-63]L2-63_01175_sbcC_Nuclease_sbcCD_subunit_C,exonuclease_subunit_SbcC,ATPase_involved_in_DNA_repair,exonuclease_SbcC_1246069:12	RbromiL2-63
Org5_Gene1143	accessory	2100	[RbromiL2-63]L2-63_01176_zupT_Zinc_transporter_ZupT,zinc_transporter_ZupT,ZIP_Zinc_transporter_1249330:1250112_forward_MW:26912	RbromiL2-63
Org5_Gene1144	accessory	3997	[RbromiL2-63]L2-63_01177_L2-63_01177_hypothetical_protein_1250256:1250513_forward_MW:9098	RbromiL2-63
Org5_Gene1146	accessory	2479	[RbromiL2-63]L2-63_01179_sdpI_Immunity_protein_sdpI,Predicted_integral_membrane_protein,Protein_of_unknown_function_(DUF1648)_1252719:125	RbromiL2-63
Org5_Gene1147	accessory	4003	[RbromiL2-63]L2-63_01180_czrA_HTH-type_transcriptional_repressor_CzrA,DNA-binding_transcriptional_repressor_ArsR,Uncharacterized_protein_conser	RbromiL2-63
Org5_Gene1148	accessory	858	[RbromiL2-63]L2-63_01181_eno_1_Enolase,enolase,L-alanine-DL-glutamate_epimerase_and_related_enzymes_of_enolase_superfamily,phosphopyruvate_	RbromiL2-63
Org5_Gene115	accessory	1979	[RbromiL2-63]L2-63_00118_L2-63_00118_hypothetical_protein_131521:132339_forward_MW:31540	RbromiL2-63
Org5_Gene1150	accessory	742	[RbromiL2-63]L2-63_01183_mepA_7_Staphylococcal_virulence_regulator_protein_A,multidrug_efflux_protein,MATE_efflux_family_protein,MatE_125582	RbromiL2-63
Org5_Gene1151	accessory	2826	[RbromiL2-63]L2-63_01184_L2-63_01184_hypothetical_protein_1257240:1257797_forward_MW:21794	RbromiL2-63
Org5_Gene1152	accessory	2632	[RbromiL2-63]L2-63_01185_L2-63_01185_Uracil_DNA_glycosylase_superfamily_1257816:1258433_forward_MW:24194	RbromiL2-63
Org5_Gene1153	accessory	1220	[RbromiL2-63]L2-63_01186_L2-63_01186_Uncharacterized_protein_conserved_in_bacteria,Acyltransferase_family_1258576:1259679_reverse_MW:41754	RbromiL2-63
Org5_Gene1154	accessory	108	[RbromiL2-63]L2-63_01187_L2-63_01187_CDP-Glycerol:Poly(glycerophosphate)_glycerophosphotransferase_1259982:1262696_reverse_MW:106437	RbromiL2-63
Org5_Gene1156	accessory	3156	[RbromiL2-63]L2-63_01189_L2-63_01189_putative_acetyltransferase,Predicted_acetyltransferase,_GNAT_superfamily,ribosomal-protein-alanine_acetyltra	RbromiL2-63
Org5_Gene1157	accessory	125	[RbromiL2-63]L2-63_01190_fus_2_Elongation_factor_G,elongation_factor_G,Predicted_RNA-binding_protein_containing_a_PIN_domain,translation_elong	RbromiL2-63
Org5_Gene1158	accessory	977	[RbromiL2-63]L2-63_01191_dapL_2_LL-diaminopimelate_aminotransferase,aspartate_aminotransferase,Alanine-alpha-ketoisovalerate_(or_valine-pyruvat	RbromiL2-63
Org5_Gene1159	accessory	4006	[RbromiL2-63]L2-63_01192_L2-63_01192_hypothetical_protein_1268861:1269115_reverse_MW:9288	RbromiL2-63
Org5_Gene1160	accessory	2931	[RbromiL2-63]L2-63_01193_L2-63_01193_hypothetical_protein_1269307:1269843_reverse_MW:20392	RbromiL2-63
Org5_Gene1161	accessory	2861	[RbromiL2-63]L2-63_01194_L2-63_01194_hypothetical_protein_1269863:1270417_reverse_MW:20700	RbromiL2-63
Org5_Gene1162	accessory	3092	[RbromiL2-63]L2-63_01195_L2-63_01195_hypothetical_protein_1270433:1270924_reverse_MW:19132	RbromiL2-63
Org5_Gene1163	accessory	2767	[RbromiL2-63]L2-63_01196_L2-63_01196_hypothetical_protein_1270938:1271519_reverse_MW:22341	RbromiL2-63
Org5_Gene1164	accessory	3419	[RbromiL2-63]L2-63_01197_def1_1_Peptide_deformylase_1,peptide_deformylase,N-formylmethionyl-tRNA_deformylase,peptide_deformylase,Polypeptid	RbromiL2-63
Org5_Gene1165	accessory	2311	[RbromiL2-63]L2-63_01198_L2-63_01198_hypothetical_protein_1272237:1272953_reverse_MW:27346	RbromiL2-63
Org5_Gene1166	accessory	3131	[RbromiL2-63]L2-63_01199_sigM_RNA_polymerase_sigma_factor_sigM,RNA_polymerase_sigma_factor,RNA_polymerase_sigma_factor,_SigM_family,Sigm	RbromiL2-63
Org5_Gene1167	accessory	4263	[RbromiL2-63]L2-63_01200_L2-63_01200_hypothetical_protein_1273561:1273752_forward_MW:7309	RbromiL2-63
Org5_Gene1168	accessory	3263	[RbromiL2-63]L2-63_01201_adhR_HTH-type_transcriptional_regulator_AdhR,zinc-responsive_transcriptional_regulator,Transcriptional_regulator,_effector	RbromiL2-63
Org5_Gene1169	accessory	1905	[RbromiL2-63]L2-63_01202_L2-63_01202_hypothetical_protein,Patatin-like_phospholipase_1274446:1275285_reverse_MW:31952	RbromiL2-63
Org5_Gene1170	accessory	2662	[RbromiL2-63]L2-63_01203_L2-63_01203_mycofactocin_system_transcriptional_regulator_1275350:1275931_forward_MW:22004	RbromiL2-63
Org5_Gene1171	accessory	2363	[RbromiL2-63]L2-63_01204_L2-63_01204_Cd1,azoreductase,Putative_NADPH-quinone_reductase_(modulator_of_drug_activity_B),Flavodoxin-like_fold_12	RbromiL2-63
Org5_Gene1174	accessory	213	[RbromiL2-63]L2-63_01207_fabF_3_3-oxoacyl-[acyl-carrier-protein]_synthase_2,3-oxoacyl-(acyl_carrier_protein)_synthase_II,Enoyl_reductase_domain_of_	RbromiL2-63
Org5_Gene1176	accessory	2422	[RbromiL2-63]L2-63_01209_aas_Bifunctional_protein_aas,2-acyl-glycerophospho-ethanolamine_acyltransferase,1-acylglycerol-3-phosphate_O-acyltransfe	RbromiL2-63
Org5_Gene1177	accessory	1299	[RbromiL2-63]L2-63_01210_trpS2_Tryptophan--tRNA_ligase_2,tryptophanyl-tRNA_synthetase_II,Tryptophanyl-tRNA_synthetase,tryptophan--tRNA_ligase,	RbromiL2-63
Org5_Gene1178	accessory	1381	[RbromiL2-63]L2-63_01211_L2-63_01211_Penicillin_V_acylase_and_related_amidases,Linear_amide_C-N_hydrolases,_choloylglycine_hydrolase_family_12	RbromiL2-63
Org5_Gene1179	accessory	840	[RbromiL2-63]L2-63_01212_L2-63_01212_sensory_histidine_kinase_DcuS,Predicted_signal_transduction_protein_with_a_C-terminal_ATPase_domain_128	RbromiL2-63
Org5_Gene1180	accessory	2284	[RbromiL2-63]L2-63_01213_yehT_Probable_transcriptional_regulatory_protein_YehT,two-component_response_regulator,Response_regulator_of_the_Ly	RbromiL2-63
Org5_Gene1181	accessory	272	[RbromiL2-63]L2-63_01214_L2-63_01214_Metallo-beta-lactamase_superfamily_1285777:1287807_forward_MW:75298	RbromiL2-63
Org5_Gene1182	accessory	2887	[RbromiL2-63]L2-63_01215_L2-63_01215_Uncharacterized_conserved_protein_1287826:1288371_forward_MW:19555	RbromiL2-63
Org5_Gene1187	accessory	1493	[RbromiL2-63]L2-63_01223_ntpC_V-type_sodium_pump_subunit_C,V-type_ATP_synthase_subunit_C,ATP_synthase_A1,_C_subunit,ATP_synthase_(C/AC3	RbromiL2-63
Org5_Gene1188	accessory	2760	[RbromiL2-63]L2-63_01224_L2-63_01224_V-type_ATP_synthase_subunit_E,ATP_synthase_(E/31_kDa)_subunit_1294339:1294920_reverse_MW:21987	RbromiL2-63
Org5_Gene1190	accessory	287	[RbromiL2-63]L2-63_01226_L2-63_01226_V-type_ATP_synthase_subunit_I,V-type_ATPase_116kDa_subunit_family_1295432:1297405_reverse_MW:7360	RbromiL2-63
Org5_Gene1191	accessory	3801	[RbromiL2-63]L2-63_01227_L2-63_01227_V-type_ATP_synthase_subunit_H_1297455:1297766_reverse_MW:11599	RbromiL2-63
Org5_Gene1192	accessory	1429	[RbromiL2-63]L2-63_01228_L2-63_01228_Protein_involved_in_cell_division,mobile_mystery_protein_B,Fic/DOC_family_1297998:1298996_reverse_MW:3	RbromiL2-63
Org5_Gene1194	accessory	32	[RbromiL2-63]L2-63_01230_swrC_Swarming_motility_protein_SwrC,multidrug_efflux_system_subunit_MdtC,Predicted_exporter,heavy_metal_efflux_pum	RbromiL2-63
Org5_Gene1195	accessory	2637	[RbromiL2-63]L2-63_01231_L2-63_01231_DNA-binding_transcriptional_repressor_AcrR,probable_dihydroxyacetone_kinase_regulator,Bacterial_regulatory	RbromiL2-63
Org5_Gene1196	accessory	3183	[RbromiL2-63]L2-63_01232_yfkJ_Low_molecular_weight_protein-tyrosine-phosphatase_yfkJ,phosphotyrosine-protein_phosphatase,Protein-tyrosine-phos	RbromiL2-63
Org5_Gene1197	accessory	4432	[RbromiL2-63]L2-63_01233_L2-63_01233_hypothetical_protein_1305363:1305467_reverse_MW:3692	RbromiL2-63
Org5_Gene1198	accessory	3000	[RbromiL2-63]L2-63_01234_L2-63_01234_hypothetical_protein_1305525:1306043_reverse_MW:20443	RbromiL2-63
Org5_Gene12	accessory	969	[RbromiL2-63]L2-63_00012_L2-63_00012_Uncharacterized_protein_involved_in_cytokinesis,_contains_TGc_(transglutaminase/protease-like)_domain,Trar	RbromiL2-63
Org5_Gene1200	accessory	2604	[RbromiL2-63]L2-63_01236_wcaJ_2_Putative_colanic_biosynthesis_UDP-glucose_lipid_carrier_transferase,putative_UDP-glucose_lipid_carrier_transferase	RbromiL2-63
Org5_Gene1202	accessory	1241	[RbromiL2-63]L2-63_01238_L2-63_01238_Glycogen_synthase,colanic_acid_biosynthesis_glycosyltransferase_WcaL,Glycogen_synthase,sugar_transferase,_	RbromiL2-63
Org5_Gene1203	accessory	1248	[RbromiL2-63]L2-63_01239_pimB_GDP-mannose-dependent_alpha-(1-6)-phosphatidylinositol_monomannoside_mannosyltransferase,Vi_polysaccharide_b	RbromiL2-63
Org5_Gene1204	accessory	1645	[RbromiL2-63]L2-63_01240_hyaD_Hyaluronan_synthase,putative_glycosyl_transferase,Predicted_glycosyl_hydrolase,poly-beta-1,6_N-acetyl-D-glucosamin	RbromiL2-63
Org5_Gene1206	accessory	1025	[RbromiL2-63]L2-63_01242_L2-63_01242_Resolvase,_N_terminal_domain_1310652:1311854_reverse_MW:45971	RbromiL2-63
Org5_Gene1207	accessory	2002	[RbromiL2-63]L2-63_01243_fliY_Sulfate_starvation-induced_protein_7,cystine_transporter_subunit,lysine-arginine-ornithine-binding_periplasmic_protein,	RbromiL2-63
Org5_Gene1210	accessory	3499	[RbromiL2-63]L2-63_01246_L2-63_01246_hypothetical_protein_1314632:1315021_forward_MW:13812	RbromiL2-63
Org5_Gene1211	accessory	878	[RbromiL2-63]L2-63_01247_yjiA_Uncharacterized_GTP-binding_protein_YjiA,putative_GTP-binding_protein_YjiA,Ni2_-binding_GTPase_involved_in_regula	RbromiL2-63
Org5_Gene1212	accessory	2837	[RbromiL2-63]L2-63_01248_maf_Septum_formation_protein_Maf,Maf-like_protein,Nucleotide-binding_protein_implicated_in_inhibition_of_septum_forn	RbromiL2-63
Org5_Gene1213	accessory	1608	[RbromiL2-63]L2-63_01249_cysE_3_Serine_acetyltransferase,serine_acetyltransferase,Acetyltransferase_(isoleucine_patch_superfamily),serine_O-acetyltr	RbromiL2-63
Org5_Gene1214	accessory	2360	[RbromiL2-63]L2-63_01250_L2-63_01250_Transglutaminase-like_superfamily_1318492:1319190_forward_MW:27133	RbromiL2-63
Org5_Gene1215	accessory	3013	[RbromiL2-63]L2-63_01251_L2-63_01251_ribosomal-protein-alanine_acetyltransferase,Acetyltransferase_(GNAT)_family_1319217:1319669_forward_MW	RbromiL2-63
Org5_Gene1216	accessory	3151	[RbromiL2-63]L2-63_01252_L2-63_01252_putative_acetyltransferase,ribosomal-protein-alanine_acetyltransferase,Acetyltransferase_(GNAT)_family_1319	RbromiL2-63
Org5_Gene1217	accessory	789	[RbromiL2-63]L2-63_01253_L2-63_01253_Na_-dependent_transporters_of_the_SNF_family,Sodium:neurotransmitter_symporter_family_1320305:132163	RbromiL2-63
Org5_Gene1218	accessory	1741	[RbromiL2-63]L2-63_01254_cynR_Cyn_operon_transcriptional_activator,DNA-binding_transcriptional_regulator_CynR,aminoethylphosphonate_catabolism	RbromiL2-63
Org5_Gene122	accessory	324	[RbromiL2-63]L2-63_00125_L2-63_00125_hypothetical_protein_142932:144824_forward_MW:67317	RbromiL2-63

Org5_Gene1220	accessory	3963	[RbromiL2-63]L2-63_01256_L2-63_01256_CotJB_protein_1322822:1323085_reverse_MW:10452	RbromiL2-63
Org5_Gene1221	accessory	4215	[RbromiL2-63]L2-63_01257_L2-63_01257_Spore_coat_associated_protein_JA_(CotJA)_1323069:1323269_reverse_MW:7358	RbromiL2-63
Org5_Gene1222	accessory	1933	[RbromiL2-63]L2-63_01258_thiT_Thiamine_ECF_transporter_S_component_ThiT,putative_proton-coupled_thiamine_transporter_YuaJ,Thiamine_transpor	RbromiL2-63
Org5_Gene1225	accessory	2700	[RbromiL2-63]L2-63_01262_L2-63_01262_Alpha/beta_hydrolase_family_1327889:1328488_forward_MW:23227	RbromiL2-63
Org5_Gene1226	accessory	3193	[RbromiL2-63]L2-63_01263_L2-63_01263_hypothetical_protein_1328516:1328983_forward_MW:17132	RbromiL2-63
Org5_Gene123	accessory	2712	[RbromiL2-63]L2-63_00126_kynB_Kynurenine_formamidase,arylformamidase,Putative_cyclase_145090:145683_forward_MW:21484	RbromiL2-63
Org5_Gene1231	accessory	4335	[RbromiL2-63]L2-63_01268_L2-63_01268_Virus_attachment_protein_p12_family_1332962:1333135_forward_MW:6062	RbromiL2-63
Org5_Gene1232	accessory	2386	[RbromiL2-63]L2-63_01269_macB_3_Macrolide_export_ATP-binding/permease_protein_MacB,macrolide_transporter_ATP-binding_/permease_protein,Pr	RbromiL2-63
Org5_Gene1233	accessory	202	[RbromiL2-63]L2-63_01270_L2-63_01270_FtsX-like_permease_family_1333987:1336230_forward_MW:85303	RbromiL2-63
Org5_Gene1234	accessory	3556	[RbromiL2-63]L2-63_01271_L2-63_01271_putative_mercuric_reductase,Copper_chaperone,copper_ion_binding_protein,Heavy-metal-associated_domain_	RbromiL2-63
Org5_Gene1235	accessory	1531	[RbromiL2-63]L2-63_01272_soxS_Regulatory_protein_soxS,DNA-binding_transcriptional_regulator_MelR,DNA_gyrase_inhibitor,YSIRK-targeted_surface_ar	RbromiL2-63
Org5_Gene1236	accessory	2862	[RbromiL2-63]L2-63_01273_L2-63_01273_Protein_of_unknown_function_(DUF3793)_1338020:1338574_forward_MW:21042	RbromiL2-63
Org5_Gene124	accessory	2517	[RbromiL2-63]L2-63_00127_gph_Phosphoglycolate_phosphatase,phosphoglycolate_phosphatase,phosphoglycolate_phosphatase,_bacterial,haloacid_deha	RbromiL2-63
Org5_Gene1241	accessory	3105	[RbromiL2-63]L2-63_01279_ywnH_Putative_phosphinothricin_acetyltransferase_YwnH,putative_acetyltransferase,Predicted_acetyltransferase,ribosomal-[illegible]	RbromiL2-63
Org5_Gene1242	accessory	3696	[RbromiL2-63]L2-63_01280_L2-63_01280_Uncharacterized_conserved_protein,_contains_double-stranded_beta-helix_domain,Cupin_domain_1343232:13	RbromiL2-63
Org5_Gene1243	accessory	4130	[RbromiL2-63]L2-63_01281_L2-63_01281_hypothetical_protein_1343647:1343868_forward_MW:8242	RbromiL2-63
Org5_Gene1244	accessory	3287	[RbromiL2-63]L2-63_01282_L2-63_01282_hypothetical_protein_1343917:1344360_reverse_MW:17355	RbromiL2-63
Org5_Gene1245	accessory	4066	[RbromiL2-63]L2-63_01283_relJ_Antitoxin_YefM,antitoxin_YefM,Phd_YefM_1344633:1344872_forward_MW:9163	RbromiL2-63
Org5_Gene1246	accessory	3921	[RbromiL2-63]L2-63_01284_L2-63_01284_addiction_module_toxin,_Txe/YoeB_family,Plasmid_encoded_toxin_Txe_1344862:1345137_forward_MW:10974	RbromiL2-63
Org5_Gene1247	accessory	1853	[RbromiL2-63]L2-63_01285_pdxK_Pyridoxine_kinase,pyridoxamine_kinase,pyridoxal_kinase,Phosphomethylpyrimidine_kinase_1345315:1346169_forward_	RbromiL2-63
Org5_Gene1248	accessory	2872	[RbromiL2-63]L2-63_01286_L2-63_01286_Predicted_membrane_protein,conserved_hypothetical_protein,Protein_of_unknown_function_(DUF1393)_1346	RbromiL2-63
Org5_Gene1249	accessory	2678	[RbromiL2-63]L2-63_01287_L2-63_01287_molybdenum_cofactor_biosynthesis_protein_A,Uncharacterized_conserved_protein_related_to_pyruvate_form	RbromiL2-63
Org5_Gene125	accessory	2420	[RbromiL2-63]L2-63_00128_L2-63_00128_hypothetical_protein,Uncharacterized_BCR,_COG1636_146301:146987_forward_MW:26915	RbromiL2-63
Org5_Gene1250	accessory	2713	[RbromiL2-63]L2-63_01288_L2-63_01288_Ion_channel_1347576:1347998_forward_MW:15757	RbromiL2-63
Org5_Gene1251	accessory	752	[RbromiL2-63]L2-63_01289_L2-63_01289_Na_-dependent_transporters_of_the_SNF_family,Sodium:neurotransmitter_symporter_family_1348083:134943	RbromiL2-63
Org5_Gene1252	accessory	1713	[RbromiL2-63]L2-63_01290_L2-63_01290_hypothetical_protein_1349692:1350588_forward_MW:34328	RbromiL2-63
Org5_Gene1253	accessory	3510	[RbromiL2-63]L2-63_01291_L2-63_01291_hypothetical_protein_1350745:1351131_forward_MW:14145	RbromiL2-63
Org5_Gene1254	accessory	2571	[RbromiL2-63]L2-63_01292_L2-63_01292_hypothetical_protein_1351209:1351847_reverse_MW:24955	RbromiL2-63
Org5_Gene1255	accessory	2783	[RbromiL2-63]L2-63_01293_hcpC_Putative_beta-lactamase_hcpC_precursor,Sel1_repeat_1352135:1352710_forward_MW:21341	RbromiL2-63
Org5_Gene1257	accessory	698	[RbromiL2-63]L2-63_01296_purP_Probable_adenine_permease_PurP,Permeases,Permease_family_1354233:1355627_forward_MW:49291	RbromiL2-63
Org5_Gene1258	accessory	3351	[RbromiL2-63]L2-63_01297_cymR_Cysteine_metabolism_repressor,DNA-binding_transcriptional_regulator_IscR,Rrf2_family_protein,Transcriptional_regul	RbromiL2-63
Org5_Gene126	accessory	430	[RbromiL2-63]L2-63_00129_ptsI_Phosphoenolpyruvate-protein_phosphotransferase,phosphoenolpyruvate-protein_phosphotransferase,Uncharacterized_	RbromiL2-63
Org5_Gene1260	accessory	1599	[RbromiL2-63]L2-63_01299_cysK_Cysteine_synthase,cysteine_synthase_A,Cysteine_synthase,cysteine_synthase_A,Pyridoxal-phosphate_dependent_enzyn	RbromiL2-63
Org5_Gene1261	accessory	2651	[RbromiL2-63]L2-63_01300_trpF_N-(5'-phosphoribosyl)anthranilate_isomerase,N-(5'-phosphoribosyl)anthranilate_isomerase,Phosphoribosylanthranilate_i	RbromiL2-63
Org5_Gene1265	accessory	3927	[RbromiL2-63]L2-63_01304_yafQ_1_mRNA_interferase_YafQ,addiction_module_toxin,_RelE/StbE_family,Plasmid_stabilisation_system_protein_1360990:1	RbromiL2-63
Org5_Gene1266	accessory	3952	[RbromiL2-63]L2-63_01305_L2-63_01305_addiction_module_antitoxin,_RelB/DinJ_family,RelB_antitoxin_1361262:1361531_reverse_MW:10212	RbromiL2-63
Org5_Gene1269	accessory	3941	[RbromiL2-63]L2-63_01308_dinJ_Antitoxin_DinJ,bifunctional_antitoxin/transcriptional_repressor_RelB,addiction_module_antitoxin,_RelB/DinJ_family,RelE	RbromiL2-63
Org5_Gene1274	accessory	2902	[RbromiL2-63]L2-63_01314_L2-63_01314_hypothetical_protein_1370051:1370593_forward_MW:20971	RbromiL2-63
Org5_Gene1275	accessory	1792	[RbromiL2-63]L2-63_01315_nnr_2_Nicotinamide_nucleotide_repair_protein,putative_carbohydrate_kinase,Uncharacterized_conserved_protein,YjeF_fami	RbromiL2-63
Org5_Gene1276	accessory	2787	[RbromiL2-63]L2-63_01316_L2-63_01316_putative_nicotinate-nucleotide_adenylyltransferase,Predicted_HD_superfamily_hydrolase,HD_domain_1371678	RbromiL2-63
Org5_Gene1277	accessory	3437	[RbromiL2-63]L2-63_01317_L2-63_01317_hypothetical_protein_1372377:1372781_forward_MW:14936	RbromiL2-63
Org5_Gene1279	accessory	699	[RbromiL2-63]L2-63_01319_mgtE_Magnesium_transporter_mgtE,Predicted_transcriptional_regulator_containing_CBS_domains,magnesium_transporter,D	RbromiL2-63
Org5_Gene128	accessory	308	[RbromiL2-63]L2-63_00131_fruA_EIIABC-Fru,PTS_system_fructose-specific_transporter_subunits_IIBC,PTS_system,_Fru_family,_IIC_component,Phosphoe	RbromiL2-63
Org5_Gene1280	accessory	2884	[RbromiL2-63]L2-63_01320_L2-63_01320_Cd1,Putative_NADPH-quinone_reductase_(modulator_of_drug_activity_B),NADPH-dependent_FMN_reductase_	RbromiL2-63
Org5_Gene1281	accessory	2988	[RbromiL2-63]L2-63_01321_L2-63_01321_hypothetical_protein_1375409:1375930_forward_MW:18771	RbromiL2-63
Org5_Gene1283	accessory	2478	[RbromiL2-63]L2-63_01323_immR_10_HTH-type_transcriptional_regulator_immR,Predicted_transcriptional_regulator,transcriptional_regulator,_y4mF_far	RbromiL2-63
Org5_Gene1286	accessory	2242	[RbromiL2-63]L2-63_01326_cpsB_Tyrosine-protein_phosphatase_CpsB,Capsular_polysaccharide_biosynthesis_protein_1379160:1379894_forward_MW:27	RbromiL2-63
Org5_Gene129	accessory	1678	[RbromiL2-63]L2-63_00132_lacC_Tagatose-6-phosphate_kinase,1-phosphofructokinase,ADP-heptose_synthase,_bifunctional_sugar_kinase/adenylyltransfe	RbromiL2-63
Org5_Gene1291	accessory	294	[RbromiL2-63]L2-63_01331_L2-63_01331_putative_bifunctional_signaling_protein/50S_ribosomal_protein_L9,Predicted_signaling_protein_consisting_of_	RbromiL2-63
Org5_Gene1294	accessory	804	[RbromiL2-63]L2-63_01334_tilS_tRNA(Ile)-lysidine_synthase,tRNA(Ile)-lysidine_synthetase,Predicted_ATPase_of_the_PP-loop_superfamily_implicated_in_	RbromiL2-63
Org5_Gene1299	accessory	4071	[RbromiL2-63]L2-63_01339_L2-63_01339_hypothetical_protein_1395778:1396014_forward_MW:8225	RbromiL2-63
Org5_Gene130	accessory	2142	[RbromiL2-63]L2-63_00133_glcR_1_HTH-type_transcriptional_repressor_glcR,DNA-binding_transcriptional_regulator_AgaR,Phosphotransferase_system,_r	RbromiL2-63
Org5_Gene1300	accessory	2112	[RbromiL2-63]L2-63_01340_L2-63_01340_N-acetylmuramoyl-L-alanine_amidase_1396007:1396786_forward_MW:27667	RbromiL2-63
Org5_Gene1301	accessory	696	[RbromiL2-63]L2-63_01341_mepA_8_Multidrug_export_protein_mepA,multidrug_efflux_pump_VmrA,MATE_efflux_family_protein,MatE_1397222:13986	RbromiL2-63
Org5_Gene1302	accessory	4191	[RbromiL2-63]L2-63_01342_L2-63_01342_hypothetical_protein_1398984:1399190_forward_MW:8226	RbromiL2-63
Org5_Gene1303	accessory	3835	[RbromiL2-63]L2-63_01343_L2-63_01343_RNA_polymerase_sigma_factor,Transcriptional_regulator,_contains_sigma_factor-related_N-terminal_domain,R	RbromiL2-63
Org5_Gene1304	accessory	2657	[RbromiL2-63]L2-63_01344_L2-63_01344_hypothetical_protein_1399491:1400099_reverse_MW:22267	RbromiL2-63
Org5_Gene1305	accessory	842	[RbromiL2-63]L2-63_01345_L2-63_01345_hypothetical_protein_1400340:1401653_forward_MW:47318	RbromiL2-63
Org5_Gene1307	accessory	2014	[RbromiL2-63]L2-63_01348_L2-63_01348_D-alanyl-D-alanine_carboxypeptidase,D-alanyl-D-alanine_carboxypeptidase_1403953:1404747_forward_MW:30	RbromiL2-63
Org5_Gene131	accessory	2569	[RbromiL2-63]L2-63_00134_L2-63_00134_transcriptional_regulator_FixK,Cyclic_nucleotide-binding_domain_153215:153856_reverse_MW:24795	RbromiL2-63
Org5_Gene1313	accessory	3818	[RbromiL2-63]L2-63_01354_L2-63_01354_Muramidase-2_precursor,membrane-bound_lytic_murein_transglycosylase_D,Oxidation_resistance_protein,spo	RbromiL2-63
Org5_Gene1314	accessory	930	[RbromiL2-63]L2-63_01355_eno_2_Enolase,enolase,L-alanine-DL-glutamate_epimerase_and_related_enzymes_of_enolase_superfamily,phosphopyruvate_	RbromiL2-63
Org5_Gene1316	accessory	71	[RbromiL2-63]L2-63_01357_pulA_3_Pullulanase_precursor,glycogen_debranching_enzyme,pullulanase,_type_I,Alpha_amylase,_catalytic_domain_141426	RbromiL2-63
Org5_Gene1321	accessory	1731	[RbromiL2-63]L2-63_01362_L2-63_01362_sporulation_inhibitor_KapD,Inhibitor_of_the_KinA_pathway_to_sporulation,_predicted_exonuclease,DNA_polyr	RbromiL2-63
Org5_Gene1322	accessory	3137	[RbromiL2-63]L2-63_01363_L2-63_01363_hypothetical_protein_1424448:1424924_forward_MW:18168	RbromiL2-63
Org5_Gene1323	accessory	427	[RbromiL2-63]L2-63_01364_htrA_2_Putative_serine_protease_HtrA,serine_endoprotease,Predicted_protease_with_the_C-terminal_PDZ_domain,peptidas	RbromiL2-63
Org5_Gene1324	accessory	2313	[RbromiL2-63]L2-63_01365_lytR_4_Sensory_transduction_protein_lytR,putative_two-component_response-regulatory_protein_YehT,Response_regulator_	RbromiL2-63
Org5_Gene1325	accessory	303	[RbromiL2-63]L2-63_01366_L2-63_01366_hypothetical_protein_1427730:1429667_reverse_MW:73207	RbromiL2-63
Org5_Gene1326	accessory	1194	[RbromiL2-63]L2-63_01367_L2-63_01367_hypothetical_protein_1429878:1430999_forward_MW:40814	RbromiL2-63
Org5_Gene1329	accessory	2542	[RbromiL2-63]L2-63_01370_L2-63_01370_hypothetical_protein_1434198:1434848_forward_MW:24212	RbromiL2-63
Org5_Gene1330	accessory	2563	[RbromiL2-63]L2-63_01371_rebM_Rebeccamycin_O-methyltransferase,hypothetical_protein,Trans-aconitate_methyltransferase,ubiquinone/menaquinone	RbromiL2-63
Org5_Gene1332	accessory	1507	[RbromiL2-63]L2-63_01373_L2-63_01373_hypothetical_protein,YihY_family_inner_membrane_protein,Ribonuclease_BN-like_family_1436333:1437298_fo	RbromiL2-63
Org5_Gene1333	accessory	2276	[RbromiL2-63]L2-63_01374_L2-63_01374_hypothetical_protein_1437350:1438075_forward_MW:26991	RbromiL2-63
Org5_Gene1334	accessory	3132	[RbromiL2-63]L2-63_01375_L2-63_01375_transcriptional_regulator_SlyA,homoprotocatechuate_degradation_operon_regulator,_HpaR,MarR_family_1438	RbromiL2-63
Org5_Gene1337	accessory	1630	[RbromiL2-63]L2-63_01378_L2-63_01378_ComEC_family_competence_protein,Predicted_metal-dependent_RNase,_consists_of_a_metallo-beta-lactamas	RbromiL2-63
Org5_Gene1338	accessory	4143	[RbromiL2-63]L2-63_01379_L2-63_01379_Protein_of_unknown_function_(DUF3006)_1443462:1443680_forward_MW:8334	RbromiL2-63
Org5_Gene1339	accessory	3133	[RbromiL2-63]L2-63_01380_L2-63_01380_hypothetical_protein_1443759:1444241_reverse_MW:18249	RbromiL2-63
Org5_Gene134	accessory	2329	[RbromiL2-63]L2-63_00137_L2-63_00137_hypothetical_protein_156973:157686_forward_MW:26264	RbromiL2-63
Org5_Gene1340	accessory	2085	[RbromiL2-63]L2-63_01381_yycF_1_Transcriptional_regulatory_protein_YycF,DNA-binding_transcriptional_regulator_BaeR,Response_regulator_containing	RbromiL2-63
Org5_Gene1342	accessory	790	[RbromiL2-63]L2-63_01383_yycG_Sensor_histidine_kinase_YycG,sensory_histidine_kinase_AtoS,Signal_transduction_histidine_kinase,_nitrogen_specific,p	RbromiL2-63
Org5_Gene1343	accessory	567	[RbromiL2-63]L2-63_01384_tycA_Tyrocidine_synthase_I,D-alanine--poly(phosphoribitol)_ligase_subunit_1,Anthranilate/para-aminobenzoate_synthases_c	RbromiL2-63
Org5_Gene1344	accessory	1073	[RbromiL2-63]L2-63_01385_lysA_1_Diaminopimelate_decarboxylase,bifunctional_aspartate_kinase/diaminopimelate_decarboxylase_protein,Diaminopime	RbromiL2-63
Org5_Gene1345	accessory	4091	[RbromiL2-63]L2-63_01386_L2-63_01386_Aminoacyl_carrier_protein_2,D-alanine--poly(phosphoribitol)_ligase_subunit_2_1449461:1449691_forward_MW	RbromiL2-63
Org5_Gene1346	accessory	482	[RbromiL2-63]L2-63_01387_dltB_2_hypothetical_protein,D-alanyl-lipoteichoic_acid_biosynthesis_protein_DltB,MBOAT_family_1449693:1451327_forward	RbromiL2-63
Org5_Gene1347	accessory	1470	[RbromiL2-63]L2-63_01388_L2-63_01388_hypothetical_protein_1451340:1452320_forward_MW:37900	RbromiL2-63
Org5_Gene1348	accessory	2829	[RbromiL2-63]L2-63_01389_L2-63_01389_HAD_hydrolase,_family_IB,haloacid_dehalogenase-like_hydrolase_1452798:1453364_reverse_MW:22048	RbromiL2-63
Org5_Gene1349	accessory	1817	[RbromiL2-63]L2-63_01390_L2-63_01390_hypothetical_protein_1453591:1454454_forward_MW:32775	RbromiL2-63
Org5_Gene1350	accessory	2650	[RbromiL2-63]L2-63_01391_dnaJ_2_Chaperone_protein_DnaJ,chaperone_protein_DnaJ,Preprotein_translocase_subunit_Sec63,chaperone_protein_DnaJ,D	RbromiL2-63
Org5_Gene1351	accessory	2699	[RbromiL2-63]L2-63_01392_L2-63_01392_hypothetical_protein_1455012:1455611_forward_MW:22192	RbromiL2-63
Org5_Gene1352	accessory	138	[RbromiL2-63]L2-63_01393_L2-63_01393_invasion_associated_secreted_endopeptidase,Phage-related_lysozyme_(muraminidase),SH3_domain_protein,Ba	RbromiL2-63
Org5_Gene1354	accessory	2477	[RbromiL2-63]L2-63_01395_L2-63_01395_stage_II_sporulation_protein_M_1459044:1459712_forward_MW:24721	RbromiL2-63
Org5_Gene1355	accessory	1547	[RbromiL2-63]L2-63_01396_L2-63_01396_Predicted_integral_membrane_protein_1459727:1460677_forward_MW:36168	RbromiL2-63
Org5_Gene1356	accessory	2541	[RbromiL2-63]L2-63_01397_L2-63_01397_hypothetical_protein_1460674:1461324_forward_MW:22616	RbromiL2-63
Org5_Gene1357	accessory	2915	[RbromiL2-63]L2-63_01398_L2-63_01398_hypothetical_protein_1461308:1461847_forward_MW:19243	RbromiL2-63
Org5_Gene1358	accessory	1790	[RbromiL2-63]L2-63_01399_L2-63_01399_Radical_SAM_superfamily_1461907:1462779_reverse_MW:33449	RbromiL2-63
Org5_Gene1361	accessory	1930	[RbromiL2-63]L2-63_01402_L2-63_01402_hypothetical_protein_1464653:1465486_forward_MW:31788	RbromiL2-63
Org5_Gene1368	accessory	3972	[RbromiL2-63]L2-63_01409_L2-63_01409_pro-sigmaK_processing_inhibitor_BofA,SigmaK-factor_processing_regulatory_protein_BofA_1474003:1474263_f	RbromiL2-63
Org5_Gene1369	accessory	2708	[RbromiL2-63]L2-63_01410_L2-63_01410_hypothetical_protein_1474319:1474915_reverse_MW:21931	RbromiL2-63
Org5_Gene137	accessory	140	[RbromiL2-63]L2-63_00140_pbpD_Penicillin-binding_protein_4_precursor,penicillin-binding_protein_1a,Membrane_carboxypeptidase/penicillin-binding_	RbromiL2-63
Org5_Gene1370	accessory	3306	[RbromiL2-63]L2-63_01411_iscR_2_HTH-type_transcriptional_regulator_iscR,DNA-binding_transcriptional_regulator_IscR,Rrf2_family_protein,Transcriptio	RbromiL2-63
Org5_Gene1371	accessory	2167	[RbromiL2-63]L2-63_01412_L2-63_01412_Probable_ABC_transporter_ATP-binding_protein_M6_Spy0273,cysteine_desulfurase_ATPase_component,ABC-t	RbromiL2-63
Org5_Gene1372	accessory	682	[RbromiL2-63]L2-63_01413_L2-63_01413_hypothetical_protein,cysteine_desulfurase_activator_complex_subunit_SufB,FeS_assembly_protein_SufB,Uncha	RbromiL2-63
Org5_Gene1373	accessory	1257	[RbromiL2-63]L2-63_01414_sufD_FeS_cluster_assembly_protein_sufD,cysteine_desulfurase_activator_complex_subunit_SufB,FeS_assembly_protein_SufD	RbromiL2-63
Org5_Gene1374	accessory	989	[RbromiL2-63]L2-63_01415_csd_1_Probable_cysteine_desulfurase,bifunctional_cysteine_desulfurase/selenocysteine_lyase,Uncharacterized_protein_cons	RbromiL2-63
Org5_Gene1375	accessory	3356	[RbromiL2-63]L2-63_01416_nifU_2_NifU-like_protein,scaffold_protein,SUF_system_FeS_assembly_protein,_NifU_family,NifU-like_N_terminal_domain_14	RbromiL2-63
Org5_Gene1376	accessory	2961	[RbromiL2-63]L2-63_01417_L2-63_01417_hypothetical_protein_1480695:1481225_forward_MW:20123	RbromiL2-63
Org5_Gene1377	accessory	3168	[RbromiL2-63]L2-63_01418_L2-63_01418_hypothetical_protein_1481390:1481863_forward_MW:17689	RbromiL2-63
Org5_Gene1378	accessory	2042	[RbromiL2-63]L2-63_01419_L2-63_01419_CAAX_prenyl_protease-related_protein,CAAX_amino_terminal_protease_self-_immunity_1482034:1482834_for	RbromiL2-63
Org5_Gene1379	accessory	2978	[RbromiL2-63]L2-63_01420_L2-63_01420_hypothetical_protein_1483205:1483729_forward_MW:19595	RbromiL2-63
Org5_Gene138	accessory	3725	[RbromiL2-63]L2-63_00141_L2-63_00141_hypothetical_protein_163253:163582_forward_MW:11855	RbromiL2-63
Org5_Gene1380	accessory	2504	[RbromiL2-63]L2-63_01421_L2-63_01421_hypothetical_protein_1484311:1484667_forward_MW:13821	RbromiL2-63
Org5_Gene1381	accessory	3537	[RbromiL2-63]L2-63_01422_L2-63_01422_hypothetical_protein_1484918:1485295_reverse_MW:14433	RbromiL2-63
Org5_Gene1382	accessory	3625	[RbromiL2-63]L2-63_01423_L2-63_01423_transcriptional_regulator,_Acidobacterial,_PadR-family,Transcriptional_regulator_PadR-like_family_1485285:14	RbromiL2-63
Org5_Gene1383	accessory	1508	[RbromiL2-63]L2-63_01424_L2-63_01424_thiol-disulfide_oxidoreductase,periplasmic_protein_thiol:disulfide_oxidoreductases,_DsbE_subfamily,AhpC/TSA_	RbromiL2-63
Org5_Gene1384	accessory	1457	[RbromiL2-63]L2-63_01425_yccM_Putative_electron_transport_protein_yccM,quinol_dehydrogenase_membrane_component,Uncharacterized_Fe-S_cent	RbromiL2-63
Org5_Gene1385	accessory	2442	[RbromiL2-63]L2-63_01426_mprA_Mycobacterial_persistence_regulator_A,DNA-binding_transcriptional_regulator_BasR,Response_regulator_of_citrate/r	RbromiL2-63
Org5_Gene1386	accessory	1157	[RbromiL2-63]L2-63_01427_arlS_2_Signal_transduction_histidine-protein_kinase_ArlS,two-component_sensor_protein,Predicted_periplasmic_ligand-bind	RbromiL2-63
Org5_Gene1387	accessory	2680	[RbromiL2-63]L2-63_01428_L2-63_01428_DNA-binding_transcriptional_repressor_AcrR,Bacterial_regulatory_proteins,_tetR_family_1490405:1491007_for	RbromiL2-63
Org5_Gene1388	accessory	293	[RbromiL2-63]L2-63_01429_L2-63_01429_Collagen_triple_helix_repeat_(20_copies)_1491004:1492830_forward_MW:64474	RbromiL2-63
Org5_Gene1389	accessory	514	[RbromiL2-63]L2-63_01430_L2-63_01430_hypothetical_protein_1496080:1497300_forward_MW:44769	RbromiL2-63
Org5_Gene1390	accessory	915	[RbromiL2-63]L2-63_01431_L2-63_01431_Peptidase_M16_inactive_domain_1497739:1499004_forward_MW:46789	RbromiL2-63
Org5_Gene1394	accessory	3777	[RbromiL2-63]L2-63_01435_L2-63_01435_hypothetical_protein,Predicted_ribosomal_protein,Protein_of_unknown_function_(DUF464)_1502405:1502722_	RbromiL2-63

Org5_Gene1398	accessory	1725	[RbromiL2-63]L2-63_01439_L2-63_01439_Uncharacterized_BCR,_YitT_family_COG1284_1504846:1505739_forward_MW:31924	RbromiL2-63
Org5_Gene1399	accessory	1295	[RbromiL2-63]L2-63_01440_L2-63_01440_glycosyltransferase,GtrA-like_protein_1505791:1506855_reverse_MW:40209	RbromiL2-63
Org5_Gene1400	accessory	425	[RbromiL2-63]L2-63_01441_amyE_2_Alpha-amylase_precursor_1507893:1508816_forward_MW:34128	RbromiL2-63
Org5_Gene1402	accessory	211	[RbromiL2-63]L2-63_01443_L2-63_01443_Dockerin_type_I_repeat_1510087:1512291_forward_MW:79232	RbromiL2-63
Org5_Gene1410	accessory	797	[RbromiL2-63]L2-63_01451_L2-63_01451_hypothetical_protein_1519446:1520774_forward_MW:48119	RbromiL2-63
Org5_Gene1414	accessory	2761	[RbromiL2-63]L2-63_01455_gmk_Guanylate_kinase,guanylate_kinase,Guanylate_kinase,guanylate_kinase,Guanylate_kinase_1522940:1523521_forward_M	RbromiL2-63
Org5_Gene1420	accessory	779	[RbromiL2-63]L2-63_01461_rsmB_Ribosomal_RNA_small_subunit_methyltransferase_B,16S_rRNA_methyltransferase_B,tRNA_and_rRNA_cytosine-C5-me	RbromiL2-63
Org5_Gene1425	accessory	2602	[RbromiL2-63]L2-63_01466_thiN_Thiamine_pyrophosphokinase,Thiamine_pyrophosphokinase,thiamine_pyrophosphokinase,Thiamin_pyrophosphokinase,	RbromiL2-63
Org5_Gene143	accessory	3163	[RbromiL2-63]L2-63_00146_accB_Biotin_carboxyl_carrier_protein_of_acetyl-CoA_carboxylase,acetyl-CoA_carboxylase_biotin_carboxyl_carrier_protein_su	RbromiL2-63
Org5_Gene1430	accessory	3045	[RbromiL2-63]L2-63_01472_L2-63_01472_hypothetical_protein_1539721:1540209_forward_MW:18224	RbromiL2-63
Org5_Gene1431	accessory	987	[RbromiL2-63]L2-63_01473_L2-63_01473_hypothetical_protein_1540206:1541426_forward_MW:47623	RbromiL2-63
Org5_Gene1432	accessory	1815	[RbromiL2-63]L2-63_01474_degV_hypothetical_protein,EDD_domain_protein,_DegV_family,Uncharacterised_protein,_DegV_family_COG1307_1541532:1	RbromiL2-63
Org5_Gene1433	accessory	438	[RbromiL2-63]L2-63_01475_L2-63_01475_conserved_hypothetical_integral_membrane_protein_1542420:1544102_reverse_MW:63481	RbromiL2-63
Org5_Gene1435	accessory	3844	[RbromiL2-63]L2-63_01478_L2-63_01478_hypothetical_protein,Uncharacterized_protein_conserved_in_bacteria,Protein_of_unknown_function_(DUF1292	RbromiL2-63
Org5_Gene1437	accessory	580	[RbromiL2-63]L2-63_01480_L2-63_01480_Lipid_A_core_-_O-antigen_ligase_and_related_enzymes,O-Antigen_ligase_1546863:1548365_forward_MW:578	RbromiL2-63
Org5_Gene1439	accessory	3054	[RbromiL2-63]L2-63_01482_L2-63_01482_hypothetical_protein_1550386:1550886_forward_MW:18867	RbromiL2-63
Org5_Gene1443	accessory	2689	[RbromiL2-63]L2-63_01486_L2-63_01486_stage_II_sporulation_protein_R,Stage_II_sporulation_protein_R_(spore_II_R)_1553681:1554283_forward_MW:	RbromiL2-63
Org5_Gene1444	accessory	60	[RbromiL2-63]L2-63_01487_addB_ATP-dependent_helicase/deoxyribonuclease_subunit_B,Inactivated_superfamily_I_helicase,helicase-exonuclease_AddA	RbromiL2-63
Org5_Gene1445	accessory	51	[RbromiL2-63]L2-63_01488_addA_ATP-dependent_helicase/nuclease_subunit_A,putative_recombination_protein_RecB,Superfamily_I_DNA_and_RNA_he	RbromiL2-63
Org5_Gene1446	accessory	2734	[RbromiL2-63]L2-63_01489_L2-63_01489_Uncharacterized_conserved_protein_1561328:1561918_reverse_MW:21824	RbromiL2-63
Org5_Gene1448	accessory	2225	[RbromiL2-63]L2-63_01491_ispD1_2-C-methyl-D-erythritol_4-phosphate_cytidylyltransferase_1,2-C-methyl-D-erythritol_4-phosphate_cytidylyltransferase,	RbromiL2-63
Org5_Gene1449	accessory	1266	[RbromiL2-63]L2-63_01492_strE_1_dTDP-glucose_4,6-dehydratase,NAD-dependent_epimerase/dehydratase_family_protein,dTDP-glucose_4,6-dehydratas	RbromiL2-63
Org5_Gene1451	accessory	328	[RbromiL2-63]L2-63_01494_L2-63_01494_LPS_biosynthesis_protein,LicD_family_1565770:1567125_forward_MW:51250	RbromiL2-63
Org5_Gene1452	accessory	2802	[RbromiL2-63]L2-63_01495_bar_Phosphinothricin_N-acetyltransferase_1567280:1567852_reverse_MW:22052	RbromiL2-63
Org5_Gene1454	accessory	96	[RbromiL2-63]L2-63_01497_L2-63_01497_Uncharacterized_protein_conserved_in_bacteria,Acyltransferase_family_1571455:1574304_reverse_MW:1084	RbromiL2-63
Org5_Gene1455	accessory	507	[RbromiL2-63]L2-63_01498_L2-63_01498_Predicted_integral_membrane_protein,conserved_hypothetical_integral_membrane_protein_1574497:1576089	RbromiL2-63
Org5_Gene1458	accessory	467	[RbromiL2-63]L2-63_01501_L2-63_01501_Uncharacterized_protein_conserved_in_bacteria_1578437:1580071_forward_MW:61367	RbromiL2-63
Org5_Gene1459	accessory	2074	[RbromiL2-63]L2-63_01502_mscS_Small-conductance_mechanosensitive_channel,mechanosensitive_channel_MscS,Mechanosensitive_ion_channel_1580	RbromiL2-63
Org5_Gene1460	accessory	3756	[RbromiL2-63]L2-63_01503_L2-63_01503_hypothetical_protein_1580960:1581283_forward_MW:11287	RbromiL2-63
Org5_Gene1462	accessory	2030	[RbromiL2-63]L2-63_01505_L2-63_01505_sigma-E_processing_peptidase_SpoIIGA,Sporulation_factor_SpoIIGA_1582078:1582881_reverse_MW:29621	RbromiL2-63
Org5_Gene1465	accessory	1497	[RbromiL2-63]L2-63_01508_L2-63_01508_hypothetical_protein_1584763:1585725_forward_MW:34628	RbromiL2-63
Org5_Gene1470	accessory	2102	[RbromiL2-63]L2-63_01514_L2-63_01514_Putative_TrmH_family_tRNA/rRNA_methyltransferase,23S_rRNA_(guanosine-2'-O-)-methyltransferase,RNA_me	RbromiL2-63
Org5_Gene1471	accessory	927	[RbromiL2-63]L2-63_01515_smf_hypothetical_protein,hypothetical_protein,DNA_protecting_protein_DprA,DNA_recombination-mediator_protein_A_158	RbromiL2-63
Org5_Gene1476	accessory	1574	[RbromiL2-63]L2-63_01520_hemN_1_Oxygen-independent_coproporphyrinogen-III_oxidase,coproporphyrinogen_III_oxidase,histone_acetyltransferase,_E	RbromiL2-63
Org5_Gene148	accessory	3314	[RbromiL2-63]L2-63_00151_L2-63_00151_hypothetical_protein_171706:172143_reverse_MW:15739	RbromiL2-63
Org5_Gene1481	accessory	2681	[RbromiL2-63]L2-63_01525_nadD_Probable_nicotinate-nucleotide_adenylyltransferase,nicotinic_acid_mononucleotide_adenylyltransferase,nicotinate_(ni	RbromiL2-63
Org5_Gene1482	accessory	2743	[RbromiL2-63]L2-63_01526_L2-63_01526_putative_nicotinate-nucleotide_adenylyltransferase,Predicted_HD_superfamily_hydrolase,putative_HD_superfa	RbromiL2-63
Org5_Gene1487	accessory	1242	[RbromiL2-63]L2-63_01531_L2-63_01531_Uncharacterized_peptidase_SA1530,aminopeptidase,3-dehydroquinate_dehydratase_II,methionine_aminopepti	RbromiL2-63
Org5_Gene1489	accessory	3110	[RbromiL2-63]L2-63_01533_L2-63_01533_hypothetical_protein_1609411:1609896_forward_MW:18305	RbromiL2-63
Org5_Gene149	accessory	545	[RbromiL2-63]L2-63_00152_L2-63_00152_Iron_hydrogenase_1,formate_dehydrogenase-H_ferredoxin_subunit,Iron_only_hydrogenase_large_subunit,_C-t	RbromiL2-63
Org5_Gene1490	accessory	2532	[RbromiL2-63]L2-63_01534_L2-63_01534_sporulation_protein_YunB,Sporulation_protein_YunB_(Spo_YunB)_1609947:1610600_forward_MW:24285	RbromiL2-63
Org5_Gene1494	accessory	229	[RbromiL2-63]L2-63_01538_mutL_DNA_mismatch_repair_protein_mutL,DNA_mismatch_repair_protein,DNA_mismatch_repair_enzyme_(predicted_ATPas	RbromiL2-63
Org5_Gene1495	accessory	1550	[RbromiL2-63]L2-63_01539_miaA_tRNA_dimethylallyltransferase,tRNA_delta(2)-isopentenylpyrophosphate_transferase,tRNA_dimethylallyltransferase,IPP	RbromiL2-63
Org5_Gene1496	accessory	877	[RbromiL2-63]L2-63_01540_L2-63_01540_putative_membrane_fusion_protein,HlyD_family_secretion_protein_1618233:1619516_forward_MW:47199	RbromiL2-63
Org5_Gene1499	accessory	2147	[RbromiL2-63]L2-63_01543_L2-63_01543_photosystem_II_S4_domain_protein_1620677:1621426_forward_MW:28151	RbromiL2-63
Org5_Gene15	accessory	2357	[RbromiL2-63]L2-63_00015_yetF_1_hypothetical_protein,Protein_of_unknown_function_(DUF421)_20098:20802_forward_MW:26608	RbromiL2-63
Org5_Gene150	accessory	2134	[RbromiL2-63]L2-63_00153_L2-63_00153_vancomycin_high_temperature_exclusion_protein,DUF218_domain_174060:174830_forward_MW:28062	RbromiL2-63
Org5_Gene1500	accessory	2446	[RbromiL2-63]L2-63_01544_divIVA_Minicell-associated_protein_DivIVA,DivIVA_domain,DivIVA_protein_1621440:1622114_forward_MW:25064	RbromiL2-63
Org5_Gene1505	accessory	2568	[RbromiL2-63]L2-63_01549_sipP_Signal_peptidase_I_P,signal_peptidase_I,Type_IV_secretory_pathway,_protease_TraF,signal_peptidase_I,Peptidase_S26_	RbromiL2-63
Org5_Gene1506	accessory	2785	[RbromiL2-63]L2-63_01550_sipT_Signal_peptidase_I_T,signal_peptidase_I,Type_IV_secretory_pathway,_protease_TraF,signal_peptidase_I,Peptidase_S24-	RbromiL2-63
Org5_Gene1509	accessory	3585	[RbromiL2-63]L2-63_01553_L2-63_01553_hypothetical_protein,hypothetical_protein,conserved_hypothetical_protein,Uncharacterised_protein_family_UP	RbromiL2-63
Org5_Gene151	accessory	3996	[RbromiL2-63]L2-63_00154_L2-63_00154_hypothetical_protein_174775:175026_reverse_MW:9762	RbromiL2-63
Org5_Gene1510	accessory	1578	[RbromiL2-63]L2-63_01554_L2-63_01554_Membrane_dipeptidase_(Peptidase_family_M19)_1630234:1631172_forward_MW:35598	RbromiL2-63
Org5_Gene152	accessory	4185	[RbromiL2-63]L2-63_00155_immR_3_HTH-type_transcriptional_regulator_immR,Predicted_transcriptional_regulator,Helix-turn-helix_175095:175304_reve	RbromiL2-63
Org5_Gene1522	accessory	2396	[RbromiL2-63]L2-63_01566_L2-63_01566_hypothetical_protein_1645239:1645934_reverse_MW:25833	RbromiL2-63
Org5_Gene1524	accessory	2333	[RbromiL2-63]L2-63_01568_amiC_N-acetylmuramoyl-L-alanine_amidase_AmiC_precursor,N-acetylmuramoyl-l-alanine_amidase_I,N-acetylmuramoyl-L-ala	RbromiL2-63
Org5_Gene1526	accessory	679	[RbromiL2-63]L2-63_01570_purF_1_Amidophosphoribosyltransferase_precursor,amidophosphoribosyltransferase,Glutamine_phosphoribosylpyrophospha	RbromiL2-63
Org5_Gene1527	accessory	114	[RbromiL2-63]L2-63_01571_ykoD_Putative_HMP/thiamine_import_ATP-binding_protein_YkoD,cobalt_transporter_ATP-binding_subunit,ABC-type_spermi	RbromiL2-63
Org5_Gene1528	accessory	1732	[RbromiL2-63]L2-63_01572_ecfT_1_Energy-coupling_factor_transporter_transmembrane_protein_EcfT,ABC-type_cobalt_transport_system,_permease_co	RbromiL2-63
Org5_Gene1529	accessory	1565	[RbromiL2-63]L2-63_01573_L2-63_01573_hypothetical_protein_1654292:1655221_reverse_MW:34186	RbromiL2-63
Org5_Gene153	accessory	3028	[RbromiL2-63]L2-63_00156_L2-63_00156_hypothetical_protein_175552:176061_forward_MW:18696	RbromiL2-63
Org5_Gene1530	accessory	326	[RbromiL2-63]L2-63_01574_L2-63_01574_hypothetical_protein_1655248:1657137_reverse_MW:67705	RbromiL2-63
Org5_Gene1531	accessory	4072	[RbromiL2-63]L2-63_01575_L2-63_01575_hypothetical_protein_1657620:1657856_forward_MW:8677	RbromiL2-63
Org5_Gene1533	accessory	2009	[RbromiL2-63]L2-63_01577_L2-63_01577_ribonuclease_Z,Low-affinity_cAMP_phosphodiesterase,ribonuclease_Z,Beta-lactamase_superfamily_domain_16	RbromiL2-63
Org5_Gene1536	accessory	686	[RbromiL2-63]L2-63_01580_speA_Arginine_decarboxylase,lysine_decarboxylase_LdcC,Serine-pyruvate_aminotransferase/archaeal_aspartate_aminotransf	RbromiL2-63
Org5_Gene1538	accessory	1229	[RbromiL2-63]L2-63_01582_hemN_2_Oxygen-independent_coproporphyrinogen-III_oxidase,coproporphyrinogen_III_oxidase,Putative_heme_iron_utilizati	RbromiL2-63
Org5_Gene1539	accessory	1690	[RbromiL2-63]L2-63_01583_L2-63_01583_hypothetical_protein_1666744:1667643_reverse_MW:32982	RbromiL2-63
Org5_Gene1540	accessory	2343	[RbromiL2-63]L2-63_01584_L2-63_01584_hypothetical_protein_1667896:1668603_reverse_MW:26094	RbromiL2-63
Org5_Gene1541	accessory	3307	[RbromiL2-63]L2-63_01585_cwlJ_Cell_wall_hydrolase_CwlJ,Cell_wall_hydrolyses_involved_in_spore_germination,spore_cortex-lytic_enzyme,Cell_Wall_Hy	RbromiL2-63
Org5_Gene1542	accessory	1996	[RbromiL2-63]L2-63_01586_L2-63_01586_hypothetical_protein_1669256:1669573_reverse_MW:11863	RbromiL2-63
Org5_Gene1543	accessory	4223	[RbromiL2-63]L2-63_01587_L2-63_01587_hypothetical_protein_1669654:1669854_reverse_MW:7741	RbromiL2-63
Org5_Gene1544	accessory	655	[RbromiL2-63]L2-63_01589_L2-63_01589_Divergent_AAA_domain_1670899:1672326_reverse_MW:53620	RbromiL2-63
Org5_Gene1545	accessory	410	[RbromiL2-63]L2-63_01590_L2-63_01590_Putative_multidrug_export_ATP-binding/permease_protein_SAV1866,cyclic_beta-1,2-glucan_ABC_transporter,A	RbromiL2-63
Org5_Gene1546	accessory	377	[RbromiL2-63]L2-63_01591_irtA_Iron_import_ATP-binding/permease_protein_IrtA,cyclic_beta-1,2-glucan_ABC_transporter,ABC-type_bacteriocin/lantibio	RbromiL2-63
Org5_Gene1547	accessory	2626	[RbromiL2-63]L2-63_01592_L2-63_01592_hypothetical_protein_1676063:1676479_reverse_MW:16021	RbromiL2-63
Org5_Gene1549	accessory	3319	[RbromiL2-63]L2-63_01594_L2-63_01594_ribosomal-protein-alanine_acetyltransferase,Acetyltransferase_(GNAT)_family_1676701:1677135_reverse_MW:	RbromiL2-63
Org5_Gene155	accessory	3763	[RbromiL2-63]L2-63_00158_celA_Endoglucanase_A_precursor_176960:177280_forward_MW:11210	RbromiL2-63
Org5_Gene1552	accessory	2337	[RbromiL2-63]L2-63_01597_ydiC_hypothetical_protein,UGMP_family_protein,universal_bacterial_protein_YeaZ,Glycoprotease_family_1678369:1679079_	RbromiL2-63
Org5_Gene1555	accessory	1095	[RbromiL2-63]L2-63_01600_ftsW_3_Cell_division_protein_FtsW,cell_wall_shape-determining_protein,rod_shape-determining_protein_RodA,Cell_cycle_p	RbromiL2-63
Org5_Gene1556	accessory	925	[RbromiL2-63]L2-63_01601_L2-63_01601_Uncharacterized_protein_conserved_in_bacteria,YbbR-like_protein_1682535:1683794_reverse_MW:45148	RbromiL2-63
Org5_Gene1558	accessory	1106	[RbromiL2-63]L2-63_01603_csd_2_Probable_cysteine_desulfurase,bifunctional_cysteine_desulfurase/selenocysteine_lyase,Serine-pyruvate_aminotransfe	RbromiL2-63
Org5_Gene1559	accessory	4103	[RbromiL2-63]L2-63_01604_L2-63_01604_Protein_of_unknown_function_(DUF3343)_1685920:1686147_reverse_MW:8288	RbromiL2-63
Org5_Gene1565	accessory	3360	[RbromiL2-63]L2-63_01610_yajC_hypothetical_protein,preprotein_translocase_subunit_YajC,preprotein_translocase,_YajC_subunit,Preprotein_translocase	RbromiL2-63
Org5_Gene1566	accessory	3480	[RbromiL2-63]L2-63_01611_L2-63_01611_putative_membrane_protein,Protein_of_unknown_function_(DUF3792)_1692095:1692487_forward_MW:13380	RbromiL2-63
Org5_Gene1567	accessory	150	[RbromiL2-63]L2-63_01612_prkC_4_Serine/threonine-protein_kinase_PrkC,Predicted_ATPase,Protein_kinase_domain_1692719:1695127_forward_MW:88	RbromiL2-63
Org5_Gene1568	accessory	414	[RbromiL2-63]L2-63_01613_L2-63_01613_hypothetical_protein_1695163:1696884_forward_MW:66239	RbromiL2-63
Org5_Gene1569	accessory	4007	[RbromiL2-63]L2-63_01614_L2-63_01614_hypothetical_protein_1697697:1697951_forward_MW:9637	RbromiL2-63
Org5_Gene1570	accessory	3911	[RbromiL2-63]L2-63_01615_yafQ_2_mRNA_interferase_YafQ,addiction_module_toxin,_RelE/StbE_family,Plasmid_stabilisation_system_protein_1697944:	RbromiL2-63
Org5_Gene1571	accessory	301	[RbromiL2-63]L2-63_01616_L2-63_01616_Uncharacterized_protein_conserved_in_bacteria,Bacterial_Ig-like_domain_(group_2)_1698314:1700233_revers	RbromiL2-63
Org5_Gene1572	accessory	1779	[RbromiL2-63]L2-63_01617_rluD_2_Ribosomal_large_subunit_pseudouridine_synthase_D,23S_rRNA_pseudouridine_synthase_D,pseudouridine_synthase,	RbromiL2-63
Org5_Gene1573	accessory	3938	[RbromiL2-63]L2-63_01618_L2-63_01618_Protein_of_unknown_function_(DUF1294)_1701937:1702209_reverse_MW:10703	RbromiL2-63
Org5_Gene1574	accessory	965	[RbromiL2-63]L2-63_01619_L2-63_01619_Uncharacterized_protein_conserved_in_bacteria,Uncharacterised_BCR,_COG1649_1702233:1703465_forward_M	RbromiL2-63
Org5_Gene1576	accessory	4057	[RbromiL2-63]L2-63_01621_L2-63_01621_hypothetical_protein_1704825:1705064_forward_MW:8916	RbromiL2-63
Org5_Gene1585	accessory	827	[RbromiL2-63]L2-63_01630_yhhT_hypothetical_protein,pheromone_autoinducer_2_transporter,sporulation_integral_membrane_protein_YtvI,Domain_of_	RbromiL2-63
Org5_Gene1588	accessory	708	[RbromiL2-63]L2-63_01633_rasP_Zinc_metalloprotease_rasP,zinc_metallopeptidase_RseP,Translation_elongation_factor_Ts,RIP_metalloprotease_RseP,Pe	RbromiL2-63
Org5_Gene1590	accessory	1939	[RbromiL2-63]L2-63_01635_cdsA_Phosphatidate_cytidylyltransferase,CDP-diglyceride_synthase,Predicted_CDP-diglyceride_synthetase/phosphatidate_cyt	RbromiL2-63
Org5_Gene1594	accessory	2091	[RbromiL2-63]L2-63_01640_L2-63_01640_hypothetical_protein_1724175:1724954_forward_MW:27855	RbromiL2-63
Org5_Gene1595	accessory	1687	[RbromiL2-63]L2-63_01641_L2-63_01641_radical_SAM_protein,Radical_SAM_superfamily_1724967:1725869_reverse_MW:33812	RbromiL2-63
Org5_Gene1596	accessory	2429	[RbromiL2-63]L2-63_01642_L2-63_01642_hypothetical_protein_1726085:1726768_reverse_MW:26134	RbromiL2-63
Org5_Gene1599	accessory	3057	[RbromiL2-63]L2-63_01645_L2-63_01645_Zn-dependent_proteases,Peptidase_family_M50_1729280:1729780_reverse_MW:18991	RbromiL2-63
Org5_Gene1600	accessory	3520	[RbromiL2-63]L2-63_01646_L2-63_01646_hypothetical_protein_1729863:1730243_reverse_MW:14073	RbromiL2-63
Org5_Gene1602	accessory	3182	[RbromiL2-63]L2-63_01648_L2-63_01648_Predicted_integral_membrane_protein,VanZ_like_family_1731709:1732179_reverse_MW:17623	RbromiL2-63
Org5_Gene1603	accessory	3152	[RbromiL2-63]L2-63_01649_L2-63_01649_Predicted_membrane_protein,Putative_small_multi-drug_export_protein_1732376:1732852_forward_MW:169	RbromiL2-63
Org5_Gene1605	accessory	966	[RbromiL2-63]L2-63_01651_L2-63_01651_hypothetical_protein,flavoprotein,_HI0933_family,HI0933-like_protein_1733358:1734137_forward_MW:28214	RbromiL2-63
Org5_Gene1607	accessory	1672	[RbromiL2-63]L2-63_01653_L2-63_01653_UDP-glucose_4-epimerase,NAD_dependent_epimerase/dehydratase_family_1735793:1736695_forward_MW:35	RbromiL2-63
Org5_Gene1608	accessory	119	[RbromiL2-63]L2-63_01654_amyE_3_Alpha-amylase_precursor,cytoplasmic_alpha-amylase,malto-oligosyltrehalose_trehalohydrolase,Alpha_amylase,_cata	RbromiL2-63
Org5_Gene161	accessory	3989	[RbromiL2-63]L2-63_00164_secE_Preprotein_translocase_subunit_secE,preprotein_translocase_subunit_SecE,preprotein_translocase,_SecE_subunit,SecE/	RbromiL2-63
Org5_Gene1611	accessory	560	[RbromiL2-63]L2-63_01658_artQ_Arginine_transport_system_permease_protein_ArtQ,amino_acid_ABC_transporter_permease,ABC-type_amino_acid_tra	RbromiL2-63
Org5_Gene1613	accessory	768	[RbromiL2-63]L2-63_01660_cca_CCA-adding_enzyme,tRNA_CCA-pyrophosphorylase,Uncharacterized_conserved_protein,CCA_tRNA_nucleotidyltransfera	RbromiL2-63
Org5_Gene1614	accessory	167	[RbromiL2-63]L2-63_01661_L2-63_01661_Calcium-transporting_ATPase_lmo0841,magnesium-transporting_ATPase_MgtA,Uncharacterized_protein_conse	RbromiL2-63
Org5_Gene1615	accessory	1382	[RbromiL2-63]L2-63_01662_spoIVB_SpoIVB_peptidase_precursor,Predicted_secreted_protein_containing_a_PDZ_domain,stage_IV_sporulation_protein_	RbromiL2-63
Org5_Gene1624	accessory	571	[RbromiL2-63]L2-63_01671_L2-63_01671_Predicted_membrane_protein,Protein_of_unknown_function_(DUF1113)_1756912:1758423_reverse_MW:5741	RbromiL2-63
Org5_Gene1626	accessory	3295	[RbromiL2-63]L2-63_01673_L2-63_01673_NfeD-like_C-terminal,_partner-binding_1759419:1759859_reverse_MW:16076	RbromiL2-63
Org5_Gene1627	accessory	1770	[RbromiL2-63]L2-63_01674_ybhF_Uncharacterized_ABC_transporter_ATP-binding_protein_YbhF,nodulation_ABC_transporter_NodI,ABC-type_Na__transp	RbromiL2-63
Org5_Gene1628	accessory	190	[RbromiL2-63]L2-63_01675_L2-63_01675_ABC-type_transport_system_involved_in_multi-copper_enzyme_maturation,_permease_component,ABC-2_fam	RbromiL2-63
Org5_Gene1629	accessory	921	[RbromiL2-63]L2-63_01676_L2-63_01676_hypothetical_protein_1763223:1764485_reverse_MW:47986	RbromiL2-63
Org5_Gene1630	accessory	3270	[RbromiL2-63]L2-63_01677_L2-63_01677_hypothetical_protein_1764508:1764954_reverse_MW:16170	RbromiL2-63
Org5_Gene1631	accessory	1114	[RbromiL2-63]L2-63_01678_L2-63_01678_Fucose_4-O-acetylase_and_related_acetyltransferases,Acyltransferase_family_1765304:1766467_reverse_MW:	RbromiL2-63
Org5_Gene1632	accessory	810	[RbromiL2-63]L2-63_01679_L2-63_01679_hypothetical_protein_1766844:1768169_reverse_MW:49853	RbromiL2-63
Org5_Gene1633	accessory	838	[RbromiL2-63]L2-63_01680_L2-63_01680_sensory_histidine_kinase_DcuS,Histidine_kinase-,_DNA_gyrase_B-,_and_HSP90-like_ATPase_1768214:1769527_	RbromiL2-63
Org5_Gene1634	accessory	2309	[RbromiL2-63]L2-63_01681_lytR_5_Sensory_transduction_protein_lytR,putative_two-component_response-regulatory_protein_YehT,Response_regulator_	RbromiL2-63
Org5_Gene1638	accessory	888	[RbromiL2-63]L2-63_01685_galK_Galactokinase,galactokinase,Galactokinase,galactokinase,Galactokinase_galactose-binding_signature_1774382:1775662_	RbromiL2-63

Org5_Gene1639	accessory	604	[RbromiL2-63]L2-63_01686_melB_Thiomethylgalactoside_permease_II,melibiose:sodium_symporter,sugar_(Glycoside-Pentoside-Hexuronide)_transporter	RbromiL2-63
Org5_Gene1640	accessory	1395	[RbromiL2-63]L2-63_01687_ccpA_Glucose-resistance_amylase_regulator,DNA-binding_transcriptional_repressor_EbgR,ATP-dependent_protease_HslVU_(	RbromiL2-63
Org5_Gene1641	accessory	2113	[RbromiL2-63]L2-63_01688_L2-63_01688_Sulfite_exporter_TauE/SafE_1778971:1779750_reverse_MW:27311	RbromiL2-63
Org5_Gene1642	accessory	1577	[RbromiL2-63]L2-63_01689_L2-63_01689_hypothetical_protein_1779793:1780719_reverse_MW:34015	RbromiL2-63
Org5_Gene1643	accessory	2997	[RbromiL2-63]L2-63_01690_L2-63_01690_dephospho-CoA_kinase/protein_folding_accessory_domain-containing_protein,Dephospho-CoA_kinase,Unchara	RbromiL2-63
Org5_Gene1644	accessory	3441	[RbromiL2-63]L2-63_01691_L2-63_01691_Transposase_and_inactivated_derivatives,Transposase_IS200_like_1781691:1782095_reverse_MW:15616	RbromiL2-63
Org5_Gene1650	accessory	3123	[RbromiL2-63]L2-63_01697_L2-63_01697_hypothetical_protein_1789184:1789669_reverse_MW:18220	RbromiL2-63
Org5_Gene1651	accessory	3381	[RbromiL2-63]L2-63_01698_L2-63_01698_hypothetical_protein_1789712:1790131_reverse_MW:15986	RbromiL2-63
Org5_Gene1652	accessory	3053	[RbromiL2-63]L2-63_01699_L2-63_01699_hypothetical_protein_1790382:1790882_reverse_MW:18326	RbromiL2-63
Org5_Gene1653	accessory	4050	[RbromiL2-63]L2-63_01700_L2-63_01700_hypothetical_protein_1791296:1791538_forward_MW:9255	RbromiL2-63
Org5_Gene1654	accessory	1957	[RbromiL2-63]L2-63_01701_soj_3_Sporulation_initiation_inhibitor_protein_soj,plasmid-partitioning_protein_RepA,Septum_formation_inhibitor-activating	RbromiL2-63
Org5_Gene1655	accessory	1576	[RbromiL2-63]L2-63_01702_parB_3_Probable_chromosome-partitioning_protein_parB,plasmid_partitioning_protein,ParB/RepB/Spo0J_family_partition_p	RbromiL2-63
Org5_Gene1656	accessory	2597	[RbromiL2-63]L2-63_01703_L2-63_01703_hypothetical_protein_1793564:1794196_forward_MW:22719	RbromiL2-63
Org5_Gene1657	accessory	24	[RbromiL2-63]L2-63_01704_L2-63_01704_Predicted_outer_membrane_protein,Cna_protein_B-type_domain_1794264:1798505_forward_MW:153793	RbromiL2-63
Org5_Gene1658	accessory	3685	[RbromiL2-63]L2-63_01705_L2-63_01705_hypothetical_protein_1798660:1798995_forward_MW:13010	RbromiL2-63
Org5_Gene1659	accessory	1581	[RbromiL2-63]L2-63_01706_L2-63_01706_hypothetical_protein_1798992:1799930_forward_MW:35978	RbromiL2-63
Org5_Gene1660	accessory	2890	[RbromiL2-63]L2-63_01707_L2-63_01707_hypothetical_protein_1799927:1800472_forward_MW:21247	RbromiL2-63
Org5_Gene1661	accessory	2428	[RbromiL2-63]L2-63_01708_L2-63_01708_Antirestriction_protein,Antirestriction_protein_(ArdA)_1800484:1801167_forward_MW:25947	RbromiL2-63
Org5_Gene1662	accessory	3628	[RbromiL2-63]L2-63_01709_L2-63_01709_anaerobic_benzoate_catabolism_transcriptional_regulator,Predicted_transcriptional_regulator,transcriptional_r	RbromiL2-63
Org5_Gene1663	accessory	3208	[RbromiL2-63]L2-63_01710_L2-63_01710_hypothetical_protein_1801724:1802188_forward_MW:17763	RbromiL2-63
Org5_Gene1664	accessory	3758	[RbromiL2-63]L2-63_01711_L2-63_01711_hypothetical_protein_1802260:1802583_forward_MW:12541	RbromiL2-63
Org5_Gene1665	accessory	1331	[RbromiL2-63]L2-63_01712_L2-63_01712_hypothetical_protein,Fic/DOC_family_1802744:1803790_forward_MW:39245	RbromiL2-63
Org5_Gene1666	accessory	2316	[RbromiL2-63]L2-63_01713_L2-63_01713_hypothetical_protein_1803802:1804518_forward_MW:27234	RbromiL2-63
Org5_Gene1667	accessory	1499	[RbromiL2-63]L2-63_01714_L2-63_01714_DNA_primase_(bacterial_type)_1804602:1805570_forward_MW:36139	RbromiL2-63
Org5_Gene1668	accessory	3729	[RbromiL2-63]L2-63_01715_L2-63_01715_hypothetical_protein_1805567:1805896_forward_MW:11967	RbromiL2-63
Org5_Gene1669	accessory	3443	[RbromiL2-63]L2-63_01716_L2-63_01716_Domain_of_unknown_function_(DUF3846)_1805886:1806287_forward_MW:15141	RbromiL2-63
Org5_Gene1670	accessory	4302	[RbromiL2-63]L2-63_01717_L2-63_01717_hypothetical_protein_1806287:1806469_forward_MW:6621	RbromiL2-63
Org5_Gene1671	accessory	601	[RbromiL2-63]L2-63_01718_hhaIM_1_Modification_methylase_HhaI,DNA_cytosine_methylase,DNA_(cytosine-5-)-methyltransferase,C-5_cytosine-specific_	RbromiL2-63
Org5_Gene1672	accessory	3722	[RbromiL2-63]L2-63_01719_L2-63_01719_Bacterial_mobilisation_protein_(MobC)_1807937:1808266_forward_MW:12694	RbromiL2-63
Org5_Gene1673	accessory	664	[RbromiL2-63]L2-63_01720_L2-63_01720_Relaxase/Mobilisation_nuclease_domain_1808271:1809692_forward_MW:55244	RbromiL2-63
Org5_Gene1674	accessory	1961	[RbromiL2-63]L2-63_01721_L2-63_01721_Protein_of_unknown_function_(DUF3801)_1809676:1810500_forward_MW:30533	RbromiL2-63
Org5_Gene1675	accessory	1698	[RbromiL2-63]L2-63_01722_L2-63_01722_hypothetical_protein_1810504:1811403_forward_MW:33950	RbromiL2-63
Org5_Gene1676	accessory	4255	[RbromiL2-63]L2-63_01723_L2-63_01723_hypothetical_protein_1811406:1811600_forward_MW:7522	RbromiL2-63
Org5_Gene1679	accessory	4158	[RbromiL2-63]L2-63_01726_L2-63_01726_hypothetical_protein_1813754:1813969_forward_MW:8168	RbromiL2-63
Org5_Gene1680	accessory	2784	[RbromiL2-63]L2-63_01727_L2-63_01727_Transcriptional_activator,_adenine-specific_DNA_methyltransferase,MT-A70_1814040:1814615_forward_MW:2	RbromiL2-63
Org5_Gene1681	accessory	1907	[RbromiL2-63]L2-63_01728_L2-63_01728_hypothetical_protein_1814602:1815441_forward_MW:30226	RbromiL2-63
Org5_Gene1682	accessory	1892	[RbromiL2-63]L2-63_01729_L2-63_01729_Domain_of_unknown_function_(DUF3560)_1815501:1816343_forward_MW:32566	RbromiL2-63
Org5_Gene1683	accessory	3614	[RbromiL2-63]L2-63_01730_L2-63_01730_PrgI_family_protein_1816358:1816714_forward_MW:13562	RbromiL2-63
Org5_Gene1684	accessory	177	[RbromiL2-63]L2-63_01731_L2-63_01731_conjugal_transfer_ATP-binding_protein_TraC,Type_IV_secretory_pathway,_VirB4_components,type-IV_secretio	RbromiL2-63
Org5_Gene1685	accessory	2051	[RbromiL2-63]L2-63_01732_L2-63_01732_Sortase_(surface_protein_transpeptidase),sortase,_SrtB_family,Sortase_family_1819081:1819878_forward_MW	RbromiL2-63
Org5_Gene1686	accessory	401	[RbromiL2-63]L2-63_01733_L2-63_01733_Uncharacterized_conserved_protein,CHAP_domain_1819871:1821616_forward_MW:62951	RbromiL2-63
Org5_Gene1687	accessory	4159	[RbromiL2-63]L2-63_01734_L2-63_01734_Predicted_transcriptional_regulator_1821753:1821968_forward_MW:8020	RbromiL2-63
Org5_Gene1688	accessory	2347	[RbromiL2-63]L2-63_01735_walR_Transcriptional_regulatory_protein_walR,DNA-binding_response_regulator_CreB,Response_regulator_of_citrate/malate	RbromiL2-63
Org5_Gene1689	accessory	1176	[RbromiL2-63]L2-63_01736_phoR_4_Alkaline_phosphatase_synthesis_sensor_protein_phoR,sensory_histidine_kinase_CreC,Predicted_periplasmic_ligand-	RbromiL2-63
Org5_Gene169	accessory	2099	[RbromiL2-63]L2-63_00173_xynZ_Endo-1,4-beta-xylanase_Z_precursor,enterobactin/ferric_enterobactin_esterase,Predicted_hydrolase_of_the_alpha/beta	RbromiL2-63
Org5_Gene1690	accessory	2926	[RbromiL2-63]L2-63_01737_L2-63_01737_D-alanyl-D-alanine_carboxypeptidase,D-alanyl-D-alanine_carboxypeptidase_1824025:1824561_forward_MW:20	RbromiL2-63
Org5_Gene1691	accessory	1960	[RbromiL2-63]L2-63_01738_L2-63_01738_Uncharacterized_vancomycin_resistance_protein,VanW_like_protein_1824574:1825398_forward_MW:31976	RbromiL2-63
Org5_Gene1692	accessory	1319	[RbromiL2-63]L2-63_01739_vanB_Vancomycin_B-type_resistance_protein_VanB,D-alanyl-alanine_synthetase_A,Formate-dependent_phosphoribosylglycir	RbromiL2-63
Org5_Gene1693	accessory	2108	[RbromiL2-63]L2-63_01740_L2-63_01740_D-alanyl-D-alanine_carboxypeptidase,D-alanyl-D-alanine_carboxypeptidase_1826446:1827228_forward_MW:30	RbromiL2-63
Org5_Gene1694	accessory	230	[RbromiL2-63]L2-63_01741_vanT_Serine/alanine_racemase,alanine_racemase,Uncharacterized_protein_conserved_in_bacteria,alanine_racemase,Alanine_	RbromiL2-63
Org5_Gene1695	accessory	3714	[RbromiL2-63]L2-63_01742_L2-63_01742_anaerobic_benzoate_catabolism_transcriptional_regulator,Predicted_transcriptional_regulator,conserved_hypot	RbromiL2-63
Org5_Gene1696	accessory	3188	[RbromiL2-63]L2-63_01743_L2-63_01743_RNA_polymerase_sigma_factor,RNA_polymerase_sigma_factor,_sigma-70_family,Sigma-70,_region_4_1830252:	RbromiL2-63
Org5_Gene1697	accessory	2980	[RbromiL2-63]L2-63_01744_ndoA_2_mRNA_interferase_EndoA,PemK-like_protein_1831358:1831882_forward_MW:19558	RbromiL2-63
Org5_Gene1698	accessory	4141	[RbromiL2-63]L2-63_01745_L2-63_01745_DNA_binding_domain,_excisionase_family,Helix-turn-helix_domain_1831894:1832112_forward_MW:8340	RbromiL2-63
Org5_Gene1699	accessory	1047	[RbromiL2-63]L2-63_01746_xerC_Tyrosine_recombinase_XerC,site-specific_tyrosine_recombinase_XerC,Site-specific_recombinase_XerD,tyrosine_recombi	RbromiL2-63
Org5_Gene17	accessory	1899	[RbromiL2-63]L2-63_00017_immR_1_HTH-type_transcriptional_regulator_immR,transcriptional_repressor_DicA,transcriptional_regulator,_y4mF_family,He	RbromiL2-63
Org5_Gene170	accessory	2969	[RbromiL2-63]L2-63_00174_paiA_Protease_synthase_and_sporulation_negative_regulatory_protein_PAI_1,ribosomal-protein-alanine_acetyltransferase,A	RbromiL2-63
Org5_Gene1700	accessory	1309	[RbromiL2-63]L2-63_01748_L2-63_01748_CAAX_amino_terminal_protease_self-_immunity_1834111:1835166_forward_MW:38788	RbromiL2-63
Org5_Gene1703	accessory	2123	[RbromiL2-63]L2-63_01751_L2-63_01751_Predicted_acetyltransferase,ribosomal-protein-alanine_acetyltransferase,FR47-like_protein_1836948:1837721_r	RbromiL2-63
Org5_Gene1704	accessory	1322	[RbromiL2-63]L2-63_01752_ysdC_1_Putative_aminopeptidase_ysdC,exoaminopeptidase,glutamyl_aminopeptidase,M42_glutamyl_aminopeptidase_18377	RbromiL2-63
Org5_Gene1705	accessory	1370	[RbromiL2-63]L2-63_01753_ysdC_2_Putative_aminopeptidase_ysdC,exoaminopeptidase,glutamyl_aminopeptidase,M42_glutamyl_aminopeptidase_1838	RbromiL2-63
Org5_Gene1707	accessory	739	[RbromiL2-63]L2-63_01755_gabR_HTH-type_transcriptional_regulatory_protein_gabR,transcriptional_regulatory_protein_PtsJ,Alanine-alpha-ketoisovalera	RbromiL2-63
Org5_Gene1708	accessory	1814	[RbromiL2-63]L2-63_01756_L2-63_01756_hypothetical_protein_1842753:1843619_reverse_MW:31520	RbromiL2-63
Org5_Gene1709	accessory	1008	[RbromiL2-63]L2-63_01757_pbpE_Penicillin-binding_protein_E,beta-lactamase/D-alanine_carboxypeptidase,Uncharacterized_protein_conserved_in_bacte	RbromiL2-63
Org5_Gene1712	accessory	3200	[RbromiL2-63]L2-63_01760_L2-63_01760_hypothetical_protein_1847239:1847703_reverse_MW:17038	RbromiL2-63
Org5_Gene1713	accessory	1458	[RbromiL2-63]L2-63_01763_dnaC_2_DNA_replication_protein_dnaC,DNA_replication_protein_DnaC,DNA_replication_protein,chromosomal_replication_in	RbromiL2-63
Org5_Gene1714	accessory	1231	[RbromiL2-63]L2-63_01764_L2-63_01764_DnaD_domain_protein,Replication_initiation_and_membrane_attachment_1849100:1850197_reverse_MW:411	RbromiL2-63
Org5_Gene1715	accessory	4008	[RbromiL2-63]L2-63_01765_L2-63_01765_uncharacterized_protein,TSCPD_domain_1850470:1850724_forward_MW:9035	RbromiL2-63
Org5_Gene1716	accessory	4412	[RbromiL2-63]L2-63_01766_L2-63_01766_hypothetical_protein_1850710:1850844_reverse_MW:5058	RbromiL2-63
Org5_Gene1719	accessory	1338	[RbromiL2-63]L2-63_01769_L2-63_01769_Protein_of_unknown_function_(DUF2628)_1853489:1854532_forward_MW:38430	RbromiL2-63
Org5_Gene1721	accessory	1969	[RbromiL2-63]L2-63_01771_polX_DNA_polymerase/3'-5'_exonuclease_PolX,histidinol-phosphatase,Histidinol_phosphatase_and_related_hydrolases_of_th	RbromiL2-63
Org5_Gene1724	accessory	3794	[RbromiL2-63]L2-63_01774_L2-63_01774_hypothetical_protein_1858279:1858590_forward_MW:12113	RbromiL2-63
Org5_Gene1726	accessory	305	[RbromiL2-63]L2-63_01776_L2-63_01776_V-type_ATP_synthase_subunit_I,Nucleoside_diphosphate_kinase,V-type_ATPase_116kDa_subunit_family_1859	RbromiL2-63
Org5_Gene1729	accessory	2707	[RbromiL2-63]L2-63_01779_L2-63_01779_ATP_synthase_(E/31_kDa)_subunit_1862462:1863058_forward_MW:22525	RbromiL2-63
Org5_Gene173	accessory	2375	[RbromiL2-63]L2-63_00177_lolD_1_Lipoprotein-releasing_system_ATP-binding_protein_LolD,DL-methionine_transporter_ATP-binding_subunit,Predicted_	RbromiL2-63
Org5_Gene1733	accessory	3755	[RbromiL2-63]L2-63_01783_L2-63_01783_hypothetical_protein_1867056:1867379_reverse_MW:11754	RbromiL2-63
Org5_Gene1735	accessory	392	[RbromiL2-63]L2-63_01785_dxs_2_1-deoxy-D-xylulose-5-phosphate_synthase,1-deoxy-D-xylulose-5-phosphate_synthase,Transketolase,_C-terminal_subun	RbromiL2-63
Org5_Gene1736	accessory	1087	[RbromiL2-63]L2-63_01790_L2-63_01790_Uncharacterized_protein_conserved_in_bacteria,CYTH_domain_1870310:1871482_reverse_MW:45208	RbromiL2-63
Org5_Gene1738	accessory	2323	[RbromiL2-63]L2-63_01792_L2-63_01792_hypothetical_protein_1872763:1873473_reverse_MW:26290	RbromiL2-63
Org5_Gene1739	accessory	1811	[RbromiL2-63]L2-63_01793_L2-63_01793_hypothetical_protein_1873529:1874395_reverse_MW:31598	RbromiL2-63
Org5_Gene174	accessory	57	[RbromiL2-63]L2-63_00178_lolE_Lipoprotein-releasing_system_transmembrane_protein_lolE,outer_membrane-specific_lipoprotein_transporter_subunit_	RbromiL2-63
Org5_Gene1740	accessory	4400	[RbromiL2-63]L2-63_01794_L2-63_01794_hypothetical_protein_1874471:1874614_reverse_MW:5340	RbromiL2-63
Org5_Gene1741	accessory	45	[RbromiL2-63]L2-63_01795_L2-63_01795_hypothetical_protein_1874636:1878226_reverse_MW:133274	RbromiL2-63
Org5_Gene1742	accessory	795	[RbromiL2-63]L2-63_01796_L2-63_01796_hypothetical_protein_1878219:1879550_reverse_MW:47052	RbromiL2-63
Org5_Gene1745	accessory	1421	[RbromiL2-63]L2-63_01799_L2-63_01799_ABC-type_uncharacterized_transport_system,_periplasmic_component,ABC_transporter_substrate_binding_pro	RbromiL2-63
Org5_Gene1747	accessory	995	[RbromiL2-63]L2-63_01801_xseA_Exodeoxyribonuclease_7_large_subunit,exodeoxyribonuclease_VII_large_subunit,exodeoxyribonuclease_VII,_large_subu	RbromiL2-63
Org5_Gene1749	accessory	3081	[RbromiL2-63]L2-63_01803_nusB_N_utilization_substance_protein_B_homolog,transcription_antitermination_protein_NusB,transcription_antiterminatior	RbromiL2-63
Org5_Gene1750	accessory	2903	[RbromiL2-63]L2-63_01804_L2-63_01804_SpoIIIAH-like_protein_1889250:1889792_reverse_MW:19553	RbromiL2-63
Org5_Gene1752	accessory	3177	[RbromiL2-63]L2-63_01806_L2-63_01806_hypothetical_protein_1890260:1890733_reverse_MW:17210	RbromiL2-63
Org5_Gene1753	accessory	1199	[RbromiL2-63]L2-63_01807_L2-63_01807_stage_III_sporulation_protein_AE,Stage_III_sporulation_protein_AE_(spore_III_AE)_1890730:1891848_reverse_	RbromiL2-63
Org5_Gene1754	accessory	3501	[RbromiL2-63]L2-63_01808_L2-63_01808_stage_III_sporulation_protein_AD,Stage_III_sporulation_protein_AC/AD_protein_family_1891858:1892247_reve	RbromiL2-63
Org5_Gene1756	accessory	3075	[RbromiL2-63]L2-63_01810_L2-63_01810_stage_III_sporulation_protein_SpoAB,stage_III_sporulation_protein_AB,Stage_III_sporulation_protein_AB_(spor	RbromiL2-63
Org5_Gene1757	accessory	1549	[RbromiL2-63]L2-63_01811_L2-63_01811_Uncharacterized_protein_conserved_in_bacteria,stage_III_sporulation_protein_AA_1892936:1893886_reverse_I	RbromiL2-63
Org5_Gene1758	accessory	3034	[RbromiL2-63]L2-63_01812_L2-63_01812_preprotein_translocase_subunit_SecD,protein-export_membrane_protein_SecD_1894002:1894490_reverse_MW	RbromiL2-63
Org5_Gene1761	accessory	1561	[RbromiL2-63]L2-63_01815_L2-63_01815_hypothetical_protein_1897263:1898210_reverse_MW:34197	RbromiL2-63
Org5_Gene1765	accessory	3277	[RbromiL2-63]L2-63_01819_L2-63_01819_Septum_formation_initiator_1900610:1901056_reverse_MW:17079	RbromiL2-63
Org5_Gene1770	accessory	345	[RbromiL2-63]L2-63_01824_ytgP_Probable_cell_division_protein_ytgP,stage_V_sporulation_protein_B,MatE_1903801:1905657_reverse_MW:66913	RbromiL2-63
Org5_Gene1771	accessory	3434	[RbromiL2-63]L2-63_01825_L2-63_01825_hypothetical_protein_1905682:1906077_reverse_MW:14398	RbromiL2-63
Org5_Gene1776	accessory	267	[RbromiL2-63]L2-63_01830_recJ_Single-stranded-DNA-specific_exonuclease_recJ,ssDNA_exonuclease_RecJ,Uncharacterized_protein_conserved_in_bacter	RbromiL2-63
Org5_Gene1778	accessory	3272	[RbromiL2-63]L2-63_01832_argR_Arginine_hydroxamate_resistance_protein,arginine_repressor,arginine_repressor,Arginine_repressor,_C-terminal_domai	RbromiL2-63
Org5_Gene1779	accessory	1849	[RbromiL2-63]L2-63_01833_ppnK_Probable_inorganic_polyphosphate/ATP-NAD_kinase,inorganic_polyphosphate/ATP-NAD_kinase,Predicted_sugar_kinas	RbromiL2-63
Org5_Gene1783	accessory	4117	[RbromiL2-63]L2-63_01837_L2-63_01837_exodeoxyribonuclease_VII_small_subunit,exodeoxyribonuclease_VII,_small_subunit,Exonuclease_VII_small_subu	RbromiL2-63
Org5_Gene1788	accessory	2738	[RbromiL2-63]L2-63_01842_L2-63_01842_germination_protein,_Ger(x)C_family_1923792:1924382_reverse_MW:21622	RbromiL2-63
Org5_Gene1789	accessory	1290	[RbromiL2-63]L2-63_01843_L2-63_01843_spore_germination_protein_(amino_acid_permease)_1924375:1925442_reverse_MW:39757	RbromiL2-63
Org5_Gene179	accessory	3369	[RbromiL2-63]L2-63_00183_L2-63_00183_hypothetical_protein_199922:200344_forward_MW:14971	RbromiL2-63
Org5_Gene1791	accessory	2312	[RbromiL2-63]L2-63_01845_yetF_2_hypothetical_protein,Protein_of_unknown_function_(DUF421)_1926930:1927646_forward_MW:26606	RbromiL2-63
Org5_Gene1792	accessory	3524	[RbromiL2-63]L2-63_01846_L2-63_01846_hypothetical_protein_1927651:1928031_forward_MW:14399	RbromiL2-63
Org5_Gene1793	accessory	1915	[RbromiL2-63]L2-63_01847_znuB_High-affinity_zinc_uptake_system_membrane_protein_znuB,high-affinity_zinc_transporter_membrane_component,ancl	RbromiL2-63
Org5_Gene1794	accessory	2203	[RbromiL2-63]L2-63_01848_lptB_3_Lipopolysaccharide_export_system_ATP-binding_protein_LptB,hemin_importer_ATP-binding_subunit,ABC-type_uncha	RbromiL2-63
Org5_Gene1795	accessory	1456	[RbromiL2-63]L2-63_01849_adcA_Probable_zinc_transport_system_zinc-binding_lipoprotein_AdcA_precursor,high-affinity_zinc_transporter_periplasmic_	RbromiL2-63
Org5_Gene1799	accessory	2289	[RbromiL2-63]L2-63_01853_ywqD_2_Tyrosine-protein_kinase_YwqD,tyrosine_kinase,Capsular_polysaccharide_biosynthesis_protein,capsular_exopolysaccl	RbromiL2-63
Org5_Gene180	accessory	2981	[RbromiL2-63]L2-63_00184_L2-63_00184_anhydro-N-acetylmuramic_acid_kinase_200530:201054_reverse_MW:19849	RbromiL2-63
Org5_Gene1800	accessory	2257	[RbromiL2-63]L2-63_01854_L2-63_01854_tyrosine_kinase,Capsular_polysaccharide_biosynthesis_protein,polysaccharide_export_protein,_MPA1_family,Ch	RbromiL2-63
Org5_Gene1802	accessory	3365	[RbromiL2-63]L2-63_01856_L2-63_01856_oxaloacetate_decarboxylase_subunit_gamma,Na_-transporting_methylmalonyl-CoA/oxaloacetate_decarboxylas	RbromiL2-63
Org5_Gene1804	accessory	564	[RbromiL2-63]L2-63_01858_L2-63_01858_Domain_of_unknown_function_(DUF3794)_1939996:1941519_reverse_MW:56729	RbromiL2-63
Org5_Gene1805	accessory	4437	[RbromiL2-63]L2-63_01859_L2-63_01859_hypothetical_protein_1941717:1941806_reverse_MW:3391	RbromiL2-63
Org5_Gene1807	accessory	2236	[RbromiL2-63]L2-63_01861_L2-63_01861_hypothetical_protein_1943102:1943839_reverse_MW:27969	RbromiL2-63
Org5_Gene1808	accessory	245	[RbromiL2-63]L2-63_01862_L2-63_01862_Subtilisin_BL,Regulatory_P_domain_of_the_subtilisin-like_proprotein_convertases_and_other_proteases,type_\	RbromiL2-63
Org5_Gene1810	accessory	4192	[RbromiL2-63]L2-63_01864_L2-63_01864_hypothetical_protein_1947179:1947385_forward_MW:7939	RbromiL2-63
Org5_Gene1812	accessory	3944	[RbromiL2-63]L2-63_01866_relG_Toxin_RelG,addiction_module_toxin,_RelE/StbE_family,Plasmid_stabilisation_system_protein_1948253:1948525_reverse	RbromiL2-63
Org5_Gene1813	accessory	4135	[RbromiL2-63]L2-63_01867_L2-63_01867_hypothetical_protein_1948522:1948743_reverse_MW:8613	RbromiL2-63
Org5_Gene1815	accessory	3899	[RbromiL2-63]L2-63_01870_L2-63_01870_hypothetical_protein_1951667:1951948_reverse_MW:10436	RbromiL2-63

Org5_Gene1818	accessory	4415	[RbromiL2-63]L2-63_01873_L2-63_01873_hypothetical_protein_1954177:1954308_forward_MW:5178	RbromiL2-63
Org5_Gene182	accessory	1642	[RbromiL2-63]L2-63_00186_pstS_Phosphate-binding_protein_pstS_precursor,phosphate_ABC_transporter_periplasmic_substrate-binding_protein_PstS,ph	RbromiL2-63
Org5_Gene1821	accessory	901	[RbromiL2-63]L2-63_01876_tagH_1_Teichoic_acids_export_ATP-binding_protein_TagH,teichoic_acids_export_protein_ATP-binding_subunit,ABC-type_Na_	RbromiL2-63
Org5_Gene1822	accessory	1891	[RbromiL2-63]L2-63_01877_L2-63_01877_inner_membrane_transport_permease,ABC-2_type_transporter_1957335:1958177_reverse_MW:31567	RbromiL2-63
Org5_Gene1823	accessory	2471	[RbromiL2-63]L2-63_01878_ppiA_Peptidyl-prolyl_cis-trans_isomerase_A_precursor,peptidyl-prolyl_cis-trans_isomerase_A_(rotamase_A),Cyclophilin_type_	RbromiL2-63
Org5_Gene1824	accessory	3179	[RbromiL2-63]L2-63_01879_yaiI_hypothetical_protein,hypothetical_protein,Uncharacterized_BCR,_YaiI/YqxD_family_COG1671_1958983:1959453_forwarc	RbromiL2-63
Org5_Gene1825	accessory	959	[RbromiL2-63]L2-63_01880_ybbD_hypothetical_protein,beta-hexosaminidase,Glycosyl_hydrolase_family_3_N_terminal_domain_1959915:1961150_revers	RbromiL2-63
Org5_Gene1826	accessory	273	[RbromiL2-63]L2-63_01881_L2-63_01881_Bacterial_Ig-like_domain_(group_2)_1961158:1963185_reverse_MW:73646	RbromiL2-63
Org5_Gene1827	accessory	3109	[RbromiL2-63]L2-63_01882_L2-63_01882_CarD-like/TRCF_domain_1963357:1963845_reverse_MW:18516	RbromiL2-63
Org5_Gene1829	accessory	3983	[RbromiL2-63]L2-63_01884_L2-63_01884_hypothetical_protein_1965839:1966099_forward_MW:9903	RbromiL2-63
Org5_Gene183	accessory	1802	[RbromiL2-63]L2-63_00187_pstC_Phosphate_transport_system_permease_protein_pstC,phosphate_transporter_permease_subunit_PstC,ABC-type_phosp	RbromiL2-63
Org5_Gene1830	accessory	4127	[RbromiL2-63]L2-63_01885_L2-63_01885_hypothetical_protein_1966115:1966336_forward_MW:8489	RbromiL2-63
Org5_Gene1833	accessory	3492	[RbromiL2-63]L2-63_01888_L2-63_01888_Helix-turn-helix_1969390:1969782_reverse_MW:15171	RbromiL2-63
Org5_Gene1834	accessory	1839	[RbromiL2-63]L2-63_01890_L2-63_01890_Predicted_integral_membrane_protein,Protein_of_unknown_function,_DUF624_1970462:1971322_reverse_MW	RbromiL2-63
Org5_Gene1839	accessory	2628	[RbromiL2-63]L2-63_01895_pgsA_2_CDP-diacylglycerol--glycerol-3-phosphate_3-phosphatidyltransferase,phosphatidylglycerophosphate_synthetase,CDP-(	RbromiL2-63
Org5_Gene184	accessory	1612	[RbromiL2-63]L2-63_00188_pstA_Phosphate_transport_system_permease_protein_pstA,phosphate_transporter_permease_subunit_PtsA,ABC-type_phos	RbromiL2-63
Org5_Gene1840	accessory	2453	[RbromiL2-63]L2-63_01896_L2-63_01896_Colicin_V_production_protein_1978546:1979220_reverse_MW:24272	RbromiL2-63
Org5_Gene1841	accessory	865	[RbromiL2-63]L2-63_01897_L2-63_01897_hypothetical_protein_1979226:1980521_reverse_MW:47262	RbromiL2-63
Org5_Gene1851	accessory	3575	[RbromiL2-63]L2-63_01907_L2-63_01907_hypothetical_protein_1988492:1988857_reverse_MW:13440	RbromiL2-63
Org5_Gene1853	accessory	3090	[RbromiL2-63]L2-63_01909_L2-63_01909_hypothetical_protein_1990221:1990712_reverse_MW:18570	RbromiL2-63
Org5_Gene1856	accessory	4435	[RbromiL2-63]L2-63_01912_L2-63_01912_hypothetical_protein_1994938:1995030_reverse_MW:3645	RbromiL2-63
Org5_Gene1857	accessory	1020	[RbromiL2-63]L2-63_01913_L2-63_01913_FtsX-like_permease_family_1995181:1996386_reverse_MW:44961	RbromiL2-63
Org5_Gene1858	accessory	2439	[RbromiL2-63]L2-63_01914_lolD_2_Lipoprotein-releasing_system_ATP-binding_protein_LolD,lipoprotein_transporter_ATP-binding_subunit,Predicted_ABC	RbromiL2-63
Org5_Gene1859	accessory	773	[RbromiL2-63]L2-63_01915_L2-63_01915_FtsX-like_permease_family_1997074:1998420_reverse_MW:50032	RbromiL2-63
Org5_Gene186	accessory	2551	[RbromiL2-63]L2-63_00190_phoU_1_Phosphate_transport_system_protein_phoU_homolog,transcriptional_regulator_PhoU,Uncharacterized_conserved_p	RbromiL2-63
Org5_Gene1862	accessory	4342	[RbromiL2-63]L2-63_01918_L2-63_01918_hypothetical_protein_2000268:2000441_forward_MW:6878	RbromiL2-63
Org5_Gene1863	accessory	2776	[RbromiL2-63]L2-63_01919_L2-63_01919_hypothetical_protein_2001130:2001708_reverse_MW:22313	RbromiL2-63
Org5_Gene1864	accessory	2523	[RbromiL2-63]L2-63_01920_L2-63_01920_Fluoroquinolones_export_ATP-binding_protein_Rv2688c/MT2762,cytochrome_c_biogenesis_protein_CcmA,ABC	RbromiL2-63
Org5_Gene1865	accessory	1919	[RbromiL2-63]L2-63_01921_L2-63_01921_ABC-2_family_transporter_protein_2002363:2003199_reverse_MW:32170	RbromiL2-63
Org5_Gene1866	accessory	2381	[RbromiL2-63]L2-63_01922_L2-63_01922_hypothetical_protein_2003196:2003897_reverse_MW:26888	RbromiL2-63
Org5_Gene1867	accessory	1954	[RbromiL2-63]L2-63_01923_L2-63_01923_ABC-2_family_transporter_protein_2003887:2004711_reverse_MW:31959	RbromiL2-63
Org5_Gene1868	accessory	2409	[RbromiL2-63]L2-63_01924_L2-63_01924_hypothetical_protein_2004730:2005422_reverse_MW:25703	RbromiL2-63
Org5_Gene1869	accessory	3212	[RbromiL2-63]L2-63_01925_L2-63_01925_hypothetical_protein_2005543:2006004_reverse_MW:17191	RbromiL2-63
Org5_Gene187	accessory	2472	[RbromiL2-63]L2-63_00191_regX3_1_Sensory_transduction_protein_regX3,DNA-binding_response_regulator_CreB,Response_regulator,phosphate_regulo	RbromiL2-63
Org5_Gene1870	accessory	3656	[RbromiL2-63]L2-63_01926_L2-63_01926_hypothetical_protein_2006147:2006473_reverse_MW:12515	RbromiL2-63
Org5_Gene1871	accessory	2377	[RbromiL2-63]L2-63_01927_L2-63_01927_hypothetical_protein_2006560:2007261_reverse_MW:27364	RbromiL2-63
Org5_Gene1872	accessory	754	[RbromiL2-63]L2-63_01928_L2-63_01928_Predicted_ATPase_2007266:2008621_reverse_MW:53245	RbromiL2-63
Org5_Gene1873	accessory	2449	[RbromiL2-63]L2-63_01929_L2-63_01929_hypothetical_protein_2009692:2010360_reverse_MW:25478	RbromiL2-63
Org5_Gene1874	accessory	3641	[RbromiL2-63]L2-63_01930_L2-63_01930_hypothetical_protein_2010398:2010748_reverse_MW:13203	RbromiL2-63
Org5_Gene1875	accessory	3165	[RbromiL2-63]L2-63_01931_L2-63_01931_flavodoxin,Flavodoxin,Flavodoxin_2010913:2011386_reverse_MW:17694	RbromiL2-63
Org5_Gene1877	accessory	3036	[RbromiL2-63]L2-63_01933_L2-63_01933_flavodoxin,Flavodoxin_2011862:2012368_reverse_MW:18670	RbromiL2-63
Org5_Gene1879	accessory	759	[RbromiL2-63]L2-63_01935_dacB_2_D-alanyl-D-alanine_carboxypeptidase_dacB_precursor,D-alanyl-D-alanine_carboxypeptidase,D-alanyl-D-alanine_carbo	RbromiL2-63
Org5_Gene188	accessory	466	[RbromiL2-63]L2-63_00192_phoR_1_Alkaline_phosphatase_synthesis_sensor_protein_phoR,sensory_histidine_kinase_CreC,Predicted_periplasmic_ligand-	RbromiL2-63
Org5_Gene1883	accessory	1077	[RbromiL2-63]L2-63_01939_L2-63_01939_sporulation_protein_YqfD,Putative_stage_IV_sporulation_protein_YqfD_2019519:2020697_reverse_MW:44472	RbromiL2-63
Org5_Gene1884	accessory	3933	[RbromiL2-63]L2-63_01940_L2-63_01940_sporulation_protein_YqfC,YabP_family_2020707:2020979_reverse_MW:10041	RbromiL2-63
Org5_Gene1885	accessory	1279	[RbromiL2-63]L2-63_01941_L2-63_01941_Uncharacterized_protein_conserved_in_bacteria,sporulation_integral_membrane_protein_YlbJ,Nucleoside_recc	RbromiL2-63
Org5_Gene1887	accessory	3822	[RbromiL2-63]L2-63_01943_ybjG_Putative_undecaprenyl-diphosphatase_ybjG,undecaprenyl_pyrophosphate_phosphatase,PAP2_superfamily_2024209:20	RbromiL2-63
Org5_Gene1888	accessory	223	[RbromiL2-63]L2-63_01944_L2-63_01944_CHAP_domain_2024625:2026784_reverse_MW:80131	RbromiL2-63
Org5_Gene1889	accessory	4090	[RbromiL2-63]L2-63_01945_L2-63_01945_Predicted_transcriptional_regulator_2026931:2027161_forward_MW:8870	RbromiL2-63
Org5_Gene189	accessory	369	[RbromiL2-63]L2-63_00193_phoU_2_Phosphate_transport_system_protein_phoU_homolog,transcriptional_regulator_PhoU,Na/Pi-cotransporter_II-relatec	RbromiL2-63
Org5_Gene1892	accessory	1906	[RbromiL2-63]L2-63_01948_L2-63_01948_ComEC_family_competence_protein,Predicted_metal-dependent_RNase,_consists_of_a_metallo-beta-lactamas	RbromiL2-63
Org5_Gene1894	accessory	104	[RbromiL2-63]L2-63_01950_spoIIIE_Stage_III_sporulation_protein_E,DNA_translocase_FtsK,DNA_segregation_ATPase_FtsK/SpoIIIE_and_related_proteins,	RbromiL2-63
Org5_Gene1898	accessory	3481	[RbromiL2-63]L2-63_01954_L2-63_01954_Predicted_membrane_protein,Protein_of_unknown_function_(DUF1113)_2038463:2038855_reverse_MW:1485	RbromiL2-63
Org5_Gene190	accessory	2554	[RbromiL2-63]L2-63_00194_yqfA_hypothetical_protein,hemolysin,Predicted_membrane_protein,_hemolysin_III_homolog,channel_protein,_hemolysin_III_	RbromiL2-63
Org5_Gene1900	accessory	1334	[RbromiL2-63]L2-63_01956_L2-63_01956_DNA_polymerase_III_subunit_delta,DNA_polymerase_III,_delta_subunit,DNA_polymerase_III,_delta_subunit_2C	RbromiL2-63
Org5_Gene1901	accessory	204	[RbromiL2-63]L2-63_01957_L2-63_01957_DNA_internalization-related_competence_protein_ComEC/Rec2,Competence_protein_2040546:2042786_revers	RbromiL2-63
Org5_Gene1902	accessory	2974	[RbromiL2-63]L2-63_01958_L2-63_01958_hypothetical_protein_2042799:2043326_reverse_MW:18995	RbromiL2-63
Org5_Gene1905	accessory	2279	[RbromiL2-63]L2-63_01961_plsY_G3P_acyltransferase,putative_glycerol-3-phosphate_acyltransferase_PlsY,Predicted_membrane_protein,acyl-phosphate_	RbromiL2-63
Org5_Gene1907	accessory	848	[RbromiL2-63]L2-63_01963_L2-63_01963_putative_FeS-containing_Cyanobacterial-specific_oxidoreductase,Protein_of_unknown_function_(DUF512)_204(	RbromiL2-63
Org5_Gene191	accessory	2321	[RbromiL2-63]L2-63_00195_ecsA_ABC-type_transporter_ATP-binding_protein_EcsA,nodulation_ABC_transporter_NodI,ABC-type_Na__transport_system,_	RbromiL2-63
Org5_Gene1910	accessory	2390	[RbromiL2-63]L2-63_01966_L2-63_01966_conserved_hypothetical_protein_2049347:2050042_forward_MW:26928	RbromiL2-63
Org5_Gene1911	accessory	2328	[RbromiL2-63]L2-63_01967_L2-63_01967_hypothetical_protein,conserved_hypothetical_integral_membrane_protein,Uncharacterized_ACR,_YhhQ_family_	RbromiL2-63
Org5_Gene1912	accessory	3526	[RbromiL2-63]L2-63_01968_L2-63_01968_Penicillinase_repressor_2051054:2051434_forward_MW:14651	RbromiL2-63
Org5_Gene1913	accessory	1033	[RbromiL2-63]L2-63_01969_blaR1_Regulatory_protein_BlaR1,Antirepressor_regulating_drug_resistance,_predicted_signal_transduction_N-terminal_mem	RbromiL2-63
Org5_Gene1914	accessory	3816	[RbromiL2-63]L2-63_01970_L2-63_01970_hypothetical_protein_2052695:2053000_forward_MW:11303	RbromiL2-63
Org5_Gene1915	accessory	1092	[RbromiL2-63]L2-63_01971_L2-63_01971_RmuC_family_2053058:2054227_reverse_MW:44132	RbromiL2-63
Org5_Gene1917	accessory	1394	[RbromiL2-63]L2-63_01973_L2-63_01973_Putative_bifunctional_phosphatase/peptidyl-prolyl_cis-trans_isomerase,peptidyl-prolyl_cis-trans_isomerase_A_	RbromiL2-63
Org5_Gene1919	accessory	1616	[RbromiL2-63]L2-63_01975_L2-63_01975_regulatory_ATPase_RavA,Uncharacterized_conserved_protein_(some_members_contain_a_von_Willebrand_fac	RbromiL2-63
Org5_Gene192	accessory	509	[RbromiL2-63]L2-63_00196_L2-63_00196_Predicted_permease_213268:214872_forward_MW:58573	RbromiL2-63
Org5_Gene1920	accessory	1511	[RbromiL2-63]L2-63_01976_L2-63_01976_Uncharacterized_conserved_protein_(some_members_contain_a_von_Willebrand_factor_type_A_(vWA)_doma	RbromiL2-63
Org5_Gene1921	accessory	226	[RbromiL2-63]L2-63_01977_L2-63_01977_Uncharacterized_protein_conserved_in_bacteria,Transglutaminase-like_superfamily_2059979:2062123_forward	RbromiL2-63
Org5_Gene1922	accessory	4304	[RbromiL2-63]L2-63_01978_L2-63_01978_hypothetical_protein_2062208:2062390_reverse_MW:7254	RbromiL2-63
Org5_Gene1923	accessory	3302	[RbromiL2-63]L2-63_01979_L2-63_01979_two-component_response_regulator,Response_regulator_of_the_LytR/AlgR_family,LytTr_DNA-binding_domain_	RbromiL2-63
Org5_Gene1924	accessory	3275	[RbromiL2-63]L2-63_01980_L2-63_01980_Protein_of_unknown_function_(DUF3021)_2063020:2063466_forward_MW:17036	RbromiL2-63
Org5_Gene1926	accessory	1598	[RbromiL2-63]L2-63_01982_L2-63_01982_ABC-2_family_transporter_protein_2064495:2065427_forward_MW:33800	RbromiL2-63
Org5_Gene1927	accessory	1207	[RbromiL2-63]L2-63_01983_metE_5-methyltetrahydropteroyltriglutamate--homocysteine_methyltransferase,5-methyltetrahydropteroyltriglutamate--hom	RbromiL2-63
Org5_Gene1928	accessory	1711	[RbromiL2-63]L2-63_01984_cysB_Cys_regulon_transcriptional_activator,transcriptional_regulator_CysB-like_protein,aminoethylphosphonate_catabolism_	RbromiL2-63
Org5_Gene1929	accessory	2985	[RbromiL2-63]L2-63_01985_nfrA2_FMN_reductase_[NAD(P)H],F420-0--gamma-glutamyl_ligase,F420_biosynthesis_protein_FbiB,_C-terminal_domain,Nitro	RbromiL2-63
Org5_Gene1930	accessory	2909	[RbromiL2-63]L2-63_01986_L2-63_01986_malonic_semialdehyde_reductase,5,6-dimethylbenzimidazole_synthase,Nitroreductase_family_2068143:206868	RbromiL2-63
Org5_Gene1931	accessory	1553	[RbromiL2-63]L2-63_01987_L2-63_01987_auxin_efflux_carrier,Membrane_transport_protein_2068744:2069670_reverse_MW:33742	RbromiL2-63
Org5_Gene1932	accessory	4303	[RbromiL2-63]L2-63_01988_L2-63_01988_Domain_of_Unknown_Function_(DUF1540)_2069740:2069922_reverse_MW:6653	RbromiL2-63
Org5_Gene1934	accessory	379	[RbromiL2-63]L2-63_01990_L2-63_01990_Protein_of_unknown_function_(DUF524)_2070814:2072592_reverse_MW:69490	RbromiL2-63
Org5_Gene1935	accessory	78	[RbromiL2-63]L2-63_01991_L2-63_01991_hypothetical_protein_2072608:2075712_reverse_MW:121012	RbromiL2-63
Org5_Gene1936	accessory	3782	[RbromiL2-63]L2-63_01992_L2-63_01992_hypothetical_protein_2076467:2076691_forward_MW:8564	RbromiL2-63
Org5_Gene1937	accessory	2434	[RbromiL2-63]L2-63_01993_L2-63_01993_hypothetical_protein_2076717:2077397_forward_MW:25685	RbromiL2-63
Org5_Gene1938	accessory	3848	[RbromiL2-63]L2-63_01994_L2-63_01994_hypothetical_protein_2077416:2077712_forward_MW:11210	RbromiL2-63
Org5_Gene1939	accessory	4426	[RbromiL2-63]L2-63_01995_L2-63_01995_hypothetical_protein_2078065:2078181_forward_MW:4172	RbromiL2-63
Org5_Gene194	accessory	1140	[RbromiL2-63]L2-63_00198_L2-63_00198_conserved_hypothetical_protein,Uncharacterised_protein_family_(UPF0104)_216774:217922_reverse_MW:433	RbromiL2-63
Org5_Gene1940	accessory	3647	[RbromiL2-63]L2-63_01996_L2-63_01996_hypothetical_protein_2078178:2078525_forward_MW:13221	RbromiL2-63
Org5_Gene1941	accessory	172	[RbromiL2-63]L2-63_01997_L2-63_01997_ski2-like_helicase,Superfamily_II_helicase,DEAD/DEAH_box_helicase_2078582:2080936_reverse_MW:92511	RbromiL2-63
Org5_Gene1942	accessory	1570	[RbromiL2-63]L2-63_01998_L2-63_01998_hypothetical_protein_2080938:2081879_reverse_MW:37056	RbromiL2-63
Org5_Gene1943	accessory	559	[RbromiL2-63]L2-63_01999_L2-63_01999_V-type_ATP_synthase_subunit_E,Resolvase,_N_terminal_domain_2082106:2083635_reverse_MW:58375	RbromiL2-63
Org5_Gene1944	accessory	1881	[RbromiL2-63]L2-63_02000_parB_4_Probable_chromosome-partitioning_protein_parB,plasmid_partitioning_protein_RepB,ParB/RepB/Spo0J_family_parti	RbromiL2-63
Org5_Gene1945	accessory	692	[RbromiL2-63]L2-63_02001_L2-63_02001_Relaxase/Mobilisation_nuclease_domain_2084750:2086144_reverse_MW:55160	RbromiL2-63
Org5_Gene1946	accessory	3610	[RbromiL2-63]L2-63_02002_L2-63_02002_hypothetical_protein_2086105:2086461_reverse_MW:13714	RbromiL2-63
Org5_Gene1947	accessory	3706	[RbromiL2-63]L2-63_02003_L2-63_02003_hypothetical_protein_2086780:2087112_reverse_MW:12750	RbromiL2-63
Org5_Gene1948	accessory	1613	[RbromiL2-63]L2-63_02004_L2-63_02004_hypothetical_protein_2087109:2088035_reverse_MW:35067	RbromiL2-63
Org5_Gene1949	accessory	4069	[RbromiL2-63]L2-63_02005_L2-63_02005_hypothetical_protein_2088040:2088276_reverse_MW:8990	RbromiL2-63
Org5_Gene195	accessory	2951	[RbromiL2-63]L2-63_00199_L2-63_00199_serine/threonine_protein_kinase,Kae1-associated_kinase_Bud32_218220:218753_forward_MW:21061	RbromiL2-63
Org5_Gene1950	accessory	1168	[RbromiL2-63]L2-63_02006_L2-63_02006_hypothetical_protein_2088559:2089695_forward_MW:44358	RbromiL2-63
Org5_Gene1951	accessory	1739	[RbromiL2-63]L2-63_02007_L2-63_02007_RNA_polymerase_sigma_factor,_sigma-70_family_2089692:2090579_forward_MW:34527	RbromiL2-63
Org5_Gene1952	accessory	602	[RbromiL2-63]L2-63_02008_L2-63_02008_hypothetical_protein_2090626:2092104_forward_MW:57966	RbromiL2-63
Org5_Gene1953	accessory	1305	[RbromiL2-63]L2-63_02009_L2-63_02009_hypothetical_protein_2092137:2093195_forward_MW:42221	RbromiL2-63
Org5_Gene1954	accessory	2417	[RbromiL2-63]L2-63_02010_L2-63_02010_hypothetical_protein_2093176:2093865_forward_MW:27166	RbromiL2-63
Org5_Gene1955	accessory	237	[RbromiL2-63]L2-63_02011_L2-63_02011_Uncharacterized_conserved_protein,Protein_of_unknown_function_DUF262_2094102:2096207_reverse_MW:81	RbromiL2-63
Org5_Gene1956	accessory	2376	[RbromiL2-63]L2-63_02012_L2-63_02012_AlwI_restriction_endonuclease_2096225:2096926_reverse_MW:27355	RbromiL2-63
Org5_Gene1957	accessory	2307	[RbromiL2-63]L2-63_02013_L2-63_02013_hypothetical_protein_2096950:2097666_reverse_MW:27405	RbromiL2-63
Org5_Gene1958	accessory	4264	[RbromiL2-63]L2-63_02014_L2-63_02014_hypothetical_protein_2097667:2097858_reverse_MW:7640	RbromiL2-63
Org5_Gene1959	accessory	3775	[RbromiL2-63]L2-63_02015_L2-63_02015_Adenine-specific_DNA_methylase,D12_class_N6_adenine-specific_DNA_methyltransferase_2097894:2098211_r	RbromiL2-63
Org5_Gene196	accessory	3229	[RbromiL2-63]L2-63_00200_L2-63_00200_hypothetical_protein_218766:219224_forward_MW:17410	RbromiL2-63
Org5_Gene1960	accessory	3544	[RbromiL2-63]L2-63_02016_L2-63_02016_hypothetical_protein_2098441:2098797_reverse_MW:13926	RbromiL2-63
Org5_Gene1961	accessory	4311	[RbromiL2-63]L2-63_02017_L2-63_02017_hypothetical_protein_2098801:2098980_reverse_MW:7050	RbromiL2-63
Org5_Gene1962	accessory	1614	[RbromiL2-63]L2-63_02019_L2-63_02019_hypothetical_protein_2099546:2100472_reverse_MW:35077	RbromiL2-63
Org5_Gene1967	accessory	395	[RbromiL2-63]L2-63_02024_dnaX_2_DNA_polymerase_III_subunit_tau,DNA_polymerase_III_subunits_gamma_and_tau,Holliday_junction_resolvasome,_h	RbromiL2-63
Org5_Gene1968	accessory	713	[RbromiL2-63]L2-63_02027_L2-63_02027_dihydrolipoamide_dehydrogenase,dihydrolipoyl_dehydrogenase,Pyridine_nucleotide-disulphide_oxidoreductase	RbromiL2-63
Org5_Gene197	accessory	3462	[RbromiL2-63]L2-63_00201_L2-63_00201_putative_manganese-dependent_inorganic_pyrophosphatase,Predicted_transcriptional_regulator_with_C-termi	RbromiL2-63
Org5_Gene1970	accessory	4123	[RbromiL2-63]L2-63_02030_L2-63_02030_hypothetical_protein_2106805:2107026_reverse_MW:8279	RbromiL2-63
Org5_Gene1971	accessory	2667	[RbromiL2-63]L2-63_02031_bcrC_Undecaprenyl-diphosphatase_BcrC,undecaprenyl_pyrophosphate_phosphatase,PAP2_(acid_phosphatase)_superfamily_	RbromiL2-63
Org5_Gene1973	accessory	2697	[RbromiL2-63]L2-63_02033_L2-63_02033_Putative_acetyltransferase_SA2342,maltose_O-acetyltransferase,Acetyltransferase_(isoleucine_patch_superfam	RbromiL2-63
Org5_Gene1974	accessory	4251	[RbromiL2-63]L2-63_02034_L2-63_02034_hypothetical_protein_2109829:2110023_forward_MW:7563	RbromiL2-63
Org5_Gene1976	accessory	2287	[RbromiL2-63]L2-63_02036_L2-63_02036_Predicted_glutamine_amidotransferase,CobB/CobQ-like_glutamine_amidotransferase_domain_2111588:211231	RbromiL2-63
Org5_Gene1978	accessory	2496	[RbromiL2-63]L2-63_02038_L2-63_02038_DNA_utilization_protein_GntX,comF_family_protein,Phosphoribosyl_transferase_domain_2114514:2115176_for	RbromiL2-63

Org5_Gene1979	accessory	1392	[RbromiL2-63]L2-63_02039_mreB_2_Rod_shape-determining_protein_MreB,rod_shape-determining_protein_MreB,Ethanolamine_utilization_protein,_po	RbromiL2-63
Org5_Gene198	accessory	2433	[RbromiL2-63]L2-63_00202_srrA_1_Staphylococcal_respiratory_response_protein_A,DNA-binding_response_regulator_CreB,Response_regulator_of_citrat	RbromiL2-63
Org5_Gene1980	accessory	2808	[RbromiL2-63]L2-63_02040_L2-63_02040_hypothetical_protein_2116414:2116983_reverse_MW:22094	RbromiL2-63
Org5_Gene1981	accessory	69	[RbromiL2-63]L2-63_02041_amyE_4_Alpha-amylase_precursor,cytoplasmic_alpha-amylase,malto-oligosyltrehalose_trehalohydrolase,Alpha_amylase,_cata	RbromiL2-63
Org5_Gene1988	accessory	1239	[RbromiL2-63]L2-63_02048_strE_2_dTDP-glucose_4,6-dehydratase,dTDP-glucose_4,6-dehydratase,dTDP-glucose_4,6-dehydratase,NAD_dependent_epime	RbromiL2-63
Org5_Gene1989	accessory	337	[RbromiL2-63]L2-63_02049_L2-63_02049_LPS_biosynthesis_protein,LicD_family_2128152:2130029_forward_MW:70865	RbromiL2-63
Org5_Gene199	accessory	417	[RbromiL2-63]L2-63_00203_qseC_Sensor_protein_qseC,sensory_histidine_kinase_CreC,Predicted_periplasmic_ligand-binding_sensor_domain,phosphate_	RbromiL2-63
Org5_Gene1990	accessory	531	[RbromiL2-63]L2-63_02052_L2-63_02052_Recombinase_2130670:2132232_reverse_MW:60892	RbromiL2-63
Org5_Gene1991	accessory	1465	[RbromiL2-63]L2-63_02053_L2-63_02053_hypothetical_protein_2132488:2133471_forward_MW:37233	RbromiL2-63
Org5_Gene1992	accessory	4120	[RbromiL2-63]L2-63_02054_L2-63_02054_hypothetical_protein_2133495:2133716_forward_MW:8611	RbromiL2-63
Org5_Gene1993	accessory	3155	[RbromiL2-63]L2-63_02055_L2-63_02055_Protein_of_unknown_function_(DUF3801)_2133758:2134234_forward_MW:17554	RbromiL2-63
Org5_Gene1995	accessory	335	[RbromiL2-63]L2-63_02057_L2-63_02057_Recombinase_2135290:2137170_forward_MW:71807	RbromiL2-63
Org5_Gene1996	accessory	3627	[RbromiL2-63]L2-63_02058_L2-63_02058_hypothetical_protein_2137197:2137550_forward_MW:13697	RbromiL2-63
Org5_Gene1997	accessory	3837	[RbromiL2-63]L2-63_02059_L2-63_02059_hypothetical_protein_2137567:2137866_forward_MW:11751	RbromiL2-63
Org5_Gene1998	accessory	3571	[RbromiL2-63]L2-63_02060_L2-63_02060_anaerobic_benzoate_catabolism_transcriptional_regulator,Helix-turn-helix_2137894:2138262_reverse_MW:137	RbromiL2-63
Org5_Gene1999	accessory	4277	[RbromiL2-63]L2-63_02061_L2-63_02061_hypothetical_protein_2138407:2138595_forward_MW:6988	RbromiL2-63
Org5_Gene2	accessory	1475	[RbromiL2-63]L2-63_00002_ymdB_O-acetyl-ADP-ribose_deacetylase,RNase_III_inhibitor,Macro_domain_2009:2986_forward_MW:36435	RbromiL2-63
Org5_Gene200	accessory	1112	[RbromiL2-63]L2-63_00204_L2-63_00204_hypothetical_protein_222859:224022_forward_MW:43691	RbromiL2-63
Org5_Gene2000	accessory	2383	[RbromiL2-63]L2-63_02062_yycF_2_Transcriptional_regulatory_protein_YycF,DNA-binding_transcriptional_regulator_CpxR,Response_regulator_of_citrate,	RbromiL2-63
Org5_Gene2001	accessory	1627	[RbromiL2-63]L2-63_02063_walK_Sensor_protein_kinase_walK,signal_transduction_histidine-protein_kinase_BaeS,Predicted_periplasmic_ligand-binding_	RbromiL2-63
Org5_Gene2002	accessory	2150	[RbromiL2-63]L2-63_02064_L2-63_02064_Uncharacterized_protein_conserved_in_bacteria,lantibiotic_protection_ABC_transporter_permease_subunit,_M	RbromiL2-63
Org5_Gene2003	accessory	3355	[RbromiL2-63]L2-63_02065_L2-63_02065_hypothetical_protein_2141101:2141526_forward_MW:15930	RbromiL2-63
Org5_Gene2004	accessory	1629	[RbromiL2-63]L2-63_02066_drrA_2_Daunorubicin/doxorubicin_resistance_ATP-binding_protein_DrrA,nodulation_ABC_transporter_NodI,ABC-type_Na__tr	RbromiL2-63
Org5_Gene2005	accessory	2259	[RbromiL2-63]L2-63_02067_L2-63_02067_Uncharacterized_protein_conserved_in_bacteria,lantibiotic_protection_ABC_transporter_permease_subunit,_M	RbromiL2-63
Org5_Gene2006	accessory	2243	[RbromiL2-63]L2-63_02068_L2-63_02068_Uncharacterized_protein_conserved_in_bacteria,ABC-2_family_transporter_protein_2143202:2143936_forward_	RbromiL2-63
Org5_Gene2007	accessory	3406	[RbromiL2-63]L2-63_02069_L2-63_02069_RNA_polymerase_sigma_factor,_sigma-70_family,Sigma-70,_region_4_2144406:2144819_forward_MW:15938	RbromiL2-63
Org5_Gene2008	accessory	3257	[RbromiL2-63]L2-63_02070_L2-63_02070_RNA_polymerase_sigma_factor,RNA_polymerase_sigma_factor,_sigma-70_family_2144923:2145375_forward_M	RbromiL2-63
Org5_Gene2009	accessory	3215	[RbromiL2-63]L2-63_02071_L2-63_02071_RNA_polymerase_sigma_factor,_sigma-70_family_2145386:2145847_forward_MW:17991	RbromiL2-63
Org5_Gene201	accessory	2886	[RbromiL2-63]L2-63_00205_proX_Prolyl-tRNA_deacylase_proX;,Uncharacterized_conserved_protein,YbaK_/_prolyl-tRNA_synthetases_associated_domain_	RbromiL2-63
Org5_Gene2010	accessory	3869	[RbromiL2-63]L2-63_02072_L2-63_02072_Protein_of_unknown_function_(DUF3847)_2145876:2146166_forward_MW:10885	RbromiL2-63
Org5_Gene2011	accessory	638	[RbromiL2-63]L2-63_02073_mobA_2_DNA_strand_transferase,conjugal_transfer_relaxase_TraA,Ti-type_conjugative_transfer_relaxase_TraA,MobA/MobL_	RbromiL2-63
Org5_Gene2012	accessory	3873	[RbromiL2-63]L2-63_02074_yafQ_3_mRNA_interferase_YafQ,addiction_module_toxin,_RelE/StbE_family,Plasmid_stabilisation_system_protein_2147906:	RbromiL2-63
Org5_Gene2013	accessory	3570	[RbromiL2-63]L2-63_02075_L2-63_02075_addiction_module_antitoxin,_RelB/DinJ_family,RelB_antitoxin_2148193:2148561_reverse_MW:13809	RbromiL2-63
Org5_Gene2014	accessory	4324	[RbromiL2-63]L2-63_02076_L2-63_02076_hypothetical_protein_2148578:2148754_forward_MW:6117	RbromiL2-63
Org5_Gene2015	accessory	2240	[RbromiL2-63]L2-63_02077_L2-63_02077_phage_replisome_organizer,_putative,_N-terminal_region,N-terminal_phage_replisome_organiser_(Phage_rep_	RbromiL2-63
Org5_Gene2016	accessory	1823	[RbromiL2-63]L2-63_02078_dnaC_3_DNA_replication_protein_dnaC,hypothetical_protein,DNA_replication_protein,chromosomal_replication_initiator_pr	RbromiL2-63
Org5_Gene2017	accessory	4252	[RbromiL2-63]L2-63_02079_L2-63_02079_hypothetical_protein_2150884:2151078_forward_MW:7233	RbromiL2-63
Org5_Gene2018	accessory	3573	[RbromiL2-63]L2-63_02080_L2-63_02080_hypothetical_protein_2151156:2151521_forward_MW:14099	RbromiL2-63
Org5_Gene202	accessory	219	[RbromiL2-63]L2-63_00206_L2-63_00206_hypothetical_protein_224753:226930_reverse_MW:80290	RbromiL2-63
Org5_Gene2020	accessory	4347	[RbromiL2-63]L2-63_02082_L2-63_02082_hypothetical_protein_2152448:2152618_forward_MW:6852	RbromiL2-63
Org5_Gene2022	accessory	3937	[RbromiL2-63]L2-63_02084_L2-63_02084_Predicted_transcriptional_regulator_2153348:2153620_forward_MW:10279	RbromiL2-63
Org5_Gene2023	accessory	3529	[RbromiL2-63]L2-63_02085_L2-63_02085_hypothetical_protein_2153580:2153957_forward_MW:14876	RbromiL2-63
Org5_Gene2024	accessory	3380	[RbromiL2-63]L2-63_02086_L2-63_02086_hypothetical_protein_2154011:2154430_forward_MW:17200	RbromiL2-63
Org5_Gene2025	accessory	3246	[RbromiL2-63]L2-63_02087_L2-63_02087_Bacterial_transcription_activator,_effector_binding_domain_2154463:2154918_forward_MW:16736	RbromiL2-63
Org5_Gene2026	accessory	3760	[RbromiL2-63]L2-63_02088_L2-63_02088_Regulator_of_competence-specific_genes,TfoX_N-terminal_domain_2154942:2155262_forward_MW:12135	RbromiL2-63
Org5_Gene2027	accessory	4025	[RbromiL2-63]L2-63_02089_L2-63_02089_Protein_of_unknown_function_(DUF3781)_2155291:2155539_forward_MW:9665	RbromiL2-63
Org5_Gene2028	accessory	3173	[RbromiL2-63]L2-63_02090_L2-63_02090_putative_acetyltransferase,ribosomal-protein-alanine_acetyltransferase,Acetyltransferase_(GNAT)_family_2155	RbromiL2-63
Org5_Gene2029	accessory	2016	[RbromiL2-63]L2-63_02091_L2-63_02091_Helix-turn-helix_domain_2156136:2156942_reverse_MW:30984	RbromiL2-63
Org5_Gene203	accessory	1716	[RbromiL2-63]L2-63_00207_fbpC_Fe(3_)_ions_import_ATP-binding_protein_FbpC,nodulation_ABC_transporter_NodI,ABC-type_uncharacterized_transport	RbromiL2-63
Org5_Gene2030	accessory	4281	[RbromiL2-63]L2-63_02092_L2-63_02092_hypothetical_protein_2157426:2157614_forward_MW:7515	RbromiL2-63
Org5_Gene2031	accessory	3665	[RbromiL2-63]L2-63_02093_L2-63_02093_hypothetical_protein_2157611:2157952_forward_MW:12372	RbromiL2-63
Org5_Gene2032	accessory	3846	[RbromiL2-63]L2-63_02094_L2-63_02094_Protein_of_unknown_function_(DUF3847)_2157945:2158241_forward_MW:11729	RbromiL2-63
Org5_Gene2033	accessory	4129	[RbromiL2-63]L2-63_02095_L2-63_02095_hypothetical_protein_2158559:2158780_forward_MW:8583	RbromiL2-63
Org5_Gene2034	accessory	523	[RbromiL2-63]L2-63_02096_mobA_3_DNA_strand_transferase,conjugal_transfer_relaxase_TraA,Ti-type_conjugative_transfer_relaxase_TraA,MobA/MobL_	RbromiL2-63
Org5_Gene2035	accessory	2559	[RbromiL2-63]L2-63_02097_dnaG_3_DNA_primase,DNA_primase,DNA_primase_(bacterial_type),DNA_primase,CHC2_zinc_finger_2160611:2161255_forwa	RbromiL2-63
Org5_Gene2036	accessory	800	[RbromiL2-63]L2-63_02098_L2-63_02098_Predicted_P-loop_ATPase_and_inactivated_derivatives,Virulence-associated_protein_E_2161233:2162561_forw	RbromiL2-63
Org5_Gene2037	accessory	4233	[RbromiL2-63]L2-63_02099_L2-63_02099_hypothetical_protein_2162803:2163000_forward_MW:7241	RbromiL2-63
Org5_Gene2038	accessory	451	[RbromiL2-63]L2-63_02100_L2-63_02100_Resolvase,_N_terminal_domain_2163001:2164671_forward_MW:64623	RbromiL2-63
Org5_Gene2039	accessory	1803	[RbromiL2-63]L2-63_02101_L2-63_02101_hypothetical_protein_2164864:2165733_forward_MW:31622	RbromiL2-63
Org5_Gene204	accessory	3536	[RbromiL2-63]L2-63_00208_L2-63_00208_transcriptional_regulatory_protein_PtsJ,phosphonate_metabolism_transcriptional_regulator_PhnF,Bacterial_reg	RbromiL2-63
Org5_Gene2040	accessory	3451	[RbromiL2-63]L2-63_02102_L2-63_02102_PrgI_family_protein_2165911:2166312_forward_MW:14996	RbromiL2-63
Org5_Gene2041	accessory	146	[RbromiL2-63]L2-63_02103_L2-63_02103_Type_IV_secretory_pathway,_VirB4_components,type-IV_secretion_system_protein_TraC,AAA-like_domain_21	RbromiL2-63
Org5_Gene2042	accessory	1526	[RbromiL2-63]L2-63_02104_yhdJ_DNA_adenine_methyltransferase_YhdJ,putative_methyltransferase,Adenine_specific_DNA_methylase_Mod,DNA_methy	RbromiL2-63
Org5_Gene2043	accessory	225	[RbromiL2-63]L2-63_02105_L2-63_02105_conserved_hypothetical_protein_2169615:2171762_forward_MW:79069	RbromiL2-63
Org5_Gene2044	accessory	3912	[RbromiL2-63]L2-63_02106_L2-63_02106_hypothetical_protein_2171797:2172075_forward_MW:10340	RbromiL2-63
Org5_Gene2045	accessory	2334	[RbromiL2-63]L2-63_02107_L2-63_02107_hypothetical_protein_2172068:2172769_forward_MW:25235	RbromiL2-63
Org5_Gene2046	accessory	4274	[RbromiL2-63]L2-63_02108_L2-63_02108_hypothetical_protein_2172766:2172954_forward_MW:7034	RbromiL2-63
Org5_Gene2048	accessory	250	[RbromiL2-63]L2-63_02110_topB_3_DNA_topoisomerase_3,DNA_topoisomerase_III,Topoisomerase_IA,DNA_topoisomerase_III,DNA_topoisomerase_2173	RbromiL2-63
Org5_Gene2049	accessory	49	[RbromiL2-63]L2-63_02111_L2-63_02111_Antirestriction_protein,Antirestriction_protein_(ArdA)_2175274:2178837_forward_MW:134141	RbromiL2-63
Org5_Gene2050	accessory	1542	[RbromiL2-63]L2-63_02112_L2-63_02112_hypothetical_protein_2178841:2179794_forward_MW:36577	RbromiL2-63
Org5_Gene2051	accessory	4199	[RbromiL2-63]L2-63_02113_L2-63_02113_hypothetical_protein_2179797:2180003_forward_MW:7601	RbromiL2-63
Org5_Gene2052	accessory	4018	[RbromiL2-63]L2-63_02114_L2-63_02114_Helix-turn-helix_domain_2180543:2180794_reverse_MW:9584	RbromiL2-63
Org5_Gene2053	accessory	771	[RbromiL2-63]L2-63_02115_L2-63_02115_Relaxase/Mobilisation_nuclease_domain_2180858:2182204_reverse_MW:50469	RbromiL2-63
Org5_Gene2054	accessory	3724	[RbromiL2-63]L2-63_02116_L2-63_02116_Bacterial_mobilisation_protein_(MobC)_2182165:2182494_reverse_MW:12518	RbromiL2-63
Org5_Gene2056	accessory	4294	[RbromiL2-63]L2-63_02118_L2-63_02118_hypothetical_protein_2183500:2183679_forward_MW:7062	RbromiL2-63
Org5_Gene2057	accessory	1452	[RbromiL2-63]L2-63_02119_rlmN_3_Ribosomal_RNA_large_subunit_methyltransferase_N,ribosomal_RNA_large_subunit_methyltransferase_N,23S_rRNA_	RbromiL2-63
Org5_Gene2058	accessory	3304	[RbromiL2-63]L2-63_02120_L2-63_02120_RNA_polymerase_sigma_factor,RNA_polymerase_sigma_factor,_sigma-70_family,Sigma-70,_region_4_2185230:	RbromiL2-63
Org5_Gene2059	accessory	3978	[RbromiL2-63]L2-63_02121_L2-63_02121_Helix-turn-helix_domain_2185651:2185911_forward_MW:9674	RbromiL2-63
Org5_Gene2060	accessory	447	[RbromiL2-63]L2-63_02122_L2-63_02122_Resolvase,_N_terminal_domain_2186657:2188333_forward_MW:65254	RbromiL2-63
Org5_Gene2061	accessory	4206	[RbromiL2-63]L2-63_02123_L2-63_02123_hypothetical_protein_2188382:2188585_reverse_MW:7540	RbromiL2-63
Org5_Gene2062	accessory	4053	[RbromiL2-63]L2-63_02124_L2-63_02124_hypothetical_protein_2188586:2188825_reverse_MW:9088	RbromiL2-63
Org5_Gene2063	accessory	1975	[RbromiL2-63]L2-63_02125_parB_5_Probable_chromosome-partitioning_protein_parB,plasmid_partitioning_protein_RepB,ParB/RepB/Spo0J_family_partit	RbromiL2-63
Org5_Gene2064	accessory	3679	[RbromiL2-63]L2-63_02126_L2-63_02126_Replication_initiator_protein_A_(RepA)_N-terminus_2189745:2190080_reverse_MW:13173	RbromiL2-63
Org5_Gene2065	accessory	4176	[RbromiL2-63]L2-63_02127_L2-63_02127_hypothetical_protein_2190177:2190386_reverse_MW:8080	RbromiL2-63
Org5_Gene2066	accessory	440	[RbromiL2-63]L2-63_02128_hsdM_Type_I_restriction_enzyme_EcoKI_M_protein,Type_I_restriction-modification_system_methyltransferase_subunit,type_	RbromiL2-63
Org5_Gene2067	accessory	1209	[RbromiL2-63]L2-63_02129_L2-63_02129_Type_I_restriction_modification_DNA_specificity_domain_2192646:2193758_forward_MW:42886	RbromiL2-63
Org5_Gene2068	accessory	362	[RbromiL2-63]L2-63_02130_L2-63_02130_KAP_family_P-loop_domain_2193774:2195591_forward_MW:70601	RbromiL2-63
Org5_Gene2069	accessory	62	[RbromiL2-63]L2-63_02131_hsdR_2_Type_I_restriction_enzyme_EcoR124II_R_protein,type_I_restriction_enzyme_EcoKI_subunit_R,Predicted_HKD_family	RbromiL2-63
Org5_Gene207	accessory	2121	[RbromiL2-63]L2-63_00211_L2-63_00211_Glycine/sarcosine_N-methyltransferase,bifunctional_3-demethylubiquinone-9_3-methyltransferase/_2-octapren	RbromiL2-63
Org5_Gene2070	accessory	3525	[RbromiL2-63]L2-63_02132_L2-63_02132_hypothetical_protein_2199285:2199659_reverse_MW:14471	RbromiL2-63
Org5_Gene2071	accessory	2340	[RbromiL2-63]L2-63_02133_L2-63_02133_hypothetical_protein_2200321:2201031_reverse_MW:26327	RbromiL2-63
Org5_Gene2072	accessory	2543	[RbromiL2-63]L2-63_02134_L2-63_02134_5'-nucleotidase,phosphoglycolate_phosphatase,phosphoglycolate_phosphatase,_bacterial,haloacid_dehalogena	RbromiL2-63
Org5_Gene2073	accessory	504	[RbromiL2-63]L2-63_02135_L2-63_02135_Lipid_A_core_-_O-antigen_ligase_and_related_enzymes,O-Antigen_ligase_2203212:2204816_forward_MW:599	RbromiL2-63
Org5_Gene2074	accessory	1828	[RbromiL2-63]L2-63_02136_pgaB_Poly-beta-1,6-N-acetyl-D-glucosamine_N-deacetylase_precursor,outer_membrane_N-deacetylase,poly-beta-1,6-N-acety	RbromiL2-63
Org5_Gene2075	accessory	3483	[RbromiL2-63]L2-63_02137_perR_Peroxide-responsive_repressor_perR,ferric_uptake_regulator,Ferric_uptake_regulator_family_2205842:2206234_forwar	RbromiL2-63
Org5_Gene2077	accessory	4372	[RbromiL2-63]L2-63_02139_L2-63_02139_Rubredoxin,anaerobic_nitric_oxide_reductase_flavorubredoxin,Rubredoxin,Rubredoxin_2206897:2207055_forw	RbromiL2-63
Org5_Gene2079	accessory	1208	[RbromiL2-63]L2-63_02141_L2-63_02141_Protein_of_unknown_function_(DUF2974)_2208075:2209187_reverse_MW:42367	RbromiL2-63
Org5_Gene2080	accessory	357	[RbromiL2-63]L2-63_02142_recQ_ATP-dependent_DNA_helicase_recQ,ATP-dependent_DNA_helicase_RecQ,Lhr-like_helicases,ATP-dependent_DNA_helic	RbromiL2-63
Org5_Gene2081	accessory	3546	[RbromiL2-63]L2-63_02143_L2-63_02143_hypothetical_protein_2211139:2211513_reverse_MW:14718	RbromiL2-63
Org5_Gene2082	accessory	3506	[RbromiL2-63]L2-63_02144_L2-63_02144_hypothetical_protein_2211674:2212060_reverse_MW:14659	RbromiL2-63
Org5_Gene2083	accessory	534	[RbromiL2-63]L2-63_02145_L2-63_02145_hypothetical_protein_2213021:2214574_reverse_MW:58267	RbromiL2-63
Org5_Gene2084	accessory	2754	[RbromiL2-63]L2-63_02146_L2-63_02146_hypothetical_protein_2214588:2215172_reverse_MW:21863	RbromiL2-63
Org5_Gene2085	accessory	3507	[RbromiL2-63]L2-63_02147_L2-63_02147_hypothetical_protein_2215189:2215575_reverse_MW:14173	RbromiL2-63
Org5_Gene2086	accessory	3446	[RbromiL2-63]L2-63_02148_L2-63_02148_hypothetical_protein_2215769:2216170_reverse_MW:13660	RbromiL2-63
Org5_Gene2087	accessory	2580	[RbromiL2-63]L2-63_02149_vraR_Response_regulator_protein_vraR,transcriptional_regulator_NarL,Response_regulator_of_citrate/malate_metabolism,tr	RbromiL2-63
Org5_Gene2088	accessory	847	[RbromiL2-63]L2-63_02150_L2-63_02150_sensory_histidine_kinase_UhpB,Signal_transduction_histidine_kinase_2216972:2218279_reverse_MW:48911	RbromiL2-63
Org5_Gene2089	accessory	454	[RbromiL2-63]L2-63_02151_L2-63_02151_hypothetical_protein_2218276:2219943_reverse_MW:63287	RbromiL2-63
Org5_Gene209	accessory	1911	[RbromiL2-63]L2-63_00213_L2-63_00213_Predicted_membrane_protein_231772:232608_reverse_MW:30415	RbromiL2-63
Org5_Gene2090	accessory	299	[RbromiL2-63]L2-63_02152_traG_3_Conjugal_transfer_protein_traG,conjugal_transfer_coupling_protein_TraG,Type_IV_secretory_pathway,_VirD4_compo	RbromiL2-63
Org5_Gene2091	accessory	1640	[RbromiL2-63]L2-63_02153_L2-63_02153_DNA_primase,DNA_primase_(bacterial_type),DNA_primase_2222279:2223199_reverse_MW:35512	RbromiL2-63
Org5_Gene2092	accessory	1464	[RbromiL2-63]L2-63_02154_L2-63_02154_hypothetical_protein_2223450:2224433_forward_MW:37906	RbromiL2-63
Org5_Gene2093	accessory	252	[RbromiL2-63]L2-63_02155_L2-63_02155_Putative_amidoligase_enzyme_2224507:2226591_reverse_MW:80055	RbromiL2-63
Org5_Gene2094	accessory	4059	[RbromiL2-63]L2-63_02156_L2-63_02156_hypothetical_protein_2226588:2226827_reverse_MW:9196	RbromiL2-63
Org5_Gene2095	accessory	1185	[RbromiL2-63]L2-63_02157_L2-63_02157_hypothetical_protein_2226842:2227879_reverse_MW:38269	RbromiL2-63
Org5_Gene2096	accessory	3077	[RbromiL2-63]L2-63_02158_pezA_Antitoxin_PezA,DNA-binding_transcriptional_repressor_PuuR,transcriptional_regulator,_y4mF_family,Helix-turn-helix_d	RbromiL2-63
Org5_Gene2097	accessory	1228	[RbromiL2-63]L2-63_02159_L2-63_02159_hypothetical_protein_2228625:2229728_reverse_MW:40696	RbromiL2-63
Org5_Gene2098	accessory	3654	[RbromiL2-63]L2-63_02160_L2-63_02160_Protein_of_unknown_function_(DUF3852)_2229775:2230119_reverse_MW:12573	RbromiL2-63
Org5_Gene2099	accessory	3636	[RbromiL2-63]L2-63_02161_L2-63_02161_hypothetical_protein_2230097:2230447_reverse_MW:13323	RbromiL2-63
Org5_Gene21	accessory	3362	[RbromiL2-63]L2-63_00021_L2-63_00021_hypothetical_protein_26981:27403_forward_MW:15857	RbromiL2-63
Org5_Gene210	accessory	1967	[RbromiL2-63]L2-63_00215_thiM_Hydroxyethylthiazole_kinase,hydroxyethylthiazole_kinase,Uncharacterized_conserved_protein,hydroxyethylthiazole_kin	RbromiL2-63
Org5_Gene2100	accessory	2244	[RbromiL2-63]L2-63_02162_L2-63_02162_Predicted_outer_membrane_protein,Cna_protein_B-type_domain_2230447:2231181_reverse_MW:27154	RbromiL2-63
Org5_Gene2101	accessory	22	[RbromiL2-63]L2-63_02163_L2-63_02163_Predicted_outer_membrane_protein,Cna_protein_B-type_domain_2231281:2232711_reverse_MW:51374	RbromiL2-63

Org5_Gene2102	accessory	2696	[RbromiL2-63]L2-63_02164_L2-63_02164_hypothetical_protein_2232791:2233390_reverse_MW:22209	RbromiL2-63
Org5_Gene2103	accessory	2148	[RbromiL2-63]L2-63_02165_L2-63_02165_hypothetical_protein_2233782:2234549_reverse_MW:29309	RbromiL2-63
Org5_Gene2104	accessory	3868	[RbromiL2-63]L2-63_02166_L2-63_02166_transcriptional_regulator,_AbrB_family_2234647:2234937_reverse_MW:11437	RbromiL2-63
Org5_Gene2105	accessory	4230	[RbromiL2-63]L2-63_02167_L2-63_02167_hypothetical_protein_2234937:2235134_reverse_MW:7147	RbromiL2-63
Org5_Gene2106	accessory	1451	[RbromiL2-63]L2-63_02168_dpnM_Modification_methylase_DpnIIA,Site-specific_DNA_methylase,DNA_adenine_methylase,D12_class_N6_adenine-specifi	RbromiL2-63
Org5_Gene2107	accessory	2057	[RbromiL2-63]L2-63_02169_L2-63_02169_DNA_cytosine_methylase,DNA_(cytosine-5-)-methyltransferase,C-5_cytosine-specific_DNA_methylase_2236236	RbromiL2-63
Org5_Gene2108	accessory	3266	[RbromiL2-63]L2-63_02170_hhaIM_2_Modification_methylase_HhaI,DNA_cytosine_methylase,DNA_(cytosine-5-)-methyltransferase,C-5_cytosine-specific_	RbromiL2-63
Org5_Gene2109	accessory	3776	[RbromiL2-63]L2-63_02171_L2-63_02171_hypothetical_protein_2237600:2237902_reverse_MW:11564	RbromiL2-63
Org5_Gene211	accessory	2565	[RbromiL2-63]L2-63_00216_thiE_Thiamine-phosphate_synthase,thiamine-phosphate_pyrophosphorylase,Thiamine_monophosphate_synthase,thiamine-pl	RbromiL2-63
Org5_Gene2110	accessory	4366	[RbromiL2-63]L2-63_02172_L2-63_02172_hypothetical_protein_2237987:2238139_reverse_MW:6195	RbromiL2-63
Org5_Gene2111	accessory	4414	[RbromiL2-63]L2-63_02173_L2-63_02173_hypothetical_protein_2238314:2238448_reverse_MW:5178	RbromiL2-63
Org5_Gene212	accessory	2498	[RbromiL2-63]L2-63_00217_L2-63_00217_Phosphorylated_carbohydrates_phosphatase_TM_1254,2-deoxyglucose-6-phosphatase,beta-phosphoglucomuta	RbromiL2-63
Org5_Gene213	accessory	1982	[RbromiL2-63]L2-63_00218_thiD_Hydroxymethylpyrimidine/phosphomethylpyrimidine_kinase,bifunctional_hydroxy-methylpyrimidine_kinase/_hydroxy-pl	RbromiL2-63
Org5_Gene214	accessory	1032	[RbromiL2-63]L2-63_00219_codB_Cytosine_permease,cytosine_permease,putative_hydroxymethylpyrimidine_transporter_CytX,Permease_for_cytosine/p	RbromiL2-63
Org5_Gene215	accessory	208	[RbromiL2-63]L2-63_00220_spoVT_Stage_V_sporulation_protein_T,stage_V_sporulation_protein_T,SpoVT_/_AbrB_like_domain_237414:237956_forward_	RbromiL2-63
Org5_Gene216	accessory	1246	[RbromiL2-63]L2-63_00221_L2-63_00221_hypothetical_protein_238126:239217_forward_MW:42212	RbromiL2-63
Org5_Gene217	accessory	3063	[RbromiL2-63]L2-63_00222_L2-63_00222_nicotinamidase/pyrazinamidase,Isochorismatase_family_239644:240144_reverse_MW:18367	RbromiL2-63
Org5_Gene218	accessory	2950	[RbromiL2-63]L2-63_00223_gpmA_2,3-bisphosphoglycerate-dependent_phosphoglycerate_mutase,phosphoglycerate_mutase,alpha-ribazole_phosphatase	RbromiL2-63
Org5_Gene219	accessory	788	[RbromiL2-63]L2-63_00224_ktrB_Ktr_system_potassium_uptake_protein_B,potassium_uptake_protein,_TrkH_family,Cation_transport_protein_241437:24	RbromiL2-63
Org5_Gene220	accessory	2553	[RbromiL2-63]L2-63_00225_ktrA_Ktr_system_potassium_uptake_protein_A,potassium_transporter_peripheral_membrane_component,Kef-type_K__trans	RbromiL2-63
Org5_Gene221	accessory	4010	[RbromiL2-63]L2-63_00226_L2-63_00226_hypothetical_protein_243453:243707_forward_MW:9697	RbromiL2-63
Org5_Gene222	accessory	286	[RbromiL2-63]L2-63_00227_kdpD_Sensor_protein_KdpD,sensor_protein_KdpD,Predicted_periplasmic_ligand-binding_sensor_domain,phosphate_regulon_	RbromiL2-63
Org5_Gene223	accessory	2378	[RbromiL2-63]L2-63_00228_kdpE_KDP_operon_transcriptional_regulatory_protein_KdpE,DNA-binding_transcriptional_activator_KdpE,Response_regulato	RbromiL2-63
Org5_Gene224	accessory	1029	[RbromiL2-63]L2-63_00229_Int-Tn_1_Integrase,site-specific_tyrosine_recombinase_XerC,Site-specific_recombinase_XerC,tyrosine_recombinase_XerC,Pha	RbromiL2-63
Org5_Gene225	accessory	3450	[RbromiL2-63]L2-63_00230_L2-63_00230_hypothetical_protein_248165:248566_reverse_MW:14943	RbromiL2-63
Org5_Gene226	accessory	2811	[RbromiL2-63]L2-63_00231_L2-63_00231_Domain_of_unknown_function_(DUF955)_248747:249316_forward_MW:22286	RbromiL2-63
Org5_Gene227	accessory	2415	[RbromiL2-63]L2-63_00232_immR_5_HTH-type_transcriptional_regulator_immR,transcriptional_repressor_DicA,Helix-turn-helix_249282:249971_reverse_	RbromiL2-63
Org5_Gene228	accessory	4082	[RbromiL2-63]L2-63_00233_L2-63_00233_Helix-turn-helix_domain_250211:250444_forward_MW:8925	RbromiL2-63
Org5_Gene229	accessory	4144	[RbromiL2-63]L2-63_00234_L2-63_00234_hypothetical_protein_250485:250703_forward_MW:8329	RbromiL2-63
Org5_Gene230	accessory	4290	[RbromiL2-63]L2-63_00235_L2-63_00235_hypothetical_protein_250696:250881_forward_MW:6898	RbromiL2-63
Org5_Gene231	accessory	4097	[RbromiL2-63]L2-63_00236_L2-63_00236_hypothetical_protein_250853:251083_reverse_MW:8856	RbromiL2-63
Org5_Gene232	accessory	4181	[RbromiL2-63]L2-63_00237_L2-63_00237_transcriptional_regulator,_y4mF_family,Helix-turn-helix_251169:251378_forward_MW:7863	RbromiL2-63
Org5_Gene233	accessory	4266	[RbromiL2-63]L2-63_00238_L2-63_00238_hypothetical_protein_251383:251574_forward_MW:7039	RbromiL2-63
Org5_Gene234	accessory	4228	[RbromiL2-63]L2-63_00239_L2-63_00239_DNA_binding_domain,_excisionase_family,Helix-turn-helix_domain_251587:251784_forward_MW:7795	RbromiL2-63
Org5_Gene235	accessory	4073	[RbromiL2-63]L2-63_00240_L2-63_00240_hypothetical_protein_251787:252023_forward_MW:9157	RbromiL2-63
Org5_Gene236	accessory	3301	[RbromiL2-63]L2-63_00241_L2-63_00241_hypothetical_protein_252020:252460_forward_MW:16185	RbromiL2-63
Org5_Gene237	accessory	549	[RbromiL2-63]L2-63_00242_L2-63_00242_hypothetical_protein_252474:254012_forward_MW:60079	RbromiL2-63
Org5_Gene238	accessory	1474	[RbromiL2-63]L2-63_00243_L2-63_00243_hypothetical_protein_253981:254958_forward_MW:38147	RbromiL2-63
Org5_Gene239	accessory	2716	[RbromiL2-63]L2-63_00244_L2-63_00244_Siphovirus_Gp157_254971:255564_forward_MW:22608	RbromiL2-63
Org5_Gene24	accessory	452	[RbromiL2-63]L2-63_00027_pinR_Putative_DNA-invertase_from_lambdoid_prophage_Rac,multiple_promoter_invertase,Transposase_and_inactivated_de	RbromiL2-63
Org5_Gene240	accessory	1673	[RbromiL2-63]L2-63_00245_L2-63_00245_hypothetical_protein_255575:256483_forward_MW:33584	RbromiL2-63
Org5_Gene241	accessory	784	[RbromiL2-63]L2-63_00246_L2-63_00246_type_I_restriction_enzyme_EcoKI_subunit_R,Predicted_HKD_family_nuclease,DNA_phosphorothioation_system	RbromiL2-63
Org5_Gene242	accessory	3784	[RbromiL2-63]L2-63_00247_L2-63_00247_VRR-NUC_domain_257795:258109_forward_MW:11528	RbromiL2-63
Org5_Gene243	accessory	2845	[RbromiL2-63]L2-63_00248_L2-63_00248_Protein_of_unknown_function_(DUF669)_258112:258672_forward_MW:21037	RbromiL2-63
Org5_Gene244	accessory	186	[RbromiL2-63]L2-63_00249_L2-63_00249_hypothetical_protein,Uncharacterized_conserved_protein,phage/plasmid_primase,_P4_family,_C-terminal_dom	RbromiL2-63
Org5_Gene245	accessory	3417	[RbromiL2-63]L2-63_00250_L2-63_00250_hypothetical_protein_261206:261616_forward_MW:16282	RbromiL2-63
Org5_Gene246	accessory	3071	[RbromiL2-63]L2-63_00251_L2-63_00251_hypothetical_protein_261622:262119_forward_MW:19207	RbromiL2-63
Org5_Gene247	accessory	4336	[RbromiL2-63]L2-63_00252_L2-63_00252_hypothetical_protein_262116:262289_forward_MW:6878	RbromiL2-63
Org5_Gene248	accessory	3414	[RbromiL2-63]L2-63_00253_L2-63_00253_hypothetical_protein_262296:262706_forward_MW:16056	RbromiL2-63
Org5_Gene249	accessory	471	[RbromiL2-63]L2-63_00254_L2-63_00254_phage_terminase,_large_subunit,_PBSX_family,Phage_terminase_large_subunit_262888:264540_forward_MW:	RbromiL2-63
Org5_Gene25	accessory	4383	[RbromiL2-63]L2-63_00028_L2-63_00028_hypothetical_protein_33663:33815_reverse_MW:5780	RbromiL2-63
Org5_Gene250	accessory	814	[RbromiL2-63]L2-63_00255_L2-63_00255_phage_portal_protein,_putative,_A118_family,Phage_portal_protein,_SPP1_Gp6-like_264540:265865_forward_	RbromiL2-63
Org5_Gene251	accessory	269	[RbromiL2-63]L2-63_00256_L2-63_00256_Putative_ADP-ribosyltransferase_Certhrax,Phage_minor_capsid_protein_2_265865:267904_forward_MW:77434	RbromiL2-63
Org5_Gene252	accessory	3749	[RbromiL2-63]L2-63_00257_L2-63_00257_hypothetical_protein_267901:268227_forward_MW:12497	RbromiL2-63
Org5_Gene253	accessory	3436	[RbromiL2-63]L2-63_00258_L2-63_00258_hypothetical_protein_268419:268823_forward_MW:15179	RbromiL2-63
Org5_Gene254	accessory	4218	[RbromiL2-63]L2-63_00259_L2-63_00259_hypothetical_protein_268870:269070_forward_MW:7481	RbromiL2-63
Org5_Gene255	accessory	2823	[RbromiL2-63]L2-63_00260_L2-63_00260_Phage_minor_structural_protein_GP20_269184:269750_forward_MW:20630	RbromiL2-63
Org5_Gene256	accessory	1784	[RbromiL2-63]L2-63_00261_L2-63_00261_hypothetical_protein_269780:270655_forward_MW:31867	RbromiL2-63
Org5_Gene257	accessory	3561	[RbromiL2-63]L2-63_00262_L2-63_00262_hypothetical_protein_270668:271039_forward_MW:13703	RbromiL2-63
Org5_Gene258	accessory	3372	[RbromiL2-63]L2-63_00263_L2-63_00263_hypothetical_protein_271036:271458_forward_MW:15725	RbromiL2-63
Org5_Gene259	accessory	3128	[RbromiL2-63]L2-63_00264_L2-63_00264_hypothetical_protein_271463:271945_forward_MW:17893	RbromiL2-63
Org5_Gene26	accessory	3609	[RbromiL2-63]L2-63_00029_ndoA_1_mRNA_interferase_EndoA,toxin_MazF,PemK-like_protein_33927:34283_reverse_MW:13111	RbromiL2-63
Org5_Gene260	accessory	3298	[RbromiL2-63]L2-63_00265_L2-63_00265_hypothetical_protein_271942:272382_forward_MW:16403	RbromiL2-63
Org5_Gene261	accessory	3051	[RbromiL2-63]L2-63_00266_L2-63_00266_hypothetical_protein_272385:272888_forward_MW:18413	RbromiL2-63
Org5_Gene262	accessory	3385	[RbromiL2-63]L2-63_00267_L2-63_00267_hypothetical_protein_272910:273329_forward_MW:15700	RbromiL2-63
Org5_Gene263	accessory	2891	[RbromiL2-63]L2-63_00268_L2-63_00268_Bacteriophage_Gp15_protein_273326:273871_forward_MW:21098	RbromiL2-63
Org5_Gene264	accessory	2710	[RbromiL2-63]L2-63_00269_L2-63_00269_hypothetical_protein_273965:274561_forward_MW:21894	RbromiL2-63
Org5_Gene265	accessory	43	[RbromiL2-63]L2-63_00270_L2-63_00270_Phage-related_protein_274674:278339_forward_MW:132574	RbromiL2-63
Org5_Gene266	accessory	3392	[RbromiL2-63]L2-63_00271_L2-63_00271_hypothetical_protein_278351:278767_forward_MW:16287	RbromiL2-63
Org5_Gene267	accessory	870	[RbromiL2-63]L2-63_00272_L2-63_00272_hypothetical_protein_278780:280072_forward_MW:47989	RbromiL2-63
Org5_Gene268	accessory	2127	[RbromiL2-63]L2-63_00273_L2-63_00273_hypothetical_protein_280076:280849_forward_MW:27820	RbromiL2-63
Org5_Gene27	accessory	3346	[RbromiL2-63]L2-63_00030_L2-63_00030_RNA_polymerase_sigma_factor_SigX,RNA_polymerase_sigma_factor,_sigma-70_family_34869:35297_reverse_M	RbromiL2-63
Org5_Gene270	accessory	3262	[RbromiL2-63]L2-63_00275_L2-63_00275_Phage-related_holin_(Lysis_protein),toxin_secretion/phage_lysis_holin,Holin_family_281116:281565_forward_M	RbromiL2-63
Org5_Gene271	accessory	1910	[RbromiL2-63]L2-63_00276_L2-63_00276_N-acetylmuramoyl-L-alanine_amidase_281568:282407_forward_MW:30775	RbromiL2-63
Org5_Gene272	accessory	2039	[RbromiL2-63]L2-63_00281_ydaD_General_stress_protein_39,3-ketoacyl-(acyl-carrier-protein)_reductase,Uncharacterized_conserved_protein,3-oxoacyl-[a	RbromiL2-63
Org5_Gene273	accessory	4345	[RbromiL2-63]L2-63_00282_L2-63_00282_hypothetical_protein_284594:284764_forward_MW:6714	RbromiL2-63
Org5_Gene274	accessory	1777	[RbromiL2-63]L2-63_00283_spoVFA_Stage_V_sporulation_protein_FA,dipicolinate_synthase_subunit_A,dipicolinic_acid_synthetase,_A_subunit_284892:2	RbromiL2-63
Org5_Gene276	accessory	473	[RbromiL2-63]L2-63_00285_L2-63_00285_hypothetical_protein_286456:288105_forward_MW:58966	RbromiL2-63
Org5_Gene277	accessory	4177	[RbromiL2-63]L2-63_00286_L2-63_00286_Predicted_transcriptional_regulator,putative_zinc_finger/helix-turn-helix_protein,_YgiT_family,Helix-turn-helix_	RbromiL2-63
Org5_Gene278	accessory	670	[RbromiL2-63]L2-63_00287_licB_Beta-glucanase_precursor_288495:289910_forward_MW:53531	RbromiL2-63
Org5_Gene279	accessory	2966	[RbromiL2-63]L2-63_00288_L2-63_00288_hypothetical_protein_290034:290564_forward_MW:19744	RbromiL2-63
Org5_Gene28	accessory	4275	[RbromiL2-63]L2-63_00031_L2-63_00031_hypothetical_protein_35771:35959_reverse_MW:7283	RbromiL2-63
Org5_Gene286	accessory	2261	[RbromiL2-63]L2-63_00295_spoIIQ_Stage_II_sporulation_protein_Q,Membrane-bound_metallopeptidase,Peptidase_family_M23_296471:297199_forward	RbromiL2-63
Org5_Gene287	accessory	2592	[RbromiL2-63]L2-63_00296_L2-63_00296_cytidylate_kinase,putative_cytidylate_kinase_297491:298123_reverse_MW:23602	RbromiL2-63
Org5_Gene288	accessory	2585	[RbromiL2-63]L2-63_00297_L2-63_00297_phosphoglycolate_phosphatase,phosphoglycolate_phosphatase,_bacterial,haloacid_dehalogenase-like_hydrolas	RbromiL2-63
Org5_Gene289	accessory	3211	[RbromiL2-63]L2-63_00298_L2-63_00298_Transposase_and_inactivated_derivatives,Transposase_IS200_like_300614:300904_reverse_MW:11159	RbromiL2-63
Org5_Gene29	accessory	711	[RbromiL2-63]L2-63_00032_mepA_1_Multidrug_export_protein_mepA,multidrug_efflux_pump_VmrA,MATE_efflux_family_protein,MatE_35972:37357_re	RbromiL2-63
Org5_Gene291	accessory	2759	[RbromiL2-63]L2-63_00303_L2-63_00303_hypothetical_protein_306886:307470_reverse_MW:22223	RbromiL2-63
Org5_Gene293	accessory	993	[RbromiL2-63]L2-63_00309_L2-63_00309_Transposase_and_inactivated_derivatives,Transposase_308750:309235_forward_MW:19036	RbromiL2-63
Org5_Gene295	accessory	4229	[RbromiL2-63]L2-63_00312_sspC2_SSP-3,Small,_acid-soluble_spore_proteins,_alpha/beta_type_312723:312920_reverse_MW:7173	RbromiL2-63
Org5_Gene296	accessory	151	[RbromiL2-63]L2-63_00313_L2-63_00313_X-X-X-Leu-X-X-Gly_heptad_repeats_313186:315615_reverse_MW:84582	RbromiL2-63
Org5_Gene298	accessory	2828	[RbromiL2-63]L2-63_00315_L2-63_00315_probable_dihydroxyacetone_kinase_regulator,Bacterial_regulatory_proteins,_tetR_family_317968:318534_reve	RbromiL2-63
Org5_Gene299	accessory	2600	[RbromiL2-63]L2-63_00316_sfp_4'-phosphopantetheinyl_transferase_sfp,holo-(acyl_carrier_protein)_synthase_2,4'-phosphopantetheinyl_transferase_sup	RbromiL2-63
Org5_Gene3	accessory	2562	[RbromiL2-63]L2-63_00003_L2-63_00003_Uncharacterized_protein_encoded_in_toxicity_protection_region_of_plasmid_R478,_contains_von_Willebrand_	RbromiL2-63
Org5_Gene30	accessory	2973	[RbromiL2-63]L2-63_00033_L2-63_00033_transcriptional_regulator,_Acidobacterial,_PadR-family,Transcriptional_regulator_PadR-like_family_37480:3800	RbromiL2-63
Org5_Gene300	accessory	3106	[RbromiL2-63]L2-63_00317_L2-63_00317_ComE_operon_protein_2,Cytidine_and_deoxycytidylate_deaminase_zinc-binding_region_319408:319896_revers	RbromiL2-63
Org5_Gene304	accessory	3624	[RbromiL2-63]L2-63_00321_L2-63_00321_hypothetical_protein_321640:321873_reverse_MW:8770	RbromiL2-63
Org5_Gene305	accessory	1245	[RbromiL2-63]L2-63_00322_L2-63_00322_Predicted_membrane_protein_322105:323196_reverse_MW:40584	RbromiL2-63
Org5_Gene306	accessory	1336	[RbromiL2-63]L2-63_00323_baeS_Signal_transduction_histidine-protein_kinase_BaeS,sensory_histidine_kinase_CreC,Predicted_periplasmic_ligand-bindin	RbromiL2-63
Org5_Gene307	accessory	2466	[RbromiL2-63]L2-63_00324_hssR_Heme_response_regulator_hssR,transcriptional_regulatory_protein_TctD,Response_regulator_of_citrate/malate_metab	RbromiL2-63
Org5_Gene308	accessory	777	[RbromiL2-63]L2-63_00325_mepA_2_Multidrug_export_protein_mepA,multidrug_efflux_pump_VmrA,MATE_efflux_family_protein,MatE_325574:326917_	RbromiL2-63
Org5_Gene31	accessory	1326	[RbromiL2-63]L2-63_00034_L2-63_00034_Predicted_choloylglycine_hydrolase,Acyl-coenzyme_A:6-aminopenicillanic_acid_acyl-transferase_38274:39323_	RbromiL2-63
Org5_Gene311	accessory	1945	[RbromiL2-63]L2-63_00328_L2-63_00328_hypothetical_protein_329746:330573_reverse_MW:31307	RbromiL2-63
Org5_Gene312	accessory	825	[RbromiL2-63]L2-63_00329_envC_Septal_ring_factor,AmiB_activator,Membrane-bound_metallopeptidase,chromosome_segregation_protein_SMC,Peptid	RbromiL2-63
Org5_Gene313	accessory	1709	[RbromiL2-63]L2-63_00330_ftsX_Cell_division_protein_FtsX,cell_division_ABC_transporter_subunit_FtsX,putative_protein_insertion_permease_FtsX,FtsX-	RbromiL2-63
Org5_Gene317	accessory	2298	[RbromiL2-63]L2-63_00334_rsuA_Ribosomal_small_subunit_pseudouridine_synthase_A,16S_rRNA_pseudouridylate_synthase_A,pseudouridine_synthase,	RbromiL2-63
Org5_Gene318	accessory	3180	[RbromiL2-63]L2-63_00335_L2-63_00335_hypothetical_protein_337296:337766_reverse_MW:17270	RbromiL2-63
Org5_Gene319	accessory	2489	[RbromiL2-63]L2-63_00336_L2-63_00336_5'-nucleotidase,phosphoglycolate_phosphatase,phosphoglycolate_phosphatase,_bacterial,haloacid_dehalogena	RbromiL2-63
Org5_Gene32	accessory	1816	[RbromiL2-63]L2-63_00035_tetD_1_hypothetical_protein,DNA-binding_transcriptional_regulator_SoxS,DNA_gyrase_inhibitor,YSIRK-targeted_surface_anti	RbromiL2-63
Org5_Gene322	accessory	4416	[RbromiL2-63]L2-63_00339_L2-63_00339_hypothetical_protein_340269:340397_forward_MW:4166	RbromiL2-63
Org5_Gene326	accessory	2630	[RbromiL2-63]L2-63_00343_L2-63_00343_Predicted_membrane_protein,Protein_of_unknown_function_(DUF3816)_343515:344132_forward_MW:21611	RbromiL2-63
Org5_Gene327	accessory	1502	[RbromiL2-63]L2-63_00344_birA_Bifunctional_protein_BirA,bifunctional_biotin--[acetyl-CoA-carboxylase]_synthetase/biotin_operon_repressor,Uncharact	RbromiL2-63
Org5_Gene328	accessory	3961	[RbromiL2-63]L2-63_00345_L2-63_00345_hypothetical_protein_345501:345764_reverse_MW:10064	RbromiL2-63
Org5_Gene33	accessory	3604	[RbromiL2-63]L2-63_00036_L2-63_00036_anaerobic_benzoate_catabolism_transcriptional_regulator,transcriptional_regulator,_y4mF_family,Helix-turn-he	RbromiL2-63
Org5_Gene330	accessory	3094	[RbromiL2-63]L2-63_00347_L2-63_00347_hypothetical_protein_346218:346709_reverse_MW:17696	RbromiL2-63
Org5_Gene331	accessory	2144	[RbromiL2-63]L2-63_00348_L2-63_00348_hypothetical_protein_346755:347522_reverse_MW:27811	RbromiL2-63
Org5_Gene332	accessory	1412	[RbromiL2-63]L2-63_00349_L2-63_00349_hypothetical_protein_347606:348613_reverse_MW:37531	RbromiL2-63
Org5_Gene334	accessory	2306	[RbromiL2-63]L2-63_00351_L2-63_00351_phosphoglycolate_phosphatase,HAD_hydrolase_352736:353452_reverse_MW:27442	RbromiL2-63
Org5_Gene336	accessory	3047	[RbromiL2-63]L2-63_00353_L2-63_00353_hypothetical_protein_355039:355542_reverse_MW:17407	RbromiL2-63
Org5_Gene337	accessory	2250	[RbromiL2-63]L2-63_00354_mrkE_hypothetical_protein,putative_two-component_response-regulatory_protein_YehT,Response_regulator_of_the_LytR/A	RbromiL2-63
Org5_Gene339	accessory	841	[RbromiL2-63]L2-63_00356_L2-63_00356_Predicted_signal_transduction_protein_with_a_C-terminal_ATPase_domain_356930:357844_forward_MW:345	RbromiL2-63
Org5_Gene34	accessory	381	[RbromiL2-63]L2-63_00037_L2-63_00037_conserved_hypothetical_protein,CHAP_domain_40833:42608_reverse_MW:63719	RbromiL2-63

Org5_Gene340	accessory	968	[RbromiL2-63]L2-63_00357_L2-63_00357_CHAP_domain_357962:359173_reverse_MW:43457	RbromiL2-63
Org5_Gene341	accessory	3584	[RbromiL2-63]L2-63_00358_L2-63_00358_hypothetical_protein,four_helix_bundle_protein,S23_ribosomal_protein_359477:359839_reverse_MW:13829	RbromiL2-63
Org5_Gene343	accessory	2624	[RbromiL2-63]L2-63_00362_slt_Soluble_lytic_murein_transglycosylase_precursor,lytic_murein_transglycosylase,Transglycosylase_SLT_domain_361742:362	RbromiL2-63
Org5_Gene344	accessory	2647	[RbromiL2-63]L2-63_00363_coaE_Dephospho-CoA_kinase,dephospho-CoA_kinase,Dephospho-CoA_kinase,dephospho-CoA_kinase,Dephospho-CoA_kinase	RbromiL2-63
Org5_Gene348	accessory	2548	[RbromiL2-63]L2-63_00367_L2-63_00367_hypothetical_protein_365395:366042_reverse_MW:23947	RbromiL2-63
Org5_Gene35	accessory	2129	[RbromiL2-63]L2-63_00038_L2-63_00038_sortase,_SrtB_family,Sortase_family_42644:43417_reverse_MW:29114	RbromiL2-63
Org5_Gene352	accessory	2780	[RbromiL2-63]L2-63_00371_L2-63_00371_probable_dihydroxyacetone_kinase_regulator_371572:372147_forward_MW:21931	RbromiL2-63
Org5_Gene353	accessory	856	[RbromiL2-63]L2-63_00372_ybaL_Inner_membrane_protein_ybaL,putative_cation:proton_antiport_protein,Kef-type_K__transport_system,_predicted_NA	RbromiL2-63
Org5_Gene354	accessory	3398	[RbromiL2-63]L2-63_00373_L2-63_00373_hypothetical_protein_373717:374133_reverse_MW:15558	RbromiL2-63
Org5_Gene355	accessory	1942	[RbromiL2-63]L2-63_00374_L2-63_00374_hypothetical_protein_374143:374973_reverse_MW:31048	RbromiL2-63
Org5_Gene356	accessory	1471	[RbromiL2-63]L2-63_00375_ansA_L-asparaginase_1,glutamyl-tRNA(Gln)_amidotransferase_subunit_D,L-asparaginase,_type_I,Asparaginase_375158:37612	RbromiL2-63
Org5_Gene357	accessory	3201	[RbromiL2-63]L2-63_00376_L2-63_00376_Predicted_flavin-nucleotide-binding_protein,PPOX_class_probable_F420-dependent_enzyme,Pyridoxamine_5'-p	RbromiL2-63
Org5_Gene358	accessory	872	[RbromiL2-63]L2-63_00377_L2-63_00377_Uncharacterized_conserved_protein,Protein_of_unknown_function_(DUF1015)_376636:377925_reverse_MW:4	RbromiL2-63
Org5_Gene359	accessory	1573	[RbromiL2-63]L2-63_00379_L2-63_00379_Uncharacterized_conserved_protein,Fic/DOC_family_378486:379427_reverse_MW:35725	RbromiL2-63
Org5_Gene36	accessory	3432	[RbromiL2-63]L2-63_00039_L2-63_00039_hypothetical_protein_43432:43836_reverse_MW:15973	RbromiL2-63
Org5_Gene360	accessory	3748	[RbromiL2-63]L2-63_00380_L2-63_00380_hypothetical_protein_379647:379895_reverse_MW:9467	RbromiL2-63
Org5_Gene361	accessory	184	[RbromiL2-63]L2-63_00381_spoIIE_Stage_II_sporulation_protein_E,stage_II_sporulation_protein_E,Stage_II_sporulation_protein_E_(SpoIIE)_380299:3826	RbromiL2-63
Org5_Gene363	accessory	4058	[RbromiL2-63]L2-63_00383_immR_6_HTH-type_transcriptional_regulator_immR,Predicted_transcriptional_regulator,putative_zinc_finger/helix-turn-helix_	RbromiL2-63
Org5_Gene366	accessory	3227	[RbromiL2-63]L2-63_00386_L2-63_00386_hypothetical_protein_391796:392254_reverse_MW:17756	RbromiL2-63
Org5_Gene368	accessory	478	[RbromiL2-63]L2-63_00388_rsbU_Phosphoserine_phosphatase_rsbU,stage_II_sporulation_protein_E,Stage_II_sporulation_protein_E_(SpoIIE)_393426:395	RbromiL2-63
Org5_Gene369	accessory	500	[RbromiL2-63]L2-63_00389_sleB_Germination-specific_amidase,Uncharacterized_conserved_protein,spore_cortex-lytic_enzyme,Putative_peptidoglycan_	RbromiL2-63
Org5_Gene37	accessory	181	[RbromiL2-63]L2-63_00040_L2-63_00040_conjugal_transfer_ATP-binding_protein_TraC,Type_IV_secretory_pathway,_VirB4_components,type-IV_secretio	RbromiL2-63
Org5_Gene372	accessory	2061	[RbromiL2-63]L2-63_00392_L2-63_00392_hypothetical_protein_401631:402422_reverse_MW:28440	RbromiL2-63
Org5_Gene373	accessory	1169	[RbromiL2-63]L2-63_00393_L2-63_00393_hypothetical_protein,Protein_of_unknown_function_(DUF795)_402702:403838_reverse_MW:41030	RbromiL2-63
Org5_Gene376	accessory	3522	[RbromiL2-63]L2-63_00396_L2-63_00396_hypothetical_protein_405727:406107_reverse_MW:14194	RbromiL2-63
Org5_Gene377	accessory	1584	[RbromiL2-63]L2-63_00397_L2-63_00397_auxin_efflux_carrier,Membrane_transport_protein_406257:407195_forward_MW:33922	RbromiL2-63
Org5_Gene38	accessory	3255	[RbromiL2-63]L2-63_00041_L2-63_00041_PrgI_family_protein_46169:46621_reverse_MW:17386	RbromiL2-63
Org5_Gene380	accessory	1752	[RbromiL2-63]L2-63_00400_L2-63_00400_hypothetical_protein_410161:411030_reverse_MW:31605	RbromiL2-63
Org5_Gene381	accessory	1035	[RbromiL2-63]L2-63_00401_L2-63_00401_hypothetical_protein_411088:412284_reverse_MW:44138	RbromiL2-63
Org5_Gene384	accessory	889	[RbromiL2-63]L2-63_00404_L2-63_00404_hypothetical_protein_416602:417882_reverse_MW:47902	RbromiL2-63
Org5_Gene388	accessory	635	[RbromiL2-63]L2-63_00408_L2-63_00408_Predicted_ATPase_(AAA__superfamily),Archaeal_ATPase_422987:424438_forward_MW:55795	RbromiL2-63
Org5_Gene389	accessory	1151	[RbromiL2-63]L2-63_00409_L2-63_00409_FtsX-like_permease_family_424490:425632_reverse_MW:42255	RbromiL2-63
Org5_Gene390	accessory	2400	[RbromiL2-63]L2-63_00410_macB_1_Macrolide_export_ATP-binding/permease_protein_MacB,DL-methionine_transporter_ATP-binding_subunit,Predicte	RbromiL2-63
Org5_Gene391	accessory	2786	[RbromiL2-63]L2-63_00411_L2-63_00411_hypothetical_protein_426329:426904_reverse_MW:21522	RbromiL2-63
Org5_Gene392	accessory	3005	[RbromiL2-63]L2-63_00412_L2-63_00412_hypothetical_protein_427145:427624_reverse_MW:18205	RbromiL2-63
Org5_Gene393	accessory	1302	[RbromiL2-63]L2-63_00413_L2-63_00413_Uncharacterized_homolog_of_phage_Mu_protein_gp47,conserved_hypothetical_protein,Baseplate_J-like_prote	RbromiL2-63
Org5_Gene394	accessory	3727	[RbromiL2-63]L2-63_00414_L2-63_00414_hypothetical_protein_428669:428998_reverse_MW:11825	RbromiL2-63
Org5_Gene395	accessory	3726	[RbromiL2-63]L2-63_00415_L2-63_00415_Mu-like_prophage_protein_gp45_429000:429329_reverse_MW:11275	RbromiL2-63
Org5_Gene396	accessory	1534	[RbromiL2-63]L2-63_00416_L2-63_00416_Mu-like_prophage_tail_protein_gpP_429304:430260_reverse_MW:35051	RbromiL2-63
Org5_Gene397	accessory	3009	[RbromiL2-63]L2-63_00417_L2-63_00417_Mu-like_prophage_DNA_circulation_protein_430254:430769_reverse_MW:19758	RbromiL2-63
Org5_Gene398	accessory	4389	[RbromiL2-63]L2-63_00418_L2-63_00418_hypothetical_protein_430766:430915_reverse_MW:5861	RbromiL2-63
Org5_Gene399	accessory	3579	[RbromiL2-63]L2-63_00419_L2-63_00419_hypothetical_protein_430912:431277_reverse_MW:13730	RbromiL2-63
Org5_Gene4	accessory	735	[RbromiL2-63]L2-63_00004_L2-63_00004_Predicted_membrane_protein_3774:5141_reverse_MW:52001	RbromiL2-63
Org5_Gene40	accessory	2436	[RbromiL2-63]L2-63_00043_L2-63_00043_hypothetical_protein_47634:48314_forward_MW:26281	RbromiL2-63
Org5_Gene400	accessory	3144	[RbromiL2-63]L2-63_00420_L2-63_00420_hypothetical_protein_431278:431709_reverse_MW:15641	RbromiL2-63
Org5_Gene401	accessory	3603	[RbromiL2-63]L2-63_00421_L2-63_00421_hypothetical_protein_431696:432055_reverse_MW:13737	RbromiL2-63
Org5_Gene402	accessory	3620	[RbromiL2-63]L2-63_00422_L2-63_00422_hypothetical_protein_432052:432405_reverse_MW:13010	RbromiL2-63
Org5_Gene404	accessory	3684	[RbromiL2-63]L2-63_00424_L2-63_00424_hypothetical_protein_433444:433776_reverse_MW:11470	RbromiL2-63
Org5_Gene405	accessory	1431	[RbromiL2-63]L2-63_00425_L2-63_00425_hypothetical_protein_433790:434785_reverse_MW:37069	RbromiL2-63
Org5_Gene406	accessory	1126	[RbromiL2-63]L2-63_00426_L2-63_00426_phage_portal_protein,_HK97_family_434775:435929_reverse_MW:42250	RbromiL2-63
Org5_Gene408	accessory	3187	[RbromiL2-63]L2-63_00428_L2-63_00428_Terminase_small_subunit_437175:437645_reverse_MW:17085	RbromiL2-63
Org5_Gene409	accessory	3803	[RbromiL2-63]L2-63_00429_trxA_1_Thioredoxin-M,thioredoxin,Thiol:disulfide_interchange_protein,thioredoxin,Thioredoxin_437904:438212_reverse_MW	RbromiL2-63
Org5_Gene41	accessory	3601	[RbromiL2-63]L2-63_00044_L2-63_00044_hypothetical_protein_48362:48718_reverse_MW:12790	RbromiL2-63
Org5_Gene410	accessory	4222	[RbromiL2-63]L2-63_00430_L2-63_00430_Ferredoxin-thioredoxin_reductase,_catalytic_subunit,Ferredoxin_thioredoxin_reductase_catalytic_beta_chain_4	RbromiL2-63
Org5_Gene411	accessory	3619	[RbromiL2-63]L2-63_00431_L2-63_00431_hypothetical_protein_439041:439394_forward_MW:13333	RbromiL2-63
Org5_Gene412	accessory	3195	[RbromiL2-63]L2-63_00432_L2-63_00432_Acetyltransferase_(GNAT)_family_439704:440171_forward_MW:18179	RbromiL2-63
Org5_Gene414	accessory	468	[RbromiL2-63]L2-63_00434_amyE_1_Alpha-amylase_precursor,triple_tyrosine_motif-containing_protein_442939:444585_reverse_MW:58568	RbromiL2-63
Org5_Gene416	accessory	462	[RbromiL2-63]L2-63_00436_L2-63_00436_Papain_family_cysteine_protease_446591:448243_reverse_MW:60430	RbromiL2-63
Org5_Gene418	accessory	1233	[RbromiL2-63]L2-63_00438_L2-63_00438_stage_II_sporulation_protein_P,Stage_II_sporulation_protein_P_(SpoIIP)_449541:450638_forward_MW:40615	RbromiL2-63
Org5_Gene42	accessory	372	[RbromiL2-63]L2-63_00045_traG_1_Conjugal_transfer_protein_traG,type_IV_secretion_system_component_VirD4,Type_IV_secretory_pathway,_VirD4_co	RbromiL2-63
Org5_Gene420	accessory	651	[RbromiL2-63]L2-63_00440_trkA_Trk_system_potassium_uptake_protein_trkA,potassium_transporter_peripheral_membrane_component,Kef-type_K__tr	RbromiL2-63
Org5_Gene423	accessory	1545	[RbromiL2-63]L2-63_00443_ldhP_L-lactate_dehydrogenase_P,L-lactate_dehydrogenase,L-lactate_dehydrogenase,lactate/malate_dehydrogenase,_alpha/b	RbromiL2-63
Org5_Gene425	accessory	2344	[RbromiL2-63]L2-63_00445_L2-63_00445_transcriptional_regulator_FixK_458664:459311_reverse_MW:24693	RbromiL2-63
Org5_Gene426	accessory	1310	[RbromiL2-63]L2-63_00446_L2-63_00446_hypothetical_protein_459673:460728_forward_MW:39566	RbromiL2-63
Org5_Gene43	accessory	4239	[RbromiL2-63]L2-63_00046_L2-63_00046_hypothetical_protein_50510:50707_reverse_MW:7698	RbromiL2-63
Org5_Gene430	accessory	897	[RbromiL2-63]L2-63_00450_L2-63_00450_corrinoid_ABC_transporter_substrate-binding_protein,ABC-type_Fe3_-citrate_transport_system,_periplasmic_c	RbromiL2-63
Org5_Gene434	accessory	113	[RbromiL2-63]L2-63_00455_L2-63_00455_copper_exporting_ATPase,heavy_metal_translocating_P-type_ATPase_468949:471609_reverse_MW:98074	RbromiL2-63
Org5_Gene436	accessory	2765	[RbromiL2-63]L2-63_00457_L2-63_00457_hypothetical_protein_472834:473415_reverse_MW:21701	RbromiL2-63
Org5_Gene438	accessory	855	[RbromiL2-63]L2-63_00459_L2-63_00459_hypothetical_protein_474594:475892_reverse_MW:48330	RbromiL2-63
Org5_Gene44	accessory	1822	[RbromiL2-63]L2-63_00047_L2-63_00047_hypothetical_protein_50723:51586_reverse_MW:32673	RbromiL2-63
Org5_Gene441	accessory	1024	[RbromiL2-63]L2-63_00462_L2-63_00462_Predicted_integral_membrane_protein_478468:479670_forward_MW:46033	RbromiL2-63
Org5_Gene442	accessory	623	[RbromiL2-63]L2-63_00463_mepA_3_Multidrug_export_protein_mepA,multidrug_efflux_pump_VmrA,MATE_efflux_family_protein,MatE_479748:481208_	RbromiL2-63
Org5_Gene443	accessory	370	[RbromiL2-63]L2-63_00464_L2-63_00464_Bacterial_protein_of_unknown_function_(DUF885)_481244:483037_reverse_MW:67629	RbromiL2-63
Org5_Gene444	accessory	1870	[RbromiL2-63]L2-63_00465_L2-63_00465_Predicted_membrane_protein,Bax_inhibitor_1_like_483377:484225_forward_MW:30659	RbromiL2-63
Org5_Gene445	accessory	2552	[RbromiL2-63]L2-63_00466_L2-63_00466_Phosphorylated_carbohydrates_phosphatase_TM_1254,2-deoxyglucose-6-phosphatase,beta-phosphoglucomuta	RbromiL2-63
Org5_Gene446	accessory	740	[RbromiL2-63]L2-63_00467_mepA_4_Staphylococcal_virulence_regulator_protein_A,multidrug_efflux_pump_VmrA,MATE_efflux_family_protein,MatE_48	RbromiL2-63
Org5_Gene45	accessory	2000	[RbromiL2-63]L2-63_00048_L2-63_00048_Protein_of_unknown_function_(DUF3801)_51593:52405_reverse_MW:29755	RbromiL2-63
Org5_Gene453	accessory	285	[RbromiL2-63]L2-63_00474_L2-63_00474_hypothetical_protein_493706:494917_reverse_MW:45824	RbromiL2-63
Org5_Gene456	accessory	1882	[RbromiL2-63]L2-63_00477_L2-63_00477_Uncharacterized_metallophosphoesterase_Cj0846,phosphodiesterase_YaeI,Uncharacterized_protein_conserved	RbromiL2-63
Org5_Gene457	accessory	1931	[RbromiL2-63]L2-63_00478_ypdC_Uncharacterized_HTH-type_transcriptional_regulator_ypdC,transcriptional_activator_RhaS,DNA_gyrase_inhibitor,YSIRK-	RbromiL2-63
Org5_Gene459	accessory	2299	[RbromiL2-63]L2-63_00480_L2-63_00480_SynChlorMet_cassette_protein_ScmC_502239:502952_reverse_MW:26580	RbromiL2-63
Org5_Gene46	accessory	674	[RbromiL2-63]L2-63_00049_L2-63_00049_Relaxase/Mobilisation_nuclease_domain_52347:53759_reverse_MW:54711	RbromiL2-63
Org5_Gene460	accessory	950	[RbromiL2-63]L2-63_00481_L2-63_00481_Fucose_4-O-acetylase_and_related_acetyltransferases,Acyltransferase_family_503195:504409_reverse_MW:46	RbromiL2-63
Org5_Gene461	accessory	1372	[RbromiL2-63]L2-63_00483_L2-63_00483_hypothetical_protein_505363:506391_reverse_MW:39122	RbromiL2-63
Org5_Gene462	accessory	1060	[RbromiL2-63]L2-63_00484_alr_1_Alanine_racemase,alanine_racemase,Uncharacterized_protein_conserved_in_bacteria,alanine_racemase,Alanine_racem	RbromiL2-63
Org5_Gene463	accessory	557	[RbromiL2-63]L2-63_00485_L2-63_00485_Uncharacterized_protein_involved_in_cytokinesis,_contains_TGc_(transglutaminase/protease-like)_domain_50	RbromiL2-63
Org5_Gene466	accessory	3569	[RbromiL2-63]L2-63_00489_L2-63_00489_hypothetical_protein_512189:512557_forward_MW:13924	RbromiL2-63
Org5_Gene467	accessory	2025	[RbromiL2-63]L2-63_00490_L2-63_00490_hypothetical_protein_512999:513790_reverse_MW:28960	RbromiL2-63
Org5_Gene468	accessory	1840	[RbromiL2-63]L2-63_00491_tetD_2_hypothetical_protein,DNA-binding_transcriptional_regulator_SoxS,DNA_gyrase_inhibitor,YSIRK-targeted_surface_anti	RbromiL2-63
Org5_Gene469	accessory	1425	[RbromiL2-63]L2-63_00492_L2-63_00492_hypothetical_protein_515182:516183_reverse_MW:38321	RbromiL2-63
Org5_Gene47	accessory	3733	[RbromiL2-63]L2-63_00050_L2-63_00050_Ribbon-helix-helix_protein,_copG_family_53759:54088_reverse_MW:13123	RbromiL2-63
Org5_Gene470	accessory	1580	[RbromiL2-63]L2-63_00493_L2-63_00493_auxin_efflux_carrier,Membrane_transport_protein_516587:517525_reverse_MW:34095	RbromiL2-63
Org5_Gene471	accessory	3241	[RbromiL2-63]L2-63_00494_L2-63_00494_hypothetical_protein_517823:518278_forward_MW:16241	RbromiL2-63
Org5_Gene473	accessory	198	[RbromiL2-63]L2-63_00496_L2-63_00496_Beta/alpha-amylase_precursor,alpha-amylase,Maltooligosyl_trehalose_synthase,alpha,alpha-phosphotrehalase,	RbromiL2-63
Org5_Gene475	accessory	2216	[RbromiL2-63]L2-63_00498_sipW_1_Signal_peptidase_I_W,signal_peptidase_I_523942:524685_reverse_MW:27502	RbromiL2-63
Org5_Gene476	accessory	982	[RbromiL2-63]L2-63_00499_L2-63_00499_hypothetical_protein_525121:526344_reverse_MW:44111	RbromiL2-63
Org5_Gene477	accessory	20	[RbromiL2-63]L2-63_00500_L2-63_00500_repeat,Listeria-Bacteroides_repeat_domain_(List_Bact_rpt)_526376:530803_reverse_MW:160297	RbromiL2-63
Org5_Gene478	accessory	394	[RbromiL2-63]L2-63_00501_L2-63_00501_hypothetical_protein_530834:532588_reverse_MW:65378	RbromiL2-63
Org5_Gene479	accessory	1371	[RbromiL2-63]L2-63_00502_L2-63_00502_hypothetical_protein_532620:533648_reverse_MW:37548	RbromiL2-63
Org5_Gene48	accessory	4384	[RbromiL2-63]L2-63_00051_L2-63_00051_Protein_of_unknown_function_(DUF3789)_54048:54200_reverse_MW:5637	RbromiL2-63
Org5_Gene480	accessory	130	[RbromiL2-63]L2-63_00503_L2-63_00503_ATPase_involved_in_DNA_repair,chromosome_segregation_protein_SMC_533658:536204_reverse_MW:97664	RbromiL2-63
Org5_Gene481	accessory	552	[RbromiL2-63]L2-63_00504_L2-63_00504_hypothetical_protein_536298:537833_reverse_MW:58469	RbromiL2-63
Org5_Gene482	accessory	2212	[RbromiL2-63]L2-63_00505_sipW_2_Signal_peptidase_I_W,signal_peptidase_I,Peptidase_S24-like_538056:538799_reverse_MW:27356	RbromiL2-63
Org5_Gene483	accessory	2062	[RbromiL2-63]L2-63_00506_L2-63_00506_hypothetical_protein_538852:539643_reverse_MW:27915	RbromiL2-63
Org5_Gene484	accessory	2017	[RbromiL2-63]L2-63_00507_L2-63_00507_hypothetical_protein_539758:540564_reverse_MW:30405	RbromiL2-63
Org5_Gene485	accessory	1981	[RbromiL2-63]L2-63_00508_L2-63_00508_hypothetical_protein_540610:541428_reverse_MW:30215	RbromiL2-63
Org5_Gene487	accessory	2715	[RbromiL2-63]L2-63_00510_yvdD_LOG_family_protein_yvdD,conserved_hypothetical_protein,Possible_lysine_decarboxylase_541970:542563_reverse_MV	RbromiL2-63
Org5_Gene488	accessory	678	[RbromiL2-63]L2-63_00511_norM_Na(_)/drug_antiporter,multidrug_efflux_protein,MATE_efflux_family_protein,MatE_542812:544221_forward_MW:507	RbromiL2-63
Org5_Gene489	accessory	1990	[RbromiL2-63]L2-63_00512_L2-63_00512_hypothetical_protein_544267:545082_forward_MW:30984	RbromiL2-63
Org5_Gene49	accessory	3862	[RbromiL2-63]L2-63_00052_L2-63_00052_hypothetical_protein_54293:54586_reverse_MW:11023	RbromiL2-63
Org5_Gene490	accessory	8	[RbromiL2-63]L2-63_00513_L2-63_00513_hypothetical_protein_545613:552602_reverse_MW:257058	RbromiL2-63
Org5_Gene491	accessory	3125	[RbromiL2-63]L2-63_00514_L2-63_00514_hypothetical_protein_552605:553090_reverse_MW:18402	RbromiL2-63
Org5_Gene492	accessory	2956	[RbromiL2-63]L2-63_00515_L2-63_00515_hypothetical_protein_553101:553634_reverse_MW:19348	RbromiL2-63
Org5_Gene493	accessory	433	[RbromiL2-63]L2-63_00516_prkC_1_Serine/threonine-protein_kinase_PrkC,serine/threonine-protein_kinase,Predicted_ATPase,TOMM_system_kinase/cycl	RbromiL2-63
Org5_Gene494	accessory	2128	[RbromiL2-63]L2-63_00517_L2-63_00517_Putative_protein_phosphatase_2C-type,putative_protein_serine/threonine_phosphatase_555351:556124_rever	RbromiL2-63
Org5_Gene495	accessory	498	[RbromiL2-63]L2-63_00518_L2-63_00518_Uncharacterized_conserved_protein,_contains_FHA_domain,type_VI_secretion_system_FHA_domain_protein,F	RbromiL2-63
Org5_Gene496	accessory	843	[RbromiL2-63]L2-63_00519_L2-63_00519_hypothetical_protein_557813:559126_reverse_MW:49810	RbromiL2-63
Org5_Gene497	accessory	2470	[RbromiL2-63]L2-63_00520_L2-63_00520_Flp_pilus_assembly_protein,_protease_CpaA,Type_IV_leader_peptidase_family_559140:559811_reverse_MW:2	RbromiL2-63
Org5_Gene498	accessory	803	[RbromiL2-63]L2-63_00521_L2-63_00521_hypothetical_protein_559822:561150_reverse_MW:47964	RbromiL2-63
Org5_Gene499	accessory	2587	[RbromiL2-63]L2-63_00522_L2-63_00522_hypothetical_protein_561174:561809_reverse_MW:23127	RbromiL2-63
Org5_Gene5	accessory	3712	[RbromiL2-63]L2-63_00005_yqjI_Transcriptional_regulator_YqjI,lineage-specific_thermal_regulator_protein,transcriptional_regulator,_Acidobacterial,_Pad	RbromiL2-63
Org5_Gene50	accessory	1448	[RbromiL2-63]L2-63_00053_L2-63_00053_hypothetical_protein_54661:55650_reverse_MW:37485	RbromiL2-63

Org5_Gene500	accessory	39	[RbromiL2-63]L2-63_00523_L2-63_00523_hypothetical_protein_561825:565559_reverse_MW:138127	RbromiL2-63
Org5_Gene501	accessory	2487	[RbromiL2-63]L2-63_00524_L2-63_00524_Flp_pilus_assembly_protein_TadG,TadE-like_protein_565575:566240_reverse_MW:24651	RbromiL2-63
Org5_Gene502	accessory	4122	[RbromiL2-63]L2-63_00525_L2-63_00525_hypothetical_protein_566341:566562_reverse_MW:8020	RbromiL2-63
Org5_Gene503	accessory	1787	[RbromiL2-63]L2-63_00526_L2-63_00526_Flp_pilus_assembly_protein_TadB_566627:567502_reverse_MW:33118	RbromiL2-63
Org5_Gene504	accessory	1831	[RbromiL2-63]L2-63_00527_L2-63_00527_Flp_pilus_assembly_protein_TadB_567515:568378_reverse_MW:32303	RbromiL2-63
Org5_Gene505	accessory	955	[RbromiL2-63]L2-63_00528_ptlH_Pertussis_toxin_liberation_protein_H,conjugal_transfer_ATPase_TrbB,Flp_pilus_assembly_protein,_ATPase_CpaF,helicas	RbromiL2-63
Org5_Gene506	accessory	1296	[RbromiL2-63]L2-63_00529_L2-63_00529_Flp_pilus_assembly_protein,_ATPase_CpaE_569642:570706_reverse_MW:39199	RbromiL2-63
Org5_Gene507	accessory	2336	[RbromiL2-63]L2-63_00530_lytR_1_Sensory_transduction_protein_lytR,putative_two-component_response-regulatory_protein_YehT,Response_regulator_	RbromiL2-63
Org5_Gene508	accessory	821	[RbromiL2-63]L2-63_00531_L2-63_00531_hypothetical_protein_571731:573011_forward_MW:49404	RbromiL2-63
Org5_Gene509	accessory	4226	[RbromiL2-63]L2-63_00532_L2-63_00532_hypothetical_protein_573190:573390_reverse_MW:7928	RbromiL2-63
Org5_Gene51	accessory	1413	[RbromiL2-63]L2-63_00054_L2-63_00054_hypothetical_protein_55733:56740_reverse_MW:39096	RbromiL2-63
Org5_Gene510	accessory	4126	[RbromiL2-63]L2-63_00533_L2-63_00533_hypothetical_protein_573387:573608_reverse_MW:8038	RbromiL2-63
Org5_Gene511	accessory	1600	[RbromiL2-63]L2-63_00534_ribF_Riboflavin_biosynthesis_protein_ribF,bifunctional_riboflavin_kinase/FMN_adenylyltransferase,FAD_synthase,riboflavin_b	RbromiL2-63
Org5_Gene513	accessory	1729	[RbromiL2-63]L2-63_00536_L2-63_00536_Gamma-D-glutamyl-L-diamino_acid_endopeptidase_1,murein_peptide_amidase_A,Zinc_carboxypeptidase_5753	RbromiL2-63
Org5_Gene515	accessory	3242	[RbromiL2-63]L2-63_00538_L2-63_00538_C_GCAxxG_C_C_family_protein,Putative_redox-active_protein_(C_GCAxxG_C_C)_578793:579248_reverse_MW:	RbromiL2-63
Org5_Gene517	accessory	3405	[RbromiL2-63]L2-63_00540_L2-63_00540_Domain_of_unknown_function_(DUF3783)_581500:581889_forward_MW:14637	RbromiL2-63
Org5_Gene518	accessory	1928	[RbromiL2-63]L2-63_00541_ywpJ_Uncharacterized_phosphatase_YwpJ,sugar_phosphate_phosphatase,Predicted_hydrolase_(HAD_superfamily),Cof-like_h	RbromiL2-63
Org5_Gene52	accessory	1016	[RbromiL2-63]L2-63_00055_L2-63_00055_hypothetical_protein_56883:58088_reverse_MW:45468	RbromiL2-63
Org5_Gene521	accessory	3343	[RbromiL2-63]L2-63_00544_L2-63_00544_hypothetical_protein_584471:584899_forward_MW:16646	RbromiL2-63
Org5_Gene522	accessory	4179	[RbromiL2-63]L2-63_00545_L2-63_00545_hypothetical_protein_585130:585339_forward_MW:7814	RbromiL2-63
Org5_Gene523	accessory	3402	[RbromiL2-63]L2-63_00546_L2-63_00546_hypothetical_protein_585551:585967_reverse_MW:15876	RbromiL2-63
Org5_Gene524	accessory	3403	[RbromiL2-63]L2-63_00547_L2-63_00547_hypothetical_protein_586063:586476_reverse_MW:15421	RbromiL2-63
Org5_Gene525	accessory	1120	[RbromiL2-63]L2-63_00548_L2-63_00548_hypothetical_protein_587470:588627_forward_MW:41289	RbromiL2-63
Org5_Gene526	accessory	2852	[RbromiL2-63]L2-63_00549_L2-63_00549_hypothetical_protein_588723:589280_reverse_MW:20123	RbromiL2-63
Org5_Gene527	accessory	1143	[RbromiL2-63]L2-63_00550_L2-63_00550_Uncharacterized_protein_conserved_in_bacteria,G5_domain_589563:590711_reverse_MW:40585	RbromiL2-63
Org5_Gene528	accessory	1893	[RbromiL2-63]L2-63_00551_L2-63_00551_DNA-damage-inducible_protein_D_591115:591957_reverse_MW:32269	RbromiL2-63
Org5_Gene529	accessory	1999	[RbromiL2-63]L2-63_00552_araC_Arabinose_operon_regulatory_protein,DNA-binding_transcriptional_regulator_AraC,Adenosine_deaminase,YSIRK-target	RbromiL2-63
Org5_Gene53	accessory	3732	[RbromiL2-63]L2-63_00056_L2-63_00056_hypothetical_protein_58207:58536_reverse_MW:12801	RbromiL2-63
Org5_Gene530	accessory	1921	[RbromiL2-63]L2-63_00553_melR_Melibiose_operon_regulatory_protein,DNA-binding_transcriptional_regulator_MelR,DNA_gyrase_inhibitor,YSIRK-target	RbromiL2-63
Org5_Gene531	accessory	1873	[RbromiL2-63]L2-63_00554_aes_Acetyl_esterase,acetyl_esterase,Poly(3-hydroxybutyrate)_depolymerase,alpha/beta_hydrolase_fold_593967:594815_reve	RbromiL2-63
Org5_Gene532	accessory	1607	[RbromiL2-63]L2-63_00555_L2-63_00555_Bacterial_Ig-like_domain_(group_2)_594830:595759_reverse_MW:33466	RbromiL2-63
Org5_Gene533	accessory	2531	[RbromiL2-63]L2-63_00556_crp_cAMP_regulatory_protein,DNA-binding_transcriptional_dual_regulator_Crp,Predicted_signal-transduction_protein_contai	RbromiL2-63
Org5_Gene536	accessory	64	[RbromiL2-63]L2-63_00559_cbhB_Exoglucanase_B_precursor,Bacterial_Ig-like_domain_(group_2)_598694:601969_reverse_MW:120228	RbromiL2-63
Org5_Gene54	accessory	539	[RbromiL2-63]L2-63_00057_L2-63_00057_DNA_methylase_58614:60167_reverse_MW:59267	RbromiL2-63
Org5_Gene544	accessory	4118	[RbromiL2-63]L2-63_00567_L2-63_00567_Helix-turn-helix_domain_612377:612601_reverse_MW:8583	RbromiL2-63
Org5_Gene545	accessory	1524	[RbromiL2-63]L2-63_00568_L2-63_00568_hypothetical_protein_612724:613683_forward_MW:37428	RbromiL2-63
Org5_Gene547	accessory	2193	[RbromiL2-63]L2-63_00570_lytR_2_Sensory_transduction_protein_lytR,two-component_response_regulator,Response_regulator_of_the_LytR/AlgR_famil	RbromiL2-63
Org5_Gene548	accessory	548	[RbromiL2-63]L2-63_00571_L2-63_00571_hypothetical_protein_615643:617181_reverse_MW:54721	RbromiL2-63
Org5_Gene549	accessory	497	[RbromiL2-63]L2-63_00572_L2-63_00572_gliding-associated_putative_ABC_transporter_substrate-binding_component_GldG,ABC-type_uncharacterized_t	RbromiL2-63
Org5_Gene55	accessory	4272	[RbromiL2-63]L2-63_00058_L2-63_00058_hypothetical_protein_60241:60429_reverse_MW:7115	RbromiL2-63
Org5_Gene553	accessory	1656	[RbromiL2-63]L2-63_00576_L2-63_00576_hypothetical_protein_621232:622146_reverse_MW:35199	RbromiL2-63
Org5_Gene554	accessory	106	[RbromiL2-63]L2-63_00577_L2-63_00577_Predicted_membrane_protein,Bacterial_membrane_protein_YfhO_622492:625230_reverse_MW:102111	RbromiL2-63
Org5_Gene556	accessory	3213	[RbromiL2-63]L2-63_00579_L2-63_00579_GtrA-like_protein_626434:626892_forward_MW:17529	RbromiL2-63
Org5_Gene559	accessory	3407	[RbromiL2-63]L2-63_00582_L2-63_00582_hypothetical_protein_628638:628919_forward_MW:10396	RbromiL2-63
Org5_Gene56	accessory	917	[RbromiL2-63]L2-63_00059_L2-63_00059_Plasmid_recombination_enzyme_60548:61813_reverse_MW:49675	RbromiL2-63
Org5_Gene562	accessory	1987	[RbromiL2-63]L2-63_00588_L2-63_00588_hypothetical_protein_632424:633239_forward_MW:29843	RbromiL2-63
Org5_Gene563	accessory	3466	[RbromiL2-63]L2-63_00589_L2-63_00589_Protein_of_unknown_function_(DUF2752)_633355:633753_reverse_MW:14840	RbromiL2-63
Org5_Gene564	accessory	3623	[RbromiL2-63]L2-63_00590_L2-63_00590_hypothetical_protein_633757:634110_reverse_MW:13044	RbromiL2-63
Org5_Gene569	accessory	2320	[RbromiL2-63]L2-63_00595_rsmG_Ribosomal_RNA_small_subunit_methyltransferase_G,16S_rRNA_methyltransferase_GidB,16S_rRNA_(guanine(527)-N(7	RbromiL2-63
Org5_Gene57	accessory	1387	[RbromiL2-63]L2-63_00060_L2-63_00060_DNA_repair_protein_RadA,RecA-superfamily_ATPases_implicated_in_signal_transduction,KaiC_61994:63010_re	RbromiL2-63
Org5_Gene573	accessory	1410	[RbromiL2-63]L2-63_00599_yidC_Oxa1Ec,membrane_protein_insertase,membrane_protein_insertase,_YidC/Oxa1_family,60Kd_inner_membrane_protein_	RbromiL2-63
Org5_Gene58	accessory	1559	[RbromiL2-63]L2-63_00061_L2-63_00061_Adenine_deaminase,adenosine_deaminase,Adenosine_deaminase,adenosine_deaminase,Adenosine/AMP_dean	RbromiL2-63
Org5_Gene582	accessory	2258	[RbromiL2-63]L2-63_00608_L2-63_00608_cxxc_20_cxxc_protein_649976:650707_forward_MW:27593	RbromiL2-63
Org5_Gene584	accessory	3328	[RbromiL2-63]L2-63_00610_L2-63_00610_hypothetical_protein,acyl-CoA_thioesterase_YbgC,acyl-CoA_thioester_hydrolase,_YbgC/YbaW_family,Thioester	RbromiL2-63
Org5_Gene586	accessory	2176	[RbromiL2-63]L2-63_00612_L2-63_00612_hypothetical_protein_656534:657289_forward_MW:28108	RbromiL2-63
Org5_Gene587	accessory	327	[RbromiL2-63]L2-63_00613_apu_Alpha-amylase/pullulanase,putative_bifunctional_4-alpha-glucanotransferase/glycogen_debranching_enzyme,Maltooligo	RbromiL2-63
Org5_Gene588	accessory	2560	[RbromiL2-63]L2-63_00614_L2-63_00614_cell_division_protein_ZapA,Uncharacterized_protein_conserved_in_bacteria,Cell_division_protein_ZapA_65976	RbromiL2-63
Org5_Gene589	accessory	262	[RbromiL2-63]L2-63_00615_yhbU_1_Uncharacterized_protease_yhbU_precursor,putative_protease,Collagenase_and_related_proteases,Peptidase_family_	RbromiL2-63
Org5_Gene59	accessory	4112	[RbromiL2-63]L2-63_00062_L2-63_00062_hypothetical_protein_64577:64801_reverse_MW:8548	RbromiL2-63
Org5_Gene592	accessory	1973	[RbromiL2-63]L2-63_00618_L2-63_00618_rod_shape-determining_protein_MreC,rod_shape-determining_protein_MreC,rod_shape-determining_protein_	RbromiL2-63
Org5_Gene593	accessory	3046	[RbromiL2-63]L2-63_00619_L2-63_00619_rod_shape-determining_protein_MreD_665091:665594_forward_MW:18738	RbromiL2-63
Org5_Gene594	accessory	276	[RbromiL2-63]L2-63_00620_penA_Penicillin-binding_protein_2B,penicillin-binding_protein_2,Membrane_carboxypeptidase/penicillin-binding_protein,pen	RbromiL2-63
Org5_Gene6	accessory	652	[RbromiL2-63]L2-63_00006_L2-63_00006_hypothetical_protein_5587:7020_reverse_MW:54540	RbromiL2-63
Org5_Gene60	accessory	234	[RbromiL2-63]L2-63_00063_L2-63_00063_Recombinase_64873:66921_reverse_MW:79012	RbromiL2-63
Org5_Gene600	accessory	2096	[RbromiL2-63]L2-63_00626_L2-63_00626_hypothetical_protein_672406:673191_forward_MW:29576	RbromiL2-63
Org5_Gene602	accessory	1188	[RbromiL2-63]L2-63_00628_L2-63_00628_hypothetical_protein_674728:675852_forward_MW:42333	RbromiL2-63
Org5_Gene603	accessory	1977	[RbromiL2-63]L2-63_00629_L2-63_00629_Domain_of_unknown_function_(DUF477)_675849:676667_forward_MW:30200	RbromiL2-63
Org5_Gene604	accessory	1405	[RbromiL2-63]L2-63_00630_bioB_Biotin_synthase,biotin_synthase,[FeFe]_hydrogenase_H-cluster_radical_SAM_maturase_HydE,Radical_SAM_superfamily	RbromiL2-63
Org5_Gene606	accessory	1066	[RbromiL2-63]L2-63_00632_mnmE_2_tRNA_modification_GTPase_MnmE,tRNA_modification_GTPase_TrmE,Predicted_GTPase,[FeFe]_hydrogenase_H-clu	RbromiL2-63
Org5_Gene607	accessory	476	[RbromiL2-63]L2-63_00633_acs_Acetyl-coenzyme_A_synthetase,acetyl-CoA_synthetase,Uncharacterized_protein_conserved_in_bacteria,acetate--CoA_lig	RbromiL2-63
Org5_Gene61	accessory	4145	[RbromiL2-63]L2-63_00064_L2-63_00064_hypothetical_protein_66999:67217_forward_MW:8524	RbromiL2-63
Org5_Gene610	accessory	620	[RbromiL2-63]L2-63_00637_trpE_Anthranilate_synthase_component_1,anthranilate_synthase_component_I,Anthranilate/para-aminobenzoate_synthases	RbromiL2-63
Org5_Gene611	accessory	2800	[RbromiL2-63]L2-63_00638_trpG_Anthranilate_synthase_component_II,bifunctional_glutamine_amidotransferase/anthranilate_phosphoribosyltransferas	RbromiL2-63
Org5_Gene612	accessory	1401	[RbromiL2-63]L2-63_00639_trpD2_Anthranilate_phosphoribosyltransferase_2,anthranilate_phosphoribosyltransferase,Phosphoribosylanthranilate_isomer	RbromiL2-63
Org5_Gene613	accessory	2046	[RbromiL2-63]L2-63_00640_trpC_Indole-3-glycerol_phosphate_synthase,indole-3-glycerol-phosphate_synthase,Indole-3-glycerol_phosphate_synthase,Ind	RbromiL2-63
Org5_Gene619	accessory	3792	[RbromiL2-63]L2-63_00646_L2-63_00646_hypothetical_protein_693510:693821_forward_MW:11655	RbromiL2-63
Org5_Gene620	accessory	1348	[RbromiL2-63]L2-63_00647_fliU_Flagellar_biosynthetic_protein_fliU_693818:694855_forward_MW:40238	RbromiL2-63
Org5_Gene625	accessory	3909	[RbromiL2-63]L2-63_00652_L2-63_00652_hypothetical_protein_699021:699299_reverse_MW:10460	RbromiL2-63
Org5_Gene626	accessory	2706	[RbromiL2-63]L2-63_00653_L2-63_00653_KilA-N_domain_699302:699898_reverse_MW:23069	RbromiL2-63
Org5_Gene627	accessory	3998	[RbromiL2-63]L2-63_00654_L2-63_00654_hypothetical_protein_699933:700169_reverse_MW:9366	RbromiL2-63
Org5_Gene628	accessory	3442	[RbromiL2-63]L2-63_00655_ytkD_Putative_8-oxo-dGTP_diphosphatase_YtkD,nucleoside_triphosphatase_YtkD,NUDIX_domain_700222:700623_reverse_M	RbromiL2-63
Org5_Gene63	accessory	3171	[RbromiL2-63]L2-63_00066_L2-63_00066_Protein_of_unknown_function_(DUF3801)_68327:68800_forward_MW:17761	RbromiL2-63
Org5_Gene630	accessory	2668	[RbromiL2-63]L2-63_00657_fic_Probable_adenosine_monophosphate-protein_transferase_fic,cell_filamentation_protein_Fic,Protein_involved_in_cell_div	RbromiL2-63
Org5_Gene631	accessory	3920	[RbromiL2-63]L2-63_00658_L2-63_00658_hypothetical_protein_702924:703199_reverse_MW:10714	RbromiL2-63
Org5_Gene632	accessory	1368	[RbromiL2-63]L2-63_00659_hrcA_Heat-inducible_transcription_repressor_HrcA,heat-inducible_transcription_repressor,heat-inducible_transcription_repre	RbromiL2-63
Org5_Gene633	accessory	2855	[RbromiL2-63]L2-63_00660_grpE_HSP-70_cofactor,heat_shock_protein_GrpE,GrpE_704473:705030_forward_MW:20549	RbromiL2-63
Org5_Gene637	accessory	1225	[RbromiL2-63]L2-63_00664_L2-63_00664_hypothetical_protein_709493:709636_reverse_MW:5269	RbromiL2-63
Org5_Gene639	accessory	1937	[RbromiL2-63]L2-63_00666_L2-63_00666_hypothetical_protein_710542:711369_reverse_MW:31240	RbromiL2-63
Org5_Gene64	accessory	364	[RbromiL2-63]L2-63_00067_L2-63_00067_conjugal_transfer_coupling_protein_TraG,Type_IV_secretory_pathway,_VirD4_components,type_IV_conjugativ	RbromiL2-63
Org5_Gene640	accessory	2746	[RbromiL2-63]L2-63_00667_L2-63_00667_hypothetical_protein_711582:712169_reverse_MW:20942	RbromiL2-63
Org5_Gene641	accessory	3416	[RbromiL2-63]L2-63_00668_L2-63_00668_hypothetical_protein,transcriptional_regulator,_AbrB_family,Helix-turn-helix_712432:712842_forward_MW:154	RbromiL2-63
Org5_Gene642	accessory	3929	[RbromiL2-63]L2-63_00669_lptB_2_Lipopolysaccharide_export_system_ATP-binding_protein_LptB,nodulation_ABC_transporter_NodI,ABC-type_Na__tran	RbromiL2-63
Org5_Gene643	accessory	2146	[RbromiL2-63]L2-63_00670_L2-63_00670_Predicted_membrane_protein,Protein_of_unknown_function_(DUF1624)_713298:714056_forward_MW:28590	RbromiL2-63
Org5_Gene644	accessory	2358	[RbromiL2-63]L2-63_00671_L2-63_00671_Predicted_membrane_protein,Protein_of_unknown_function_(DUF1275)_714125:714829_reverse_MW:25739	RbromiL2-63
Org5_Gene65	accessory	4163	[RbromiL2-63]L2-63_00068_L2-63_00068_Maff2_family_70646:70804_forward_MW:5429	RbromiL2-63
Org5_Gene653	accessory	3576	[RbromiL2-63]L2-63_00680_L2-63_00680_Uncharacterized_protein_encoded_in_hypervariable_junctions_of_pilus_gene_clusters,Uncharacterised_protei	RbromiL2-63
Org5_Gene655	accessory	30	[RbromiL2-63]L2-63_00682_malS_Alpha-amylase_precursor,alpha-amylase,Maltooligosyl_trehalose_synthase,alpha,alpha-phosphotrehalase,Alpha_amylas	RbromiL2-63
Org5_Gene656	accessory	991	[RbromiL2-63]L2-63_00683_senX3_Signal-transduction_histidine_kinase_senX3,sensory_histidine_kinase_CreC,Predicted_periplasmic_ligand-binding_sens	RbromiL2-63
Org5_Gene657	accessory	2549	[RbromiL2-63]L2-63_00684_phoB_1_Phosphate_regulon_transcriptional_regulatory_protein_phoB,DNA-binding_response_regulator_CreB,Response_regu	RbromiL2-63
Org5_Gene658	accessory	116	[RbromiL2-63]L2-63_00685_L2-63_00685_acidobacterial_duplicated_orphan_permease,FtsX-like_permease_family_732028:734676_reverse_MW:97650	RbromiL2-63
Org5_Gene659	accessory	2465	[RbromiL2-63]L2-63_00686_macB_2_Macrolide_export_ATP-binding/permease_protein_MacB,macrolide_transporter_ATP-binding_/permease_protein,Pr	RbromiL2-63
Org5_Gene660	accessory	1648	[RbromiL2-63]L2-63_00687_truB_tRNA_pseudouridine_synthase_B,tRNA_pseudouridine_synthase_B,Pseudouridine_synthase,tRNA_pseudouridine(55)_s	RbromiL2-63
Org5_Gene661	accessory	1575	[RbromiL2-63]L2-63_00688_nrnA_Bifunctional_oligoribonuclease_and_PAP_phosphatase_nrnA,putative_bifunctional_signaling_protein/50S_ribosomal_p	RbromiL2-63
Org5_Gene664	accessory	3711	[RbromiL2-63]L2-63_00691_L2-63_00691_hypothetical_protein,Ribosomal_protein_L30E,Ribosomal_protein_L7Ae/L30e/S12e/Gadd45_family_740526:740	RbromiL2-63
Org5_Gene665	accessory	3805	[RbromiL2-63]L2-63_00692_L2-63_00692_hypothetical_protein,Protein_of_unknown_function_(DUF448)_740874:741182_reverse_MW:11566	RbromiL2-63
Org5_Gene668	accessory	4240	[RbromiL2-63]L2-63_00696_L2-63_00696_hypothetical_protein_744390:744587_forward_MW:7327	RbromiL2-63
Org5_Gene669	accessory	2984	[RbromiL2-63]L2-63_00697_L2-63_00697_hypothetical_protein_744883:745404_forward_MW:19165	RbromiL2-63
Org5_Gene67	accessory	2975	[RbromiL2-63]L2-63_00070_L2-63_00070_hypothetical_protein_71372:71899_forward_MW:18586	RbromiL2-63
Org5_Gene674	accessory	2636	[RbromiL2-63]L2-63_00702_L2-63_00702_hypothetical_protein_748591:749196_forward_MW:22336	RbromiL2-63
Org5_Gene677	accessory	2043	[RbromiL2-63]L2-63_00705_L2-63_00705_hypothetical_protein_753872:754669_forward_MW:29708	RbromiL2-63
Org5_Gene678	accessory	406	[RbromiL2-63]L2-63_00706_htrA_1_Serine_protease_Do-like_HtrA,serine_endoprotease,Predicted_secreted_protein_containing_a_PDZ_domain,peptidas	RbromiL2-63
Org5_Gene68	accessory	3861	[RbromiL2-63]L2-63_00071_L2-63_00071_hypothetical_protein_72075:72368_forward_MW:10800	RbromiL2-63
Org5_Gene680	accessory	2881	[RbromiL2-63]L2-63_00708_bioY_Biotin_ECF_transporter_S_component_BioY,BioY_family_758285:758833_forward_MW:19285	RbromiL2-63
Org5_Gene681	accessory	3590	[RbromiL2-63]L2-63_00709_L2-63_00709_Protein_of_unknown_function_(DUF1284)_758859:759221_forward_MW:13303	RbromiL2-63
Org5_Gene685	accessory	19	[RbromiL2-63]L2-63_00713_L2-63_00713_fibro-slime_domain_766617:771104_forward_MW:165429	RbromiL2-63
Org5_Gene686	accessory	662	[RbromiL2-63]L2-63_00714_L2-63_00714_Predicted_outer_membrane_protein,fimbrial_isopeptide_formation_D2_domain,Cna_protein_B-type_domain_	RbromiL2-63
Org5_Gene687	accessory	1569	[RbromiL2-63]L2-63_00715_L2-63_00715_Sortase_(surface_protein_transpeptidase),sortase,Sortase_family_772913:773857_forward_MW:35052	RbromiL2-63
Org5_Gene688	accessory	2076	[RbromiL2-63]L2-63_00716_L2-63_00716_Sortase_(surface_protein_transpeptidase),sortase,Sortase_family_773850:774638_forward_MW:28969	RbromiL2-63
Org5_Gene689	accessory	1665	[RbromiL2-63]L2-63_00717_L2-63_00717_hypothetical_protein_774733:775641_forward_MW:33550	RbromiL2-63
Org5_Gene69	accessory	1972	[RbromiL2-63]L2-63_00072_immR_2_HTH-type_transcriptional_regulator_immR,Helix-turn-helix_domain_72413:73234_reverse_MW:31763	RbromiL2-63
Org5_Gene690	accessory	2556	[RbromiL2-63]L2-63_00718_L2-63_00718_hypothetical_protein_776147:776788_reverse_MW:24224	RbromiL2-63
Org5_Gene693	accessory	2561	[RbromiL2-63]L2-63_00721_L2-63_00721_Vitamin_B12_dependent_methionine_synthase,_activation_domain_779793:780437_forward_MW:24068	RbromiL2-63

Org5_Gene695	accessory	3393	[RbromiL2-63]L2-63_00723_L2-63_00723_hypothetical_protein_782939:783355_forward_MW:16516	RbromiL2-63
Org5_Gene697	accessory	1054	[RbromiL2-63]L2-63_00725_L2-63_00725_L,D-transpeptidase_catalytic_domain_788177:789364_reverse_MW:42855	RbromiL2-63
Org5_Gene699	accessory	3085	[RbromiL2-63]L2-63_00727_L2-63_00727_Predicted_HD_superfamily_hydrolase,HD_domain_790270:790761_forward_MW:18648	RbromiL2-63
Org5_Gene7	accessory	680	[RbromiL2-63]L2-63_00007_L2-63_00007_hypothetical_protein_7041:8450_reverse_MW:53892	RbromiL2-63
Org5_Gene70	accessory	3703	[RbromiL2-63]L2-63_00073_L2-63_00073_hypothetical_protein_73956:74288_forward_MW:12070	RbromiL2-63
Org5_Gene701	accessory	2180	[RbromiL2-63]L2-63_00729_pflA_Pyruvate_formate-lyase-activating_enzyme,pyruvate_formate_lyase-activating_enzyme_1,Predicted_Fe-S_oxidoreductas	RbromiL2-63
Org5_Gene702	accessory	4146	[RbromiL2-63]L2-63_00730_L2-63_00730_hypothetical_protein_794565:794783_forward_MW:7946	RbromiL2-63
Org5_Gene703	accessory	342	[RbromiL2-63]L2-63_00731_L2-63_00731_Predicted_integral_membrane_protein_linked_to_a_cation_pump,FixH_795358:797220_forward_MW:66774	RbromiL2-63
Org5_Gene704	accessory	3663	[RbromiL2-63]L2-63_00732_L2-63_00732_hypothetical_protein_797213:797554_reverse_MW:13373	RbromiL2-63
Org5_Gene705	accessory	2764	[RbromiL2-63]L2-63_00733_cysE_1_Serine_acetyltransferase,serine_acetyltransferase,Acetyltransferase_(isoleucine_patch_superfamily),serine_O-acetyltr	RbromiL2-63
Org5_Gene706	accessory	1127	[RbromiL2-63]L2-63_00734_L2-63_00734_NADH_dehydrogenase_subunit_I,Uncharacterized_Fe-S_center_protein,NADH-quinone_oxidoreductase,_chain_	RbromiL2-63
Org5_Gene707	accessory	1254	[RbromiL2-63]L2-63_00735_L2-63_00735_Polysaccharide_pyruvyl_transferase_799568:800653_reverse_MW:41248	RbromiL2-63
Org5_Gene708	accessory	1689	[RbromiL2-63]L2-63_00736_kfoC_1_Chondroitin_polymerase,N-glycosyltransferase,Predicted_glycosyl_hydrolase,poly-beta-1,6_N-acetyl-D-glucosamine_s	RbromiL2-63
Org5_Gene709	accessory	547	[RbromiL2-63]L2-63_00737_L2-63_00737_Polysaccharide_biosynthesis_protein_801585:803114_reverse_MW:55519	RbromiL2-63
Org5_Gene71	accessory	3830	[RbromiL2-63]L2-63_00074_L2-63_00074_Protein_of_unknown_function_(DUF3847)_74285:74587_forward_MW:11529	RbromiL2-63
Org5_Gene710	accessory	1203	[RbromiL2-63]L2-63_00738_L2-63_00738_Polysaccharide_pyruvyl_transferase_803114:804232_reverse_MW:42871	RbromiL2-63
Org5_Gene711	accessory	910	[RbromiL2-63]L2-63_00739_L2-63_00739_Lipid_A_core_-_O-antigen_ligase_and_related_enzymes,probable_O-glycosylation_ligase,_exosortase_A-associa	RbromiL2-63
Org5_Gene712	accessory	1491	[RbromiL2-63]L2-63_00740_kfoC_2_Chondroitin_polymerase,putative_glycosyl_transferase,Predicted_glycosyl_hydrolase,poly-beta-1,6_N-acetyl-D-glucos	RbromiL2-63
Org5_Gene714	accessory	1049	[RbromiL2-63]L2-63_00742_L2-63_00742_NADH_dehydrogenase_subunit_I,PUA_domain_(predicted_RNA-binding_domain),NADH-quinone_oxidoreductas	RbromiL2-63
Org5_Gene715	accessory	1097	[RbromiL2-63]L2-63_00743_L2-63_00743_Polysaccharide_pyruvyl_transferase_807737:808906_reverse_MW:45155	RbromiL2-63
Org5_Gene716	accessory	942	[RbromiL2-63]L2-63_00744_L2-63_00744_Glycogen_synthase,colanic_acid_biosynthesis_glycosyltransferase_WcaL,Glycogen_synthase,N-acetyl-alpha-D-gl	RbromiL2-63
Org5_Gene717	accessory	1411	[RbromiL2-63]L2-63_00745_gspA_General_stress_protein_A,lipopolysaccharide_1,3-galactosyltransferase,Glycosyl_transferase_family_8_810161:811168_	RbromiL2-63
Org5_Gene72	accessory	3599	[RbromiL2-63]L2-63_00075_L2-63_00075_hypothetical_protein_74875:75234_forward_MW:13170	RbromiL2-63
Org5_Gene722	accessory	2462	[RbromiL2-63]L2-63_00750_wcaJ_1_Putative_colanic_biosynthesis_UDP-glucose_lipid_carrier_transferase,undecaprenyl-phosphate_galactose_phosphotra	RbromiL2-63
Org5_Gene723	accessory	2326	[RbromiL2-63]L2-63_00751_ywqD_1_Tyrosine-protein_kinase_YwqD,tyrosine_kinase,Capsular_polysaccharide_biosynthesis_protein,capsular_exopolysacch	RbromiL2-63
Org5_Gene724	accessory	2288	[RbromiL2-63]L2-63_00752_cap8A_Capsular_polysaccharide_type_8_biosynthesis_protein_cap8A,Capsular_polysaccharide_biosynthesis_protein,polysacch	RbromiL2-63
Org5_Gene725	accessory	2727	[RbromiL2-63]L2-63_00753_L2-63_00753_hypothetical_protein_817596:818186_forward_MW:20686	RbromiL2-63
Org5_Gene726	accessory	2281	[RbromiL2-63]L2-63_00754_L2-63_00754_Sortase_(surface_protein_transpeptidase),sortase,_marine_proteobacterial_type,Sortase_family_818192:81891	RbromiL2-63
Org5_Gene727	accessory	1941	[RbromiL2-63]L2-63_00755_L2-63_00755_hypothetical_protein_818917:819747_forward_MW:31046	RbromiL2-63
Org5_Gene73	accessory	519	[RbromiL2-63]L2-63_00076_mobA_1_DNA_strand_transferase,conjugal_transfer_relaxase_TraA,Ti-type_conjugative_transfer_relaxase_TraA,MobA/MobL_	RbromiL2-63
Org5_Gene730	accessory	751	[RbromiL2-63]L2-63_00758_L2-63_00758_Na_-dependent_transporters_of_the_SNF_family,Sodium:neurotransmitter_symporter_family_823181:824536_	RbromiL2-63
Org5_Gene731	accessory	218	[RbromiL2-63]L2-63_00759_ppk_Polyphosphate_kinase,polyphosphate_kinase,polyphosphate_kinase_1,Polyphosphate_kinase_824862:827042_forward_N	RbromiL2-63
Org5_Gene732	accessory	1269	[RbromiL2-63]L2-63_00760_pfp_Pyrophosphate--fructose_6-phosphate_1-phosphotransferase,6-phosphofructokinase,P_pilus_assembly/Cpx_signaling_pa	RbromiL2-63
Org5_Gene733	accessory	4322	[RbromiL2-63]L2-63_00761_L2-63_00761_hypothetical_protein_828195:828371_forward_MW:6544	RbromiL2-63
Org5_Gene734	accessory	3449	[RbromiL2-63]L2-63_00762_L2-63_00762_hypothetical_protein,Uncharacterized_protein_conserved_in_bacteria,Protein_of_unknown_function_(DUF419)	RbromiL2-63
Org5_Gene735	accessory	2930	[RbromiL2-63]L2-63_00763_L2-63_00763_hypothetical_protein_828930:829466_forward_MW:19796	RbromiL2-63
Org5_Gene738	accessory	4279	[RbromiL2-63]L2-63_00766_L2-63_00766_hypothetical_protein_832153:832341_reverse_MW:7394	RbromiL2-63
Org5_Gene739	accessory	3666	[RbromiL2-63]L2-63_00767_L2-63_00767_hypothetical_protein_832451:832792_forward_MW:12874	RbromiL2-63
Org5_Gene74	accessory	2577	[RbromiL2-63]L2-63_00077_dnaG_1_DNA_primase,DNA_primase,DNA_primase_(bacterial_type),DNA_primase,CHC2_zinc_finger_77041:77682_forward_N	RbromiL2-63
Org5_Gene742	accessory	422	[RbromiL2-63]L2-63_00770_L2-63_00770_Uncharacterized_vancomycin_resistance_protein,VanW_like_protein_834693:836396_reverse_MW:61415	RbromiL2-63
Org5_Gene743	accessory	1154	[RbromiL2-63]L2-63_00771_ugtP_Processive_diacylglycerol_glucosyltransferase,diacylglycerol_glucosyltransferase,Spore_coat_polysaccharide_biosynthesi	RbromiL2-63
Org5_Gene744	accessory	762	[RbromiL2-63]L2-63_00772_L2-63_00772_Serine_protease_inhibitor,Serpin_(serine_protease_inhibitor)_838000:839349_forward_MW:49540	RbromiL2-63
Org5_Gene749	accessory	786	[RbromiL2-63]L2-63_00777_cycB_Cyclodextrin-binding_protein_precursor,maltose_ABC_transporter_periplasmic_protein,Maltose-binding_periplasmic_pr	RbromiL2-63
Org5_Gene760	accessory	295	[RbromiL2-63]L2-63_00788_L2-63_00788_sortase,_SrtB_family,Sortase_family_860204:862153_forward_MW:73732	RbromiL2-63
Org5_Gene761	accessory	3382	[RbromiL2-63]L2-63_00789_L2-63_00789_hypothetical_protein_862165:862578_forward_MW:15299	RbromiL2-63
Org5_Gene762	accessory	1041	[RbromiL2-63]L2-63_00790_metB_Cystathionine_gamma-synthase,cystathionine_gamma-synthase,Cystathionine_beta-lyase_family_protein_involved_in_	RbromiL2-63
Org5_Gene763	accessory	4128	[RbromiL2-63]L2-63_00791_L2-63_00791_hypothetical_protein_864149:864370_forward_MW:8490	RbromiL2-63
Org5_Gene764	accessory	1447	[RbromiL2-63]L2-63_00792_L2-63_00792_Glycyl-glycine_endopeptidase_ALE-1_precursor,putative_peptidase,Peptidase_family_M23_864992:865960_for	RbromiL2-63
Org5_Gene765	accessory	3376	[RbromiL2-63]L2-63_00793_L2-63_00793_hypothetical_protein_866081:866500_forward_MW:15053	RbromiL2-63
Org5_Gene767	accessory	1121	[RbromiL2-63]L2-63_00795_L2-63_00795_hypothetical_protein_868193:869350_forward_MW:43507	RbromiL2-63
Org5_Gene768	accessory	3012	[RbromiL2-63]L2-63_00796_btuR_Cob(I)yrinic_acid_a,c-diamide_adenosyltransferase,cob(I)alamin_adenolsyltransferase/cobinamide_ATP-dependent_ade	RbromiL2-63
Org5_Gene78	accessory	1819	[RbromiL2-63]L2-63_00081_L2-63_00081_hypothetical_protein_81346:82209_forward_MW:31421	RbromiL2-63
Org5_Gene795	accessory	4431	[RbromiL2-63]L2-63_00823_rpmJ_Ribosomal_protein_II,50S_ribosomal_protein_L36,ribosomal_protein_L36,Ribosomal_protein_L36_882981:883094_forw	RbromiL2-63
Org5_Gene8	accessory	660	[RbromiL2-63]L2-63_00008_L2-63_00008_hypothetical_protein_8467:9891_reverse_MW:54412	RbromiL2-63
Org5_Gene80	accessory	3485	[RbromiL2-63]L2-63_00083_L2-63_00083_PrgI_family_protein_82770:83162_forward_MW:14715	RbromiL2-63
Org5_Gene801	accessory	1513	[RbromiL2-63]L2-63_00829_L2-63_00829_hypothetical_protein_886358:887320_reverse_MW:34098	RbromiL2-63
Org5_Gene802	accessory	281	[RbromiL2-63]L2-63_00830_L2-63_00830_CotH_protein_887568:889556_forward_MW:73507	RbromiL2-63
Org5_Gene804	accessory	3031	[RbromiL2-63]L2-63_00832_aroL_Shikimate_kinase_2,shikimate_kinase,Dephospho-CoA_kinase,Shikimate_kinase_891208:891717_forward_MW:18688	RbromiL2-63
Org5_Gene808	accessory	845	[RbromiL2-63]L2-63_00836_fgs_Folylpolyglutamate_synthase,bifunctional_folylpolyglutamate_synthase/_dihydrofolate_synthase,Folylpolyglutamate_synt	RbromiL2-63
Org5_Gene810	accessory	2222	[RbromiL2-63]L2-63_00838_yfiC_tRNA1(Val)_(adenine(37)-N6)-methyltransferase,N5-glutamine_S-adenosyl-L-methionine-dependent_methyltransferase,f	RbromiL2-63
Org5_Gene813	accessory	1827	[RbromiL2-63]L2-63_00841_dnaX_1_DNA_polymerase_III_subunit_tau,DNA_polymerase_III_subunit_delta',DNA_polymerase_III,_delta'_subunit_897438:8	RbromiL2-63
Org5_Gene814	accessory	3178	[RbromiL2-63]L2-63_00842_yfcE_Phosphodiesterase_yfcE,phosphodiesterase,phosphodiesterase,_MJ0936_family,Calcineurin-like_phosphoesterase_supe	RbromiL2-63
Org5_Gene815	accessory	4070	[RbromiL2-63]L2-63_00843_L2-63_00843_hypothetical_protein_898891:899127_forward_MW:8509	RbromiL2-63
Org5_Gene816	accessory	3622	[RbromiL2-63]L2-63_00844_L2-63_00844_hypothetical_protein_899129:899482_forward_MW:13127	RbromiL2-63
Org5_Gene817	accessory	3891	[RbromiL2-63]L2-63_00845_L2-63_00845_hypothetical_protein_899497:899781_forward_MW:11027	RbromiL2-63
Org5_Gene82	accessory	1536	[RbromiL2-63]L2-63_00085_dpnA_Modification_methylase_DpnIIB,putative_methyltransferase,Adenine_specific_DNA_methylase_Mod,DNA_methylase_8	RbromiL2-63
Org5_Gene824	accessory	2932	[RbromiL2-63]L2-63_00852_aroK_Shikimate_kinase,shikimate_kinase,Shikimate_kinase_906110:906646_forward_MW:19661	RbromiL2-63
Org5_Gene83	accessory	289	[RbromiL2-63]L2-63_00086_cwlS_D-gamma-glutamyl-meso-diaminopimelic_acid_endopeptidase_CwlS_precursor,invasion_associated_secreted_endopept	RbromiL2-63
Org5_Gene831	accessory	3612	[RbromiL2-63]L2-63_00859_immR_7_HTH-type_transcriptional_regulator_immR,hypothetical_protein,Predicted_transcriptional_regulator,Helix-turn-helix	RbromiL2-63
Org5_Gene832	accessory	629	[RbromiL2-63]L2-63_00860_L2-63_00860_hypothetical_protein_913944:915401_forward_MW:54138	RbromiL2-63
Org5_Gene833	accessory	3079	[RbromiL2-63]L2-63_00861_L2-63_00861_Double_zinc_ribbon_915412:915906_forward_MW:17935	RbromiL2-63
Org5_Gene835	accessory	2863	[RbromiL2-63]L2-63_00863_L2-63_00863_Negative_regulator_of_genetic_competence,_sporulation_and_motility_917748:918302_forward_MW:20856	RbromiL2-63
Org5_Gene836	accessory	4325	[RbromiL2-63]L2-63_00864_L2-63_00864_hypothetical_protein_918392:918568_reverse_MW:7156	RbromiL2-63
Org5_Gene837	accessory	3672	[RbromiL2-63]L2-63_00865_L2-63_00865_hypothetical_protein_918683:919021_forward_MW:12644	RbromiL2-63
Org5_Gene838	accessory	3532	[RbromiL2-63]L2-63_00866_L2-63_00866_hypothetical_protein_919145:919522_reverse_MW:13638	RbromiL2-63
Org5_Gene84	accessory	2290	[RbromiL2-63]L2-63_00087_L2-63_00087_hypothetical_protein,PUA_domain_(predicted_RNA-binding_domain)_88452:89174_forward_MW:27966	RbromiL2-63
Org5_Gene840	accessory	1232	[RbromiL2-63]L2-63_00868_tdh_L-threonine_3-dehydrogenase,L-threonine_3-dehydrogenase,L-threonine_3-dehydrogenase,Zinc-binding_dehydrogenase_	RbromiL2-63
Org5_Gene841	accessory	2373	[RbromiL2-63]L2-63_00869_alsE_D-allulose-6-phosphate_3-epimerase,allulose-6-phosphate_3-epimerase,Pentose-5-phosphate-3-epimerase,ribulose-phos	RbromiL2-63
Org5_Gene842	accessory	704	[RbromiL2-63]L2-63_00870_ydjE_Inner_membrane_metabolite_transport_protein_ydjE,putative_3-hydroxyphenylpropionic_transporter_MhpT,Arabinose	RbromiL2-63
Org5_Gene843	accessory	1308	[RbromiL2-63]L2-63_00871_gutB_Sorbitol_dehydrogenase,L-threonine_3-dehydrogenase,L-threonine_3-dehydrogenase,Alcohol_dehydrogenase_GroES-lik	RbromiL2-63
Org5_Gene844	accessory	1913	[RbromiL2-63]L2-63_00872_gatY_D-tagatose-1,6-bisphosphate_aldolase_subunit_GatY,tagatose-bisphosphate_aldolase,Fructose/tagatose_bisphosphate_	RbromiL2-63
Org5_Gene845	accessory	1487	[RbromiL2-63]L2-63_00873_ydjH_Uncharacterized_sugar_kinase_ydjH,ribokinase,Uncharacterized_protein_conserved_in_bacteria,ribokinase,pfkB_family_	RbromiL2-63
Org5_Gene846	accessory	1438	[RbromiL2-63]L2-63_00874_yhdN_General_stress_protein_69,putative_oxidoreductase,Predicted_oxidoreductase,voltage-dependent_potassium_channel	RbromiL2-63
Org5_Gene847	accessory	2185	[RbromiL2-63]L2-63_00875_glcR_2_HTH-type_transcriptional_repressor_glcR,DNA-binding_transcriptional_regulator_AgaR,Phosphotransferase_system,_r	RbromiL2-63
Org5_Gene848	accessory	728	[RbromiL2-63]L2-63_00876_gltP_Glutamate-aspartate_carrier_protein,C4-dicarboxylate_transporter_DctA,Sodium:dicarboxylate_symporter_family_93189	RbromiL2-63
Org5_Gene849	accessory	1189	[RbromiL2-63]L2-63_00877_gldA_Glycerol_dehydrogenase,glycerol_dehydrogenase,Glycerol_dehydrogenase_and_related_enzymes,Iron-containing_alcoh	RbromiL2-63
Org5_Gene85	accessory	3959	[RbromiL2-63]L2-63_00088_L2-63_00088_hypothetical_protein_89181:89447_forward_MW:9970	RbromiL2-63
Org5_Gene850	accessory	373	[RbromiL2-63]L2-63_00878_adeC_Adenine_deaminase,cryptic_adenine_deaminase,N-acyl-D-aspartate/D-glutamate_deacylase,adenine_deaminase,Amido	RbromiL2-63
Org5_Gene851	accessory	3269	[RbromiL2-63]L2-63_00879_L2-63_00879_Protein_of_unknown_function_(DUF2712)_937158:937604_forward_MW:16764	RbromiL2-63
Org5_Gene852	accessory	2165	[RbromiL2-63]L2-63_00880_L2-63_00880_hypothetical_protein_937717:938478_forward_MW:28515	RbromiL2-63
Org5_Gene853	accessory	2172	[RbromiL2-63]L2-63_00881_L2-63_00881_hypothetical_protein_938465:939223_forward_MW:29120	RbromiL2-63
Org5_Gene854	accessory	2402	[RbromiL2-63]L2-63_00882_L2-63_00882_Protein_of_unknown_function_(DUF2705)_939210:939902_forward_MW:26169	RbromiL2-63
Org5_Gene855	accessory	2621	[RbromiL2-63]L2-63_00883_metN_Methionine_import_ATP-binding_protein_MetN,cytochrome_c_biogenesis_protein_CcmA,ABC-type_uncharacterized_t	RbromiL2-63
Org5_Gene856	accessory	1432	[RbromiL2-63]L2-63_00884_L2-63_00884_hypothetical_protein_940525:941520_forward_MW:37193	RbromiL2-63
Org5_Gene857	accessory	1052	[RbromiL2-63]L2-63_00885_L2-63_00885_HAD_phosphoserine_phosphatase-like_hydrolase,_family_IB,haloacid_dehalogenase-like_hydrolase_942102:94	RbromiL2-63
Org5_Gene858	accessory	616	[RbromiL2-63]L2-63_00886_dgt_Deoxyguanosinetriphosphate_triphosphohydrolase,deoxyguanosinetriphosphate_triphosphohydrolase-like_protein,putati	RbromiL2-63
Org5_Gene859	accessory	2464	[RbromiL2-63]L2-63_00887_L2-63_00887_hypothetical_protein_944849:945520_forward_MW:26096	RbromiL2-63
Org5_Gene86	accessory	2199	[RbromiL2-63]L2-63_00089_L2-63_00089_hypothetical_protein_89437:90186_forward_MW:26600	RbromiL2-63
Org5_Gene860	accessory	2456	[RbromiL2-63]L2-63_00888_L2-63_00888_hypothetical_protein_945974:946648_reverse_MW:26474	RbromiL2-63
Org5_Gene861	accessory	854	[RbromiL2-63]L2-63_00889_L2-63_00889_Predicted_ATPase_946761:947948_reverse_MW:44996	RbromiL2-63
Org5_Gene862	accessory	235	[RbromiL2-63]L2-63_00890_L2-63_00890_hypothetical_protein_948173:949306_reverse_MW:41195	RbromiL2-63
Org5_Gene863	accessory	2170	[RbromiL2-63]L2-63_00891_L2-63_00891_hypothetical_protein_951624:952382_forward_MW:29965	RbromiL2-63
Org5_Gene864	accessory	2809	[RbromiL2-63]L2-63_00892_L2-63_00892_hypothetical_protein_952626:953195_forward_MW:21771	RbromiL2-63
Org5_Gene865	accessory	68	[RbromiL2-63]L2-63_00893_hsdR_1_Type-1_restriction_enzyme_R_protein,type_I_restriction_enzyme_EcoKI_subunit_R,type_I_site-specific_deoxyribonu	RbromiL2-63
Org5_Gene866	accessory	895	[RbromiL2-63]L2-63_00894_L2-63_00894_EcoKI_restriction-modification_system_protein_HsdS,Type_I_restriction_modification_DNA_specificity_domain_	RbromiL2-63
Org5_Gene867	accessory	515	[RbromiL2-63]L2-63_00895_L2-63_00895_Probable_type_I_restriction_enzyme_BthVORF4518P_M_protein,N5-glutamine_S-adenosyl-L-methionine-depen	RbromiL2-63
Org5_Gene868	accessory	1596	[RbromiL2-63]L2-63_00896_Int-Tn_2_Integrase,site-specific_tyrosine_recombinase_XerC,Site-specific_recombinase_XerD,tyrosine_recombinase_XerD,Pha	RbromiL2-63
Org5_Gene869	accessory	2663	[RbromiL2-63]L2-63_00897_L2-63_00897_Type_I_restriction_modification_DNA_specificity_domain_960286:960894_reverse_MW:22898	RbromiL2-63
Org5_Gene87	accessory	255	[RbromiL2-63]L2-63_00090_topB_1_DNA_topoisomerase_3,DNA_topoisomerase_III,Topoisomerase_IA,DNA_topoisomerase_III,DNA_topoisomerase_9018	RbromiL2-63
Org5_Gene870	accessory	3032	[RbromiL2-63]L2-63_00898_L2-63_00898_EcoKI_restriction-modification_system_protein_HsdS,Type_I_restriction_modification_DNA_specificity_domain_	RbromiL2-63
Org5_Gene871	accessory	2641	[RbromiL2-63]L2-63_00899_L2-63_00899_hypothetical_protein_962891:963505_forward_MW:23675	RbromiL2-63
Org5_Gene872	accessory	3731	[RbromiL2-63]L2-63_00900_L2-63_00900_hypothetical_protein_963630:963959_forward_MW:12120	RbromiL2-63
Org5_Gene873	accessory	1488	[RbromiL2-63]L2-63_00901_L2-63_00901_Putative_viral_replication_protein_964075:965046_forward_MW:37654	RbromiL2-63
Org5_Gene874	accessory	851	[RbromiL2-63]L2-63_00902_ftsK_DNA_translocase_FtsK,DNA_translocase_FtsK,DNA_segregation_ATPase_FtsK/SpoIIIE_and_related_proteins,type_VII_sec	RbromiL2-63
Org5_Gene875	accessory	4172	[RbromiL2-63]L2-63_00903_L2-63_00903_DNA_binding_domain,_excisionase_family,Helix-turn-helix_domain_966362:966574_forward_MW:8083	RbromiL2-63
Org5_Gene876	accessory	1081	[RbromiL2-63]L2-63_00904_Int-Tn_3_Integrase,site-specific_tyrosine_recombinase_XerC,Site-specific_recombinase_XerD,tyrosine_recombinase_XerC,Pha	RbromiL2-63
Org5_Gene877	accessory	4198	[RbromiL2-63]L2-63_00905_immR_8_HTH-type_transcriptional_regulator_immR,hypothetical_protein,Helix-turn-helix_968327:968533_reverse_MW:7822	RbromiL2-63
Org5_Gene878	accessory	2492	[RbromiL2-63]L2-63_00906_L2-63_00906_hypothetical_protein_968644:969309_forward_MW:23646	RbromiL2-63
Org5_Gene879	accessory	863	[RbromiL2-63]L2-63_00907_L2-63_00907_KWG_Leptospira_969331:970626_forward_MW:47586	RbromiL2-63
Org5_Gene88	accessory	58	[RbromiL2-63]L2-63_00091_L2-63_00091_Antirestriction_protein_(ArdA)_92749:96096_forward_MW:126817	RbromiL2-63
Org5_Gene880	accessory	2178	[RbromiL2-63]L2-63_00908_L2-63_00908_hypothetical_protein_970663:971418_forward_MW:27560	RbromiL2-63
Org5_Gene881	accessory	4355	[RbromiL2-63]L2-63_00909_L2-63_00909_hypothetical_protein_971519:971686_reverse_MW:6396	RbromiL2-63

Org5_Gene883	accessory	3750	[RbromiL2-63]L2-63_00911_immR_9_HTH-type_transcriptional_regulator_immR,hypothetical_protein,conserved_hypothetical_protein,Helix-turn-helix_97	RbromiL2-63
Org5_Gene884	accessory	254	[RbromiL2-63]L2-63_00912_cgkA_Kappa-carrageenase_precursor,bacterial_surface_protein_26-residue_repeat,Bacterial_Ig-like_domain_(group_2)_9742	RbromiL2-63
Org5_Gene886	accessory	2455	[RbromiL2-63]L2-63_00914_tmk_Thymidylate_kinase,thymidylate_kinase,thymidylate_kinase,Thymidylate_kinase_977312:977986_forward_MW:25408	RbromiL2-63
Org5_Gene887	accessory	1737	[RbromiL2-63]L2-63_00915_L2-63_00915_Uncharacterized_conserved_protein,Uncharacterized_conserved_protein_(DUF2156)_977973:978863_forward_	RbromiL2-63
Org5_Gene888	accessory	2072	[RbromiL2-63]L2-63_00916_L2-63_00916_Predicted_acetyltransferase_involved_in_intracellular_survival_and_related_acetyltransferases,Acetyltransferas	RbromiL2-63
Org5_Gene89	accessory	4183	[RbromiL2-63]L2-63_00092_L2-63_00092_hypothetical_protein_96101:96310_forward_MW:7966	RbromiL2-63
Org5_Gene890	accessory	2684	[RbromiL2-63]L2-63_00918_yqgN_hypothetical_protein,5-formyltetrahydrofolate_cyclo-ligase_family_protein,5-formyltetrahydrofolate_cyclo-ligase,5-forr	RbromiL2-63
Org5_Gene891	accessory	749	[RbromiL2-63]L2-63_00919_yceG_hypothetical_protein,putative_aminodeoxychorismate_lyase,conserved_hypothetical_protein,_YceG_family,YceG-like_f	RbromiL2-63
Org5_Gene894	accessory	1159	[RbromiL2-63]L2-63_00923_L2-63_00923_hypothetical_protein_986772:987854_forward_MW:37295	RbromiL2-63
Org5_Gene895	accessory	1932	[RbromiL2-63]L2-63_00925_nfo_Probable_endonuclease_4,endonuclease_IV,Endonuclease_IV,apurinic_endonuclease_(APN1),Xylose_isomerase-like_TIM	RbromiL2-63
Org5_Gene896	accessory	3720	[RbromiL2-63]L2-63_00926_trxA_2_Thioredoxin,thioredoxin,Thiol:disulfide_interchange_protein,thioredoxin,Thioredoxin_989236:989565_forward_MW:1	RbromiL2-63
Org5_Gene897	accessory	1726	[RbromiL2-63]L2-63_00927_trxB_1_Thioredoxin_reductase,alkyl_hydroperoxide_reductase_subunit_F,Alkyl_hydroperoxide_reductase,_large_subunit,thio	RbromiL2-63
Org5_Gene898	accessory	1625	[RbromiL2-63]L2-63_00928_eamA_Probable_amino-acid_metabolite_efflux_pump,putative_DMT_superfamily_transporter_inner_membrane_protein,carb	RbromiL2-63
Org5_Gene9	accessory	284	[RbromiL2-63]L2-63_00009_fbp_Fructose-1,6-bisphosphatase_class_3,Firmicute_fructose-1,6-bisphosphatase_10428:12410_reverse_MW:75100	RbromiL2-63
Org5_Gene90	accessory	792	[RbromiL2-63]L2-63_00093_L2-63_00093_Relaxase/Mobilisation_nuclease_domain_96512:97846_reverse_MW:50915	RbromiL2-63
Org5_Gene900	accessory	653	[RbromiL2-63]L2-63_00930_L2-63_00930_Na_/alanine_symporter,amino_acid_carrier_protein,Sodium:alanine_symporter_family_992171:993598_forwarc	RbromiL2-63
Org5_Gene901	accessory	2709	[RbromiL2-63]L2-63_00931_L2-63_00931_hypothetical_protein_993655:994251_forward_MW:23073	RbromiL2-63
Org5_Gene904	accessory	1710	[RbromiL2-63]L2-63_00934_L2-63_00934_Uncharacterized_BCR,_YitT_family_COG1284_998604:999500_forward_MW:32506	RbromiL2-63
Org5_Gene905	accessory	1072	[RbromiL2-63]L2-63_00935_fieF_Ferrous-iron_efflux_pump_FieF,ferrous_iron_efflux_protein_F,Predicted_Co/Zn/Cd_cation_transporters,cation_diffusion_	RbromiL2-63
Org5_Gene91	accessory	3735	[RbromiL2-63]L2-63_00094_L2-63_00094_Bacterial_mobilisation_protein_(MobC)_97807:98136_reverse_MW:13080	RbromiL2-63
Org5_Gene912	accessory	4300	[RbromiL2-63]L2-63_00942_L2-63_00942_hypothetical_protein_1009060:1009242_forward_MW:6547	RbromiL2-63
Org5_Gene913	accessory	3516	[RbromiL2-63]L2-63_00943_L2-63_00943_hypothetical_protein_1009295:1009678_reverse_MW:14336	RbromiL2-63
Org5_Gene914	accessory	3762	[RbromiL2-63]L2-63_00944_L2-63_00944_hypothetical_protein_1009693:1010013_reverse_MW:11389	RbromiL2-63
Org5_Gene915	accessory	3577	[RbromiL2-63]L2-63_00945_L2-63_00945_hypothetical_protein_1009994:1010359_reverse_MW:13473	RbromiL2-63
Org5_Gene916	accessory	4291	[RbromiL2-63]L2-63_00946_L2-63_00946_hypothetical_protein_1010514:1010699_forward_MW:6829	RbromiL2-63
Org5_Gene917	accessory	526	[RbromiL2-63]L2-63_00947_nlpD_Murein_hydrolase_activator_NlpD_precursor,lipoprotein_NlpD,Membrane-bound_metallopeptidase,Peptidase_family_	RbromiL2-63
Org5_Gene919	accessory	1670	[RbromiL2-63]L2-63_00949_lyc_Autolytic_lysozyme,Lyzozyme_M1_(1,4-beta-N-acetylmuramidase),Glycosyl_hydrolases_family_25_1012942:1013850_reve	RbromiL2-63
Org5_Gene92	accessory	3615	[RbromiL2-63]L2-63_00095_L2-63_00095_anaerobic_benzoate_catabolism_transcriptional_regulator,Predicted_transcriptional_regulator,Helix-turn-helix_	RbromiL2-63
Org5_Gene920	accessory	3701	[RbromiL2-63]L2-63_00950_L2-63_00950_hypothetical_protein_1014103:1014435_forward_MW:12773	RbromiL2-63
Org5_Gene921	accessory	588	[RbromiL2-63]L2-63_00951_L2-63_00951_hypothetical_protein,Fibronectin_type_III_domain_1014541:1016019_forward_MW:54587	RbromiL2-63
Org5_Gene922	accessory	2135	[RbromiL2-63]L2-63_00952_L2-63_00952_hypothetical_protein_1016281:1017051_forward_MW:28158	RbromiL2-63
Org5_Gene923	accessory	3751	[RbromiL2-63]L2-63_00953_L2-63_00953_transcriptional_repressor_DicA,transcriptional_regulator,_y4mF_family,Helix-turn-helix_1017311:1017637_rever	RbromiL2-63
Org5_Gene924	accessory	2812	[RbromiL2-63]L2-63_00954_L2-63_00954_hypothetical_protein_1017805:1018374_forward_MW:21692	RbromiL2-63
Org5_Gene932	accessory	1895	[RbromiL2-63]L2-63_00962_prmC_Release_factor_glutamine_methyltransferase,N5-glutamine_S-adenosyl-L-methionine-dependent_methyltransferase,M	RbromiL2-63
Org5_Gene936	accessory	785	[RbromiL2-63]L2-63_00966_L2-63_00966_hypothetical_protein_1029518:1030855_reverse_MW:49191	RbromiL2-63
Org5_Gene938	accessory	2171	[RbromiL2-63]L2-63_00968_L2-63_00968_hypothetical_protein,metal-binding_protein,Uncharacterized_protein_conserved_in_bacteria,dinuclear_metal_	RbromiL2-63
Org5_Gene939	accessory	2486	[RbromiL2-63]L2-63_00969_trmK_tRNA_(adenine(22)-N(1))-methyltransferase,Family_of_unknown_function_(DUF633)_1032430:1033095_reverse_MW:2	RbromiL2-63
Org5_Gene94	accessory	3309	[RbromiL2-63]L2-63_00097_L2-63_00097_RNA_polymerase_sigma_factor,RNA_polymerase_sigma_factor,_sigma-70_family,Sigma-70,_region_4_100752:1	RbromiL2-63
Org5_Gene941	accessory	556	[RbromiL2-63]L2-63_00971_comM_Competence_protein_ComM,putative_ATP-dependent_protease,Mg-chelatase_subunit_ChlI,Mg_chelatase-like_protei	RbromiL2-63
Org5_Gene942	accessory	2728	[RbromiL2-63]L2-63_00972_sigH_Stage_0_sporulation_protein_H,RNA_polymerase_factor_sigma-70,RNA_polymerase_sigma-H_factor,Sigma-70,_region_	RbromiL2-63
Org5_Gene945	accessory	1187	[RbromiL2-63]L2-63_00975_L2-63_00975_Uncharacterized_conserved_protein,Protein_of_unknown_function_DUF262_1037917:1039041_forward_MW:4	RbromiL2-63
Org5_Gene946	accessory	3142	[RbromiL2-63]L2-63_00976_L2-63_00976_hypothetical_protein_1039199:1039678_forward_MW:17602	RbromiL2-63
Org5_Gene947	accessory	3124	[RbromiL2-63]L2-63_00977_L2-63_00977_hypothetical_protein_1039739:1040224_reverse_MW:18239	RbromiL2-63
Org5_Gene948	accessory	3198	[RbromiL2-63]L2-63_00978_L2-63_00978_hypothetical_protein_1040243:1040707_reverse_MW:16856	RbromiL2-63
Org5_Gene95	accessory	4045	[RbromiL2-63]L2-63_00098_L2-63_00098_Helix-turn-helix_domain_101173:101415_forward_MW:9010	RbromiL2-63
Org5_Gene950	accessory	3141	[RbromiL2-63]L2-63_00980_L2-63_00980_hypothetical_protein_1041222:1041701_reverse_MW:18327	RbromiL2-63
Org5_Gene951	accessory	2938	[RbromiL2-63]L2-63_00981_L2-63_00981_hypothetical_protein_1041727:1042263_reverse_MW:20289	RbromiL2-63
Org5_Gene952	accessory	3185	[RbromiL2-63]L2-63_00982_L2-63_00982_hypothetical_protein_1042442:1042912_forward_MW:18071	RbromiL2-63
Org5_Gene957	accessory	3082	[RbromiL2-63]L2-63_00987_L2-63_00987_hypothetical_protein_1047736:1048230_forward_MW:18556	RbromiL2-63
Org5_Gene96	accessory	4334	[RbromiL2-63]L2-63_00099_L2-63_00099_hypothetical_protein_101874:102047_forward_MW:6422	RbromiL2-63
Org5_Gene964	accessory	3080	[RbromiL2-63]L2-63_00994_dgkA_Undecaprenol_kinase,Prokaryotic_diacylglycerol_kinase_1052871:1053365_forward_MW:18337	RbromiL2-63
Org5_Gene974	accessory	3116	[RbromiL2-63]L2-63_01004_coaD_Phosphopantetheine_adenylyltransferase,phosphopantetheine_adenylyltransferase,Predicted_ATPase/kinase_involved_	RbromiL2-63
Org5_Gene976	accessory	4436	[RbromiL2-63]L2-63_01006_L2-63_01006_hypothetical_protein_1063257:1063346_forward_MW:3131	RbromiL2-63
Org5_Gene977	accessory	3379	[RbromiL2-63]L2-63_01007_mraZ_hypothetical_protein,cell_division_protein_MraZ,protein_MraZ,MraZ_protein_1063669:1064088_forward_MW:15525	RbromiL2-63
Org5_Gene979	accessory	2882	[RbromiL2-63]L2-63_01009_L2-63_01009_hypothetical_protein_1065296:1065844_forward_MW:20458	RbromiL2-63
Org5_Gene985	accessory	631	[RbromiL2-63]L2-63_01015_L2-63_01015_cell_division_protein_FtsQ,POTRA_domain,_FtsQ-type_1073192:1074604_forward_MW:52602	RbromiL2-63
Org5_Gene987	accessory	1530	[RbromiL2-63]L2-63_01017_L2-63_01017_ABC-type_taurine_transport_system,_periplasmic_component,TRAP_transporter_solute_receptor,_TAXI_family,	RbromiL2-63
Org5_Gene988	accessory	1055	[RbromiL2-63]L2-63_01018_gltS_Glutamate_permease,sodium/glutamate_symporter,Sodium/glutamate_symporter_1077012:1078199_forward_MW:421	RbromiL2-63
Org5_Gene989	accessory	4268	[RbromiL2-63]L2-63_01019_L2-63_01019_hypothetical_protein_1078217:1078405_forward_MW:7209	RbromiL2-63
Org5_Gene99	accessory	11	[RbromiL2-63]L2-63_00102_L2-63_00102_DNA_methylase,SNF2_family_N-terminal_domain_104030:110206_reverse_MW:234094	RbromiL2-63
Org5_Gene990	accessory	1482	[RbromiL2-63]L2-63_01020_cbh_Choloylglycine_hydrolase,Penicillin_V_acylase_and_related_amidases,Linear_amide_C-N_hydrolases,_choloylglycine_hyd	RbromiL2-63
Org5_Gene991	accessory	994	[RbromiL2-63]L2-63_01021_clcA_H(_)/Cl(-)_exchange_transporter_ClcA,putative_voltage-gated_ClC-type_chloride_channel_ClcB,Voltage_gated_chloride_	RbromiL2-63
Org5_Gene992	accessory	1843	[RbromiL2-63]L2-63_01022_L2-63_01022_hypothetical_protein_1081002:1081853_reverse_MW:32007	RbromiL2-63
Org5_Gene993	accessory	3461	[RbromiL2-63]L2-63_01023_L2-63_01023_hypothetical_protein_1082000:1082398_forward_MW:14430	RbromiL2-63
Org5_Gene994	accessory	4285	[RbromiL2-63]L2-63_01024_L2-63_01024_hypothetical_protein_1082502:1082684_reverse_MW:7043	RbromiL2-63
Org5_Gene995	accessory	4288	[RbromiL2-63]L2-63_01025_L2-63_01025_hypothetical_protein_1082769:1082954_reverse_MW:7023	RbromiL2-63
Org5_Gene998	accessory	935	[RbromiL2-63]L2-63_01028_L2-63_01028_hypothetical_protein_1085687:1086940_forward_MW:48551	RbromiL2-63
Org5_Gene999	accessory	1862	[RbromiL2-63]L2-63_01029_L2-63_01029_error-prone_DNA_polymerase,Histidinol_phosphatase_and_related_hydrolases_of_the_PHP_family,DNA_polym	RbromiL2-63
Org1_Gene10	core	1902	2595397931_IE32DRAFT_00012_nicotinate-nucleotide_pyrophosphorylase_[carboxylating]_(EC_2.4.2.19)_[Ruminococcus_bromii_YE282]	Ruminococcus_bromii_YE282
Org1_Gene1000	core	137	2595398955_IE32DRAFT_01039_copper-(or_silver)-translocating_P-type_ATPase_[Ruminococcus_bromii_YE282]	Ruminococcus_bromii_YE282
Org1_Gene1015	core	118	2595398971_IE32DRAFT_01055_alanyl-tRNA_synthetase_(EC_6.1.1.7)_[Ruminococcus_bromii_YE282]	Ruminococcus_bromii_YE282
Org1_Gene1016	core	1316	2595398972_IE32DRAFT_01058_phenylalanyl-tRNA_synthetase,_alpha_subunit_(EC_6.1.1.20)_[Ruminococcus_bromii_YE282]	Ruminococcus_bromii_YE282
Org1_Gene1017	core	169	2595398973_IE32DRAFT_01059_phenylalanyl-tRNA_synthetase_beta_subunit_(EC_6.1.1.20)_[Ruminococcus_bromii_YE282]	Ruminococcus_bromii_YE282
Org1_Gene1018	core	4032	2595398974_IE32DRAFT_01060_Uncharacterized_protein_conserved_in_bacteria_[Ruminococcus_bromii_YE282]	Ruminococcus_bromii_YE282
Org1_Gene1019	core	3817	2595398975_IE32DRAFT_01061_hypothetical_protein_[Ruminococcus_bromii_YE282]	Ruminococcus_bromii_YE282
Org1_Gene1023	core	675	2595398979_IE32DRAFT_01065_Aspartyl_aminopeptidase_[Ruminococcus_bromii_YE282]	Ruminococcus_bromii_YE282
Org1_Gene1026	core	1337	2595398982_IE32DRAFT_01068_tRNA_threonylcarbamoyl_adenosine_modification_protein,_Sua5/YciO/YrdC/YwlC_family_[Ruminococcus_bromii_YE282]	Ruminococcus_bromii_YE282
Org1_Gene1027	core	1276	2595398983_IE32DRAFT_01069_peptide_chain_release_factor_1_[Ruminococcus_bromii_YE282]	Ruminococcus_bromii_YE282
Org1_Gene1028	core	4312	2595398984_IE32DRAFT_01070_Uncharacterized_protein_conserved_in_bacteria_[Ruminococcus_bromii_YE282]	Ruminococcus_bromii_YE282
Org1_Gene1029	core	1050	2595398985_IE32DRAFT_01071_LL-diaminopimelate_aminotransferase_apoenzyme_(EC_2.6.1.83)_[Ruminococcus_bromii_YE282]	Ruminococcus_bromii_YE282
Org1_Gene1030	core	492	2595398986_IE32DRAFT_01072_CTP_synthase_(EC_6.3.4.2)_[Ruminococcus_bromii_YE282]	Ruminococcus_bromii_YE282
Org1_Gene1031	core	31	2595398987_IE32DRAFT_01073_diaminopimelate_epimerase_[Ruminococcus_bromii_YE282]	Ruminococcus_bromii_YE282
Org1_Gene1032	core	1263	2595398988_IE32DRAFT_01074_carbamoyl-phosphate_synthase,_small_subunit_[Ruminococcus_bromii_YE282]	Ruminococcus_bromii_YE282
Org1_Gene1033	core	2810	2595398989_IE32DRAFT_01075_response_regulator_receiver_and_ANTAR_domain_protein_[Ruminococcus_bromii_YE282]	Ruminococcus_bromii_YE282
Org1_Gene1034	core	432	2595398990_IE32DRAFT_01076_ammonium_transporter_[Ruminococcus_bromii_YE282]	Ruminococcus_bromii_YE282
Org1_Gene1035	core	246	2595398991_IE32DRAFT_01077_Uncharacterized_protein_related_to_glutamine_synthetase_[Ruminococcus_bromii_YE282]	Ruminococcus_bromii_YE282
Org1_Gene1036	core	18	2595398992_IE32DRAFT_01078_Glutamate_synthase_domain_2_[Ruminococcus_bromii_YE282]	Ruminococcus_bromii_YE282
Org1_Gene1037	core	593	2595398993_IE32DRAFT_01079_glutamate_synthases,_NADH/NADPH,_small_subunit_[Ruminococcus_bromii_YE282]	Ruminococcus_bromii_YE282
Org1_Gene1038	core	594	2595398994_IE32DRAFT_01080_23S_rRNA_(uracil-5-)-methyltransferase_RumA_[Ruminococcus_bromii_YE282]	Ruminococcus_bromii_YE282
Org1_Gene1049	core	1288	2595399005_IE32DRAFT_01091_Aldose_1-epimerase_[Ruminococcus_bromii_YE282]	Ruminococcus_bromii_YE282
Org1_Gene1052	core	572	2595399008_IE32DRAFT_01094_Galactose-1-phosphate_uridyl_transferase,_N-terminal_domain_[Ruminococcus_bromii_YE282]	Ruminococcus_bromii_YE282
Org1_Gene1053	core	1440	2595399009_IE32DRAFT_01095_UDP-glucose-4-epimerase_GalE_[Ruminococcus_bromii_YE282]	Ruminococcus_bromii_YE282
Org1_Gene1056	core	1658	2595399012_IE32DRAFT_01098_dihydroorotate_dehydrogenase_(subfamily_1)_family_protein_[Ruminococcus_bromii_YE282]	Ruminococcus_bromii_YE282
Org1_Gene1057	core	2164	2595399013_IE32DRAFT_01099_2-polyprenylphenol_hydroxylase_and_related_flavodoxin_oxidoreductases_[Ruminococcus_bromii_YE282]	Ruminococcus_bromii_YE282
Org1_Gene1066	core	4093	2595399022_IE32DRAFT_01109_Uncharacterized_conserved_protein_[Ruminococcus_bromii_YE282]	Ruminococcus_bromii_YE282
Org1_Gene1072	core	449	2595399028_IE32DRAFT_01115_hydroxylamine_reductase_[Ruminococcus_bromii_YE282]	Ruminococcus_bromii_YE282
Org1_Gene1089	core	1015	2595399046_IE32DRAFT_01133_aspartate_kinase_(EC_2.7.2.4)_[Ruminococcus_bromii_YE282]	Ruminococcus_bromii_YE282
Org1_Gene1090	core	957	2595399047_IE32DRAFT_01134_homoserine_dehydrogenase_(EC_1.1.1.3)_[Ruminococcus_bromii_YE282]	Ruminococcus_bromii_YE282
Org1_Gene1093	core	1551	2595399050_IE32DRAFT_01137_putative_enoyl-[acyl-carrier-protein]_reductase_II_[Ruminococcus_bromii_YE282]	Ruminococcus_bromii_YE282
Org1_Gene1094	core	1688	2595399051_IE32DRAFT_01138_(acyl-carrier-protein)_S-malonyltransferase_[Ruminococcus_bromii_YE282]	Ruminococcus_bromii_YE282
Org1_Gene1095	core	2214	2595399052_IE32DRAFT_01139_3-oxoacyl-[acyl-carrier-protein]_reductase_(EC_1.1.1.100)_[Ruminococcus_bromii_YE282]	Ruminococcus_bromii_YE282
Org1_Gene1096	core	970	2595399053_IE32DRAFT_01140_beta-ketoacyl-acyl-carrier-protein_synthase_II_[Ruminococcus_bromii_YE282]	Ruminococcus_bromii_YE282
Org1_Gene1098	core	3377	2595399055_IE32DRAFT_01142_3-hydroxymyristoyl/3-hydroxydecanoyl-(acyl_carrier_protein)_dehydratases_[Ruminococcus_bromii_YE282]	Ruminococcus_bromii_YE282
Org1_Gene1099	core	748	2595399056_IE32DRAFT_01143_acetyl-CoA_carboxylase,_biotin_carboxylase_subunit_[Ruminococcus_bromii_YE282]	Ruminococcus_bromii_YE282
Org1_Gene11	core	1775	2595397932_IE32DRAFT_00013_UTP--glucose-1-phosphate_uridylyltransferase_[Ruminococcus_bromii_YE282]	Ruminococcus_bromii_YE282
Org1_Gene1100	core	1826	2595399057_IE32DRAFT_01144_acetyl-CoA_carboxylase,_carboxyl_transferase,_beta_subunit_[Ruminococcus_bromii_YE282]	Ruminococcus_bromii_YE282
Org1_Gene1101	core	2088	2595399058_IE32DRAFT_01145_acetyl-CoA_carboxylase,_carboxyl_transferase,_alpha_subunit_[Ruminococcus_bromii_YE282]	Ruminococcus_bromii_YE282
Org1_Gene1185	core	4248	2595399142_IE32DRAFT_01229_hybrid_cluster_protein-associated_redox_disulfide_domain_[Ruminococcus_bromii_YE282]	Ruminococcus_bromii_YE282
Org1_Gene1188	core	2484	2595399145_IE32DRAFT_01232_tRNA_(guanine-N(7)-)-methyltransferase_[Ruminococcus_bromii_YE282]	Ruminococcus_bromii_YE282
Org1_Gene1190	core	2345	2595399147_IE32DRAFT_01234_Response_regulators_consisting_of_a_CheY-like_receiver_domain_and_a_winged-helix_DNA-binding_domain_[Ruminoc	Ruminococcus_bromii_YE282
Org1_Gene1191	core	605	2595399148_IE32DRAFT_01235_Signal_transduction_histidine_kinase_[Ruminococcus_bromii_YE282]	Ruminococcus_bromii_YE282
Org1_Gene1192	core	1443	2595399149_IE32DRAFT_01236_Predicted_metal-dependent_enzyme_[Ruminococcus_bromii_YE282]	Ruminococcus_bromii_YE282
Org1_Gene1194	core	1195	2595399151_IE32DRAFT_01238_protein_RecA_[Ruminococcus_bromii_YE282]	Ruminococcus_bromii_YE282
Org1_Gene1195	core	2648	2595399152_IE32DRAFT_01239_Uncharacterized_protein_conserved_in_bacteria_[Ruminococcus_bromii_YE282]	Ruminococcus_bromii_YE282
Org1_Gene1196	core	778	2595399153_IE32DRAFT_01240_ribosomal_protein_S12_methylthiotransferase_RimO_[Ruminococcus_bromii_YE282]	Ruminococcus_bromii_YE282
Org1_Gene1197	core	2725	2595399154_IE32DRAFT_01241_CDP-diacylglycerol--glycerol-3-phosphate_3-phosphatidyltransferase_[Ruminococcus_bromii_YE282]	Ruminococcus_bromii_YE282
Org1_Gene1198	core	2491	2595399155_IE32DRAFT_01242_serine_O-acetyltransferase_[Ruminococcus_bromii_YE282]	Ruminococcus_bromii_YE282
Org1_Gene1199	core	685	2595399156_IE32DRAFT_01243_cysteinyl-tRNA_synthetase_(EC_6.1.1.16)_[Ruminococcus_bromii_YE282]	Ruminococcus_bromii_YE282
Org1_Gene1200	core	2056	2595399157_IE32DRAFT_01244_Predicted_xylanase/chitin_deacetylase_[Ruminococcus_bromii_YE282]	Ruminococcus_bromii_YE282
Org1_Gene1202	core	415	2595399159_IE32DRAFT_01246_hypothetical_protein_[Ruminococcus_bromii_YE282]	Ruminococcus_bromii_YE282
Org1_Gene1203	core	460	2595399160_IE32DRAFT_01247_Glycosidases_[Ruminococcus_bromii_YE282]	Ruminococcus_bromii_YE282
Org1_Gene1204	core	1824	2595399161_IE32DRAFT_01248_ABC-type_maltose_transport_systems,_permease_component_[Ruminococcus_bromii_YE282]	Ruminococcus_bromii_YE282

Org1_Gene1205	core	581	2595399162_IE32DRAFT_01249_ABC-type_sugar_transport_systems,_permease_components_[Ruminococcus_bromii_YE282]	Ruminococcus_bromii_YE282
Org1_Gene1207	core	607	2595399164_IE32DRAFT_01251_UDP-N-acetylmuramoylalanyl-D-glutamate--2,6-diaminopimelate_ligase_(EC_6.3.2.13)_[Ruminococcus_bromii_YE282]	Ruminococcus_bromii_YE282
Org1_Gene1208	core	641	2595399165_IE32DRAFT_01252_putative_nicotinate_phosphoribosyltransferase_[Ruminococcus_bromii_YE282]	Ruminococcus_bromii_YE282
Org1_Gene1215	core	2067	2595399172_IE32DRAFT_01259_hypothetical_protein_[Ruminococcus_bromii_YE282]	Ruminococcus_bromii_YE282
Org1_Gene1216	core	1978	2595399173_IE32DRAFT_01260_glutamate_racemase_(EC_5.1.1.3)_[Ruminococcus_bromii_YE282]	Ruminococcus_bromii_YE282
Org1_Gene1217	core	3199	2595399174_IE32DRAFT_01261_Uncharacterized_protein_conserved_in_bacteria_[Ruminococcus_bromii_YE282]	Ruminococcus_bromii_YE282
Org1_Gene1218	core	408	2595399175_IE32DRAFT_01262_arginyl-tRNA_synthetase_(EC_6.1.1.19)_[Ruminococcus_bromii_YE282]	Ruminococcus_bromii_YE282
Org1_Gene1220	core	2912	2595399177_IE32DRAFT_01264_Rubrerythrin_[Ruminococcus_bromii_YE282]	Ruminococcus_bromii_YE282
Org1_Gene1221	core	2897	2595399178_IE32DRAFT_01265_N-acetylmuramoyl-L-alanine_amidase_[Ruminococcus_bromii_YE282]	Ruminococcus_bromii_YE282
Org1_Gene1223	core	2500	2595399180_IE32DRAFT_01267_Uncharacterised_ACR,_YkgG_family_COG1556_[Ruminococcus_bromii_YE282]	Ruminococcus_bromii_YE282
Org1_Gene1224	core	818	2595399181_IE32DRAFT_01268_tRNA_and_rRNA_cytosine-C5-methylases_[Ruminococcus_bromii_YE282]	Ruminococcus_bromii_YE282
Org1_Gene1225	core	1855	2595399182_IE32DRAFT_01269_ribosomal_RNA_small_subunit_methyltransferase_A_[Ruminococcus_bromii_YE282]	Ruminococcus_bromii_YE282
Org1_Gene1227	core	124	2595399184_IE32DRAFT_01271_ATPase,_P-type_(transporting),_HAD_superfamily,_subfamily_IC_[Ruminococcus_bromii_YE282]	Ruminococcus_bromii_YE282
Org1_Gene1228	core	2927	2595399185_IE32DRAFT_01272_Inorganic_pyrophosphatase_[Ruminococcus_bromii_YE282]	Ruminococcus_bromii_YE282
Org1_Gene1230	core	1923	2595399187_IE32DRAFT_01274_YfiH_family_protein_[Ruminococcus_bromii_YE282]	Ruminococcus_bromii_YE282
Org1_Gene1236	core	446	2595399193_IE32DRAFT_01281_2-isopropylmalate_synthase,_yeast_type_[Ruminococcus_bromii_YE282]	Ruminococcus_bromii_YE282
Org1_Gene1239	core	1660	2595399196_IE32DRAFT_01284_ABC-type_multidrug_transport_system,_ATPase_component_[Ruminococcus_bromii_YE282]	Ruminococcus_bromii_YE282
Org1_Gene1242	core	3631	2595399199_IE32DRAFT_01287_Uncharacterized_protein_conserved_in_bacteria_[Ruminococcus_bromii_YE282]	Ruminococcus_bromii_YE282
Org1_Gene1243	core	676	2595399200_IE32DRAFT_01288_signal_recognition_particle_subunit_FFH/SRP54_(srp54)_[Ruminococcus_bromii_YE282]	Ruminococcus_bromii_YE282
Org1_Gene1244	core	4034	2595399201_IE32DRAFT_01289_ribosomal_protein_S16_[Ruminococcus_bromii_YE282]	Ruminococcus_bromii_YE282
Org1_Gene1245	core	4080	2595399202_IE32DRAFT_01290_Predicted_RNA-binding_protein_(contains_KH_domain)_[Ruminococcus_bromii_YE282]	Ruminococcus_bromii_YE282
Org1_Gene1265	core	2793	2595399223_IE32DRAFT_01311_dTDP-4-dehydrorhamnose_3,5-epimerase_[Ruminococcus_bromii_YE282]	Ruminococcus_bromii_YE282
Org1_Gene1266	core	1601	2595399224_IE32DRAFT_01312_Nucleoside-diphosphate-sugar_epimerases_[Ruminococcus_bromii_YE282]	Ruminococcus_bromii_YE282
Org1_Gene1267	core	1311	2595399225_IE32DRAFT_01313_CDP-glucose_4,6-dehydratase_[Ruminococcus_bromii_YE282]	Ruminococcus_bromii_YE282
Org1_Gene1268	core	2109	2595399226_IE32DRAFT_01314_glucose-1-phosphate_cytidylyltransferase_[Ruminococcus_bromii_YE282]	Ruminococcus_bromii_YE282
Org1_Gene1272	core	1321	2595399230_IE32DRAFT_01318_Phosphate_starvation-inducible_protein_PhoH,_predicted_ATPase_[Ruminococcus_bromii_YE282]	Ruminococcus_bromii_YE282
Org1_Gene1273	core	3007	2595399231_IE32DRAFT_01319_probable_rRNA_maturation_factor_YbeY_[Ruminococcus_bromii_YE282]	Ruminococcus_bromii_YE282
Org1_Gene1275	core	1643	2595399233_IE32DRAFT_01321_GTP-binding_protein_Era_[Ruminococcus_bromii_YE282]	Ruminococcus_bromii_YE282
Org1_Gene1278	core	4338	2595399236_IE32DRAFT_01324_hypothetical_protein_[Ruminococcus_bromii_YE282]	Ruminococcus_bromii_YE282
Org1_Gene1279	core	2204	2595399237_IE32DRAFT_01325_DNA_replication_and_repair_protein_RecO_[Ruminococcus_bromii_YE282]	Ruminococcus_bromii_YE282
Org1_Gene1280	core	135	2595399238_IE32DRAFT_01326_MutS2_family_protein_[Ruminococcus_bromii_YE282]	Ruminococcus_bromii_YE282
Org1_Gene1281	core	3006	2595399239_IE32DRAFT_01327_16S_rRNA_processing_protein_RimM_[Ruminococcus_bromii_YE282]	Ruminococcus_bromii_YE282
Org1_Gene1282	core	2404	2595399240_IE32DRAFT_01328_tRNA_(guanine-N1)-methyltransferase_[Ruminococcus_bromii_YE282]	Ruminococcus_bromii_YE282
Org1_Gene1283	core	3960	2595399241_IE32DRAFT_01329_Phosphotransferase_System_HPr_(HPr)_Family_[Ruminococcus_bromii_YE282]	Ruminococcus_bromii_YE282
Org1_Gene1284	core	347	2595399242_IE32DRAFT_01330_Excinuclease_ABC_subunit_C_[Ruminococcus_bromii_YE282]	Ruminococcus_bromii_YE282
Org1_Gene1285	core	2851	2595399243_IE32DRAFT_01331_16S_rRNA_(guanine(966)-N(2))-methyltransferase_RsmD_[Ruminococcus_bromii_YE282]	Ruminococcus_bromii_YE282
Org1_Gene1287	core	3206	2595399245_IE32DRAFT_01333_hypothetical_protein_[Ruminococcus_bromii_YE282]	Ruminococcus_bromii_YE282
Org1_Gene1289	core	1626	2595399247_IE32DRAFT_01335_16S_rRNA_(cytosine(1402)-N(4))-methyltransferase_[Ruminococcus_bromii_YE282]	Ruminococcus_bromii_YE282
Org1_Gene1291	core	194	2595399249_IE32DRAFT_01337_Cell_division_protein_FtsI/penicillin-binding_protein_2_[Ruminococcus_bromii_YE282]	Ruminococcus_bromii_YE282
Org1_Gene1292	core	1356	2595399250_IE32DRAFT_01338_Phospho-N-acetylmuramoyl-pentapeptide-transferase_(EC_2.7.8.13)_[Ruminococcus_bromii_YE282]	Ruminococcus_bromii_YE282
Org1_Gene1293	core	928	2595399251_IE32DRAFT_01339_Bacterial_cell_division_membrane_protein_[Ruminococcus_bromii_YE282]	Ruminococcus_bromii_YE282
Org1_Gene1294	core	1196	2595399252_IE32DRAFT_01340_UDP-N-acetylglucosamine-N-acetylmuramylpentapeptide_N-acetylglucosamine_transferase_[Ruminococcus_bromii_YE28	Ruminococcus_bromii_YE282
Org1_Gene1295	core	933	2595399253_IE32DRAFT_01341_UDP-N-acetylglucosamine_1-carboxyvinyltransferase_[Ruminococcus_bromii_YE282]	Ruminococcus_bromii_YE282
Org1_Gene1297	core	1214	2595399255_IE32DRAFT_01343_cell_division_protein_FtsZ_[Ruminococcus_bromii_YE282]	Ruminococcus_bromii_YE282
Org1_Gene1299	core	701	2595399257_IE32DRAFT_01345_Predicted_membrane_protein_involved_in_D-alanine_export_[Ruminococcus_bromii_YE282]	Ruminococcus_bromii_YE282
Org1_Gene13	core	1201	2595397934_IE32DRAFT_00015_peptide_chain_release_factor_2_[Ruminococcus_bromii_YE282]	Ruminococcus_bromii_YE282
Org1_Gene1302	core	3832	2595399260_IE32DRAFT_01348_Trp_operon_repressor_family_[Ruminococcus_bromii_YE282]	Ruminococcus_bromii_YE282
Org1_Gene1304	core	1730	2595399262_IE32DRAFT_01350_Disulfide_bond_chaperones_of_the_HSP33_family_[Ruminococcus_bromii_YE282]	Ruminococcus_bromii_YE282
Org1_Gene1306	core	2177	2595399268_IE32DRAFT_01356_imidazoleglycerol_phosphate_synthase,_cyclase_subunit_[Ruminococcus_bromii_YE282]	Ruminococcus_bromii_YE282
Org1_Gene1307	core	2620	2595399269_IE32DRAFT_01357_imidazole_glycerol_phosphate_synthase,_glutamine_amidotransferase_subunit_[Ruminococcus_bromii_YE282]	Ruminococcus_bromii_YE282
Org1_Gene1308	core	1715	2595399270_IE32DRAFT_01358_transcriptional_regulator,_RpiR_family_[Ruminococcus_bromii_YE282]	Ruminococcus_bromii_YE282
Org1_Gene1309	core	952	2595399271_IE32DRAFT_01359_flavoprotein,_HI0933_family_[Ruminococcus_bromii_YE282]	Ruminococcus_bromii_YE282
Org1_Gene1310	core	2482	2595399272_IE32DRAFT_01360_cytidylate_kinase_[Ruminococcus_bromii_YE282]	Ruminococcus_bromii_YE282
Org1_Gene1311	core	2586	2595399273_IE32DRAFT_01361_1-acyl-sn-glycerol-3-phosphate_acyltransferase_[Ruminococcus_bromii_YE282]	Ruminococcus_bromii_YE282
Org1_Gene1312	core	279	2595399274_IE32DRAFT_01362_4-hydroxy-3-methylbut-2-enyl_diphosphate_reductase_(EC_1.17.1.2)_[Ruminococcus_bromii_YE282]	Ruminococcus_bromii_YE282
Org1_Gene1313	core	2771	2595399275_IE32DRAFT_01363_hypothetical_protein_[Ruminococcus_bromii_YE282]	Ruminococcus_bromii_YE282
Org1_Gene1315	core	283	2595399277_IE32DRAFT_01365_DNA_ligase,_NAD-dependent_[Ruminococcus_bromii_YE282]	Ruminococcus_bromii_YE282
Org1_Gene1318	core	4250	2595399280_IE32DRAFT_01368_stage_III_sporulation_protein_AC_[Ruminococcus_bromii_YE282]	Ruminococcus_bromii_YE282
Org1_Gene1322	core	3249	2595399284_IE32DRAFT_01372_hypothetical_protein_[Ruminococcus_bromii_YE282]	Ruminococcus_bromii_YE282
Org1_Gene1325	core	1552	2595399287_IE32DRAFT_01375_Metal-dependent_proteases_with_possible_chaperone_activity_[Ruminococcus_bromii_YE282]	Ruminococcus_bromii_YE282
Org1_Gene1327	core	52	2595399289_IE32DRAFT_01377_DNA_polymerase_III_catalytic_subunit,_DnaE_type_[Ruminococcus_bromii_YE282]	Ruminococcus_bromii_YE282
Org1_Gene1329	core	1485	2595399291_IE32DRAFT_01379_ABC-type_uncharacterized_transport_system,_permease_component_[Ruminococcus_bromii_YE282]	Ruminococcus_bromii_YE282
Org1_Gene1330	core	2031	2595399292_IE32DRAFT_01380_ABC-type_uncharacterized_transport_system,_ATPase_component_[Ruminococcus_bromii_YE282]	Ruminococcus_bromii_YE282
Org1_Gene1331	core	1898	2595399293_IE32DRAFT_01381_Formate/nitrite_family_of_transporters_[Ruminococcus_bromii_YE282]	Ruminococcus_bromii_YE282
Org1_Gene1333	core	3886	2595399295_IE32DRAFT_01383_Co-chaperonin_GroES_(HSP10)_[Ruminococcus_bromii_YE282]	Ruminococcus_bromii_YE282
Org1_Gene1334	core	487	2595399296_IE32DRAFT_01384_chaperonin_GroL_[Ruminococcus_bromii_YE282]	Ruminococcus_bromii_YE282
Org1_Gene1335	core	3967	2595399297_IE32DRAFT_01385_Coenzyme_PQQ_synthesis_protein_D_(PqqD)_[Ruminococcus_bromii_YE282]	Ruminococcus_bromii_YE282
Org1_Gene1339	core	1512	2595399301_IE32DRAFT_01389_Glycerol-3-phosphate_dehydrogenase_[Ruminococcus_bromii_YE282]	Ruminococcus_bromii_YE282
Org1_Gene1341	core	2431	2595399303_IE32DRAFT_01391_Sugar_transferases_involved_in_lipopolysaccharide_synthesis_[Ruminococcus_bromii_YE282]	Ruminococcus_bromii_YE282
Org1_Gene1343	core	313	2595399305_IE32DRAFT_01393_NAD+_synthetase_[Ruminococcus_bromii_YE282]	Ruminococcus_bromii_YE282
Org1_Gene1345	core	3130	2595399307_IE32DRAFT_01395_Transcriptional_regulators_[Ruminococcus_bromii_YE282]	Ruminococcus_bromii_YE282
Org1_Gene1346	core	1075	2595399308_IE32DRAFT_01396_Aspartate/tyrosine/aromatic_aminotransferase_[Ruminococcus_bromii_YE282]	Ruminococcus_bromii_YE282
Org1_Gene1347	core	1801	2595399309_IE32DRAFT_01397_4-diphosphocytidyl-2-C-methyl-D-erythritol_kinase_(EC_2.7.1.148)_[Ruminococcus_bromii_YE282]	Ruminococcus_bromii_YE282
Org1_Gene1364	core	536	2595399326_IE32DRAFT_01414_GMP_synthase_(glutamine-hydrolyzing),_C-terminal_domain_or_B_subunit/GMP_synthase_(glutamine-hydrolyzing),_N-t	Ruminococcus_bromii_YE282
Org1_Gene1365	core	894	2595399327_IE32DRAFT_01415_Adenylosuccinate_synthetase_(EC_6.3.4.4)_[Ruminococcus_bromii_YE282]	Ruminococcus_bromii_YE282
Org1_Gene1366	core	647	2595399328_IE32DRAFT_01416_adenylosuccinate_lyase_[Ruminococcus_bromii_YE282]	Ruminococcus_bromii_YE282
Org1_Gene1367	core	613	2595399329_IE32DRAFT_01417_amidophosphoribosyltransferase_(EC_2.4.2.14)_[Ruminococcus_bromii_YE282]	Ruminococcus_bromii_YE282
Org1_Gene1368	core	2350	2595399330_IE32DRAFT_01418_phosphoribosylaminoimidazole-succinocarboxamide_synthase_(EC_6.3.2.6)_[Ruminococcus_bromii_YE282]	Ruminococcus_bromii_YE282
Org1_Gene1369	core	914	2595399332_IE32DRAFT_01420_phosphoribosylamine--glycine_ligase_(EC_6.3.4.13)_[Ruminococcus_bromii_YE282]	Ruminococcus_bromii_YE282
Org1_Gene1370	core	1076	2595399333_IE32DRAFT_01421_AICARFT/IMPCHase_bienzyme_[Ruminococcus_bromii_YE282]	Ruminococcus_bromii_YE282
Org1_Gene1371	core	2308	2595399334_IE32DRAFT_01422_IMP_cyclohydrolase-like_protein_[Ruminococcus_bromii_YE282]	Ruminococcus_bromii_YE282
Org1_Gene1372	core	2614	2595399335_IE32DRAFT_01423_phosphoribosylglycinamide_formyltransferase,_formyltetrahydrofolate-dependent_[Ruminococcus_bromii_YE282]	Ruminococcus_bromii_YE282
Org1_Gene1373	core	1351	2595399336_IE32DRAFT_01424_phosphoribosylformylglycinamidine_cyclo-ligase_(EC_6.3.3.1)_[Ruminococcus_bromii_YE282]	Ruminococcus_bromii_YE282
Org1_Gene1374	core	633	2595399337_IE32DRAFT_01425_amidophosphoribosyltransferase_(EC_2.4.2.14)_[Ruminococcus_bromii_YE282]	Ruminococcus_bromii_YE282
Org1_Gene1375	core	3072	2595399338_IE32DRAFT_01426_phosphoribosylaminoimidazole_carboxylase,_PurE_protein_[Ruminococcus_bromii_YE282]	Ruminococcus_bromii_YE282
Org1_Gene1377	core	1537	2595399340_IE32DRAFT_01428_Membrane_protease_subunits,_stomatin/prohibitin_homologs_[Ruminococcus_bromii_YE282]	Ruminococcus_bromii_YE282
Org1_Gene1381	core	4015	2595399344_IE32DRAFT_01432_protein_translocase,_SecG_subunit_[Ruminococcus_bromii_YE282]	Ruminococcus_bromii_YE282
Org1_Gene1382	core	203	2595399345_IE32DRAFT_01433_Type_IIA_topoisomerase_(DNA_gyrase/topo_II,_topoisomerase_IV),_A_subunit_[Ruminococcus_bromii_YE282]	Ruminococcus_bromii_YE282
Org1_Gene1383	core	282	2595399346_IE32DRAFT_01434_Type_IIA_topoisomerase_(DNA_gyrase/topo_II,_topoisomerase_IV),_B_subunit_[Ruminococcus_bromii_YE282]	Ruminococcus_bromii_YE282
Org1_Gene1384	core	290	2595399347_IE32DRAFT_01435_Excinuclease_ABC_subunit_B_[Ruminococcus_bromii_YE282]	Ruminococcus_bromii_YE282
Org1_Gene1385	core	2348	2595399348_IE32DRAFT_01436_DNA_repair_proteins_[Ruminococcus_bromii_YE282]	Ruminococcus_bromii_YE282
Org1_Gene1386	core	2443	2595399349_IE32DRAFT_01437_orotate_phosphoribosyltransferase_(EC_2.4.2.10)_[Ruminococcus_bromii_YE282]	Ruminococcus_bromii_YE282
Org1_Gene1387	core	898	2595399350_IE32DRAFT_01438_Nucleotidyltransferase/DNA_polymerase_involved_in_DNA_repair_[Ruminococcus_bromii_YE282]	Ruminococcus_bromii_YE282
Org1_Gene1388	core	4043	2595399351_IE32DRAFT_01439_hypothetical_protein_[Ruminococcus_bromii_YE282]	Ruminococcus_bromii_YE282
Org1_Gene1393	core	990	2595399356_IE32DRAFT_01444_Alcohol_dehydrogenase,_class_IV_[Ruminococcus_bromii_YE282]	Ruminococcus_bromii_YE282
Org1_Gene14	core	1002	2595397935_IE32DRAFT_00016_proposed_homoserine_kinase_[Ruminococcus_bromii_YE282]	Ruminococcus_bromii_YE282
Org1_Gene1412	core	659	2595399376_IE32DRAFT_01464_ABC-type_spermidine/putrescine_transport_systems,_ATPase_components_[Ruminococcus_bromii_YE282]	Ruminococcus_bromii_YE282
Org1_Gene1413	core	2069	2595399377_IE32DRAFT_01465_ABC-type_spermidine/putrescine_transport_system,_permease_component_I_[Ruminococcus_bromii_YE282]	Ruminococcus_bromii_YE282
Org1_Gene1414	core	1988	2595399378_IE32DRAFT_01466_ABC-type_spermidine/putrescine_transport_system,_permease_component_II_[Ruminococcus_bromii_YE282]	Ruminococcus_bromii_YE282
Org1_Gene1415	core	1251	2595399379_IE32DRAFT_01467_Spermidine/putrescine-binding_periplasmic_protein_[Ruminococcus_bromii_YE282]	Ruminococcus_bromii_YE282
Org1_Gene1421	core	4042	2595399385_IE32DRAFT_01473_Ribosome-associated_heat_shock_protein_implicated_in_the_recycling_of_the_50S_subunit_(S4_paralog)_[Ruminococcu	Ruminococcus_bromii_YE282
Org1_Gene1422	core	153	2595399386_IE32DRAFT_01474_Glycosidases_[Ruminococcus_bromii_YE282]	Ruminococcus_bromii_YE282
Org1_Gene1424	core	913	2595399388_IE32DRAFT_01476_ATPase_related_to_the_helicase_subunit_of_the_Holliday_junction_resolvase_[Ruminococcus_bromii_YE282]	Ruminococcus_bromii_YE282
Org1_Gene1425	core	931	2595399389_IE32DRAFT_01477_Glycine/serine_hydroxymethyltransferase_[Ruminococcus_bromii_YE282]	Ruminococcus_bromii_YE282
Org1_Gene1426	core	1439	2595399390_IE32DRAFT_01478_tRNA-U20-dihydrouridine_synthase_[Ruminococcus_bromii_YE282]	Ruminococcus_bromii_YE282
Org1_Gene1429	core	1782	2595399393_IE32DRAFT_01481_Germination_protease_[Ruminococcus_bromii_YE282]	Ruminococcus_bromii_YE282
Org1_Gene1493	core	2103	2595399457_IE32DRAFT_01545_RNA_polymerase,_sigma_subunit,_RpsG/SigG_[Ruminococcus_bromii_YE282]	Ruminococcus_bromii_YE282
Org1_Gene1494	core	2876	2595399458_IE32DRAFT_01546_PAP2_superfamily_[Ruminococcus_bromii_YE282]	Ruminococcus_bromii_YE282
Org1_Gene1495	core	1402	2595399459_IE32DRAFT_01547_stage_V_sporulation_protein_AD_[Ruminococcus_bromii_YE282]	Ruminococcus_bromii_YE282
Org1_Gene1499	core	3350	2595399463_IE32DRAFT_01551_RNAse_H-fold_protein_YqgF_[Ruminococcus_bromii_YE282]	Ruminococcus_bromii_YE282
Org1_Gene1500	core	657	2595399464_IE32DRAFT_01552_glycogen/starch_synthase,_ADP-glucose_type_[Ruminococcus_bromii_YE282]	Ruminococcus_bromii_YE282
Org1_Gene1501	core	1212	2595399465_IE32DRAFT_01553_glucose-1-phosphate_adenylyltransferase,_GlgD_subunit_[Ruminococcus_bromii_YE282]	Ruminococcus_bromii_YE282
Org1_Gene1502	core	999	2595399466_IE32DRAFT_01554_glucose-1-phosphate_adenylyltransferase_[Ruminococcus_bromii_YE282]	Ruminococcus_bromii_YE282
Org1_Gene1504	core	1352	2595399468_IE32DRAFT_01556_glyceraldehyde-3-phosphate_dehydrogenase,_type_I_[Ruminococcus_bromii_YE282]	Ruminococcus_bromii_YE282
Org1_Gene1505	core	537	2595399469_IE32DRAFT_01557_ribonuclease_Y_[Ruminococcus_bromii_YE282]	Ruminococcus_bromii_YE282
Org1_Gene1506	core	44	2595399470_IE32DRAFT_01558_DNA-directed_RNA_polymerase,_beta'_subunit,_predominant_form_[Ruminococcus_bromii_YE282]	Ruminococcus_bromii_YE282
Org1_Gene1507	core	40	2595399471_IE32DRAFT_01559_DNA-directed_RNA_polymerase_subunit_beta_(EC_2.7.7.6)_[Ruminococcus_bromii_YE282]	Ruminococcus_bromii_YE282
Org1_Gene1509	core	1834	2595399473_IE32DRAFT_01561_yjeF_N-terminal_region_[Ruminococcus_bromii_YE282]	Ruminococcus_bromii_YE282
Org1_Gene1511	core	3607	2595399475_IE32DRAFT_01563_Protein_of_unknown_function_(DUF1292)_[Ruminococcus_bromii_YE282]	Ruminococcus_bromii_YE282
Org1_Gene1514	core	926	2595399478_IE32DRAFT_01566_Chloride_channel_protein_EriC_[Ruminococcus_bromii_YE282]	Ruminococcus_bromii_YE282
Org1_Gene1516	core	1555	2595399480_IE32DRAFT_01568_Lactate_dehydrogenase_and_related_dehydrogenases_[Ruminococcus_bromii_YE282]	Ruminococcus_bromii_YE282
Org1_Gene1518	core	330	2595399482_IE32DRAFT_01570_Molecular_chaperone,_HSP90_family_[Ruminococcus_bromii_YE282]	Ruminococcus_bromii_YE282

Org1_Gene1544	core	4046	2595399508_IE32DRAFT_01596_looped-hinge_helix_DNA_binding_domain,_AbrB_family_[Ruminococcus_bromii_YE282]	Ruminococcus_bromii_YE282
Org1_Gene1545	core	2757	2595399509_IE32DRAFT_01597_Uncharacterized_membrane_protein,_required_for_spore_maturation_in_B.subtilis._[Ruminococcus_bromii_YE282]	Ruminococcus_bromii_YE282
Org1_Gene1546	core	3055	2595399510_IE32DRAFT_01598_Uncharacterized_membrane_protein_[Ruminococcus_bromii_YE282]	Ruminococcus_bromii_YE282
Org1_Gene1548	core	2166	2595399512_IE32DRAFT_01600_rRNA_methylase,_putative,_group_3_[Ruminococcus_bromii_YE282]	Ruminococcus_bromii_YE282
Org1_Gene1550	core	2522	2595399514_IE32DRAFT_01602_ribulose-phosphate_3-epimerase_[Ruminococcus_bromii_YE282]	Ruminococcus_bromii_YE282
Org1_Gene1560	core	2501	2595399524_IE32DRAFT_01612_RNA_polymerase_sigma_factor,_sigma-70_family_[Ruminococcus_bromii_YE282]	Ruminococcus_bromii_YE282
Org1_Gene1561	core	3296	2595399525_IE32DRAFT_01613_anti-sigma_F_factor_[Ruminococcus_bromii_YE282]	Ruminococcus_bromii_YE282
Org1_Gene1562	core	3765	2595399526_IE32DRAFT_01614_anti-anti-sigma_factor_[Ruminococcus_bromii_YE282]	Ruminococcus_bromii_YE282
Org1_Gene1564	core	1955	2595399528_IE32DRAFT_01616_16S_rRNA_(cytidine(1402)-2'-O)-methyltransferase_[Ruminococcus_bromii_YE282]	Ruminococcus_bromii_YE282
Org1_Gene1566	core	4343	2595399530_IE32DRAFT_01618_4Fe-4S_dicluster_domain_[Ruminococcus_bromii_YE282]	Ruminococcus_bromii_YE282
Org1_Gene1567	core	1810	2595399531_IE32DRAFT_01619_Uncharacterized_homolog_of_PSP1_[Ruminococcus_bromii_YE282]	Ruminococcus_bromii_YE282
Org1_Gene1573	core	945	2595399537_IE32DRAFT_01625_glutamate-5-semialdehyde_dehydrogenase_(EC_1.2.1.41)_[Ruminococcus_bromii_YE282]	Ruminococcus_bromii_YE282
Org1_Gene1574	core	1993	2595399538_IE32DRAFT_01626_glutamate_5-kinase_[Ruminococcus_bromii_YE282]	Ruminococcus_bromii_YE282
Org1_Gene1580	core	639	2595399544_IE32DRAFT_01632_pyruvate_kinase_[Ruminococcus_bromii_YE282]	Ruminococcus_bromii_YE282
Org1_Gene1604	core	1145	2595399568_IE32DRAFT_01656_Purine_catabolism_regulatory_protein-like_family/PucR_C-terminal_helix-turn-helix_domain_[Ruminococcus_bromii_YE2	Ruminococcus_bromii_YE282
Org1_Gene1609	core	2221	2595399573_IE32DRAFT_01661_Dehydrogenases_with_different_specificities_(related_to_short-chain_alcohol_dehydrogenases)_[Ruminococcus_bromii_	Ruminococcus_bromii_YE282
Org1_Gene1611	core	4393	2595399575_IE32DRAFT_01663_ribosomal_protein_L33,_bacterial_type_[Ruminococcus_bromii_YE282]	Ruminococcus_bromii_YE282
Org1_Gene1613	core	2971	2595399577_IE32DRAFT_01665_transcription_antitermination_protein_nusG_[Ruminococcus_bromii_YE282]	Ruminococcus_bromii_YE282
Org1_Gene1614	core	3352	2595399578_IE32DRAFT_01666_LSU_ribosomal_protein_L11P_[Ruminococcus_bromii_YE282]	Ruminococcus_bromii_YE282
Org1_Gene1615	core	2398	2595399579_IE32DRAFT_01667_ribosomal_protein_L1,_bacterial/chloroplast_[Ruminococcus_bromii_YE282]	Ruminococcus_bromii_YE282
Org1_Gene1616	core	2838	2595399580_IE32DRAFT_01668_Ribosomal_protein_L10_[Ruminococcus_bromii_YE282]	Ruminococcus_bromii_YE282
Org1_Gene1617	core	3530	2595399581_IE32DRAFT_01669_ribosomal_protein_L7/L12_[Ruminococcus_bromii_YE282]	Ruminococcus_bromii_YE282
Org1_Gene1619	core	434	2595399583_IE32DRAFT_01671_glutaminyl-tRNA_synthetase_(EC_6.1.1.18)_[Ruminococcus_bromii_YE282]	Ruminococcus_bromii_YE282
Org1_Gene1620	core	626	2595399584_IE32DRAFT_01672_glutamyl-tRNA_synthetase,_bacterial_family_[Ruminococcus_bromii_YE282]	Ruminococcus_bromii_YE282
Org1_Gene1622	core	3338	2595399586_IE32DRAFT_01674_hypothetical_protein_[Ruminococcus_bromii_YE282]	Ruminococcus_bromii_YE282
Org1_Gene1624	core	2814	2595399588_IE32DRAFT_01676_Propanediol_utilization_protein_[Ruminococcus_bromii_YE282]	Ruminococcus_bromii_YE282
Org1_Gene1625	core	2781	2595399589_IE32DRAFT_01677_uracil-DNA_glycosylase,_family_4_[Ruminococcus_bromii_YE282]	Ruminococcus_bromii_YE282
Org1_Gene1626	core	399	2595399590_IE32DRAFT_01678_aspartyl-tRNA_synthetase,_bacterial_type_[Ruminococcus_bromii_YE282]	Ruminococcus_bromii_YE282
Org1_Gene1627	core	3910	2595399591_IE32DRAFT_01679_aspartyl/glutamyl-tRNA(Asn/Gln)_amidotransferase_subunit_C_(EC_6.3.5.-)_[Ruminococcus_bromii_YE282]	Ruminococcus_bromii_YE282
Org1_Gene1628	core	628	2595399592_IE32DRAFT_01680_aspartyl/glutamyl-tRNA(Asn/Gln)_amidotransferase_subunit_A_(EC_6.3.5.-)_[Ruminococcus_bromii_YE282]	Ruminococcus_bromii_YE282
Org1_Gene1629	core	654	2595399593_IE32DRAFT_01681_aspartyl/glutamyl-tRNA(Asn/Gln)_amidotransferase_subunit_B_(EC_6.3.5.-)_[Ruminococcus_bromii_YE282]	Ruminococcus_bromii_YE282
Org1_Gene1631	core	2136	2595399595_IE32DRAFT_01683_sporulation_transcription_factor_Spo0A_[Ruminococcus_bromii_YE282]	Ruminococcus_bromii_YE282
Org1_Gene1640	core	796	2595399604_IE32DRAFT_01692_Glutamate_dehydrogenase/leucine_dehydrogenase_[Ruminococcus_bromii_YE282]	Ruminococcus_bromii_YE282
Org1_Gene1641	core	1460	2595399605_IE32DRAFT_01693_Site-specific_recombinase_XerD_[Ruminococcus_bromii_YE282]	Ruminococcus_bromii_YE282
Org1_Gene1643	core	734	2595399607_IE32DRAFT_01695_DNA_repair_protein_RadA_[Ruminococcus_bromii_YE282]	Ruminococcus_bromii_YE282
Org1_Gene1645	core	1789	2595399609_IE32DRAFT_01697_methionine_aminopeptidase,_type_I_[Ruminococcus_bromii_YE282]	Ruminococcus_bromii_YE282
Org1_Gene1647	core	318	2595399611_IE32DRAFT_01699_Fe2+_transport_system_protein_B_[Ruminococcus_bromii_YE282]	Ruminococcus_bromii_YE282
Org1_Gene1648	core	724	2595399612_IE32DRAFT_01700_SCIFF_radical_SAM_maturase_[Ruminococcus_bromii_YE282]	Ruminococcus_bromii_YE282
Org1_Gene1649	core	4381	2595399613_IE32DRAFT_01701_six-cysteine_peptide_SCIFF_[Ruminococcus_bromii_YE282]	Ruminococcus_bromii_YE282
Org1_Gene1651	core	520	2595399615_IE32DRAFT_01703_peptide_chain_release_factor_3_[Ruminococcus_bromii_YE282]	Ruminococcus_bromii_YE282
Org1_Gene1652	core	413	2595399616_IE32DRAFT_01704_Phosphomannomutase_[Ruminococcus_bromii_YE282]	Ruminococcus_bromii_YE282
Org1_Gene1654	core	1540	2595399618_IE32DRAFT_01706_Glycosyltransferases_involved_in_cell_wall_biogenesis_[Ruminococcus_bromii_YE282]	Ruminococcus_bromii_YE282
Org1_Gene1655	core	1483	2595399619_IE32DRAFT_01707_6-phosphofructokinase_(EC_2.7.1.11)_[Ruminococcus_bromii_YE282]	Ruminococcus_bromii_YE282
Org1_Gene1669	core	2892	2595399633_IE32DRAFT_01721_Cys-tRNA(Pro)_deacylase_[Ruminococcus_bromii_YE282]	Ruminococcus_bromii_YE282
Org1_Gene1672	core	3160	2595399636_IE32DRAFT_01724_ATP_synthase_subunit_C_[Ruminococcus_bromii_YE282]	Ruminococcus_bromii_YE282
Org1_Gene1675	core	3757	2595399639_IE32DRAFT_01727_Archaeal/vacuolar-type_H+-ATPase_subunit_F_[Ruminococcus_bromii_YE282]	Ruminococcus_bromii_YE282
Org1_Gene1676	core	384	2595399640_IE32DRAFT_01728_Archaeal/vacuolar-type_H+-ATPase_subunit_A_[Ruminococcus_bromii_YE282]	Ruminococcus_bromii_YE282
Org1_Gene1677	core	723	2595399641_IE32DRAFT_01729_Archaeal/vacuolar-type_H+-ATPase_subunit_B_[Ruminococcus_bromii_YE282]	Ruminococcus_bromii_YE282
Org1_Gene1678	core	2494	2595399642_IE32DRAFT_01730_H(+)-transporting_ATP_synthase,_vacuolar_type,_subunit_D_[Ruminococcus_bromii_YE282]	Ruminococcus_bromii_YE282
Org1_Gene1682	core	199	2595399646_IE32DRAFT_01734_formate_acetyltransferase_1_[Ruminococcus_bromii_YE282]	Ruminococcus_bromii_YE282
Org1_Gene1684	core	510	2595399648_IE32DRAFT_01736_phosphoenolpyruvate_carboxykinase_(ATP)_[Ruminococcus_bromii_YE282]	Ruminococcus_bromii_YE282
Org1_Gene1692	core	738	2595399656_IE32DRAFT_01744_UDP-N-acetylglucosamine_1-carboxyvinyltransferase_[Ruminococcus_bromii_YE282]	Ruminococcus_bromii_YE282
Org1_Gene1695	core	3710	2595399659_IE32DRAFT_01747_hypothetical_protein_[Ruminococcus_bromii_YE282]	Ruminococcus_bromii_YE282
Org1_Gene1696	core	717	2595399660_IE32DRAFT_01748_pyridoxal-phosphate_dependent_TrpB-like_enzyme_[Ruminococcus_bromii_YE282]	Ruminococcus_bromii_YE282
Org1_Gene1697	core	189	2595399661_IE32DRAFT_01749_alpha-1,4-glucan:alpha-1,4-glucan_6-glycosyltransferase_[Ruminococcus_bromii_YE282]	Ruminococcus_bromii_YE282
Org1_Gene1699	core	996	2595399663_IE32DRAFT_01751_acetate_kinase_[Ruminococcus_bromii_YE282]	Ruminococcus_bromii_YE282
Org1_Gene1700	core	403	2595399664_IE32DRAFT_01752_Predicted_RNA-binding_protein_homologous_to_eukaryotic_snRNP_[Ruminococcus_bromii_YE282]	Ruminococcus_bromii_YE282
Org1_Gene1701	core	1238	2595399665_IE32DRAFT_01753_GTP-binding_protein_YchF_[Ruminococcus_bromii_YE282]	Ruminococcus_bromii_YE282
Org1_Gene1702	core	3496	2595399666_IE32DRAFT_01754_Predicted_thioesterase_[Ruminococcus_bromii_YE282]	Ruminococcus_bromii_YE282
Org1_Gene1706	core	1217	2595399670_IE32DRAFT_01758_ABC-type_sugar_transport_systems,_ATPase_components_[Ruminococcus_bromii_YE282]	Ruminococcus_bromii_YE282
Org1_Gene1707	core	1258	2595399671_IE32DRAFT_01759_Sugar_diacid_utilization_regulator_[Ruminococcus_bromii_YE282]	Ruminococcus_bromii_YE282
Org1_Gene1708	core	2132	2595399672_IE32DRAFT_01760_cell_division_ATP-binding_protein_FtsE_[Ruminococcus_bromii_YE282]	Ruminococcus_bromii_YE282
Org1_Gene1712	core	3129	2595399676_IE32DRAFT_01764_transcription_elongation_factor_GreA_[Ruminococcus_bromii_YE282]	Ruminococcus_bromii_YE282
Org1_Gene1713	core	310	2595399677_IE32DRAFT_01765_lysyl-tRNA_synthetase,_class_II_(EC_6.1.1.6)_[Ruminococcus_bromii_YE282]	Ruminococcus_bromii_YE282
Org1_Gene1725	core	319	2595399690_IE32DRAFT_01778_Alpha-glucosidases,_family_31_of_glycosyl_hydrolases_[Ruminococcus_bromii_YE282]	Ruminococcus_bromii_YE282
Org1_Gene1726	core	164	2595399691_IE32DRAFT_01779_asparagine_synthase_(glutamine-hydrolyzing)_[Ruminococcus_bromii_YE282]	Ruminococcus_bromii_YE282
Org1_Gene1728	core	2888	2595399693_IE32DRAFT_01781_NTP_pyrophosphohydrolases_including_oxidative_damage_repair_enzymes_[Ruminococcus_bromii_YE282]	Ruminococcus_bromii_YE282
Org1_Gene1730	core	578	2595399695_IE32DRAFT_01783_Phosphomannomutase_[Ruminococcus_bromii_YE282]	Ruminococcus_bromii_YE282
Org1_Gene1731	core	3957	2595399696_IE32DRAFT_01784_hypothetical_protein_[Ruminococcus_bromii_YE282]	Ruminococcus_bromii_YE282
Org1_Gene1735	core	2195	2595399701_IE32DRAFT_01789_Phosphotransferase_enzyme_family_[Ruminococcus_bromii_YE282]	Ruminococcus_bromii_YE282
Org1_Gene1736	core	636	2595399702_IE32DRAFT_01790_Trk-type_K+_transport_systems,_membrane_components_[Ruminococcus_bromii_YE282]	Ruminococcus_bromii_YE282
Org1_Gene1742	core	123	2595399708_IE32DRAFT_01796_acetaldehyde_dehydrogenase_(EC_1.2.1.10)/alcohol_dehydrogenase_AdhE_(EC_1.1.1.1)_[Ruminococcus_bromii_YE282]	Ruminococcus_bromii_YE282
Org1_Gene1743	core	718	2595399709_IE32DRAFT_01797_glycyl-tRNA_synthetase_(EC_6.1.1.14)_[Ruminococcus_bromii_YE282]	Ruminococcus_bromii_YE282
Org1_Gene1749	core	869	2595399715_IE32DRAFT_01803_MiaB-like_tRNA_modifying_enzyme_[Ruminococcus_bromii_YE282]	Ruminococcus_bromii_YE282
Org1_Gene1751	core	3697	2595399717_IE32DRAFT_01805_phosphoribosyl-ATP_pyrophosphatase_(EC_3.6.1.31)_[Ruminococcus_bromii_YE282]	Ruminococcus_bromii_YE282
Org1_Gene1752	core	3785	2595399718_IE32DRAFT_01806_phosphoribosyl-AMP_cyclohydrolase_(EC_3.5.4.19)_[Ruminococcus_bromii_YE282]	Ruminococcus_bromii_YE282
Org1_Gene1753	core	2300	2595399719_IE32DRAFT_01807_1-(5-phosphoribosyl)-5-[(5-phosphoribosylamino)methylideneamino]_imidazole-4-carboxamide_isomerase_(EC_5.3.1.16)_	Ruminococcus_bromii_YE282
Org1_Gene1754	core	2773	2595399720_IE32DRAFT_01808_imidazoleglycerol-phosphate_dehydratase_(EC_4.2.1.19)_[Ruminococcus_bromii_YE282]	Ruminococcus_bromii_YE282
Org1_Gene1755	core	1314	2595399721_IE32DRAFT_01809_histidinol-phosphate_aminotransferase_[Ruminococcus_bromii_YE282]	Ruminococcus_bromii_YE282
Org1_Gene1756	core	864	2595399722_IE32DRAFT_01810_histidinol_dehydrogenase_[Ruminococcus_bromii_YE282]	Ruminococcus_bromii_YE282
Org1_Gene1757	core	2601	2595399723_IE32DRAFT_01811_ATP_phosphoribosyltransferase_[Ruminococcus_bromii_YE282]	Ruminococcus_bromii_YE282
Org1_Gene1758	core	1098	2595399724_IE32DRAFT_01812_ATP_phosphoribosyltransferase,_regulatory_subunit_[Ruminococcus_bromii_YE282]	Ruminococcus_bromii_YE282
Org1_Gene1760	core	21	2595399726_IE32DRAFT_01814_DNA_polymerase_III,_alpha_chain,_Gram-positive_type_[Ruminococcus_bromii_YE282]	Ruminococcus_bromii_YE282
Org1_Gene1761	core	1335	2595399727_IE32DRAFT_01815_4-hydroxy-3-methylbut-2-en-1-yl_diphosphate_synthase_(EC_1.17.4.3)_[Ruminococcus_bromii_YE282]	Ruminococcus_bromii_YE282
Org1_Gene1763	core	1147	2595399729_IE32DRAFT_01817_1-deoxy-D-xylulose_5-phosphate_reductoisomerase_(EC_1.1.1.267)_[Ruminococcus_bromii_YE282]	Ruminococcus_bromii_YE282
Org1_Gene1765	core	2189	2595399731_IE32DRAFT_01819_undecaprenyl_diphosphate_synthase_[Ruminococcus_bromii_YE282]	Ruminococcus_bromii_YE282
Org1_Gene1766	core	2866	2595399732_IE32DRAFT_01820_ribosome_recycling_factor_[Ruminococcus_bromii_YE282]	Ruminococcus_bromii_YE282
Org1_Gene1767	core	2319	2595399733_IE32DRAFT_01821_uridylate_kinase_(EC_2.7.4.22)_[Ruminococcus_bromii_YE282]	Ruminococcus_bromii_YE282
Org1_Gene1768	core	2223	2595399734_IE32DRAFT_01822_ABC-type_polar_amino_acid_transport_system,_ATPase_component_[Ruminococcus_bromii_YE282]	Ruminococcus_bromii_YE282
Org1_Gene1773	core	981	2595399739_IE32DRAFT_01827_beta-ketoacyl-acyl-carrier-protein_synthase_II_[Ruminococcus_bromii_YE282]	Ruminococcus_bromii_YE282
Org1_Gene1774	core	461	2595399740_IE32DRAFT_01828_Acyl-CoA_synthetases_(AMP-forming)/AMP-acid_ligases_II_[Ruminococcus_bromii_YE282]	Ruminococcus_bromii_YE282
Org1_Gene1778	core	3378	2595399744_IE32DRAFT_01832_SSU_ribosomal_protein_S12P_[Ruminococcus_bromii_YE282]	Ruminococcus_bromii_YE282
Org1_Gene1779	core	2922	2595399745_IE32DRAFT_01833_ribosomal_protein_S7,_bacterial/organelle_[Ruminococcus_bromii_YE282]	Ruminococcus_bromii_YE282
Org1_Gene1780	core	240	2595399746_IE32DRAFT_01834_translation_elongation_factor_EF-G_[Ruminococcus_bromii_YE282]	Ruminococcus_bromii_YE282
Org1_Gene1781	core	1023	2595399747_IE32DRAFT_01835_translation_elongation_factor_TU_[Ruminococcus_bromii_YE282]	Ruminococcus_bromii_YE282
Org1_Gene1782	core	592	2595399748_IE32DRAFT_01836_Xanthine/uracil_permeases_[Ruminococcus_bromii_YE282]	Ruminococcus_bromii_YE282
Org1_Gene1785	core	714	2595399751_IE32DRAFT_01839_Citrate_synthase_[Ruminococcus_bromii_YE282]	Ruminococcus_bromii_YE282
Org1_Gene1787	core	2387	2595399753_IE32DRAFT_01841_CpXC_protein_[Ruminococcus_bromii_YE282]	Ruminococcus_bromii_YE282
Org1_Gene1792	core	727	2595399758_IE32DRAFT_01846_Mg2+_transporter_(mgtE)_[Ruminococcus_bromii_YE282]	Ruminococcus_bromii_YE282
Org1_Gene1795	core	1291	2595399761_IE32DRAFT_01849_methyltransferase,_FkbM_family_[Ruminococcus_bromii_YE282]	Ruminococcus_bromii_YE282
Org1_Gene1797	core	1605	2595399764_IE32DRAFT_01852_ornithine_carbamoyltransferase_[Ruminococcus_bromii_YE282]	Ruminococcus_bromii_YE282
Org1_Gene1798	core	1088	2595399765_IE32DRAFT_01853_transaminase,_acetylornithine/succinylornithine_family_[Ruminococcus_bromii_YE282]	Ruminococcus_bromii_YE282
Org1_Gene1799	core	3359	2595399766_IE32DRAFT_01854_Acetyltransferases_[Ruminococcus_bromii_YE282]	Ruminococcus_bromii_YE282
Org1_Gene18	core	191	2595397941_IE32DRAFT_00022_Superfamily_I_DNA_and_RNA_helicases_[Ruminococcus_bromii_YE282]	Ruminococcus_bromii_YE282
Org1_Gene1800	core	1865	2595399767_IE32DRAFT_01855_N-acetylglutamate_kinase_(EC_2.7.2.8)_[Ruminococcus_bromii_YE282]	Ruminococcus_bromii_YE282
Org1_Gene1801	core	973	2595399768_IE32DRAFT_01856_glutamate_N-acetyltransferase_(EC_2.3.1.35)_[Ruminococcus_bromii_YE282]	Ruminococcus_bromii_YE282
Org1_Gene1802	core	1349	2595399769_IE32DRAFT_01857_N-acetyl-gamma-glutamyl-phosphate_reductase_(EC_1.2.1.38)_[Ruminococcus_bromii_YE282]	Ruminococcus_bromii_YE282
Org1_Gene1803	core	719	2595399770_IE32DRAFT_01858_argininosuccinate_lyase_[Ruminococcus_bromii_YE282]	Ruminococcus_bromii_YE282
Org1_Gene1804	core	983	2595399771_IE32DRAFT_01859_argininosuccinate_synthase_(EC_6.3.4.5)_[Ruminococcus_bromii_YE282]	Ruminococcus_bromii_YE282
Org1_Gene1805	core	3024	2595399772_IE32DRAFT_01860_Conserved_protein/domain_typically_associated_with_flavoprotein_oxygenases,_DIM6/NTAB_family_[Ruminococcus_bro	Ruminococcus_bromii_YE282
Org1_Gene1808	core	444	2595399775_IE32DRAFT_01863_Bacterial_cell_division_membrane_protein_[Ruminococcus_bromii_YE282]	Ruminococcus_bromii_YE282
Org1_Gene1809	core	695	2595399776_IE32DRAFT_01864_Cell_division_protein_FtsI/penicillin-binding_protein_2_[Ruminococcus_bromii_YE282]	Ruminococcus_bromii_YE282
Org1_Gene1810	core	95	2595399777_IE32DRAFT_01865_preprotein_translocase,_SecA_subunit_[Ruminococcus_bromii_YE282]	Ruminococcus_bromii_YE282
Org1_Gene1811	core	951	2595399778_IE32DRAFT_01866_Putative_virion_core_protein_(lumpy_skin_disease_virus)_[Ruminococcus_bromii_YE282]	Ruminococcus_bromii_YE282
Org1_Gene1816	core	648	2595399783_IE32DRAFT_01871_[FeFe]_hydrogenase_H-cluster_radical_SAM_maturase_HydG_[Ruminococcus_bromii_YE282]	Ruminococcus_bromii_YE282
Org1_Gene1819	core	2133	2595399787_IE32DRAFT_01875_tryptophan_synthase,_alpha_chain_(EC_4.2.1.20)_[Ruminococcus_bromii_YE282]	Ruminococcus_bromii_YE282
Org1_Gene1820	core	1062	2595399788_IE32DRAFT_01876_tryptophan_synthase,_beta_subunit_[Ruminococcus_bromii_YE282]	Ruminococcus_bromii_YE282
Org1_Gene1823	core	2111	2595399791_IE32DRAFT_01880_hydrolase,_TatD_family_[Ruminococcus_bromii_YE282]	Ruminococcus_bromii_YE282
Org1_Gene1824	core	297	2595399792_IE32DRAFT_01881_methionyl-tRNA_synthetase_(EC_6.1.1.10)_[Ruminococcus_bromii_YE282]	Ruminococcus_bromii_YE282
Org1_Gene1833	core	378	2595399802_IE32DRAFT_01891_aspartyl-tRNA_synthetase,_bacterial_type_[Ruminococcus_bromii_YE282]	Ruminococcus_bromii_YE282
Org1_Gene1835	core	805	2595399804_IE32DRAFT_01893_histidyl-tRNA_synthetase_(EC_6.1.1.21)_[Ruminococcus_bromii_YE282]	Ruminococcus_bromii_YE282
Org1_Gene1836	core	3475	2595399805_IE32DRAFT_01894_methylglyoxal_synthase_[Ruminococcus_bromii_YE282]	Ruminococcus_bromii_YE282

Org1_Gene1837	core	2226	2595399806_IE32DRAFT_01895_Septum_formation_inhibitor-activating_ATPase_[Ruminococcus_bromii_YE282]	Ruminococcus_bromii_YE282
Org1_Gene1841	core	1398	2595399810_IE32DRAFT_01899_cell_shape_determining_protein,_MreB/Mrl_family_[Ruminococcus_bromii_YE282]	Ruminococcus_bromii_YE282
Org1_Gene1842	core	3264	2595399811_IE32DRAFT_01900_deoxyuridine_5'-triphosphate_nucleotidohydrolase_(dut)_[Ruminococcus_bromii_YE282]	Ruminococcus_bromii_YE282
Org1_Gene1846	core	746	2595399815_IE32DRAFT_01904_ABC-type_sugar_transport_system,_periplasmic_component_[Ruminococcus_bromii_YE282]	Ruminococcus_bromii_YE282
Org1_Gene1847	core	1769	2595399816_IE32DRAFT_01905_ABC-type_sugar_transport_systems,_permease_components_[Ruminococcus_bromii_YE282]	Ruminococcus_bromii_YE282
Org1_Gene1848	core	1768	2595399817_IE32DRAFT_01906_ABC-type_sugar_transport_system,_permease_component_[Ruminococcus_bromii_YE282]	Ruminococcus_bromii_YE282
Org1_Gene1849	core	2898	2595399818_IE32DRAFT_01907_TIGR01440_family_protein_[Ruminococcus_bromii_YE282]	Ruminococcus_bromii_YE282
Org1_Gene1852	core	811	2595399821_IE32DRAFT_01910_Uncharacterized_protein_conserved_in_bacteria_[Ruminococcus_bromii_YE282]	Ruminococcus_bromii_YE282
Org1_Gene1853	core	90	2595399822_IE32DRAFT_01911_CoA-substrate-specific_enzyme_activase,_putative_[Ruminococcus_bromii_YE282]	Ruminococcus_bromii_YE282
Org1_Gene1929	core	4378	2595399908_IE32DRAFT_01997_hypothetical_protein_[Ruminococcus_bromii_YE282]	Ruminococcus_bromii_YE282
Org1_Gene1930	core	3981	2595399909_IE32DRAFT_01998_ribosomal_protein_S20_[Ruminococcus_bromii_YE282]	Ruminococcus_bromii_YE282
Org1_Gene1934	core	344	2595399913_IE32DRAFT_02002_chaperone_protein_DnaK_[Ruminococcus_bromii_YE282]	Ruminococcus_bromii_YE282
Org1_Gene1935	core	1153	2595399914_IE32DRAFT_02003_chaperone_protein_DnaJ_[Ruminococcus_bromii_YE282]	Ruminococcus_bromii_YE282
Org1_Gene1936	core	1591	2595399915_IE32DRAFT_02004_ribosomal_protein_L11_methyltransferase_[Ruminococcus_bromii_YE282]	Ruminococcus_bromii_YE282
Org1_Gene1939	core	1958	2595399918_IE32DRAFT_02007_ATPases_involved_in_chromosome_partitioning_[Ruminococcus_bromii_YE282]	Ruminococcus_bromii_YE282
Org1_Gene1941	core	2836	2595399920_IE32DRAFT_02009_Predicted_membrane_protein_[Ruminococcus_bromii_YE282]	Ruminococcus_bromii_YE282
Org1_Gene1956	core	210	2595399935_IE32DRAFT_02024_helicase,_putative,_RecD/TraA_family_[Ruminococcus_bromii_YE282]	Ruminococcus_bromii_YE282
Org1_Gene1958	core	215	2595399937_IE32DRAFT_02026_ribonucleoside-triphosphate_reductase_class_III_catalytic_subunit_(EC_1.17.4.2)_[Ruminococcus_bromii_YE282]	Ruminococcus_bromii_YE282
Org1_Gene1959	core	2925	2595399938_IE32DRAFT_02027_anaerobic_ribonucleoside-triphosphate_reductase_activating_protein_[Ruminococcus_bromii_YE282]	Ruminococcus_bromii_YE282
Org1_Gene1961	core	243	2595399940_IE32DRAFT_02029_Predicted_exporters_of_the_RND_superfamily_[Ruminococcus_bromii_YE282]	Ruminococcus_bromii_YE282
Org1_Gene1964	core	4101	2595399943_IE32DRAFT_02032_acyl_carrier_protein_[Ruminococcus_bromii_YE282]	Ruminococcus_bromii_YE282
Org1_Gene1965	core	1884	2595399944_IE32DRAFT_02033_Shikimate_5-dehydrogenase_[Ruminococcus_bromii_YE282]	Ruminococcus_bromii_YE282
Org1_Gene1967	core	1379	2595399946_IE32DRAFT_02035_3-deoxy-D-arabinoheptulosonate-7-phosphate_synthase_(EC_2.5.1.54)_[Ruminococcus_bromii_YE282]	Ruminococcus_bromii_YE282
Org1_Gene1968	core	1936	2595399947_IE32DRAFT_02036_Prephenate_dehydrogenase_[Ruminococcus_bromii_YE282]	Ruminococcus_bromii_YE282
Org1_Gene1969	core	1306	2595399948_IE32DRAFT_02037_3-dehydroquinate_synthase_(EC_4.2.3.4)_[Ruminococcus_bromii_YE282]	Ruminococcus_bromii_YE282
Org1_Gene1970	core	859	2595399949_IE32DRAFT_02038_3-phosphoshikimate_1-carboxyvinyltransferase_(EC_2.5.1.19)_[Ruminococcus_bromii_YE282]	Ruminococcus_bromii_YE282
Org1_Gene1971	core	1278	2595399950_IE32DRAFT_02039_chorismate_synthase_(EC_4.2.3.5)_[Ruminococcus_bromii_YE282]	Ruminococcus_bromii_YE282
Org1_Gene1972	core	1155	2595399951_IE32DRAFT_02040_Prephenate_dehydratase_[Ruminococcus_bromii_YE282]	Ruminococcus_bromii_YE282
Org1_Gene2	core	3221	2595397922_IE32DRAFT_00003_SsrA-binding_protein_[Ruminococcus_bromii_YE282]	Ruminococcus_bromii_YE282
Org1_Gene2010	core	3035	2595399989_IE32DRAFT_02078_Holliday_junction_endonuclease_RuvC_(EC_3.1.22.4)_[Ruminococcus_bromii_YE282]	Ruminococcus_bromii_YE282
Org1_Gene2011	core	2683	2595399990_IE32DRAFT_02079_Holliday_junction_DNA_helicase_subunit_RuvA_[Ruminococcus_bromii_YE282]	Ruminococcus_bromii_YE282
Org1_Gene2012	core	1247	2595399991_IE32DRAFT_02080_Holliday_junction_DNA_helicase_subunit_RuvB_[Ruminococcus_bromii_YE282]	Ruminococcus_bromii_YE282
Org1_Gene2013	core	761	2595399992_IE32DRAFT_02081_phosphoglucosamine_mutase_(EC_5.4.2.10)_[Ruminococcus_bromii_YE282]	Ruminococcus_bromii_YE282
Org1_Gene2014	core	1821	2595399993_IE32DRAFT_02082_S-adenosylmethionine-dependent_methyltransferase_[Ruminococcus_bromii_YE282]	Ruminococcus_bromii_YE282
Org1_Gene2015	core	1885	2595399994_IE32DRAFT_02083_ABC-type_cobalt_transport_system,_ATPase_component_[Ruminococcus_bromii_YE282]	Ruminococcus_bromii_YE282
Org1_Gene2016	core	1805	2595399995_IE32DRAFT_02084_ABC-type_cobalt_transport_system,_ATPase_component_[Ruminococcus_bromii_YE282]	Ruminococcus_bromii_YE282
Org1_Gene2017	core	1998	2595399996_IE32DRAFT_02085_ABC-type_cobalt_transport_system,_permease_component_CbiQ_and_related_transporters_[Ruminococcus_bromii_YE2	Ruminococcus_bromii_YE282
Org1_Gene2018	core	2028	2595399997_IE32DRAFT_02086_tRNA_pseudouridine(38-40)_synthase_[Ruminococcus_bromii_YE282]	Ruminococcus_bromii_YE282
Org1_Gene2022	core	174	2595400001_IE32DRAFT_02090_ATPases_with_chaperone_activity,_ATP-binding_subunit_[Ruminococcus_bromii_YE282]	Ruminococcus_bromii_YE282
Org1_Gene2067	core	2184	2595400047_IE32DRAFT_02136_phosphate_ABC_transporter,_ATP-binding_protein_[Ruminococcus_bromii_YE282]	Ruminococcus_bromii_YE282
Org1_Gene2072	core	892	2595400052_IE32DRAFT_02141_Predicted_Zn-dependent_peptidases_[Ruminococcus_bromii_YE282]	Ruminococcus_bromii_YE282
Org1_Gene2073	core	1866	2595400053_IE32DRAFT_02142_5,10-methylene-tetrahydrofolate_dehydrogenase/Methenyl_tetrahydrofolate_cyclohydrolase_[Ruminococcus_bromii_YE	Ruminococcus_bromii_YE282
Org1_Gene2074	core	3807	2595400054_IE32DRAFT_02143_ribosomal_protein_L21_[Ruminococcus_bromii_YE282]	Ruminococcus_bromii_YE282
Org1_Gene2076	core	3887	2595400056_IE32DRAFT_02145_ribosomal_protein_L27_[Ruminococcus_bromii_YE282]	Ruminococcus_bromii_YE282
Org1_Gene2077	core	909	2595400057_IE32DRAFT_02146_Obg_family_GTPase_CgtA_[Ruminococcus_bromii_YE282]	Ruminococcus_bromii_YE282
Org1_Gene2078	core	3444	2595400058_IE32DRAFT_02147_Uncharacterized_protein_conserved_in_bacteria_[Ruminococcus_bromii_YE282]	Ruminococcus_bromii_YE282
Org1_Gene2084	core	1964	2595400064_IE32DRAFT_02153_SseB_protein_C-terminal_domain/SseB_protein_N-terminal_domain_[Ruminococcus_bromii_YE282]	Ruminococcus_bromii_YE282
Org1_Gene2089	core	480	2595400069_IE32DRAFT_02158_PASTA_domain/Protein_kinase_domain_[Ruminococcus_bromii_YE282]	Ruminococcus_bromii_YE282
Org1_Gene2091	core	1753	2595400072_IE32DRAFT_02161_ribosome_small_subunit-dependent_GTPase_A_[Ruminococcus_bromii_YE282]	Ruminococcus_bromii_YE282
Org1_Gene2092	core	238	2595400073_IE32DRAFT_02162_Serine/threonine_protein_kinase_[Ruminococcus_bromii_YE282]	Ruminococcus_bromii_YE282
Org1_Gene2093	core	2278	2595400074_IE32DRAFT_02163_Serine/threonine_protein_phosphatase_[Ruminococcus_bromii_YE282]	Ruminococcus_bromii_YE282
Org1_Gene2094	core	1357	2595400075_IE32DRAFT_02164_23S_rRNA_(adenine(2503)-C(2))-methyltransferase_[Ruminococcus_bromii_YE282]	Ruminococcus_bromii_YE282
Org1_Gene2096	core	2364	2595400077_IE32DRAFT_02166_Predicted_Zn-dependent_protease_[Ruminococcus_bromii_YE282]	Ruminococcus_bromii_YE282
Org1_Gene2097	core	1641	2595400078_IE32DRAFT_02167_methionyl-tRNA_formyltransferase_[Ruminococcus_bromii_YE282]	Ruminococcus_bromii_YE282
Org1_Gene2098	core	3150	2595400079_IE32DRAFT_02168_peptide_deformylase_[Ruminococcus_bromii_YE282]	Ruminococcus_bromii_YE282
Org1_Gene2099	core	144	2595400080_IE32DRAFT_02169_replication_restart_DNA_helicase_PriA_[Ruminococcus_bromii_YE282]	Ruminococcus_bromii_YE282
Org1_Gene2100	core	4190	2595400081_IE32DRAFT_02170_DNA-directed_RNA_polymerase,_omega_subunit_[Ruminococcus_bromii_YE282]	Ruminococcus_bromii_YE282
Org1_Gene2102	core	3994	2595400083_IE32DRAFT_02172_Uncharacterized_protein_conserved_in_bacteria_[Ruminococcus_bromii_YE282]	Ruminococcus_bromii_YE282
Org1_Gene2103	core	1747	2595400084_IE32DRAFT_02173_TIGR00255_family_protein_[Ruminococcus_bromii_YE282]	Ruminococcus_bromii_YE282
Org1_Gene2106	core	2379	2595400087_IE32DRAFT_02176_RNA_polymerase,_sigma_29_subunit,_SigE_[Ruminococcus_bromii_YE282]	Ruminococcus_bromii_YE282
Org1_Gene2109	core	1369	2595400094_IE32DRAFT_02183_branched-chain_amino_acid_aminotransferase,_group_II_[Ruminococcus_bromii_YE282]	Ruminococcus_bromii_YE282
Org1_Gene2115	core	4278	2595400100_IE32DRAFT_02189_ribosomal_protein_L28_[Ruminococcus_bromii_YE282]	Ruminococcus_bromii_YE282
Org1_Gene2117	core	2124	2595400102_IE32DRAFT_02191_condensin_subunit_ScpA_[Ruminococcus_bromii_YE282]	Ruminococcus_bromii_YE282
Org1_Gene2118	core	2631	2595400103_IE32DRAFT_02192_segregation_and_condensation_protein_B_[Ruminococcus_bromii_YE282]	Ruminococcus_bromii_YE282
Org1_Gene2120	core	3344	2595400105_IE32DRAFT_02194_sporulation_protein_YtfJ_[Ruminococcus_bromii_YE282]	Ruminococcus_bromii_YE282
Org1_Gene2122	core	2196	2595400107_IE32DRAFT_02196_pseudouridine_synthase_[Ruminococcus_bromii_YE282]	Ruminococcus_bromii_YE282
Org1_Gene2123	core	839	2595400108_IE32DRAFT_02197_Acetyl-CoA_carboxylase,_carboxyltransferase_component_(subunits_alpha_and_beta)_[Ruminococcus_bromii_YE282]	Ruminococcus_bromii_YE282
Org1_Gene2124	core	3572	2595400109_IE32DRAFT_02198_Biotin-requiring_enzyme_[Ruminococcus_bromii_YE282]	Ruminococcus_bromii_YE282
Org1_Gene2125	core	687	2595400110_IE32DRAFT_02199_Pyruvate/oxaloacetate_carboxyltransferase_[Ruminococcus_bromii_YE282]	Ruminococcus_bromii_YE282
Org1_Gene2126	core	2029	2595400111_IE32DRAFT_02200_ABC-type_polar_amino_acid_transport_system,_ATPase_component_[Ruminococcus_bromii_YE282]	Ruminococcus_bromii_YE282
Org1_Gene2127	core	2544	2595400112_IE32DRAFT_02201_ABC-type_amino_acid_transport_system,_permease_component_[Ruminococcus_bromii_YE282]	Ruminococcus_bromii_YE282
Org1_Gene2144	core	3661	2595400129_IE32DRAFT_02218_ribosomal_protein_L17_[Ruminococcus_bromii_YE282]	Ruminococcus_bromii_YE282
Org1_Gene2145	core	1541	2595400130_IE32DRAFT_02219_DNA-directed_RNA_polymerase_subunit_alpha_(EC_2.7.7.6)_[Ruminococcus_bromii_YE282]	Ruminococcus_bromii_YE282
Org1_Gene2146	core	2686	2595400131_IE32DRAFT_02220_ribosomal_protein_S4,_bacterial/organelle_type_[Ruminococcus_bromii_YE282]	Ruminococcus_bromii_YE282
Org1_Gene2147	core	3445	2595400132_IE32DRAFT_02221_30S_ribosomal_protein_S11_[Ruminococcus_bromii_YE282]	Ruminococcus_bromii_YE282
Org1_Gene2148	core	3568	2595400133_IE32DRAFT_02222_SSU_ribosomal_protein_S13P_[Ruminococcus_bromii_YE282]	Ruminococcus_bromii_YE282
Org1_Gene2149	core	4142	2595400134_IE32DRAFT_02223_translation_initiation_factor_IF-1_[Ruminococcus_bromii_YE282]	Ruminococcus_bromii_YE282
Org1_Gene2150	core	4037	2595400135_IE32DRAFT_02224_hypothetical_protein_[Ruminococcus_bromii_YE282]	Ruminococcus_bromii_YE282
Org1_Gene2151	core	2186	2595400136_IE32DRAFT_02225_methionine_aminopeptidase,_type_I_[Ruminococcus_bromii_YE282]	Ruminococcus_bromii_YE282
Org1_Gene2152	core	2593	2595400137_IE32DRAFT_02226_Adenylate_kinase_(EC_2.7.4.3)_[Ruminococcus_bromii_YE282]	Ruminococcus_bromii_YE282
Org1_Gene2153	core	782	2595400138_IE32DRAFT_02227_protein_translocase_subunit_secY/sec61_alpha_[Ruminococcus_bromii_YE282]	Ruminococcus_bromii_YE282
Org1_Gene2154	core	3283	2595400139_IE32DRAFT_02228_LSU_ribosomal_protein_L15P_[Ruminococcus_bromii_YE282]	Ruminococcus_bromii_YE282
Org1_Gene2155	core	4346	2595400140_IE32DRAFT_02229_ribosomal_protein_L30,_bacterial/organelle_[Ruminococcus_bromii_YE282]	Ruminococcus_bromii_YE282
Org1_Gene2156	core	3067	2595400141_IE32DRAFT_02230_ribosomal_protein_S5,_bacterial/organelle_type_[Ruminococcus_bromii_YE282]	Ruminococcus_bromii_YE282
Org1_Gene2157	core	3597	2595400142_IE32DRAFT_02231_ribosomal_protein_L18,_bacterial_type_[Ruminococcus_bromii_YE282]	Ruminococcus_bromii_YE282
Org1_Gene2158	core	2900	2595400143_IE32DRAFT_02232_ribosomal_protein_L6,_bacterial_type_[Ruminococcus_bromii_YE282]	Ruminococcus_bromii_YE282
Org1_Gene2159	core	3469	2595400144_IE32DRAFT_02233_Ribosomal_protein_S8_[Ruminococcus_bromii_YE282]	Ruminococcus_bromii_YE282
Org1_Gene2160	core	4289	2595400145_IE32DRAFT_02234_Ribosomal_protein_S14_[Ruminococcus_bromii_YE282]	Ruminococcus_bromii_YE282
Org1_Gene2161	core	2914	2595400146_IE32DRAFT_02235_Ribosomal_protein_L5_[Ruminococcus_bromii_YE282]	Ruminococcus_bromii_YE282
Org1_Gene2162	core	3773	2595400147_IE32DRAFT_02236_ribosomal_protein_L24,_bacterial/organelle_[Ruminococcus_bromii_YE282]	Ruminococcus_bromii_YE282
Org1_Gene2163	core	3565	2595400148_IE32DRAFT_02237_ribosomal_protein_L14,_bacterial/organelle_[Ruminococcus_bromii_YE282]	Ruminococcus_bromii_YE282
Org1_Gene2164	core	3990	2595400149_IE32DRAFT_02238_30S_ribosomal_protein_S17_[Ruminococcus_bromii_YE282]	Ruminococcus_bromii_YE282
Org1_Gene2165	core	4200	2595400150_IE32DRAFT_02239_ribosomal_protein_L29_[Ruminococcus_bromii_YE282]	Ruminococcus_bromii_YE282
Org1_Gene2166	core	3375	2595400151_IE32DRAFT_02240_ribosomal_protein_L16,_bacterial/organelle_[Ruminococcus_bromii_YE282]	Ruminococcus_bromii_YE282
Org1_Gene2167	core	2426	2595400152_IE32DRAFT_02241_ribosomal_protein_S3,_bacterial_type_[Ruminococcus_bromii_YE282]	Ruminococcus_bromii_YE282
Org1_Gene2168	core	3667	2595400153_IE32DRAFT_02242_ribosomal_protein_L22,_bacterial_type_[Ruminococcus_bromii_YE282]	Ruminococcus_bromii_YE282
Org1_Gene2169	core	3940	2595400154_IE32DRAFT_02243_ribosomal_protein_S19,_bacterial/organelle_[Ruminococcus_bromii_YE282]	Ruminococcus_bromii_YE282
Org1_Gene2170	core	1925	2595400155_IE32DRAFT_02244_ribosomal_protein_L2,_bacterial/organellar_[Ruminococcus_bromii_YE282]	Ruminococcus_bromii_YE282
Org1_Gene2171	core	3858	2595400156_IE32DRAFT_02245_Ribosomal_protein_L23_[Ruminococcus_bromii_YE282]	Ruminococcus_bromii_YE282
Org1_Gene2172	core	2622	2595400157_IE32DRAFT_02246_LSU_ribosomal_protein_L4P_[Ruminococcus_bromii_YE282]	Ruminococcus_bromii_YE282
Org1_Gene2173	core	2594	2595400158_IE32DRAFT_02247_50S_ribosomal_protein_L3,_bacterial_[Ruminococcus_bromii_YE282]	Ruminococcus_bromii_YE282
Org1_Gene2174	core	3796	2595400159_IE32DRAFT_02248_ribosomal_protein_S10,_bacterial/organelle_[Ruminococcus_bromii_YE282]	Ruminococcus_bromii_YE282
Org1_Gene2179	core	1419	2595400164_IE32DRAFT_02253_GGGtGRT_protein_[Ruminococcus_bromii_YE282]	Ruminococcus_bromii_YE282
Org1_Gene2180	core	2401	2595400165_IE32DRAFT_02254_hypothetical_protein_[Ruminococcus_bromii_YE282]	Ruminococcus_bromii_YE282
Org1_Gene2181	core	3136	2595400166_IE32DRAFT_02255_3-isopropylmalate_dehydratase,_small_subunit_[Ruminococcus_bromii_YE282]	Ruminococcus_bromii_YE282
Org1_Gene2182	core	918	2595400167_IE32DRAFT_02256_3-isopropylmalate_dehydratase,_large_subunit_[Ruminococcus_bromii_YE282]	Ruminococcus_bromii_YE282
Org1_Gene2185	core	2359	2595400170_IE32DRAFT_02259_ABC-2_family_transporter_protein_[Ruminococcus_bromii_YE282]	Ruminococcus_bromii_YE282
Org1_Gene2186	core	1527	2595400171_IE32DRAFT_02260_ABC-type_multidrug_transport_system,_ATPase_component_[Ruminococcus_bromii_YE282]	Ruminococcus_bromii_YE282
Org1_Gene2187	core	3621	2595400172_IE32DRAFT_02261_stage_V_sporulation_protein_AE_[Ruminococcus_bromii_YE282]	Ruminococcus_bromii_YE282
Org1_Gene2188	core	50	2595400173_IE32DRAFT_02262_pyruvate:ferredoxin_(flavodoxin)_oxidoreductase,_homodimeric_[Ruminococcus_bromii_YE282]	Ruminococcus_bromii_YE282
Org1_Gene2190	core	1812	2595400175_IE32DRAFT_02264_fructose-1,6-bisphosphate_aldolase,_class_II,_various_bacterial_and_amitochondriate_protist_[Ruminococcus_bromii_YE	Ruminococcus_bromii_YE282
Org1_Gene2193	core	1875	2595400179_IE32DRAFT_02268_HAD-superfamily_hydrolase,_subfamily_IIB_[Ruminococcus_bromii_YE282]	Ruminococcus_bromii_YE282
Org1_Gene2195	core	1890	2595400181_IE32DRAFT_02270_2-polyprenylphenol_hydroxylase_and_related_flavodoxin_oxidoreductases_[Ruminococcus_bromii_YE282]	Ruminococcus_bromii_YE282
Org1_Gene2196	core	700	2595400182_IE32DRAFT_02271_glutamate_synthase_(NADPH),_homotetrameric_[Ruminococcus_bromii_YE282]	Ruminococcus_bromii_YE282
Org1_Gene2200	core	37	2595400186_IE32DRAFT_02275_phosphoribosylformylglycinamidine_synthase_(EC_6.3.5.3)_[Ruminococcus_bromii_YE282]	Ruminococcus_bromii_YE282
Org1_Gene2226	core	1085	2595400213_IE32DRAFT_02303_Malic_enzyme_[Ruminococcus_bromii_YE282]	Ruminococcus_bromii_YE282
Org1_Gene2227	core	517	2595400215_IE32DRAFT_02305_Uncharacterized_FAD-dependent_dehydrogenases_[Ruminococcus_bromii_YE282]	Ruminococcus_bromii_YE282
Org1_Gene2231	core	690	2595400219_IE32DRAFT_02309_putative_efflux_protein,_MATE_family_[Ruminococcus_bromii_YE282]	Ruminococcus_bromii_YE282
Org1_Gene2234	core	346	2595400222_IE32DRAFT_02312_radical_SAM_family_uncharacterized_protein_[Ruminococcus_bromii_YE282]	Ruminococcus_bromii_YE282
Org1_Gene2235	core	2509	2595400223_IE32DRAFT_02313_radical_SAM-linked_protein_[Ruminococcus_bromii_YE282]	Ruminococcus_bromii_YE282
Org1_Gene227	core	3361	2595398171_IE32DRAFT_00252_flavodoxin,_short_chain_[Ruminococcus_bromii_YE282]	Ruminococcus_bromii_YE282
Org1_Gene2271	core	3939	2595400260_IE32DRAFT_02350_sporulation_protein_YabP_[Ruminococcus_bromii_YE282]	Ruminococcus_bromii_YE282

Org1_Gene2274	core	3096	2595400263_IE32DRAFT_02353_Predicted_RNA_binding_protein_(contains_ribosomal_protein_S1_domain)_[Ruminococcus_bromii_YE282]	Ruminococcus_bromii_YE282
Org1_Gene2275	core	2936	2595400264_IE32DRAFT_02354_Ribosome-associated_protein_Y_(PSrp-1)_[Ruminococcus_bromii_YE282]	Ruminococcus_bromii_YE282
Org1_Gene2276	core	1128	2595400265_IE32DRAFT_02355_Predicted_N6-adenine-specific_DNA_methylase_[Ruminococcus_bromii_YE282]	Ruminococcus_bromii_YE282
Org1_Gene2277	core	2241	2595400266_IE32DRAFT_02356_DNA-binding_regulatory_protein,_YebC/PmpR_family_[Ruminococcus_bromii_YE282]	Ruminococcus_bromii_YE282
Org1_Gene2288	core	4243	2595400277_IE32DRAFT_02367_Predicted_transcriptional_regulators_[Ruminococcus_bromii_YE282]	Ruminococcus_bromii_YE282
Org1_Gene230	core	231	2595398174_IE32DRAFT_00255_ATPase,_P-type_(transporting),_HAD_superfamily,_subfamily_IC/heavy_metal_translocating_P-type_ATPase_[Ruminococc	Ruminococcus_bromii_YE282
Org1_Gene2309	core	1408	2595400298_IE32DRAFT_02388_Protein_of_unknown_function_(DUF2804)_[Ruminococcus_bromii_YE282]	Ruminococcus_bromii_YE282
Org1_Gene2311	core	92	2595400300_IE32DRAFT_02390_pullulanase,_type_I_[Ruminococcus_bromii_YE282]	Ruminococcus_bromii_YE282
Org1_Gene2336	core	1602	2595400326_IE32DRAFT_02416_homoserine_O-succinyltransferase_(EC_2.3.1.46)_[Ruminococcus_bromii_YE282]	Ruminococcus_bromii_YE282
Org1_Gene2337	core	644	2595400327_IE32DRAFT_02417_prolyl-tRNA_synthetase_(EC_6.1.1.15)_[Ruminococcus_bromii_YE282]	Ruminococcus_bromii_YE282
Org1_Gene234	core	1659	2595398178_IE32DRAFT_00259_translation_elongation_factor_Ts_[Ruminococcus_bromii_YE282]	Ruminococcus_bromii_YE282
Org1_Gene235	core	2264	2595398179_IE32DRAFT_00260_ribosomal_protein_S2,_bacterial_type_[Ruminococcus_bromii_YE282]	Ruminococcus_bromii_YE282
Org1_Gene2354	core	435	2595400345_IE32DRAFT_02435_Glycosidases_[Ruminococcus_bromii_YE282]	Ruminococcus_bromii_YE282
Org1_Gene2357	core	4031	2595400348_IE32DRAFT_02438_Acyl_carrier_protein_[Ruminococcus_bromii_YE282]	Ruminococcus_bromii_YE282
Org1_Gene2359	core	2246	2595400350_IE32DRAFT_02440_Dehydrogenases_with_different_specificities_(related_to_short-chain_alcohol_dehydrogenases)_[Ruminococcus_bromii_	Ruminococcus_bromii_YE282
Org1_Gene2360	core	3244	2595400351_IE32DRAFT_02441_3-hydroxymyristoyl/3-hydroxydecanoyl-(acyl_carrier_protein)_dehydratases_[Ruminococcus_bromii_YE282]	Ruminococcus_bromii_YE282
Org1_Gene237	core	3854	2595398181_IE32DRAFT_00262_sporulation_protein,_YlmC/YmxH_family_[Ruminococcus_bromii_YE282]	Ruminococcus_bromii_YE282
Org1_Gene2375	core	1105	2595400367_IE32DRAFT_02457_Uncharacterized_flavoproteins_[Ruminococcus_bromii_YE282]	Ruminococcus_bromii_YE282
Org1_Gene238	core	1754	2595398182_IE32DRAFT_00263_EDD_domain_protein,_DegV_family_[Ruminococcus_bromii_YE282]	Ruminococcus_bromii_YE282
Org1_Gene2382	core	2370	2595400374_IE32DRAFT_02464_Predicted_membrane_protein_[Ruminococcus_bromii_YE282]	Ruminococcus_bromii_YE282
Org1_Gene2383	core	4164	2595400375_IE32DRAFT_02465_Fe2+_transport_system_protein_A_[Ruminococcus_bromii_YE282]	Ruminococcus_bromii_YE282
Org1_Gene2384	core	4121	2595400376_IE32DRAFT_02466_Fe2+_transport_system_protein_A_[Ruminococcus_bromii_YE282]	Ruminococcus_bromii_YE282
Org1_Gene2385	core	134	2595400377_IE32DRAFT_02467_Ferrous_iron_transport_protein_B_C_terminus/Ferrous_iron_transport_protein_B/Nucleoside_recognition_[Ruminococcu	Ruminococcus_bromii_YE282
Org1_Gene240	core	1282	2595398184_IE32DRAFT_00265_aspartate_carbamoyltransferase_(EC_2.1.3.2)_[Ruminococcus_bromii_YE282]	Ruminococcus_bromii_YE282
Org1_Gene241	core	224	2595398185_IE32DRAFT_00266_Transcriptional_accessory_protein_[Ruminococcus_bromii_YE282]	Ruminococcus_bromii_YE282
Org1_Gene2428	core	3322	2595400420_IE32DRAFT_02510_Uncharacterized_conserved_protein,_contains_double-stranded_beta-helix_domain_[Ruminococcus_bromii_YE282]	Ruminococcus_bromii_YE282
Org1_Gene2429	core	3740	2595400421_IE32DRAFT_02511_Uncharacterized_homolog_of_gamma-carboxymuconolactone_decarboxylase_subunit_[Ruminococcus_bromii_YE282]	Ruminococcus_bromii_YE282
Org1_Gene2432	core	317	2595400425_IE32DRAFT_02515_ATPase_components_of_ABC_transporters_with_duplicated_ATPase_domains_[Ruminococcus_bromii_YE282]	Ruminococcus_bromii_YE282
Org1_Gene2441	core	817	2595400440_IE32DRAFT_02531_UDP-N-acetylmuramyl_tripeptide_synthase_[Ruminococcus_bromii_YE282]	Ruminococcus_bromii_YE282
Org1_Gene2442	core	1118	2595400441_IE32DRAFT_02532_Site-specific_recombinases,_DNA_invertase_Pin_homologs_[Ruminococcus_bromii_YE282]	Ruminococcus_bromii_YE282
Org1_Gene2458	core	4026	2595400457_IE32DRAFT_02548_hypothetical_protein_[Ruminococcus_bromii_YE282]	Ruminococcus_bromii_YE282
Org1_Gene2466	core	852	2595400465_IE32DRAFT_02556_OAH/OAS_sulfhydrylase_[Ruminococcus_bromii_YE282]	Ruminococcus_bromii_YE282
Org1_Gene248	core	3557	2595398192_IE32DRAFT_00273_hypothetical_protein_[Ruminococcus_bromii_YE282]	Ruminococcus_bromii_YE282
Org1_Gene2482	core	2075	2595400481_IE32DRAFT_02572_Metal-dependent_hydrolases_of_the_beta-lactamase_superfamily_I_[Ruminococcus_bromii_YE282]	Ruminococcus_bromii_YE282
Org1_Gene2483	core	3115	2595400482_IE32DRAFT_02573_Uncharacterized_conserved_protein_[Ruminococcus_bromii_YE282]	Ruminococcus_bromii_YE282
Org1_Gene249	core	1216	2595398193_IE32DRAFT_00274_RNA_polymerase_sigma_factor,_sigma-70_family_[Ruminococcus_bromii_YE282]	Ruminococcus_bromii_YE282
Org1_Gene25	core	2038	2595397948_IE32DRAFT_00029_pyrroline-5-carboxylate_reductase_[Ruminococcus_bromii_YE282]	Ruminococcus_bromii_YE282
Org1_Gene250	core	383	2595398194_IE32DRAFT_00275_DNA_primase,_catalytic_core_[Ruminococcus_bromii_YE282]	Ruminococcus_bromii_YE282
Org1_Gene251	core	2603	2595398195_IE32DRAFT_00276_uncharacterized_protein,_YigZ_family_[Ruminococcus_bromii_YE282]	Ruminococcus_bromii_YE282
Org1_Gene252	core	4201	2595398196_IE32DRAFT_00277_ribosomal_protein_L31_[Ruminococcus_bromii_YE282]	Ruminococcus_bromii_YE282
Org1_Gene254	core	298	2595398198_IE32DRAFT_00279_threonyl-tRNA_synthetase_(EC_6.1.1.3)_[Ruminococcus_bromii_YE282]	Ruminococcus_bromii_YE282
Org1_Gene259	core	2611	2595398203_IE32DRAFT_00284_Mn-containing_catalase_[Ruminococcus_bromii_YE282]	Ruminococcus_bromii_YE282
Org1_Gene262	core	4002	2595398206_IE32DRAFT_00287_Helix-turn-helix_domain_[Ruminococcus_bromii_YE282]	Ruminococcus_bromii_YE282
Org1_Gene263	core	3252	2595398207_IE32DRAFT_00288_ribose-5-phosphate_isomerase_(EC_5.3.1.6)_[Ruminococcus_bromii_YE282]	Ruminococcus_bromii_YE282
Org1_Gene265	core	3357	2595398209_IE32DRAFT_00290_tRNA_threonylcarbamoyl_adenosine_modification_protein_YjeE_[Ruminococcus_bromii_YE282]	Ruminococcus_bromii_YE282
Org1_Gene266	core	456	2595398210_IE32DRAFT_00291_Formate-tetrahydrofolate_ligase_(EC_6.3.4.3)_[Ruminococcus_bromii_YE282]	Ruminococcus_bromii_YE282
Org1_Gene269	core	1734	2595398213_IE32DRAFT_00294_TIGR00159_family_protein_[Ruminococcus_bromii_YE282]	Ruminococcus_bromii_YE282
Org1_Gene274	core	2158	2595398218_IE32DRAFT_00299_dihydrodipicolinate_reductase_(EC_1.3.1.26)_[Ruminococcus_bromii_YE282]	Ruminococcus_bromii_YE282
Org1_Gene275	core	1686	2595398219_IE32DRAFT_00300_4-hydroxy-tetrahydrodipicolinate_synthase_[Ruminococcus_bromii_YE282]	Ruminococcus_bromii_YE282
Org1_Gene276	core	1256	2595398220_IE32DRAFT_00301_aspartate-semialdehyde_dehydrogenase_(non-peptidoglycan_organisms)_[Ruminococcus_bromii_YE282]	Ruminococcus_bromii_YE282
Org1_Gene277	core	1375	2595398221_IE32DRAFT_00302_S-adenosylmethionine:tRNA_ribosyltransferase-isomerase_[Ruminococcus_bromii_YE282]	Ruminococcus_bromii_YE282
Org1_Gene278	core	1179	2595398222_IE32DRAFT_00303_tRNA-guanine_transglycosylase_[Ruminococcus_bromii_YE282]	Ruminococcus_bromii_YE282
Org1_Gene286	core	359	2595398230_IE32DRAFT_00311_GTP-binding_protein_TypA/BipA_[Ruminococcus_bromii_YE282]	Ruminococcus_bromii_YE282
Org1_Gene287	core	307	2595398231_IE32DRAFT_00312_aconitase_(EC_4.2.1.3)_[Ruminococcus_bromii_YE282]	Ruminococcus_bromii_YE282
Org1_Gene288	core	1011	2595398232_IE32DRAFT_00313_isocitrate_dehydrogenase_(NADP)_(EC_1.1.1.42)_[Ruminococcus_bromii_YE282]	Ruminococcus_bromii_YE282
Org1_Gene289	core	2589	2595398233_IE32DRAFT_00314_AT-rich_DNA-binding_protein_[Ruminococcus_bromii_YE282]	Ruminococcus_bromii_YE282
Org1_Gene290	core	857	2595398234_IE32DRAFT_00315_diaminopimelate_decarboxylase_[Ruminococcus_bromii_YE282]	Ruminococcus_bromii_YE282
Org1_Gene291	core	2688	2595398235_IE32DRAFT_00316_ribosome_biogenesis_GTP-binding_protein_YsxC/EngB_[Ruminococcus_bromii_YE282]	Ruminococcus_bromii_YE282
Org1_Gene292	core	156	2595398236_IE32DRAFT_00317_endopeptidase_La_[Ruminococcus_bromii_YE282]	Ruminococcus_bromii_YE282
Org1_Gene293	core	781	2595398237_IE32DRAFT_00318_endopeptidase_Clp_ATP-binding_regulatory_subunit_(clpX)_[Ruminococcus_bromii_YE282]	Ruminococcus_bromii_YE282
Org1_Gene294	core	2763	2595398238_IE32DRAFT_00319_ATP-dependent_Clp_endopeptidase,_proteolytic_subunit_ClpP_[Ruminococcus_bromii_YE282]	Ruminococcus_bromii_YE282
Org1_Gene295	core	890	2595398239_IE32DRAFT_00320_trigger_factor_[Ruminococcus_bromii_YE282]	Ruminococcus_bromii_YE282
Org1_Gene296	core	2412	2595398240_IE32DRAFT_00321_Predicted_phosphohydrolase_[Ruminococcus_bromii_YE282]	Ruminococcus_bromii_YE282
Org1_Gene297	core	574	2595398241_IE32DRAFT_00322_threonine_synthase_[Ruminococcus_bromii_YE282]	Ruminococcus_bromii_YE282
Org1_Gene3	core	2679	2595397923_IE32DRAFT_00004_DNA_replication_and_repair_protein_RecR_[Ruminococcus_bromii_YE282]	Ruminococcus_bromii_YE282
Org1_Gene300	core	2656	2595398244_IE32DRAFT_00325_RNase_HII_(EC_3.1.26.4)_[Ruminococcus_bromii_YE282]	Ruminococcus_bromii_YE282
Org1_Gene301	core	1771	2595398245_IE32DRAFT_00326_ribosome_biogenesis_GTP-binding_protein_YlqF_[Ruminococcus_bromii_YE282]	Ruminococcus_bromii_YE282
Org1_Gene303	core	3645	2595398247_IE32DRAFT_00328_ribosomal_protein_L19,_bacterial_type_[Ruminococcus_bromii_YE282]	Ruminococcus_bromii_YE282
Org1_Gene304	core	1638	2595398248_IE32DRAFT_00329_pseudouridine_synthase,_RluA_family_[Ruminococcus_bromii_YE282]	Ruminococcus_bromii_YE282
Org1_Gene305	core	3126	2595398249_IE32DRAFT_00330_lipoprotein_signal_peptidase_[Ruminococcus_bromii_YE282]	Ruminococcus_bromii_YE282
Org1_Gene306	core	102	2595398250_IE32DRAFT_00331_Isoleucyl-tRNA_synthetase_(EC_6.1.1.5)_[Ruminococcus_bromii_YE282]	Ruminococcus_bromii_YE282
Org1_Gene309	core	3192	2595398253_IE32DRAFT_00334_Uncharacterized_protein_conserved_in_bacteria_[Ruminococcus_bromii_YE282]	Ruminococcus_bromii_YE282
Org1_Gene310	core	2317	2595398254_IE32DRAFT_00335_pyridoxal_phosphate_enzyme,_YggS_family_[Ruminococcus_bromii_YE282]	Ruminococcus_bromii_YE282
Org1_Gene314	core	127	2595398258_IE32DRAFT_00339_DNA_mismatch_repair_protein_MutS_[Ruminococcus_bromii_YE282]	Ruminococcus_bromii_YE282
Org1_Gene315	core	3428	2595398259_IE32DRAFT_00340_Uncharacterized_conserved_protein_[Ruminococcus_bromii_YE282]	Ruminococcus_bromii_YE282
Org1_Gene316	core	673	2595398260_IE32DRAFT_00341_tRNA-N(6)-(isopentenyl)adenosine-37_thiotransferase_enzyme_MiaB_[Ruminococcus_bromii_YE282]	Ruminococcus_bromii_YE282
Org1_Gene319	core	2854	2595398263_IE32DRAFT_00344_translation_elongation_factor_P_[Ruminococcus_bromii_YE282]	Ruminococcus_bromii_YE282
Org1_Gene321	core	3327	2595398265_IE32DRAFT_00346_3-dehydroquinate_dehydratase_(EC_4.2.1.10)_[Ruminococcus_bromii_YE282]	Ruminococcus_bromii_YE282
Org1_Gene322	core	4323	2595398266_IE32DRAFT_00347_ribosomal_protein_S21_[Ruminococcus_bromii_YE282]	Ruminococcus_bromii_YE282
Org1_Gene323	core	155	2595398267_IE32DRAFT_00348_leucyl-tRNA_synthetase_(EC_6.1.1.4)_[Ruminococcus_bromii_YE282]	Ruminococcus_bromii_YE282
Org1_Gene324	core	3626	2595398268_IE32DRAFT_00349_ribosome_silencing_factor_RsfS/YbeB/iojap_[Ruminococcus_bromii_YE282]	Ruminococcus_bromii_YE282
Org1_Gene327	core	3821	2595398271_IE32DRAFT_00352_Predicted_RNA-binding_protein_containing_KH_domain,_possibly_ribosomal_protein_[Ruminococcus_bromii_YE282]	Ruminococcus_bromii_YE282
Org1_Gene328	core	2674	2595398272_IE32DRAFT_00353_non-canonical_purine_NTP_pyrophosphatase,_RdgB/HAM1_family_[Ruminococcus_bromii_YE282]	Ruminococcus_bromii_YE282
Org1_Gene329	core	1644	2595398273_IE32DRAFT_00354_signal_recognition_particle-docking_protein_FtsY_[Ruminococcus_bromii_YE282]	Ruminococcus_bromii_YE282
Org1_Gene330	core	47	2595398274_IE32DRAFT_00355_condensin_subunit_Smc_[Ruminococcus_bromii_YE282]	Ruminococcus_bromii_YE282
Org1_Gene332	core	2444	2595398276_IE32DRAFT_00357_ribonuclease_III,_bacterial_[Ruminococcus_bromii_YE282]	Ruminococcus_bromii_YE282
Org1_Gene333	core	1426	2595398277_IE32DRAFT_00358_phosphate:acyl-[acyl_carrier_protein]_acyltransferase_[Ruminococcus_bromii_YE282]	Ruminococcus_bromii_YE282
Org1_Gene334	core	828	2595398278_IE32DRAFT_00359_tRNA:m(5)U-54_methyltransferase_[Ruminococcus_bromii_YE282]	Ruminococcus_bromii_YE282
Org1_Gene335	core	249	2595398279_IE32DRAFT_00360_DNA_topoisomerase_I,_bacterial_[Ruminococcus_bromii_YE282]	Ruminococcus_bromii_YE282
Org1_Gene338	core	3634	2595398282_IE32DRAFT_00363_ribosomal_protein_L20_[Ruminococcus_bromii_YE282]	Ruminococcus_bromii_YE282
Org1_Gene339	core	4231	2595398283_IE32DRAFT_00364_ribosomal_protein_L35_[Ruminococcus_bromii_YE282]	Ruminococcus_bromii_YE282
Org1_Gene348	core	323	2595398293_IE32DRAFT_00374_ABC-type_multidrug_transport_system,_ATPase_and_permease_components_[Ruminococcus_bromii_YE282]	Ruminococcus_bromii_YE282
Org1_Gene349	core	398	2595398294_IE32DRAFT_00375_ABC-type_multidrug_transport_system,_ATPase_and_permease_components_[Ruminococcus_bromii_YE282]	Ruminococcus_bromii_YE282
Org1_Gene35	core	2815	2595397958_IE32DRAFT_00039_hypothetical_protein_[Ruminococcus_bromii_YE282]	Ruminococcus_bromii_YE282
Org1_Gene353	core	2145	2595398298_IE32DRAFT_00379_Short-chain_dehydrogenases_of_various_substrate_specificities_[Ruminococcus_bromii_YE282]	Ruminococcus_bromii_YE282
Org1_Gene355	core	729	2595398300_IE32DRAFT_00381_23S_rRNA_(uracil-5-)-methyltransferase_RumA_[Ruminococcus_bromii_YE282]	Ruminococcus_bromii_YE282
Org1_Gene356	core	710	2595398301_IE32DRAFT_00382_UDP-N-acetylmuramate--L-alanine_ligase_(EC_6.3.2.8)_[Ruminococcus_bromii_YE282]	Ruminococcus_bromii_YE282
Org1_Gene361	core	3947	2595398306_IE32DRAFT_00387_ACT_domain-containing_protein_[Ruminococcus_bromii_YE282]	Ruminococcus_bromii_YE282
Org1_Gene362	core	744	2595398307_IE32DRAFT_00388_Uncharacterized_conserved_protein_[Ruminococcus_bromii_YE282]	Ruminococcus_bromii_YE282
Org1_Gene364	core	1355	2595398309_IE32DRAFT_00390_Archaeal/vacuolar-type_H+-ATPase_subunit_C_[Ruminococcus_bromii_YE282]	Ruminococcus_bromii_YE282
Org1_Gene366	core	3337	2595398311_IE32DRAFT_00392_F0F1-type_ATP_synthase,_subunit_c/Archaeal/vacuolar-type_H+-ATPase,_subunit_K_[Ruminococcus_bromii_YE282]	Ruminococcus_bromii_YE282
Org1_Gene367	core	3802	2595398312_IE32DRAFT_00393_Archaeal/vacuolar-type_H+-ATPase_subunit_F_[Ruminococcus_bromii_YE282]	Ruminococcus_bromii_YE282
Org1_Gene369	core	393	2595398314_IE32DRAFT_00395_Archaeal/vacuolar-type_H+-ATPase_subunit_A_[Ruminococcus_bromii_YE282]	Ruminococcus_bromii_YE282
Org1_Gene37	core	371	2595397960_IE32DRAFT_00041_dihydroxy-acid_dehydratase_[Ruminococcus_bromii_YE282]	Ruminococcus_bromii_YE282
Org1_Gene370	core	716	2595398315_IE32DRAFT_00396_Archaeal/vacuolar-type_H+-ATPase_subunit_B_[Ruminococcus_bromii_YE282]	Ruminococcus_bromii_YE282
Org1_Gene371	core	2499	2595398316_IE32DRAFT_00397_H(+)-transporting_ATP_synthase,_vacuolar_type,_subunit_D_[Ruminococcus_bromii_YE282]	Ruminococcus_bromii_YE282
Org1_Gene38	core	258	2595397961_IE32DRAFT_00042_translation_elongation_factor_2_(EF-2/EF-G)_[Ruminococcus_bromii_YE282]	Ruminococcus_bromii_YE282
Org1_Gene4	core	3632	2595397924_IE32DRAFT_00005_DNA-binding_protein,_YbaB/EbfC_family_[Ruminococcus_bromii_YE282]	Ruminococcus_bromii_YE282
Org1_Gene401	core	891	2595398346_IE32DRAFT_00427_phage_terminase,_large_subunit,_PBSX_family_[Ruminococcus_bromii_YE282]	Ruminococcus_bromii_YE282
Org1_Gene405	core	1409	2595398350_IE32DRAFT_00431_phage_major_capsid_protein,_HK97_family_[Ruminococcus_bromii_YE282]	Ruminococcus_bromii_YE282
Org1_Gene42	core	396	2595397965_IE32DRAFT_00046_ABC-type_multidrug_transport_system,_ATPase_and_permease_components_[Ruminococcus_bromii_YE282]	Ruminococcus_bromii_YE282
Org1_Gene420	core	2018	2595398366_IE32DRAFT_00447_thymidylate_synthase,_flavin-dependent_[Ruminococcus_bromii_YE282]	Ruminococcus_bromii_YE282
Org1_Gene422	core	2911	2595398368_IE32DRAFT_00449_DJ-1_family_protein_[Ruminococcus_bromii_YE282]	Ruminococcus_bromii_YE282
Org1_Gene425	core	971	2595398371_IE32DRAFT_00452_Collagenase_and_related_proteases_[Ruminococcus_bromii_YE282]	Ruminococcus_bromii_YE282
Org1_Gene429	core	416	2595398375_IE32DRAFT_00456_4-amino-4-deoxy-L-arabinose_transferase_and_related_glycosyltransferases_of_PMT_family_[Ruminococcus_bromii_YE2	Ruminococcus_bromii_YE282
Org1_Gene43	core	405	2595397966_IE32DRAFT_00047_ABC-type_multidrug_transport_system,_ATPase_and_permease_components_[Ruminococcus_bromii_YE282]	Ruminococcus_bromii_YE282
Org1_Gene430	core	1671	2595398376_IE32DRAFT_00457_Patatin-like_phospholipase_[Ruminococcus_bromii_YE282]	Ruminococcus_bromii_YE282
Org1_Gene433	core	56	2595398379_IE32DRAFT_00460_transcription-repair_coupling_factor_[Ruminococcus_bromii_YE282]	Ruminococcus_bromii_YE282
Org1_Gene434	core	2720	2595398380_IE32DRAFT_00461_aminoacyl-tRNA_hydrolase_[Ruminococcus_bromii_YE282]	Ruminococcus_bromii_YE282
Org1_Gene438	core	1519	2595398384_IE32DRAFT_00465_ribose-phosphate_pyrophosphokinase_[Ruminococcus_bromii_YE282]	Ruminococcus_bromii_YE282
Org1_Gene439	core	2182	2595398385_IE32DRAFT_00466_Nucleotidyl_transferase_[Ruminococcus_bromii_YE282]	Ruminococcus_bromii_YE282
Org1_Gene440	core	120	2595398386_IE32DRAFT_00467_pyruvate_phosphate_dikinase_(EC_2.7.9.1)_[Ruminococcus_bromii_YE282]	Ruminococcus_bromii_YE282

Org1_Gene442	core	4115	2595398388_IE32DRAFT_00469_Uncharacterized_protein_conserved_in_bacteria_[Ruminococcus_bromii_YE282]	Ruminococcus_bromii_YE282
Org1_Gene443	core	3702	2595398389_IE32DRAFT_00470_Predicted_transcriptional_regulators_[Ruminococcus_bromii_YE282]	Ruminococcus_bromii_YE282
Org1_Gene446	core	494	2595398392_IE32DRAFT_00473_acetolactate_synthase,_large_subunit_(EC_2.2.1.6)_[Ruminococcus_bromii_YE282]	Ruminococcus_bromii_YE282
Org1_Gene447	core	3087	2595398393_IE32DRAFT_00474_acetolactate_synthase,_small_subunit_(EC_2.2.1.6)_[Ruminococcus_bromii_YE282]	Ruminococcus_bromii_YE282
Org1_Gene448	core	1341	2595398394_IE32DRAFT_00475_ketol-acid_reductoisomerase_(EC_1.1.1.86)_[Ruminococcus_bromii_YE282]	Ruminococcus_bromii_YE282
Org1_Gene449	core	3504	2595398395_IE32DRAFT_00476_nitrogen_regulatory_protein_P-II_family_[Ruminococcus_bromii_YE282]	Ruminococcus_bromii_YE282
Org1_Gene450	core	938	2595398396_IE32DRAFT_00477_ammonium_transporter_(TC_1.A.11)_[Ruminococcus_bromii_YE282]	Ruminococcus_bromii_YE282
Org1_Gene451	core	1074	2595398397_IE32DRAFT_00478_alanine_racemase_[Ruminococcus_bromii_YE282]	Ruminococcus_bromii_YE282
Org1_Gene452	core	129	2595398398_IE32DRAFT_00479_DNA_polymerase_I_(EC_2.7.7.7)_[Ruminococcus_bromii_YE282]	Ruminococcus_bromii_YE282
Org1_Gene455	core	1404	2595398401_IE32DRAFT_00482_tRNA_threonylcarbamoyl_adenosine_modification_protein_TsaD_[Ruminococcus_bromii_YE282]	Ruminococcus_bromii_YE282
Org1_Gene457	core	2990	2595398403_IE32DRAFT_00484_Peptidyl-prolyl_cis-trans_isomerase_(rotamase)_-_cyclophilin_family_[Ruminococcus_bromii_YE282]	Ruminococcus_bromii_YE282
Org1_Gene458	core	1397	2595398404_IE32DRAFT_00485_UDP-glucose-4-epimerase_GalE_[Ruminococcus_bromii_YE282]	Ruminococcus_bromii_YE282
Org1_Gene46	core	570	2595397969_IE32DRAFT_00050_phosphoglycerate_mutase_(EC_5.4.2.1)_[Ruminococcus_bromii_YE282]	Ruminococcus_bromii_YE282
Org1_Gene463	core	3447	2595398410_IE32DRAFT_00491_Ribosomal_protein_S9_[Ruminococcus_bromii_YE282]	Ruminococcus_bromii_YE282
Org1_Gene464	core	3339	2595398411_IE32DRAFT_00492_LSU_ribosomal_protein_L13P_[Ruminococcus_bromii_YE282]	Ruminococcus_bromii_YE282
Org1_Gene467	core	525	2595398414_IE32DRAFT_00495_Signal_transduction_histidine_kinase_[Ruminococcus_bromii_YE282]	Ruminococcus_bromii_YE282
Org1_Gene468	core	2424	2595398415_IE32DRAFT_00496_Response_regulators_consisting_of_a_CheY-like_receiver_domain_and_a_winged-helix_DNA-binding_domain_[Ruminoco	Ruminococcus_bromii_YE282
Org1_Gene469	core	1863	2595398416_IE32DRAFT_00497_hydro-lyases,_Fe-S_type,_tartrate/fumarate_subfamily,_alpha_region_[Ruminococcus_bromii_YE282]	Ruminococcus_bromii_YE282
Org1_Gene47	core	2143	2595397970_IE32DRAFT_00051_triosephosphate_isomerase_(EC_5.3.1.1)_[Ruminococcus_bromii_YE282]	Ruminococcus_bromii_YE282
Org1_Gene470	core	2878	2595398417_IE32DRAFT_00498_hydro-lyases,_Fe-S_type,_tartrate/fumarate_subfamily,_beta_region_[Ruminococcus_bromii_YE282]	Ruminococcus_bromii_YE282
Org1_Gene473	core	885	2595398420_IE32DRAFT_00501_seryl-tRNA_synthetase_(EC_6.1.1.11)_[Ruminococcus_bromii_YE282]	Ruminococcus_bromii_YE282
Org1_Gene474	core	1877	2595398421_IE32DRAFT_00502_ParB/RepB/Spo0J_family_partition_protein_[Ruminococcus_bromii_YE282]	Ruminococcus_bromii_YE282
Org1_Gene475	core	2141	2595398422_IE32DRAFT_00503_chromosome_segregation_ATPase_[Ruminococcus_bromii_YE282]	Ruminococcus_bromii_YE282
Org1_Gene476	core	1848	2595398423_IE32DRAFT_00504_ParB/RepB/Spo0J_family_partition_protein_[Ruminococcus_bromii_YE282]	Ruminococcus_bromii_YE282
Org1_Gene478	core	332	2595398425_IE32DRAFT_00506_glucose-inhibited_division_protein_A_[Ruminococcus_bromii_YE282]	Ruminococcus_bromii_YE282
Org1_Gene479	core	715	2595398426_IE32DRAFT_00507_tRNA_modification_GTPase_TrmE_[Ruminococcus_bromii_YE282]	Ruminococcus_bromii_YE282
Org1_Gene48	core	1009	2595397971_IE32DRAFT_00052_phosphoglycerate_kinase_(EC_2.7.2.3)_[Ruminococcus_bromii_YE282]	Ruminococcus_bromii_YE282
Org1_Gene480	core	2011	2595398427_IE32DRAFT_00508_Predicted_RNA-binding_protein_[Ruminococcus_bromii_YE282]	Ruminococcus_bromii_YE282
Org1_Gene482	core	4063	2595398429_IE32DRAFT_00510_putative_membrane_protein_insertion_efficiency_factor_[Ruminococcus_bromii_YE282]	Ruminococcus_bromii_YE282
Org1_Gene483	core	3616	2595398430_IE32DRAFT_00511_ribonuclease_P_protein_component,_eubacterial_[Ruminococcus_bromii_YE282]	Ruminococcus_bromii_YE282
Org1_Gene484	core	4411	2595398431_IE32DRAFT_00512_ribosomal_protein_L34,_bacterial_type_[Ruminococcus_bromii_YE282]	Ruminococcus_bromii_YE282
Org1_Gene485	core	600	2595398432_IE32DRAFT_00513_inosine-5'-monophosphate_dehydrogenase_[Ruminococcus_bromii_YE282]	Ruminococcus_bromii_YE282
Org1_Gene486	core	1533	2595398433_IE32DRAFT_00514_Transcriptional_regulator/sugar_kinase_[Ruminococcus_bromii_YE282]	Ruminococcus_bromii_YE282
Org1_Gene49	core	2998	2595397972_IE32DRAFT_00053_tRNA_(cytidine(34)-2'-O)-methyltransferase_[Ruminococcus_bromii_YE282]	Ruminococcus_bromii_YE282
Org1_Gene493	core	553	2595398440_IE32DRAFT_00521_Glycosidases_[Ruminococcus_bromii_YE282]	Ruminococcus_bromii_YE282
Org1_Gene496	core	1383	2595398443_IE32DRAFT_00524_tryptophanyl-tRNA_synthetase_(EC_6.1.1.2)_[Ruminococcus_bromii_YE282]	Ruminococcus_bromii_YE282
Org1_Gene497	core	1056	2595398444_IE32DRAFT_00525_Transcriptional_regulators_containing_a_DNA-binding_HTH_domain_and_an_aminotransferase_domain_(MocR_family)_	Ruminococcus_bromii_YE282
Org1_Gene499	core	2391	2595398446_IE32DRAFT_00527_ADP-ribose_pyrophosphatase_[Ruminococcus_bromii_YE282]	Ruminococcus_bromii_YE282
Org1_Gene501	core	453	2595398448_IE32DRAFT_00529_acetolactate_synthase,_large_subunit_(EC_2.2.1.6)_[Ruminococcus_bromii_YE282]	Ruminococcus_bromii_YE282
Org1_Gene502	core	2393	2595398449_IE32DRAFT_00530_5'-methylthioadenosine/S-adenosylhomocysteine_nucleosidase_[Ruminococcus_bromii_YE282]	Ruminococcus_bromii_YE282
Org1_Gene503	core	2255	2595398450_IE32DRAFT_00531_RNA_methyltransferase,_RsmE_family_[Ruminococcus_bromii_YE282]	Ruminococcus_bromii_YE282
Org1_Gene504	core	2857	2595398451_IE32DRAFT_00532_amidohydrolase,_PncC_family_[Ruminococcus_bromii_YE282]	Ruminococcus_bromii_YE282
Org1_Gene505	core	590	2595398452_IE32DRAFT_00533_4-alpha-glucanotransferase_[Ruminococcus_bromii_YE282]	Ruminococcus_bromii_YE282
Org1_Gene506	core	171	2595398453_IE32DRAFT_00534_glycogen/starch/alpha-glucan_phosphorylases_[Ruminococcus_bromii_YE282]	Ruminococcus_bromii_YE282
Org1_Gene507	core	363	2595398454_IE32DRAFT_00535_elongation_factor_4_[Ruminococcus_bromii_YE282]	Ruminococcus_bromii_YE282
Org1_Gene509	core	2188	2595398456_IE32DRAFT_00537_Dinucleotide-utilizing_enzymes_involved_in_molybdopterin_and_thiamine_biosynthesis_family_1_[Ruminococcus_bromi	Ruminococcus_bromii_YE282
Org1_Gene511	core	1484	2595398458_IE32DRAFT_00539_Glycosyltransferases_involved_in_cell_wall_biogenesis_[Ruminococcus_bromii_YE282]	Ruminococcus_bromii_YE282
Org1_Gene513	core	697	2595398460_IE32DRAFT_00541_chromosomal_replication_initiator_protein_DnaA_[Ruminococcus_bromii_YE282]	Ruminococcus_bromii_YE282
Org1_Gene514	core	1224	2595398461_IE32DRAFT_00542_DNA_polymerase_III,_beta_subunit_[Ruminococcus_bromii_YE282]	Ruminococcus_bromii_YE282
Org1_Gene515	core	4166	2595398462_IE32DRAFT_00543_Uncharacterized_conserved_protein_[Ruminococcus_bromii_YE282]	Ruminococcus_bromii_YE282
Org1_Gene516	core	1180	2595398463_IE32DRAFT_00544_DNA_replication_and_repair_protein_RecF_[Ruminococcus_bromii_YE282]	Ruminococcus_bromii_YE282
Org1_Gene517	core	3932	2595398464_IE32DRAFT_00545_hypothetical_protein_[Ruminococcus_bromii_YE282]	Ruminococcus_bromii_YE282
Org1_Gene520	core	256	2595398467_IE32DRAFT_00548_DNA_gyrase,_B_subunit_[Ruminococcus_bromii_YE282]	Ruminococcus_bromii_YE282
Org1_Gene522	core	128	2595398469_IE32DRAFT_00550_DNA_gyrase,_A_subunit_[Ruminococcus_bromii_YE282]	Ruminococcus_bromii_YE282
Org1_Gene525	core	1289	2595398475_IE32DRAFT_00556_D-alanine--D-alanine_ligase_[Ruminococcus_bromii_YE282]	Ruminococcus_bromii_YE282
Org1_Gene526	core	758	2595398476_IE32DRAFT_00557_UDP-N-acetylmuramoyl-tripeptide--D-alanyl-D-alanine_ligase_(EC_6.3.2.10)_[Ruminococcus_bromii_YE282]	Ruminococcus_bromii_YE282
Org1_Gene527	core	2595	2595398477_IE32DRAFT_00558_uracil_phosphoribosyltransferase_(EC_2.4.2.9)_[Ruminococcus_bromii_YE282]	Ruminococcus_bromii_YE282
Org1_Gene528	core	2658	2595398479_IE32DRAFT_00560_Uncharacterized_protein_conserved_in_bacteria_[Ruminococcus_bromii_YE282]	Ruminococcus_bromii_YE282
Org1_Gene529	core	1117	2595398480_IE32DRAFT_00561_NusA_antitermination_factor_[Ruminococcus_bromii_YE282]	Ruminococcus_bromii_YE282
Org1_Gene532	core	165	2595398483_IE32DRAFT_00564_translation_initiation_factor_IF-2_[Ruminococcus_bromii_YE282]	Ruminococcus_bromii_YE282
Org1_Gene533	core	3541	2595398484_IE32DRAFT_00565_ribosome-binding_factor_A_[Ruminococcus_bromii_YE282]	Ruminococcus_bromii_YE282
Org1_Gene536	core	1554	2595398487_IE32DRAFT_00568_Transketolase,_C-terminal_subunit_[Ruminococcus_bromii_YE282]	Ruminococcus_bromii_YE282
Org1_Gene537	core	1872	2595398488_IE32DRAFT_00569_Transketolase,_N-terminal_subunit_[Ruminococcus_bromii_YE282]	Ruminococcus_bromii_YE282
Org1_Gene538	core	2140	2595398489_IE32DRAFT_00570_pantothenate_kinase,_type_III_[Ruminococcus_bromii_YE282]	Ruminococcus_bromii_YE282
Org1_Gene54	core	3239	2595397977_IE32DRAFT_00058_S-ribosylhomocysteine_lyase_(EC_4.4.1.21)/quorum-sensing_autoinducer_2_(AI-2)_synthesis_protein_LuxS_[Ruminococc	Ruminococcus_bromii_YE282
Org1_Gene542	core	2506	2595398493_IE32DRAFT_00574_haloacid_dehalogenase_superfamily,_subfamily_IA,_variant_3_with_third_motif_having_DD_or_ED_[Ruminococcus_bror	Ruminococcus_bromii_YE282
Org1_Gene547	core	2275	2595398498_IE32DRAFT_00579_NAD-dependent_protein_deacetylases,_SIR2_family_[Ruminococcus_bromii_YE282]	Ruminococcus_bromii_YE282
Org1_Gene551	core	4333	2595398502_IE32DRAFT_00583_hypothetical_protein_[Ruminococcus_bromii_YE282]	Ruminococcus_bromii_YE282
Org1_Gene553	core	2521	2595398504_IE32DRAFT_00585_SOS_regulatory_protein_LexA_[Ruminococcus_bromii_YE282]	Ruminococcus_bromii_YE282
Org1_Gene559	core	1904	2595398510_IE32DRAFT_00591_3-methyladenine_DNA_glycosylase/8-oxoguanine_DNA_glycosylase_[Ruminococcus_bromii_YE282]	Ruminococcus_bromii_YE282
Org1_Gene560	core	2411	2595398511_IE32DRAFT_00592_hypothetical_protein_[Ruminococcus_bromii_YE282]	Ruminococcus_bromii_YE282
Org1_Gene561	core	2513	2595398512_IE32DRAFT_00593_DNA-(apurinic_or_apyrimidinic_site)_lyase_(EC_4.2.99.18)/endonuclease_III_(EC_3.2.2.-)_[Ruminococcus_bromii_YE282]	Ruminococcus_bromii_YE282
Org1_Gene562	core	2252	2595398513_IE32DRAFT_00594_haloacid_dehalogenase_superfamily,_subfamily_IA,_variant_1_with_third_motif_having_Dx(3-4)D_or_Dx(3-4)E_[Ruminoc	Ruminococcus_bromii_YE282
Org1_Gene6	core	2452	2595397927_IE32DRAFT_00008_Bacterial_transferase_hexapeptide_(six_repeats)_[Ruminococcus_bromii_YE282]	Ruminococcus_bromii_YE282
Org1_Gene65	core	112	2595397988_IE32DRAFT_00069_valyl-tRNA_synthetase_(EC_6.1.1.9)_[Ruminococcus_bromii_YE282]	Ruminococcus_bromii_YE282
Org1_Gene682	core	1067	2595398634_IE32DRAFT_00715_methionine_adenosyltransferase_(EC_2.5.1.6)_[Ruminococcus_bromii_YE282]	Ruminococcus_bromii_YE282
Org1_Gene688	core	1509	2595398640_IE32DRAFT_00722_pseudouridine_synthase,_RluA_family_[Ruminococcus_bromii_YE282]	Ruminococcus_bromii_YE282
Org1_Gene690	core	603	2595398642_IE32DRAFT_00724_stage_IV_sporulation_protein_A_[Ruminococcus_bromii_YE282]	Ruminococcus_bromii_YE282
Org1_Gene692	core	956	2595398644_IE32DRAFT_00726_sodium_ion-translocating_decarboxylase,_beta_subunit_[Ruminococcus_bromii_YE282]	Ruminococcus_bromii_YE282
Org1_Gene696	core	886	2595398648_IE32DRAFT_00730_Alanine-glyoxylate_amino-transferase_[Ruminococcus_bromii_YE282]	Ruminococcus_bromii_YE282
Org1_Gene697	core	663	2595398649_IE32DRAFT_00731_protein-export_membrane_protein,_SecD/SecF_family_[Ruminococcus_bromii_YE282]	Ruminococcus_bromii_YE282
Org1_Gene698	core	1378	2595398650_IE32DRAFT_00732_Preprotein_translocase_subunit_SecF_[Ruminococcus_bromii_YE282]	Ruminococcus_bromii_YE282
Org1_Gene701	core	622	2595398653_IE32DRAFT_00735_Bacillus/Clostridium_GerA_spore_germination_protein_[Ruminococcus_bromii_YE282]	Ruminococcus_bromii_YE282
Org1_Gene704	core	1495	2595398656_IE32DRAFT_00738_Hpr(Ser)_kinase/phosphatase_(EC_2.7.1.-)_[Ruminococcus_bromii_YE282]	Ruminococcus_bromii_YE282
Org1_Gene705	core	1679	2595398657_IE32DRAFT_00739_UDP-N-acetylmuramate_dehydrogenase_(EC_1.1.1.158)_[Ruminococcus_bromii_YE282]	Ruminococcus_bromii_YE282
Org1_Gene706	core	1818	2595398658_IE32DRAFT_00740_Predicted_P-loop-containing_kinase_[Ruminococcus_bromii_YE282]	Ruminococcus_bromii_YE282
Org1_Gene707	core	1590	2595398659_IE32DRAFT_00741_conserved_hypothetical_protein_[Ruminococcus_bromii_YE282]	Ruminococcus_bromii_YE282
Org1_Gene709	core	1774	2595398661_IE32DRAFT_00743_Geranylgeranyl_pyrophosphate_synthase_[Ruminococcus_bromii_YE282]	Ruminococcus_bromii_YE282
Org1_Gene71	core	3646	2595397994_IE32DRAFT_00075_Growth_inhibitor_[Ruminococcus_bromii_YE282]	Ruminococcus_bromii_YE282
Org1_Gene710	core	340	2595398662_IE32DRAFT_00744_1-deoxy-D-xylulose-5-phosphate_synthase_(EC_2.2.1.7)_[Ruminococcus_bromii_YE282]	Ruminococcus_bromii_YE282
Org1_Gene711	core	2015	2595398663_IE32DRAFT_00745_TlyA_family_rRNA_methyltransferase/putative_hemolysin_[Ruminococcus_bromii_YE282]	Ruminococcus_bromii_YE282
Org1_Gene714	core	457	2595398666_IE32DRAFT_00748_DNA_replication_and_repair_protein_RecN_[Ruminococcus_bromii_YE282]	Ruminococcus_bromii_YE282
Org1_Gene716	core	212	2595398668_IE32DRAFT_00750_(p)ppGpp_synthetase,_RelA/SpoT_family_[Ruminococcus_bromii_YE282]	Ruminococcus_bromii_YE282
Org1_Gene717	core	3237	2595398669_IE32DRAFT_00751_D-tyrosyl-tRNA(Tyr)_deacylase_[Ruminococcus_bromii_YE282]	Ruminococcus_bromii_YE282
Org1_Gene718	core	2613	2595398670_IE32DRAFT_00752_Zn-dependent_hydrolases,_including_glyoxylases_[Ruminococcus_bromii_YE282]	Ruminococcus_bromii_YE282
Org1_Gene719	core	624	2595398671_IE32DRAFT_00753_coproporphyrinogen_dehydrogenase_HemZ_[Ruminococcus_bromii_YE282]	Ruminococcus_bromii_YE282
Org1_Gene722	core	2036	2595398674_IE32DRAFT_00756_MazG_family_protein_[Ruminococcus_bromii_YE282]	Ruminococcus_bromii_YE282
Org1_Gene723	core	3918	2595398675_IE32DRAFT_00757_Bacterial_nucleoid_DNA-binding_protein_[Ruminococcus_bromii_YE282]	Ruminococcus_bromii_YE282
Org1_Gene724	core	3251	2595398676_IE32DRAFT_00758_Transcriptional_repressor_of_class_III_stress_genes_[Ruminococcus_bromii_YE282]	Ruminococcus_bromii_YE282
Org1_Gene725	core	3043	2595398677_IE32DRAFT_00759_Uncharacterized_protein_with_conserved_CXXC_pairs_[Ruminococcus_bromii_YE282]	Ruminococcus_bromii_YE282
Org1_Gene726	core	1444	2595398678_IE32DRAFT_00760_Arginine_kinase_[Ruminococcus_bromii_YE282]	Ruminococcus_bromii_YE282
Org1_Gene727	core	141	2595398679_IE32DRAFT_00761_ATPases_with_chaperone_activity,_ATP-binding_subunit_[Ruminococcus_bromii_YE282]	Ruminococcus_bromii_YE282
Org1_Gene729	core	251	2595398681_IE32DRAFT_00763_Type_II_secretory_pathway,_pullulanase_PulA_and_related_glycosidases_[Ruminococcus_bromii_YE282]	Ruminococcus_bromii_YE282
Org1_Gene739	core	702	2595398692_IE32DRAFT_00774_UDP-N-acetylmuramoylalanine--D-glutamate_ligase_(EC_6.3.2.9)_[Ruminococcus_bromii_YE282]	Ruminococcus_bromii_YE282
Org1_Gene74	core	3965	2595397997_IE32DRAFT_00078_Phosphotransferase_System_HPr_(HPr)_Family_[Ruminococcus_bromii_YE282]	Ruminococcus_bromii_YE282
Org1_Gene743	core	902	2595398696_IE32DRAFT_00778_Cystathionine_beta-lyase_family_protein_involved_in_aluminum_resistance_[Ruminococcus_bromii_YE282]	Ruminococcus_bromii_YE282
Org1_Gene748	core	2169	2595398701_IE32DRAFT_00783_exodeoxyribonuclease_III_[Ruminococcus_bromii_YE282]	Ruminococcus_bromii_YE282
Org1_Gene749	core	2395	2595398702_IE32DRAFT_00784_RNA_polymerase_sigma_factor,_sigma-70_family_[Ruminococcus_bromii_YE282]	Ruminococcus_bromii_YE282
Org1_Gene750	core	3230	2595398703_IE32DRAFT_00785_Cytosine/adenosine_deaminases_[Ruminococcus_bromii_YE282]	Ruminococcus_bromii_YE282
Org1_Gene773	core	3540	2595398726_IE32DRAFT_00808_iron_(metal)_dependent_repressor,_DtxR_family_[Ruminococcus_bromii_YE282]	Ruminococcus_bromii_YE282
Org1_Gene774	core	1043	2595398727_IE32DRAFT_00809_threonine_ammonia-lyase,_medium_form_[Ruminococcus_bromii_YE282]	Ruminococcus_bromii_YE282
Org1_Gene775	core	3555	2595398728_IE32DRAFT_00810_reactive_intermediate/imine_deaminase_[Ruminococcus_bromii_YE282]	Ruminococcus_bromii_YE282
Org1_Gene777	core	2795	2595398730_IE32DRAFT_00812_dipicolinic_acid_synthetase,_B_subunit_[Ruminococcus_bromii_YE282]	Ruminococcus_bromii_YE282
Org1_Gene779	core	905	2595398732_IE32DRAFT_00814_OAH/OAS_sulfhydrylase_[Ruminococcus_bromii_YE282]	Ruminococcus_bromii_YE282
Org1_Gene780	core	2362	2595398733_IE32DRAFT_00815_2-C-methyl-D-erythritol_4-phosphate_cytidylyltransferase_[Ruminococcus_bromii_YE282]	Ruminococcus_bromii_YE282
Org1_Gene781	core	3164	2595398734_IE32DRAFT_00816_2-C-methyl-D-erythritol_2,4-cyclodiphosphate_synthase_(EC_4.6.1.12)_[Ruminococcus_bromii_YE282]	Ruminococcus_bromii_YE282
Org1_Gene783	core	1141	2595398736_IE32DRAFT_00818_D-alanyl-D-alanine_carboxypeptidase_[Ruminococcus_bromii_YE282]	Ruminococcus_bromii_YE282
Org1_Gene784	core	787	2595398737_IE32DRAFT_00819_Glucose-6-phosphate_isomerase_[Ruminococcus_bromii_YE282]	Ruminococcus_bromii_YE282
Org1_Gene785	core	3391	2595398738_IE32DRAFT_00820_hypothetical_protein_[Ruminococcus_bromii_YE282]	Ruminococcus_bromii_YE282
Org1_Gene786	core	1129	2595398739_IE32DRAFT_00821_Predicted_ATPase_of_the_PP-loop_superfamily_implicated_in_cell_cycle_control_[Ruminococcus_bromii_YE282]	Ruminococcus_bromii_YE282
Org1_Gene787	core	3074	2595398740_IE32DRAFT_00822_Predicted_metal-binding,_possibly_nucleic_acid-binding_protein_[Ruminococcus_bromii_YE282]	Ruminococcus_bromii_YE282
Org1_Gene788	core	4271	2595398741_IE32DRAFT_00823_ribosomal_protein_L32_[Ruminococcus_bromii_YE282]	Ruminococcus_bromii_YE282

Org1_Gene789	core	819	2595398742_IE32DRAFT_00824_ribosome-associated_GTPase_EngA_[Ruminococcus_bromii_YE282]	Ruminococcus_bromii_YE282
Org1_Gene791	core	3658	2595398744_IE32DRAFT_00826_Diadenosine_tetraphosphate_(Ap4A)_hydrolase_and_other_HIT_family_hydrolases_[Ruminococcus_bromii_YE282]	Ruminococcus_bromii_YE282
Org1_Gene792	core	2935	2595398745_IE32DRAFT_00827_Adenine/guanine_phosphoribosyltransferases_and_related_PRPP-binding_proteins_[Ruminococcus_bromii_YE282]	Ruminococcus_bromii_YE282
Org1_Gene795	core	2718	2595398748_IE32DRAFT_00830_Small_primase-like_proteins_(Toprim_domain)_[Ruminococcus_bromii_YE282]	Ruminococcus_bromii_YE282
Org1_Gene797	core	830	2595398750_IE32DRAFT_00832_Hemolysins_and_related_proteins_containing_CBS_domains_[Ruminococcus_bromii_YE282]	Ruminococcus_bromii_YE282
Org1_Gene798	core	1989	2595398751_IE32DRAFT_00833_Protease_subunit_of_ATP-dependent_Clp_proteases_[Ruminococcus_bromii_YE282]	Ruminococcus_bromii_YE282
Org1_Gene799	core	1521	2595398752_IE32DRAFT_00834_Uncharacterized_bacitracin_resistance_protein_[Ruminococcus_bromii_YE282]	Ruminococcus_bromii_YE282
Org1_Gene8	core	937	2595397929_IE32DRAFT_00010_GTP-binding_protein_HflX_[Ruminococcus_bromii_YE282]	Ruminococcus_bromii_YE282
Org1_Gene801	core	316	2595398754_IE32DRAFT_00836_uncharacterized_radical_SAM_protein_YgiQ_[Ruminococcus_bromii_YE282]	Ruminococcus_bromii_YE282
Org1_Gene803	core	979	2595398756_IE32DRAFT_00838_tyrosyl-tRNA_synthetase_(EC_6.1.1.1)_[Ruminococcus_bromii_YE282]	Ruminococcus_bromii_YE282
Org1_Gene804	core	3312	2595398757_IE32DRAFT_00839_large_conductance_mechanosensitive_channel_protein_[Ruminococcus_bromii_YE282]	Ruminococcus_bromii_YE282
Org1_Gene806	core	354	2595398759_IE32DRAFT_00841_asparagine_synthase_(glutamine-hydrolyzing)_[Ruminococcus_bromii_YE282]	Ruminococcus_bromii_YE282
Org1_Gene810	core	2126	2595398763_IE32DRAFT_00845_metallophosphoesterase,_MG_246/BB_0505_family_[Ruminococcus_bromii_YE282]	Ruminococcus_bromii_YE282
Org1_Gene811	core	470	2595398764_IE32DRAFT_00846_DAK2_domain_fusion_protein_YloV_[Ruminococcus_bromii_YE282]	Ruminococcus_bromii_YE282
Org1_Gene812	core	265	2595398765_IE32DRAFT_00847_ATP-dependent_DNA_helicase_RecG_(EC_3.6.1.-)_[Ruminococcus_bromii_YE282]	Ruminococcus_bromii_YE282
Org1_Gene814	core	958	2595398767_IE32DRAFT_00849_Predicted_ATPase_(AAA+_superfamily)_[Ruminococcus_bromii_YE282]	Ruminococcus_bromii_YE282
Org1_Gene817	core	3962	2595398770_IE32DRAFT_00852_ribosomal_protein_S15,_bacterial/organelle_[Ruminococcus_bromii_YE282]	Ruminococcus_bromii_YE282
Org1_Gene818	core	207	2595398771_IE32DRAFT_00853_polyribonucleotide_nucleotidyltransferase_[Ruminococcus_bromii_YE282]	Ruminococcus_bromii_YE282
Org1_Gene828	core	1065	2595398781_IE32DRAFT_00863_tRNA_sulfurtransferase_ThiI_[Ruminococcus_bromii_YE282]	Ruminococcus_bromii_YE282
Org1_Gene829	core	1182	2595398782_IE32DRAFT_00864_Cysteine_sulfinate_desulfinase/cysteine_desulfurase_and_related_enzymes_[Ruminococcus_bromii_YE282]	Ruminococcus_bromii_YE282
Org1_Gene830	core	3243	2595398783_IE32DRAFT_00865_stage_V_sporulation_protein_AC_[Ruminococcus_bromii_YE282]	Ruminococcus_bromii_YE282
Org1_Gene835	core	1125	2595398788_IE32DRAFT_00870_Phosphoglycerate_dehydrogenase_and_related_dehydrogenases_[Ruminococcus_bromii_YE282]	Ruminococcus_bromii_YE282
Org1_Gene836	core	1255	2595398789_IE32DRAFT_00871_phosphoserine_aminotransferase_apoenzyme_(EC_2.6.1.52)_[Ruminococcus_bromii_YE282]	Ruminococcus_bromii_YE282
Org1_Gene837	core	1588	2595398790_IE32DRAFT_00872_orotidine-5'-phosphate_decarboxylase_(EC_4.1.1.23)_[Ruminococcus_bromii_YE282]	Ruminococcus_bromii_YE282
Org1_Gene839	core	1469	2595398792_IE32DRAFT_00874_HMGL-like_[Ruminococcus_bromii_YE282]	Ruminococcus_bromii_YE282
Org1_Gene840	core	907	2595398793_IE32DRAFT_00875_dihydroorotase,_multifunctional_complex_type_[Ruminococcus_bromii_YE282]	Ruminococcus_bromii_YE282
Org1_Gene849	core	356	2595398802_IE32DRAFT_00884_glucosamine--fructose-6-phosphate_aminotransferase_(isomerizing)_[Ruminococcus_bromii_YE282]	Ruminococcus_bromii_YE282
Org1_Gene850	core	336	2595398803_IE32DRAFT_00885_Predicted_nucleoside-diphosphate_sugar_epimerases_[Ruminococcus_bromii_YE282]	Ruminococcus_bromii_YE282
Org1_Gene875	core	154	2595398828_IE32DRAFT_00910_Homocysteine_S-methyltransferase/B12_binding_domain/Pterin_binding_enzyme_[Ruminococcus_bromii_YE282]	Ruminococcus_bromii_YE282
Org1_Gene877	core	1832	2595398830_IE32DRAFT_00912_5,10-methylenetetrahydrofolate_reductase_(NAD(P))_(EC_1.5.1.20)_[Ruminococcus_bromii_YE282]	Ruminococcus_bromii_YE282
Org1_Gene880	core	1017	2595398833_IE32DRAFT_00915_Predicted_pyridoxal_phosphate-dependent_enzyme_apparently_involved_in_regulation_of_cell_wall_biogenesis_[Rumin	Ruminococcus_bromii_YE282
Org1_Gene881	core	3922	2595398834_IE32DRAFT_00916_sporulation_transcriptional_regulator_SpoIIID_[Ruminococcus_bromii_YE282]	Ruminococcus_bromii_YE282
Org1_Gene890	core	1766	2595398843_IE32DRAFT_00925_Radical_SAM_superfamily_[Ruminococcus_bromii_YE282]	Ruminococcus_bromii_YE282
Org1_Gene896	core	1274	2595398849_IE32DRAFT_00931_tRNA_(5-methylaminomethyl-2-thiouridylate)-methyltransferase_[Ruminococcus_bromii_YE282]	Ruminococcus_bromii_YE282
Org1_Gene897	core	1649	2595398850_IE32DRAFT_00932_quinolinate_synthetase_(EC_2.5.1.72)_[Ruminococcus_bromii_YE282]	Ruminococcus_bromii_YE282
Org1_Gene9	core	611	2595397930_IE32DRAFT_00011_L-aspartate_oxidase_(EC_1.4.3.16)_[Ruminococcus_bromii_YE282]	Ruminococcus_bromii_YE282
Org1_Gene90	core	3905	2595398016_IE32DRAFT_00097_CxxC-x17-CxxC_domain_[Ruminococcus_bromii_YE282]	Ruminococcus_bromii_YE282
Org1_Gene905	core	1069	2595398858_IE32DRAFT_00940_transcriptional_attenuator,_LytR_family_[Ruminococcus_bromii_YE282]	Ruminococcus_bromii_YE282
Org1_Gene906	core	2744	2595398859_IE32DRAFT_00941_Predicted_hydrolases_of_HD_superfamily_[Ruminococcus_bromii_YE282]	Ruminococcus_bromii_YE282
Org1_Gene908	core	743	2595398861_IE32DRAFT_00943_dipeptidase,_putative_[Ruminococcus_bromii_YE282]	Ruminococcus_bromii_YE282
Org1_Gene910	core	1262	2595398863_IE32DRAFT_00945_Glycosyltransferases,_probably_involved_in_cell_wall_biogenesis_[Ruminococcus_bromii_YE282]	Ruminococcus_bromii_YE282
Org1_Gene911	core	1664	2595398864_IE32DRAFT_00946_LPS_biosynthesis_protein_[Ruminococcus_bromii_YE282]	Ruminococcus_bromii_YE282
Org1_Gene915	core	3228	2595398868_IE32DRAFT_00952_transcriptional_regulator_NrdR_[Ruminococcus_bromii_YE282]	Ruminococcus_bromii_YE282
Org1_Gene916	core	3330	2595398869_IE32DRAFT_00953_FeS_cluster_assembly_scaffold_protein_NifU,_Clostridium_type_[Ruminococcus_bromii_YE282]	Ruminococcus_bromii_YE282
Org1_Gene917	core	1027	2595398870_IE32DRAFT_00954_cysteine_desulfurase_NifS_[Ruminococcus_bromii_YE282]	Ruminococcus_bromii_YE282
Org1_Gene918	core	3091	2595398871_IE32DRAFT_00955_Ribonuclease_HI_[Ruminococcus_bromii_YE282]	Ruminococcus_bromii_YE282
Org1_Gene919	core	320	2595398872_IE32DRAFT_00956_beta-CASP_ribonuclease,_RNase_J_family_[Ruminococcus_bromii_YE282]	Ruminococcus_bromii_YE282
Org1_Gene921	core	3258	2595398874_IE32DRAFT_00958_ribosomal_protein_L9_[Ruminococcus_bromii_YE282]	Ruminococcus_bromii_YE282
Org1_Gene922	core	725	2595398875_IE32DRAFT_00959_replicative_DNA_helicase_[Ruminococcus_bromii_YE282]	Ruminococcus_bromii_YE282
Org1_Gene924	core	2937	2595398877_IE32DRAFT_00961_hypoxanthine_phosphoribosyltransferase_[Ruminococcus_bromii_YE282]	Ruminococcus_bromii_YE282
Org1_Gene925	core	270	2595398878_IE32DRAFT_00962_ATP-dependent_metalloprotease_FtsH_[Ruminococcus_bromii_YE282]	Ruminococcus_bromii_YE282
Org1_Gene926	core	99	2595398879_IE32DRAFT_00963_excinuclease_ABC,_A_subunit_[Ruminococcus_bromii_YE282]	Ruminococcus_bromii_YE282
Org1_Gene927	core	3992	2595398880_IE32DRAFT_00964_hypothetical_protein_[Ruminococcus_bromii_YE282]	Ruminococcus_bromii_YE282
Org1_Gene929	core	1871	2595398882_IE32DRAFT_00966_haloacid_dehalogenase-like_hydrolase_[Ruminococcus_bromii_YE282]	Ruminococcus_bromii_YE282
Org1_Gene931	core	1618	2595398884_IE32DRAFT_00968_S1_RNA_binding_domain_[Ruminococcus_bromii_YE282]	Ruminococcus_bromii_YE282
Org1_Gene949	core	1285	2595398903_IE32DRAFT_00987_3-isopropylmalate_dehydrogenase_[Ruminococcus_bromii_YE282]	Ruminococcus_bromii_YE282
Org1_Gene950	core	2610	2595398904_IE32DRAFT_00988_Fructose-2,6-bisphosphatase_[Ruminococcus_bromii_YE282]	Ruminococcus_bromii_YE282
Org1_Gene994	core	2205	2595398949_IE32DRAFT_01033_ABC-type_polysaccharide/polyol_phosphate_transport_system,_ATPase_component_[Ruminococcus_bromii_YE282]	Ruminococcus_bromii_YE282
Org1_Gene995	core	1968	2595398950_IE32DRAFT_01034_ABC-type_polysaccharide/polyol_phosphate_export_systems,_permease_component_[Ruminococcus_bromii_YE282]	Ruminococcus_bromii_YE282
Org1_Gene996	core	3849	2595398951_IE32DRAFT_01035_ribosomal_protein_S6_[Ruminococcus_bromii_YE282]	Ruminococcus_bromii_YE282
Org1_Gene997	core	3056	2595398952_IE32DRAFT_01036_single_stranded_DNA-binding_protein_(ssb)_[Ruminococcus_bromii_YE282]	Ruminococcus_bromii_YE282
Org1_Gene998	core	4065	2595398953_IE32DRAFT_01037_ribosomal_protein_S18_[Ruminococcus_bromii_YE282]	Ruminococcus_bromii_YE282
Org1_Gene999	core	3823	2595398954_IE32DRAFT_01038_Uncharacterized_protein_conserved_in_bacteria_[Ruminococcus_bromii_YE282]	Ruminococcus_bromii_YE282
Org2_Gene1006	core	2188	[Rbromii5AMG]RB5AMG_01037_Sulfur_carrier_protein_ThiS_adenylyltransferase	Rbromii5AMG
Org2_Gene1041	core	231	[Rbromii5AMG]RB5AMG_01072_Silver_exporting_P-type_ATPase	Rbromii5AMG
Org2_Gene1049	core	2898	[Rbromii5AMG]RB5AMG_01080_hypothetical_protein	Rbromii5AMG
Org2_Gene1068	core	3244	[Rbromii5AMG]RB5AMG_01099_3-hydroxyacyl-[acyl-carrier-protein]_dehydratase_FabZ	Rbromii5AMG
Org2_Gene1069	core	2246	[Rbromii5AMG]RB5AMG_01100_3-oxoacyl-[acyl-carrier-protein]_reductase_FabG	Rbromii5AMG
Org2_Gene1071	core	4031	[Rbromii5AMG]RB5AMG_01102_Acyl_carrier_protein	Rbromii5AMG
Org2_Gene1079	core	2494	[Rbromii5AMG]RB5AMG_01113_V-type_sodium_pump_subunit_D	Rbromii5AMG
Org2_Gene1080	core	723	[Rbromii5AMG]RB5AMG_01114_V-type_sodium_pump_subunit_B	Rbromii5AMG
Org2_Gene1081	core	384	[Rbromii5AMG]RB5AMG_01115_V-type_sodium_ATPase_catalytic_subunit_A	Rbromii5AMG
Org2_Gene1082	core	3757	[Rbromii5AMG]RB5AMG_01116_V-type_sodium_pump_subunit_G	Rbromii5AMG
Org2_Gene1085	core	3160	[Rbromii5AMG]RB5AMG_01119_Sodium_ATPase_proteolipid_component	Rbromii5AMG
Org2_Gene1089	core	2892	[Rbromii5AMG]RB5AMG_01123_Cys-tRNA(Pro)/Cys-tRNA(Cys)_deacylase_YbaK	Rbromii5AMG
Org2_Gene1119	core	2611	[Rbromii5AMG]RB5AMG_01153_putative_manganese_catalase	Rbromii5AMG
Org2_Gene1122	core	2544	[Rbromii5AMG]RB5AMG_01156_L-cystine_transport_system_permease_protein_TcyB	Rbromii5AMG
Org2_Gene1123	core	2029	[Rbromii5AMG]RB5AMG_01157_L-cystine_import_ATP-binding_protein_TcyC	Rbromii5AMG
Org2_Gene1137	core	1898	[Rbromii5AMG]RB5AMG_01172_Formate_channel_1	Rbromii5AMG
Org2_Gene1140	core	224	[Rbromii5AMG]RB5AMG_01175_30S_ribosomal_protein_S1	Rbromii5AMG
Org2_Gene1142	core	3540	[Rbromii5AMG]RB5AMG_01177_Manganese_transport_regulator	Rbromii5AMG
Org2_Gene1143	core	4164	[Rbromii5AMG]RB5AMG_01178_FeoA_domain_protein	Rbromii5AMG
Org2_Gene1144	core	4121	[Rbromii5AMG]RB5AMG_01179_Ferrous_iron_transport_protein_A	Rbromii5AMG
Org2_Gene1145	core	134	[Rbromii5AMG]RB5AMG_01180_Ferrous_iron_transport_protein_B	Rbromii5AMG
Org2_Gene1152	core	3361	[Rbromii5AMG]RB5AMG_01187_Flavodoxin	Rbromii5AMG
Org2_Gene1153	core	1043	[Rbromii5AMG]RB5AMG_01188_L-threonine_dehydratase_catabolic_TdcB	Rbromii5AMG
Org2_Gene1154	core	3555	[Rbromii5AMG]RB5AMG_01189_Enamine/imine_deaminase	Rbromii5AMG
Org2_Gene1155	core	1067	[Rbromii5AMG]RB5AMG_01190_S-adenosylmethionine_synthase	Rbromii5AMG
Org2_Gene1171	core	1397	[Rbromii5AMG]RB5AMG_01207_UDP-glucose_4-epimerase	Rbromii5AMG
Org2_Gene1172	core	2990	[Rbromii5AMG]RB5AMG_01208_Peptidyl-prolyl_cis-trans_isomerase_B	Rbromii5AMG
Org2_Gene1173	core	1404	[Rbromii5AMG]RB5AMG_01209_t(6)A37_threonylcarbamoyladenosine_biosynthesis_protein	Rbromii5AMG
Org2_Gene1176	core	129	[Rbromii5AMG]RB5AMG_01212_DNA_polymerase_I	Rbromii5AMG
Org2_Gene1177	core	1074	[Rbromii5AMG]RB5AMG_01213_Alanine_racemase	Rbromii5AMG
Org2_Gene1180	core	1145	[Rbromii5AMG]RB5AMG_01217_Purine_catabolism_regulatory_protein	Rbromii5AMG
Org2_Gene119	core	2223	[Rbromii5AMG]RB5AMG_00127_Arginine_transport_ATP-binding_protein_ArtM	Rbromii5AMG
Org2_Gene1198	core	1902	[Rbromii5AMG]RB5AMG_01235_putative_nicotinate-nucleotide_pyrophosphorylase_[carboxylating]	Rbromii5AMG
Org2_Gene1199	core	611	[Rbromii5AMG]RB5AMG_01236_L-aspartate_oxidase	Rbromii5AMG
Org2_Gene12	core	1105	[Rbromii5AMG]RB5AMG_00012_H(2)O-forming_NADH_oxidase	Rbromii5AMG
Org2_Gene1208	core	1214	[Rbromii5AMG]RB5AMG_01245_Cell_division_protein_FtsZ	Rbromii5AMG
Org2_Gene121	core	1085	[Rbromii5AMG]RB5AMG_00129_NADP-dependent_malic_enzyme	Rbromii5AMG
Org2_Gene1210	core	933	[Rbromii5AMG]RB5AMG_01247_UDP-N-acetylglucosamine_1-carboxyvinyltransferase_1	Rbromii5AMG
Org2_Gene1211	core	1196	[Rbromii5AMG]RB5AMG_01248_UDP-N-acetylglucosamine--N-acetylmuramyl-(pentapeptide)_pyrophosphoryl-undecaprenol_N-acetylglucosamine_transfe	Rbromii5AMG
Org2_Gene1212	core	928	[Rbromii5AMG]RB5AMG_01249_Cell_division_protein_FtsW	Rbromii5AMG
Org2_Gene1213	core	1356	[Rbromii5AMG]RB5AMG_01250_Phospho-N-acetylmuramoyl-pentapeptide-transferase	Rbromii5AMG
Org2_Gene1214	core	194	[Rbromii5AMG]RB5AMG_01251_Sporulation-specific_penicillin-binding_protein	Rbromii5AMG
Org2_Gene1216	core	1626	[Rbromii5AMG]RB5AMG_01253_Ribosomal_RNA_small_subunit_methyltransferase_H	Rbromii5AMG
Org2_Gene1218	core	3206	[Rbromii5AMG]RB5AMG_01255_hypothetical_protein	Rbromii5AMG
Org2_Gene1220	core	2851	[Rbromii5AMG]RB5AMG_01257_Ribosomal_RNA_small_subunit_methyltransferase_D	Rbromii5AMG
Org2_Gene1221	core	347	[Rbromii5AMG]RB5AMG_01258_Excinuclease_ABC_subunit_C	Rbromii5AMG
Org2_Gene1222	core	3960	[Rbromii5AMG]RB5AMG_01259_Catabolite_repression_HPr	Rbromii5AMG
Org2_Gene1223	core	2404	[Rbromii5AMG]RB5AMG_01260_tRNA_(guanine-N(1)-)-methyltransferase	Rbromii5AMG
Org2_Gene1224	core	3006	[Rbromii5AMG]RB5AMG_01261_Ribosome_maturation_factor_RimM	Rbromii5AMG
Org2_Gene1225	core	135	[Rbromii5AMG]RB5AMG_01262_MutS2_protein	Rbromii5AMG
Org2_Gene1226	core	2204	[Rbromii5AMG]RB5AMG_01263_Recombination_protein_O	Rbromii5AMG
Org2_Gene1227	core	4338	[Rbromii5AMG]RB5AMG_01264_hypothetical_protein	Rbromii5AMG
Org2_Gene1228	core	1643	[Rbromii5AMG]RB5AMG_01265_Bex_protein	Rbromii5AMG
Org2_Gene1230	core	3007	[Rbromii5AMG]RB5AMG_01267_Endoribonuclease_YbeY	Rbromii5AMG
Org2_Gene1231	core	1321	[Rbromii5AMG]RB5AMG_01268_PhoH-like_protein	Rbromii5AMG
Org2_Gene1232	core	3631	[Rbromii5AMG]RB5AMG_01269_putative_DNA-binding_protein	Rbromii5AMG
Org2_Gene1233	core	676	[Rbromii5AMG]RB5AMG_01270_hypothetical_protein	Rbromii5AMG
Org2_Gene1234	core	4034	[Rbromii5AMG]RB5AMG_01271_hypothetical_protein	Rbromii5AMG
Org2_Gene1235	core	4080	[Rbromii5AMG]RB5AMG_01272_putative_RNA-binding_protein_(contains_KH_domain)	Rbromii5AMG

Org2_Gene1237	core	141	[Rbromii5AMG]RB5AMG_01274_Negative_regulator_of_genetic_competence_ClpC/MecB	Rbromii5AMG
Org2_Gene1238	core	1444	[Rbromii5AMG]RB5AMG_01275_Putative_ATP:guanido_phosphotransferase	Rbromii5AMG
Org2_Gene1239	core	3043	[Rbromii5AMG]RB5AMG_01276_hypothetical_protein	Rbromii5AMG
Org2_Gene1240	core	3251	[Rbromii5AMG]RB5AMG_01277_Class_three_stress_gene_repressor	Rbromii5AMG
Org2_Gene1249	core	3905	[Rbromii5AMG]RB5AMG_01286_CxxC-x17-CxxC_domain_protein	Rbromii5AMG
Org2_Gene125	core	517	[Rbromii5AMG]RB5AMG_00134_anaerobic_glycerol-3-phosphate_dehydrogenase_subunit_B	Rbromii5AMG
Org2_Gene1252	core	4248	[Rbromii5AMG]RB5AMG_01289_hybrid_cluster_protein-associated_redox_disulfide_domain_protein	Rbromii5AMG
Org2_Gene1255	core	2484	[Rbromii5AMG]RB5AMG_01292_tRNA_(guanine-N(7)-)-methyltransferase	Rbromii5AMG
Org2_Gene1257	core	2345	[Rbromii5AMG]RB5AMG_01294_Sensory_transduction_protein_regX3	Rbromii5AMG
Org2_Gene1258	core	605	[Rbromii5AMG]RB5AMG_01295_Alkaline_phosphatase_synthesis_sensor_protein_PhoR	Rbromii5AMG
Org2_Gene1259	core	1443	[Rbromii5AMG]RB5AMG_01296_putative_metal-dependent_enzyme	Rbromii5AMG
Org2_Gene1261	core	1195	[Rbromii5AMG]RB5AMG_01298_Recombinase_A	Rbromii5AMG
Org2_Gene1262	core	2648	[Rbromii5AMG]RB5AMG_01299_Regulatory_protein_RecX	Rbromii5AMG
Org2_Gene1263	core	778	[Rbromii5AMG]RB5AMG_01300_Ribosomal_protein_S12_methylthiotransferase_RimO	Rbromii5AMG
Org2_Gene1264	core	2725	[Rbromii5AMG]RB5AMG_01301_CDP-diacylglycerol--glycerol-3-phosphate_3-phosphatidyltransferase	Rbromii5AMG
Org2_Gene1265	core	2491	[Rbromii5AMG]RB5AMG_01302_Serine_acetyltransferase	Rbromii5AMG
Org2_Gene1266	core	685	[Rbromii5AMG]RB5AMG_01303_Cysteine--tRNA_ligase	Rbromii5AMG
Org2_Gene1267	core	2056	[Rbromii5AMG]RB5AMG_01304_Bifunctional_xylanase/deacetylase_precursor	Rbromii5AMG
Org2_Gene1274	core	3239	[Rbromii5AMG]RB5AMG_01311_S-ribosylhomocysteine_lyase	Rbromii5AMG
Org2_Gene1277	core	2998	[Rbromii5AMG]RB5AMG_01314_tRNA_(cytidine(34)-2'-O)-methyltransferase	Rbromii5AMG
Org2_Gene1278	core	1009	[Rbromii5AMG]RB5AMG_01315_Phosphoglycerate_kinase	Rbromii5AMG
Org2_Gene1279	core	2143	[Rbromii5AMG]RB5AMG_01316_Triosephosphate_isomerase	Rbromii5AMG
Org2_Gene1280	core	570	[Rbromii5AMG]RB5AMG_01317_2,3-bisphosphoglycerate-independent_phosphoglycerate_mutase	Rbromii5AMG
Org2_Gene1281	core	405	[Rbromii5AMG]RB5AMG_01318_putative_ABC_transporter_ATP-binding_protein	Rbromii5AMG
Org2_Gene1282	core	396	[Rbromii5AMG]RB5AMG_01319_putative_ABC_transporter_ATP-binding_protein	Rbromii5AMG
Org2_Gene1285	core	258	[Rbromii5AMG]RB5AMG_01322_Elongation_factor_G	Rbromii5AMG
Org2_Gene1286	core	371	[Rbromii5AMG]RB5AMG_01323_Dihydroxy-acid_dehydratase	Rbromii5AMG
Org2_Gene1289	core	2506	[Rbromii5AMG]RB5AMG_01326_Phosphorylated_carbohydrates_phosphatase	Rbromii5AMG
Org2_Gene129	core	690	[Rbromii5AMG]RB5AMG_00138_Multidrug_export_protein_MepA	Rbromii5AMG
Org2_Gene1295	core	118	[Rbromii5AMG]RB5AMG_01333_Alanine--tRNA_ligase	Rbromii5AMG
Org2_Gene1296	core	971	[Rbromii5AMG]RB5AMG_01335_putative_protease_YhbU_precursor	Rbromii5AMG
Org2_Gene1299	core	2911	[Rbromii5AMG]RB5AMG_01338_Chaperone_protein_YajL	Rbromii5AMG
Org2_Gene1303	core	2018	[Rbromii5AMG]RB5AMG_01342_Thymidylate_synthase_ThyX	Rbromii5AMG
Org2_Gene1306	core	641	[Rbromii5AMG]RB5AMG_01345_Nicotinate_phosphoribosyltransferase_pncB2	Rbromii5AMG
Org2_Gene132	core	346	[Rbromii5AMG]RB5AMG_00141_B12-binding_domain/radical_SAM_domain_protein,_family	Rbromii5AMG
Org2_Gene133	core	2509	[Rbromii5AMG]RB5AMG_00142_radical_SAM-linked_protein	Rbromii5AMG
Org2_Gene137	core	2319	[Rbromii5AMG]RB5AMG_00147_Uridylate_kinase	Rbromii5AMG
Org2_Gene138	core	2866	[Rbromii5AMG]RB5AMG_00148_Ribosome-releasing_factor	Rbromii5AMG
Org2_Gene139	core	2189	[Rbromii5AMG]RB5AMG_00149_Ditrans,polycis-undecaprenyl-diphosphate_synthase_((2E,6E)-farnesyl-diphosphate_specific)	Rbromii5AMG
Org2_Gene141	core	1147	[Rbromii5AMG]RB5AMG_00151_1-deoxy-D-xylulose_5-phosphate_reductoisomerase	Rbromii5AMG
Org2_Gene1412	core	112	[Rbromii5AMG]RB5AMG_01452_Valine--tRNA_ligase	Rbromii5AMG
Org2_Gene1417	core	553	[Rbromii5AMG]RB5AMG_01457_Trehalose_synthase/amylase_TreS	Rbromii5AMG
Org2_Gene1420	core	1155	[Rbromii5AMG]RB5AMG_01460_P-protein	Rbromii5AMG
Org2_Gene1421	core	1278	[Rbromii5AMG]RB5AMG_01461_Chorismate_synthase	Rbromii5AMG
Org2_Gene1422	core	859	[Rbromii5AMG]RB5AMG_01462_3-phosphoshikimate_1-carboxyvinyltransferase	Rbromii5AMG
Org2_Gene1423	core	1306	[Rbromii5AMG]RB5AMG_01463_3-dehydroquinate_synthase	Rbromii5AMG
Org2_Gene1424	core	1936	[Rbromii5AMG]RB5AMG_01464_Arogenate_dehydrogenase	Rbromii5AMG
Org2_Gene1425	core	1379	[Rbromii5AMG]RB5AMG_01465_Phospho-2-dehydro-3-deoxyheptonate_aldolase	Rbromii5AMG
Org2_Gene1427	core	1884	[Rbromii5AMG]RB5AMG_01467_Shikimate_dehydrogenase	Rbromii5AMG
Org2_Gene1428	core	4101	[Rbromii5AMG]RB5AMG_01468_Acyl_carrier_protein	Rbromii5AMG
Org2_Gene1429	core	644	[Rbromii5AMG]RB5AMG_01469_Proline--tRNA_ligase	Rbromii5AMG
Org2_Gene143	core	1335	[Rbromii5AMG]RB5AMG_00153_4-hydroxy-3-methylbut-2-en-1-yl_diphosphate_synthase	Rbromii5AMG
Org2_Gene1431	core	1602	[Rbromii5AMG]RB5AMG_01471_Homoserine_O-succinyltransferase	Rbromii5AMG
Org2_Gene1432	core	1993	[Rbromii5AMG]RB5AMG_01472_Glutamate_5-kinase_1	Rbromii5AMG
Org2_Gene1433	core	945	[Rbromii5AMG]RB5AMG_01473_Gamma-glutamyl_phosphate_reductase	Rbromii5AMG
Org2_Gene1439	core	1810	[Rbromii5AMG]RB5AMG_01479_hypothetical_protein	Rbromii5AMG
Org2_Gene144	core	21	[Rbromii5AMG]RB5AMG_00154_DNA_polymerase_III_PolC-type	Rbromii5AMG
Org2_Gene1440	core	4343	[Rbromii5AMG]RB5AMG_01480_Ferredoxin	Rbromii5AMG
Org2_Gene1442	core	1955	[Rbromii5AMG]RB5AMG_01482_Ribosomal_RNA_small_subunit_methyltransferase_I	Rbromii5AMG
Org2_Gene1444	core	3765	[Rbromii5AMG]RB5AMG_01484_Stage_II_sporulation_protein_AA	Rbromii5AMG
Org2_Gene1445	core	3296	[Rbromii5AMG]RB5AMG_01485_Anti-sigma_F_factor	Rbromii5AMG
Org2_Gene1446	core	2501	[Rbromii5AMG]RB5AMG_01486_Stage_II_sporulation_protein_AC	Rbromii5AMG
Org2_Gene1448	core	639	[Rbromii5AMG]RB5AMG_01488_Pyruvate_kinase	Rbromii5AMG
Org2_Gene1451	core	3661	[Rbromii5AMG]RB5AMG_01491_hypothetical_protein	Rbromii5AMG
Org2_Gene1452	core	1541	[Rbromii5AMG]RB5AMG_01492_DNA-directed_RNA_polymerase_subunit_alpha	Rbromii5AMG
Org2_Gene1453	core	2686	[Rbromii5AMG]RB5AMG_01493_30S_ribosomal_protein_S4	Rbromii5AMG
Org2_Gene1454	core	3445	[Rbromii5AMG]RB5AMG_01494_30S_ribosomal_protein_S11	Rbromii5AMG
Org2_Gene1455	core	3568	[Rbromii5AMG]RB5AMG_01495_30S_ribosomal_protein_S13	Rbromii5AMG
Org2_Gene1457	core	4142	[Rbromii5AMG]RB5AMG_01497_Translation_initiation_factor_IF-1	Rbromii5AMG
Org2_Gene1458	core	4037	[Rbromii5AMG]RB5AMG_01498_hypothetical_protein	Rbromii5AMG
Org2_Gene1459	core	2186	[Rbromii5AMG]RB5AMG_01499_Methionine_aminopeptidase_1	Rbromii5AMG
Org2_Gene146	core	1098	[Rbromii5AMG]RB5AMG_00156_ATP_phosphoribosyltransferase_regulatory_subunit	Rbromii5AMG
Org2_Gene1460	core	2593	[Rbromii5AMG]RB5AMG_01500_Adenylate_kinase	Rbromii5AMG
Org2_Gene1461	core	782	[Rbromii5AMG]RB5AMG_01501_preprotein_translocase_subunit_SecY	Rbromii5AMG
Org2_Gene1462	core	3283	[Rbromii5AMG]RB5AMG_01502_50S_ribosomal_protein_L15	Rbromii5AMG
Org2_Gene1463	core	4346	[Rbromii5AMG]RB5AMG_01503_50S_ribosomal_protein_L30	Rbromii5AMG
Org2_Gene1464	core	3067	[Rbromii5AMG]RB5AMG_01504_30S_ribosomal_protein_S5	Rbromii5AMG
Org2_Gene1465	core	3597	[Rbromii5AMG]RB5AMG_01505_hypothetical_protein	Rbromii5AMG
Org2_Gene1466	core	2900	[Rbromii5AMG]RB5AMG_01506_hypothetical_protein	Rbromii5AMG
Org2_Gene1467	core	3469	[Rbromii5AMG]RB5AMG_01507_30S_ribosomal_protein_S8	Rbromii5AMG
Org2_Gene1468	core	4289	[Rbromii5AMG]RB5AMG_01508_hypothetical_protein	Rbromii5AMG
Org2_Gene1469	core	2914	[Rbromii5AMG]RB5AMG_01509_50S_ribosomal_protein_L5	Rbromii5AMG
Org2_Gene147	core	2601	[Rbromii5AMG]RB5AMG_00157_ATP_phosphoribosyltransferase	Rbromii5AMG
Org2_Gene1470	core	3773	[Rbromii5AMG]RB5AMG_01510_50S_ribosomal_protein_L24	Rbromii5AMG
Org2_Gene1471	core	3565	[Rbromii5AMG]RB5AMG_01511_50S_ribosomal_protein_L14	Rbromii5AMG
Org2_Gene1472	core	3990	[Rbromii5AMG]RB5AMG_01512_30S_ribosomal_protein_S17	Rbromii5AMG
Org2_Gene1473	core	4200	[Rbromii5AMG]RB5AMG_01513_50S_ribosomal_protein_L29	Rbromii5AMG
Org2_Gene1474	core	3375	[Rbromii5AMG]RB5AMG_01514_50S_ribosomal_protein_L16	Rbromii5AMG
Org2_Gene1475	core	2426	[Rbromii5AMG]RB5AMG_01515_hypothetical_protein	Rbromii5AMG
Org2_Gene1476	core	3667	[Rbromii5AMG]RB5AMG_01516_50S_ribosomal_protein_L22	Rbromii5AMG
Org2_Gene1477	core	3940	[Rbromii5AMG]RB5AMG_01517_hypothetical_protein	Rbromii5AMG
Org2_Gene1478	core	1925	[Rbromii5AMG]RB5AMG_01518_hypothetical_protein	Rbromii5AMG
Org2_Gene1479	core	3858	[Rbromii5AMG]RB5AMG_01519_50S_ribosomal_protein_L23	Rbromii5AMG
Org2_Gene148	core	864	[Rbromii5AMG]RB5AMG_00158_Histidinol_dehydrogenase	Rbromii5AMG
Org2_Gene1480	core	2622	[Rbromii5AMG]RB5AMG_01520_50S_ribosomal_protein_L4	Rbromii5AMG
Org2_Gene1481	core	2594	[Rbromii5AMG]RB5AMG_01521_50S_ribosomal_protein_L3	Rbromii5AMG
Org2_Gene1482	core	3796	[Rbromii5AMG]RB5AMG_01522_hypothetical_protein	Rbromii5AMG
Org2_Gene1485	core	701	[Rbromii5AMG]RB5AMG_01525_D-alanyl-lipoteichoic_acid_biosynthesis_protein_DltB	Rbromii5AMG
Org2_Gene149	core	1314	[Rbromii5AMG]RB5AMG_00159_Histidinol-phosphate_aminotransferase	Rbromii5AMG
Org2_Gene1492	core	1065	[Rbromii5AMG]RB5AMG_01532_putative_tRNA_sulfurtransferase	Rbromii5AMG
Org2_Gene1493	core	1182	[Rbromii5AMG]RB5AMG_01533_Cysteine_desulfurase	Rbromii5AMG
Org2_Gene1494	core	3243	[Rbromii5AMG]RB5AMG_01534_stage_V_sporulation_protein_AC	Rbromii5AMG
Org2_Gene1495	core	1023	[Rbromii5AMG]RB5AMG_01535_Elongation_factor_Tu	Rbromii5AMG
Org2_Gene1496	core	240	[Rbromii5AMG]RB5AMG_01536_Vegetative_protein_19	Rbromii5AMG
Org2_Gene1497	core	2922	[Rbromii5AMG]RB5AMG_01537_30S_ribosomal_protein_S7	Rbromii5AMG
Org2_Gene1498	core	3378	[Rbromii5AMG]RB5AMG_01538_30S_ribosomal_protein_S12	Rbromii5AMG
Org2_Gene1499	core	460	[Rbromii5AMG]RB5AMG_01539_Alpha-amylase_precursor	Rbromii5AMG
Org2_Gene15	core	207	[Rbromii5AMG]RB5AMG_00015_Polyribonucleotide_nucleotidyltransferase	Rbromii5AMG
Org2_Gene150	core	2773	[Rbromii5AMG]RB5AMG_00160_Imidazoleglycerol-phosphate_dehydratase	Rbromii5AMG
Org2_Gene1500	core	1824	[Rbromii5AMG]RB5AMG_01540_Maltose_transport_system_permease_protein_MalG	Rbromii5AMG
Org2_Gene1501	core	581	[Rbromii5AMG]RB5AMG_01541_Maltose_transport_system_permease_protein_MalF	Rbromii5AMG
Org2_Gene1503	core	607	[Rbromii5AMG]RB5AMG_01543_UDP-N-acetylmuramoyl-L-alanyl-D-glutamate--L-lysine_ligase	Rbromii5AMG
Org2_Gene1504	core	2221	[Rbromii5AMG]RB5AMG_01544_3-oxoacyl-[acyl-carrier-protein]_reductase_FabG	Rbromii5AMG
Org2_Gene1505	core	434	[Rbromii5AMG]RB5AMG_01545_Glutamine--tRNA_ligase	Rbromii5AMG
Org2_Gene1506	core	626	[Rbromii5AMG]RB5AMG_01546_Glutamate--tRNA_ligase	Rbromii5AMG
Org2_Gene151	core	2300	[Rbromii5AMG]RB5AMG_00161_1-(5-phosphoribosyl)-5-[(5-phosphoribosylamino)methylideneamino]_imidazole-4-carboxamide_isomerase	Rbromii5AMG
Org2_Gene1510	core	1402	[Rbromii5AMG]RB5AMG_01550_Stage_V_sporulation_protein_AD	Rbromii5AMG
Org2_Gene1511	core	2876	[Rbromii5AMG]RB5AMG_01551_undecaprenyl_pyrophosphate_phosphatase	Rbromii5AMG
Org2_Gene1514	core	931	[Rbromii5AMG]RB5AMG_01554_Serine_hydroxymethyltransferase	Rbromii5AMG
Org2_Gene1515	core	1439	[Rbromii5AMG]RB5AMG_01555_putative_tRNA-dihydrouridine_synthase	Rbromii5AMG
Org2_Gene152	core	3785	[Rbromii5AMG]RB5AMG_00162_phosphoribosyl-AMP_cyclohydrolase	Rbromii5AMG
Org2_Gene1522	core	297	[Rbromii5AMG]RB5AMG_01562_Methionine--tRNA_ligase	Rbromii5AMG

Org2_Gene1523	core	2111	[Rbromii5AMG]RB5AMG_01563_putative_deoxyribonuclease_YcfH	Rbromii5AMG
Org2_Gene153	core	3697	[Rbromii5AMG]RB5AMG_00163_Phosphoribosyl-ATP_pyrophosphatase	Rbromii5AMG
Org2_Gene1530	core	2109	[Rbromii5AMG]RB5AMG_01570_Glucose-1-phosphate_cytidylyltransferase	Rbromii5AMG
Org2_Gene1531	core	1311	[Rbromii5AMG]RB5AMG_01571_CDP-glucose_4,6-dehydratase	Rbromii5AMG
Org2_Gene1532	core	1601	[Rbromii5AMG]RB5AMG_01572_dTDP-glucose_4,6-dehydratase	Rbromii5AMG
Org2_Gene1533	core	2793	[Rbromii5AMG]RB5AMG_01573_dTDP-4-dehydrorhamnose_3,5-epimerase	Rbromii5AMG
Org2_Gene155	core	869	[Rbromii5AMG]RB5AMG_00165_Threonylcarbamoyladenosine_tRNA_methylthiotransferase_MtaB	Rbromii5AMG
Org2_Gene1551	core	4278	[Rbromii5AMG]RB5AMG_01591_50S_ribosomal_protein_L28	Rbromii5AMG
Org2_Gene1553	core	2124	[Rbromii5AMG]RB5AMG_01593_Segregation_and_condensation_protein_A	Rbromii5AMG
Org2_Gene1554	core	2631	[Rbromii5AMG]RB5AMG_01594_Segregation_and_condensation_protein_B	Rbromii5AMG
Org2_Gene1556	core	3344	[Rbromii5AMG]RB5AMG_01596_putative_spore_protein_YtfJ	Rbromii5AMG
Org2_Gene1558	core	2196	[Rbromii5AMG]RB5AMG_01598_Ribosomal_large_subunit_pseudouridine_synthase_B	Rbromii5AMG
Org2_Gene1559	core	839	[Rbromii5AMG]RB5AMG_01599_Methylmalonyl-CoA_carboxyltransferase_12S_subunit	Rbromii5AMG
Org2_Gene1560	core	3572	[Rbromii5AMG]RB5AMG_01600_Glutaconyl-CoA_decarboxylase_subunit_gamma	Rbromii5AMG
Org2_Gene1561	core	687	[Rbromii5AMG]RB5AMG_01601_Methylmalonyl-CoA_carboxyltransferase_5S_subunit	Rbromii5AMG
Org2_Gene1564	core	1341	[Rbromii5AMG]RB5AMG_01604_Ketol-acid_reductoisomerase	Rbromii5AMG
Org2_Gene1565	core	3087	[Rbromii5AMG]RB5AMG_01605_Acetolactate_synthase_small_subunit	Rbromii5AMG
Org2_Gene1566	core	494	[Rbromii5AMG]RB5AMG_01606_Acetolactate_synthase_large_subunit	Rbromii5AMG
Org2_Gene1569	core	3702	[Rbromii5AMG]RB5AMG_01609_hypothetical_protein	Rbromii5AMG
Org2_Gene1571	core	738	[Rbromii5AMG]RB5AMG_01611_UDP-N-acetylglucosamine_1-carboxyvinyltransferase_2	Rbromii5AMG
Org2_Gene1572	core	2075	[Rbromii5AMG]RB5AMG_01612_Putative_metallo-hydrolase_YycJ	Rbromii5AMG
Org2_Gene1573	core	3115	[Rbromii5AMG]RB5AMG_01613_Ribosomal_RNA_large_subunit_methyltransferase_H	Rbromii5AMG
Org2_Gene1574	core	403	[Rbromii5AMG]RB5AMG_01614_hypothetical_protein	Rbromii5AMG
Org2_Gene1575	core	1766	[Rbromii5AMG]RB5AMG_01615_anaerobic_ribonucleoside-triphosphate_reductase_activating_protein	Rbromii5AMG
Org2_Gene1578	core	1274	[Rbromii5AMG]RB5AMG_01618_tRNA-specific_2-thiouridylase_MnmA	Rbromii5AMG
Org2_Gene1579	core	1649	[Rbromii5AMG]RB5AMG_01619_Quinolinate_synthase_A	Rbromii5AMG
Org2_Gene1583	core	1069	[Rbromii5AMG]RB5AMG_01623_Putative_transcriptional_regulator_YvhJ	Rbromii5AMG
Org2_Gene1584	core	2744	[Rbromii5AMG]RB5AMG_01624_5'-nucleotidase_YfbR	Rbromii5AMG
Org2_Gene1586	core	743	[Rbromii5AMG]RB5AMG_01626_Beta-Ala-Xaa_dipeptidase	Rbromii5AMG
Org2_Gene1588	core	1262	[Rbromii5AMG]RB5AMG_01628_N-glycosyltransferase	Rbromii5AMG
Org2_Gene1589	core	1664	[Rbromii5AMG]RB5AMG_01629_LPS_biosynthesis_protein	Rbromii5AMG
Org2_Gene1593	core	657	[Rbromii5AMG]RB5AMG_01635_Glycogen_synthase	Rbromii5AMG
Org2_Gene1594	core	1212	[Rbromii5AMG]RB5AMG_01636_Glycogen_biosynthesis_protein_GlgD	Rbromii5AMG
Org2_Gene1595	core	999	[Rbromii5AMG]RB5AMG_01637_Glucose-1-phosphate_adenylyltransferase	Rbromii5AMG
Org2_Gene1597	core	1352	[Rbromii5AMG]RB5AMG_01639_Glyceraldehyde-3-phosphate_dehydrogenase	Rbromii5AMG
Org2_Gene1598	core	537	[Rbromii5AMG]RB5AMG_01640_Ribonuclease_Y	Rbromii5AMG
Org2_Gene1599	core	938	[Rbromii5AMG]RB5AMG_01641_Ammonia_transporter	Rbromii5AMG
Org2_Gene16	core	3962	[Rbromii5AMG]RB5AMG_00016_hypothetical_protein	Rbromii5AMG
Org2_Gene1600	core	3504	[Rbromii5AMG]RB5AMG_01642_Nitrogen_regulatory_protein_P-II	Rbromii5AMG
Org2_Gene1602	core	461	[Rbromii5AMG]RB5AMG_01644_Long-chain-fatty-acid--CoA_ligase	Rbromii5AMG
Org2_Gene1603	core	981	[Rbromii5AMG]RB5AMG_01645_3-oxoacyl-[acyl-carrier-protein]_synthase_2	Rbromii5AMG
Org2_Gene1605	core	990	[Rbromii5AMG]RB5AMG_01647_Aldehyde-alcohol_dehydrogenase	Rbromii5AMG
Org2_Gene1614	core	2136	[Rbromii5AMG]RB5AMG_01656_Stage_0_sporulation_protein_G	Rbromii5AMG
Org2_Gene1615	core	914	[Rbromii5AMG]RB5AMG_01657_Phosphoribosylamine--glycine_ligase	Rbromii5AMG
Org2_Gene1616	core	1076	[Rbromii5AMG]RB5AMG_01658_Bifunctional_purine_biosynthesis_protein_PurH	Rbromii5AMG
Org2_Gene1617	core	2308	[Rbromii5AMG]RB5AMG_01659_IMP_cyclohydrolase	Rbromii5AMG
Org2_Gene1618	core	2614	[Rbromii5AMG]RB5AMG_01660_Phosphoribosylglycinamide_formyltransferase	Rbromii5AMG
Org2_Gene1619	core	1351	[Rbromii5AMG]RB5AMG_01661_Phosphoribosylformylglycinamidine_cyclo-ligase	Rbromii5AMG
Org2_Gene1620	core	633	[Rbromii5AMG]RB5AMG_01662_Amidophosphoribosyltransferase_precursor	Rbromii5AMG
Org2_Gene1621	core	3072	[Rbromii5AMG]RB5AMG_01663_N5-carboxyaminoimidazole_ribonucleotide_mutase	Rbromii5AMG
Org2_Gene1623	core	1537	[Rbromii5AMG]RB5AMG_01665_Modulator_of_FtsH_protease_HflK	Rbromii5AMG
Org2_Gene1672	core	648	[Rbromii5AMG]RB5AMG_01714_2-iminoacetate_synthase	Rbromii5AMG
Org2_Gene1676	core	951	[Rbromii5AMG]RB5AMG_01718_Putative_virion_core_protein_(lumpy_skin_disease_virus)	Rbromii5AMG
Org2_Gene1678	core	378	[Rbromii5AMG]RB5AMG_01720_Aspartate--tRNA_ligase	Rbromii5AMG
Org2_Gene1679	core	805	[Rbromii5AMG]RB5AMG_01721_Histidine--tRNA_ligase	Rbromii5AMG
Org2_Gene1680	core	3475	[Rbromii5AMG]RB5AMG_01722_Methylglyoxal_synthase	Rbromii5AMG
Org2_Gene1681	core	2226	[Rbromii5AMG]RB5AMG_01723_Cell_division_inhibitor_MinD	Rbromii5AMG
Org2_Gene1685	core	1398	[Rbromii5AMG]RB5AMG_01727_Rod_shape-determining_protein_MreB	Rbromii5AMG
Org2_Gene1686	core	3264	[Rbromii5AMG]RB5AMG_01728_Deoxyuridine_5'-triphosphate_nucleotidohydrolase	Rbromii5AMG
Org2_Gene1691	core	128	[Rbromii5AMG]RB5AMG_01733_DNA_gyrase_subunit_A	Rbromii5AMG
Org2_Gene1693	core	256	[Rbromii5AMG]RB5AMG_01735_DNA_gyrase_subunit_B	Rbromii5AMG
Org2_Gene1695	core	3932	[Rbromii5AMG]RB5AMG_01737_hypothetical_protein	Rbromii5AMG
Org2_Gene1696	core	1180	[Rbromii5AMG]RB5AMG_01738_DNA_replication_and_repair_protein_RecF	Rbromii5AMG
Org2_Gene1697	core	4166	[Rbromii5AMG]RB5AMG_01739_ribosome-associated_protein	Rbromii5AMG
Org2_Gene1698	core	1224	[Rbromii5AMG]RB5AMG_01740_DNA_polymerase_III_subunit_beta	Rbromii5AMG
Org2_Gene1699	core	697	[Rbromii5AMG]RB5AMG_01741_Chromosomal_replication_initiator_protein_DnaA	Rbromii5AMG
Org2_Gene1700	core	4411	[Rbromii5AMG]RB5AMG_01742_50S_ribosomal_protein_L34	Rbromii5AMG
Org2_Gene1701	core	3616	[Rbromii5AMG]RB5AMG_01743_Ribonuclease_P_protein_component	Rbromii5AMG
Org2_Gene1702	core	4063	[Rbromii5AMG]RB5AMG_01744_Putative_membrane_protein_insertion_efficiency_factor	Rbromii5AMG
Org2_Gene1704	core	2011	[Rbromii5AMG]RB5AMG_01746_R3H_domain_protein	Rbromii5AMG
Org2_Gene1705	core	715	[Rbromii5AMG]RB5AMG_01747_tRNA_modification_GTPase_MnmE	Rbromii5AMG
Org2_Gene1706	core	332	[Rbromii5AMG]RB5AMG_01748_Glucose-inhibited_division_protein_A	Rbromii5AMG
Org2_Gene1708	core	1848	[Rbromii5AMG]RB5AMG_01750_Nucleoid_occlusion_protein	Rbromii5AMG
Org2_Gene1709	core	2141	[Rbromii5AMG]RB5AMG_01751_Sporulation_initiation_inhibitor_protein_soj	Rbromii5AMG
Org2_Gene1710	core	1877	[Rbromii5AMG]RB5AMG_01752_putative_chromosome-partitioning_protein_ParB	Rbromii5AMG
Org2_Gene1711	core	885	[Rbromii5AMG]RB5AMG_01753_Serine--tRNA_ligase	Rbromii5AMG
Org2_Gene1715	core	600	[Rbromii5AMG]RB5AMG_01757_Inosine-5'-monophosphate_dehydrogenase	Rbromii5AMG
Org2_Gene1716	core	1533	[Rbromii5AMG]RB5AMG_01758_Glucokinase	Rbromii5AMG
Org2_Gene1720	core	3339	[Rbromii5AMG]RB5AMG_01765_50S_ribosomal_protein_L13	Rbromii5AMG
Org2_Gene1721	core	3447	[Rbromii5AMG]RB5AMG_01766_30S_ribosomal_protein_S9	Rbromii5AMG
Org2_Gene1723	core	1484	[Rbromii5AMG]RB5AMG_01768_Putative_glycosyltransferase_CsbB	Rbromii5AMG
Org2_Gene1726	core	3621	[Rbromii5AMG]RB5AMG_01771_stage_V_sporulation_protein_AE	Rbromii5AMG
Org2_Gene1727	core	1527	[Rbromii5AMG]RB5AMG_01772_putative_ABC_transporter_ATP-binding_protein_YxlF	Rbromii5AMG
Org2_Gene1728	core	2359	[Rbromii5AMG]RB5AMG_01773_gliding_motility-associated_ABC_transporter_permease_protein_GldF	Rbromii5AMG
Org2_Gene1732	core	2595	[Rbromii5AMG]RB5AMG_01777_Uracil_phosphoribosyltransferase	Rbromii5AMG
Org2_Gene1733	core	592	[Rbromii5AMG]RB5AMG_01778_Uracil_transporter	Rbromii5AMG
Org2_Gene1734	core	453	[Rbromii5AMG]RB5AMG_01779_Acetolactate_synthase_large_subunit	Rbromii5AMG
Org2_Gene1735	core	2393	[Rbromii5AMG]RB5AMG_01780_5'-methylthioadenosine/S-adenosylhomocysteine_nucleosidase	Rbromii5AMG
Org2_Gene1736	core	2255	[Rbromii5AMG]RB5AMG_01781_Ribosomal_RNA_small_subunit_methyltransferase_E	Rbromii5AMG
Org2_Gene1737	core	2857	[Rbromii5AMG]RB5AMG_01782_Nicotinamide-nucleotide_amidohydrolase_PncC	Rbromii5AMG
Org2_Gene1738	core	590	[Rbromii5AMG]RB5AMG_01783_4-alpha-glucanotransferase	Rbromii5AMG
Org2_Gene1739	core	171	[Rbromii5AMG]RB5AMG_01784_Glycogen_phosphorylase	Rbromii5AMG
Org2_Gene1742	core	3136	[Rbromii5AMG]RB5AMG_01787_2,3-dimethylmalate_dehydratase_small_subunit	Rbromii5AMG
Org2_Gene1743	core	918	[Rbromii5AMG]RB5AMG_01788_2,3-dimethylmalate_dehydratase_large_subunit	Rbromii5AMG
Org2_Gene1752	core	1730	[Rbromii5AMG]RB5AMG_01801_hypothetical_protein	Rbromii5AMG
Org2_Gene1754	core	3832	[Rbromii5AMG]RB5AMG_01803_hypothetical_protein	Rbromii5AMG
Org2_Gene1755	core	2103	[Rbromii5AMG]RB5AMG_01804_Stage_II_sporulation_protein_AC	Rbromii5AMG
Org2_Gene1767	core	449	[Rbromii5AMG]RB5AMG_01816_Hydroxylamine_reductase	Rbromii5AMG
Org2_Gene1775	core	2184	[Rbromii5AMG]RB5AMG_01824_Phosphate_import_ATP-binding_protein_PstB_3	Rbromii5AMG
Org2_Gene1779	core	717	[Rbromii5AMG]RB5AMG_01828_Tryptophan_synthase_beta_chain	Rbromii5AMG
Org2_Gene1781	core	654	[Rbromii5AMG]RB5AMG_01830_Aspartyl/glutamyl-tRNA(Asn/Gln)_amidotransferase_subunit_B	Rbromii5AMG
Org2_Gene1782	core	628	[Rbromii5AMG]RB5AMG_01831_Glutamyl-tRNA(Gln)_amidotransferase_subunit_A	Rbromii5AMG
Org2_Gene1783	core	3910	[Rbromii5AMG]RB5AMG_01832_Aspartyl/glutamyl-tRNA(Asn/Gln)_amidotransferase_subunit_C	Rbromii5AMG
Org2_Gene1784	core	399	[Rbromii5AMG]RB5AMG_01833_Aspartate--tRNA_ligase	Rbromii5AMG
Org2_Gene1787	core	2781	[Rbromii5AMG]RB5AMG_01836_uracil-DNA_glycosylase,_family_4	Rbromii5AMG
Org2_Gene1788	core	2814	[Rbromii5AMG]RB5AMG_01837_Phosphate_propanoyltransferase	Rbromii5AMG
Org2_Gene1792	core	3338	[Rbromii5AMG]RB5AMG_01841_hypothetical_protein	Rbromii5AMG
Org2_Gene1793	core	510	[Rbromii5AMG]RB5AMG_01842_Phosphoenolpyruvate_carboxykinase_[ATP]	Rbromii5AMG
Org2_Gene1794	core	3530	[Rbromii5AMG]RB5AMG_01843_hypothetical_protein	Rbromii5AMG
Org2_Gene1795	core	2838	[Rbromii5AMG]RB5AMG_01844_50S_ribosomal_protein_L10	Rbromii5AMG
Org2_Gene1796	core	2398	[Rbromii5AMG]RB5AMG_01846_50S_ribosomal_protein_L1	Rbromii5AMG
Org2_Gene1797	core	3352	[Rbromii5AMG]RB5AMG_01847_50S_ribosomal_protein_L11	Rbromii5AMG
Org2_Gene1798	core	2971	[Rbromii5AMG]RB5AMG_01848_hypothetical_protein	Rbromii5AMG
Org2_Gene1800	core	4393	[Rbromii5AMG]RB5AMG_01850_50S_ribosomal_protein_L33	Rbromii5AMG
Org2_Gene1801	core	2067	[Rbromii5AMG]RB5AMG_01851_hypothetical_protein	Rbromii5AMG
Org2_Gene1802	core	1978	[Rbromii5AMG]RB5AMG_01852_Glutamate_racemase	Rbromii5AMG
Org2_Gene1803	core	3199	[Rbromii5AMG]RB5AMG_01853_hypothetical_protein	Rbromii5AMG
Org2_Gene1804	core	408	[Rbromii5AMG]RB5AMG_01854_Arginine--tRNA_ligase	Rbromii5AMG
Org2_Gene1811	core	2088	[Rbromii5AMG]RB5AMG_01861_Acetyl-coenzyme_A_carboxylase_carboxyl_transferase_subunit_alpha	Rbromii5AMG
Org2_Gene1812	core	1826	[Rbromii5AMG]RB5AMG_01862_Acetyl-coenzyme_A_carboxylase_carboxyl_transferase_subunit_beta	Rbromii5AMG
Org2_Gene1813	core	748	[Rbromii5AMG]RB5AMG_01863_2-oxoglutarate_carboxylase_small_subunit	Rbromii5AMG
Org2_Gene1814	core	3377	[Rbromii5AMG]RB5AMG_01864_3-hydroxyacyl-[acyl-carrier-protein]_dehydratase_FabZ	Rbromii5AMG

Org2_Gene1816	core	970	[Rbromii5AMG]RB5AMG_01866_3-oxoacyl-[acyl-carrier-protein]_synthase_2	Rbromii5AMG
Org2_Gene1817	core	2214	[Rbromii5AMG]RB5AMG_01867_3-oxoacyl-[acyl-carrier-protein]_reductase_FabG	Rbromii5AMG
Org2_Gene1818	core	1688	[Rbromii5AMG]RB5AMG_01868_Malonyl_CoA-acyl_carrier_protein_transacylase	Rbromii5AMG
Org2_Gene1819	core	1551	[Rbromii5AMG]RB5AMG_01869_Nitronate_monooxygenase	Rbromii5AMG
Org2_Gene1822	core	957	[Rbromii5AMG]RB5AMG_01872_Homoserine_dehydrogenase	Rbromii5AMG
Org2_Gene1823	core	1015	[Rbromii5AMG]RB5AMG_01873_Aspartokinase	Rbromii5AMG
Org2_Gene1825	core	2925	[Rbromii5AMG]RB5AMG_01875_anaerobic_ribonucleotide_reductase-activating_protein	Rbromii5AMG
Org2_Gene1826	core	215	[Rbromii5AMG]RB5AMG_01876_Anaerobic_ribonucleoside-triphosphate_reductase	Rbromii5AMG
Org2_Gene1831	core	3965	[Rbromii5AMG]RB5AMG_01881_Phosphocarrier_protein_HPr	Rbromii5AMG
Org2_Gene1837	core	718	[Rbromii5AMG]RB5AMG_01887_Glycine--tRNA_ligase	Rbromii5AMG
Org2_Gene1838	core	1050	[Rbromii5AMG]RB5AMG_01888_LL-diaminopimelate_aminotransferase	Rbromii5AMG
Org2_Gene1839	core	492	[Rbromii5AMG]RB5AMG_01889_CTP_synthase	Rbromii5AMG
Org2_Gene1840	core	31	[Rbromii5AMG]RB5AMG_01890_Carbamoyl-phosphate_synthase_large_chain	Rbromii5AMG
Org2_Gene1841	core	1263	[Rbromii5AMG]RB5AMG_01891_Carbamoyl-phosphate_synthase_small_chain	Rbromii5AMG
Org2_Gene1842	core	2810	[Rbromii5AMG]RB5AMG_01892_putative_transcriptional_regulatory_protein_pdtaR	Rbromii5AMG
Org2_Gene1843	core	432	[Rbromii5AMG]RB5AMG_01893_Ammonia_transporter	Rbromii5AMG
Org2_Gene1844	core	246	[Rbromii5AMG]RB5AMG_01894_Glutamine_synthetase	Rbromii5AMG
Org2_Gene1845	core	18	[Rbromii5AMG]RB5AMG_01895_Ferredoxin-dependent_glutamate_synthase_1	Rbromii5AMG
Org2_Gene1846	core	593	[Rbromii5AMG]RB5AMG_01896_Glutamate_synthase_[NADPH]_small_chain	Rbromii5AMG
Org2_Gene1847	core	594	[Rbromii5AMG]RB5AMG_01897_23S_rRNA_(uracil-C(5))-methyltransferase_RlmCD	Rbromii5AMG
Org2_Gene1856	core	199	[Rbromii5AMG]RB5AMG_01908_Formate_acetyltransferase	Rbromii5AMG
Org2_Gene1858	core	2815	[Rbromii5AMG]RB5AMG_01910_hypothetical_protein	Rbromii5AMG
Org2_Gene1860	core	50	[Rbromii5AMG]RB5AMG_01912_Pyruvate-flavodoxin_oxidoreductase	Rbromii5AMG
Org2_Gene1863	core	154	[Rbromii5AMG]RB5AMG_01915_Methionine_synthase	Rbromii5AMG
Org2_Gene1865	core	1832	[Rbromii5AMG]RB5AMG_01917_5,10-methylenetetrahydrofolate_reductase	Rbromii5AMG
Org2_Gene1866	core	727	[Rbromii5AMG]RB5AMG_01918_Magnesium_transporter_MgtE	Rbromii5AMG
Org2_Gene1875	core	95	[Rbromii5AMG]RB5AMG_01927_preprotein_translocase_subunit_SecA	Rbromii5AMG
Org2_Gene1876	core	695	[Rbromii5AMG]RB5AMG_01928_Beta-lactam-inducible_penicillin-binding_protein	Rbromii5AMG
Org2_Gene1877	core	444	[Rbromii5AMG]RB5AMG_01929_Cell_division_protein_FtsW	Rbromii5AMG
Org2_Gene1881	core	1812	[Rbromii5AMG]RB5AMG_01933_Fructose-bisphosphate_aldolase	Rbromii5AMG
Org2_Gene1884	core	811	[Rbromii5AMG]RB5AMG_01936_2-hydroxyglutaryl-CoA_dehydratase,_D-component	Rbromii5AMG
Org2_Gene1885	core	90	[Rbromii5AMG]RB5AMG_01937_2-hydroxyglutaryl-CoA_dehydratase_component_A	Rbromii5AMG
Org2_Gene1887	core	2387	[Rbromii5AMG]RB5AMG_01939_hypothetical_protein	Rbromii5AMG
Org2_Gene1888	core	1419	[Rbromii5AMG]RB5AMG_01940_hypothetical_protein	Rbromii5AMG
Org2_Gene1889	core	2401	[Rbromii5AMG]RB5AMG_01941_hypothetical_protein	Rbromii5AMG
Org2_Gene1890	core	4243	[Rbromii5AMG]RB5AMG_01942_anaerobic_benzoate_catabolism_transcriptional_regulator	Rbromii5AMG
Org2_Gene1895	core	2658	[Rbromii5AMG]RB5AMG_01948_Ribosome_maturation_factor_RimP	Rbromii5AMG
Org2_Gene1896	core	1117	[Rbromii5AMG]RB5AMG_01949_hypothetical_protein	Rbromii5AMG
Org2_Gene1899	core	165	[Rbromii5AMG]RB5AMG_01952_Translation_initiation_factor_IF-2	Rbromii5AMG
Org2_Gene19	core	746	[Rbromii5AMG]RB5AMG_00020_Maltose-binding_periplasmic_proteins/domains	Rbromii5AMG
Org2_Gene1900	core	3541	[Rbromii5AMG]RB5AMG_01953_Ribosome-binding_factor_A	Rbromii5AMG
Org2_Gene1908	core	3024	[Rbromii5AMG]RB5AMG_01961_Flavoredoxin	Rbromii5AMG
Org2_Gene1911	core	983	[Rbromii5AMG]RB5AMG_01964_Argininosuccinate_synthase	Rbromii5AMG
Org2_Gene1912	core	719	[Rbromii5AMG]RB5AMG_01965_Argininosuccinate_lyase_1	Rbromii5AMG
Org2_Gene1913	core	1349	[Rbromii5AMG]RB5AMG_01966_N-acetyl-gamma-glutamyl-phosphate_reductase	Rbromii5AMG
Org2_Gene1914	core	973	[Rbromii5AMG]RB5AMG_01967_Arginine_biosynthesis_bifunctional_protein_ArgJ	Rbromii5AMG
Org2_Gene1915	core	1865	[Rbromii5AMG]RB5AMG_01968_Acetylglutamate_kinase	Rbromii5AMG
Org2_Gene1916	core	3359	[Rbromii5AMG]RB5AMG_01969_Acetyltransferase_YpeA	Rbromii5AMG
Org2_Gene1917	core	1088	[Rbromii5AMG]RB5AMG_01970_Acetylornithine_aminotransferase	Rbromii5AMG
Org2_Gene1918	core	1605	[Rbromii5AMG]RB5AMG_01971_Ornithine_carbamoyltransferase	Rbromii5AMG
Org2_Gene192	core	1282	[Rbromii5AMG]RB5AMG_00202_Aspartate_carbamoyltransferase_catalytic_chain	Rbromii5AMG
Org2_Gene1926	core	1958	[Rbromii5AMG]RB5AMG_01979_antiporter_inner_membrane_protein	Rbromii5AMG
Org2_Gene1928	core	1591	[Rbromii5AMG]RB5AMG_01981_Ribosomal_protein_L11_methyltransferase	Rbromii5AMG
Org2_Gene1929	core	1153	[Rbromii5AMG]RB5AMG_01982_Chaperone_protein_DnaJ	Rbromii5AMG
Org2_Gene1930	core	344	[Rbromii5AMG]RB5AMG_01983_Heat_shock_protein_70	Rbromii5AMG
Org2_Gene1935	core	2521	[Rbromii5AMG]RB5AMG_01989_LexA_repressor	Rbromii5AMG
Org2_Gene1938	core	2878	[Rbromii5AMG]RB5AMG_01992_Fumarate_hydratase_class_I,_anaerobic	Rbromii5AMG
Org2_Gene1939	core	1863	[Rbromii5AMG]RB5AMG_01993_L(+)-tartrate_dehydratase_subunit_alpha	Rbromii5AMG
Org2_Gene1940	core	2424	[Rbromii5AMG]RB5AMG_01994_Staphylococcal_respiratory_response_protein_A	Rbromii5AMG
Org2_Gene1941	core	525	[Rbromii5AMG]RB5AMG_01995_Alkaline_phosphatase_synthesis_sensor_protein_PhoR	Rbromii5AMG
Org2_Gene1944	core	4378	[Rbromii5AMG]RB5AMG_01998_hypothetical_protein	Rbromii5AMG
Org2_Gene1945	core	3981	[Rbromii5AMG]RB5AMG_01999_30S_ribosomal_protein_S20	Rbromii5AMG
Org2_Gene1946	core	2275	[Rbromii5AMG]RB5AMG_02000_NAD-dependent_protein_deacetylase	Rbromii5AMG
Org2_Gene1947	core	2133	[Rbromii5AMG]RB5AMG_02001_Tryptophan_synthase_alpha_chain	Rbromii5AMG
Org2_Gene1948	core	1062	[Rbromii5AMG]RB5AMG_02002_Tryptophan_synthase_beta_chain	Rbromii5AMG
Org2_Gene1953	core	758	[Rbromii5AMG]RB5AMG_02008_UDP-N-acetylmuramoyl-tripeptide--D-alanyl-D-alanine_ligase	Rbromii5AMG
Org2_Gene1954	core	1289	[Rbromii5AMG]RB5AMG_02009_D-alanine--D-alanine_ligase	Rbromii5AMG
Org2_Gene197	core	1801	[Rbromii5AMG]RB5AMG_00207_4-diphosphocytidyl-2-C-methyl-D-erythritol_kinase	Rbromii5AMG
Org2_Gene1971	core	330	[Rbromii5AMG]RB5AMG_02026_High_temperature_protein_G	Rbromii5AMG
Org2_Gene1975	core	2164	[Rbromii5AMG]RB5AMG_02030_Dihydroorotate_oxidase_B,_electron_transfer_subunit	Rbromii5AMG
Org2_Gene1976	core	1658	[Rbromii5AMG]RB5AMG_02031_Dihydroorotate_dehydrogenase_B_(NAD(+)),_catalytic_subunit	Rbromii5AMG
Org2_Gene1978	core	319	[Rbromii5AMG]RB5AMG_02033_Alpha-xylosidase	Rbromii5AMG
Org2_Gene198	core	1075	[Rbromii5AMG]RB5AMG_00208_Putative_N-acetyl-LL-diaminopimelate_aminotransferase	Rbromii5AMG
Org2_Gene1980	core	578	[Rbromii5AMG]RB5AMG_02035_Phosphomannomutase/phosphoglucomutase	Rbromii5AMG
Org2_Gene1981	core	3957	[Rbromii5AMG]RB5AMG_02036_hypothetical_protein	Rbromii5AMG
Org2_Gene1982	core	3607	[Rbromii5AMG]RB5AMG_02037_hypothetical_protein	Rbromii5AMG
Org2_Gene1984	core	317	[Rbromii5AMG]RB5AMG_02039_putative_ABC_transporter_ATP-binding_protein_YheS	Rbromii5AMG
Org2_Gene199	core	3130	[Rbromii5AMG]RB5AMG_00209_Leucine-responsive_regulatory_protein	Rbromii5AMG
Org2_Gene20	core	1769	[Rbromii5AMG]RB5AMG_00021_L-arabinose_transport_system_permease_protein_AraP	Rbromii5AMG
Org2_Gene2008	core	2169	[Rbromii5AMG]RB5AMG_02065_Exodeoxyribonuclease	Rbromii5AMG
Org2_Gene201	core	313	[Rbromii5AMG]RB5AMG_00211_Glutamine-dependent_NAD(+)_synthetase	Rbromii5AMG
Org2_Gene2010	core	2912	[Rbromii5AMG]RB5AMG_02067_NADH_peroxidase	Rbromii5AMG
Org2_Gene2028	core	2205	[Rbromii5AMG]RB5AMG_02087_Teichoic_acids_export_ATP-binding_protein_TagH	Rbromii5AMG
Org2_Gene2029	core	1968	[Rbromii5AMG]RB5AMG_02088_Teichoic_acid_translocation_permease_protein_TagG	Rbromii5AMG
Org2_Gene203	core	2431	[Rbromii5AMG]RB5AMG_00213_Putative_undecaprenyl-phosphate_N-acetylgalactosaminyl_1-phosphate_transferase	Rbromii5AMG
Org2_Gene2030	core	1251	[Rbromii5AMG]RB5AMG_02089_Spermidine/putrescine-binding_periplasmic_protein_precursor	Rbromii5AMG
Org2_Gene2031	core	1988	[Rbromii5AMG]RB5AMG_02090_Inner_membrane_ABC_transporter_permease_protein_YdcV	Rbromii5AMG
Org2_Gene2032	core	2069	[Rbromii5AMG]RB5AMG_02091_Putrescine_transport_system_permease_protein_PotH	Rbromii5AMG
Org2_Gene2033	core	659	[Rbromii5AMG]RB5AMG_02092_Spermidine/putrescine_import_ATP-binding_protein_PotA	Rbromii5AMG
Org2_Gene2038	core	210	[Rbromii5AMG]RB5AMG_02097_Exodeoxyribonuclease_V_alpha_chain	Rbromii5AMG
Org2_Gene2040	core	817	[Rbromii5AMG]RB5AMG_02099_putative_bifunctional_UDP-N-acetylmuramoylalanyl-D-glutamate--2,6-diaminopimelate_ligase/UDP-N-acetylmuramoyl-tr	Rbromii5AMG
Org2_Gene2043	core	937	[Rbromii5AMG]RB5AMG_02102_GTP-binding_protein_HflX	Rbromii5AMG
Org2_Gene2046	core	2452	[Rbromii5AMG]RB5AMG_02105_Bifunctional_protein_GlmU	Rbromii5AMG
Org2_Gene2049	core	3632	[Rbromii5AMG]RB5AMG_02111_Nucleoid-associated_protein	Rbromii5AMG
Org2_Gene205	core	1512	[Rbromii5AMG]RB5AMG_00216_Glycerol-3-phosphate_dehydrogenase_[NAD(P)+]	Rbromii5AMG
Org2_Gene2050	core	2679	[Rbromii5AMG]RB5AMG_02112_Recombination_protein_RecR	Rbromii5AMG
Org2_Gene2051	core	3221	[Rbromii5AMG]RB5AMG_02113_SsrA-binding_protein	Rbromii5AMG
Org2_Gene2071	core	1118	[Rbromii5AMG]RB5AMG_02134_hypothetical_protein	Rbromii5AMG
Org2_Gene2075	core	3322	[Rbromii5AMG]RB5AMG_02138_Cupin_domain_protein	Rbromii5AMG
Org2_Gene2077	core	3228	[Rbromii5AMG]RB5AMG_02140_Transcriptional_repressor_NrdR	Rbromii5AMG
Org2_Gene2079	core	265	[Rbromii5AMG]RB5AMG_02142_ATP-dependent_DNA_helicase_RecG	Rbromii5AMG
Org2_Gene2080	core	470	[Rbromii5AMG]RB5AMG_02143_dihydroxyacetone_kinase	Rbromii5AMG
Org2_Gene2081	core	2126	[Rbromii5AMG]RB5AMG_02144_metallophosphoesterase,_MG_246/_family	Rbromii5AMG
Org2_Gene2085	core	354	[Rbromii5AMG]RB5AMG_02148_Asparagine_synthetase_[glutamine-hydrolyzing]_3	Rbromii5AMG
Org2_Gene2087	core	3312	[Rbromii5AMG]RB5AMG_02150_Large-conductance_mechanosensitive_channel	Rbromii5AMG
Org2_Gene2088	core	979	[Rbromii5AMG]RB5AMG_02151_Tyrosine--tRNA_ligase	Rbromii5AMG
Org2_Gene2090	core	316	[Rbromii5AMG]RB5AMG_02153_putative_radical_SAM_protein_YgiQ	Rbromii5AMG
Org2_Gene2092	core	1521	[Rbromii5AMG]RB5AMG_02155_Undecaprenyl-diphosphatase	Rbromii5AMG
Org2_Gene2093	core	1989	[Rbromii5AMG]RB5AMG_02156_Translocation-enhancing_protein_TepA	Rbromii5AMG
Org2_Gene2094	core	830	[Rbromii5AMG]RB5AMG_02157_Magnesium_and_cobalt_efflux_protein_CorC	Rbromii5AMG
Org2_Gene2096	core	2718	[Rbromii5AMG]RB5AMG_02159_Ribonuclease_M5	Rbromii5AMG
Org2_Gene2099	core	2935	[Rbromii5AMG]RB5AMG_02162_Pur_operon_repressor	Rbromii5AMG
Org2_Gene21	core	1768	[Rbromii5AMG]RB5AMG_00022_L-arabinose_transport_system_permease_protein_AraQ	Rbromii5AMG
Org2_Gene210	core	3967	[Rbromii5AMG]RB5AMG_00221_hypothetical_protein	Rbromii5AMG
Org2_Gene2100	core	3658	[Rbromii5AMG]RB5AMG_02163_HIT-like_protein	Rbromii5AMG
Org2_Gene2102	core	819	[Rbromii5AMG]RB5AMG_02165_GTP-binding_protein_EngA	Rbromii5AMG
Org2_Gene2104	core	4271	[Rbromii5AMG]RB5AMG_02167_50S_ribosomal_protein_L32	Rbromii5AMG
Org2_Gene2105	core	3074	[Rbromii5AMG]RB5AMG_02168_hypothetical_protein	Rbromii5AMG
Org2_Gene211	core	487	[Rbromii5AMG]RB5AMG_00222_Stress_protein_H5	Rbromii5AMG
Org2_Gene2112	core	1618	[Rbromii5AMG]RB5AMG_02175_30S_ribosomal_protein_S1	Rbromii5AMG
Org2_Gene2114	core	1871	[Rbromii5AMG]RB5AMG_02177_HAD_phosphoserine_phosphatase-like_hydrolase,_family_IB	Rbromii5AMG
Org2_Gene212	core	3886	[Rbromii5AMG]RB5AMG_00223_co-chaperonin_GroES	Rbromii5AMG

Org2_Gene2121	core	1660	[Rbromii5AMG]RB5AMG_02184_Daunorubicin/doxorubicin_resistance_ATP-binding_protein_DrrA	Rbromii5AMG
Org2_Gene2129	core	3710	[Rbromii5AMG]RB5AMG_02192_hypothetical_protein	Rbromii5AMG
Org2_Gene213	core	480	[Rbromii5AMG]RB5AMG_00224_Serine/threonine-protein_kinase_PrkC	Rbromii5AMG
Org2_Gene2135	core	2252	[Rbromii5AMG]RB5AMG_02206_Putative_HAD-hydrolase_YfnB	Rbromii5AMG
Org2_Gene2136	core	2513	[Rbromii5AMG]RB5AMG_02207_UV-endonuclease	Rbromii5AMG
Org2_Gene2139	core	363	[Rbromii5AMG]RB5AMG_02210_Elongation_factor_4	Rbromii5AMG
Org2_Gene2141	core	164	[Rbromii5AMG]RB5AMG_02212_Asparagine_synthetase_[glutamine-hydrolyzing]_1	Rbromii5AMG
Org2_Gene2144	core	2888	[Rbromii5AMG]RB5AMG_02215_ADP-ribose_pyrophosphatase	Rbromii5AMG
Org2_Gene2149	core	4333	[Rbromii5AMG]RB5AMG_02220_hypothetical_protein	Rbromii5AMG
Org2_Gene215	core	1753	[Rbromii5AMG]RB5AMG_00227_Putative_ribosome_biogenesis_GTPase_RsgA	Rbromii5AMG
Org2_Gene216	core	238	[Rbromii5AMG]RB5AMG_00228_Serine/threonine-protein_kinase_PrkC	Rbromii5AMG
Org2_Gene2161	core	1875	[Rbromii5AMG]RB5AMG_02232_Sugar_phosphatase_YidA	Rbromii5AMG
Org2_Gene2163	core	714	[Rbromii5AMG]RB5AMG_02234_2-methylcitrate_synthase	Rbromii5AMG
Org2_Gene2169	core	1017	[Rbromii5AMG]RB5AMG_02240_UDP-4-amino-4-deoxy-L-arabinose--oxoglutarate_aminotransferase	Rbromii5AMG
Org2_Gene217	core	2278	[Rbromii5AMG]RB5AMG_00229_Serine/threonine_phosphatase_stp	Rbromii5AMG
Org2_Gene2170	core	3922	[Rbromii5AMG]RB5AMG_02241_14_kDa_transcription_factor	Rbromii5AMG
Org2_Gene218	core	1357	[Rbromii5AMG]RB5AMG_00230_putative_dual-specificity_RNA_methyltransferase_RlmN	Rbromii5AMG
Org2_Gene22	core	174	[Rbromii5AMG]RB5AMG_00023_Heat_shock_protein_HSP1	Rbromii5AMG
Org2_Gene220	core	2364	[Rbromii5AMG]RB5AMG_00232_Putative_neutral_zinc_metallopeptidase	Rbromii5AMG
Org2_Gene221	core	1641	[Rbromii5AMG]RB5AMG_00233_Methionyl-tRNA_formyltransferase	Rbromii5AMG
Org2_Gene222	core	3150	[Rbromii5AMG]RB5AMG_00234_Peptide_deformylase_1	Rbromii5AMG
Org2_Gene223	core	144	[Rbromii5AMG]RB5AMG_00235_Primosomal_protein_N'	Rbromii5AMG
Org2_Gene224	core	4190	[Rbromii5AMG]RB5AMG_00236_DNA-directed_RNA_polymerase_subunit_omega	Rbromii5AMG
Org2_Gene226	core	3994	[Rbromii5AMG]RB5AMG_00238_hypothetical_protein	Rbromii5AMG
Org2_Gene227	core	1747	[Rbromii5AMG]RB5AMG_00239_hypothetical_protein	Rbromii5AMG
Org2_Gene228	core	2241	[Rbromii5AMG]RB5AMG_00240_putative_transcriptional_regulatory_protein_YebC	Rbromii5AMG
Org2_Gene230	core	279	[Rbromii5AMG]RB5AMG_00242_hypothetical_protein	Rbromii5AMG
Org2_Gene231	core	2586	[Rbromii5AMG]RB5AMG_00243_1-acyl-sn-glycerol-3-phosphate_acyltransferase	Rbromii5AMG
Org2_Gene232	core	2482	[Rbromii5AMG]RB5AMG_00244_Cytidylate_kinase	Rbromii5AMG
Org2_Gene233	core	952	[Rbromii5AMG]RB5AMG_00245_tricarballylate_dehydrogenase	Rbromii5AMG
Org2_Gene234	core	1715	[Rbromii5AMG]RB5AMG_00246_MurPQ_operon_repressor	Rbromii5AMG
Org2_Gene235	core	2620	[Rbromii5AMG]RB5AMG_00247_Imidazole_glycerol_phosphate_synthase_subunit_HisH_1	Rbromii5AMG
Org2_Gene236	core	2177	[Rbromii5AMG]RB5AMG_00248_Imidazole_glycerol_phosphate_synthase_subunit_HisF	Rbromii5AMG
Org2_Gene238	core	1964	[Rbromii5AMG]RB5AMG_00250_enhanced_serine_sensitivity_protein_SseB	Rbromii5AMG
Org2_Gene243	core	3444	[Rbromii5AMG]RB5AMG_00255_Mini-ribonuclease_3	Rbromii5AMG
Org2_Gene244	core	909	[Rbromii5AMG]RB5AMG_00256_Spo0B-associated_GTP-binding_protein	Rbromii5AMG
Org2_Gene245	core	3887	[Rbromii5AMG]RB5AMG_00257_50S_ribosomal_protein_L27	Rbromii5AMG
Org2_Gene247	core	3807	[Rbromii5AMG]RB5AMG_00259_50S_ribosomal_protein_L21	Rbromii5AMG
Org2_Gene248	core	1866	[Rbromii5AMG]RB5AMG_00260_Bifunctional_protein_FolD_protein	Rbromii5AMG
Org2_Gene249	core	892	[Rbromii5AMG]RB5AMG_00261_Putative_zinc_protease_AlbF	Rbromii5AMG
Org2_Gene256	core	1288	[Rbromii5AMG]RB5AMG_00268_Aldose_1-epimerase_precursor	Rbromii5AMG
Org2_Gene257	core	572	[Rbromii5AMG]RB5AMG_00269_galactose-1-phosphate_uridylyltransferase	Rbromii5AMG
Org2_Gene258	core	1440	[Rbromii5AMG]RB5AMG_00270_UDP-glucose_4-epimerase	Rbromii5AMG
Org2_Gene26	core	2028	[Rbromii5AMG]RB5AMG_00027_tRNA_pseudouridine_synthase_A	Rbromii5AMG
Org2_Gene27	core	1998	[Rbromii5AMG]RB5AMG_00028_Energy-coupling_factor_transporter_transmembrane_protein_EcfT	Rbromii5AMG
Org2_Gene28	core	1805	[Rbromii5AMG]RB5AMG_00029_Energy-coupling_factor_transporter_ATP-binding_protein_EcfA2	Rbromii5AMG
Org2_Gene29	core	1885	[Rbromii5AMG]RB5AMG_00030_Energy-coupling_factor_transporter_ATP-binding_protein_EcfA1	Rbromii5AMG
Org2_Gene30	core	1821	[Rbromii5AMG]RB5AMG_00031_Ribosomal_RNA_large_subunit_methyltransferase_K	Rbromii5AMG
Org2_Gene31	core	761	[Rbromii5AMG]RB5AMG_00032_Phosphoglucosamine_mutase	Rbromii5AMG
Org2_Gene313	core	446	[Rbromii5AMG]RB5AMG_00326_2-isopropylmalate_synthase	Rbromii5AMG
Org2_Gene317	core	243	[Rbromii5AMG]RB5AMG_00330_Membrane_protein_YdfJ	Rbromii5AMG
Org2_Gene32	core	1247	[Rbromii5AMG]RB5AMG_00033_Holliday_junction_ATP-dependent_DNA_helicase_RuvB	Rbromii5AMG
Org2_Gene321	core	4046	[Rbromii5AMG]RB5AMG_00334_transcriptional_regulator,_AbrB_family	Rbromii5AMG
Org2_Gene322	core	2757	[Rbromii5AMG]RB5AMG_00335_Spore_maturation_protein_A	Rbromii5AMG
Org2_Gene323	core	3055	[Rbromii5AMG]RB5AMG_00336_Spore_maturation_protein_B	Rbromii5AMG
Org2_Gene33	core	2683	[Rbromii5AMG]RB5AMG_00034_Holliday_junction_ATP-dependent_DNA_helicase_RuvA	Rbromii5AMG
Org2_Gene34	core	3035	[Rbromii5AMG]RB5AMG_00035_Crossover_junction_endodeoxyribonuclease_RuvC	Rbromii5AMG
Org2_Gene36	core	1408	[Rbromii5AMG]RB5AMG_00037_hypothetical_protein	Rbromii5AMG
Org2_Gene38	core	435	[Rbromii5AMG]RB5AMG_00039_Neopullulanase	Rbromii5AMG
Org2_Gene39	core	251	[Rbromii5AMG]RB5AMG_00040_Glycogen_debranching_enzyme	Rbromii5AMG
Org2_Gene407	core	310	[Rbromii5AMG]RB5AMG_00420_Lysine--tRNA_ligase	Rbromii5AMG
Org2_Gene408	core	3129	[Rbromii5AMG]RB5AMG_00421_Transcript_cleavage_factor_GreA	Rbromii5AMG
Org2_Gene412	core	2132	[Rbromii5AMG]RB5AMG_00425_Cell_division_ATP-binding_protein_FtsE	Rbromii5AMG
Org2_Gene413	core	1258	[Rbromii5AMG]RB5AMG_00426_Sugar_diacid_regulator	Rbromii5AMG
Org2_Gene414	core	1217	[Rbromii5AMG]RB5AMG_00427_Trehalose_import_ATP-binding_protein_SugC	Rbromii5AMG
Org2_Gene418	core	3496	[Rbromii5AMG]RB5AMG_00431_Fluoroacetyl-CoA_thioesterase	Rbromii5AMG
Org2_Gene419	core	1238	[Rbromii5AMG]RB5AMG_00432_Ribosome-binding_ATPase_YchF	Rbromii5AMG
Org2_Gene421	core	1554	[Rbromii5AMG]RB5AMG_00434_1-deoxy-D-xylulose-5-phosphate_synthase	Rbromii5AMG
Org2_Gene422	core	1872	[Rbromii5AMG]RB5AMG_00435_Transketolase	Rbromii5AMG
Org2_Gene423	core	2140	[Rbromii5AMG]RB5AMG_00436_Type_III_pantothenate_kinase	Rbromii5AMG
Org2_Gene426	core	37	[Rbromii5AMG]RB5AMG_00439_Phosphoribosylformylglycinamidine_synthase_2	Rbromii5AMG
Org2_Gene428	core	1555	[Rbromii5AMG]RB5AMG_00441_Putative_2-hydroxyacid_dehydrogenase	Rbromii5AMG
Org2_Gene434	core	675	[Rbromii5AMG]RB5AMG_00449_putative_M18_family_aminopeptidase_1	Rbromii5AMG
Org2_Gene437	core	1337	[Rbromii5AMG]RB5AMG_00452_t(6)A37_threonylcarbamoyladenosine_biosynthesis_protein_RimN	Rbromii5AMG
Org2_Gene438	core	1276	[Rbromii5AMG]RB5AMG_00453_Peptide_chain_release_factor_1	Rbromii5AMG
Org2_Gene439	core	4312	[Rbromii5AMG]RB5AMG_00454_hypothetical_protein	Rbromii5AMG
Org2_Gene441	core	1671	[Rbromii5AMG]RB5AMG_00456_NTE_family_protein_RssA	Rbromii5AMG
Org2_Gene442	core	416	[Rbromii5AMG]RB5AMG_00457_putative_membrane_protein	Rbromii5AMG
Org2_Gene443	core	189	[Rbromii5AMG]RB5AMG_00458_1,4-alpha-glucan_branching_enzyme_GlgB	Rbromii5AMG
Org2_Gene453	core	3350	[Rbromii5AMG]RB5AMG_00469_Putative_Holliday_junction_resolvase	Rbromii5AMG
Org2_Gene457	core	44	[Rbromii5AMG]RB5AMG_00473_DNA-directed_RNA_polymerase_subunit_beta'	Rbromii5AMG
Org2_Gene458	core	40	[Rbromii5AMG]RB5AMG_00474_DNA-directed_RNA_polymerase_subunit_beta	Rbromii5AMG
Org2_Gene459	core	1129	[Rbromii5AMG]RB5AMG_00475_tRNA_2-thiocytidine_biosynthesis_protein_TtcA	Rbromii5AMG
Org2_Gene491	core	4015	[Rbromii5AMG]RB5AMG_00511_preprotein_translocase_subunit_SecG	Rbromii5AMG
Org2_Gene492	core	203	[Rbromii5AMG]RB5AMG_00512_DNA_gyrase_subunit_A	Rbromii5AMG
Org2_Gene493	core	282	[Rbromii5AMG]RB5AMG_00513_DNA_gyrase_subunit_B	Rbromii5AMG
Org2_Gene494	core	290	[Rbromii5AMG]RB5AMG_00514_Excinuclease_ABC_subunit_B	Rbromii5AMG
Org2_Gene495	core	2348	[Rbromii5AMG]RB5AMG_00515_DNA_repair_protein_RadC	Rbromii5AMG
Org2_Gene496	core	2443	[Rbromii5AMG]RB5AMG_00516_Orotate_phosphoribosyltransferase	Rbromii5AMG
Org2_Gene499	core	898	[Rbromii5AMG]RB5AMG_00519_DNA_polymerase_IV	Rbromii5AMG
Org2_Gene500	core	4043	[Rbromii5AMG]RB5AMG_00520_hypothetical_protein	Rbromii5AMG
Org2_Gene506	core	2038	[Rbromii5AMG]RB5AMG_00526_Pyrroline-5-carboxylate_reductase	Rbromii5AMG
Org2_Gene524	core	323	[Rbromii5AMG]RB5AMG_00546_putative_ABC_transporter_ATP-binding_protein	Rbromii5AMG
Org2_Gene525	core	398	[Rbromii5AMG]RB5AMG_00547_putative_ABC_transporter_ATP-binding_protein	Rbromii5AMG
Org2_Gene529	core	2145	[Rbromii5AMG]RB5AMG_00551_putative_oxidoreductase	Rbromii5AMG
Org2_Gene535	core	729	[Rbromii5AMG]RB5AMG_00557_putative_RNA_methyltransferase	Rbromii5AMG
Org2_Gene536	core	710	[Rbromii5AMG]RB5AMG_00558_UDP-N-acetylmuramate--L-alanine_ligase	Rbromii5AMG
Org2_Gene540	core	359	[Rbromii5AMG]RB5AMG_00562_Tyrosine_phosphorylated_protein_A	Rbromii5AMG
Org2_Gene541	core	307	[Rbromii5AMG]RB5AMG_00563_2,3-dimethylmalate_dehydratase_large_subunit	Rbromii5AMG
Org2_Gene542	core	1011	[Rbromii5AMG]RB5AMG_00564_Isocitrate_dehydrogenase_[NADP]	Rbromii5AMG
Org2_Gene543	core	2589	[Rbromii5AMG]RB5AMG_00565_Redox-sensing_transcriptional_repressor_rex	Rbromii5AMG
Org2_Gene545	core	92	[Rbromii5AMG]RB5AMG_00567_Pullulanase_precursor	Rbromii5AMG
Org2_Gene548	core	3557	[Rbromii5AMG]RB5AMG_00570_hypothetical_protein	Rbromii5AMG
Org2_Gene549	core	1216	[Rbromii5AMG]RB5AMG_00571_RNA_polymerase_sigma_factor_RpoD	Rbromii5AMG
Org2_Gene550	core	383	[Rbromii5AMG]RB5AMG_00572_DNA_primase	Rbromii5AMG
Org2_Gene551	core	2603	[Rbromii5AMG]RB5AMG_00573_IMPACT_family_member_YigZ	Rbromii5AMG
Org2_Gene552	core	4201	[Rbromii5AMG]RB5AMG_00574_50S_ribosomal_protein_L31	Rbromii5AMG
Org2_Gene554	core	298	[Rbromii5AMG]RB5AMG_00576_Threonine--tRNA_ligase_1	Rbromii5AMG
Org2_Gene562	core	3992	[Rbromii5AMG]RB5AMG_00585_hypothetical_protein	Rbromii5AMG
Org2_Gene564	core	99	[Rbromii5AMG]RB5AMG_00587_Excinuclease_ABC_subunit_A	Rbromii5AMG
Org2_Gene565	core	270	[Rbromii5AMG]RB5AMG_00588_ATP-dependent_zinc_metalloprotease_FtsH	Rbromii5AMG
Org2_Gene566	core	2937	[Rbromii5AMG]RB5AMG_00589_Hypoxanthine-guanine_phosphoribosyltransferase	Rbromii5AMG
Org2_Gene568	core	725	[Rbromii5AMG]RB5AMG_00591_Replicative_DNA_helicase	Rbromii5AMG
Org2_Gene569	core	3258	[Rbromii5AMG]RB5AMG_00592_hypothetical_protein	Rbromii5AMG
Org2_Gene571	core	320	[Rbromii5AMG]RB5AMG_00594_Ribonuclease_J_1	Rbromii5AMG
Org2_Gene572	core	3091	[Rbromii5AMG]RB5AMG_00595_Ribonuclease_HI	Rbromii5AMG
Org2_Gene573	core	1027	[Rbromii5AMG]RB5AMG_00596_Cysteine_desulfurase	Rbromii5AMG
Org2_Gene574	core	3330	[Rbromii5AMG]RB5AMG_00597_NifU-like_protein	Rbromii5AMG
Org2_Gene576	core	1285	[Rbromii5AMG]RB5AMG_00599_3-isopropylmalate_dehydrogenase	Rbromii5AMG
Org2_Gene577	core	2610	[Rbromii5AMG]RB5AMG_00600_Alpha-ribazole_phosphatase	Rbromii5AMG
Org2_Gene579	core	3646	[Rbromii5AMG]RB5AMG_00602_mRNA_interferase_MazF	Rbromii5AMG
Org2_Gene586	core	1316	[Rbromii5AMG]RB5AMG_00610_Phenylalanine--tRNA_ligase_alpha_subunit	Rbromii5AMG

Org2_Gene587	core	169	[Rbromii5AMG]RB5AMG_00611_Phenylalanine--tRNA_ligase_beta_subunit	Rbromii5AMG
Org2_Gene588	core	4032	[Rbromii5AMG]RB5AMG_00612_hypothetical_protein	Rbromii5AMG
Org2_Gene589	core	3817	[Rbromii5AMG]RB5AMG_00613_hypothetical_protein	Rbromii5AMG
Org2_Gene590	core	137	[Rbromii5AMG]RB5AMG_00614_putative_copper-importing_P-type_ATPase_A	Rbromii5AMG
Org2_Gene591	core	3823	[Rbromii5AMG]RB5AMG_00615_Copper-sensitive_operon_repressor	Rbromii5AMG
Org2_Gene594	core	4065	[Rbromii5AMG]RB5AMG_00618_hypothetical_protein	Rbromii5AMG
Org2_Gene595	core	3056	[Rbromii5AMG]RB5AMG_00619_Helix-destabilizing_protein	Rbromii5AMG
Org2_Gene596	core	3849	[Rbromii5AMG]RB5AMG_00620_30S_ribosomal_protein_S6	Rbromii5AMG
Org2_Gene599	core	852	[Rbromii5AMG]RB5AMG_00623_Methionine_gamma-lyase	Rbromii5AMG
Org2_Gene602	core	2395	[Rbromii5AMG]RB5AMG_00626_RNA_polymerase_sigma-28_factor_precursor	Rbromii5AMG
Org2_Gene610	core	2897	[Rbromii5AMG]RB5AMG_00635_N-acetylmuramoyl-L-alanine_amidase_CwlD	Rbromii5AMG
Org2_Gene611	core	2500	[Rbromii5AMG]RB5AMG_00636_hypothetical_protein	Rbromii5AMG
Org2_Gene613	core	818	[Rbromii5AMG]RB5AMG_00638_Ribosomal_RNA_small_subunit_methyltransferase_F	Rbromii5AMG
Org2_Gene614	core	1855	[Rbromii5AMG]RB5AMG_00639_Ribosomal_RNA_small_subunit_methyltransferase_A	Rbromii5AMG
Org2_Gene616	core	124	[Rbromii5AMG]RB5AMG_00642_Calcium-transporting_ATPase	Rbromii5AMG
Org2_Gene619	core	2927	[Rbromii5AMG]RB5AMG_00645_Inorganic_pyrophosphatase	Rbromii5AMG
Org2_Gene621	core	1923	[Rbromii5AMG]RB5AMG_00647_Laccase_domain_protein	Rbromii5AMG
Org2_Gene622	core	1509	[Rbromii5AMG]RB5AMG_00648_Ribosomal_large_subunit_pseudouridine_synthase_C	Rbromii5AMG
Org2_Gene625	core	603	[Rbromii5AMG]RB5AMG_00651_Stage_IV_sporulation_protein_A	Rbromii5AMG
Org2_Gene627	core	956	[Rbromii5AMG]RB5AMG_00653_Glutaconyl-CoA_decarboxylase_subunit_beta	Rbromii5AMG
Org2_Gene630	core	886	[Rbromii5AMG]RB5AMG_00656_Putative_aminotransferase/MSMEI_6121	Rbromii5AMG
Org2_Gene631	core	663	[Rbromii5AMG]RB5AMG_00657_preprotein_translocase_subunit_SecD	Rbromii5AMG
Org2_Gene632	core	1378	[Rbromii5AMG]RB5AMG_00658_preprotein_translocase_subunit_SecF	Rbromii5AMG
Org2_Gene640	core	622	[Rbromii5AMG]RB5AMG_00666_Spore_germination_protein_B1	Rbromii5AMG
Org2_Gene643	core	1495	[Rbromii5AMG]RB5AMG_00669_HPr_kinase/phosphorylase	Rbromii5AMG
Org2_Gene644	core	1679	[Rbromii5AMG]RB5AMG_00670_UDP-N-acetylenolpyruvoylglucosamine_reductase	Rbromii5AMG
Org2_Gene645	core	1818	[Rbromii5AMG]RB5AMG_00671_glmZ(sRNA)-inactivating_NTPase	Rbromii5AMG
Org2_Gene646	core	1590	[Rbromii5AMG]RB5AMG_00672_Putative_sporulation_transcription_regulator_WhiA	Rbromii5AMG
Org2_Gene648	core	1774	[Rbromii5AMG]RB5AMG_00674_Farnesyl_diphosphate_synthase	Rbromii5AMG
Org2_Gene649	core	340	[Rbromii5AMG]RB5AMG_00675_1-deoxy-D-xylulose-5-phosphate_synthase	Rbromii5AMG
Org2_Gene650	core	2015	[Rbromii5AMG]RB5AMG_00676_Hemolysin_A	Rbromii5AMG
Org2_Gene653	core	457	[Rbromii5AMG]RB5AMG_00679_Recombination_protein_N	Rbromii5AMG
Org2_Gene655	core	212	[Rbromii5AMG]RB5AMG_00681_GTP_pyrophosphokinase	Rbromii5AMG
Org2_Gene656	core	3237	[Rbromii5AMG]RB5AMG_00682_D-tyrosyl-tRNA(Tyr)_deacylase	Rbromii5AMG
Org2_Gene657	core	2613	[Rbromii5AMG]RB5AMG_00683_putative_polyketide_biosynthesis_zinc-dependent_hydrolase_PksB	Rbromii5AMG
Org2_Gene658	core	624	[Rbromii5AMG]RB5AMG_00684_Oxygen-independent_coproporphyrinogen-III_oxidase_2	Rbromii5AMG
Org2_Gene661	core	2036	[Rbromii5AMG]RB5AMG_00687_Nucleoside_triphosphate_pyrophosphohydrolase	Rbromii5AMG
Org2_Gene662	core	3918	[Rbromii5AMG]RB5AMG_00688_hypothetical_protein	Rbromii5AMG
Org2_Gene663	core	4042	[Rbromii5AMG]RB5AMG_00689_ribosome-associated_heat_shock_protein_Hsp15	Rbromii5AMG
Org2_Gene664	core	3939	[Rbromii5AMG]RB5AMG_00690_Spore_protein_YabP	Rbromii5AMG
Org2_Gene666	core	3096	[Rbromii5AMG]RB5AMG_00692_General_stress_protein_13	Rbromii5AMG
Org2_Gene667	core	2936	[Rbromii5AMG]RB5AMG_00693_SigL_modulation_protein	Rbromii5AMG
Org2_Gene668	core	1128	[Rbromii5AMG]RB5AMG_00694_Ribosomal_RNA_large_subunit_methyltransferase_K/L	Rbromii5AMG
Org2_Gene670	core	2771	[Rbromii5AMG]RB5AMG_00696_putative_HD-superfamily_hydrolase	Rbromii5AMG
Org2_Gene671	core	283	[Rbromii5AMG]RB5AMG_00697_DNA_ligase	Rbromii5AMG
Org2_Gene675	core	4250	[Rbromii5AMG]RB5AMG_00701_stage_III_sporulation_protein_AC	Rbromii5AMG
Org2_Gene679	core	3249	[Rbromii5AMG]RB5AMG_00705_stage_III_sporulation_protein_AG	Rbromii5AMG
Org2_Gene682	core	1552	[Rbromii5AMG]RB5AMG_00708_t(6)A37_threonylcarbamoyladenosine_biosynthesis_protein	Rbromii5AMG
Org2_Gene684	core	52	[Rbromii5AMG]RB5AMG_00710_DNA_polymerase_III_subunit_alpha	Rbromii5AMG
Org2_Gene686	core	1485	[Rbromii5AMG]RB5AMG_00712_autoinducer_2_ABC_transporter_permease_LsrC	Rbromii5AMG
Org2_Gene687	core	2031	[Rbromii5AMG]RB5AMG_00713_Spermidine/putrescine_import_ATP-binding_protein_PotA	Rbromii5AMG
Org2_Gene692	core	1369	[Rbromii5AMG]RB5AMG_00718_Branched-chain-amino-acid_aminotransferase	Rbromii5AMG
Org2_Gene695	core	3740	[Rbromii5AMG]RB5AMG_00721_4-carboxymuconolactone_decarboxylase	Rbromii5AMG
Org2_Gene698	core	2499	[Rbromii5AMG]RB5AMG_00724_V-type_sodium_pump_subunit_D	Rbromii5AMG
Org2_Gene699	core	716	[Rbromii5AMG]RB5AMG_00725_V-type_sodium_pump_subunit_B	Rbromii5AMG
Org2_Gene700	core	393	[Rbromii5AMG]RB5AMG_00726_V-type_ATP_synthase_alpha_chain	Rbromii5AMG
Org2_Gene702	core	3802	[Rbromii5AMG]RB5AMG_00728_V-type_ATP_synthase_subunit_F	Rbromii5AMG
Org2_Gene703	core	3337	[Rbromii5AMG]RB5AMG_00729_V-type_ATP_synthase_subunit_K	Rbromii5AMG
Org2_Gene705	core	1355	[Rbromii5AMG]RB5AMG_00731_V-type_ATP_synthase_subunit_C	Rbromii5AMG
Org2_Gene707	core	744	[Rbromii5AMG]RB5AMG_00733_hypothetical_protein	Rbromii5AMG
Org2_Gene708	core	3947	[Rbromii5AMG]RB5AMG_00734_formyltetrahydrofolate_deformylase	Rbromii5AMG
Org2_Gene710	core	1775	[Rbromii5AMG]RB5AMG_00736_UTP--glucose-1-phosphate_uridylyltransferase	Rbromii5AMG
Org2_Gene712	core	1201	[Rbromii5AMG]RB5AMG_00738_Peptide_chain_release_factor_2	Rbromii5AMG
Org2_Gene713	core	1002	[Rbromii5AMG]RB5AMG_00739_cofactor-independent_phosphoglycerate_mutase	Rbromii5AMG
Org2_Gene718	core	191	[Rbromii5AMG]RB5AMG_00746_ATP-dependent_DNA_helicase_PcrA	Rbromii5AMG
Org2_Gene722	core	702	[Rbromii5AMG]RB5AMG_00750_UDP-N-acetylmuramoylalanine--D-glutamate_ligase	Rbromii5AMG
Org2_Gene726	core	902	[Rbromii5AMG]RB5AMG_00754_cystathionine_gamma-synthase	Rbromii5AMG
Org2_Gene727	core	3230	[Rbromii5AMG]RB5AMG_00755_tRNA-specific_adenosine_deaminase	Rbromii5AMG
Org2_Gene730	core	2350	[Rbromii5AMG]RB5AMG_00758_Phosphoribosylaminoimidazole-succinocarboxamide_synthase	Rbromii5AMG
Org2_Gene731	core	613	[Rbromii5AMG]RB5AMG_00759_Amidophosphoribosyltransferase_precursor	Rbromii5AMG
Org2_Gene732	core	647	[Rbromii5AMG]RB5AMG_00760_Adenylosuccinate_lyase	Rbromii5AMG
Org2_Gene733	core	894	[Rbromii5AMG]RB5AMG_00761_Adenylosuccinate_synthetase	Rbromii5AMG
Org2_Gene734	core	536	[Rbromii5AMG]RB5AMG_00762_GMP_synthase_[glutamine-hydrolyzing]	Rbromii5AMG
Org2_Gene757	core	413	[Rbromii5AMG]RB5AMG_00785_Phosphoglucomutase	Rbromii5AMG
Org2_Gene760	core	1540	[Rbromii5AMG]RB5AMG_00788_putative_glycosyltransferase_YkoT	Rbromii5AMG
Org2_Gene761	core	1483	[Rbromii5AMG]RB5AMG_00789_6-phosphofructokinase	Rbromii5AMG
Org2_Gene766	core	2379	[Rbromii5AMG]RB5AMG_00794_RNA_polymerase_sigma-35_factor_precursor	Rbromii5AMG
Org2_Gene768	core	1754	[Rbromii5AMG]RB5AMG_00796_DegV_domain-containing_protein	Rbromii5AMG
Org2_Gene769	core	3854	[Rbromii5AMG]RB5AMG_00797_sporulation_protein,_YlmC/YmxH_family	Rbromii5AMG
Org2_Gene771	core	2264	[Rbromii5AMG]RB5AMG_00799_Vegetative_protein_209	Rbromii5AMG
Org2_Gene772	core	1659	[Rbromii5AMG]RB5AMG_00800_Elongation_factor_Ts	Rbromii5AMG
Org2_Gene775	core	4231	[Rbromii5AMG]RB5AMG_00804_Ribosomal_protein_A	Rbromii5AMG
Org2_Gene776	core	3634	[Rbromii5AMG]RB5AMG_00805_50S_ribosomal_protein_L20	Rbromii5AMG
Org2_Gene779	core	249	[Rbromii5AMG]RB5AMG_00808_DNA_topoisomerase_1	Rbromii5AMG
Org2_Gene780	core	828	[Rbromii5AMG]RB5AMG_00809_Methylenetetrahydrofolate--tRNA-(uracil-5-)-methyltransferase_TrmFO	Rbromii5AMG
Org2_Gene781	core	1426	[Rbromii5AMG]RB5AMG_00810_Phosphate_acyltransferase	Rbromii5AMG
Org2_Gene782	core	2444	[Rbromii5AMG]RB5AMG_00811_Ribonuclease_3	Rbromii5AMG
Org2_Gene784	core	47	[Rbromii5AMG]RB5AMG_00813_Chromosome_partition_protein_Smc	Rbromii5AMG
Org2_Gene785	core	1644	[Rbromii5AMG]RB5AMG_00814_Signal_recognition_particle_receptor_FtsY	Rbromii5AMG
Org2_Gene786	core	2674	[Rbromii5AMG]RB5AMG_00815_Non-canonical_purine_NTP_pyrophosphatase	Rbromii5AMG
Org2_Gene787	core	3821	[Rbromii5AMG]RB5AMG_00816_RNA-binding_protein	Rbromii5AMG
Org2_Gene790	core	3626	[Rbromii5AMG]RB5AMG_00819_Ribosomal_silencing_factor_RsfS	Rbromii5AMG
Org2_Gene791	core	155	[Rbromii5AMG]RB5AMG_00820_Leucine--tRNA_ligase	Rbromii5AMG
Org2_Gene792	core	4323	[Rbromii5AMG]RB5AMG_00821_30S_ribosomal_protein_S21	Rbromii5AMG
Org2_Gene793	core	3327	[Rbromii5AMG]RB5AMG_00822_3-dehydroquinate_dehydratase	Rbromii5AMG
Org2_Gene795	core	2854	[Rbromii5AMG]RB5AMG_00824_Elongation_factor_P	Rbromii5AMG
Org2_Gene798	core	673	[Rbromii5AMG]RB5AMG_00827_(Dimethylallyl)adenosine_tRNA_methylthiotransferase_MiaB	Rbromii5AMG
Org2_Gene799	core	3428	[Rbromii5AMG]RB5AMG_00828_hypothetical_protein	Rbromii5AMG
Org2_Gene800	core	127	[Rbromii5AMG]RB5AMG_00829_DNA_mismatch_repair_protein_MutS	Rbromii5AMG
Org2_Gene804	core	2317	[Rbromii5AMG]RB5AMG_00833_alanine_racemase	Rbromii5AMG
Org2_Gene805	core	3192	[Rbromii5AMG]RB5AMG_00834_Cell_division_protein_SepF	Rbromii5AMG
Org2_Gene808	core	102	[Rbromii5AMG]RB5AMG_00837_Isoleucine--tRNA_ligase	Rbromii5AMG
Org2_Gene809	core	3126	[Rbromii5AMG]RB5AMG_00838_Lipoprotein_signal_peptidase	Rbromii5AMG
Org2_Gene810	core	1638	[Rbromii5AMG]RB5AMG_00839_Ribosomal_large_subunit_pseudouridine_synthase_D	Rbromii5AMG
Org2_Gene811	core	3645	[Rbromii5AMG]RB5AMG_00840_50S_ribosomal_protein_L19	Rbromii5AMG
Org2_Gene814	core	1771	[Rbromii5AMG]RB5AMG_00843_Ribosome_biogenesis_GTPase_A	Rbromii5AMG
Org2_Gene815	core	2656	[Rbromii5AMG]RB5AMG_00844_Ribonuclease_HII	Rbromii5AMG
Org2_Gene818	core	574	[Rbromii5AMG]RB5AMG_00847_Threonine_synthase	Rbromii5AMG
Org2_Gene819	core	2412	[Rbromii5AMG]RB5AMG_00848_putative_phosphoesterase_or_phosphohydrolase	Rbromii5AMG
Org2_Gene820	core	890	[Rbromii5AMG]RB5AMG_00849_Trigger_factor	Rbromii5AMG
Org2_Gene821	core	2763	[Rbromii5AMG]RB5AMG_00850_ATP-dependent_Clp_protease_proteolytic_subunit	Rbromii5AMG
Org2_Gene822	core	781	[Rbromii5AMG]RB5AMG_00851_ATP-dependent_Clp_protease_ATP-binding_subunit_ClpX	Rbromii5AMG
Org2_Gene823	core	156	[Rbromii5AMG]RB5AMG_00852_Lon_protease_1	Rbromii5AMG
Org2_Gene824	core	2688	[Rbromii5AMG]RB5AMG_00853_putative_GTP-binding_protein_EngB	Rbromii5AMG
Org2_Gene825	core	857	[Rbromii5AMG]RB5AMG_00854_Diaminopimelate_decarboxylase	Rbromii5AMG
Org2_Gene826	core	2370	[Rbromii5AMG]RB5AMG_00855_putative_membrane_protein	Rbromii5AMG
Org2_Gene828	core	796	[Rbromii5AMG]RB5AMG_00857_NADP-specific_glutamate_dehydrogenase	Rbromii5AMG
Org2_Gene829	core	1460	[Rbromii5AMG]RB5AMG_00858_Tyrosine_recombinase_XerD	Rbromii5AMG
Org2_Gene83	core	2795	[Rbromii5AMG]RB5AMG_00089_Dipicolinate_synthase_subunit_B	Rbromii5AMG
Org2_Gene831	core	734	[Rbromii5AMG]RB5AMG_00860_Lon_protease_2	Rbromii5AMG
Org2_Gene833	core	1789	[Rbromii5AMG]RB5AMG_00862_Methionine_aminopeptidase	Rbromii5AMG
Org2_Gene840	core	318	[Rbromii5AMG]RB5AMG_00869_Ferrous_iron_transport_protein_B	Rbromii5AMG

Org2_Gene842	core	724	[Rbromii5AMG]RB5AMG_00871_Anaerobic_sulfatase-maturating_enzyme	Rbromii5AMG
Org2_Gene843	core	4381	[Rbromii5AMG]RB5AMG_00872_six-cysteine_peptide_SCIFF	Rbromii5AMG
Org2_Gene845	core	520	[Rbromii5AMG]RB5AMG_00874_Peptide_chain_release_factor_3	Rbromii5AMG
Org2_Gene862	core	4002	[Rbromii5AMG]RB5AMG_00891_Helix-turn-helix_domain_protein	Rbromii5AMG
Org2_Gene863	core	3252	[Rbromii5AMG]RB5AMG_00892_Ribose-5-phosphate_isomerase_B	Rbromii5AMG
Org2_Gene865	core	3357	[Rbromii5AMG]RB5AMG_00894_ADP-binding_protein	Rbromii5AMG
Org2_Gene866	core	456	[Rbromii5AMG]RB5AMG_00895_Formate--tetrahydrofolate_ligase	Rbromii5AMG
Org2_Gene869	core	1734	[Rbromii5AMG]RB5AMG_00898_DisA_bacterial_checkpoint_controller_nucleotide-binding_protein	Rbromii5AMG
Org2_Gene872	core	2158	[Rbromii5AMG]RB5AMG_00901_4-hydroxy-tetrahydrodipicolinate_reductase	Rbromii5AMG
Org2_Gene873	core	1686	[Rbromii5AMG]RB5AMG_00902_4-hydroxy-tetrahydrodipicolinate_synthase	Rbromii5AMG
Org2_Gene874	core	1256	[Rbromii5AMG]RB5AMG_00903_Aspartate-semialdehyde_dehydrogenase_2	Rbromii5AMG
Org2_Gene875	core	1375	[Rbromii5AMG]RB5AMG_00904_S-adenosylmethionine:tRNA_ribosyltransferase-isomerase	Rbromii5AMG
Org2_Gene876	core	1179	[Rbromii5AMG]RB5AMG_00905_Queuine_tRNA-ribosyltransferase	Rbromii5AMG
Org2_Gene88	core	905	[Rbromii5AMG]RB5AMG_00094_Methionine_gamma-lyase	Rbromii5AMG
Org2_Gene89	core	2362	[Rbromii5AMG]RB5AMG_00095_2-C-methyl-D-erythritol_4-phosphate_cytidylyltransferase	Rbromii5AMG
Org2_Gene894	core	2195	[Rbromii5AMG]RB5AMG_00923_serine/threonine_protein_kinase	Rbromii5AMG
Org2_Gene895	core	123	[Rbromii5AMG]RB5AMG_00924_Aldehyde-alcohol_dehydrogenase	Rbromii5AMG
Org2_Gene898	core	996	[Rbromii5AMG]RB5AMG_00927_Acetate_kinase	Rbromii5AMG
Org2_Gene899	core	4093	[Rbromii5AMG]RB5AMG_00928_hypothetical_protein	Rbromii5AMG
Org2_Gene90	core	3164	[Rbromii5AMG]RB5AMG_00096_2-C-methyl-D-erythritol_2,4-cyclodiphosphate_synthase	Rbromii5AMG
Org2_Gene91	core	1141	[Rbromii5AMG]RB5AMG_00097_D-alanyl-D-alanine_carboxypeptidase_DacF_precursor	Rbromii5AMG
Org2_Gene917	core	1834	[Rbromii5AMG]RB5AMG_00947_Nicotinamide_nucleotide_repair_protein	Rbromii5AMG
Org2_Gene92	core	787	[Rbromii5AMG]RB5AMG_00098_Glucose-6-phosphate_isomerase	Rbromii5AMG
Org2_Gene920	core	336	[Rbromii5AMG]RB5AMG_00950_UDP-N-acetyl-alpha-D-glucosamine_C6_dehydratase	Rbromii5AMG
Org2_Gene921	core	356	[Rbromii5AMG]RB5AMG_00951_Glutamine--fructose-6-phosphate_aminotransferase_[isomerizing]	Rbromii5AMG
Org2_Gene923	core	907	[Rbromii5AMG]RB5AMG_00953_Dihydroorotase	Rbromii5AMG
Org2_Gene924	core	1469	[Rbromii5AMG]RB5AMG_00954_4-hydroxy-2-oxovalerate_aldolase	Rbromii5AMG
Org2_Gene925	core	1588	[Rbromii5AMG]RB5AMG_00955_orotidine_5'-phosphate_decarboxylase	Rbromii5AMG
Org2_Gene926	core	1255	[Rbromii5AMG]RB5AMG_00956_Phosphoserine_aminotransferase	Rbromii5AMG
Org2_Gene927	core	1125	[Rbromii5AMG]RB5AMG_00957_D-3-phosphoglycerate_dehydrogenase	Rbromii5AMG
Org2_Gene93	core	3391	[Rbromii5AMG]RB5AMG_00099_hypothetical_protein	Rbromii5AMG
Org2_Gene933	core	1383	[Rbromii5AMG]RB5AMG_00963_Tryptophan--tRNA_ligase	Rbromii5AMG
Org2_Gene934	core	1056	[Rbromii5AMG]RB5AMG_00964_2-aminoadipate_transaminase	Rbromii5AMG
Org2_Gene936	core	2391	[Rbromii5AMG]RB5AMG_00966_Putative_mutator_protein_MutT4	Rbromii5AMG
Org2_Gene938	core	2166	[Rbromii5AMG]RB5AMG_00968_Putative_TrmH_family_tRNA/rRNA_methyltransferase	Rbromii5AMG
Org2_Gene940	core	2522	[Rbromii5AMG]RB5AMG_00970_Ribulose-phosphate_3-epimerase	Rbromii5AMG
Org2_Gene942	core	1291	[Rbromii5AMG]RB5AMG_00972_methyltransferase,_FkbM_family	Rbromii5AMG
Org2_Gene943	core	958	[Rbromii5AMG]RB5AMG_00974_orc1/cdc6_family_replication_initiation_protein	Rbromii5AMG
Org2_Gene945	core	1904	[Rbromii5AMG]RB5AMG_00976_DNA-3-methyladenine_glycosylase	Rbromii5AMG
Org2_Gene946	core	2411	[Rbromii5AMG]RB5AMG_00977_hypothetical_protein	Rbromii5AMG
Org2_Gene947	core	4026	[Rbromii5AMG]RB5AMG_00978_hypothetical_protein	Rbromii5AMG
Org2_Gene950	core	2836	[Rbromii5AMG]RB5AMG_00981_putative_manganese_efflux_pump_MntP	Rbromii5AMG
Org2_Gene952	core	1890	[Rbromii5AMG]RB5AMG_00983_Dihydroorotate_oxidase_B,_electron_transfer_subunit	Rbromii5AMG
Org2_Gene953	core	700	[Rbromii5AMG]RB5AMG_00984_Glutamate_synthase_[NADPH]_small_chain	Rbromii5AMG
Org2_Gene955	core	636	[Rbromii5AMG]RB5AMG_00986_Trk_system_potassium_uptake_protein_TrkG	Rbromii5AMG
Org2_Gene957	core	1782	[Rbromii5AMG]RB5AMG_00988_Germination_protease_precursor	Rbromii5AMG
Org2_Gene959	core	913	[Rbromii5AMG]RB5AMG_00990_Replication-associated_recombination_protein_A	Rbromii5AMG
Org2_Gene961	core	153	[Rbromii5AMG]RB5AMG_00992_Beta/alpha-amylase_precursor	Rbromii5AMG
Org2_Gene966	core	891	[Rbromii5AMG]RB5AMG_00997_phage_terminase,_large_subunit,_PBSX_family	Rbromii5AMG
Org2_Gene970	core	1409	[Rbromii5AMG]RB5AMG_01001_phage_major_capsid_protein,_HK97_family	Rbromii5AMG
Org2_Gene983	core	120	[Rbromii5AMG]RB5AMG_01014_Pyruvate,_phosphate_dikinase	Rbromii5AMG
Org2_Gene984	core	2182	[Rbromii5AMG]RB5AMG_01015_Bifunctional_protein_GlmU	Rbromii5AMG
Org2_Gene985	core	1519	[Rbromii5AMG]RB5AMG_01016_Ribose-phosphate_pyrophosphokinase	Rbromii5AMG
Org2_Gene987	core	2720	[Rbromii5AMG]RB5AMG_01018_Peptidyl-tRNA_hydrolase	Rbromii5AMG
Org2_Gene988	core	56	[Rbromii5AMG]RB5AMG_01019_Transcription-repair-coupling_factor	Rbromii5AMG
Org2_Gene991	core	4115	[Rbromii5AMG]RB5AMG_01022_hypothetical_protein	Rbromii5AMG
Org2_Gene992	core	415	[Rbromii5AMG]RB5AMG_01023_MutS2_protein	Rbromii5AMG
Org2_Gene997	core	926	[Rbromii5AMG]RB5AMG_01028_putative_ion_channel_protein	Rbromii5AMG
Org3_Gene1003	core	3992	[RbromiiATCC27255]RBATCC27255_01031_hypothetical_protein	RbromiiATCC27255
Org3_Gene1004	core	99	[RbromiiATCC27255]RBATCC27255_01032_Excinuclease_ABC_subunit_A	RbromiiATCC27255
Org3_Gene1005	core	270	[RbromiiATCC27255]RBATCC27255_01033_ATP-dependent_zinc_metalloprotease_FtsH	RbromiiATCC27255
Org3_Gene1006	core	2937	[RbromiiATCC27255]RBATCC27255_01034_Hypoxanthine-guanine_phosphoribosyltransferase	RbromiiATCC27255
Org3_Gene1008	core	725	[RbromiiATCC27255]RBATCC27255_01036_Replicative_DNA_helicase	RbromiiATCC27255
Org3_Gene1009	core	3258	[RbromiiATCC27255]RBATCC27255_01037_50S_ribosomal_protein_L9	RbromiiATCC27255
Org3_Gene1011	core	320	[RbromiiATCC27255]RBATCC27255_01039_Ribonuclease_J_1	RbromiiATCC27255
Org3_Gene1012	core	3091	[RbromiiATCC27255]RBATCC27255_01040_Ribonuclease_HI	RbromiiATCC27255
Org3_Gene1013	core	1027	[RbromiiATCC27255]RBATCC27255_01041_Cysteine_desulfurase	RbromiiATCC27255
Org3_Gene1014	core	3330	[RbromiiATCC27255]RBATCC27255_01042_NifU-like_protein	RbromiiATCC27255
Org3_Gene1017	core	1285	[RbromiiATCC27255]RBATCC27255_01045_3-isopropylmalate_dehydrogenase	RbromiiATCC27255
Org3_Gene1018	core	2610	[RbromiiATCC27255]RBATCC27255_01046_Alpha-ribazole_phosphatase	RbromiiATCC27255
Org3_Gene1020	core	3646	[RbromiiATCC27255]RBATCC27255_01048_mRNA_interferase_MazF	RbromiiATCC27255
Org3_Gene1028	core	207	[RbromiiATCC27255]RBATCC27255_01056_Polyribonucleotide_nucleotidyltransferase	RbromiiATCC27255
Org3_Gene1029	core	3962	[RbromiiATCC27255]RBATCC27255_01057_hypothetical_protein	RbromiiATCC27255
Org3_Gene1053	core	2252	[RbromiiATCC27255]RBATCC27255_01083_Putative_HAD-hydrolase_YfnB	RbromiiATCC27255
Org3_Gene1054	core	2513	[RbromiiATCC27255]RBATCC27255_01084_UV-endonuclease	RbromiiATCC27255
Org3_Gene1057	core	727	[RbromiiATCC27255]RBATCC27255_01087_Magnesium_transporter_MgtE	RbromiiATCC27255
Org3_Gene1059	core	1812	[RbromiiATCC27255]RBATCC27255_01089_Fructose-bisphosphate_aldolase	RbromiiATCC27255
Org3_Gene106	core	3239	[RbromiiATCC27255]RBATCC27255_00110_S-ribosylhomocysteine_lyase	RbromiiATCC27255
Org3_Gene1063	core	446	[RbromiiATCC27255]RBATCC27255_01098_2-isopropylmalate_synthase	RbromiiATCC27255
Org3_Gene1073	core	1288	[RbromiiATCC27255]RBATCC27255_01108_Aldose_1-epimerase_precursor	RbromiiATCC27255
Org3_Gene1074	core	572	[RbromiiATCC27255]RBATCC27255_01109_galactose-1-phosphate_uridylyltransferase	RbromiiATCC27255
Org3_Gene1075	core	1440	[RbromiiATCC27255]RBATCC27255_01110_UDP-glucose_4-epimerase	RbromiiATCC27255
Org3_Gene1100	core	3322	[RbromiiATCC27255]RBATCC27255_01135_Cupin_domain_protein	RbromiiATCC27255
Org3_Gene1102	core	3228	[RbromiiATCC27255]RBATCC27255_01137_Transcriptional_repressor_NrdR	RbromiiATCC27255
Org3_Gene1104	core	265	[RbromiiATCC27255]RBATCC27255_01139_ATP-dependent_DNA_helicase_RecG	RbromiiATCC27255
Org3_Gene1105	core	470	[RbromiiATCC27255]RBATCC27255_01140_dihydroxyacetone_kinase	RbromiiATCC27255
Org3_Gene1106	core	2126	[RbromiiATCC27255]RBATCC27255_01141_metallophosphoesterase,_MG_246/_family	RbromiiATCC27255
Org3_Gene111	core	1484	[RbromiiATCC27255]RBATCC27255_00115_Putative_glycosyltransferase_CsbB	RbromiiATCC27255
Org3_Gene1110	core	354	[RbromiiATCC27255]RBATCC27255_01145_Asparagine_synthetase_[glutamine-hydrolyzing]_3	RbromiiATCC27255
Org3_Gene1112	core	3312	[RbromiiATCC27255]RBATCC27255_01147_Large-conductance_mechanosensitive_channel	RbromiiATCC27255
Org3_Gene1113	core	979	[RbromiiATCC27255]RBATCC27255_01148_Tyrosine--tRNA_ligase	RbromiiATCC27255
Org3_Gene1115	core	316	[RbromiiATCC27255]RBATCC27255_01150_putative_radical_SAM_protein_YgiQ	RbromiiATCC27255
Org3_Gene1117	core	1521	[RbromiiATCC27255]RBATCC27255_01152_Undecaprenyl-diphosphatase	RbromiiATCC27255
Org3_Gene1118	core	1989	[RbromiiATCC27255]RBATCC27255_01153_Translocation-enhancing_protein_TepA	RbromiiATCC27255
Org3_Gene1119	core	830	[RbromiiATCC27255]RBATCC27255_01154_Hemolysin_C	RbromiiATCC27255
Org3_Gene1121	core	2718	[RbromiiATCC27255]RBATCC27255_01156_Ribonuclease_M5	RbromiiATCC27255
Org3_Gene1125	core	2935	[RbromiiATCC27255]RBATCC27255_01160_Pur_operon_repressor	RbromiiATCC27255
Org3_Gene1126	core	3658	[RbromiiATCC27255]RBATCC27255_01161_HIT-like_protein	RbromiiATCC27255
Org3_Gene1128	core	819	[RbromiiATCC27255]RBATCC27255_01163_GTP-binding_protein_EngA	RbromiiATCC27255
Org3_Gene113	core	2878	[RbromiiATCC27255]RBATCC27255_00117_Fumarate_hydratase_class_I,_aerobic	RbromiiATCC27255
Org3_Gene1130	core	4271	[RbromiiATCC27255]RBATCC27255_01165_50S_ribosomal_protein_L32	RbromiiATCC27255
Org3_Gene1131	core	3074	[RbromiiATCC27255]RBATCC27255_01166_hypothetical_protein	RbromiiATCC27255
Org3_Gene1138	core	1618	[RbromiiATCC27255]RBATCC27255_01173_30S_ribosomal_protein_S1	RbromiiATCC27255
Org3_Gene114	core	1863	[RbromiiATCC27255]RBATCC27255_00118_L(+)-tartrate_dehydratase_subunit_alpha	RbromiiATCC27255
Org3_Gene1140	core	1871	[RbromiiATCC27255]RBATCC27255_01175_HAD_phosphoserine_phosphatase-like_hydrolase,_family_IB	RbromiiATCC27255
Org3_Gene1149	core	1660	[RbromiiATCC27255]RBATCC27255_01184_Daunorubicin/doxorubicin_resistance_ATP-binding_protein_DrrA	RbromiiATCC27255
Org3_Gene115	core	2424	[RbromiiATCC27255]RBATCC27255_00119_Staphylococcal_respiratory_response_protein_A	RbromiiATCC27255
Org3_Gene1157	core	3710	[RbromiiATCC27255]RBATCC27255_01192_hypothetical_protein	RbromiiATCC27255
Org3_Gene116	core	525	[RbromiiATCC27255]RBATCC27255_00120_Alkaline_phosphatase_synthesis_sensor_protein_PhoR	RbromiiATCC27255
Org3_Gene1161	core	817	[RbromiiATCC27255]RBATCC27255_01196_putative_bifunctional_UDP-N-acetylmuramoylalanyl-D-glutamate--2,6-diaminopimelate_ligase/UDP-N-acetylmu	RbromiiATCC27255
Org3_Gene1163	core	210	[RbromiiATCC27255]RBATCC27255_01198_Exodeoxyribonuclease_V_alpha_chain	RbromiiATCC27255
Org3_Gene1168	core	659	[RbromiiATCC27255]RBATCC27255_01203_Spermidine/putrescine_import_ATP-binding_protein_PotA	RbromiiATCC27255
Org3_Gene1169	core	2069	[RbromiiATCC27255]RBATCC27255_01204_Putrescine_transport_system_permease_protein_PotH	RbromiiATCC27255
Org3_Gene1170	core	1988	[RbromiiATCC27255]RBATCC27255_01205_Inner_membrane_ABC_transporter_permease_protein_YdcV	RbromiiATCC27255
Org3_Gene1171	core	1251	[RbromiiATCC27255]RBATCC27255_01206_Spermidine/putrescine-binding_periplasmic_protein_precursor	RbromiiATCC27255
Org3_Gene1172	core	1968	[RbromiiATCC27255]RBATCC27255_01207_Teichoic_acid_translocation_permease_protein_TagG	RbromiiATCC27255
Org3_Gene1173	core	2205	[RbromiiATCC27255]RBATCC27255_01208_Teichoic_acids_export_ATP-binding_protein_TagH	RbromiiATCC27255
Org3_Gene119	core	4378	[RbromiiATCC27255]RBATCC27255_00123_hypothetical_protein	RbromiiATCC27255
Org3_Gene12	core	2195	[RbromiiATCC27255]RBATCC27255_00014_serine/threonine_protein_kinase	RbromiiATCC27255
Org3_Gene120	core	3981	[RbromiiATCC27255]RBATCC27255_00124_30S_ribosomal_protein_S20	RbromiiATCC27255
Org3_Gene1202	core	536	[RbromiiATCC27255]RBATCC27255_01238_GMP_synthase_[glutamine-hydrolyzing]	RbromiiATCC27255
Org3_Gene1203	core	894	[RbromiiATCC27255]RBATCC27255_01239_Adenylosuccinate_synthetase	RbromiiATCC27255

Org3_Gene1204	core	647	[RbromiiATCC27255]RBATCC27255_01240_Adenylosuccinate_lyase	RbromiiATCC27255
Org3_Gene1205	core	613	[RbromiiATCC27255]RBATCC27255_01241_Amidophosphoribosyltransferase_precursor	RbromiiATCC27255
Org3_Gene1206	core	2350	[RbromiiATCC27255]RBATCC27255_01242_Phosphoribosylaminoimidazole-succinocarboxamide_synthase	RbromiiATCC27255
Org3_Gene1208	core	1282	[RbromiiATCC27255]RBATCC27255_01245_Aspartate_carbamoyltransferase_catalytic_chain	RbromiiATCC27255
Org3_Gene121	core	2275	[RbromiiATCC27255]RBATCC27255_00125_NAD-dependent_protein_deacetylase	RbromiiATCC27255
Org3_Gene1213	core	1801	[RbromiiATCC27255]RBATCC27255_01250_4-diphosphocytidyl-2-C-methyl-D-erythritol_kinase	RbromiiATCC27255
Org3_Gene1214	core	1075	[RbromiiATCC27255]RBATCC27255_01251_Putative_N-acetyl-LL-diaminopimelate_aminotransferase	RbromiiATCC27255
Org3_Gene1215	core	3130	[RbromiiATCC27255]RBATCC27255_01252_Leucine-responsive_regulatory_protein	RbromiiATCC27255
Org3_Gene1217	core	313	[RbromiiATCC27255]RBATCC27255_01254_Glutamine-dependent_NAD(+)_synthetase	RbromiiATCC27255
Org3_Gene1219	core	2431	[RbromiiATCC27255]RBATCC27255_01256_Putative_undecaprenyl-phosphate_N-acetylgalactosaminyl_1-phosphate_transferase	RbromiiATCC27255
Org3_Gene122	core	2133	[RbromiiATCC27255]RBATCC27255_00126_Tryptophan_synthase_alpha_chain	RbromiiATCC27255
Org3_Gene1221	core	1512	[RbromiiATCC27255]RBATCC27255_01259_Glycerol-3-phosphate_dehydrogenase_[NAD(P)+]	RbromiiATCC27255
Org3_Gene1226	core	3967	[RbromiiATCC27255]RBATCC27255_01264_hypothetical_protein	RbromiiATCC27255
Org3_Gene1227	core	487	[RbromiiATCC27255]RBATCC27255_01265_Stress_protein_H5	RbromiiATCC27255
Org3_Gene1228	core	3886	[RbromiiATCC27255]RBATCC27255_01266_co-chaperonin_GroES	RbromiiATCC27255
Org3_Gene1229	core	480	[RbromiiATCC27255]RBATCC27255_01267_Serine/threonine-protein_kinase_PrkC	RbromiiATCC27255
Org3_Gene123	core	1062	[RbromiiATCC27255]RBATCC27255_00127_Tryptophan_synthase_beta_chain	RbromiiATCC27255
Org3_Gene1231	core	1753	[RbromiiATCC27255]RBATCC27255_01270_Putative_ribosome_biogenesis_GTPase_RsgA	RbromiiATCC27255
Org3_Gene1232	core	238	[RbromiiATCC27255]RBATCC27255_01271_Serine/threonine-protein_kinase_PrkC	RbromiiATCC27255
Org3_Gene1233	core	2278	[RbromiiATCC27255]RBATCC27255_01272_Serine/threonine_phosphatase_stp	RbromiiATCC27255
Org3_Gene1234	core	1357	[RbromiiATCC27255]RBATCC27255_01273_putative_dual-specificity_RNA_methyltransferase_RlmN	RbromiiATCC27255
Org3_Gene1236	core	2364	[RbromiiATCC27255]RBATCC27255_01275_Putative_neutral_zinc_metallopeptidase	RbromiiATCC27255
Org3_Gene1237	core	1641	[RbromiiATCC27255]RBATCC27255_01276_Methionyl-tRNA_formyltransferase	RbromiiATCC27255
Org3_Gene1238	core	3150	[RbromiiATCC27255]RBATCC27255_01277_Peptide_deformylase_1	RbromiiATCC27255
Org3_Gene1239	core	144	[RbromiiATCC27255]RBATCC27255_01278_Primosomal_protein_N'	RbromiiATCC27255
Org3_Gene1240	core	4190	[RbromiiATCC27255]RBATCC27255_01279_DNA-directed_RNA_polymerase_subunit_omega	RbromiiATCC27255
Org3_Gene1242	core	3994	[RbromiiATCC27255]RBATCC27255_01281_hypothetical_protein	RbromiiATCC27255
Org3_Gene1243	core	1747	[RbromiiATCC27255]RBATCC27255_01282_hypothetical_protein	RbromiiATCC27255
Org3_Gene1244	core	2241	[RbromiiATCC27255]RBATCC27255_01283_putative_transcriptional_regulatory_protein_YebC	RbromiiATCC27255
Org3_Gene1246	core	279	[RbromiiATCC27255]RBATCC27255_01285_hypothetical_protein	RbromiiATCC27255
Org3_Gene1247	core	2586	[RbromiiATCC27255]RBATCC27255_01286_1-acyl-sn-glycerol-3-phosphate_acyltransferase	RbromiiATCC27255
Org3_Gene1248	core	2482	[RbromiiATCC27255]RBATCC27255_01287_Cytidylate_kinase	RbromiiATCC27255
Org3_Gene1249	core	952	[RbromiiATCC27255]RBATCC27255_01288_tricarballylate_dehydrogenase	RbromiiATCC27255
Org3_Gene1250	core	1715	[RbromiiATCC27255]RBATCC27255_01289_MurPQ_operon_repressor	RbromiiATCC27255
Org3_Gene1251	core	2620	[RbromiiATCC27255]RBATCC27255_01290_Imidazole_glycerol_phosphate_synthase_subunit_HisH_1	RbromiiATCC27255
Org3_Gene1252	core	2177	[RbromiiATCC27255]RBATCC27255_01291_Imidazole_glycerol_phosphate_synthase_subunit_HisF	RbromiiATCC27255
Org3_Gene1254	core	1964	[RbromiiATCC27255]RBATCC27255_01293_enhanced_serine_sensitivity_protein_SseB	RbromiiATCC27255
Org3_Gene1257	core	2031	[RbromiiATCC27255]RBATCC27255_01296_Spermidine/putrescine_import_ATP-binding_protein_PotA	RbromiiATCC27255
Org3_Gene1258	core	1485	[RbromiiATCC27255]RBATCC27255_01297_autoinducer_2_ABC_transporter_permease_LsrC	RbromiiATCC27255
Org3_Gene1260	core	52	[RbromiiATCC27255]RBATCC27255_01299_DNA_polymerase_III_subunit_alpha	RbromiiATCC27255
Org3_Gene1262	core	1552	[RbromiiATCC27255]RBATCC27255_01301_t(6)A37_threonylcarbamoyladenosine_biosynthesis_protein	RbromiiATCC27255
Org3_Gene1268	core	3230	[RbromiiATCC27255]RBATCC27255_01307_tRNA-specific_adenosine_deaminase	RbromiiATCC27255
Org3_Gene1269	core	902	[RbromiiATCC27255]RBATCC27255_01308_cystathionine_gamma-synthase	RbromiiATCC27255
Org3_Gene1273	core	702	[RbromiiATCC27255]RBATCC27255_01312_UDP-N-acetylmuramoylalanine--D-glutamate_ligase	RbromiiATCC27255
Org3_Gene1277	core	191	[RbromiiATCC27255]RBATCC27255_01316_ATP-dependent_DNA_helicase_PcrA	RbromiiATCC27255
Org3_Gene128	core	758	[RbromiiATCC27255]RBATCC27255_00133_UDP-N-acetylmuramoyl-tripeptide--D-alanyl-D-alanine_ligase	RbromiiATCC27255
Org3_Gene1282	core	1002	[RbromiiATCC27255]RBATCC27255_01321_cofactor-independent_phosphoglycerate_mutase	RbromiiATCC27255
Org3_Gene1283	core	1201	[RbromiiATCC27255]RBATCC27255_01322_Peptide_chain_release_factor_2	RbromiiATCC27255
Org3_Gene1285	core	1775	[RbromiiATCC27255]RBATCC27255_01324_UTP--glucose-1-phosphate_uridylyltransferase	RbromiiATCC27255
Org3_Gene1287	core	3947	[RbromiiATCC27255]RBATCC27255_01326_formyltetrahydrofolate_deformylase	RbromiiATCC27255
Org3_Gene1288	core	744	[RbromiiATCC27255]RBATCC27255_01327_hypothetical_protein	RbromiiATCC27255
Org3_Gene129	core	1289	[RbromiiATCC27255]RBATCC27255_00134_D-alanine--D-alanine_ligase	RbromiiATCC27255
Org3_Gene1290	core	1355	[RbromiiATCC27255]RBATCC27255_01329_V-type_ATP_synthase_subunit_C	RbromiiATCC27255
Org3_Gene1292	core	3337	[RbromiiATCC27255]RBATCC27255_01331_V-type_ATP_synthase_subunit_K	RbromiiATCC27255
Org3_Gene1293	core	3802	[RbromiiATCC27255]RBATCC27255_01332_V-type_ATP_synthase_subunit_F	RbromiiATCC27255
Org3_Gene1295	core	393	[RbromiiATCC27255]RBATCC27255_01334_V-type_ATP_synthase_alpha_chain	RbromiiATCC27255
Org3_Gene1296	core	716	[RbromiiATCC27255]RBATCC27255_01335_V-type_sodium_pump_subunit_B	RbromiiATCC27255
Org3_Gene1297	core	2499	[RbromiiATCC27255]RBATCC27255_01336_V-type_sodium_pump_subunit_D	RbromiiATCC27255
Org3_Gene1300	core	3740	[RbromiiATCC27255]RBATCC27255_01339_4-carboxymuconolactone_decarboxylase	RbromiiATCC27255
Org3_Gene1303	core	1369	[RbromiiATCC27255]RBATCC27255_01346_Branched-chain-amino-acid_aminotransferase	RbromiiATCC27255
Org3_Gene1309	core	3444	[RbromiiATCC27255]RBATCC27255_01352_Mini-ribonuclease_3	RbromiiATCC27255
Org3_Gene1310	core	909	[RbromiiATCC27255]RBATCC27255_01353_Spo0B-associated_GTP-binding_protein	RbromiiATCC27255
Org3_Gene1311	core	3887	[RbromiiATCC27255]RBATCC27255_01354_50S_ribosomal_protein_L27	RbromiiATCC27255
Org3_Gene1313	core	3807	[RbromiiATCC27255]RBATCC27255_01356_50S_ribosomal_protein_L21	RbromiiATCC27255
Org3_Gene1314	core	1866	[RbromiiATCC27255]RBATCC27255_01357_Bifunctional_protein_FolD_protein	RbromiiATCC27255
Org3_Gene1315	core	892	[RbromiiATCC27255]RBATCC27255_01358_Putative_zinc_protease_AlbF	RbromiiATCC27255
Org3_Gene1318	core	435	[RbromiiATCC27255]RBATCC27255_01361_Neopullulanase	RbromiiATCC27255
Org3_Gene1319	core	251	[RbromiiATCC27255]RBATCC27255_01362_Glycogen_debranching_enzyme	RbromiiATCC27255
Org3_Gene1339	core	1118	[RbromiiATCC27255]RBATCC27255_01382_hypothetical_protein	RbromiiATCC27255
Org3_Gene134	core	885	[RbromiiATCC27255]RBATCC27255_00139_Serine--tRNA_ligase	RbromiiATCC27255
Org3_Gene135	core	1877	[RbromiiATCC27255]RBATCC27255_00140_putative_chromosome-partitioning_protein_ParB	RbromiiATCC27255
Org3_Gene136	core	2141	[RbromiiATCC27255]RBATCC27255_00141_Sporulation_initiation_inhibitor_protein_soj	RbromiiATCC27255
Org3_Gene1361	core	2892	[RbromiiATCC27255]RBATCC27255_01404_Cys-tRNA(Pro)/Cys-tRNA(Cys)_deacylase_YbaK	RbromiiATCC27255
Org3_Gene1364	core	3160	[RbromiiATCC27255]RBATCC27255_01407_Sodium_ATPase_proteolipid_component	RbromiiATCC27255
Org3_Gene1367	core	3757	[RbromiiATCC27255]RBATCC27255_01410_V-type_sodium_pump_subunit_G	RbromiiATCC27255
Org3_Gene1368	core	384	[RbromiiATCC27255]RBATCC27255_01411_V-type_sodium_ATPase_catalytic_subunit_A	RbromiiATCC27255
Org3_Gene1369	core	723	[RbromiiATCC27255]RBATCC27255_01412_V-type_sodium_pump_subunit_B	RbromiiATCC27255
Org3_Gene137	core	1848	[RbromiiATCC27255]RBATCC27255_00142_Nucleoid_occlusion_protein	RbromiiATCC27255
Org3_Gene1370	core	2494	[RbromiiATCC27255]RBATCC27255_01413_V-type_sodium_pump_subunit_D	RbromiiATCC27255
Org3_Gene1375	core	231	[RbromiiATCC27255]RBATCC27255_01418_Silver_exporting_P-type_ATPase	RbromiiATCC27255
Org3_Gene139	core	332	[RbromiiATCC27255]RBATCC27255_00144_Glucose-inhibited_division_protein_A	RbromiiATCC27255
Org3_Gene1397	core	4031	[RbromiiATCC27255]RBATCC27255_01443_Acyl_carrier_protein	RbromiiATCC27255
Org3_Gene1399	core	2246	[RbromiiATCC27255]RBATCC27255_01445_3-oxoacyl-[acyl-carrier-protein]_reductase_FabG	RbromiiATCC27255
Org3_Gene140	core	715	[RbromiiATCC27255]RBATCC27255_00145_tRNA_modification_GTPase_MnmE	RbromiiATCC27255
Org3_Gene1400	core	3244	[RbromiiATCC27255]RBATCC27255_01446_3-hydroxyacyl-[acyl-carrier-protein]_dehydratase_FabZ	RbromiiATCC27255
Org3_Gene141	core	2011	[RbromiiATCC27255]RBATCC27255_00146_R3H_domain_protein	RbromiiATCC27255
Org3_Gene1418	core	2898	[RbromiiATCC27255]RBATCC27255_01464_hypothetical_protein	RbromiiATCC27255
Org3_Gene143	core	4063	[RbromiiATCC27255]RBATCC27255_00148_Putative_membrane_protein_insertion_efficiency_factor	RbromiiATCC27255
Org3_Gene144	core	3616	[RbromiiATCC27255]RBATCC27255_00149_Ribonuclease_P_protein_component	RbromiiATCC27255
Org3_Gene1442	core	2188	[RbromiiATCC27255]RBATCC27255_01488_Sulfur_carrier_protein_ThiS_adenylyltransferase	RbromiiATCC27255
Org3_Gene145	core	4411	[RbromiiATCC27255]RBATCC27255_00150_50S_ribosomal_protein_L34	RbromiiATCC27255
Org3_Gene1451	core	926	[RbromiiATCC27255]RBATCC27255_01497_putative_ion_channel_protein	RbromiiATCC27255
Org3_Gene1456	core	990	[RbromiiATCC27255]RBATCC27255_01502_Aldehyde-alcohol_dehydrogenase	RbromiiATCC27255
Org3_Gene1458	core	981	[RbromiiATCC27255]RBATCC27255_01504_3-oxoacyl-[acyl-carrier-protein]_synthase_2	RbromiiATCC27255
Org3_Gene1459	core	461	[RbromiiATCC27255]RBATCC27255_01505_Long-chain-fatty-acid--CoA_ligase	RbromiiATCC27255
Org3_Gene146	core	697	[RbromiiATCC27255]RBATCC27255_00151_Chromosomal_replication_initiator_protein_DnaA	RbromiiATCC27255
Org3_Gene1461	core	3504	[RbromiiATCC27255]RBATCC27255_01507_Nitrogen_regulatory_protein_P-II	RbromiiATCC27255
Org3_Gene1462	core	938	[RbromiiATCC27255]RBATCC27255_01508_Ammonia_transporter	RbromiiATCC27255
Org3_Gene1463	core	537	[RbromiiATCC27255]RBATCC27255_01509_Ribonuclease_Y	RbromiiATCC27255
Org3_Gene1464	core	1352	[RbromiiATCC27255]RBATCC27255_01510_Glyceraldehyde-3-phosphate_dehydrogenase	RbromiiATCC27255
Org3_Gene1466	core	999	[RbromiiATCC27255]RBATCC27255_01512_Glucose-1-phosphate_adenylyltransferase	RbromiiATCC27255
Org3_Gene1467	core	1212	[RbromiiATCC27255]RBATCC27255_01513_Glycogen_biosynthesis_protein_GlgD	RbromiiATCC27255
Org3_Gene1468	core	657	[RbromiiATCC27255]RBATCC27255_01514_Glycogen_synthase	RbromiiATCC27255
Org3_Gene147	core	1224	[RbromiiATCC27255]RBATCC27255_00152_DNA_polymerase_III_subunit_beta	RbromiiATCC27255
Org3_Gene1472	core	1664	[RbromiiATCC27255]RBATCC27255_01520_LPS_biosynthesis_protein	RbromiiATCC27255
Org3_Gene1473	core	1262	[RbromiiATCC27255]RBATCC27255_01521_N-glycosyltransferase	RbromiiATCC27255
Org3_Gene1475	core	743	[RbromiiATCC27255]RBATCC27255_01523_Beta-Ala-Xaa_dipeptidase	RbromiiATCC27255
Org3_Gene1477	core	2744	[RbromiiATCC27255]RBATCC27255_01525_5'-nucleotidase_YfbR	RbromiiATCC27255
Org3_Gene1478	core	1069	[RbromiiATCC27255]RBATCC27255_01526_Putative_transcriptional_regulator_YvhJ	RbromiiATCC27255
Org3_Gene148	core	4166	[RbromiiATCC27255]RBATCC27255_00153_ribosome-associated_protein	RbromiiATCC27255
Org3_Gene1482	core	1649	[RbromiiATCC27255]RBATCC27255_01530_Quinolinate_synthase_A	RbromiiATCC27255
Org3_Gene1483	core	1274	[RbromiiATCC27255]RBATCC27255_01531_tRNA-specific_2-thiouridylase_MnmA	RbromiiATCC27255
Org3_Gene149	core	1180	[RbromiiATCC27255]RBATCC27255_00154_DNA_replication_and_repair_protein_RecF	RbromiiATCC27255
Org3_Gene15	core	1017	[RbromiiATCC27255]RBATCC27255_00017_UDP-4-amino-4-deoxy-L-arabinose--oxoglutarate_aminotransferase	RbromiiATCC27255
Org3_Gene150	core	3932	[RbromiiATCC27255]RBATCC27255_00155_hypothetical_protein	RbromiiATCC27255
Org3_Gene1506	core	2912	[RbromiiATCC27255]RBATCC27255_01555_NADH_peroxidase	RbromiiATCC27255
Org3_Gene1508	core	2169	[RbromiiATCC27255]RBATCC27255_01557_Exodeoxyribonuclease	RbromiiATCC27255
Org3_Gene1512	core	1408	[RbromiiATCC27255]RBATCC27255_01561_hypothetical_protein	RbromiiATCC27255
Org3_Gene1514	core	3035	[RbromiiATCC27255]RBATCC27255_01563_Crossover_junction_endodeoxyribonuclease_RuvC	RbromiiATCC27255
Org3_Gene1515	core	2683	[RbromiiATCC27255]RBATCC27255_01564_Holliday_junction_ATP-dependent_DNA_helicase_RuvA	RbromiiATCC27255
Org3_Gene1516	core	1247	[RbromiiATCC27255]RBATCC27255_01565_Holliday_junction_ATP-dependent_DNA_helicase_RuvB	RbromiiATCC27255
Org3_Gene1517	core	761	[RbromiiATCC27255]RBATCC27255_01566_Phosphoglucosamine_mutase	RbromiiATCC27255

Org3_Gene1518	core	1821	[RbromiiATCC27255]RBATCC27255_01567_Ribosomal_RNA_large_subunit_methyltransferase_K	RbromiiATCC27255
Org3_Gene1519	core	1885	[RbromiiATCC27255]RBATCC27255_01568_Energy-coupling_factor_transporter_ATP-binding_protein_EcfA2	RbromiiATCC27255
Org3_Gene152	core	256	[RbromiiATCC27255]RBATCC27255_00157_DNA_gyrase_subunit_B	RbromiiATCC27255
Org3_Gene1520	core	1805	[RbromiiATCC27255]RBATCC27255_01569_Energy-coupling_factor_transporter_ATP-binding_protein_EcfA2	RbromiiATCC27255
Org3_Gene1521	core	1998	[RbromiiATCC27255]RBATCC27255_01570_Energy-coupling_factor_transporter_transmembrane_protein_EcfT	RbromiiATCC27255
Org3_Gene1522	core	2028	[RbromiiATCC27255]RBATCC27255_01571_tRNA_pseudouridine_synthase_A	RbromiiATCC27255
Org3_Gene1526	core	174	[RbromiiATCC27255]RBATCC27255_01575_Heat_shock_protein_HSP1	RbromiiATCC27255
Org3_Gene1527	core	1768	[RbromiiATCC27255]RBATCC27255_01576_L-arabinose_transport_system_permease_protein_AraQ	RbromiiATCC27255
Org3_Gene1528	core	1769	[RbromiiATCC27255]RBATCC27255_01577_L-arabinose_transport_system_permease_protein_AraP	RbromiiATCC27255
Org3_Gene1529	core	746	[RbromiiATCC27255]RBATCC27255_01578_Maltose-binding_periplasmic_proteins/domains	RbromiiATCC27255
Org3_Gene1531	core	413	[RbromiiATCC27255]RBATCC27255_01580_Phosphoglucomutase	RbromiiATCC27255
Org3_Gene1532	core	2897	[RbromiiATCC27255]RBATCC27255_01581_N-acetylmuramoyl-L-alanine_amidase_CwlD	RbromiiATCC27255
Org3_Gene1533	core	2500	[RbromiiATCC27255]RBATCC27255_01582_hypothetical_protein	RbromiiATCC27255
Org3_Gene1535	core	818	[RbromiiATCC27255]RBATCC27255_01584_Ribosomal_RNA_small_subunit_methyltransferase_F	RbromiiATCC27255
Org3_Gene1536	core	1855	[RbromiiATCC27255]RBATCC27255_01585_Ribosomal_RNA_small_subunit_methyltransferase_A	RbromiiATCC27255
Org3_Gene1538	core	124	[RbromiiATCC27255]RBATCC27255_01588_Calcium-transporting_ATPase	RbromiiATCC27255
Org3_Gene154	core	128	[RbromiiATCC27255]RBATCC27255_00159_DNA_gyrase_subunit_A	RbromiiATCC27255
Org3_Gene1541	core	2927	[RbromiiATCC27255]RBATCC27255_01591_Inorganic_pyrophosphatase	RbromiiATCC27255
Org3_Gene1543	core	1923	[RbromiiATCC27255]RBATCC27255_01593_Laccase_domain_protein	RbromiiATCC27255
Org3_Gene1544	core	1509	[RbromiiATCC27255]RBATCC27255_01594_Ribosomal_large_subunit_pseudouridine_synthase_C	RbromiiATCC27255
Org3_Gene1546	core	603	[RbromiiATCC27255]RBATCC27255_01596_Stage_IV_sporulation_protein_A	RbromiiATCC27255
Org3_Gene1548	core	956	[RbromiiATCC27255]RBATCC27255_01598_Glutaconyl-CoA_decarboxylase_subunit_beta	RbromiiATCC27255
Org3_Gene1551	core	886	[RbromiiATCC27255]RBATCC27255_01601_Putative_aminotransferase/MSMEI_6121	RbromiiATCC27255
Org3_Gene1552	core	663	[RbromiiATCC27255]RBATCC27255_01602_preprotein_translocase_subunit_SecD	RbromiiATCC27255
Org3_Gene1553	core	1378	[RbromiiATCC27255]RBATCC27255_01603_preprotein_translocase_subunit_SecF	RbromiiATCC27255
Org3_Gene1559	core	622	[RbromiiATCC27255]RBATCC27255_01609_Spore_germination_protein_B1	RbromiiATCC27255
Org3_Gene1562	core	1495	[RbromiiATCC27255]RBATCC27255_01612_HPr_kinase/phosphorylase	RbromiiATCC27255
Org3_Gene1563	core	1679	[RbromiiATCC27255]RBATCC27255_01613_UDP-N-acetylenolpyruvoylglucosamine_reductase	RbromiiATCC27255
Org3_Gene1564	core	1818	[RbromiiATCC27255]RBATCC27255_01614_glmZ(sRNA)-inactivating_NTPase	RbromiiATCC27255
Org3_Gene1565	core	1590	[RbromiiATCC27255]RBATCC27255_01615_Putative_sporulation_transcription_regulator_WhiA	RbromiiATCC27255
Org3_Gene1567	core	1774	[RbromiiATCC27255]RBATCC27255_01617_Farnesyl_diphosphate_synthase	RbromiiATCC27255
Org3_Gene1568	core	340	[RbromiiATCC27255]RBATCC27255_01618_1-deoxy-D-xylulose-5-phosphate_synthase	RbromiiATCC27255
Org3_Gene1569	core	2015	[RbromiiATCC27255]RBATCC27255_01619_Hemolysin_A	RbromiiATCC27255
Org3_Gene1572	core	457	[RbromiiATCC27255]RBATCC27255_01622_Recombination_protein_N	RbromiiATCC27255
Org3_Gene1577	core	212	[RbromiiATCC27255]RBATCC27255_01627_GTP_pyrophosphokinase	RbromiiATCC27255
Org3_Gene1578	core	3237	[RbromiiATCC27255]RBATCC27255_01628_D-tyrosyl-tRNA(Tyr)_deacylase	RbromiiATCC27255
Org3_Gene1579	core	2613	[RbromiiATCC27255]RBATCC27255_01629_putative_polyketide_biosynthesis_zinc-dependent_hydrolase_PksB	RbromiiATCC27255
Org3_Gene1580	core	624	[RbromiiATCC27255]RBATCC27255_01630_Oxygen-independent_coproporphyrinogen-III_oxidase_2	RbromiiATCC27255
Org3_Gene1583	core	2036	[RbromiiATCC27255]RBATCC27255_01633_Nucleoside_triphosphate_pyrophosphohydrolase	RbromiiATCC27255
Org3_Gene1584	core	3918	[RbromiiATCC27255]RBATCC27255_01634_hypothetical_protein	RbromiiATCC27255
Org3_Gene1585	core	4042	[RbromiiATCC27255]RBATCC27255_01635_ribosome-associated_heat_shock_protein_Hsp15	RbromiiATCC27255
Org3_Gene1586	core	3939	[RbromiiATCC27255]RBATCC27255_01636_Spore_protein_YabP	RbromiiATCC27255
Org3_Gene1589	core	3096	[RbromiiATCC27255]RBATCC27255_01639_General_stress_protein_13	RbromiiATCC27255
Org3_Gene1590	core	2936	[RbromiiATCC27255]RBATCC27255_01640_SigL_modulation_protein	RbromiiATCC27255
Org3_Gene1591	core	1128	[RbromiiATCC27255]RBATCC27255_01641_Ribosomal_RNA_large_subunit_methyltransferase_L	RbromiiATCC27255
Org3_Gene1593	core	2771	[RbromiiATCC27255]RBATCC27255_01643_putative_HD-superfamily_hydrolase	RbromiiATCC27255
Org3_Gene1594	core	283	[RbromiiATCC27255]RBATCC27255_01644_DNA_ligase	RbromiiATCC27255
Org3_Gene1599	core	4250	[RbromiiATCC27255]RBATCC27255_01649_stage_III_sporulation_protein_AC	RbromiiATCC27255
Org3_Gene16	core	3922	[RbromiiATCC27255]RBATCC27255_00018_14_kDa_transcription_factor	RbromiiATCC27255
Org3_Gene1603	core	3249	[RbromiiATCC27255]RBATCC27255_01653_stage_III_sporulation_protein_AG	RbromiiATCC27255
Org3_Gene1606	core	4231	[RbromiiATCC27255]RBATCC27255_01656_Ribosomal_protein_A	RbromiiATCC27255
Org3_Gene1607	core	3634	[RbromiiATCC27255]RBATCC27255_01657_50S_ribosomal_protein_L20	RbromiiATCC27255
Org3_Gene161	core	3264	[RbromiiATCC27255]RBATCC27255_00166_Deoxyuridine_5'-triphosphate_nucleotidohydrolase	RbromiiATCC27255
Org3_Gene1610	core	249	[RbromiiATCC27255]RBATCC27255_01660_DNA_topoisomerase_1	RbromiiATCC27255
Org3_Gene1611	core	828	[RbromiiATCC27255]RBATCC27255_01661_Methylenetetrahydrofolate--tRNA-(uracil-5-)-methyltransferase_TrmFO	RbromiiATCC27255
Org3_Gene1612	core	1426	[RbromiiATCC27255]RBATCC27255_01662_Phosphate_acyltransferase	RbromiiATCC27255
Org3_Gene1613	core	2444	[RbromiiATCC27255]RBATCC27255_01663_Ribonuclease_3	RbromiiATCC27255
Org3_Gene1615	core	47	[RbromiiATCC27255]RBATCC27255_01665_Chromosome_partition_protein_Smc	RbromiiATCC27255
Org3_Gene1616	core	1644	[RbromiiATCC27255]RBATCC27255_01666_Signal_recognition_particle_receptor_FtsY	RbromiiATCC27255
Org3_Gene1617	core	2674	[RbromiiATCC27255]RBATCC27255_01667_Non-canonical_purine_NTP_pyrophosphatase	RbromiiATCC27255
Org3_Gene1618	core	3821	[RbromiiATCC27255]RBATCC27255_01668_RNA-binding_protein	RbromiiATCC27255
Org3_Gene162	core	1398	[RbromiiATCC27255]RBATCC27255_00167_Rod_shape-determining_protein_MreB	RbromiiATCC27255
Org3_Gene1621	core	3626	[RbromiiATCC27255]RBATCC27255_01671_Ribosomal_silencing_factor_RsfS	RbromiiATCC27255
Org3_Gene1622	core	155	[RbromiiATCC27255]RBATCC27255_01672_Leucine--tRNA_ligase	RbromiiATCC27255
Org3_Gene1623	core	4323	[RbromiiATCC27255]RBATCC27255_01673_30S_ribosomal_protein_S21	RbromiiATCC27255
Org3_Gene1624	core	3327	[RbromiiATCC27255]RBATCC27255_01674_3-dehydroquinate_dehydratase	RbromiiATCC27255
Org3_Gene1626	core	2854	[RbromiiATCC27255]RBATCC27255_01676_Elongation_factor_P	RbromiiATCC27255
Org3_Gene1629	core	673	[RbromiiATCC27255]RBATCC27255_01679_(Dimethylallyl)adenosine_tRNA_methylthiotransferase_MiaB	RbromiiATCC27255
Org3_Gene1630	core	3428	[RbromiiATCC27255]RBATCC27255_01680_hypothetical_protein	RbromiiATCC27255
Org3_Gene1631	core	127	[RbromiiATCC27255]RBATCC27255_01681_DNA_mismatch_repair_protein_MutS	RbromiiATCC27255
Org3_Gene1635	core	2317	[RbromiiATCC27255]RBATCC27255_01685_alanine_racemase	RbromiiATCC27255
Org3_Gene1636	core	3192	[RbromiiATCC27255]RBATCC27255_01686_Cell_division_protein_SepF	RbromiiATCC27255
Org3_Gene1639	core	102	[RbromiiATCC27255]RBATCC27255_01689_Isoleucine--tRNA_ligase	RbromiiATCC27255
Org3_Gene1640	core	3126	[RbromiiATCC27255]RBATCC27255_01690_Lipoprotein_signal_peptidase	RbromiiATCC27255
Org3_Gene1641	core	1638	[RbromiiATCC27255]RBATCC27255_01691_Ribosomal_large_subunit_pseudouridine_synthase_D	RbromiiATCC27255
Org3_Gene1642	core	3645	[RbromiiATCC27255]RBATCC27255_01692_50S_ribosomal_protein_L19	RbromiiATCC27255
Org3_Gene1645	core	1771	[RbromiiATCC27255]RBATCC27255_01695_Ribosome_biogenesis_GTPase_A	RbromiiATCC27255
Org3_Gene1646	core	2656	[RbromiiATCC27255]RBATCC27255_01696_Ribonuclease_HII	RbromiiATCC27255
Org3_Gene1649	core	574	[RbromiiATCC27255]RBATCC27255_01699_Threonine_synthase	RbromiiATCC27255
Org3_Gene1650	core	2412	[RbromiiATCC27255]RBATCC27255_01700_putative_phosphoesterase_or_phosphohydrolase	RbromiiATCC27255
Org3_Gene1651	core	890	[RbromiiATCC27255]RBATCC27255_01701_Trigger_factor	RbromiiATCC27255
Org3_Gene1652	core	2763	[RbromiiATCC27255]RBATCC27255_01702_ATP-dependent_Clp_protease_proteolytic_subunit	RbromiiATCC27255
Org3_Gene1653	core	781	[RbromiiATCC27255]RBATCC27255_01703_ATP-dependent_Clp_protease_ATP-binding_subunit_ClpX	RbromiiATCC27255
Org3_Gene1654	core	156	[RbromiiATCC27255]RBATCC27255_01704_Lon_protease_1	RbromiiATCC27255
Org3_Gene1655	core	2688	[RbromiiATCC27255]RBATCC27255_01705_putative_GTP-binding_protein_EngB	RbromiiATCC27255
Org3_Gene1656	core	857	[RbromiiATCC27255]RBATCC27255_01706_Diaminopimelate_decarboxylase	RbromiiATCC27255
Org3_Gene1657	core	2370	[RbromiiATCC27255]RBATCC27255_01707_putative_membrane_protein	RbromiiATCC27255
Org3_Gene166	core	2226	[RbromiiATCC27255]RBATCC27255_00171_Cell_division_inhibitor_MinD	RbromiiATCC27255
Org3_Gene167	core	3475	[RbromiiATCC27255]RBATCC27255_00172_Methylglyoxal_synthase	RbromiiATCC27255
Org3_Gene1671	core	1958	[RbromiiATCC27255]RBATCC27255_01721_antiporter_inner_membrane_protein	RbromiiATCC27255
Org3_Gene1675	core	3541	[RbromiiATCC27255]RBATCC27255_01725_Ribosome-binding_factor_A	RbromiiATCC27255
Org3_Gene1676	core	165	[RbromiiATCC27255]RBATCC27255_01726_Translation_initiation_factor_IF-2	RbromiiATCC27255
Org3_Gene1679	core	1117	[RbromiiATCC27255]RBATCC27255_01729_hypothetical_protein	RbromiiATCC27255
Org3_Gene168	core	805	[RbromiiATCC27255]RBATCC27255_00173_Histidine--tRNA_ligase	RbromiiATCC27255
Org3_Gene1680	core	2658	[RbromiiATCC27255]RBATCC27255_01730_Ribosome_maturation_factor_RimP	RbromiiATCC27255
Org3_Gene1683	core	4243	[RbromiiATCC27255]RBATCC27255_01734_anaerobic_benzoate_catabolism_transcriptional_regulator	RbromiiATCC27255
Org3_Gene1684	core	2401	[RbromiiATCC27255]RBATCC27255_01735_hypothetical_protein	RbromiiATCC27255
Org3_Gene1685	core	1419	[RbromiiATCC27255]RBATCC27255_01736_hypothetical_protein	RbromiiATCC27255
Org3_Gene1687	core	154	[RbromiiATCC27255]RBATCC27255_01738_Methionine_synthase	RbromiiATCC27255
Org3_Gene1689	core	1832	[RbromiiATCC27255]RBATCC27255_01740_5,10-methylenetetrahydrofolate_reductase	RbromiiATCC27255
Org3_Gene169	core	378	[RbromiiATCC27255]RBATCC27255_00174_Aspartate--tRNA_ligase	RbromiiATCC27255
Org3_Gene1690	core	171	[RbromiiATCC27255]RBATCC27255_01741_Glycogen_phosphorylase	RbromiiATCC27255
Org3_Gene1691	core	590	[RbromiiATCC27255]RBATCC27255_01742_4-alpha-glucanotransferase	RbromiiATCC27255
Org3_Gene1692	core	2857	[RbromiiATCC27255]RBATCC27255_01743_Nicotinamide-nucleotide_amidohydrolase_PncC	RbromiiATCC27255
Org3_Gene1693	core	2255	[RbromiiATCC27255]RBATCC27255_01744_Ribosomal_RNA_small_subunit_methyltransferase_E	RbromiiATCC27255
Org3_Gene1694	core	2393	[RbromiiATCC27255]RBATCC27255_01745_5'-methylthioadenosine/S-adenosylhomocysteine_nucleosidase	RbromiiATCC27255
Org3_Gene1695	core	453	[RbromiiATCC27255]RBATCC27255_01746_Acetolactate_synthase_large_subunit	RbromiiATCC27255
Org3_Gene1696	core	592	[RbromiiATCC27255]RBATCC27255_01747_Uracil_transporter	RbromiiATCC27255
Org3_Gene1700	core	1383	[RbromiiATCC27255]RBATCC27255_01751_Tryptophan--tRNA_ligase	RbromiiATCC27255
Org3_Gene1701	core	1056	[RbromiiATCC27255]RBATCC27255_01752_2-aminoadipate_transaminase	RbromiiATCC27255
Org3_Gene1703	core	2391	[RbromiiATCC27255]RBATCC27255_01754_Putative_mutator_protein_MutT4	RbromiiATCC27255
Org3_Gene1705	core	2166	[RbromiiATCC27255]RBATCC27255_01756_Putative_TrmH_family_tRNA/rRNA_methyltransferase	RbromiiATCC27255
Org3_Gene1707	core	2522	[RbromiiATCC27255]RBATCC27255_01758_Ribulose-phosphate_3-epimerase	RbromiiATCC27255
Org3_Gene171	core	951	[RbromiiATCC27255]RBATCC27255_00176_Putative_virion_core_protein_(lumpy_skin_disease_virus)	RbromiiATCC27255
Org3_Gene1718	core	2793	[RbromiiATCC27255]RBATCC27255_01769_dTDP-4-dehydrorhamnose_3,5-epimerase	RbromiiATCC27255
Org3_Gene1719	core	1601	[RbromiiATCC27255]RBATCC27255_01770_GDP-6-deoxy-D-mannose_reductase	RbromiiATCC27255
Org3_Gene1720	core	1311	[RbromiiATCC27255]RBATCC27255_01771_CDP-glucose_4,6-dehydratase	RbromiiATCC27255
Org3_Gene1721	core	2109	[RbromiiATCC27255]RBATCC27255_01772_Glucose-1-phosphate_cytidylyltransferase	RbromiiATCC27255
Org3_Gene1728	core	2111	[RbromiiATCC27255]RBATCC27255_01779_putative_deoxyribonuclease_YcfH	RbromiiATCC27255
Org3_Gene1729	core	297	[RbromiiATCC27255]RBATCC27255_01780_Methionine--tRNA_ligase	RbromiiATCC27255
Org3_Gene1737	core	1439	[RbromiiATCC27255]RBATCC27255_01788_putative_tRNA-dihydrouridine_synthase	RbromiiATCC27255
Org3_Gene1738	core	931	[RbromiiATCC27255]RBATCC27255_01789_Serine_hydroxymethyltransferase	RbromiiATCC27255

Org3_Gene175	core	648	[RbromiiATCC27255]RBATCC27255_00180_2-iminoacetate_synthase	RbromiiATCC27255
Org3_Gene1757	core	641	[RbromiiATCC27255]RBATCC27255_01809_Nicotinate_phosphoribosyltransferase_pncB2	RbromiiATCC27255
Org3_Gene1758	core	553	[RbromiiATCC27255]RBATCC27255_01810_Trehalose_synthase/amylase_TreS	RbromiiATCC27255
Org3_Gene1763	core	112	[RbromiiATCC27255]RBATCC27255_01815_Valine--tRNA_ligase	RbromiiATCC27255
Org3_Gene1767	core	3350	[RbromiiATCC27255]RBATCC27255_01819_Putative_Holliday_junction_resolvase	RbromiiATCC27255
Org3_Gene177	core	600	[RbromiiATCC27255]RBATCC27255_00182_Inosine-5'-monophosphate_dehydrogenase	RbromiiATCC27255
Org3_Gene1771	core	639	[RbromiiATCC27255]RBATCC27255_01823_Pyruvate_kinase	RbromiiATCC27255
Org3_Gene1773	core	1155	[RbromiiATCC27255]RBATCC27255_01825_P-protein	RbromiiATCC27255
Org3_Gene1774	core	1278	[RbromiiATCC27255]RBATCC27255_01826_Chorismate_synthase	RbromiiATCC27255
Org3_Gene1775	core	859	[RbromiiATCC27255]RBATCC27255_01827_3-phosphoshikimate_1-carboxyvinyltransferase	RbromiiATCC27255
Org3_Gene1776	core	1306	[RbromiiATCC27255]RBATCC27255_01828_3-dehydroquinate_synthase	RbromiiATCC27255
Org3_Gene1777	core	1936	[RbromiiATCC27255]RBATCC27255_01829_Arogenate_dehydrogenase	RbromiiATCC27255
Org3_Gene1778	core	1379	[RbromiiATCC27255]RBATCC27255_01830_Phospho-2-dehydro-3-deoxyheptonate_aldolase	RbromiiATCC27255
Org3_Gene178	core	1533	[RbromiiATCC27255]RBATCC27255_00183_Glucokinase	RbromiiATCC27255
Org3_Gene1780	core	1884	[RbromiiATCC27255]RBATCC27255_01832_Shikimate_dehydrogenase	RbromiiATCC27255
Org3_Gene1781	core	4101	[RbromiiATCC27255]RBATCC27255_01833_Acyl_carrier_protein	RbromiiATCC27255
Org3_Gene1782	core	644	[RbromiiATCC27255]RBATCC27255_01834_Proline--tRNA_ligase	RbromiiATCC27255
Org3_Gene1784	core	1602	[RbromiiATCC27255]RBATCC27255_01836_Homoserine_O-succinyltransferase	RbromiiATCC27255
Org3_Gene1785	core	1993	[RbromiiATCC27255]RBATCC27255_01837_Glutamate_5-kinase_1	RbromiiATCC27255
Org3_Gene1786	core	945	[RbromiiATCC27255]RBATCC27255_01838_Gamma-glutamyl_phosphate_reductase	RbromiiATCC27255
Org3_Gene1792	core	1810	[RbromiiATCC27255]RBATCC27255_01844_hypothetical_protein	RbromiiATCC27255
Org3_Gene1793	core	4343	[RbromiiATCC27255]RBATCC27255_01845_Ferredoxin	RbromiiATCC27255
Org3_Gene1795	core	1955	[RbromiiATCC27255]RBATCC27255_01847_Ribosomal_RNA_small_subunit_methyltransferase_I	RbromiiATCC27255
Org3_Gene1797	core	3765	[RbromiiATCC27255]RBATCC27255_01849_Stage_II_sporulation_protein_AA	RbromiiATCC27255
Org3_Gene1798	core	3296	[RbromiiATCC27255]RBATCC27255_01850_Anti-sigma_F_factor	RbromiiATCC27255
Org3_Gene1799	core	2501	[RbromiiATCC27255]RBATCC27255_01851_Stage_II_sporulation_protein_AC	RbromiiATCC27255
Org3_Gene1802	core	120	[RbromiiATCC27255]RBATCC27255_01855_Pyruvate,_phosphate_dikinase	RbromiiATCC27255
Org3_Gene1803	core	2182	[RbromiiATCC27255]RBATCC27255_01856_Bifunctional_protein_GlmU	RbromiiATCC27255
Org3_Gene1804	core	1519	[RbromiiATCC27255]RBATCC27255_01857_Ribose-phosphate_pyrophosphokinase	RbromiiATCC27255
Org3_Gene1806	core	2720	[RbromiiATCC27255]RBATCC27255_01859_Peptidyl-tRNA_hydrolase	RbromiiATCC27255
Org3_Gene1807	core	56	[RbromiiATCC27255]RBATCC27255_01860_Transcription-repair-coupling_factor	RbromiiATCC27255
Org3_Gene1810	core	4115	[RbromiiATCC27255]RBATCC27255_01863_hypothetical_protein	RbromiiATCC27255
Org3_Gene1811	core	415	[RbromiiATCC27255]RBATCC27255_01864_MutS2_protein	RbromiiATCC27255
Org3_Gene1818	core	4093	[RbromiiATCC27255]RBATCC27255_01871_hypothetical_protein	RbromiiATCC27255
Org3_Gene1819	core	996	[RbromiiATCC27255]RBATCC27255_01872_Acetate_kinase	RbromiiATCC27255
Org3_Gene182	core	3339	[RbromiiATCC27255]RBATCC27255_00189_50S_ribosomal_protein_L13	RbromiiATCC27255
Org3_Gene1822	core	123	[RbromiiATCC27255]RBATCC27255_01875_Aldehyde-alcohol_dehydrogenase	RbromiiATCC27255
Org3_Gene1824	core	1782	[RbromiiATCC27255]RBATCC27255_01877_Germination_protease_precursor	RbromiiATCC27255
Org3_Gene1827	core	1129	[RbromiiATCC27255]RBATCC27255_01880_tRNA_2-thiocytidine_biosynthesis_protein_TtcA	RbromiiATCC27255
Org3_Gene1829	core	636	[RbromiiATCC27255]RBATCC27255_01882_Trk_system_potassium_uptake_protein_TrkG	RbromiiATCC27255
Org3_Gene183	core	3447	[RbromiiATCC27255]RBATCC27255_00190_30S_ribosomal_protein_S9	RbromiiATCC27255
Org3_Gene1832	core	50	[RbromiiATCC27255]RBATCC27255_01885_Pyruvate-flavodoxin_oxidoreductase	RbromiiATCC27255
Org3_Gene1833	core	2876	[RbromiiATCC27255]RBATCC27255_01886_undecaprenyl_pyrophosphate_phosphatase	RbromiiATCC27255
Org3_Gene1834	core	1402	[RbromiiATCC27255]RBATCC27255_01887_Stage_V_sporulation_protein_AD	RbromiiATCC27255
Org3_Gene1841	core	626	[RbromiiATCC27255]RBATCC27255_01894_Glutamate--tRNA_ligase	RbromiiATCC27255
Org3_Gene1842	core	434	[RbromiiATCC27255]RBATCC27255_01895_Glutamine--tRNA_ligase	RbromiiATCC27255
Org3_Gene1843	core	2221	[RbromiiATCC27255]RBATCC27255_01896_3-oxoacyl-[acyl-carrier-protein]_reductase_FabG	RbromiiATCC27255
Org3_Gene1844	core	607	[RbromiiATCC27255]RBATCC27255_01897_UDP-N-acetylmuramoyl-L-alanyl-D-glutamate--L-lysine_ligase	RbromiiATCC27255
Org3_Gene1846	core	581	[RbromiiATCC27255]RBATCC27255_01899_Maltose_transport_system_permease_protein_MalF	RbromiiATCC27255
Org3_Gene1847	core	1824	[RbromiiATCC27255]RBATCC27255_01900_Maltose_transport_system_permease_protein_MalG	RbromiiATCC27255
Org3_Gene1848	core	460	[RbromiiATCC27255]RBATCC27255_01901_Alpha-amylase_precursor	RbromiiATCC27255
Org3_Gene1851	core	3378	[RbromiiATCC27255]RBATCC27255_01904_30S_ribosomal_protein_S12	RbromiiATCC27255
Org3_Gene1852	core	2922	[RbromiiATCC27255]RBATCC27255_01905_30S_ribosomal_protein_S7	RbromiiATCC27255
Org3_Gene1853	core	240	[RbromiiATCC27255]RBATCC27255_01906_Vegetative_protein_19	RbromiiATCC27255
Org3_Gene1854	core	1023	[RbromiiATCC27255]RBATCC27255_01907_Elongation_factor_Tu	RbromiiATCC27255
Org3_Gene1860	core	189	[RbromiiATCC27255]RBATCC27255_01913_1,4-alpha-glucan_branching_enzyme_GlgB	RbromiiATCC27255
Org3_Gene1861	core	416	[RbromiiATCC27255]RBATCC27255_01914_putative_membrane_protein	RbromiiATCC27255
Org3_Gene1862	core	1671	[RbromiiATCC27255]RBATCC27255_01915_NTE_family_protein_RssA	RbromiiATCC27255
Org3_Gene1864	core	4312	[RbromiiATCC27255]RBATCC27255_01917_hypothetical_protein	RbromiiATCC27255
Org3_Gene1865	core	1276	[RbromiiATCC27255]RBATCC27255_01918_Peptide_chain_release_factor_1	RbromiiATCC27255
Org3_Gene1866	core	1337	[RbromiiATCC27255]RBATCC27255_01919_t(6)A37_threonylcarbamoyladenosine_biosynthesis_protein_RimN	RbromiiATCC27255
Org3_Gene1869	core	675	[RbromiiATCC27255]RBATCC27255_01922_putative_M18_family_aminopeptidase_1	RbromiiATCC27255
Org3_Gene1873	core	2506	[RbromiiATCC27255]RBATCC27255_01927_Phosphorylated_carbohydrates_phosphatase	RbromiiATCC27255
Org3_Gene1876	core	371	[RbromiiATCC27255]RBATCC27255_01930_Dihydroxy-acid_dehydratase	RbromiiATCC27255
Org3_Gene1877	core	258	[RbromiiATCC27255]RBATCC27255_01931_Elongation_factor_G	RbromiiATCC27255
Org3_Gene1879	core	2815	[RbromiiATCC27255]RBATCC27255_01933_hypothetical_protein	RbromiiATCC27255
Org3_Gene1880	core	2411	[RbromiiATCC27255]RBATCC27255_01934_hypothetical_protein	RbromiiATCC27255
Org3_Gene1881	core	4026	[RbromiiATCC27255]RBATCC27255_01935_hypothetical_protein	RbromiiATCC27255
Org3_Gene1883	core	1730	[RbromiiATCC27255]RBATCC27255_01937_hypothetical_protein	RbromiiATCC27255
Org3_Gene1885	core	3832	[RbromiiATCC27255]RBATCC27255_01939_hypothetical_protein	RbromiiATCC27255
Org3_Gene1886	core	2103	[RbromiiATCC27255]RBATCC27255_01940_Stage_II_sporulation_protein_AC	RbromiiATCC27255
Org3_Gene1894	core	687	[RbromiiATCC27255]RBATCC27255_01948_Methylmalonyl-CoA_carboxyltransferase_5S_subunit	RbromiiATCC27255
Org3_Gene1895	core	3572	[RbromiiATCC27255]RBATCC27255_01949_Glutaconyl-CoA_decarboxylase_subunit_gamma	RbromiiATCC27255
Org3_Gene1896	core	839	[RbromiiATCC27255]RBATCC27255_01950_Methylmalonyl-CoA_carboxyltransferase_12S_subunit	RbromiiATCC27255
Org3_Gene1897	core	2196	[RbromiiATCC27255]RBATCC27255_01951_Ribosomal_large_subunit_pseudouridine_synthase_B	RbromiiATCC27255
Org3_Gene1899	core	3344	[RbromiiATCC27255]RBATCC27255_01953_putative_spore_protein_YtfJ	RbromiiATCC27255
Org3_Gene19	core	1834	[RbromiiATCC27255]RBATCC27255_00022_Nicotinamide_nucleotide_repair_protein	RbromiiATCC27255
Org3_Gene1901	core	2631	[RbromiiATCC27255]RBATCC27255_01955_Segregation_and_condensation_protein_B	RbromiiATCC27255
Org3_Gene1902	core	2124	[RbromiiATCC27255]RBATCC27255_01956_Segregation_and_condensation_protein_A	RbromiiATCC27255
Org3_Gene1904	core	4278	[RbromiiATCC27255]RBATCC27255_01958_50S_ribosomal_protein_L28	RbromiiATCC27255
Org3_Gene1909	core	1397	[RbromiiATCC27255]RBATCC27255_01963_UDP-glucose_4-epimerase	RbromiiATCC27255
Org3_Gene1910	core	2990	[RbromiiATCC27255]RBATCC27255_01964_Peptidyl-prolyl_cis-trans_isomerase_B	RbromiiATCC27255
Org3_Gene1911	core	1404	[RbromiiATCC27255]RBATCC27255_01965_t(6)A37_threonylcarbamoyladenosine_biosynthesis_protein	RbromiiATCC27255
Org3_Gene1914	core	129	[RbromiiATCC27255]RBATCC27255_01968_DNA_polymerase_I	RbromiiATCC27255
Org3_Gene1915	core	1074	[RbromiiATCC27255]RBATCC27255_01969_Alanine_racemase	RbromiiATCC27255
Org3_Gene1918	core	1145	[RbromiiATCC27255]RBATCC27255_01973_Purine_catabolism_regulatory_protein	RbromiiATCC27255
Org3_Gene1935	core	1902	[RbromiiATCC27255]RBATCC27255_01990_putative_nicotinate-nucleotide_pyrophosphorylase_[carboxylating]	RbromiiATCC27255
Org3_Gene1936	core	611	[RbromiiATCC27255]RBATCC27255_01991_L-aspartate_oxidase	RbromiiATCC27255
Org3_Gene1943	core	1766	[RbromiiATCC27255]RBATCC27255_02000_anaerobic_ribonucleoside-triphosphate_reductase_activating_protein	RbromiiATCC27255
Org3_Gene207	core	3136	[RbromiiATCC27255]RBATCC27255_00219_2,3-dimethylmalate_dehydratase_small_subunit	RbromiiATCC27255
Org3_Gene208	core	918	[RbromiiATCC27255]RBATCC27255_00220_2,3-dimethylmalate_dehydratase_large_subunit	RbromiiATCC27255
Org3_Gene217	core	811	[RbromiiATCC27255]RBATCC27255_00229_2-hydroxyglutaryl-CoA_dehydratase,_D-component	RbromiiATCC27255
Org3_Gene218	core	90	[RbromiiATCC27255]RBATCC27255_00230_2-hydroxyglutaryl-CoA_dehydratase_component_A	RbromiiATCC27255
Org3_Gene22	core	701	[RbromiiATCC27255]RBATCC27255_00025_D-alanyl-lipoteichoic_acid_biosynthesis_protein_DltB	RbromiiATCC27255
Org3_Gene220	core	2387	[RbromiiATCC27255]RBATCC27255_00232_hypothetical_protein	RbromiiATCC27255
Org3_Gene222	core	1605	[RbromiiATCC27255]RBATCC27255_00234_Ornithine_carbamoyltransferase	RbromiiATCC27255
Org3_Gene223	core	1088	[RbromiiATCC27255]RBATCC27255_00235_Acetylornithine_aminotransferase	RbromiiATCC27255
Org3_Gene224	core	3359	[RbromiiATCC27255]RBATCC27255_00236_Acetyltransferase_YpeA	RbromiiATCC27255
Org3_Gene225	core	1865	[RbromiiATCC27255]RBATCC27255_00237_Acetylglutamate_kinase	RbromiiATCC27255
Org3_Gene226	core	973	[RbromiiATCC27255]RBATCC27255_00238_Arginine_biosynthesis_bifunctional_protein_ArgJ	RbromiiATCC27255
Org3_Gene227	core	1349	[RbromiiATCC27255]RBATCC27255_00239_N-acetyl-gamma-glutamyl-phosphate_reductase	RbromiiATCC27255
Org3_Gene228	core	719	[RbromiiATCC27255]RBATCC27255_00240_Argininosuccinate_lyase_1	RbromiiATCC27255
Org3_Gene229	core	983	[RbromiiATCC27255]RBATCC27255_00241_Argininosuccinate_synthase	RbromiiATCC27255
Org3_Gene231	core	3024	[RbromiiATCC27255]RBATCC27255_00243_Flavoredoxin	RbromiiATCC27255
Org3_Gene237	core	1591	[RbromiiATCC27255]RBATCC27255_00249_Ribosomal_protein_L11_methyltransferase	RbromiiATCC27255
Org3_Gene238	core	1153	[RbromiiATCC27255]RBATCC27255_00250_Chaperone_protein_DnaJ	RbromiiATCC27255
Org3_Gene239	core	344	[RbromiiATCC27255]RBATCC27255_00251_Heat_shock_protein_70	RbromiiATCC27255
Org3_Gene245	core	2521	[RbromiiATCC27255]RBATCC27255_00257_LexA_repressor	RbromiiATCC27255
Org3_Gene246	core	2595	[RbromiiATCC27255]RBATCC27255_00258_Uracil_phosphoribosyltransferase	RbromiiATCC27255
Org3_Gene250	core	2359	[RbromiiATCC27255]RBATCC27255_00262_gliding_motility-associated_ABC_transporter_permease_protein_GldF	RbromiiATCC27255
Org3_Gene251	core	1527	[RbromiiATCC27255]RBATCC27255_00263_putative_ABC_transporter_ATP-binding_protein_YxlF	RbromiiATCC27255
Org3_Gene252	core	3621	[RbromiiATCC27255]RBATCC27255_00264_stage_V_sporulation_protein_AE	RbromiiATCC27255
Org3_Gene256	core	2836	[RbromiiATCC27255]RBATCC27255_00268_putative_manganese_efflux_pump_MntP	RbromiiATCC27255
Org3_Gene258	core	1890	[RbromiiATCC27255]RBATCC27255_00270_Dihydroorotate_oxidase_B,_electron_transfer_subunit	RbromiiATCC27255
Org3_Gene259	core	700	[RbromiiATCC27255]RBATCC27255_00271_Glutamate_synthase_[NADPH]_small_chain	RbromiiATCC27255
Org3_Gene26	core	913	[RbromiiATCC27255]RBATCC27255_00029_Replication-associated_recombination_protein_A	RbromiiATCC27255
Org3_Gene260	core	40	[RbromiiATCC27255]RBATCC27255_00272_DNA-directed_RNA_polymerase_subunit_beta	RbromiiATCC27255
Org3_Gene261	core	44	[RbromiiATCC27255]RBATCC27255_00273_DNA-directed_RNA_polymerase_subunit_beta'	RbromiiATCC27255
Org3_Gene265	core	2795	[RbromiiATCC27255]RBATCC27255_00277_Dipicolinate_synthase_subunit_B	RbromiiATCC27255
Org3_Gene270	core	905	[RbromiiATCC27255]RBATCC27255_00282_Methionine_gamma-lyase	RbromiiATCC27255
Org3_Gene271	core	2362	[RbromiiATCC27255]RBATCC27255_00283_2-C-methyl-D-erythritol_4-phosphate_cytidylyltransferase	RbromiiATCC27255
Org3_Gene272	core	3164	[RbromiiATCC27255]RBATCC27255_00284_2-C-methyl-D-erythritol_2,4-cyclodiphosphate_synthase	RbromiiATCC27255

Org3_Gene273	core	1141	[RbromiiATCC27255]RBATCC27255_00285_D-alanyl-D-alanine_carboxypeptidase_DacF_precursor	RbromiiATCC27255
Org3_Gene274	core	787	[RbromiiATCC27255]RBATCC27255_00286_Glucose-6-phosphate_isomerase	RbromiiATCC27255
Org3_Gene275	core	3391	[RbromiiATCC27255]RBATCC27255_00287_hypothetical_protein	RbromiiATCC27255
Org3_Gene28	core	153	[RbromiiATCC27255]RBATCC27255_00031_Beta/alpha-amylase_precursor	RbromiiATCC27255
Org3_Gene290	core	1105	[RbromiiATCC27255]RBATCC27255_00302_H(2)O-forming_NADH_oxidase	RbromiiATCC27255
Org3_Gene301	core	1540	[RbromiiATCC27255]RBATCC27255_00313_putative_glycosyltransferase_YkoT	RbromiiATCC27255
Org3_Gene302	core	1483	[RbromiiATCC27255]RBATCC27255_00314_6-phosphofructokinase	RbromiiATCC27255
Org3_Gene306	core	2379	[RbromiiATCC27255]RBATCC27255_00318_Stage_II_sporulation_protein_GB	RbromiiATCC27255
Org3_Gene308	core	1754	[RbromiiATCC27255]RBATCC27255_00320_DegV_domain-containing_protein	RbromiiATCC27255
Org3_Gene309	core	3854	[RbromiiATCC27255]RBATCC27255_00321_sporulation_protein,_YlmC/YmxH_family	RbromiiATCC27255
Org3_Gene311	core	2264	[RbromiiATCC27255]RBATCC27255_00323_Vegetative_protein_209	RbromiiATCC27255
Org3_Gene312	core	1659	[RbromiiATCC27255]RBATCC27255_00324_Elongation_factor_Ts	RbromiiATCC27255
Org3_Gene314	core	2319	[RbromiiATCC27255]RBATCC27255_00326_Uridylate_kinase	RbromiiATCC27255
Org3_Gene315	core	2866	[RbromiiATCC27255]RBATCC27255_00327_Ribosome-releasing_factor	RbromiiATCC27255
Org3_Gene316	core	2189	[RbromiiATCC27255]RBATCC27255_00328_Ditrans,polycis-undecaprenyl-diphosphate_synthase_((2E,6E)-farnesyl-diphosphate_specific)	RbromiiATCC27255
Org3_Gene318	core	1147	[RbromiiATCC27255]RBATCC27255_00330_1-deoxy-D-xylulose_5-phosphate_reductoisomerase	RbromiiATCC27255
Org3_Gene320	core	1335	[RbromiiATCC27255]RBATCC27255_00332_4-hydroxy-3-methylbut-2-en-1-yl_diphosphate_synthase	RbromiiATCC27255
Org3_Gene321	core	21	[RbromiiATCC27255]RBATCC27255_00333_DNA_polymerase_III_PolC-type	RbromiiATCC27255
Org3_Gene323	core	1098	[RbromiiATCC27255]RBATCC27255_00335_ATP_phosphoribosyltransferase_regulatory_subunit	RbromiiATCC27255
Org3_Gene324	core	2601	[RbromiiATCC27255]RBATCC27255_00336_ATP_phosphoribosyltransferase	RbromiiATCC27255
Org3_Gene325	core	864	[RbromiiATCC27255]RBATCC27255_00337_Histidinol_dehydrogenase	RbromiiATCC27255
Org3_Gene326	core	1314	[RbromiiATCC27255]RBATCC27255_00338_Histidinol-phosphate_aminotransferase	RbromiiATCC27255
Org3_Gene327	core	2773	[RbromiiATCC27255]RBATCC27255_00339_Imidazoleglycerol-phosphate_dehydratase	RbromiiATCC27255
Org3_Gene328	core	2300	[RbromiiATCC27255]RBATCC27255_00340_1-(5-phosphoribosyl)-5-[(5-phosphoribosylamino)methylideneamino]_imidazole-4-carboxamide_isomerase	RbromiiATCC27255
Org3_Gene329	core	3785	[RbromiiATCC27255]RBATCC27255_00341_phosphoribosyl-AMP_cyclohydrolase	RbromiiATCC27255
Org3_Gene330	core	3697	[RbromiiATCC27255]RBATCC27255_00342_Phosphoribosyl-ATP_pyrophosphatase	RbromiiATCC27255
Org3_Gene332	core	869	[RbromiiATCC27255]RBATCC27255_00344_Threonylcarbamoyladenosine_tRNA_methylthiotransferase_MtaB	RbromiiATCC27255
Org3_Gene340	core	1179	[RbromiiATCC27255]RBATCC27255_00352_Queuine_tRNA-ribosyltransferase	RbromiiATCC27255
Org3_Gene341	core	1375	[RbromiiATCC27255]RBATCC27255_00353_S-adenosylmethionine:tRNA_ribosyltransferase-isomerase	RbromiiATCC27255
Org3_Gene342	core	1256	[RbromiiATCC27255]RBATCC27255_00354_Aspartate-semialdehyde_dehydrogenase_2	RbromiiATCC27255
Org3_Gene343	core	1686	[RbromiiATCC27255]RBATCC27255_00355_4-hydroxy-tetrahydrodipicolinate_synthase	RbromiiATCC27255
Org3_Gene344	core	2158	[RbromiiATCC27255]RBATCC27255_00356_4-hydroxy-tetrahydrodipicolinate_reductase	RbromiiATCC27255
Org3_Gene347	core	1734	[RbromiiATCC27255]RBATCC27255_00359_DisA_bacterial_checkpoint_controller_nucleotide-binding_protein	RbromiiATCC27255
Org3_Gene350	core	456	[RbromiiATCC27255]RBATCC27255_00362_Formate--tetrahydrofolate_ligase	RbromiiATCC27255
Org3_Gene351	core	3357	[RbromiiATCC27255]RBATCC27255_00363_ADP-binding_protein	RbromiiATCC27255
Org3_Gene353	core	3252	[RbromiiATCC27255]RBATCC27255_00365_Ribose-5-phosphate_isomerase_B	RbromiiATCC27255
Org3_Gene354	core	4002	[RbromiiATCC27255]RBATCC27255_00366_Helix-turn-helix_domain_protein	RbromiiATCC27255
Org3_Gene36	core	3221	[RbromiiATCC27255]RBATCC27255_00039_SsrA-binding_protein	RbromiiATCC27255
Org3_Gene361	core	520	[RbromiiATCC27255]RBATCC27255_00374_Peptide_chain_release_factor_3	RbromiiATCC27255
Org3_Gene363	core	4381	[RbromiiATCC27255]RBATCC27255_00376_six-cysteine_peptide_SCIFF	RbromiiATCC27255
Org3_Gene364	core	724	[RbromiiATCC27255]RBATCC27255_00377_Anaerobic_sulfatase-maturating_enzyme	RbromiiATCC27255
Org3_Gene366	core	318	[RbromiiATCC27255]RBATCC27255_00379_Ferrous_iron_transport_protein_B	RbromiiATCC27255
Org3_Gene373	core	1789	[RbromiiATCC27255]RBATCC27255_00386_Methionine_aminopeptidase	RbromiiATCC27255
Org3_Gene375	core	734	[RbromiiATCC27255]RBATCC27255_00388_Lon_protease_2	RbromiiATCC27255
Org3_Gene377	core	1460	[RbromiiATCC27255]RBATCC27255_00390_Tyrosine_recombinase_XerD	RbromiiATCC27255
Org3_Gene378	core	796	[RbromiiATCC27255]RBATCC27255_00391_NADP-specific_glutamate_dehydrogenase	RbromiiATCC27255
Org3_Gene38	core	1588	[RbromiiATCC27255]RBATCC27255_00042_orotidine_5'-phosphate_decarboxylase	RbromiiATCC27255
Org3_Gene39	core	1255	[RbromiiATCC27255]RBATCC27255_00043_Phosphoserine_aminotransferase	RbromiiATCC27255
Org3_Gene394	core	4015	[RbromiiATCC27255]RBATCC27255_00407_preprotein_translocase_subunit_SecG	RbromiiATCC27255
Org3_Gene395	core	203	[RbromiiATCC27255]RBATCC27255_00408_DNA_gyrase_subunit_A	RbromiiATCC27255
Org3_Gene396	core	282	[RbromiiATCC27255]RBATCC27255_00409_DNA_gyrase_subunit_B	RbromiiATCC27255
Org3_Gene397	core	290	[RbromiiATCC27255]RBATCC27255_00410_Excinuclease_ABC_subunit_B	RbromiiATCC27255
Org3_Gene398	core	2348	[RbromiiATCC27255]RBATCC27255_00411_DNA_repair_protein_RadC	RbromiiATCC27255
Org3_Gene399	core	2443	[RbromiiATCC27255]RBATCC27255_00412_Orotate_phosphoribosyltransferase	RbromiiATCC27255
Org3_Gene40	core	1125	[RbromiiATCC27255]RBATCC27255_00044_D-3-phosphoglycerate_dehydrogenase	RbromiiATCC27255
Org3_Gene400	core	898	[RbromiiATCC27255]RBATCC27255_00413_DNA_polymerase_IV	RbromiiATCC27255
Org3_Gene401	core	4043	[RbromiiATCC27255]RBATCC27255_00414_hypothetical_protein	RbromiiATCC27255
Org3_Gene407	core	2038	[RbromiiATCC27255]RBATCC27255_00420_Pyrroline-5-carboxylate_reductase	RbromiiATCC27255
Org3_Gene415	core	2509	[RbromiiATCC27255]RBATCC27255_00429_radical_SAM-linked_protein	RbromiiATCC27255
Org3_Gene416	core	346	[RbromiiATCC27255]RBATCC27255_00430_B12-binding_domain/radical_SAM_domain_protein,_family	RbromiiATCC27255
Org3_Gene419	core	690	[RbromiiATCC27255]RBATCC27255_00433_Multidrug_export_protein_MepA	RbromiiATCC27255
Org3_Gene423	core	517	[RbromiiATCC27255]RBATCC27255_00437_flavoprotein,_family	RbromiiATCC27255
Org3_Gene425	core	1085	[RbromiiATCC27255]RBATCC27255_00440_NADP-dependent_malic_enzyme	RbromiiATCC27255
Org3_Gene427	core	2223	[RbromiiATCC27255]RBATCC27255_00442_Arginine_transport_ATP-binding_protein_ArtM	RbromiiATCC27255
Org3_Gene43	core	891	[RbromiiATCC27255]RBATCC27255_00047_phage_terminase,_large_subunit,_PBSX_family	RbromiiATCC27255
Org3_Gene431	core	2136	[RbromiiATCC27255]RBATCC27255_00446_Stage_0_sporulation_protein_A	RbromiiATCC27255
Org3_Gene432	core	914	[RbromiiATCC27255]RBATCC27255_00447_Phosphoribosylamine--glycine_ligase	RbromiiATCC27255
Org3_Gene433	core	1076	[RbromiiATCC27255]RBATCC27255_00448_Bifunctional_purine_biosynthesis_protein_PurH	RbromiiATCC27255
Org3_Gene434	core	2308	[RbromiiATCC27255]RBATCC27255_00449_IMP_cyclohydrolase	RbromiiATCC27255
Org3_Gene435	core	2614	[RbromiiATCC27255]RBATCC27255_00450_Phosphoribosylglycinamide_formyltransferase	RbromiiATCC27255
Org3_Gene436	core	1351	[RbromiiATCC27255]RBATCC27255_00451_Phosphoribosylformylglycinamidine_cyclo-ligase	RbromiiATCC27255
Org3_Gene437	core	633	[RbromiiATCC27255]RBATCC27255_00452_Amidophosphoribosyltransferase_precursor	RbromiiATCC27255
Org3_Gene438	core	3072	[RbromiiATCC27255]RBATCC27255_00453_N5-carboxyaminoimidazole_ribonucleotide_mutase	RbromiiATCC27255
Org3_Gene440	core	1537	[RbromiiATCC27255]RBATCC27255_00455_Modulator_of_FtsH_protease_HflK	RbromiiATCC27255
Org3_Gene459	core	2679	[RbromiiATCC27255]RBATCC27255_00474_Recombination_protein_RecR	RbromiiATCC27255
Org3_Gene460	core	3632	[RbromiiATCC27255]RBATCC27255_00475_Nucleoid-associated_protein	RbromiiATCC27255
Org3_Gene463	core	2452	[RbromiiATCC27255]RBATCC27255_00481_Bifunctional_protein_GlmU	RbromiiATCC27255
Org3_Gene466	core	937	[RbromiiATCC27255]RBATCC27255_00484_GTP-binding_protein_HflX	RbromiiATCC27255
Org3_Gene47	core	1409	[RbromiiATCC27255]RBATCC27255_00051_phage_major_capsid_protein,_HK97_family	RbromiiATCC27255
Org3_Gene474	core	1067	[RbromiiATCC27255]RBATCC27255_00493_S-adenosylmethionine_synthase	RbromiiATCC27255
Org3_Gene475	core	3555	[RbromiiATCC27255]RBATCC27255_00494_Enamine/imine_deaminase	RbromiiATCC27255
Org3_Gene476	core	1043	[RbromiiATCC27255]RBATCC27255_00495_L-threonine_dehydratase_catabolic_TdcB	RbromiiATCC27255
Org3_Gene477	core	3361	[RbromiiATCC27255]RBATCC27255_00496_Flavodoxin	RbromiiATCC27255
Org3_Gene482	core	134	[RbromiiATCC27255]RBATCC27255_00501_Ferrous_iron_transport_protein_B	RbromiiATCC27255
Org3_Gene483	core	4121	[RbromiiATCC27255]RBATCC27255_00502_Ferrous_iron_transport_protein_A	RbromiiATCC27255
Org3_Gene484	core	4164	[RbromiiATCC27255]RBATCC27255_00503_FeoA_domain_protein	RbromiiATCC27255
Org3_Gene485	core	3540	[RbromiiATCC27255]RBATCC27255_00504_Manganese_transport_regulator	RbromiiATCC27255
Org3_Gene487	core	224	[RbromiiATCC27255]RBATCC27255_00506_30S_ribosomal_protein_S1	RbromiiATCC27255
Org3_Gene488	core	1898	[RbromiiATCC27255]RBATCC27255_00507_Formate_channel_1	RbromiiATCC27255
Org3_Gene500	core	2029	[RbromiiATCC27255]RBATCC27255_00520_L-cystine_import_ATP-binding_protein_TcyC	RbromiiATCC27255
Org3_Gene501	core	2544	[RbromiiATCC27255]RBATCC27255_00521_L-cystine_transport_system_permease_protein_TcyB	RbromiiATCC27255
Org3_Gene504	core	2611	[RbromiiATCC27255]RBATCC27255_00524_putative_manganese_catalase	RbromiiATCC27255
Org3_Gene572	core	1469	[RbromiiATCC27255]RBATCC27255_00592_4-hydroxy-2-oxovalerate_aldolase	RbromiiATCC27255
Org3_Gene573	core	907	[RbromiiATCC27255]RBATCC27255_00593_Dihydroorotase	RbromiiATCC27255
Org3_Gene576	core	958	[RbromiiATCC27255]RBATCC27255_00596_orc1/cdc6_family_replication_initiation_protein	RbromiiATCC27255
Org3_Gene577	core	1291	[RbromiiATCC27255]RBATCC27255_00598_methyltransferase,_FkbM_family	RbromiiATCC27255
Org3_Gene581	core	199	[RbromiiATCC27255]RBATCC27255_00602_Formate_acetyltransferase	RbromiiATCC27255
Org3_Gene583	core	1904	[RbromiiATCC27255]RBATCC27255_00604_DNA-3-methyladenine_glycosylase	RbromiiATCC27255
Org3_Gene584	core	356	[RbromiiATCC27255]RBATCC27255_00605_Glutamine--fructose-6-phosphate_aminotransferase_[isomerizing]	RbromiiATCC27255
Org3_Gene585	core	336	[RbromiiATCC27255]RBATCC27255_00606_UDP-N-acetyl-alpha-D-glucosamine_C6_dehydratase	RbromiiATCC27255
Org3_Gene590	core	1065	[RbromiiATCC27255]RBATCC27255_00611_putative_tRNA_sulfurtransferase	RbromiiATCC27255
Org3_Gene591	core	1182	[RbromiiATCC27255]RBATCC27255_00612_Cysteine_desulfurase	RbromiiATCC27255
Org3_Gene592	core	3243	[RbromiiATCC27255]RBATCC27255_00613_stage_V_sporulation_protein_AC	RbromiiATCC27255
Org3_Gene594	core	714	[RbromiiATCC27255]RBATCC27255_00615_2-methylcitrate_synthase	RbromiiATCC27255
Org3_Gene596	core	1875	[RbromiiATCC27255]RBATCC27255_00617_Sugar_phosphatase_YidA	RbromiiATCC27255
Org3_Gene608	core	4333	[RbromiiATCC27255]RBATCC27255_00629_hypothetical_protein	RbromiiATCC27255
Org3_Gene613	core	2888	[RbromiiATCC27255]RBATCC27255_00634_ADP-ribose_pyrophosphatase	RbromiiATCC27255
Org3_Gene615	core	164	[RbromiiATCC27255]RBATCC27255_00636_Asparagine_synthetase_[glutamine-hydrolyzing]_1	RbromiiATCC27255
Org3_Gene617	core	363	[RbromiiATCC27255]RBATCC27255_00638_Elongation_factor_4	RbromiiATCC27255
Org3_Gene619	core	95	[RbromiiATCC27255]RBATCC27255_00640_preprotein_translocase_subunit_SecA	RbromiiATCC27255
Org3_Gene62	core	3796	[RbromiiATCC27255]RBATCC27255_00066_hypothetical_protein	RbromiiATCC27255
Org3_Gene620	core	695	[RbromiiATCC27255]RBATCC27255_00641_Beta-lactam-inducible_penicillin-binding_protein	RbromiiATCC27255
Org3_Gene621	core	444	[RbromiiATCC27255]RBATCC27255_00642_Cell_division_protein_FtsW	RbromiiATCC27255
Org3_Gene63	core	2594	[RbromiiATCC27255]RBATCC27255_00067_50S_ribosomal_protein_L3	RbromiiATCC27255
Org3_Gene64	core	2622	[RbromiiATCC27255]RBATCC27255_00068_50S_ribosomal_protein_L4	RbromiiATCC27255
Org3_Gene65	core	3858	[RbromiiATCC27255]RBATCC27255_00069_50S_ribosomal_protein_L23	RbromiiATCC27255
Org3_Gene66	core	1925	[RbromiiATCC27255]RBATCC27255_00070_hypothetical_protein	RbromiiATCC27255
Org3_Gene67	core	3940	[RbromiiATCC27255]RBATCC27255_00071_hypothetical_protein	RbromiiATCC27255
Org3_Gene68	core	3667	[RbromiiATCC27255]RBATCC27255_00072_50S_ribosomal_protein_L22	RbromiiATCC27255
Org3_Gene69	core	2426	[RbromiiATCC27255]RBATCC27255_00073_hypothetical_protein	RbromiiATCC27255
Org3_Gene692	core	403	[RbromiiATCC27255]RBATCC27255_00714_hypothetical_protein	RbromiiATCC27255
Org3_Gene693	core	3115	[RbromiiATCC27255]RBATCC27255_00715_Ribosomal_RNA_large_subunit_methyltransferase_H	RbromiiATCC27255

Org3_Gene694	core	2075	[RbromiiATCC27255]RBATCC27255_00716_Putative_metallo-hydrolase_YycJ	RbromiiATCC27255
Org3_Gene695	core	738	[RbromiiATCC27255]RBATCC27255_00717_UDP-N-acetylglucosamine_1-carboxyvinyltransferase_2	RbromiiATCC27255
Org3_Gene696	core	3702	[RbromiiATCC27255]RBATCC27255_00718_hypothetical_protein	RbromiiATCC27255
Org3_Gene699	core	494	[RbromiiATCC27255]RBATCC27255_00721_Acetolactate_synthase_large_subunit	RbromiiATCC27255
Org3_Gene70	core	3375	[RbromiiATCC27255]RBATCC27255_00074_50S_ribosomal_protein_L16	RbromiiATCC27255
Org3_Gene700	core	3087	[RbromiiATCC27255]RBATCC27255_00722_Acetolactate_synthase_small_subunit	RbromiiATCC27255
Org3_Gene701	core	1341	[RbromiiATCC27255]RBATCC27255_00723_Ketol-acid_reductoisomerase	RbromiiATCC27255
Org3_Gene71	core	4200	[RbromiiATCC27255]RBATCC27255_00075_50S_ribosomal_protein_L29	RbromiiATCC27255
Org3_Gene710	core	449	[RbromiiATCC27255]RBATCC27255_00732_Hydroxylamine_reductase	RbromiiATCC27255
Org3_Gene719	core	2184	[RbromiiATCC27255]RBATCC27255_00741_Phosphate_import_ATP-binding_protein_PstB_3	RbromiiATCC27255
Org3_Gene72	core	3990	[RbromiiATCC27255]RBATCC27255_00076_30S_ribosomal_protein_S17	RbromiiATCC27255
Org3_Gene723	core	717	[RbromiiATCC27255]RBATCC27255_00745_Tryptophan_synthase_beta_chain	RbromiiATCC27255
Org3_Gene725	core	654	[RbromiiATCC27255]RBATCC27255_00747_Aspartyl/glutamyl-tRNA(Asn/Gln)_amidotransferase_subunit_B	RbromiiATCC27255
Org3_Gene726	core	628	[RbromiiATCC27255]RBATCC27255_00748_Glutamyl-tRNA(Gln)_amidotransferase_subunit_A	RbromiiATCC27255
Org3_Gene727	core	3910	[RbromiiATCC27255]RBATCC27255_00749_Aspartyl/glutamyl-tRNA(Asn/Gln)_amidotransferase_subunit_C	RbromiiATCC27255
Org3_Gene728	core	399	[RbromiiATCC27255]RBATCC27255_00750_Aspartate--tRNA_ligase	RbromiiATCC27255
Org3_Gene73	core	3565	[RbromiiATCC27255]RBATCC27255_00077_50S_ribosomal_protein_L14	RbromiiATCC27255
Org3_Gene731	core	2781	[RbromiiATCC27255]RBATCC27255_00753_uracil-DNA_glycosylase,_family_4	RbromiiATCC27255
Org3_Gene732	core	2814	[RbromiiATCC27255]RBATCC27255_00754_Phosphate_propanoyltransferase	RbromiiATCC27255
Org3_Gene734	core	3338	[RbromiiATCC27255]RBATCC27255_00756_hypothetical_protein	RbromiiATCC27255
Org3_Gene735	core	510	[RbromiiATCC27255]RBATCC27255_00757_Phosphoenolpyruvate_carboxykinase_[ATP]	RbromiiATCC27255
Org3_Gene736	core	3530	[RbromiiATCC27255]RBATCC27255_00758_hypothetical_protein	RbromiiATCC27255
Org3_Gene737	core	2838	[RbromiiATCC27255]RBATCC27255_00759_50S_ribosomal_protein_L10	RbromiiATCC27255
Org3_Gene738	core	2398	[RbromiiATCC27255]RBATCC27255_00761_50S_ribosomal_protein_L1	RbromiiATCC27255
Org3_Gene739	core	3352	[RbromiiATCC27255]RBATCC27255_00762_50S_ribosomal_protein_L11	RbromiiATCC27255
Org3_Gene74	core	3773	[RbromiiATCC27255]RBATCC27255_00078_50S_ribosomal_protein_L24	RbromiiATCC27255
Org3_Gene740	core	2971	[RbromiiATCC27255]RBATCC27255_00763_hypothetical_protein	RbromiiATCC27255
Org3_Gene742	core	4393	[RbromiiATCC27255]RBATCC27255_00765_50S_ribosomal_protein_L33	RbromiiATCC27255
Org3_Gene743	core	2067	[RbromiiATCC27255]RBATCC27255_00766_hypothetical_protein	RbromiiATCC27255
Org3_Gene744	core	1978	[RbromiiATCC27255]RBATCC27255_00767_Glutamate_racemase	RbromiiATCC27255
Org3_Gene745	core	3199	[RbromiiATCC27255]RBATCC27255_00768_hypothetical_protein	RbromiiATCC27255
Org3_Gene746	core	408	[RbromiiATCC27255]RBATCC27255_00769_Arginine--tRNA_ligase	RbromiiATCC27255
Org3_Gene75	core	2914	[RbromiiATCC27255]RBATCC27255_00079_50S_ribosomal_protein_L5	RbromiiATCC27255
Org3_Gene750	core	1015	[RbromiiATCC27255]RBATCC27255_00773_Aspartokinase	RbromiiATCC27255
Org3_Gene751	core	957	[RbromiiATCC27255]RBATCC27255_00774_Homoserine_dehydrogenase	RbromiiATCC27255
Org3_Gene754	core	1551	[RbromiiATCC27255]RBATCC27255_00777_Nitronate_monooxygenase	RbromiiATCC27255
Org3_Gene755	core	1688	[RbromiiATCC27255]RBATCC27255_00778_Malonyl_CoA-acyl_carrier_protein_transacylase	RbromiiATCC27255
Org3_Gene756	core	2214	[RbromiiATCC27255]RBATCC27255_00779_3-oxoacyl-[acyl-carrier-protein]_reductase_FabG	RbromiiATCC27255
Org3_Gene757	core	970	[RbromiiATCC27255]RBATCC27255_00780_3-oxoacyl-[acyl-carrier-protein]_synthase_2	RbromiiATCC27255
Org3_Gene759	core	3377	[RbromiiATCC27255]RBATCC27255_00782_3-hydroxyacyl-[acyl-carrier-protein]_dehydratase_FabZ	RbromiiATCC27255
Org3_Gene76	core	4289	[RbromiiATCC27255]RBATCC27255_00080_hypothetical_protein	RbromiiATCC27255
Org3_Gene760	core	748	[RbromiiATCC27255]RBATCC27255_00783_2-oxoglutarate_carboxylase_small_subunit	RbromiiATCC27255
Org3_Gene761	core	1826	[RbromiiATCC27255]RBATCC27255_00784_Acetyl-coenzyme_A_carboxylase_carboxyl_transferase_subunit_beta	RbromiiATCC27255
Org3_Gene762	core	2088	[RbromiiATCC27255]RBATCC27255_00785_Acetyl-coenzyme_A_carboxylase_carboxyl_transferase_subunit_alpha	RbromiiATCC27255
Org3_Gene764	core	1626	[RbromiiATCC27255]RBATCC27255_00787_Ribosomal_RNA_small_subunit_methyltransferase_H	RbromiiATCC27255
Org3_Gene766	core	194	[RbromiiATCC27255]RBATCC27255_00789_Sporulation-specific_penicillin-binding_protein	RbromiiATCC27255
Org3_Gene767	core	1356	[RbromiiATCC27255]RBATCC27255_00790_Phospho-N-acetylmuramoyl-pentapeptide-transferase	RbromiiATCC27255
Org3_Gene768	core	928	[RbromiiATCC27255]RBATCC27255_00791_Cell_division_protein_FtsW	RbromiiATCC27255
Org3_Gene769	core	1196	[RbromiiATCC27255]RBATCC27255_00792_UDP-N-acetylglucosamine--N-acetylmuramyl-(pentapeptide)_pyrophosphoryl-undecaprenol_N-acetylglucosami	RbromiiATCC27255
Org3_Gene77	core	3469	[RbromiiATCC27255]RBATCC27255_00081_30S_ribosomal_protein_S8	RbromiiATCC27255
Org3_Gene770	core	933	[RbromiiATCC27255]RBATCC27255_00793_UDP-N-acetylglucosamine_1-carboxyvinyltransferase_1	RbromiiATCC27255
Org3_Gene772	core	1214	[RbromiiATCC27255]RBATCC27255_00795_Cell_division_protein_FtsZ	RbromiiATCC27255
Org3_Gene78	core	2900	[RbromiiATCC27255]RBATCC27255_00082_hypothetical_protein	RbromiiATCC27255
Org3_Gene783	core	594	[RbromiiATCC27255]RBATCC27255_00806_23S_rRNA_(uracil-C(5))-methyltransferase_RlmCD	RbromiiATCC27255
Org3_Gene784	core	593	[RbromiiATCC27255]RBATCC27255_00807_Glutamate_synthase_[NADPH]_small_chain	RbromiiATCC27255
Org3_Gene785	core	18	[RbromiiATCC27255]RBATCC27255_00808_Ferredoxin-dependent_glutamate_synthase_1	RbromiiATCC27255
Org3_Gene786	core	246	[RbromiiATCC27255]RBATCC27255_00809_Glutamine_synthetase	RbromiiATCC27255
Org3_Gene787	core	432	[RbromiiATCC27255]RBATCC27255_00810_Ammonia_transporter	RbromiiATCC27255
Org3_Gene79	core	3597	[RbromiiATCC27255]RBATCC27255_00083_hypothetical_protein	RbromiiATCC27255
Org3_Gene794	core	330	[RbromiiATCC27255]RBATCC27255_00817_High_temperature_protein_G	RbromiiATCC27255
Org3_Gene797	core	2164	[RbromiiATCC27255]RBATCC27255_00820_Dihydroorotate_oxidase_B,_electron_transfer_subunit	RbromiiATCC27255
Org3_Gene798	core	1658	[RbromiiATCC27255]RBATCC27255_00821_Dihydroorotate_dehydrogenase_B_(NAD(+)),_catalytic_subunit	RbromiiATCC27255
Org3_Gene80	core	3067	[RbromiiATCC27255]RBATCC27255_00084_30S_ribosomal_protein_S5	RbromiiATCC27255
Org3_Gene800	core	319	[RbromiiATCC27255]RBATCC27255_00823_Alpha-xylosidase	RbromiiATCC27255
Org3_Gene802	core	578	[RbromiiATCC27255]RBATCC27255_00825_Phosphomannomutase/phosphoglucomutase	RbromiiATCC27255
Org3_Gene803	core	3957	[RbromiiATCC27255]RBATCC27255_00826_hypothetical_protein	RbromiiATCC27255
Org3_Gene804	core	3607	[RbromiiATCC27255]RBATCC27255_00827_hypothetical_protein	RbromiiATCC27255
Org3_Gene806	core	317	[RbromiiATCC27255]RBATCC27255_00829_putative_ABC_transporter_ATP-binding_protein_YheS	RbromiiATCC27255
Org3_Gene81	core	4346	[RbromiiATCC27255]RBATCC27255_00085_50S_ribosomal_protein_L30	RbromiiATCC27255
Org3_Gene813	core	118	[RbromiiATCC27255]RBATCC27255_00838_Alanine--tRNA_ligase	RbromiiATCC27255
Org3_Gene814	core	971	[RbromiiATCC27255]RBATCC27255_00840_putative_protease_YhbU_precursor	RbromiiATCC27255
Org3_Gene817	core	2911	[RbromiiATCC27255]RBATCC27255_00843_Chaperone_protein_YajL	RbromiiATCC27255
Org3_Gene82	core	3283	[RbromiiATCC27255]RBATCC27255_00086_50S_ribosomal_protein_L15	RbromiiATCC27255
Org3_Gene821	core	2018	[RbromiiATCC27255]RBATCC27255_00847_Thymidylate_synthase_ThyX	RbromiiATCC27255
Org3_Gene823	core	1555	[RbromiiATCC27255]RBATCC27255_00849_Glycerate_dehydrogenase	RbromiiATCC27255
Org3_Gene825	core	37	[RbromiiATCC27255]RBATCC27255_00851_Phosphoribosylformylglycinamidine_synthase_2	RbromiiATCC27255
Org3_Gene828	core	2140	[RbromiiATCC27255]RBATCC27255_00854_Type_III_pantothenate_kinase	RbromiiATCC27255
Org3_Gene829	core	1872	[RbromiiATCC27255]RBATCC27255_00855_Transketolase	RbromiiATCC27255
Org3_Gene83	core	782	[RbromiiATCC27255]RBATCC27255_00087_preprotein_translocase_subunit_SecY	RbromiiATCC27255
Org3_Gene830	core	1554	[RbromiiATCC27255]RBATCC27255_00856_1-deoxy-D-xylulose-5-phosphate_synthase	RbromiiATCC27255
Org3_Gene831	core	4080	[RbromiiATCC27255]RBATCC27255_00857_putative_RNA-binding_protein_(contains_KH_domain)	RbromiiATCC27255
Org3_Gene832	core	4034	[RbromiiATCC27255]RBATCC27255_00858_hypothetical_protein	RbromiiATCC27255
Org3_Gene833	core	676	[RbromiiATCC27255]RBATCC27255_00859_hypothetical_protein	RbromiiATCC27255
Org3_Gene834	core	3631	[RbromiiATCC27255]RBATCC27255_00860_putative_DNA-binding_protein	RbromiiATCC27255
Org3_Gene835	core	1321	[RbromiiATCC27255]RBATCC27255_00861_PhoH-like_protein	RbromiiATCC27255
Org3_Gene836	core	3007	[RbromiiATCC27255]RBATCC27255_00862_Endoribonuclease_YbeY	RbromiiATCC27255
Org3_Gene838	core	1643	[RbromiiATCC27255]RBATCC27255_00864_Bex_protein	RbromiiATCC27255
Org3_Gene839	core	4338	[RbromiiATCC27255]RBATCC27255_00865_hypothetical_protein	RbromiiATCC27255
Org3_Gene84	core	2593	[RbromiiATCC27255]RBATCC27255_00088_Adenylate_kinase	RbromiiATCC27255
Org3_Gene840	core	2204	[RbromiiATCC27255]RBATCC27255_00866_Recombination_protein_O	RbromiiATCC27255
Org3_Gene841	core	135	[RbromiiATCC27255]RBATCC27255_00867_MutS2_protein	RbromiiATCC27255
Org3_Gene842	core	3006	[RbromiiATCC27255]RBATCC27255_00868_Ribosome_maturation_factor_RimM	RbromiiATCC27255
Org3_Gene843	core	2404	[RbromiiATCC27255]RBATCC27255_00869_tRNA_(guanine-N(1)-)-methyltransferase	RbromiiATCC27255
Org3_Gene844	core	3960	[RbromiiATCC27255]RBATCC27255_00870_Catabolite_repression_HPr	RbromiiATCC27255
Org3_Gene845	core	347	[RbromiiATCC27255]RBATCC27255_00871_Excinuclease_ABC_subunit_C	RbromiiATCC27255
Org3_Gene846	core	2851	[RbromiiATCC27255]RBATCC27255_00872_Ribosomal_RNA_small_subunit_methyltransferase_D	RbromiiATCC27255
Org3_Gene848	core	3206	[RbromiiATCC27255]RBATCC27255_00874_hypothetical_protein	RbromiiATCC27255
Org3_Gene849	core	2810	[RbromiiATCC27255]RBATCC27255_00875_putative_transcriptional_regulatory_protein_pdtaR	RbromiiATCC27255
Org3_Gene85	core	2186	[RbromiiATCC27255]RBATCC27255_00089_Methionine_aminopeptidase_1	RbromiiATCC27255
Org3_Gene850	core	1263	[RbromiiATCC27255]RBATCC27255_00876_Carbamoyl-phosphate_synthase_small_chain	RbromiiATCC27255
Org3_Gene851	core	31	[RbromiiATCC27255]RBATCC27255_00877_Carbamoyl-phosphate_synthase_large_chain	RbromiiATCC27255
Org3_Gene852	core	492	[RbromiiATCC27255]RBATCC27255_00878_CTP_synthase	RbromiiATCC27255
Org3_Gene853	core	1050	[RbromiiATCC27255]RBATCC27255_00879_LL-diaminopimelate_aminotransferase	RbromiiATCC27255
Org3_Gene854	core	718	[RbromiiATCC27255]RBATCC27255_00880_Glycine--tRNA_ligase	RbromiiATCC27255
Org3_Gene86	core	4037	[RbromiiATCC27255]RBATCC27255_00090_hypothetical_protein	RbromiiATCC27255
Org3_Gene860	core	3965	[RbromiiATCC27255]RBATCC27255_00886_Phosphocarrier_protein_HPr	RbromiiATCC27255
Org3_Gene865	core	215	[RbromiiATCC27255]RBATCC27255_00891_Anaerobic_ribonucleoside-triphosphate_reductase	RbromiiATCC27255
Org3_Gene866	core	2925	[RbromiiATCC27255]RBATCC27255_00892_anaerobic_ribonucleotide_reductase-activating_protein	RbromiiATCC27255
Org3_Gene87	core	4142	[RbromiiATCC27255]RBATCC27255_00091_Translation_initiation_factor_IF-1	RbromiiATCC27255
Org3_Gene872	core	1316	[RbromiiATCC27255]RBATCC27255_00899_Phenylalanine--tRNA_ligase_alpha_subunit	RbromiiATCC27255
Org3_Gene873	core	169	[RbromiiATCC27255]RBATCC27255_00900_Phenylalanine--tRNA_ligase_beta_subunit	RbromiiATCC27255
Org3_Gene874	core	4032	[RbromiiATCC27255]RBATCC27255_00901_hypothetical_protein	RbromiiATCC27255
Org3_Gene875	core	3817	[RbromiiATCC27255]RBATCC27255_00902_hypothetical_protein	RbromiiATCC27255
Org3_Gene878	core	137	[RbromiiATCC27255]RBATCC27255_00905_putative_copper-importing_P-type_ATPase_A	RbromiiATCC27255
Org3_Gene879	core	3823	[RbromiiATCC27255]RBATCC27255_00906_Copper-sensitive_operon_repressor	RbromiiATCC27255
Org3_Gene882	core	4065	[RbromiiATCC27255]RBATCC27255_00909_hypothetical_protein	RbromiiATCC27255
Org3_Gene883	core	3056	[RbromiiATCC27255]RBATCC27255_00910_Helix-destabilizing_protein	RbromiiATCC27255
Org3_Gene884	core	3849	[RbromiiATCC27255]RBATCC27255_00911_30S_ribosomal_protein_S6	RbromiiATCC27255
Org3_Gene887	core	852	[RbromiiATCC27255]RBATCC27255_00914_Methionine_gamma-lyase	RbromiiATCC27255
Org3_Gene89	core	3568	[RbromiiATCC27255]RBATCC27255_00093_30S_ribosomal_protein_S13	RbromiiATCC27255
Org3_Gene890	core	2395	[RbromiiATCC27255]RBATCC27255_00917_RNA_polymerase_sigma-28_factor_precursor	RbromiiATCC27255
Org3_Gene898	core	243	[RbromiiATCC27255]RBATCC27255_00925_Membrane_protein_YdfJ	RbromiiATCC27255

Org3_Gene90	core	3445	[RbromiiATCC27255]RBATCC27255_00094_30S_ribosomal_protein_S11	RbromiiATCC27255
Org3_Gene902	core	4046	[RbromiiATCC27255]RBATCC27255_00929_transcriptional_regulator,_AbrB_family	RbromiiATCC27255
Org3_Gene903	core	2757	[RbromiiATCC27255]RBATCC27255_00930_Spore_maturation_protein_A	RbromiiATCC27255
Org3_Gene904	core	3055	[RbromiiATCC27255]RBATCC27255_00931_Spore_maturation_protein_B	RbromiiATCC27255
Org3_Gene907	core	1238	[RbromiiATCC27255]RBATCC27255_00934_Ribosome-binding_ATPase_YchF	RbromiiATCC27255
Org3_Gene908	core	3496	[RbromiiATCC27255]RBATCC27255_00935_Fluoroacetyl-CoA_thioesterase	RbromiiATCC27255
Org3_Gene91	core	2686	[RbromiiATCC27255]RBATCC27255_00095_30S_ribosomal_protein_S4	RbromiiATCC27255
Org3_Gene912	core	1217	[RbromiiATCC27255]RBATCC27255_00939_Trehalose_import_ATP-binding_protein_SugC	RbromiiATCC27255
Org3_Gene913	core	1258	[RbromiiATCC27255]RBATCC27255_00940_Purine_catabolism_regulatory_protein	RbromiiATCC27255
Org3_Gene914	core	2132	[RbromiiATCC27255]RBATCC27255_00941_Cell_division_ATP-binding_protein_FtsE	RbromiiATCC27255
Org3_Gene918	core	3129	[RbromiiATCC27255]RBATCC27255_00945_Transcript_cleavage_factor_GreA	RbromiiATCC27255
Org3_Gene919	core	310	[RbromiiATCC27255]RBATCC27255_00946_Lysine--tRNA_ligase	RbromiiATCC27255
Org3_Gene92	core	1541	[RbromiiATCC27255]RBATCC27255_00096_DNA-directed_RNA_polymerase_subunit_alpha	RbromiiATCC27255
Org3_Gene927	core	141	[RbromiiATCC27255]RBATCC27255_00954_Negative_regulator_of_genetic_competence_ClpC/MecB	RbromiiATCC27255
Org3_Gene928	core	1444	[RbromiiATCC27255]RBATCC27255_00955_Putative_ATP:guanido_phosphotransferase	RbromiiATCC27255
Org3_Gene929	core	3043	[RbromiiATCC27255]RBATCC27255_00956_hypothetical_protein	RbromiiATCC27255
Org3_Gene93	core	3661	[RbromiiATCC27255]RBATCC27255_00097_hypothetical_protein	RbromiiATCC27255
Org3_Gene930	core	3251	[RbromiiATCC27255]RBATCC27255_00957_Class_three_stress_gene_repressor	RbromiiATCC27255
Org3_Gene934	core	3905	[RbromiiATCC27255]RBATCC27255_00961_CxxC-x17-CxxC_domain_protein	RbromiiATCC27255
Org3_Gene937	core	4248	[RbromiiATCC27255]RBATCC27255_00964_hybrid_cluster_protein-associated_redox_disulfide_domain_protein	RbromiiATCC27255
Org3_Gene94	core	396	[RbromiiATCC27255]RBATCC27255_00098_putative_ABC_transporter_ATP-binding_protein	RbromiiATCC27255
Org3_Gene940	core	2484	[RbromiiATCC27255]RBATCC27255_00967_tRNA_(guanine-N(7)-)-methyltransferase	RbromiiATCC27255
Org3_Gene942	core	2345	[RbromiiATCC27255]RBATCC27255_00969_Sensory_transduction_protein_regX3	RbromiiATCC27255
Org3_Gene943	core	605	[RbromiiATCC27255]RBATCC27255_00970_Alkaline_phosphatase_synthesis_sensor_protein_PhoR	RbromiiATCC27255
Org3_Gene944	core	1443	[RbromiiATCC27255]RBATCC27255_00971_putative_metal-dependent_enzyme	RbromiiATCC27255
Org3_Gene946	core	1195	[RbromiiATCC27255]RBATCC27255_00973_Recombinase_A	RbromiiATCC27255
Org3_Gene947	core	2648	[RbromiiATCC27255]RBATCC27255_00974_Regulatory_protein_RecX	RbromiiATCC27255
Org3_Gene948	core	778	[RbromiiATCC27255]RBATCC27255_00975_Ribosomal_protein_S12_methylthiotransferase_RimO	RbromiiATCC27255
Org3_Gene949	core	2725	[RbromiiATCC27255]RBATCC27255_00976_CDP-diacylglycerol--glycerol-3-phosphate_3-phosphatidyltransferase	RbromiiATCC27255
Org3_Gene95	core	405	[RbromiiATCC27255]RBATCC27255_00099_putative_ABC_transporter_ATP-binding_protein	RbromiiATCC27255
Org3_Gene950	core	2491	[RbromiiATCC27255]RBATCC27255_00977_Serine_acetyltransferase	RbromiiATCC27255
Org3_Gene951	core	685	[RbromiiATCC27255]RBATCC27255_00978_Cysteine--tRNA_ligase	RbromiiATCC27255
Org3_Gene952	core	2056	[RbromiiATCC27255]RBATCC27255_00979_Bifunctional_xylanase/deacetylase_precursor	RbromiiATCC27255
Org3_Gene96	core	570	[RbromiiATCC27255]RBATCC27255_00100_2,3-bisphosphoglycerate-independent_phosphoglycerate_mutase	RbromiiATCC27255
Org3_Gene965	core	323	[RbromiiATCC27255]RBATCC27255_00992_putative_ABC_transporter_ATP-binding_protein	RbromiiATCC27255
Org3_Gene966	core	398	[RbromiiATCC27255]RBATCC27255_00993_putative_ABC_transporter_ATP-binding_protein	RbromiiATCC27255
Org3_Gene97	core	2143	[RbromiiATCC27255]RBATCC27255_00101_Triosephosphate_isomerase	RbromiiATCC27255
Org3_Gene970	core	2145	[RbromiiATCC27255]RBATCC27255_00997_putative_oxidoreductase	RbromiiATCC27255
Org3_Gene973	core	729	[RbromiiATCC27255]RBATCC27255_01000_putative_RNA_methyltransferase	RbromiiATCC27255
Org3_Gene974	core	710	[RbromiiATCC27255]RBATCC27255_01001_UDP-N-acetylmuramate--L-alanine_ligase	RbromiiATCC27255
Org3_Gene98	core	1009	[RbromiiATCC27255]RBATCC27255_00102_Phosphoglycerate_kinase	RbromiiATCC27255
Org3_Gene981	core	359	[RbromiiATCC27255]RBATCC27255_01008_Tyrosine_phosphorylated_protein_A	RbromiiATCC27255
Org3_Gene982	core	307	[RbromiiATCC27255]RBATCC27255_01009_2,3-dimethylmalate_dehydratase_large_subunit	RbromiiATCC27255
Org3_Gene983	core	1011	[RbromiiATCC27255]RBATCC27255_01010_Isocitrate_dehydrogenase_[NADP]	RbromiiATCC27255
Org3_Gene984	core	2589	[RbromiiATCC27255]RBATCC27255_01011_Redox-sensing_transcriptional_repressor_rex	RbromiiATCC27255
Org3_Gene986	core	92	[RbromiiATCC27255]RBATCC27255_01013_Pullulanase_precursor	RbromiiATCC27255
Org3_Gene989	core	3557	[RbromiiATCC27255]RBATCC27255_01016_hypothetical_protein	RbromiiATCC27255
Org3_Gene99	core	2998	[RbromiiATCC27255]RBATCC27255_00103_tRNA_(cytidine(34)-2'-O)-methyltransferase	RbromiiATCC27255
Org3_Gene990	core	1216	[RbromiiATCC27255]RBATCC27255_01017_RNA_polymerase_sigma_factor_RpoD	RbromiiATCC27255
Org3_Gene991	core	383	[RbromiiATCC27255]RBATCC27255_01018_DNA_primase	RbromiiATCC27255
Org3_Gene992	core	2603	[RbromiiATCC27255]RBATCC27255_01019_IMPACT_family_member_YigZ	RbromiiATCC27255
Org3_Gene993	core	4201	[RbromiiATCC27255]RBATCC27255_01020_50S_ribosomal_protein_L31	RbromiiATCC27255
Org3_Gene995	core	298	[RbromiiATCC27255]RBATCC27255_01022_Threonine--tRNA_ligase_1	RbromiiATCC27255
Org4_Gene10	core	1866	[RbromiiL2-36]RBL236_00010_Bifunctional_protein_FolD_protein	RbromiiL2-36
Org4_Gene1001	core	4343	[RbromiiL2-36]RBL236_01051_Ferredoxin	RbromiiL2-36
Org4_Gene1002	core	1810	[RbromiiL2-36]RBL236_01052_hypothetical_protein	RbromiiL2-36
Org4_Gene1008	core	945	[RbromiiL2-36]RBL236_01058_Gamma-glutamyl_phosphate_reductase	RbromiiL2-36
Org4_Gene1009	core	1993	[RbromiiL2-36]RBL236_01059_Glutamate_5-kinase_1	RbromiiL2-36
Org4_Gene1010	core	1602	[RbromiiL2-36]RBL236_01060_Homoserine_O-succinyltransferase	RbromiiL2-36
Org4_Gene1011	core	644	[RbromiiL2-36]RBL236_01061_Proline--tRNA_ligase	RbromiiL2-36
Org4_Gene1012	core	4101	[RbromiiL2-36]RBL236_01062_Acyl_carrier_protein	RbromiiL2-36
Org4_Gene1013	core	1884	[RbromiiL2-36]RBL236_01063_Shikimate_dehydrogenase	RbromiiL2-36
Org4_Gene1015	core	1379	[RbromiiL2-36]RBL236_01065_Phospho-2-dehydro-3-deoxyheptonate_aldolase	RbromiiL2-36
Org4_Gene1016	core	1936	[RbromiiL2-36]RBL236_01066_Arogenate_dehydrogenase	RbromiiL2-36
Org4_Gene1017	core	1306	[RbromiiL2-36]RBL236_01067_3-dehydroquinate_synthase	RbromiiL2-36
Org4_Gene1018	core	859	[RbromiiL2-36]RBL236_01068_3-phosphoshikimate_1-carboxyvinyltransferase	RbromiiL2-36
Org4_Gene1019	core	1278	[RbromiiL2-36]RBL236_01069_Chorismate_synthase	RbromiiL2-36
Org4_Gene1020	core	1155	[RbromiiL2-36]RBL236_01070_P-protein	RbromiiL2-36
Org4_Gene1024	core	553	[RbromiiL2-36]RBL236_01074_Trehalose_synthase/amylase_TreS	RbromiiL2-36
Org4_Gene1029	core	112	[RbromiiL2-36]RBL236_01079_Valine--tRNA_ligase	RbromiiL2-36
Org4_Gene1063	core	330	[RbromiiL2-36]RBL236_01115_High_temperature_protein_G	RbromiiL2-36
Org4_Gene1067	core	2164	[RbromiiL2-36]RBL236_01119_Dihydroorotate_oxidase_B,_electron_transfer_subunit	RbromiiL2-36
Org4_Gene1068	core	1658	[RbromiiL2-36]RBL236_01120_Dihydroorotate_dehydrogenase_B_(NAD(+)),_catalytic_subunit	RbromiiL2-36
Org4_Gene1070	core	319	[RbromiiL2-36]RBL236_01122_Alpha-xylosidase	RbromiiL2-36
Org4_Gene1072	core	578	[RbromiiL2-36]RBL236_01124_Phosphomannomutase/phosphoglucomutase	RbromiiL2-36
Org4_Gene1073	core	3957	[RbromiiL2-36]RBL236_01125_hypothetical_protein	RbromiiL2-36
Org4_Gene1074	core	3607	[RbromiiL2-36]RBL236_01126_hypothetical_protein	RbromiiL2-36
Org4_Gene1076	core	317	[RbromiiL2-36]RBL236_01128_putative_ABC_transporter_ATP-binding_protein_YheS	RbromiiL2-36
Org4_Gene11	core	3807	[RbromiiL2-36]RBL236_00011_50S_ribosomal_protein_L21	RbromiiL2-36
Org4_Gene1163	core	594	[RbromiiL2-36]RBL236_01218_23S_rRNA_(uracil-C(5))-methyltransferase_RlmCD	RbromiiL2-36
Org4_Gene1164	core	593	[RbromiiL2-36]RBL236_01219_Glutamate_synthase_[NADPH]_small_chain	RbromiiL2-36
Org4_Gene1165	core	18	[RbromiiL2-36]RBL236_01220_Ferredoxin-dependent_glutamate_synthase_1	RbromiiL2-36
Org4_Gene1166	core	246	[RbromiiL2-36]RBL236_01221_Glutamine_synthetase	RbromiiL2-36
Org4_Gene1167	core	432	[RbromiiL2-36]RBL236_01222_Ammonia_transporter	RbromiiL2-36
Org4_Gene1170	core	2810	[RbromiiL2-36]RBL236_01225_putative_transcriptional_regulatory_protein_pdtaR	RbromiiL2-36
Org4_Gene1171	core	1263	[RbromiiL2-36]RBL236_01226_Carbamoyl-phosphate_synthase_small_chain	RbromiiL2-36
Org4_Gene1172	core	31	[RbromiiL2-36]RBL236_01227_Carbamoyl-phosphate_synthase_large_chain	RbromiiL2-36
Org4_Gene1173	core	492	[RbromiiL2-36]RBL236_01228_CTP_synthase	RbromiiL2-36
Org4_Gene1174	core	1050	[RbromiiL2-36]RBL236_01229_LL-diaminopimelate_aminotransferase	RbromiiL2-36
Org4_Gene1175	core	718	[RbromiiL2-36]RBL236_01230_Glycine--tRNA_ligase	RbromiiL2-36
Org4_Gene1217	core	2350	[RbromiiL2-36]RBL236_01274_Phosphoribosylaminoimidazole-succinocarboxamide_synthase	RbromiiL2-36
Org4_Gene1218	core	613	[RbromiiL2-36]RBL236_01275_Amidophosphoribosyltransferase_precursor	RbromiiL2-36
Org4_Gene1219	core	647	[RbromiiL2-36]RBL236_01276_Adenylosuccinate_lyase	RbromiiL2-36
Org4_Gene1220	core	894	[RbromiiL2-36]RBL236_01277_Adenylosuccinate_synthetase	RbromiiL2-36
Org4_Gene1221	core	536	[RbromiiL2-36]RBL236_01278_GMP_synthase_[glutamine-hydrolyzing]	RbromiiL2-36
Org4_Gene1229	core	1440	[RbromiiL2-36]RBL236_01286_UDP-glucose_4-epimerase	RbromiiL2-36
Org4_Gene1230	core	572	[RbromiiL2-36]RBL236_01287_galactose-1-phosphate_uridylyltransferase	RbromiiL2-36
Org4_Gene1231	core	1288	[RbromiiL2-36]RBL236_01288_Aldose_1-epimerase_precursor	RbromiiL2-36
Org4_Gene1241	core	1537	[RbromiiL2-36]RBL236_01298_Modulator_of_FtsH_protease_HflK	RbromiiL2-36
Org4_Gene1243	core	3072	[RbromiiL2-36]RBL236_01300_N5-carboxyaminoimidazole_ribonucleotide_mutase	RbromiiL2-36
Org4_Gene1244	core	633	[RbromiiL2-36]RBL236_01301_Amidophosphoribosyltransferase_precursor	RbromiiL2-36
Org4_Gene1245	core	1351	[RbromiiL2-36]RBL236_01302_Phosphoribosylformylglycinamidine_cyclo-ligase	RbromiiL2-36
Org4_Gene1246	core	2614	[RbromiiL2-36]RBL236_01303_Phosphoribosylglycinamide_formyltransferase	RbromiiL2-36
Org4_Gene1247	core	2308	[RbromiiL2-36]RBL236_01304_IMP_cyclohydrolase	RbromiiL2-36
Org4_Gene1248	core	1076	[RbromiiL2-36]RBL236_01305_Bifunctional_purine_biosynthesis_protein_PurH	RbromiiL2-36
Org4_Gene1249	core	914	[RbromiiL2-36]RBL236_01306_Phosphoribosylamine--glycine_ligase	RbromiiL2-36
Org4_Gene1250	core	2136	[RbromiiL2-36]RBL236_01307_Stage_0_sporulation_protein_G	RbromiiL2-36
Org4_Gene1254	core	2223	[RbromiiL2-36]RBL236_01311_Arginine_transport_ATP-binding_protein_ArtM	RbromiiL2-36
Org4_Gene1256	core	1085	[RbromiiL2-36]RBL236_01313_NADP-dependent_malic_enzyme	RbromiiL2-36
Org4_Gene1259	core	517	[RbromiiL2-36]RBL236_01317_anaerobic_glycerol-3-phosphate_dehydrogenase_subunit_B	RbromiiL2-36
Org4_Gene1263	core	690	[RbromiiL2-36]RBL236_01321_Multidrug_export_protein_MepA	RbromiiL2-36
Org4_Gene1266	core	346	[RbromiiL2-36]RBL236_01324_B12-binding_domain/radical_SAM_domain_protein,_family	RbromiiL2-36
Org4_Gene1267	core	2509	[RbromiiL2-36]RBL236_01325_radical_SAM-linked_protein	RbromiiL2-36
Org4_Gene1271	core	2319	[RbromiiL2-36]RBL236_01330_Uridylate_kinase	RbromiiL2-36
Org4_Gene1272	core	2866	[RbromiiL2-36]RBL236_01331_Ribosome-releasing_factor	RbromiiL2-36
Org4_Gene1273	core	2189	[RbromiiL2-36]RBL236_01332_Ditrans,polycis-undecaprenyl-diphosphate_synthase_((2E,6E)-farnesyl-diphosphate_specific)	RbromiiL2-36
Org4_Gene1275	core	1147	[RbromiiL2-36]RBL236_01334_1-deoxy-D-xylulose_5-phosphate_reductoisomerase	RbromiiL2-36
Org4_Gene1277	core	1335	[RbromiiL2-36]RBL236_01336_4-hydroxy-3-methylbut-2-en-1-yl_diphosphate_synthase	RbromiiL2-36
Org4_Gene1278	core	21	[RbromiiL2-36]RBL236_01337_DNA_polymerase_III_PolC-type	RbromiiL2-36
Org4_Gene1280	core	1098	[RbromiiL2-36]RBL236_01339_ATP_phosphoribosyltransferase_regulatory_subunit	RbromiiL2-36
Org4_Gene1281	core	2601	[RbromiiL2-36]RBL236_01340_ATP_phosphoribosyltransferase	RbromiiL2-36
Org4_Gene1282	core	864	[RbromiiL2-36]RBL236_01341_Histidinol_dehydrogenase	RbromiiL2-36

Org4_Gene1283	core	1314	[RbromiiL2-36]RBL236_01342_Histidinol-phosphate_aminotransferase	RbromiiL2-36
Org4_Gene1284	core	2773	[RbromiiL2-36]RBL236_01343_Imidazoleglycerol-phosphate_dehydratase	RbromiiL2-36
Org4_Gene1285	core	2300	[RbromiiL2-36]RBL236_01344_1-(5-phosphoribosyl)-5-[(5-phosphoribosylamino)methylideneamino]_imidazole-4-carboxamide_isomerase	RbromiiL2-36
Org4_Gene1286	core	3785	[RbromiiL2-36]RBL236_01345_phosphoribosyl-AMP_cyclohydrolase	RbromiiL2-36
Org4_Gene1287	core	3697	[RbromiiL2-36]RBL236_01346_Phosphoribosyl-ATP_pyrophosphatase	RbromiiL2-36
Org4_Gene1289	core	869	[RbromiiL2-36]RBL236_01348_Threonylcarbamoyladenosine_tRNA_methylthiotransferase_MtaB	RbromiiL2-36
Org4_Gene13	core	3887	[RbromiiL2-36]RBL236_00013_50S_ribosomal_protein_L27	RbromiiL2-36
Org4_Gene1300	core	1179	[RbromiiL2-36]RBL236_01359_Queuine_tRNA-ribosyltransferase	RbromiiL2-36
Org4_Gene1301	core	1375	[RbromiiL2-36]RBL236_01360_S-adenosylmethionine:tRNA_ribosyltransferase-isomerase	RbromiiL2-36
Org4_Gene1302	core	1256	[RbromiiL2-36]RBL236_01361_Aspartate-semialdehyde_dehydrogenase_2	RbromiiL2-36
Org4_Gene1303	core	1686	[RbromiiL2-36]RBL236_01362_4-hydroxy-tetrahydrodipicolinate_synthase	RbromiiL2-36
Org4_Gene1304	core	2158	[RbromiiL2-36]RBL236_01363_4-hydroxy-tetrahydrodipicolinate_reductase	RbromiiL2-36
Org4_Gene1307	core	1734	[RbromiiL2-36]RBL236_01366_DisA_bacterial_checkpoint_controller_nucleotide-binding_protein	RbromiiL2-36
Org4_Gene1310	core	456	[RbromiiL2-36]RBL236_01369_Formate--tetrahydrofolate_ligase	RbromiiL2-36
Org4_Gene1311	core	3357	[RbromiiL2-36]RBL236_01370_ADP-binding_protein	RbromiiL2-36
Org4_Gene1313	core	3252	[RbromiiL2-36]RBL236_01372_Ribose-5-phosphate_isomerase_B	RbromiiL2-36
Org4_Gene1314	core	4002	[RbromiiL2-36]RBL236_01373_Helix-turn-helix_domain_protein	RbromiiL2-36
Org4_Gene1327	core	520	[RbromiiL2-36]RBL236_01387_Peptide_chain_release_factor_3	RbromiiL2-36
Org4_Gene1329	core	4381	[RbromiiL2-36]RBL236_01389_six-cysteine_peptide_SCIFF	RbromiiL2-36
Org4_Gene1330	core	724	[RbromiiL2-36]RBL236_01390_Anaerobic_sulfatase-maturating_enzyme	RbromiiL2-36
Org4_Gene1332	core	318	[RbromiiL2-36]RBL236_01392_Ferrous_iron_transport_protein_B	RbromiiL2-36
Org4_Gene1339	core	1789	[RbromiiL2-36]RBL236_01399_Methionine_aminopeptidase	RbromiiL2-36
Org4_Gene1341	core	734	[RbromiiL2-36]RBL236_01401_Lon_protease_2	RbromiiL2-36
Org4_Gene1343	core	1460	[RbromiiL2-36]RBL236_01403_Tyrosine_recombinase_XerD	RbromiiL2-36
Org4_Gene1344	core	796	[RbromiiL2-36]RBL236_01404_NADP-specific_glutamate_dehydrogenase	RbromiiL2-36
Org4_Gene1345	core	2370	[RbromiiL2-36]RBL236_01405_putative_membrane_protein	RbromiiL2-36
Org4_Gene1346	core	857	[RbromiiL2-36]RBL236_01406_Diaminopimelate_decarboxylase	RbromiiL2-36
Org4_Gene1347	core	2688	[RbromiiL2-36]RBL236_01407_putative_GTP-binding_protein_EngB	RbromiiL2-36
Org4_Gene1348	core	156	[RbromiiL2-36]RBL236_01408_Lon_protease_1	RbromiiL2-36
Org4_Gene1349	core	781	[RbromiiL2-36]RBL236_01409_ATP-dependent_Clp_protease_ATP-binding_subunit_ClpX	RbromiiL2-36
Org4_Gene1350	core	2763	[RbromiiL2-36]RBL236_01410_ATP-dependent_Clp_protease_proteolytic_subunit	RbromiiL2-36
Org4_Gene1351	core	890	[RbromiiL2-36]RBL236_01411_Trigger_factor	RbromiiL2-36
Org4_Gene1352	core	2412	[RbromiiL2-36]RBL236_01412_putative_phosphoesterase_or_phosphohydrolase	RbromiiL2-36
Org4_Gene1353	core	574	[RbromiiL2-36]RBL236_01413_Threonine_synthase	RbromiiL2-36
Org4_Gene1356	core	2656	[RbromiiL2-36]RBL236_01416_Ribonuclease_HII	RbromiiL2-36
Org4_Gene1357	core	1771	[RbromiiL2-36]RBL236_01417_Ribosome_biogenesis_GTPase_A	RbromiiL2-36
Org4_Gene1360	core	3645	[RbromiiL2-36]RBL236_01420_50S_ribosomal_protein_L19	RbromiiL2-36
Org4_Gene1361	core	1638	[RbromiiL2-36]RBL236_01421_Ribosomal_large_subunit_pseudouridine_synthase_D	RbromiiL2-36
Org4_Gene1362	core	3126	[RbromiiL2-36]RBL236_01422_Lipoprotein_signal_peptidase	RbromiiL2-36
Org4_Gene1363	core	102	[RbromiiL2-36]RBL236_01423_Isoleucine--tRNA_ligase	RbromiiL2-36
Org4_Gene1366	core	3192	[RbromiiL2-36]RBL236_01426_Cell_division_protein_SepF	RbromiiL2-36
Org4_Gene1367	core	2317	[RbromiiL2-36]RBL236_01427_alanine_racemase	RbromiiL2-36
Org4_Gene1371	core	127	[RbromiiL2-36]RBL236_01431_DNA_mismatch_repair_protein_MutS	RbromiiL2-36
Org4_Gene1372	core	3428	[RbromiiL2-36]RBL236_01432_hypothetical_protein	RbromiiL2-36
Org4_Gene1373	core	673	[RbromiiL2-36]RBL236_01433_(Dimethylallyl)adenosine_tRNA_methylthiotransferase_MiaB	RbromiiL2-36
Org4_Gene1376	core	2854	[RbromiiL2-36]RBL236_01436_Elongation_factor_P	RbromiiL2-36
Org4_Gene1378	core	3327	[RbromiiL2-36]RBL236_01438_3-dehydroquinate_dehydratase	RbromiiL2-36
Org4_Gene1379	core	4323	[RbromiiL2-36]RBL236_01439_30S_ribosomal_protein_S21	RbromiiL2-36
Org4_Gene1380	core	155	[RbromiiL2-36]RBL236_01440_Leucine--tRNA_ligase	RbromiiL2-36
Org4_Gene1381	core	3626	[RbromiiL2-36]RBL236_01441_Ribosomal_silencing_factor_RsfS	RbromiiL2-36
Org4_Gene1384	core	3821	[RbromiiL2-36]RBL236_01444_RNA-binding_protein	RbromiiL2-36
Org4_Gene1385	core	2674	[RbromiiL2-36]RBL236_01445_Non-canonical_purine_NTP_pyrophosphatase	RbromiiL2-36
Org4_Gene1386	core	1644	[RbromiiL2-36]RBL236_01446_Signal_recognition_particle_receptor_FtsY	RbromiiL2-36
Org4_Gene1387	core	47	[RbromiiL2-36]RBL236_01447_Chromosome_partition_protein_Smc	RbromiiL2-36
Org4_Gene1389	core	2444	[RbromiiL2-36]RBL236_01449_Ribonuclease_3	RbromiiL2-36
Org4_Gene1390	core	1426	[RbromiiL2-36]RBL236_01450_Phosphate_acyltransferase	RbromiiL2-36
Org4_Gene1391	core	828	[RbromiiL2-36]RBL236_01451_Methylenetetrahydrofolate--tRNA-(uracil-5-)-methyltransferase_TrmFO	RbromiiL2-36
Org4_Gene1392	core	249	[RbromiiL2-36]RBL236_01452_DNA_topoisomerase_1	RbromiiL2-36
Org4_Gene1395	core	3634	[RbromiiL2-36]RBL236_01455_50S_ribosomal_protein_L20	RbromiiL2-36
Org4_Gene1396	core	4231	[RbromiiL2-36]RBL236_01456_Ribosomal_protein_A	RbromiiL2-36
Org4_Gene1398	core	1659	[RbromiiL2-36]RBL236_01459_Elongation_factor_Ts	RbromiiL2-36
Org4_Gene1399	core	2264	[RbromiiL2-36]RBL236_01460_Vegetative_protein_209	RbromiiL2-36
Org4_Gene14	core	909	[RbromiiL2-36]RBL236_00014_Spo0B-associated_GTP-binding_protein	RbromiiL2-36
Org4_Gene1401	core	3854	[RbromiiL2-36]RBL236_01462_sporulation_protein,_YlmC/YmxH_family	RbromiiL2-36
Org4_Gene1402	core	1754	[RbromiiL2-36]RBL236_01463_DegV_domain-containing_protein	RbromiiL2-36
Org4_Gene1404	core	2379	[RbromiiL2-36]RBL236_01465_RNA_polymerase_sigma-35_factor_precursor	RbromiiL2-36
Org4_Gene1408	core	1483	[RbromiiL2-36]RBL236_01469_6-phosphofructokinase	RbromiiL2-36
Org4_Gene1409	core	1540	[RbromiiL2-36]RBL236_01470_putative_glycosyltransferase_YkoT	RbromiiL2-36
Org4_Gene1412	core	413	[RbromiiL2-36]RBL236_01473_Phosphoglucomutase	RbromiiL2-36
Org4_Gene1417	core	1282	[RbromiiL2-36]RBL236_01478_Aspartate_carbamoyltransferase_catalytic_chain	RbromiiL2-36
Org4_Gene1422	core	1801	[RbromiiL2-36]RBL236_01483_4-diphosphocytidyl-2-C-methyl-D-erythritol_kinase	RbromiiL2-36
Org4_Gene1423	core	1075	[RbromiiL2-36]RBL236_01484_Putative_N-acetyl-LL-diaminopimelate_aminotransferase	RbromiiL2-36
Org4_Gene1424	core	3130	[RbromiiL2-36]RBL236_01485_Leucine-responsive_regulatory_protein	RbromiiL2-36
Org4_Gene1426	core	313	[RbromiiL2-36]RBL236_01487_Glutamine-dependent_NAD(+)_synthetase	RbromiiL2-36
Org4_Gene1428	core	2431	[RbromiiL2-36]RBL236_01489_UDP-glucose:undecaprenyl-phosphate_glucose-1-phosphate_transferase	RbromiiL2-36
Org4_Gene143	core	2205	[RbromiiL2-36]RBL236_00146_Teichoic_acids_export_ATP-binding_protein_TagH	RbromiiL2-36
Org4_Gene1430	core	1512	[RbromiiL2-36]RBL236_01492_Glycerol-3-phosphate_dehydrogenase_[NAD(P)+]	RbromiiL2-36
Org4_Gene1435	core	3967	[RbromiiL2-36]RBL236_01497_hypothetical_protein	RbromiiL2-36
Org4_Gene1436	core	487	[RbromiiL2-36]RBL236_01498_Stress_protein_H5	RbromiiL2-36
Org4_Gene1437	core	3886	[RbromiiL2-36]RBL236_01499_co-chaperonin_GroES	RbromiiL2-36
Org4_Gene1438	core	480	[RbromiiL2-36]RBL236_01500_Serine/threonine-protein_kinase_PrkC	RbromiiL2-36
Org4_Gene144	core	1968	[RbromiiL2-36]RBL236_00147_Teichoic_acid_translocation_permease_protein_TagG	RbromiiL2-36
Org4_Gene1440	core	1753	[RbromiiL2-36]RBL236_01503_Putative_ribosome_biogenesis_GTPase_RsgA	RbromiiL2-36
Org4_Gene1441	core	238	[RbromiiL2-36]RBL236_01504_Serine/threonine-protein_kinase_PrkC	RbromiiL2-36
Org4_Gene1442	core	2278	[RbromiiL2-36]RBL236_01505_Serine/threonine_phosphatase_stp	RbromiiL2-36
Org4_Gene1443	core	1357	[RbromiiL2-36]RBL236_01506_putative_dual-specificity_RNA_methyltransferase_RlmN	RbromiiL2-36
Org4_Gene1445	core	2364	[RbromiiL2-36]RBL236_01508_Putative_neutral_zinc_metallopeptidase	RbromiiL2-36
Org4_Gene1446	core	1641	[RbromiiL2-36]RBL236_01509_Methionyl-tRNA_formyltransferase	RbromiiL2-36
Org4_Gene1447	core	3150	[RbromiiL2-36]RBL236_01510_Peptide_deformylase_1	RbromiiL2-36
Org4_Gene1448	core	144	[RbromiiL2-36]RBL236_01511_Primosomal_protein_N'	RbromiiL2-36
Org4_Gene1449	core	4190	[RbromiiL2-36]RBL236_01512_DNA-directed_RNA_polymerase_subunit_omega	RbromiiL2-36
Org4_Gene145	core	1251	[RbromiiL2-36]RBL236_00148_Spermidine/putrescine-binding_periplasmic_protein_precursor	RbromiiL2-36
Org4_Gene1451	core	3994	[RbromiiL2-36]RBL236_01514_hypothetical_protein	RbromiiL2-36
Org4_Gene1452	core	1747	[RbromiiL2-36]RBL236_01515_hypothetical_protein	RbromiiL2-36
Org4_Gene1453	core	2241	[RbromiiL2-36]RBL236_01516_putative_transcriptional_regulatory_protein_YebC	RbromiiL2-36
Org4_Gene1458	core	3965	[RbromiiL2-36]RBL236_01521_Phosphocarrier_protein_HPr	RbromiiL2-36
Org4_Gene146	core	1988	[RbromiiL2-36]RBL236_00149_Inner_membrane_ABC_transporter_permease_protein_YdcV	RbromiiL2-36
Org4_Gene1463	core	215	[RbromiiL2-36]RBL236_01526_Anaerobic_ribonucleoside-triphosphate_reductase	RbromiiL2-36
Org4_Gene1464	core	2925	[RbromiiL2-36]RBL236_01527_anaerobic_ribonucleotide_reductase-activating_protein	RbromiiL2-36
Org4_Gene1466	core	1015	[RbromiiL2-36]RBL236_01529_Aspartokinase	RbromiiL2-36
Org4_Gene1467	core	957	[RbromiiL2-36]RBL236_01530_Homoserine_dehydrogenase	RbromiiL2-36
Org4_Gene147	core	2069	[RbromiiL2-36]RBL236_00150_Putrescine_transport_system_permease_protein_PotH	RbromiiL2-36
Org4_Gene1470	core	1551	[RbromiiL2-36]RBL236_01533_Nitronate_monooxygenase	RbromiiL2-36
Org4_Gene1471	core	1688	[RbromiiL2-36]RBL236_01534_Malonyl_CoA-acyl_carrier_protein_transacylase	RbromiiL2-36
Org4_Gene1472	core	2214	[RbromiiL2-36]RBL236_01535_3-oxoacyl-[acyl-carrier-protein]_reductase_FabG	RbromiiL2-36
Org4_Gene1473	core	970	[RbromiiL2-36]RBL236_01536_3-oxoacyl-[acyl-carrier-protein]_synthase_2	RbromiiL2-36
Org4_Gene1475	core	3377	[RbromiiL2-36]RBL236_01538_3-hydroxyacyl-[acyl-carrier-protein]_dehydratase_FabZ	RbromiiL2-36
Org4_Gene1476	core	748	[RbromiiL2-36]RBL236_01539_2-oxoglutarate_carboxylase_small_subunit	RbromiiL2-36
Org4_Gene1477	core	1826	[RbromiiL2-36]RBL236_01540_Acetyl-coenzyme_A_carboxylase_carboxyl_transferase_subunit_beta	RbromiiL2-36
Org4_Gene1478	core	2088	[RbromiiL2-36]RBL236_01541_Acetyl-coenzyme_A_carboxylase_carboxyl_transferase_subunit_alpha	RbromiiL2-36
Org4_Gene148	core	659	[RbromiiL2-36]RBL236_00151_Spermidine/putrescine_import_ATP-binding_protein_PotA	RbromiiL2-36
Org4_Gene1487	core	408	[RbromiiL2-36]RBL236_01550_Arginine--tRNA_ligase	RbromiiL2-36
Org4_Gene1488	core	3199	[RbromiiL2-36]RBL236_01551_hypothetical_protein	RbromiiL2-36
Org4_Gene1489	core	1978	[RbromiiL2-36]RBL236_01552_Glutamate_racemase	RbromiiL2-36
Org4_Gene1490	core	2067	[RbromiiL2-36]RBL236_01553_hypothetical_protein	RbromiiL2-36
Org4_Gene1491	core	4393	[RbromiiL2-36]RBL236_01554_50S_ribosomal_protein_L33	RbromiiL2-36
Org4_Gene1493	core	2971	[RbromiiL2-36]RBL236_01556_hypothetical_protein	RbromiiL2-36
Org4_Gene1494	core	3352	[RbromiiL2-36]RBL236_01557_50S_ribosomal_protein_L11	RbromiiL2-36
Org4_Gene1495	core	2398	[RbromiiL2-36]RBL236_01558_50S_ribosomal_protein_L1	RbromiiL2-36
Org4_Gene1496	core	2838	[RbromiiL2-36]RBL236_01560_50S_ribosomal_protein_L10	RbromiiL2-36
Org4_Gene1497	core	3530	[RbromiiL2-36]RBL236_01561_hypothetical_protein	RbromiiL2-36
Org4_Gene1498	core	510	[RbromiiL2-36]RBL236_01562_Phosphoenolpyruvate_carboxykinase_[ATP]	RbromiiL2-36

Org4_Gene1499	core	3338	[RbromiiL2-36]RBL236_01563_hypothetical_protein	RbromiiL2-36
Org4_Gene15	core	3444	[RbromiiL2-36]RBL236_00015_Mini-ribonuclease_3	RbromiiL2-36
Org4_Gene1502	core	2814	[RbromiiL2-36]RBL236_01566_Phosphate_propanoyltransferase	RbromiiL2-36
Org4_Gene1503	core	2781	[RbromiiL2-36]RBL236_01567_uracil-DNA_glycosylase,_family_4	RbromiiL2-36
Org4_Gene1506	core	399	[RbromiiL2-36]RBL236_01570_Aspartate--tRNA_ligase	RbromiiL2-36
Org4_Gene1507	core	3910	[RbromiiL2-36]RBL236_01571_Aspartyl/glutamyl-tRNA(Asn/Gln)_amidotransferase_subunit_C	RbromiiL2-36
Org4_Gene1508	core	628	[RbromiiL2-36]RBL236_01572_Glutamyl-tRNA(Gln)_amidotransferase_subunit_A	RbromiiL2-36
Org4_Gene1509	core	654	[RbromiiL2-36]RBL236_01573_Aspartyl/glutamyl-tRNA(Asn/Gln)_amidotransferase_subunit_B	RbromiiL2-36
Org4_Gene1512	core	717	[RbromiiL2-36]RBL236_01576_Tryptophan_synthase_beta_chain	RbromiiL2-36
Org4_Gene1516	core	2184	[RbromiiL2-36]RBL236_01580_Phosphate_import_ATP-binding_protein_PstB_3	RbromiiL2-36
Org4_Gene1524	core	449	[RbromiiL2-36]RBL236_01588_Hydroxylamine_reductase	RbromiiL2-36
Org4_Gene153	core	210	[RbromiiL2-36]RBL236_00156_Exodeoxyribonuclease_V_alpha_chain	RbromiiL2-36
Org4_Gene1536	core	2103	[RbromiiL2-36]RBL236_01600_Stage_II_sporulation_protein_AC	RbromiiL2-36
Org4_Gene1537	core	3832	[RbromiiL2-36]RBL236_01601_hypothetical_protein	RbromiiL2-36
Org4_Gene1539	core	1730	[RbromiiL2-36]RBL236_01603_hypothetical_protein	RbromiiL2-36
Org4_Gene1541	core	2031	[RbromiiL2-36]RBL236_01608_Spermidine/putrescine_import_ATP-binding_protein_PotA	RbromiiL2-36
Org4_Gene1542	core	1485	[RbromiiL2-36]RBL236_01609_autoinducer_2_ABC_transporter_permease_LsrC	RbromiiL2-36
Org4_Gene1544	core	52	[RbromiiL2-36]RBL236_01611_DNA_polymerase_III_subunit_alpha	RbromiiL2-36
Org4_Gene1546	core	1552	[RbromiiL2-36]RBL236_01613_t(6)A37_threonylcarbamoyladenosine_biosynthesis_protein	RbromiiL2-36
Org4_Gene1549	core	3249	[RbromiiL2-36]RBL236_01616_stage_III_sporulation_protein_AG	RbromiiL2-36
Org4_Gene155	core	817	[RbromiiL2-36]RBL236_00158_putative_bifunctional_UDP-N-acetylmuramoylalanyl-D-glutamate--2,6-diaminopimelate_ligase/UDP-N-acetylmuramoyl-tripe	RbromiiL2-36
Org4_Gene1553	core	4250	[RbromiiL2-36]RBL236_01620_stage_III_sporulation_protein_AC	RbromiiL2-36
Org4_Gene1557	core	283	[RbromiiL2-36]RBL236_01624_DNA_ligase	RbromiiL2-36
Org4_Gene1558	core	2771	[RbromiiL2-36]RBL236_01625_putative_HD-superfamily_hydrolase	RbromiiL2-36
Org4_Gene1560	core	1128	[RbromiiL2-36]RBL236_01627_Ribosomal_RNA_large_subunit_methyltransferase_K/L	RbromiiL2-36
Org4_Gene1561	core	2936	[RbromiiL2-36]RBL236_01628_SigL_modulation_protein	RbromiiL2-36
Org4_Gene1562	core	3096	[RbromiiL2-36]RBL236_01629_General_stress_protein_13	RbromiiL2-36
Org4_Gene1564	core	3939	[RbromiiL2-36]RBL236_01631_Spore_protein_YabP	RbromiiL2-36
Org4_Gene1565	core	4042	[RbromiiL2-36]RBL236_01632_ribosome-associated_heat_shock_protein_Hsp15	RbromiiL2-36
Org4_Gene1566	core	3918	[RbromiiL2-36]RBL236_01633_hypothetical_protein	RbromiiL2-36
Org4_Gene1567	core	2036	[RbromiiL2-36]RBL236_01634_Nucleoside_triphosphate_pyrophosphohydrolase	RbromiiL2-36
Org4_Gene1570	core	624	[RbromiiL2-36]RBL236_01637_Oxygen-independent_coproporphyrinogen-III_oxidase_2	RbromiiL2-36
Org4_Gene1571	core	2613	[RbromiiL2-36]RBL236_01638_putative_polyketide_biosynthesis_zinc-dependent_hydrolase_PksB	RbromiiL2-36
Org4_Gene1572	core	3237	[RbromiiL2-36]RBL236_01639_D-tyrosyl-tRNA(Tyr)_deacylase	RbromiiL2-36
Org4_Gene1573	core	212	[RbromiiL2-36]RBL236_01640_GTP_pyrophosphokinase	RbromiiL2-36
Org4_Gene1575	core	457	[RbromiiL2-36]RBL236_01642_Recombination_protein_N	RbromiiL2-36
Org4_Gene1578	core	2015	[RbromiiL2-36]RBL236_01645_Hemolysin_A	RbromiiL2-36
Org4_Gene1579	core	340	[RbromiiL2-36]RBL236_01646_1-deoxy-D-xylulose-5-phosphate_synthase	RbromiiL2-36
Org4_Gene158	core	937	[RbromiiL2-36]RBL236_00161_GTP-binding_protein_HflX	RbromiiL2-36
Org4_Gene1580	core	1774	[RbromiiL2-36]RBL236_01647_Farnesyl_diphosphate_synthase	RbromiiL2-36
Org4_Gene1582	core	1590	[RbromiiL2-36]RBL236_01649_Putative_sporulation_transcription_regulator_WhiA	RbromiiL2-36
Org4_Gene1583	core	1818	[RbromiiL2-36]RBL236_01650_glmZ(sRNA)-inactivating_NTPase	RbromiiL2-36
Org4_Gene1584	core	1679	[RbromiiL2-36]RBL236_01651_UDP-N-acetylenolpyruvoylglucosamine_reductase	RbromiiL2-36
Org4_Gene1585	core	1495	[RbromiiL2-36]RBL236_01652_HPr_kinase/phosphorylase	RbromiiL2-36
Org4_Gene1588	core	622	[RbromiiL2-36]RBL236_01655_Spore_germination_protein_B1	RbromiiL2-36
Org4_Gene1594	core	1378	[RbromiiL2-36]RBL236_01661_preprotein_translocase_subunit_SecF	RbromiiL2-36
Org4_Gene1595	core	663	[RbromiiL2-36]RBL236_01662_preprotein_translocase_subunit_SecD	RbromiiL2-36
Org4_Gene1596	core	886	[RbromiiL2-36]RBL236_01663_Putative_aminotransferase/MSMEI_6121	RbromiiL2-36
Org4_Gene1599	core	956	[RbromiiL2-36]RBL236_01666_Glutaconyl-CoA_decarboxylase_subunit_beta	RbromiiL2-36
Org4_Gene1601	core	603	[RbromiiL2-36]RBL236_01668_Stage_IV_sporulation_protein_A	RbromiiL2-36
Org4_Gene1604	core	1509	[RbromiiL2-36]RBL236_01671_Ribosomal_large_subunit_pseudouridine_synthase_C	RbromiiL2-36
Org4_Gene1607	core	1923	[RbromiiL2-36]RBL236_01674_Laccase_domain_protein	RbromiiL2-36
Org4_Gene1609	core	2927	[RbromiiL2-36]RBL236_01676_Inorganic_pyrophosphatase	RbromiiL2-36
Org4_Gene161	core	2452	[RbromiiL2-36]RBL236_00164_Bifunctional_protein_GlmU	RbromiiL2-36
Org4_Gene1612	core	124	[RbromiiL2-36]RBL236_01679_Calcium-transporting_ATPase	RbromiiL2-36
Org4_Gene1614	core	1855	[RbromiiL2-36]RBL236_01682_Ribosomal_RNA_small_subunit_methyltransferase_A	RbromiiL2-36
Org4_Gene1615	core	818	[RbromiiL2-36]RBL236_01683_Ribosomal_RNA_small_subunit_methyltransferase_F	RbromiiL2-36
Org4_Gene1617	core	2500	[RbromiiL2-36]RBL236_01685_hypothetical_protein	RbromiiL2-36
Org4_Gene1618	core	2897	[RbromiiL2-36]RBL236_01686_N-acetylmuramoyl-L-alanine_amidase_CwlD	RbromiiL2-36
Org4_Gene1626	core	1105	[RbromiiL2-36]RBL236_01694_H(2)O-forming_NADH_oxidase	RbromiiL2-36
Org4_Gene1629	core	207	[RbromiiL2-36]RBL236_01697_Polyribonucleotide_nucleotidyltransferase	RbromiiL2-36
Org4_Gene1630	core	3962	[RbromiiL2-36]RBL236_01698_hypothetical_protein	RbromiiL2-36
Org4_Gene1633	core	746	[RbromiiL2-36]RBL236_01702_Maltose-binding_periplasmic_proteins/domains	RbromiiL2-36
Org4_Gene1634	core	1769	[RbromiiL2-36]RBL236_01703_L-arabinose_transport_system_permease_protein_AraP	RbromiiL2-36
Org4_Gene1635	core	1768	[RbromiiL2-36]RBL236_01704_L-arabinose_transport_system_permease_protein_AraQ	RbromiiL2-36
Org4_Gene1636	core	174	[RbromiiL2-36]RBL236_01705_Heat_shock_protein_HSP1	RbromiiL2-36
Org4_Gene164	core	3632	[RbromiiL2-36]RBL236_00170_Nucleoid-associated_protein	RbromiiL2-36
Org4_Gene1640	core	2028	[RbromiiL2-36]RBL236_01709_tRNA_pseudouridine_synthase_A	RbromiiL2-36
Org4_Gene1641	core	1998	[RbromiiL2-36]RBL236_01710_Energy-coupling_factor_transporter_transmembrane_protein_EcfT	RbromiiL2-36
Org4_Gene1642	core	1805	[RbromiiL2-36]RBL236_01711_Energy-coupling_factor_transporter_ATP-binding_protein_EcfA2	RbromiiL2-36
Org4_Gene1643	core	1885	[RbromiiL2-36]RBL236_01712_Energy-coupling_factor_transporter_ATP-binding_protein_EcfA1	RbromiiL2-36
Org4_Gene1644	core	1821	[RbromiiL2-36]RBL236_01713_Ribosomal_RNA_large_subunit_methyltransferase_K	RbromiiL2-36
Org4_Gene1645	core	761	[RbromiiL2-36]RBL236_01714_Phosphoglucosamine_mutase	RbromiiL2-36
Org4_Gene1646	core	1247	[RbromiiL2-36]RBL236_01715_Holliday_junction_ATP-dependent_DNA_helicase_RuvB	RbromiiL2-36
Org4_Gene1647	core	2683	[RbromiiL2-36]RBL236_01716_Holliday_junction_ATP-dependent_DNA_helicase_RuvA	RbromiiL2-36
Org4_Gene1648	core	3035	[RbromiiL2-36]RBL236_01717_Crossover_junction_endodeoxyribonuclease_RuvC	RbromiiL2-36
Org4_Gene165	core	2679	[RbromiiL2-36]RBL236_00171_Recombination_protein_RecR	RbromiiL2-36
Org4_Gene1650	core	1408	[RbromiiL2-36]RBL236_01719_hypothetical_protein	RbromiiL2-36
Org4_Gene1652	core	435	[RbromiiL2-36]RBL236_01721_Neopullulanase	RbromiiL2-36
Org4_Gene1653	core	251	[RbromiiL2-36]RBL236_01722_Glycogen_debranching_enzyme	RbromiiL2-36
Org4_Gene166	core	3221	[RbromiiL2-36]RBL236_00172_SsrA-binding_protein	RbromiiL2-36
Org4_Gene1718	core	4026	[RbromiiL2-36]RBL236_01794_hypothetical_protein	RbromiiL2-36
Org4_Gene1724	core	2795	[RbromiiL2-36]RBL236_01804_Dipicolinate_synthase_subunit_B	RbromiiL2-36
Org4_Gene1729	core	905	[RbromiiL2-36]RBL236_01809_Methionine_gamma-lyase	RbromiiL2-36
Org4_Gene1730	core	2362	[RbromiiL2-36]RBL236_01810_2-C-methyl-D-erythritol_4-phosphate_cytidylyltransferase	RbromiiL2-36
Org4_Gene1731	core	3164	[RbromiiL2-36]RBL236_01811_2-C-methyl-D-erythritol_2,4-cyclodiphosphate_synthase	RbromiiL2-36
Org4_Gene1732	core	1141	[RbromiiL2-36]RBL236_01812_D-alanyl-D-alanine_carboxypeptidase_DacF_precursor	RbromiiL2-36
Org4_Gene1733	core	787	[RbromiiL2-36]RBL236_01813_Glucose-6-phosphate_isomerase	RbromiiL2-36
Org4_Gene1734	core	3391	[RbromiiL2-36]RBL236_01814_hypothetical_protein	RbromiiL2-36
Org4_Gene1741	core	2912	[RbromiiL2-36]RBL236_01821_NADH_peroxidase	RbromiiL2-36
Org4_Gene1743	core	2169	[RbromiiL2-36]RBL236_01823_Exodeoxyribonuclease	RbromiiL2-36
Org4_Gene1759	core	1341	[RbromiiL2-36]RBL236_01839_Ketol-acid_reductoisomerase	RbromiiL2-36
Org4_Gene1760	core	3087	[RbromiiL2-36]RBL236_01840_Acetolactate_synthase_small_subunit	RbromiiL2-36
Org4_Gene1761	core	494	[RbromiiL2-36]RBL236_01841_Acetolactate_synthase_large_subunit	RbromiiL2-36
Org4_Gene1764	core	3702	[RbromiiL2-36]RBL236_01844_hypothetical_protein	RbromiiL2-36
Org4_Gene1765	core	738	[RbromiiL2-36]RBL236_01845_UDP-N-acetylglucosamine_1-carboxyvinyltransferase_2	RbromiiL2-36
Org4_Gene1766	core	2075	[RbromiiL2-36]RBL236_01846_Putative_metallo-hydrolase_YycJ	RbromiiL2-36
Org4_Gene1767	core	3115	[RbromiiL2-36]RBL236_01847_Ribosomal_RNA_large_subunit_methyltransferase_H	RbromiiL2-36
Org4_Gene1768	core	403	[RbromiiL2-36]RBL236_01848_hypothetical_protein	RbromiiL2-36
Org4_Gene1769	core	1766	[RbromiiL2-36]RBL236_01849_anaerobic_ribonucleoside-triphosphate_reductase_activating_protein	RbromiiL2-36
Org4_Gene1772	core	1274	[RbromiiL2-36]RBL236_01852_tRNA-specific_2-thiouridylase_MnmA	RbromiiL2-36
Org4_Gene1773	core	1649	[RbromiiL2-36]RBL236_01853_Quinolinate_synthase_A	RbromiiL2-36
Org4_Gene1777	core	1069	[RbromiiL2-36]RBL236_01857_Putative_transcriptional_regulator_YvhJ	RbromiiL2-36
Org4_Gene1778	core	2744	[RbromiiL2-36]RBL236_01858_5'-nucleotidase_YfbR	RbromiiL2-36
Org4_Gene1780	core	743	[RbromiiL2-36]RBL236_01860_Beta-Ala-Xaa_dipeptidase	RbromiiL2-36
Org4_Gene1782	core	1262	[RbromiiL2-36]RBL236_01862_N-glycosyltransferase	RbromiiL2-36
Org4_Gene1783	core	1664	[RbromiiL2-36]RBL236_01863_LPS_biosynthesis_protein	RbromiiL2-36
Org4_Gene1787	core	657	[RbromiiL2-36]RBL236_01869_Glycogen_synthase	RbromiiL2-36
Org4_Gene1788	core	1212	[RbromiiL2-36]RBL236_01870_Glycogen_biosynthesis_protein_GlgD	RbromiiL2-36
Org4_Gene1789	core	999	[RbromiiL2-36]RBL236_01871_Glucose-1-phosphate_adenylyltransferase	RbromiiL2-36
Org4_Gene1791	core	1352	[RbromiiL2-36]RBL236_01873_Glyceraldehyde-3-phosphate_dehydrogenase	RbromiiL2-36
Org4_Gene1792	core	537	[RbromiiL2-36]RBL236_01874_Ribonuclease_Y	RbromiiL2-36
Org4_Gene1793	core	938	[RbromiiL2-36]RBL236_01875_Ammonia_transporter	RbromiiL2-36
Org4_Gene1794	core	3504	[RbromiiL2-36]RBL236_01876_Nitrogen_regulatory_protein_P-II	RbromiiL2-36
Org4_Gene1796	core	461	[RbromiiL2-36]RBL236_01878_Long-chain-fatty-acid--CoA_ligase	RbromiiL2-36
Org4_Gene1797	core	981	[RbromiiL2-36]RBL236_01879_3-oxoacyl-[acyl-carrier-protein]_synthase_2	RbromiiL2-36
Org4_Gene1799	core	990	[RbromiiL2-36]RBL236_01881_Aldehyde-alcohol_dehydrogenase	RbromiiL2-36
Org4_Gene1804	core	926	[RbromiiL2-36]RBL236_01886_putative_ion_channel_protein	RbromiiL2-36
Org4_Gene1814	core	2188	[RbromiiL2-36]RBL236_01896_Sulfur_carrier_protein_ThiS_adenylyltransferase	RbromiiL2-36
Org4_Gene1853	core	231	[RbromiiL2-36]RBL236_01935_Silver_exporting_P-type_ATPase	RbromiiL2-36
Org4_Gene1862	core	2898	[RbromiiL2-36]RBL236_01944_hypothetical_protein	RbromiiL2-36
Org4_Gene1879	core	3244	[RbromiiL2-36]RBL236_01961_3-hydroxyacyl-[acyl-carrier-protein]_dehydratase_FabZ	RbromiiL2-36

Org4_Gene1880	core	2246	[RbromiiL2-36]RBL236_01962_3-oxoacyl-[acyl-carrier-protein]_reductase_FabG	RbromiiL2-36
Org4_Gene1882	core	4031	[RbromiiL2-36]RBL236_01964_Acyl_carrier_protein	RbromiiL2-36
Org4_Gene1890	core	2494	[RbromiiL2-36]RBL236_01974_V-type_sodium_pump_subunit_D	RbromiiL2-36
Org4_Gene1891	core	723	[RbromiiL2-36]RBL236_01975_V-type_sodium_pump_subunit_B	RbromiiL2-36
Org4_Gene1892	core	384	[RbromiiL2-36]RBL236_01976_V-type_sodium_ATPase_catalytic_subunit_A	RbromiiL2-36
Org4_Gene1893	core	3757	[RbromiiL2-36]RBL236_01977_V-type_sodium_pump_subunit_G	RbromiiL2-36
Org4_Gene1896	core	3160	[RbromiiL2-36]RBL236_01980_Sodium_ATPase_proteolipid_component	RbromiiL2-36
Org4_Gene19	core	1964	[RbromiiL2-36]RBL236_00019_enhanced_serine_sensitivity_protein_SseB	RbromiiL2-36
Org4_Gene1900	core	2892	[RbromiiL2-36]RBL236_01984_Cys-tRNA(Pro)/Cys-tRNA(Cys)_deacylase_YbaK	RbromiiL2-36
Org4_Gene1923	core	1118	[RbromiiL2-36]RBL236_02007_hypothetical_protein	RbromiiL2-36
Org4_Gene1931	core	2038	[RbromiiL2-36]RBL236_02015_Pyrroline-5-carboxylate_reductase	RbromiiL2-36
Org4_Gene1937	core	4043	[RbromiiL2-36]RBL236_02021_hypothetical_protein	RbromiiL2-36
Org4_Gene1938	core	898	[RbromiiL2-36]RBL236_02022_DNA_polymerase_IV	RbromiiL2-36
Org4_Gene1940	core	2443	[RbromiiL2-36]RBL236_02024_Orotate_phosphoribosyltransferase	RbromiiL2-36
Org4_Gene1941	core	2348	[RbromiiL2-36]RBL236_02025_DNA_repair_protein_RadC	RbromiiL2-36
Org4_Gene1942	core	290	[RbromiiL2-36]RBL236_02026_Excinuclease_ABC_subunit_B	RbromiiL2-36
Org4_Gene1943	core	282	[RbromiiL2-36]RBL236_02027_DNA_gyrase_subunit_B	RbromiiL2-36
Org4_Gene1944	core	203	[RbromiiL2-36]RBL236_02028_DNA_gyrase_subunit_A	RbromiiL2-36
Org4_Gene1945	core	4015	[RbromiiL2-36]RBL236_02029_preprotein_translocase_subunit_SecG	RbromiiL2-36
Org4_Gene196	core	3710	[RbromiiL2-36]RBL236_00203_hypothetical_protein	RbromiiL2-36
Org4_Gene1970	core	907	[RbromiiL2-36]RBL236_02054_Dihydroorotase	RbromiiL2-36
Org4_Gene1971	core	1469	[RbromiiL2-36]RBL236_02055_4-hydroxy-2-oxovalerate_aldolase	RbromiiL2-36
Org4_Gene1972	core	1588	[RbromiiL2-36]RBL236_02056_orotidine_5'-phosphate_decarboxylase	RbromiiL2-36
Org4_Gene1973	core	1255	[RbromiiL2-36]RBL236_02057_Phosphoserine_aminotransferase	RbromiiL2-36
Org4_Gene1974	core	1125	[RbromiiL2-36]RBL236_02058_D-3-phosphoglycerate_dehydrogenase	RbromiiL2-36
Org4_Gene1981	core	1383	[RbromiiL2-36]RBL236_02065_Tryptophan--tRNA_ligase	RbromiiL2-36
Org4_Gene1982	core	1056	[RbromiiL2-36]RBL236_02066_2-aminoadipate_transaminase	RbromiiL2-36
Org4_Gene1984	core	2391	[RbromiiL2-36]RBL236_02068_Putative_mutator_protein_MutT4	RbromiiL2-36
Org4_Gene1986	core	2166	[RbromiiL2-36]RBL236_02070_Putative_TrmH_family_tRNA/rRNA_methyltransferase	RbromiiL2-36
Org4_Gene1988	core	2522	[RbromiiL2-36]RBL236_02072_Ribulose-phosphate_3-epimerase	RbromiiL2-36
Org4_Gene1989	core	1291	[RbromiiL2-36]RBL236_02073_methyltransferase,_FkbM_family	RbromiiL2-36
Org4_Gene1990	core	958	[RbromiiL2-36]RBL236_02075_orc1/cdc6_family_replication_initiation_protein	RbromiiL2-36
Org4_Gene1992	core	1904	[RbromiiL2-36]RBL236_02077_DNA-3-methyladenine_glycosylase	RbromiiL2-36
Org4_Gene1993	core	2411	[RbromiiL2-36]RBL236_02078_hypothetical_protein	RbromiiL2-36
Org4_Gene1997	core	2836	[RbromiiL2-36]RBL236_02082_putative_manganese_efflux_pump_MntP	RbromiiL2-36
Org4_Gene1999	core	1890	[RbromiiL2-36]RBL236_02084_Dihydroorotate_oxidase_B,_electron_transfer_subunit	RbromiiL2-36
Org4_Gene2000	core	700	[RbromiiL2-36]RBL236_02085_Glutamate_synthase_[NADPH]_small_chain	RbromiiL2-36
Org4_Gene2002	core	636	[RbromiiL2-36]RBL236_02087_Trk_system_potassium_uptake_protein_TrkG	RbromiiL2-36
Org4_Gene2004	core	1782	[RbromiiL2-36]RBL236_02089_Germination_protease_precursor	RbromiiL2-36
Org4_Gene2006	core	913	[RbromiiL2-36]RBL236_02091_Replication-associated_recombination_protein_A	RbromiiL2-36
Org4_Gene2008	core	153	[RbromiiL2-36]RBL236_02093_Beta/alpha-amylase_precursor	RbromiiL2-36
Org4_Gene2014	core	891	[RbromiiL2-36]RBL236_02099_phage_terminase,_large_subunit,_PBSX_family	RbromiiL2-36
Org4_Gene2018	core	1409	[RbromiiL2-36]RBL236_02103_phage_major_capsid_protein,_HK97_family	RbromiiL2-36
Org4_Gene2034	core	120	[RbromiiL2-36]RBL236_02119_Pyruvate,_phosphate_dikinase	RbromiiL2-36
Org4_Gene2035	core	2182	[RbromiiL2-36]RBL236_02120_Bifunctional_protein_GlmU	RbromiiL2-36
Org4_Gene2036	core	1519	[RbromiiL2-36]RBL236_02121_Ribose-phosphate_pyrophosphokinase	RbromiiL2-36
Org4_Gene2038	core	2720	[RbromiiL2-36]RBL236_02123_Peptidyl-tRNA_hydrolase	RbromiiL2-36
Org4_Gene2039	core	56	[RbromiiL2-36]RBL236_02124_Transcription-repair-coupling_factor	RbromiiL2-36
Org4_Gene204	core	1660	[RbromiiL2-36]RBL236_00211_Daunorubicin/doxorubicin_resistance_ATP-binding_protein_DrrA	RbromiiL2-36
Org4_Gene2042	core	4115	[RbromiiL2-36]RBL236_02127_hypothetical_protein	RbromiiL2-36
Org4_Gene2043	core	415	[RbromiiL2-36]RBL236_02128_MutS2_protein	RbromiiL2-36
Org4_Gene2046	core	4093	[RbromiiL2-36]RBL236_02131_hypothetical_protein	RbromiiL2-36
Org4_Gene2047	core	996	[RbromiiL2-36]RBL236_02132_Acetate_kinase	RbromiiL2-36
Org4_Gene2050	core	123	[RbromiiL2-36]RBL236_02135_Aldehyde-alcohol_dehydrogenase	RbromiiL2-36
Org4_Gene2051	core	2195	[RbromiiL2-36]RBL236_02136_serine/threonine_protein_kinase	RbromiiL2-36
Org4_Gene2054	core	1129	[RbromiiL2-36]RBL236_02139_tRNA_2-thiocytidine_biosynthesis_protein_TtcA	RbromiiL2-36
Org4_Gene2056	core	40	[RbromiiL2-36]RBL236_02141_DNA-directed_RNA_polymerase_subunit_beta	RbromiiL2-36
Org4_Gene2057	core	44	[RbromiiL2-36]RBL236_02142_DNA-directed_RNA_polymerase_subunit_beta'	RbromiiL2-36
Org4_Gene2059	core	3350	[RbromiiL2-36]RBL236_02144_Putative_Holliday_junction_resolvase	RbromiiL2-36
Org4_Gene2070	core	189	[RbromiiL2-36]RBL236_02156_1,4-alpha-glucan_branching_enzyme_GlgB	RbromiiL2-36
Org4_Gene2071	core	416	[RbromiiL2-36]RBL236_02157_putative_membrane_protein	RbromiiL2-36
Org4_Gene2072	core	1671	[RbromiiL2-36]RBL236_02158_NTE_family_protein_RssA	RbromiiL2-36
Org4_Gene2074	core	4312	[RbromiiL2-36]RBL236_02160_hypothetical_protein	RbromiiL2-36
Org4_Gene2075	core	1276	[RbromiiL2-36]RBL236_02161_Peptide_chain_release_factor_1	RbromiiL2-36
Org4_Gene2076	core	1337	[RbromiiL2-36]RBL236_02162_t(6)A37_threonylcarbamoyladenosine_biosynthesis_protein_RimN	RbromiiL2-36
Org4_Gene2079	core	675	[RbromiiL2-36]RBL236_02165_putative_M18_family_aminopeptidase_1	RbromiiL2-36
Org4_Gene2086	core	1555	[RbromiiL2-36]RBL236_02174_Glycerate_dehydrogenase	RbromiiL2-36
Org4_Gene2088	core	37	[RbromiiL2-36]RBL236_02176_Phosphoribosylformylglycinamidine_synthase_2	RbromiiL2-36
Org4_Gene2096	core	2140	[RbromiiL2-36]RBL236_02184_Type_III_pantothenate_kinase	RbromiiL2-36
Org4_Gene2097	core	1872	[RbromiiL2-36]RBL236_02185_Transketolase	RbromiiL2-36
Org4_Gene2098	core	1554	[RbromiiL2-36]RBL236_02186_1-deoxy-D-xylulose-5-phosphate_synthase	RbromiiL2-36
Org4_Gene21	core	2177	[RbromiiL2-36]RBL236_00021_Imidazole_glycerol_phosphate_synthase_subunit_HisF	RbromiiL2-36
Org4_Gene2100	core	1238	[RbromiiL2-36]RBL236_02188_Ribosome-binding_ATPase_YchF	RbromiiL2-36
Org4_Gene2101	core	3496	[RbromiiL2-36]RBL236_02189_Fluoroacetyl-CoA_thioesterase	RbromiiL2-36
Org4_Gene2105	core	1217	[RbromiiL2-36]RBL236_02193_Trehalose_import_ATP-binding_protein_SugC	RbromiiL2-36
Org4_Gene2106	core	1258	[RbromiiL2-36]RBL236_02194_Sugar_diacid_regulator	RbromiiL2-36
Org4_Gene2107	core	2132	[RbromiiL2-36]RBL236_02195_Cell_division_ATP-binding_protein_FtsE	RbromiiL2-36
Org4_Gene2111	core	3129	[RbromiiL2-36]RBL236_02199_Transcript_cleavage_factor_GreA	RbromiiL2-36
Org4_Gene2112	core	310	[RbromiiL2-36]RBL236_02200_Lysine--tRNA_ligase	RbromiiL2-36
Org4_Gene2118	core	3055	[RbromiiL2-36]RBL236_02206_Spore_maturation_protein_B	RbromiiL2-36
Org4_Gene2119	core	2757	[RbromiiL2-36]RBL236_02207_Spore_maturation_protein_A	RbromiiL2-36
Org4_Gene2120	core	4046	[RbromiiL2-36]RBL236_02208_transcriptional_regulator,_AbrB_family	RbromiiL2-36
Org4_Gene2124	core	243	[RbromiiL2-36]RBL236_02212_Membrane_protein_YdfJ	RbromiiL2-36
Org4_Gene2127	core	446	[RbromiiL2-36]RBL236_02215_2-isopropylmalate_synthase	RbromiiL2-36
Org4_Gene22	core	2620	[RbromiiL2-36]RBL236_00022_Imidazole_glycerol_phosphate_synthase_subunit_HisH_1	RbromiiL2-36
Org4_Gene221	core	918	[RbromiiL2-36]RBL236_00232_2,3-dimethylmalate_dehydratase_large_subunit	RbromiiL2-36
Org4_Gene222	core	3136	[RbromiiL2-36]RBL236_00233_2,3-dimethylmalate_dehydratase_small_subunit	RbromiiL2-36
Org4_Gene224	core	171	[RbromiiL2-36]RBL236_00235_Glycogen_phosphorylase	RbromiiL2-36
Org4_Gene225	core	590	[RbromiiL2-36]RBL236_00236_4-alpha-glucanotransferase	RbromiiL2-36
Org4_Gene226	core	2857	[RbromiiL2-36]RBL236_00237_Nicotinamide-nucleotide_amidohydrolase_PncC	RbromiiL2-36
Org4_Gene227	core	2255	[RbromiiL2-36]RBL236_00238_Ribosomal_RNA_small_subunit_methyltransferase_E	RbromiiL2-36
Org4_Gene228	core	2393	[RbromiiL2-36]RBL236_00239_5'-methylthioadenosine/S-adenosylhomocysteine_nucleosidase	RbromiiL2-36
Org4_Gene229	core	453	[RbromiiL2-36]RBL236_00240_Acetolactate_synthase_large_subunit	RbromiiL2-36
Org4_Gene23	core	1715	[RbromiiL2-36]RBL236_00023_MurPQ_operon_repressor	RbromiiL2-36
Org4_Gene230	core	592	[RbromiiL2-36]RBL236_00241_Uracil_transporter	RbromiiL2-36
Org4_Gene233	core	2595	[RbromiiL2-36]RBL236_00244_Uracil_phosphoribosyltransferase	RbromiiL2-36
Org4_Gene237	core	2359	[RbromiiL2-36]RBL236_00248_gliding_motility-associated_ABC_transporter_permease_protein_GldF	RbromiiL2-36
Org4_Gene238	core	1527	[RbromiiL2-36]RBL236_00249_putative_ABC_transporter_ATP-binding_protein_YxlF	RbromiiL2-36
Org4_Gene239	core	3621	[RbromiiL2-36]RBL236_00250_stage_V_sporulation_protein_AE	RbromiiL2-36
Org4_Gene24	core	952	[RbromiiL2-36]RBL236_00024_tricarballylate_dehydrogenase	RbromiiL2-36
Org4_Gene242	core	1484	[RbromiiL2-36]RBL236_00253_Putative_glycosyltransferase_CsbB	RbromiiL2-36
Org4_Gene244	core	3447	[RbromiiL2-36]RBL236_00255_30S_ribosomal_protein_S9	RbromiiL2-36
Org4_Gene245	core	3339	[RbromiiL2-36]RBL236_00256_50S_ribosomal_protein_L13	RbromiiL2-36
Org4_Gene247	core	1533	[RbromiiL2-36]RBL236_00261_Glucokinase	RbromiiL2-36
Org4_Gene248	core	600	[RbromiiL2-36]RBL236_00262_Inosine-5'-monophosphate_dehydrogenase	RbromiiL2-36
Org4_Gene25	core	2482	[RbromiiL2-36]RBL236_00025_Cytidylate_kinase	RbromiiL2-36
Org4_Gene252	core	885	[RbromiiL2-36]RBL236_00266_Serine--tRNA_ligase	RbromiiL2-36
Org4_Gene253	core	1877	[RbromiiL2-36]RBL236_00267_putative_chromosome-partitioning_protein_ParB	RbromiiL2-36
Org4_Gene254	core	2141	[RbromiiL2-36]RBL236_00268_Sporulation_initiation_inhibitor_protein_soj	RbromiiL2-36
Org4_Gene255	core	1848	[RbromiiL2-36]RBL236_00269_Nucleoid_occlusion_protein	RbromiiL2-36
Org4_Gene257	core	332	[RbromiiL2-36]RBL236_00271_Glucose-inhibited_division_protein_A	RbromiiL2-36
Org4_Gene258	core	715	[RbromiiL2-36]RBL236_00272_tRNA_modification_GTPase_MnmE	RbromiiL2-36
Org4_Gene259	core	2011	[RbromiiL2-36]RBL236_00273_R3H_domain_protein	RbromiiL2-36
Org4_Gene26	core	2586	[RbromiiL2-36]RBL236_00026_1-acyl-sn-glycerol-3-phosphate_acyltransferase	RbromiiL2-36
Org4_Gene261	core	4063	[RbromiiL2-36]RBL236_00275_Putative_membrane_protein_insertion_efficiency_factor	RbromiiL2-36
Org4_Gene262	core	3616	[RbromiiL2-36]RBL236_00276_Ribonuclease_P_protein_component	RbromiiL2-36
Org4_Gene263	core	4411	[RbromiiL2-36]RBL236_00277_50S_ribosomal_protein_L34	RbromiiL2-36
Org4_Gene264	core	697	[RbromiiL2-36]RBL236_00278_Chromosomal_replication_initiator_protein_DnaA	RbromiiL2-36
Org4_Gene265	core	1224	[RbromiiL2-36]RBL236_00279_DNA_polymerase_III_subunit_beta	RbromiiL2-36
Org4_Gene266	core	4166	[RbromiiL2-36]RBL236_00280_ribosome-associated_protein	RbromiiL2-36
Org4_Gene267	core	1180	[RbromiiL2-36]RBL236_00281_DNA_replication_and_repair_protein_RecF	RbromiiL2-36
Org4_Gene268	core	3932	[RbromiiL2-36]RBL236_00282_hypothetical_protein	RbromiiL2-36

Org4_Gene27	core	279	[RbromiiL2-36]RBL236_00027_hypothetical_protein	RbromiiL2-36
Org4_Gene270	core	256	[RbromiiL2-36]RBL236_00284_DNA_gyrase_subunit_B	RbromiiL2-36
Org4_Gene272	core	128	[RbromiiL2-36]RBL236_00286_DNA_gyrase_subunit_A	RbromiiL2-36
Org4_Gene277	core	3264	[RbromiiL2-36]RBL236_00291_Deoxyuridine_5'-triphosphate_nucleotidohydrolase	RbromiiL2-36
Org4_Gene278	core	1398	[RbromiiL2-36]RBL236_00292_Rod_shape-determining_protein_MreB	RbromiiL2-36
Org4_Gene282	core	2226	[RbromiiL2-36]RBL236_00296_Cell_division_inhibitor_MinD	RbromiiL2-36
Org4_Gene283	core	3475	[RbromiiL2-36]RBL236_00297_Methylglyoxal_synthase	RbromiiL2-36
Org4_Gene284	core	805	[RbromiiL2-36]RBL236_00298_Histidine--tRNA_ligase	RbromiiL2-36
Org4_Gene285	core	378	[RbromiiL2-36]RBL236_00299_Aspartate--tRNA_ligase	RbromiiL2-36
Org4_Gene287	core	951	[RbromiiL2-36]RBL236_00301_Putative_virion_core_protein_(lumpy_skin_disease_virus)	RbromiiL2-36
Org4_Gene29	core	1397	[RbromiiL2-36]RBL236_00029_UDP-glucose_4-epimerase	RbromiiL2-36
Org4_Gene291	core	648	[RbromiiL2-36]RBL236_00305_2-iminoacetate_synthase	RbromiiL2-36
Org4_Gene294	core	1289	[RbromiiL2-36]RBL236_00308_D-alanine--D-alanine_ligase	RbromiiL2-36
Org4_Gene295	core	758	[RbromiiL2-36]RBL236_00309_UDP-N-acetylmuramoyl-tripeptide--D-alanyl-D-alanine_ligase	RbromiiL2-36
Org4_Gene30	core	2990	[RbromiiL2-36]RBL236_00030_Peptidyl-prolyl_cis-trans_isomerase_B	RbromiiL2-36
Org4_Gene300	core	1062	[RbromiiL2-36]RBL236_00315_Tryptophan_synthase_beta_chain	RbromiiL2-36
Org4_Gene301	core	2133	[RbromiiL2-36]RBL236_00316_Tryptophan_synthase_alpha_chain	RbromiiL2-36
Org4_Gene302	core	2275	[RbromiiL2-36]RBL236_00317_NAD-dependent_protein_deacetylase	RbromiiL2-36
Org4_Gene303	core	3981	[RbromiiL2-36]RBL236_00318_30S_ribosomal_protein_S20	RbromiiL2-36
Org4_Gene304	core	4378	[RbromiiL2-36]RBL236_00319_hypothetical_protein	RbromiiL2-36
Org4_Gene307	core	525	[RbromiiL2-36]RBL236_00322_Alkaline_phosphatase_synthesis_sensor_protein_PhoR	RbromiiL2-36
Org4_Gene308	core	2424	[RbromiiL2-36]RBL236_00323_Staphylococcal_respiratory_response_protein_A	RbromiiL2-36
Org4_Gene309	core	1863	[RbromiiL2-36]RBL236_00324_L(+)-tartrate_dehydratase_subunit_alpha	RbromiiL2-36
Org4_Gene31	core	1404	[RbromiiL2-36]RBL236_00031_t(6)A37_threonylcarbamoyladenosine_biosynthesis_protein	RbromiiL2-36
Org4_Gene310	core	2878	[RbromiiL2-36]RBL236_00325_Fumarate_hydratase_class_I,_aerobic	RbromiiL2-36
Org4_Gene315	core	2521	[RbromiiL2-36]RBL236_00330_LexA_repressor	RbromiiL2-36
Org4_Gene320	core	344	[RbromiiL2-36]RBL236_00335_Heat_shock_protein_70	RbromiiL2-36
Org4_Gene321	core	1153	[RbromiiL2-36]RBL236_00336_Chaperone_protein_DnaJ	RbromiiL2-36
Org4_Gene322	core	1591	[RbromiiL2-36]RBL236_00337_Ribosomal_protein_L11_methyltransferase	RbromiiL2-36
Org4_Gene324	core	1958	[RbromiiL2-36]RBL236_00339_antiporter_inner_membrane_protein	RbromiiL2-36
Org4_Gene331	core	1605	[RbromiiL2-36]RBL236_00346_Ornithine_carbamoyltransferase	RbromiiL2-36
Org4_Gene332	core	1088	[RbromiiL2-36]RBL236_00347_Acetylornithine_aminotransferase	RbromiiL2-36
Org4_Gene333	core	3359	[RbromiiL2-36]RBL236_00348_Acetyltransferase_YpeA	RbromiiL2-36
Org4_Gene334	core	1865	[RbromiiL2-36]RBL236_00349_Acetylglutamate_kinase	RbromiiL2-36
Org4_Gene335	core	973	[RbromiiL2-36]RBL236_00350_Arginine_biosynthesis_bifunctional_protein_ArgJ	RbromiiL2-36
Org4_Gene336	core	1349	[RbromiiL2-36]RBL236_00351_N-acetyl-gamma-glutamyl-phosphate_reductase	RbromiiL2-36
Org4_Gene337	core	719	[RbromiiL2-36]RBL236_00352_Argininosuccinate_lyase_1	RbromiiL2-36
Org4_Gene338	core	983	[RbromiiL2-36]RBL236_00353_Argininosuccinate_synthase	RbromiiL2-36
Org4_Gene34	core	129	[RbromiiL2-36]RBL236_00034_DNA_polymerase_I	RbromiiL2-36
Org4_Gene340	core	3024	[RbromiiL2-36]RBL236_00355_Flavoredoxin	RbromiiL2-36
Org4_Gene348	core	3541	[RbromiiL2-36]RBL236_00363_Ribosome-binding_factor_A	RbromiiL2-36
Org4_Gene349	core	165	[RbromiiL2-36]RBL236_00364_Translation_initiation_factor_IF-2	RbromiiL2-36
Org4_Gene35	core	1074	[RbromiiL2-36]RBL236_00035_Alanine_racemase	RbromiiL2-36
Org4_Gene352	core	1117	[RbromiiL2-36]RBL236_00367_hypothetical_protein	RbromiiL2-36
Org4_Gene353	core	2658	[RbromiiL2-36]RBL236_00368_Ribosome_maturation_factor_RimP	RbromiiL2-36
Org4_Gene356	core	4243	[RbromiiL2-36]RBL236_00372_anaerobic_benzoate_catabolism_transcriptional_regulator	RbromiiL2-36
Org4_Gene357	core	2401	[RbromiiL2-36]RBL236_00373_hypothetical_protein	RbromiiL2-36
Org4_Gene358	core	1419	[RbromiiL2-36]RBL236_00374_hypothetical_protein	RbromiiL2-36
Org4_Gene359	core	2387	[RbromiiL2-36]RBL236_00375_hypothetical_protein	RbromiiL2-36
Org4_Gene361	core	90	[RbromiiL2-36]RBL236_00377_2-hydroxyglutaryl-CoA_dehydratase_component_A	RbromiiL2-36
Org4_Gene362	core	811	[RbromiiL2-36]RBL236_00378_2-hydroxyglutaryl-CoA_dehydratase,_D-component	RbromiiL2-36
Org4_Gene365	core	1812	[RbromiiL2-36]RBL236_00381_Fructose-bisphosphate_aldolase	RbromiiL2-36
Org4_Gene368	core	444	[RbromiiL2-36]RBL236_00384_Cell_division_protein_FtsW	RbromiiL2-36
Org4_Gene369	core	695	[RbromiiL2-36]RBL236_00385_Penicillin-binding_protein_A	RbromiiL2-36
Org4_Gene370	core	95	[RbromiiL2-36]RBL236_00386_preprotein_translocase_subunit_SecA	RbromiiL2-36
Org4_Gene377	core	727	[RbromiiL2-36]RBL236_00393_Magnesium_transporter_MgtE	RbromiiL2-36
Org4_Gene378	core	1832	[RbromiiL2-36]RBL236_00394_5,10-methylenetetrahydrofolate_reductase	RbromiiL2-36
Org4_Gene380	core	154	[RbromiiL2-36]RBL236_00396_Methionine_synthase	RbromiiL2-36
Org4_Gene382	core	50	[RbromiiL2-36]RBL236_00398_Pyruvate-flavodoxin_oxidoreductase	RbromiiL2-36
Org4_Gene384	core	2815	[RbromiiL2-36]RBL236_00400_hypothetical_protein	RbromiiL2-36
Org4_Gene386	core	199	[RbromiiL2-36]RBL236_00402_Formate_acetyltransferase	RbromiiL2-36
Org4_Gene403	core	2793	[RbromiiL2-36]RBL236_00419_dTDP-4-dehydrorhamnose_3,5-epimerase	RbromiiL2-36
Org4_Gene404	core	1601	[RbromiiL2-36]RBL236_00420_dTDP-glucose_4,6-dehydratase	RbromiiL2-36
Org4_Gene405	core	1311	[RbromiiL2-36]RBL236_00421_CDP-glucose_4,6-dehydratase	RbromiiL2-36
Org4_Gene406	core	2109	[RbromiiL2-36]RBL236_00422_Glucose-1-phosphate_cytidylyltransferase	RbromiiL2-36
Org4_Gene413	core	2111	[RbromiiL2-36]RBL236_00429_putative_deoxyribonuclease_YcfH	RbromiiL2-36
Org4_Gene414	core	297	[RbromiiL2-36]RBL236_00430_Methionine--tRNA_ligase	RbromiiL2-36
Org4_Gene421	core	1439	[RbromiiL2-36]RBL236_00437_putative_tRNA-dihydrouridine_synthase	RbromiiL2-36
Org4_Gene422	core	931	[RbromiiL2-36]RBL236_00438_Serine_hydroxymethyltransferase	RbromiiL2-36
Org4_Gene425	core	2876	[RbromiiL2-36]RBL236_00441_undecaprenyl_pyrophosphate_phosphatase	RbromiiL2-36
Org4_Gene426	core	1402	[RbromiiL2-36]RBL236_00442_Stage_V_sporulation_protein_AD	RbromiiL2-36
Org4_Gene430	core	626	[RbromiiL2-36]RBL236_00446_Glutamate--tRNA_ligase	RbromiiL2-36
Org4_Gene431	core	434	[RbromiiL2-36]RBL236_00447_Glutamine--tRNA_ligase	RbromiiL2-36
Org4_Gene432	core	2221	[RbromiiL2-36]RBL236_00448_3-oxoacyl-[acyl-carrier-protein]_reductase_FabG	RbromiiL2-36
Org4_Gene433	core	607	[RbromiiL2-36]RBL236_00449_UDP-N-acetylmuramoyl-L-alanyl-D-glutamate--L-lysine_ligase	RbromiiL2-36
Org4_Gene435	core	581	[RbromiiL2-36]RBL236_00451_Maltose_transport_system_permease_protein_MalF	RbromiiL2-36
Org4_Gene436	core	1824	[RbromiiL2-36]RBL236_00452_Maltose_transport_system_permease_protein_MalG	RbromiiL2-36
Org4_Gene437	core	460	[RbromiiL2-36]RBL236_00453_Alpha-amylase_precursor	RbromiiL2-36
Org4_Gene438	core	3378	[RbromiiL2-36]RBL236_00454_30S_ribosomal_protein_S12	RbromiiL2-36
Org4_Gene439	core	2922	[RbromiiL2-36]RBL236_00455_30S_ribosomal_protein_S7	RbromiiL2-36
Org4_Gene440	core	240	[RbromiiL2-36]RBL236_00456_Vegetative_protein_19	RbromiiL2-36
Org4_Gene441	core	1023	[RbromiiL2-36]RBL236_00457_Elongation_factor_Tu	RbromiiL2-36
Org4_Gene442	core	3243	[RbromiiL2-36]RBL236_00458_stage_V_sporulation_protein_AC	RbromiiL2-36
Org4_Gene443	core	1182	[RbromiiL2-36]RBL236_00459_Cysteine_desulfurase	RbromiiL2-36
Org4_Gene444	core	1065	[RbromiiL2-36]RBL236_00460_putative_tRNA_sulfurtransferase	RbromiiL2-36
Org4_Gene453	core	2252	[RbromiiL2-36]RBL236_00477_Putative_HAD-hydrolase_YfnB	RbromiiL2-36
Org4_Gene454	core	2513	[RbromiiL2-36]RBL236_00478_UV-endonuclease	RbromiiL2-36
Org4_Gene457	core	363	[RbromiiL2-36]RBL236_00481_Elongation_factor_4	RbromiiL2-36
Org4_Gene459	core	164	[RbromiiL2-36]RBL236_00483_Asparagine_synthetase_[glutamine-hydrolyzing]_1	RbromiiL2-36
Org4_Gene461	core	2888	[RbromiiL2-36]RBL236_00485_ADP-ribose_pyrophosphatase	RbromiiL2-36
Org4_Gene48	core	4278	[RbromiiL2-36]RBL236_00049_50S_ribosomal_protein_L28	RbromiiL2-36
Org4_Gene487	core	4333	[RbromiiL2-36]RBL236_00511_hypothetical_protein	RbromiiL2-36
Org4_Gene499	core	1875	[RbromiiL2-36]RBL236_00523_Sugar_phosphatase_YidA	RbromiiL2-36
Org4_Gene50	core	2124	[RbromiiL2-36]RBL236_00051_Segregation_and_condensation_protein_A	RbromiiL2-36
Org4_Gene501	core	714	[RbromiiL2-36]RBL236_00525_2-methylcitrate_synthase	RbromiiL2-36
Org4_Gene508	core	1017	[RbromiiL2-36]RBL236_00533_UDP-4-amino-4-deoxy-L-arabinose--oxoglutarate_aminotransferase	RbromiiL2-36
Org4_Gene509	core	3922	[RbromiiL2-36]RBL236_00534_14_kDa_transcription_factor	RbromiiL2-36
Org4_Gene51	core	2631	[RbromiiL2-36]RBL236_00052_Segregation_and_condensation_protein_B	RbromiiL2-36
Org4_Gene515	core	1834	[RbromiiL2-36]RBL236_00541_Nicotinamide_nucleotide_repair_protein	RbromiiL2-36
Org4_Gene518	core	336	[RbromiiL2-36]RBL236_00544_UDP-N-acetyl-alpha-D-glucosamine_C6_dehydratase	RbromiiL2-36
Org4_Gene519	core	356	[RbromiiL2-36]RBL236_00545_Glutamine--fructose-6-phosphate_aminotransferase_[isomerizing]	RbromiiL2-36
Org4_Gene53	core	3344	[RbromiiL2-36]RBL236_00054_putative_spore_protein_YtfJ	RbromiiL2-36
Org4_Gene533	core	1145	[RbromiiL2-36]RBL236_00567_4-aminobutyrate_aminotransferase	RbromiiL2-36
Org4_Gene541	core	1902	[RbromiiL2-36]RBL236_00575_putative_nicotinate-nucleotide_pyrophosphorylase_[carboxylating]	RbromiiL2-36
Org4_Gene542	core	611	[RbromiiL2-36]RBL236_00576_L-aspartate_oxidase	RbromiiL2-36
Org4_Gene55	core	2196	[RbromiiL2-36]RBL236_00056_Ribosomal_large_subunit_pseudouridine_synthase_B	RbromiiL2-36
Org4_Gene552	core	1214	[RbromiiL2-36]RBL236_00586_Cell_division_protein_FtsZ	RbromiiL2-36
Org4_Gene554	core	933	[RbromiiL2-36]RBL236_00588_UDP-N-acetylglucosamine_1-carboxyvinyltransferase_1	RbromiiL2-36
Org4_Gene555	core	1196	[RbromiiL2-36]RBL236_00589_UDP-N-acetylglucosamine--N-acetylmuramyl-(pentapeptide)_pyrophosphoryl-undecaprenol_N-acetylglucosamine_transfera	RbromiiL2-36
Org4_Gene556	core	928	[RbromiiL2-36]RBL236_00590_Cell_division_protein_FtsW	RbromiiL2-36
Org4_Gene557	core	1356	[RbromiiL2-36]RBL236_00591_Phospho-N-acetylmuramoyl-pentapeptide-transferase	RbromiiL2-36
Org4_Gene558	core	194	[RbromiiL2-36]RBL236_00592_Sporulation-specific_penicillin-binding_protein	RbromiiL2-36
Org4_Gene56	core	839	[RbromiiL2-36]RBL236_00057_Methylmalonyl-CoA_carboxyltransferase_12S_subunit	RbromiiL2-36
Org4_Gene560	core	1626	[RbromiiL2-36]RBL236_00594_Ribosomal_RNA_small_subunit_methyltransferase_H	RbromiiL2-36
Org4_Gene563	core	3206	[RbromiiL2-36]RBL236_00597_hypothetical_protein	RbromiiL2-36
Org4_Gene565	core	2851	[RbromiiL2-36]RBL236_00599_Ribosomal_RNA_small_subunit_methyltransferase_D	RbromiiL2-36
Org4_Gene566	core	347	[RbromiiL2-36]RBL236_00600_Excinuclease_ABC_subunit_C	RbromiiL2-36
Org4_Gene567	core	3960	[RbromiiL2-36]RBL236_00601_Catabolite_repression_HPr	RbromiiL2-36
Org4_Gene568	core	2404	[RbromiiL2-36]RBL236_00602_tRNA_(guanine-N(1)-)-methyltransferase	RbromiiL2-36
Org4_Gene569	core	3006	[RbromiiL2-36]RBL236_00603_Ribosome_maturation_factor_RimM	RbromiiL2-36
Org4_Gene57	core	3572	[RbromiiL2-36]RBL236_00058_Glutaconyl-CoA_decarboxylase_subunit_gamma	RbromiiL2-36
Org4_Gene570	core	135	[RbromiiL2-36]RBL236_00604_MutS2_protein	RbromiiL2-36

Org4_Gene571	core	2204	[RbromiiL2-36]RBL236_00605_Recombination_protein_O	RbromiiL2-36
Org4_Gene572	core	4338	[RbromiiL2-36]RBL236_00606_hypothetical_protein	RbromiiL2-36
Org4_Gene573	core	1643	[RbromiiL2-36]RBL236_00607_Bex_protein	RbromiiL2-36
Org4_Gene575	core	3007	[RbromiiL2-36]RBL236_00609_Endoribonuclease_YbeY	RbromiiL2-36
Org4_Gene576	core	1321	[RbromiiL2-36]RBL236_00610_PhoH-like_protein	RbromiiL2-36
Org4_Gene577	core	3631	[RbromiiL2-36]RBL236_00611_putative_DNA-binding_protein	RbromiiL2-36
Org4_Gene578	core	676	[RbromiiL2-36]RBL236_00612_hypothetical_protein	RbromiiL2-36
Org4_Gene579	core	4034	[RbromiiL2-36]RBL236_00613_hypothetical_protein	RbromiiL2-36
Org4_Gene58	core	687	[RbromiiL2-36]RBL236_00059_Methylmalonyl-CoA_carboxyltransferase_5S_subunit	RbromiiL2-36
Org4_Gene580	core	4080	[RbromiiL2-36]RBL236_00614_putative_RNA-binding_protein_(contains_KH_domain)	RbromiiL2-36
Org4_Gene582	core	141	[RbromiiL2-36]RBL236_00616_Negative_regulator_of_genetic_competence_ClpC/MecB	RbromiiL2-36
Org4_Gene583	core	1444	[RbromiiL2-36]RBL236_00617_Putative_ATP:guanido_phosphotransferase	RbromiiL2-36
Org4_Gene584	core	3043	[RbromiiL2-36]RBL236_00618_hypothetical_protein	RbromiiL2-36
Org4_Gene585	core	3251	[RbromiiL2-36]RBL236_00619_Class_three_stress_gene_repressor	RbromiiL2-36
Org4_Gene595	core	3905	[RbromiiL2-36]RBL236_00629_CxxC-x17-CxxC_domain_protein	RbromiiL2-36
Org4_Gene598	core	4248	[RbromiiL2-36]RBL236_00632_hybrid_cluster_protein-associated_redox_disulfide_domain_protein	RbromiiL2-36
Org4_Gene601	core	2484	[RbromiiL2-36]RBL236_00635_tRNA_(guanine-N(7)-)-methyltransferase	RbromiiL2-36
Org4_Gene603	core	2345	[RbromiiL2-36]RBL236_00637_Sensory_transduction_protein_regX3	RbromiiL2-36
Org4_Gene604	core	605	[RbromiiL2-36]RBL236_00638_Alkaline_phosphatase_synthesis_sensor_protein_PhoR	RbromiiL2-36
Org4_Gene605	core	1443	[RbromiiL2-36]RBL236_00639_putative_metal-dependent_enzyme	RbromiiL2-36
Org4_Gene607	core	1195	[RbromiiL2-36]RBL236_00641_Recombinase_A	RbromiiL2-36
Org4_Gene608	core	2648	[RbromiiL2-36]RBL236_00642_Regulatory_protein_RecX	RbromiiL2-36
Org4_Gene609	core	778	[RbromiiL2-36]RBL236_00643_Ribosomal_protein_S12_methylthiotransferase_RimO	RbromiiL2-36
Org4_Gene610	core	2725	[RbromiiL2-36]RBL236_00644_CDP-diacylglycerol--glycerol-3-phosphate_3-phosphatidyltransferase	RbromiiL2-36
Org4_Gene611	core	2491	[RbromiiL2-36]RBL236_00645_Serine_acetyltransferase	RbromiiL2-36
Org4_Gene612	core	685	[RbromiiL2-36]RBL236_00646_Cysteine--tRNA_ligase	RbromiiL2-36
Org4_Gene613	core	2056	[RbromiiL2-36]RBL236_00647_Bifunctional_xylanase/deacetylase_precursor	RbromiiL2-36
Org4_Gene620	core	3239	[RbromiiL2-36]RBL236_00654_S-ribosylhomocysteine_lyase	RbromiiL2-36
Org4_Gene627	core	3322	[RbromiiL2-36]RBL236_00661_Cupin_domain_protein	RbromiiL2-36
Org4_Gene629	core	3228	[RbromiiL2-36]RBL236_00663_Transcriptional_repressor_NrdR	RbromiiL2-36
Org4_Gene631	core	265	[RbromiiL2-36]RBL236_00665_ATP-dependent_DNA_helicase_RecG	RbromiiL2-36
Org4_Gene632	core	470	[RbromiiL2-36]RBL236_00666_dihydroxyacetone_kinase	RbromiiL2-36
Org4_Gene633	core	2126	[RbromiiL2-36]RBL236_00667_metallophosphoesterase,_MG_246/_family	RbromiiL2-36
Org4_Gene637	core	354	[RbromiiL2-36]RBL236_00671_Asparagine_synthetase_[glutamine-hydrolyzing]_3	RbromiiL2-36
Org4_Gene641	core	3312	[RbromiiL2-36]RBL236_00675_Large-conductance_mechanosensitive_channel	RbromiiL2-36
Org4_Gene642	core	979	[RbromiiL2-36]RBL236_00676_Tyrosine--tRNA_ligase	RbromiiL2-36
Org4_Gene644	core	316	[RbromiiL2-36]RBL236_00678_putative_radical_SAM_protein_YgiQ	RbromiiL2-36
Org4_Gene646	core	1521	[RbromiiL2-36]RBL236_00680_Undecaprenyl-diphosphatase	RbromiiL2-36
Org4_Gene647	core	1989	[RbromiiL2-36]RBL236_00681_Translocation-enhancing_protein_TepA	RbromiiL2-36
Org4_Gene648	core	830	[RbromiiL2-36]RBL236_00682_Magnesium_and_cobalt_efflux_protein_CorC	RbromiiL2-36
Org4_Gene650	core	2718	[RbromiiL2-36]RBL236_00684_Ribonuclease_M5	RbromiiL2-36
Org4_Gene654	core	2935	[RbromiiL2-36]RBL236_00688_Pur_operon_repressor	RbromiiL2-36
Org4_Gene655	core	3658	[RbromiiL2-36]RBL236_00689_HIT-like_protein	RbromiiL2-36
Org4_Gene657	core	819	[RbromiiL2-36]RBL236_00691_GTP-binding_protein_EngA	RbromiiL2-36
Org4_Gene659	core	4271	[RbromiiL2-36]RBL236_00693_50S_ribosomal_protein_L32	RbromiiL2-36
Org4_Gene660	core	3074	[RbromiiL2-36]RBL236_00694_hypothetical_protein	RbromiiL2-36
Org4_Gene667	core	1618	[RbromiiL2-36]RBL236_00701_30S_ribosomal_protein_S1	RbromiiL2-36
Org4_Gene669	core	1871	[RbromiiL2-36]RBL236_00703_HAD_phosphoserine_phosphatase-like_hydrolase,_family_IB	RbromiiL2-36
Org4_Gene671	core	2611	[RbromiiL2-36]RBL236_00705_putative_manganese_catalase	RbromiiL2-36
Org4_Gene674	core	2544	[RbromiiL2-36]RBL236_00708_L-cystine_transport_system_permease_protein_TcyB	RbromiiL2-36
Org4_Gene675	core	2029	[RbromiiL2-36]RBL236_00709_L-cystine_import_ATP-binding_protein_TcyC	RbromiiL2-36
Org4_Gene689	core	1898	[RbromiiL2-36]RBL236_00724_Formate_channel_1	RbromiiL2-36
Org4_Gene690	core	224	[RbromiiL2-36]RBL236_00725_30S_ribosomal_protein_S1	RbromiiL2-36
Org4_Gene693	core	3540	[RbromiiL2-36]RBL236_00728_Manganese_transport_regulator	RbromiiL2-36
Org4_Gene694	core	4164	[RbromiiL2-36]RBL236_00729_FeoA_domain_protein	RbromiiL2-36
Org4_Gene695	core	4121	[RbromiiL2-36]RBL236_00730_Ferrous_iron_transport_protein_A	RbromiiL2-36
Org4_Gene696	core	134	[RbromiiL2-36]RBL236_00731_Ferrous_iron_transport_protein_B	RbromiiL2-36
Org4_Gene703	core	3361	[RbromiiL2-36]RBL236_00738_Flavodoxin	RbromiiL2-36
Org4_Gene704	core	1043	[RbromiiL2-36]RBL236_00739_L-threonine_dehydratase_catabolic_TdcB	RbromiiL2-36
Org4_Gene705	core	3555	[RbromiiL2-36]RBL236_00740_Enamine/imine_deaminase	RbromiiL2-36
Org4_Gene706	core	1067	[RbromiiL2-36]RBL236_00741_S-adenosylmethionine_synthase	RbromiiL2-36
Org4_Gene722	core	2395	[RbromiiL2-36]RBL236_00759_RNA_polymerase_sigma-28_factor_precursor	RbromiiL2-36
Org4_Gene725	core	852	[RbromiiL2-36]RBL236_00762_Methionine_gamma-lyase	RbromiiL2-36
Org4_Gene728	core	3849	[RbromiiL2-36]RBL236_00765_30S_ribosomal_protein_S6	RbromiiL2-36
Org4_Gene729	core	3056	[RbromiiL2-36]RBL236_00766_Helix-destabilizing_protein	RbromiiL2-36
Org4_Gene730	core	4065	[RbromiiL2-36]RBL236_00767_hypothetical_protein	RbromiiL2-36
Org4_Gene733	core	3823	[RbromiiL2-36]RBL236_00770_Copper-sensitive_operon_repressor	RbromiiL2-36
Org4_Gene734	core	137	[RbromiiL2-36]RBL236_00771_putative_copper-importing_P-type_ATPase_A	RbromiiL2-36
Org4_Gene736	core	3817	[RbromiiL2-36]RBL236_00773_hypothetical_protein	RbromiiL2-36
Org4_Gene737	core	4032	[RbromiiL2-36]RBL236_00774_hypothetical_protein	RbromiiL2-36
Org4_Gene738	core	169	[RbromiiL2-36]RBL236_00775_Phenylalanine--tRNA_ligase_beta_subunit	RbromiiL2-36
Org4_Gene739	core	1316	[RbromiiL2-36]RBL236_00776_Phenylalanine--tRNA_ligase_alpha_subunit	RbromiiL2-36
Org4_Gene748	core	3646	[RbromiiL2-36]RBL236_00786_mRNA_interferase_MazF	RbromiiL2-36
Org4_Gene750	core	2610	[RbromiiL2-36]RBL236_00788_Alpha-ribazole_phosphatase	RbromiiL2-36
Org4_Gene751	core	1285	[RbromiiL2-36]RBL236_00789_3-isopropylmalate_dehydrogenase	RbromiiL2-36
Org4_Gene753	core	3330	[RbromiiL2-36]RBL236_00791_NifU-like_protein	RbromiiL2-36
Org4_Gene754	core	1027	[RbromiiL2-36]RBL236_00792_Cysteine_desulfurase	RbromiiL2-36
Org4_Gene755	core	3091	[RbromiiL2-36]RBL236_00793_Ribonuclease_HI	RbromiiL2-36
Org4_Gene756	core	320	[RbromiiL2-36]RBL236_00794_Ribonuclease_J_1	RbromiiL2-36
Org4_Gene758	core	3258	[RbromiiL2-36]RBL236_00796_hypothetical_protein	RbromiiL2-36
Org4_Gene759	core	725	[RbromiiL2-36]RBL236_00797_Replicative_DNA_helicase	RbromiiL2-36
Org4_Gene761	core	2937	[RbromiiL2-36]RBL236_00799_Hypoxanthine-guanine_phosphoribosyltransferase	RbromiiL2-36
Org4_Gene762	core	270	[RbromiiL2-36]RBL236_00800_ATP-dependent_zinc_metalloprotease_FtsH	RbromiiL2-36
Org4_Gene763	core	99	[RbromiiL2-36]RBL236_00801_Excinuclease_ABC_subunit_A	RbromiiL2-36
Org4_Gene764	core	3992	[RbromiiL2-36]RBL236_00802_hypothetical_protein	RbromiiL2-36
Org4_Gene772	core	298	[RbromiiL2-36]RBL236_00811_Threonine--tRNA_ligase_1	RbromiiL2-36
Org4_Gene774	core	4201	[RbromiiL2-36]RBL236_00813_50S_ribosomal_protein_L31	RbromiiL2-36
Org4_Gene775	core	2603	[RbromiiL2-36]RBL236_00814_IMPACT_family_member_YigZ	RbromiiL2-36
Org4_Gene776	core	383	[RbromiiL2-36]RBL236_00815_DNA_primase	RbromiiL2-36
Org4_Gene777	core	1216	[RbromiiL2-36]RBL236_00816_RNA_polymerase_sigma_factor_RpoD	RbromiiL2-36
Org4_Gene778	core	3557	[RbromiiL2-36]RBL236_00817_hypothetical_protein	RbromiiL2-36
Org4_Gene781	core	92	[RbromiiL2-36]RBL236_00820_Pullulanase_precursor	RbromiiL2-36
Org4_Gene783	core	2589	[RbromiiL2-36]RBL236_00822_Redox-sensing_transcriptional_repressor_rex	RbromiiL2-36
Org4_Gene784	core	1011	[RbromiiL2-36]RBL236_00823_Isocitrate_dehydrogenase_[NADP]	RbromiiL2-36
Org4_Gene785	core	307	[RbromiiL2-36]RBL236_00824_2,3-dimethylmalate_dehydratase_large_subunit	RbromiiL2-36
Org4_Gene786	core	359	[RbromiiL2-36]RBL236_00825_Tyrosine_phosphorylated_protein_A	RbromiiL2-36
Org4_Gene793	core	710	[RbromiiL2-36]RBL236_00832_UDP-N-acetylmuramate--L-alanine_ligase	RbromiiL2-36
Org4_Gene794	core	729	[RbromiiL2-36]RBL236_00833_putative_RNA_methyltransferase	RbromiiL2-36
Org4_Gene797	core	2145	[RbromiiL2-36]RBL236_00836_putative_oxidoreductase	RbromiiL2-36
Org4_Gene801	core	398	[RbromiiL2-36]RBL236_00840_putative_ABC_transporter_ATP-binding_protein	RbromiiL2-36
Org4_Gene802	core	323	[RbromiiL2-36]RBL236_00841_putative_ABC_transporter_ATP-binding_protein	RbromiiL2-36
Org4_Gene819	core	1369	[RbromiiL2-36]RBL236_00859_Branched-chain-amino-acid_aminotransferase	RbromiiL2-36
Org4_Gene822	core	3740	[RbromiiL2-36]RBL236_00866_4-carboxymuconolactone_decarboxylase	RbromiiL2-36
Org4_Gene824	core	2499	[RbromiiL2-36]RBL236_00868_V-type_sodium_pump_subunit_D	RbromiiL2-36
Org4_Gene825	core	716	[RbromiiL2-36]RBL236_00869_V-type_sodium_pump_subunit_B	RbromiiL2-36
Org4_Gene826	core	393	[RbromiiL2-36]RBL236_00870_V-type_ATP_synthase_alpha_chain	RbromiiL2-36
Org4_Gene828	core	3802	[RbromiiL2-36]RBL236_00872_V-type_ATP_synthase_subunit_F	RbromiiL2-36
Org4_Gene829	core	3337	[RbromiiL2-36]RBL236_00873_V-type_ATP_synthase_subunit_K	RbromiiL2-36
Org4_Gene831	core	1355	[RbromiiL2-36]RBL236_00875_V-type_ATP_synthase_subunit_C	RbromiiL2-36
Org4_Gene833	core	744	[RbromiiL2-36]RBL236_00877_hypothetical_protein	RbromiiL2-36
Org4_Gene834	core	3947	[RbromiiL2-36]RBL236_00878_formyltetrahydrofolate_deformylase	RbromiiL2-36
Org4_Gene836	core	1775	[RbromiiL2-36]RBL236_00880_UTP--glucose-1-phosphate_uridylyltransferase	RbromiiL2-36
Org4_Gene838	core	1201	[RbromiiL2-36]RBL236_00882_Peptide_chain_release_factor_2	RbromiiL2-36
Org4_Gene839	core	1002	[RbromiiL2-36]RBL236_00883_cofactor-independent_phosphoglycerate_mutase	RbromiiL2-36
Org4_Gene845	core	191	[RbromiiL2-36]RBL236_00891_ATP-dependent_DNA_helicase_PcrA	RbromiiL2-36
Org4_Gene850	core	702	[RbromiiL2-36]RBL236_00896_UDP-N-acetylmuramoylalanine--D-glutamate_ligase	RbromiiL2-36
Org4_Gene854	core	902	[RbromiiL2-36]RBL236_00900_cystathionine_gamma-synthase	RbromiiL2-36
Org4_Gene855	core	3230	[RbromiiL2-36]RBL236_00901_tRNA-specific_adenosine_deaminase	RbromiiL2-36
Org4_Gene9	core	892	[RbromiiL2-36]RBL236_00009_Putative_zinc_protease_AlbF	RbromiiL2-36
Org4_Gene916	core	641	[RbromiiL2-36]RBL236_00964_Nicotinate_phosphoribosyltransferase_pncB2	RbromiiL2-36
Org4_Gene919	core	2018	[RbromiiL2-36]RBL236_00967_Thymidylate_synthase_ThyX	RbromiiL2-36
Org4_Gene923	core	2911	[RbromiiL2-36]RBL236_00971_Chaperone_protein_YajL	RbromiiL2-36

Org4_Gene926	core	971	[RbromiiL2-36]RBL236_00974_putative_protease_YhbU_precursor	RbromiiL2-36
Org4_Gene927	core	118	[RbromiiL2-36]RBL236_00976_Alanine--tRNA_ligase	RbromiiL2-36
Org4_Gene933	core	2506	[RbromiiL2-36]RBL236_00983_Phosphorylated_carbohydrates_phosphatase	RbromiiL2-36
Org4_Gene936	core	371	[RbromiiL2-36]RBL236_00986_Dihydroxy-acid_dehydratase	RbromiiL2-36
Org4_Gene937	core	258	[RbromiiL2-36]RBL236_00987_Elongation_factor_G	RbromiiL2-36
Org4_Gene940	core	396	[RbromiiL2-36]RBL236_00990_putative_ABC_transporter_ATP-binding_protein	RbromiiL2-36
Org4_Gene941	core	405	[RbromiiL2-36]RBL236_00991_putative_ABC_transporter_ATP-binding_protein	RbromiiL2-36
Org4_Gene942	core	570	[RbromiiL2-36]RBL236_00992_2,3-bisphosphoglycerate-independent_phosphoglycerate_mutase	RbromiiL2-36
Org4_Gene943	core	2143	[RbromiiL2-36]RBL236_00993_Triosephosphate_isomerase	RbromiiL2-36
Org4_Gene944	core	1009	[RbromiiL2-36]RBL236_00994_Phosphoglycerate_kinase	RbromiiL2-36
Org4_Gene945	core	2998	[RbromiiL2-36]RBL236_00995_tRNA_(cytidine(34)-2'-O)-methyltransferase	RbromiiL2-36
Org4_Gene956	core	701	[RbromiiL2-36]RBL236_01006_D-alanyl-lipoteichoic_acid_biosynthesis_protein_DltB	RbromiiL2-36
Org4_Gene959	core	3796	[RbromiiL2-36]RBL236_01009_hypothetical_protein	RbromiiL2-36
Org4_Gene960	core	2594	[RbromiiL2-36]RBL236_01010_50S_ribosomal_protein_L3	RbromiiL2-36
Org4_Gene961	core	2622	[RbromiiL2-36]RBL236_01011_50S_ribosomal_protein_L4	RbromiiL2-36
Org4_Gene962	core	3858	[RbromiiL2-36]RBL236_01012_50S_ribosomal_protein_L23	RbromiiL2-36
Org4_Gene963	core	1925	[RbromiiL2-36]RBL236_01013_hypothetical_protein	RbromiiL2-36
Org4_Gene964	core	3940	[RbromiiL2-36]RBL236_01014_hypothetical_protein	RbromiiL2-36
Org4_Gene965	core	3667	[RbromiiL2-36]RBL236_01015_50S_ribosomal_protein_L22	RbromiiL2-36
Org4_Gene966	core	2426	[RbromiiL2-36]RBL236_01016_hypothetical_protein	RbromiiL2-36
Org4_Gene967	core	3375	[RbromiiL2-36]RBL236_01017_50S_ribosomal_protein_L16	RbromiiL2-36
Org4_Gene968	core	4200	[RbromiiL2-36]RBL236_01018_50S_ribosomal_protein_L29	RbromiiL2-36
Org4_Gene969	core	3990	[RbromiiL2-36]RBL236_01019_30S_ribosomal_protein_S17	RbromiiL2-36
Org4_Gene970	core	3565	[RbromiiL2-36]RBL236_01020_50S_ribosomal_protein_L14	RbromiiL2-36
Org4_Gene971	core	3773	[RbromiiL2-36]RBL236_01021_50S_ribosomal_protein_L24	RbromiiL2-36
Org4_Gene972	core	2914	[RbromiiL2-36]RBL236_01022_50S_ribosomal_protein_L5	RbromiiL2-36
Org4_Gene973	core	4289	[RbromiiL2-36]RBL236_01023_hypothetical_protein	RbromiiL2-36
Org4_Gene974	core	3469	[RbromiiL2-36]RBL236_01024_30S_ribosomal_protein_S8	RbromiiL2-36
Org4_Gene975	core	2900	[RbromiiL2-36]RBL236_01025_hypothetical_protein	RbromiiL2-36
Org4_Gene976	core	3597	[RbromiiL2-36]RBL236_01026_hypothetical_protein	RbromiiL2-36
Org4_Gene977	core	3067	[RbromiiL2-36]RBL236_01027_30S_ribosomal_protein_S5	RbromiiL2-36
Org4_Gene978	core	4346	[RbromiiL2-36]RBL236_01028_50S_ribosomal_protein_L30	RbromiiL2-36
Org4_Gene979	core	3283	[RbromiiL2-36]RBL236_01029_50S_ribosomal_protein_L15	RbromiiL2-36
Org4_Gene980	core	782	[RbromiiL2-36]RBL236_01030_preprotein_translocase_subunit_SecY	RbromiiL2-36
Org4_Gene981	core	2593	[RbromiiL2-36]RBL236_01031_Adenylate_kinase	RbromiiL2-36
Org4_Gene982	core	2186	[RbromiiL2-36]RBL236_01032_Methionine_aminopeptidase_1	RbromiiL2-36
Org4_Gene983	core	4037	[RbromiiL2-36]RBL236_01033_hypothetical_protein	RbromiiL2-36
Org4_Gene984	core	4142	[RbromiiL2-36]RBL236_01034_Translation_initiation_factor_IF-1	RbromiiL2-36
Org4_Gene986	core	3568	[RbromiiL2-36]RBL236_01036_30S_ribosomal_protein_S13	RbromiiL2-36
Org4_Gene987	core	3445	[RbromiiL2-36]RBL236_01037_30S_ribosomal_protein_S11	RbromiiL2-36
Org4_Gene988	core	2686	[RbromiiL2-36]RBL236_01038_30S_ribosomal_protein_S4	RbromiiL2-36
Org4_Gene989	core	1541	[RbromiiL2-36]RBL236_01039_DNA-directed_RNA_polymerase_subunit_alpha	RbromiiL2-36
Org4_Gene990	core	3661	[RbromiiL2-36]RBL236_01040_hypothetical_protein	RbromiiL2-36
Org4_Gene993	core	639	[RbromiiL2-36]RBL236_01043_Pyruvate_kinase	RbromiiL2-36
Org4_Gene995	core	2501	[RbromiiL2-36]RBL236_01045_Stage_II_sporulation_protein_AC	RbromiiL2-36
Org4_Gene996	core	3296	[RbromiiL2-36]RBL236_01046_Anti-sigma_F_factor	RbromiiL2-36
Org4_Gene997	core	3765	[RbromiiL2-36]RBL236_01047_Stage_II_sporulation_protein_AA	RbromiiL2-36
Org4_Gene999	core	1955	[RbromiiL2-36]RBL236_01049_Ribosomal_RNA_small_subunit_methyltransferase_I	RbromiiL2-36
Org5_Gene10	core	330	[RbromiL2-63]L2-63_00010_htpG_High_temperature_protein_G,heat_shock_protein_90,Molecular_chaperone,_HSP90_family,Hsp90_protein_12649:1452	RbromiL2-63
Org5_Gene1005	core	1145	[RbromiL2-63]L2-63_01035_L2-63_01035_hypothetical_protein,Sugar_diacid_utilization_regulator,Aminotransferase_class-III_1093336:1094178_forward_	RbromiL2-63
Org5_Gene1026	core	1074	[RbromiL2-63]L2-63_01057_alr_2_Alanine_racemase,alanine_racemase,Uncharacterized_protein_conserved_in_bacteria,alanine_racemase,Alanine_racem	RbromiL2-63
Org5_Gene1027	core	129	[RbromiL2-63]L2-63_01058_polA_DNA_polymerase_I,DNA_polymerase_I,DNA_polymerase_I_-_3'-5'_exonuclease_and_polymerase_domains,DNA_polyme	RbromiL2-63
Org5_Gene1030	core	1404	[RbromiL2-63]L2-63_01061_gcp_1_t(6)A37_threonylcarbamoyladenosine_biosynthesis_protein,UGMP_family_protein,Transcription_initiation_factor_TFII	RbromiL2-63
Org5_Gene1031	core	2990	[RbromiL2-63]L2-63_01062_ppiB_Peptidyl-prolyl_cis-trans_isomerase_B,peptidyl-prolyl_cis-trans_isomerase_A_(rotamase_A),Cyclophilin_type_peptidyl-p	RbromiL2-63
Org5_Gene1032	core	1397	[RbromiL2-63]L2-63_01063_galE_1_UDP-glucose_4-epimerase,UDP-galactose-4-epimerase,UDP-glucose_4-epimerase,NAD_dependent_epimerase/dehydr	RbromiL2-63
Org5_Gene1037	core	4278	[RbromiL2-63]L2-63_01068_rpmB_50S_ribosomal_protein_L28,50S_ribosomal_protein_L28,Ribosomal_protein_L28,ribosomal_protein_L28,Ribosomal_L2	RbromiL2-63
Org5_Gene1039	core	2124	[RbromiL2-63]L2-63_01070_scpA_Segregation_and_condensation_protein_A,segregation_and_condensation_protein_A,ScpA/B_protein_1127768:112854	RbromiL2-63
Org5_Gene1040	core	2631	[RbromiL2-63]L2-63_01071_scpB_Segregation_and_condensation_protein_B,segregation_and_condensation_protein_B,segregation_and_condensation_p	RbromiL2-63
Org5_Gene1042	core	3344	[RbromiL2-63]L2-63_01073_ytfJ_Uncharacterized_spore_protein_ytfJ,Uncharacterized_conserved_protein,sporulation_protein_YtfJ,Sporulation_protein_Y	RbromiL2-63
Org5_Gene1044	core	2196	[RbromiL2-63]L2-63_01075_rluB_Ribosomal_large_subunit_pseudouridine_synthase_B,23S_rRNA_pseudouridine_synthase_F,pseudouridine_synthase,RN	RbromiL2-63
Org5_Gene1045	core	839	[RbromiL2-63]L2-63_01076_L2-63_01076_Methylmalonyl-CoA_carboxyltransferase_12S_subunit,malonate_decarboxylase_subunit_beta,Acetyl-CoA_carbo	RbromiL2-63
Org5_Gene1046	core	3572	[RbromiL2-63]L2-63_01077_gcdC_Glutaconyl-CoA_decarboxylase_subunit_gamma,putative_acetyl-CoA_carboxylase_biotin_carboxyl_carrier_protein_sub	RbromiL2-63
Org5_Gene1047	core	687	[RbromiL2-63]L2-63_01078_L2-63_01078_Methylmalonyl-CoA_carboxyltransferase_5S_subunit,oxaloacetate_decarboxylase,Pyruvate/oxaloacetate_carbo	RbromiL2-63
Org5_Gene1051	core	1341	[RbromiL2-63]L2-63_01082_ilvC_Ketol-acid_reductoisomerase,ketol-acid_reductoisomerase,ketol-acid_reductoisomerase,Acetohydroxy_acid_isomeroredu	RbromiL2-63
Org5_Gene1052	core	3087	[RbromiL2-63]L2-63_01083_ilvH_Acetolactate_synthase_small_subunit,acetolactate_synthase_3_regulatory_subunit,Acetolactate_synthase_(isozyme_II),_	RbromiL2-63
Org5_Gene1053	core	494	[RbromiL2-63]L2-63_01084_ilvB_2_Acetolactate_synthase_large_subunit,acetolactate_synthase_3_catalytic_subunit,Glyoxylate_carboligase,acetolactate_	RbromiL2-63
Org5_Gene1056	core	3702	[RbromiL2-63]L2-63_01087_ziaR_Transcriptional_repressor_smtB_homolog,DNA-binding_transcriptional_repressor_ArsR,Uncharacterized_protein_conser	RbromiL2-63
Org5_Gene1057	core	738	[RbromiL2-63]L2-63_01088_murAB_UDP-N-acetylglucosamine_1-carboxyvinyltransferase_2,UDP-N-acetylglucosamine_1-carboxyvinyltransferase,ATP_pho	RbromiL2-63
Org5_Gene1058	core	2075	[RbromiL2-63]L2-63_01089_L2-63_01089_ribonuclease_Z,Predicted_metal-dependent_RNase,_consists_of_a_metallo-beta-lactamase_domain_and_an_R	RbromiL2-63
Org5_Gene1059	core	3115	[RbromiL2-63]L2-63_01090_rlmH_Ribosomal_RNA_large_subunit_methyltransferase_H,rRNA_large_subunit_methyltransferase,Uncharacterized_conserve	RbromiL2-63
Org5_Gene1060	core	403	[RbromiL2-63]L2-63_01091_L2-63_01091_Fibronectin-binding_protein_A_N-terminus_(FbpA)_1153027:1154766_forward_MW:65715	RbromiL2-63
Org5_Gene1061	core	1766	[RbromiL2-63]L2-63_01092_L2-63_01092_anaerobic_ribonucleoside-triphosphate_reductase_activating_protein,Radical_SAM_superfamily_1155075:1155	RbromiL2-63
Org5_Gene1064	core	1274	[RbromiL2-63]L2-63_01095_mnmA_tRNA-specific_2-thiouridylase_mnmA,tRNA-specific_2-thiouridylase_MnmA,Predicted_ATPase_of_the_PP-loop_super	RbromiL2-63
Org5_Gene1065	core	1649	[RbromiL2-63]L2-63_01096_nadA_Quinolinate_synthase_A,quinolinate_synthetase,quinolinate_synthetase_complex,_A_subunit,Quinolinate_synthetase_	RbromiL2-63
Org5_Gene1069	core	1069	[RbromiL2-63]L2-63_01100_yvhJ_2_Putative_transcriptional_regulator_yvhJ,membrane-bound_transcriptional_regulator_LytR,cell_envelope-related_func	RbromiL2-63
Org5_Gene1070	core	2744	[RbromiL2-63]L2-63_01101_yfbR_5'-nucleotidase_yfbR,5'-nucleotidase,HD_containing_hydrolase-like_enzyme_1167546:1168133_reverse_MW:22320	RbromiL2-63
Org5_Gene1072	core	743	[RbromiL2-63]L2-63_01103_pepV_Beta-Ala-Xaa_dipeptidase,dipeptidase_PepV,putative_dipeptidase,Peptidase_family_M20/M25/M40_1170887:1172248	RbromiL2-63
Org5_Gene1074	core	1262	[RbromiL2-63]L2-63_01105_L2-63_01105_N-glycosyltransferase,Predicted_glycosyl_hydrolase,poly-beta-1,6_N-acetyl-D-glucosamine_synthase,Glycosyl_tr	RbromiL2-63
Org5_Gene1075	core	1664	[RbromiL2-63]L2-63_01106_L2-63_01106_LPS_biosynthesis_protein,LicD_family_1174203:1175078_forward_MW:33999	RbromiL2-63
Org5_Gene1079	core	657	[RbromiL2-63]L2-63_01112_glgA_Glycogen_synthase,glycogen_synthase,Glycogen_synthase,glycogen/starch_synthase,_ADP-glucose_type,Starch_synthas	RbromiL2-63
Org5_Gene1080	core	1212	[RbromiL2-63]L2-63_01113_glgC_1_Glucose-1-phosphate_adenylyltransferase,glucose-1-phosphate_adenylyltransferase,glucose-1-phosphate_adenylyltra	RbromiL2-63
Org5_Gene1081	core	999	[RbromiL2-63]L2-63_01114_glgC_2_Glucose-1-phosphate_adenylyltransferase,glucose-1-phosphate_adenylyltransferase,glucose-1-phosphate_adenylyltra	RbromiL2-63
Org5_Gene1083	core	1352	[RbromiL2-63]L2-63_01116_gap_Glyceraldehyde-3-phosphate_dehydrogenase,glyceraldehyde-3-phosphate_dehydrogenase,Transketolase,glyceraldehyde-	RbromiL2-63
Org5_Gene1084	core	537	[RbromiL2-63]L2-63_01117_rny_Ribonuclease_Y,phosphodiesterase,Predicted_HD_superfamily_hydrolase,conserved_hypothetical_protein_YmdA/YtgF,D	RbromiL2-63
Org5_Gene1085	core	938	[RbromiL2-63]L2-63_01118_amt_2_Ammonia_transporter,ammonium_transporter,Ammonia_permease,ammonium_transporter,Ammonium_Transporter_	RbromiL2-63
Org5_Gene1086	core	3504	[RbromiL2-63]L2-63_01119_glnB_Nitrogen_regulatory_protein_P-II,nitrogen_regulatory_protein_P-II_2,Nitrogen_regulatory_protein_P-II_1188878:11892	RbromiL2-63
Org5_Gene1088	core	461	[RbromiL2-63]L2-63_01121_fadD_Long-chain-fatty-acid--CoA_ligase,long-chain-fatty-acid--CoA_ligase,Uncharacterized_protein_conserved_in_bacteria,O-s	RbromiL2-63
Org5_Gene1089	core	981	[RbromiL2-63]L2-63_01122_fabF_2_3-oxoacyl-[acyl-carrier-protein]_synthase_2,3-oxoacyl-(acyl_carrier_protein)_synthase_II,Enoyl_reductase_domain_of_	RbromiL2-63
Org5_Gene109	core	594	[RbromiL2-63]L2-63_00112_rlmCD_23S_rRNA_(uracil-C(5))-methyltransferase_RlmCD,23S_rRNA_methyluridine_methyltransferase,Predicted_O-methyltra	RbromiL2-63
Org5_Gene1091	core	990	[RbromiL2-63]L2-63_01124_adhE_2_Aldehyde-alcohol_dehydrogenase,bifunctional_acetaldehyde-CoA/alcohol_dehydrogenase,Glycerol_dehydrogenase_	RbromiL2-63
Org5_Gene1096	core	926	[RbromiL2-63]L2-63_01129_L2-63_01129_putative_ion_channel_protein,Voltage_gated_chloride_channel_1198693:1199943_reverse_MW:44809	RbromiL2-63
Org5_Gene110	core	593	[RbromiL2-63]L2-63_00113_gltD_1_Glutamate_synthase_[NADPH]_small_chain,glutamate_synthase_subunit_beta,Monoamine_oxidase,glutamate_synth	RbromiL2-63
Org5_Gene1106	core	2188	[RbromiL2-63]L2-63_01139_thiF_Sulfur_carrier_protein_ThiS_adenylyltransferase,sulfur_acceptor_protein_CsdL,thiazole_biosynthesis_adenylyltransferase	RbromiL2-63
Org5_Gene111	core	18	[RbromiL2-63]L2-63_00114_gltB_Ferredoxin-dependent_glutamate_synthase_1,glutamate_synthase_subunit_alpha,Uncharacterized_conserved_protein,f	RbromiL2-63
Org5_Gene112	core	246	[RbromiL2-63]L2-63_00115_glnA_Glutamine_synthetase,glutamine_synthetase,glutamine_synthetase,_type_I,Glutamine_synthetase_type_III_N_terminal	RbromiL2-63
Org5_Gene113	core	432	[RbromiL2-63]L2-63_00116_amt_1_Ammonia_transporter,ammonium_transporter,Ammonia_permease,ammonium_transporter,Ammonium_Transporter_	RbromiL2-63
Org5_Gene1145	core	231	[RbromiL2-63]L2-63_01178_actP_Copper-transporting_P-type_ATPase,zinc/cadmium/mercury/lead-transporting_ATPase,Uncharacterized_protein_conser	RbromiL2-63
Org5_Gene1155	core	2898	[RbromiL2-63]L2-63_01188_L2-63_01188_TT1679,hypothetical_protein,Uncharacterized_protein_conserved_in_bacteria,conserved_hypothetical_protein,	RbromiL2-63
Org5_Gene116	core	2810	[RbromiL2-63]L2-63_00119_pdtaR_Probable_transcriptional_regulatory_protein_pdtaR,Response_regulator_of_citrate/malate_metabolism,ANTAR_domai	RbromiL2-63
Org5_Gene117	core	1263	[RbromiL2-63]L2-63_00120_carA_Carbamoyl-phosphate_synthase_small_chain,carbamoyl_phosphate_synthase_small_subunit,Carbamoylphosphate_synt	RbromiL2-63
Org5_Gene1172	core	3244	[RbromiL2-63]L2-63_01205_fabZ_2_(3R)-hydroxymyristoyl-[acyl-carrier-protein]_dehydratase,(3R)-hydroxymyristoyl-ACP_dehydratase,beta-hydroxyacyl-(a	RbromiL2-63
Org5_Gene1173	core	2246	[RbromiL2-63]L2-63_01206_fabG_3_3-oxoacyl-[acyl-carrier-protein]_reductase_FabG,3-ketoacyl-(acyl-carrier-protein)_reductase,Uncharacterized_conserv	RbromiL2-63
Org5_Gene1175	core	4031	[RbromiL2-63]L2-63_01208_acpP_2_Acyl_carrier_protein,acyl_carrier_protein,Acyl_carrier_protein,acyl_carrier_protein,Phosphopantetheine_attachment_	RbromiL2-63
Org5_Gene118	core	31	[RbromiL2-63]L2-63_00121_carB_Carbamoyl-phosphate_synthase_large_chain,carbamoyl_phosphate_synthase_large_subunit,Carbamoylphosphate_synth	RbromiL2-63
Org5_Gene1183	core	2494	[RbromiL2-63]L2-63_01219_ntpD_1_V-type_sodium_pump_subunit_D,V-type_ATP_synthase_subunit_D,V-type_ATPase,_D_subunit,ATP_synthase_subuni	RbromiL2-63
Org5_Gene1184	core	723	[RbromiL2-63]L2-63_01220_ntpB_1_V-type_sodium_pump_subunit_B,V-type_ATP_synthase_subunit_B,F0F1-type_ATP_synthase,_beta_subunit,ATP_synt	RbromiL2-63
Org5_Gene1185	core	384	[RbromiL2-63]L2-63_01221_ntpA_V-type_sodium_ATPase_catalytic_subunit_A,V-type_ATP_synthase_subunit_A,F0F1-type_ATP_synthase,_beta_subunit,	RbromiL2-63
Org5_Gene1186	core	3757	[RbromiL2-63]L2-63_01222_ntpG_V-type_sodium_pump_subunit_G,V-type_ATP_synthase_subunit_F,ATP_synthase_(F/14-kDa)_subunit_1293040:129336	RbromiL2-63
Org5_Gene1189	core	3160	[RbromiL2-63]L2-63_01225_ntpK_Sodium_ATPase_proteolipid_component,V-type_ATP_synthase_subunit_K,ATP_synthase_subunit_C_1294962:1295435_	RbromiL2-63
Org5_Gene119	core	492	[RbromiL2-63]L2-63_00122_pyrG_CTP_synthase,CTP_synthetase,CTP_synthase,CTP_synthase_N-terminus_138328:139950_forward_MW:60493	RbromiL2-63
Org5_Gene1193	core	2892	[RbromiL2-63]L2-63_01229_ybaK_Cys-tRNA(Pro)/Cys-tRNA(Cys)_deacylase_ybaK,hypothetical_protein,Uncharacterized_conserved_protein,YbaK/EbsC_pr	RbromiL2-63
Org5_Gene120	core	1050	[RbromiL2-63]L2-63_00123_dapL_1_LL-diaminopimelate_aminotransferase,L,L-diaminopimelate_aminotransferase,Alanine-alpha-ketoisovalerate_(or_vali	RbromiL2-63
Org5_Gene1205	core	2611	[RbromiL2-63]L2-63_01241_ydbD_Probable_manganese_catalase,Mn-containing_catalase,Manganese_containing_catalase_1310096:1310581_reverse_M	RbromiL2-63
Org5_Gene1208	core	2544	[RbromiL2-63]L2-63_01244_tcyB_L-cystine_transport_system_permease_protein_tcyB,amino_acid_ABC_transporter_permease,ABC-type_amino_acid_tra	RbromiL2-63
Org5_Gene1209	core	2029	[RbromiL2-63]L2-63_01245_tcyC_L-cystine_import_ATP-binding_protein_TcyC,putative_amino-acid_ABC_transporter_ATP-binding_protein_YecC,ABC-typ	RbromiL2-63
Org5_Gene121	core	718	[RbromiL2-63]L2-63_00124_glyQS_Glycine--tRNA_ligase,glycyl-tRNA_synthetase,Glycyl-tRNA_synthetase_(class_II),glycine--tRNA_ligase,tRNA_synthetase_	RbromiL2-63
Org5_Gene1223	core	1898	[RbromiL2-63]L2-63_01260_focA_Formate_channel_1,formate_transporter,Formate/nitrite_family_of_transporters,formate/nitrite_transporter,Formate/	RbromiL2-63
Org5_Gene1224	core	224	[RbromiL2-63]L2-63_01261_yhgF_hypothetical_protein,30S_ribosomal_protein_S1,ribosomal_protein_S1,Tex-like_protein_N-terminal_domain_1325646:1	RbromiL2-63
Org5_Gene1227	core	3540	[RbromiL2-63]L2-63_01264_mntR_Manganese_transport_regulator,manganese_transport_regulator_MntR,Iron_dependent_repressor,_metal_binding_an	RbromiL2-63
Org5_Gene1228	core	4164	[RbromiL2-63]L2-63_01265_L2-63_01265_FeoA_domain_1329827:1330039_forward_MW:7486	RbromiL2-63
Org5_Gene1229	core	4121	[RbromiL2-63]L2-63_01266_feoA_Ferrous_iron_transport_protein_A,ferrous_iron_transport_protein_A,FeoA_domain_1330125:1330346_forward_MW:82	RbromiL2-63
Org5_Gene1230	core	134	[RbromiL2-63]L2-63_01267_feoB_1_Ferrous_iron_transport_protein_B,ferrous_iron_transport_protein_B,Fe2__transport_system_protein_B,ferrous_iron_	RbromiL2-63

Org5_Gene1237	core	3361	[RbromiL2-63]L2-63_01274_L2-63_01274_Flavodoxin,flavodoxin,Flavodoxin,flavodoxin,Flavodoxin_1338620:1339042_forward_MW:14608	RbromiL2-63
Org5_Gene1238	core	1043	[RbromiL2-63]L2-63_01275_tdcB_L-threonine_dehydratase_catabolic_TdcB,threonine_dehydratase,Threonine_dehydratase,threonine_ammonia-lyase,Pyr	RbromiL2-63
Org5_Gene1239	core	3555	[RbromiL2-63]L2-63_01276_yabJ_Enamine/imine_deaminase,putative_endoribonuclease_L-PSP,putative_endoribonuclease_L-PSP,Endoribonuclease_L-PSI	RbromiL2-63
Org5_Gene1240	core	1067	[RbromiL2-63]L2-63_01277_metK_S-adenosylmethionine_synthase,S-adenosylmethionine_synthetase,methionine_adenosyltransferase,S-adenosylmethior	RbromiL2-63
Org5_Gene1256	core	2395	[RbromiL2-63]L2-63_01295_sigK_RNA_polymerase_sigma-28_factor_precursor,sporulation_sigma_factor_SigK,RNA_polymerase_sigma-K_factor,Sigma-70_	RbromiL2-63
Org5_Gene1259	core	852	[RbromiL2-63]L2-63_01298_mdeA_3_Methionine_gamma-lyase,O-acetylhomoserine_aminocarboxypropyltransferase,Protoheme_ferro-lyase_(ferrochelat	RbromiL2-63
Org5_Gene1262	core	3849	[RbromiL2-63]L2-63_01301_rpsF_30S_ribosomal_protein_S6,30S_ribosomal_protein_S6,ribosomal_protein_S6,Ribosomal_protein_S6_1359781:1360077_f	RbromiL2-63
Org5_Gene1263	core	3056	[RbromiL2-63]L2-63_01302_ssb_Helix-destabilizing_protein,single-stranded_DNA-binding_protein,single-stranded_DNA-binding_protein,Single-strand_bin	RbromiL2-63
Org5_Gene1264	core	4065	[RbromiL2-63]L2-63_01303_rpsR_BS21,30S_ribosomal_protein_S18,Ribosomal_protein_S18,ribosomal_protein_S18,Ribosomal_protein_S18_1360663:136	RbromiL2-63
Org5_Gene1267	core	3823	[RbromiL2-63]L2-63_01306_csoR_Copper-sensitive_operon_repressor,regulator_protein_FrmR,Uncharacterised_BCR,_COG1937_1361739:1362044_forwar	RbromiL2-63
Org5_Gene1268	core	137	[RbromiL2-63]L2-63_01307_copA_Probable_copper-importing_P-type_ATPase_A,copper_exporting_ATPase,Copper_chaperone,heavy_metal_translocating	RbromiL2-63
Org5_Gene127	core	3965	[RbromiL2-63]L2-63_00130_ptsH_Phosphocarrier_protein_HPr,phosphocarrier_protein_HPr,Uncharacterized_protein_conserved_in_bacteria,phosphocarr	RbromiL2-63
Org5_Gene1270	core	3817	[RbromiL2-63]L2-63_01309_L2-63_01309_hypothetical_protein_1365447:1365752_reverse_MW:11606	RbromiL2-63
Org5_Gene1271	core	4032	[RbromiL2-63]L2-63_01310_L2-63_01310_hypothetical_protein,hypothetical_protein,Uncharacterized_protein_conserved_in_bacteria,Bacterial_protein_o	RbromiL2-63
Org5_Gene1272	core	169	[RbromiL2-63]L2-63_01311_pheT_Phenylalanine--tRNA_ligase_beta_subunit,phenylalanyl-tRNA_synthetase_subunit_beta,Phenylalanyl-tRNA_synthetase_	RbromiL2-63
Org5_Gene1273	core	1316	[RbromiL2-63]L2-63_01312_pheS_Phenylalanine--tRNA_ligase_alpha_subunit,phenylalanyl-tRNA_synthetase_subunit_alpha,Phenylalanyl-tRNA_synthetas	RbromiL2-63
Org5_Gene1282	core	3646	[RbromiL2-63]L2-63_01322_mazF_mRNA_interferase_MazF,toxin_MazF,PemK-like_protein_1376034:1376381_forward_MW:12412	RbromiL2-63
Org5_Gene1284	core	2610	[RbromiL2-63]L2-63_01324_cobC_Alpha-ribazole_phosphatase,bifunctional_RNase_H/acid_phosphatase,alpha-ribazole_phosphatase,Histidine_phosphata	RbromiL2-63
Org5_Gene1285	core	1285	[RbromiL2-63]L2-63_01325_leuB_3-isopropylmalate_dehydrogenase,3-isopropylmalate_dehydrogenase,3-isopropylmalate_dehydrogenase,Isocitrate/isopr	RbromiL2-63
Org5_Gene1287	core	3330	[RbromiL2-63]L2-63_01327_nifU_1_NifU-like_protein,scaffold_protein,FeS_cluster_assembly_scaffold_protein_NifU,NifU-like_N_terminal_domain_13800	RbromiL2-63
Org5_Gene1288	core	1027	[RbromiL2-63]L2-63_01328_iscS_2_Cysteine_desulfurase,cysteine_desulfurase,Uncharacterized_protein_conserved_in_bacteria,cysteine_desulfurase_NifS	RbromiL2-63
Org5_Gene1289	core	3091	[RbromiL2-63]L2-63_01329_rnhA_Ribonuclease_HI,ribonuclease_H,Predicted_double-stranded_RNA/RNA-DNA_hybrid_binding_protein,RNase_H_138176	RbromiL2-63
Org5_Gene1290	core	320	[RbromiL2-63]L2-63_01330_rnjA_Ribonuclease_J_1,Predicted_metal-dependent_RNase,_consists_of_a_metallo-beta-lactamase_domain_and_an_RNA-bin	RbromiL2-63
Org5_Gene1292	core	3258	[RbromiL2-63]L2-63_01332_rplI_BL17,50S_ribosomal_protein_L9,Ribosomal_protein_L9,ribosomal_protein_L9,Ribosomal_protein_L9,_C-terminal_domain	RbromiL2-63
Org5_Gene1293	core	725	[RbromiL2-63]L2-63_01333_dnaC_1_Replicative_DNA_helicase,replicative_DNA_helicase,Replicative_DNA_helicase,replicative_DNA_helicase,DnaB-like_he	RbromiL2-63
Org5_Gene1295	core	2937	[RbromiL2-63]L2-63_01335_hpt_Hypoxanthine-guanine_phosphoribosyltransferase,hypoxanthine_phosphoribosyltransferase,Predicted_ATPase_of_the_P	RbromiL2-63
Org5_Gene1296	core	270	[RbromiL2-63]L2-63_01336_ftsH_ATP-dependent_zinc_metalloprotease_FtsH,ATP-dependent_metalloprotease,Ribosome-associated_chaperone_zuotin,A	RbromiL2-63
Org5_Gene1297	core	99	[RbromiL2-63]L2-63_01337_uvrA_Excinuclease_ABC_subunit_A,excinuclease_ABC_subunit_A,Predicted_ABC-type_transport_system_involved_in_lysopho	RbromiL2-63
Org5_Gene1298	core	3992	[RbromiL2-63]L2-63_01338_L2-63_01338_hypothetical_protein_1395516:1395773_forward_MW:9701	RbromiL2-63
Org5_Gene13	core	2164	[RbromiL2-63]L2-63_00013_pyrK_1_Dihydrdoorotate_oxidase_B,_electron_transfer_subunit,dihydroorotate_dehydrogenase_electron_transfer_subunit,su	RbromiL2-63
Org5_Gene1306	core	298	[RbromiL2-63]L2-63_01347_thrS_Threonine--tRNA_ligase,threonyl-tRNA_synthetase,Prolyl-tRNA_synthetase,threonine--tRNA_ligase,tRNA_synthetase_cla	RbromiL2-63
Org5_Gene1308	core	4201	[RbromiL2-63]L2-63_01349_rpmE_50S_ribosomal_protein_L31,50S_ribosomal_protein_L31,Ribosomal_protein_L31,ribosomal_protein_L31,Ribosomal_pro	RbromiL2-63
Org5_Gene1309	core	2603	[RbromiL2-63]L2-63_01350_yigZ_IMPACT_family_member_yigZ,hypothetical_protein,uncharacterized_protein,_YigZ_family,Uncharacterized_protein_fami	RbromiL2-63
Org5_Gene1310	core	383	[RbromiL2-63]L2-63_01351_dnaG_2_DNA_primase,DNA_primase,DNA_primase_(bacterial_type),DNA_primase,DNA_primase_catalytic_core,_N-terminal_	RbromiL2-63
Org5_Gene1311	core	1216	[RbromiL2-63]L2-63_01352_rpoD_RNA_polymerase_sigma_factor_rpoD,RNA_polymerase_sigma_factor_RpoD,RNA_polymerase_sigma_factor_RpoD,Sigm	RbromiL2-63
Org5_Gene1312	core	3557	[RbromiL2-63]L2-63_01353_L2-63_01353_hypothetical_protein_1408818:1409189_forward_MW:14362	RbromiL2-63
Org5_Gene1315	core	92	[RbromiL2-63]L2-63_01356_pulA_2_Pullulanase_precursor,glycogen_debranching_enzyme,pullulanase,_type_I,Alpha_amylase,_catalytic_domain_141137	RbromiL2-63
Org5_Gene1317	core	2589	[RbromiL2-63]L2-63_01358_rex_Redox-sensing_transcriptional_repressor_rex,redox-sensing_transcriptional_repressor_Rex,Putative_DNA-binding_protein	RbromiL2-63
Org5_Gene1318	core	1011	[RbromiL2-63]L2-63_01359_icd_Isocitrate_dehydrogenase_[NADP],isocitrate_dehydrogenase,isocitrate_dehydrogenase,_NADP-dependent,Isocitrate/isop	RbromiL2-63
Org5_Gene1319	core	307	[RbromiL2-63]L2-63_01360_dmdA_2_2,3-dimethylmalate_dehydratase_large_subunit,aconitate_hydratase,3-isopropylmalate_dehydratase_large_subunit,	RbromiL2-63
Org5_Gene132	core	215	[RbromiL2-63]L2-63_00135_nrdD_Anaerobic_ribonucleoside-triphosphate_reductase,anaerobic_ribonucleoside_triphosphate_reductase,anaerobic_ribonu	RbromiL2-63
Org5_Gene1320	core	359	[RbromiL2-63]L2-63_01361_typA_Tyrosine_phosphorylated_protein_A,GTP-binding_protein,GTPases_-_translation_elongation_factors,GTP-binding_prote	RbromiL2-63
Org5_Gene1327	core	710	[RbromiL2-63]L2-63_01368_murC_UDP-N-acetylmuramate--L-alanine_ligase,UDP-N-acetylmuramate--L-alanine_ligase,Folylpolyglutamate_synthase,UDP-N	RbromiL2-63
Org5_Gene1328	core	729	[RbromiL2-63]L2-63_01369_L2-63_01369_Uncharacterized_RNA_methyltransferase_SAV1897,23S_rRNA_m(5)U1939_methyltransferase,Predicted_RNA-bi	RbromiL2-63
Org5_Gene133	core	2925	[RbromiL2-63]L2-63_00136_L2-63_00136_anaerobic_ribonucleotide_reductase-activating_protein,anaerobic_ribonucleoside-triphosphate_reductase_acti	RbromiL2-63
Org5_Gene1331	core	2145	[RbromiL2-63]L2-63_01372_L2-63_01372_Uncharacterized_oxidoreductase_SAV2478,3-ketoacyl-(acyl-carrier-protein)_reductase,Uncharacterized_conserv	RbromiL2-63
Org5_Gene1335	core	398	[RbromiL2-63]L2-63_01376_L2-63_01376_Putative_multidrug_export_ATP-binding/permease_protein_SAV1866,cyclic_beta-1,2-glucan_ABC_transporter,A	RbromiL2-63
Org5_Gene1336	core	323	[RbromiL2-63]L2-63_01377_yheH_Probable_multidrug_resistance_ABC_transporter_ATP-binding/permease_protein_YheH,lipid_transporter_ATP-binding/	RbromiL2-63
Org5_Gene135	core	1015	[RbromiL2-63]L2-63_00138_lysC_Aspartokinase_2,aspartate_kinase,Uridylate_kinase,aspartate_kinase,Amino_acid_kinase_family_157964:159169_reverse	RbromiL2-63
Org5_Gene1353	core	2038	[RbromiL2-63]L2-63_01394_proC_Pyrroline-5-carboxylate_reductase,pyrroline-5-carboxylate_reductase,Pyrroline-5-carboxylate_reductase,pyrroline-5-carb	RbromiL2-63
Org5_Gene1359	core	4043	[RbromiL2-63]L2-63_01400_L2-63_01400_hypothetical_protein_1462792:1463025_reverse_MW:9039	RbromiL2-63
Org5_Gene136	core	957	[RbromiL2-63]L2-63_00139_hom_Homoserine_dehydrogenase,homoserine_dehydrogenase,Homoserine_dehydrogenase,Homoserine_dehydrogenase_15	RbromiL2-63
Org5_Gene1360	core	898	[RbromiL2-63]L2-63_01401_dinB_DNA_polymerase_IV,DNA_polymerase_IV,Nucleotidyltransferase/DNA_polymerase_involved_in_DNA_repair,impB/muc	RbromiL2-63
Org5_Gene1362	core	2443	[RbromiL2-63]L2-63_01403_pyrE_Orotate_phosphoribosyltransferase,orotate_phosphoribosyltransferase,orotate_phosphoribosyltransferase,Phosphoribo	RbromiL2-63
Org5_Gene1363	core	2348	[RbromiL2-63]L2-63_01404_L2-63_01404_hypothetical_protein,hypothetical_protein,DNA_repair_protein_RadC,Protein_of_unknown_function_(DUF2466	RbromiL2-63
Org5_Gene1364	core	290	[RbromiL2-63]L2-63_01405_uvrB_hypothetical_protein,excinuclease_ABC_subunit_B,Superfamily_II_DNA/RNA_helicase_required_for_DNA_uptake_(late_	RbromiL2-63
Org5_Gene1365	core	282	[RbromiL2-63]L2-63_01406_gyrB_2_DNA_gyrase_subunit_B,DNA_topoisomerase_IV_subunit_B,DNA_gyrase,_B_subunit,DNA_gyrase_B_1469296:1471281	RbromiL2-63
Org5_Gene1366	core	203	[RbromiL2-63]L2-63_01407_gyrA_2_DNA_gyrase_subunit_A,DNA_topoisomerase_IV_subunit_A,Type_IIA_topoisomerase_(DNA_gyrase/topo_II,_topoisom	RbromiL2-63
Org5_Gene1367	core	4015	[RbromiL2-63]L2-63_01408_L2-63_01408_preprotein_translocase_subunit_SecG,preprotein_translocase,_SecG_subunit,Preprotein_translocase_SecG_sub	RbromiL2-63
Org5_Gene139	core	1551	[RbromiL2-63]L2-63_00142_L2-63_00142_Nitronate_monooxygenase,dihydroorotate_dehydrogenase_1B,Dihydroorotate_dehydrogenase,putative_enoyl-	RbromiL2-63
Org5_Gene1391	core	892	[RbromiL2-63]L2-63_01432_L2-63_01432_protease3,coenzyme_PQQ_biosynthesis_protein_PqqF,Insulinase_(Peptidase_family_M16)_1499019:1500296_f	RbromiL2-63
Org5_Gene1392	core	1866	[RbromiL2-63]L2-63_01433_folD_Bifunctional_protein_FolD,bifunctional_5,10-methylene-tetrahydrofolate_dehydrogenase/_5,10-methylene-tetrahydrofo	RbromiL2-63
Org5_Gene1393	core	3807	[RbromiL2-63]L2-63_01434_rplU_50S_ribosomal_protein_L21,50S_ribosomal_protein_L21,ribosomal_protein_L21,Ribosomal_prokaryotic_L21_protein_15	RbromiL2-63
Org5_Gene1395	core	3887	[RbromiL2-63]L2-63_01436_rpmA_50S_ribosomal_protein_L27,50S_ribosomal_protein_L27,Ribosomal_protein_L27,ribosomal_protein_L27,Ribosomal_L2	RbromiL2-63
Org5_Gene1396	core	909	[RbromiL2-63]L2-63_01437_obg_Spo0B-associated_GTP-binding_protein,GTPase_CgtA,Predicted_GTPase,_probable_translation_factor,Obg_family_GTPas	RbromiL2-63
Org5_Gene1397	core	3444	[RbromiL2-63]L2-63_01438_mrnC_Mini-ribonuclease_3,ribonuclease_III_1504440:1504841_forward_MW:14890	RbromiL2-63
Org5_Gene14	core	1658	[RbromiL2-63]L2-63_00014_pyrDB_Dihydroorotate_dehydrogenase_B_(NAD(_)),_catalytic_subunit,dihydroorotate_dehydrogenase_1B,Dihydroorotate_de	RbromiL2-63
Org5_Gene140	core	1688	[RbromiL2-63]L2-63_00143_fenF_Malonyl_CoA-acyl_carrier_protein_transacylase,(acyl-carrier-protein)_S-malonyltransferase,malonyl_CoA-acyl_carrier_pr	RbromiL2-63
Org5_Gene1401	core	1964	[RbromiL2-63]L2-63_01442_L2-63_01442_enhanced_serine_sensitivity_protein_SseB,SseB_protein_1509028:1509846_forward_MW:30802	RbromiL2-63
Org5_Gene1403	core	2177	[RbromiL2-63]L2-63_01444_hisF_Imidazole_glycerol_phosphate_synthase_subunit_HisF,imidazole_glycerol_phosphate_synthase_subunit_HisF,Imidazoleg	RbromiL2-63
Org5_Gene1404	core	2620	[RbromiL2-63]L2-63_01445_hisH1_Imidazole_glycerol_phosphate_synthase_subunit_HisH_1,imidazole_glycerol_phosphate_synthase_subunit_HisH,Imida	RbromiL2-63
Org5_Gene1405	core	1715	[RbromiL2-63]L2-63_01446_murR_MurPQ_operon_repressor,putative_DNA-binding_transcriptional_regulator,6-phospho_3-hexuloisomerase,SIS_domain_	RbromiL2-63
Org5_Gene1406	core	952	[RbromiL2-63]L2-63_01447_L2-63_01447_hypothetical_protein,tricarballylate_dehydrogenase,Uncharacterized_conserved_protein,flavoprotein,_HI0933_f	RbromiL2-63
Org5_Gene1407	core	2482	[RbromiL2-63]L2-63_01448_cmk_Cytidylate_kinase,cytidylate_kinase,Cytidylate_kinase,cytidylate_kinase,Cytidylate_kinase_1516133:1516801_forward_M	RbromiL2-63
Org5_Gene1408	core	2586	[RbromiL2-63]L2-63_01449_plsC_1-acyl-sn-glycerol-3-phosphate_acyltransferase,2-acyl-glycerophospho-ethanolamine_acyltransferase,Glycerol-3-phospha	RbromiL2-63
Org5_Gene1409	core	279	[RbromiL2-63]L2-63_01450_ypfD_30S_ribosomal_protein_S1_homolog,4-hydroxy-3-methylbut-2-enyl_diphosphate_reductase/S1_RNA-binding_domain_p	RbromiL2-63
Org5_Gene141	core	2214	[RbromiL2-63]L2-63_00144_fabG_1_3-oxoacyl-[acyl-carrier-protein]_reductase_FabG,3-ketoacyl-(acyl-carrier-protein)_reductase,Uncharacterized_conserv	RbromiL2-63
Org5_Gene1411	core	2241	[RbromiL2-63]L2-63_01452_yebC_Probable_transcriptional_regulatory_protein_YebC,hypothetical_protein,DNA-binding_regulatory_protein,_YebC/PmpR_	RbromiL2-63
Org5_Gene1412	core	1747	[RbromiL2-63]L2-63_01453_L2-63_01453_hypothetical_protein,hypothetical_protein,conserved_hypothetical_protein,YicC-like_family,_N-terminal_region	RbromiL2-63
Org5_Gene1413	core	3994	[RbromiL2-63]L2-63_01454_L2-63_01454_hypothetical_protein,Uncharacterized_protein_conserved_in_bacteria,Domain_of_unknown_function_(DUF370)	RbromiL2-63
Org5_Gene1415	core	4190	[RbromiL2-63]L2-63_01456_L2-63_01456_DNA-directed_RNA_polymerase_subunit_omega,DNA-directed_RNA_polymerase,_omega_subunit,RNA_polyme	RbromiL2-63
Org5_Gene1416	core	144	[RbromiL2-63]L2-63_01457_priA_Primosomal_protein_N',primosome_assembly_protein_PriA,ATP-dependent_protease_HslVU_(ClpYQ),_peptidase_subur	RbromiL2-63
Org5_Gene1417	core	3150	[RbromiL2-63]L2-63_01458_def1_2_Peptide_deformylase_1,peptide_deformylase,N-formylmethionyl-tRNA_deformylase,peptide_deformylase,Polypeptid	RbromiL2-63
Org5_Gene1418	core	1641	[RbromiL2-63]L2-63_01459_fmt_Methionyl-tRNA_formyltransferase,methionyl-tRNA_formyltransferase,Folate-dependent_phosphoribosylglycinamide_for	RbromiL2-63
Org5_Gene1419	core	2364	[RbromiL2-63]L2-63_01460_L2-63_01460_Putative_neutral_zinc_metallopeptidase_1527679:1528383_forward_MW:26065	RbromiL2-63
Org5_Gene142	core	970	[RbromiL2-63]L2-63_00145_fabF_1_3-oxoacyl-[acyl-carrier-protein]_synthase_2,3-oxoacyl-(acyl_carrier_protein)_synthase_II,Enoyl_reductase_domain_of_	RbromiL2-63
Org5_Gene1421	core	1357	[RbromiL2-63]L2-63_01462_rlmN_2_Ribosomal_RNA_large_subunit_methyltransferase_N,ribosomal_RNA_large_subunit_methyltransferase_N,23S_rRNA_	RbromiL2-63
Org5_Gene1422	core	2278	[RbromiL2-63]L2-63_01463_stp_Serine/threonine_phosphatase_stp,putative_protein_serine/threonine_phosphatase,Protein_phosphatase_2C_1530784:1	RbromiL2-63
Org5_Gene1423	core	238	[RbromiL2-63]L2-63_01464_prkC_2_Serine/threonine-protein_kinase_PrkC,serine/threonine-protein_kinase,Predicted_ATPase,TOMM_system_kinase/cycl	RbromiL2-63
Org5_Gene1424	core	1753	[RbromiL2-63]L2-63_01465_rsgA_Putative_ribosome_biogenesis_GTPase_RsgA,GTPase_RsgA,ribosome_small_subunit-dependent_GTPase_A,Protein_of_	RbromiL2-63
Org5_Gene1426	core	480	[RbromiL2-63]L2-63_01468_prkC_3_Serine/threonine-protein_kinase_PrkC,Predicted_ATPase,PASTA_domain_1535473:1537107_forward_MW:60298	RbromiL2-63
Org5_Gene1427	core	3886	[RbromiL2-63]L2-63_01469_groS_hypothetical_protein,co-chaperonin_GroES,Chaperonin_10_Kd_subunit_1537374:1537658_forward_MW:10009	RbromiL2-63
Org5_Gene1428	core	487	[RbromiL2-63]L2-63_01470_groL_hypothetical_protein,chaperonin_GroEL,chaperonin_GroL,TCP-1/cpn60_chaperonin_family_1537687:1539315_forward_	RbromiL2-63
Org5_Gene1429	core	3967	[RbromiL2-63]L2-63_01471_L2-63_01471_hypothetical_protein_1539471:1539734_forward_MW:9924	RbromiL2-63
Org5_Gene1434	core	1512	[RbromiL2-63]L2-63_01476_gpsA_Glycerol-3-phosphate_dehydrogenase_[NAD(P)_],NAD(P)H-dependent_glycerol-3-phosphate_dehydrogenase,Glycerol-3	RbromiL2-63
Org5_Gene1436	core	2431	[RbromiL2-63]L2-63_01479_wcaJ_3_Putative_colanic_biosynthesis_UDP-glucose_lipid_carrier_transferase,putative_UDP-glucose_lipid_carrier_transferase	RbromiL2-63
Org5_Gene1438	core	313	[RbromiL2-63]L2-63_01481_nadE_Glutamine-dependent_NAD(_)_synthetase,NAD_synthetase,NAD_synthase,NAD__synthetase,NAD_synthase_1548450:1	RbromiL2-63
Org5_Gene144	core	3377	[RbromiL2-63]L2-63_00147_fabZ_1_(3R)-hydroxymyristoyl-[acyl-carrier-protein]_dehydratase,bifunctional_UDP-3-O-[3-hydroxymyristoyl]_N-acetylglucosa	RbromiL2-63
Org5_Gene1440	core	3130	[RbromiL2-63]L2-63_01483_lrp_Leucine-responsive_regulatory_protein,leucine-responsive_transcriptional_regulator,AsnC_family_1551077:1551559_forw	RbromiL2-63
Org5_Gene1441	core	1075	[RbromiL2-63]L2-63_01484_patA_Putative_aminotransferase_A,hypothetical_protein,Prephenate_dehydrogenase,LL-diaminopimelate_aminotransferase,	RbromiL2-63
Org5_Gene1442	core	1801	[RbromiL2-63]L2-63_01485_ispE_4-diphosphocytidyl-2-C-methyl-D-erythritol_kinase,4-diphosphocytidyl-2-C-methyl-D-erythritol_kinase,Archaeal_shikimat	RbromiL2-63
Org5_Gene1447	core	1282	[RbromiL2-63]L2-63_01490_pyrB_Aspartate_carbamoyltransferase_catalytic_chain,aspartate_carbamoyltransferase_catalytic_subunit,Aspartate_carbamo	RbromiL2-63
Org5_Gene145	core	748	[RbromiL2-63]L2-63_00148_cfiB_2-oxoglutarate_carboxylase_small_subunit,acetyl-CoA_carboxylase_biotin_carboxylase_subunit,Acetyl/propionyl-CoA_ca	RbromiL2-63
Org5_Gene1453	core	413	[RbromiL2-63]L2-63_01496_pgcA_Phosphoglucomutase,phosphoglucomutase,Deoxyribose-phosphate_aldolase,phosphoglucosamine_mutase,Phosphoglu	RbromiL2-63
Org5_Gene1456	core	1540	[RbromiL2-63]L2-63_01499_ykoT_Uncharacterized_glycosyltransferase_ykoT,undecaprenyl_phosphate_4-deoxy-4-formamido-L-arabinose_transferase,Pre	RbromiL2-63
Org5_Gene1457	core	1483	[RbromiL2-63]L2-63_01500_pfkA_6-phosphofructokinase,6-phosphofructokinase,P_pilus_assembly/Cpx_signaling_pathway,_periplasmic_inhibitor/zinc-res	RbromiL2-63
Org5_Gene146	core	1826	[RbromiL2-63]L2-63_00149_accD_Acetyl-coenzyme_A_carboxylase_carboxyl_transferase_subunit_beta,acetyl-CoA_carboxylase_subunit_beta,Acetyl-CoA_	RbromiL2-63
Org5_Gene1461	core	2379	[RbromiL2-63]L2-63_01504_sigE_RNA_polymerase_sigma-35_factor_precursor,sporulation_sigma_factor_SigE,RNA_polymerase_sigma-E_factor,Sigma-70_	RbromiL2-63
Org5_Gene1463	core	1754	[RbromiL2-63]L2-63_01506_L2-63_01506_DegV_domain-containing_protein_SAV1425,EDD_domain_protein,_DegV_family,Uncharacterised_protein,_Deg	RbromiL2-63
Org5_Gene1464	core	3854	[RbromiL2-63]L2-63_01507_L2-63_01507_Uncharacterized_protein_conserved_in_bacteria,sporulation_protein,_YlmC/YmxH_family,PRC-barrel_domain_1	RbromiL2-63
Org5_Gene1466	core	2264	[RbromiL2-63]L2-63_01509_rpsB_Vegetative_protein_209,30S_ribosomal_protein_S2,Ribosomal_protein_S2,ribosomal_protein_S2,Ribosomal_protein_S2	RbromiL2-63
Org5_Gene1467	core	1659	[RbromiL2-63]L2-63_01510_tsf_Elongation_factor_Ts,elongation_factor_Ts,Translation_elongation_factor_Ts,translation_elongation_factor_Ts,Elongation	RbromiL2-63
Org5_Gene1468	core	4231	[RbromiL2-63]L2-63_01512_rpmI_Ribosomal_protein_A,50S_ribosomal_protein_L35,Ribosomal_protein_L35,ribosomal_protein_L35,Ribosomal_protein_L	RbromiL2-63
Org5_Gene1469	core	3634	[RbromiL2-63]L2-63_01513_rplT_50S_ribosomal_protein_L20,50S_ribosomal_protein_L20,ribosomal_protein_L20,Ribosomal_protein_L20_1588774:15891	RbromiL2-63
Org5_Gene147	core	2088	[RbromiL2-63]L2-63_00150_accA_Acetyl-coenzyme_A_carboxylase_carboxyl_transferase_subunit_alpha,acetyl-CoA_carboxylase_carboxyltransferase_sub	RbromiL2-63
Org5_Gene1472	core	249	[RbromiL2-63]L2-63_01516_topA_DNA_topoisomerase_1,DNA_topoisomerase_I,Zn-finger_domain_associated_with_topoisomerase_type_I,DNA_topoison	RbromiL2-63
Org5_Gene1473	core	828	[RbromiL2-63]L2-63_01517_trmFO_Methylenetetrahydrofolate--tRNA-(uracil-5-)-methyltransferase_TrmFO,tRNA_(uracil-5-)-methyltransferase_Gid,tRNA:	RbromiL2-63
Org5_Gene1474	core	1426	[RbromiL2-63]L2-63_01518_plsX_Phosphate_acyltransferase,putative_phosphate_acyltransferase,Fatty_acid/phospholipid_biosynthesis_enzyme,fatty_aci	RbromiL2-63
Org5_Gene1475	core	2444	[RbromiL2-63]L2-63_01519_rnc_Ribonuclease_3,ribonuclease_III,ribonuclease_III,RNase3_domain_1595741:1596418_forward_MW:25141	RbromiL2-63
Org5_Gene1477	core	47	[RbromiL2-63]L2-63_01521_smc_Chromosome_partition_protein_Smc,chromosome_segregation_protein,ATPase_involved_in_DNA_repair,chromosome_s	RbromiL2-63
Org5_Gene1478	core	1644	[RbromiL2-63]L2-63_01522_ftsY_Cell_division_protein_FtsY_homolog,signal_recognition_particle-docking_protein_FtsY,Signal_recognition_particle_GTPas	RbromiL2-63
Org5_Gene1479	core	2674	[RbromiL2-63]L2-63_01523_L2-63_01523_Non-canonical_purine_NTP_pyrophosphatase,dITP/XTP_pyrophosphatase,Xanthosine_triphosphate_pyrophosp	RbromiL2-63

Org5_Gene1480	core	3821	[RbromiL2-63]L2-63_01524_L2-63_01524_RNA-binding_protein_HI_1333,RNA-binding_protein_YhbY,putative_RNA-binding_protein,_YhbY_family,CRS1_/_	RbromiL2-63
Org5_Gene1483	core	3626	[RbromiL2-63]L2-63_01527_ybeB_hypothetical_protein,ribosome-associated_protein,iojap-like_ribosome-associated_protein,Domain_of_unknown_functi	RbromiL2-63
Org5_Gene1484	core	155	[RbromiL2-63]L2-63_01528_leuS_Leucine--tRNA_ligase,leucyl-tRNA_synthetase,Leucyl-tRNA_synthetase,leucine--tRNA_ligase,tRNA_synthetases_class_I_(	RbromiL2-63
Org5_Gene1485	core	4323	[RbromiL2-63]L2-63_01529_rpsU_30S_ribosomal_protein_S21,30S_ribosomal_protein_S21,ribosomal_protein_S21,Ribosomal_protein_S21_1606957:1607	RbromiL2-63
Org5_Gene1486	core	3327	[RbromiL2-63]L2-63_01530_yqhS_3-dehydroquinate_dehydratase,3-dehydroquinate_dehydratase,3-dehydroquinate_dehydratase_II,3-dehydroquinate_de	RbromiL2-63
Org5_Gene1488	core	2854	[RbromiL2-63]L2-63_01532_efp_Elongation_factor_P,elongation_factor_P,translation_elongation_factor_P,Elongation_factor_P,_C-terminal_1608833:160	RbromiL2-63
Org5_Gene1491	core	673	[RbromiL2-63]L2-63_01535_miaB_(Dimethylallyl)adenosine_tRNA_methylthiotransferase_MiaB,(dimethylallyl)adenosine_tRNA_methylthiotransferase,tRN	RbromiL2-63
Org5_Gene1492	core	3428	[RbromiL2-63]L2-63_01536_L2-63_01536_Protein_of_unknown_function_(DUF964)_1612108:1612515_forward_MW:14980	RbromiL2-63
Org5_Gene1493	core	127	[RbromiL2-63]L2-63_01537_mutS_DNA_mismatch_repair_protein_mutS,DNA_mismatch_repair_protein_MutS,ATP_phosphoribosyltransferase,DNA_mism	RbromiL2-63
Org5_Gene1497	core	2317	[RbromiL2-63]L2-63_01541_yggS_hypothetical_protein,alanine_racemase,Predicted_enzyme_with_a_TIM-barrel_fold,pyridoxal_phosphate_enzyme,_YggS	RbromiL2-63
Org5_Gene1498	core	3192	[RbromiL2-63]L2-63_01542_sepF_Cell_division_protein_SepF,Protein_of_unknown_function_(DUF552)_1620226:1620693_forward_MW:17612	RbromiL2-63
Org5_Gene1501	core	102	[RbromiL2-63]L2-63_01545_ileS_Isoleucine--tRNA_ligase,isoleucyl-tRNA_synthetase,Isoleucyl-tRNA_synthetase,isoleucine--tRNA_ligase,tRNA_synthetases	RbromiL2-63
Org5_Gene1502	core	3126	[RbromiL2-63]L2-63_01546_lspA_Lipoprotein_signal_peptidase,lipoprotein_signal_peptidase,signal_peptidase_II,Signal_peptidase_(SPase)_II_1624980:16	RbromiL2-63
Org5_Gene1503	core	1638	[RbromiL2-63]L2-63_01547_rluD_1_Ribosomal_large_subunit_pseudouridine_synthase_D,23S_rRNA_pseudouridine_synthase_D,pseudouridine_synthase,	RbromiL2-63
Org5_Gene1504	core	3645	[RbromiL2-63]L2-63_01548_rplS_50S_ribosomal_protein_L19,50S_ribosomal_protein_L19,Ribosomal_protein_L19,ribosomal_protein_L19,Ribosomal_prot	RbromiL2-63
Org5_Gene1507	core	1771	[RbromiL2-63]L2-63_01551_rbgA_Ribosome_biogenesis_GTPase_A,GTPase_YlqF,Predicted_GTPase,ribosome_biogenesis_GTP-binding_protein_YlqF,GTPa	RbromiL2-63
Org5_Gene1508	core	2656	[RbromiL2-63]L2-63_01552_rnhB_Ribonuclease_HII,ribonuclease_HII,ribonuclease_HII,Ribonuclease_HII_1629257:1629862_forward_MW:22399	RbromiL2-63
Org5_Gene1511	core	574	[RbromiL2-63]L2-63_01555_thrC_Threonine_synthase,threonine_synthase,threonine_synthase,Pyridoxal-phosphate_dependent_enzyme_1631215:163272	RbromiL2-63
Org5_Gene1512	core	2412	[RbromiL2-63]L2-63_01556_L2-63_01556_Predicted_phosphoesterase_or_phosphohydrolase,Calcineurin-like_phosphoesterase_1632723:1633412_forwar	RbromiL2-63
Org5_Gene1513	core	890	[RbromiL2-63]L2-63_01557_tig_Trigger_factor,trigger_factor,FKBP-type_peptidyl-prolyl_cis-trans_isomerase_(trigger_factor),trigger_factor,Bacterial_trigg	RbromiL2-63
Org5_Gene1514	core	2763	[RbromiL2-63]L2-63_01558_clpP_ATP-dependent_Clp_protease_proteolytic_subunit,ATP-dependent_Clp_protease_proteolytic_subunit,ATP-dependent_C	RbromiL2-63
Org5_Gene1515	core	781	[RbromiL2-63]L2-63_01559_clpX_ATP-dependent_Clp_protease_ATP-binding_subunit_ClpX,ATP-dependent_protease_ATP-binding_subunit_ClpX,DnaK_su	RbromiL2-63
Org5_Gene1516	core	156	[RbromiL2-63]L2-63_01560_lon1_Lon_protease_1,DNA-binding_ATP-dependent_protease_La,Archaeal_serine_proteases,ATP-dependent_protease_La,Lor	RbromiL2-63
Org5_Gene1517	core	2688	[RbromiL2-63]L2-63_01561_engB_Probable_GTP-binding_protein_EngB,GTP-binding_protein_YsxC,Predicted_GTPase,ribosome_biogenesis_GTP-binding_	RbromiL2-63
Org5_Gene1518	core	857	[RbromiL2-63]L2-63_01562_lysA_2_Diaminopimelate_decarboxylase,bifunctional_aspartate_kinase/diaminopimelate_decarboxylase_protein,Diaminopime	RbromiL2-63
Org5_Gene1519	core	2370	[RbromiL2-63]L2-63_01563_L2-63_01563_Predicted_membrane_protein,Protein_of_unknown_function_(DUF1275)_1641262:1641954_forward_MW:2604	RbromiL2-63
Org5_Gene1520	core	796	[RbromiL2-63]L2-63_01564_gdhA_NADP-specific_glutamate_dehydrogenase,glutamate_dehydrogenase,Glutamate/Leucine/Phenylalanine/Valine_dehydr	RbromiL2-63
Org5_Gene1521	core	1460	[RbromiL2-63]L2-63_01565_xerD_Tyrosine_recombinase_XerD,site-specific_tyrosine_recombinase_XerC,Site-specific_recombinase_XerD,tyrosine_recomb	RbromiL2-63
Org5_Gene1523	core	734	[RbromiL2-63]L2-63_01567_L2-63_01567_DNA_repair_protein_RadA,RecA-superfamily_ATPases_implicated_in_signal_transduction,DNA_repair_protein_I	RbromiL2-63
Org5_Gene1525	core	1789	[RbromiL2-63]L2-63_01569_map_2_Methionine_aminopeptidase,methionine_aminopeptidase,Xaa-Pro_aminopeptidase,methionine_aminopeptidase,_typ	RbromiL2-63
Org5_Gene1532	core	318	[RbromiL2-63]L2-63_01576_feoB_2_Ferrous_iron_transport_protein_B,ferrous_iron_transport_protein_B,Fe2__transport_system_protein_B,ferrous_iron_	RbromiL2-63
Org5_Gene1534	core	724	[RbromiL2-63]L2-63_01578_chuR_Anaerobic_sulfatase-maturating_enzyme,anaerobic_sulfatase-maturase,Predicted_metal-binding_protein,SCIFF_radical_	RbromiL2-63
Org5_Gene1535	core	4381	[RbromiL2-63]L2-63_01579_L2-63_01579_six-cysteine_peptide_SCIFF_1662107:1662259_reverse_MW:5447	RbromiL2-63
Org5_Gene1537	core	520	[RbromiL2-63]L2-63_01581_prfC_Peptide_chain_release_factor_3,peptide_chain_release_factor_3,EMAP_domain,peptide_chain_release_factor_3,Elonga	RbromiL2-63
Org5_Gene1550	core	4002	[RbromiL2-63]L2-63_01595_L2-63_01595_Helix-turn-helix_domain_1677280:1677528_reverse_MW:9181	RbromiL2-63
Org5_Gene1551	core	3252	[RbromiL2-63]L2-63_01596_rpiB_Ribose-5-phosphate_isomerase_B,ribose-5-phosphate_isomerase_B,sugar-phosphate_isomerase,_RpiB/LacA/LacB_famil	RbromiL2-63
Org5_Gene1553	core	3357	[RbromiL2-63]L2-63_01598_L2-63_01598_hypothetical_protein,ADP-binding_protein,ATPase,_YjeE_family,Uncharacterised_P-loop_hydrolase_UPF0079_1	RbromiL2-63
Org5_Gene1554	core	456	[RbromiL2-63]L2-63_01599_fhs_Formate--tetrahydrofolate_ligase,formate--tetrahydrofolate_ligase,Formate--tetrahydrofolate_ligase_1679500:1681167_r	RbromiL2-63
Org5_Gene1557	core	1734	[RbromiL2-63]L2-63_01602_L2-63_01602_conserved_hypothetical_protein,DisA_bacterial_checkpoint_controller_nucleotide-binding_1683791:1684681_r	RbromiL2-63
Org5_Gene156	core	408	[RbromiL2-63]L2-63_00159_argS_Arginine--tRNA_ligase,arginyl-tRNA_synthetase,Arginyl-tRNA_synthetase,arginine--tRNA_ligase,tRNA_synthetases_class_	RbromiL2-63
Org5_Gene1560	core	2158	[RbromiL2-63]L2-63_01605_dapB_Dihydrodipicolinate_reductase,dihydrodipicolinate_reductase,Dihydrodipicolinate_reductase,dihydrodipicolinate_reduc	RbromiL2-63
Org5_Gene1561	core	1686	[RbromiL2-63]L2-63_01606_dapA_Dihydrodipicolinate_synthase,dihydrodipicolinate_synthase,dihydrodipicolinate_synthase,Dihydrodipicolinate_syntheta	RbromiL2-63
Org5_Gene1562	core	1256	[RbromiL2-63]L2-63_01607_asd2_Aspartate-semialdehyde_dehydrogenase_2,aspartate-semialdehyde_dehydrogenase,Aspartate-semialdehyde_dehydrog	RbromiL2-63
Org5_Gene1563	core	1375	[RbromiL2-63]L2-63_01608_queA_S-adenosylmethionine:tRNA_ribosyltransferase-isomerase,S-adenosylmethionine:tRNA_ribosyltransferase-isomerase,S-	RbromiL2-63
Org5_Gene1564	core	1179	[RbromiL2-63]L2-63_01609_tgt_Queuine_tRNA-ribosyltransferase,queuine_tRNA-ribosyltransferase,queuine_tRNA-ribosyltransferase,Queuine_tRNA-ribos	RbromiL2-63
Org5_Gene157	core	3199	[RbromiL2-63]L2-63_00160_L2-63_00160_Uncharacterized_protein_conserved_in_bacteria,Domain_of_unknown_function_(DUF1934)_179317:179781_re	RbromiL2-63
Org5_Gene1575	core	869	[RbromiL2-63]L2-63_01620_L2-63_01620_hypothetical_protein_1703457:1703681_reverse_MW:8560	RbromiL2-63
Org5_Gene1577	core	3697	[RbromiL2-63]L2-63_01622_hisE_Phosphoribosyl-ATP_pyrophosphatase,phosphoribosyl-ATP_pyrophosphatase,Phosphoribosyl-AMP_cyclohydrolase,phos	RbromiL2-63
Org5_Gene1578	core	3785	[RbromiL2-63]L2-63_01623_L2-63_01623_phosphoribosyl-AMP_cyclohydrolase,Phosphoribosyl-AMP_cyclohydrolase,Phosphoribosyl-AMP_cyclohydrolase_	RbromiL2-63
Org5_Gene1579	core	2300	[RbromiL2-63]L2-63_01624_hisA_1-(5-phosphoribosyl)-5-[(5-phosphoribosylamino)methylideneamino]_imidazole-4-carboxamide_isomerase,1-(5-phospho	RbromiL2-63
Org5_Gene158	core	1978	[RbromiL2-63]L2-63_00161_murI_Glutamate_racemase,glutamate_racemase,Xanthosine_triphosphate_pyrophosphatase,glutamate_racemase,Asp/Glu/H	RbromiL2-63
Org5_Gene1580	core	2773	[RbromiL2-63]L2-63_01625_hisB_Imidazoleglycerol-phosphate_dehydratase,imidazoleglycerol-phosphate_dehydratase,Imidazoleglycerol-phosphate_dehy	RbromiL2-63
Org5_Gene1581	core	1314	[RbromiL2-63]L2-63_01626_hisC_Histidinol-phosphate_aminotransferase,histidinol-phosphate_aminotransferase,Histidinol_dehydrogenase,histidinol-pho	RbromiL2-63
Org5_Gene1582	core	864	[RbromiL2-63]L2-63_01627_hisD_Histidinol_dehydrogenase,bifunctional_histidinal_dehydrogenase/_histidinol_dehydrogenase,Histidinol_dehydrogenase,	RbromiL2-63
Org5_Gene1583	core	2601	[RbromiL2-63]L2-63_01628_hisG_ATP_phosphoribosyltransferase,ATP_phosphoribosyltransferase_catalytic_subunit,ATP_phosphoribosyltransferase,ATP_	RbromiL2-63
Org5_Gene1584	core	1098	[RbromiL2-63]L2-63_01629_hisZ_ATP_phosphoribosyltransferase_regulatory_subunit,ATP_phosphoribosyltransferase_regulatory_subunit,ATP_phosphori	RbromiL2-63
Org5_Gene1586	core	21	[RbromiL2-63]L2-63_01631_polC_DNA_polymerase_III_polC-type,DNA_polymerase_III_PolC,Histidinol_phosphatase_and_related_hydrolases_of_the_PHP	RbromiL2-63
Org5_Gene1587	core	1335	[RbromiL2-63]L2-63_01632_ispG_4-hydroxy-3-methylbut-2-en-1-yl_diphosphate_synthase,4-hydroxy-3-methylbut-2-en-1-yl_diphosphate_synthase,4-hydr	RbromiL2-63
Org5_Gene1589	core	1147	[RbromiL2-63]L2-63_01634_dxr_1-deoxy-D-xylulose_5-phosphate_reductoisomerase,1-deoxy-D-xylulose_5-phosphate_reductoisomerase,Translation_elon	RbromiL2-63
Org5_Gene159	core	2067	[RbromiL2-63]L2-63_00162_L2-63_00162_hypothetical_protein_180750:181538_forward_MW:29974	RbromiL2-63
Org5_Gene1591	core	2189	[RbromiL2-63]L2-63_01636_uppS_Undecaprenyl_pyrophosphate_synthase,undecaprenyl_pyrophosphate_synthase,Undecaprenyl_pyrophosphate_synthas	RbromiL2-63
Org5_Gene1592	core	2866	[RbromiL2-63]L2-63_01637_frr_Ribosome-releasing_factor,ribosome_recycling_factor,Translation_elongation_factor_Ts,ribosome_recycling_factor,Riboso	RbromiL2-63
Org5_Gene1593	core	2319	[RbromiL2-63]L2-63_01638_pyrH_Uridylate_kinase,uridylate_kinase,Uridylate_kinase,UMP_kinase,Amino_acid_kinase_family_1722988:1723701_reverse_	RbromiL2-63
Org5_Gene1597	core	2509	[RbromiL2-63]L2-63_01643_L2-63_01643_Uncharacterized_protein_conserved_in_bacteria,radical_SAM-linked_protein,Uncharacterized_protein_conserve	RbromiL2-63
Org5_Gene1598	core	346	[RbromiL2-63]L2-63_01644_L2-63_01644_radical_SAM_family_uncharacterized_protein,Radical_SAM_superfamily_1727411:1729267_reverse_MW:70352	RbromiL2-63
Org5_Gene16	core	319	[RbromiL2-63]L2-63_00016_yicI_Alpha-xylosidase,putative_alpha-glucosidase,Glycosyl_hydrolases_family_31_21008:22912_forward_MW:72213	RbromiL2-63
Org5_Gene160	core	4393	[RbromiL2-63]L2-63_00163_rpmG_50S_ribosomal_protein_L33,50S_ribosomal_protein_L33,Ribosomal_protein_L33,ribosomal_protein_L33,Ribosomal_pr	RbromiL2-63
Org5_Gene1601	core	690	[RbromiL2-63]L2-63_01647_mepA_9_Multidrug_export_protein_mepA,multidrug_efflux_pump_VmrA,MATE_efflux_family_protein,MatE_1730295:173167	RbromiL2-63
Org5_Gene1606	core	517	[RbromiL2-63]L2-63_01652_L2-63_01652_hypothetical_protein,Uncharacterized_conserved_protein,flavoprotein,_HI0933_family,FAD_dependent_oxidore	RbromiL2-63
Org5_Gene1610	core	1085	[RbromiL2-63]L2-63_01657_maeB_2_NADP-dependent_malic_enzyme,bifunctional_malic_enzyme_oxidoreductase/phosphotransacetylase,Malic_enzyme,	RbromiL2-63
Org5_Gene1612	core	2223	[RbromiL2-63]L2-63_01659_artM_Arginine_transport_ATP-binding_protein_ArtM,glutamine_ABC_transporter_ATP-binding_protein,ABC-type_histidine_tr	RbromiL2-63
Org5_Gene1616	core	2136	[RbromiL2-63]L2-63_01663_spo0A_Stage_0_sporulation_protein_G,chemotaxis-specific_methylesterase,Response_regulator_of_citrate/malate_metabolis	RbromiL2-63
Org5_Gene1617	core	914	[RbromiL2-63]L2-63_01664_purD_Phosphoribosylamine--glycine_ligase,phosphoribosylamine--glycine_ligase,Phosphoribosylamine-glycine_ligase,phospho	RbromiL2-63
Org5_Gene1618	core	1076	[RbromiL2-63]L2-63_01665_purH_Bifunctional_purine_biosynthesis_protein_PurH,5-aminoimidazole-4-carboxamide_ribonucleotide_transformylase,phos	RbromiL2-63
Org5_Gene1619	core	2308	[RbromiL2-63]L2-63_01666_L2-63_01666_IMP_cyclohydrolase,IMP_cyclohydrolase,IMP_cyclohydrolase-like_protein_1752328:1753044_reverse_MW:2648	RbromiL2-63
Org5_Gene162	core	2971	[RbromiL2-63]L2-63_00165_nusG_Transcription_antitermination_protein_nusG,transcription_antitermination_protein_NusG,transcription_termination/an	RbromiL2-63
Org5_Gene1620	core	2614	[RbromiL2-63]L2-63_01667_purN_Phosphoribosylglycinamide_formyltransferase,phosphoribosylglycinamide_formyltransferase,Folate-dependent_phosph	RbromiL2-63
Org5_Gene1621	core	1351	[RbromiL2-63]L2-63_01668_purM_Phosphoribosylformylglycinamidine_cyclo-ligase,phosphoribosylaminoimidazole_synthetase,Phosphoribosylamine-glyci	RbromiL2-63
Org5_Gene1622	core	633	[RbromiL2-63]L2-63_01669_purF_2_Amidophosphoribosyltransferase_precursor,amidophosphoribosyltransferase,Glutamine_phosphoribosylpyrophospha	RbromiL2-63
Org5_Gene1623	core	3072	[RbromiL2-63]L2-63_01670_purE_N5-carboxyaminoimidazole_ribonucleotide_mutase,Phosphoribosylcarboxyaminoimidazole_(NCAIR)_mutase,phosphori	RbromiL2-63
Org5_Gene1625	core	1537	[RbromiL2-63]L2-63_01672_qmcA_hypothetical_protein,FtsH_protease_regulator_HflK,Uncharacterized_protein_conserved_in_bacteria,HflK_protein,SPF	RbromiL2-63
Org5_Gene163	core	3352	[RbromiL2-63]L2-63_00166_rplK_50S_ribosomal_protein_L11,50S_ribosomal_protein_L11,ribosomal_protein_L11,Ribosomal_protein_L11,_N-terminal_do	RbromiL2-63
Org5_Gene1635	core	1288	[RbromiL2-63]L2-63_01682_mro_Aldose_1-epimerase_precursor,galactose-1-epimerase,galactose_mutarotase,Aldose_1-epimerase_1770749:1771816_re	RbromiL2-63
Org5_Gene1636	core	572	[RbromiL2-63]L2-63_01683_L2-63_01683_galactose-1-phosphate_uridylyltransferase,Galactose-1-phosphate_uridyltransferase,galactose-1-phosphate_uri	RbromiL2-63
Org5_Gene1637	core	1440	[RbromiL2-63]L2-63_01684_galE_2_UDP-glucose_4-epimerase,UDP-galactose-4-epimerase,Putative_NADH-flavin_reductase,UDP-glucose_4-epimerase,NA	RbromiL2-63
Org5_Gene164	core	2398	[RbromiL2-63]L2-63_00167_rplA_50S_ribosomal_protein_L1,50S_ribosomal_protein_L1,ribosomal_protein_L1,Ribosomal_protein_L1p/L10e_family_18333	RbromiL2-63
Org5_Gene1645	core	536	[RbromiL2-63]L2-63_01692_guaA_GMP_synthase_[glutamine-hydrolyzing],GMP_synthase,3-methyladenine_DNA_glycosylase,GMP_synthase_(glutamine-	RbromiL2-63
Org5_Gene1646	core	894	[RbromiL2-63]L2-63_01693_purA_Adenylosuccinate_synthetase,adenylosuccinate_synthetase,adenylosuccinate_synthase,Adenylosuccinate_synthetase_1	RbromiL2-63
Org5_Gene1647	core	647	[RbromiL2-63]L2-63_01694_purB_Adenylosuccinate_lyase,adenylosuccinate_lyase,Adenylosuccinate_lyase,adenylosuccinate_lyase,Lyase_1785193:178662	RbromiL2-63
Org5_Gene1648	core	613	[RbromiL2-63]L2-63_01695_purF_3_Amidophosphoribosyltransferase_precursor,amidophosphoribosyltransferase,Glutamine_phosphoribosylpyrophospha	RbromiL2-63
Org5_Gene1649	core	2350	[RbromiL2-63]L2-63_01696_purC_Phosphoribosylaminoimidazole-succinocarboxamide_synthase,phosphoribosylaminoimidazole-succinocarboxamide_synt	RbromiL2-63
Org5_Gene165	core	2838	[RbromiL2-63]L2-63_00169_rplJ_50S_ribosomal_protein_L10,50S_ribosomal_protein_L10,Ribosomal_protein_L10,Ribosomal_protein_L10_184228:184788	RbromiL2-63
Org5_Gene166	core	3530	[RbromiL2-63]L2-63_00170_rplL_MA1/MA2,50S_ribosomal_protein_L7/L12,ribosomal_protein_L7/L12,Ribosomal_protein_L7/L12_C-terminal_domain_18	RbromiL2-63
Org5_Gene167	core	510	[RbromiL2-63]L2-63_00171_pckA_Phosphoenolpyruvate_carboxykinase_[ATP],phosphoenolpyruvate_carboxykinase,Phosphoenolpyruvate_carboxykinase_	RbromiL2-63
Org5_Gene168	core	3338	[RbromiL2-63]L2-63_00172_L2-63_00172_hypothetical_protein_187245:187673_reverse_MW:16126	RbromiL2-63
Org5_Gene1701	core	3230	[RbromiL2-63]L2-63_01749_tadA_tRNA-specific_adenosine_deaminase,tRNA-specific_adenosine_deaminase,Pyrimidine_deaminase,riboflavin_biosynthesi	RbromiL2-63
Org5_Gene1702	core	902	[RbromiL2-63]L2-63_01750_L2-63_01750_cystathionine_gamma-synthase,Cystathionine_beta-lyase_family_protein_involved_in_aluminum_resistance,Alu	RbromiL2-63
Org5_Gene1706	core	702	[RbromiL2-63]L2-63_01754_murD_UDP-N-acetylmuramoylalanine--D-glutamate_ligase,UDP-N-acetylmuramoyl-L-alanyl-D-glutamate_synthetase,UDP-N-ac	RbromiL2-63
Org5_Gene171	core	2814	[RbromiL2-63]L2-63_00175_pduL_Phosphate_propanoyltransferase,propanediol_utilization_phosphotransacylase,Propanediol_utilization_protein,Propane	RbromiL2-63
Org5_Gene1711	core	191	[RbromiL2-63]L2-63_01759_pcrA_ATP-dependent_DNA_helicase_pcrA,DNA-dependent_helicase_II,Superfamily_I_DNA_and_RNA_helicases,ATP-depender	RbromiL2-63
Org5_Gene1717	core	1002	[RbromiL2-63]L2-63_01767_L2-63_01767_cofactor-independent_phosphoglycerate_mutase,Predicted_phosphoglycerate_mutase,_AP_superfamily,propos	RbromiL2-63
Org5_Gene1718	core	1201	[RbromiL2-63]L2-63_01768_prfB_Peptide_chain_release_factor_2,peptide_chain_release_factor_2,peptide_chain_release_factor_2,RF-1_domain_185217	RbromiL2-63
Org5_Gene172	core	2781	[RbromiL2-63]L2-63_00176_L2-63_00176_uracil-DNA_glycosylase,_family_4,Uracil_DNA_glycosylase_superfamily_189865:190440_forward_MW:21936	RbromiL2-63
Org5_Gene1720	core	1775	[RbromiL2-63]L2-63_01770_gtaB_UTP--glucose-1-phosphate_uridylyltransferase,UTP--glucose-1-phosphate_uridylyltransferase_subunit_GalU,CTP:phosph	RbromiL2-63
Org5_Gene1722	core	3947	[RbromiL2-63]L2-63_01772_L2-63_01772_hypothetical_protein,hypothetical_protein,ACT_domain-containing_protein,ACT_domain_1856594:1856863_for	RbromiL2-63
Org5_Gene1723	core	744	[RbromiL2-63]L2-63_01773_L2-63_01773_hypothetical_protein,hypothetical_protein,Uncharacterized_conserved_protein,Uncharacterized_ACR_(DUF711)	RbromiL2-63
Org5_Gene1725	core	1355	[RbromiL2-63]L2-63_01775_L2-63_01775_V-type_ATP_synthase_subunit_C,ATP_synthase_A1,_C_subunit,ATP_synthase_(C/AC39)_subunit_1858629:1859	RbromiL2-63
Org5_Gene1727	core	3337	[RbromiL2-63]L2-63_01777_L2-63_01777_V-type_ATP_synthase_subunit_K,ATP_synthase_subunit_C_1861652:1862077_forward_MW:14100	RbromiL2-63
Org5_Gene1728	core	3802	[RbromiL2-63]L2-63_01778_L2-63_01778_V-type_ATP_synthase_subunit_F,ATP_synthase_(F/14-kDa)_subunit_1862114:1862422_forward_MW:11540	RbromiL2-63
Org5_Gene1730	core	393	[RbromiL2-63]L2-63_01780_atpA_V-type_ATP_synthase_alpha_chain,V-type_ATP_synthase_subunit_A,F0F1-type_ATP_synthase,_beta_subunit,ATP_synth	RbromiL2-63
Org5_Gene1731	core	716	[RbromiL2-63]L2-63_01781_ntpB_2_V-type_sodium_pump_subunit_B,V-type_ATP_synthase_subunit_B,F0F1-type_ATP_synthase,_beta_subunit,ATP_synt	RbromiL2-63
Org5_Gene1732	core	2499	[RbromiL2-63]L2-63_01782_ntpD_2_V-type_sodium_pump_subunit_D,V-type_ATP_synthase_subunit_D,V-type_ATPase,_D_subunit,ATP_synthase_subuni	RbromiL2-63
Org5_Gene1734	core	3740	[RbromiL2-63]L2-63_01784_L2-63_01784_4-carboxymuconolactone_decarboxylase,Carboxymuconolactone_decarboxylase_family_1867461:1867784_reve	RbromiL2-63
Org5_Gene1737	core	1369	[RbromiL2-63]L2-63_01791_ilvE_Branched-chain-amino-acid_aminotransferase,branched-chain_amino_acid_aminotransferase,Branched-chain_amino_aci	RbromiL2-63
Org5_Gene1743	core	2031	[RbromiL2-63]L2-63_01797_potA_1_Spermidine/putrescine_import_ATP-binding_protein_PotA,putrescine/spermidine_ABC_transporter_ATPase_protein,	RbromiL2-63
Org5_Gene1744	core	1485	[RbromiL2-63]L2-63_01798_L2-63_01798_autoinducer_2_ABC_transporter_permease_LsrC,ABC-type_uncharacterized_transport_system,_permease_com	RbromiL2-63
Org5_Gene1746	core	52	[RbromiL2-63]L2-63_01800_dnaE_DNA_polymerase_III_subunit_alpha,DNA_polymerase_III_DnaE,Histidinol_phosphatase_and_related_hydrolases_of_the	RbromiL2-63
Org5_Gene1748	core	1552	[RbromiL2-63]L2-63_01802_gcp_2_t(6)A37_threonylcarbamoyladenosine_biosynthesis_protein,UGMP_family_protein,putative_glycoprotease_GCP,Glyco	RbromiL2-63
Org5_Gene175	core	399	[RbromiL2-63]L2-63_00179_aspS_1_Aspartate--tRNA_ligase,aspartyl-tRNA_synthetase,Aspartyl/asparaginyl-tRNA_synthetases,aspartate--tRNA_ligase,tRN	RbromiL2-63
Org5_Gene1751	core	3249	[RbromiL2-63]L2-63_01805_L2-63_01805_stage_III_sporulation_protein_AG_1889807:1890256_reverse_MW:16203	RbromiL2-63
Org5_Gene1755	core	4250	[RbromiL2-63]L2-63_01809_L2-63_01809_stage_III_sporulation_protein_AC,Stage_III_sporulation_protein_AC/AD_protein_family_1892249:1892443_reve	RbromiL2-63
Org5_Gene1759	core	283	[RbromiL2-63]L2-63_01813_ligA_DNA_ligase,NAD-dependent_DNA_ligase_LigA,3-methyladenine_DNA_glycosylase,DNA_ligase,_NAD-dependent,NAD-dep	RbromiL2-63

Org5_Gene176	core	3910	[RbromiL2-63]L2-63_00180_gatC_Aspartyl/glutamyl-tRNA(Asn/Gln)_amidotransferase_subunit_C,aspartyl/glutamyl-tRNA_amidotransferase_subunit_C,as	RbromiL2-63
Org5_Gene1760	core	2771	[RbromiL2-63]L2-63_01814_L2-63_01814_Predicted_HD-superfamily_hydrolase_1896601:1897179_reverse_MW:22410	RbromiL2-63
Org5_Gene1762	core	1128	[RbromiL2-63]L2-63_01816_rlmL_1_Ribosomal_RNA_large_subunit_methyltransferase_L,23S_rRNA_m(2)G2445_methyltransferase,Type_I_restriction-mo	RbromiL2-63
Org5_Gene1763	core	2936	[RbromiL2-63]L2-63_01817_yvyD_SigL_modulation_protein,ribosome_hibernation_promoting_factor_HPF,ribosomal_subunit_interface_protein,Sigma_54	RbromiL2-63
Org5_Gene1764	core	3096	[RbromiL2-63]L2-63_01818_yugI_General_stress_protein_13,hypothetical_protein,polyribonucleotide_nucleotidyltransferase,S1_RNA_binding_domain_1	RbromiL2-63
Org5_Gene1766	core	3939	[RbromiL2-63]L2-63_01820_L2-63_01820_sporulation_protein_YabP,YabP_family_1901459:1901731_reverse_MW:9788	RbromiL2-63
Org5_Gene1767	core	4042	[RbromiL2-63]L2-63_01821_L2-63_01821_hypothetical_protein,ribosome-associated_heat_shock_protein_Hsp15,S4_domain_1902313:1902555_reverse_	RbromiL2-63
Org5_Gene1768	core	3918	[RbromiL2-63]L2-63_01822_hupA_HB,transcriptional_regulator_HU_subunit_alpha,integration_host_factor,_beta_subunit,Bacterial_DNA-binding_protein_	RbromiL2-63
Org5_Gene1769	core	2036	[RbromiL2-63]L2-63_01823_mazG_Nucleoside_triphosphate_pyrophosphohydrolase,nucleoside_triphosphate_pyrophosphohydrolase,MazG_family_protei	RbromiL2-63
Org5_Gene177	core	628	[RbromiL2-63]L2-63_00181_gatA_Glutamyl-tRNA(Gln)_amidotransferase_subunit_A,aspartyl/glutamyl-tRNA_amidotransferase_subunit_A,Allophanate_hy	RbromiL2-63
Org5_Gene1772	core	624	[RbromiL2-63]L2-63_01826_hemZ_Oxygen-independent_coproporphyrinogen-III_oxidase_2,coproporphyrinogen_III_oxidase,Putative_heme_iron_utilizati	RbromiL2-63
Org5_Gene1773	core	2613	[RbromiL2-63]L2-63_01827_L2-63_01827_hypothetical_protein,hydroxyacylglutathione_hydrolase,hydroxyacylglutathione_hydrolase,Metallo-beta-lactama	RbromiL2-63
Org5_Gene1774	core	3237	[RbromiL2-63]L2-63_01828_dtd_D-tyrosyl-tRNA(Tyr)_deacylase,D-tyrosyl-tRNA(Tyr)_deacylase,D-tyrosyl-tRNA(Tyr)_deacylase,D-Tyr-tRNA(Tyr)_deacylase_	RbromiL2-63
Org5_Gene1775	core	212	[RbromiL2-63]L2-63_01829_relA_GTP_pyrophosphokinase,bifunctional_(p)ppGpp_synthetase_II/_guanosine-3',5'-bis_pyrophosphate_3'-pyrophosphohydr	RbromiL2-63
Org5_Gene1777	core	457	[RbromiL2-63]L2-63_01831_recN_Recombination_protein_N,recombination_and_repair_protein,Predicted_ATPase,DNA_repair_protein_RecN,RecF/RecN/	RbromiL2-63
Org5_Gene178	core	654	[RbromiL2-63]L2-63_00182_gatB_Aspartyl/glutamyl-tRNA(Asn/Gln)_amidotransferase_subunit_B,aspartyl/glutamyl-tRNA_amidotransferase_subunit_B,as	RbromiL2-63
Org5_Gene1780	core	2015	[RbromiL2-63]L2-63_01834_tlyA_16S/23S_rRNA_(cytidine-2'-O)-methyltransferase_TlyA,30S_ribosomal_protein_S4,hemolysin_TlyA_family_protein,FtsJ-lik	RbromiL2-63
Org5_Gene1781	core	340	[RbromiL2-63]L2-63_01835_dxs_3_1-deoxy-D-xylulose-5-phosphate_synthase,1-deoxy-D-xylulose-5-phosphate_synthase,Transketolase,_C-terminal_subun	RbromiL2-63
Org5_Gene1782	core	1774	[RbromiL2-63]L2-63_01836_L2-63_01836_Farnesyl_diphosphate_synthase,geranyltranstransferase,Geranylgeranyl_pyrophosphate_synthase,heptaprenyl_	RbromiL2-63
Org5_Gene1784	core	1590	[RbromiL2-63]L2-63_01838_whiA_Putative_sporulation_transcription_regulator_WhiA,Uncharacterized_protein_conserved_in_bacteria,conserved_hypoth	RbromiL2-63
Org5_Gene1785	core	1818	[RbromiL2-63]L2-63_01839_L2-63_01839_hypothetical_protein,glmZ(sRNA)-inactivating_NTPase,Predicted_P-loop-containing_kinase,P-loop_ATPase_prot	RbromiL2-63
Org5_Gene1786	core	1679	[RbromiL2-63]L2-63_01840_murB_UDP-N-acetylenolpyruvoylglucosamine_reductase,UDP-N-acetylenolpyruvoylglucosamine_reductase,UDP-N-acetylmura	RbromiL2-63
Org5_Gene1787	core	1495	[RbromiL2-63]L2-63_01841_hprK_HPr_kinase/phosphorylase,HPr_kinase/phosphorylase,HPr(Ser)_kinase/phosphatase,HPr_Serine_kinase_C-terminal_dom	RbromiL2-63
Org5_Gene1790	core	622	[RbromiL2-63]L2-63_01844_L2-63_01844_Bacillus/Clostridium_GerA_spore_germination_protein_1925442:1926896_reverse_MW:53679	RbromiL2-63
Org5_Gene1796	core	1378	[RbromiL2-63]L2-63_01850_secDF_1_hypothetical_protein,preprotein_translocase_subunit_SecF,protein-export_membrane_protein_SecF,Protein_export_	RbromiL2-63
Org5_Gene1797	core	663	[RbromiL2-63]L2-63_01851_secDF_2_hypothetical_protein,preprotein_translocase_subunit_SecD,protein-export_membrane_protein_SecD,Protein_export	RbromiL2-63
Org5_Gene1798	core	886	[RbromiL2-63]L2-63_01852_L2-63_01852_Putative_aminotransferase_MSMEG_6286,Alanine-glyoxylate_amino-transferase_1933405:1934685_reverse_M	RbromiL2-63
Org5_Gene18	core	578	[RbromiL2-63]L2-63_00018_algC_Phosphomannomutase/phosphoglucomutase,phosphomannomutase/phosphoglucomutase,phosphoglucosamine_mutas	RbromiL2-63
Org5_Gene1801	core	956	[RbromiL2-63]L2-63_01855_gcdB_Glutaconyl-CoA_decarboxylase_subunit_beta,oxaloacetate_decarboxylase_subunit_beta,Na_-transporting_methylmalor	RbromiL2-63
Org5_Gene1803	core	603	[RbromiL2-63]L2-63_01857_spoIVA_Stage_IV_sporulation_protein_A,stage_IV_sporulation_protein_A,Stage_IV_sporulation_protein_A_(spore_IV_A)_193	RbromiL2-63
Org5_Gene1806	core	1509	[RbromiL2-63]L2-63_01860_rluC_Ribosomal_large_subunit_pseudouridine_synthase_C,23S_rRNA_pseudouridylate_synthase_C,pseudouridine_synthase,_	RbromiL2-63
Org5_Gene1809	core	1923	[RbromiL2-63]L2-63_01863_L2-63_01863_Laccase_domain_protein_SAV1187,hypothetical_protein,uncharacterized_protein,_YfiH_family,Multi-copper_po	RbromiL2-63
Org5_Gene181	core	717	[RbromiL2-63]L2-63_00185_trpB_1_Tryptophan_synthase_beta_chain,tryptophan_synthase_subunit_beta,Predicted_alternative_tryptophan_synthase_be	RbromiL2-63
Org5_Gene1811	core	2927	[RbromiL2-63]L2-63_01865_ppa_Inorganic_pyrophosphatase,inorganic_pyrophosphatase,Inorganic_pyrophosphatase,Inorganic_pyrophosphatase_194751	RbromiL2-63
Org5_Gene1814	core	124	[RbromiL2-63]L2-63_01868_L2-63_01868_Calcium-transporting_ATPase_lmo0841,magnesium-transporting_ATPase_MgtA,Uncharacterized_protein_conse	RbromiL2-63
Org5_Gene1816	core	1855	[RbromiL2-63]L2-63_01871_rsmA_Ribosomal_RNA_small_subunit_methyltransferase_A,16S_ribosomal_RNA_methyltransferase_KsgA/Dim1_family_prote	RbromiL2-63
Org5_Gene1817	core	818	[RbromiL2-63]L2-63_01872_rsmF_Ribosomal_RNA_small_subunit_methyltransferase_F,rRNA_(cytosine-C(5)-)-methyltransferase_RsmF,tRNA_and_rRNA_c	RbromiL2-63
Org5_Gene1819	core	2500	[RbromiL2-63]L2-63_01874_L2-63_01874_Uncharacterised_ACR,_YkgG_family_COG1556_1954368:1955030_reverse_MW:24746	RbromiL2-63
Org5_Gene1820	core	2897	[RbromiL2-63]L2-63_01875_L2-63_01875_N-acetylmuramoyl-L-alanine_amidase_CwlD,N-acetylmuramoyl-L-alanine_amidase_1955314:1955856_forward_	RbromiL2-63
Org5_Gene1828	core	1105	[RbromiL2-63]L2-63_01883_fprA1_H(2)O-forming_NADH_oxidase,metallo-beta-lactamase/flavodoxin_domain-containing_protein,Rubredoxin,flavodoxin,N	RbromiL2-63
Org5_Gene1831	core	207	[RbromiL2-63]L2-63_01886_pnp_Polyribonucleotide_nucleotidyltransferase,polynucleotide_phosphorylase/polyadenylase,RNase_PH-related_exoribonucle	RbromiL2-63
Org5_Gene1832	core	3962	[RbromiL2-63]L2-63_01887_rpsO_BS18,30S_ribosomal_protein_S15,ribosomal_protein_S15,Ribosomal_protein_S15_1968987:1969250_reverse_MW:1010	RbromiL2-63
Org5_Gene1835	core	746	[RbromiL2-63]L2-63_01891_L2-63_01891_Maltose-binding_periplasmic_proteins/domains_1971689:1973047_forward_MW:49231	RbromiL2-63
Org5_Gene1836	core	1769	[RbromiL2-63]L2-63_01892_ugpA_sn-glycerol-3-phosphate_transport_system_permease_protein_ugpA,glycerol-3-phosphate_transporter_permease,ABC-	RbromiL2-63
Org5_Gene1837	core	1768	[RbromiL2-63]L2-63_01893_ycjP_Inner_membrane_ABC_transporter_permease_protein_ycjP,glycerol-3-phosphate_transporter_membrane_protein,ABC-t	RbromiL2-63
Org5_Gene1838	core	174	[RbromiL2-63]L2-63_01894_clpE_Heat_shock_protein_HSP1,protein_disaggregation_chaperone,Predicted_ATPase,ATP-dependent_chaperone_protein_Cl	RbromiL2-63
Org5_Gene1842	core	2028	[RbromiL2-63]L2-63_01898_truA_tRNA_pseudouridine_synthase_A,tRNA_pseudouridine_synthase_A,tRNA_pseudouridine(38-40)_synthase,tRNA_pseudo	RbromiL2-63
Org5_Gene1843	core	1998	[RbromiL2-63]L2-63_01899_ecfT_2_Energy-coupling_factor_transporter_transmembrane_protein_EcfT,ABC-type_cobalt_transport_system,_permease_co	RbromiL2-63
Org5_Gene1844	core	1805	[RbromiL2-63]L2-63_01900_ecfA2_Energy-coupling_factor_transporter_ATP-binding_protein_EcfA2,cobalt_transporter_ATP-binding_subunit,ABC-type_sp	RbromiL2-63
Org5_Gene1845	core	1885	[RbromiL2-63]L2-63_01901_ecfA1_Energy-coupling_factor_transporter_ATP-binding_protein_EcfA1,cobalt_transporter_ATP-binding_subunit,ABC-type_sp	RbromiL2-63
Org5_Gene1846	core	1821	[RbromiL2-63]L2-63_01902_rlmL_2_Ribosomal_RNA_large_subunit_methyltransferase_L,23S_rRNA_m(2)G2445_methyltransferase,protein-(glutamine-N5	RbromiL2-63
Org5_Gene1847	core	761	[RbromiL2-63]L2-63_01903_glmM_Phosphoglucosamine_mutase,phosphoglucosamine_mutase,Predicted_glutamine_amidotransferase,phosphoglucosami	RbromiL2-63
Org5_Gene1848	core	1247	[RbromiL2-63]L2-63_01904_ruvB_Holliday_junction_ATP-dependent_DNA_helicase_RuvB,Holliday_junction_DNA_helicase_RuvB,Holliday_junction_resolv	RbromiL2-63
Org5_Gene1849	core	2683	[RbromiL2-63]L2-63_01905_ruvA_Holliday_junction_ATP-dependent_DNA_helicase_RuvA,Holliday_junction_DNA_helicase_RuvA,Holliday_junction_DNA_	RbromiL2-63
Org5_Gene185	core	2184	[RbromiL2-63]L2-63_00189_pstB3_Phosphate_import_ATP-binding_protein_PstB_3,phosphate_transporter_ATP-binding_protein,ABC-type_spermidine/p	RbromiL2-63
Org5_Gene1850	core	3035	[RbromiL2-63]L2-63_01906_ruvC_Crossover_junction_endodeoxyribonuclease_RuvC,Holliday_junction_resolvase,crossover_junction_endodeoxyribonucle	RbromiL2-63
Org5_Gene1852	core	1408	[RbromiL2-63]L2-63_01908_L2-63_01908_Protein_of_unknown_function_(DUF2804)_1989052:1990059_forward_MW:38251	RbromiL2-63
Org5_Gene1854	core	435	[RbromiL2-63]L2-63_01910_nplT_Neopullulanase,maltodextrin_glucosidase,Uncharacterized_protein_conserved_in_bacteria,alpha,alpha-phosphotrehalas	RbromiL2-63
Org5_Gene1855	core	251	[RbromiL2-63]L2-63_01911_glgX_Glycogen_debranching_enzyme,glycogen_debranching_enzyme,glycogen_debranching_enzyme_GlgX,Alpha_amylase,_ca	RbromiL2-63
Org5_Gene1861	core	1118	[RbromiL2-63]L2-63_01917_L2-63_01917_hypothetical_protein_2000057:2000278_forward_MW:8565	RbromiL2-63
Org5_Gene1876	core	3322	[RbromiL2-63]L2-63_01932_L2-63_01932_Cupin_domain_2011407:2011841_reverse_MW:16095	RbromiL2-63
Org5_Gene1878	core	3228	[RbromiL2-63]L2-63_01934_nrdR_Transcriptional_repressor_NrdR,transcriptional_regulator_NrdR,transcriptional_regulator_NrdR,ATP_cone_domain_201	RbromiL2-63
Org5_Gene1880	core	265	[RbromiL2-63]L2-63_01936_recG_ATP-dependent_DNA_helicase_recG,ATP-dependent_DNA_helicase_RecG,Superfamily_II_DNA/RNA_helicase_required_	RbromiL2-63
Org5_Gene1881	core	470	[RbromiL2-63]L2-63_01937_L2-63_01937_hypothetical_protein,dihydroxyacetone_kinase,DAK2_domain_fusion_protein_YloV,DAK2_domain_2016796:201	RbromiL2-63
Org5_Gene1882	core	2126	[RbromiL2-63]L2-63_01938_L2-63_01938_hypothetical_protein,metallophosphoesterase,_MG_246/BB_0505_family_2018664:2019437_reverse_MW:2800	RbromiL2-63
Org5_Gene1886	core	354	[RbromiL2-63]L2-63_01942_asnO_Asparagine_synthetase_[glutamine-hydrolyzing]_3,asparagine_synthetase_B,Asparagine_synthase_(glutamine-hydrolyzi	RbromiL2-63
Org5_Gene1890	core	3312	[RbromiL2-63]L2-63_01946_mscL_Large-conductance_mechanosensitive_channel,large-conductance_mechanosensitive_channel,large_conductance_mech	RbromiL2-63
Org5_Gene1891	core	979	[RbromiL2-63]L2-63_01947_tyrS_Tyrosine--tRNA_ligase,tyrosyl-tRNA_synthetase,Tyrosyl-tRNA_synthetase,tyrosine--tRNA_ligase,tRNA_synthetases_class_	RbromiL2-63
Org5_Gene1893	core	316	[RbromiL2-63]L2-63_01949_L2-63_01949_hypothetical_protein,uncharacterized_radical_SAM_protein_YgiQ,Radical_SAM_N-terminal_2030239:2032152_r	RbromiL2-63
Org5_Gene1895	core	1521	[RbromiL2-63]L2-63_01951_uppP_Undecaprenyl-diphosphatase,undecaprenyl_pyrophosphate_phosphatase,undecaprenyl-diphosphatase_UppP,Bacitracin	RbromiL2-63
Org5_Gene1896	core	1989	[RbromiL2-63]L2-63_01952_L2-63_01952_ATP-dependent_Clp_protease_proteolytic_subunit,signal_peptide_peptidase_SppA,_36K_type,Clp_protease_20	RbromiL2-63
Org5_Gene1897	core	830	[RbromiL2-63]L2-63_01953_L2-63_01953_hypothetical_protein,hypothetical_protein,Putative_Mg2__and_Co2__transporter_CorB,gliding_motility-associa	RbromiL2-63
Org5_Gene1899	core	2718	[RbromiL2-63]L2-63_01955_L2-63_01955_ribonuclease_M5_2038923:2039501_reverse_MW:21286	RbromiL2-63
Org5_Gene19	core	3957	[RbromiL2-63]L2-63_00019_L2-63_00019_hypothetical_protein_26115:26381_forward_MW:9641	RbromiL2-63
Org5_Gene1903	core	2935	[RbromiL2-63]L2-63_01959_purR_Pur_operon_repressor,adenine_phosphoribosyltransferase,pur_operon_repressor_PurR,Phosphoribosyl_transferase_do	RbromiL2-63
Org5_Gene1904	core	3658	[RbromiL2-63]L2-63_01960_L2-63_01960_HIT-like_protein_HI_0961,purine_nucleoside_phosphoramidase,HIT_domain_2044244:2044585_reverse_MW:1	RbromiL2-63
Org5_Gene1906	core	819	[RbromiL2-63]L2-63_01962_der_GTP-binding_protein_EngA,GTP-binding_protein_Der,Cytidylate_kinase,ribosome-associated_GTPase_EngA,GTPase_of_u	RbromiL2-63
Org5_Gene1908	core	4271	[RbromiL2-63]L2-63_01964_rpmF_50S_ribosomal_protein_L32,50S_ribosomal_protein_L32,ribosomal_protein_L32,Ribosomal_L32p_protein_family_2048	RbromiL2-63
Org5_Gene1909	core	3074	[RbromiL2-63]L2-63_01965_L2-63_01965_Uncharacterized_ACR,_COG1399_2048431:2048925_reverse_MW:18726	RbromiL2-63
Org5_Gene1916	core	1618	[RbromiL2-63]L2-63_01972_rpsA_30S_ribosomal_protein_S1,4-hydroxy-3-methylbut-2-enyl_diphosphate_reductase/S1_RNA-binding_domain_protein,ribo	RbromiL2-63
Org5_Gene1918	core	1871	[RbromiL2-63]L2-63_01974_L2-63_01974_HAD_phosphoserine_phosphatase-like_hydrolase,_family_IB,haloacid_dehalogenase-like_hydrolase_2056642:2	RbromiL2-63
Org5_Gene1925	core	1660	[RbromiL2-63]L2-63_01981_drrA_1_Daunorubicin/doxorubicin_resistance_ATP-binding_protein_DrrA,nodulation_ABC_transporter_NodI,ABC-type_Na__tr	RbromiL2-63
Org5_Gene193	core	449	[RbromiL2-63]L2-63_00197_hcp_Hydroxylamine_reductase,hybrid_cluster_protein,hydroxylamine_reductase,Prismane/CO_dehydrogenase_family_21495	RbromiL2-63
Org5_Gene1933	core	3710	[RbromiL2-63]L2-63_01989_L2-63_01989_hypothetical_protein_2070043:2070375_forward_MW:12605	RbromiL2-63
Org5_Gene1963	core	3221	[RbromiL2-63]L2-63_02020_smpB_SsrA-binding_protein,SsrA-binding_protein,SsrA-binding_protein,SmpB_protein_2100485:2100934_reverse_MW:17319	RbromiL2-63
Org5_Gene1964	core	2679	[RbromiL2-63]L2-63_02021_recR_Recombination_protein_RecR,recombination_protein_RecR,Recombinational_DNA_repair_protein_(RecF_pathway),reco	RbromiL2-63
Org5_Gene1965	core	3632	[RbromiL2-63]L2-63_02022_L2-63_02022_hypothetical_protein,hypothetical_protein,DNA-binding_protein,_YbaB/EbfC_family,Uncharacterised_BCR,_Yba	RbromiL2-63
Org5_Gene1969	core	2452	[RbromiL2-63]L2-63_02029_glmU_2_Bifunctional_protein_GlmU,bifunctional_N-acetylglucosamine-1-phosphate_uridyltransferase/glucosamine-1-phosph	RbromiL2-63
Org5_Gene1972	core	937	[RbromiL2-63]L2-63_02032_hflX_GTP-binding_protein_HflX,GTPase_HflX,Fe2__transport_system_protein_B,GTP-binding_protein_HflX,GTPase_of_unkno	RbromiL2-63
Org5_Gene1975	core	817	[RbromiL2-63]L2-63_02035_L2-63_02035_putative_bifunctional_UDP-N-acetylmuramoylalanyl-D-glutamate--2,6-diaminopimelate_ligase/UDP-N-acetylmu	RbromiL2-63
Org5_Gene1977	core	210	[RbromiL2-63]L2-63_02037_recD_Exodeoxyribonuclease_V_alpha_chain,exonuclease_V_subunit_alpha,helicase,_RecD/TraA_family,Viral_(Superfamily_1)_	RbromiL2-63
Org5_Gene1982	core	659	[RbromiL2-63]L2-63_02042_potA_2_Spermidine/putrescine_import_ATP-binding_protein_PotA,putrescine/spermidine_ABC_transporter_ATPase_protein,	RbromiL2-63
Org5_Gene1983	core	2069	[RbromiL2-63]L2-63_02043_potH_Putrescine_transport_system_permease_protein_PotH,putrescine_transporter_subunit:_membrane_component_of_AB	RbromiL2-63
Org5_Gene1984	core	1988	[RbromiL2-63]L2-63_02044_ydcV_Inner_membrane_ABC_transporter_permease_protein_ydcV,spermidine/putrescine_ABC_transporter_membrane_prot	RbromiL2-63
Org5_Gene1985	core	1251	[RbromiL2-63]L2-63_02045_potD_Spermidine/putrescine-binding_periplasmic_protein_precursor,spermidine/putrescine_ABC_transporter_periplasmic_su	RbromiL2-63
Org5_Gene1986	core	1968	[RbromiL2-63]L2-63_02046_L2-63_02046_ABC-2_type_transporter_2125406:2126227_forward_MW:31803	RbromiL2-63
Org5_Gene1987	core	2205	[RbromiL2-63]L2-63_02047_tagH_2_Teichoic_acids_export_ATP-binding_protein_TagH,teichoic_acids_export_protein_ATP-binding_subunit,ABC-type_Na_	RbromiL2-63
Org5_Gene20	core	3607	[RbromiL2-63]L2-63_00020_L2-63_00020_hypothetical_protein,Uncharacterized_protein_conserved_in_bacteria,Protein_of_unknown_function_(DUF1292	RbromiL2-63
Org5_Gene205	core	2103	[RbromiL2-63]L2-63_00209_sigF_1_Stage_II_sporulation_protein_AC,sporulation_sigma_factor_SigG,RNA_polymerase_sigma-G_factor,Sigma-70,_region_	RbromiL2-63
Org5_Gene206	core	3832	[RbromiL2-63]L2-63_00210_L2-63_00210_Uncharacterized_protein_conserved_in_bacteria,TrpR_homolog_YerC/YecD,Trp_repressor_protein_229372:229	RbromiL2-63
Org5_Gene2076	core	2912	[RbromiL2-63]L2-63_02138_rbr3A_NADH_peroxidase,Uncharacterized_conserved_protein,Rubrerythrin_2206260:2206799_forward_MW:19873	RbromiL2-63
Org5_Gene2078	core	2169	[RbromiL2-63]L2-63_02140_exoA_Exodeoxyribonuclease,exodeoxyribonuclease_III,Uncharacterized_protein_conserved_in_bacteria,exodeoxyribonuclease	RbromiL2-63
Org5_Gene208	core	1730	[RbromiL2-63]L2-63_00212_hslO_Heat_shock_protein_33_homolog,Hsp33-like_chaperonin,Phosphoenolpyruvate_carboxykinase_(ATP),Hsp33_protein_23	RbromiL2-63
Org5_Gene22	core	317	[RbromiL2-63]L2-63_00022_yheS_Uncharacterized_ABC_transporter_ATP-binding_protein_YheS,putative_ABC_transporter_ATP-binding_protein,ATP-dep	RbromiL2-63
Org5_Gene269	core	4026	[RbromiL2-63]L2-63_00274_L2-63_00274_hypothetical_protein_280864:281112_forward_MW:9333	RbromiL2-63
Org5_Gene275	core	2795	[RbromiL2-63]L2-63_00284_L2-63_00284_dipicolinate_synthase_subunit_B,Phosphopantothenoylcysteine_synthetase/decarboxylase,dipicolinic_acid_synt	RbromiL2-63
Org5_Gene280	core	905	[RbromiL2-63]L2-63_00289_mdeA_1_Methionine_gamma-lyase,O-acetylhomoserine_aminocarboxypropyltransferase,Protoheme_ferro-lyase_(ferrochelat	RbromiL2-63
Org5_Gene281	core	2362	[RbromiL2-63]L2-63_00290_ispD_2-C-methyl-D-erythritol_4-phosphate_cytidylyltransferase,2-C-methyl-D-erythritol_4-phosphate_cytidylyltransferase,2C-	RbromiL2-63
Org5_Gene282	core	3164	[RbromiL2-63]L2-63_00291_ispF_2-C-methyl-D-erythritol_2,4-cyclodiphosphate_synthase,2-C-methyl-D-erythritol_2,4-cyclodiphosphate_synthase,2C-met	RbromiL2-63
Org5_Gene283	core	1141	[RbromiL2-63]L2-63_00292_dacC_D-alanyl-D-alanine_carboxypeptidase_dacC_precursor,D-alanyl-D-alanine_carboxypeptidase,D-alanyl-D-alanine_carboxy	RbromiL2-63
Org5_Gene284	core	787	[RbromiL2-63]L2-63_00293_pgi_Glucose-6-phosphate_isomerase,glucose-6-phosphate_isomerase,Transaldolase,Phosphoglucose_isomerase_294561:2958	RbromiL2-63
Org5_Gene285	core	3391	[RbromiL2-63]L2-63_00294_L2-63_00294_hypothetical_protein_295934:296350_forward_MW:14790	RbromiL2-63
Org5_Gene294	core	446	[RbromiL2-63]L2-63_00311_leuA_1_2-isopropylmalate_synthase,2-isopropylmalate_synthase,Isopropylmalate/homocitrate/citramalate_synthases,2-isopr	RbromiL2-63
Org5_Gene297	core	243	[RbromiL2-63]L2-63_00314_ydgH_Putative_membrane_protein_ydgH,Predicted_exporter,transport_protein,MMPL_family_315615:317708_reverse_MW:	RbromiL2-63
Org5_Gene301	core	4046	[RbromiL2-63]L2-63_00318_L2-63_00318_transcriptional_regulator,_AbrB_family_319966:320202_reverse_MW:8844	RbromiL2-63
Org5_Gene302	core	2757	[RbromiL2-63]L2-63_00319_spmA_Spore_maturation_protein_A,Uncharacterized_membrane_protein,Nucleoside_recognition_320363:320947_forward_	RbromiL2-63
Org5_Gene303	core	3055	[RbromiL2-63]L2-63_00320_spmB_Spore_maturation_protein_B,hypothetical_protein,Uncharacterized_membrane_protein_320976:321476_forward_MW	RbromiL2-63
Org5_Gene309	core	310	[RbromiL2-63]L2-63_00326_lysS_Lysine--tRNA_ligase,lysyl-tRNA_synthetase,Aspartyl/asparaginyl-tRNA_synthetases,lysine--tRNA_ligase,tRNA_synthetase	RbromiL2-63
Org5_Gene310	core	3129	[RbromiL2-63]L2-63_00327_greA_Transcript_cleavage_factor_greA,transcription_elongation_factor_GreA,Transcription_elongation_factor,transcription_e	RbromiL2-63
Org5_Gene314	core	2132	[RbromiL2-63]L2-63_00331_ftsE_Cell_division_ATP-binding_protein_FtsE,DL-methionine_transporter_ATP-binding_subunit,ABC-type_phosphate/phospho	RbromiL2-63
Org5_Gene315	core	1258	[RbromiL2-63]L2-63_00332_cdaR_Sugar_diacid_regulator,carbohydrate_diacid_transcriptional_activator_CdaR,Sugar_diacid_utilization_regulator_333809	RbromiL2-63
Org5_Gene316	core	1217	[RbromiL2-63]L2-63_00333_ugpC_sn-glycerol-3-phosphate_import_ATP-binding_protein_UgpC,glycerol-3-phosphate_transporter_ATP-binding_subunit,A	RbromiL2-63

Org5_Gene320	core	3496	[RbromiL2-63]L2-63_00337_L2-63_00337_hypothetical_protein_338506:338895_reverse_MW:13818	RbromiL2-63
Org5_Gene321	core	1238	[RbromiL2-63]L2-63_00338_engD_GTP-dependent_nucleic_acid-binding_protein_engD,GTP-binding_protein_YchF,Predicted_GTPase,_probable_translatio	RbromiL2-63
Org5_Gene323	core	1554	[RbromiL2-63]L2-63_00340_dxs_1_1-deoxy-D-xylulose-5-phosphate_synthase,transketolase,Transketolase,_C-terminal_subunit,1-deoxy-D-xylulose-5-phos	RbromiL2-63
Org5_Gene324	core	1872	[RbromiL2-63]L2-63_00341_tkt_Transketolase,transketolase,Transketolase,_N-terminal_subunit,transketolase,Transketolase,_thiamine_diphosphate_bind	RbromiL2-63
Org5_Gene325	core	2140	[RbromiL2-63]L2-63_00342_coaX_Type_III_pantothenate_kinase,pantothenate_kinase,pantothenate_kinase,_type_III,Bordetella_pertussis_Bvg_accessory	RbromiL2-63
Org5_Gene333	core	37	[RbromiL2-63]L2-63_00350_purL_Phosphoribosylformylglycinamidine_synthase_2,phosphoribosylformylglycinamidine_synthase,Phosphoribosylformylglyc	RbromiL2-63
Org5_Gene335	core	1555	[RbromiL2-63]L2-63_00352_hprA_Glycerate_dehydrogenase,glycerate_dehydrogenase,Phosphoserine_aminotransferase,phosphoglycerate_dehydrogenas	RbromiL2-63
Org5_Gene342	core	675	[RbromiL2-63]L2-63_00361_apeA_Probable_M18_family_aminopeptidase_1,putative_aminopeptidase_1,Aminopeptidase_I_zinc_metalloprotease_(M18)_	RbromiL2-63
Org5_Gene345	core	1337	[RbromiL2-63]L2-63_00364_rimN_t(6)A37_threonylcarbamoyladenosine_biosynthesis_protein_RimN,tRNA(ANN)_t(6)A37_threonylcarbamoyladenosine_m	RbromiL2-63
Org5_Gene346	core	1276	[RbromiL2-63]L2-63_00365_prfA_Peptide_chain_release_factor_1,peptide_chain_release_factor_1,peptide_chain_release_factor_1,RF-1_domain_364013	RbromiL2-63
Org5_Gene347	core	4312	[RbromiL2-63]L2-63_00366_L2-63_00366_Bacterial_protein_of_unknown_function_(DUF951)_365095:365274_reverse_MW:6779	RbromiL2-63
Org5_Gene349	core	1671	[RbromiL2-63]L2-63_00368_rssA_NTE_family_protein_rssA,hypothetical_protein,Patatin,Patatin-like_phospholipase_366105:367013_reverse_MW:33021	RbromiL2-63
Org5_Gene350	core	416	[RbromiL2-63]L2-63_00369_L2-63_00369_Predicted_membrane_protein_367028:368746_reverse_MW:64890	RbromiL2-63
Org5_Gene351	core	189	[RbromiL2-63]L2-63_00370_glgB_1_1,4-alpha-glucan_branching_enzyme_GlgB,glycogen_branching_enzyme,Type_IV_secretory_pathway,_VirB10_compor	RbromiL2-63
Org5_Gene362	core	3350	[RbromiL2-63]L2-63_00382_yrrK_Putative_Holliday_junction_resolvase,Holliday_junction_resolvase-like_protein,RNAse_H_domain_protein,_YqgF_family,	RbromiL2-63
Org5_Gene364	core	44	[RbromiL2-63]L2-63_00384_rpoC_DNA-directed_RNA_polymerase_subunit_beta',DNA-directed_RNA_polymerase_subunit_beta',DNA-directed_RNA_polyr	RbromiL2-63
Org5_Gene365	core	40	[RbromiL2-63]L2-63_00385_rpoB_DNA-directed_RNA_polymerase_subunit_beta,DNA-directed_RNA_polymerase_subunit_beta,DNA-directed_RNA_polym	RbromiL2-63
Org5_Gene367	core	1129	[RbromiL2-63]L2-63_00387_ttcA_tRNA_2-thiocytidine_biosynthesis_protein_TtcA,tRNA_2-thiocytidine_biosynthesis_protein_TtcA,Predicted_ATPase_of_t	RbromiL2-63
Org5_Gene370	core	2195	[RbromiL2-63]L2-63_00390_L2-63_00390_serine/threonine_protein_kinase,fibrobacter_succinogenes_paralogous_family,Phosphotransferase_enzyme_far	RbromiL2-63
Org5_Gene371	core	123	[RbromiL2-63]L2-63_00391_adhE_1_Aldehyde-alcohol_dehydrogenase,bifunctional_acetaldehyde-CoA/alcohol_dehydrogenase,Glycerol_dehydrogenase_a	RbromiL2-63
Org5_Gene374	core	996	[RbromiL2-63]L2-63_00394_ackA_Acetate_kinase,acetate_kinase_A/propionate_kinase_2,Acetate_kinase,acetate_kinase,Acetokinase_family_404043:405	RbromiL2-63
Org5_Gene375	core	4093	[RbromiL2-63]L2-63_00395_L2-63_00395_Domain_of_unknown_function_(DUF378)_405436:405660_reverse_MW:7874	RbromiL2-63
Org5_Gene378	core	415	[RbromiL2-63]L2-63_00398_mutS2_1_MutS2_protein,DNA_mismatch_repair_protein_MutS,DNA_mismatch_repair_protein_MutS,MutS_domain_V_40794	RbromiL2-63
Org5_Gene379	core	4115	[RbromiL2-63]L2-63_00399_L2-63_00399_hypothetical_protein,hypothetical_protein,Uncharacterized_protein_conserved_in_bacteria,Bacterial_protein_o	RbromiL2-63
Org5_Gene382	core	56	[RbromiL2-63]L2-63_00402_mfd_Transcription-repair-coupling_factor,transcription-repair_coupling_factor,Superfamily_II_DNA/RNA_helicase_required_fc	RbromiL2-63
Org5_Gene383	core	2720	[RbromiL2-63]L2-63_00403_pth_Peptidyl-tRNA_hydrolase,peptidyl-tRNA_hydrolase,Peptidyl-tRNA_hydrolase,peptidyl-tRNA_hydrolase,Peptidyl-tRNA_hyd	RbromiL2-63
Org5_Gene385	core	1519	[RbromiL2-63]L2-63_00405_prs_Ribose-phosphate_pyrophosphokinase,ribose-phosphate_pyrophosphokinase,ribose-phosphate_diphosphokinase,Phosph	RbromiL2-63
Org5_Gene386	core	2182	[RbromiL2-63]L2-63_00406_glmU_1_Bifunctional_protein_GlmU,bifunctional_N-acetylglucosamine-1-phosphate_uridyltransferase/glucosamine-1-phosph	RbromiL2-63
Org5_Gene387	core	120	[RbromiL2-63]L2-63_00407_ppdK_Pyruvate,_phosphate_dikinase,pyruvate_phosphate_dikinase,Uncharacterized_protein_conserved_in_bacteria,pyruvate	RbromiL2-63
Org5_Gene403	core	1409	[RbromiL2-63]L2-63_00423_L2-63_00423_Predicted_phage_phi-C31_gp36_major_capsid-like_protein,phage_major_capsid_protein,_HK97_family,Phage_c	RbromiL2-63
Org5_Gene407	core	891	[RbromiL2-63]L2-63_00427_L2-63_00427_Bacteriophage_terminase_large_(ATPase)_subunit_and_inactivated_derivatives,phage_terminase,_large_subun	RbromiL2-63
Org5_Gene413	core	153	[RbromiL2-63]L2-63_00433_L2-63_00433_Beta/alpha-amylase_precursor,alpha-amylase,Maltooligosyl_trehalose_synthase,trehalose_synthase,Alpha_amy	RbromiL2-63
Org5_Gene415	core	913	[RbromiL2-63]L2-63_00435_rarA_Replication-associated_recombination_protein_A,recombination_factor_protein_RarA,Holliday_junction_resolvasome,_	RbromiL2-63
Org5_Gene417	core	1782	[RbromiL2-63]L2-63_00437_gpr_Germination_protease_precursor,germination_protease,GPR_endopeptidase,Germination_protease_448572:449447_forv	RbromiL2-63
Org5_Gene419	core	636	[RbromiL2-63]L2-63_00439_trkG_Trk_system_potassium_uptake_protein_trkG,potassium_transporter,potassium_uptake_protein,_TrkH_family,Cation_tra	RbromiL2-63
Org5_Gene421	core	700	[RbromiL2-63]L2-63_00441_gltD_2_Glutamate_synthase_[NADPH]_small_chain,putative_oxidoreductase,Alkyl_hydroperoxide_reductase,_large_subunit,g	RbromiL2-63
Org5_Gene422	core	1890	[RbromiL2-63]L2-63_00442_pyrK_2_Dihydrdoorotate_oxidase_B,_electron_transfer_subunit,ferredoxin-NADP(_)_reductase_subunit_alpha,sulfite_reducta	RbromiL2-63
Org5_Gene424	core	2836	[RbromiL2-63]L2-63_00444_yebN_hypothetical_protein,hypothetical_protein,putative_sporulation_protein_YtaF,Domain_of_unknown_function_DUF_458	RbromiL2-63
Org5_Gene428	core	2411	[RbromiL2-63]L2-63_00448_L2-63_00448_hypothetical_protein_461618:462307_reverse_MW:27065	RbromiL2-63
Org5_Gene429	core	1904	[RbromiL2-63]L2-63_00449_alkA_DNA-3-methyladenine_glycosylase,3-methyladenine_DNA_glycosylase/8-oxoguanine_DNA_glycosylase,8-oxoguanine_DN	RbromiL2-63
Org5_Gene431	core	958	[RbromiL2-63]L2-63_00451_L2-63_00451_Predicted_ATPase_(AAA__superfamily),orc1/cdc6_family_replication_initiation_protein,Protein_of_unknown_fu	RbromiL2-63
Org5_Gene432	core	1291	[RbromiL2-63]L2-63_00453_L2-63_00453_methyltransferase,_FkbM_family_467122:468189_forward_MW:40006	RbromiL2-63
Org5_Gene433	core	2522	[RbromiL2-63]L2-63_00454_rpe_Ribulose-phosphate_3-epimerase,ribulose-phosphate_3-epimerase,Pentose-5-phosphate-3-epimerase,ribulose-phosphat	RbromiL2-63
Org5_Gene435	core	2166	[RbromiL2-63]L2-63_00456_L2-63_00456_Putative_TrmH_family_tRNA/rRNA_methyltransferase,23S_rRNA_(guanosine-2'-O-)-methyltransferase,RNA_me	RbromiL2-63
Org5_Gene437	core	2391	[RbromiL2-63]L2-63_00458_L2-63_00458_dihydroneopterin_triphosphate_pyrophosphatase,Predicted_NTP_pyrophosphohydrolase,mutator_mutT_protei	RbromiL2-63
Org5_Gene439	core	1056	[RbromiL2-63]L2-63_00460_lysN_2-aminoadipate_transaminase,aspartate_aminotransferase,Alanine-alpha-ketoisovalerate_(or_valine-pyruvate)_aminotr	RbromiL2-63
Org5_Gene440	core	1383	[RbromiL2-63]L2-63_00461_trpS_Tryptophan--tRNA_ligase,tryptophanyl-tRNA_synthetase,Tryptophanyl-tRNA_synthetase,tryptophan--tRNA_ligase,tRNA_	RbromiL2-63
Org5_Gene447	core	1125	[RbromiL2-63]L2-63_00468_serA_D-3-phosphoglycerate_dehydrogenase,D-3-phosphoglycerate_dehydrogenase,Phosphoserine_aminotransferase,phosph	RbromiL2-63
Org5_Gene448	core	1255	[RbromiL2-63]L2-63_00469_serC_Phosphoserine_aminotransferase,3-phosphoserine/phosphohydroxythreonine_aminotransferase,Phosphoserine_aminot	RbromiL2-63
Org5_Gene449	core	1588	[RbromiL2-63]L2-63_00470_L2-63_00470_orotidine_5'-phosphate_decarboxylase,Orotidine-5'-phosphate_decarboxylase,orotidine_5'-phosphate_decarbo	RbromiL2-63
Org5_Gene450	core	1469	[RbromiL2-63]L2-63_00471_leuA_2_2-isopropylmalate_synthase,4-hyroxy-2-oxovalerate/4-hydroxy-2-oxopentanoic_acid_aldolase,,Isopropylmalate/homo	RbromiL2-63
Org5_Gene451	core	907	[RbromiL2-63]L2-63_00472_pyrC_Dihydroorotase,dihydroorotase,N-acyl-D-aspartate/D-glutamate_deacylase,dihydroorotase,_multifunctional_complex_ty	RbromiL2-63
Org5_Gene454	core	356	[RbromiL2-63]L2-63_00475_glmS_Glucosamine--fructose-6-phosphate_aminotransferase_[isomerizing],glucosamine--fructose-6-phosphate_aminotransfe	RbromiL2-63
Org5_Gene455	core	336	[RbromiL2-63]L2-63_00476_capD_UDP-glucose_4-epimerase,dTDP-glucose_4,6_dehydratase,UDP-N-acetylglucosamine_4,6-dehydratase,Polysaccharide_b	RbromiL2-63
Org5_Gene458	core	1834	[RbromiL2-63]L2-63_00479_nnr_1_Nicotinamide_nucleotide_repair_protein,putative_carbohydrate_kinase,Uncharacterized_conserved_protein,YjeF_fami	RbromiL2-63
Org5_Gene464	core	3922	[RbromiL2-63]L2-63_00486_spoIIID_14_kDa_transcription_factor,sporulation_transcriptional_regulator_SpoIIID,Stage_III_sporulation_protein_D_509822:	RbromiL2-63
Org5_Gene465	core	1017	[RbromiL2-63]L2-63_00487_arnB_UDP-4-amino-4-deoxy-L-arabinose--oxoglutarate_aminotransferase,UDP-4-amino-4-deoxy-L-arabinose--oxoglutarate_am	RbromiL2-63
Org5_Gene472	core	714	[RbromiL2-63]L2-63_00495_prpC_2-methylcitrate_synthase,citrate_synthase,2-methylcitrate_synthase/citrate_synthase_II,Citrate_synthase_518723:5200	RbromiL2-63
Org5_Gene474	core	1875	[RbromiL2-63]L2-63_00497_yidA_Phosphatase_YidA,sugar_phosphate_phosphatase,ACT_domain-containing_protein,Cof-like_hydrolase,haloacid_dehalog	RbromiL2-63
Org5_Gene486	core	4333	[RbromiL2-63]L2-63_00509_L2-63_00509_hypothetical_protein_541804:541977_reverse_MW:6862	RbromiL2-63
Org5_Gene512	core	2888	[RbromiL2-63]L2-63_00535_nudF_ADP-ribose_pyrophosphatase,adenosine_nucleotide_hydrolase_NudE,nudix-type_nucleoside_diphosphatase,_YffH/Adp	RbromiL2-63
Org5_Gene514	core	164	[RbromiL2-63]L2-63_00537_asnB_Asparagine_synthetase_[glutamine-hydrolyzing]_1,asparagine_synthetase_B,Asparagine_synthase_(glutamine-hydrolyzi	RbromiL2-63
Org5_Gene516	core	363	[RbromiL2-63]L2-63_00539_lepA_Elongation_factor_4,GTP-binding_protein_LepA,GTPases_-_translation_elongation_factors,GTP-binding_protein_LepA,G	RbromiL2-63
Org5_Gene519	core	2513	[RbromiL2-63]L2-63_00542_pdg_UV-endonuclease,endonuclease_III,3-methyladenine_DNA_glycosylase/8-oxoguanine_DNA_glycosylase,endonuclease_III,	RbromiL2-63
Org5_Gene520	core	2252	[RbromiL2-63]L2-63_00543_yfnB_Putative_HAD-hydrolase_yfnB,phosphoglycolate_phosphatase,Predicted_hydrolase_(HAD_superfamily),HAD_hydrolase,	RbromiL2-63
Org5_Gene534	core	918	[RbromiL2-63]L2-63_00557_dmdA_1_2,3-dimethylmalate_dehydratase_large_subunit,3-isopropylmalate_dehydratase_large_subunit,3-isopropylmalate_d	RbromiL2-63
Org5_Gene535	core	3136	[RbromiL2-63]L2-63_00558_DmdB_2,3-dimethylmalate_dehydratase_small_subunit,3-isopropylmalate_dehydratase_small_subunit,3-isopropylmalate_deh	RbromiL2-63
Org5_Gene537	core	171	[RbromiL2-63]L2-63_00560_malP_Maltodextrin_phosphorylase,maltodextrin_phosphorylase,glycogen/starch/alpha-glucan_phosphorylases,Carbohydrate_	RbromiL2-63
Org5_Gene538	core	590	[RbromiL2-63]L2-63_00561_malQ_4-alpha-glucanotransferase,4-alpha-glucanotransferase,4-alpha-glucanotransferase,4-alpha-glucanotransferase_604584	RbromiL2-63
Org5_Gene539	core	2857	[RbromiL2-63]L2-63_00562_ygaD_hypothetical_protein,competence_damage-inducible_protein_A,Uncharacterized_protein_(competence-_and_mitomyci	RbromiL2-63
Org5_Gene540	core	2255	[RbromiL2-63]L2-63_00563_rsmE_Ribosomal_RNA_small_subunit_methyltransferase_E,16S_ribosomal_RNA_methyltransferase_RsmE,RNA_methyltransfe	RbromiL2-63
Org5_Gene541	core	2393	[RbromiL2-63]L2-63_00564_mtnN_5'-methylthioadenosine/S-adenosylhomocysteine_nucleosidase,5'-methylthioadenosine/S-adenosylhomocysteine_nucl	RbromiL2-63
Org5_Gene542	core	453	[RbromiL2-63]L2-63_00565_ilvB_1_Acetolactate_synthase_large_subunit,acetolactate_synthase_3_catalytic_subunit,Glyoxylate_carboligase,acetolactate_	RbromiL2-63
Org5_Gene543	core	592	[RbromiL2-63]L2-63_00566_pyrP_Uracil_transporter,uracil_transporter,uracil-xanthine_permease,Permease_family_610764:612248_reverse_MW:52095	RbromiL2-63
Org5_Gene546	core	2595	[RbromiL2-63]L2-63_00569_upp_Uracil_phosphoribosyltransferase,uracil_phosphoribosyltransferase,uracil_phosphoribosyltransferase,Phosphoribosyl_tra	RbromiL2-63
Org5_Gene550	core	2359	[RbromiL2-63]L2-63_00573_L2-63_00573_ABC-type_transport_system_involved_in_multi-copper_enzyme_maturation,_permease_component,gliding_mo	RbromiL2-63
Org5_Gene551	core	1527	[RbromiL2-63]L2-63_00574_lptB_1_Lipopolysaccharide_export_system_ATP-binding_protein_LptB,nodulation_factor_exporter_subunit_NodI,ABC-type_N	RbromiL2-63
Org5_Gene552	core	3621	[RbromiL2-63]L2-63_00575_L2-63_00575_stage_V_sporulation_protein_AE,SpoVA_protein_620709:621062_forward_MW:11671	RbromiL2-63
Org5_Gene555	core	1484	[RbromiL2-63]L2-63_00578_yfdH_Bactoprenol_glucosyl_transferase_homolog_from_prophage_CPS-53,undecaprenyl_phosphate_4-deoxy-4-formamido-L-	RbromiL2-63
Org5_Gene557	core	3447	[RbromiL2-63]L2-63_00580_rpsI_30S_ribosomal_protein_S9,30S_ribosomal_protein_S9,Ribosomal_protein_S9,archaeal_ribosomal_protein_S9P,Ribosoma	RbromiL2-63
Org5_Gene558	core	3339	[RbromiL2-63]L2-63_00581_rplM_50S_ribosomal_protein_L13,50S_ribosomal_protein_L13,Ribosomal_protein_L13,ribosomal_protein_L13,Ribosomal_pro	RbromiL2-63
Org5_Gene560	core	1533	[RbromiL2-63]L2-63_00586_glkA_Glucokinase,fructokinase,Predicted_phosphosugar_isomerases,ROK_family_protein_(putative_glucokinase),ROK_family_	RbromiL2-63
Org5_Gene561	core	600	[RbromiL2-63]L2-63_00587_guaB_Inosine-5'-monophosphate_dehydrogenase,inosine_5'-monophosphate_dehydrogenase,Predicted_signal-transduction_	RbromiL2-63
Org5_Gene565	core	885	[RbromiL2-63]L2-63_00591_serS_Serine--tRNA_ligase,seryl-tRNA_synthetase,Prolyl-tRNA_synthetase,serine--tRNA_ligase,Seryl-tRNA_synthetase_N-termi	RbromiL2-63
Org5_Gene566	core	1877	[RbromiL2-63]L2-63_00592_parB_2_Probable_chromosome-partitioning_protein_parB,plasmid_partitioning_protein_RepB,ParB/RepB/Spo0J_family_parti	RbromiL2-63
Org5_Gene567	core	2141	[RbromiL2-63]L2-63_00593_soj_2_Sporulation_initiation_inhibitor_protein_soj,plasmid-partitioning_protein_RepA,Septum_formation_inhibitor-activating	RbromiL2-63
Org5_Gene568	core	1848	[RbromiL2-63]L2-63_00594_noc_Nucleoid_occlusion_protein,plasmid_partitioning_protein,ParB/RepB/Spo0J_family_partition_protein,ParB-like_nuclease_	RbromiL2-63
Org5_Gene570	core	332	[RbromiL2-63]L2-63_00596_mnmG_Glucose-inhibited_division_protein_A,tRNA_uridine_5-carboxymethylaminomethyl_modification_enzyme_GidA,Protei	RbromiL2-63
Org5_Gene571	core	715	[RbromiL2-63]L2-63_00597_mnmE_1_tRNA_modification_GTPase_MnmE,tRNA_modification_GTPase_TrmE,Predicted_GTPase,tRNA_modification_GTPas	RbromiL2-63
Org5_Gene572	core	2011	[RbromiL2-63]L2-63_00598_L2-63_00598_R3H_domain_642638:643447_reverse_MW:29913	RbromiL2-63
Org5_Gene574	core	4063	[RbromiL2-63]L2-63_00600_yidD_Putative_membrane_protein_insertion_efficiency_factor,hypothetical_protein,conserved_hypothetical_protein_YidD,Do	RbromiL2-63
Org5_Gene575	core	3616	[RbromiL2-63]L2-63_00601_rnpA_Ribonuclease_P_protein_component,ribonuclease_P,ribonuclease_P_protein_component,Ribonuclease_P_644713:6450	RbromiL2-63
Org5_Gene576	core	4411	[RbromiL2-63]L2-63_00602_rpmH_50S_ribosomal_protein_L34,50S_ribosomal_protein_L34,Ribosomal_protein_L34,ribosomal_protein_L34,Ribosomal_pr	RbromiL2-63
Org5_Gene577	core	697	[RbromiL2-63]L2-63_00603_dnaA_Chromosomal_replication_initiator_protein_DnaA,chromosomal_replication_initiation_protein,DNA_replication_protei	RbromiL2-63
Org5_Gene578	core	1224	[RbromiL2-63]L2-63_00604_dnaN_DNA_polymerase_III_subunit_beta,DNA_polymerase_III_subunit_beta,DNA_polymerase_III,_beta_subunit,DNA_polym	RbromiL2-63
Org5_Gene579	core	4166	[RbromiL2-63]L2-63_00605_ybcJ_hypothetical_protein,ribosome-associated_protein,Uncharacterized_conserved_protein,S4_domain_protein_YaaA_6483	RbromiL2-63
Org5_Gene580	core	1180	[RbromiL2-63]L2-63_00606_recF_DNA_replication_and_repair_protein_recF,recombination_protein_F,Recombinational_DNA_repair_ATPase_(RecF_pathw	RbromiL2-63
Org5_Gene581	core	3932	[RbromiL2-63]L2-63_00607_L2-63_00607_hypothetical_protein_649677:649949_forward_MW:10329	RbromiL2-63
Org5_Gene583	core	256	[RbromiL2-63]L2-63_00609_gyrB_1_DNA_gyrase_subunit_B,DNA_gyrase_subunit_B,DNA_gyrase,_B_subunit,DNA_gyrase_B_651120:653195_forward_MW	RbromiL2-63
Org5_Gene585	core	128	[RbromiL2-63]L2-63_00611_gyrA_1_DNA_gyrase_subunit_A,DNA_gyrase_subunit_A,Type_IIA_topoisomerase_(DNA_gyrase/topo_II,_topoisomerase_IV),_	RbromiL2-63
Org5_Gene590	core	3264	[RbromiL2-63]L2-63_00616_dut_Deoxyuridine_5'-triphosphate_nucleotidohydrolase,deoxyuridine_5'-triphosphate_nucleotidohydrolase,dUTP_diphosphat	RbromiL2-63
Org5_Gene591	core	1398	[RbromiL2-63]L2-63_00617_mreB_1_Rod_shape-determining_protein_MreB,rod_shape-determining_protein_MreB,Ethanolamine_utilization_protein,_po	RbromiL2-63
Org5_Gene595	core	2226	[RbromiL2-63]L2-63_00621_minD_Cell_division_inhibitor_MinD,cell_division_inhibitor_MinD,Septum_formation_inhibitor-activating_ATPase,septum_site-	RbromiL2-63
Org5_Gene596	core	3475	[RbromiL2-63]L2-63_00622_mgsA_Methylglyoxal_synthase,methylglyoxal_synthase,Methylglyoxal_synthase,methylglyoxal_synthase,MGS-like_domain_66	RbromiL2-63
Org5_Gene598	core	805	[RbromiL2-63]L2-63_00624_hisS_2_Histidine--tRNA_ligase,histidyl-tRNA_synthetase,Histidyl-tRNA_synthetase,histidine--tRNA_ligase,Anticodon_binding_	RbromiL2-63
Org5_Gene599	core	378	[RbromiL2-63]L2-63_00625_aspS_2_Aspartate--tRNA_ligase,aspartyl-tRNA_synthetase,Aspartyl/asparaginyl-tRNA_synthetases,aspartate--tRNA_ligase,tRN	RbromiL2-63
Org5_Gene601	core	951	[RbromiL2-63]L2-63_00627_L2-63_00627_Putative_virion_core_protein_(lumpy_skin_disease_virus),Double_zinc_ribbon_673409:674650_forward_MW:4	RbromiL2-63
Org5_Gene605	core	648	[RbromiL2-63]L2-63_00631_thiH_2-iminoacetate_synthase,thiamine_biosynthesis_protein_ThiH,Thiamine_monophosphate_synthase,[FeFe]_hydrogenase	RbromiL2-63
Org5_Gene608	core	1289	[RbromiL2-63]L2-63_00634_ddl_D-alanine--D-alanine_ligase,D-alanine--D-alanine_ligase,Glutathione_synthase/Ribosomal_protein_S6_modification_enzy	RbromiL2-63
Org5_Gene609	core	758	[RbromiL2-63]L2-63_00635_murF_UDP-N-acetylmuramoyl-tripeptide--D-alanyl-D-alanine_ligase,putative_bifunctional_UDP-N-acetylmuramoylalanyl-D-glu	RbromiL2-63
Org5_Gene614	core	1062	[RbromiL2-63]L2-63_00641_trpB_2_Tryptophan_synthase_beta_chain,tryptophan_synthase_subunit_beta,Tryptophan_synthase_alpha_chain,tryptophan_	RbromiL2-63
Org5_Gene615	core	2133	[RbromiL2-63]L2-63_00642_trpA_Tryptophan_synthase_alpha_chain,tryptophan_synthase_subunit_alpha,Tryptophan_synthase_alpha_chain,tryptophan_	RbromiL2-63
Org5_Gene616	core	2275	[RbromiL2-63]L2-63_00643_cobB_NAD-dependent_deacetylase,NAD-dependent_deacetylase,Sir2_family_691949:692674_forward_MW:26714	RbromiL2-63
Org5_Gene617	core	3981	[RbromiL2-63]L2-63_00644_rpsT_30S_ribosomal_protein_S20,30S_ribosomal_protein_S20,Ribosomal_protein_S20,ribosomal_protein_S20,Ribosomal_pro	RbromiL2-63
Org5_Gene618	core	4378	[RbromiL2-63]L2-63_00645_L2-63_00645_hypothetical_protein_693294:693449_forward_MW:5755	RbromiL2-63
Org5_Gene621	core	525	[RbromiL2-63]L2-63_00648_phoR_2_Alkaline_phosphatase_synthesis_sensor_protein_phoR,phosphate_regulon_sensor_protein,Predicted_periplasmic_li	RbromiL2-63
Org5_Gene622	core	2424	[RbromiL2-63]L2-63_00649_srrA_2_Staphylococcal_respiratory_response_protein_A,DNA-binding_response_regulator_CreB,Response_regulator_of_citrat	RbromiL2-63
Org5_Gene623	core	1863	[RbromiL2-63]L2-63_00650_ttdA_L(_)-tartrate_dehydratase_subunit_alpha,fumarate_hydratase,Tartrate_dehydratase_alpha_subunit/Fumarate_hydratas	RbromiL2-63
Org5_Gene624	core	2878	[RbromiL2-63]L2-63_00651_fumA_Fumarate_hydratase_class_I,_aerobic,fumarate_hydratase,Tartrate_dehydratase_beta_subunit/Fumarate_hydratase_cl	RbromiL2-63
Org5_Gene629	core	2521	[RbromiL2-63]L2-63_00656_lexA_LexA_repressor,LexA_repressor,repressor_LexA,LexA_DNA_binding_domain_700634:701290_reverse_MW:24535	RbromiL2-63
Org5_Gene634	core	344	[RbromiL2-63]L2-63_00661_dnaK_Heat_shock_protein_70,molecular_chaperone_DnaK,Ethanolamine_utilization_protein,_possible_chaperonin,chaperone	RbromiL2-63
Org5_Gene635	core	1153	[RbromiL2-63]L2-63_00662_dnaJ_1_Heat_shock_protein_J,chaperone_protein_DnaJ,Preprotein_translocase_subunit_Sec63,chaperone_protein_DnaJ,DnaJ	RbromiL2-63

Org5_Gene636	core	1591	[RbromiL2-63]L2-63_00663_prmA_Ribosomal_protein_L11_methyltransferase,ribosomal_protein_L11_methyltransferase,Ribosomal_protein_L11_methyla	RbromiL2-63
Org5_Gene638	core	1958	[RbromiL2-63]L2-63_00665_L2-63_00665_antiporter_inner_membrane_protein,CO_dehydrogenase_maturation_factor,cell_division_ATPase_MinD,ParA/M	RbromiL2-63
Org5_Gene645	core	1605	[RbromiL2-63]L2-63_00672_argF_Ornithine_carbamoyltransferase,ornithine_carbamoyltransferase,Ornithine_carbamoyltransferase,ornithine_carbamoyltr	RbromiL2-63
Org5_Gene646	core	1088	[RbromiL2-63]L2-63_00673_argD_Acetylornithine_aminotransferase,acetylornithine_aminotransferase,Ornithine/acetylornithine_aminotransferase,transa	RbromiL2-63
Org5_Gene647	core	3359	[RbromiL2-63]L2-63_00674_ypeA_1_Acetyltransferase_YpeA,putative_acetyltransferase,Predicted_acetyltransferase,ribosomal-protein-alanine_acetyltran	RbromiL2-63
Org5_Gene648	core	1865	[RbromiL2-63]L2-63_00675_argB_Acetylglutamate_kinase,acetylglutamate_kinase,Acetylglutamate_kinase,acetylglutamate_kinase,Amino_acid_kinase_far	RbromiL2-63
Org5_Gene649	core	973	[RbromiL2-63]L2-63_00676_argJ_Arginine_biosynthesis_bifunctional_protein_ArgJ,bifunctional_ornithine_acetyltransferase/N-acetylglutamate_synthase_	RbromiL2-63
Org5_Gene650	core	1349	[RbromiL2-63]L2-63_00677_argC_N-acetyl-gamma-glutamyl-phosphate_reductase,N-acetyl-gamma-glutamyl-phosphate_reductase,Acetylglutamate_semi	RbromiL2-63
Org5_Gene651	core	719	[RbromiL2-63]L2-63_00678_argH_Argininosuccinate_lyase,argininosuccinate_lyase,Argininosuccinate_lyase,argininosuccinate_lyase,Lyase_721237:722613_	RbromiL2-63
Org5_Gene652	core	983	[RbromiL2-63]L2-63_00679_argG_Argininosuccinate_synthase,argininosuccinate_synthase,Argininosuccinate_synthase,argininosuccinate_synthase,Arginos	RbromiL2-63
Org5_Gene654	core	3024	[RbromiL2-63]L2-63_00681_flr_Flavoredoxin,Flavin_reductase_like_domain_724949:725458_forward_MW:19553	RbromiL2-63
Org5_Gene662	core	3541	[RbromiL2-63]L2-63_00689_rbfA_Ribosome-binding_factor_A,ribosome-binding_factor_A,ribosome-binding_factor_A,Ribosome-binding_factor_A_737692	RbromiL2-63
Org5_Gene663	core	165	[RbromiL2-63]L2-63_00690_infB_Translation_initiation_factor_IF-2,translation_initiation_factor_IF-2,EMAP_domain,translation_initiation_factor_IF-2,Tran	RbromiL2-63
Org5_Gene666	core	1117	[RbromiL2-63]L2-63_00693_nusA_Transcription_elongation_protein_nusA,transcription_elongation_factor_NusA,Transcription_elongation_factor,transcri	RbromiL2-63
Org5_Gene667	core	2658	[RbromiL2-63]L2-63_00694_rimP_Ribosome_maturation_factor_RimP,ribosome_maturation_protein_RimP,Uncharacterised_BCR,_YhbC_family_COG0779_	RbromiL2-63
Org5_Gene670	core	4243	[RbromiL2-63]L2-63_00698_L2-63_00698_hypothetical_protein,Uncharacterized_protein_conserved_in_bacteria,transcriptional_regulator,_y4mF_family,H	RbromiL2-63
Org5_Gene671	core	2401	[RbromiL2-63]L2-63_00699_L2-63_00699_hypothetical_protein_745809:746501_forward_MW:25035	RbromiL2-63
Org5_Gene672	core	1419	[RbromiL2-63]L2-63_00700_L2-63_00700_hypothetical_protein_746523:747527_forward_MW:35480	RbromiL2-63
Org5_Gene673	core	2387	[RbromiL2-63]L2-63_00701_L2-63_00701_hypothetical_protein_747700:748398_reverse_MW:27244	RbromiL2-63
Org5_Gene675	core	90	[RbromiL2-63]L2-63_00703_hgdC_2-hydroxyglutaryl-CoA_dehydratase_component_A,pantothenate_kinase,Uncharacterized_protein_conserved_in_bacte	RbromiL2-63
Org5_Gene676	core	811	[RbromiL2-63]L2-63_00704_L2-63_00704_Uncharacterized_protein_conserved_in_bacteria,2-hydroxyglutaryl-CoA_dehydratase,_D-component_752403:75	RbromiL2-63
Org5_Gene679	core	1812	[RbromiL2-63]L2-63_00707_fba_Fructose-bisphosphate_aldolase,fructose-bisphosphate_aldolase,Fructose/tagatose_bisphosphate_aldolase,fructose-1,6-	RbromiL2-63
Org5_Gene682	core	444	[RbromiL2-63]L2-63_00710_ftsW_1_Cell_division_protein_FtsW,cell_division_protein_FtsW,Uncharacterized_conserved_protein,_contains_FHA_domain,c	RbromiL2-63
Org5_Gene683	core	695	[RbromiL2-63]L2-63_00711_pbpA_Penicillin-binding_protein_A,peptidoglycan_synthase_FtsI,Membrane_carboxypeptidase/penicillin-binding_protein,peni	RbromiL2-63
Org5_Gene684	core	95	[RbromiL2-63]L2-63_00712_secA_hypothetical_protein,preprotein_translocase_subunit_SecA,Predicted_metal-binding_protein_related_to_the_C-termin	RbromiL2-63
Org5_Gene691	core	727	[RbromiL2-63]L2-63_00719_L2-63_00719_Magnesium_transporter_mgtE,inosine_5'-monophosphate_dehydrogenase,Predicted_transcriptional_regulator_	RbromiL2-63
Org5_Gene692	core	1832	[RbromiL2-63]L2-63_00720_metF_5,10-methylenetetrahydrofolate_reductase,5,10-methylenetetrahydrofolate_reductase,5,10-methylenetetrahydrofolate	RbromiL2-63
Org5_Gene694	core	154	[RbromiL2-63]L2-63_00722_metH_Methionine_synthase,B12-dependent_methionine_synthase,Predicted_cobalamin_binding_protein,methionine_syntha	RbromiL2-63
Org5_Gene696	core	50	[RbromiL2-63]L2-63_00724_porA_Pyruvate_synthase_subunit_porA,pyruvate_flavodoxin_oxidoreductase_subunit_alpha,Sulfite_reductase,_beta_subunit_	RbromiL2-63
Org5_Gene698	core	2815	[RbromiL2-63]L2-63_00726_L2-63_00726_hypothetical_protein_789541:790065_forward_MW:20704	RbromiL2-63
Org5_Gene700	core	199	[RbromiL2-63]L2-63_00728_pflB_Formate_acetyltransferase,putative_formate_acetyltransferase_2,formate_acetyltransferase,Pyruvate_formate_lyase_7	RbromiL2-63
Org5_Gene718	core	2793	[RbromiL2-63]L2-63_00746_rfbC_dTDP-4-dehydrorhamnose_3,5-epimerase,dTDP-4-dehydrorhamnose_3,5-epimerase,dTDP-4-dehydrorhamnose_3,5-epim	RbromiL2-63
Org5_Gene719	core	1601	[RbromiL2-63]L2-63_00747_rffG_dTDP-glucose_4,6-dehydratase_2,dTDP-glucose_4,6-dehydratase,dTDP-glucose_4,6-dehydratase,NAD_dependent_epime	RbromiL2-63
Org5_Gene720	core	1311	[RbromiL2-63]L2-63_00748_rfbG_CDP-glucose_4,6-dehydratase,dTDP-glucose_4,6-dehydratase,CDP-glucose_4,6-dehydratase,NAD_dependent_epimerase_	RbromiL2-63
Org5_Gene721	core	2109	[RbromiL2-63]L2-63_00749_rfbF_Glucose-1-phosphate_cytidylyltransferase,glucose-1-phosphate_adenylyltransferase,CTP:phosphocholine_cytidylyltransf	RbromiL2-63
Org5_Gene728	core	2111	[RbromiL2-63]L2-63_00756_ycfH_Uncharacterized_deoxyribonuclease_YcfH,putative_DNAse,Mg-dependent_DNase,hydrolase,_TatD_family,TatD_related_	RbromiL2-63
Org5_Gene729	core	297	[RbromiL2-63]L2-63_00757_metG_Methionine--tRNA_ligase,methionyl-tRNA_synthetase,Methionyl-tRNA_synthetase,methionine--tRNA_ligase,tRNA_syn	RbromiL2-63
Org5_Gene736	core	1439	[RbromiL2-63]L2-63_00764_dus_Probable_tRNA-dihydrouridine_synthase,tRNA-dihydrouridine_synthase_B,tRNA-dihydrouridine_synthase,putative_TIM-	RbromiL2-63
Org5_Gene737	core	931	[RbromiL2-63]L2-63_00765_glyA_Pyridoxal-phosphate-dependent_serine_hydroxymethyltransferase,serine_hydroxymethyltransferase,8-amino-7-oxonon	RbromiL2-63
Org5_Gene740	core	2876	[RbromiL2-63]L2-63_00768_L2-63_00768_undecaprenyl_pyrophosphate_phosphatase,PAP2_superfamily_832921:833469_reverse_MW:20566	RbromiL2-63
Org5_Gene741	core	1402	[RbromiL2-63]L2-63_00769_spoVAD_Stage_V_sporulation_protein_AD,stage_V_sporulation_protein_AD,stage_V_sporulation_protein_AD,Stage_V_sporul	RbromiL2-63
Org5_Gene745	core	626	[RbromiL2-63]L2-63_00773_gltX_Glutamate--tRNA_ligase,glutamyl-tRNA_synthetase,Glutamyl-_and_glutaminyl-tRNA_synthetases,glutamate--tRNA_ligas	RbromiL2-63
Org5_Gene746	core	434	[RbromiL2-63]L2-63_00774_glnS_Glutamine--tRNA_ligase,glutaminyl-tRNA_synthetase,Glutamyl-_and_glutaminyl-tRNA_synthetases,glutamine--tRNA_lig	RbromiL2-63
Org5_Gene747	core	2221	[RbromiL2-63]L2-63_00775_fabG_2_3-oxoacyl-[acyl-carrier-protein]_reductase_FabG,3-ketoacyl-(acyl-carrier-protein)_reductase,Uncharacterized_conserv	RbromiL2-63
Org5_Gene748	core	607	[RbromiL2-63]L2-63_00776_murE_UDP-N-acetylmuramoyl-L-alanyl-D-glutamate--L-lysine_ligase,UDP-N-acetylmuramoylalanyl-D-glutamate--2,6-diaminopi	RbromiL2-63
Org5_Gene750	core	581	[RbromiL2-63]L2-63_00778_malF_Maltose_transport_system_permease_protein_malF,maltose_transporter_membrane_protein,ABC-type_polysaccharide	RbromiL2-63
Org5_Gene751	core	1824	[RbromiL2-63]L2-63_00779_malG_Maltose_transport_system_permease_protein_malG,maltose_transporter_permease,ABC-type_maltose_transport_syst	RbromiL2-63
Org5_Gene752	core	460	[RbromiL2-63]L2-63_00780_L2-63_00780_Alpha-amylase_precursor,trehalose-6-phosphate_hydrolase,Maltooligosyl_trehalose_synthase,alpha,alpha-phos	RbromiL2-63
Org5_Gene753	core	3378	[RbromiL2-63]L2-63_00781_rpsL_30S_ribosomal_protein_S12,30S_ribosomal_protein_S12,Ribosomal_protein_S12,ribosomal_protein_S12,Ribosomal_prot	RbromiL2-63
Org5_Gene754	core	2922	[RbromiL2-63]L2-63_00782_rpsG_30S_ribosomal_protein_S7,30S_ribosomal_protein_S7,Ribosomal_protein_S7,ribosomal_protein_S7,Ribosomal_protein_	RbromiL2-63
Org5_Gene755	core	240	[RbromiL2-63]L2-63_00783_fusA_Vegetative_protein_19,elongation_factor_G,GTPases_-_translation_elongation_factors,translation_elongation_factor_G	RbromiL2-63
Org5_Gene756	core	1023	[RbromiL2-63]L2-63_00784_tuf_Elongation_factor_Tu,elongation_factor_Tu,GTPases_-_translation_elongation_factors,translation_elongation_factor_Tu,	RbromiL2-63
Org5_Gene757	core	3243	[RbromiL2-63]L2-63_00785_L2-63_00785_stage_V_sporulation_protein_AC,SpoVA_protein_857225:857680_reverse_MW:15951	RbromiL2-63
Org5_Gene758	core	1182	[RbromiL2-63]L2-63_00786_iscS_1_Cysteine_desulfurase,cysteine_desulfurase,Uncharacterized_protein_conserved_in_bacteria,cysteine_desulfurase_Nif	RbromiL2-63
Org5_Gene759	core	1065	[RbromiL2-63]L2-63_00787_thiI_Probable_tRNA_sulfurtransferase,thiamine_biosynthesis_protein_ThiI,Predicted_ATPase_of_the_PP-loop_superfamily_i	RbromiL2-63
Org5_Gene766	core	701	[RbromiL2-63]L2-63_00794_dltB_1_hypothetical_protein,D-alanyl-lipoteichoic_acid_biosynthesis_protein_DltB,MBOAT_family_866759:868153_forward_	RbromiL2-63
Org5_Gene769	core	3796	[RbromiL2-63]L2-63_00797_rpsJ_BS13,30S_ribosomal_protein_S10,ribosomal_protein_S10,Ribosomal_protein_S10p/S20e_870606:870917_forward_MW:	RbromiL2-63
Org5_Gene770	core	2594	[RbromiL2-63]L2-63_00798_rplC_50S_ribosomal_protein_L3,50S_ribosomal_protein_L3,Ribosomal_protein_L3,50S_ribosomal_protein_L3,Ribosomal_prot	RbromiL2-63
Org5_Gene771	core	2622	[RbromiL2-63]L2-63_00799_rplD_50S_ribosomal_protein_L4,50S_ribosomal_protein_L4,50S_ribosomal_protein_L4,Ribosomal_protein_L4/L1_family_8716	RbromiL2-63
Org5_Gene772	core	3858	[RbromiL2-63]L2-63_00800_rplW_50S_ribosomal_protein_L23,50S_ribosomal_protein_L23,Ribosomal_protein_L23,archaeal_ribosomal_protein_L23,Ribos	RbromiL2-63
Org5_Gene773	core	1925	[RbromiL2-63]L2-63_00801_rplB_L3,50S_ribosomal_protein_L2,Ribosomal_protein_L2,ribosomal_protein_L2,Ribosomal_Proteins_L2,_C-terminal_domain_	RbromiL2-63
Org5_Gene774	core	3940	[RbromiL2-63]L2-63_00802_rpsS_BS17,30S_ribosomal_protein_S19,Ribosomal_protein_S19,ribosomal_protein_S19,Ribosomal_protein_S19_873509:8737	RbromiL2-63
Org5_Gene775	core	3667	[RbromiL2-63]L2-63_00803_rplV_50S_ribosomal_protein_L22,50S_ribosomal_protein_L22,ribosomal_protein_L22,Ribosomal_protein_L22p/L17e_873802:	RbromiL2-63
Org5_Gene776	core	2426	[RbromiL2-63]L2-63_00804_rpsC_BS2,30S_ribosomal_protein_S3,Uncharacterized_conserved_protein,ribosomal_protein_S3,Ribosomal_protein_S3,_C-ter	RbromiL2-63
Org5_Gene777	core	3375	[RbromiL2-63]L2-63_00805_rplP_50S_ribosomal_protein_L16,50S_ribosomal_protein_L16,Ribosomal_protein_L16/L10E,ribosomal_protein_L16,Ribosomal	RbromiL2-63
Org5_Gene778	core	4200	[RbromiL2-63]L2-63_00806_rpmC_50S_ribosomal_protein_L29,50S_ribosomal_protein_L29,Ribosomal_protein_L29,ribosomal_protein_L29,Ribosomal_L2	RbromiL2-63
Org5_Gene779	core	3990	[RbromiL2-63]L2-63_00807_rpsQ_30S_ribosomal_protein_S17,30S_ribosomal_protein_S17,30S_ribosomal_protein_S17,Ribosomal_protein_S17_875469:8	RbromiL2-63
Org5_Gene780	core	3565	[RbromiL2-63]L2-63_00808_rplN_50S_ribosomal_protein_L14,50S_ribosomal_protein_L14,Ribosomal_protein_L14,ribosomal_protein_L14,Ribosomal_prot	RbromiL2-63
Org5_Gene781	core	3773	[RbromiL2-63]L2-63_00809_rplX_50S_ribosomal_protein_L24,50S_ribosomal_protein_L24,ribosomal_protein_L24,KOW_motif_876149:876466_forward_M	RbromiL2-63
Org5_Gene782	core	2914	[RbromiL2-63]L2-63_00810_rplE_50S_ribosomal_protein_L5,50S_ribosomal_protein_L5,Ribosomal_protein_L5,ribosomal_L5P_family_C-terminus_876481:	RbromiL2-63
Org5_Gene783	core	4289	[RbromiL2-63]L2-63_00811_rpsN1_BS-A,30S_ribosomal_protein_S14,Ribosomal_protein_S14p/S29e_877033:877218_forward_MW:7212	RbromiL2-63
Org5_Gene784	core	3469	[RbromiL2-63]L2-63_00812_rpsH_30S_ribosomal_protein_S8,30S_ribosomal_protein_S8,Ribosomal_protein_S8,Ribosomal_protein_S8_877238:877636_fo	RbromiL2-63
Org5_Gene785	core	2900	[RbromiL2-63]L2-63_00813_rplF_BL10,50S_ribosomal_protein_L6,ribosomal_protein_L6,Ribosomal_protein_L6_877652:878194_forward_MW:19765	RbromiL2-63
Org5_Gene786	core	3597	[RbromiL2-63]L2-63_00814_rplR_50S_ribosomal_protein_L18,50S_ribosomal_protein_L18,ribosomal_protein_L18,Ribosomal_L18p/L5e_family_878213:87	RbromiL2-63
Org5_Gene787	core	3067	[RbromiL2-63]L2-63_00815_rpsE_30S_ribosomal_protein_S5,30S_ribosomal_protein_S5,Ribosomal_protein_S5,ribosomal_protein_S5,Ribosomal_protein_	RbromiL2-63
Org5_Gene788	core	4346	[RbromiL2-63]L2-63_00816_rpmD_50S_ribosomal_protein_L30,50S_ribosomal_protein_L30,ribosomal_protein_L30,Ribosomal_protein_L30p/L7e_879105:	RbromiL2-63
Org5_Gene789	core	3283	[RbromiL2-63]L2-63_00817_rplO_50S_ribosomal_protein_L15,50S_ribosomal_protein_L15,ribosomal_protein_L15,Ribosomal_protein_L18e/L15_879294:8	RbromiL2-63
Org5_Gene790	core	782	[RbromiL2-63]L2-63_00818_secY_hypothetical_protein,preprotein_translocase_subunit_SecY,Preprotein_translocase_subunit_SecY,preprotein_transloca	RbromiL2-63
Org5_Gene791	core	2593	[RbromiL2-63]L2-63_00819_L2-63_00819_Adenylate_kinase,adenylate_kinase,adenylate_kinase,Adenylate_kinase_881072:881704_forward_MW:22950	RbromiL2-63
Org5_Gene792	core	2186	[RbromiL2-63]L2-63_00820_map_1_Methionine_aminopeptidase_1,methionine_aminopeptidase,Xaa-Pro_aminopeptidase,methionine_aminopeptidase,_	RbromiL2-63
Org5_Gene793	core	4037	[RbromiL2-63]L2-63_00821_L2-63_00821_hypothetical_protein_882471:882716_forward_MW:9001	RbromiL2-63
Org5_Gene794	core	4142	[RbromiL2-63]L2-63_00822_infA_Translation_initiation_factor_IF-1,translation_initiation_factor_IF-1,translation_initiation_factor_IF-1,Translation_initiati	RbromiL2-63
Org5_Gene796	core	3568	[RbromiL2-63]L2-63_00824_rpsM_30S_ribosomal_protein_S13,30S_ribosomal_protein_S13,Ribosomal_protein_S13,30S_ribosomal_protein_S13,Ribosoma	RbromiL2-63
Org5_Gene797	core	3445	[RbromiL2-63]L2-63_00825_rpsK_30S_ribosomal_protein_S11,30S_ribosomal_protein_S11,Ribosomal_protein_S11,30S_ribosomal_protein_S11,Ribosomal_	RbromiL2-63
Org5_Gene798	core	2686	[RbromiL2-63]L2-63_00826_rpsD_30S_ribosomal_protein_S4,30S_ribosomal_protein_S4,ribosomal_protein_S4,S4_domain_883961:884563_forward_MW:	RbromiL2-63
Org5_Gene799	core	1541	[RbromiL2-63]L2-63_00827_rpoA_DNA-directed_RNA_polymerase_subunit_alpha,DNA-directed_RNA_polymerase_subunit_alpha,DNA-directed_RNA_poly	RbromiL2-63
Org5_Gene800	core	3661	[RbromiL2-63]L2-63_00828_rplQ_BL21,50S_ribosomal_protein_L17,Ribosomal_protein_L17,ribosomal_protein_L17,Ribosomal_protein_L17_885664:8860	RbromiL2-63
Org5_Gene803	core	639	[RbromiL2-63]L2-63_00831_pyk_Pyruvate_kinase,pyruvate_kinase,pyruvate_kinase,Pyruvate_kinase,_barrel_domain_889764:891206_forward_MW:52101	RbromiL2-63
Org5_Gene805	core	2501	[RbromiL2-63]L2-63_00833_sigF_2_Stage_II_sporulation_protein_AC,sporulation_sigma_factor_SigF,RNA_polymerase_sigma-F_factor,Sigma-70,_region_4_	RbromiL2-63
Org5_Gene806	core	3296	[RbromiL2-63]L2-63_00834_spoIIAB_Anti-sigma_F_factor,anti-sigma_F_factor,Signal_transduction_histidine_kinase,anti-sigma_F_factor,Histidine_kinase-,	RbromiL2-63
Org5_Gene807	core	3765	[RbromiL2-63]L2-63_00835_spoIIAA_1_Stage_II_sporulation_protein_AA,Anti-anti-sigma_regulatory_factor_(antagonist_of_anti-sigma_factor),anti-sigma_	RbromiL2-63
Org5_Gene809	core	1955	[RbromiL2-63]L2-63_00837_rsmI_Ribosomal_RNA_small_subunit_methyltransferase_I,SAM-dependent_16S_ribosomal_RNA_C1402_ribose_2'-O-methyltr	RbromiL2-63
Org5_Gene811	core	4343	[RbromiL2-63]L2-63_00839_fer_Ferredoxin,NADH_dehydrogenase_subunit_I,Uncharacterized_Fe-S_center_protein,archaeoflavoprotein,_MJ0208_family,4	RbromiL2-63
Org5_Gene812	core	1810	[RbromiL2-63]L2-63_00840_tpl_hypothetical_protein,PSP1_C-terminal_conserved_region_896584:897450_reverse_MW:32255	RbromiL2-63
Org5_Gene818	core	945	[RbromiL2-63]L2-63_00846_proA_Gamma-glutamyl_phosphate_reductase,gamma-glutamyl_phosphate_reductase,Gamma-glutamyl_phosphate_reductas	RbromiL2-63
Org5_Gene819	core	1993	[RbromiL2-63]L2-63_00847_proB_Glutamate_5-kinase_1,gamma-glutamyl_kinase,Gamma-glutamyl_phosphate_reductase,glutamate_5-kinase,Amino_acid	RbromiL2-63
Org5_Gene820	core	1602	[RbromiL2-63]L2-63_00848_metA_Homoserine_O-succinyltransferase,homoserine_O-succinyltransferase,homoserine_O-succinyltransferase,Homoserine_	RbromiL2-63
Org5_Gene821	core	644	[RbromiL2-63]L2-63_00849_proS_Proline--tRNA_ligase,prolyl-tRNA_synthetase,Prolyl-tRNA_synthetase,proline--tRNA_ligase,Prolyl-tRNA_synthetase,_C-te	RbromiL2-63
Org5_Gene822	core	4101	[RbromiL2-63]L2-63_00850_acpP_1_Acyl_carrier_protein,acyl_carrier_protein,Acyl_carrier_protein,acyl_carrier_protein,Phosphopantetheine_attachment_	RbromiL2-63
Org5_Gene823	core	1884	[RbromiL2-63]L2-63_00851_aroE_Shikimate_dehydrogenase,shikimate_5-dehydrogenase,5-enolpyruvylshikimate-3-phosphate_synthase,shikimate_5-dehy	RbromiL2-63
Org5_Gene825	core	1379	[RbromiL2-63]L2-63_00853_aroF_Phospho-2-dehydro-3-deoxyheptonate_aldolase,3-deoxy-7-phosphoheptulonate_synthase,3-deoxy-D-arabino-heptuloso	RbromiL2-63
Org5_Gene826	core	1936	[RbromiL2-63]L2-63_00854_tyrC_Arogenate_dehydrogenase,prephenate_dehydrogenase,Prephenate_dehydrogenase,Prephenate_dehydrogenase_90786	RbromiL2-63
Org5_Gene827	core	1306	[RbromiL2-63]L2-63_00855_aroB_3-dehydroquinate_synthase,3-dehydroquinate_synthase,3-dehydroquinate_synthetase,3-dehydroquinate_synthase,3-de	RbromiL2-63
Org5_Gene828	core	859	[RbromiL2-63]L2-63_00856_aroA_3-phosphoshikimate_1-carboxyvinyltransferase,3-phosphoshikimate_1-carboxyvinyltransferase,5-enolpyruvylshikimate-	RbromiL2-63
Org5_Gene829	core	1278	[RbromiL2-63]L2-63_00857_aroC_Chorismate_synthase,chorismate_synthase,Chorismate_synthase,chorismate_synthase,Chorismate_synthase_911076:91	RbromiL2-63
Org5_Gene830	core	1155	[RbromiL2-63]L2-63_00858_pheA_P-protein,prephenate_dehydratase,Prephenate_dehydrogenase,chorismate_mutase,Prephenate_dehydratase_912154:	RbromiL2-63
Org5_Gene834	core	553	[RbromiL2-63]L2-63_00862_treS_Trehalose_synthase/amylase_TreS,trehalose-6-phosphate_hydrolase,Maltooligosyl_trehalose_synthase,trehalose_syntha	RbromiL2-63
Org5_Gene839	core	112	[RbromiL2-63]L2-63_00867_valS_Valine--tRNA_ligase,valyl-tRNA_synthetase,Valyl-tRNA_synthetase,valine--tRNA_ligase,tRNA_synthetases_class_I_(I,_L,_	RbromiL2-63
Org5_Gene882	core	641	[RbromiL2-63]L2-63_00910_L2-63_00910_nicotinate_phosphoribosyltransferase,nicotinate_phosphoribosyltransferase,Nicotinate_phosphoribosyltransfer	RbromiL2-63
Org5_Gene885	core	2018	[RbromiL2-63]L2-63_00913_thyX_Thymidylate_synthase_thyX,FAD-dependent_thymidylate_synthase,thymidylate_synthase,_flavin-dependent,Thymidylat	RbromiL2-63
Org5_Gene889	core	2911	[RbromiL2-63]L2-63_00917_yajL_Chaperone_protein_YajL,oxidative-stress-resistance_chaperone,Uncharacterized_protein_conserved_in_bacteria,DJ-1_fa	RbromiL2-63
Org5_Gene892	core	971	[RbromiL2-63]L2-63_00920_yhbU_2_Uncharacterized_protease_yhbU_precursor,putative_protease,Collagenase_and_related_proteases,Peptidase_family_	RbromiL2-63
Org5_Gene893	core	118	[RbromiL2-63]L2-63_00922_alaS_Alanine--tRNA_ligase,alanyl-tRNA_synthetase,Alanyl-tRNA_synthetase,alanine--tRNA_ligase,tRNA_synthetases_class_II_(	RbromiL2-63
Org5_Gene899	core	2506	[RbromiL2-63]L2-63_00929_L2-63_00929_Phosphorylated_carbohydrates_phosphatase_TM_1254,2-deoxyglucose-6-phosphatase,beta-phosphoglucomuta	RbromiL2-63
Org5_Gene902	core	371	[RbromiL2-63]L2-63_00932_ilvD_Dihydroxy-acid_dehydratase,dihydroxy-acid_dehydratase,Dihydroxyacid_dehydratase/phosphogluconate_dehydratase,di	RbromiL2-63
Org5_Gene903	core	258	[RbromiL2-63]L2-63_00933_fus_1_Elongation_factor_G,elongation_factor_G,translation_elongation_factor_G,Elongation_factor_G,_domain_IV_996399:9	RbromiL2-63
Org5_Gene906	core	396	[RbromiL2-63]L2-63_00936_L2-63_00936_Putative_multidrug_export_ATP-binding/permease_protein_SAV1866,cyclic_beta-1,2-glucan_ABC_transporter,A	RbromiL2-63
Org5_Gene907	core	405	[RbromiL2-63]L2-63_00937_msbA_Lipid_A_export_ATP-binding/permease_protein_MsbA,lipid_transporter_ATP-binding/permease_protein,ABC-type_bac	RbromiL2-63
Org5_Gene908	core	570	[RbromiL2-63]L2-63_00938_gpmI_2,3-bisphosphoglycerate-independent_phosphoglycerate_mutase,phosphoglyceromutase,Predicted_phosphoglycerate_	RbromiL2-63
Org5_Gene909	core	2143	[RbromiL2-63]L2-63_00939_tpiA_Triosephosphate_isomerase,bifunctional_phosphoglycerate_kinase/triosephosphate_isomerase,triose-phosphate_isome	RbromiL2-63
Org5_Gene910	core	1009	[RbromiL2-63]L2-63_00940_pgk_Phosphoglycerate_kinase,bifunctional_phosphoglycerate_kinase/triosephosphate_isomerase,3-phosphoglycerate_kinase,	RbromiL2-63
Org5_Gene911	core	2998	[RbromiL2-63]L2-63_00941_trmL_tRNA_(cytidine(34)-2'-O)-methyltransferase,putative_rRNA_methylase,tRNA_(cytidine(34)-2'-O)-methyltransferase,SpoU	RbromiL2-63

Org5_Gene918	core	3239	[RbromiL2-63]L2-63_00948_luxS_S-ribosylhomocysteine_lyase,S-ribosylhomocysteinase,LuxS_protein_involved_in_autoinducer_AI2_synthesis,S-Ribosylho	RbromiL2-63
Org5_Gene925	core	2056	[RbromiL2-63]L2-63_00955_xynD_Bifunctional_xylanase/deacetylase_precursor,polysaccharide_deacetylase_family_sporulation_protein_PdaB,Polysaccha	RbromiL2-63
Org5_Gene926	core	685	[RbromiL2-63]L2-63_00956_cysS_Cysteine--tRNA_ligase,cysteinyl-tRNA_synthetase,Cysteinyl-tRNA_synthetase,cysteine--tRNA_ligase,tRNA_synthetases_c	RbromiL2-63
Org5_Gene927	core	2491	[RbromiL2-63]L2-63_00957_cysE_2_Serine_acetyltransferase,serine_acetyltransferase,Acetyltransferase_(isoleucine_patch_superfamily),serine_O-acetyltr	RbromiL2-63
Org5_Gene928	core	2725	[RbromiL2-63]L2-63_00958_pgsA_1_CDP-diacylglycerol--glycerol-3-phosphate_3-phosphatidyltransferase,phosphatidylglycerophosphate_synthetase,CDP-	RbromiL2-63
Org5_Gene929	core	778	[RbromiL2-63]L2-63_00959_rimO_Ribosomal_protein_S12_methylthiotransferase_RimO,ribosomal_protein_S12_methylthiotransferase,ribosomal_protein	RbromiL2-63
Org5_Gene930	core	2648	[RbromiL2-63]L2-63_00960_recX_Regulatory_protein_recX,recombination_regulator_RecX,Uncharacterized_protein_conserved_in_bacteria,RecX_family_	RbromiL2-63
Org5_Gene931	core	1195	[RbromiL2-63]L2-63_00961_recA_Recombinase_A,recombinase_A,RecA-superfamily_ATPases_implicated_in_signal_transduction,protein_RecA,recA_bact	RbromiL2-63
Org5_Gene933	core	1443	[RbromiL2-63]L2-63_00963_L2-63_00963_Predicted_metal-dependent_enzyme,Protein_of_unknown_function_(DUF1385)_1026241:1027224_reverse_MV	RbromiL2-63
Org5_Gene934	core	605	[RbromiL2-63]L2-63_00964_phoR_3_Alkaline_phosphatase_synthesis_sensor_protein_phoR,signal_transduction_histidine-protein_kinase_BaeS,Signal_tra	RbromiL2-63
Org5_Gene935	core	2345	[RbromiL2-63]L2-63_00965_regX3_2_Sensory_transduction_protein_regX3,osmolarity_response_regulator,Response_regulator_containing_a_CheY-like_re	RbromiL2-63
Org5_Gene937	core	2484	[RbromiL2-63]L2-63_00967_trmB_tRNA_(guanine-N(7)-)-methyltransferase,tRNA_(guanine-N(7)-)-methyltransferase,tRNA_(guanine-N(7)-)-methyltransfer	RbromiL2-63
Org5_Gene940	core	4248	[RbromiL2-63]L2-63_00970_L2-63_00970_hybrid_cluster_protein-associated_redox_disulfide_domain,Domain_of_unknown_function_(DUF1858)_103309	RbromiL2-63
Org5_Gene943	core	3905	[RbromiL2-63]L2-63_00973_L2-63_00973_CxxC-x17-CxxC_domain_1035875:1036153_forward_MW:10491	RbromiL2-63
Org5_Gene953	core	3251	[RbromiL2-63]L2-63_00983_ctsR_Class_three_stress_gene_repressor,Transcriptional_repressor_of_class_III_stress_genes,Firmicute_transcriptional_repre	RbromiL2-63
Org5_Gene954	core	3043	[RbromiL2-63]L2-63_00984_L2-63_00984_Uncharacterized_protein_with_conserved_CXXC_pairs,UvrB/uvrC_motif_1043595:1044098_forward_MW:18521	RbromiL2-63
Org5_Gene955	core	1444	[RbromiL2-63]L2-63_00985_L2-63_00985_Putative_ATP:guanido_phosphotransferase_SAV0524,ATP:guanido_phosphotransferase,Arginine_kinase,ATP:gu	RbromiL2-63
Org5_Gene956	core	141	[RbromiL2-63]L2-63_00986_clpC_Negative_regulator_of_genetic_competence_ClpC/MecB,protein_disaggregation_chaperone,Transcriptional_regulator_c	RbromiL2-63
Org5_Gene958	core	4080	[RbromiL2-63]L2-63_00988_L2-63_00988_hypothetical_protein,hypothetical_protein,Predicted_RNA-binding_protein_(contains_KH_domain)_1048480:10	RbromiL2-63
Org5_Gene959	core	4034	[RbromiL2-63]L2-63_00989_rpsP_BS17,30S_ribosomal_protein_S16,ribosomal_protein_S16,Ribosomal_protein_S16_1048737:1048982_reverse_MW:9194	RbromiL2-63
Org5_Gene960	core	676	[RbromiL2-63]L2-63_00990_ffh_Fifty-four_homolog,signal_recognition_particle_protein,Signal_recognition_particle_GTPase,signal_recognition_particle_p	RbromiL2-63
Org5_Gene961	core	3631	[RbromiL2-63]L2-63_00991_L2-63_00991_hypothetical_protein,putative_DNA-binding_protein,Uncharacterized_protein_conserved_in_bacteria,RNA_poly	RbromiL2-63
Org5_Gene962	core	1321	[RbromiL2-63]L2-63_00992_ybeZ_PhoH-like_protein,hypothetical_protein,Predicted_ATPase_related_to_phosphate_starvation-inducible_protein_PhoH,P	RbromiL2-63
Org5_Gene963	core	3007	[RbromiL2-63]L2-63_00993_L2-63_00993_Probable_rRNA_maturation_factor,metal-binding_heat_shock_protein,metalloprotein,_YbeY/UPF0054_family,U	RbromiL2-63
Org5_Gene965	core	1643	[RbromiL2-63]L2-63_00995_era_Bex_protein,GTPase_Era,Predicted_GTPase,GTP-binding_protein_Era,GTPase_of_unknown_function_1053415:1054332_f	RbromiL2-63
Org5_Gene966	core	4338	[RbromiL2-63]L2-63_00996_L2-63_00996_hypothetical_protein_1054436:1054609_forward_MW:6797	RbromiL2-63
Org5_Gene967	core	2204	[RbromiL2-63]L2-63_00997_recO_Recombination_protein_O,DNA_repair_protein_RecO,DNA_repair_protein_RecO,Recombination_protein_O_N_terminal	RbromiL2-63
Org5_Gene968	core	135	[RbromiL2-63]L2-63_00998_mutS2_2_MutS2_protein,recombination_and_DNA_strand_exchange_inhibitor_protein,Uncharacterized_protein_conserved_i	RbromiL2-63
Org5_Gene969	core	3006	[RbromiL2-63]L2-63_00999_rimM_Ribosome_maturation_factor_rimM,16S_rRNA-processing_protein_RimM,16S_rRNA_processing_protein_RimM,RimM_	RbromiL2-63
Org5_Gene970	core	2404	[RbromiL2-63]L2-63_01000_trmD_tRNA_(guanine-N(1)-)-methyltransferase,tRNA_(guanine-N(1)-)-methyltransferase,tRNA-(guanine-N1)-methyltransferas	RbromiL2-63
Org5_Gene971	core	3960	[RbromiL2-63]L2-63_01001_crh_Catabolite_repression_HPr,phosphocarrier_protein_Chr,Uncharacterized_protein_conserved_in_bacteria,phosphocarrier,_	RbromiL2-63
Org5_Gene972	core	347	[RbromiL2-63]L2-63_01002_uvrC_Excinuclease_ABC_subunit_C,excinuclease_ABC_subunit_C,Nuclease_subunit_of_the_excinuclease_complex,excinucleas	RbromiL2-63
Org5_Gene973	core	2851	[RbromiL2-63]L2-63_01003_rsmD_Ribosomal_RNA_small_subunit_methyltransferase_D,16S_rRNA_m(2)G966-methyltransferase,Predicted_O-methyltrans	RbromiL2-63
Org5_Gene975	core	3206	[RbromiL2-63]L2-63_01005_L2-63_01005_hypothetical_protein_1062763:1063224_forward_MW:17080	RbromiL2-63
Org5_Gene978	core	1626	[RbromiL2-63]L2-63_01008_rsmH_Ribosomal_RNA_small_subunit_methyltransferase_H,16S_rRNA_m(4)C1402_methyltranserfase,S-adenosyl-methyltrans	RbromiL2-63
Org5_Gene980	core	194	[RbromiL2-63]L2-63_01010_ftsI_Peptidoglycan_synthase_FtsI_precursor,peptidoglycan_synthase_FtsI,Membrane_carboxypeptidase/penicillin-binding_pr	RbromiL2-63
Org5_Gene981	core	1356	[RbromiL2-63]L2-63_01011_mraY_Phospho-N-acetylmuramoyl-pentapeptide-transferase,phospho-N-acetylmuramoyl-pentapeptide-transferase,phospho-	RbromiL2-63
Org5_Gene982	core	928	[RbromiL2-63]L2-63_01012_ftsW_2_Cell_division_protein_FtsW,cell_wall_shape-determining_protein,stage_V_sporulation_protein_E,Cell_cycle_protein_	RbromiL2-63
Org5_Gene983	core	1196	[RbromiL2-63]L2-63_01013_murG_UDP-N-acetylglucosamine--N-acetylmuramyl-(pentapeptide)_pyrophosphoryl-undecaprenol_N-acetylglucosamine_tran	RbromiL2-63
Org5_Gene984	core	933	[RbromiL2-63]L2-63_01014_murAA_UDP-N-acetylglucosamine_1-carboxyvinyltransferase_1,UDP-N-acetylglucosamine_1-carboxyvinyltransferase,ATP_pho	RbromiL2-63
Org5_Gene986	core	1214	[RbromiL2-63]L2-63_01016_ftsZ_Cell_division_protein_FtsZ,cell_division_protein_FtsZ,Cell_division_GTPase,cell_division_protein_FtsZ,Tubulin/FtsZ_famil	RbromiL2-63
Org5_Gene996	core	611	[RbromiL2-63]L2-63_01026_nadB_L-aspartate_oxidase,L-aspartate_oxidase,Aspartate_oxidase,L-aspartate_oxidase,FAD_binding_domain_1083093:108455	RbromiL2-63
Org5_Gene997	core	1902	[RbromiL2-63]L2-63_01027_nadC_Probable_nicotinate-nucleotide_pyrophosphorylase_[carboxylating],nicotinate-nucleotide_pyrophosphorylase,Aspartat	RbromiL2-63
Org1_Gene100	unique	2481	2595398026_IE32DRAFT_00107_hypothetical_protein_[Ruminococcus_bromii_YE282]	Ruminococcus_bromii_YE282
Org1_Gene1001	unique	2181	2595398956_IE32DRAFT_01040_Membrane_proteins_related_to_metalloendopeptidases_[Ruminococcus_bromii_YE282]	Ruminococcus_bromii_YE282
Org1_Gene1002	unique	2596	2595398957_IE32DRAFT_01041_Cytidylate_kinase_[Ruminococcus_bromii_YE282]	Ruminococcus_bromii_YE282
Org1_Gene1003	unique	3317	2595398958_IE32DRAFT_01042_Transcriptional_regulators_[Ruminococcus_bromii_YE282]	Ruminococcus_bromii_YE282
Org1_Gene1004	unique	3478	2595398959_IE32DRAFT_01043_MatE_[Ruminococcus_bromii_YE282]	Ruminococcus_bromii_YE282
Org1_Gene1005	unique	3114	2595398960_IE32DRAFT_01044_Polysaccharide_biosynthesis_C-terminal_domain_[Ruminococcus_bromii_YE282]	Ruminococcus_bromii_YE282
Org1_Gene1006	unique	3500	2595398961_IE32DRAFT_01045_MatE_[Ruminococcus_bromii_YE282]	Ruminococcus_bromii_YE282
Org1_Gene1007	unique	3788	2595398963_IE32DRAFT_01047_hypothetical_protein_[Ruminococcus_bromii_YE282]	Ruminococcus_bromii_YE282
Org1_Gene1008	unique	3404	2595398964_IE32DRAFT_01048_hypothetical_protein_[Ruminococcus_bromii_YE282]	Ruminococcus_bromii_YE282
Org1_Gene1009	unique	4391	2595398965_IE32DRAFT_01049_hypothetical_protein_[Ruminococcus_bromii_YE282]	Ruminococcus_bromii_YE282
Org1_Gene101	unique	808	2595398027_IE32DRAFT_00108_Reverse_transcriptase_(RNA-dependent_DNA_polymerase)_[Ruminococcus_bromii_YE282]	Ruminococcus_bromii_YE282
Org1_Gene1010	unique	2883	2595398966_IE32DRAFT_01050_two_component_transcriptional_regulator,_LytTR_family_[Ruminococcus_bromii_YE282]	Ruminococcus_bromii_YE282
Org1_Gene1011	unique	831	2595398967_IE32DRAFT_01051_GHKL_domain_[Ruminococcus_bromii_YE282]	Ruminococcus_bromii_YE282
Org1_Gene1012	unique	868	2595398968_IE32DRAFT_01052_FAD_dependent_oxidoreductase/Rieske_[2Fe-2S]_domain_[Ruminococcus_bromii_YE282]	Ruminococcus_bromii_YE282
Org1_Gene1013	unique	1991	2595398969_IE32DRAFT_01053_Tocopherol_cyclase_[Ruminococcus_bromii_YE282]	Ruminococcus_bromii_YE282
Org1_Gene1014	unique	1286	2595398970_IE32DRAFT_01054_Merozoite_surface_protein_1_(MSP1)_C-terminus_[Ruminococcus_bromii_YE282]	Ruminococcus_bromii_YE282
Org1_Gene102	unique	2060	2595398028_IE32DRAFT_00109_hypothetical_protein_[Ruminococcus_bromii_YE282]	Ruminococcus_bromii_YE282
Org1_Gene1020	unique	627	2595398976_IE32DRAFT_01062_Histidine_kinase-,_DNA_gyrase_B-,_and_HSP90-like_ATPase_[Ruminococcus_bromii_YE282]	Ruminococcus_bromii_YE282
Org1_Gene1021	unique	1192	2595398977_IE32DRAFT_01063_hypothetical_protein_[Ruminococcus_bromii_YE282]	Ruminococcus_bromii_YE282
Org1_Gene1022	unique	1166	2595398978_IE32DRAFT_01064_cation_diffusion_facilitator_family_transporter_[Ruminococcus_bromii_YE282]	Ruminococcus_bromii_YE282
Org1_Gene1024	unique	2690	2595398980_IE32DRAFT_01066_Transglycosylase_SLT_domain_[Ruminococcus_bromii_YE282]	Ruminococcus_bromii_YE282
Org1_Gene1025	unique	2666	2595398981_IE32DRAFT_01067_dephospho-CoA_kinase_[Ruminococcus_bromii_YE282]	Ruminococcus_bromii_YE282
Org1_Gene103	unique	7	2595398029_IE32DRAFT_00110_hypothetical_protein_[Ruminococcus_bromii_YE282]	Ruminococcus_bromii_YE282
Org1_Gene1039	unique	3563	2595398995_IE32DRAFT_01081_Transposon-encoded_protein_TnpV_[Ruminococcus_bromii_YE282]	Ruminococcus_bromii_YE282
Org1_Gene104	unique	1277	2595398030_IE32DRAFT_00111_hypothetical_protein_[Ruminococcus_bromii_YE282]	Ruminococcus_bromii_YE282
Org1_Gene1040	unique	2574	2595398996_IE32DRAFT_01082_hypothetical_protein_[Ruminococcus_bromii_YE282]	Ruminococcus_bromii_YE282
Org1_Gene1041	unique	2941	2595398997_IE32DRAFT_01083_hypothetical_protein_[Ruminococcus_bromii_YE282]	Ruminococcus_bromii_YE282
Org1_Gene1042	unique	126	2595398998_IE32DRAFT_01084_Uncharacterized_protein_conserved_in_bacteria_[Ruminococcus_bromii_YE282]	Ruminococcus_bromii_YE282
Org1_Gene1043	unique	397	2595398999_IE32DRAFT_01085_Adenine_specific_DNA_methylase_Mod_[Ruminococcus_bromii_YE282]	Ruminococcus_bromii_YE282
Org1_Gene1044	unique	1042	2595399000_IE32DRAFT_01086_Predicted_ATPase_(AAA+_superfamily)_[Ruminococcus_bromii_YE282]	Ruminococcus_bromii_YE282
Org1_Gene1045	unique	3828	2595399001_IE32DRAFT_01087_hypothetical_protein_[Ruminococcus_bromii_YE282]	Ruminococcus_bromii_YE282
Org1_Gene1046	unique	1896	2595399002_IE32DRAFT_01088_ParB/RepB/Spo0J_family_partition_protein_[Ruminococcus_bromii_YE282]	Ruminococcus_bromii_YE282
Org1_Gene1047	unique	4317	2595399003_IE32DRAFT_01089_hypothetical_protein_[Ruminococcus_bromii_YE282]	Ruminococcus_bromii_YE282
Org1_Gene1048	unique	450	2595399004_IE32DRAFT_01090_Site-specific_recombinases,_DNA_invertase_Pin_homologs_[Ruminococcus_bromii_YE282]	Ruminococcus_bromii_YE282
Org1_Gene105	unique	274	2595398031_IE32DRAFT_00112_hypothetical_protein_[Ruminococcus_bromii_YE282]	Ruminococcus_bromii_YE282
Org1_Gene1054	unique	3639	2595399010_IE32DRAFT_01096_hypothetical_protein_[Ruminococcus_bromii_YE282]	Ruminococcus_bromii_YE282
Org1_Gene1055	unique	3879	2595399011_IE32DRAFT_01097_HTH-like_domain_[Ruminococcus_bromii_YE282]	Ruminococcus_bromii_YE282
Org1_Gene1058	unique	844	2595399014_IE32DRAFT_01100_Transglutaminase-like_superfamily_[Ruminococcus_bromii_YE282]	Ruminococcus_bromii_YE282
Org1_Gene1059	unique	2954	2595399015_IE32DRAFT_01101_Predicted_membrane_protein_[Ruminococcus_bromii_YE282]	Ruminococcus_bromii_YE282
Org1_Gene106	unique	105	2595398032_IE32DRAFT_00113_hypothetical_protein_[Ruminococcus_bromii_YE282]	Ruminococcus_bromii_YE282
Org1_Gene1060	unique	2591	2595399016_IE32DRAFT_01102_phosphoribosylanthranilate_isomerase_(EC_5.3.1.24)_[Ruminococcus_bromii_YE282]	Ruminococcus_bromii_YE282
Org1_Gene1061	unique	2089	2595399017_IE32DRAFT_01103_indole-3-glycerol_phosphate_synthase_(EC_4.1.1.48)_[Ruminococcus_bromii_YE282]	Ruminococcus_bromii_YE282
Org1_Gene1062	unique	1417	2595399018_IE32DRAFT_01104_anthranilate_phosphoribosyltransferase_[Ruminococcus_bromii_YE282]	Ruminococcus_bromii_YE282
Org1_Gene1063	unique	2794	2595399019_IE32DRAFT_01105_glutamine_amidotransferase_of_anthranilate_synthase_or_aminodeoxychorismate_synthase_[Ruminococcus_bromii_YE2	Ruminococcus_bromii_YE282
Org1_Gene1064	unique	606	2595399020_IE32DRAFT_01106_anthranilate_synthase_component_I,_non-proteobacterial_lineages_[Ruminococcus_bromii_YE282]	Ruminococcus_bromii_YE282
Org1_Gene1065	unique	3523	2595399021_IE32DRAFT_01108_hypothetical_protein_[Ruminococcus_bromii_YE282]	Ruminococcus_bromii_YE282
Org1_Gene1067	unique	2530	2595399023_IE32DRAFT_01110_Putative_TM_nitroreductase_[Ruminococcus_bromii_YE282]	Ruminococcus_bromii_YE282
Org1_Gene1068	unique	3297	2595399024_IE32DRAFT_01111_Transcriptional_regulators_[Ruminococcus_bromii_YE282]	Ruminococcus_bromii_YE282
Org1_Gene1069	unique	3798	2595399025_IE32DRAFT_01112_thioredoxin_[Ruminococcus_bromii_YE282]	Ruminococcus_bromii_YE282
Org1_Gene107	unique	1107	2595398033_IE32DRAFT_00114_hypothetical_protein_[Ruminococcus_bromii_YE282]	Ruminococcus_bromii_YE282
Org1_Gene1070	unique	1795	2595399026_IE32DRAFT_01113_Thioredoxin_reductase_[Ruminococcus_bromii_YE282]	Ruminococcus_bromii_YE282
Org1_Gene1071	unique	2356	2595399027_IE32DRAFT_01114_Predicted_membrane_protein_[Ruminococcus_bromii_YE282]	Ruminococcus_bromii_YE282
Org1_Gene1074	unique	3367	2595399030_IE32DRAFT_01117_hypothetical_protein_[Ruminococcus_bromii_YE282]	Ruminococcus_bromii_YE282
Org1_Gene1075	unique	2536	2595399032_IE32DRAFT_01119_Predicted_phosphatases_[Ruminococcus_bromii_YE282]	Ruminococcus_bromii_YE282
Org1_Gene1076	unique	481	2595399033_IE32DRAFT_01120_O-Antigen_ligase_[Ruminococcus_bromii_YE282]	Ruminococcus_bromii_YE282
Org1_Gene1077	unique	3026	2595399034_IE32DRAFT_01121_Copper/zinc_superoxide_dismutase_(SODC)_[Ruminococcus_bromii_YE282]	Ruminococcus_bromii_YE282
Org1_Gene1078	unique	985	2595399035_IE32DRAFT_01122_Alkaline_phosphatase_[Ruminococcus_bromii_YE282]	Ruminococcus_bromii_YE282
Org1_Gene1079	unique	1944	2595399036_IE32DRAFT_01123_Methyltransferase_domain_[Ruminococcus_bromii_YE282]	Ruminococcus_bromii_YE282
Org1_Gene108	unique	34	2595398034_IE32DRAFT_00115_hypothetical_protein_[Ruminococcus_bromii_YE282]	Ruminococcus_bromii_YE282
Org1_Gene1080	unique	3971	2595399037_IE32DRAFT_01124_Phosphotransferase_system,_HPr-related_proteins_[Ruminococcus_bromii_YE282]	Ruminococcus_bromii_YE282
Org1_Gene1081	unique	2389	2595399038_IE32DRAFT_01125_hypothetical_protein_[Ruminococcus_bromii_YE282]	Ruminococcus_bromii_YE282
Org1_Gene1082	unique	1676	2595399039_IE32DRAFT_01126_hypothetical_protein_[Ruminococcus_bromii_YE282]	Ruminococcus_bromii_YE282
Org1_Gene1084	unique	2510	2595399041_IE32DRAFT_01128_haloacid_dehalogenase_superfamily,_subfamily_IA,_variant_1_with_third_motif_having_Dx(3-4)D_or_Dx(3-4)E_[Rumino	Ruminococcus_bromii_YE282
Org1_Gene1085	unique	2567	2595399042_IE32DRAFT_01129_Uncharacterized_protein_conserved_in_bacteria_[Ruminococcus_bromii_YE282]	Ruminococcus_bromii_YE282
Org1_Gene1086	unique	3139	2595399043_IE32DRAFT_01130_hypothetical_protein_[Ruminococcus_bromii_YE282]	Ruminococcus_bromii_YE282
Org1_Gene1087	unique	2879	2595399044_IE32DRAFT_01131_Predicted_transcriptional_regulators_[Ruminococcus_bromii_YE282]	Ruminococcus_bromii_YE282
Org1_Gene1088	unique	2163	2595399045_IE32DRAFT_01132_4Fe-4S_binding_domain/4Fe-4S_dicluster_domain_[Ruminococcus_bromii_YE282]	Ruminococcus_bromii_YE282
Org1_Gene109	unique	3713	2595398035_IE32DRAFT_00116_hypothetical_protein_[Ruminococcus_bromii_YE282]	Ruminococcus_bromii_YE282
Org1_Gene1091	unique	158	2595399048_IE32DRAFT_01135_Membrane_carboxypeptidase_(penicillin-binding_protein)_[Ruminococcus_bromii_YE282]	Ruminococcus_bromii_YE282
Org1_Gene1092	unique	3739	2595399049_IE32DRAFT_01136_hypothetical_protein_[Ruminococcus_bromii_YE282]	Ruminococcus_bromii_YE282
Org1_Gene1097	unique	3202	2595399054_IE32DRAFT_01141_acetyl-CoA_carboxylase,_biotin_carboxyl_carrier_protein_[Ruminococcus_bromii_YE282]	Ruminococcus_bromii_YE282
Org1_Gene110	unique	163	2595398036_IE32DRAFT_00117_phage_tail_tape_measure_protein,_TP901_family,_core_region_[Ruminococcus_bromii_YE282]	Ruminococcus_bromii_YE282
Org1_Gene1102	unique	1785	2595399059_IE32DRAFT_01146_hypothetical_protein_[Ruminococcus_bromii_YE282]	Ruminococcus_bromii_YE282
Org1_Gene1103	unique	3149	2595399060_IE32DRAFT_01147_Transcriptional_regulators_[Ruminococcus_bromii_YE282]	Ruminococcus_bromii_YE282
Org1_Gene1104	unique	1619	2595399061_IE32DRAFT_01148_Transcriptional_regulator_[Ruminococcus_bromii_YE282]	Ruminococcus_bromii_YE282
Org1_Gene1105	unique	2229	2595399062_IE32DRAFT_01149_CRISPR-associated_endoribonuclease_Cas6_[Ruminococcus_bromii_YE282]	Ruminococcus_bromii_YE282
Org1_Gene1106	unique	643	2595399063_IE32DRAFT_01150_CRISPR-associated_protein_Cas8a1/Csx8,_subtype_I_[Ruminococcus_bromii_YE282]	Ruminococcus_bromii_YE282
Org1_Gene1107	unique	1764	2595399064_IE32DRAFT_01151_CRISPR-associated_autoregulator_DevR_family_[Ruminococcus_bromii_YE282]	Ruminococcus_bromii_YE282
Org1_Gene1108	unique	1345	2595399065_IE32DRAFT_01152_CRISPR-associated_protein_Cas5,_N-terminal_domain_[Ruminococcus_bromii_YE282]	Ruminococcus_bromii_YE282

Org1_Gene1109	unique	353	2595399066_IE32DRAFT_01153_CRISPR-associated_helicase_Cas3/CRISPR-associated_endonuclease_Cas3-HD_[Ruminococcus_bromii_YE282]	Ruminococcus_bromii_YE282
Org1_Gene111	unique	1329	2595398037_IE32DRAFT_00118_Site-specific_recombinase_XerD_[Ruminococcus_bromii_YE282]	Ruminococcus_bromii_YE282
Org1_Gene1110	unique	3145	2595399067_IE32DRAFT_01154_hypothetical_protein_[Ruminococcus_bromii_YE282]	Ruminococcus_bromii_YE282
Org1_Gene1111	unique	2868	2595399068_IE32DRAFT_01155_CRISPR-associated_protein_Cas4_[Ruminococcus_bromii_YE282]	Ruminococcus_bromii_YE282
Org1_Gene1112	unique	2833	2595399069_IE32DRAFT_01156_CRISPR-associated_endonuclease_Cas1_[Ruminococcus_bromii_YE282]	Ruminococcus_bromii_YE282
Org1_Gene1113	unique	3851	2595399070_IE32DRAFT_01157_CRISPR-associated_endonuclease_Cas2_[Ruminococcus_bromii_YE282]	Ruminococcus_bromii_YE282
Org1_Gene1114	unique	3644	2595399071_IE32DRAFT_01158_Predicted_transcriptional_regulators_[Ruminococcus_bromii_YE282]	Ruminococcus_bromii_YE282
Org1_Gene1115	unique	876	2595399072_IE32DRAFT_01159_Bacterial_cell_division_membrane_protein_[Ruminococcus_bromii_YE282]	Ruminococcus_bromii_YE282
Org1_Gene1116	unique	2732	2595399073_IE32DRAFT_01160_Glycopeptide_antibiotics_resistance_protein_[Ruminococcus_bromii_YE282]	Ruminococcus_bromii_YE282
Org1_Gene1117	unique	3370	2595399074_IE32DRAFT_01161_hypothetical_protein_[Ruminococcus_bromii_YE282]	Ruminococcus_bromii_YE282
Org1_Gene1118	unique	1436	2595399075_IE32DRAFT_01162_MobA/MobL_family_[Ruminococcus_bromii_YE282]	Ruminococcus_bromii_YE282
Org1_Gene112	unique	3282	2595398038_IE32DRAFT_00119_hypothetical_protein_[Ruminococcus_bromii_YE282]	Ruminococcus_bromii_YE282
Org1_Gene1120	unique	3422	2595399077_IE32DRAFT_01164_hypothetical_protein_[Ruminococcus_bromii_YE282]	Ruminococcus_bromii_YE282
Org1_Gene1121	unique	4225	2595399078_IE32DRAFT_01165_Conjugal_transfer_protein_TraD_[Ruminococcus_bromii_YE282]	Ruminococcus_bromii_YE282
Org1_Gene1123	unique	3371	2595399080_IE32DRAFT_01167_hypothetical_protein_[Ruminococcus_bromii_YE282]	Ruminococcus_bromii_YE282
Org1_Gene1124	unique	2581	2595399081_IE32DRAFT_01168_hypothetical_protein_[Ruminococcus_bromii_YE282]	Ruminococcus_bromii_YE282
Org1_Gene1125	unique	1965	2595399082_IE32DRAFT_01169_Site-specific_DNA_methylase_[Ruminococcus_bromii_YE282]	Ruminococcus_bromii_YE282
Org1_Gene1126	unique	3865	2595399083_IE32DRAFT_01170_hypothetical_protein_[Ruminococcus_bromii_YE282]	Ruminococcus_bromii_YE282
Org1_Gene1127	unique	3613	2595399084_IE32DRAFT_01171_hypothetical_protein_[Ruminococcus_bromii_YE282]	Ruminococcus_bromii_YE282
Org1_Gene1128	unique	1717	2595399085_IE32DRAFT_01172_hypothetical_protein_[Ruminococcus_bromii_YE282]	Ruminococcus_bromii_YE282
Org1_Gene1129	unique	1343	2595399086_IE32DRAFT_01173_methionine_adenosyltransferase_(EC_2.5.1.6)_[Ruminococcus_bromii_YE282]	Ruminococcus_bromii_YE282
Org1_Gene113	unique	3107	2595398039_IE32DRAFT_00120_hypothetical_protein_[Ruminococcus_bromii_YE282]	Ruminococcus_bromii_YE282
Org1_Gene1130	unique	3892	2595399087_IE32DRAFT_01174_hypothetical_protein_[Ruminococcus_bromii_YE282]	Ruminococcus_bromii_YE282
Org1_Gene1131	unique	502	2595399088_IE32DRAFT_01175_thermosome_subunit_[Ruminococcus_bromii_YE282]	Ruminococcus_bromii_YE282
Org1_Gene1132	unique	1525	2595399089_IE32DRAFT_01176_Predicted_Fe-S-cluster_redox_enzyme_[Ruminococcus_bromii_YE282]	Ruminococcus_bromii_YE282
Org1_Gene1133	unique	2640	2595399090_IE32DRAFT_01177_DNA_modification_methylase_[Ruminococcus_bromii_YE282]	Ruminococcus_bromii_YE282
Org1_Gene1134	unique	3637	2595399091_IE32DRAFT_01178_hypothetical_protein_[Ruminococcus_bromii_YE282]	Ruminococcus_bromii_YE282
Org1_Gene1135	unique	2672	2595399092_IE32DRAFT_01179_Domain_of_unknown_function_(DUF955)_[Ruminococcus_bromii_YE282]	Ruminococcus_bromii_YE282
Org1_Gene1136	unique	3653	2595399093_IE32DRAFT_01180_Helix-turn-helix_domain_[Ruminococcus_bromii_YE282]	Ruminococcus_bromii_YE282
Org1_Gene1137	unique	3974	2595399094_IE32DRAFT_01181_hypothetical_protein_[Ruminococcus_bromii_YE282]	Ruminococcus_bromii_YE282
Org1_Gene1138	unique	4356	2595399095_IE32DRAFT_01182_hypothetical_protein_[Ruminococcus_bromii_YE282]	Ruminococcus_bromii_YE282
Org1_Gene1139	unique	3968	2595399096_IE32DRAFT_01183_hypothetical_protein_[Ruminococcus_bromii_YE282]	Ruminococcus_bromii_YE282
Org1_Gene114	unique	3027	2595398040_IE32DRAFT_00121_Recombination_protein_U_[Ruminococcus_bromii_YE282]	Ruminococcus_bromii_YE282
Org1_Gene1140	unique	2058	2595399097_IE32DRAFT_01184_ADP-ribosylglycohydrolase_[Ruminococcus_bromii_YE282]	Ruminococcus_bromii_YE282
Org1_Gene1141	unique	1582	2595399098_IE32DRAFT_01185_NUMOD1_domain/Domain_of_unknown_function_(DUF4379)_[Ruminococcus_bromii_YE282]	Ruminococcus_bromii_YE282
Org1_Gene1142	unique	1365	2595399099_IE32DRAFT_01186_Predicted_nucleotidyltransferase_[Ruminococcus_bromii_YE282]	Ruminococcus_bromii_YE282
Org1_Gene1143	unique	1563	2595399100_IE32DRAFT_01187_hypothetical_protein_[Ruminococcus_bromii_YE282]	Ruminococcus_bromii_YE282
Org1_Gene1144	unique	862	2595399101_IE32DRAFT_01188_tRNA_nucleotidyltransferase/poly(A)_polymerase_[Ruminococcus_bromii_YE282]	Ruminococcus_bromii_YE282
Org1_Gene1145	unique	3734	2595399102_IE32DRAFT_01189_hypothetical_protein_[Ruminococcus_bromii_YE282]	Ruminococcus_bromii_YE282
Org1_Gene1146	unique	2251	2595399103_IE32DRAFT_01190_Site-specific_DNA_methylase_[Ruminococcus_bromii_YE282]	Ruminococcus_bromii_YE282
Org1_Gene1147	unique	4273	2595399104_IE32DRAFT_01191_hypothetical_protein_[Ruminococcus_bromii_YE282]	Ruminococcus_bromii_YE282
Org1_Gene1148	unique	3448	2595399105_IE32DRAFT_01192_hypothetical_protein_[Ruminococcus_bromii_YE282]	Ruminococcus_bromii_YE282
Org1_Gene1149	unique	2197	2595399106_IE32DRAFT_01193_Site-specific_DNA_methylase_[Ruminococcus_bromii_YE282]	Ruminococcus_bromii_YE282
Org1_Gene115	unique	4084	2595398041_IE32DRAFT_00122_hypothetical_protein_[Ruminococcus_bromii_YE282]	Ruminococcus_bromii_YE282
Org1_Gene1150	unique	4254	2595399107_IE32DRAFT_01194_hypothetical_protein_[Ruminococcus_bromii_YE282]	Ruminococcus_bromii_YE282
Org1_Gene1151	unique	3117	2595399108_IE32DRAFT_01195_hypothetical_protein_[Ruminococcus_bromii_YE282]	Ruminococcus_bromii_YE282
Org1_Gene1152	unique	3652	2595399109_IE32DRAFT_01196_hypothetical_protein_[Ruminococcus_bromii_YE282]	Ruminococcus_bromii_YE282
Org1_Gene1153	unique	4044	2595399110_IE32DRAFT_01197_hypothetical_protein_[Ruminococcus_bromii_YE282]	Ruminococcus_bromii_YE282
Org1_Gene1154	unique	2130	2595399111_IE32DRAFT_01198_DNA_modification_methylase_[Ruminococcus_bromii_YE282]	Ruminococcus_bromii_YE282
Org1_Gene1155	unique	4321	2595399112_IE32DRAFT_01199_hypothetical_protein_[Ruminococcus_bromii_YE282]	Ruminococcus_bromii_YE282
Org1_Gene1156	unique	2791	2595399113_IE32DRAFT_01200_Phosphoadenosine_phosphosulfate_reductase_family_[Ruminococcus_bromii_YE282]	Ruminococcus_bromii_YE282
Org1_Gene1157	unique	3856	2595399114_IE32DRAFT_01201_Phosphoadenosine_phosphosulfate_reductase_family_[Ruminococcus_bromii_YE282]	Ruminococcus_bromii_YE282
Org1_Gene1158	unique	3662	2595399115_IE32DRAFT_01202_hypothetical_protein_[Ruminococcus_bromii_YE282]	Ruminococcus_bromii_YE282
Org1_Gene1159	unique	1510	2595399116_IE32DRAFT_01203_Predicted_kinase_[Ruminococcus_bromii_YE282]	Ruminococcus_bromii_YE282
Org1_Gene116	unique	3995	2595398042_IE32DRAFT_00123_hypothetical_protein_[Ruminococcus_bromii_YE282]	Ruminococcus_bromii_YE282
Org1_Gene1160	unique	3670	2595399117_IE32DRAFT_01204_hypothetical_protein_[Ruminococcus_bromii_YE282]	Ruminococcus_bromii_YE282
Org1_Gene1161	unique	2576	2595399118_IE32DRAFT_01205_Protein_of_unknown_function_(DUF1071)_[Ruminococcus_bromii_YE282]	Ruminococcus_bromii_YE282
Org1_Gene1162	unique	3281	2595399119_IE32DRAFT_01206_hypothetical_protein_[Ruminococcus_bromii_YE282]	Ruminococcus_bromii_YE282
Org1_Gene1163	unique	2645	2595399120_IE32DRAFT_01207_hypothetical_protein_[Ruminococcus_bromii_YE282]	Ruminococcus_bromii_YE282
Org1_Gene1164	unique	2928	2595399121_IE32DRAFT_01208_hypothetical_protein_[Ruminococcus_bromii_YE282]	Ruminococcus_bromii_YE282
Org1_Gene1165	unique	794	2595399122_IE32DRAFT_01209_Replicative_DNA_helicase_[Ruminococcus_bromii_YE282]	Ruminococcus_bromii_YE282
Org1_Gene1166	unique	1390	2595399123_IE32DRAFT_01210_CHC2_zinc_finger/DNA_primase_catalytic_core,_N-terminal_domain_[Ruminococcus_bromii_YE282]	Ruminococcus_bromii_YE282
Org1_Gene1167	unique	2044	2595399124_IE32DRAFT_01211_PD-(D/E)XK_nuclease_superfamily_[Ruminococcus_bromii_YE282]	Ruminococcus_bromii_YE282
Org1_Gene1168	unique	81	2595399125_IE32DRAFT_01212_DNA_polymerase_III,_alpha_subunit_[Ruminococcus_bromii_YE282]	Ruminococcus_bromii_YE282
Org1_Gene1169	unique	3286	2595399126_IE32DRAFT_01213_hypothetical_protein_[Ruminococcus_bromii_YE282]	Ruminococcus_bromii_YE282
Org1_Gene117	unique	922	2595398043_IE32DRAFT_00124_hypothetical_protein_[Ruminococcus_bromii_YE282]	Ruminococcus_bromii_YE282
Org1_Gene1170	unique	1749	2595399127_IE32DRAFT_01214_group_I_intron_endonuclease_[Ruminococcus_bromii_YE282]	Ruminococcus_bromii_YE282
Org1_Gene1171	unique	953	2595399128_IE32DRAFT_01215_Uncharacterized_conserved_protein_[Ruminococcus_bromii_YE282]	Ruminococcus_bromii_YE282
Org1_Gene1172	unique	2438	2595399129_IE32DRAFT_01216_HNH_endonuclease/AP2_domain_[Ruminococcus_bromii_YE282]	Ruminococcus_bromii_YE282
Org1_Gene1173	unique	3901	2595399130_IE32DRAFT_01217_hypothetical_protein_[Ruminococcus_bromii_YE282]	Ruminococcus_bromii_YE282
Org1_Gene1174	unique	2110	2595399131_IE32DRAFT_01218_hypothetical_protein_[Ruminococcus_bromii_YE282]	Ruminococcus_bromii_YE282
Org1_Gene1175	unique	3761	2595399132_IE32DRAFT_01219_hypothetical_protein_[Ruminococcus_bromii_YE282]	Ruminococcus_bromii_YE282
Org1_Gene1176	unique	2839	2595399133_IE32DRAFT_01220_Guanylate_kinase_[Ruminococcus_bromii_YE282]	Ruminococcus_bromii_YE282
Org1_Gene1177	unique	3332	2595399134_IE32DRAFT_01221_hypothetical_protein_[Ruminococcus_bromii_YE282]	Ruminococcus_bromii_YE282
Org1_Gene1178	unique	182	2595399135_IE32DRAFT_01222_ribonucleoside-triphosphate_reductase_class_III_catalytic_subunit_(EC_1.17.4.2)_[Ruminococcus_bromii_YE282]	Ruminococcus_bromii_YE282
Org1_Gene1179	unique	2933	2595399136_IE32DRAFT_01223_anaerobic_ribonucleoside-triphosphate_reductase_activating_protein_[Ruminococcus_bromii_YE282]	Ruminococcus_bromii_YE282
Org1_Gene118	unique	2201	2595398044_IE32DRAFT_00125_hypothetical_protein_[Ruminococcus_bromii_YE282]	Ruminococcus_bromii_YE282
Org1_Gene1180	unique	1084	2595399137_IE32DRAFT_01224_hypothetical_protein_[Ruminococcus_bromii_YE282]	Ruminococcus_bromii_YE282
Org1_Gene1181	unique	3411	2595399138_IE32DRAFT_01225_hypothetical_protein_[Ruminococcus_bromii_YE282]	Ruminococcus_bromii_YE282
Org1_Gene1182	unique	3334	2595399139_IE32DRAFT_01226_dUTPase_[Ruminococcus_bromii_YE282]	Ruminococcus_bromii_YE282
Org1_Gene1183	unique	3335	2595399140_IE32DRAFT_01227_hypothetical_protein_[Ruminococcus_bromii_YE282]	Ruminococcus_bromii_YE282
Org1_Gene1184	unique	486	2595399141_IE32DRAFT_01228_Site-specific_recombinases,_DNA_invertase_Pin_homologs_[Ruminococcus_bromii_YE282]	Ruminococcus_bromii_YE282
Org1_Gene1186	unique	2483	2595399143_IE32DRAFT_01230_Predicted_SAM-dependent_methyltransferase_[Ruminococcus_bromii_YE282]	Ruminococcus_bromii_YE282
Org1_Gene1187	unique	2173	2595399144_IE32DRAFT_01231_dinuclear_metal_center_protein,_YbgI/SA1388_family_[Ruminococcus_bromii_YE282]	Ruminococcus_bromii_YE282
Org1_Gene1189	unique	780	2595399146_IE32DRAFT_01233_hypothetical_protein_[Ruminococcus_bromii_YE282]	Ruminococcus_bromii_YE282
Org1_Gene119	unique	2047	2595398045_IE32DRAFT_00126_hypothetical_protein_[Ruminococcus_bromii_YE282]	Ruminococcus_bromii_YE282
Org1_Gene1193	unique	1864	2595399150_IE32DRAFT_01237_protein-(glutamine-N5)_methyltransferase,_release_factor-specific_[Ruminococcus_bromii_YE282]	Ruminococcus_bromii_YE282
Org1_Gene12	unique	1399	2595397933_IE32DRAFT_00014_DNA-directed_RNA_polymerase,_subunit_M/Transcription_elongation_factor_TFIIS_[Ruminococcus_bromii_YE282]	Ruminococcus_bromii_YE282
Org1_Gene120	unique	3113	2595398046_IE32DRAFT_00127_hypothetical_protein_[Ruminococcus_bromii_YE282]	Ruminococcus_bromii_YE282
Org1_Gene1206	unique	832	2595399163_IE32DRAFT_01250_Maltose-binding_periplasmic_proteins/domains_[Ruminococcus_bromii_YE282]	Ruminococcus_bromii_YE282
Org1_Gene1209	unique	4337	2595399166_IE32DRAFT_01253_Restriction_alleviation_protein_Lar_[Ruminococcus_bromii_YE282]	Ruminococcus_bromii_YE282
Org1_Gene121	unique	1281	2595398047_IE32DRAFT_00128_hypothetical_protein_[Ruminococcus_bromii_YE282]	Ruminococcus_bromii_YE282
Org1_Gene1210	unique	1662	2595399167_IE32DRAFT_01254_Cysteine_synthase_[Ruminococcus_bromii_YE282]	Ruminococcus_bromii_YE282
Org1_Gene1211	unique	1122	2595399168_IE32DRAFT_01255_Cystathionine_beta-lyases/cystathionine_gamma-synthases_[Ruminococcus_bromii_YE282]	Ruminococcus_bromii_YE282
Org1_Gene1212	unique	3222	2595399169_IE32DRAFT_01256_S-ribosylhomocysteine_lyase_(EC_4.4.1.21)/quorum-sensing_autoinducer_2_(AI-2)_synthesis_protein_LuxS_[Ruminococc	Ruminococcus_bromii_YE282
Org1_Gene1213	unique	1604	2595399170_IE32DRAFT_01257_methyltransferase,_TIGR00027_family_[Ruminococcus_bromii_YE282]	Ruminococcus_bromii_YE282
Org1_Gene1214	unique	121	2595399171_IE32DRAFT_01258_Leucine_rich_repeats_(6_copies)_[Ruminococcus_bromii_YE282]	Ruminococcus_bromii_YE282
Org1_Gene1219	unique	170	2595399176_IE32DRAFT_01263_Peptidase_family_M28/PA_domain_[Ruminococcus_bromii_YE282]	Ruminococcus_bromii_YE282
Org1_Gene122	unique	3503	2595398048_IE32DRAFT_00129_hypothetical_protein_[Ruminococcus_bromii_YE282]	Ruminococcus_bromii_YE282
Org1_Gene1222	unique	4326	2595399179_IE32DRAFT_01266_hypothetical_protein_[Ruminococcus_bromii_YE282]	Ruminococcus_bromii_YE282
Org1_Gene1226	unique	3903	2595399183_IE32DRAFT_01270_hypothetical_protein_[Ruminococcus_bromii_YE282]	Ruminococcus_bromii_YE282
Org1_Gene1229	unique	4194	2595399186_IE32DRAFT_01273_hypothetical_protein_[Ruminococcus_bromii_YE282]	Ruminococcus_bromii_YE282
Org1_Gene123	unique	2953	2595398049_IE32DRAFT_00130_hypothetical_protein_[Ruminococcus_bromii_YE282]	Ruminococcus_bromii_YE282
Org1_Gene1231	unique	3315	2595399188_IE32DRAFT_01275_transcriptional_regulator,_BadM/Rrf2_family_[Ruminococcus_bromii_YE282]	Ruminococcus_bromii_YE282
Org1_Gene1232	unique	2993	2595399189_IE32DRAFT_01276_Predicted_membrane_protein_[Ruminococcus_bromii_YE282]	Ruminococcus_bromii_YE282
Org1_Gene1233	unique	3545	2595399190_IE32DRAFT_01277_Uncharacterized_protein_conserved_in_bacteria_[Ruminococcus_bromii_YE282]	Ruminococcus_bromii_YE282
Org1_Gene1234	unique	2896	2595399191_IE32DRAFT_01278_Nitroreductase_[Ruminococcus_bromii_YE282]	Ruminococcus_bromii_YE282
Org1_Gene1235	unique	2294	2595399192_IE32DRAFT_01279_Predicted_glutamine_amidotransferases_[Ruminococcus_bromii_YE282]	Ruminococcus_bromii_YE282
Org1_Gene1237	unique	3305	2595399194_IE32DRAFT_01282_transcriptional_regulator,_LytTR_family_[Ruminococcus_bromii_YE282]	Ruminococcus_bromii_YE282
Org1_Gene1238	unique	3321	2595399195_IE32DRAFT_01283_Protein_of_unknown_function_(DUF3021)_[Ruminococcus_bromii_YE282]	Ruminococcus_bromii_YE282
Org1_Gene124	unique	4297	2595398050_IE32DRAFT_00131_hypothetical_protein_[Ruminococcus_bromii_YE282]	Ruminococcus_bromii_YE282
Org1_Gene1240	unique	1594	2595399197_IE32DRAFT_01285_ABC-2_type_transporter_[Ruminococcus_bromii_YE282]	Ruminococcus_bromii_YE282
Org1_Gene1241	unique	2755	2595399198_IE32DRAFT_01286_hypothetical_protein_[Ruminococcus_bromii_YE282]	Ruminococcus_bromii_YE282
Org1_Gene1246	unique	769	2595399203_IE32DRAFT_01291_putative_efflux_protein,_MATE_family_[Ruminococcus_bromii_YE282]	Ruminococcus_bromii_YE282
Org1_Gene1247	unique	1272	2595399204_IE32DRAFT_01292_Leucine_rich_repeats_(6_copies)_[Ruminococcus_bromii_YE282]	Ruminococcus_bromii_YE282
Org1_Gene1248	unique	3730	2595399205_IE32DRAFT_01293_Predicted_transcriptional_regulators_[Ruminococcus_bromii_YE282]	Ruminococcus_bromii_YE282
Org1_Gene1249	unique	765	2595399206_IE32DRAFT_01294_putative_efflux_protein,_MATE_family_[Ruminococcus_bromii_YE282]	Ruminococcus_bromii_YE282
Org1_Gene125	unique	1222	2595398051_IE32DRAFT_00132_hypothetical_protein_[Ruminococcus_bromii_YE282]	Ruminococcus_bromii_YE282
Org1_Gene1250	unique	656	2595399207_IE32DRAFT_01295_Permeases_[Ruminococcus_bromii_YE282]	Ruminococcus_bromii_YE282
Org1_Gene1251	unique	3900	2595399209_IE32DRAFT_01297_hypothetical_protein_[Ruminococcus_bromii_YE282]	Ruminococcus_bromii_YE282
Org1_Gene1253	unique	1146	2595399211_IE32DRAFT_01299_Coenzyme_F420_hydrogenase/dehydrogenase,_beta_subunit_C_terminus/4Fe-4S_dicluster_domain_[Ruminococcus_bro	Ruminococcus_bromii_YE282
Org1_Gene1254	unique	1265	2595399212_IE32DRAFT_01300_Polysaccharide_pyruvyl_transferase_[Ruminococcus_bromii_YE282]	Ruminococcus_bromii_YE282

Org1_Gene1258	unique	820	2595399216_IE32DRAFT_01304_Lipid_A_core_-_O-antigen_ligase_and_related_enzymes_[Ruminococcus_bromii_YE282]	Ruminococcus_bromii_YE282
Org1_Gene126	unique	3310	2595398052_IE32DRAFT_00133_hypothetical_protein_[Ruminococcus_bromii_YE282]	Ruminococcus_bromii_YE282
Org1_Gene1260	unique	1044	2595399218_IE32DRAFT_01306_Coenzyme_F420-reducing_hydrogenase,_beta_subunit_[Ruminococcus_bromii_YE282]	Ruminococcus_bromii_YE282
Org1_Gene1269	unique	2469	2595399227_IE32DRAFT_01315_Sugar_transferases_involved_in_lipopolysaccharide_synthesis_[Ruminococcus_bromii_YE282]	Ruminococcus_bromii_YE282
Org1_Gene127	unique	1013	2595398053_IE32DRAFT_00134_hypothetical_protein_[Ruminococcus_bromii_YE282]	Ruminococcus_bromii_YE282
Org1_Gene1270	unique	2384	2595399228_IE32DRAFT_01316_capsular_exopolysaccharide_family_[Ruminococcus_bromii_YE282]	Ruminococcus_bromii_YE282
Org1_Gene1271	unique	2371	2595399229_IE32DRAFT_01317_Capsular_polysaccharide_biosynthesis_protein_[Ruminococcus_bromii_YE282]	Ruminococcus_bromii_YE282
Org1_Gene1274	unique	3018	2595399232_IE32DRAFT_01320_Diacylglycerol_kinase_[Ruminococcus_bromii_YE282]	Ruminococcus_bromii_YE282
Org1_Gene1276	unique	3342	2595399234_IE32DRAFT_01322_ADP-ribose_pyrophosphatase_[Ruminococcus_bromii_YE282]	Ruminococcus_bromii_YE282
Org1_Gene1277	unique	3253	2595399235_IE32DRAFT_01323_Acetyltransferase_(GNAT)_domain_[Ruminococcus_bromii_YE282]	Ruminococcus_bromii_YE282
Org1_Gene128	unique	477	2595398054_IE32DRAFT_00135_hypothetical_protein_[Ruminococcus_bromii_YE282]	Ruminococcus_bromii_YE282
Org1_Gene1286	unique	3104	2595399244_IE32DRAFT_01332_Phosphopantetheine_adenylyltransferase_(EC_2.7.7.3)_[Ruminococcus_bromii_YE282]	Ruminococcus_bromii_YE282
Org1_Gene1288	unique	3383	2595399246_IE32DRAFT_01334_mraZ_protein_[Ruminococcus_bromii_YE282]	Ruminococcus_bromii_YE282
Org1_Gene129	unique	333	2595398055_IE32DRAFT_00136_Terminase-like_family_[Ruminococcus_bromii_YE282]	Ruminococcus_bromii_YE282
Org1_Gene1290	unique	2858	2595399248_IE32DRAFT_01336_hypothetical_protein_[Ruminococcus_bromii_YE282]	Ruminococcus_bromii_YE282
Org1_Gene1296	unique	694	2595399254_IE32DRAFT_01342_Cell_division_septal_protein_[Ruminococcus_bromii_YE282]	Ruminococcus_bromii_YE282
Org1_Gene1298	unique	1344	2595399256_IE32DRAFT_01344_Membrane_proteins_related_to_metalloendopeptidases_[Ruminococcus_bromii_YE282]	Ruminococcus_bromii_YE282
Org1_Gene130	unique	1674	2595398056_IE32DRAFT_00137_hypothetical_protein_[Ruminococcus_bromii_YE282]	Ruminococcus_bromii_YE282
Org1_Gene1300	unique	1001	2595399258_IE32DRAFT_01346_DHHW_protein_[Ruminococcus_bromii_YE282]	Ruminococcus_bromii_YE282
Org1_Gene1301	unique	3999	2595399259_IE32DRAFT_01347_hypothetical_protein_[Ruminococcus_bromii_YE282]	Ruminococcus_bromii_YE282
Org1_Gene1303	unique	2228	2595399261_IE32DRAFT_01349_Methyltransferase_domain_[Ruminococcus_bromii_YE282]	Ruminococcus_bromii_YE282
Org1_Gene131	unique	2770	2595398057_IE32DRAFT_00138_hypothetical_protein_[Ruminococcus_bromii_YE282]	Ruminococcus_bromii_YE282
Org1_Gene1314	unique	669	2595399276_IE32DRAFT_01364_Transposase_domain_(DUF772)/Transposase_DDE_domain_[Ruminococcus_bromii_YE282]	Ruminococcus_bromii_YE282
Org1_Gene1316	unique	1558	2595399278_IE32DRAFT_01366_Uncharacterized_protein_conserved_in_bacteria_[Ruminococcus_bromii_YE282]	Ruminococcus_bromii_YE282
Org1_Gene1317	unique	3176	2595399279_IE32DRAFT_01367_Stage_III_sporulation_protein_AB_(spore_III_AB)_[Ruminococcus_bromii_YE282]	Ruminococcus_bromii_YE282
Org1_Gene1319	unique	3497	2595399281_IE32DRAFT_01369_Stage_III_sporulation_protein_AC/AD_protein_family_[Ruminococcus_bromii_YE282]	Ruminococcus_bromii_YE282
Org1_Gene132	unique	3860	2595398058_IE32DRAFT_00139_Bacterial_nucleoid_DNA-binding_protein_[Ruminococcus_bromii_YE282]	Ruminococcus_bromii_YE282
Org1_Gene1320	unique	1200	2595399282_IE32DRAFT_01370_Stage_III_sporulation_protein_AE_(spore_III_AE)_[Ruminococcus_bromii_YE282]	Ruminococcus_bromii_YE282
Org1_Gene1321	unique	3170	2595399283_IE32DRAFT_01371_Stage_III_sporulation_protein_AF_(Spore_III_AF)_[Ruminococcus_bromii_YE282]	Ruminococcus_bromii_YE282
Org1_Gene1323	unique	2895	2595399285_IE32DRAFT_01373_SpoIIIAH-like_protein_[Ruminococcus_bromii_YE282]	Ruminococcus_bromii_YE282
Org1_Gene1324	unique	3303	2595399286_IE32DRAFT_01374_NusB_antitermination_factor_[Ruminococcus_bromii_YE282]	Ruminococcus_bromii_YE282
Org1_Gene1326	unique	1006	2595399288_IE32DRAFT_01376_Exodeoxyribonuclease_VII_large_subunit_(EC_3.1.11.6)_[Ruminococcus_bromii_YE282]	Ruminococcus_bromii_YE282
Org1_Gene1328	unique	1442	2595399290_IE32DRAFT_01378_ABC-type_uncharacterized_transport_system,_periplasmic_component_[Ruminococcus_bromii_YE282]	Ruminococcus_bromii_YE282
Org1_Gene133	unique	1012	2595398059_IE32DRAFT_00140_Calcineurin-like_phosphoesterase_superfamily_domain_[Ruminococcus_bromii_YE282]	Ruminococcus_bromii_YE282
Org1_Gene1332	unique	2992	2595399294_IE32DRAFT_01382_hypothetical_protein_[Ruminococcus_bromii_YE282]	Ruminococcus_bromii_YE282
Org1_Gene1336	unique	3068	2595399298_IE32DRAFT_01386_hypothetical_protein_[Ruminococcus_bromii_YE282]	Ruminococcus_bromii_YE282
Org1_Gene1337	unique	974	2595399299_IE32DRAFT_01387_Uncharacterised_nucleotidyltransferase_[Ruminococcus_bromii_YE282]	Ruminococcus_bromii_YE282
Org1_Gene1338	unique	443	2595399300_IE32DRAFT_01388_Dolichyl-phosphate-mannose-protein_mannosyltransferase_[Ruminococcus_bromii_YE282]	Ruminococcus_bromii_YE282
Org1_Gene134	unique	2615	2595398060_IE32DRAFT_00141_Protease_subunit_of_ATP-dependent_Clp_proteases_[Ruminococcus_bromii_YE282]	Ruminococcus_bromii_YE282
Org1_Gene1340	unique	3843	2595399302_IE32DRAFT_01390_Protein_of_unknown_function_(DUF1292)_[Ruminococcus_bromii_YE282]	Ruminococcus_bromii_YE282
Org1_Gene1342	unique	618	2595399304_IE32DRAFT_01392_O-antigen_ligase_like_membrane_protein_[Ruminococcus_bromii_YE282]	Ruminococcus_bromii_YE282
Org1_Gene1344	unique	3135	2595399306_IE32DRAFT_01394_hypothetical_protein_[Ruminococcus_bromii_YE282]	Ruminococcus_bromii_YE282
Org1_Gene1348	unique	2649	2595399310_IE32DRAFT_01398_stage_II_sporulation_protein_R_[Ruminococcus_bromii_YE282]	Ruminococcus_bromii_YE282
Org1_Gene1349	unique	59	2595399311_IE32DRAFT_01399_ATP-dependent_nuclease,_subunit_B_[Ruminococcus_bromii_YE282]	Ruminococcus_bromii_YE282
Org1_Gene135	unique	649	2595398061_IE32DRAFT_00142_Predicted_exonuclease_of_the_beta-lactamase_fold_involved_in_RNA_processing_[Ruminococcus_bromii_YE282]	Ruminococcus_bromii_YE282
Org1_Gene1350	unique	48	2595399312_IE32DRAFT_01400_DNA_helicase/exodeoxyribonuclease_V,_subunit_A_(EC_3.1.11.5)_[Ruminococcus_bromii_YE282]	Ruminococcus_bromii_YE282
Org1_Gene1352	unique	2248	2595399314_IE32DRAFT_01402_Methyltransferase_domain_[Ruminococcus_bromii_YE282]	Ruminococcus_bromii_YE282
Org1_Gene1354	unique	2906	2595399316_IE32DRAFT_01404_Predicted_acetyltransferase_[Ruminococcus_bromii_YE282]	Ruminococcus_bromii_YE282
Org1_Gene1355	unique	1772	2595399317_IE32DRAFT_01405_Streptomycin_adenylyltransferase_[Ruminococcus_bromii_YE282]	Ruminococcus_bromii_YE282
Org1_Gene1356	unique	2157	2595399318_IE32DRAFT_01406_Phosphorylase_superfamily_[Ruminococcus_bromii_YE282]	Ruminococcus_bromii_YE282
Org1_Gene1359	unique	1018	2595399321_IE32DRAFT_01409_RecA-family_ATPase_[Ruminococcus_bromii_YE282]	Ruminococcus_bromii_YE282
Org1_Gene136	unique	4350	2595398062_IE32DRAFT_00143_hypothetical_protein_[Ruminococcus_bromii_YE282]	Ruminococcus_bromii_YE282
Org1_Gene1360	unique	3694	2595399322_IE32DRAFT_01410_hypothetical_protein_[Ruminococcus_bromii_YE282]	Ruminococcus_bromii_YE282
Org1_Gene1362	unique	4257	2595399324_IE32DRAFT_01412_hypothetical_protein_[Ruminococcus_bromii_YE282]	Ruminococcus_bromii_YE282
Org1_Gene137	unique	2546	2595398064_IE32DRAFT_00145_hypothetical_protein_[Ruminococcus_bromii_YE282]	Ruminococcus_bromii_YE282
Org1_Gene1376	unique	630	2595399339_IE32DRAFT_01427_Predicted_membrane_protein_[Ruminococcus_bromii_YE282]	Ruminococcus_bromii_YE282
Org1_Gene1378	unique	3279	2595399341_IE32DRAFT_01429_Membrane_protein_implicated_in_regulation_of_membrane_protease_activity_[Ruminococcus_bromii_YE282]	Ruminococcus_bromii_YE282
Org1_Gene1379	unique	2655	2595399342_IE32DRAFT_01430_Domain_of_unknown_function_(DUF4364)_[Ruminococcus_bromii_YE282]	Ruminococcus_bromii_YE282
Org1_Gene138	unique	1010	2595398068_IE32DRAFT_00149_hypothetical_protein_[Ruminococcus_bromii_YE282]	Ruminococcus_bromii_YE282
Org1_Gene1380	unique	3986	2595399343_IE32DRAFT_01431_SigmaK-factor_processing_regulatory_protein_BofA_[Ruminococcus_bromii_YE282]	Ruminococcus_bromii_YE282
Org1_Gene1389	unique	428	2595399352_IE32DRAFT_01440_Leucine_rich_repeats_(6_copies)_[Ruminococcus_bromii_YE282]	Ruminococcus_bromii_YE282
Org1_Gene139	unique	1799	2595398069_IE32DRAFT_00150_hypothetical_protein_[Ruminococcus_bromii_YE282]	Ruminococcus_bromii_YE282
Org1_Gene1390	unique	542	2595399353_IE32DRAFT_01441_hypothetical_protein_[Ruminococcus_bromii_YE282]	Ruminococcus_bromii_YE282
Org1_Gene1391	unique	2864	2595399354_IE32DRAFT_01442_MAF_protein_[Ruminococcus_bromii_YE282]	Ruminococcus_bromii_YE282
Org1_Gene1392	unique	1603	2595399355_IE32DRAFT_01443_Transcriptional_regulator_[Ruminococcus_bromii_YE282]	Ruminococcus_bromii_YE282
Org1_Gene1394	unique	3049	2595399357_IE32DRAFT_01445_hypothetical_protein_[Ruminococcus_bromii_YE282]	Ruminococcus_bromii_YE282
Org1_Gene1396	unique	3348	2595399359_IE32DRAFT_01447_hypothetical_protein_[Ruminococcus_bromii_YE282]	Ruminococcus_bromii_YE282
Org1_Gene1397	unique	426	2595399360_IE32DRAFT_01448_MobA/MobL_family_[Ruminococcus_bromii_YE282]	Ruminococcus_bromii_YE282
Org1_Gene1398	unique	4061	2595399361_IE32DRAFT_01449_hypothetical_protein_[Ruminococcus_bromii_YE282]	Ruminococcus_bromii_YE282
Org1_Gene1399	unique	2094	2595399362_IE32DRAFT_01450_ABC-2_family_transporter_protein_[Ruminococcus_bromii_YE282]	Ruminococcus_bromii_YE282
Org1_Gene140	unique	4029	2595398070_IE32DRAFT_00151_hypothetical_protein_[Ruminococcus_bromii_YE282]	Ruminococcus_bromii_YE282
Org1_Gene1400	unique	2035	2595399363_IE32DRAFT_01451_ABC-2_family_transporter_protein_[Ruminococcus_bromii_YE282]	Ruminococcus_bromii_YE282
Org1_Gene1401	unique	1745	2595399364_IE32DRAFT_01452_ABC-type_multidrug_transport_system,_ATPase_component_[Ruminococcus_bromii_YE282]	Ruminococcus_bromii_YE282
Org1_Gene1402	unique	2642	2595399365_IE32DRAFT_01453_transcriptional_regulator,_TetR_family_[Ruminococcus_bromii_YE282]	Ruminococcus_bromii_YE282
Org1_Gene1403	unique	586	2595399366_IE32DRAFT_01454_Site-specific_recombinases,_DNA_invertase_Pin_homologs_[Ruminococcus_bromii_YE282]	Ruminococcus_bromii_YE282
Org1_Gene1404	unique	2063	2595399368_IE32DRAFT_01456_ABC-2_family_transporter_protein_[Ruminococcus_bromii_YE282]	Ruminococcus_bromii_YE282
Org1_Gene1405	unique	2507	2595399369_IE32DRAFT_01457_ABC_transporter_[Ruminococcus_bromii_YE282]	Ruminococcus_bromii_YE282
Org1_Gene1406	unique	3593	2595399370_IE32DRAFT_01458_Helix-turn-helix_[Ruminococcus_bromii_YE282]	Ruminococcus_bromii_YE282
Org1_Gene1407	unique	4404	2595399371_IE32DRAFT_01459_cyclic_lactone_autoinducer_peptide_[Ruminococcus_bromii_YE282]	Ruminococcus_bromii_YE282
Org1_Gene1408	unique	2756	2595399372_IE32DRAFT_01460_Membrane_protein_putatively_involved_in_post-translational_modification_of_the_autoinducing_quorum-sensing_pept	Ruminococcus_bromii_YE282
Org1_Gene1409	unique	801	2595399373_IE32DRAFT_01461_GHKL_domain_[Ruminococcus_bromii_YE282]	Ruminococcus_bromii_YE282
Org1_Gene141	unique	98	2595398071_IE32DRAFT_00152_hypothetical_protein_[Ruminococcus_bromii_YE282]	Ruminococcus_bromii_YE282
Org1_Gene1410	unique	2382	2595399374_IE32DRAFT_01462_two_component_transcriptional_regulator,_LytTR_family_[Ruminococcus_bromii_YE282]	Ruminococcus_bromii_YE282
Org1_Gene1411	unique	161	2595399375_IE32DRAFT_01463_Alpha_amylase,_catalytic_domain_[Ruminococcus_bromii_YE282]	Ruminococcus_bromii_YE282
Org1_Gene1416	unique	111	2595399380_IE32DRAFT_01468_Uncharacterized_low-complexity_proteins_[Ruminococcus_bromii_YE282]	Ruminococcus_bromii_YE282
Org1_Gene1417	unique	2361	2595399381_IE32DRAFT_01469_hypothetical_protein_[Ruminococcus_bromii_YE282]	Ruminococcus_bromii_YE282
Org1_Gene1418	unique	1724	2595399382_IE32DRAFT_01470_Dockerin_type_I_repeat/Leucine_rich_repeats_(6_copies)_[Ruminococcus_bromii_YE282]	Ruminococcus_bromii_YE282
Org1_Gene1419	unique	4406	2595399383_IE32DRAFT_01471_hypothetical_protein_[Ruminococcus_bromii_YE282]	Ruminococcus_bromii_YE282
Org1_Gene142	unique	2989	2595398072_IE32DRAFT_00153_hypothetical_protein_[Ruminococcus_bromii_YE282]	Ruminococcus_bromii_YE282
Org1_Gene1420	unique	29	2595399384_IE32DRAFT_01472_fibro-slime_domain_[Ruminococcus_bromii_YE282]	Ruminococcus_bromii_YE282
Org1_Gene1423	unique	331	2595399387_IE32DRAFT_01475_Dockerin_type_I_repeat_[Ruminococcus_bromii_YE282]	Ruminococcus_bromii_YE282
Org1_Gene1427	unique	2918	2595399391_IE32DRAFT_01479_hypothetical_protein_[Ruminococcus_bromii_YE282]	Ruminococcus_bromii_YE282
Org1_Gene1428	unique	1223	2595399392_IE32DRAFT_01480_stage_II_sporulation_protein_P_[Ruminococcus_bromii_YE282]	Ruminococcus_bromii_YE282
Org1_Gene143	unique	3768	2595398073_IE32DRAFT_00154_hypothetical_protein_[Ruminococcus_bromii_YE282]	Ruminococcus_bromii_YE282
Org1_Gene1430	unique	479	2595399394_IE32DRAFT_01482_Papain_family_cysteine_protease_[Ruminococcus_bromii_YE282]	Ruminococcus_bromii_YE282
Org1_Gene1431	unique	2813	2595399395_IE32DRAFT_01483_hypothetical_protein_[Ruminococcus_bromii_YE282]	Ruminococcus_bromii_YE282
Org1_Gene1433	unique	3430	2595399397_IE32DRAFT_01485_hypothetical_protein_[Ruminococcus_bromii_YE282]	Ruminococcus_bromii_YE282
Org1_Gene1434	unique	798	2595399398_IE32DRAFT_01486_Helicase_conserved_C-terminal_domain/SNF2_family_N-terminal_domain_[Ruminococcus_bromii_YE282]	Ruminococcus_bromii_YE282
Org1_Gene1435	unique	3946	2595399399_IE32DRAFT_01487_hypothetical_protein_[Ruminococcus_bromii_YE282]	Ruminococcus_bromii_YE282
Org1_Gene1436	unique	3274	2595399400_IE32DRAFT_01488_hypothetical_protein_[Ruminococcus_bromii_YE282]	Ruminococcus_bromii_YE282
Org1_Gene1437	unique	368	2595399401_IE32DRAFT_01489_PD-(D/E)XK_nuclease_superfamily/AAA_domain_[Ruminococcus_bromii_YE282]	Ruminococcus_bromii_YE282
Org1_Gene1438	unique	3233	2595399402_IE32DRAFT_01490_Protein_of_unknown_function_(DUF669)_[Ruminococcus_bromii_YE282]	Ruminococcus_bromii_YE282
Org1_Gene1439	unique	421	2595399403_IE32DRAFT_01491_hypothetical_protein_[Ruminococcus_bromii_YE282]	Ruminococcus_bromii_YE282
Org1_Gene144	unique	3774	2595398074_IE32DRAFT_00155_hypothetical_protein_[Ruminococcus_bromii_YE282]	Ruminococcus_bromii_YE282
Org1_Gene1440	unique	3752	2595399404_IE32DRAFT_01492_hypothetical_protein_[Ruminococcus_bromii_YE282]	Ruminococcus_bromii_YE282
Org1_Gene1441	unique	329	2595399405_IE32DRAFT_01493_phage/plasmid_primase,_P4_family,_C-terminal_domain_[Ruminococcus_bromii_YE282]	Ruminococcus_bromii_YE282
Org1_Gene1442	unique	4132	2595399406_IE32DRAFT_01494_hypothetical_protein_[Ruminococcus_bromii_YE282]	Ruminococcus_bromii_YE282
Org1_Gene1443	unique	4133	2595399407_IE32DRAFT_01495_hypothetical_protein_[Ruminococcus_bromii_YE282]	Ruminococcus_bromii_YE282
Org1_Gene1444	unique	3587	2595399408_IE32DRAFT_01496_hypothetical_protein_[Ruminococcus_bromii_YE282]	Ruminococcus_bromii_YE282
Org1_Gene1445	unique	4205	2595399409_IE32DRAFT_01497_hypothetical_protein_[Ruminococcus_bromii_YE282]	Ruminococcus_bromii_YE282
Org1_Gene1446	unique	3799	2595399410_IE32DRAFT_01498_hypothetical_protein_[Ruminococcus_bromii_YE282]	Ruminococcus_bromii_YE282
Org1_Gene1447	unique	3850	2595399411_IE32DRAFT_01499_hypothetical_protein_[Ruminococcus_bromii_YE282]	Ruminococcus_bromii_YE282
Org1_Gene1448	unique	4047	2595399412_IE32DRAFT_01500_hypothetical_protein_[Ruminococcus_bromii_YE282]	Ruminococcus_bromii_YE282
Org1_Gene1449	unique	4111	2595399413_IE32DRAFT_01501_hypothetical_protein_[Ruminococcus_bromii_YE282]	Ruminococcus_bromii_YE282
Org1_Gene145	unique	4397	2595398075_IE32DRAFT_00156_hypothetical_protein_[Ruminococcus_bromii_YE282]	Ruminococcus_bromii_YE282
Org1_Gene1450	unique	4284	2595399414_IE32DRAFT_01502_hypothetical_protein_[Ruminococcus_bromii_YE282]	Ruminococcus_bromii_YE282
Org1_Gene1451	unique	3366	2595399415_IE32DRAFT_01503_hypothetical_protein_[Ruminococcus_bromii_YE282]	Ruminococcus_bromii_YE282
Org1_Gene1452	unique	3098	2595399416_IE32DRAFT_01504_Terminase_small_subunit_[Ruminococcus_bromii_YE282]	Ruminococcus_bromii_YE282
Org1_Gene1453	unique	939	2595399417_IE32DRAFT_01505_phage_terminase,_large_subunit,_PBSX_family_[Ruminococcus_bromii_YE282]	Ruminococcus_bromii_YE282
Org1_Gene1454	unique	632	2595399418_IE32DRAFT_01506_Phage_portal_protein,_SPP1_Gp6-like_[Ruminococcus_bromii_YE282]	Ruminococcus_bromii_YE282
Org1_Gene1455	unique	474	2595399419_IE32DRAFT_01507_Phage_Mu_protein_F_like_protein_[Ruminococcus_bromii_YE282]	Ruminococcus_bromii_YE282
Org1_Gene1456	unique	4387	2595399420_IE32DRAFT_01508_hypothetical_protein_[Ruminococcus_bromii_YE282]	Ruminococcus_bromii_YE282

Org1_Gene1457	unique	2726	2595399421_IE32DRAFT_01509_Phage_minor_structural_protein_GP20_[Ruminococcus_bromii_YE282]	Ruminococcus_bromii_YE282
Org1_Gene1458	unique	1422	2595399422_IE32DRAFT_01510_hypothetical_protein_[Ruminococcus_bromii_YE282]	Ruminococcus_bromii_YE282
Org1_Gene1459	unique	4055	2595399423_IE32DRAFT_01511_hypothetical_protein_[Ruminococcus_bromii_YE282]	Ruminococcus_bromii_YE282
Org1_Gene146	unique	3457	2595398076_IE32DRAFT_00157_hypothetical_protein_[Ruminococcus_bromii_YE282]	Ruminococcus_bromii_YE282
Org1_Gene1460	unique	2963	2595399424_IE32DRAFT_01512_hypothetical_protein_[Ruminococcus_bromii_YE282]	Ruminococcus_bromii_YE282
Org1_Gene1461	unique	3384	2595399425_IE32DRAFT_01513_hypothetical_protein_[Ruminococcus_bromii_YE282]	Ruminococcus_bromii_YE282
Org1_Gene1462	unique	3423	2595399426_IE32DRAFT_01514_hypothetical_protein_[Ruminococcus_bromii_YE282]	Ruminococcus_bromii_YE282
Org1_Gene1463	unique	3558	2595399427_IE32DRAFT_01515_hypothetical_protein_[Ruminococcus_bromii_YE282]	Ruminococcus_bromii_YE282
Org1_Gene1464	unique	2324	2595399428_IE32DRAFT_01516_hypothetical_protein_[Ruminococcus_bromii_YE282]	Ruminococcus_bromii_YE282
Org1_Gene1465	unique	3440	2595399429_IE32DRAFT_01517_hypothetical_protein_[Ruminococcus_bromii_YE282]	Ruminococcus_bromii_YE282
Org1_Gene1466	unique	3907	2595399430_IE32DRAFT_01518_hypothetical_protein_[Ruminococcus_bromii_YE282]	Ruminococcus_bromii_YE282
Org1_Gene1467	unique	132	2595399431_IE32DRAFT_01519_Phage-related_protein_[Ruminococcus_bromii_YE282]	Ruminococcus_bromii_YE282
Org1_Gene1468	unique	2339	2595399432_IE32DRAFT_01520_hypothetical_protein_[Ruminococcus_bromii_YE282]	Ruminococcus_bromii_YE282
Org1_Gene1469	unique	196	2595399433_IE32DRAFT_01521_phage_minor_structural_protein,_N-terminal_region_[Ruminococcus_bromii_YE282]	Ruminococcus_bromii_YE282
Org1_Gene147	unique	2820	2595398077_IE32DRAFT_00158_hypothetical_protein_[Ruminococcus_bromii_YE282]	Ruminococcus_bromii_YE282
Org1_Gene1470	unique	4418	2595399434_IE32DRAFT_01522_hypothetical_protein_[Ruminococcus_bromii_YE282]	Ruminococcus_bromii_YE282
Org1_Gene1471	unique	4016	2595399435_IE32DRAFT_01523_hypothetical_protein_[Ruminococcus_bromii_YE282]	Ruminococcus_bromii_YE282
Org1_Gene1472	unique	244	2595399436_IE32DRAFT_01524_hypothetical_protein_[Ruminococcus_bromii_YE282]	Ruminococcus_bromii_YE282
Org1_Gene1473	unique	3966	2595399437_IE32DRAFT_01525_hypothetical_protein_[Ruminococcus_bromii_YE282]	Ruminococcus_bromii_YE282
Org1_Gene1474	unique	4422	2595399438_IE32DRAFT_01526_hypothetical_protein_[Ruminococcus_bromii_YE282]	Ruminococcus_bromii_YE282
Org1_Gene1475	unique	4327	2595399439_IE32DRAFT_01527_hypothetical_protein_[Ruminococcus_bromii_YE282]	Ruminococcus_bromii_YE282
Org1_Gene1476	unique	3855	2595399440_IE32DRAFT_01528_hypothetical_protein_[Ruminococcus_bromii_YE282]	Ruminococcus_bromii_YE282
Org1_Gene1477	unique	3925	2595399441_IE32DRAFT_01529_hypothetical_protein_[Ruminococcus_bromii_YE282]	Ruminococcus_bromii_YE282
Org1_Gene1478	unique	2073	2595399442_IE32DRAFT_01530_N-acetylmuramoyl-L-alanine_amidase/Bacterial_SH3_domain_[Ruminococcus_bromii_YE282]	Ruminococcus_bromii_YE282
Org1_Gene1479	unique	1950	2595399443_IE32DRAFT_01531_3-methyl-2-oxobutanoate_hydroxymethyltransferase_[Ruminococcus_bromii_YE282]	Ruminococcus_bromii_YE282
Org1_Gene148	unique	4214	2595398078_IE32DRAFT_00159_hypothetical_protein_[Ruminococcus_bromii_YE282]	Ruminococcus_bromii_YE282
Org1_Gene1480	unique	1903	2595399444_IE32DRAFT_01532_pantoate--beta-alanine_ligase_[Ruminococcus_bromii_YE282]	Ruminococcus_bromii_YE282
Org1_Gene1481	unique	3505	2595399445_IE32DRAFT_01533_L-aspartate_1-decarboxylase_(EC_4.1.1.11)_[Ruminococcus_bromii_YE282]	Ruminococcus_bromii_YE282
Org1_Gene1482	unique	1022	2595399446_IE32DRAFT_01534_phosphopantothenoylcysteine_decarboxylase_/_phosphopantothenate--cysteine_ligase_[Ruminococcus_bromii_YE282]	Ruminococcus_bromii_YE282
Org1_Gene1483	unique	2550	2595399447_IE32DRAFT_01535_Site-specific_recombinases,_DNA_invertase_Pin_homologs_[Ruminococcus_bromii_YE282]	Ruminococcus_bromii_YE282
Org1_Gene1484	unique	3041	2595399448_IE32DRAFT_01536_UvrD/REP_helicase_N-terminal_domain_[Ruminococcus_bromii_YE282]	Ruminococcus_bromii_YE282
Org1_Gene1487	unique	338	2595399451_IE32DRAFT_01539_KAP_family_P-loop_domain_[Ruminococcus_bromii_YE282]	Ruminococcus_bromii_YE282
Org1_Gene1488	unique	2451	2595399452_IE32DRAFT_01540_hypothetical_protein_[Ruminococcus_bromii_YE282]	Ruminococcus_bromii_YE282
Org1_Gene1489	unique	722	2595399453_IE32DRAFT_01541_hypothetical_protein_[Ruminococcus_bromii_YE282]	Ruminococcus_bromii_YE282
Org1_Gene149	unique	4188	2595398079_IE32DRAFT_00160_hypothetical_protein_[Ruminococcus_bromii_YE282]	Ruminococcus_bromii_YE282
Org1_Gene1490	unique	2269	2595399454_IE32DRAFT_01542_Mg-dependent_DNase_[Ruminococcus_bromii_YE282]	Ruminococcus_bromii_YE282
Org1_Gene1491	unique	2004	2595399455_IE32DRAFT_01543_hypothetical_protein_[Ruminococcus_bromii_YE282]	Ruminococcus_bromii_YE282
Org1_Gene1492	unique	17	2595399456_IE32DRAFT_01544_Glycosidases_[Ruminococcus_bromii_YE282]	Ruminococcus_bromii_YE282
Org1_Gene1496	unique	489	2595399460_IE32DRAFT_01548_Uncharacterized_vancomycin_resistance_protein_[Ruminococcus_bromii_YE282]	Ruminococcus_bromii_YE282
Org1_Gene1497	unique	1184	2595399461_IE32DRAFT_01549_UDP-N-acetylglucosamine:LPS_N-acetylglucosamine_transferase_[Ruminococcus_bromii_YE282]	Ruminococcus_bromii_YE282
Org1_Gene1498	unique	188	2595399462_IE32DRAFT_01550_Stage_II_sporulation_protein_E_(SpoIIE)_[Ruminococcus_bromii_YE282]	Ruminococcus_bromii_YE282
Org1_Gene15	unique	1243	2595397936_IE32DRAFT_00017_DnaD_and_phage-associated_domain_[Ruminococcus_bromii_YE282]	Ruminococcus_bromii_YE282
Org1_Gene150	unique	4362	2595398080_IE32DRAFT_00161_hypothetical_protein_[Ruminococcus_bromii_YE282]	Ruminococcus_bromii_YE282
Org1_Gene1503	unique	322	2595399467_IE32DRAFT_01555_alpha-1,4-glucan:alpha-1,4-glucan_6-glycosyltransferase_[Ruminococcus_bromii_YE282]	Ruminococcus_bromii_YE282
Org1_Gene1508	unique	2283	2595399472_IE32DRAFT_01560_hypothetical_protein_[Ruminococcus_bromii_YE282]	Ruminococcus_bromii_YE282
Org1_Gene151	unique	3078	2595398081_IE32DRAFT_00162_hypothetical_protein_[Ruminococcus_bromii_YE282]	Ruminococcus_bromii_YE282
Org1_Gene1510	unique	3396	2595399474_IE32DRAFT_01562_hypothetical_protein_[Ruminococcus_bromii_YE282]	Ruminococcus_bromii_YE282
Org1_Gene1512	unique	2024	2595399476_IE32DRAFT_01564_hypothetical_protein_[Ruminococcus_bromii_YE282]	Ruminococcus_bromii_YE282
Org1_Gene1513	unique	3718	2595399477_IE32DRAFT_01565_transcriptional_regulator,_HxlR_family_[Ruminococcus_bromii_YE282]	Ruminococcus_bromii_YE282
Org1_Gene1515	unique	2365	2595399479_IE32DRAFT_01567_Predicted_hydrolase_(HAD_superfamily)_[Ruminococcus_bromii_YE282]	Ruminococcus_bromii_YE282
Org1_Gene1517	unique	1617	2595399481_IE32DRAFT_01569_Mg2+_and_Co2+_transporters_[Ruminococcus_bromii_YE282]	Ruminococcus_bromii_YE282
Org1_Gene1519	unique	3044	2595399483_IE32DRAFT_01571_hypothetical_protein_[Ruminococcus_bromii_YE282]	Ruminococcus_bromii_YE282
Org1_Gene152	unique	4210	2595398082_IE32DRAFT_00163_hypothetical_protein_[Ruminococcus_bromii_YE282]	Ruminococcus_bromii_YE282
Org1_Gene1521	unique	1677	2595399485_IE32DRAFT_01573_Transcriptional_regulator_[Ruminococcus_bromii_YE282]	Ruminococcus_bromii_YE282
Org1_Gene1522	unique	3583	2595399486_IE32DRAFT_01574_Cobalamin-independent_synthase,_Catalytic_domain_[Ruminococcus_bromii_YE282]	Ruminococcus_bromii_YE282
Org1_Gene1523	unique	533	2595399487_IE32DRAFT_01575_Site-specific_recombinases,_DNA_invertase_Pin_homologs_[Ruminococcus_bromii_YE282]	Ruminococcus_bromii_YE282
Org1_Gene1524	unique	4085	2595399488_IE32DRAFT_01576_hypothetical_protein_[Ruminococcus_bromii_YE282]	Ruminococcus_bromii_YE282
Org1_Gene1525	unique	1000	2595399489_IE32DRAFT_01577_Relaxase/Mobilisation_nuclease_domain_[Ruminococcus_bromii_YE282]	Ruminococcus_bromii_YE282
Org1_Gene1526	unique	4131	2595399490_IE32DRAFT_01578_Bacterial_mobilisation_protein_(MobC)_[Ruminococcus_bromii_YE282]	Ruminococcus_bromii_YE282
Org1_Gene1527	unique	610	2595399491_IE32DRAFT_01579_Domain_of_unknown_function_(DUF927)_[Ruminococcus_bromii_YE282]	Ruminococcus_bromii_YE282
Org1_Gene1528	unique	3581	2595399492_IE32DRAFT_01580_hypothetical_protein_[Ruminococcus_bromii_YE282]	Ruminococcus_bromii_YE282
Org1_Gene1529	unique	3936	2595399493_IE32DRAFT_01581_hypothetical_protein_[Ruminococcus_bromii_YE282]	Ruminococcus_bromii_YE282
Org1_Gene153	unique	4385	2595398083_IE32DRAFT_00164_hypothetical_protein_[Ruminococcus_bromii_YE282]	Ruminococcus_bromii_YE282
Org1_Gene1530	unique	3924	2595399494_IE32DRAFT_01582_hypothetical_protein_[Ruminococcus_bromii_YE282]	Ruminococcus_bromii_YE282
Org1_Gene1531	unique	978	2595399495_IE32DRAFT_01583_hypothetical_protein_[Ruminococcus_bromii_YE282]	Ruminococcus_bromii_YE282
Org1_Gene1532	unique	2227	2595399496_IE32DRAFT_01584_Peptidase_C39_like_family_[Ruminococcus_bromii_YE282]	Ruminococcus_bromii_YE282
Org1_Gene1533	unique	1152	2595399497_IE32DRAFT_01585_hypothetical_protein_[Ruminococcus_bromii_YE282]	Ruminococcus_bromii_YE282
Org1_Gene1534	unique	2570	2595399498_IE32DRAFT_01586_hypothetical_protein_[Ruminococcus_bromii_YE282]	Ruminococcus_bromii_YE282
Org1_Gene1535	unique	2835	2595399499_IE32DRAFT_01587_RNA_polymerase_sigma_factor,_sigma-70_family_[Ruminococcus_bromii_YE282]	Ruminococcus_bromii_YE282
Org1_Gene1536	unique	2159	2595399500_IE32DRAFT_01588_Cobalamin-independent_synthase,_Catalytic_domain_[Ruminococcus_bromii_YE282]	Ruminococcus_bromii_YE282
Org1_Gene1537	unique	2904	2595399501_IE32DRAFT_01589_looped-hinge_helix_DNA_binding_domain,_AbrB_family_[Ruminococcus_bromii_YE282]	Ruminococcus_bromii_YE282
Org1_Gene1538	unique	887	2595399502_IE32DRAFT_01590_Gamma-glutamylcysteine_synthetase_[Ruminococcus_bromii_YE282]	Ruminococcus_bromii_YE282
Org1_Gene1539	unique	2131	2595399503_IE32DRAFT_01591_Predicted_glutamine_amidotransferase_[Ruminococcus_bromii_YE282]	Ruminococcus_bromii_YE282
Org1_Gene154	unique	4339	2595398084_IE32DRAFT_00165_hypothetical_protein_[Ruminococcus_bromii_YE282]	Ruminococcus_bromii_YE282
Org1_Gene1540	unique	4098	2595399504_IE32DRAFT_01592_hypothetical_protein_[Ruminococcus_bromii_YE282]	Ruminococcus_bromii_YE282
Org1_Gene1541	unique	1846	2595399505_IE32DRAFT_01593_hypothetical_protein_[Ruminococcus_bromii_YE282]	Ruminococcus_bromii_YE282
Org1_Gene1542	unique	3930	2595399506_IE32DRAFT_01594_hypothetical_protein_[Ruminococcus_bromii_YE282]	Ruminococcus_bromii_YE282
Org1_Gene1543	unique	2702	2595399507_IE32DRAFT_01595_Phosphopantetheinyl_transferase_[Ruminococcus_bromii_YE282]	Ruminococcus_bromii_YE282
Org1_Gene1547	unique	2766	2595399511_IE32DRAFT_01599_hypothetical_protein_[Ruminococcus_bromii_YE282]	Ruminococcus_bromii_YE282
Org1_Gene1549	unique	110	2595399513_IE32DRAFT_01601_hypothetical_protein_[Ruminococcus_bromii_YE282]	Ruminococcus_bromii_YE282
Org1_Gene155	unique	2607	2595398085_IE32DRAFT_00166_hypothetical_protein_[Ruminococcus_bromii_YE282]	Ruminococcus_bromii_YE282
Org1_Gene1551	unique	2588	2595399515_IE32DRAFT_01603_hypothetical_protein_[Ruminococcus_bromii_YE282]	Ruminococcus_bromii_YE282
Org1_Gene1552	unique	2	2595399516_IE32DRAFT_01604_PKD_domain/Fibronectin_type_III_domain_[Ruminococcus_bromii_YE282]	Ruminococcus_bromii_YE282
Org1_Gene1553	unique	431	2595399517_IE32DRAFT_01605_hypothetical_protein_[Ruminococcus_bromii_YE282]	Ruminococcus_bromii_YE282
Org1_Gene1554	unique	1838	2595399518_IE32DRAFT_01606_Cohesin_domain_[Ruminococcus_bromii_YE282]	Ruminococcus_bromii_YE282
Org1_Gene1555	unique	1284	2595399519_IE32DRAFT_01607_hypothetical_protein_[Ruminococcus_bromii_YE282]	Ruminococcus_bromii_YE282
Org1_Gene1556	unique	871	2595399520_IE32DRAFT_01608_putative_efflux_protein,_MATE_family_[Ruminococcus_bromii_YE282]	Ruminococcus_bromii_YE282
Org1_Gene1557	unique	1786	2595399521_IE32DRAFT_01609_AraC-type_DNA-binding_domain-containing_proteins_[Ruminococcus_bromii_YE282]	Ruminococcus_bromii_YE282
Org1_Gene1558	unique	1037	2595399522_IE32DRAFT_01610_CHAP_domain/Bacterial_SH3_domain_[Ruminococcus_bromii_YE282]	Ruminococcus_bromii_YE282
Org1_Gene1559	unique	2153	2595399523_IE32DRAFT_01611_Trans-aconitate_methyltransferase_[Ruminococcus_bromii_YE282]	Ruminococcus_bromii_YE282
Org1_Gene156	unique	4153	2595398086_IE32DRAFT_00167_hypothetical_protein_[Ruminococcus_bromii_YE282]	Ruminococcus_bromii_YE282
Org1_Gene1563	unique	867	2595399527_IE32DRAFT_01615_folylpolyglutamate_synthase/dihydrofolate_synthase_[Ruminococcus_bromii_YE282]	Ruminococcus_bromii_YE282
Org1_Gene1565	unique	2233	2595399529_IE32DRAFT_01617_Predicted_O-methyltransferase_[Ruminococcus_bromii_YE282]	Ruminococcus_bromii_YE282
Org1_Gene1568	unique	1825	2595399532_IE32DRAFT_01620_DNA_polymerase_III,_delta_subunit_[Ruminococcus_bromii_YE282]	Ruminococcus_bromii_YE282
Org1_Gene1569	unique	3186	2595399533_IE32DRAFT_01621_phosphoesterase,_MJ0936_family_[Ruminococcus_bromii_YE282]	Ruminococcus_bromii_YE282
Org1_Gene157	unique	4261	2595398087_IE32DRAFT_00168_hypothetical_protein_[Ruminococcus_bromii_YE282]	Ruminococcus_bromii_YE282
Org1_Gene1570	unique	4030	2595399534_IE32DRAFT_01622_hypothetical_protein_[Ruminococcus_bromii_YE282]	Ruminococcus_bromii_YE282
Org1_Gene1571	unique	3594	2595399535_IE32DRAFT_01623_hypothetical_protein_[Ruminococcus_bromii_YE282]	Ruminococcus_bromii_YE282
Org1_Gene1572	unique	3786	2595399536_IE32DRAFT_01624_hypothetical_protein_[Ruminococcus_bromii_YE282]	Ruminococcus_bromii_YE282
Org1_Gene1575	unique	2661	2595399539_IE32DRAFT_01627_radical_SAM_enzyme,_TIGR04100_family_[Ruminococcus_bromii_YE282]	Ruminococcus_bromii_YE282
Org1_Gene1576	unique	2849	2595399540_IE32DRAFT_01628_Predicted_membrane_protein_[Ruminococcus_bromii_YE282]	Ruminococcus_bromii_YE282
Org1_Gene1577	unique	1887	2595399541_IE32DRAFT_01629_Pyridoxal/pyridoxine/pyridoxamine_kinase_[Ruminococcus_bromii_YE282]	Ruminococcus_bromii_YE282
Org1_Gene1578	unique	1615	2595399542_IE32DRAFT_01630_hypothetical_protein_[Ruminococcus_bromii_YE282]	Ruminococcus_bromii_YE282
Org1_Gene1579	unique	288	2595399543_IE32DRAFT_01631_CotH_protein_[Ruminococcus_bromii_YE282]	Ruminococcus_bromii_YE282
Org1_Gene158	unique	4209	2595398088_IE32DRAFT_00169_hypothetical_protein_[Ruminococcus_bromii_YE282]	Ruminococcus_bromii_YE282
Org1_Gene1581	unique	3058	2595399545_IE32DRAFT_01633_shikimate_kinase_(EC_2.7.1.71)_[Ruminococcus_bromii_YE282]	Ruminococcus_bromii_YE282
Org1_Gene1582	unique	2084	2595399546_IE32DRAFT_01634_Amidohydrolase_[Ruminococcus_bromii_YE282]	Ruminococcus_bromii_YE282
Org1_Gene1583	unique	1221	2595399547_IE32DRAFT_01635_ABC-type_nitrate/sulfonate/bicarbonate_transport_systems,_periplasmic_components_[Ruminococcus_bromii_YE282]	Ruminococcus_bromii_YE282
Org1_Gene1584	unique	1994	2595399548_IE32DRAFT_01636_ABC-type_nitrate/sulfonate/bicarbonate_transport_system,_ATPase_component_[Ruminococcus_bromii_YE282]	Ruminococcus_bromii_YE282
Org1_Gene1585	unique	1639	2595399549_IE32DRAFT_01637_ABC-type_nitrate/sulfonate/bicarbonate_transport_system,_permease_component_[Ruminococcus_bromii_YE282]	Ruminococcus_bromii_YE282
Org1_Gene1586	unique	2960	2595399550_IE32DRAFT_01638_2-oxoacid:acceptor_oxidoreductase,_gamma_subunit,_pyruvate/2-ketoisovalerate_family_[Ruminococcus_bromii_YE282]	Ruminococcus_bromii_YE282
Org1_Gene1587	unique	3866	2595399551_IE32DRAFT_01639_2-oxoacid:acceptor_oxidoreductase,_delta_subunit,_pyruvate/2-ketoisovalerate_family_[Ruminococcus_bromii_YE282]	Ruminococcus_bromii_YE282
Org1_Gene1588	unique	1091	2595399552_IE32DRAFT_01640_Pyruvate:ferredoxin_oxidoreductase_and_related_2-oxoacid:ferredoxin_oxidoreductases,_alpha_subunit_[Ruminococcus_	Ruminococcus_bromii_YE282
Org1_Gene1589	unique	1701	2595399553_IE32DRAFT_01641_Pyruvate:ferredoxin_oxidoreductase_and_related_2-oxoacid:ferredoxin_oxidoreductases,_beta_subunit_[Ruminococcus_	Ruminococcus_bromii_YE282
Org1_Gene159	unique	3955	2595398089_IE32DRAFT_00170_hypothetical_protein_[Ruminococcus_bromii_YE282]	Ruminococcus_bromii_YE282
Org1_Gene1590	unique	775	2595399554_IE32DRAFT_01642_Coenzyme_F390_synthetase_[Ruminococcus_bromii_YE282]	Ruminococcus_bromii_YE282
Org1_Gene1591	unique	1093	2595399555_IE32DRAFT_01643_Aspartate/tyrosine/aromatic_aminotransferase_[Ruminococcus_bromii_YE282]	Ruminococcus_bromii_YE282
Org1_Gene1592	unique	4211	2595399556_IE32DRAFT_01644_hypothetical_protein_[Ruminococcus_bromii_YE282]	Ruminococcus_bromii_YE282
Org1_Gene16	unique	1455	2595397937_IE32DRAFT_00018_IstB-like_ATP_binding_protein_[Ruminococcus_bromii_YE282]	Ruminococcus_bromii_YE282
Org1_Gene160	unique	3147	2595398090_IE32DRAFT_00171_hypothetical_protein_[Ruminococcus_bromii_YE282]	Ruminococcus_bromii_YE282
Org1_Gene1605	unique	2514	2595399569_IE32DRAFT_01657_cAMP-binding_proteins_-_catabolite_gene_activator_and_regulatory_subunit_of_cAMP-dependent_protein_kinases_[Ru	Ruminococcus_bromii_YE282
Org1_Gene1606	unique	2330	2595399570_IE32DRAFT_01658_4Fe-4S_binding_domain_[Ruminococcus_bromii_YE282]	Ruminococcus_bromii_YE282

Org1_Gene1607	unique	4074	2595399571_IE32DRAFT_01659_Domain_of_unknown_function_(DUF1858)_[Ruminococcus_bromii_YE282]	Ruminococcus_bromii_YE282
Org1_Gene1608	unique	2547	2595399572_IE32DRAFT_01660_Cupin_domain_[Ruminococcus_bromii_YE282]	Ruminococcus_bromii_YE282
Org1_Gene161	unique	4220	2595398091_IE32DRAFT_00172_hypothetical_protein_[Ruminococcus_bromii_YE282]	Ruminococcus_bromii_YE282
Org1_Gene1610	unique	94	2595399574_IE32DRAFT_01662_Bacterial_membrane_protein_YfhO_[Ruminococcus_bromii_YE282]	Ruminococcus_bromii_YE282
Org1_Gene1612	unique	3958	2595399576_IE32DRAFT_01664_preprotein_translocase,_SecE_subunit,_bacterial_[Ruminococcus_bromii_YE282]	Ruminococcus_bromii_YE282
Org1_Gene1618	unique	3203	2595399582_IE32DRAFT_01670_hypothetical_protein_[Ruminococcus_bromii_YE282]	Ruminococcus_bromii_YE282
Org1_Gene162	unique	3065	2595398092_IE32DRAFT_00173_hypothetical_protein_[Ruminococcus_bromii_YE282]	Ruminococcus_bromii_YE282
Org1_Gene1621	unique	3514	2595399585_IE32DRAFT_01673_hypothetical_protein_[Ruminococcus_bromii_YE282]	Ruminococcus_bromii_YE282
Org1_Gene1623	unique	1876	2595399587_IE32DRAFT_01675_Enterochelin_esterase_and_related_enzymes_[Ruminococcus_bromii_YE282]	Ruminococcus_bromii_YE282
Org1_Gene163	unique	4319	2595398093_IE32DRAFT_00174_hypothetical_protein_[Ruminococcus_bromii_YE282]	Ruminococcus_bromii_YE282
Org1_Gene1632	unique	1380	2595399596_IE32DRAFT_01684_stage_IV_sporulation_protein_B_[Ruminococcus_bromii_YE282]	Ruminococcus_bromii_YE282
Org1_Gene1633	unique	180	2595399597_IE32DRAFT_01685_ATPase,_P-type_(transporting),_HAD_superfamily,_subfamily_IC_[Ruminococcus_bromii_YE282]	Ruminococcus_bromii_YE282
Org1_Gene1634	unique	783	2595399598_IE32DRAFT_01686_tRNA_nucleotidyltransferase/poly(A)_polymerase_[Ruminococcus_bromii_YE282]	Ruminococcus_bromii_YE282
Org1_Gene1635	unique	3374	2595399599_IE32DRAFT_01687_Predicted_acyltransferase_[Ruminococcus_bromii_YE282]	Ruminococcus_bromii_YE282
Org1_Gene1636	unique	3744	2595399600_IE32DRAFT_01688_hypothetical_protein_[Ruminococcus_bromii_YE282]	Ruminococcus_bromii_YE282
Org1_Gene1637	unique	3881	2595399601_IE32DRAFT_01689_hypothetical_protein_[Ruminococcus_bromii_YE282]	Ruminococcus_bromii_YE282
Org1_Gene1638	unique	2653	2595399602_IE32DRAFT_01690_Uncharacterized_conserved_protein_[Ruminococcus_bromii_YE282]	Ruminococcus_bromii_YE282
Org1_Gene164	unique	4310	2595398094_IE32DRAFT_00175_hypothetical_protein_[Ruminococcus_bromii_YE282]	Ruminococcus_bromii_YE282
Org1_Gene1642	unique	2394	2595399606_IE32DRAFT_01694_hypothetical_protein_[Ruminococcus_bromii_YE282]	Ruminococcus_bromii_YE282
Org1_Gene1644	unique	2335	2595399608_IE32DRAFT_01696_N-acetylmuramoyl-L-alanine_amidase_[Ruminococcus_bromii_YE282]	Ruminococcus_bromii_YE282
Org1_Gene1646	unique	4033	2595399610_IE32DRAFT_01698_Fe2+_transport_system_protein_A_[Ruminococcus_bromii_YE282]	Ruminococcus_bromii_YE282
Org1_Gene165	unique	4062	2595398095_IE32DRAFT_00176_hypothetical_protein_[Ruminococcus_bromii_YE282]	Ruminococcus_bromii_YE282
Org1_Gene1650	unique	677	2595399614_IE32DRAFT_01702_Arginine/lysine/ornithine_decarboxylases_[Ruminococcus_bromii_YE282]	Ruminococcus_bromii_YE282
Org1_Gene1653	unique	93	2595399617_IE32DRAFT_01705_Uncharacterized_protein_conserved_in_bacteria_[Ruminococcus_bromii_YE282]	Ruminococcus_bromii_YE282
Org1_Gene1656	unique	637	2595399620_IE32DRAFT_01708_MORN_repeat_[Ruminococcus_bromii_YE282]	Ruminococcus_bromii_YE282
Org1_Gene1657	unique	2022	2595399621_IE32DRAFT_01709_Small-conductance_mechanosensitive_channel_[Ruminococcus_bromii_YE282]	Ruminococcus_bromii_YE282
Org1_Gene1658	unique	1450	2595399622_IE32DRAFT_01710_Enterochelin_esterase_and_related_enzymes_[Ruminococcus_bromii_YE282]	Ruminococcus_bromii_YE282
Org1_Gene1659	unique	1237	2595399623_IE32DRAFT_01711_putative_oxygen-independent_coproporphyrinogen_III_oxidase_[Ruminococcus_bromii_YE282]	Ruminococcus_bromii_YE282
Org1_Gene166	unique	3247	2595398096_IE32DRAFT_00177_Uncharacterized_protein_conserved_in_bacteria_[Ruminococcus_bromii_YE282]	Ruminococcus_bromii_YE282
Org1_Gene1660	unique	730	2595399624_IE32DRAFT_01712_Transcriptional_regulators_containing_a_DNA-binding_HTH_domain_and_an_aminotransferase_domain_(MocR_family)_	Ruminococcus_bromii_YE282
Org1_Gene1662	unique	1160	2595399626_IE32DRAFT_01714_hypothetical_protein_[Ruminococcus_bromii_YE282]	Ruminococcus_bromii_YE282
Org1_Gene1663	unique	495	2595399627_IE32DRAFT_01715_Predicted_membrane_protein_involved_in_D-alanine_export_[Ruminococcus_bromii_YE282]	Ruminococcus_bromii_YE282
Org1_Gene1664	unique	1083	2595399628_IE32DRAFT_01716_Diaminopimelate_decarboxylase_[Ruminococcus_bromii_YE282]	Ruminococcus_bromii_YE282
Org1_Gene1665	unique	4110	2595399629_IE32DRAFT_01717_Phosphopantetheine_attachment_site_[Ruminococcus_bromii_YE282]	Ruminococcus_bromii_YE282
Org1_Gene1666	unique	577	2595399630_IE32DRAFT_01718_AMP-binding_enzyme_C-terminal_domain/AMP-binding_enzyme_[Ruminococcus_bromii_YE282]	Ruminococcus_bromii_YE282
Org1_Gene1667	unique	33	2595399631_IE32DRAFT_01719_Cation/multidrug_efflux_pump_[Ruminococcus_bromii_YE282]	Ruminococcus_bromii_YE282
Org1_Gene1668	unique	2634	2595399632_IE32DRAFT_01720_transcriptional_regulator,_TetR_family_[Ruminococcus_bromii_YE282]	Ruminococcus_bromii_YE282
Org1_Gene167	unique	1544	2595398097_IE32DRAFT_00178_hypothetical_protein_[Ruminococcus_bromii_YE282]	Ruminococcus_bromii_YE282
Org1_Gene1670	unique	3797	2595399634_IE32DRAFT_01722_hypothetical_protein_[Ruminococcus_bromii_YE282]	Ruminococcus_bromii_YE282
Org1_Gene1671	unique	291	2595399635_IE32DRAFT_01723_Archaeal/vacuolar-type_H+-ATPase_subunit_I_[Ruminococcus_bromii_YE282]	Ruminococcus_bromii_YE282
Org1_Gene1673	unique	2768	2595399637_IE32DRAFT_01725_Archaeal/vacuolar-type_H+-ATPase_subunit_E_[Ruminococcus_bromii_YE282]	Ruminococcus_bromii_YE282
Org1_Gene1674	unique	1500	2595399638_IE32DRAFT_01726_Archaeal/vacuolar-type_H+-ATPase_subunit_C_[Ruminococcus_bromii_YE282]	Ruminococcus_bromii_YE282
Org1_Gene1679	unique	2910	2595399643_IE32DRAFT_01731_hypothetical_protein_[Ruminococcus_bromii_YE282]	Ruminococcus_bromii_YE282
Org1_Gene168	unique	3795	2595398098_IE32DRAFT_00179_hypothetical_protein_[Ruminococcus_bromii_YE282]	Ruminococcus_bromii_YE282
Org1_Gene1680	unique	737	2595399644_IE32DRAFT_01732_Arabinogalactan_endo-1,4-beta-galactosidase_[Ruminococcus_bromii_YE282]	Ruminococcus_bromii_YE282
Org1_Gene1681	unique	2237	2595399645_IE32DRAFT_01733_pyruvate_formate-lyase_1-activating_enzyme_[Ruminococcus_bromii_YE282]	Ruminococcus_bromii_YE282
Org1_Gene1683	unique	2406	2595399647_IE32DRAFT_01735_cAMP-binding_proteins_-_catabolite_gene_activator_and_regulatory_subunit_of_cAMP-dependent_protein_kinases_[Ru	Ruminococcus_bromii_YE282
Org1_Gene1685	unique	131	2595399649_IE32DRAFT_01737_ABC-type_transport_system,_involved_in_lipoprotein_release,_permease_component_[Ruminococcus_bromii_YE282]	Ruminococcus_bromii_YE282
Org1_Gene1686	unique	2425	2595399650_IE32DRAFT_01738_ABC-type_antimicrobial_peptide_transport_system,_ATPase_component_[Ruminococcus_bromii_YE282]	Ruminococcus_bromii_YE282
Org1_Gene1687	unique	1445	2595399651_IE32DRAFT_01739_Signal_transduction_histidine_kinase_[Ruminococcus_bromii_YE282]	Ruminococcus_bromii_YE282
Org1_Gene1688	unique	2445	2595399652_IE32DRAFT_01740_Response_regulators_consisting_of_a_CheY-like_receiver_domain_and_a_winged-helix_DNA-binding_domain_[Ruminocc	Ruminococcus_bromii_YE282
Org1_Gene169	unique	3508	2595398099_IE32DRAFT_00180_hypothetical_protein_[Ruminococcus_bromii_YE282]	Ruminococcus_bromii_YE282
Org1_Gene1691	unique	1340	2595399655_IE32DRAFT_01743_Domain_of_unknown_function_(DUF4365)_[Ruminococcus_bromii_YE282]	Ruminococcus_bromii_YE282
Org1_Gene1693	unique	2555	2595399657_IE32DRAFT_01745_hypothetical_protein_[Ruminococcus_bromii_YE282]	Ruminococcus_bromii_YE282
Org1_Gene1694	unique	4296	2595399658_IE32DRAFT_01746_Domain_of_Unknown_Function_(DUF1540)_[Ruminococcus_bromii_YE282]	Ruminococcus_bromii_YE282
Org1_Gene1698	unique	1170	2595399662_IE32DRAFT_01750_Predicted_nucleotidyltransferase_[Ruminococcus_bromii_YE282]	Ruminococcus_bromii_YE282
Org1_Gene17	unique	3234	2595397940_IE32DRAFT_00021_Domain_of_unknown_function_(DUF4358)_[Ruminococcus_bromii_YE282]	Ruminococcus_bromii_YE282
Org1_Gene170	unique	3674	2595398100_IE32DRAFT_00181_hypothetical_protein_[Ruminococcus_bromii_YE282]	Ruminococcus_bromii_YE282
Org1_Gene1703	unique	2518	2595399667_IE32DRAFT_01755_Predicted_phosphatases_[Ruminococcus_bromii_YE282]	Ruminococcus_bromii_YE282
Org1_Gene1704	unique	3111	2595399668_IE32DRAFT_01756_hypothetical_protein_[Ruminococcus_bromii_YE282]	Ruminococcus_bromii_YE282
Org1_Gene1705	unique	2295	2595399669_IE32DRAFT_01757_ribosomal_small_subunit_pseudouridine_synthase_A_(EC_5.4.99.-)_[Ruminococcus_bromii_YE282]	Ruminococcus_bromii_YE282
Org1_Gene1709	unique	1691	2595399673_IE32DRAFT_01761_Cell_division_protein_[Ruminococcus_bromii_YE282]	Ruminococcus_bromii_YE282
Org1_Gene171	unique	3747	2595398101_IE32DRAFT_00182_hypothetical_protein_[Ruminococcus_bromii_YE282]	Ruminococcus_bromii_YE282
Org1_Gene1710	unique	884	2595399674_IE32DRAFT_01762_Membrane-bound_metallopeptidase_[Ruminococcus_bromii_YE282]	Ruminococcus_bromii_YE282
Org1_Gene1711	unique	1926	2595399675_IE32DRAFT_01763_hypothetical_protein_[Ruminococcus_bromii_YE282]	Ruminococcus_bromii_YE282
Org1_Gene1714	unique	3276	2595399678_IE32DRAFT_01766_hypothetical_protein_[Ruminococcus_bromii_YE282]	Ruminococcus_bromii_YE282
Org1_Gene1715	unique	1986	2595399679_IE32DRAFT_01767_hypothetical_protein_[Ruminococcus_bromii_YE282]	Ruminococcus_bromii_YE282
Org1_Gene1717	unique	1757	2595399682_IE32DRAFT_01770_Uncharacterized_protein_conserved_in_bacteria_[Ruminococcus_bromii_YE282]	Ruminococcus_bromii_YE282
Org1_Gene1719	unique	4208	2595399684_IE32DRAFT_01772_hypothetical_protein_[Ruminococcus_bromii_YE282]	Ruminococcus_bromii_YE282
Org1_Gene172	unique	3311	2595398102_IE32DRAFT_00183_hypothetical_protein_[Ruminococcus_bromii_YE282]	Ruminococcus_bromii_YE282
Org1_Gene1720	unique	2687	2595399685_IE32DRAFT_01773_hypothetical_protein_[Ruminococcus_bromii_YE282]	Ruminococcus_bromii_YE282
Org1_Gene1722	unique	767	2595399687_IE32DRAFT_01775_putative_efflux_protein,_MATE_family_[Ruminococcus_bromii_YE282]	Ruminococcus_bromii_YE282
Org1_Gene1724	unique	1705	2595399689_IE32DRAFT_01777_Lysophospholipase_[Ruminococcus_bromii_YE282]	Ruminococcus_bromii_YE282
Org1_Gene1727	unique	1762	2595399692_IE32DRAFT_01780_Zinc_carboxypeptidase_[Ruminococcus_bromii_YE282]	Ruminococcus_bromii_YE282
Org1_Gene1729	unique	1632	2595399694_IE32DRAFT_01782_riboflavin_kinase/FMN_adenylyltransferase_[Ruminococcus_bromii_YE282]	Ruminococcus_bromii_YE282
Org1_Gene173	unique	1110	2595398103_IE32DRAFT_00184_DNA_polymerase_sliding_clamp_subunit_(PCNA_homolog)_[Ruminococcus_bromii_YE282]	Ruminococcus_bromii_YE282
Org1_Gene1732	unique	1005	2595399697_IE32DRAFT_01785_Uncharacterized_conserved_protein_[Ruminococcus_bromii_YE282]	Ruminococcus_bromii_YE282
Org1_Gene1733	unique	3167	2595399699_IE32DRAFT_01787_Predicted_flavin-nucleotide-binding_protein_[Ruminococcus_bromii_YE282]	Ruminococcus_bromii_YE282
Org1_Gene1734	unique	1476	2595399700_IE32DRAFT_01788_L-asparaginase/archaeal_Glu-tRNAGln_amidotransferase_subunit_D_[Ruminococcus_bromii_YE282]	Ruminococcus_bromii_YE282
Org1_Gene1737	unique	757	2595399703_IE32DRAFT_01791_K+_transport_systems,_NAD-binding_component_[Ruminococcus_bromii_YE282]	Ruminococcus_bromii_YE282
Org1_Gene1738	unique	3158	2595399704_IE32DRAFT_01792_Deoxycytidylate_deaminase_[Ruminococcus_bromii_YE282]	Ruminococcus_bromii_YE282
Org1_Gene1739	unique	2714	2595399705_IE32DRAFT_01793_HD_domain_[Ruminococcus_bromii_YE282]	Ruminococcus_bromii_YE282
Org1_Gene174	unique	3197	2595398104_IE32DRAFT_00185_hypothetical_protein_[Ruminococcus_bromii_YE282]	Ruminococcus_bromii_YE282
Org1_Gene1740	unique	569	2595399706_IE32DRAFT_01794_Putative_peptidoglycan-binding_domain-containing_protein_[Ruminococcus_bromii_YE282]	Ruminococcus_bromii_YE282
Org1_Gene1741	unique	2301	2595399707_IE32DRAFT_01795_Leucine_rich_repeats_(6_copies)_[Ruminococcus_bromii_YE282]	Ruminococcus_bromii_YE282
Org1_Gene1744	unique	671	2595399710_IE32DRAFT_01798_hypothetical_protein_[Ruminococcus_bromii_YE282]	Ruminococcus_bromii_YE282
Org1_Gene1746	unique	1780	2595399712_IE32DRAFT_01800_pseudouridine_synthase,_RluA_family_[Ruminococcus_bromii_YE282]	Ruminococcus_bromii_YE282
Org1_Gene1747	unique	3904	2595399713_IE32DRAFT_01801_Predicted_membrane_protein_[Ruminococcus_bromii_YE282]	Ruminococcus_bromii_YE282
Org1_Gene1748	unique	941	2595399714_IE32DRAFT_01802_Uncharacterized_protein_conserved_in_bacteria_[Ruminococcus_bromii_YE282]	Ruminococcus_bromii_YE282
Org1_Gene175	unique	4105	2595398105_IE32DRAFT_00186_hypothetical_protein_[Ruminococcus_bromii_YE282]	Ruminococcus_bromii_YE282
Org1_Gene1750	unique	4100	2595399716_IE32DRAFT_01804_hypothetical_protein_[Ruminococcus_bromii_YE282]	Ruminococcus_bromii_YE282
Org1_Gene1759	unique	707	2595399725_IE32DRAFT_01813_Predicted_permease_[Ruminococcus_bromii_YE282]	Ruminococcus_bromii_YE282
Org1_Gene176	unique	1920	2595398106_IE32DRAFT_00187_hypothetical_protein_[Ruminococcus_bromii_YE282]	Ruminococcus_bromii_YE282
Org1_Gene1762	unique	709	2595399728_IE32DRAFT_01816_RIP_metalloprotease_RseP_[Ruminococcus_bromii_YE282]	Ruminococcus_bromii_YE282
Org1_Gene1764	unique	2106	2595399730_IE32DRAFT_01818_CDP-diglyceride_synthetase_[Ruminococcus_bromii_YE282]	Ruminococcus_bromii_YE282
Org1_Gene1769	unique	2260	2595399735_IE32DRAFT_01823_amino_acid_ABC_transporter_membrane_protein,_PAAT_family_(TC_3.A.1.3.-)_[Ruminococcus_bromii_YE282]	Ruminococcus_bromii_YE282
Org1_Gene177	unique	3764	2595398107_IE32DRAFT_00188_hypothetical_protein_[Ruminococcus_bromii_YE282]	Ruminococcus_bromii_YE282
Org1_Gene1770	unique	2053	2595399736_IE32DRAFT_01824_Bacterial_extracellular_solute-binding_proteins,_family_3_[Ruminococcus_bromii_YE282]	Ruminococcus_bromii_YE282
Org1_Gene1771	unique	1859	2595399737_IE32DRAFT_01825_EDD_domain_protein,_DegV_family_[Ruminococcus_bromii_YE282]	Ruminococcus_bromii_YE282
Org1_Gene1772	unique	1668	2595399738_IE32DRAFT_01826_Transcriptional_regulator_[Ruminococcus_bromii_YE282]	Ruminococcus_bromii_YE282
Org1_Gene1775	unique	3373	2595399741_IE32DRAFT_01829_acyl-CoA_thioester_hydrolase,_YbgC/YbaW_family_[Ruminococcus_bromii_YE282]	Ruminococcus_bromii_YE282
Org1_Gene1776	unique	3559	2595399742_IE32DRAFT_01830_hypothetical_protein_[Ruminococcus_bromii_YE282]	Ruminococcus_bromii_YE282
Org1_Gene178	unique	4353	2595398108_IE32DRAFT_00189_hypothetical_protein_[Ruminococcus_bromii_YE282]	Ruminococcus_bromii_YE282
Org1_Gene1783	unique	1847	2595399749_IE32DRAFT_01837_ABC-type_polysaccharide/polyol_phosphate_export_systems,_permease_component_[Ruminococcus_bromii_YE282]	Ruminococcus_bromii_YE282
Org1_Gene1784	unique	793	2595399750_IE32DRAFT_01838_ABC-type_polysaccharide/polyol_phosphate_transport_system,_ATPase_component_[Ruminococcus_bromii_YE282]	Ruminococcus_bromii_YE282
Org1_Gene1786	unique	2474	2595399752_IE32DRAFT_01840_Predicted_membrane_protein_[Ruminococcus_bromii_YE282]	Ruminococcus_bromii_YE282
Org1_Gene1788	unique	2821	2595399754_IE32DRAFT_01842_hypothetical_protein_[Ruminococcus_bromii_YE282]	Ruminococcus_bromii_YE282
Org1_Gene1789	unique	3567	2595399755_IE32DRAFT_01843_hypothetical_protein_[Ruminococcus_bromii_YE282]	Ruminococcus_bromii_YE282
Org1_Gene179	unique	3719	2595398109_IE32DRAFT_00190_hypothetical_protein_[Ruminococcus_bromii_YE282]	Ruminococcus_bromii_YE282
Org1_Gene1790	unique	1651	2595399756_IE32DRAFT_01844_Predicted_permeases_[Ruminococcus_bromii_YE282]	Ruminococcus_bromii_YE282
Org1_Gene1791	unique	1407	2595399757_IE32DRAFT_01845_Predicted_transcriptional_regulator_[Ruminococcus_bromii_YE282]	Ruminococcus_bromii_YE282
Org1_Gene1793	unique	2606	2595399759_IE32DRAFT_01847_hypothetical_protein_[Ruminococcus_bromii_YE282]	Ruminococcus_bromii_YE282
Org1_Gene1794	unique	1102	2595399760_IE32DRAFT_01848_Uncharacterized_oxidoreductases,_Fe-dependent_alcohol_dehydrogenase_family_[Ruminococcus_bromii_YE282]	Ruminococcus_bromii_YE282
Org1_Gene180	unique	4175	2595398110_IE32DRAFT_00191_hypothetical_protein_[Ruminococcus_bromii_YE282]	Ruminococcus_bromii_YE282
Org1_Gene1807	unique	2273	2595399774_IE32DRAFT_01862_hypothetical_protein_[Ruminococcus_bromii_YE282]	Ruminococcus_bromii_YE282
Org1_Gene181	unique	2856	2595398111_IE32DRAFT_00192_hypothetical_protein_[Ruminococcus_bromii_YE282]	Ruminococcus_bromii_YE282
Org1_Gene1812	unique	1236	2595399779_IE32DRAFT_01867_hypothetical_protein_[Ruminococcus_bromii_YE282]	Ruminococcus_bromii_YE282
Org1_Gene1813	unique	1974	2595399780_IE32DRAFT_01868_Beta-propeller_domains_of_methanol_dehydrogenase_type_[Ruminococcus_bromii_YE282]	Ruminococcus_bromii_YE282
Org1_Gene1814	unique	3880	2595399781_IE32DRAFT_01869_putative_iron-only_hydrogenase_system_regulator_[Ruminococcus_bromii_YE282]	Ruminococcus_bromii_YE282
Org1_Gene1815	unique	1293	2595399782_IE32DRAFT_01870_[FeFe]_hydrogenase_H-cluster_radical_SAM_maturase_HydE_[Ruminococcus_bromii_YE282]	Ruminococcus_bromii_YE282
Org1_Gene1817	unique	1034	2595399784_IE32DRAFT_01872_[FeFe]_hydrogenase_H-cluster_maturation_GTPase_HydF_[Ruminococcus_bromii_YE282]	Ruminococcus_bromii_YE282
Org1_Gene1818	unique	904	2595399786_IE32DRAFT_01874_Chloride_channel_protein_EriC_[Ruminococcus_bromii_YE282]	Ruminococcus_bromii_YE282

Org1_Gene182	unique	1556	2595398112_IE32DRAFT_00193_Phage_integrase_family_[Ruminococcus_bromii_YE282]	Ruminococcus_bromii_YE282
Org1_Gene1821	unique	2070	2595399789_IE32DRAFT_01878_hypothetical_protein_[Ruminococcus_bromii_YE282]	Ruminococcus_bromii_YE282
Org1_Gene1822	unique	173	2595399790_IE32DRAFT_01879_ferrous_iron_transporter_FeoB_[Ruminococcus_bromii_YE282]	Ruminococcus_bromii_YE282
Org1_Gene1825	unique	3789	2595399794_IE32DRAFT_01883_Fe2+/Zn2+_uptake_regulation_proteins_[Ruminococcus_bromii_YE282]	Ruminococcus_bromii_YE282
Org1_Gene1826	unique	3033	2595399795_IE32DRAFT_01884_Ferritin-like_protein_[Ruminococcus_bromii_YE282]	Ruminococcus_bromii_YE282
Org1_Gene1827	unique	2842	2595399796_IE32DRAFT_01885_peroxiredoxin_[Ruminococcus_bromii_YE282]	Ruminococcus_bromii_YE282
Org1_Gene1828	unique	496	2595399797_IE32DRAFT_01886_putative_alkyl_hydroperoxide_reductase_F_subunit_[Ruminococcus_bromii_YE282]	Ruminococcus_bromii_YE282
Org1_Gene183	unique	3259	2595398113_IE32DRAFT_00194_hypothetical_protein_[Ruminococcus_bromii_YE282]	Ruminococcus_bromii_YE282
Org1_Gene1830	unique	3550	2595399799_IE32DRAFT_01888_Desulfoferrodoxin_[Ruminococcus_bromii_YE282]	Ruminococcus_bromii_YE282
Org1_Gene1831	unique	77	2595399800_IE32DRAFT_01889_Leucine_rich_repeats_(6_copies)_[Ruminococcus_bromii_YE282]	Ruminococcus_bromii_YE282
Org1_Gene1832	unique	2122	2595399801_IE32DRAFT_01890_zinc-ribbon_domain_[Ruminococcus_bromii_YE282]	Ruminococcus_bromii_YE282
Org1_Gene1834	unique	2711	2595399803_IE32DRAFT_01892_Acetyltransferase_(isoleucine_patch_superfamily)_[Ruminococcus_bromii_YE282]	Ruminococcus_bromii_YE282
Org1_Gene1838	unique	277	2595399807_IE32DRAFT_01896_Cell_division_protein_FtsI/penicillin-binding_protein_2_[Ruminococcus_bromii_YE282]	Ruminococcus_bromii_YE282
Org1_Gene1839	unique	3003	2595399808_IE32DRAFT_01897_hypothetical_protein_[Ruminococcus_bromii_YE282]	Ruminococcus_bromii_YE282
Org1_Gene184	unique	4305	2595398114_IE32DRAFT_00195_hypothetical_protein_[Ruminococcus_bromii_YE282]	Ruminococcus_bromii_YE282
Org1_Gene1840	unique	1963	2595399809_IE32DRAFT_01898_rod_shape-determining_protein_MreC_[Ruminococcus_bromii_YE282]	Ruminococcus_bromii_YE282
Org1_Gene1843	unique	253	2595399812_IE32DRAFT_01901_Collagenase_and_related_proteases_[Ruminococcus_bromii_YE282]	Ruminococcus_bromii_YE282
Org1_Gene1844	unique	2920	2595399813_IE32DRAFT_01902_Uncharacterized_protein_conserved_in_bacteria_[Ruminococcus_bromii_YE282]	Ruminococcus_bromii_YE282
Org1_Gene1845	unique	1922	2595399814_IE32DRAFT_01903_Protein_of_unknown_function,_DUF624_[Ruminococcus_bromii_YE282]	Ruminococcus_bromii_YE282
Org1_Gene185	unique	4245	2595398115_IE32DRAFT_00196_hypothetical_protein_[Ruminococcus_bromii_YE282]	Ruminococcus_bromii_YE282
Org1_Gene1851	unique	1167	2595399820_IE32DRAFT_01909_Glycosyl_hydrolases_family_25_[Ruminococcus_bromii_YE282]	Ruminococcus_bromii_YE282
Org1_Gene1855	unique	2270	2595399829_IE32DRAFT_01918_Fic/DOC_family_[Ruminococcus_bromii_YE282]	Ruminococcus_bromii_YE282
Org1_Gene1856	unique	908	2595399830_IE32DRAFT_01919_DNA-methyltransferase_(dcm)_[Ruminococcus_bromii_YE282]	Ruminococcus_bromii_YE282
Org1_Gene1857	unique	4420	2595399831_IE32DRAFT_01920_Protein_of_unknown_function_(DUF3789)_[Ruminococcus_bromii_YE282]	Ruminococcus_bromii_YE282
Org1_Gene1859	unique	4221	2595399833_IE32DRAFT_01922_hypothetical_protein_[Ruminococcus_bromii_YE282]	Ruminococcus_bromii_YE282
Org1_Gene186	unique	3527	2595398116_IE32DRAFT_00197_Growth_inhibitor_[Ruminococcus_bromii_YE282]	Ruminococcus_bromii_YE282
Org1_Gene1861	unique	2419	2595399835_IE32DRAFT_01924_Metallopeptidase_family_M24_[Ruminococcus_bromii_YE282]	Ruminococcus_bromii_YE282
Org1_Gene1862	unique	2191	2595399836_IE32DRAFT_01925_hypothetical_protein_[Ruminococcus_bromii_YE282]	Ruminococcus_bromii_YE282
Org1_Gene1864	unique	115	2595399838_IE32DRAFT_01927_Cna_protein_B-type_domain_[Ruminococcus_bromii_YE282]	Ruminococcus_bromii_YE282
Org1_Gene1865	unique	3704	2595399839_IE32DRAFT_01928_hypothetical_protein_[Ruminococcus_bromii_YE282]	Ruminococcus_bromii_YE282
Org1_Gene1867	unique	3698	2595399841_IE32DRAFT_01930_Helix-turn-helix_domain_[Ruminococcus_bromii_YE282]	Ruminococcus_bromii_YE282
Org1_Gene1868	unique	3062	2595399842_IE32DRAFT_01931_Uncharacterized_protein_conserved_in_bacteria_[Ruminococcus_bromii_YE282]	Ruminococcus_bromii_YE282
Org1_Gene187	unique	1434	2595398117_IE32DRAFT_00198_hypothetical_protein_[Ruminococcus_bromii_YE282]	Ruminococcus_bromii_YE282
Org1_Gene1870	unique	3655	2595399844_IE32DRAFT_01933_hypothetical_protein_[Ruminococcus_bromii_YE282]	Ruminococcus_bromii_YE282
Org1_Gene1872	unique	2055	2595399846_IE32DRAFT_01935_Uncharacterized_protein_conserved_in_bacteria_[Ruminococcus_bromii_YE282]	Ruminococcus_bromii_YE282
Org1_Gene1874	unique	248	2595399848_IE32DRAFT_01937_AAA_ATPase_domain_[Ruminococcus_bromii_YE282]	Ruminococcus_bromii_YE282
Org1_Gene1875	unique	2889	2595399849_IE32DRAFT_01938_hypothetical_protein_[Ruminococcus_bromii_YE282]	Ruminococcus_bromii_YE282
Org1_Gene1877	unique	4036	2595399851_IE32DRAFT_01940_hypothetical_protein_[Ruminococcus_bromii_YE282]	Ruminococcus_bromii_YE282
Org1_Gene1878	unique	551	2595399852_IE32DRAFT_01941_hypothetical_protein_[Ruminococcus_bromii_YE282]	Ruminococcus_bromii_YE282
Org1_Gene188	unique	2598	2595398118_IE32DRAFT_00199_hypothetical_protein_[Ruminococcus_bromii_YE282]	Ruminococcus_bromii_YE282
Org1_Gene1881	unique	3488	2595399855_IE32DRAFT_01944_hypothetical_protein_[Ruminococcus_bromii_YE282]	Ruminococcus_bromii_YE282
Org1_Gene1882	unique	3452	2595399856_IE32DRAFT_01945_hypothetical_protein_[Ruminococcus_bromii_YE282]	Ruminococcus_bromii_YE282
Org1_Gene1887	unique	4247	2595399866_IE32DRAFT_01955_hypothetical_protein_[Ruminococcus_bromii_YE282]	Ruminococcus_bromii_YE282
Org1_Gene1888	unique	2737	2595399867_IE32DRAFT_01956_hypothetical_protein_[Ruminococcus_bromii_YE282]	Ruminococcus_bromii_YE282
Org1_Gene189	unique	3902	2595398119_IE32DRAFT_00200_RyR_domain_[Ruminococcus_bromii_YE282]	Ruminococcus_bromii_YE282
Org1_Gene1897	unique	1478	2595399876_IE32DRAFT_01965_Restriction_endonuclease_[Ruminococcus_bromii_YE282]	Ruminococcus_bromii_YE282
Org1_Gene1898	unique	2097	2595399877_IE32DRAFT_01966_hypothetical_protein_[Ruminococcus_bromii_YE282]	Ruminococcus_bromii_YE282
Org1_Gene19	unique	1857	2595397942_IE32DRAFT_00023_hypothetical_protein_[Ruminococcus_bromii_YE282]	Ruminococcus_bromii_YE282
Org1_Gene190	unique	3174	2595398120_IE32DRAFT_00201_Amidases_related_to_nicotinamidase_[Ruminococcus_bromii_YE282]	Ruminococcus_bromii_YE282
Org1_Gene1908	unique	2277	2595399887_IE32DRAFT_01976_hypothetical_protein_[Ruminococcus_bromii_YE282]	Ruminococcus_bromii_YE282
Org1_Gene191	unique	3509	2595398121_IE32DRAFT_00202_hypothetical_protein_[Ruminococcus_bromii_YE282]	Ruminococcus_bromii_YE282
Org1_Gene1915	unique	667	2595399894_IE32DRAFT_01983_hypothetical_protein_[Ruminococcus_bromii_YE282]	Ruminococcus_bromii_YE282
Org1_Gene1916	unique	3586	2595399895_IE32DRAFT_01984_hypothetical_protein_[Ruminococcus_bromii_YE282]	Ruminococcus_bromii_YE282
Org1_Gene1919	unique	1503	2595399898_IE32DRAFT_01987_hypothetical_protein_[Ruminococcus_bromii_YE282]	Ruminococcus_bromii_YE282
Org1_Gene192	unique	2539	2595398122_IE32DRAFT_00203_hypothetical_protein_[Ruminococcus_bromii_YE282]	Ruminococcus_bromii_YE282
Org1_Gene1920	unique	1744	2595399899_IE32DRAFT_01988_AraC-type_DNA-binding_domain-containing_proteins_[Ruminococcus_bromii_YE282]	Ruminococcus_bromii_YE282
Org1_Gene1921	unique	691	2595399900_IE32DRAFT_01989_Domain_of_unknown_function_(DUF4367)_[Ruminococcus_bromii_YE282]	Ruminococcus_bromii_YE282
Org1_Gene1922	unique	3491	2595399901_IE32DRAFT_01990_hypothetical_protein_[Ruminococcus_bromii_YE282]	Ruminococcus_bromii_YE282
Org1_Gene1923	unique	3112	2595399902_IE32DRAFT_01991_RNA_polymerase_sigma_factor,_sigma-70_family_[Ruminococcus_bromii_YE282]	Ruminococcus_bromii_YE282
Org1_Gene1924	unique	3617	2595399903_IE32DRAFT_01992_hypothetical_protein_[Ruminococcus_bromii_YE282]	Ruminococcus_bromii_YE282
Org1_Gene1925	unique	2934	2595399904_IE32DRAFT_01993_hypothetical_protein_[Ruminococcus_bromii_YE282]	Ruminococcus_bromii_YE282
Org1_Gene1926	unique	1320	2595399905_IE32DRAFT_01994_hypothetical_protein_[Ruminococcus_bromii_YE282]	Ruminococcus_bromii_YE282
Org1_Gene1927	unique	3878	2595399906_IE32DRAFT_01995_hypothetical_protein_[Ruminococcus_bromii_YE282]	Ruminococcus_bromii_YE282
Org1_Gene1928	unique	1693	2595399907_IE32DRAFT_01996_Uncharacterized_conserved_protein_[Ruminococcus_bromii_YE282]	Ruminococcus_bromii_YE282
Org1_Gene193	unique	3589	2595398123_IE32DRAFT_00204_hypothetical_protein_[Ruminococcus_bromii_YE282]	Ruminococcus_bromii_YE282
Org1_Gene1931	unique	3864	2595399910_IE32DRAFT_01999_hypothetical_protein_[Ruminococcus_bromii_YE282]	Ruminococcus_bromii_YE282
Org1_Gene1932	unique	1373	2595399911_IE32DRAFT_02000_heat-inducible_transcription_repressor_HrcA_[Ruminococcus_bromii_YE282]	Ruminococcus_bromii_YE282
Org1_Gene1933	unique	2843	2595399912_IE32DRAFT_02001_Molecular_chaperone_GrpE_(heat_shock_protein)_[Ruminococcus_bromii_YE282]	Ruminococcus_bromii_YE282
Org1_Gene1937	unique	2695	2595399916_IE32DRAFT_02005_hypothetical_protein_[Ruminococcus_bromii_YE282]	Ruminococcus_bromii_YE282
Org1_Gene1938	unique	1995	2595399917_IE32DRAFT_02006_hypothetical_protein_[Ruminococcus_bromii_YE282]	Ruminococcus_bromii_YE282
Org1_Gene194	unique	666	2595398124_IE32DRAFT_00205_Protein_of_unknown_function_(DUF2493)/Reverse_transcriptase_(RNA-dependent_DNA_polymerase)_[Ruminococcus_b	Ruminococcus_bromii_YE282
Org1_Gene1940	unique	3742	2595399919_IE32DRAFT_02008_hypothetical_protein_[Ruminococcus_bromii_YE282]	Ruminococcus_bromii_YE282
Org1_Gene1942	unique	3708	2595399921_IE32DRAFT_02010_Predicted_branched-chain_amino_acid_permeases_(azaleucine_resistance)_[Ruminococcus_bromii_YE282]	Ruminococcus_bromii_YE282
Org1_Gene1943	unique	2262	2595399922_IE32DRAFT_02011_Predicted_branched-chain_amino_acid_permease_(azaleucine_resistance)_[Ruminococcus_bromii_YE282]	Ruminococcus_bromii_YE282
Org1_Gene1944	unique	2351	2595399923_IE32DRAFT_02012_cAMP-binding_proteins_-_catabolite_gene_activator_and_regulatory_subunit_of_cAMP-dependent_protein_kinases_[Ru	Ruminococcus_bromii_YE282
Org1_Gene1945	unique	823	2595399924_IE32DRAFT_02013_electron_transport_complex,_RnfABCDGE_type,_C_subunit_[Ruminococcus_bromii_YE282]	Ruminococcus_bromii_YE282
Org1_Gene1946	unique	1636	2595399925_IE32DRAFT_02014_electron_transport_complex,_RnfABCDGE_type,_D_subunit_[Ruminococcus_bromii_YE282]	Ruminococcus_bromii_YE282
Org1_Gene1947	unique	2940	2595399926_IE32DRAFT_02015_Predicted_NADH:ubiquinone_oxidoreductase,_subunit_RnfG_[Ruminococcus_bromii_YE282]	Ruminococcus_bromii_YE282
Org1_Gene1948	unique	2457	2595399927_IE32DRAFT_02016_electron_transport_complex,_RnfABCDGE_type,_E_subunit_[Ruminococcus_bromii_YE282]	Ruminococcus_bromii_YE282
Org1_Gene1949	unique	2735	2595399928_IE32DRAFT_02017_electron_transport_complex,_RnfABCDGE_type,_A_subunit_[Ruminococcus_bromii_YE282]	Ruminococcus_bromii_YE282
Org1_Gene195	unique	205	2595398134_IE32DRAFT_00215_TerD_domain_[Ruminococcus_bromii_YE282]	Ruminococcus_bromii_YE282
Org1_Gene1950	unique	1935	2595399929_IE32DRAFT_02018_Predicted_NADH:ubiquinone_oxidoreductase,_subunit_RnfB_[Ruminococcus_bromii_YE282]	Ruminococcus_bromii_YE282
Org1_Gene1951	unique	3754	2595399930_IE32DRAFT_02019_O-6-methylguanine_DNA_methyltransferase_[Ruminococcus_bromii_YE282]	Ruminococcus_bromii_YE282
Org1_Gene1952	unique	2638	2595399931_IE32DRAFT_02020_Putative_phosphatase_regulatory_subunit_[Ruminococcus_bromii_YE282]	Ruminococcus_bromii_YE282
Org1_Gene1953	unique	4152	2595399932_IE32DRAFT_02021_hypothetical_protein_[Ruminococcus_bromii_YE282]	Ruminococcus_bromii_YE282
Org1_Gene1954	unique	1406	2595399933_IE32DRAFT_02022_Actin-like_ATPase_involved_in_cell_morphogenesis_[Ruminococcus_bromii_YE282]	Ruminococcus_bromii_YE282
Org1_Gene1955	unique	2476	2595399934_IE32DRAFT_02023_Predicted_amidophosphoribosyltransferases_[Ruminococcus_bromii_YE282]	Ruminococcus_bromii_YE282
Org1_Gene1957	unique	2617	2595399936_IE32DRAFT_02025_cAMP-binding_proteins_-_catabolite_gene_activator_and_regulatory_subunit_of_cAMP-dependent_protein_kinases_[Ru	Ruminococcus_bromii_YE282
Org1_Gene196	unique	3867	2595398136_IE32DRAFT_00217_hypothetical_protein_[Ruminococcus_bromii_YE282]	Ruminococcus_bromii_YE282
Org1_Gene1960	unique	166	2595399939_IE32DRAFT_02028_X-X-X-Leu-X-X-Gly_heptad_repeats_[Ruminococcus_bromii_YE282]	Ruminococcus_bromii_YE282
Org1_Gene1962	unique	2817	2595399941_IE32DRAFT_02030_transcriptional_regulator,_TetR_family_[Ruminococcus_bromii_YE282]	Ruminococcus_bromii_YE282
Org1_Gene1963	unique	2116	2595399942_IE32DRAFT_02031_Uncharacterized_protein_conserved_in_bacteria_[Ruminococcus_bromii_YE282]	Ruminococcus_bromii_YE282
Org1_Gene1966	unique	3038	2595399945_IE32DRAFT_02034_shikimate_kinase_(EC_2.7.1.71)_[Ruminococcus_bromii_YE282]	Ruminococcus_bromii_YE282
Org1_Gene197	unique	614	2595398139_IE32DRAFT_00220_transposase,_IS605_OrfB_family,_central_region_[Ruminococcus_bromii_YE282]	Ruminococcus_bromii_YE282
Org1_Gene1973	unique	529	2595399952_IE32DRAFT_02041_Bacterial_SH3_domain_[Ruminococcus_bromii_YE282]	Ruminococcus_bromii_YE282
Org1_Gene1974	unique	3333	2595399953_IE32DRAFT_02042_Mannose-6-phosphate_isomerase_[Ruminococcus_bromii_YE282]	Ruminococcus_bromii_YE282
Org1_Gene1978	unique	875	2595399957_IE32DRAFT_02046_GHKL_domain_[Ruminococcus_bromii_YE282]	Ruminococcus_bromii_YE282
Org1_Gene1979	unique	2354	2595399958_IE32DRAFT_02047_two_component_transcriptional_regulator,_LytTR_family_[Ruminococcus_bromii_YE282]	Ruminococcus_bromii_YE282
Org1_Gene198	unique	3949	2595398141_IE32DRAFT_00222_hypothetical_protein_[Ruminococcus_bromii_YE282]	Ruminococcus_bromii_YE282
Org1_Gene1980	unique	3657	2595399959_IE32DRAFT_02048_Helix-turn-helix_domain_[Ruminococcus_bromii_YE282]	Ruminococcus_bromii_YE282
Org1_Gene1981	unique	1100	2595399960_IE32DRAFT_02049_Cysteinyl-tRNA_synthetase_[Ruminococcus_bromii_YE282]	Ruminococcus_bromii_YE282
Org1_Gene1982	unique	1707	2595399961_IE32DRAFT_02050_Nucleoside_diphosphate_kinase_[Ruminococcus_bromii_YE282]	Ruminococcus_bromii_YE282
Org1_Gene1983	unique	4049	2595399962_IE32DRAFT_02051_MoaD_family_protein,_archaeal_[Ruminococcus_bromii_YE282]	Ruminococcus_bromii_YE282
Org1_Gene1984	unique	2071	2595399963_IE32DRAFT_02052_Dinucleotide-utilizing_enzymes_involved_in_molybdopterin_and_thiamine_biosynthesis_family_2_[Ruminococcus_bromi	Ruminococcus_bromii_YE282
Org1_Gene1985	unique	2432	2595399964_IE32DRAFT_02053_Predicted_phosphoribosyltransferases_[Ruminococcus_bromii_YE282]	Ruminococcus_bromii_YE282
Org1_Gene1986	unique	1583	2595399965_IE32DRAFT_02054_Branched-chain_amino_acid_aminotransferase/4-amino-4-deoxychorismate_lyase_[Ruminococcus_bromii_YE282]	Ruminococcus_bromii_YE282
Org1_Gene1987	unique	2066	2595399966_IE32DRAFT_02055_Predicted_metal-dependent_hydrolase_of_the_TIM-barrel_fold_[Ruminococcus_bromii_YE282]	Ruminococcus_bromii_YE282
Org1_Gene1988	unique	1330	2595399967_IE32DRAFT_02056_Uncharacterised_nucleotidyltransferase_[Ruminococcus_bromii_YE282]	Ruminococcus_bromii_YE282
Org1_Gene1989	unique	2799	2595399968_IE32DRAFT_02057_Uncharacterized_protein_conserved_in_bacteria_[Ruminococcus_bromii_YE282]	Ruminococcus_bromii_YE282
Org1_Gene199	unique	2010	2595398143_IE32DRAFT_00224_Uncharacterized_conserved_protein_[Ruminococcus_bromii_YE282]	Ruminococcus_bromii_YE282
Org1_Gene1990	unique	1183	2595399969_IE32DRAFT_02058_hypothetical_protein_[Ruminococcus_bromii_YE282]	Ruminococcus_bromii_YE282
Org1_Gene1991	unique	4021	2595399970_IE32DRAFT_02059_Phosphopantetheine_attachment_site_[Ruminococcus_bromii_YE282]	Ruminococcus_bromii_YE282
Org1_Gene1992	unique	1367	2595399971_IE32DRAFT_02060_NlpC/p60-like_transpeptidase_[Ruminococcus_bromii_YE282]	Ruminococcus_bromii_YE282
Org1_Gene1993	unique	1446	2595399972_IE32DRAFT_02061_ABC-type_uncharacterized_transport_system,_ATPase_component_[Ruminococcus_bromii_YE282]	Ruminococcus_bromii_YE282
Org1_Gene1994	unique	2090	2595399973_IE32DRAFT_02062_ABC-type_uncharacterized_transport_system,_permease_component_[Ruminococcus_bromii_YE282]	Ruminococcus_bromii_YE282
Org1_Gene1995	unique	1983	2595399974_IE32DRAFT_02063_ABC-type_uncharacterized_transport_system,_permease_component_[Ruminococcus_bromii_YE282]	Ruminococcus_bromii_YE282
Org1_Gene1996	unique	441	2595399975_IE32DRAFT_02064_AMP-binding_enzyme_C-terminal_domain/AMP-binding_enzyme/Phosphopantetheine_attachment_site_[Ruminococcus_	Ruminococcus_bromii_YE282
Org1_Gene1997	unique	1273	2595399976_IE32DRAFT_02065_NlpC/p60-like_transpeptidase_[Ruminococcus_bromii_YE282]	Ruminococcus_bromii_YE282
Org1_Gene1998	unique	3562	2595399977_IE32DRAFT_02066_Helix-turn-helix_domain_[Ruminococcus_bromii_YE282]	Ruminococcus_bromii_YE282
Org1_Gene1999	unique	4196	2595399978_IE32DRAFT_02067_hypothetical_protein_[Ruminococcus_bromii_YE282]	Ruminococcus_bromii_YE282
Org1_Gene20	unique	2827	2595397943_IE32DRAFT_00024_Haloacid_Dehalogenase_superfamily,_subfamily_IB,_phosphoserine_phosphatase-like_[Ruminococcus_bromii_YE282]	Ruminococcus_bromii_YE282
Org1_Gene200	unique	3842	2595398144_IE32DRAFT_00225_hypothetical_protein_[Ruminococcus_bromii_YE282]	Ruminococcus_bromii_YE282

Org1_Gene2004	unique	2893	2595399983_IE32DRAFT_02072_hypothetical_protein_[Ruminococcus_bromii_YE282]	Ruminococcus_bromii_YE282
Org1_Gene2005	unique	4169	2595399984_IE32DRAFT_02073_AAA_domain_[Ruminococcus_bromii_YE282]	Ruminococcus_bromii_YE282
Org1_Gene2006	unique	573	2595399985_IE32DRAFT_02074_AAA_domain_[Ruminococcus_bromii_YE282]	Ruminococcus_bromii_YE282
Org1_Gene2009	unique	3650	2595399988_IE32DRAFT_02077_hypothetical_protein_[Ruminococcus_bromii_YE282]	Ruminococcus_bromii_YE282
Org1_Gene201	unique	2019	2595398145_IE32DRAFT_00226_hypothetical_protein_[Ruminococcus_bromii_YE282]	Ruminococcus_bromii_YE282
Org1_Gene2019	unique	880	2595399998_IE32DRAFT_02087_hypothetical_protein_[Ruminococcus_bromii_YE282]	Ruminococcus_bromii_YE282
Org1_Gene202	unique	2218	2595398146_IE32DRAFT_00227_hypothetical_protein_[Ruminococcus_bromii_YE282]	Ruminococcus_bromii_YE282
Org1_Gene2020	unique	2468	2595399999_IE32DRAFT_02088_Colicin_V_production_protein_[Ruminococcus_bromii_YE282]	Ruminococcus_bromii_YE282
Org1_Gene2021	unique	2722	2595400000_IE32DRAFT_02089_Phosphatidylglycerophosphate_synthase_[Ruminococcus_bromii_YE282]	Ruminococcus_bromii_YE282
Org1_Gene2024	unique	4056	2595400004_IE32DRAFT_02093_Predicted_transcriptional_regulator_[Ruminococcus_bromii_YE282]	Ruminococcus_bromii_YE282
Org1_Gene2025	unique	3973	2595400005_IE32DRAFT_02094_hypothetical_protein_[Ruminococcus_bromii_YE282]	Ruminococcus_bromii_YE282
Org1_Gene2026	unique	1463	2595400006_IE32DRAFT_02095_Zeta_toxin_[Ruminococcus_bromii_YE282]	Ruminococcus_bromii_YE282
Org1_Gene2027	unique	1592	2595400007_IE32DRAFT_02096_Fic/DOC_family_[Ruminococcus_bromii_YE282]	Ruminococcus_bromii_YE282
Org1_Gene2028	unique	3030	2595400008_IE32DRAFT_02097_Predicted_phosphatase_homologous_to_the_C-terminal_domain_of_histone_macroH2A1_[Ruminococcus_bromii_YE282	Ruminococcus_bromii_YE282
Org1_Gene2029	unique	1901	2595400009_IE32DRAFT_02098_hypothetical_protein_[Ruminococcus_bromii_YE282]	Ruminococcus_bromii_YE282
Org1_Gene203	unique	2408	2595398147_IE32DRAFT_00228_hypothetical_protein_[Ruminococcus_bromii_YE282]	Ruminococcus_bromii_YE282
Org1_Gene2030	unique	1517	2595400010_IE32DRAFT_02099_Protein_of_unknown_function_(DUF3991)/Toprim-like_[Ruminococcus_bromii_YE282]	Ruminococcus_bromii_YE282
Org1_Gene2031	unique	3824	2595400011_IE32DRAFT_02100_hypothetical_protein_[Ruminococcus_bromii_YE282]	Ruminococcus_bromii_YE282
Org1_Gene2032	unique	665	2595400012_IE32DRAFT_02101_Bacterial_Ig-like_domain_(group_2)_[Ruminococcus_bromii_YE282]	Ruminococcus_bromii_YE282
Org1_Gene2033	unique	148	2595400013_IE32DRAFT_02102_AAA-like_domain_[Ruminococcus_bromii_YE282]	Ruminococcus_bromii_YE282
Org1_Gene2034	unique	3743	2595400014_IE32DRAFT_02103_PrgI_family_protein_[Ruminococcus_bromii_YE282]	Ruminococcus_bromii_YE282
Org1_Gene2035	unique	1193	2595400015_IE32DRAFT_02104_TrbL/VirB6_plasmid_conjugal_transfer_protein_[Ruminococcus_bromii_YE282]	Ruminococcus_bromii_YE282
Org1_Gene2038	unique	712	2595400018_IE32DRAFT_02107_Predicted_transcriptional_regulators_[Ruminococcus_bromii_YE282]	Ruminococcus_bromii_YE282
Org1_Gene2039	unique	1696	2595400019_IE32DRAFT_02108_hypothetical_protein_[Ruminococcus_bromii_YE282]	Ruminococcus_bromii_YE282
Org1_Gene204	unique	2965	2595398148_IE32DRAFT_00229_hypothetical_protein_[Ruminococcus_bromii_YE282]	Ruminococcus_bromii_YE282
Org1_Gene2040	unique	3495	2595400020_IE32DRAFT_02109_hypothetical_protein_[Ruminococcus_bromii_YE282]	Ruminococcus_bromii_YE282
Org1_Gene2041	unique	76	2595400021_IE32DRAFT_02110_DNA_or_RNA_helicases_of_superfamily_II_[Ruminococcus_bromii_YE282]	Ruminococcus_bromii_YE282
Org1_Gene2043	unique	3791	2595400023_IE32DRAFT_02112_hypothetical_protein_[Ruminococcus_bromii_YE282]	Ruminococcus_bromii_YE282
Org1_Gene2044	unique	3479	2595400024_IE32DRAFT_02113_Ribbon-helix-helix_protein,_copG_family_[Ruminococcus_bromii_YE282]	Ruminococcus_bromii_YE282
Org1_Gene2045	unique	732	2595400025_IE32DRAFT_02114_Relaxase/Mobilisation_nuclease_domain_[Ruminococcus_bromii_YE282]	Ruminococcus_bromii_YE282
Org1_Gene2046	unique	1879	2595400026_IE32DRAFT_02115_ParB/RepB/Spo0J_family_partition_protein_[Ruminococcus_bromii_YE282]	Ruminococcus_bromii_YE282
Org1_Gene2047	unique	4269	2595400027_IE32DRAFT_02116_DNA_binding_domain,_excisionase_family_[Ruminococcus_bromii_YE282]	Ruminococcus_bromii_YE282
Org1_Gene2048	unique	4270	2595400028_IE32DRAFT_02117_DNA_binding_domain,_excisionase_family_[Ruminococcus_bromii_YE282]	Ruminococcus_bromii_YE282
Org1_Gene2049	unique	4253	2595400029_IE32DRAFT_02118_DNA_binding_domain,_excisionase_family_[Ruminococcus_bromii_YE282]	Ruminococcus_bromii_YE282
Org1_Gene205	unique	3888	2595398149_IE32DRAFT_00230_hypothetical_protein_[Ruminococcus_bromii_YE282]	Ruminococcus_bromii_YE282
Org1_Gene2050	unique	1094	2595400030_IE32DRAFT_02119_Site-specific_recombinase_XerD_[Ruminococcus_bromii_YE282]	Ruminococcus_bromii_YE282
Org1_Gene2055	unique	3231	2595400035_IE32DRAFT_02124_hypothetical_protein_[Ruminococcus_bromii_YE282]	Ruminococcus_bromii_YE282
Org1_Gene2056	unique	2050	2595400036_IE32DRAFT_02125_hypothetical_protein_[Ruminococcus_bromii_YE282]	Ruminococcus_bromii_YE282
Org1_Gene2057	unique	2161	2595400037_IE32DRAFT_02126_hypothetical_protein_[Ruminococcus_bromii_YE282]	Ruminococcus_bromii_YE282
Org1_Gene2058	unique	1959	2595400038_IE32DRAFT_02127_ABC-2_family_transporter_protein_[Ruminococcus_bromii_YE282]	Ruminococcus_bromii_YE282
Org1_Gene2059	unique	1889	2595400039_IE32DRAFT_02128_ABC-2_family_transporter_protein_[Ruminococcus_bromii_YE282]	Ruminococcus_bromii_YE282
Org1_Gene206	unique	3836	2595398150_IE32DRAFT_00231_hypothetical_protein_[Ruminococcus_bromii_YE282]	Ruminococcus_bromii_YE282
Org1_Gene2060	unique	2497	2595400040_IE32DRAFT_02129_ABC-type_multidrug_transport_system,_ATPase_component_[Ruminococcus_bromii_YE282]	Ruminococcus_bromii_YE282
Org1_Gene2061	unique	2807	2595400041_IE32DRAFT_02130_hypothetical_protein_[Ruminococcus_bromii_YE282]	Ruminococcus_bromii_YE282
Org1_Gene2062	unique	375	2595400042_IE32DRAFT_02131_deoxyguanosinetriphosphate_triphosphohydrolase,_putative_[Ruminococcus_bromii_YE282]	Ruminococcus_bromii_YE282
Org1_Gene2063	unique	4078	2595400043_IE32DRAFT_02132_Helix-turn-helix_domain_[Ruminococcus_bromii_YE282]	Ruminococcus_bromii_YE282
Org1_Gene2064	unique	465	2595400044_IE32DRAFT_02133_Signal_transduction_histidine_kinase_[Ruminococcus_bromii_YE282]	Ruminococcus_bromii_YE282
Org1_Gene2065	unique	2459	2595400045_IE32DRAFT_02134_Response_regulators_consisting_of_a_CheY-like_receiver_domain_and_a_winged-helix_DNA-binding_domain_[Ruminoco	Ruminococcus_bromii_YE282
Org1_Gene2066	unique	2566	2595400046_IE32DRAFT_02135_phosphate_transport_system_regulatory_protein_PhoU_[Ruminococcus_bromii_YE282]	Ruminococcus_bromii_YE282
Org1_Gene2068	unique	1833	2595400048_IE32DRAFT_02137_phosphate_ABC_transporter,_permease_protein_PstA_[Ruminococcus_bromii_YE282]	Ruminococcus_bromii_YE282
Org1_Gene2069	unique	1763	2595400049_IE32DRAFT_02138_phosphate_ABC_transporter,_permease_protein_PstC_[Ruminococcus_bromii_YE282]	Ruminococcus_bromii_YE282
Org1_Gene207	unique	3950	2595398151_IE32DRAFT_00232_hypothetical_protein_[Ruminococcus_bromii_YE282]	Ruminococcus_bromii_YE282
Org1_Gene2070	unique	1798	2595400050_IE32DRAFT_02139_phosphate_binding_protein_[Ruminococcus_bromii_YE282]	Ruminococcus_bromii_YE282
Org1_Gene2071	unique	919	2595400051_IE32DRAFT_02140_Predicted_Zn-dependent_peptidases_[Ruminococcus_bromii_YE282]	Ruminococcus_bromii_YE282
Org1_Gene2075	unique	3772	2595400055_IE32DRAFT_02144_Predicted_ribosomal_protein_[Ruminococcus_bromii_YE282]	Ruminococcus_bromii_YE282
Org1_Gene2079	unique	1791	2595400059_IE32DRAFT_02148_Uncharacterized_conserved_protein_[Ruminococcus_bromii_YE282]	Ruminococcus_bromii_YE282
Org1_Gene208	unique	4219	2595398152_IE32DRAFT_00233_hypothetical_protein_[Ruminococcus_bromii_YE282]	Ruminococcus_bromii_YE282
Org1_Gene2080	unique	822	2595400060_IE32DRAFT_02149_putative_efflux_protein,_MATE_family_[Ruminococcus_bromii_YE282]	Ruminococcus_bromii_YE282
Org1_Gene2081	unique	1949	2595400061_IE32DRAFT_02150_hypothetical_protein_[Ruminococcus_bromii_YE282]	Ruminococcus_bromii_YE282
Org1_Gene2082	unique	1358	2595400062_IE32DRAFT_02151_Predicted_membrane_protein_[Ruminococcus_bromii_YE282]	Ruminococcus_bromii_YE282
Org1_Gene2083	unique	280	2595400063_IE32DRAFT_02152_Alpha_amylase,_catalytic_domain_[Ruminococcus_bromii_YE282]	Ruminococcus_bromii_YE282
Org1_Gene2085	unique	1992	2595400065_IE32DRAFT_02154_Predicted_metal-dependent_hydrolase_of_the_TIM-barrel_fold_[Ruminococcus_bromii_YE282]	Ruminococcus_bromii_YE282
Org1_Gene2086	unique	2627	2595400066_IE32DRAFT_02155_transcriptional_regulator,_TetR_family_[Ruminococcus_bromii_YE282]	Ruminococcus_bromii_YE282
Org1_Gene209	unique	1634	2595398153_IE32DRAFT_00234_Predicted_phosphohydrolase_(DHH_superfamily)_[Ruminococcus_bromii_YE282]	Ruminococcus_bromii_YE282
Org1_Gene2090	unique	2599	2595400071_IE32DRAFT_02160_thiamine_pyrophosphokinase_[Ruminococcus_bromii_YE282]	Ruminococcus_bromii_YE282
Org1_Gene2095	unique	791	2595400076_IE32DRAFT_02165_16S_rRNA_(cytosine(967)-C(5))-methyltransferase_[Ruminococcus_bromii_YE282]	Ruminococcus_bromii_YE282
Org1_Gene21	unique	1813	2595397944_IE32DRAFT_00025_hypothetical_protein_[Ruminococcus_bromii_YE282]	Ruminococcus_bromii_YE282
Org1_Gene210	unique	2213	2595398154_IE32DRAFT_00235_hypothetical_protein_[Ruminococcus_bromii_YE282]	Ruminococcus_bromii_YE282
Org1_Gene2101	unique	2782	2595400082_IE32DRAFT_02171_guanylate_kinase_(EC_2.7.4.8)_[Ruminococcus_bromii_YE282]	Ruminococcus_bromii_YE282
Org1_Gene2104	unique	1498	2595400085_IE32DRAFT_02174_hypothetical_protein_[Ruminococcus_bromii_YE282]	Ruminococcus_bromii_YE282
Org1_Gene2105	unique	2405	2595400086_IE32DRAFT_02175_Sporulation_factor_SpoIIGA_[Ruminococcus_bromii_YE282]	Ruminococcus_bromii_YE282
Org1_Gene2107	unique	3834	2595400088_IE32DRAFT_02177_hypothetical_protein_[Ruminococcus_bromii_YE282]	Ruminococcus_bromii_YE282
Org1_Gene2108	unique	1433	2595400093_IE32DRAFT_02182_Uncharacterized_protein_conserved_in_bacteria_[Ruminococcus_bromii_YE282]	Ruminococcus_bromii_YE282
Org1_Gene211	unique	3707	2595398155_IE32DRAFT_00236_hypothetical_protein_[Ruminococcus_bromii_YE282]	Ruminococcus_bromii_YE282
Org1_Gene2110	unique	668	2595400095_IE32DRAFT_02184_Transposase_and_inactivated_derivatives,_IS30_family_[Ruminococcus_bromii_YE282]	Ruminococcus_bromii_YE282
Org1_Gene2113	unique	1528	2595400098_IE32DRAFT_02187_hypothetical_protein_[Ruminococcus_bromii_YE282]	Ruminococcus_bromii_YE282
Org1_Gene2114	unique	609	2595400099_IE32DRAFT_02188_hypothetical_protein_[Ruminococcus_bromii_YE282]	Ruminococcus_bromii_YE282
Org1_Gene2116	unique	2423	2595400101_IE32DRAFT_02190_Zn-dependent_proteases_[Ruminococcus_bromii_YE282]	Ruminococcus_bromii_YE282
Org1_Gene2119	unique	2633	2595400104_IE32DRAFT_02193_Protein_of_unknown_function_(DUF2953)_[Ruminococcus_bromii_YE282]	Ruminococcus_bromii_YE282
Org1_Gene212	unique	3389	2595398156_IE32DRAFT_00237_hypothetical_protein_[Ruminococcus_bromii_YE282]	Ruminococcus_bromii_YE282
Org1_Gene2121	unique	1172	2595400106_IE32DRAFT_02195_D-alanyl-D-alanine_carboxypeptidase_[Ruminococcus_bromii_YE282]	Ruminococcus_bromii_YE282
Org1_Gene2128	unique	1845	2595400113_IE32DRAFT_02202_ABC-type_amino_acid_transport/signal_transduction_systems,_periplasmic_component/domain_[Ruminococcus_bromii_	Ruminococcus_bromii_YE282
Org1_Gene2129	unique	1793	2595400114_IE32DRAFT_02203_Predicted_membrane_protein_[Ruminococcus_bromii_YE282]	Ruminococcus_bromii_YE282
Org1_Gene213	unique	2991	2595398157_IE32DRAFT_00238_Calcineurin-like_phosphoesterase_[Ruminococcus_bromii_YE282]	Ruminococcus_bromii_YE282
Org1_Gene2130	unique	2899	2595400115_IE32DRAFT_02204_RNA_polymerase_sigma_factor,_sigma-70_family_[Ruminococcus_bromii_YE282]	Ruminococcus_bromii_YE282
Org1_Gene2131	unique	3897	2595400116_IE32DRAFT_02205_hypothetical_protein_[Ruminococcus_bromii_YE282]	Ruminococcus_bromii_YE282
Org1_Gene2132	unique	1647	2595400117_IE32DRAFT_02206_hypothetical_protein_[Ruminococcus_bromii_YE282]	Ruminococcus_bromii_YE282
Org1_Gene2133	unique	2508	2595400118_IE32DRAFT_02207_E1-E2_ATPase_[Ruminococcus_bromii_YE282]	Ruminococcus_bromii_YE282
Org1_Gene2134	unique	528	2595400119_IE32DRAFT_02208_haloacid_dehalogenase-like_hydrolase_[Ruminococcus_bromii_YE282]	Ruminococcus_bromii_YE282
Org1_Gene2135	unique	2830	2595400120_IE32DRAFT_02209_Membrane-associated_phospholipid_phosphatase_[Ruminococcus_bromii_YE282]	Ruminococcus_bromii_YE282
Org1_Gene2136	unique	1820	2595400121_IE32DRAFT_02210_hypothetical_protein_[Ruminococcus_bromii_YE282]	Ruminococcus_bromii_YE282
Org1_Gene2137	unique	3883	2595400122_IE32DRAFT_02211_hypothetical_protein_[Ruminococcus_bromii_YE282]	Ruminococcus_bromii_YE282
Org1_Gene2138	unique	4150	2595400123_IE32DRAFT_02212_hypothetical_protein_[Ruminococcus_bromii_YE282]	Ruminococcus_bromii_YE282
Org1_Gene2139	unique	3838	2595400124_IE32DRAFT_02213_hypothetical_protein_[Ruminococcus_bromii_YE282]	Ruminococcus_bromii_YE282
Org1_Gene214	unique	3474	2595398158_IE32DRAFT_00239_hypothetical_protein_[Ruminococcus_bromii_YE282]	Ruminococcus_bromii_YE282
Org1_Gene2140	unique	4236	2595400125_IE32DRAFT_02214_hypothetical_protein_[Ruminococcus_bromii_YE282]	Ruminococcus_bromii_YE282
Org1_Gene2141	unique	4237	2595400126_IE32DRAFT_02215_hypothetical_protein_[Ruminococcus_bromii_YE282]	Ruminococcus_bromii_YE282
Org1_Gene2143	unique	3166	2595400128_IE32DRAFT_02217_hypothetical_protein_[Ruminococcus_bromii_YE282]	Ruminococcus_bromii_YE282
Org1_Gene215	unique	2341	2595398159_IE32DRAFT_00240_hypothetical_protein_[Ruminococcus_bromii_YE282]	Ruminococcus_bromii_YE282
Org1_Gene216	unique	3099	2595398160_IE32DRAFT_00241_Dual_specificity_phosphatase,_catalytic_domain_[Ruminococcus_bromii_YE282]	Ruminococcus_bromii_YE282
Org1_Gene217	unique	2831	2595398161_IE32DRAFT_00242_hypothetical_protein_[Ruminococcus_bromii_YE282]	Ruminococcus_bromii_YE282
Org1_Gene2175	unique	3016	2595400160_IE32DRAFT_02249_ATP:corrinoid_adenosyltransferase_[Ruminococcus_bromii_YE282]	Ruminococcus_bromii_YE282
Org1_Gene2177	unique	2777	2595400162_IE32DRAFT_02251_hypothetical_protein_[Ruminococcus_bromii_YE282]	Ruminococcus_bromii_YE282
Org1_Gene2178	unique	2098	2595400163_IE32DRAFT_02252_Nucleotidyltransferase_domain/Domain_of_unknown_function_(DUF4037)_[Ruminococcus_bromii_YE282]	Ruminococcus_bromii_YE282
Org1_Gene218	unique	967	2595398162_IE32DRAFT_00243_Arabinose_efflux_permease_[Ruminococcus_bromii_YE282]	Ruminococcus_bromii_YE282
Org1_Gene2183	unique	566	2595400168_IE32DRAFT_02257_Domain_of_unknown_function_(DUF4340)_[Ruminococcus_bromii_YE282]	Ruminococcus_bromii_YE282
Org1_Gene2184	unique	488	2595400169_IE32DRAFT_02258_ABC-type_uncharacterized_transport_system_[Ruminococcus_bromii_YE282]	Ruminococcus_bromii_YE282
Org1_Gene2189	unique	361	2595400174_IE32DRAFT_02263_Trypsin-like_serine_proteases,_typically_periplasmic,_contain_C-terminal_PDZ_domain_[Ruminococcus_bromii_YE282]	Ruminococcus_bromii_YE282
Org1_Gene219	unique	259	2595398163_IE32DRAFT_00244_DNA_ligase,_NAD-dependent_[Ruminococcus_bromii_YE282]	Ruminococcus_bromii_YE282
Org1_Gene2191	unique	3169	2595400176_IE32DRAFT_02265_Uncharacterized_conserved_protein_[Ruminococcus_bromii_YE282]	Ruminococcus_bromii_YE282
Org1_Gene2194	unique	200	2595400180_IE32DRAFT_02269_Glycosidases_[Ruminococcus_bromii_YE282]	Ruminococcus_bromii_YE282
Org1_Gene2197	unique	1694	2595400183_IE32DRAFT_02272_NLPA_lipoprotein_[Ruminococcus_bromii_YE282]	Ruminococcus_bromii_YE282
Org1_Gene2198	unique	1427	2595400184_IE32DRAFT_02273_ABC-type_metal_ion_transport_system,_ATPase_component_[Ruminococcus_bromii_YE282]	Ruminococcus_bromii_YE282
Org1_Gene2199	unique	2368	2595400185_IE32DRAFT_02274_ABC-type_metal_ion_transport_system,_permease_component_[Ruminococcus_bromii_YE282]	Ruminococcus_bromii_YE282
Org1_Gene220	unique	3945	2595398164_IE32DRAFT_00245_hypothetical_protein_[Ruminococcus_bromii_YE282]	Ruminococcus_bromii_YE282
Org1_Gene2201	unique	2917	2595400187_IE32DRAFT_02276_Tic20-like_protein/zinc-ribbon_domain_[Ruminococcus_bromii_YE282]	Ruminococcus_bromii_YE282
Org1_Gene2202	unique	642	2595400188_IE32DRAFT_02277_hypothetical_protein_[Ruminococcus_bromii_YE282]	Ruminococcus_bromii_YE282
Org1_Gene2203	unique	1940	2595400189_IE32DRAFT_02278_Predicted_membrane_protein_[Ruminococcus_bromii_YE282]	Ruminococcus_bromii_YE282
Org1_Gene2204	unique	3793	2595400190_IE32DRAFT_02280_hypothetical_protein_[Ruminococcus_bromii_YE282]	Ruminococcus_bromii_YE282
Org1_Gene2205	unique	2736	2595400191_IE32DRAFT_02281_YcaO-type_kinase_domain_[Ruminococcus_bromii_YE282]	Ruminococcus_bromii_YE282
Org1_Gene2206	unique	4184	2595400192_IE32DRAFT_02282_hypothetical_protein_[Ruminococcus_bromii_YE282]	Ruminococcus_bromii_YE282
Org1_Gene2208	unique	1099	2595400194_IE32DRAFT_02284_Site-specific_recombinase_XerD_[Ruminococcus_bromii_YE282]	Ruminococcus_bromii_YE282

Org1_Gene221	unique	2082	2595398165_IE32DRAFT_00246_DNA-methyltransferase_(dcm)_[Ruminococcus_bromii_YE282]	Ruminococcus_bromii_YE282
Org1_Gene2210	unique	2101	2595400196_IE32DRAFT_02286_Replication_initiator_protein_A_(RepA)_N-terminus_[Ruminococcus_bromii_YE282]	Ruminococcus_bromii_YE282
Org1_Gene2211	unique	835	2595400197_IE32DRAFT_02287_Predicted_ATPase_(AAA+_superfamily)_[Ruminococcus_bromii_YE282]	Ruminococcus_bromii_YE282
Org1_Gene2212	unique	63	2595400198_IE32DRAFT_02288_Helicase_conserved_C-terminal_domain/PLD-like_domain/SNF2_family_N-terminal_domain_[Ruminococcus_bromii_YE28	Ruminococcus_bromii_YE282
Org1_Gene2213	unique	149	2595400199_IE32DRAFT_02289_Eco57I_restriction-modification_methylase_[Ruminococcus_bromii_YE282]	Ruminococcus_bromii_YE282
Org1_Gene2214	unique	4081	2595400200_IE32DRAFT_02290_TaqI-like_C-terminal_specificity_domain_[Ruminococcus_bromii_YE282]	Ruminococcus_bromii_YE282
Org1_Gene2215	unique	2079	2595400201_IE32DRAFT_02291_TaqI-like_C-terminal_specificity_domain_[Ruminococcus_bromii_YE282]	Ruminococcus_bromii_YE282
Org1_Gene2216	unique	2685	2595400202_IE32DRAFT_02292_Site-specific_recombinases,_DNA_invertase_Pin_homologs_[Ruminococcus_bromii_YE282]	Ruminococcus_bromii_YE282
Org1_Gene2217	unique	1623	2595400203_IE32DRAFT_02293_hypothetical_protein_[Ruminococcus_bromii_YE282]	Ruminococcus_bromii_YE282
Org1_Gene2218	unique	3618	2595400204_IE32DRAFT_02294_Transposon-encoded_protein_TnpV_[Ruminococcus_bromii_YE282]	Ruminococcus_bromii_YE282
Org1_Gene222	unique	4116	2595398166_IE32DRAFT_00247_hypothetical_protein_[Ruminococcus_bromii_YE282]	Ruminococcus_bromii_YE282
Org1_Gene2220	unique	3812	2595400206_IE32DRAFT_02296_hypothetical_protein_[Ruminococcus_bromii_YE282]	Ruminococcus_bromii_YE282
Org1_Gene2221	unique	168	2595400208_IE32DRAFT_02298_D-alanyl-D-alanine_carboxypeptidase_[Ruminococcus_bromii_YE282]	Ruminococcus_bromii_YE282
Org1_Gene2224	unique	2572	2595400211_IE32DRAFT_02301_hypothetical_protein_[Ruminococcus_bromii_YE282]	Ruminococcus_bromii_YE282
Org1_Gene2225	unique	1270	2595400212_IE32DRAFT_02302_Transposase_and_inactivated_derivatives_[Ruminococcus_bromii_YE282]	Ruminococcus_bromii_YE282
Org1_Gene2228	unique	997	2595400216_IE32DRAFT_02306_flavoprotein,_HI0933_family_[Ruminococcus_bromii_YE282]	Ruminococcus_bromii_YE282
Org1_Gene2229	unique	3064	2595400217_IE32DRAFT_02307_Predicted_membrane_protein_[Ruminococcus_bromii_YE282]	Ruminococcus_bromii_YE282
Org1_Gene223	unique	4331	2595398167_IE32DRAFT_00248_hypothetical_protein_[Ruminococcus_bromii_YE282]	Ruminococcus_bromii_YE282
Org1_Gene2230	unique	3205	2595400218_IE32DRAFT_02308_Predicted_integral_membrane_protein_[Ruminococcus_bromii_YE282]	Ruminococcus_bromii_YE282
Org1_Gene2232	unique	3629	2595400220_IE32DRAFT_02310_hypothetical_protein_[Ruminococcus_bromii_YE282]	Ruminococcus_bromii_YE282
Org1_Gene2233	unique	2660	2595400221_IE32DRAFT_02311_Peptidase_family_M50_[Ruminococcus_bromii_YE282]	Ruminococcus_bromii_YE282
Org1_Gene2236	unique	1692	2595400224_IE32DRAFT_02314_Predicted_Fe-S_oxidoreductase_[Ruminococcus_bromii_YE282]	Ruminococcus_bromii_YE282
Org1_Gene2237	unique	2162	2595400225_IE32DRAFT_02315_hypothetical_protein_[Ruminococcus_bromii_YE282]	Ruminococcus_bromii_YE282
Org1_Gene2238	unique	3190	2595400226_IE32DRAFT_02316_Protein_of_unknown_function_(DUF3021)_[Ruminococcus_bromii_YE282]	Ruminococcus_bromii_YE282
Org1_Gene2239	unique	3268	2595400227_IE32DRAFT_02317_LytTr_DNA-binding_domain_[Ruminococcus_bromii_YE282]	Ruminococcus_bromii_YE282
Org1_Gene224	unique	4235	2595398168_IE32DRAFT_00249_hypothetical_protein_[Ruminococcus_bromii_YE282]	Ruminococcus_bromii_YE282
Org1_Gene2240	unique	3829	2595400228_IE32DRAFT_02318_hypothetical_protein_[Ruminococcus_bromii_YE282]	Ruminococcus_bromii_YE282
Org1_Gene2241	unique	1053	2595400229_IE32DRAFT_02319_hypothetical_protein_[Ruminococcus_bromii_YE282]	Ruminococcus_bromii_YE282
Org1_Gene2242	unique	916	2595400230_IE32DRAFT_02320_Aminopeptidase_C_[Ruminococcus_bromii_YE282]	Ruminococcus_bromii_YE282
Org1_Gene2243	unique	1719	2595400231_IE32DRAFT_02321_4Fe-4S_single_cluster_domain_[Ruminococcus_bromii_YE282]	Ruminococcus_bromii_YE282
Org1_Gene2244	unique	1523	2595400232_IE32DRAFT_02322_hypothetical_protein_[Ruminococcus_bromii_YE282]	Ruminococcus_bromii_YE282
Org1_Gene2245	unique	1572	2595400233_IE32DRAFT_02323_hypothetical_protein_[Ruminococcus_bromii_YE282]	Ruminococcus_bromii_YE282
Org1_Gene2246	unique	4358	2595400234_IE32DRAFT_02324_hypothetical_protein_[Ruminococcus_bromii_YE282]	Ruminococcus_bromii_YE282
Org1_Gene2247	unique	1635	2595400235_IE32DRAFT_02325_Radical_SAM_superfamily_[Ruminococcus_bromii_YE282]	Ruminococcus_bromii_YE282
Org1_Gene2248	unique	3896	2595400236_IE32DRAFT_02326_hypothetical_protein_[Ruminococcus_bromii_YE282]	Ruminococcus_bromii_YE282
Org1_Gene2249	unique	720	2595400237_IE32DRAFT_02327_radical_SAM_additional_4Fe4S-binding_SPASM_domain_[Ruminococcus_bromii_YE282]	Ruminococcus_bromii_YE282
Org1_Gene225	unique	3560	2595398169_IE32DRAFT_00250_C-5_cytosine-specific_DNA_methylase_[Ruminococcus_bromii_YE282]	Ruminococcus_bromii_YE282
Org1_Gene2250	unique	2578	2595400238_IE32DRAFT_02328_hypothetical_protein_[Ruminococcus_bromii_YE282]	Ruminococcus_bromii_YE282
Org1_Gene2251	unique	2676	2595400239_IE32DRAFT_02329_isopentenyl-diphosphate_delta-isomerase_(EC_5.3.3.2)_[Ruminococcus_bromii_YE282]	Ruminococcus_bromii_YE282
Org1_Gene2252	unique	2414	2595400240_IE32DRAFT_02330_Transposase_DDE_domain_[Ruminococcus_bromii_YE282]	Ruminococcus_bromii_YE282
Org1_Gene2254	unique	3299	2595400242_IE32DRAFT_02332_Sigma-70,_region_4_[Ruminococcus_bromii_YE282]	Ruminococcus_bromii_YE282
Org1_Gene2255	unique	4309	2595400243_IE32DRAFT_02333_hypothetical_protein_[Ruminococcus_bromii_YE282]	Ruminococcus_bromii_YE282
Org1_Gene2256	unique	4364	2595400244_IE32DRAFT_02334_hypothetical_protein_[Ruminococcus_bromii_YE282]	Ruminococcus_bromii_YE282
Org1_Gene2257	unique	516	2595400245_IE32DRAFT_02335_Site-specific_recombinases,_DNA_invertase_Pin_homologs_[Ruminococcus_bromii_YE282]	Ruminococcus_bromii_YE282
Org1_Gene2259	unique	2152	2595400248_IE32DRAFT_02338_LPXTG-site_transpeptidase_(sortase)_family_protein_[Ruminococcus_bromii_YE282]	Ruminococcus_bromii_YE282
Org1_Gene226	unique	3175	2595398170_IE32DRAFT_00251_hypothetical_protein_[Ruminococcus_bromii_YE282]	Ruminococcus_bromii_YE282
Org1_Gene2260	unique	3630	2595400249_IE32DRAFT_02339_hypothetical_protein_[Ruminococcus_bromii_YE282]	Ruminococcus_bromii_YE282
Org1_Gene2261	unique	4051	2595400250_IE32DRAFT_02340_SpoVG_[Ruminococcus_bromii_YE282]	Ruminococcus_bromii_YE282
Org1_Gene2262	unique	1123	2595400251_IE32DRAFT_02341_Predicted_transcriptional_regulators_[Ruminococcus_bromii_YE282]	Ruminococcus_bromii_YE282
Org1_Gene2263	unique	2083	2595400252_IE32DRAFT_02342_ATPases_involved_in_chromosome_partitioning_[Ruminococcus_bromii_YE282]	Ruminococcus_bromii_YE282
Org1_Gene2264	unique	3787	2595400253_IE32DRAFT_02343_hypothetical_protein_[Ruminococcus_bromii_YE282]	Ruminococcus_bromii_YE282
Org1_Gene2265	unique	3159	2595400254_IE32DRAFT_02344_Phage_integrase_family_[Ruminococcus_bromii_YE282]	Ruminococcus_bromii_YE282
Org1_Gene2266	unique	3021	2595400255_IE32DRAFT_02345_hypothetical_protein_[Ruminococcus_bromii_YE282]	Ruminococcus_bromii_YE282
Org1_Gene2267	unique	2198	2595400256_IE32DRAFT_02346_CAAX_protease_self-immunity_[Ruminococcus_bromii_YE282]	Ruminococcus_bromii_YE282
Org1_Gene2268	unique	2982	2595400257_IE32DRAFT_02347_Amidases_related_to_nicotinamidase_[Ruminococcus_bromii_YE282]	Ruminococcus_bromii_YE282
Org1_Gene2269	unique	3122	2595400258_IE32DRAFT_02348_transcriptional_regulator,_CarD_family_[Ruminococcus_bromii_YE282]	Ruminococcus_bromii_YE282
Org1_Gene2270	unique	1070	2595400259_IE32DRAFT_02349_Beta-glucosidase-related_glycosidases_[Ruminococcus_bromii_YE282]	Ruminococcus_bromii_YE282
Org1_Gene2272	unique	2824	2595400261_IE32DRAFT_02351_Spore_cortex_protein_YabQ_(Spore_YabQ)_[Ruminococcus_bromii_YE282]	Ruminococcus_bromii_YE282
Org1_Gene2273	unique	3753	2595400262_IE32DRAFT_02352_Septum_formation_initiator_[Ruminococcus_bromii_YE282]	Ruminococcus_bromii_YE282
Org1_Gene2278	unique	2524	2595400267_IE32DRAFT_02357_ABC-type_multidrug_transport_system,_ATPase_component_[Ruminococcus_bromii_YE282]	Ruminococcus_bromii_YE282
Org1_Gene2279	unique	2187	2595400268_IE32DRAFT_02358_ABC-2_family_transporter_protein_[Ruminococcus_bromii_YE282]	Ruminococcus_bromii_YE282
Org1_Gene228	unique	2859	2595398172_IE32DRAFT_00253_Protein_of_unknown_function_(DUF3793)_[Ruminococcus_bromii_YE282]	Ruminococcus_bromii_YE282
Org1_Gene2280	unique	3894	2595400269_IE32DRAFT_02359_Radical_SAM_superfamily_[Ruminococcus_bromii_YE282]	Ruminococcus_bromii_YE282
Org1_Gene2281	unique	3194	2595400270_IE32DRAFT_02360_hypothetical_protein_[Ruminococcus_bromii_YE282]	Ruminococcus_bromii_YE282
Org1_Gene2282	unique	3226	2595400271_IE32DRAFT_02361_hypothetical_protein_[Ruminococcus_bromii_YE282]	Ruminococcus_bromii_YE282
Org1_Gene2286	unique	2875	2595400275_IE32DRAFT_02365_Acetyltransferases,_including_N-acetylases_of_ribosomal_proteins_[Ruminococcus_bromii_YE282]	Ruminococcus_bromii_YE282
Org1_Gene2287	unique	3008	2595400276_IE32DRAFT_02366_hypothetical_protein_[Ruminococcus_bromii_YE282]	Ruminococcus_bromii_YE282
Org1_Gene2289	unique	4013	2595400278_IE32DRAFT_02368_hypothetical_protein_[Ruminococcus_bromii_YE282]	Ruminococcus_bromii_YE282
Org1_Gene229	unique	3859	2595398173_IE32DRAFT_00254_Uncharacterized_protein_conserved_in_bacteria_(DUF2325)_[Ruminococcus_bromii_YE282]	Ruminococcus_bromii_YE282
Org1_Gene2290	unique	3898	2595400279_IE32DRAFT_02369_Mor_transcription_activator_family_[Ruminococcus_bromii_YE282]	Ruminococcus_bromii_YE282
Org1_Gene2291	unique	3265	2595400280_IE32DRAFT_02370_Acetyltransferase_(GNAT)_domain_[Ruminococcus_bromii_YE282]	Ruminococcus_bromii_YE282
Org1_Gene2292	unique	3687	2595400281_IE32DRAFT_02371_Domain_of_unknown_function_(DUF3784)_[Ruminococcus_bromii_YE282]	Ruminococcus_bromii_YE282
Org1_Gene2293	unique	3100	2595400282_IE32DRAFT_02372_NUDIX_domain_[Ruminococcus_bromii_YE282]	Ruminococcus_bromii_YE282
Org1_Gene2294	unique	2968	2595400283_IE32DRAFT_02373_Predicted_transcriptional_regulators_[Ruminococcus_bromii_YE282]	Ruminococcus_bromii_YE282
Org1_Gene2298	unique	4388	2595400287_IE32DRAFT_02377_hypothetical_protein_[Ruminococcus_bromii_YE282]	Ruminococcus_bromii_YE282
Org1_Gene2299	unique	1548	2595400288_IE32DRAFT_02378_AAA_domain_[Ruminococcus_bromii_YE282]	Ruminococcus_bromii_YE282
Org1_Gene23	unique	2871	2595397946_IE32DRAFT_00027_hypothetical_protein_[Ruminococcus_bromii_YE282]	Ruminococcus_bromii_YE282
Org1_Gene2300	unique	598	2595400289_IE32DRAFT_02379_NUDIX_domain/Plasmid_recombination_enzyme_[Ruminococcus_bromii_YE282]	Ruminococcus_bromii_YE282
Org1_Gene2301	unique	1518	2595400290_IE32DRAFT_02380_ADP-ribose_pyrophosphatase_[Ruminococcus_bromii_YE282]	Ruminococcus_bromii_YE282
Org1_Gene2302	unique	3191	2595400291_IE32DRAFT_02381_Acetyltransferases_[Ruminococcus_bromii_YE282]	Ruminococcus_bromii_YE282
Org1_Gene2303	unique	2137	2595400292_IE32DRAFT_02382_Protein_kinase_domain_[Ruminococcus_bromii_YE282]	Ruminococcus_bromii_YE282
Org1_Gene2304	unique	3893	2595400293_IE32DRAFT_02383_hypothetical_protein_[Ruminococcus_bromii_YE282]	Ruminococcus_bromii_YE282
Org1_Gene2305	unique	2232	2595400294_IE32DRAFT_02384_Phosphorylase_superfamily_[Ruminococcus_bromii_YE282]	Ruminococcus_bromii_YE282
Org1_Gene2306	unique	3220	2595400295_IE32DRAFT_02385_Acetyltransferase_(GNAT)_domain_[Ruminococcus_bromii_YE282]	Ruminococcus_bromii_YE282
Org1_Gene2308	unique	1096	2595400297_IE32DRAFT_02387_Transposase_and_inactivated_derivatives_[Ruminococcus_bromii_YE282]	Ruminococcus_bromii_YE282
Org1_Gene231	unique	4001	2595398175_IE32DRAFT_00256_hypothetical_protein_[Ruminococcus_bromii_YE282]	Ruminococcus_bromii_YE282
Org1_Gene2310	unique	72	2595400299_IE32DRAFT_02389_pullulanase,_type_I_[Ruminococcus_bromii_YE282]	Ruminococcus_bromii_YE282
Org1_Gene2312	unique	3826	2595400301_IE32DRAFT_02391_LysM_domain_[Ruminococcus_bromii_YE282]	Ruminococcus_bromii_YE282
Org1_Gene2313	unique	1868	2595400302_IE32DRAFT_02392_ABC-type_Mn2+/Zn2+_transport_systems,_permease_components_[Ruminococcus_bromii_YE282]	Ruminococcus_bromii_YE282
Org1_Gene2314	unique	2318	2595400303_IE32DRAFT_02393_ABC-type_Mn/Zn_transport_systems,_ATPase_component_[Ruminococcus_bromii_YE282]	Ruminococcus_bromii_YE282
Org1_Gene2315	unique	1494	2595400304_IE32DRAFT_02394_ABC-type_metal_ion_transport_system,_periplasmic_component/surface_adhesin_[Ruminococcus_bromii_YE282]	Ruminococcus_bromii_YE282
Org1_Gene2316	unique	3412	2595400305_IE32DRAFT_02395_Fe2+/Zn2+_uptake_regulation_proteins_[Ruminococcus_bromii_YE282]	Ruminococcus_bromii_YE282
Org1_Gene2318	unique	2972	2595400308_IE32DRAFT_02398_Predicted_transcriptional_regulators_[Ruminococcus_bromii_YE282]	Ruminococcus_bromii_YE282
Org1_Gene2319	unique	2525	2595400309_IE32DRAFT_02399_Protein_of_unknown_function_(DUF998)_[Ruminococcus_bromii_YE282]	Ruminococcus_bromii_YE282
Org1_Gene232	unique	109	2595398176_IE32DRAFT_00257_MacB-like_periplasmic_core_domain/FtsX-like_permease_family_[Ruminococcus_bromii_YE282]	Ruminococcus_bromii_YE282
Org1_Gene2320	unique	4227	2595400310_IE32DRAFT_02400_Cysteine-rich_KTR_[Ruminococcus_bromii_YE282]	Ruminococcus_bromii_YE282
Org1_Gene2321	unique	2682	2595400311_IE32DRAFT_02401_CAAX_protease_self-immunity_[Ruminococcus_bromii_YE282]	Ruminococcus_bromii_YE282
Org1_Gene2322	unique	2327	2595400312_IE32DRAFT_02402_HEAT_repeats_[Ruminococcus_bromii_YE282]	Ruminococcus_bromii_YE282
Org1_Gene2323	unique	2427	2595400313_IE32DRAFT_02403_hypothetical_protein_[Ruminococcus_bromii_YE282]	Ruminococcus_bromii_YE282
Org1_Gene2329	unique	1300	2595400319_IE32DRAFT_02409_Plasmid_recombination_enzyme_[Ruminococcus_bromii_YE282]	Ruminococcus_bromii_YE282
Org1_Gene233	unique	2380	2595398177_IE32DRAFT_00258_ABC-type_antimicrobial_peptide_transport_system,_ATPase_component_[Ruminococcus_bromii_YE282]	Ruminococcus_bromii_YE282
Org1_Gene2330	unique	3519	2595400320_IE32DRAFT_02410_Transposon-encoded_protein_TnpV_[Ruminococcus_bromii_YE282]	Ruminococcus_bromii_YE282
Org1_Gene2332	unique	4204	2595400322_IE32DRAFT_02412_hypothetical_protein_[Ruminococcus_bromii_YE282]	Ruminococcus_bromii_YE282
Org1_Gene2333	unique	3531	2595400323_IE32DRAFT_02413_transcriptional_regulator,_HxlR_family_[Ruminococcus_bromii_YE282]	Ruminococcus_bromii_YE282
Org1_Gene2334	unique	3870	2595400324_IE32DRAFT_02414_hypothetical_protein_[Ruminococcus_bromii_YE282]	Ruminococcus_bromii_YE282
Org1_Gene2335	unique	4359	2595400325_IE32DRAFT_02415_hypothetical_protein_[Ruminococcus_bromii_YE282]	Ruminococcus_bromii_YE282
Org1_Gene2338	unique	3089	2595400328_IE32DRAFT_02418_hypothetical_protein_[Ruminococcus_bromii_YE282]	Ruminococcus_bromii_YE282
Org1_Gene2339	unique	4022	2595400329_IE32DRAFT_02419_hypothetical_protein_[Ruminococcus_bromii_YE282]	Ruminococcus_bromii_YE282
Org1_Gene2340	unique	988	2595400330_IE32DRAFT_02420_ABC-type_branched-chain_amino_acid_transport_systems,_periplasmic_component_[Ruminococcus_bromii_YE282]	Ruminococcus_bromii_YE282
Org1_Gene2341	unique	1746	2595400331_IE32DRAFT_02421_Branched-chain_amino_acid_ABC-type_transport_system,_permease_components_[Ruminococcus_bromii_YE282]	Ruminococcus_bromii_YE282
Org1_Gene2342	unique	1226	2595400332_IE32DRAFT_02422_amino_acid/amide_ABC_transporter_membrane_protein_2,_HAAT_family_(TC_3.A.1.4.-)_[Ruminococcus_bromii_YE282]	Ruminococcus_bromii_YE282
Org1_Gene2343	unique	1808	2595400333_IE32DRAFT_02423_ABC-type_branched-chain_amino_acid_transport_systems,_ATPase_component_[Ruminococcus_bromii_YE282]	Ruminococcus_bromii_YE282
Org1_Gene2344	unique	2388	2595400334_IE32DRAFT_02424_amino_acid/amide_ABC_transporter_ATP-binding_protein_2,_HAAT_family_(TC_3.A.1.4.-)_[Ruminococcus_bromii_YE282	Ruminococcus_bromii_YE282
Org1_Gene2346	unique	3	2595400336_IE32DRAFT_02426_PKD_domain/Fibronectin_type_III_domain/Leucine_rich_repeats_(6_copies)_[Ruminococcus_bromii_YE282]	Ruminococcus_bromii_YE282
Org1_Gene2347	unique	2643	2595400337_IE32DRAFT_02427_hypothetical_protein_[Ruminococcus_bromii_YE282]	Ruminococcus_bromii_YE282
Org1_Gene2348	unique	3969	2595400339_IE32DRAFT_02429_Bacterial_regulatory_protein,_arsR_family_[Ruminococcus_bromii_YE282]	Ruminococcus_bromii_YE282
Org1_Gene2349	unique	2558	2595400340_IE32DRAFT_02430_SdpI/YhfL_protein_family/Protein_of_unknown_function_(DUF1648)_[Ruminococcus_bromii_YE282]	Ruminococcus_bromii_YE282
Org1_Gene2352	unique	2403	2595400343_IE32DRAFT_02433_nicotinamide_mononucleotide_transporter_PnuC_[Ruminococcus_bromii_YE282]	Ruminococcus_bromii_YE282
Org1_Gene2353	unique	2704	2595400344_IE32DRAFT_02434_Sortase_and_related_acyltransferases_[Ruminococcus_bromii_YE282]	Ruminococcus_bromii_YE282
Org1_Gene2355	unique	2987	2595400346_IE32DRAFT_02436_hypothetical_protein_[Ruminococcus_bromii_YE282]	Ruminococcus_bromii_YE282
Org1_Gene2356	unique	2480	2595400347_IE32DRAFT_02437_Acyltransferase_[Ruminococcus_bromii_YE282]	Ruminococcus_bromii_YE282
Org1_Gene2358	unique	175	2595400349_IE32DRAFT_02439_3-oxoacyl-(acyl-carrier-protein)_synthase_[Ruminococcus_bromii_YE282]	Ruminococcus_bromii_YE282

Org1_Gene236	unique	1557	2595398180_IE32DRAFT_00261_hypothetical_protein_[Ruminococcus_bromii_YE282]	Ruminococcus_bromii_YE282
Org1_Gene2361	unique	2790	2595400352_IE32DRAFT_02442_NADPH-dependent_FMN_reductase_[Ruminococcus_bromii_YE282]	Ruminococcus_bromii_YE282
Org1_Gene2364	unique	2331	2595400355_IE32DRAFT_02445_two_component_transcriptional_regulator,_LytTR_family_[Ruminococcus_bromii_YE282]	Ruminococcus_bromii_YE282
Org1_Gene2365	unique	815	2595400356_IE32DRAFT_02446_GHKL_domain_[Ruminococcus_bromii_YE282]	Ruminococcus_bromii_YE282
Org1_Gene2366	unique	3324	2595400357_IE32DRAFT_02447_hypothetical_protein_[Ruminococcus_bromii_YE282]	Ruminococcus_bromii_YE282
Org1_Gene2367	unique	2342	2595400358_IE32DRAFT_02448_hypothetical_protein_[Ruminococcus_bromii_YE282]	Ruminococcus_bromii_YE282
Org1_Gene2368	unique	1837	2595400359_IE32DRAFT_02449_ABC-2_family_transporter_protein_[Ruminococcus_bromii_YE282]	Ruminococcus_bromii_YE282
Org1_Gene2369	unique	1880	2595400360_IE32DRAFT_02450_ABC-2_family_transporter_protein_[Ruminococcus_bromii_YE282]	Ruminococcus_bromii_YE282
Org1_Gene2370	unique	2526	2595400361_IE32DRAFT_02451_ABC-type_multidrug_transport_system,_ATPase_component_[Ruminococcus_bromii_YE282]	Ruminococcus_bromii_YE282
Org1_Gene2371	unique	2692	2595400362_IE32DRAFT_02452_hypothetical_protein_[Ruminococcus_bromii_YE282]	Ruminococcus_bromii_YE282
Org1_Gene2372	unique	4402	2595400363_IE32DRAFT_02453_hypothetical_protein_[Ruminococcus_bromii_YE282]	Ruminococcus_bromii_YE282
Org1_Gene2374	unique	4060	2595400365_IE32DRAFT_02455_Transposase_[Ruminococcus_bromii_YE282]	Ruminococcus_bromii_YE282
Org1_Gene2376	unique	300	2595400368_IE32DRAFT_02458_Domain_of_unknown_function_(DUF4353)_[Ruminococcus_bromii_YE282]	Ruminococcus_bromii_YE282
Org1_Gene2377	unique	2448	2595400369_IE32DRAFT_02459_hypothetical_protein_[Ruminococcus_bromii_YE282]	Ruminococcus_bromii_YE282
Org1_Gene2378	unique	2296	2595400370_IE32DRAFT_02460_VTC_domain_[Ruminococcus_bromii_YE282]	Ruminococcus_bromii_YE282
Org1_Gene2379	unique	2454	2595400371_IE32DRAFT_02461_Response_regulators_consisting_of_a_CheY-like_receiver_domain_and_a_winged-helix_DNA-binding_domain_[Ruminoc	Ruminococcus_bromii_YE282
Org1_Gene2380	unique	946	2595400372_IE32DRAFT_02462_His_Kinase_A_(phospho-acceptor)_domain/Histidine_kinase-,_DNA_gyrase_B-,_and_HSP90-like_ATPase_[Ruminococcus_	Ruminococcus_bromii_YE282
Org1_Gene2381	unique	1755	2595400373_IE32DRAFT_02463_Transcriptional_regulator_[Ruminococcus_bromii_YE282]	Ruminococcus_bromii_YE282
Org1_Gene2386	unique	4348	2595400378_IE32DRAFT_02468_Virus_attachment_protein_p12_family_[Ruminococcus_bromii_YE282]	Ruminococcus_bromii_YE282
Org1_Gene2387	unique	3574	2595400379_IE32DRAFT_02469_Mn-dependent_transcriptional_regulator_[Ruminococcus_bromii_YE282]	Ruminococcus_bromii_YE282
Org1_Gene2388	unique	3993	2595400380_IE32DRAFT_02470_Copper_chaperone_[Ruminococcus_bromii_YE282]	Ruminococcus_bromii_YE282
Org1_Gene2389	unique	1068	2595400381_IE32DRAFT_02471_Leucine_rich_repeats_(6_copies)_[Ruminococcus_bromii_YE282]	Ruminococcus_bromii_YE282
Org1_Gene239	unique	836	2595398183_IE32DRAFT_00264_hypothetical_protein_[Ruminococcus_bromii_YE282]	Ruminococcus_bromii_YE282
Org1_Gene2392	unique	1396	2595400384_IE32DRAFT_02474_Site-specific_recombinase_XerD_[Ruminococcus_bromii_YE282]	Ruminococcus_bromii_YE282
Org1_Gene2393	unique	3542	2595400385_IE32DRAFT_02475_hypothetical_protein_[Ruminococcus_bromii_YE282]	Ruminococcus_bromii_YE282
Org1_Gene2394	unique	3408	2595400386_IE32DRAFT_02476_Predicted_transcriptional_regulators_[Ruminococcus_bromii_YE282]	Ruminococcus_bromii_YE282
Org1_Gene2395	unique	3363	2595400387_IE32DRAFT_02477_hypothetical_protein_[Ruminococcus_bromii_YE282]	Ruminococcus_bromii_YE282
Org1_Gene2396	unique	3463	2595400388_IE32DRAFT_02478_hypothetical_protein_[Ruminococcus_bromii_YE282]	Ruminococcus_bromii_YE282
Org1_Gene2397	unique	2701	2595400389_IE32DRAFT_02479_hypothetical_protein_[Ruminococcus_bromii_YE282]	Ruminococcus_bromii_YE282
Org1_Gene2398	unique	4370	2595400390_IE32DRAFT_02480_hypothetical_protein_[Ruminococcus_bromii_YE282]	Ruminococcus_bromii_YE282
Org1_Gene2399	unique	4186	2595400391_IE32DRAFT_02481_Helix-turn-helix_domain_[Ruminococcus_bromii_YE282]	Ruminococcus_bromii_YE282
Org1_Gene24	unique	147	2595397947_IE32DRAFT_00028_SH3_domain_protein_[Ruminococcus_bromii_YE282]	Ruminococcus_bromii_YE282
Org1_Gene2400	unique	3438	2595400392_IE32DRAFT_02482_Helix-turn-helix_[Ruminococcus_bromii_YE282]	Ruminococcus_bromii_YE282
Org1_Gene2401	unique	3460	2595400393_IE32DRAFT_02483_Pentapeptide_repeats_(8_copies)_[Ruminococcus_bromii_YE282]	Ruminococcus_bromii_YE282
Org1_Gene2403	unique	160	2595400395_IE32DRAFT_02485_Cna_protein_B-type_domain_[Ruminococcus_bromii_YE282]	Ruminococcus_bromii_YE282
Org1_Gene2404	unique	597	2595400396_IE32DRAFT_02486_Cna_protein_B-type_domain_[Ruminococcus_bromii_YE282]	Ruminococcus_bromii_YE282
Org1_Gene2405	unique	1908	2595400397_IE32DRAFT_02487_LPXTG-site_transpeptidase_(sortase)_family_protein_[Ruminococcus_bromii_YE282]	Ruminococcus_bromii_YE282
Org1_Gene2406	unique	1720	2595400398_IE32DRAFT_02488_hypothetical_protein_[Ruminococcus_bromii_YE282]	Ruminococcus_bromii_YE282
Org1_Gene2408	unique	960	2595400400_IE32DRAFT_02490_hypothetical_protein_[Ruminococcus_bromii_YE282]	Ruminococcus_bromii_YE282
Org1_Gene2409	unique	195	2595400401_IE32DRAFT_02491_hypothetical_protein_[Ruminococcus_bromii_YE282]	Ruminococcus_bromii_YE282
Org1_Gene2410	unique	1441	2595400402_IE32DRAFT_02492_CotH_protein_[Ruminococcus_bromii_YE282]	Ruminococcus_bromii_YE282
Org1_Gene2411	unique	2538	2595400403_IE32DRAFT_02493_hypothetical_protein_[Ruminococcus_bromii_YE282]	Ruminococcus_bromii_YE282
Org1_Gene2412	unique	4125	2595400404_IE32DRAFT_02494_hypothetical_protein_[Ruminococcus_bromii_YE282]	Ruminococcus_bromii_YE282
Org1_Gene2413	unique	1280	2595400405_IE32DRAFT_02495_hypothetical_protein_[Ruminococcus_bromii_YE282]	Ruminococcus_bromii_YE282
Org1_Gene2414	unique	3001	2595400406_IE32DRAFT_02496_zinc-ribbon_domain_[Ruminococcus_bromii_YE282]	Ruminococcus_bromii_YE282
Org1_Gene2415	unique	1807	2595400407_IE32DRAFT_02497_STAS_domain_[Ruminococcus_bromii_YE282]	Ruminococcus_bromii_YE282
Org1_Gene2416	unique	1007	2595400408_IE32DRAFT_02498_Sulfate_permease_and_related_transporters_(MFS_superfamily)_[Ruminococcus_bromii_YE282]	Ruminococcus_bromii_YE282
Org1_Gene2418	unique	3538	2595400410_IE32DRAFT_02500_Transposon-encoded_protein_TnpV_[Ruminococcus_bromii_YE282]	Ruminococcus_bromii_YE282
Org1_Gene2419	unique	1377	2595400411_IE32DRAFT_02501_Virulence_protein_[Ruminococcus_bromii_YE282]	Ruminococcus_bromii_YE282
Org1_Gene242	unique	3498	2595398186_IE32DRAFT_00267_putative_membrane_protein,_TIGR04086_family_[Ruminococcus_bromii_YE282]	Ruminococcus_bromii_YE282
Org1_Gene2420	unique	1624	2595400412_IE32DRAFT_02502_hypothetical_protein_[Ruminococcus_bromii_YE282]	Ruminococcus_bromii_YE282
Org1_Gene2426	unique	3289	2595400418_IE32DRAFT_02508_Predicted_transcriptional_regulators_[Ruminococcus_bromii_YE282]	Ruminococcus_bromii_YE282
Org1_Gene2427	unique	3103	2595400419_IE32DRAFT_02509_Aldo/keto_reductase_family_[Ruminococcus_bromii_YE282]	Ruminococcus_bromii_YE282
Org1_Gene243	unique	3236	2595398187_IE32DRAFT_00268_TspO_and_MBR_related_proteins_[Ruminococcus_bromii_YE282]	Ruminococcus_bromii_YE282
Org1_Gene2430	unique	3148	2595400422_IE32DRAFT_02512_Flavodoxins_[Ruminococcus_bromii_YE282]	Ruminococcus_bromii_YE282
Org1_Gene2431	unique	563	2595400423_IE32DRAFT_02513_LXG_domain_of_WXG_superfamily_[Ruminococcus_bromii_YE282]	Ruminococcus_bromii_YE282
Org1_Gene2433	unique	3487	2595400426_IE32DRAFT_02516_hypothetical_protein_[Ruminococcus_bromii_YE282]	Ruminococcus_bromii_YE282
Org1_Gene2434	unique	1057	2595400429_IE32DRAFT_02519_Site-specific_recombinase_XerD_[Ruminococcus_bromii_YE282]	Ruminococcus_bromii_YE282
Org1_Gene2435	unique	3975	2595400430_IE32DRAFT_02520_hypothetical_protein_[Ruminococcus_bromii_YE282]	Ruminococcus_bromii_YE282
Org1_Gene2438	unique	2612	2595400437_IE32DRAFT_02528_Uridine_phosphorylase_[Ruminococcus_bromii_YE282]	Ruminococcus_bromii_YE282
Org1_Gene2439	unique	1809	2595400438_IE32DRAFT_02529_Predicted_membrane_protein_[Ruminococcus_bromii_YE282]	Ruminococcus_bromii_YE282
Org1_Gene244	unique	201	2595398188_IE32DRAFT_00269_PASTA_domain/YARHG_domain/Protein_kinase_domain_[Ruminococcus_bromii_YE282]	Ruminococcus_bromii_YE282
Org1_Gene2440	unique	2286	2595400439_IE32DRAFT_02530_Predicted_glutamine_amidotransferase_[Ruminococcus_bromii_YE282]	Ruminococcus_bromii_YE282
Org1_Gene2443	unique	4234	2595400442_IE32DRAFT_02533_Transposon-encoded_protein_TnpW_[Ruminococcus_bromii_YE282]	Ruminococcus_bromii_YE282
Org1_Gene2444	unique	3608	2595400443_IE32DRAFT_02534_Cysteine-rich_VLP_[Ruminococcus_bromii_YE282]	Ruminococcus_bromii_YE282
Org1_Gene2445	unique	2304	2595400444_IE32DRAFT_02535_hypothetical_protein_[Ruminococcus_bromii_YE282]	Ruminococcus_bromii_YE282
Org1_Gene2449	unique	2519	2595400448_IE32DRAFT_02539_hypothetical_protein_[Ruminococcus_bromii_YE282]	Ruminococcus_bromii_YE282
Org1_Gene245	unique	418	2595398189_IE32DRAFT_00270_hypothetical_protein_[Ruminococcus_bromii_YE282]	Ruminococcus_bromii_YE282
Org1_Gene2450	unique	2913	2595400449_IE32DRAFT_02540_hypothetical_protein_[Ruminococcus_bromii_YE282]	Ruminococcus_bromii_YE282
Org1_Gene2451	unique	2869	2595400450_IE32DRAFT_02541_hypothetical_protein_[Ruminococcus_bromii_YE282]	Ruminococcus_bromii_YE282
Org1_Gene2452	unique	1953	2595400451_IE32DRAFT_02542_hypothetical_protein_[Ruminococcus_bromii_YE282]	Ruminococcus_bromii_YE282
Org1_Gene2454	unique	3162	2595400453_IE32DRAFT_02544_hypothetical_protein_[Ruminococcus_bromii_YE282]	Ruminococcus_bromii_YE282
Org1_Gene2455	unique	1400	2595400454_IE32DRAFT_02545_CAAX_protease_self-immunity_[Ruminococcus_bromii_YE282]	Ruminococcus_bromii_YE282
Org1_Gene2456	unique	4207	2595400455_IE32DRAFT_02546_hypothetical_protein_[Ruminococcus_bromii_YE282]	Ruminococcus_bromii_YE282
Org1_Gene2459	unique	4027	2595400458_IE32DRAFT_02549_hypothetical_protein_[Ruminococcus_bromii_YE282]	Ruminococcus_bromii_YE282
Org1_Gene246	unique	3240	2595398190_IE32DRAFT_00271_hypothetical_protein_[Ruminococcus_bromii_YE282]	Ruminococcus_bromii_YE282
Org1_Gene2460	unique	4180	2595400459_IE32DRAFT_02550_hypothetical_protein_[Ruminococcus_bromii_YE282]	Ruminococcus_bromii_YE282
Org1_Gene2461	unique	1415	2595400460_IE32DRAFT_02551_Lyzozyme_M1_(1,4-beta-N-acetylmuramidase)_[Ruminococcus_bromii_YE282]	Ruminococcus_bromii_YE282
Org1_Gene2462	unique	4292	2595400461_IE32DRAFT_02552_Ribbon-helix-helix_protein,_copG_family_[Ruminococcus_bromii_YE282]	Ruminococcus_bromii_YE282
Org1_Gene2463	unique	4408	2595400462_IE32DRAFT_02553_hypothetical_protein_[Ruminococcus_bromii_YE282]	Ruminococcus_bromii_YE282
Org1_Gene2464	unique	4009	2595400463_IE32DRAFT_02554_hypothetical_protein_[Ruminococcus_bromii_YE282]	Ruminococcus_bromii_YE282
Org1_Gene2467	unique	3935	2595400466_IE32DRAFT_02557_hypothetical_protein_[Ruminococcus_bromii_YE282]	Ruminococcus_bromii_YE282
Org1_Gene2468	unique	3353	2595400467_IE32DRAFT_02558_Predicted_transcriptional_regulators_[Ruminococcus_bromii_YE282]	Ruminococcus_bromii_YE282
Org1_Gene2469	unique	4024	2595400468_IE32DRAFT_02559_Aldo/keto_reductase_family_[Ruminococcus_bromii_YE282]	Ruminococcus_bromii_YE282
Org1_Gene247	unique	763	2595398191_IE32DRAFT_00272_Putative_peptidoglycan-binding_domain-containing_protein_[Ruminococcus_bromii_YE282]	Ruminococcus_bromii_YE282
Org1_Gene2470	unique	2778	2595400469_IE32DRAFT_02560_Aldo/keto_reductase_family_[Ruminococcus_bromii_YE282]	Ruminococcus_bromii_YE282
Org1_Gene2471	unique	2801	2595400470_IE32DRAFT_02561_hypothetical_protein_[Ruminococcus_bromii_YE282]	Ruminococcus_bromii_YE282
Org1_Gene2472	unique	3418	2595400471_IE32DRAFT_02562_hypothetical_protein_[Ruminococcus_bromii_YE282]	Ruminococcus_bromii_YE282
Org1_Gene2473	unique	4332	2595400472_IE32DRAFT_02563_ABC_transporter_[Ruminococcus_bromii_YE282]	Ruminococcus_bromii_YE282
Org1_Gene2474	unique	4064	2595400473_IE32DRAFT_02564_hypothetical_protein_[Ruminococcus_bromii_YE282]	Ruminococcus_bromii_YE282
Org1_Gene2475	unique	1514	2595400474_IE32DRAFT_02565_Predicted_Zn-dependent_protease_[Ruminococcus_bromii_YE282]	Ruminococcus_bromii_YE282
Org1_Gene2476	unique	2280	2595400475_IE32DRAFT_02566_transcriptional_regulator,_MerR_family_[Ruminococcus_bromii_YE282]	Ruminococcus_bromii_YE282
Org1_Gene2477	unique	2535	2595400476_IE32DRAFT_02567_Predicted_phosphatase/phosphohexomutase_[Ruminococcus_bromii_YE282]	Ruminococcus_bromii_YE282
Org1_Gene2478	unique	3877	2595400477_IE32DRAFT_02568_hypothetical_protein_[Ruminococcus_bromii_YE282]	Ruminococcus_bromii_YE282
Org1_Gene2479	unique	3890	2595400478_IE32DRAFT_02569_hypothetical_protein_[Ruminococcus_bromii_YE282]	Ruminococcus_bromii_YE282
Org1_Gene2480	unique	1292	2595400479_IE32DRAFT_02570_Glycosyl_hydrolases_family_25/Bacterial_Ig-like_domain_(group_2)_[Ruminococcus_bromii_YE282]	Ruminococcus_bromii_YE282
Org1_Gene2481	unique	2355	2595400480_IE32DRAFT_02571_Bacterial_Ig-like_domain_(group_2)_[Ruminococcus_bromii_YE282]	Ruminococcus_bromii_YE282
Org1_Gene2485	unique	540	2595400484_IE32DRAFT_02575_ABC-type_bacteriocin/lantibiotic_exporters,_contain_an_N-terminal_double-glycine_peptidase_domain_[Ruminococcus_	Ruminococcus_bromii_YE282
Org1_Gene2486	unique	1039	2595400485_IE32DRAFT_02576_Site-specific_recombinase_XerD_[Ruminococcus_bromii_YE282]	Ruminococcus_bromii_YE282
Org1_Gene2487	unique	4399	2595400486_IE32DRAFT_02577_hypothetical_protein_[Ruminococcus_bromii_YE282]	Ruminococcus_bromii_YE282
Org1_Gene2488	unique	2230	2595400487_IE32DRAFT_02578_Biotin-lipoyl_like_[Ruminococcus_bromii_YE282]	Ruminococcus_bromii_YE282
Org1_Gene2489	unique	2675	2595400488_IE32DRAFT_02579_ABC_transporter_[Ruminococcus_bromii_YE282]	Ruminococcus_bromii_YE282
Org1_Gene253	unique	2049	2595398197_IE32DRAFT_00278_D-alanyl-D-alanine_carboxypeptidase_[Ruminococcus_bromii_YE282]	Ruminococcus_bromii_YE282
Org1_Gene255	unique	1124	2595398199_IE32DRAFT_00280_hypothetical_protein_[Ruminococcus_bromii_YE282]	Ruminococcus_bromii_YE282
Org1_Gene256	unique	2605	2595398200_IE32DRAFT_00281_hypothetical_protein_[Ruminococcus_bromii_YE282]	Ruminococcus_bromii_YE282
Org1_Gene257	unique	4295	2595398201_IE32DRAFT_00282_Spore_coat_associated_protein_JA_(CotJA)_[Ruminococcus_bromii_YE282]	Ruminococcus_bromii_YE282
Org1_Gene258	unique	4011	2595398202_IE32DRAFT_00283_CotJB_protein_[Ruminococcus_bromii_YE282]	Ruminococcus_bromii_YE282
Org1_Gene26	unique	2463	2595397949_IE32DRAFT_00030_hypothetical_protein_[Ruminococcus_bromii_YE282]	Ruminococcus_bromii_YE282
Org1_Gene260	unique	2939	2595398204_IE32DRAFT_00285_Acetyltransferases,_including_N-acetylases_of_ribosomal_proteins_[Ruminococcus_bromii_YE282]	Ruminococcus_bromii_YE282
Org1_Gene261	unique	2958	2595398205_IE32DRAFT_00286_Acetyltransferase_(GNAT)_domain_[Ruminococcus_bromii_YE282]	Ruminococcus_bromii_YE282
Org1_Gene264	unique	2353	2595398208_IE32DRAFT_00289_tRNA_threonylcarbamoyl_adenosine_modification_protein_YeaZ_[Ruminococcus_bromii_YE282]	Ruminococcus_bromii_YE282
Org1_Gene267	unique	1101	2595398211_IE32DRAFT_00292_Bacterial_cell_division_membrane_protein_[Ruminococcus_bromii_YE282]	Ruminococcus_bromii_YE282
Org1_Gene268	unique	924	2595398212_IE32DRAFT_00293_Uncharacterized_protein_conserved_in_bacteria_[Ruminococcus_bromii_YE282]	Ruminococcus_bromii_YE282
Org1_Gene27	unique	1473	2595397950_IE32DRAFT_00031_Predicted_integral_membrane_protein_[Ruminococcus_bromii_YE282]	Ruminococcus_bromii_YE282
Org1_Gene270	unique	1078	2595398214_IE32DRAFT_00295_cysteine_desulfurase_family_protein_[Ruminococcus_bromii_YE282]	Ruminococcus_bromii_YE282
Org1_Gene271	unique	4106	2595398215_IE32DRAFT_00296_Protein_of_unknown_function_(DUF3343)_[Ruminococcus_bromii_YE282]	Ruminococcus_bromii_YE282
Org1_Gene272	unique	2041	2595398216_IE32DRAFT_00297_ABC-2_family_transporter_protein_[Ruminococcus_bromii_YE282]	Ruminococcus_bromii_YE282
Org1_Gene273	unique	1728	2595398217_IE32DRAFT_00298_ABC-type_multidrug_transport_system,_ATPase_component_[Ruminococcus_bromii_YE282]	Ruminococcus_bromii_YE282
Org1_Gene279	unique	3325	2595398223_IE32DRAFT_00304_preprotein_translocase,_YajC_subunit_[Ruminococcus_bromii_YE282]	Ruminococcus_bromii_YE282
Org1_Gene28	unique	2527	2595397951_IE32DRAFT_00032_hypothetical_protein_[Ruminococcus_bromii_YE282]	Ruminococcus_bromii_YE282
Org1_Gene280	unique	1014	2595398224_IE32DRAFT_00305_FtsX-like_permease_family_[Ruminococcus_bromii_YE282]	Ruminococcus_bromii_YE282
Org1_Gene282	unique	731	2595398226_IE32DRAFT_00307_FtsX-like_permease_family_[Ruminococcus_bromii_YE282]	Ruminococcus_bromii_YE282

Org1_Gene283	unique	3326	2595398227_IE32DRAFT_00308_hypothetical_protein_[Ruminococcus_bromii_YE282]	Ruminococcus_bromii_YE282
Org1_Gene284	unique	420	2595398228_IE32DRAFT_00309_Trypsin-like_serine_proteases,_typically_periplasmic,_contain_C-terminal_PDZ_domain_[Ruminococcus_bromii_YE282]	Ruminococcus_bromii_YE282
Org1_Gene285	unique	1714	2595398229_IE32DRAFT_00310_Exonuclease_[Ruminococcus_bromii_YE282]	Ruminococcus_bromii_YE282
Org1_Gene29	unique	3048	2595397952_IE32DRAFT_00033_hypothetical_protein_[Ruminococcus_bromii_YE282]	Ruminococcus_bromii_YE282
Org1_Gene298	unique	1587	2595398242_IE32DRAFT_00323_Zn-dependent_dipeptidase,_microsomal_dipeptidase_homolog_[Ruminococcus_bromii_YE282]	Ruminococcus_bromii_YE282
Org1_Gene299	unique	3494	2595398243_IE32DRAFT_00324_TIGR00252_family_protein_[Ruminococcus_bromii_YE282]	Ruminococcus_bromii_YE282
Org1_Gene30	unique	1333	2595397953_IE32DRAFT_00034_Fucose_4-O-acetylase_and_related_acetyltransferases_[Ruminococcus_bromii_YE282]	Ruminococcus_bromii_YE282
Org1_Gene302	unique	2450	2595398246_IE32DRAFT_00327_signal_peptidase_I,_bacterial_type_[Ruminococcus_bromii_YE282]	Ruminococcus_bromii_YE282
Org1_Gene307	unique	2206	2595398251_IE32DRAFT_00332_DivIVA_domain_[Ruminococcus_bromii_YE282]	Ruminococcus_bromii_YE282
Org1_Gene308	unique	2219	2595398252_IE32DRAFT_00333_Uncharacterized_conserved_protein,_contains_S4-like_domain_[Ruminococcus_bromii_YE282]	Ruminococcus_bromii_YE282
Org1_Gene31	unique	1501	2595397954_IE32DRAFT_00035_Putative_GTPases_(G3E_family)_[Ruminococcus_bromii_YE282]	Ruminococcus_bromii_YE282
Org1_Gene311	unique	883	2595398255_IE32DRAFT_00336_HlyD_family_secretion_protein_[Ruminococcus_bromii_YE282]	Ruminococcus_bromii_YE282
Org1_Gene312	unique	1532	2595398256_IE32DRAFT_00337_tRNA_dimethylallyltransferase_[Ruminococcus_bromii_YE282]	Ruminococcus_bromii_YE282
Org1_Gene313	unique	264	2595398257_IE32DRAFT_00338_DNA_mismatch_repair_protein_MutL_[Ruminococcus_bromii_YE282]	Ruminococcus_bromii_YE282
Org1_Gene317	unique	2516	2595398261_IE32DRAFT_00342_Sporulation_protein_YunB_(Spo_YunB)_[Ruminococcus_bromii_YE282]	Ruminococcus_bromii_YE282
Org1_Gene318	unique	3060	2595398262_IE32DRAFT_00343_hypothetical_protein_[Ruminococcus_bromii_YE282]	Ruminococcus_bromii_YE282
Org1_Gene32	unique	1761	2595397955_IE32DRAFT_00036_Predicted_xylanase/chitin_deacetylase_[Ruminococcus_bromii_YE282]	Ruminococcus_bromii_YE282
Org1_Gene320	unique	1253	2595398264_IE32DRAFT_00345_Xaa-Pro_aminopeptidase_[Ruminococcus_bromii_YE282]	Ruminococcus_bromii_YE282
Org1_Gene325	unique	2796	2595398269_IE32DRAFT_00350_putative_HD_superfamily_hydrolase_of_NAD_metabolism_[Ruminococcus_bromii_YE282]	Ruminococcus_bromii_YE282
Org1_Gene326	unique	2671	2595398270_IE32DRAFT_00351_nicotinate-nucleotide_adenylyltransferase_(EC_2.7.7.18)_[Ruminococcus_bromii_YE282]	Ruminococcus_bromii_YE282
Org1_Gene33	unique	4283	2595397956_IE32DRAFT_00037_Maltose_acetyltransferase_[Ruminococcus_bromii_YE282]	Ruminococcus_bromii_YE282
Org1_Gene331	unique	1797	2595398275_IE32DRAFT_00356_Radical_SAM_superfamily_[Ruminococcus_bromii_YE282]	Ruminococcus_bromii_YE282
Org1_Gene336	unique	949	2595398280_IE32DRAFT_00361_DNA_protecting_protein_DprA_[Ruminococcus_bromii_YE282]	Ruminococcus_bromii_YE282
Org1_Gene337	unique	2107	2595398281_IE32DRAFT_00362_rRNA_methylases_[Ruminococcus_bromii_YE282]	Ruminococcus_bromii_YE282
Org1_Gene34	unique	1031	2595397957_IE32DRAFT_00038_Bacterial_Ig-like_domain_(group_2)/L,D-transpeptidase_catalytic_domain_[Ruminococcus_bromii_YE282]	Ruminococcus_bromii_YE282
Org1_Gene341	unique	382	2595398286_IE32DRAFT_00367_Putative_glycosyl/glycerophosphate_transferases_involved_in_teichoic_acid_biosynthesis_TagF/TagB/EpsJ/RodC_[Rumin	Ruminococcus_bromii_YE282
Org1_Gene342	unique	2493	2595398287_IE32DRAFT_00368_Pentose-5-phosphate-3-epimerase_[Ruminococcus_bromii_YE282]	Ruminococcus_bromii_YE282
Org1_Gene343	unique	1325	2595398288_IE32DRAFT_00369_Threonine_dehydrogenase_and_related_Zn-dependent_dehydrogenases_[Ruminococcus_bromii_YE282]	Ruminococcus_bromii_YE282
Org1_Gene344	unique	2399	2595398289_IE32DRAFT_00370_2-C-methyl-D-erythritol_4-phosphate_cytidylyltransferase_[Ruminococcus_bromii_YE282]	Ruminococcus_bromii_YE282
Org1_Gene345	unique	538	2595398290_IE32DRAFT_00371_Pectate_lyase_superfamily_protein_[Ruminococcus_bromii_YE282]	Ruminococcus_bromii_YE282
Org1_Gene346	unique	4157	2595398291_IE32DRAFT_00372_Protein_of_unknown_function_(DUF3006)_[Ruminococcus_bromii_YE282]	Ruminococcus_bromii_YE282
Org1_Gene347	unique	1695	2595398292_IE32DRAFT_00373_Predicted_hydrolase_(metallo-beta-lactamase_superfamily)_[Ruminococcus_bromii_YE282]	Ruminococcus_bromii_YE282
Org1_Gene350	unique	3260	2595398295_IE32DRAFT_00376_Transcriptional_regulators_[Ruminococcus_bromii_YE282]	Ruminococcus_bromii_YE282
Org1_Gene351	unique	2303	2595398296_IE32DRAFT_00377_hypothetical_protein_[Ruminococcus_bromii_YE282]	Ruminococcus_bromii_YE282
Org1_Gene352	unique	1708	2595398297_IE32DRAFT_00378_Predicted_membrane_protein_[Ruminococcus_bromii_YE282]	Ruminococcus_bromii_YE282
Org1_Gene354	unique	2608	2595398299_IE32DRAFT_00380_Methyltransferase_domain_[Ruminococcus_bromii_YE282]	Ruminococcus_bromii_YE282
Org1_Gene357	unique	3397	2595398302_IE32DRAFT_00383_Transcriptional_regulators_[Ruminococcus_bromii_YE282]	Ruminococcus_bromii_YE282
Org1_Gene358	unique	2430	2595398303_IE32DRAFT_00384_Predicted_membrane_protein_[Ruminococcus_bromii_YE282]	Ruminococcus_bromii_YE282
Org1_Gene359	unique	3223	2595398304_IE32DRAFT_00385_hypothetical_protein_[Ruminococcus_bromii_YE282]	Ruminococcus_bromii_YE282
Org1_Gene36	unique	3120	2595397959_IE32DRAFT_00040_G/U_mismatch-specific_uracil-DNA_glycosylase_(EC_3.2.2.-)_[Ruminococcus_bromii_YE282]	Ruminococcus_bromii_YE282
Org1_Gene360	unique	1970	2595398305_IE32DRAFT_00386_histidinol_phosphate_phosphatase,_HisJ_family_[Ruminococcus_bromii_YE282]	Ruminococcus_bromii_YE282
Org1_Gene363	unique	3811	2595398308_IE32DRAFT_00389_hypothetical_protein_[Ruminococcus_bromii_YE282]	Ruminococcus_bromii_YE282
Org1_Gene365	unique	306	2595398310_IE32DRAFT_00391_Archaeal/vacuolar-type_H+-ATPase_subunit_I_[Ruminococcus_bromii_YE282]	Ruminococcus_bromii_YE282
Org1_Gene368	unique	2724	2595398313_IE32DRAFT_00394_Archaeal/vacuolar-type_H+-ATPase_subunit_E_[Ruminococcus_bromii_YE282]	Ruminococcus_bromii_YE282
Org1_Gene372	unique	3097	2595398317_IE32DRAFT_00398_hypothetical_protein_[Ruminococcus_bromii_YE282]	Ruminococcus_bromii_YE282
Org1_Gene373	unique	0	2595398318_IE32DRAFT_00399_Polyketide_synthase_modules_and_related_proteins_[Ruminococcus_bromii_YE282]	Ruminococcus_bromii_YE282
Org1_Gene374	unique	13	2595398319_IE32DRAFT_00400_KR_domain/Beta-ketoacyl_synthase,_N-terminal_domain/Beta-ketoacyl_synthase,_C-terminal_domain/Phosphopantethe	Ruminococcus_bromii_YE282
Org1_Gene375	unique	12	2595398320_IE32DRAFT_00401_KR_domain/Beta-ketoacyl_synthase,_N-terminal_domain/Beta-ketoacyl_synthase,_C-terminal_domain/Phosphopantethe	Ruminococcus_bromii_YE282
Org1_Gene376	unique	1874	2595398321_IE32DRAFT_00402_Phosphopantetheine_attachment_site_[Ruminococcus_bromii_YE282]	Ruminococcus_bromii_YE282
Org1_Gene377	unique	1	2595398322_IE32DRAFT_00403_Polyketide_synthase_modules_and_related_proteins_[Ruminococcus_bromii_YE282]	Ruminococcus_bromii_YE282
Org1_Gene378	unique	9	2595398323_IE32DRAFT_00404_KR_domain/Beta-ketoacyl_synthase,_N-terminal_domain/Beta-ketoacyl_synthase,_C-terminal_domain/Phosphopantethe	Ruminococcus_bromii_YE282
Org1_Gene379	unique	16	2595398324_IE32DRAFT_00405_Polyketide_synthase_dehydratase/Thioesterase_domain/Beta-ketoacyl_synthase,_N-terminal_domain/Beta-ketoacyl_syn	Ruminococcus_bromii_YE282
Org1_Gene380	unique	1620	2595398325_IE32DRAFT_00406_ABC-type_multidrug_transport_system,_ATPase_component_[Ruminococcus_bromii_YE282]	Ruminococcus_bromii_YE282
Org1_Gene381	unique	2054	2595398326_IE32DRAFT_00407_ABC-2_family_transporter_protein_[Ruminococcus_bromii_YE282]	Ruminococcus_bromii_YE282
Org1_Gene382	unique	2003	2595398327_IE32DRAFT_00408_hypothetical_protein_[Ruminococcus_bromii_YE282]	Ruminococcus_bromii_YE282
Org1_Gene383	unique	4170	2595398328_IE32DRAFT_00409_hypothetical_protein_[Ruminococcus_bromii_YE282]	Ruminococcus_bromii_YE282
Org1_Gene384	unique	2268	2595398329_IE32DRAFT_00410_ABC-type_multidrug_transport_system,_ATPase_component_[Ruminococcus_bromii_YE282]	Ruminococcus_bromii_YE282
Org1_Gene385	unique	2254	2595398330_IE32DRAFT_00411_ABC-2_family_transporter_protein_[Ruminococcus_bromii_YE282]	Ruminococcus_bromii_YE282
Org1_Gene386	unique	2272	2595398331_IE32DRAFT_00412_Phosphopantetheinyl_transferase_[Ruminococcus_bromii_YE282]	Ruminococcus_bromii_YE282
Org1_Gene387	unique	2441	2595398332_IE32DRAFT_00413_Zn-dependent_hydrolases,_including_glyoxylases_[Ruminococcus_bromii_YE282]	Ruminococcus_bromii_YE282
Org1_Gene388	unique	1538	2595398333_IE32DRAFT_00414_Acyl_transferase_domain_[Ruminococcus_bromii_YE282]	Ruminococcus_bromii_YE282
Org1_Gene389	unique	193	2595398334_IE32DRAFT_00415_malonyl_CoA-acyl_carrier_protein_transacylase_[Ruminococcus_bromii_YE282]	Ruminococcus_bromii_YE282
Org1_Gene39	unique	1479	2595397962_IE32DRAFT_00043_N-acetylglutamate_synthase_and_related_acetyltransferases_[Ruminococcus_bromii_YE282]	Ruminococcus_bromii_YE282
Org1_Gene390	unique	912	2595398335_IE32DRAFT_00416_hydroxymethylglutaryl-CoA_synthase_(EC_2.3.3.10)_[Ruminococcus_bromii_YE282]	Ruminococcus_bromii_YE282
Org1_Gene391	unique	2174	2595398336_IE32DRAFT_00417_Enoyl-CoA_hydratase/carnithine_racemase_[Ruminococcus_bromii_YE282]	Ruminococcus_bromii_YE282
Org1_Gene392	unique	2156	2595398337_IE32DRAFT_00418_Enoyl-CoA_hydratase/carnithine_racemase_[Ruminococcus_bromii_YE282]	Ruminococcus_bromii_YE282
Org1_Gene393	unique	4040	2595398338_IE32DRAFT_00419_hypothetical_protein_[Ruminococcus_bromii_YE282]	Ruminococcus_bromii_YE282
Org1_Gene394	unique	899	2595398339_IE32DRAFT_00420_3-oxoacyl-(acyl-carrier-protein)_synthase_[Ruminococcus_bromii_YE282]	Ruminococcus_bromii_YE282
Org1_Gene395	unique	2235	2595398340_IE32DRAFT_00421_3-oxoacyl-[acyl-carrier-protein]_reductase_(EC_1.1.1.100)_[Ruminococcus_bromii_YE282]	Ruminococcus_bromii_YE282
Org1_Gene396	unique	1366	2595398341_IE32DRAFT_00422_Membrane-associated_lipoprotein_involved_in_thiamine_biosynthesis_[Ruminococcus_bromii_YE282]	Ruminococcus_bromii_YE282
Org1_Gene397	unique	1680	2595398342_IE32DRAFT_00423_FtsX-like_permease_family_[Ruminococcus_bromii_YE282]	Ruminococcus_bromii_YE282
Org1_Gene398	unique	2322	2595398343_IE32DRAFT_00424_ABC-type_antimicrobial_peptide_transport_system,_ATPase_component_[Ruminococcus_bromii_YE282]	Ruminococcus_bromii_YE282
Org1_Gene399	unique	2762	2595398344_IE32DRAFT_00425_Transcriptional_regulator_[Ruminococcus_bromii_YE282]	Ruminococcus_bromii_YE282
Org1_Gene40	unique	3906	2595397963_IE32DRAFT_00044_Mor_transcription_activator_family_[Ruminococcus_bromii_YE282]	Ruminococcus_bromii_YE282
Org1_Gene400	unique	3889	2595398345_IE32DRAFT_00426_hypothetical_protein_[Ruminococcus_bromii_YE282]	Ruminococcus_bromii_YE282
Org1_Gene402	unique	1156	2595398347_IE32DRAFT_00428_hypothetical_protein_[Ruminococcus_bromii_YE282]	Ruminococcus_bromii_YE282
Org1_Gene403	unique	1435	2595398348_IE32DRAFT_00429_hypothetical_protein_[Ruminococcus_bromii_YE282]	Ruminococcus_bromii_YE282
Org1_Gene404	unique	3682	2595398349_IE32DRAFT_00430_hypothetical_protein_[Ruminococcus_bromii_YE282]	Ruminococcus_bromii_YE282
Org1_Gene406	unique	3640	2595398351_IE32DRAFT_00432_hypothetical_protein_[Ruminococcus_bromii_YE282]	Ruminococcus_bromii_YE282
Org1_Gene407	unique	3554	2595398352_IE32DRAFT_00433_hypothetical_protein_[Ruminococcus_bromii_YE282]	Ruminococcus_bromii_YE282
Org1_Gene408	unique	3316	2595398353_IE32DRAFT_00434_hypothetical_protein_[Ruminococcus_bromii_YE282]	Ruminococcus_bromii_YE282
Org1_Gene409	unique	3543	2595398354_IE32DRAFT_00435_hypothetical_protein_[Ruminococcus_bromii_YE282]	Ruminococcus_bromii_YE282
Org1_Gene41	unique	1858	2595397964_IE32DRAFT_00045_Acetyltransferases_[Ruminococcus_bromii_YE282]	Ruminococcus_bromii_YE282
Org1_Gene410	unique	4409	2595398355_IE32DRAFT_00436_hypothetical_protein_[Ruminococcus_bromii_YE282]	Ruminococcus_bromii_YE282
Org1_Gene411	unique	3010	2595398356_IE32DRAFT_00437_LysM_domain_[Ruminococcus_bromii_YE282]	Ruminococcus_bromii_YE282
Org1_Gene412	unique	1579	2595398357_IE32DRAFT_00438_hypothetical_protein_[Ruminococcus_bromii_YE282]	Ruminococcus_bromii_YE282
Org1_Gene413	unique	3700	2595398358_IE32DRAFT_00439_Bacteriophage_Mu_Gp45_protein_[Ruminococcus_bromii_YE282]	Ruminococcus_bromii_YE282
Org1_Gene414	unique	3668	2595398359_IE32DRAFT_00440_hypothetical_protein_[Ruminococcus_bromii_YE282]	Ruminococcus_bromii_YE282
Org1_Gene415	unique	1304	2595398360_IE32DRAFT_00441_Uncharacterized_homolog_of_phage_Mu_protein_gp47_[Ruminococcus_bromii_YE282]	Ruminococcus_bromii_YE282
Org1_Gene416	unique	3153	2595398361_IE32DRAFT_00442_Uncharacterized_protein_conserved_in_bacteria_(DUF2313)_[Ruminococcus_bromii_YE282]	Ruminococcus_bromii_YE282
Org1_Gene418	unique	2068	2595398363_IE32DRAFT_00444_Predicted_divalent_heavy-metal_cations_transporter_[Ruminococcus_bromii_YE282]	Ruminococcus_bromii_YE282
Org1_Gene419	unique	83	2595398365_IE32DRAFT_00446_Alpha_amylase,_catalytic_domain_[Ruminococcus_bromii_YE282]	Ruminococcus_bromii_YE282
Org1_Gene421	unique	2467	2595398367_IE32DRAFT_00448_Thymidylate_kinase_[Ruminococcus_bromii_YE282]	Ruminococcus_bromii_YE282
Org1_Gene423	unique	2731	2595398369_IE32DRAFT_00450_5,10-methenyltetrahydrofolate_synthetase_[Ruminococcus_bromii_YE282]	Ruminococcus_bromii_YE282
Org1_Gene424	unique	750	2595398370_IE32DRAFT_00451_conserved_hypothetical_protein,_YceG_family_[Ruminococcus_bromii_YE282]	Ruminococcus_bromii_YE282
Org1_Gene426	unique	1204	2595398372_IE32DRAFT_00453_alanine_racemase_[Ruminococcus_bromii_YE282]	Ruminococcus_bromii_YE282
Org1_Gene427	unique	1363	2595398373_IE32DRAFT_00454_Sugar-transfer_associated_ATP-grasp_[Ruminococcus_bromii_YE282]	Ruminococcus_bromii_YE282
Org1_Gene428	unique	4424	2595398374_IE32DRAFT_00455_hypothetical_protein_[Ruminococcus_bromii_YE282]	Ruminococcus_bromii_YE282
Org1_Gene431	unique	1869	2595398377_IE32DRAFT_00458_hypothetical_protein_[Ruminococcus_bromii_YE282]	Ruminococcus_bromii_YE282
Org1_Gene432	unique	1026	2595398378_IE32DRAFT_00459_hypothetical_protein_[Ruminococcus_bromii_YE282]	Ruminococcus_bromii_YE282
Org1_Gene435	unique	2885	2595398381_IE32DRAFT_00462_hypothetical_protein_[Ruminococcus_bromii_YE282]	Ruminococcus_bromii_YE282
Org1_Gene436	unique	3810	2595398382_IE32DRAFT_00463_hypothetical_protein_[Ruminococcus_bromii_YE282]	Ruminococcus_bromii_YE282
Org1_Gene437	unique	3678	2595398383_IE32DRAFT_00464_hypothetical_protein_[Ruminococcus_bromii_YE282]	Ruminococcus_bromii_YE282
Org1_Gene44	unique	964	2595397967_IE32DRAFT_00048_ABC-type_Na+_efflux_pump,_permease_component_[Ruminococcus_bromii_YE282]	Ruminococcus_bromii_YE282
Org1_Gene441	unique	3238	2595398387_IE32DRAFT_00468_Acetyltransferases_[Ruminococcus_bromii_YE282]	Ruminococcus_bromii_YE282
Org1_Gene444	unique	2665	2595398390_IE32DRAFT_00471_Methylase_involved_in_ubiquinone/menaquinone_biosynthesis_[Ruminococcus_bromii_YE282]	Ruminococcus_bromii_YE282
Org1_Gene445	unique	257	2595398391_IE32DRAFT_00472_heavy_metal-(Cd/Co/Hg/Pb/Zn)-translocating_P-type_ATPase_[Ruminococcus_bromii_YE282]	Ruminococcus_bromii_YE282
Org1_Gene45	unique	1685	2595397968_IE32DRAFT_00049_ABC-type_uncharacterized_transport_system,_ATPase_component_[Ruminococcus_bromii_YE282]	Ruminococcus_bromii_YE282
Org1_Gene453	unique	3410	2595398399_IE32DRAFT_00480_Protein-tyrosine-phosphatase_[Ruminococcus_bromii_YE282]	Ruminococcus_bromii_YE282
Org1_Gene454	unique	3386	2595398400_IE32DRAFT_00481_ribosomal-protein-alanine_acetyltransferase_[Ruminococcus_bromii_YE282]	Ruminococcus_bromii_YE282
Org1_Gene456	unique	2774	2595398402_IE32DRAFT_00483_Domain_of_unknown_function_(DUF4190)_[Ruminococcus_bromii_YE282]	Ruminococcus_bromii_YE282
Org1_Gene459	unique	582	2595398405_IE32DRAFT_00486_hypothetical_protein_[Ruminococcus_bromii_YE282]	Ruminococcus_bromii_YE282
Org1_Gene460	unique	1198	2595398407_IE32DRAFT_00488_Uncharacterized_protein_conserved_in_bacteria_[Ruminococcus_bromii_YE282]	Ruminococcus_bromii_YE282
Org1_Gene461	unique	2797	2595398408_IE32DRAFT_00489_hypothetical_protein_[Ruminococcus_bromii_YE282]	Ruminococcus_bromii_YE282
Org1_Gene465	unique	3401	2595398412_IE32DRAFT_00493_Universal_stress_protein_family_[Ruminococcus_bromii_YE282]	Ruminococcus_bromii_YE282
Org1_Gene466	unique	1844	2595398413_IE32DRAFT_00494_HAD-superfamily_hydrolase,_subfamily_IIB_[Ruminococcus_bromii_YE282]	Ruminococcus_bromii_YE282
Org1_Gene471	unique	2026	2595398418_IE32DRAFT_00499_Prolipoprotein_diacylglyceryltransferase_[Ruminococcus_bromii_YE282]	Ruminococcus_bromii_YE282
Org1_Gene472	unique	3578	2595398419_IE32DRAFT_00500_hypothetical_protein_[Ruminococcus_bromii_YE282]	Ruminococcus_bromii_YE282
Org1_Gene477	unique	2314	2595398424_IE32DRAFT_00505_16S_rRNA_(guanine(527)-N(7))-methyltransferase_RsmG_[Ruminococcus_bromii_YE282]	Ruminococcus_bromii_YE282
Org1_Gene481	unique	1428	2595398428_IE32DRAFT_00509_membrane_protein_insertase,_YidC/Oxa1_family,_C-terminal_domain_[Ruminococcus_bromii_YE282]	Ruminococcus_bromii_YE282
Org1_Gene487	unique	756	2595398434_IE32DRAFT_00515_His_Kinase_A_(phospho-acceptor)_domain/Histidine_kinase-,_DNA_gyrase_B-,_and_HSP90-like_ATPase_[Ruminococcus_	Ruminococcus_bromii_YE282
Org1_Gene488	unique	2473	2595398435_IE32DRAFT_00516_Response_regulators_consisting_of_a_CheY-like_receiver_domain_and_a_winged-helix_DNA-binding_domain_[Ruminococ	Ruminococcus_bromii_YE282

Org1_Gene489	unique	2693	2595398436_IE32DRAFT_00517_Uncharacterized_protein_conserved_in_bacteria_[Ruminococcus_bromii_YE282]	Ruminococcus_bromii_YE282
Org1_Gene490	unique	3118	2595398437_IE32DRAFT_00518_hypothetical_protein_[Ruminococcus_bromii_YE282]	Ruminococcus_bromii_YE282
Org1_Gene491	unique	2850	2595398438_IE32DRAFT_00519_hypothetical_protein_[Ruminococcus_bromii_YE282]	Ruminococcus_bromii_YE282
Org1_Gene492	unique	4000	2595398439_IE32DRAFT_00520_hypothetical_protein_[Ruminococcus_bromii_YE282]	Ruminococcus_bromii_YE282
Org1_Gene494	unique	2870	2595398441_IE32DRAFT_00522_hypothetical_protein_[Ruminococcus_bromii_YE282]	Ruminococcus_bromii_YE282
Org1_Gene495	unique	1004	2595398442_IE32DRAFT_00523_Predicted_integral_membrane_protein_[Ruminococcus_bromii_YE282]	Ruminococcus_bromii_YE282
Org1_Gene498	unique	693	2595398445_IE32DRAFT_00526_DHHW_protein_[Ruminococcus_bromii_YE282]	Ruminococcus_bromii_YE282
Org1_Gene5	unique	402	2595397925_IE32DRAFT_00006_DNA_polymerase_III,_subunit_gamma_and_tau_[Ruminococcus_bromii_YE282]	Ruminococcus_bromii_YE282
Org1_Gene50	unique	4298	2595397973_IE32DRAFT_00054_hypothetical_protein_[Ruminococcus_bromii_YE282]	Ruminococcus_bromii_YE282
Org1_Gene500	unique	3025	2595398447_IE32DRAFT_00528_hypothetical_protein_[Ruminococcus_bromii_YE282]	Ruminococcus_bromii_YE282
Org1_Gene508	unique	3235	2595398455_IE32DRAFT_00536_C_GCAxxG_C_C_family_probable_redox_protein_[Ruminococcus_bromii_YE282]	Ruminococcus_bromii_YE282
Org1_Gene51	unique	3534	2595397974_IE32DRAFT_00055_Predicted_transcriptional_regulators_[Ruminococcus_bromii_YE282]	Ruminococcus_bromii_YE282
Org1_Gene510	unique	97	2595398457_IE32DRAFT_00538_Predicted_membrane_protein_[Ruminococcus_bromii_YE282]	Ruminococcus_bromii_YE282
Org1_Gene512	unique	755	2595398459_IE32DRAFT_00540_Predicted_membrane_protein_[Ruminococcus_bromii_YE282]	Ruminococcus_bromii_YE282
Org1_Gene518	unique	2092	2595398465_IE32DRAFT_00546_hypothetical_protein_[Ruminococcus_bromii_YE282]	Ruminococcus_bromii_YE282
Org1_Gene519	unique	3831	2595398466_IE32DRAFT_00547_hypothetical_protein_[Ruminococcus_bromii_YE282]	Ruminococcus_bromii_YE282
Org1_Gene521	unique	3320	2595398468_IE32DRAFT_00549_acyl-CoA_thioester_hydrolase,_YbgC/YbaW_family_[Ruminococcus_bromii_YE282]	Ruminococcus_bromii_YE282
Org1_Gene523	unique	2297	2595398470_IE32DRAFT_00551_hypothetical_protein_[Ruminococcus_bromii_YE282]	Ruminococcus_bromii_YE282
Org1_Gene524	unique	4041	2595398474_IE32DRAFT_00555_Dockerin_type_I_repeat_[Ruminococcus_bromii_YE282]	Ruminococcus_bromii_YE282
Org1_Gene53	unique	565	2595397976_IE32DRAFT_00057_Peptidase_family_M23/G5_domain_[Ruminococcus_bromii_YE282]	Ruminococcus_bromii_YE282
Org1_Gene530	unique	3857	2595398481_IE32DRAFT_00562_Predicted_nucleic-acid-binding_protein_implicated_in_transcription_termination_[Ruminococcus_bromii_YE282]	Ruminococcus_bromii_YE282
Org1_Gene531	unique	3717	2595398482_IE32DRAFT_00563_Ribosomal_protein_HS6-type_(S12/L30/L7a)_[Ruminococcus_bromii_YE282]	Ruminococcus_bromii_YE282
Org1_Gene534	unique	1589	2595398485_IE32DRAFT_00566_Exopolyphosphatase-related_proteins_[Ruminococcus_bromii_YE282]	Ruminococcus_bromii_YE282
Org1_Gene535	unique	1682	2595398486_IE32DRAFT_00567_tRNA_pseudouridine(55)_synthase_[Ruminococcus_bromii_YE282]	Ruminococcus_bromii_YE282
Org1_Gene539	unique	2698	2595398490_IE32DRAFT_00571_Predicted_membrane_protein_[Ruminococcus_bromii_YE282]	Ruminococcus_bromii_YE282
Org1_Gene540	unique	1490	2595398491_IE32DRAFT_00572_birA,_biotin-[acetyl-CoA-carboxylase]_ligase_region_[Ruminococcus_bromii_YE282]	Ruminococcus_bromii_YE282
Org1_Gene541	unique	1646	2595398492_IE32DRAFT_00573_Permeases_of_the_drug/metabolite_transporter_(DMT)_superfamily_[Ruminococcus_bromii_YE282]	Ruminococcus_bromii_YE282
Org1_Gene543	unique	419	2595398494_IE32DRAFT_00575_Sodium:alanine_symporter_family_[Ruminococcus_bromii_YE282]	Ruminococcus_bromii_YE282
Org1_Gene544	unique	1909	2595398495_IE32DRAFT_00576_AraC-type_DNA-binding_domain-containing_proteins_[Ruminococcus_bromii_YE282]	Ruminococcus_bromii_YE282
Org1_Gene545	unique	1997	2595398496_IE32DRAFT_00577_AraC-type_DNA-binding_domain-containing_proteins_[Ruminococcus_bromii_YE282]	Ruminococcus_bromii_YE282
Org1_Gene546	unique	2929	2595398497_IE32DRAFT_00578_Rubrerythrin_[Ruminococcus_bromii_YE282]	Ruminococcus_bromii_YE282
Org1_Gene548	unique	1886	2595398499_IE32DRAFT_00580_Predicted_transcriptional_regulators_[Ruminococcus_bromii_YE282]	Ruminococcus_bromii_YE282
Org1_Gene549	unique	1735	2595398500_IE32DRAFT_00581_Leucine_rich_repeats_(6_copies)_[Ruminococcus_bromii_YE282]	Ruminococcus_bromii_YE282
Org1_Gene55	unique	1684	2595397978_IE32DRAFT_00059_Lyzozyme_M1_(1,4-beta-N-acetylmuramidase)_[Ruminococcus_bromii_YE282]	Ruminococcus_bromii_YE282
Org1_Gene550	unique	3980	2595398501_IE32DRAFT_00582_uncharacterized_protein_TIGR03905_[Ruminococcus_bromii_YE282]	Ruminococcus_bromii_YE282
Org1_Gene552	unique	2832	2595398503_IE32DRAFT_00584_TIGR00730_family_protein_[Ruminococcus_bromii_YE282]	Ruminococcus_bromii_YE282
Org1_Gene554	unique	334	2595398505_IE32DRAFT_00586_Prenyltransferase-like/Cadherin-like_beta_sandwich_domain_[Ruminococcus_bromii_YE282]	Ruminococcus_bromii_YE282
Org1_Gene555	unique	1593	2595398506_IE32DRAFT_00587_Domain_of_unknown_function_(DUF4430)_[Ruminococcus_bromii_YE282]	Ruminococcus_bromii_YE282
Org1_Gene556	unique	1733	2595398507_IE32DRAFT_00588_ABC-type_cobalt_transport_system,_permease_component_CbiQ_and_related_transporters_[Ruminococcus_bromii_YE2	Ruminococcus_bromii_YE282
Org1_Gene557	unique	117	2595398508_IE32DRAFT_00589_ATPase_components_of_various_ABC-type_transport_systems,_contain_duplicated_ATPase_[Ruminococcus_bromii_YE2	Ruminococcus_bromii_YE282
Org1_Gene558	unique	2848	2595398509_IE32DRAFT_00590_hypothetical_protein_[Ruminococcus_bromii_YE282]	Ruminococcus_bromii_YE282
Org1_Gene56	unique	3875	2595397979_IE32DRAFT_00060_hypothetical_protein_[Ruminococcus_bromii_YE282]	Ruminococcus_bromii_YE282
Org1_Gene563	unique	4316	2595398514_IE32DRAFT_00595_hypothetical_protein_[Ruminococcus_bromii_YE282]	Ruminococcus_bromii_YE282
Org1_Gene567	unique	3819	2595398518_IE32DRAFT_00599_hypothetical_protein_[Ruminococcus_bromii_YE282]	Ruminococcus_bromii_YE282
Org1_Gene568	unique	2582	2595398519_IE32DRAFT_00600_hypothetical_protein_[Ruminococcus_bromii_YE282]	Ruminococcus_bromii_YE282
Org1_Gene569	unique	3915	2595398520_IE32DRAFT_00601_hypothetical_protein_[Ruminococcus_bromii_YE282]	Ruminococcus_bromii_YE282
Org1_Gene57	unique	2583	2595397980_IE32DRAFT_00061_Domain_of_unknown_function_(DUF4097)_[Ruminococcus_bromii_YE282]	Ruminococcus_bromii_YE282
Org1_Gene570	unique	3790	2595398521_IE32DRAFT_00602_hypothetical_protein_[Ruminococcus_bromii_YE282]	Ruminococcus_bromii_YE282
Org1_Gene571	unique	1021	2595398522_IE32DRAFT_00603_DNA-methyltransferase_(dcm)_[Ruminococcus_bromii_YE282]	Ruminococcus_bromii_YE282
Org1_Gene572	unique	3690	2595398523_IE32DRAFT_00604_hypothetical_protein_[Ruminococcus_bromii_YE282]	Ruminococcus_bromii_YE282
Org1_Gene573	unique	1317	2595398524_IE32DRAFT_00605_Site-specific_DNA_methylase_[Ruminococcus_bromii_YE282]	Ruminococcus_bromii_YE282
Org1_Gene574	unique	4099	2595398525_IE32DRAFT_00606_hypothetical_protein_[Ruminococcus_bromii_YE282]	Ruminococcus_bromii_YE282
Org1_Gene575	unique	3769	2595398526_IE32DRAFT_00607_Regulators_of_stationary/sporulation_gene_expression_[Ruminococcus_bromii_YE282]	Ruminococcus_bromii_YE282
Org1_Gene576	unique	2435	2595398527_IE32DRAFT_00608_Domain_of_unknown_function_(DUF3846)/YodL-like_[Ruminococcus_bromii_YE282]	Ruminococcus_bromii_YE282
Org1_Gene577	unique	3271	2595398528_IE32DRAFT_00609_Uncharacterized_protein,_involved_in_the_regulation_of_septum_location_[Ruminococcus_bromii_YE282]	Ruminococcus_bromii_YE282
Org1_Gene578	unique	4014	2595398529_IE32DRAFT_00610_hypothetical_protein_[Ruminococcus_bromii_YE282]	Ruminococcus_bromii_YE282
Org1_Gene579	unique	2080	2595398530_IE32DRAFT_00611_hypothetical_protein_[Ruminococcus_bromii_YE282]	Ruminococcus_bromii_YE282
Org1_Gene58	unique	2907	2595397981_IE32DRAFT_00062_hypothetical_protein_[Ruminococcus_bromii_YE282]	Ruminococcus_bromii_YE282
Org1_Gene580	unique	1131	2595398531_IE32DRAFT_00612_Antirestriction_protein_(ArdA)_[Ruminococcus_bromii_YE282]	Ruminococcus_bromii_YE282
Org1_Gene581	unique	2945	2595398532_IE32DRAFT_00613_hypothetical_protein_[Ruminococcus_bromii_YE282]	Ruminococcus_bromii_YE282
Org1_Gene582	unique	3689	2595398533_IE32DRAFT_00614_hypothetical_protein_[Ruminococcus_bromii_YE282]	Ruminococcus_bromii_YE282
Org1_Gene583	unique	1897	2595398534_IE32DRAFT_00615_Domain_of_unknown_function_(DUF4314)_[Ruminococcus_bromii_YE282]	Ruminococcus_bromii_YE282
Org1_Gene584	unique	1449	2595398535_IE32DRAFT_00616_hypothetical_protein_[Ruminococcus_bromii_YE282]	Ruminococcus_bromii_YE282
Org1_Gene585	unique	535	2595398536_IE32DRAFT_00617_Antirestriction_protein_(ArdA)_[Ruminococcus_bromii_YE282]	Ruminococcus_bromii_YE282
Org1_Gene586	unique	2694	2595398537_IE32DRAFT_00618_hypothetical_protein_[Ruminococcus_bromii_YE282]	Ruminococcus_bromii_YE282
Org1_Gene587	unique	3635	2595398538_IE32DRAFT_00619_hypothetical_protein_[Ruminococcus_bromii_YE282]	Ruminococcus_bromii_YE282
Org1_Gene588	unique	4360	2595398539_IE32DRAFT_00620_hypothetical_protein_[Ruminococcus_bromii_YE282]	Ruminococcus_bromii_YE282
Org1_Gene589	unique	42	2595398540_IE32DRAFT_00621_Cna_protein_B-type_domain_[Ruminococcus_bromii_YE282]	Ruminococcus_bromii_YE282
Org1_Gene59	unique	1783	2595397982_IE32DRAFT_00063_yjeF_C-terminal_region,_hydroxyethylthiazole_kinase-related_[Ruminococcus_bromii_YE282]	Ruminococcus_bromii_YE282
Org1_Gene590	unique	3602	2595398541_IE32DRAFT_00622_hypothetical_protein_[Ruminococcus_bromii_YE282]	Ruminococcus_bromii_YE282
Org1_Gene592	unique	1667	2595398543_IE32DRAFT_00624_hypothetical_protein_[Ruminococcus_bromii_YE282]	Ruminococcus_bromii_YE282
Org1_Gene593	unique	3511	2595398544_IE32DRAFT_00625_hypothetical_protein_[Ruminococcus_bromii_YE282]	Ruminococcus_bromii_YE282
Org1_Gene594	unique	3943	2595398545_IE32DRAFT_00626_hypothetical_protein_[Ruminococcus_bromii_YE282]	Ruminococcus_bromii_YE282
Org1_Gene595	unique	2590	2595398546_IE32DRAFT_00627_hypothetical_protein_[Ruminococcus_bromii_YE282]	Ruminococcus_bromii_YE282
Org1_Gene596	unique	1929	2595398547_IE32DRAFT_00628_Uncharacterized_protein_conserved_in_bacteria_[Ruminococcus_bromii_YE282]	Ruminococcus_bromii_YE282
Org1_Gene598	unique	2986	2595398549_IE32DRAFT_00630_hypothetical_protein_[Ruminococcus_bromii_YE282]	Ruminococcus_bromii_YE282
Org1_Gene599	unique	3218	2595398550_IE32DRAFT_00631_Acetyltransferase_(GNAT)_family_[Ruminococcus_bromii_YE282]	Ruminococcus_bromii_YE282
Org1_Gene60	unique	3539	2595397983_IE32DRAFT_00064_Predicted_transcriptional_regulators_[Ruminococcus_bromii_YE282]	Ruminococcus_bromii_YE282
Org1_Gene600	unique	1211	2595398551_IE32DRAFT_00632_ADP-ribosylglycohydrolase_[Ruminococcus_bromii_YE282]	Ruminococcus_bromii_YE282
Org1_Gene601	unique	2511	2595398552_IE32DRAFT_00633_Predicted_membrane_protein_[Ruminococcus_bromii_YE282]	Ruminococcus_bromii_YE282
Org1_Gene602	unique	929	2595398553_IE32DRAFT_00634_CHAP_domain_[Ruminococcus_bromii_YE282]	Ruminococcus_bromii_YE282
Org1_Gene603	unique	1328	2595398554_IE32DRAFT_00635_Pentapeptide_repeats_(9_copies)_[Ruminococcus_bromii_YE282]	Ruminococcus_bromii_YE282
Org1_Gene604	unique	1327	2595398555_IE32DRAFT_00636_Pentapeptide_repeats_(9_copies)_[Ruminococcus_bromii_YE282]	Ruminococcus_bromii_YE282
Org1_Gene605	unique	4017	2595398556_IE32DRAFT_00637_hypothetical_protein_[Ruminococcus_bromii_YE282]	Ruminococcus_bromii_YE282
Org1_Gene606	unique	2505	2595398557_IE32DRAFT_00638_hypothetical_protein_[Ruminococcus_bromii_YE282]	Ruminococcus_bromii_YE282
Org1_Gene607	unique	3250	2595398558_IE32DRAFT_00639_hypothetical_protein_[Ruminococcus_bromii_YE282]	Ruminococcus_bromii_YE282
Org1_Gene608	unique	3415	2595398559_IE32DRAFT_00640_hypothetical_protein_[Ruminococcus_bromii_YE282]	Ruminococcus_bromii_YE282
Org1_Gene609	unique	216	2595398560_IE32DRAFT_00641_FOG:_TPR_repeat,_SEL1_subfamily_[Ruminococcus_bromii_YE282]	Ruminococcus_bromii_YE282
Org1_Gene61	unique	1702	2595397984_IE32DRAFT_00065_ABC-type_multidrug_transport_system,_ATPase_component_[Ruminococcus_bromii_YE282]	Ruminococcus_bromii_YE282
Org1_Gene610	unique	1666	2595398561_IE32DRAFT_00642_Protein_of_unknown_function_(DUF3991)/Toprim-like_[Ruminococcus_bromii_YE282]	Ruminococcus_bromii_YE282
Org1_Gene611	unique	261	2595398562_IE32DRAFT_00643_Type_IV_secretory_system_Conjugative_DNA_transfer_[Ruminococcus_bromii_YE282]	Ruminococcus_bromii_YE282
Org1_Gene612	unique	3977	2595398563_IE32DRAFT_00644_Protein_of_unknown_function_(DUF3782)_[Ruminococcus_bromii_YE282]	Ruminococcus_bromii_YE282
Org1_Gene613	unique	4369	2595398564_IE32DRAFT_00645_Arc-like_DNA_binding_domain_[Ruminococcus_bromii_YE282]	Ruminococcus_bromii_YE282
Org1_Gene614	unique	3800	2595398565_IE32DRAFT_00646_Protein_of_unknown_function_(DUF1273)_[Ruminococcus_bromii_YE282]	Ruminococcus_bromii_YE282
Org1_Gene615	unique	1800	2595398566_IE32DRAFT_00647_Site-specific_recombinase_XerD_[Ruminococcus_bromii_YE282]	Ruminococcus_bromii_YE282
Org1_Gene616	unique	136	2595398567_IE32DRAFT_00648_ABC-type_transport_system,_involved_in_lipoprotein_release,_permease_component_[Ruminococcus_bromii_YE282]	Ruminococcus_bromii_YE282
Org1_Gene617	unique	2458	2595398568_IE32DRAFT_00649_ABC-type_antimicrobial_peptide_transport_system,_ATPase_component_[Ruminococcus_bromii_YE282]	Ruminococcus_bromii_YE282
Org1_Gene618	unique	585	2595398569_IE32DRAFT_00650_Phytoene_dehydrogenase_and_related_proteins_[Ruminococcus_bromii_YE282]	Ruminococcus_bromii_YE282
Org1_Gene619	unique	2168	2595398570_IE32DRAFT_00651_Zn-dependent_hydrolases,_including_glyoxylases_[Ruminococcus_bromii_YE282]	Ruminococcus_bromii_YE282
Org1_Gene62	unique	260	2595397985_IE32DRAFT_00066_ABC-2_family_transporter_protein_[Ruminococcus_bromii_YE282]	Ruminococcus_bromii_YE282
Org1_Gene620	unique	3988	2595398571_IE32DRAFT_00652_Acyl_carrier_protein_[Ruminococcus_bromii_YE282]	Ruminococcus_bromii_YE282
Org1_Gene621	unique	1454	2595398572_IE32DRAFT_00653_Collagenase_and_related_proteases_[Ruminococcus_bromii_YE282]	Ruminococcus_bromii_YE282
Org1_Gene622	unique	1727	2595398573_IE32DRAFT_00654_hypothetical_protein_[Ruminococcus_bromii_YE282]	Ruminococcus_bromii_YE282
Org1_Gene623	unique	187	2595398574_IE32DRAFT_00655_3-oxoacyl-(acyl-carrier-protein)_synthase_[Ruminococcus_bromii_YE282]	Ruminococcus_bromii_YE282
Org1_Gene624	unique	1759	2595398575_IE32DRAFT_00656_EDD_domain_protein,_DegV_family_[Ruminococcus_bromii_YE282]	Ruminococcus_bromii_YE282
Org1_Gene625	unique	2245	2595398576_IE32DRAFT_00657_3-oxoacyl-[acyl-carrier-protein]_reductase_(EC_1.1.1.100)_[Ruminococcus_bromii_YE282]	Ruminococcus_bromii_YE282
Org1_Gene626	unique	3245	2595398577_IE32DRAFT_00658_3-hydroxyacyl-[acyl-carrier-protein]_dehydratase_(EC_4.2.1.-)_[Ruminococcus_bromii_YE282]	Ruminococcus_bromii_YE282
Org1_Gene627	unique	1888	2595398578_IE32DRAFT_00659_ABC-type_multidrug_transport_system,_ATPase_component_[Ruminococcus_bromii_YE282]	Ruminococcus_bromii_YE282
Org1_Gene628	unique	2374	2595398579_IE32DRAFT_00660_ABC-2_family_transporter_protein_[Ruminococcus_bromii_YE282]	Ruminococcus_bromii_YE282
Org1_Gene629	unique	1213	2595398580_IE32DRAFT_00661_3-deoxy-D-arabinoheptulosonate-7-phosphate_synthase_(EC_2.5.1.54)_[Ruminococcus_bromii_YE282]	Ruminococcus_bromii_YE282
Org1_Gene63	unique	1275	2595397986_IE32DRAFT_00067_6-phosphofructokinase_[Ruminococcus_bromii_YE282]	Ruminococcus_bromii_YE282
Org1_Gene630	unique	1468	2595398581_IE32DRAFT_00662_Predicted_transcriptional_regulator_[Ruminococcus_bromii_YE282]	Ruminococcus_bromii_YE282
Org1_Gene631	unique	2644	2595398582_IE32DRAFT_00663_SOS-response_transcriptional_repressors_(RecA-mediated_autopeptidases)_[Ruminococcus_bromii_YE282]	Ruminococcus_bromii_YE282
Org1_Gene632	unique	4259	2595398583_IE32DRAFT_00664_hypothetical_protein_[Ruminococcus_bromii_YE282]	Ruminococcus_bromii_YE282
Org1_Gene633	unique	4173	2595398584_IE32DRAFT_00665_hypothetical_protein_[Ruminococcus_bromii_YE282]	Ruminococcus_bromii_YE282
Org1_Gene634	unique	4067	2595398585_IE32DRAFT_00666_hypothetical_protein_[Ruminococcus_bromii_YE282]	Ruminococcus_bromii_YE282
Org1_Gene635	unique	2962	2595398586_IE32DRAFT_00667_hypothetical_protein_[Ruminococcus_bromii_YE282]	Ruminococcus_bromii_YE282
Org1_Gene636	unique	1350	2595398587_IE32DRAFT_00668_Uncharacterized_conserved_protein_[Ruminococcus_bromii_YE282]	Ruminococcus_bromii_YE282
Org1_Gene637	unique	3737	2595398588_IE32DRAFT_00669_Growth_inhibitor_[Ruminococcus_bromii_YE282]	Ruminococcus_bromii_YE282
Org1_Gene638	unique	4390	2595398589_IE32DRAFT_00670_Protein_of_unknown_function_(DUF3789)_[Ruminococcus_bromii_YE282]	Ruminococcus_bromii_YE282
Org1_Gene639	unique	4299	2595398590_IE32DRAFT_00671_hypothetical_protein_[Ruminococcus_bromii_YE282]	Ruminococcus_bromii_YE282
Org1_Gene64	unique	228	2595397987_IE32DRAFT_00068_polyphosphate_kinase_1_[Ruminococcus_bromii_YE282]	Ruminococcus_bromii_YE282

Org1_Gene640	unique	513	2595398591_IE32DRAFT_00672_Site-specific_recombinases,_DNA_invertase_Pin_homologs_[Ruminococcus_bromii_YE282]	Ruminococcus_bromii_YE282
Org1_Gene641	unique	2139	2595398592_IE32DRAFT_00673_Protein_of_unknown_function_(DUF2971)_[Ruminococcus_bromii_YE282]	Ruminococcus_bromii_YE282
Org1_Gene643	unique	3465	2595398594_IE32DRAFT_00675_hypothetical_protein_[Ruminococcus_bromii_YE282]	Ruminococcus_bromii_YE282
Org1_Gene644	unique	85	2595398595_IE32DRAFT_00676_Type_I_restriction-modification_system_methyltransferase_subunit_[Ruminococcus_bromii_YE282]	Ruminococcus_bromii_YE282
Org1_Gene645	unique	2495	2595398596_IE32DRAFT_00677_hypothetical_protein_[Ruminococcus_bromii_YE282]	Ruminococcus_bromii_YE282
Org1_Gene646	unique	961	2595398598_IE32DRAFT_00679_Phage_integrase_family_[Ruminococcus_bromii_YE282]	Ruminococcus_bromii_YE282
Org1_Gene647	unique	1633	2595398599_IE32DRAFT_00680_Double_zinc_ribbon_[Ruminococcus_bromii_YE282]	Ruminococcus_bromii_YE282
Org1_Gene648	unique	2372	2595398600_IE32DRAFT_00681_two_component_transcriptional_regulator,_LytTR_family_[Ruminococcus_bromii_YE282]	Ruminococcus_bromii_YE282
Org1_Gene649	unique	3600	2595398601_IE32DRAFT_00682_Predicted_transcriptional_regulators_[Ruminococcus_bromii_YE282]	Ruminococcus_bromii_YE282
Org1_Gene650	unique	4376	2595398602_IE32DRAFT_00683_hypothetical_protein_[Ruminococcus_bromii_YE282]	Ruminococcus_bromii_YE282
Org1_Gene651	unique	3914	2595398603_IE32DRAFT_00684_hypothetical_protein_[Ruminococcus_bromii_YE282]	Ruminococcus_bromii_YE282
Org1_Gene652	unique	4314	2595398604_IE32DRAFT_00685_hypothetical_protein_[Ruminococcus_bromii_YE282]	Ruminococcus_bromii_YE282
Org1_Gene653	unique	4403	2595398605_IE32DRAFT_00686_hypothetical_protein_[Ruminococcus_bromii_YE282]	Ruminococcus_bromii_YE282
Org1_Gene654	unique	4246	2595398606_IE32DRAFT_00687_hypothetical_protein_[Ruminococcus_bromii_YE282]	Ruminococcus_bromii_YE282
Org1_Gene655	unique	4174	2595398607_IE32DRAFT_00688_hypothetical_protein_[Ruminococcus_bromii_YE282]	Ruminococcus_bromii_YE282
Org1_Gene656	unique	2853	2595398608_IE32DRAFT_00689_hypothetical_protein_[Ruminococcus_bromii_YE282]	Ruminococcus_bromii_YE282
Org1_Gene657	unique	3580	2595398609_IE32DRAFT_00690_VRR-NUC_domain_[Ruminococcus_bromii_YE282]	Ruminococcus_bromii_YE282
Org1_Gene658	unique	4102	2595398610_IE32DRAFT_00691_hypothetical_protein_[Ruminococcus_bromii_YE282]	Ruminococcus_bromii_YE282
Org1_Gene659	unique	2421	2595398611_IE32DRAFT_00692_hypothetical_protein_[Ruminococcus_bromii_YE282]	Ruminococcus_bromii_YE282
Org1_Gene66	unique	4108	2595397989_IE32DRAFT_00070_hypothetical_protein_[Ruminococcus_bromii_YE282]	Ruminococcus_bromii_YE282
Org1_Gene660	unique	2266	2595398612_IE32DRAFT_00693_hypothetical_protein_[Ruminococcus_bromii_YE282]	Ruminococcus_bromii_YE282
Org1_Gene661	unique	3551	2595398613_IE32DRAFT_00694_VRR-NUC_domain_[Ruminococcus_bromii_YE282]	Ruminococcus_bromii_YE282
Org1_Gene662	unique	3649	2595398614_IE32DRAFT_00695_hypothetical_protein_[Ruminococcus_bromii_YE282]	Ruminococcus_bromii_YE282
Org1_Gene663	unique	3820	2595398615_IE32DRAFT_00696_HNH_endonuclease_[Ruminococcus_bromii_YE282]	Ruminococcus_bromii_YE282
Org1_Gene664	unique	2860	2595398616_IE32DRAFT_00697_hypothetical_protein_[Ruminococcus_bromii_YE282]	Ruminococcus_bromii_YE282
Org1_Gene665	unique	3814	2595398617_IE32DRAFT_00698_hypothetical_protein_[Ruminococcus_bromii_YE282]	Ruminococcus_bromii_YE282
Org1_Gene666	unique	472	2595398618_IE32DRAFT_00699_Phage_terminase-like_protein,_large_subunit_[Ruminococcus_bromii_YE282]	Ruminococcus_bromii_YE282
Org1_Gene667	unique	1115	2595398619_IE32DRAFT_00700_phage_portal_protein,_HK97_family_[Ruminococcus_bromii_YE282]	Ruminococcus_bromii_YE282
Org1_Gene668	unique	2052	2595398620_IE32DRAFT_00701_Protease_subunit_of_ATP-dependent_Clp_proteases_[Ruminococcus_bromii_YE282]	Ruminococcus_bromii_YE282
Org1_Gene669	unique	975	2595398621_IE32DRAFT_00702_phage_major_capsid_protein,_HK97_family_[Ruminococcus_bromii_YE282]	Ruminococcus_bromii_YE282
Org1_Gene67	unique	1788	2595397990_IE32DRAFT_00071_uncharacterized_protein,_YkwD_family_[Ruminococcus_bromii_YE282]	Ruminococcus_bromii_YE282
Org1_Gene670	unique	3778	2595398622_IE32DRAFT_00703_hypothetical_protein_[Ruminococcus_bromii_YE282]	Ruminococcus_bromii_YE282
Org1_Gene671	unique	3638	2595398623_IE32DRAFT_00704_hypothetical_protein_[Ruminococcus_bromii_YE282]	Ruminococcus_bromii_YE282
Org1_Gene672	unique	3699	2595398624_IE32DRAFT_00705_hypothetical_protein_[Ruminococcus_bromii_YE282]	Ruminococcus_bromii_YE282
Org1_Gene673	unique	3456	2595398625_IE32DRAFT_00706_Bacteriophage_HK97-gp10,_putative_tail-component_[Ruminococcus_bromii_YE282]	Ruminococcus_bromii_YE282
Org1_Gene674	unique	2646	2595398626_IE32DRAFT_00707_phage_major_tail_protein,_phi13_family_[Ruminococcus_bromii_YE282]	Ruminococcus_bromii_YE282
Org1_Gene675	unique	3549	2595398627_IE32DRAFT_00708_hypothetical_protein_[Ruminococcus_bromii_YE282]	Ruminococcus_bromii_YE282
Org1_Gene676	unique	4320	2595398628_IE32DRAFT_00709_hypothetical_protein_[Ruminococcus_bromii_YE282]	Ruminococcus_bromii_YE282
Org1_Gene677	unique	28	2595398629_IE32DRAFT_00710_Phage-related_minor_tail_protein_[Ruminococcus_bromii_YE282]	Ruminococcus_bromii_YE282
Org1_Gene678	unique	2138	2595398630_IE32DRAFT_00711_Phage-related_protein_[Ruminococcus_bromii_YE282]	Ruminococcus_bromii_YE282
Org1_Gene679	unique	1058	2595398631_IE32DRAFT_00712_phage_minor_structural_protein,_N-terminal_region_[Ruminococcus_bromii_YE282]	Ruminococcus_bromii_YE282
Org1_Gene68	unique	532	2595397991_IE32DRAFT_00072_hypothetical_protein_[Ruminococcus_bromii_YE282]	Ruminococcus_bromii_YE282
Org1_Gene680	unique	360	2595398632_IE32DRAFT_00713_Pectate_lyase_superfamily_protein_[Ruminococcus_bromii_YE282]	Ruminococcus_bromii_YE282
Org1_Gene683	unique	3095	2595398635_IE32DRAFT_00717_Acetyltransferases_[Ruminococcus_bromii_YE282]	Ruminococcus_bromii_YE282
Org1_Gene684	unique	3728	2595398636_IE32DRAFT_00718_Cupin_domain_[Ruminococcus_bromii_YE282]	Ruminococcus_bromii_YE282
Org1_Gene685	unique	3853	2595398637_IE32DRAFT_00719_RNA_polymerase_sigma_factor,_sigma-70_family_[Ruminococcus_bromii_YE282]	Ruminococcus_bromii_YE282
Org1_Gene686	unique	4171	2595398638_IE32DRAFT_00720_hypothetical_protein_[Ruminococcus_bromii_YE282]	Ruminococcus_bromii_YE282
Org1_Gene687	unique	684	2595398639_IE32DRAFT_00721_putative_efflux_protein,_MATE_family_[Ruminococcus_bromii_YE282]	Ruminococcus_bromii_YE282
Org1_Gene689	unique	558	2595398641_IE32DRAFT_00723_Domain_of_unknown_function_(DUF3794)_[Ruminococcus_bromii_YE282]	Ruminococcus_bromii_YE282
Org1_Gene69	unique	2834	2595397992_IE32DRAFT_00073_Uncharacterized_conserved_protein_[Ruminococcus_bromii_YE282]	Ruminococcus_bromii_YE282
Org1_Gene691	unique	3291	2595398643_IE32DRAFT_00725_Oxaloacetate_decarboxylase,_gamma_chain_[Ruminococcus_bromii_YE282]	Ruminococcus_bromii_YE282
Org1_Gene693	unique	2048	2595398645_IE32DRAFT_00727_Capsular_polysaccharide_biosynthesis_protein_[Ruminococcus_bromii_YE282]	Ruminococcus_bromii_YE282
Org1_Gene694	unique	2037	2595398646_IE32DRAFT_00728_capsular_exopolysaccharide_family_[Ruminococcus_bromii_YE282]	Ruminococcus_bromii_YE282
Org1_Gene695	unique	4138	2595398647_IE32DRAFT_00729_hypothetical_protein_[Ruminococcus_bromii_YE282]	Ruminococcus_bromii_YE282
Org1_Gene699	unique	3528	2595398651_IE32DRAFT_00733_Domain_of_unknown_function_(DUF4363)_[Ruminococcus_bromii_YE282]	Ruminococcus_bromii_YE282
Org1_Gene7	unique	3088	2595397928_IE32DRAFT_00009_PAP2_superfamily_[Ruminococcus_bromii_YE282]	Ruminococcus_bromii_YE282
Org1_Gene70	unique	2979	2595397993_IE32DRAFT_00074_hypothetical_protein_[Ruminococcus_bromii_YE282]	Ruminococcus_bromii_YE282
Org1_Gene700	unique	2291	2595398652_IE32DRAFT_00734_Predicted_membrane_protein_[Ruminococcus_bromii_YE282]	Ruminococcus_bromii_YE282
Org1_Gene702	unique	1307	2595398654_IE32DRAFT_00736_hypothetical_protein_[Ruminococcus_bromii_YE282]	Ruminococcus_bromii_YE282
Org1_Gene703	unique	2733	2595398655_IE32DRAFT_00737_hypothetical_protein_[Ruminococcus_bromii_YE282]	Ruminococcus_bromii_YE282
Org1_Gene708	unique	4137	2595398660_IE32DRAFT_00742_Exodeoxyribonuclease_VII_small_subunit_(EC_3.1.11.6)_[Ruminococcus_bromii_YE282]	Ruminococcus_bromii_YE282
Org1_Gene712	unique	1852	2595398664_IE32DRAFT_00746_Predicted_sugar_kinase_[Ruminococcus_bromii_YE282]	Ruminococcus_bromii_YE282
Org1_Gene713	unique	3280	2595398665_IE32DRAFT_00747_transcriptional_regulator,_ArgR_family_[Ruminococcus_bromii_YE282]	Ruminococcus_bromii_YE282
Org1_Gene715	unique	266	2595398667_IE32DRAFT_00749_exonuclease_RecJ_(EC_3.1.-.-)_[Ruminococcus_bromii_YE282]	Ruminococcus_bromii_YE282
Org1_Gene72	unique	2253	2595397995_IE32DRAFT_00076_PEP-utilising_enzyme,_TIM_barrel_domain_[Ruminococcus_bromii_YE282]	Ruminococcus_bromii_YE282
Org1_Gene720	unique	3204	2595398672_IE32DRAFT_00754_Uncharacterised_protein_family_UPF0547_[Ruminococcus_bromii_YE282]	Ruminococcus_bromii_YE282
Org1_Gene721	unique	367	2595398673_IE32DRAFT_00755_Membrane_protein_involved_in_the_export_of_O-antigen_and_teichoic_acid_[Ruminococcus_bromii_YE282]	Ruminococcus_bromii_YE282
Org1_Gene728	unique	2748	2595398680_IE32DRAFT_00762_HD_domain_[Ruminococcus_bromii_YE282]	Ruminococcus_bromii_YE282
Org1_Gene73	unique	1504	2595397996_IE32DRAFT_00077_PEP-utilising_enzyme,_TIM_barrel_domain/PEP-utilising_enzyme,_mobile_domain/PEP-utilising_enzyme,_N-terminal_[Ru	Ruminococcus_bromii_YE282
Org1_Gene730	unique	3020	2595398682_IE32DRAFT_00764_NADH:ubiquinone_oxidoreductase_24_kD_subunit_[Ruminococcus_bromii_YE282]	Ruminococcus_bromii_YE282
Org1_Gene731	unique	325	2595398683_IE32DRAFT_00765_NADH:ubiquinone_oxidoreductase,_NADH-binding_(51_kD)_subunit_[Ruminococcus_bromii_YE282]	Ruminococcus_bromii_YE282
Org1_Gene732	unique	409	2595398684_IE32DRAFT_00766_[FeFe]_hydrogenase,_group_A_[Ruminococcus_bromii_YE282]	Ruminococcus_bromii_YE282
Org1_Gene733	unique	661	2595398685_IE32DRAFT_00767_[FeFe]_hydrogenase,_group_B1/B3_[Ruminococcus_bromii_YE282]	Ruminococcus_bromii_YE282
Org1_Gene734	unique	1089	2595398686_IE32DRAFT_00768_Stage_II_sporulation_protein_E_(SpoIIE)_[Ruminococcus_bromii_YE282]	Ruminococcus_bromii_YE282
Org1_Gene735	unique	448	2595398687_IE32DRAFT_00769_Iron_only_hydrogenase_large_subunit,_C-terminal_domain_[Ruminococcus_bromii_YE282]	Ruminococcus_bromii_YE282
Org1_Gene736	unique	3982	2595398688_IE32DRAFT_00770_Thioredoxin-like_[2Fe-2S]_ferredoxin_[Ruminococcus_bromii_YE282]	Ruminococcus_bromii_YE282
Org1_Gene737	unique	4039	2595398689_IE32DRAFT_00771_Thioredoxin-like_[2Fe-2S]_ferredoxin_[Ruminococcus_bromii_YE282]	Ruminococcus_bromii_YE282
Org1_Gene738	unique	2194	2595398690_IE32DRAFT_00772_Phosphotransferase_enzyme_family_[Ruminococcus_bromii_YE282]	Ruminococcus_bromii_YE282
Org1_Gene740	unique	1364	2595398693_IE32DRAFT_00775_Cellulase_M_and_related_proteins_[Ruminococcus_bromii_YE282]	Ruminococcus_bromii_YE282
Org1_Gene741	unique	1324	2595398694_IE32DRAFT_00776_Cellulase_M_and_related_proteins_[Ruminococcus_bromii_YE282]	Ruminococcus_bromii_YE282
Org1_Gene742	unique	2114	2595398695_IE32DRAFT_00777_Acetyltransferase_(GNAT)_domain_[Ruminococcus_bromii_YE282]	Ruminococcus_bromii_YE282
Org1_Gene744	unique	3473	2595398697_IE32DRAFT_00779_CBS_domain_[Ruminococcus_bromii_YE282]	Ruminococcus_bromii_YE282
Org1_Gene745	unique	3293	2595398698_IE32DRAFT_00780_hypothetical_protein_[Ruminococcus_bromii_YE282]	Ruminococcus_bromii_YE282
Org1_Gene746	unique	1142	2595398699_IE32DRAFT_00781_conserved_hypothetical_protein_[Ruminococcus_bromii_YE282]	Ruminococcus_bromii_YE282
Org1_Gene747	unique	1133	2595398700_IE32DRAFT_00782_Protein_of_unknown_function_(DUF2974)_[Ruminococcus_bromii_YE282]	Ruminococcus_bromii_YE282
Org1_Gene75	unique	2249	2595397998_IE32DRAFT_00079_PTS_system,_fructose_subfamily,_IIC_component_[Ruminococcus_bromii_YE282]	Ruminococcus_bromii_YE282
Org1_Gene751	unique	1391	2595398704_IE32DRAFT_00786_Predicted_metal-dependent_membrane_protease_[Ruminococcus_bromii_YE282]	Ruminococcus_bromii_YE282
Org1_Gene752	unique	239	2595398705_IE32DRAFT_00787_hypothetical_protein_[Ruminococcus_bromii_YE282]	Ruminococcus_bromii_YE282
Org1_Gene754	unique	3991	2595398707_IE32DRAFT_00789_hypothetical_protein_[Ruminococcus_bromii_YE282]	Ruminococcus_bromii_YE282
Org1_Gene755	unique	1946	2595398708_IE32DRAFT_00790_ParB/RepB/Spo0J_family_partition_protein_[Ruminococcus_bromii_YE282]	Ruminococcus_bromii_YE282
Org1_Gene76	unique	1197	2595397999_IE32DRAFT_00080_PTS_system,_fructose-specific,_IIB_component_[Ruminococcus_bromii_YE282]	Ruminococcus_bromii_YE282
Org1_Gene763	unique	4182	2595398716_IE32DRAFT_00798_Predicted_transcriptional_regulator_[Ruminococcus_bromii_YE282]	Ruminococcus_bromii_YE282
Org1_Gene764	unique	103	2595398717_IE32DRAFT_00799_type_I_restriction_system_adenine_methylase_(hsdM)_[Ruminococcus_bromii_YE282]	Ruminococcus_bromii_YE282
Org1_Gene765	unique	943	2595398718_IE32DRAFT_00800_Restriction_endonuclease_S_subunits_[Ruminococcus_bromii_YE282]	Ruminococcus_bromii_YE282
Org1_Gene766	unique	79	2595398719_IE32DRAFT_00801_type_I_site-specific_deoxyribonuclease,_HsdR_family_[Ruminococcus_bromii_YE282]	Ruminococcus_bromii_YE282
Org1_Gene767	unique	596	2595398720_IE32DRAFT_00802_hypothetical_protein_[Ruminococcus_bromii_YE282]	Ruminococcus_bromii_YE282
Org1_Gene768	unique	2265	2595398721_IE32DRAFT_00803_hypothetical_protein_[Ruminococcus_bromii_YE282]	Ruminococcus_bromii_YE282
Org1_Gene769	unique	1051	2595398722_IE32DRAFT_00804_Predicted_Zn_peptidase_[Ruminococcus_bromii_YE282]	Ruminococcus_bromii_YE282
Org1_Gene77	unique	1669	2595398000_IE32DRAFT_00081_1-phosphofructokinase_[Ruminococcus_bromii_YE282]	Ruminococcus_bromii_YE282
Org1_Gene770	unique	3521	2595398723_IE32DRAFT_00805_hypothetical_protein_[Ruminococcus_bromii_YE282]	Ruminococcus_bromii_YE282
Org1_Gene776	unique	1781	2595398729_IE32DRAFT_00811_D-isomer_specific_2-hydroxyacid_dehydrogenase,_NAD_binding_domain_[Ruminococcus_bromii_YE282]	Ruminococcus_bromii_YE282
Org1_Gene778	unique	3015	2595398731_IE32DRAFT_00813_hypothetical_protein_[Ruminococcus_bromii_YE282]	Ruminococcus_bromii_YE282
Org1_Gene78	unique	1119	2595398001_IE32DRAFT_00082_Collagen_triple_helix_repeat_(20_copies)_[Ruminococcus_bromii_YE282]	Ruminococcus_bromii_YE282
Org1_Gene782	unique	2996	2595398735_IE32DRAFT_00817_Tic20-like_protein_[Ruminococcus_bromii_YE282]	Ruminococcus_bromii_YE282
Org1_Gene79	unique	2609	2595398002_IE32DRAFT_00083_transcriptional_regulator,_TetR_family_[Ruminococcus_bromii_YE282]	Ruminococcus_bromii_YE282
Org1_Gene790	unique	2325	2595398743_IE32DRAFT_00825_acyl-phosphate_glycerol_3-phosphate_acyltransferase_[Ruminococcus_bromii_YE282]	Ruminococcus_bromii_YE282
Org1_Gene793	unique	220	2595398746_IE32DRAFT_00828_ComEC/Rec2-related_protein_[Ruminococcus_bromii_YE282]	Ruminococcus_bromii_YE282
Org1_Gene794	unique	1298	2595398747_IE32DRAFT_00829_DNA_polymerase_III,_delta_subunit_(EC_2.7.7.7)_[Ruminococcus_bromii_YE282]	Ruminococcus_bromii_YE282
Org1_Gene796	unique	3484	2595398749_IE32DRAFT_00831_Protein_of_unknown_function_(DUF1113)_[Ruminococcus_bromii_YE282]	Ruminococcus_bromii_YE282
Org1_Gene80	unique	4256	2595398003_IE32DRAFT_00084_Cysteine-rich_KTR_[Ruminococcus_bromii_YE282]	Ruminococcus_bromii_YE282
Org1_Gene800	unique	84	2595398753_IE32DRAFT_00835_DNA_translocase_FtsK_[Ruminococcus_bromii_YE282]	Ruminococcus_bromii_YE282
Org1_Gene802	unique	1611	2595398755_IE32DRAFT_00837_Predicted_hydrolase_(metallo-beta-lactamase_superfamily)_[Ruminococcus_bromii_YE282]	Ruminococcus_bromii_YE282
Org1_Gene805	unique	3181	2595398758_IE32DRAFT_00840_PAP2_superfamily_[Ruminococcus_bromii_YE282]	Ruminococcus_bromii_YE282
Org1_Gene807	unique	1294	2595398760_IE32DRAFT_00842_hypothetical_protein_[Ruminococcus_bromii_YE282]	Ruminococcus_bromii_YE282
Org1_Gene808	unique	3926	2595398761_IE32DRAFT_00843_YabP_family_[Ruminococcus_bromii_YE282]	Ruminococcus_bromii_YE282
Org1_Gene809	unique	1079	2595398762_IE32DRAFT_00844_Putative_stage_IV_sporulation_protein_YqfD_[Ruminococcus_bromii_YE282]	Ruminococcus_bromii_YE282
Org1_Gene81	unique	3039	2595398004_IE32DRAFT_00085_Flavodoxin_domain_[Ruminococcus_bromii_YE282]	Ruminococcus_bromii_YE282
Org1_Gene813	unique	772	2595398766_IE32DRAFT_00848_D-alanyl-D-alanine_carboxypeptidase_[Ruminococcus_bromii_YE282]	Ruminococcus_bromii_YE282
Org1_Gene815	unique	992	2595398768_IE32DRAFT_00850_ABC-type_Fe3+-hydroxamate_transport_system,_periplasmic_component_[Ruminococcus_bromii_YE282]	Ruminococcus_bromii_YE282
Org1_Gene816	unique	3502	2595398769_IE32DRAFT_00851_Predicted_transcriptional_regulators_[Ruminococcus_bromii_YE282]	Ruminococcus_bromii_YE282
Org1_Gene819	unique	236	2595398772_IE32DRAFT_00854_hypothetical_protein_[Ruminococcus_bromii_YE282]	Ruminococcus_bromii_YE282
Org1_Gene82	unique	4161	2595398005_IE32DRAFT_00086_hypothetical_protein_[Ruminococcus_bromii_YE282]	Ruminococcus_bromii_YE282

Org1_Gene820	unique	3596	2595398773_IE32DRAFT_00855_hypothetical_protein_[Ruminococcus_bromii_YE282]	Ruminococcus_bromii_YE282
Org1_Gene821	unique	3336	2595398774_IE32DRAFT_00856_hypothetical_protein_[Ruminococcus_bromii_YE282]	Ruminococcus_bromii_YE282
Org1_Gene822	unique	2894	2595398775_IE32DRAFT_00857_hypothetical_protein_[Ruminococcus_bromii_YE282]	Ruminococcus_bromii_YE282
Org1_Gene823	unique	1610	2595398776_IE32DRAFT_00858_cysteine_synthase_A_[Ruminococcus_bromii_YE282]	Ruminococcus_bromii_YE282
Org1_Gene825	unique	932	2595398778_IE32DRAFT_00860_diaminobutyrate_aminotransferase_apoenzyme_(EC_2.6.1.76)_[Ruminococcus_bromii_YE282]	Ruminococcus_bromii_YE282
Org1_Gene826	unique	3399	2595398779_IE32DRAFT_00861_hypothetical_protein_[Ruminococcus_bromii_YE282]	Ruminococcus_bromii_YE282
Org1_Gene827	unique	304	2595398780_IE32DRAFT_00862_sortase,_SrtB_family_[Ruminococcus_bromii_YE282]	Ruminococcus_bromii_YE282
Org1_Gene83	unique	3426	2595398006_IE32DRAFT_00087_hypothetical_protein_[Ruminococcus_bromii_YE282]	Ruminococcus_bromii_YE282
Org1_Gene831	unique	3771	2595398784_IE32DRAFT_00866_Predicted_transcriptional_regulators_[Ruminococcus_bromii_YE282]	Ruminococcus_bromii_YE282
Org1_Gene832	unique	1948	2595398785_IE32DRAFT_00867_hypothetical_protein_[Ruminococcus_bromii_YE282]	Ruminococcus_bromii_YE282
Org1_Gene833	unique	3413	2595398786_IE32DRAFT_00868_hypothetical_protein_[Ruminococcus_bromii_YE282]	Ruminococcus_bromii_YE282
Org1_Gene834	unique	3154	2595398787_IE32DRAFT_00869_hypothetical_protein_[Ruminococcus_bromii_YE282]	Ruminococcus_bromii_YE282
Org1_Gene838	unique	4425	2595398791_IE32DRAFT_00873_hypothetical_protein_[Ruminococcus_bromii_YE282]	Ruminococcus_bromii_YE282
Org1_Gene84	unique	4413	2595398007_IE32DRAFT_00088_hypothetical_protein_[Ruminococcus_bromii_YE282]	Ruminococcus_bromii_YE282
Org1_Gene841	unique	309	2595398794_IE32DRAFT_00876_hypothetical_protein_[Ruminococcus_bromii_YE282]	Ruminococcus_bromii_YE282
Org1_Gene842	unique	1038	2595398795_IE32DRAFT_00877_DHHW_protein_[Ruminococcus_bromii_YE282]	Ruminococcus_bromii_YE282
Org1_Gene843	unique	683	2595398796_IE32DRAFT_00878_Predicted_membrane_protein_involved_in_D-alanine_export_[Ruminococcus_bromii_YE282]	Ruminococcus_bromii_YE282
Org1_Gene845	unique	1804	2595398798_IE32DRAFT_00880_dTDP-4-dehydrorhamnose_reductase_(EC_1.1.1.133)_[Ruminococcus_bromii_YE282]	Ruminococcus_bromii_YE282
Org1_Gene846	unique	1385	2595398799_IE32DRAFT_00881_hypothetical_protein_[Ruminococcus_bromii_YE282]	Ruminococcus_bromii_YE282
Org1_Gene847	unique	1388	2595398800_IE32DRAFT_00882_dTDP-glucose_4,6-dehydratase_(EC_4.2.1.46)_[Ruminococcus_bromii_YE282]	Ruminococcus_bromii_YE282
Org1_Gene848	unique	1743	2595398801_IE32DRAFT_00883_glucose-1-phosphate_thymidylyltransferase,_short_form_[Ruminococcus_bromii_YE282]	Ruminococcus_bromii_YE282
Org1_Gene85	unique	2557	2595398008_IE32DRAFT_00089_transcriptional_regulator,_TetR_family_[Ruminococcus_bromii_YE282]	Ruminococcus_bromii_YE282
Org1_Gene851	unique	1259	2595398804_IE32DRAFT_00886_Predicted_acyltransferases_[Ruminococcus_bromii_YE282]	Ruminococcus_bromii_YE282
Org1_Gene852	unique	3093	2595398805_IE32DRAFT_00887_3-deoxy-D-manno-octulosonate_8-phosphate_phosphatase,_YrbI_family_[Ruminococcus_bromii_YE282]	Ruminococcus_bromii_YE282
Org1_Gene853	unique	491	2595398806_IE32DRAFT_00888_HMGL-like_[Ruminococcus_bromii_YE282]	Ruminococcus_bromii_YE282
Org1_Gene854	unique	2238	2595398807_IE32DRAFT_00889_CMP-2-keto-3-deoxyoctulosonic_acid_synthetase_[Ruminococcus_bromii_YE282]	Ruminococcus_bromii_YE282
Org1_Gene855	unique	2086	2595398808_IE32DRAFT_00890_Protein_of_unknown_function_DUF115_[Ruminococcus_bromii_YE282]	Ruminococcus_bromii_YE282
Org1_Gene856	unique	1489	2595398809_IE32DRAFT_00891_Glycosyl_transferase_family_2_[Ruminococcus_bromii_YE282]	Ruminococcus_bromii_YE282
Org1_Gene857	unique	544	2595398810_IE32DRAFT_00892_Membrane_protein_involved_in_the_export_of_O-antigen_and_teichoic_acid_[Ruminococcus_bromii_YE282]	Ruminococcus_bromii_YE282
Org1_Gene858	unique	1416	2595398811_IE32DRAFT_00893_Glycosyltransferases_involved_in_cell_wall_biogenesis_[Ruminococcus_bromii_YE282]	Ruminococcus_bromii_YE282
Org1_Gene859	unique	1486	2595398812_IE32DRAFT_00894_Glycosyl_transferase_family_2_[Ruminococcus_bromii_YE282]	Ruminococcus_bromii_YE282
Org1_Gene86	unique	621	2595398009_IE32DRAFT_00090_Protease_subunit_of_ATP-dependent_Clp_proteases_[Ruminococcus_bromii_YE282]	Ruminococcus_bromii_YE282
Org1_Gene860	unique	1430	2595398813_IE32DRAFT_00895_Glycosyl_transferase_family_2_[Ruminococcus_bromii_YE282]	Ruminococcus_bromii_YE282
Org1_Gene861	unique	1046	2595398814_IE32DRAFT_00896_Glycosyltransferases_involved_in_cell_wall_biogenesis_[Ruminococcus_bromii_YE282]	Ruminococcus_bromii_YE282
Org1_Gene862	unique	1205	2595398815_IE32DRAFT_00897_Glycosyltransferase_[Ruminococcus_bromii_YE282]	Ruminococcus_bromii_YE282
Org1_Gene863	unique	1721	2595398816_IE32DRAFT_00898_Glycosyltransferases_involved_in_cell_wall_biogenesis_[Ruminococcus_bromii_YE282]	Ruminococcus_bromii_YE282
Org1_Gene864	unique	1393	2595398817_IE32DRAFT_00899_Glycosyl_transferase_family_2_[Ruminococcus_bromii_YE282]	Ruminococcus_bromii_YE282
Org1_Gene865	unique	1332	2595398818_IE32DRAFT_00900_Glycosyl_transferase_family_2_[Ruminococcus_bromii_YE282]	Ruminococcus_bromii_YE282
Org1_Gene866	unique	1346	2595398819_IE32DRAFT_00901_EpsG_family_[Ruminococcus_bromii_YE282]	Ruminococcus_bromii_YE282
Org1_Gene867	unique	1190	2595398820_IE32DRAFT_00902_Glycosyltransferase_[Ruminococcus_bromii_YE282]	Ruminococcus_bromii_YE282
Org1_Gene868	unique	499	2595398821_IE32DRAFT_00903_hypothetical_protein_[Ruminococcus_bromii_YE282]	Ruminococcus_bromii_YE282
Org1_Gene869	unique	1663	2595398822_IE32DRAFT_00904_Phosphoglycerate_dehydrogenase_and_related_dehydrogenases_[Ruminococcus_bromii_YE282]	Ruminococcus_bromii_YE282
Org1_Gene87	unique	3029	2595398010_IE32DRAFT_00091_Predicted_acetyltransferase_[Ruminococcus_bromii_YE282]	Ruminococcus_bromii_YE282
Org1_Gene870	unique	1943	2595398823_IE32DRAFT_00905_HpcH/HpaI_aldolase/citrate_lyase_family_[Ruminococcus_bromii_YE282]	Ruminococcus_bromii_YE282
Org1_Gene871	unique	2575	2595398824_IE32DRAFT_00906_Sugar_transferases_involved_in_lipopolysaccharide_synthesis_[Ruminococcus_bromii_YE282]	Ruminococcus_bromii_YE282
Org1_Gene872	unique	1914	2595398825_IE32DRAFT_00907_Predicted_phosphohydrolases_[Ruminococcus_bromii_YE282]	Ruminococcus_bromii_YE282
Org1_Gene873	unique	1287	2595398826_IE32DRAFT_00908_Uncharacterized_protein_conserved_in_bacteria_[Ruminococcus_bromii_YE282]	Ruminococcus_bromii_YE282
Org1_Gene874	unique	1315	2595398827_IE32DRAFT_00909_Uncharacterized_protein_conserved_in_bacteria_[Ruminococcus_bromii_YE282]	Ruminococcus_bromii_YE282
Org1_Gene876	unique	2564	2595398829_IE32DRAFT_00911_Vitamin_B12_dependent_methionine_synthase,_activation_domain_[Ruminococcus_bromii_YE282]	Ruminococcus_bromii_YE282
Org1_Gene878	unique	1376	2595398831_IE32DRAFT_00913_CAAX_protease_self-immunity_[Ruminococcus_bromii_YE282]	Ruminococcus_bromii_YE282
Org1_Gene879	unique	3951	2595398832_IE32DRAFT_00914_hypothetical_protein_[Ruminococcus_bromii_YE282]	Ruminococcus_bromii_YE282
Org1_Gene88	unique	4260	2595398011_IE32DRAFT_00092_hypothetical_protein_[Ruminococcus_bromii_YE282]	Ruminococcus_bromii_YE282
Org1_Gene882	unique	645	2595398835_IE32DRAFT_00917_hypothetical_protein_[Ruminococcus_bromii_YE282]	Ruminococcus_bromii_YE282
Org1_Gene883	unique	1842	2595398836_IE32DRAFT_00918_hypothetical_protein_[Ruminococcus_bromii_YE282]	Ruminococcus_bromii_YE282
Org1_Gene885	unique	3648	2595398838_IE32DRAFT_00920_Dockerin_type_I_repeat_[Ruminococcus_bromii_YE282]	Ruminococcus_bromii_YE282
Org1_Gene886	unique	176	2595398839_IE32DRAFT_00921_FtsX-like_permease_family_[Ruminococcus_bromii_YE282]	Ruminococcus_bromii_YE282
Org1_Gene887	unique	2154	2595398840_IE32DRAFT_00922_ABC-type_antimicrobial_peptide_transport_system,_ATPase_component_[Ruminococcus_bromii_YE282]	Ruminococcus_bromii_YE282
Org1_Gene888	unique	2490	2595398841_IE32DRAFT_00923_Response_regulators_consisting_of_a_CheY-like_receiver_domain_and_a_winged-helix_DNA-binding_domain_[Ruminoc	Ruminococcus_bromii_YE282
Org1_Gene889	unique	972	2595398842_IE32DRAFT_00924_Signal_transduction_histidine_kinase_[Ruminococcus_bromii_YE282]	Ruminococcus_bromii_YE282
Org1_Gene89	unique	3518	2595398012_IE32DRAFT_00093_Bacterial_transferase_hexapeptide_(six_repeats)_[Ruminococcus_bromii_YE282]	Ruminococcus_bromii_YE282
Org1_Gene891	unique	4148	2595398844_IE32DRAFT_00926_hypothetical_protein_[Ruminococcus_bromii_YE282]	Ruminococcus_bromii_YE282
Org1_Gene892	unique	1389	2595398845_IE32DRAFT_00927_ABC-type_uncharacterized_transport_system,_ATPase_component_[Ruminococcus_bromii_YE282]	Ruminococcus_bromii_YE282
Org1_Gene893	unique	1985	2595398846_IE32DRAFT_00928_ABC-type_uncharacterized_transport_system,_permease_component_[Ruminococcus_bromii_YE282]	Ruminococcus_bromii_YE282
Org1_Gene894	unique	2045	2595398847_IE32DRAFT_00929_ABC-type_uncharacterized_transport_system,_permease_component_[Ruminococcus_bromii_YE282]	Ruminococcus_bromii_YE282
Org1_Gene895	unique	35	2595398848_IE32DRAFT_00930_pullulanase,_type_I_[Ruminococcus_bromii_YE282]	Ruminococcus_bromii_YE282
Org1_Gene898	unique	3970	2595398851_IE32DRAFT_00933_hypothetical_protein_[Ruminococcus_bromii_YE282]	Ruminococcus_bromii_YE282
Org1_Gene899	unique	2023	2595398852_IE32DRAFT_00934_sortase,_SrtB_family_[Ruminococcus_bromii_YE282]	Ruminococcus_bromii_YE282
Org1_Gene900	unique	770	2595398853_IE32DRAFT_00935_transcriptional_attenuator,_LytR_family_[Ruminococcus_bromii_YE282]	Ruminococcus_bromii_YE282
Org1_Gene901	unique	2844	2595398854_IE32DRAFT_00936_hypothetical_protein_[Ruminococcus_bromii_YE282]	Ruminococcus_bromii_YE282
Org1_Gene902	unique	2012	2595398855_IE32DRAFT_00937_sortase,_SrtB_family_[Ruminococcus_bromii_YE282]	Ruminococcus_bromii_YE282
Org1_Gene903	unique	1045	2595398856_IE32DRAFT_00938_transcriptional_attenuator,_LytR_family_[Ruminococcus_bromii_YE282]	Ruminococcus_bromii_YE282
Org1_Gene904	unique	458	2595398857_IE32DRAFT_00939_Na+/phosphate_symporter_[Ruminococcus_bromii_YE282]	Ruminococcus_bromii_YE282
Org1_Gene907	unique	142	2595398860_IE32DRAFT_00942_Bacterial_membrane_protein_YfhO_[Ruminococcus_bromii_YE282]	Ruminococcus_bromii_YE282
Org1_Gene909	unique	2078	2595398862_IE32DRAFT_00944_hypothetical_protein_[Ruminococcus_bromii_YE282]	Ruminococcus_bromii_YE282
Org1_Gene91	unique	2749	2595398017_IE32DRAFT_00098_RNA_polymerase_sigma_factor,_sigma-70_family_[Ruminococcus_bromii_YE282]	Ruminococcus_bromii_YE282
Org1_Gene912	unique	2256	2595398865_IE32DRAFT_00947_Predicted_DNA_alkylation_repair_enzyme_[Ruminococcus_bromii_YE282]	Ruminococcus_bromii_YE282
Org1_Gene913	unique	2573	2595398866_IE32DRAFT_00949_Predicted_membrane_protein_[Ruminococcus_bromii_YE282]	Ruminococcus_bromii_YE282
Org1_Gene914	unique	2789	2595398867_IE32DRAFT_00951_ECF-type_riboflavin_transporter,_S_component_[Ruminococcus_bromii_YE282]	Ruminococcus_bromii_YE282
Org1_Gene92	unique	807	2595398018_IE32DRAFT_00099_Mg_chelatase-related_protein_[Ruminococcus_bromii_YE282]	Ruminococcus_bromii_YE282
Org1_Gene920	unique	296	2595398873_IE32DRAFT_00957_Predicted_signaling_protein_consisting_of_a_modified_GGDEF_domain_and_a_DHH_domain_[Ruminococcus_bromii_YE	Ruminococcus_bromii_YE282
Org1_Gene923	unique	824	2595398876_IE32DRAFT_00960_tRNA(Ile)-lysidine_synthetase,_N-terminal_domain/tRNA(Ile)-lysidine_synthetase,_C-terminal_domain_[Ruminococcus_b	Ruminococcus_bromii_YE282
Org1_Gene928	unique	576	2595398881_IE32DRAFT_00965_Lyzozyme_M1_(1,4-beta-N-acetylmuramidase)_[Ruminococcus_bromii_YE282]	Ruminococcus_bromii_YE282
Org1_Gene93	unique	3102	2595398019_IE32DRAFT_00100_hypothetical_protein_[Ruminococcus_bromii_YE282]	Ruminococcus_bromii_YE282
Org1_Gene930	unique	1360	2595398883_IE32DRAFT_00967_Peptidyl-prolyl_cis-trans_isomerase_(rotamase)_-_cyclophilin_family_[Ruminococcus_bromii_YE282]	Ruminococcus_bromii_YE282
Org1_Gene932	unique	911	2595398885_IE32DRAFT_00969_Uncharacterized_protein_conserved_in_bacteria_[Ruminococcus_bromii_YE282]	Ruminococcus_bromii_YE282
Org1_Gene933	unique	1861	2595398886_IE32DRAFT_00970_Aldo/keto_reductases,_related_to_diketogulonate_reductase_[Ruminococcus_bromii_YE282]	Ruminococcus_bromii_YE282
Org1_Gene934	unique	1924	2595398887_IE32DRAFT_00971_Dehydrogenases_with_different_specificities_(related_to_short-chain_alcohol_dehydrogenases)_[Ruminococcus_bromii_	Ruminococcus_bromii_YE282
Org1_Gene935	unique	3022	2595398888_IE32DRAFT_00972_Flavodoxins_[Ruminococcus_bromii_YE282]	Ruminococcus_bromii_YE282
Org1_Gene936	unique	1718	2595398889_IE32DRAFT_00973_Transcriptional_regulator_[Ruminococcus_bromii_YE282]	Ruminococcus_bromii_YE282
Org1_Gene937	unique	2175	2595398890_IE32DRAFT_00974_Flavodoxin_domain/4Fe-4S_dicluster_domain_[Ruminococcus_bromii_YE282]	Ruminococcus_bromii_YE282
Org1_Gene938	unique	3683	2595398891_IE32DRAFT_00975_Integrase_core_domain_[Ruminococcus_bromii_YE282]	Ruminococcus_bromii_YE282
Org1_Gene94	unique	1111	2595398020_IE32DRAFT_00101_Glycosyl_hydrolases_family_25_[Ruminococcus_bromii_YE282]	Ruminococcus_bromii_YE282
Org1_Gene940	unique	1090	2595398893_IE32DRAFT_00977_hypothetical_protein_[Ruminococcus_bromii_YE282]	Ruminococcus_bromii_YE282
Org1_Gene941	unique	4187	2595398894_IE32DRAFT_00978_transcriptional_regulator,_AlpA_family_[Ruminococcus_bromii_YE282]	Ruminococcus_bromii_YE282
Org1_Gene942	unique	3605	2595398895_IE32DRAFT_00979_Helix-turn-helix_domain_[Ruminococcus_bromii_YE282]	Ruminococcus_bromii_YE282
Org1_Gene943	unique	3455	2595398896_IE32DRAFT_00980_hypothetical_protein_[Ruminococcus_bromii_YE282]	Ruminococcus_bromii_YE282
Org1_Gene944	unique	809	2595398897_IE32DRAFT_00981_hypothetical_protein_[Ruminococcus_bromii_YE282]	Ruminococcus_bromii_YE282
Org1_Gene946	unique	315	2595398899_IE32DRAFT_00983_Type_I_restriction-modification_system_methyltransferase_subunit_[Ruminococcus_bromii_YE282]	Ruminococcus_bromii_YE282
Org1_Gene947	unique	2717	2595398900_IE32DRAFT_00984_Type_I_restriction_enzyme_R_protein_N_terminus_(HSDR_N)_[Ruminococcus_bromii_YE282]	Ruminococcus_bromii_YE282
Org1_Gene948	unique	2267	2595398902_IE32DRAFT_00986_Capsular_polysaccharide_biosynthesis_protein_[Ruminococcus_bromii_YE282]	Ruminococcus_bromii_YE282
Org1_Gene95	unique	1520	2595398021_IE32DRAFT_00102_CHAP_domain_[Ruminococcus_bromii_YE282]	Ruminococcus_bromii_YE282
Org1_Gene951	unique	2461	2595398905_IE32DRAFT_00989_Helix-turn-helix_[Ruminococcus_bromii_YE282]	Ruminococcus_bromii_YE282
Org1_Gene952	unique	2418	2595398906_IE32DRAFT_00990_Peptidyl-prolyl_cis-trans_isomerase_(rotamase)_-_cyclophilin_family_[Ruminococcus_bromii_YE282]	Ruminococcus_bromii_YE282
Org1_Gene953	unique	1796	2595398907_IE32DRAFT_00991_Bacteriophage_protein_gp37_[Ruminococcus_bromii_YE282]	Ruminococcus_bromii_YE282
Org1_Gene954	unique	1776	2595398908_IE32DRAFT_00992_NAD-dependent_protein_deacetylases,_SIR2_family_[Ruminococcus_bromii_YE282]	Ruminococcus_bromii_YE282
Org1_Gene955	unique	1546	2595398909_IE32DRAFT_00993_NADPH:quinone_reductase_and_related_Zn-dependent_oxidoreductases_[Ruminococcus_bromii_YE282]	Ruminococcus_bromii_YE282
Org1_Gene956	unique	1230	2595398910_IE32DRAFT_00994_radical_SAM/SPASM_domain_protein,_ACGX_system_[Ruminococcus_bromii_YE282]	Ruminococcus_bromii_YE282
Org1_Gene957	unique	4361	2595398911_IE32DRAFT_00995_ACGX-repeat_peptide_[Ruminococcus_bromii_YE282]	Ruminococcus_bromii_YE282
Org1_Gene958	unique	3721	2595398912_IE32DRAFT_00996_transcriptional_regulator,_HxlR_family_[Ruminococcus_bromii_YE282]	Ruminococcus_bromii_YE282
Org1_Gene959	unique	475	2595398913_IE32DRAFT_00997_Acyl-coenzyme_A_synthetases/AMP-(fatty)_acid_ligases_[Ruminococcus_bromii_YE282]	Ruminococcus_bromii_YE282
Org1_Gene96	unique	3453	2595398022_IE32DRAFT_00103_hypothetical_protein_[Ruminococcus_bromii_YE282]	Ruminococcus_bromii_YE282
Org1_Gene960	unique	4352	2595398914_IE32DRAFT_00998_TIGR04076_family_protein_[Ruminococcus_bromii_YE282]	Ruminococcus_bromii_YE282
Org1_Gene961	unique	3420	2595398915_IE32DRAFT_00999_hypothetical_protein_[Ruminococcus_bromii_YE282]	Ruminococcus_bromii_YE282
Org1_Gene962	unique	2207	2595398916_IE32DRAFT_01000_hypothetical_protein_[Ruminococcus_bromii_YE282]	Ruminococcus_bromii_YE282
Org1_Gene963	unique	2520	2595398917_IE32DRAFT_01001_hypothetical_protein_[Ruminococcus_bromii_YE282]	Ruminococcus_bromii_YE282
Org1_Gene964	unique	2095	2595398918_IE32DRAFT_01002_Methyltransferase_domain_[Ruminococcus_bromii_YE282]	Ruminococcus_bromii_YE282
Org1_Gene965	unique	385	2595398919_IE32DRAFT_01003_ABC-type_multidrug_transport_system,_ATPase_and_permease_components_[Ruminococcus_bromii_YE282]	Ruminococcus_bromii_YE282
Org1_Gene966	unique	365	2595398920_IE32DRAFT_01004_ABC-type_multidrug_transport_system,_ATPase_and_permease_components_[Ruminococcus_bromii_YE282]	Ruminococcus_bromii_YE282
Org1_Gene967	unique	1927	2595398921_IE32DRAFT_01005_O-Methyltransferase_involved_in_polyketide_biosynthesis_[Ruminococcus_bromii_YE282]	Ruminococcus_bromii_YE282
Org1_Gene968	unique	1756	2595398922_IE32DRAFT_01006_Zn-dependent_hydrolases,_including_glyoxylases_[Ruminococcus_bromii_YE282]	Ruminococcus_bromii_YE282
Org1_Gene969	unique	3086	2595398923_IE32DRAFT_01007_Putative_protein-S-isoprenylcysteine_methyltransferase_[Ruminococcus_bromii_YE282]	Ruminococcus_bromii_YE282
Org1_Gene97	unique	3267	2595398023_IE32DRAFT_00104_hypothetical_protein_[Ruminococcus_bromii_YE282]	Ruminococcus_bromii_YE282

Org1_Gene970	unique	4119	2595398924_IE32DRAFT_01008_Predicted_transcriptional_regulator_[Ruminococcus_bromii_YE282]	Ruminococcus_bromii_YE282
Org1_Gene971	unique	2623	2595398925_IE32DRAFT_01009_Methyltransferase_domain_[Ruminococcus_bromii_YE282]	Ruminococcus_bromii_YE282
Org1_Gene972	unique	3042	2595398926_IE32DRAFT_01010_Putative_protein-S-isoprenylcysteine_methyltransferase_[Ruminococcus_bromii_YE282]	Ruminococcus_bromii_YE282
Org1_Gene973	unique	2943	2595398927_IE32DRAFT_01011_Leucine_carboxyl_methyltransferase_[Ruminococcus_bromii_YE282]	Ruminococcus_bromii_YE282
Org1_Gene974	unique	4136	2595398928_IE32DRAFT_01012_hypothetical_protein_[Ruminococcus_bromii_YE282]	Ruminococcus_bromii_YE282
Org1_Gene975	unique	1323	2595398929_IE32DRAFT_01013_Esterase/lipase_[Ruminococcus_bromii_YE282]	Ruminococcus_bromii_YE282
Org1_Gene976	unique	2723	2595398930_IE32DRAFT_01014_conserved_hypothetical_integral_membrane_protein_TIGR02185_[Ruminococcus_bromii_YE282]	Ruminococcus_bromii_YE282
Org1_Gene977	unique	2211	2595398931_IE32DRAFT_01015_ABC-type_cobalt_transport_system,_permease_component_CbiQ_and_related_transporters_[Ruminococcus_bromii_YE2	Ruminococcus_bromii_YE282
Org1_Gene978	unique	587	2595398932_IE32DRAFT_01016_ATPase_components_of_various_ABC-type_transport_systems,_contain_duplicated_ATPase_[Ruminococcus_bromii_YE2	Ruminococcus_bromii_YE282
Org1_Gene979	unique	1353	2595398933_IE32DRAFT_01017_AraC-type_DNA-binding_domain-containing_proteins_[Ruminococcus_bromii_YE282]	Ruminococcus_bromii_YE282
Org1_Gene98	unique	3471	2595398024_IE32DRAFT_00105_GIY-YIG_catalytic_domain_[Ruminococcus_bromii_YE282]	Ruminococcus_bromii_YE282
Org1_Gene980	unique	2033	2595398934_IE32DRAFT_01018_Recombinase_[Ruminococcus_bromii_YE282]	Ruminococcus_bromii_YE282
Org1_Gene981	unique	1916	2595398935_IE32DRAFT_01019_hypothetical_protein_[Ruminococcus_bromii_YE282]	Ruminococcus_bromii_YE282
Org1_Gene982	unique	82	2595398936_IE32DRAFT_01020_RNA_dependent_RNA_polymerase_[Ruminococcus_bromii_YE282]	Ruminococcus_bromii_YE282
Org1_Gene983	unique	650	2595398937_IE32DRAFT_01021_hypothetical_protein_[Ruminococcus_bromii_YE282]	Ruminococcus_bromii_YE282
Org1_Gene984	unique	3840	2595398938_IE32DRAFT_01022_hypothetical_protein_[Ruminococcus_bromii_YE282]	Ruminococcus_bromii_YE282
Org1_Gene985	unique	3779	2595398939_IE32DRAFT_01023_Bacterial_mobilisation_protein_(MobC)_[Ruminococcus_bromii_YE282]	Ruminococcus_bromii_YE282
Org1_Gene986	unique	1459	2595398940_IE32DRAFT_01024_Relaxase/Mobilisation_nuclease_domain_[Ruminococcus_bromii_YE282]	Ruminococcus_bromii_YE282
Org1_Gene987	unique	4354	2595398941_IE32DRAFT_01025_hypothetical_protein_[Ruminococcus_bromii_YE282]	Ruminococcus_bromii_YE282
Org1_Gene988	unique	1061	2595398942_IE32DRAFT_01026_Site-specific_recombinases,_DNA_invertase_Pin_homologs_[Ruminococcus_bromii_YE282]	Ruminococcus_bromii_YE282
Org1_Gene989	unique	3642	2595398943_IE32DRAFT_01027_hypothetical_protein_[Ruminococcus_bromii_YE282]	Ruminococcus_bromii_YE282
Org1_Gene99	unique	3341	2595398025_IE32DRAFT_00106_Protein_of_unknown_function_(DUF1113)_[Ruminococcus_bromii_YE282]	Ruminococcus_bromii_YE282
Org1_Gene990	unique	4147	2595398945_IE32DRAFT_01029_hypothetical_protein_[Ruminococcus_bromii_YE282]	Ruminococcus_bromii_YE282
Org1_Gene991	unique	339	2595398946_IE32DRAFT_01030_LPS_biosynthesis_protein_[Ruminococcus_bromii_YE282]	Ruminococcus_bromii_YE282
Org1_Gene992	unique	1250	2595398947_IE32DRAFT_01031_Nucleoside-diphosphate-sugar_epimerases_[Ruminococcus_bromii_YE282]	Ruminococcus_bromii_YE282
Org1_Gene993	unique	2234	2595398948_IE32DRAFT_01032_4-diphosphocytidyl-2-methyl-D-erithritol_synthase_[Ruminococcus_bromii_YE282]	Ruminococcus_bromii_YE282
Org2_Gene10	unique	302	[Rbromii5AMG]RB5AMG_00010_Cell_wall-associated_polypeptide_CWBP200	Rbromii5AMG
Org2_Gene1054	unique	3023	[Rbromii5AMG]RB5AMG_01085_hypothetical_protein	Rbromii5AMG
Org2_Gene1058	unique	3224	[Rbromii5AMG]RB5AMG_01089_hypothetical_protein	Rbromii5AMG
Org2_Gene106	unique	833	[Rbromii5AMG]RB5AMG_00114_hypothetical_protein	Rbromii5AMG
Org2_Gene107	unique	2332	[Rbromii5AMG]RB5AMG_00115_Transcriptional_regulatory_protein_YehT	Rbromii5AMG
Org2_Gene108	unique	834	[Rbromii5AMG]RB5AMG_00116_Maltose-binding_periplasmic_proteins/domains	Rbromii5AMG
Org2_Gene1090	unique	2087	[Rbromii5AMG]RB5AMG_01124_putative_methyltransferase_YcgJ	Rbromii5AMG
Org2_Gene1098	unique	1496	[Rbromii5AMG]RB5AMG_01132_putative_glycosyltransferase_EpsJ	Rbromii5AMG
Org2_Gene1099	unique	1736	[Rbromii5AMG]RB5AMG_01133_Chondroitin_polymerase	Rbromii5AMG
Org2_Gene11	unique	1149	[Rbromii5AMG]RB5AMG_00011_hypothetical_protein	Rbromii5AMG
Org2_Gene1100	unique	1384	[Rbromii5AMG]RB5AMG_01134_putative_glycosyltransferase_EpsJ	Rbromii5AMG
Org2_Gene1101	unique	1210	[Rbromii5AMG]RB5AMG_01135_N-acetylgalactosamine-N,N'-diacetylbacillosaminyl-diphospho-undecaprenol_4-alpha-N-acetylgalactosaminyltransferase	Rbromii5AMG
Org2_Gene1102	unique	1048	[Rbromii5AMG]RB5AMG_01136_Putative_glycosyltransferase_EpsH	Rbromii5AMG
Org2_Gene1103	unique	1462	[Rbromii5AMG]RB5AMG_01137_putative_glycosyltransferase_EpsJ	Rbromii5AMG
Org2_Gene1104	unique	1480	[Rbromii5AMG]RB5AMG_01138_putative_glycosyltransferase_EpsJ	Rbromii5AMG
Org2_Gene1105	unique	1424	[Rbromii5AMG]RB5AMG_01139_putative_glycosyltransferase_EpsJ	Rbromii5AMG
Org2_Gene1106	unique	3884	[Rbromii5AMG]RB5AMG_01140_hypothetical_protein	Rbromii5AMG
Org2_Gene1107	unique	3919	[Rbromii5AMG]RB5AMG_01141_hypothetical_protein	Rbromii5AMG
Org2_Gene1108	unique	4433	[Rbromii5AMG]RB5AMG_01142_hypothetical_protein	Rbromii5AMG
Org2_Gene1109	unique	554	[Rbromii5AMG]RB5AMG_01143_Polysaccharide_biosynthesis_protein	Rbromii5AMG
Org2_Gene111	unique	733	[Rbromii5AMG]RB5AMG_00119_Maltose-binding_periplasmic_proteins/domains	Rbromii5AMG
Org2_Gene1110	unique	2093	[Rbromii5AMG]RB5AMG_01144_hypothetical_protein	Rbromii5AMG
Org2_Gene1111	unique	1515	[Rbromii5AMG]RB5AMG_01145_hypothetical_protein	Rbromii5AMG
Org2_Gene1112	unique	2540	[Rbromii5AMG]RB5AMG_01146_3-deoxy-manno-octulosonate_cytidylyltransferase	Rbromii5AMG
Org2_Gene1113	unique	2119	[Rbromii5AMG]RB5AMG_01147_2-dehydro-3-deoxyphosphooctonate_aldolase	Rbromii5AMG
Org2_Gene1114	unique	1505	[Rbromii5AMG]RB5AMG_01148_Arabinose_5-phosphate_isomerase_KdsD	Rbromii5AMG
Org2_Gene1115	unique	3217	[Rbromii5AMG]RB5AMG_01149_3-deoxy-D-manno-octulosonate_8-phosphate_phosphatase_KdsC	Rbromii5AMG
Org2_Gene1116	unique	936	[Rbromii5AMG]RB5AMG_01150_Capsular_polysaccharide_biosynthesis_protein	Rbromii5AMG
Org2_Gene1117	unique	1361	[Rbromii5AMG]RB5AMG_01151_Fucose_4-O-acetylase	Rbromii5AMG
Org2_Gene1118	unique	4428	[Rbromii5AMG]RB5AMG_01152_hypothetical_protein	Rbromii5AMG
Org2_Gene1129	unique	3606	[Rbromii5AMG]RB5AMG_01163_Spermine/spermidine_acetyltransferase	Rbromii5AMG
Org2_Gene1138	unique	2677	[Rbromii5AMG]RB5AMG_01173_hypothetical_protein	Rbromii5AMG
Org2_Gene116	unique	3273	[Rbromii5AMG]RB5AMG_00124_hypothetical_protein	Rbromii5AMG
Org2_Gene123	unique	4392	[Rbromii5AMG]RB5AMG_00132_hypothetical_protein	Rbromii5AMG
Org2_Gene1242	unique	2905	[Rbromii5AMG]RB5AMG_01279_hypothetical_protein	Rbromii5AMG
Org2_Gene1243	unique	2919	[Rbromii5AMG]RB5AMG_01280_hypothetical_protein	Rbromii5AMG
Org2_Gene1268	unique	3161	[Rbromii5AMG]RB5AMG_01305_putative_integral_membrane_protein	Rbromii5AMG
Org2_Gene1304	unique	263	[Rbromii5AMG]RB5AMG_01343_Exoglucanase_B_precursor	Rbromii5AMG
Org2_Gene1308	unique	1161	[Rbromii5AMG]RB5AMG_01348_Integrase	Rbromii5AMG
Org2_Gene1309	unique	4162	[Rbromii5AMG]RB5AMG_01349_DNA_binding_domain,_excisionase_family	Rbromii5AMG
Org2_Gene1310	unique	2806	[Rbromii5AMG]RB5AMG_01350_mRNA_interferase_MazF	Rbromii5AMG
Org2_Gene1311	unique	2865	[Rbromii5AMG]RB5AMG_01351_RNA_polymerase_sigma_factor	Rbromii5AMG
Org2_Gene1312	unique	122	[Rbromii5AMG]RB5AMG_01352_Restriction_endonuclease	Rbromii5AMG
Org2_Gene1313	unique	387	[Rbromii5AMG]RB5AMG_01353_putative_methyltransferase	Rbromii5AMG
Org2_Gene1314	unique	3146	[Rbromii5AMG]RB5AMG_01354_Bifunctional_(p)ppGpp_synthase/hydrolase_RelA	Rbromii5AMG
Org2_Gene1315	unique	2703	[Rbromii5AMG]RB5AMG_01355_putative_membrane_protein	Rbromii5AMG
Org2_Gene1316	unique	3214	[Rbromii5AMG]RB5AMG_01356_hypothetical_protein	Rbromii5AMG
Org2_Gene1317	unique	1108	[Rbromii5AMG]RB5AMG_01357_hypothetical_protein	Rbromii5AMG
Org2_Gene1318	unique	3121	[Rbromii5AMG]RB5AMG_01358_hypothetical_protein	Rbromii5AMG
Org2_Gene1319	unique	159	[Rbromii5AMG]RB5AMG_01359_hypothetical_protein	Rbromii5AMG
Org2_Gene1320	unique	36	[Rbromii5AMG]RB5AMG_01360_Type_IV_secretory_pathway,_VirB4_components	Rbromii5AMG
Org2_Gene1321	unique	3138	[Rbromii5AMG]RB5AMG_01361_hypothetical_protein	Rbromii5AMG
Org2_Gene1322	unique	3535	[Rbromii5AMG]RB5AMG_01362_hypothetical_protein	Rbromii5AMG
Org2_Gene1323	unique	55	[Rbromii5AMG]RB5AMG_01363_Type_VII_secretion_system_protein_EccCa1	Rbromii5AMG
Org2_Gene1324	unique	1347	[Rbromii5AMG]RB5AMG_01364_hypothetical_protein	Rbromii5AMG
Org2_Gene1325	unique	3014	[Rbromii5AMG]RB5AMG_01365_hypothetical_protein	Rbromii5AMG
Org2_Gene1326	unique	2077	[Rbromii5AMG]RB5AMG_01366_prolipoprotein_diacylglyceryl_transferase	Rbromii5AMG
Org2_Gene1327	unique	3916	[Rbromii5AMG]RB5AMG_01367_hypothetical_protein	Rbromii5AMG
Org2_Gene1328	unique	3736	[Rbromii5AMG]RB5AMG_01368_HTH-type_transcriptional_regulator_ImmR	Rbromii5AMG
Org2_Gene1329	unique	366	[Rbromii5AMG]RB5AMG_01369_hypothetical_protein	Rbromii5AMG
Org2_Gene1330	unique	411	[Rbromii5AMG]RB5AMG_01370_putative_ATPase	Rbromii5AMG
Org2_Gene1331	unique	2007	[Rbromii5AMG]RB5AMG_01371_sortase,_SrtB_family	Rbromii5AMG
Org2_Gene1333	unique	3582	[Rbromii5AMG]RB5AMG_01373_PrgI_family_protein	Rbromii5AMG
Org2_Gene1335	unique	3552	[Rbromii5AMG]RB5AMG_01375_hypothetical_protein	Rbromii5AMG
Org2_Gene1337	unique	4241	[Rbromii5AMG]RB5AMG_01377_hypothetical_protein	Rbromii5AMG
Org2_Gene1338	unique	1750	[Rbromii5AMG]RB5AMG_01378_hypothetical_protein	Rbromii5AMG
Org2_Gene1339	unique	2027	[Rbromii5AMG]RB5AMG_01379_hypothetical_protein	Rbromii5AMG
Org2_Gene1340	unique	726	[Rbromii5AMG]RB5AMG_01380_Relaxase/Mobilisation_nuclease_domain_protein	Rbromii5AMG
Org2_Gene1341	unique	3675	[Rbromii5AMG]RB5AMG_01381_Bacterial_mobilisation_protein_(MobC)	Rbromii5AMG
Org2_Gene1342	unique	4405	[Rbromii5AMG]RB5AMG_01382_hypothetical_protein	Rbromii5AMG
Org2_Gene1343	unique	2220	[Rbromii5AMG]RB5AMG_01383_hypothetical_protein	Rbromii5AMG
Org2_Gene1344	unique	1516	[Rbromii5AMG]RB5AMG_01384_DNA_primase_(bacterial_type)	Rbromii5AMG
Org2_Gene1345	unique	3659	[Rbromii5AMG]RB5AMG_01385_hypothetical_protein	Rbromii5AMG
Org2_Gene1346	unique	10	[Rbromii5AMG]RB5AMG_01386_DNA_mismatch_repair_protein_MutS	Rbromii5AMG
Org2_Gene1347	unique	681	[Rbromii5AMG]RB5AMG_01387_Recombinase	Rbromii5AMG
Org2_Gene1361	unique	2190	[Rbromii5AMG]RB5AMG_01401_hypothetical_protein	Rbromii5AMG
Org2_Gene1363	unique	61	[Rbromii5AMG]RB5AMG_01403_Antirestriction_protein	Rbromii5AMG
Org2_Gene1375	unique	4216	[Rbromii5AMG]RB5AMG_01415_hypothetical_protein	Rbromii5AMG
Org2_Gene1376	unique	4224	[Rbromii5AMG]RB5AMG_01416_hypothetical_protein	Rbromii5AMG
Org2_Gene1377	unique	4258	[Rbromii5AMG]RB5AMG_01417_hypothetical_protein	Rbromii5AMG
Org2_Gene1378	unique	1894	[Rbromii5AMG]RB5AMG_01418_Primosomal_protein_DnaI	Rbromii5AMG
Org2_Gene1379	unique	2059	[Rbromii5AMG]RB5AMG_01419_hypothetical_protein	Rbromii5AMG
Org2_Gene1380	unique	490	[Rbromii5AMG]RB5AMG_01420_Relaxase/Mobilisation_nuclease_domain_protein	Rbromii5AMG
Org2_Gene1381	unique	3464	[Rbromii5AMG]RB5AMG_01421_hypothetical_protein	Rbromii5AMG
Org2_Gene1382	unique	4168	[Rbromii5AMG]RB5AMG_01422_Helix-turn-helix_domain_protein	Rbromii5AMG
Org2_Gene1383	unique	2149	[Rbromii5AMG]RB5AMG_01423_Mg-protoporphyrin_IX_methyl_transferase	Rbromii5AMG
Org2_Gene1384	unique	312	[Rbromii5AMG]RB5AMG_01424_Tetracycline_resistance_protein_TetO	Rbromii5AMG
Org2_Gene1385	unique	583	[Rbromii5AMG]RB5AMG_01425_DNA_methylase	Rbromii5AMG
Org2_Gene1386	unique	1597	[Rbromii5AMG]RB5AMG_01426_hypothetical_protein	Rbromii5AMG
Org2_Gene1387	unique	2819	[Rbromii5AMG]RB5AMG_01427_hypothetical_protein	Rbromii5AMG
Org2_Gene1388	unique	4054	[Rbromii5AMG]RB5AMG_01428_hypothetical_protein	Rbromii5AMG
Org2_Gene1390	unique	25	[Rbromii5AMG]RB5AMG_01430_T6_antigen	Rbromii5AMG
Org2_Gene1391	unique	1571	[Rbromii5AMG]RB5AMG_01431_putative_chromosome-partitioning_protein_ParB	Rbromii5AMG
Org2_Gene1392	unique	1956	[Rbromii5AMG]RB5AMG_01432_Sporulation_initiation_inhibitor_protein_soj	Rbromii5AMG
Org2_Gene1393	unique	4307	[Rbromii5AMG]RB5AMG_01433_hypothetical_protein	Rbromii5AMG
Org2_Gene1394	unique	321	[Rbromii5AMG]RB5AMG_01434_putative_ATP-binding_protein_involved_in_virulence	Rbromii5AMG

Org2_Gene1418	unique	1362	[Rbromii5AMG]RB5AMG_01458_Endoglucanase_A_precursor	Rbromii5AMG
Org2_Gene1486	unique	3388	[Rbromii5AMG]RB5AMG_01526_hypothetical_protein	Rbromii5AMG
Org2_Gene1534	unique	1453	[Rbromii5AMG]RB5AMG_01574_General_stress_protein_A	Rbromii5AMG
Org2_Gene1535	unique	1178	[Rbromii5AMG]RB5AMG_01575_Putative_glycosyltransferase_EpsF	Rbromii5AMG
Org2_Gene1536	unique	1738	[Rbromii5AMG]RB5AMG_01576_Spore_coat_polysaccharide_biosynthesis_protein_SpsA	Rbromii5AMG
Org2_Gene1537	unique	906	[Rbromii5AMG]RB5AMG_01577_Lipid_A_core_-_O-antigen_ligase	Rbromii5AMG
Org2_Gene1538	unique	1951	[Rbromii5AMG]RB5AMG_01578_polysaccharide_pyruvyl_transferase_CsaB	Rbromii5AMG
Org2_Gene1539	unique	543	[Rbromii5AMG]RB5AMG_01579_putative_membrane_protein_EpsK	Rbromii5AMG
Org2_Gene1540	unique	1650	[Rbromii5AMG]RB5AMG_01580_Chondroitin_polymerase	Rbromii5AMG
Org2_Gene1541	unique	1267	[Rbromii5AMG]RB5AMG_01581_Polysaccharide_pyruvyl_transferase	Rbromii5AMG
Org2_Gene1542	unique	4375	[Rbromii5AMG]RB5AMG_01582_hypothetical_protein	Rbromii5AMG
Org2_Gene1543	unique	386	[Rbromii5AMG]RB5AMG_01583_2-succinyl-5-enolpyruvyl-6-hydroxy-3-cyclohexene-1-carboxylate_synthase	Rbromii5AMG
Org2_Gene1544	unique	1063	[Rbromii5AMG]RB5AMG_01584_UDP-glucose_6-dehydrogenase	Rbromii5AMG
Org2_Gene1545	unique	1139	[Rbromii5AMG]RB5AMG_01585_hypothetical_protein	Rbromii5AMG
Org2_Gene1570	unique	2315	[Rbromii5AMG]RB5AMG_01610_hypothetical_protein	Rbromii5AMG
Org2_Gene159	unique	3876	[Rbromii5AMG]RB5AMG_00169_hypothetical_protein	Rbromii5AMG
Org2_Gene160	unique	2247	[Rbromii5AMG]RB5AMG_00170_Capsular_polysaccharide_type_8_biosynthesis_protein_cap8A	Rbromii5AMG
Org2_Gene1606	unique	4280	[Rbromii5AMG]RB5AMG_01648_addiction_module_antitoxin,_RelB/DinJ_family	Rbromii5AMG
Org2_Gene1609	unique	4088	[Rbromii5AMG]RB5AMG_01651_hypothetical_protein	Rbromii5AMG
Org2_Gene161	unique	2302	[Rbromii5AMG]RB5AMG_00171_Tyrosine-protein_kinase_YwqD	Rbromii5AMG
Org2_Gene1610	unique	1301	[Rbromii5AMG]RB5AMG_01652_Beta-glucanase_precursor	Rbromii5AMG
Org2_Gene162	unique	2369	[Rbromii5AMG]RB5AMG_00172_Undecaprenyl_phosphate_N,N'-diacetylbacillosamine_1-phosphate_transferase	Rbromii5AMG
Org2_Gene1625	unique	2822	[Rbromii5AMG]RB5AMG_01667_RNA_polymerase_sigma_factor	Rbromii5AMG
Org2_Gene1626	unique	2503	[Rbromii5AMG]RB5AMG_01668_hypothetical_protein	Rbromii5AMG
Org2_Gene1627	unique	1134	[Rbromii5AMG]RB5AMG_01669_macrolide_transporter_ATP-binding_/permease_protein	Rbromii5AMG
Org2_Gene1628	unique	3429	[Rbromii5AMG]RB5AMG_01670_hypothetical_protein	Rbromii5AMG
Org2_Gene163	unique	4421	[Rbromii5AMG]RB5AMG_00173_hypothetical_protein	Rbromii5AMG
Org2_Gene1631	unique	222	[Rbromii5AMG]RB5AMG_01673_Lactococcin-G-processing_and_transport_ATP-binding_protein_LagD	Rbromii5AMG
Org2_Gene1632	unique	829	[Rbromii5AMG]RB5AMG_01674_Maltose-binding_periplasmic_proteins/domains	Rbromii5AMG
Org2_Gene1633	unique	4005	[Rbromii5AMG]RB5AMG_01675_hypothetical_protein	Rbromii5AMG
Org2_Gene1634	unique	376	[Rbromii5AMG]RB5AMG_01676_hypothetical_protein	Rbromii5AMG
Org2_Gene1635	unique	2719	[Rbromii5AMG]RB5AMG_01677_putative_accessory_gene_regulator_protein	Rbromii5AMG
Org2_Gene1642	unique	3472	[Rbromii5AMG]RB5AMG_01684_hypothetical_protein	Rbromii5AMG
Org2_Gene1644	unique	3390	[Rbromii5AMG]RB5AMG_01686_hypothetical_protein	Rbromii5AMG
Org2_Gene165	unique	1722	[Rbromii5AMG]RB5AMG_00175_Glucose-1-phosphate_thymidylyltransferase	Rbromii5AMG
Org2_Gene1650	unique	1568	[Rbromii5AMG]RB5AMG_01692_hypothetical_protein	Rbromii5AMG
Org2_Gene1659	unique	2179	[Rbromii5AMG]RB5AMG_01701_hypothetical_protein	Rbromii5AMG
Org2_Gene166	unique	1297	[Rbromii5AMG]RB5AMG_00176_dTDP-glucose_4,6-dehydratase	Rbromii5AMG
Org2_Gene1660	unique	2921	[Rbromii5AMG]RB5AMG_01702_Cd1	Rbromii5AMG
Org2_Gene167	unique	1653	[Rbromii5AMG]RB5AMG_00177_dTDP-4-dehydrorhamnose_reductase	Rbromii5AMG
Org2_Gene169	unique	1164	[Rbromii5AMG]RB5AMG_00179_Putative_glycosyltransferase_EpsD	Rbromii5AMG
Org2_Gene170	unique	1177	[Rbromii5AMG]RB5AMG_00180_Polysaccharide_pyruvyl_transferase	Rbromii5AMG
Org2_Gene171	unique	1103	[Rbromii5AMG]RB5AMG_00181_ferredoxin	Rbromii5AMG
Org2_Gene172	unique	1030	[Rbromii5AMG]RB5AMG_00182_GDP-mannose-dependent_alpha-mannosyltransferase	Rbromii5AMG
Org2_Gene1724	unique	3705	[Rbromii5AMG]RB5AMG_01769_hypothetical_protein	Rbromii5AMG
Org2_Gene173	unique	1723	[Rbromii5AMG]RB5AMG_00183_rhamnosyltransferase	Rbromii5AMG
Org2_Gene174	unique	1856	[Rbromii5AMG]RB5AMG_00184_Putative_teichuronic_acid_biosynthesis_glycosyltransferase_TuaG	Rbromii5AMG
Org2_Gene1740	unique	91	[Rbromii5AMG]RB5AMG_01785_Exoglucanase_B_precursor	Rbromii5AMG
Org2_Gene1741	unique	4052	[Rbromii5AMG]RB5AMG_01786_hypothetical_protein	Rbromii5AMG
Org2_Gene175	unique	1437	[Rbromii5AMG]RB5AMG_00185_hypothetical_protein	Rbromii5AMG
Org2_Gene176	unique	1218	[Rbromii5AMG]RB5AMG_00186_hypothetical_protein	Rbromii5AMG
Org2_Gene177	unique	640	[Rbromii5AMG]RB5AMG_00187_Putative_O-antigen_transporter	Rbromii5AMG
Org2_Gene178	unique	3210	[Rbromii5AMG]RB5AMG_00188_2-dehydropantoate_2-reductase	Rbromii5AMG
Org2_Gene1789	unique	2983	[Rbromii5AMG]RB5AMG_01838_hypothetical_protein	Rbromii5AMG
Org2_Gene179	unique	2239	[Rbromii5AMG]RB5AMG_00189_Chromosomal_replication_initiator_protein_DnaA	Rbromii5AMG
Org2_Gene180	unique	4429	[Rbromii5AMG]RB5AMG_00190_hypothetical_protein	Rbromii5AMG
Org2_Gene181	unique	2488	[Rbromii5AMG]RB5AMG_00191_hypothetical_protein	Rbromii5AMG
Org2_Gene182	unique	2475	[Rbromii5AMG]RB5AMG_00192_putative_transposase_OrfB	Rbromii5AMG
Org2_Gene183	unique	1697	[Rbromii5AMG]RB5AMG_00193_UDP-N-acetylglucosamine_diphosphorylase/glucosamine-1-phosphate_N-acetyltransferase	Rbromii5AMG
Org2_Gene184	unique	3673	[Rbromii5AMG]RB5AMG_00194_VanZ_like_family_protein	Rbromii5AMG
Org2_Gene1848	unique	4340	[Rbromii5AMG]RB5AMG_01898_hypothetical_protein	Rbromii5AMG
Org2_Gene185	unique	390	[Rbromii5AMG]RB5AMG_00195_hypothetical_protein	Rbromii5AMG
Org2_Gene186	unique	3827	[Rbromii5AMG]RB5AMG_00196_hypothetical_protein	Rbromii5AMG
Org2_Gene1861	unique	4087	[Rbromii5AMG]RB5AMG_01913_hypothetical_protein	Rbromii5AMG
Org2_Gene1868	unique	1675	[Rbromii5AMG]RB5AMG_01920_hypothetical_protein	Rbromii5AMG
Org2_Gene1869	unique	1652	[Rbromii5AMG]RB5AMG_01921_hypothetical_protein	Rbromii5AMG
Org2_Gene1873	unique	599	[Rbromii5AMG]RB5AMG_01925_Fimbrial_subunit_type_1_precursor	Rbromii5AMG
Org2_Gene1878	unique	2923	[Rbromii5AMG]RB5AMG_01930_hypothetical_protein	Rbromii5AMG
Org2_Gene188	unique	3680	[Rbromii5AMG]RB5AMG_00198_hypothetical_protein	Rbromii5AMG
Org2_Gene1893	unique	4086	[Rbromii5AMG]RB5AMG_01945_YgiT-type_zinc_finger_domain_protein	Rbromii5AMG
Org2_Gene1894	unique	3767	[Rbromii5AMG]RB5AMG_01946_hypothetical_protein	Rbromii5AMG
Org2_Gene1910	unique	4004	[Rbromii5AMG]RB5AMG_01963_hypothetical_protein	Rbromii5AMG
Org2_Gene1956	unique	1260	[Rbromii5AMG]RB5AMG_02011_D-alanine--D-alanine_ligase	Rbromii5AMG
Org2_Gene1957	unique	183	[Rbromii5AMG]RB5AMG_02012_hypothetical_protein	Rbromii5AMG
Org2_Gene1958	unique	233	[Rbromii5AMG]RB5AMG_02013_hypothetical_protein	Rbromii5AMG
Org2_Gene1959	unique	3804	[Rbromii5AMG]RB5AMG_02014_hypothetical_protein	Rbromii5AMG
Org2_Gene1960	unique	2747	[Rbromii5AMG]RB5AMG_02015_hypothetical_protein	Rbromii5AMG
Org2_Gene1961	unique	87	[Rbromii5AMG]RB5AMG_02016_Calcineurin-like_phosphoesterase	Rbromii5AMG
Org2_Gene1969	unique	703	[Rbromii5AMG]RB5AMG_02024_hypothetical_protein	Rbromii5AMG
Org2_Gene1985	unique	2367	[Rbromii5AMG]RB5AMG_02041_thymidylate_kinase	Rbromii5AMG
Org2_Gene1986	unique	152	[Rbromii5AMG]RB5AMG_02042_hypothetical_protein	Rbromii5AMG
Org2_Gene1987	unique	1841	[Rbromii5AMG]RB5AMG_02043_hypothetical_protein	Rbromii5AMG
Org2_Gene1988	unique	1339	[Rbromii5AMG]RB5AMG_02044_hypothetical_protein	Rbromii5AMG
Org2_Gene1989	unique	595	[Rbromii5AMG]RB5AMG_02045_hypothetical_protein	Rbromii5AMG
Org2_Gene1990	unique	3874	[Rbromii5AMG]RB5AMG_02047_Transposase	Rbromii5AMG
Org2_Gene1994	unique	2263	[Rbromii5AMG]RB5AMG_02051_hypothetical_protein	Rbromii5AMG
Org2_Gene1995	unique	4368	[Rbromii5AMG]RB5AMG_02052_hypothetical_protein	Rbromii5AMG
Org2_Gene2017	unique	4394	[Rbromii5AMG]RB5AMG_02074_hypothetical_protein	Rbromii5AMG
Org2_Gene2018	unique	4357	[Rbromii5AMG]RB5AMG_02075_hypothetical_protein	Rbromii5AMG
Org2_Gene2019	unique	4242	[Rbromii5AMG]RB5AMG_02076_hypothetical_protein	Rbromii5AMG
Org2_Gene2021	unique	3723	[Rbromii5AMG]RB5AMG_02078_hypothetical_protein	Rbromii5AMG
Org2_Gene2022	unique	3011	[Rbromii5AMG]RB5AMG_02079_hypothetical_protein	Rbromii5AMG
Org2_Gene2023	unique	3676	[Rbromii5AMG]RB5AMG_02080_hypothetical_protein	Rbromii5AMG
Org2_Gene2024	unique	501	[Rbromii5AMG]RB5AMG_02081_Superfamily_II_helicase	Rbromii5AMG
Org2_Gene2025	unique	459	[Rbromii5AMG]RB5AMG_02082_Transposase	Rbromii5AMG
Org2_Gene2053	unique	4396	[Rbromii5AMG]RB5AMG_02116_hypothetical_protein	Rbromii5AMG
Org2_Gene2054	unique	3976	[Rbromii5AMG]RB5AMG_02117_hypothetical_protein	Rbromii5AMG
Org2_Gene2055	unique	2635	[Rbromii5AMG]RB5AMG_02118_hypothetical_protein	Rbromii5AMG
Org2_Gene2056	unique	1984	[Rbromii5AMG]RB5AMG_02119_hypothetical_protein	Rbromii5AMG
Org2_Gene2057	unique	1059	[Rbromii5AMG]RB5AMG_02120_ADP-ribosyl-[dinitrogen_reductase]_hydrolase	Rbromii5AMG
Org2_Gene2130	unique	38	[Rbromii5AMG]RB5AMG_02193_hypothetical_protein	Rbromii5AMG
Org2_Gene2134	unique	3331	[Rbromii5AMG]RB5AMG_02205_hypothetical_protein	Rbromii5AMG
Org2_Gene2142	unique	469	[Rbromii5AMG]RB5AMG_02213_Divergent_AAA_domain_protein	Rbromii5AMG
Org2_Gene2176	unique	2924	[Rbromii5AMG]RB5AMG_02247_Transposase	Rbromii5AMG
Org2_Gene2177	unique	355	[Rbromii5AMG]RB5AMG_02248_putative_membrane_protein	Rbromii5AMG
Org2_Gene2178	unique	802	[Rbromii5AMG]RB5AMG_02249_Putative_virion_core_protein_(lumpy_skin_disease_virus)	Rbromii5AMG
Org2_Gene2179	unique	1683	[Rbromii5AMG]RB5AMG_02250_Double_zinc_ribbon	Rbromii5AMG
Org2_Gene2180	unique	4156	[Rbromii5AMG]RB5AMG_02251_putative_transcriptional_regulator	Rbromii5AMG
Org2_Gene2181	unique	4244	[Rbromii5AMG]RB5AMG_02252_hypothetical_protein	Rbromii5AMG
Org2_Gene2182	unique	4095	[Rbromii5AMG]RB5AMG_02253_hypothetical_protein	Rbromii5AMG
Org2_Gene2183	unique	1567	[Rbromii5AMG]RB5AMG_02254_Uracil_DNA_glycosylase_superfamily_protein	Rbromii5AMG
Org2_Gene2184	unique	1234	[Rbromii5AMG]RB5AMG_02255_hypothetical_protein	Rbromii5AMG
Org2_Gene2185	unique	2115	[Rbromii5AMG]RB5AMG_02256_hypothetical_protein	Rbromii5AMG
Org2_Gene2186	unique	3232	[Rbromii5AMG]RB5AMG_02257_hypothetical_protein	Rbromii5AMG
Org2_Gene239	unique	4238	[Rbromii5AMG]RB5AMG_00251_hypothetical_protein	Rbromii5AMG
Org2_Gene252	unique	3083	[Rbromii5AMG]RB5AMG_00264_hypothetical_protein	Rbromii5AMG
Org2_Gene264	unique	3254	[Rbromii5AMG]RB5AMG_00276_Phosphatase_NudJ	Rbromii5AMG
Org2_Gene265	unique	3476	[Rbromii5AMG]RB5AMG_00277_hypothetical_protein	Rbromii5AMG
Org2_Gene266	unique	209	[Rbromii5AMG]RB5AMG_00278_hypothetical_protein	Rbromii5AMG
Org2_Gene267	unique	4124	[Rbromii5AMG]RB5AMG_00279_transcriptional_regulator,_y4mF_family	Rbromii5AMG
Org2_Gene268	unique	1760	[Rbromii5AMG]RB5AMG_00280_hypothetical_protein	Rbromii5AMG
Org2_Gene269	unique	1466	[Rbromii5AMG]RB5AMG_00281_Modification_methylase_HindIII	Rbromii5AMG

Org2_Gene270	unique	4193	[Rbromii5AMG]RB5AMG_00282_putative_transcriptional_regulator	Rbromii5AMG
Org2_Gene275	unique	1654	[Rbromii5AMG]RB5AMG_00287_Chromosome-partitioning_protein_ParB	Rbromii5AMG
Org2_Gene276	unique	2639	[Rbromii5AMG]RB5AMG_00288_hypothetical_protein	Rbromii5AMG
Org2_Gene277	unique	3964	[Rbromii5AMG]RB5AMG_00289_hypothetical_protein	Rbromii5AMG
Org2_Gene278	unique	3050	[Rbromii5AMG]RB5AMG_00290_hypothetical_protein	Rbromii5AMG
Org2_Gene279	unique	2104	[Rbromii5AMG]RB5AMG_00291_PUA_domain_(predicted_RNA-binding_domain)	Rbromii5AMG
Org2_Gene280	unique	1918	[Rbromii5AMG]RB5AMG_00292_hypothetical_protein	Rbromii5AMG
Org2_Gene281	unique	3651	[Rbromii5AMG]RB5AMG_00293_hypothetical_protein	Rbromii5AMG
Org2_Gene282	unique	3157	[Rbromii5AMG]RB5AMG_00294_hypothetical_protein	Rbromii5AMG
Org2_Gene283	unique	374	[Rbromii5AMG]RB5AMG_00295_conjugal_transfer_coupling_protein_TraG	Rbromii5AMG
Org2_Gene285	unique	1806	[Rbromii5AMG]RB5AMG_00297_hypothetical_protein	Rbromii5AMG
Org2_Gene286	unique	3300	[Rbromii5AMG]RB5AMG_00298_hypothetical_protein	Rbromii5AMG
Org2_Gene288	unique	423	[Rbromii5AMG]RB5AMG_00300_putative_endopeptidase_p60_precursor	Rbromii5AMG
Org2_Gene289	unique	4068	[Rbromii5AMG]RB5AMG_00301_hypothetical_protein	Rbromii5AMG
Org2_Gene290	unique	1359	[Rbromii5AMG]RB5AMG_00302_Bacteriocin_BCN5	Rbromii5AMG
Org2_Gene291	unique	4286	[Rbromii5AMG]RB5AMG_00303_hypothetical_protein	Rbromii5AMG
Org2_Gene292	unique	268	[Rbromii5AMG]RB5AMG_00304_DNA_topoisomerase_3	Rbromii5AMG
Org2_Gene293	unique	3019	[Rbromii5AMG]RB5AMG_00305_hypothetical_protein	Rbromii5AMG
Org2_Gene294	unique	217	[Rbromii5AMG]RB5AMG_00306_Antirestriction_protein	Rbromii5AMG
Org2_Gene295	unique	4092	[Rbromii5AMG]RB5AMG_00307_hypothetical_protein	Rbromii5AMG
Org2_Gene296	unique	5	[Rbromii5AMG]RB5AMG_00308_DNA_mismatch_repair_protein_MutS	Rbromii5AMG
Org2_Gene298	unique	1564	[Rbromii5AMG]RB5AMG_00310_D-alanyl-D-alanine_carboxypeptidase_precursor	Rbromii5AMG
Org2_Gene301	unique	436	[Rbromii5AMG]RB5AMG_00313_DNA_methylase	Rbromii5AMG
Org2_Gene302	unique	3069	[Rbromii5AMG]RB5AMG_00314_hypothetical_protein	Rbromii5AMG
Org2_Gene303	unique	3917	[Rbromii5AMG]RB5AMG_00315_hypothetical_protein	Rbromii5AMG
Org2_Gene304	unique	4380	[Rbromii5AMG]RB5AMG_00316_hypothetical_protein	Rbromii5AMG
Org2_Gene305	unique	3882	[Rbromii5AMG]RB5AMG_00317_hypothetical_protein	Rbromii5AMG
Org2_Gene306	unique	658	[Rbromii5AMG]RB5AMG_00318_Relaxase/Mobilisation_nuclease_domain_protein	Rbromii5AMG
Org2_Gene307	unique	2739	[Rbromii5AMG]RB5AMG_00319_hypothetical_protein	Rbromii5AMG
Org2_Gene308	unique	2285	[Rbromii5AMG]RB5AMG_00320_cobalt_ABC_transporter,_permease_protein_CbiQ	Rbromii5AMG
Org2_Gene309	unique	619	[Rbromii5AMG]RB5AMG_00321_Putative_HMP/thiamine_import_ATP-binding_protein_YkoD	Rbromii5AMG
Org2_Gene310	unique	1535	[Rbromii5AMG]RB5AMG_00322_Regulatory_protein_PchR	Rbromii5AMG
Org2_Gene311	unique	1271	[Rbromii5AMG]RB5AMG_00323_Putative_multidrug_export_ATP-binding/permease_protein	Rbromii5AMG
Org2_Gene330	unique	963	[Rbromii5AMG]RB5AMG_00343_Integrase	Rbromii5AMG
Org2_Gene331	unique	4019	[Rbromii5AMG]RB5AMG_00344_Excisionase_from_transposon_Tn916	Rbromii5AMG
Org2_Gene332	unique	4079	[Rbromii5AMG]RB5AMG_00345_DNA_binding_domain,_excisionase_family	Rbromii5AMG
Org2_Gene333	unique	4094	[Rbromii5AMG]RB5AMG_00346_Excisionase_from_transposon_Tn916	Rbromii5AMG
Org2_Gene334	unique	4139	[Rbromii5AMG]RB5AMG_00347_Excisionase_from_transposon_Tn916	Rbromii5AMG
Org2_Gene335	unique	2846	[Rbromii5AMG]RB5AMG_00348_mRNA_interferase_EndoA	Rbromii5AMG
Org2_Gene336	unique	2752	[Rbromii5AMG]RB5AMG_00349_hypothetical_protein	Rbromii5AMG
Org2_Gene337	unique	2231	[Rbromii5AMG]RB5AMG_00350_hypothetical_protein	Rbromii5AMG
Org2_Gene338	unique	4023	[Rbromii5AMG]RB5AMG_00351_hypothetical_protein	Rbromii5AMG
Org2_Gene339	unique	3841	[Rbromii5AMG]RB5AMG_00352_hypothetical_protein	Rbromii5AMG
Org2_Gene340	unique	962	[Rbromii5AMG]RB5AMG_00353_DNA_polymerase_IV	Rbromii5AMG
Org2_Gene341	unique	3427	[Rbromii5AMG]RB5AMG_00354_transcriptional_repressor_DicA	Rbromii5AMG
Org2_Gene342	unique	2948	[Rbromii5AMG]RB5AMG_00355_Metallopeptidase_ImmA	Rbromii5AMG
Org2_Gene343	unique	1621	[Rbromii5AMG]RB5AMG_00356_Thioredoxin_reductase	Rbromii5AMG
Org2_Gene344	unique	3780	[Rbromii5AMG]RB5AMG_00357_Thioredoxin	Rbromii5AMG
Org2_Gene345	unique	2515	[Rbromii5AMG]RB5AMG_00358_Global_nitrogen_regulator	Rbromii5AMG
Org2_Gene346	unique	437	[Rbromii5AMG]RB5AMG_00359_Coenzyme_A_disulfide_reductase	Rbromii5AMG
Org2_Gene347	unique	3770	[Rbromii5AMG]RB5AMG_00360_Thiosulfate_sulfurtransferase_PspE_precursor	Rbromii5AMG
Org2_Gene348	unique	3825	[Rbromii5AMG]RB5AMG_00361_hypothetical_protein	Rbromii5AMG
Org2_Gene349	unique	2208	[Rbromii5AMG]RB5AMG_00362_Bile_acid_7-dehydroxylase_1/3	Rbromii5AMG
Org2_Gene350	unique	4377	[Rbromii5AMG]RB5AMG_00363_Rubredoxin	Rbromii5AMG
Org2_Gene351	unique	3547	[Rbromii5AMG]RB5AMG_00364_Desulfoferrodoxin	Rbromii5AMG
Org2_Gene352	unique	1637	[Rbromii5AMG]RB5AMG_00365_L-lactate_dehydrogenase	Rbromii5AMG
Org2_Gene354	unique	3431	[Rbromii5AMG]RB5AMG_00367_Peroxide-responsive_repressor_PerR	Rbromii5AMG
Org2_Gene355	unique	3813	[Rbromii5AMG]RB5AMG_00368_hypothetical_protein	Rbromii5AMG
Org2_Gene356	unique	1934	[Rbromii5AMG]RB5AMG_00369_methyltransferase	Rbromii5AMG
Org2_Gene357	unique	1655	[Rbromii5AMG]RB5AMG_00370_2-dehydropantoate_2-reductase	Rbromii5AMG
Org2_Gene358	unique	2618	[Rbromii5AMG]RB5AMG_00371_Demethylmenaquinone_methyltransferase	Rbromii5AMG
Org2_Gene359	unique	1461	[Rbromii5AMG]RB5AMG_00372_hypothetical_protein	Rbromii5AMG
Org2_Gene360	unique	3329	[Rbromii5AMG]RB5AMG_00373_hypothetical_protein	Rbromii5AMG
Org2_Gene362	unique	2652	[Rbromii5AMG]RB5AMG_00375_Demethylrebeccamycin-D-glucose_O-methyltransferase	Rbromii5AMG
Org2_Gene363	unique	562	[Rbromii5AMG]RB5AMG_00376_DNA_polymerase_IV	Rbromii5AMG
Org2_Gene364	unique	866	[Rbromii5AMG]RB5AMG_00377_EcoKI_restriction-modification_system_protein_HsdS	Rbromii5AMG
Org2_Gene365	unique	1467	[Rbromii5AMG]RB5AMG_00378_Tyrosine_recombinase_XerC	Rbromii5AMG
Org2_Gene366	unique	2750	[Rbromii5AMG]RB5AMG_00379_hypothetical_protein	Rbromii5AMG
Org2_Gene367	unique	2804	[Rbromii5AMG]RB5AMG_00380_Type_I_restriction_modification_DNA_specificity_domain_protein	Rbromii5AMG
Org2_Gene368	unique	484	[Rbromii5AMG]RB5AMG_00381_Type_I_restriction_enzyme_EcoKI_M_protein	Rbromii5AMG
Org2_Gene369	unique	70	[Rbromii5AMG]RB5AMG_00382_Type_I_restriction_enzyme_EcoR124II_R_protein	Rbromii5AMG
Org2_Gene370	unique	4197	[Rbromii5AMG]RB5AMG_00383_putative_transcriptional_regulator	Rbromii5AMG
Org2_Gene371	unique	4155	[Rbromii5AMG]RB5AMG_00384_hypothetical_protein	Rbromii5AMG
Org2_Gene372	unique	3686	[Rbromii5AMG]RB5AMG_00385_anaerobic_benzoate_catabolism_transcriptional_regulator	Rbromii5AMG
Org2_Gene373	unique	3256	[Rbromii5AMG]RB5AMG_00386_hypothetical_protein	Rbromii5AMG
Org2_Gene374	unique	407	[Rbromii5AMG]RB5AMG_00387_CHAP_domain_protein	Rbromii5AMG
Org2_Gene375	unique	3323	[Rbromii5AMG]RB5AMG_00388_hypothetical_protein	Rbromii5AMG
Org2_Gene376	unique	2155	[Rbromii5AMG]RB5AMG_00389_sortase,_SrtB_family	Rbromii5AMG
Org2_Gene377	unique	3454	[Rbromii5AMG]RB5AMG_00390_hypothetical_protein	Rbromii5AMG
Org2_Gene380	unique	3885	[Rbromii5AMG]RB5AMG_00393_hypothetical_protein	Rbromii5AMG
Org2_Gene381	unique	4344	[Rbromii5AMG]RB5AMG_00394_hypothetical_protein	Rbromii5AMG
Org2_Gene385	unique	4212	[Rbromii5AMG]RB5AMG_00398_hypothetical_protein	Rbromii5AMG
Org2_Gene386	unique	1751	[Rbromii5AMG]RB5AMG_00399_hypothetical_protein	Rbromii5AMG
Org2_Gene387	unique	2021	[Rbromii5AMG]RB5AMG_00400_hypothetical_protein	Rbromii5AMG
Org2_Gene388	unique	741	[Rbromii5AMG]RB5AMG_00401_Relaxase/Mobilisation_nuclease_domain_protein	Rbromii5AMG
Org2_Gene389	unique	3671	[Rbromii5AMG]RB5AMG_00402_Bacterial_mobilisation_protein_(MobC)	Rbromii5AMG
Org2_Gene390	unique	4373	[Rbromii5AMG]RB5AMG_00403_hypothetical_protein	Rbromii5AMG
Org2_Gene391	unique	3439	[Rbromii5AMG]RB5AMG_00404_hypothetical_protein	Rbromii5AMG
Org2_Gene392	unique	1539	[Rbromii5AMG]RB5AMG_00405_DNA_primase	Rbromii5AMG
Org2_Gene393	unique	4	[Rbromii5AMG]RB5AMG_00406_DNA_methylase	Rbromii5AMG
Org2_Gene394	unique	3908	[Rbromii5AMG]RB5AMG_00407_hypothetical_protein	Rbromii5AMG
Org2_Gene395	unique	4104	[Rbromii5AMG]RB5AMG_00408_hypothetical_protein	Rbromii5AMG
Org2_Gene396	unique	3872	[Rbromii5AMG]RB5AMG_00409_hypothetical_protein	Rbromii5AMG
Org2_Gene397	unique	4438	[Rbromii5AMG]RB5AMG_00410_hypothetical_protein	Rbromii5AMG
Org2_Gene398	unique	23	[Rbromii5AMG]RB5AMG_00411_putative_outer_membrane_protein	Rbromii5AMG
Org2_Gene399	unique	2440	[Rbromii5AMG]RB5AMG_00412_hypothetical_protein	Rbromii5AMG
Org2_Gene402	unique	2619	[Rbromii5AMG]RB5AMG_00415_Demethylmenaquinone_methyltransferase	Rbromii5AMG
Org2_Gene403	unique	2013	[Rbromii5AMG]RB5AMG_00416_methyltransferase	Rbromii5AMG
Org2_Gene404	unique	4349	[Rbromii5AMG]RB5AMG_00417_hypothetical_protein	Rbromii5AMG
Org2_Gene405	unique	706	[Rbromii5AMG]RB5AMG_00418_hypothetical_protein	Rbromii5AMG
Org2_Gene406	unique	3040	[Rbromii5AMG]RB5AMG_00419_transcriptional_repressor_DicA	Rbromii5AMG
Org2_Gene44	unique	3517	[Rbromii5AMG]RB5AMG_00045_Transcriptional_activator_protein_CopR	Rbromii5AMG
Org2_Gene45	unique	1158	[Rbromii5AMG]RB5AMG_00046_Cell_wall-associated_polypeptide_CWBP200	Rbromii5AMG
Org2_Gene455	unique	3294	[Rbromii5AMG]RB5AMG_00471_putative_HTH-type_transcriptional_regulator	Rbromii5AMG
Org2_Gene456	unique	3219	[Rbromii5AMG]RB5AMG_00472_hypothetical_protein	Rbromii5AMG
Org2_Gene462	unique	2040	[Rbromii5AMG]RB5AMG_00478_putative_transposase_OrfB	Rbromii5AMG
Org2_Gene470	unique	2005	[Rbromii5AMG]RB5AMG_00490_hypothetical_protein	Rbromii5AMG
Org2_Gene473	unique	2949	[Rbromii5AMG]RB5AMG_00493_hypothetical_protein	Rbromii5AMG
Org2_Gene475	unique	3368	[Rbromii5AMG]RB5AMG_00495_hypothetical_protein	Rbromii5AMG
Org2_Gene482	unique	2841	[Rbromii5AMG]RB5AMG_00502_hypothetical_protein	Rbromii5AMG
Org2_Gene497	unique	1851	[Rbromii5AMG]RB5AMG_00517_hypothetical_protein	Rbromii5AMG
Org2_Gene498	unique	197	[Rbromii5AMG]RB5AMG_00518_Papain_family_cysteine_protease	Rbromii5AMG
Org2_Gene512	unique	4160	[Rbromii5AMG]RB5AMG_00532_hypothetical_protein	Rbromii5AMG
Org2_Gene53	unique	1706	[Rbromii5AMG]RB5AMG_00055_hypothetical_protein	Rbromii5AMG
Org2_Gene532	unique	3143	[Rbromii5AMG]RB5AMG_00554_putative_methylated_DNA-protein_cysteine_methyltransferase	Rbromii5AMG
Org2_Gene533	unique	1354	[Rbromii5AMG]RB5AMG_00555_Virulence_protein	Rbromii5AMG
Org2_Gene534	unique	3308	[Rbromii5AMG]RB5AMG_00556_putative_acetyltransferase	Rbromii5AMG
Org2_Gene55	unique	4329	[Rbromii5AMG]RB5AMG_00057_Stage_V_sporulation_protein_T	Rbromii5AMG
Org2_Gene56	unique	3564	[Rbromii5AMG]RB5AMG_00058_hypothetical_protein	Rbromii5AMG
Org2_Gene57	unique	107	[Rbromii5AMG]RB5AMG_00059_putative_DNA_helicase	Rbromii5AMG
Org2_Gene58	unique	380	[Rbromii5AMG]RB5AMG_00060_hypothetical_protein	Rbromii5AMG
Org2_Gene59	unique	74	[Rbromii5AMG]RB5AMG_00061_Type-1_restriction_enzyme_R_protein	Rbromii5AMG

Org2_Gene60	unique	1854	[Rbromii5AMG]RB5AMG_00062_hypothetical_protein	Rbromii5AMG
Org2_Gene61	unique	1191	[Rbromii5AMG]RB5AMG_00063_putative_nucleotidyltransferases	Rbromii5AMG
Org2_Gene62	unique	527	[Rbromii5AMG]RB5AMG_00064_Type_I_restriction_enzyme_EcoKI_M_protein	Rbromii5AMG
Org2_Gene63	unique	1543	[Rbromii5AMG]RB5AMG_00065_Serine/threonine-protein_kinase_HipA	Rbromii5AMG
Org2_Gene636	unique	3592	[Rbromii5AMG]RB5AMG_00662_Phage-related_protein	Rbromii5AMG
Org2_Gene637	unique	3839	[Rbromii5AMG]RB5AMG_00663_DNA-binding_transcriptional_repressor_PuuR	Rbromii5AMG
Org2_Gene64	unique	3741	[Rbromii5AMG]RB5AMG_00066_Serine/threonine-protein_kinase_HipA	Rbromii5AMG
Org2_Gene65	unique	4203	[Rbromii5AMG]RB5AMG_00067_transcriptional_regulator,_y4mF_family	Rbromii5AMG
Org2_Gene66	unique	3681	[Rbromii5AMG]RB5AMG_00068_hypothetical_protein	Rbromii5AMG
Org2_Gene67	unique	4020	[Rbromii5AMG]RB5AMG_00069_hypothetical_protein	Rbromii5AMG
Org2_Gene688	unique	14	[Rbromii5AMG]RB5AMG_00714_hypothetical_protein	Rbromii5AMG
Org2_Gene689	unique	4407	[Rbromii5AMG]RB5AMG_00715_hypothetical_protein	Rbromii5AMG
Org2_Gene735	unique	2959	[Rbromii5AMG]RB5AMG_00763_Helix-turn-helix_domain_protein	Rbromii5AMG
Org2_Gene741	unique	3533	[Rbromii5AMG]RB5AMG_00769_hypothetical_protein	Rbromii5AMG
Org2_Gene742	unique	541	[Rbromii5AMG]RB5AMG_00770_putative_ABC_transporter_ATP-binding_protein_YheS	Rbromii5AMG
Org2_Gene743	unique	3070	[Rbromii5AMG]RB5AMG_00771_Lincosamide_resistance_protein	Rbromii5AMG
Org2_Gene747	unique	561	[Rbromii5AMG]RB5AMG_00775_Recombinase	Rbromii5AMG
Org2_Gene748	unique	464	[Rbromii5AMG]RB5AMG_00776_Recombinase	Rbromii5AMG
Org2_Gene749	unique	485	[Rbromii5AMG]RB5AMG_00777_hypothetical_protein	Rbromii5AMG
Org2_Gene750	unique	4114	[Rbromii5AMG]RB5AMG_00778_hypothetical_protein	Rbromii5AMG
Org2_Gene751	unique	3134	[Rbromii5AMG]RB5AMG_00779_hypothetical_protein	Rbromii5AMG
Org2_Gene752	unique	3746	[Rbromii5AMG]RB5AMG_00780_transcriptional_regulator,_y4mF_family	Rbromii5AMG
Org2_Gene753	unique	3566	[Rbromii5AMG]RB5AMG_00781_Manganese_transport_regulator	Rbromii5AMG
Org2_Gene754	unique	400	[Rbromii5AMG]RB5AMG_00782_Putative_multidrug_export_ATP-binding/permease_protein	Rbromii5AMG
Org2_Gene755	unique	1938	[Rbromii5AMG]RB5AMG_00783_Putative_multidrug_export_ATP-binding/permease_protein	Rbromii5AMG
Org2_Gene76	unique	2118	[Rbromii5AMG]RB5AMG_00078_Prolipoprotein_diacylglyceryl_transferase	Rbromii5AMG
Org2_Gene765	unique	4267	[Rbromii5AMG]RB5AMG_00793_hypothetical_protein	Rbromii5AMG
Org2_Gene77	unique	2818	[Rbromii5AMG]RB5AMG_00079_Chromate_transport_protein	Rbromii5AMG
Org2_Gene773	unique	4401	[Rbromii5AMG]RB5AMG_00801_hypothetical_protein	Rbromii5AMG
Org2_Gene78	unique	2792	[Rbromii5AMG]RB5AMG_00080_Chromate_transport_protein	Rbromii5AMG
Org2_Gene79	unique	4382	[Rbromii5AMG]RB5AMG_00085_hypothetical_protein	Rbromii5AMG
Org2_Gene8	unique	2816	[Rbromii5AMG]RB5AMG_00008_Cell_wall-associated_polypeptide_CWBP200	Rbromii5AMG
Org2_Gene850	unique	4012	[Rbromii5AMG]RB5AMG_00879_hypothetical_protein	Rbromii5AMG
Org2_Gene851	unique	1163	[Rbromii5AMG]RB5AMG_00880_Ribonuclease_3	Rbromii5AMG
Org2_Gene852	unique	4341	[Rbromii5AMG]RB5AMG_00881_hypothetical_protein	Rbromii5AMG
Org2_Gene853	unique	343	[Rbromii5AMG]RB5AMG_00882_putative_AAA-ATPase	Rbromii5AMG
Org2_Gene854	unique	65	[Rbromii5AMG]RB5AMG_00883_type_I_restriction_enzyme_EcoKI_subunit_R	Rbromii5AMG
Org2_Gene855	unique	672	[Rbromii5AMG]RB5AMG_00884_Type_I_restriction_enzyme_EcoKI_M_protein	Rbromii5AMG
Org2_Gene856	unique	689	[Rbromii5AMG]RB5AMG_00885_Type_I_restriction_enzyme_EcoKI_specificity_protein	Rbromii5AMG
Org2_Gene86	unique	1130	[Rbromii5AMG]RB5AMG_00092_hypothetical_protein	Rbromii5AMG
Org2_Gene881	unique	3340	[Rbromii5AMG]RB5AMG_00910_hypothetical_protein	Rbromii5AMG
Org2_Gene884	unique	3225	[Rbromii5AMG]RB5AMG_00913_hypothetical_protein	Rbromii5AMG
Org2_Gene885	unique	3216	[Rbromii5AMG]RB5AMG_00914_hypothetical_protein	Rbromii5AMG
Org2_Gene886	unique	278	[Rbromii5AMG]RB5AMG_00915_Stage_V_sporulation_protein_K	Rbromii5AMG
Org2_Gene887	unique	853	[Rbromii5AMG]RB5AMG_00916_hypothetical_protein	Rbromii5AMG
Org2_Gene888	unique	1206	[Rbromii5AMG]RB5AMG_00917_ATPase_family_associated_with_various_cellular_activities_(AAA)	Rbromii5AMG
Org2_Gene889	unique	53	[Rbromii5AMG]RB5AMG_00918_DNA_methylase	Rbromii5AMG
Org2_Gene9	unique	3066	[Rbromii5AMG]RB5AMG_00009_hypothetical_protein	Rbromii5AMG
Org2_Gene904	unique	391	[Rbromii5AMG]RB5AMG_00933_hypothetical_protein	Rbromii5AMG
Org2_Gene906	unique	3061	[Rbromii5AMG]RB5AMG_00935_hypothetical_protein	Rbromii5AMG
Org2_Gene941	unique	505	[Rbromii5AMG]RB5AMG_00971_hypothetical_protein	Rbromii5AMG
Org2_Gene96	unique	2125	[Rbromii5AMG]RB5AMG_00102_hypothetical_protein	Rbromii5AMG
Org2_Gene963	unique	3548	[Rbromii5AMG]RB5AMG_00994_hypothetical_protein	Rbromii5AMG
Org2_Gene98	unique	2545	[Rbromii5AMG]RB5AMG_00104_hypothetical_protein	Rbromii5AMG
Org2_Gene982	unique	4430	[Rbromii5AMG]RB5AMG_01013_hypothetical_protein	Rbromii5AMG
Org3_Gene1	unique	617	[RbromiiATCC27255]RBATCC27255_00001_Divergent_AAA_domain_protein	RbromiiATCC27255
Org3_Gene10	unique	3845	[RbromiiATCC27255]RBATCC27255_00012_hypothetical_protein	RbromiiATCC27255
Org3_Gene1015	unique	3643	[RbromiiATCC27255]RBATCC27255_01043_Cupin_domain_protein	RbromiiATCC27255
Org3_Gene1026	unique	4395	[RbromiiATCC27255]RBATCC27255_01054_hypothetical_protein	RbromiiATCC27255
Org3_Gene1027	unique	3633	[RbromiiATCC27255]RBATCC27255_01055_hypothetical_protein	RbromiiATCC27255
Org3_Gene1031	unique	358	[RbromiiATCC27255]RBATCC27255_01060_putative_ATP-binding_protein_involved_in_virulence	RbromiiATCC27255
Org3_Gene1032	unique	2200	[RbromiiATCC27255]RBATCC27255_01061_Modification_methylase_DpnIIB	RbromiiATCC27255
Org3_Gene1033	unique	1878	[RbromiiATCC27255]RBATCC27255_01062_Restriction_endonuclease	RbromiiATCC27255
Org3_Gene1034	unique	1767	[RbromiiATCC27255]RBATCC27255_01063_Type-2_restriction_enzyme_DpnII	RbromiiATCC27255
Org3_Gene1035	unique	2942	[RbromiiATCC27255]RBATCC27255_01064_hypothetical_protein	RbromiiATCC27255
Org3_Gene1036	unique	4315	[RbromiiATCC27255]RBATCC27255_01065_hypothetical_protein	RbromiiATCC27255
Org3_Gene1041	unique	1472	[RbromiiATCC27255]RBATCC27255_01070_putative_membrane_protein	RbromiiATCC27255
Org3_Gene1042	unique	2460	[RbromiiATCC27255]RBATCC27255_01071_putative_membrane_protein	RbromiiATCC27255
Org3_Gene1043	unique	86	[RbromiiATCC27255]RBATCC27255_01072_cobaltochelatase_subunit	RbromiiATCC27255
Org3_Gene1044	unique	1181	[RbromiiATCC27255]RBATCC27255_01073_hypothetical_protein	RbromiiATCC27255
Org3_Gene1045	unique	625	[RbromiiATCC27255]RBATCC27255_01074_hypothetical_protein	RbromiiATCC27255
Org3_Gene1046	unique	101	[RbromiiATCC27255]RBATCC27255_01075_hypothetical_protein	RbromiiATCC27255
Org3_Gene1047	unique	3409	[RbromiiATCC27255]RBATCC27255_01076_hypothetical_protein	RbromiiATCC27255
Org3_Gene1048	unique	2202	[RbromiiATCC27255]RBATCC27255_01077_Double_zinc_ribbon	RbromiiATCC27255
Org3_Gene1049	unique	1240	[RbromiiATCC27255]RBATCC27255_01078_hypothetical_protein	RbromiiATCC27255
Org3_Gene1050	unique	954	[RbromiiATCC27255]RBATCC27255_01080_hypothetical_protein	RbromiiATCC27255
Org3_Gene1051	unique	2745	[RbromiiATCC27255]RBATCC27255_01081_hypothetical_protein	RbromiiATCC27255
Org3_Gene1064	unique	1631	[RbromiiATCC27255]RBATCC27255_01099_hypothetical_protein	RbromiiATCC27255
Org3_Gene1067	unique	178	[RbromiiATCC27255]RBATCC27255_01102_ABC-type_transport_system_involved_in_multi-copper_enzyme_maturation,_permease_component	RbromiiATCC27255
Org3_Gene1068	unique	900	[RbromiiATCC27255]RBATCC27255_01103_hypothetical_protein	RbromiiATCC27255
Org3_Gene1081	unique	157	[RbromiiATCC27255]RBATCC27255_01116_ABC-type_transport_system_involved_in_multi-copper_enzyme_maturation,_permease_component	RbromiiATCC27255
Org3_Gene1082	unique	429	[RbromiiATCC27255]RBATCC27255_01117_hypothetical_protein	RbromiiATCC27255
Org3_Gene1083	unique	3207	[RbromiiATCC27255]RBATCC27255_01118_hypothetical_protein	RbromiiATCC27255
Org3_Gene1084	unique	4351	[RbromiiATCC27255]RBATCC27255_01119_cyclic_lactone_autoinducer_peptide	RbromiiATCC27255
Org3_Gene1085	unique	4287	[RbromiiATCC27255]RBATCC27255_01120_hypothetical_protein	RbromiiATCC27255
Org3_Gene1086	unique	4363	[RbromiiATCC27255]RBATCC27255_01121_hypothetical_protein	RbromiiATCC27255
Org3_Gene1087	unique	2741	[RbromiiATCC27255]RBATCC27255_01122_putative_accessory_gene_regulator_protein	RbromiiATCC27255
Org3_Gene1088	unique	806	[RbromiiATCC27255]RBATCC27255_01123_hypothetical_protein	RbromiiATCC27255
Org3_Gene1089	unique	2310	[RbromiiATCC27255]RBATCC27255_01124_two-component_response_regulator	RbromiiATCC27255
Org3_Gene1090	unique	4134	[RbromiiATCC27255]RBATCC27255_01125_hypothetical_protein	RbromiiATCC27255
Org3_Gene1095	unique	3691	[RbromiiATCC27255]RBATCC27255_01130_hypothetical_protein	RbromiiATCC27255
Org3_Gene1097	unique	4265	[RbromiiATCC27255]RBATCC27255_01132_hypothetical_protein	RbromiiATCC27255
Org3_Gene1111	unique	3349	[RbromiiATCC27255]RBATCC27255_01146_hypothetical_protein	RbromiiATCC27255
Org3_Gene1145	unique	3002	[RbromiiATCC27255]RBATCC27255_01180_hypothetical_protein	RbromiiATCC27255
Org3_Gene1146	unique	3017	[RbromiiATCC27255]RBATCC27255_01181_hypothetical_protein	RbromiiATCC27255
Org3_Gene1158	unique	2282	[RbromiiATCC27255]RBATCC27255_01193_hypothetical_protein	RbromiiATCC27255
Org3_Gene1159	unique	2947	[RbromiiATCC27255]RBATCC27255_01194_hypothetical_protein	RbromiiATCC27255
Org3_Gene1160	unique	1976	[RbromiiATCC27255]RBATCC27255_01195_hypothetical_protein	RbromiiATCC27255
Org3_Gene1166	unique	2775	[RbromiiATCC27255]RBATCC27255_01201_hypothetical_protein	RbromiiATCC27255
Org3_Gene1178	unique	2533	[RbromiiATCC27255]RBATCC27255_01214_hypothetical_protein	RbromiiATCC27255
Org3_Gene1179	unique	483	[RbromiiATCC27255]RBATCC27255_01215_Cell_wall-associated_polypeptide_CWBP200	RbromiiATCC27255
Org3_Gene1180	unique	3493	[RbromiiATCC27255]RBATCC27255_01216_hypothetical_protein	RbromiiATCC27255
Org3_Gene1182	unique	4293	[RbromiiATCC27255]RBATCC27255_01218_hypothetical_protein	RbromiiATCC27255
Org3_Gene1190	unique	2967	[RbromiiATCC27255]RBATCC27255_01226_Helix-turn-helix_domain_protein	RbromiiATCC27255
Org3_Gene1191	unique	2805	[RbromiiATCC27255]RBATCC27255_01227_hypothetical_protein	RbromiiATCC27255
Org3_Gene1192	unique	2670	[RbromiiATCC27255]RBATCC27255_01228_hypothetical_protein	RbromiiATCC27255
Org3_Gene1194	unique	3073	[RbromiiATCC27255]RBATCC27255_01230_hypothetical_protein	RbromiiATCC27255
Org3_Gene1195	unique	352	[RbromiiATCC27255]RBATCC27255_01231_putative_methyltransferase	RbromiiATCC27255
Org3_Gene1196	unique	214	[RbromiiATCC27255]RBATCC27255_01232_Restriction_endonuclease	RbromiiATCC27255
Org3_Gene1197	unique	1628	[RbromiiATCC27255]RBATCC27255_01233_hypothetical_protein	RbromiiATCC27255
Org3_Gene1198	unique	2916	[RbromiiATCC27255]RBATCC27255_01234_hypothetical_protein	RbromiiATCC27255
Org3_Gene1245	unique	747	[RbromiiATCC27255]RBATCC27255_01284_hypothetical_protein	RbromiiATCC27255
Org3_Gene13	unique	3288	[RbromiiATCC27255]RBATCC27255_00015_hypothetical_protein	RbromiiATCC27255
Org3_Gene1306	unique	4419	[RbromiiATCC27255]RBATCC27255_01349_hypothetical_protein	RbromiiATCC27255
Org3_Gene1307	unique	15	[RbromiiATCC27255]RBATCC27255_01350_hypothetical_protein	RbromiiATCC27255
Org3_Gene1320	unique	4249	[RbromiiATCC27255]RBATCC27255_01363_Recombination_directionality_factor_xis	RbromiiATCC27255
Org3_Gene1326	unique	608	[RbromiiATCC27255]RBATCC27255_01369_hypothetical_protein	RbromiiATCC27255
Org3_Gene1327	unique	3863	[RbromiiATCC27255]RBATCC27255_01370_Transposase	RbromiiATCC27255
Org3_Gene1329	unique	2769	[RbromiiATCC27255]RBATCC27255_01372_putative_transposase_OrfB	RbromiiATCC27255
Org3_Gene1330	unique	3923	[RbromiiATCC27255]RBATCC27255_01373_Transposase	RbromiiATCC27255
Org3_Gene1341	unique	3076	[RbromiiATCC27255]RBATCC27255_01384_hypothetical_protein	RbromiiATCC27255
Org3_Gene1343	unique	66	[RbromiiATCC27255]RBATCC27255_01386_hypothetical_protein	RbromiiATCC27255

Org3_Gene1344	unique	3588	[RbromiiATCC27255]RBATCC27255_01387_hypothetical_protein	RbromiiATCC27255
Org3_Gene1345	unique	3400	[RbromiiATCC27255]RBATCC27255_01388_hypothetical_protein	RbromiiATCC27255
Org3_Gene1346	unique	145	[RbromiiATCC27255]RBATCC27255_01389_hypothetical_protein	RbromiiATCC27255
Org3_Gene1347	unique	80	[RbromiiATCC27255]RBATCC27255_01390_Subtilisin_DY	RbromiiATCC27255
Org3_Gene1348	unique	1742	[RbromiiATCC27255]RBATCC27255_01391_hypothetical_protein	RbromiiATCC27255
Org3_Gene1353	unique	3985	[RbromiiATCC27255]RBATCC27255_01396_SPFH_domain_/_Band_7_family_protein	RbromiiATCC27255
Org3_Gene1355	unique	2413	[RbromiiATCC27255]RBATCC27255_01398_Sensory_transduction_protein_LytR	RbromiiATCC27255
Org3_Gene1357	unique	1860	[RbromiiATCC27255]RBATCC27255_01400_sensory_histidine_kinase_DcuS	RbromiiATCC27255
Org3_Gene1413	unique	2020	[RbromiiATCC27255]RBATCC27255_01459_hypothetical_protein	RbromiiATCC27255
Org3_Gene1486	unique	2183	[RbromiiATCC27255]RBATCC27255_01534_hypothetical_protein	RbromiiATCC27255
Org3_Gene1487	unique	2293	[RbromiiATCC27255]RBATCC27255_01535_type_VII_secretion_protein_EccCb	RbromiiATCC27255
Org3_Gene1488	unique	2008	[RbromiiATCC27255]RBATCC27255_01536_Helix-turn-helix	RbromiiATCC27255
Org3_Gene1489	unique	3660	[RbromiiATCC27255]RBATCC27255_01537_hypothetical_protein	RbromiiATCC27255
Org3_Gene1495	unique	721	[RbromiiATCC27255]RBATCC27255_01543_Exoglucanase_B_precursor	RbromiiATCC27255
Org3_Gene1499	unique	1109	[RbromiiATCC27255]RBATCC27255_01548_hypothetical_protein	RbromiiATCC27255
Org3_Gene1500	unique	463	[RbromiiATCC27255]RBATCC27255_01549_Bacterial_Ig-like_domain_(group_2)	RbromiiATCC27255
Org3_Gene1501	unique	221	[RbromiiATCC27255]RBATCC27255_01550_Fibronectin_type_III_domain_protein	RbromiiATCC27255
Org3_Gene1510	unique	3489	[RbromiiATCC27255]RBATCC27255_01559_hypothetical_protein	RbromiiATCC27255
Org3_Gene1511	unique	1162	[RbromiiATCC27255]RBATCC27255_01560_hypothetical_protein	RbromiiATCC27255
Org3_Gene156	unique	100	[RbromiiATCC27255]RBATCC27255_00161_hypothetical_protein	RbromiiATCC27255
Org3_Gene157	unique	404	[RbromiiATCC27255]RBATCC27255_00162_hypothetical_protein	RbromiiATCC27255
Org3_Gene1573	unique	3954	[RbromiiATCC27255]RBATCC27255_01623_hypothetical_protein	RbromiiATCC27255
Org3_Gene1574	unique	4140	[RbromiiATCC27255]RBATCC27255_01624_hypothetical_protein	RbromiiATCC27255
Org3_Gene1575	unique	3913	[RbromiiATCC27255]RBATCC27255_01625_hypothetical_protein	RbromiiATCC27255
Org3_Gene1587	unique	3084	[RbromiiATCC27255]RBATCC27255_01637_spore_cortex_biosynthesis_protein_YabQ	RbromiiATCC27255
Org3_Gene1592	unique	1566	[RbromiiATCC27255]RBATCC27255_01642_hypothetical_protein	RbromiiATCC27255
Org3_Gene1596	unique	3979	[RbromiiATCC27255]RBATCC27255_01646_Antitoxin_DinJ	RbromiiATCC27255
Org3_Gene1672	unique	1318	[RbromiiATCC27255]RBATCC27255_01722_hypothetical_protein	RbromiiATCC27255
Org3_Gene1708	unique	232	[RbromiiATCC27255]RBATCC27255_01759_hypothetical_protein	RbromiiATCC27255
Org3_Gene1709	unique	766	[RbromiiATCC27255]RBATCC27255_01760_Polysaccharide_biosynthesis_protein	RbromiiATCC27255
Org3_Gene1710	unique	984	[RbromiiATCC27255]RBATCC27255_01761_Lipid_A_core_-_O-antigen_ligase	RbromiiATCC27255
Org3_Gene1711	unique	179	[RbromiiATCC27255]RBATCC27255_01762_Ferredoxin	RbromiiATCC27255
Org3_Gene1712	unique	1174	[RbromiiATCC27255]RBATCC27255_01763_Putative_glycosyltransferase_EpsF	RbromiiATCC27255
Org3_Gene1713	unique	1113	[RbromiiATCC27255]RBATCC27255_01764_F420H2_dehydrogenase_subunit_F	RbromiiATCC27255
Org3_Gene1714	unique	1202	[RbromiiATCC27255]RBATCC27255_01765_Polysaccharide_pyruvyl_transferase	RbromiiATCC27255
Org3_Gene1734	unique	2751	[RbromiiATCC27255]RBATCC27255_01785_putative_adenosine_monophosphate-protein_transferase_fic	RbromiiATCC27255
Org3_Gene1740	unique	4330	[RbromiiATCC27255]RBATCC27255_01791_hypothetical_protein	RbromiiATCC27255
Org3_Gene1742	unique	3196	[RbromiiATCC27255]RBATCC27255_01793_hypothetical_protein	RbromiiATCC27255
Org3_Gene1743	unique	4048	[RbromiiATCC27255]RBATCC27255_01794_Helix-turn-helix_domain_protein	RbromiiATCC27255
Org3_Gene1744	unique	1219	[RbromiiATCC27255]RBATCC27255_01795_hypothetical_protein	RbromiiATCC27255
Org3_Gene1745	unique	349	[RbromiiATCC27255]RBATCC27255_01796_Type_IV_secretory_pathway,_VirB4_components	RbromiiATCC27255
Org3_Gene1746	unique	1175	[RbromiiATCC27255]RBATCC27255_01797_hypothetical_protein	RbromiiATCC27255
Org3_Gene1747	unique	1606	[RbromiiATCC27255]RBATCC27255_01798_Modification_methylase_HhaI	RbromiiATCC27255
Org3_Gene1748	unique	1137	[RbromiiATCC27255]RBATCC27255_01799_HpaII_restriction_endonuclease	RbromiiATCC27255
Org3_Gene1749	unique	1418	[RbromiiATCC27255]RBATCC27255_01800_Modification_methylase_HpaII	RbromiiATCC27255
Org3_Gene1750	unique	3052	[RbromiiATCC27255]RBATCC27255_01801_hypothetical_protein	RbromiiATCC27255
Org3_Gene1751	unique	3394	[RbromiiATCC27255]RBATCC27255_01802_Very_short_patch_repair_protein	RbromiiATCC27255
Org3_Gene1754	unique	2529	[RbromiiATCC27255]RBATCC27255_01805_ADP_compounds_hydrolase_NudE	RbromiiATCC27255
Org3_Gene1755	unique	753	[RbromiiATCC27255]RBATCC27255_01806_hypothetical_protein	RbromiiATCC27255
Org3_Gene1766	unique	2410	[RbromiiATCC27255]RBATCC27255_01818_putative_membrane_protein	RbromiiATCC27255
Org3_Gene1769	unique	1414	[RbromiiATCC27255]RBATCC27255_01821_hypothetical_protein	RbromiiATCC27255
Org3_Gene1800	unique	2579	[RbromiiATCC27255]RBATCC27255_01852_putative_membrane_protein	RbromiiATCC27255
Org3_Gene1812	unique	4149	[RbromiiATCC27255]RBATCC27255_01865_transcriptional_repressor_DicA	RbromiiATCC27255
Org3_Gene1813	unique	2034	[RbromiiATCC27255]RBATCC27255_01866_hypothetical_protein	RbromiiATCC27255
Org3_Gene1814	unique	3611	[RbromiiATCC27255]RBATCC27255_01867_hypothetical_protein	RbromiiATCC27255
Org3_Gene1815	unique	3358	[RbromiiATCC27255]RBATCC27255_01868_hypothetical_protein	RbromiiATCC27255
Org3_Gene184	unique	3852	[RbromiiATCC27255]RBATCC27255_00193_Group_II_intron-encoded_protein_LtrA	RbromiiATCC27255
Org3_Gene1849	unique	550	[RbromiiATCC27255]RBATCC27255_01902_hypothetical_protein	RbromiiATCC27255
Org3_Gene185	unique	3984	[RbromiiATCC27255]RBATCC27255_00196_hypothetical_protein	RbromiiATCC27255
Org3_Gene1850	unique	2995	[RbromiiATCC27255]RBATCC27255_01903_hypothetical_protein	RbromiiATCC27255
Org3_Gene186	unique	1562	[RbromiiATCC27255]RBATCC27255_00197_Integrase	RbromiiATCC27255
Org3_Gene187	unique	4096	[RbromiiATCC27255]RBATCC27255_00198_hypothetical_protein	RbromiiATCC27255
Org3_Gene188	unique	2305	[RbromiiATCC27255]RBATCC27255_00199_Recombinase	RbromiiATCC27255
Org3_Gene1882	unique	1374	[RbromiiATCC27255]RBATCC27255_01936_hypothetical_protein	RbromiiATCC27255
Org3_Gene1889	unique	227	[RbromiiATCC27255]RBATCC27255_01943_hypothetical_protein	RbromiiATCC27255
Org3_Gene189	unique	705	[RbromiiATCC27255]RBATCC27255_00200_Transposon_Tn3_resolvase	RbromiiATCC27255
Org3_Gene1891	unique	3285	[RbromiiATCC27255]RBATCC27255_01945_hypothetical_protein	RbromiiATCC27255
Org3_Gene190	unique	4076	[RbromiiATCC27255]RBATCC27255_00201_hypothetical_protein	RbromiiATCC27255
Org3_Gene1905	unique	521	[RbromiiATCC27255]RBATCC27255_01959_hypothetical_protein	RbromiiATCC27255
Org3_Gene1906	unique	1132	[RbromiiATCC27255]RBATCC27255_01960_hypothetical_protein	RbromiiATCC27255
Org3_Gene1907	unique	1773	[RbromiiATCC27255]RBATCC27255_01961_hypothetical_protein	RbromiiATCC27255
Org3_Gene1908	unique	46	[RbromiiATCC27255]RBATCC27255_01962_hypothetical_protein	RbromiiATCC27255
Org3_Gene191	unique	54	[RbromiiATCC27255]RBATCC27255_00202_PEGA_domain_protein	RbromiiATCC27255
Org3_Gene192	unique	3387	[RbromiiATCC27255]RBATCC27255_00203_hypothetical_protein	RbromiiATCC27255
Org3_Gene193	unique	1036	[RbromiiATCC27255]RBATCC27255_00204_hypothetical_protein	RbromiiATCC27255
Org3_Gene194	unique	2151	[RbromiiATCC27255]RBATCC27255_00205_hypothetical_protein	RbromiiATCC27255
Org3_Gene1944	unique	2944	[RbromiiATCC27255]RBATCC27255_02004_Modification_methylase_DpnIIB	RbromiiATCC27255
Org3_Gene1946	unique	3953	[RbromiiATCC27255]RBATCC27255_02006_hypothetical_protein	RbromiiATCC27255
Org3_Gene1947	unique	4165	[RbromiiATCC27255]RBATCC27255_02007_hypothetical_protein	RbromiiATCC27255
Org3_Gene1948	unique	3766	[RbromiiATCC27255]RBATCC27255_02008_hypothetical_protein	RbromiiATCC27255
Org3_Gene1949	unique	3477	[RbromiiATCC27255]RBATCC27255_02009_hypothetical_protein	RbromiiATCC27255
Org3_Gene195	unique	1082	[RbromiiATCC27255]RBATCC27255_00206_Clostripain_family_protein	RbromiiATCC27255
Org3_Gene196	unique	812	[RbromiiATCC27255]RBATCC27255_00207_Sensor_histidine_kinase_YpdA	RbromiiATCC27255
Org3_Gene197	unique	2064	[RbromiiATCC27255]RBATCC27255_00208_Sensory_transduction_protein_LytR	RbromiiATCC27255
Org3_Gene198	unique	3664	[RbromiiATCC27255]RBATCC27255_00209_hypothetical_protein	RbromiiATCC27255
Org3_Gene199	unique	241	[RbromiiATCC27255]RBATCC27255_00210_hypothetical_protein	RbromiiATCC27255
Org3_Gene200	unique	837	[RbromiiATCC27255]RBATCC27255_00211_hypothetical_protein	RbromiiATCC27255
Org3_Gene201	unique	4262	[RbromiiATCC27255]RBATCC27255_00212_hypothetical_protein	RbromiiATCC27255
Org3_Gene202	unique	1477	[RbromiiATCC27255]RBATCC27255_00213_hypothetical_protein	RbromiiATCC27255
Org3_Gene204	unique	2664	[RbromiiATCC27255]RBATCC27255_00215_hypothetical_protein	RbromiiATCC27255
Org3_Gene205	unique	1173	[RbromiiATCC27255]RBATCC27255_00216_Cell_wall-associated_polypeptide_CWBP200	RbromiiATCC27255
Org3_Gene206	unique	1148	[RbromiiATCC27255]RBATCC27255_00218_hypothetical_protein	RbromiiATCC27255
Org3_Gene24	unique	3716	[RbromiiATCC27255]RBATCC27255_00027_hypothetical_protein	RbromiiATCC27255
Org3_Gene254	unique	4077	[RbromiiATCC27255]RBATCC27255_00266_hypothetical_protein	RbromiiATCC27255
Org3_Gene268	unique	1227	[RbromiiATCC27255]RBATCC27255_00280_hypothetical_protein	RbromiiATCC27255
Org3_Gene280	unique	139	[RbromiiATCC27255]RBATCC27255_00292_Endo-1,4-beta-xylanase_Z_precursor	RbromiiATCC27255
Org3_Gene3	unique	1794	[RbromiiATCC27255]RBATCC27255_00003_Transposase	RbromiiATCC27255
Org3_Gene381	unique	2192	[RbromiiATCC27255]RBATCC27255_00394_hypothetical_protein	RbromiiATCC27255
Org3_Gene382	unique	3424	[RbromiiATCC27255]RBATCC27255_00395_hypothetical_protein	RbromiiATCC27255
Org3_Gene389	unique	2946	[RbromiiATCC27255]RBATCC27255_00402_hypothetical_protein	RbromiiATCC27255
Org3_Gene390	unique	3127	[RbromiiATCC27255]RBATCC27255_00403_hypothetical_protein	RbromiiATCC27255
Org3_Gene391	unique	2873	[RbromiiATCC27255]RBATCC27255_00404_hypothetical_protein	RbromiiATCC27255
Org3_Gene4	unique	4398	[RbromiiATCC27255]RBATCC27255_00004_hypothetical_protein	RbromiiATCC27255
Org3_Gene410	unique	2673	[RbromiiATCC27255]RBATCC27255_00423_Chaperone_protein_DnaJ	RbromiiATCC27255
Org3_Gene443	unique	2654	[RbromiiATCC27255]RBATCC27255_00458_hypothetical_protein	RbromiiATCC27255
Org3_Gene444	unique	3004	[RbromiiATCC27255]RBATCC27255_00459_hypothetical_protein	RbromiiATCC27255
Org3_Gene458	unique	2209	[RbromiiATCC27255]RBATCC27255_00473_Bacterial_Ig-like_domain_(group_2)	RbromiiATCC27255
Org3_Gene469	unique	3395	[RbromiiATCC27255]RBATCC27255_00487_putative_N-acetyltransferase_YjcF	RbromiiATCC27255
Org3_Gene5	unique	2880	[RbromiiATCC27255]RBATCC27255_00005_5-methylcytosine-specific_restriction_enzyme_subunit_McrC	RbromiiATCC27255
Org3_Gene505	unique	1609	[RbromiiATCC27255]RBATCC27255_00525_LPS_biosynthesis_protein	RbromiiATCC27255
Org3_Gene507	unique	555	[RbromiiATCC27255]RBATCC27255_00527_Polysaccharide_biosynthesis_protein	RbromiiATCC27255
Org3_Gene508	unique	881	[RbromiiATCC27255]RBATCC27255_00528_F420H2_dehydrogenase_subunit_F	RbromiiATCC27255
Org3_Gene509	unique	1080	[RbromiiATCC27255]RBATCC27255_00529_polysaccharide_pyruvyl_transferase_CsaB	RbromiiATCC27255
Org3_Gene510	unique	1622	[RbromiiATCC27255]RBATCC27255_00530_Putative_glycosyltransferase_EpsH	RbromiiATCC27255
Org3_Gene511	unique	3709	[RbromiiATCC27255]RBATCC27255_00531_Maltose_O-acetyltransferase	RbromiiATCC27255
Org3_Gene512	unique	1249	[RbromiiATCC27255]RBATCC27255_00532_UDP-N-acetylglucosamine_2-epimerase	RbromiiATCC27255
Org3_Gene513	unique	1506	[RbromiiATCC27255]RBATCC27255_00533_putative_glycosyltransferase_EpsJ	RbromiiATCC27255
Org3_Gene514	unique	998	[RbromiiATCC27255]RBATCC27255_00534_hypothetical_protein	RbromiiATCC27255
Org3_Gene515	unique	2964	[RbromiiATCC27255]RBATCC27255_00535_Galactoside_O-acetyltransferase	RbromiiATCC27255
Org3_Gene516	unique	1215	[RbromiiATCC27255]RBATCC27255_00536_N-acetylgalactosamine-N,N'-diacetylbacillosaminyl-diphospho-undecaprenol_4-alpha-N-acetylgalactosaminyltra	RbromiiATCC27255
Org3_Gene517	unique	2908	[RbromiiATCC27255]RBATCC27255_00537_Serine_acetyltransferase	RbromiiATCC27255
Org3_Gene519	unique	1529	[RbromiiATCC27255]RBATCC27255_00539_hypothetical_protein	RbromiiATCC27255

Org3_Gene524	unique	4083	[RbromiiATCC27255]RBATCC27255_00544_hypothetical_protein	RbromiiATCC27255
Org3_Gene525	unique	4423	[RbromiiATCC27255]RBATCC27255_00545_hypothetical_protein	RbromiiATCC27255
Org3_Gene526	unique	2485	[RbromiiATCC27255]RBATCC27255_00546_hypothetical_protein	RbromiiATCC27255
Org3_Gene527	unique	4365	[RbromiiATCC27255]RBATCC27255_00547_hypothetical_protein	RbromiiATCC27255
Org3_Gene528	unique	3808	[RbromiiATCC27255]RBATCC27255_00548_hypothetical_protein	RbromiiATCC27255
Org3_Gene529	unique	4301	[RbromiiATCC27255]RBATCC27255_00549_hypothetical_protein	RbromiiATCC27255
Org3_Gene531	unique	568	[RbromiiATCC27255]RBATCC27255_00551_Reverse_transcriptase_(RNA-dependent_DNA_polymerase)	RbromiiATCC27255
Org3_Gene532	unique	1386	[RbromiiATCC27255]RBATCC27255_00552_5-methylcytosine-specific_restriction_enzyme_subunit_McrC	RbromiiATCC27255
Org3_Gene533	unique	133	[RbromiiATCC27255]RBATCC27255_00553_5-methylcytosine-specific_restriction_enzyme_B	RbromiiATCC27255
Org3_Gene534	unique	3809	[RbromiiATCC27255]RBATCC27255_00554_hypothetical_protein	RbromiiATCC27255
Org3_Gene535	unique	3928	[RbromiiATCC27255]RBATCC27255_00555_hypothetical_protein	RbromiiATCC27255
Org3_Gene536	unique	445	[RbromiiATCC27255]RBATCC27255_00556_Recombinase	RbromiiATCC27255
Org3_Gene537	unique	615	[RbromiiATCC27255]RBATCC27255_00557_Deoxyguanosinetriphosphate_triphosphohydrolase	RbromiiATCC27255
Org3_Gene538	unique	271	[RbromiiATCC27255]RBATCC27255_00558_hypothetical_protein	RbromiiATCC27255
Org3_Gene539	unique	3847	[RbromiiATCC27255]RBATCC27255_00559_hypothetical_protein	RbromiiATCC27255
Org3_Gene540	unique	1522	[RbromiiATCC27255]RBATCC27255_00560_hypothetical_protein	RbromiiATCC27255
Org3_Gene541	unique	2758	[RbromiiATCC27255]RBATCC27255_00561_Coenzyme_F420:L-glutamate_ligase	RbromiiATCC27255
Org3_Gene542	unique	192	[RbromiiATCC27255]RBATCC27255_00562_radical_SAM_protein,_family	RbromiiATCC27255
Org3_Gene543	unique	511	[RbromiiATCC27255]RBATCC27255_00563_hypothetical_protein	RbromiiATCC27255
Org3_Gene551	unique	2730	[RbromiiATCC27255]RBATCC27255_00571_hypothetical_protein	RbromiiATCC27255
Org3_Gene552	unique	1586	[RbromiiATCC27255]RBATCC27255_00572_hypothetical_protein	RbromiiATCC27255
Org3_Gene553	unique	3738	[RbromiiATCC27255]RBATCC27255_00573_hypothetical_protein	RbromiiATCC27255
Org3_Gene554	unique	3486	[RbromiiATCC27255]RBATCC27255_00574_hypothetical_protein	RbromiiATCC27255
Org3_Gene555	unique	3513	[RbromiiATCC27255]RBATCC27255_00575_hypothetical_protein	RbromiiATCC27255
Org3_Gene556	unique	3482	[RbromiiATCC27255]RBATCC27255_00576_hypothetical_protein	RbromiiATCC27255
Org3_Gene557	unique	3290	[RbromiiATCC27255]RBATCC27255_00577_hypothetical_protein	RbromiiATCC27255
Org3_Gene558	unique	3781	[RbromiiATCC27255]RBATCC27255_00578_hypothetical_protein	RbromiiATCC27255
Org3_Gene559	unique	4109	[RbromiiATCC27255]RBATCC27255_00579_hypothetical_protein	RbromiiATCC27255
Org3_Gene560	unique	2224	[RbromiiATCC27255]RBATCC27255_00580_putative_membrane_protein	RbromiiATCC27255
Org3_Gene561	unique	88	[RbromiiATCC27255]RBATCC27255_00581_Phage-related_protein	RbromiiATCC27255
Org3_Gene562	unique	3459	[RbromiiATCC27255]RBATCC27255_00582_hypothetical_protein	RbromiiATCC27255
Org3_Gene563	unique	1003	[RbromiiATCC27255]RBATCC27255_00583_hypothetical_protein	RbromiiATCC27255
Org3_Gene564	unique	849	[RbromiiATCC27255]RBATCC27255_00584_putative_nucleotide-binding_protein_containing_TIR-like_domain_protein	RbromiiATCC27255
Org3_Gene565	unique	75	[RbromiiATCC27255]RBATCC27255_00585_Type-1_restriction_enzyme_R_protein	RbromiiATCC27255
Org3_Gene566	unique	1883	[RbromiiATCC27255]RBATCC27255_00586_hypothetical_protein	RbromiiATCC27255
Org3_Gene567	unique	1028	[RbromiiATCC27255]RBATCC27255_00587_EcoKI_restriction-modification_system_protein_HsdS	RbromiiATCC27255
Org3_Gene568	unique	341	[RbromiiATCC27255]RBATCC27255_00588_Type_I_restriction_enzyme_EcoKI_M_protein	RbromiiATCC27255
Org3_Gene571	unique	26	[RbromiiATCC27255]RBATCC27255_00591_Exoglucanase_B_precursor	RbromiiATCC27255
Org3_Gene587	unique	4386	[RbromiiATCC27255]RBATCC27255_00608_hypothetical_protein	RbromiiATCC27255
Org3_Gene6	unique	89	[RbromiiATCC27255]RBATCC27255_00006_Type-1_restriction_enzyme_R_protein	RbromiiATCC27255
Org3_Gene625	unique	3871	[RbromiiATCC27255]RBATCC27255_00646_Virulence_protein	RbromiiATCC27255
Org3_Gene628	unique	2788	[RbromiiATCC27255]RBATCC27255_00649_hypothetical_protein	RbromiiATCC27255
Org3_Gene636	unique	764	[RbromiiATCC27255]RBATCC27255_00657_Maltose-binding_periplasmic_proteins/domains	RbromiiATCC27255
Org3_Gene637	unique	2537	[RbromiiATCC27255]RBATCC27255_00658_hypothetical_protein	RbromiiATCC27255
Org3_Gene638	unique	1980	[RbromiiATCC27255]RBATCC27255_00659_putative_ABC_transporter_ATP-binding_protein_YxlF	RbromiiATCC27255
Org3_Gene639	unique	388	[RbromiiATCC27255]RBATCC27255_00660_hypothetical_protein	RbromiiATCC27255
Org3_Gene644	unique	1681	[RbromiiATCC27255]RBATCC27255_00665_hypothetical_protein	RbromiiATCC27255
Org3_Gene645	unique	3669	[RbromiiATCC27255]RBATCC27255_00666_hypothetical_protein	RbromiiATCC27255
Org3_Gene646	unique	874	[RbromiiATCC27255]RBATCC27255_00667_hypothetical_protein	RbromiiATCC27255
Org3_Gene647	unique	2105	[RbromiiATCC27255]RBATCC27255_00668_hypothetical_protein	RbromiiATCC27255
Org3_Gene650	unique	4371	[RbromiiATCC27255]RBATCC27255_00671_hypothetical_protein	RbromiiATCC27255
Org3_Gene651	unique	412	[RbromiiATCC27255]RBATCC27255_00672_Phage_minor_capsid_protein_2	RbromiiATCC27255
Org3_Gene652	unique	508	[RbromiiATCC27255]RBATCC27255_00673_hypothetical_protein	RbromiiATCC27255
Org3_Gene653	unique	745	[RbromiiATCC27255]RBATCC27255_00674_phage_terminase,_large_subunit,_PBSX_family	RbromiiATCC27255
Org3_Gene654	unique	3354	[RbromiiATCC27255]RBATCC27255_00675_Terminase_small_subunit	RbromiiATCC27255
Org3_Gene655	unique	3595	[RbromiiATCC27255]RBATCC27255_00676_hypothetical_protein	RbromiiATCC27255
Org3_Gene657	unique	4089	[RbromiiATCC27255]RBATCC27255_00678_hypothetical_protein	RbromiiATCC27255
Org3_Gene658	unique	1703	[RbromiiATCC27255]RBATCC27255_00679_hypothetical_protein	RbromiiATCC27255
Org3_Gene659	unique	3677	[RbromiiATCC27255]RBATCC27255_00680_hypothetical_protein	RbromiiATCC27255
Org3_Gene660	unique	4151	[RbromiiATCC27255]RBATCC27255_00681_hypothetical_protein	RbromiiATCC27255
Org3_Gene661	unique	3458	[RbromiiATCC27255]RBATCC27255_00682_Holliday_junction_resolvase	RbromiiATCC27255
Org3_Gene662	unique	4028	[RbromiiATCC27255]RBATCC27255_00683_hypothetical_protein	RbromiiATCC27255
Org3_Gene663	unique	206	[RbromiiATCC27255]RBATCC27255_00684_Regulatory_protein_RepA	RbromiiATCC27255
Org3_Gene664	unique	506	[RbromiiATCC27255]RBATCC27255_00685_type_I_restriction_enzyme_EcoKI_subunit_R	RbromiiATCC27255
Org3_Gene665	unique	2877	[RbromiiATCC27255]RBATCC27255_00686_hypothetical_protein	RbromiiATCC27255
Org3_Gene666	unique	1235	[RbromiiATCC27255]RBATCC27255_00687_phage_nucleotide-binding_protein	RbromiiATCC27255
Org3_Gene667	unique	3948	[RbromiiATCC27255]RBATCC27255_00688_hypothetical_protein	RbromiiATCC27255
Org3_Gene668	unique	873	[RbromiiATCC27255]RBATCC27255_00689_chromosome_segregation_protein	RbromiiATCC27255
Org3_Gene669	unique	3895	[RbromiiATCC27255]RBATCC27255_00690_hypothetical_protein	RbromiiATCC27255
Org3_Gene671	unique	2999	[RbromiiATCC27255]RBATCC27255_00692_hypothetical_protein	RbromiiATCC27255
Org3_Gene672	unique	2847	[RbromiiATCC27255]RBATCC27255_00693_hypothetical_protein	RbromiiATCC27255
Org3_Gene673	unique	4202	[RbromiiATCC27255]RBATCC27255_00694_hypothetical_protein	RbromiiATCC27255
Org3_Gene674	unique	2584	[RbromiiATCC27255]RBATCC27255_00695_LexA_repressor	RbromiiATCC27255
Org3_Gene675	unique	986	[RbromiiATCC27255]RBATCC27255_00696_Adenosine_monophosphate-protein_transferase_SoFic	RbromiiATCC27255
Org3_Gene7	unique	522	[RbromiiATCC27255]RBATCC27255_00007_putative_type_I_restriction_enzymeP_M_protein	RbromiiATCC27255
Org3_Gene711	unique	2803	[RbromiiATCC27255]RBATCC27255_00733_hypothetical_protein	RbromiiATCC27255
Org3_Gene749	unique	3209	[RbromiiATCC27255]RBATCC27255_00772_hypothetical_protein	RbromiiATCC27255
Org3_Gene782	unique	1252	[RbromiiATCC27255]RBATCC27255_00805_Endoglucanase_A_precursor	RbromiiATCC27255
Org3_Gene788	unique	1313	[RbromiiATCC27255]RBATCC27255_00811_Resuscitation-promoting_factor_Rpf2_precursor	RbromiiATCC27255
Org3_Gene792	unique	3101	[RbromiiATCC27255]RBATCC27255_00815_hypothetical_protein	RbromiiATCC27255
Org3_Gene827	unique	2705	[RbromiiATCC27255]RBATCC27255_00853_putative_membrane_protein	RbromiiATCC27255
Org3_Gene855	unique	292	[RbromiiATCC27255]RBATCC27255_00881_hypothetical_protein	RbromiiATCC27255
Org3_Gene856	unique	2740	[RbromiiATCC27255]RBATCC27255_00882_Kynurenine_formamidase	RbromiiATCC27255
Org3_Gene869	unique	1150	[RbromiiATCC27255]RBATCC27255_00895_putative_transposase_OrfB	RbromiiATCC27255
Org3_Gene876	unique	3942	[RbromiiATCC27255]RBATCC27255_00903_mRNA_interferase_YafQ	RbromiiATCC27255
Org3_Gene880	unique	4075	[RbromiiATCC27255]RBATCC27255_00907_mRNA_interferase_YafQ	RbromiiATCC27255
Org3_Gene881	unique	3931	[RbromiiATCC27255]RBATCC27255_00908_addiction_module_antitoxin,_RelB/DinJ_family	RbromiiATCC27255
Org3_Gene895	unique	1748	[RbromiiATCC27255]RBATCC27255_00922_hypothetical_protein	RbromiiATCC27255
Org3_Gene9	unique	2512	[RbromiiATCC27255]RBATCC27255_00011_hypothetical_protein	RbromiiATCC27255
Org3_Gene905	unique	2629	[RbromiiATCC27255]RBATCC27255_00932_CRISPR-associated_helicase_Cas3	RbromiiATCC27255
Org3_Gene920	unique	4276	[RbromiiATCC27255]RBATCC27255_00947_hypothetical_protein	RbromiiATCC27255
Org3_Gene932	unique	2867	[RbromiiATCC27255]RBATCC27255_00959_hypothetical_protein	RbromiiATCC27255
Org3_Gene996	unique	612	[RbromiiATCC27255]RBATCC27255_01024_hypothetical_protein	RbromiiATCC27255
Org4_Gene1	unique	3987	[RbromiiL2-36]RBL236_00001_Phage_protein_C	RbromiiL2-36
Org4_Gene1047	unique	4410	[RbromiiL2-36]RBL236_01097_hypothetical_protein	RbromiiL2-36
Org4_Gene1967	unique	1064	[RbromiiL2-36]RBL236_02051_hypothetical_protein	RbromiiL2-36
Org4_Gene2	unique	546	[RbromiiL2-36]RBL236_00002_DNA_replication_initiation_protein_gpA	RbromiiL2-36
Org4_Gene3	unique	1595	[RbromiiL2-36]RBL236_00003_minor_spike_protein	RbromiiL2-36
Org4_Gene4	unique	2976	[RbromiiL2-36]RBL236_00004_major_spike_protein	RbromiiL2-36
Org4_Gene5	unique	882	[RbromiiL2-36]RBL236_00005_capsid_protein	RbromiiL2-36
Org4_Gene6	unique	4427	[RbromiiL2-36]RBL236_00006_DNA_packaging_protein	RbromiiL2-36
Org5_Gene1149	unique	4313	[RbromiL2-63]L2-63_01182_L2-63_01182_hypothetical_protein_1255666:1255845_forward_MW:7133	RbromiL2-63
Org5_Gene944	unique	4374	[RbromiL2-63]L2-63_00974_L2-63_00974_hypothetical_protein_1037591:1037749_forward_MW:6158	RbromiL2-63
